# Prediction of N-linked glycosylation sites using position relative features and statistical moments

**DOI:** 10.1371/journal.pone.0181966

**Published:** 2017-08-10

**Authors:** Muhammad Aizaz Akmal, Nouman Rasool, Yaser Daanial Khan

**Affiliations:** 1 Department of Computer Science, School of Systems and Technology, University of Management and Technology, Lahore, Pakistan; 2 Department of Life Sciences, School of Sciences, University of Management and Technology, Lahore, Pakistan; Harbin Institute of Technology Shenzhen Graduate School, CHINA

## Abstract

Glycosylation is one of the most complex post translation modification in eukaryotic cells. Almost 50% of the human proteome is glycosylated as glycosylation plays a vital role in various biological functions such as antigen’s recognition, cell-cell communication, expression of genes and protein folding. It is a significant challenge to identify glycosylation sites in protein sequences as experimental methods are time taking and expensive. A reliable computational method is desirable for the identification of glycosylation sites. In this study, a comprehensive technique for the identification of N-linked glycosylation sites has been proposed using machine learning. The proposed predictor was trained using an up-to-date dataset through back propagation algorithm for multilayer neural network. The results of ten-fold cross-validation and other performance measures such as accuracy, sensitivity, specificity and Mathew’s correlation coefficient inferred that the accuracy of proposed tool is far better than the existing systems such as Glyomine, GlycoEP, Ensemble SVM and GPP.

## Introduction

**Fig 1:**
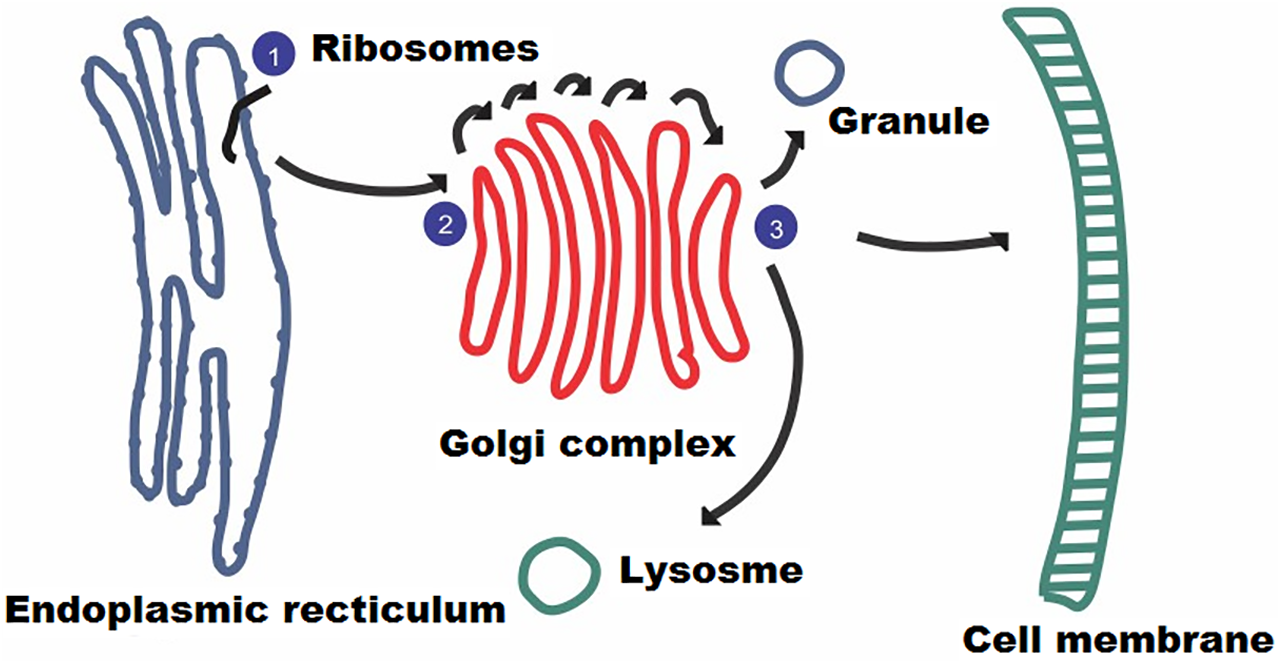
The process of glycosylation. Ribosomes attach to the cytoplasmic side of ER synthesis proteins. As protein moves, special enzymes attach to oligosaccharides via N-linkage.

Nascent protein after synthesis may undergo a variety of changes known as the post translation modification. Most of the proteins are unable to perform their normal physiological functions without undergoing such modifications. Each cell has a very accurate, sophisticated and flawless machinery incorporating specific enzymes responsible for modification of newly synthesized proteins. Glycosylation mainly manifests itself in the endoplasmic reticulum in eukaryotes, when protein after synthesis from ribosomes enters into the lumen of this organelle as shown in Fig 1. Almost 200 different kinds of post-translation modifications have been identified in various cells. Among these modifications, glycosylation holds an important position in which a carbohydrate moiety gets attached to a protein molecule. The addition of sugars to a specific amino acid of a protein results in the heterogeneity of protein, which helps it in performing a variety of cellular functions. Glycosylation plays a crucial role in a multitude of cell functions such as recognition of antigens, establishment of histocompatibility complex, protein turnover, expression of genes, controlling metabolism, protein folding, safeguarding against proteolysis and cell-cell adhesion and communication [1].

Various monosaccharides, oligosaccharides and their derivative form bonds with different amino acid residues within a protein as result of glycosylation. There are five classes of glycosylation: N-linked, O-linked, C-linked, Phospho glycosylation and glypiation. Every kind of glycosylation imparts a special characteristic to the modified protein as required by its role in cellular process. N-linked glycosylation is common amongst all types as it holds 90% share in total glycosylations [2]. The exposed asparagine residues of a protein are found to form N-linked bond with sugars. Any asparagine (N) residue appearing within a consensus pattern of sequence will form N-linked bond with sugars [3]. This modification is processed in endoplasmic reticulum (ER) lumen before exporting the modified protein to the cytoplasm or outside of the cell. In ER lumen dolichol molecule plays a pivotal role in this process [4]. The membrane-bound dolichol molecule has a long chain isoprene whose one end is attached with isoprenoid group and other with saturated alcohol [5].

It is difficult to identify such modifications experimentally after isolating proteins from a eukaryotic cell, without disrupting the native structure of the protein. Such analysis can be performed through mass spectrometry, which is a time consuming and costly technique. Computational determination of such modifications proves helpful for biologists saving their time and effort. Various researchers have proposed computational methods for determining glycosylation sites on the surface of protein using its primary structure.

Significant success has been achieved in the development of glycosylation predictive models, but still problems exist in such models that need to be addressed in order to develop better models, some of such shortcomings are listed as follows. (i) The quantity of dataset used for training limits the power and diversity of the prediction model because of inconclusive dataset diversity. (ii) The datasets used in existing models are outdated as many of experimentally verified newly discovered glycosylation sites has not been included in existing models.(iii) The feature space used by existing methods to construct models is indecisive and not comprehensive. Other potentially useful features are left uncovered that need to be characterized. The construction of the feature vectors used by the existing model for training does not meticulously extract the sequence and composition information that is crucial to identify an attribute of a protein. (iv) The accuracy of the existing models needs to be improved as some models hardly exhibit an accuracy up to 90%. Given these insufficiencies, it would be very useful to develop more accurate models that enable the systematic prediction of glycosylation.

In this study, computational method using machine learning and a comprehensive feature extraction technique is proposed for prediction of N-linked glycosylation sites. The dataset for prediction of N-linked glycosylated sites is collected from the *UniProt* database. Features pertinent to post-translational modification sites are extracted. Based on the extracted features, a neural network is trained using back propagation approach [6, 7]. Subsequently, validation of the model is performed using several quantitative measures including receiver operating characteristics, regression metric, accuracy metric, Mathew correlation coefficient, sensitivity, specificity, cross-validation and jackknife testing.

### Literature review

Researchers have made numerous contributions in developing several computational models to predict an attribute of a protein [8]. Studies showed that attributes of a protein are reliant not only on the composition of amino acids but also on the sequence in which amino acids occur in the polypeptide chain [9]. The recent work in [10] reviews design of effective feature extraction techniques based upon composition as well as the sequence of component amino acids. Enhancing the work of other researchers, the authors in [11] developed a universal technique suitable for feature extraction from proteomic as well as genomic data. Caragea et al. [12] proposed glycosylation predictor for N-, O- and C-linked sites. In this method, the authors used support vector machine (SVM) having Ensemble and Single classifier. Both of the classifiers are trained on the given dataset, performance comparison shows that Ensemble SVM performed better than Single SVM having an accuracy of 95%. The recent work of Liu et al enhanced the performance of ensemble classifiers by incorporating clustering and dynamic selection strategies [13]. Hamby and Hirst [14] proposed predictive tool GPP that was developed for the identification of glycosylation sites. Random forests algorithm based on decision tree was used to develop this model. In this algorithm a tree consists of nodes and paths, at each node it has to choose the path according to the defined rules. Using the random selection of input and features several trees are generated. A voting mechanism selects a particular class against given input trees. Chauhan et al. [15] developed GlycoEP tool for N-, O- and C- linked glycosylation site identification using different kernel functions like linear, polynomial and radial basis function (RBF) with diverse learning parameters. The results showed that RBF kernel outperforms other functions. Recently another predictive model GlycoMine was developed by Li, Fuyi et al. [16] for glycosylation identification in human proteomes. The random forest algorithm along with a novel feature extraction technique was used in order to improve performance. The feature selection was based on information gain (IG) and minimum redundancy maximum relevance (MRMR) principles.

## Materials and methods

**Fig 2:**
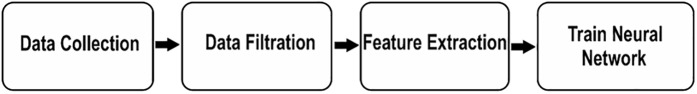
The proposed model workflow. The work flow of the proposed model is shown which includes four phases: Data Collection, Data Filtration, Feature Extraction and TNN.

The process developed for prediction of N-Linked glycosylation sites is illustrated in the current section. It comprises of four phases data collection, data filtration, feature extraction and training as shown in Fig 2. The first phase involved collection of benchmark data from a well-known online database of proteins namely *UniProt*. In the second phase, subsequences which were most relevant to N-linked glycosylation were extracted from the raw data containing primary structure. After removing duplication, carefully selected sequences forming a representative subset of overall data was used for training purposes. In the third phase, a variety of features were extracted, including position and composition variant features, raw, Hahn and central moments. In the last phase, input feature matrix comprising of feature vectors and an output matrix comprising of expected output were used to train the multilayered neural network through back propagation approach. The trained model is then validated on various test datasets will be described later in validation section.

### The dataset collection

**Fig 3:**
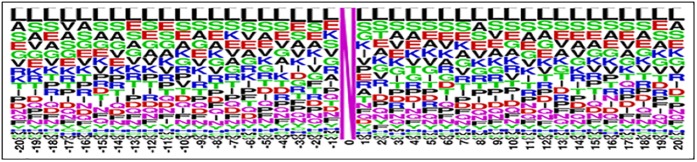
Sequence logo for (–ve) N-linked glycosylation sites. The logo depicts residues occurring on specific positions. All sites were aligned with non-glycosylated N-linked at position 0.

**Fig 4:**
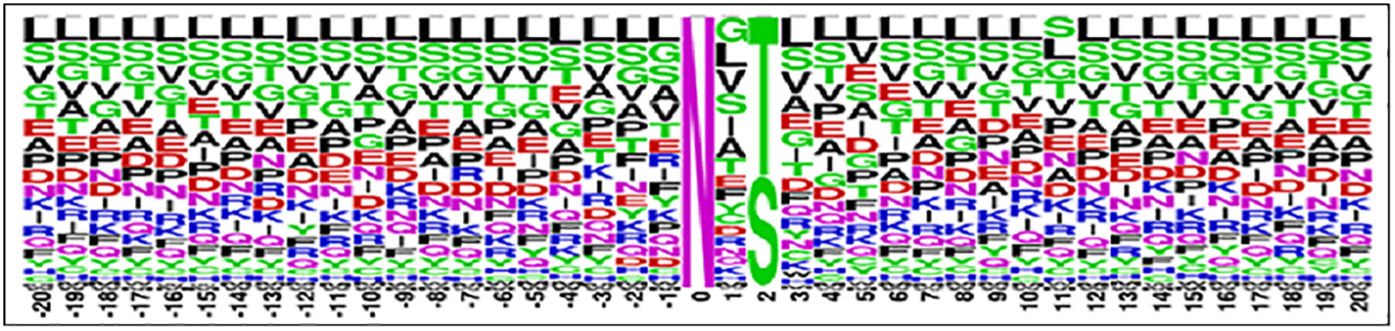
Sequence logo for (+ve) N-linked glycosylation sites. The logo illustrates residues occurring on specific positions. All sites were aligned with glycosylated N-linked at position 0.

The dataset for the prediction of the N-linked glycosylation is collected from one of the most authentic databases *UniProt* available at http://www.uniprot.org. The UniProt database is a comprehensive database which has been meticulously annotated on the basis of protein functionality and characteristics. In order to accumulate positive samples a database is formed by collecting the subset of protein in which some experimental evidence of N-linked glycosylation has been observed. To serve this purpose only those proteins were listed which were annotated with the field PTM/Processing. Additionally, within the obtained list only those proteins were included that contained the term glycosylation in feature (FT) attribute. To further ascertain the credibility of dataset only those proteins were collected in which this observation was based on experimental assertion. From the obtained dataset those proteins were left out which were not reviewed. Ultimately, this query ended up with 2964 proteins where each protein may contain a number of glycosylation sites. The sequence of amino acid residues at the glycosylation site and its vicinity bore more relevance than the entire primary structure of the protein [17]. Based upon this principle in the next step a subsequence of amino acid residues is extracted from each glycosylation site. Subsequences were extracted from the position attribute within the location element of only those features whose type attribute was “glycosylation site” and the type attribute was “*N-linked (GlcNAc…)”*. The formation of a query string to extract data from the database included all of these described features. Furthermore, in case of negative dataset a converse query string is generated. As a result, the sequences of N-linked glycosylation sites extracted from the raw data contained a total of 23761 instances. Out of these sites 11601 were N-linked glycosylation positive sites while the rest of the 12160 sites were negative. Each instance of these subsequences had a length of 41 residues. Twenty neighboring amino acid residues on both ends of Asparagine (N) residue were selected. The decision regarding the number of neighboring residues was made based on probing and experimentation such that the most optimal outcome is achieved. The collected dataset is then filtered by removing the duplicated entries, only 11461 positive (S2 File) and 12000 negative (S1 File) instances of N-linked glycosylation are left in the dataset. Alignment diagrams depicting the positive and negative datasets are shown in Fig 3 and Fig 4 respectively [18].

### Feature vector construction

The biophysical characteristics of proteins are determined by the sequence in which amino acids are incorporated in the polypeptide chain. The mere presence or absence of an amino acid does not represent a characteristic. Composition of amino acids is not the only factor that affects the proteins' behavior rather relative positioning of constituent amino acid residues is extremely significant. It has been observed through known data and experience that a minute change in the relative positioning of amino acids may alter the characteristics of the protein altogether [10]. These facts edict that a mathematical model which extracts features from the primary structure of the protein should not only be based on the information pertaining to the constituents of the proteins, but should also regard the relative positioning of the amino acids as an important factor [19].

#### Site vicinity vector

It has been observed that some sites are susceptible to Post Translational Modification (PTM) while some are not. There are number of factors that contribute towards such modifications. Most of the factors are environmental, the work of [20] shows that susceptibility of a potential site is dependent upon its neighboring residues in the peptide chain. Let *α*_*q*_ depict the potential PTM site, then the neighboring residues in the polypeptide chain are illustrated as

$$Ρ=\{\alpha_{1}\ldots \alpha_{q-2},\alpha_{q-1},\alpha_{q},\alpha_{q+1},\alpha_{q+2},\ldots ..\alpha_{n}\}$$ (1)

The Site Vicinity Vector (SVV) is derived as a sub-structure of the primary sequence which contains the potential site along with its neighbors given as

$$\alpha_{q-r}\ldots \alpha_{q-2},\alpha_{q-1},\alpha_{q},\alpha_{q+1},\alpha_{q+2},\ldots ..\alpha_{q+r}$$ (2)

Where *r* is a small integer value which is optimally selected through probing and experimentation. The SVV, which forms a component of the inclusive feature vector, is assigned unique numerical values substituting each residue position. Only 20 amino acids are significant in terms of protein synthesis, in order to extract a feature vector, each amino acid is assigned a unique integer value. As long as the values are unique, integral and are assigned consistently, it does not matter which value is assigned to which amino acid.

#### Statistical moments of primary structure

Statistical moments are a quantitative measure used for describing a collection of data. Various orders of moments describe various properties of data. Some moments may be used to evaluate the size of the data while some are indicative of its orientation and eccentricity. Mathematicians and statisticians have formed various moments based on certain well known polynomials and distribution functions. The moments used in order to elucidate the proposed problem are raw; central and Hahn moments. Raw moments are used for calculating mean, variance and asymmetry of the probability distribution, formed by the collected dataset. Raw moments are neither scale-invariant nor location-invariant [6, 21 & 22]. The central moments also provide similar information, but these moments are computed along the centroid of the data, which makes it location invariant with respect to the centroid nonetheless it is still scale-variant [21, 22 & 23]. Hahn moments are based on Hahn polynomials; these moments are neither scale-invariant nor location-variant. The obvious reason behind the choice of these moments is their sensitivity to sequence ordered information which is of prime significance as discussed earlier. Consequently, use of scale invariant moments has been avoided. The quantified values returned from each method describe data in its own way. Furthermore, variation in the quantified value of moments for arbitrary datasets implies variations in the characteristics of the data source [20, 24 & 25]. A two dimensional version of these moments is used therefore the one dimensional primary sub-sequence is firstly transformed into a two dimensional notation.

Let a protein sequence/sub-sequence P be represented as given below

P={a1,a2,a3,….ak} (3)

Where α_i_ is the *i*^*th*^ amino acid residue component in a primary sub-sequence containing k residues, also let,

$$n=\lceil \sqrt{k}\rceil$$ (4)

A matrix P’ is formed of dimension n*n to accommodate all the amino acid components of the protein P.

$$P’=\left[\begin{array}{ccccc} \beta_{11} & \beta_{12} & \cdots & & \beta_{1n}\\ \beta_{21} & \beta_{22} & \cdots & & \beta_{2n}\\ \vdots & \vdots & & \ddots & \vdots \\ \beta_{n1} & \beta_{n2} & \cdots & & \beta_{nn} \end{array}\right]$$ (5)

The 2-dimensional matrix P’ corresponds to the primary structure P. A mapping function ω is used to transform the matrix P into P’.

$$\omega (a_{m})=\beta_{ij}$$ (6)

Where $i=\frac{m}{n}+1$ and *j = m mod n* if P’ is populated in a row major manner.

The contents of the 2D matrix P’ are used for computation of moments up to degree 3; raw moments are computed using the following relation

$$M_{ij}=\sum_{p=1}^{n}\sum_{q=1}^{n}p^{i}q^{j}\;\beta_{pq}$$ (7)

Where *i + j* is the order of the moments. Moments up to order 3 were computed which are listed as *M*_00_, *M*_01_, *M*_10_, *M*_11_, *M*_12_, *M*_21_, *M*_30_ and *M*_03_.

The centroid of the data is like the center of gravity. The centroid is the point in data where data is evenly distributed in all directions in terms of its weighted average. It is easily computed after the computation of raw moments. It is given as a point $\bar{x},\bar{y}$ where

$$\bar{x}=M_{10}/M_{00}\;and\;\bar{y}=M_{01}/M_{00}$$ (8)

The centroid is used to compute the central moments. Central moments, are more like moments, used in physics along the center of gravity where the centroid behaves as the center of gravity of data. They are computed using the following relation

$$\eta_{ij}=\sum_{p=1}^{n}\;\sum_{q=1}^{n}{\left(p-\bar{x}\right)}^{i}{\left(q-\bar{y}\right)}^{j}\;\beta_{pq}$$ (9)

The one dimensional notation P was transformed into a square matrix notation P’. This transformation has to offer a greater dividend as Hahn moments can be computed on such an even dimensional organization of data. Two-dimensional discrete Hahn moments are orthogonal moments that require a square matrix as a two dimensional input data. Another leverage offered by Hahn moments is they are orthogonal which implies they have reversible property. This property renders it possible to reconstruct the original data using the inverse functions of discrete Hahn moments. This further connotes that the positional and compositional information of a primary sequence is somehow conserved within the computed moments. The Hahn polynomial order of n is given as

$$h_{n}^{u,v}(r,N)={\left(N+V-1\right)}_{n}{\left(N-1\right)}_{n}\times \sum_{k=0}^{n}{\left(-1\right)}^{k}\frac{{\left(-n\right)}_{k}{\left(-r\right)}_{k}{\left(2N+u+v-n-1\right)}_{k}}{{\left(N+v-1\right)}_{k}{\left(N-1\right)}_{k}}\frac{1}{k!}$$ (10)

The above expression uses the pochhammer symbol generalized as

$${\left(a\right)}_{k}=a.(a+1)\cdots (a+k-1)$$ (11)

And is simplified using the Gamma operator

$${\left(a\right)}_{k}=\frac{\Gamma (a+k)}{\Gamma (a)}$$ (12)

The raw values of Hahn moments are usually scaled using a weighting function and square norm given as

$$h_{n}^{\tilde{u,v}}(r,N)=h_{n}^{u,v}(r,N)\sqrt{\frac{p(r)}{d_{n}^{2}}},\;n=0,1,\cdots ,N-1$$ (13)

While

$$p(r)=\frac{\Gamma (u+r+v)(v+r+1){\left(u+v+r+1\right)}_{N}}{(u+v+2r+1)n!(N-r-1)!}$$ (14)

The orthogonal normalized Hahn moments for two dimensional discrete data matrix are computed using the following equation,

$$H_{ij}=\sum_{q=0}^{N-1}\sum_{p=0}^{N-1}\beta_{ij}h_{i}^{\tilde{u,v}}\;(q,N)h_{j}^{\tilde{u,v}}(p,N),\;m,n=0,1,\cdots N-1$$ (15)

Two dimensional raw, central and Hahn moments are computed for each primary sequence up to order 3 and are later incorporated into the miscellany feature vector.

#### Position relative incidence matrix

Sequence order information forms the basis of any mathematical model used to predict the behavior of proteins. The relative positioning of amino acid residues is one of the core paradigms governing the physical attributes of the protein. It is also important to quantize how amino acids are relatively placed in the polypeptide chain. Position Relative Incidence Matrix (PRIM) excerpts the relative positioning information of amino acid components in the polypeptide chain. PRIM is formed as a matrix with dimensions of 20x20 elements as shown below:

$$S_{PRIM}=\left[\begin{array}{cccc} A_{1\to 1} & A_{1\to 2}\cdots & A_{1\to j}\cdots & A_{1\to 20}\\ A_{2\to 1} & A_{2\to 2}\cdots & A_{2\to j}\cdots & A_{2\to 20}\\ A_{i\to 1}^{\vdots } & A_{i\to 2}^{\vdots }\cdots & A_{i\to j}^{\vdots }\cdots & A_{i\to 20}^{\vdots }\\ A_{N\to 1}^{\vdots } & A_{N\to 2}^{\vdots }\cdots & A_{N\to j}^{\vdots }\cdots & A_{N\to 20}^{\vdots } \end{array}\right]$$ (16)

An element *A*_*i*→*j*_ holds the sum of relative position of *j*^*th*^ residue with respect to the first occurrence of the *i*^*th*^ residue. PRIM yields 400 coefficients which is a large number. To further reduce the number of coefficients, moments are computed using PRIM as the input. This generates another set of data containing 24 elements [4].

#### Reverse position relative incidence matrix

The efficiency and the accuracy of any machine learning algorithm is vastly dependent on the thoroughness and the meticulousness by which the most relevant aspects of data have been extracted. A machine learning algorithm has the capability to adapt itself in understanding and uncovering obscure patterns embedded within data. The PRIM matrix uncovers or extracts information regarding the relative positioning of amino acids within the polypeptide chain. Another matrix, namely Reverse Position Relative Incidence Matrix (RPRIM) is formed which works the same way as PRIM but on the reverse primary sequence. Introduction of RPRIM helps uncover yet further hidden patterns and alleviate ambiguities among proteins with seemingly resembling polypeptide sequences.

RPRIM is again a matrix with 400 elements having dimensions of 20x20. Formally, it is given as

$$S_{RPRIM}=\left[\begin{array}{cccc} Z_{1\to 1} & Z_{1\to 2}\cdots & Z_{1\to j}\cdots & Z_{1\to 20}\\ Z_{2\to 1} & Z_{2\to 2}\cdots & Z_{2\to j}\cdots & Z_{2\to 20}\\ Z_{i\to 1}^{\vdots } & Z_{i\to 2}^{\vdots }\cdots & Z_{i\to j}^{\vdots }\cdots & Z_{i\to 20}^{\vdots }\\ Z_{N\to 1}^{\vdots } & Z_{N\to 2}^{\vdots }\cdots & Z_{N\to j}^{\vdots }\cdots & Z_{N\to 20}^{\vdots } \end{array}\right]$$ (17)

Dimensionality of the large RPRIM matrix is reduced by computing it’s raw, central and Hahn moments to transform it into a feature vector with only 24 coefficients.

#### Frequency matrix

A frequency matrix is formed, which is the distribution of occurrence of each amino acid residue within the primary structure. The Frequency matrix is given as

$$\xi =\{\tau_{1},\tau_{2},\cdots ,\tau_{20}\}$$ (18)

Where τ_i_ is the frequency of occurrence of *i*^*th*^ native amino acid. The frequency matrix contains information regarding the composition of the protein. It is evident that the frequency matrix leaves out the sequence information. The sequence information has already been extracted into PRIM.

#### Accumulative absolute position incidence vector (AAPIV)

The frequency matrix provides the accumulative frequency occurrence of the amino acid residues in the polypeptide chain while AAPIV provides the information regarding the composition of the protein. Evidently, accumulative frequency matrix discards information regarding relative positioning of the amino acid residues. AAPIV is formed to extract the information regarding the positioning of the amino acid residues in the polypeptide chain. A vector is formed with 20 elements such that each element holds the sum of all the ordinal values at which the corresponding residue occurs in the primary structure. Formally, it is described by means of the following representation of primary sequence which depicts the occurrence of a specific residue in the primary structure

$$\alpha_{p1}^{i}\ldots \alpha_{p2}^{i}\ldots \alpha_{p3}^{i}\ldots ..\alpha_{pn}^{i}$$ (19)

It depicts that a specific residue *α*^*i*^ occurs at locations *p*_1_,*p*_2_,*p*_3_,…*p*_*n*_.

Let AAPIV vector be denoted as

$$Κ=\{\mu_{1},\mu_{2},\mu_{3},\ldots ,\mu_{20}\}$$ (20)

Hence an arbitrary *i*^*th*^ element of AAPIV is computed as

$$\mu_{i}=\sum_{k=1}^{n}p_{k}$$ (21)

#### Reverse accumulative absolute position incidence vector (RAAPIV)

As discussed earlier, it is desirable, for a feature extraction method, to be capable of uncovering deeply obscure patterns. A RAAPIV is formed to do just the same. RAAPIV is built by reversing the primary structure string and then extracting AAPIV from the reversed string. Formally the RAAPIV is illustrated as 20 element vector denoted as

$$\Lambda =\{\eta_{1},\eta_{2},\eta_{3},\ldots ,\eta_{20}\}$$ (22)

Let the occurrences of a specific residue in the reversed sequence be depicted as

$$\alpha_{l1}^{i}\ldots \alpha_{l2}^{i}\ldots \alpha_{l3}^{i}\ldots ..\alpha_{ln}^{i}$$ (23)

Where *l*_1_,*l*_2_,*l*_3_,…*l*_*n*_ are the ordinal locations where the residue *α*^*i*^ occurs in the reverse sequence. The values of an arbitrary element of Λ is given as

$$\eta_{i}=\sum_{k=1}^{n}l_{k}$$ (24)

### Training neural network

**Fig 5:**
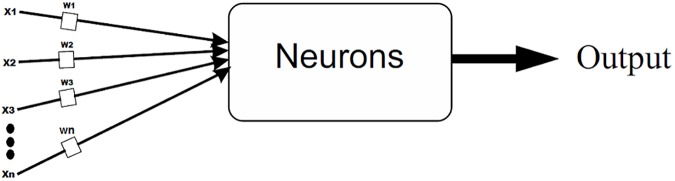
Process of neural network. In neural network input values and initial weights are assigned to the network and based on these values network start its learning.

The neural network is one of the most powerful techniques used to solve decision problems. A Neural Network works in a way similar to human nervous system. The human brain receives information from the environment and learns from its experience, the neural network adopts a similar approach. It receives labelled input and based on the experience gained from each input, it develops an opinion regarding each input during the training process. After training process is completed the network seemingly behaves in a way that makes it capable to classify each given input within an acceptable degree of accuracy Fig 5. During the learning process the goal of the neural network is to reduce the error. During each iteration the network adjusts its weights such that the error is minimized which essentially translates into improved learning and increased accuracy in the prediction of relevant class for an arbitrary input.

**Fig 6:**
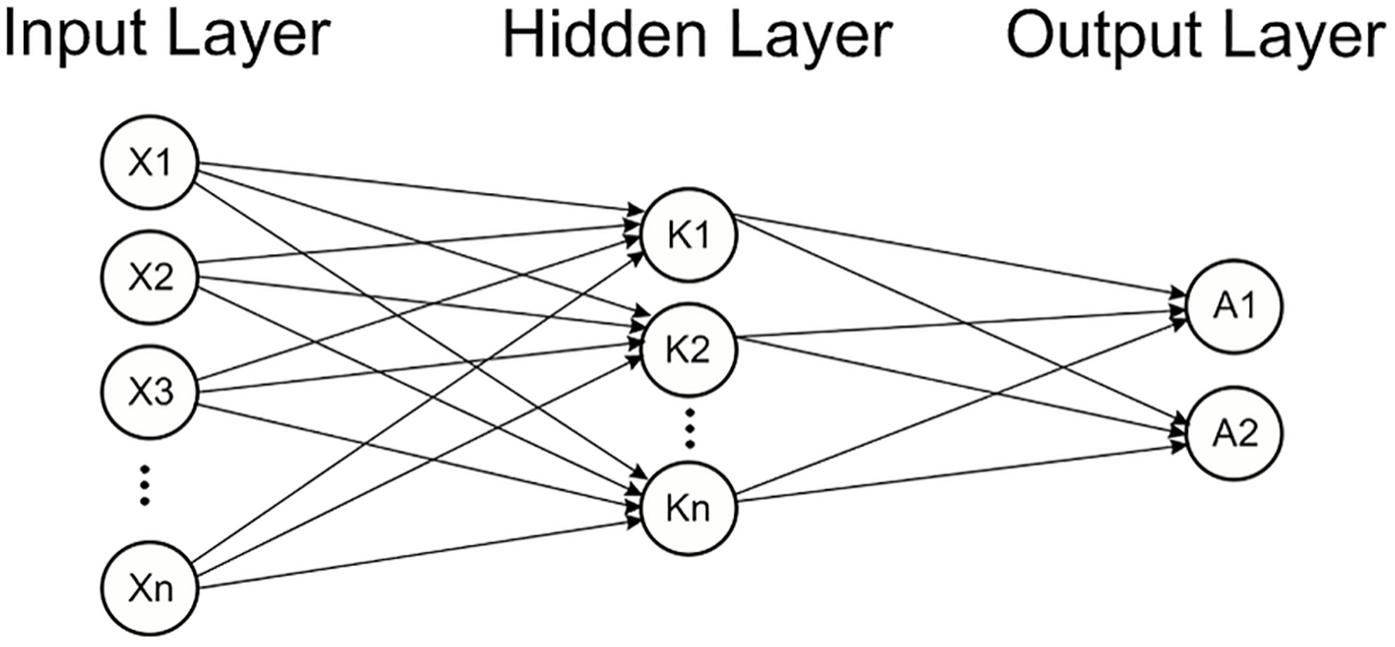
Multiple layer back propagation neural network. Artificial Neural Network having multiple layers is used for the prediction of N-linked glycosylation sites.

The artificial neural network approach is very effective for developing a classifier in a supervised or an unsupervised manner. The prediction algorithm developed for prediction of N-linked glycosylation sites also employs supervised learning. A multilayer back propagation neural network quite similar to the one used in [7] has been employed to tackle this problem as shown in Fig 6. The depths and details pulled out into the feature vector from raw data plays a vital role. A feature vector (FV) capable of discriminating data semantically is bound to provide assiduous results. The FV constructed for the prediction of the N-linked glycosylation sites consist of a large number of coefficients. The main discriminating attributes in the FV are SVV, FM, AAPIV and RAAPIV along with the raw, central and Hahn moments of PRIM, RPRIM and the two dimensional primary structure as discussed in the previous sections.

The proposed methodology for the prediction of the N-linked glycosylation in this paper consists of several phases. In the first phase, the dataset of the N-linked glycosylation was collected from the *UniProt* database as described previously. Initially, the data is in the form embedded within XML text, from which sequences are extracted using a parsing script. The second phase deals with the filtration of data in which duplicate entries has been removed to eliminate homology bias. Features were extracted from this data to form FVs. The dataset formed for this study consists of experimentally obtained data for negative as well as positive N-linked glycosylation sites. Both the FVs are combined to form an input file while each input vector is labelled as a positive or a negative sample in another expected output file. The training of the multilayered neural network is performed using back propagation technique. In order to reduce the error and increase the prediction accuracy gradient descent technique was used along with an adaptive learning rate.

### Gradient descent and adaptive learning

Gradient descent is one of the most commonly used training functions. The objective of the gradient descent algorithm is to iteratively find the set of parameters that minimizes the function [26]. This minimization is performed by moving in a direction opposite to the direction of the function gradient. The function gradient is calculated by computing the rate of change in successive outcomes. Assuming that the objective function K(*θ*) is parameterized by variable $\theta \in R^{d}$ then its gradient function is given as ∇_*θ*_*K*(*θ*). The function is minimized by moving in a direction opposite to the direction of the gradient. Based on this concept the parameters are re-calculated at each step as given in the following equation

$$\theta =\theta -\gamma \nabla_{\theta }K(\theta )$$ (25)

Where γ is the learning rate. The learning rate is usually kept constant. The performance of the algorithm greatly depends on the learning rate. It determines how quickly the function is minimized. If the learning rate is too small, then too much time might be required to reach convergence. In case, the learning rate is too large the function may oscillate and never reach the optimal point. Hence the learning rate must be kept at an optimal value. Adaptive learning algorithm varies the value of the learning rate, depending upon the performance of the algorithm. Parameters computed for a successive iteration are discarded in case the error increased in successive iterations. The learning rate is varied such that the function is minimized in each iteration. Formally, let *θ*_*i*_ and *θ*_*i*+1_ be two successively computed parameters. The weights are recalculated, using these parameters and the corresponding outputs, and subsequently the errors are also computed. Consequently, if the errors are greater as compared to the previous epoch then the learning rate is decreased; weights are discarded and the newer value of *θ*_*i*+1_ is computed. Similarly, if it is lesser, then the learning rate is increased. As a result the learning rate keeps on varying depending on the progress of the algorithm. Theoretically, the learning rate can vary on each epoch, formally if (θ_0_, θ_1_, θ_2_, θ_3_,…) are the parameters computed for each successive epoch, then they are computed using the following equation

$$\theta_{m+1}=\theta_{m}-\gamma_{m}\nabla K(\theta_{m})$$ (26)

Where *γ*_*m*_ is the learning rate used for *m*^*th*^ epoch. The adaptive learning algorithm ensures that learning rate is moderated in a way that the function is minimized at each epoch. The selection of learning rate always satisfies the following condition.

$$K(\theta_{0})\geq K(\theta_{1})\geq K(\theta_{2}),\cdots \;\cdots$$ (27)

## Experimentation and results

The proposed model endeavors to predict N-linked glycosylation sites in protein molecules that resides in prokaryotic as well as eukaryotic cells. N-linked glycosylation plays a pivotal role in protein folding and subsequent restructuring of protein molecule. The prediction model is based on a position and composition variant feature extraction technique. Benchmark test data is contrived in a number of ways to carry out experiments which justify effectiveness of the prediction model [8 & 9]. The results obtained from these experiments are described in this section.

### Self-consistency test

**Fig 7:**
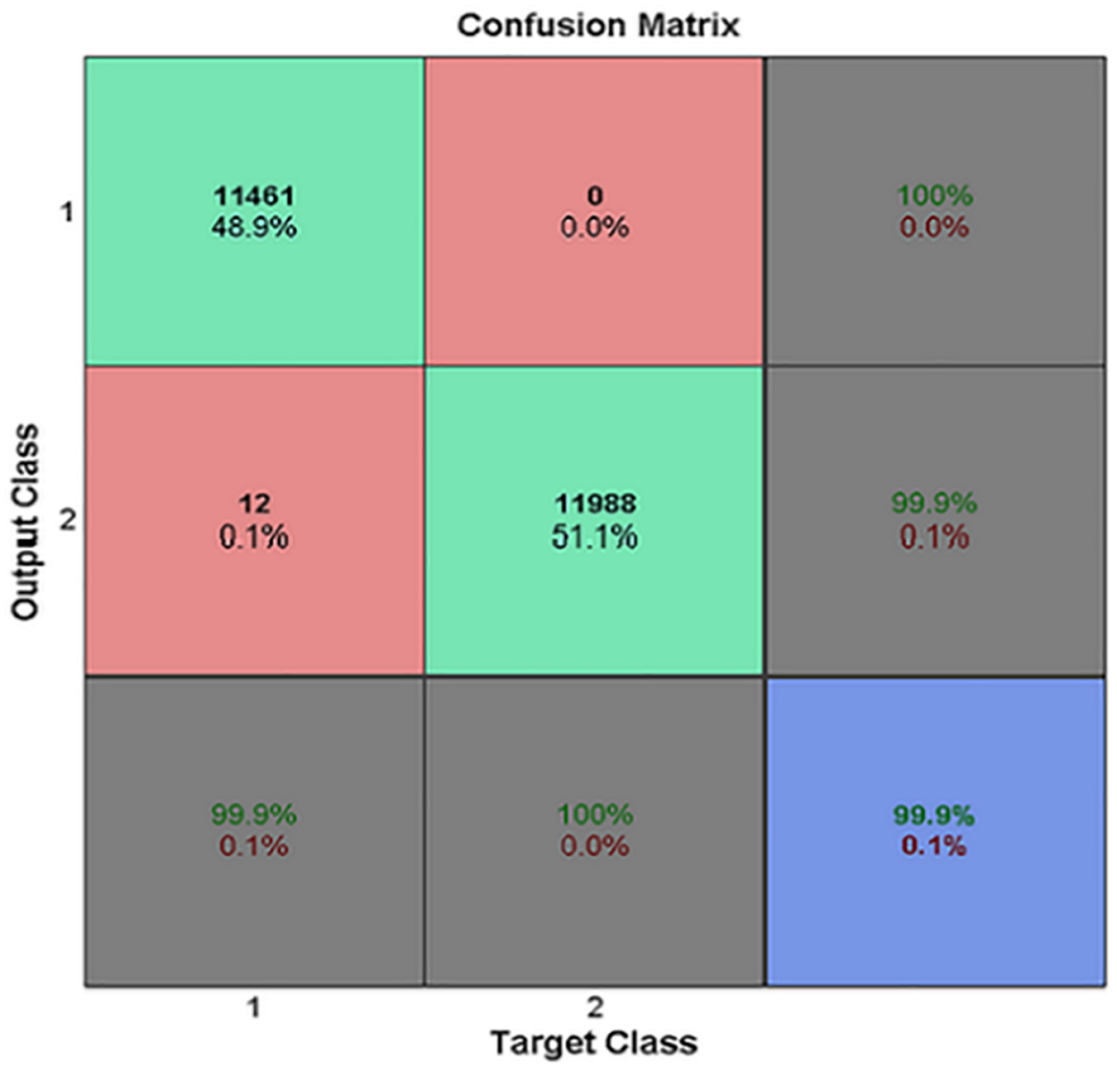
A confusion matrix of the prediction model. The values of TP, FN, FP and TN are 11461, 12, 0 and 11988 respectively. Overall accuracy also is 99.9% as shown.

Self-consistency test is the most fundamental test used by various researchers to prove the effectiveness of a predictor. Self-consistency test is carried out by gathering test case from the training domain. The results obtained from self-consistency test are elaborated using confusion matrix. Confusion matrix is an illustrious tool used to describe the accuracy of a model. It describes the prediction result against the actual data. True positive (TP) means an item correctly identified by the model as a positive N-linked glycosylation site while false negative (FN) means that a positive site was incorrectly marked as a negative site. False positive (FP) means that the model incorrectly marked a negative site as a positive site, and true negative (TN) means the model correctly identified the negative N-linked glycosylation site. An illustration of these parameters for the proposed predictive model obtained from self-consistency test is presented in Fig 7. The prediction rate for identification of positive N-linked sites is 99.9% while for negative sites it is 100%.

**Fig 8:**
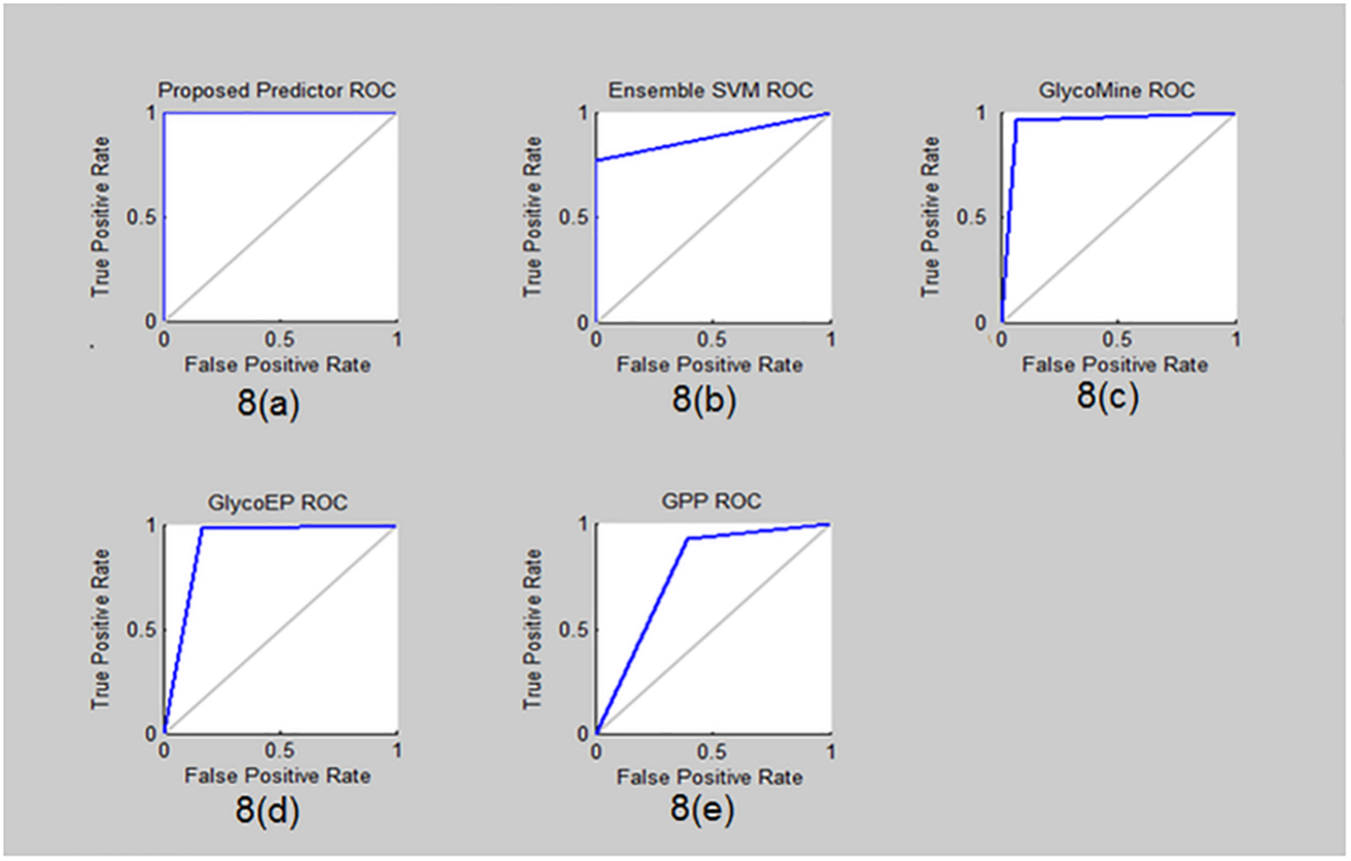
ROC comparison graph. The ROC graph comparison between proposed and other predictors like Ensemble SVM, Glycomine, GlycoEP and GPP.

Similarly Receiver Operating Characteristics (ROC) graph is another illustrative tool used to describe the results of the predictive model [27,28,29]. The ROC graph for the proposed prediction model based on the self-consistency test is depicted in Fig 8(A). It is apparent that area under the curve line in blue color in almost maximal as the curve touched the left top corner which implies that it has a True Positive Rate (TPR) approaching 1 and it also suggests that the accuracy of the predictive model is nearing 100%.The comparative results illustrated in the ROC graph amongst proposed and the existing predictors are shown in Fig 8(A) and 8B, 8C, 8D and 8E respectively, it is clearly observed that the accuracy of the proposed model is much higher than the existing ones.

**Fig 9:**
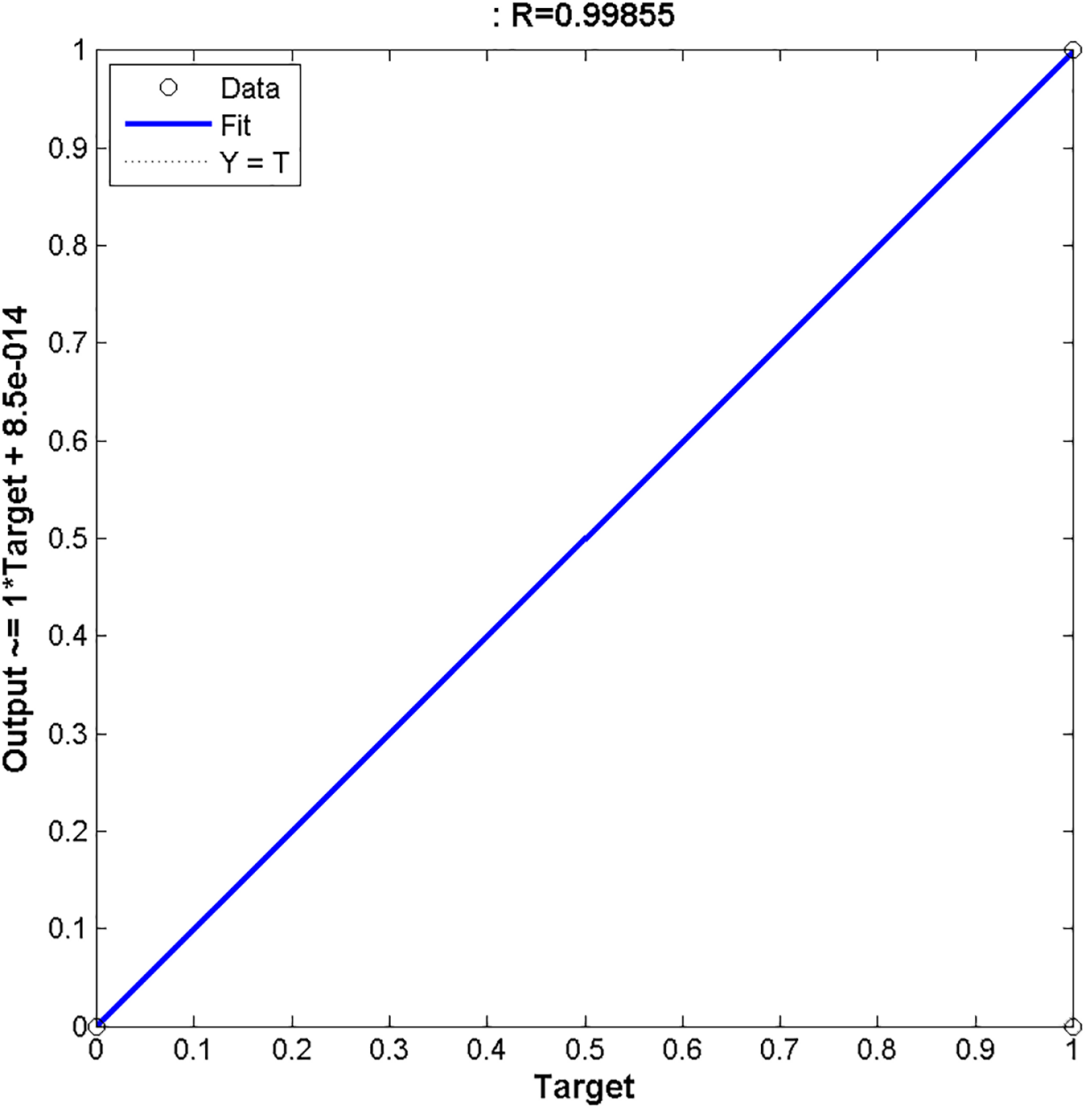
Regression metric. Regression Metric of proposed N-Linked predictor is shown. The regression value is 0.99 which shows it has a negligible error rate.

Furthermore, regression metric is another useful tool to measure the accuracy of the predictive model by calculating estimation of error. It is basically a statistical tool for the investigation of the relationship between a responsive variable (X) and one or more predictive variables (Y) [30]. For instance an arbitrary point *P*_i_(*X*_*i*,_ *Y*_*i*_*)* is defined by variables *X*_*i*_ and *Y*_*i*_. Within a dataset several such points exist, if all the points of given data lie on a straight line then it implies that the accuracy of the model is outstanding and if the data point are scattered on *XY* plane then it is indicative that poor accuracy is being exhibited by the predictor. The regression analysis of proposed predictive model is shown in Fig 9. The Figure clearly depicts that all the data points lie on a straight line. It also shows that the regression value is 0.99 which indicates excellent accuracy.

Sensitivity (Sn), specificity (Sp), Accuracy (Acc) and Mathew’s correlation coefficient are the most common quantitative metrics used to gauge the performance of a predictor [27,28,29,31,32,33,34]. The following equations demonstrate how these metrics are computed using the results of self-consistency test

$$Sn=\frac{\sum True\;Positive}{\sum Postive\;Sample\;Space}$$ (28)

$$Sn=\frac{TP}{TP+FN}$$ (29)

$$Sp=\frac{\sum True\;Negative}{\sum Negative\;Sample\;Space}$$ (30)

$$Sp=\frac{TN}{FP+TN}$$ (31)

$$Acc=\frac{\sum True\;Negative+\sum True\;Positive}{\sum Total\;Sample\;Space}$$ (32)

$$Acc=\frac{TN+TP}{TP+TN+FP+FN}$$ (33)

$$MCC=\frac{\left(\left(TN*TP)-(FN*FP\right)\right)}{\sqrt{\left(FP+TP)(FP+TN)(FN+TP)(FN+TN\right)}}$$ (34)

The benchmark dataset collected contained a comparable number of positives and negatives. Specifically, positive samples were 11461 and negative samples were 12000. The values of accuracy parameters obtained as result of the self—consistency test were *TP* = 11461, *FN* = 12, *FP* = 0 and *TN* = 11988. After putting these values in above equations the following values are yielded, Sn = 0.9989, Sp = 1, Acc = 0.9994 and MCC = 0.9989. As all these metrics are nearing 1 therefore it is inferred that the proposed model is highly accurate.

Furthermore to prove the effectiveness of the predictor and to highlight the improvement it offers, its predictive response for self-consistency test is compared with existing ones. Table 1 shows that the proposed model exhibit a higher accuracy rate than any of the existing predictors.

**Table 1:** Comparison of accuracy metrics.

Predictor	ACC (%)	MCC	SN (%)	SP (%)	ROC
**Proposed N-linked**	99.9	0.99	99.8	99.9	0.99
**Ensemble SVM**	95.0	0.84	98.0	77.0	0.91
**GPP**	92.8	0.85	96.0	91.0	-
**GlycoEP**	84.2	0.54	98.1	77.0	0.93
**GlycoMine**	94.0	0.88	92.7	95.0	0.97

The comparison of accuracy metrics of proposed and existing predictors Is illustrated.

### Validation of the model

**Fig 10:**
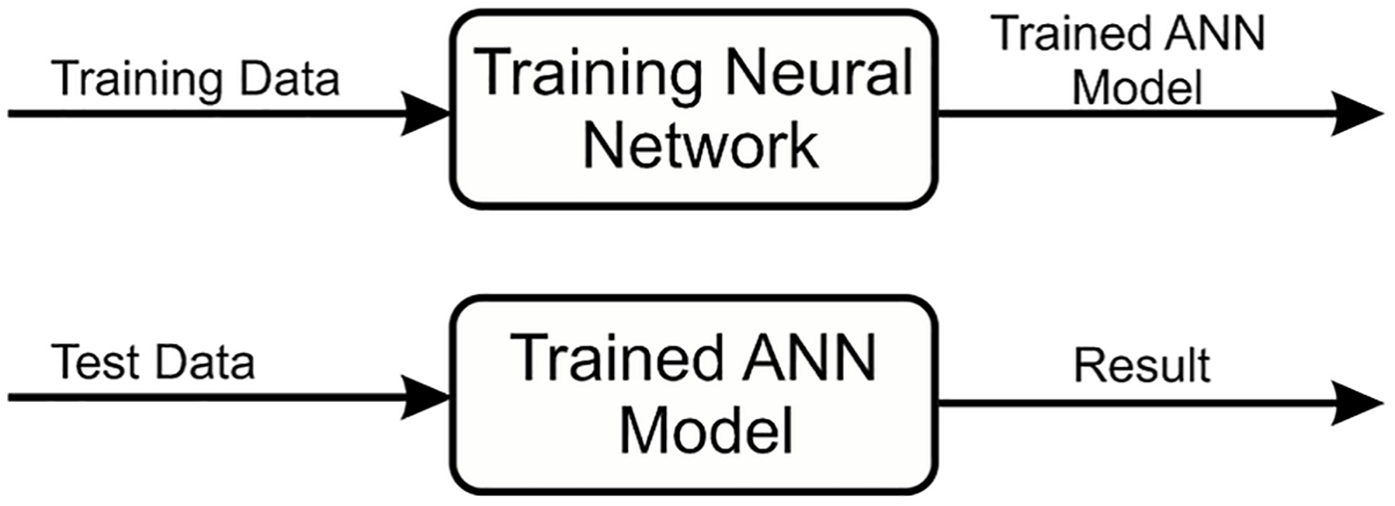
The validation of prediction model. The validation process is illustrated. The accuracy of trained model is verified by testing it over partitioned test data.

Several methods are used to validate a prediction model [8 & 9]. Some of the most reliable method used for the validation of a predictive model is K-fold cross validation and Jackknife testing. In a typical validation method the dataset is divided into two sets, i.e. training data and test data. The predictor is trained using training data. Once the model is fully trained and convergence is achieved then the accuracy of the trained model is tested on untrained data (previously partitioned test data) as shown in the Fig 10. Each method of the validation has a different approach for the selection of training and test data. Cross-validation and jackknife testing are rigorous testing and data partitioning techniques which aim to exhaustively determine the performance of the predictor [27,28,29].

#### K-fold cross validation

**Fig 11:**
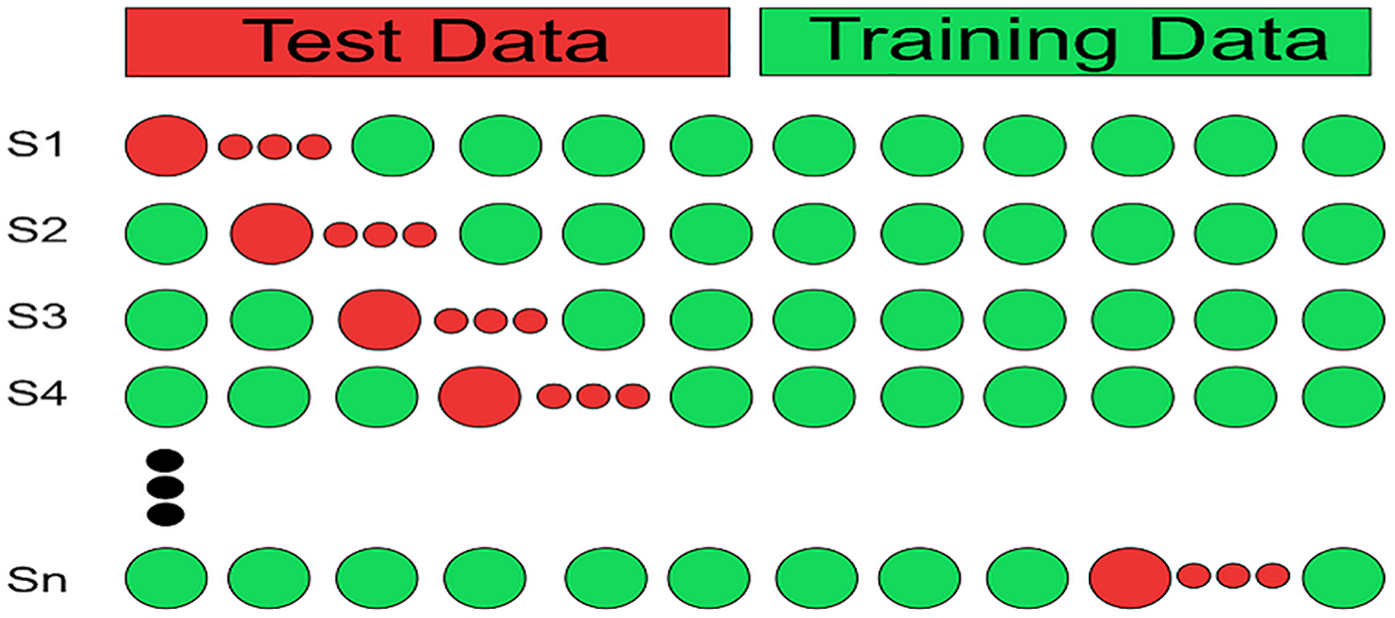
K-fold cross validation. The process of K-fold cross validation is shown. Red circles show the test data and green circles show the training data.

Cross-validation is a way to develop an expectation that the proposed method is perfect or more acceptable when an obvious validation set is not available. Available data is split into *k*-folds where *k* is some constant. All the partitions are disjoint. The system is tested for each partition while it has been trained for the rest of the data. The test is iterated *k* times for each partition as shown in Fig 11. The overall average of the accuracy in each iteration is reported as the cross-validation result.

Formally, let *S* be the total population of samples containing positive and negative samples given as:

$$S=\{s_{1,}s_{2,}s_{3,}\ldots s_{n}\}$$ (35)

Where *s*_*i*_ is any arbitrary positive or negative sample. The dataset is split into *k* comparable size subsets *S*_*i*_ such that

$${⋃}_{i=1}^{k}S_{i}=S$$ (36)

And

$${⋂}_{i=1}^{k}S_{i}=\emptyset$$ (37)

Also the subsets are selected randomly such that their sizes are comparable i.e.

$$\left|S_{i}|≅|S_{j}\right|$$ (38)

Where *S*_*i*_ and *S*_*j*_ are any distinct arbitrary sets. In a single iteration the elements of set *S*_*i*_ are left out and the model is trained on rest of the data. The trained model is used to test the left out data and an accuracy rate *R*_*i*_ is computed. The overall cross-validation result *R*_*a*_ is computed by taking the mean of outcomes for all the *k* iterations

$$R_{a}=\frac{\sum_{i=1}^{k}R_{i}}{k}$$ (39)

In this study, 10 fold cross validation has been performed separately for positive and negative sites. Initially, 10-fold cross validation is performed on negative sites wherein the data is divided into training data and test data. For dataset is partitioned into 10 folds, in each iteration a partition is left out as test data while the neural network is trained on the remaining. After sufficient training the network is simulated to check its accuracy on test data. This process is repeated on all the ten datasets for positive and negative glycosylation sites. The average of these values describe the prediction accuracy of the model which is ultimately computed as 99.998% and 99.81% for negative and positive sites respectively Table 2.

**Table 2:** Cross validation result.

10-fold CV	Positive	Negative
**K1**	99.92	99.92
**K2**	99.75	99.92
**K3**	99.75	100.00
**K4**	99.58	99.92
**K5**	99.75	99.92
**K6**	99.83	99.83
**K7**	99.92	100.00
**K8**	100.00	99.83
**K9**	99.75	99.83
**K10**	99.83	99.83
**Avg.**	99.81	99.9
**Total Avg.**	99.9	

The 10-fold cross validation of proposed predictor for both positive and negative sites are listed.

The accuracy of the predictive model is 99.9% by aggregating the positive and negative results of 10-fold cross validation. Some of the existing glycosylation prediction models have also used the cross validation approach to define accuracy of their proposed model [9, 11, 31, 35]. A comparison of their outcomes based on the cross validation test along with the result of the proposed model is depicted in Table 3.

**Table 3:** Comparison of cross validation.

Predictor	Cross-Validation (%)
**Proposed N-linked**	99.9
**Ensemble SVM**	98.0
**GPP**	88.0
**GlycoEP**	93.0
**GlycoMine**	97.0

A comparison of 10-fold cross validation of proposed and existing predictors is illustrated.

#### Jackknife testing

Cross validation works well if the data is diverse and unbiased. Some researchers have used jackknife testing to validate their results [36, 37, 38]. Jackknife testing is one of the most commonly used and mature re-sampling technique. Other validating techniques use randomly selected or partitioned dataset for testing the predictor. Usually there is no rule that governs the partitioning of this data [39]. The data can be partitioned in many different ways, hence it is possible that a certain partition may produce good results while another partition may not behave likewise. In such sub sampling technique very small selection is used for testing and different selection may produce entirely different results. Therefore, such methods may never produce unique results. The strength of jackknifing lies in its ability to produce unique results [40]. The jackknife method computes the overall accuracy of the predictor by thoroughly leaving out each observation from a dataset and training the model on left out data [41]. Ultimately, all these calculations are averaged. The output of this validation is unique for the provided dataset which consequently mitigates the issues raised by data independency and sub sampling. Considering that *X* is the total sample space having *n* elements, given as

$$X=\{x_{1},x_{1},x_{3},\ldots x_{n}\}$$ (40)

**Fig 12:**
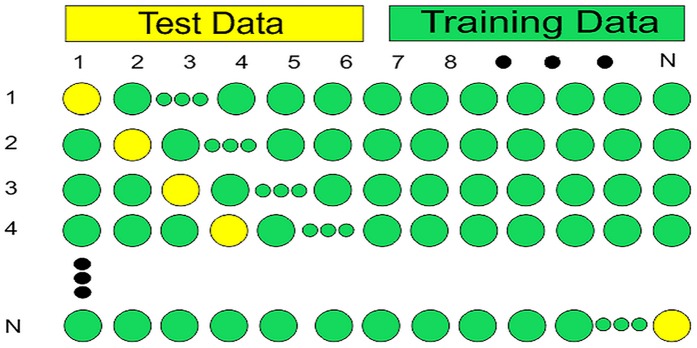
The process of Jackknife validation. The jackknife validation is shown in which yellow circles show the test data and green circles shows the training data.

Jackknife testing is an iterative method that computes the accuracy of the predictor for all permutations of the population of size *n-1* as shown in Fig 12 [32, 42, 43, 44].

Let *A*_*i*_ be the accuracy rate computed for the *i*^*th*^ iteration of the jackknife test. The data set used to compute *A*_*i*_ leaves out the *i*^*th*^ element in the population within the dataset *X*_*i*_ given as

$$X_{i}=\{x_{1},x_{1},x_{3},\ldots ,x_{i-1},x_{i+1},x_{n}\}$$ (41)

The trained neural network is simulated with the feature vector of all the samples in *X*_*i*_. The number of false positive and negatives and true positives and negatives is used to compute the accuracy for this permutation *A*_*i*_. The mean of all the values of *Ai* is computed as A*where.

$$A^{*}=\frac{1}{n}\sum_{i=1}^{n}A_{i}$$ (42)

A* represents the overall average accuracy of the predictor and *n* represents the number of observations. The dataset used in this study is too large as described earlier, therefore an estimation of jackknife test is computed using a random selection of data containing 100 samples of both positive and negative sites. In each iteration an item is left out of the training set and the outcome of the predictor is observed for the left out item. The process iterates for all the selected dataset, after aggregating the prediction results it yields an accuracy of 99.84% and 99.78% for negative and positive sites respectively.

The significance of the N-linked glycosylation has been emphasized in various existing studies. Researchers have proposed various computational approaches in order to identify N-linked glycosylation sites. The authors of each era put their best effort to enhance the prediction accuracy and to identify N-linked glycosylation site within glycoprotein sequences. In this study, we focus to achieve maximum accuracy by overcoming drawbacks in the existing methodologies. Several key features make proposed approach distinguished and more accurate from existing ones. Firstly, the benchmark dataset compiled is up-to-date and balanced dataset as only experimentally annotations have been included. Secondly the data is non redundant and is comprehensive and conclusive in size. Furthermore, the data is diverse in nature as it primary sequences originate from diverse organisms. Most importantly the feature extraction technique is scale and position variant and is capable of rigorously extracting deep obscure patterns. Additionally, exhaustive 10 fold cross validation and jackknife testing is performed to evaluate the predictive performance of the model [45, 46]. Existing methodologies described earlier have different loopholes in their approaches. In [12] the authors tried to convert unbalanced dataset (unbalanced ratio of positive and negative sample) into the balanced dataset by truncating significant data elements which resulted in an insufficient data set for mining diverse patterns. The dataset used by the author in [16] only consists of human proteome, hence this dataset tends to leave out essential patterns crucial for classification decision. Similarly the feature selection approach used by [14] does not extract the crucial details and also dataset is outdated. In this study, non-redundant, verified, reviewed and updated dataset of huge size has been used and also extensive features have been extracted. The initial experiments were conducted using a smaller feature vector. Through constant probing and experimentation the feature set was expanded until most accurate results were achieved. The design of feature sets aimed at uncovering deep obscure patterns, regarding position and composition the utmost importance. Along with this, various accuracy metrics have been computed and compared with existing model as shown in Table 1. The accuracy of the model was verified and validated by performing rigorous 10 fold cross validation as shown Table 2 and jackknife testing.

## Conclusion

Several protein functions are dependent on the glycosylation process, which is one of the most complex post-translational modifications. Any anomaly in N-linked glycosylation may result in problems in proper functioning of cell, sometimes leading to cell death. The understanding and the knowledge of N-linked glycosylation sites can help in numerous ways. The distinct function of such modified proteins, mainly depends upon structural features along with the type and details of attached carbohydrate moieties. There are many impediments, on mining such information during biochemical analysis, including small sample size, efficiency of detection, separation and analysis of vast structural heterogeneity of carbohydrates. In this study, a machine learning model using the back propagation methodology is developed for the identification of N-linked glycosylation sites. The feature vector is formed by combining different approaches, including position and composition variant features, raw moments, Hahn moments and central moments. The results yielded by the trained model are then validated using cross-validation, jackknife testing and self-consistency testing. It shows that the proposed model outperforms existing models such as Hamby random forest, GlycoMine and GlycoEP. Furthermore, the accuracy of the model is illustrated using different benchmark accuracy metrics such as Matthew Correlation Coefficient, sensitivity, specificity and accuracy. It is demonstrated with overwhelming experimental results that the proposed computational method provides an accurate cost and time effective approach as compared to existing in silico and in vitro methods.

## Supporting information

S1 File Sequences of negative N-linked sites. This file contain negative n-linked sites of glycosylation along with the accession number.
(PDF)

S2 File Sequences of postive N-linked sites. This file contain positive n-linked sites of glycosylation along with the accession number.
(PDF)
