# Supplementary material for: Prediction of N-linked glycosylation sites using position relative features and statistical moments
Source: PLoS One. 2017 Aug 10;12(8):e0181966. doi: 10.1371/journal.pone.0181966 (PMC5552137; doi:10.1371/journal.pone.0181966)
Supplement: S1 File — This file contain negative n-linked sites of glycosylation along with the accession number. (PDF) [file pone.0181966.s001.pdf]

Supplementary Data Set (S1) of Negative N-Linked Site

**Accession No**	**List of Sequences for Negative N-Linked Sites**
P62593	KVKDAEDQLGARVGYIELDLNSGKILESFRPEERFPMMSTF
Q17QI8	YPDTDVFLACFSVVQPSSFQNITEKWLPEIRTHNPQAPVLL
P52843	VPIWNRSPWIETDIGYSALINKEGPRLITSHLPIHLFSKSF
Q3LRV9	VLTTSPEETTDSSINGTGHRNQSSSSPGWTSPGLLVSVQYG
Q13145	ARNHSGTTIPTLECCHEDMCNYRGLHDVLSPPRGEASGQGN
Q9BUP3	DRDYVLKSAELAKAGGCKHFNLLSSKGADKSSNFLYLQVKG
Q8VCR9	DLGPRLNPVPDYELKYPPVTNRRDRSRYAAVFQDQYGEFSE
Q9NZQ0	TIGIDYGVTKVHVRDREIKVNIFDMAGHPFFYEVRNEFYKD
Q8VBX0	HVACAREHLDCVKVLLNAGANVNAAKLHETALHHAAKVKNV
Q9CQK7	FTYSEKYPDETPLYEIFSQENLEDNDVSDILKLLALQAEEN
Q9CYZ2	KLGDMSSYSIRHSISMPVMRNSATFKSFEDRVGTIKSKVVG
Q58DW4	DKHMNLILCDCDEFRKIKPKNSKQAEREEKRVLGLVLLRGE
Q8R197	ICFPWAGGGSTHFAKWGRKINGLLEVHAVRLAGRETRFEEP
O55101	VDPTPDPNTAYASYPSASVENYQQPPFTQNVETTEGYQPPP
O43752	LRSIEWDLEDLDETISIVEANPRKFNLDATELSIRKAFITS
Q08DY9	KNFHENTGMACRSGTDVDAANLRETFMNLKYEVRIKNDLTC
P18181	SNVRKEDKGTYYMRVLRETENELKITLEVFDPVPKPSIEIN
Q9TU03	TEKAPEPHVEEDDDELDGKLNYKPPPQKSLKELQEMDKDDE
Q96DR5	QKSSAWQLAKQKAQEAEKLLNNVISKLLPTNTDIFGLKISN
P55789	RNHPDTRTRACFTQWLCHLHNEVNRKLGKPDFDCSKVDERW
Q9CQF3	QDGVLQDWVIDDCIGNWWRPNFEPPQYPYIPAHITKPKEHK
Q8NB37	SESKPICAVGHGVAALCCATNEDRSWVFDSYSLTGPSVCEL
P12401	ELRNMKNLSEAVISSAMEIENMSEKLQAFIESQFRKIIVPV
Q9Y2R9	AEEQATIERNPYTIFHQALKNCEPMIGLVPILKGGRFYQVP
Q3UNB8	ITLELRRAPCIWKVRAMMISNTFNLILGFVAVVIEVMKTAL
Q1LZ95	SIWGEHEIDYILLVKKNVTLNPDPNEIKSYCYVTKEELEEL
Q96DC9	SFNLISEKCDILSILRDHPENRIYRRKIEELSKRFTAIRKT
O88822	PATWPEDNIIRQTISLLIVTNLGAYILYFFCATLSYYFVYD
Q9CWQ3	RHLACSEIRAASLSGDCSLVNELFRLRFGLKQHHQIETSCV
Q13326	NSEGEVTGRLKVGPKMVEVQNQQFQINSNDGKPLFTVDEKE
P05937	SGFIETEELKNFLKDLLEKANKTVDDTKLAEYTDLMLKLFD
P12004	ARLVQGSILKKVLEALKDLINEACWDISSSGVNLQSMDSSH
Q08DU1	LLSMVEDGNPHHYFLATQDQNLSMKVKKKPGIPLMFIIQNT
Q0VCP1	AVILGPPGSGKGTVCQRIAQNFGLQHLSSGHFLRENIKANT
P61106	SWLTDARNLTNPNTVIILIGNKADLEAQRDVTYEEAKQFAE
Q5IBH6	AGLFSWQYRPIHFFLALYLANDMEEDNQAPKQDIFYFLYGK
Q9HAP6	PGGVADRHGGLKRGDQLLSVNGVSVEGEQHEKAVELLKAAQ
Q2KIR7	KSLRKSLPMSLKVYGTVMHMNHGNPFNLKALVDKWPDFQTV

Q9HBL8	QGAEVVQGDQDDQVIMELALNGAYATFIVTNYWESCSQEQE
Q9QWG7	LDEEALDRIVHHTSFEMMKENPLVNYTHLPTAMMDHSKSPF
Q8TDU5	LFESWNIQNNDLKCKIITFLNRVMRGVSICTTCLLSVLQAI
Q8VBT0	LWAVPGAHGRRNNVRVLTDENWTSLLEGEWMIEFYAPWCPA
O43704	MKENPKEEIKKIIRFLEKNLNDEILDRIIHHTSFEVMKDNP
Q5E9L0	AGASLCLIGGVIFCFSISDNNKAPRMGYTYNGATSVMSSRT
Q96CT7	RAAFTAFEEAQLPRLKQENPNMRLSQLKQLLKKEWLRSPDN
P82348	LYAKEIRSRVDSSLLLQSTQNVTVSARNSEGEVTGRVKVGA
Q91ZT9	KEDSCFELLHRAVGQFELRKNGIMPREVTKDQQLCEKLTVL
Q5KR47	KLEEAEKAADESERGMKVIENRALKDEEKMELQEIQLKEAK
Q96EY4	REAHKQEKKEKLKNEKALRLNLVGEKLQWFQNHLDPQKKRY
Q9Y296	GQRDGIRVGHAVLAINGMDVNGRYTADGKEVLEYLGNPANY
Q8BVZ5	LSTYNDQSVSFVLENGCYVINVDDSGKDQEQDQVLLRYYES
P15949	QHRLVSKSFLHPGYNRSLHRNHIRHPEYDYSNDLMLLRLSK
Q4JM65	TWFQNQRMKCKKWQKNNWPRNSNGMPQGPAMAEYPGFYSYH
Q9Y624	SLALGSVTVHSSEPEVRIPENNPVKLSCAYSGFSSPRVEWK
P50876	CPKCKVYIERDEGCAQMMCKNCKHAFCWYCLESLDDDFLLI
Q9BVC5	VFDGSSTSTSIKVKKTENGDNDRLKPPPQASFTSNAFRKLS
Q0VD59	DATSARVYRSKTKEKEREEQNEKTLGHSMSHSSNISKAGGS
P33792	ISAEPADIIISSGEQVKIKINKNYTPVGGDRIFGINFSPDT
O89116	LEAGYQIAVETEQIGQEMLENLSHDREKIQRARDRLRDADA
Q3ZBK3	KEEERMVEMFLEYQNQICRQNKLIQEKKENLLKLIAEVKDK
Q8NET5	RLAGGQSVTHTGLPIMASLANTAISFSCRITYPYTPQFKVF
Q3KPI0	SVVLTCHTNNTGTSFQWIFNNQRLQVTKRMKLSWFNHMLTI
P04229	NSQKDLLEQRRAAVDTYCRHNYGVGESFTVQRRVEPKVTVY
Q2KIU7	MDSLEHDIPKGSWSVQMERGNALVVLRSLLWPGLTFYHAPR
P20827	SLAAADRHTVFWNSSNPKFRNEDYTIHVQLNDYVDIICPHY
Q9H741	SLVYFLSSTFKQEERAVRDRNLLQVHDHNQPIPWKVQFNLG
Q5E9I1	LQLYYSLLQENVPHERKSSLNFERLEAQLKACYCRIIFSKA
P00416	LLASGVSITWAHHSLMEGKRNHMNQALLITIMLGLYFTILQ
P13135	SGTIGSSELPGAFEAAGFRLNEHLYNMIIRRYSDEGGNMDF
Q2TBU3	GMLKSIQSKMNTLANRFPAVNSLIQRINLRKRRDSLILGGV
Q8IWD4	DDDCPVRKKRITEAELCAGPNDWILCAHQDVEGHGVNPSVS
Q53R12	LMAIPATTMSLTARKRACCNNRTGMFLSSLFSVITVIGALY
Q3SZW3	FIGIFAFLWVERPIRAKEKLNFTLLLKALLLSSYGKLLLIP
Q9NVZ3	ESVLCVKPDVHVYRIPPRATNRGYRAAEWQLDQPSWSGRLR
Q9WTP6	VSDEMVVELIEKNLETPSCKNGFLLDGFPRTVRQAEMLDDL
P43407	EEKLGPAIKSTDVYTEKHSDNLFKRTEVLAAVIAGGVIGFL
A7E2S9	TMDLNIRDAKKRTALYWACANGHAEVVTLLVDRKCQLDVLD
Q9H9Q4	AKELNKRLTAPPAAFLCHLDNLLRPLLKDAAHPSEATFSCD
Q9UJQ1	TCLMAEFAAKFIVPYDVWASNYVDLITEQADIALTRGAEVK

P07628	PGFNMSLLTLKEIPPGADFSNDLMLLRLSKPADITDAVKPI
P56857	NVAYRPGGFKASTGFGSNTRNKKIYDGGARTEDDEQSHPTK
Q9CR58	RDGRCAGYKGTLDCLLQTWKNEGFFALYKGFWPNWLRLGPW
Q60660	SKEESQQIGDRPSKLDSAARNSVPNRQKCAYLSSFSTEEDD
P0DMM9	SPFMRKGMAGDWKTTFTVAQNERFDADYAEKMAGCSLSFRS
Q9P0G3	LLLTALQVLAIAMTQSQEDENKIIGGHTCTRSSQPWQAALL
Q60961	AVNIQYEVIGNYYSSERMADNACVLFAVSVLMFIISSMLVY
P25732	EVYRVYFEAVKPDSKENVIDNKKLTTELSVNIIYAALIRSL
Q9Y2V0	SFGDECSHHAYLHDQFWSYWNRFGPGLVIYWYGFIQELDCN
P50194	AVDFIHYDEKNNEWNLLQVKNRDNTENSSSSKIRDNTTIKK
B9A014	YLNPLDSAWSSLKWFIINNRNEFCLQSIDSGYSYQCILFSN
Q8C767	GMVAPAVQEKKVKKRVSFADNQGLALTMVKVFSEFDDPLDI
Q9NQ31	GPGGSYQISEHAPEASQPAENISKDLYIEVYPGTYSVTVGS
P29084	KKTKVEHGGSSGSKQNSDHSNGSFNLKALSGSSGYKFGVLA
Q9CQT2	KLKQFAFVNFKHEVSVPYAMNLLNGIKLFGRPIKIQFRSGS
Q3TCJ8	KEGRILEGRHEEAGQVPQTSNAQEKVSLSDCIQEAKASLQN
P22005	EGDTLANSSDLLKELLGTGDNRAKDSHQQESTNNDEDMSKR
Q921N7	VRCFHTQVDKPENGRLIYTGNLARTIFGVKCFSYSTSVVSL
Q9P015	AKVNIEVQLASELAIAAIEKNGGVVTTAFYDPRSLDIVCKP
Q9P299	LAKYYDDTFPSMKEQMVFEKNVFNKTSRTESEIAFFGGMTI
Q9BZJ3	SETFPPGMPCWVTGWGDVDNNVHLPPPYPLKEVEVPVVENH
Q810M6	DILNPHKDHGLRAEPRDKEENRQTLPYSYSINHPCFAEIED
O43399	TSAALSTVGSAISRKLGDMRNSATFKSFEDRVGTIKSKVVG
P97461	VAETPDIKLFGKWSTDDVQINDISLQDYIAVKEKYAKYLPH
P50592	KTLGQKIESWESSRKGHSFLNHVLFRNGELVIEQEGLYYIY
Q9BXT2	TLAVLSVGTEFWVELNTYKANGSAVCEAAHLGLWKACTKRL
O75558	VSGDQIEDMFEQGKWDVFSENLLADVKGARAALNEIESRHR
A6QNL6	CVLERQIFDFLGYQWAPILANFVHIIIVILGLFGTIQYRPR
Q9D8X5	VRPIGEFRSSIDYQYQLLRCNVDLLKIIQLGLTFTNEKGEY
Q8R0W6	RDEDFVGRDDFDDTDQLRIGNDGIFMLTFFMAFLFNWIGFF
Q8BRU4	HNWIQNGKSCYYVFERWEMWNISKKSCLKEGASLFQIDSKE
Q3UGC7	RLKKLQEESDLELAKETFGVNNTVYGIDAMNPSSRDDFTEF
Q8K2J7	INENSDTVGQIVQYIMKNEANADILKAMVADNSVGDIESPV
P0CH99	LLTHSGKKPCVSKQCGKSLRNLLSPKPRKQIHTKGKSYQCN
Q9QZN4	RNLLRNPCAEENLSSWRIDSNGGDRWKVETLPGSCGTSFPD
Q8BTF8	AQKKQLEESIELIQDECVSENADHSTEEPAEGGQEADGGEM
Q8TCD6	DVAMPRKGYTLQKTLSRMSQNLEPMEYSVVVWSSGVDIISH
P01236	CQVTLRDLFDRAVVLSHYIHNLSSEMFSEFDKRYTHGRGFI
P31041	AGVLFCYGLLVTVALCVIWTNSRRNRLLQSDYMNMTPRRPG
Q13432	FTVGDKPVNNFRMIERHYFRNQLLKSFDFHFGFCIPSSKNT
Q0P565	VKQLDQCEMILQASEYEDLENKPGRLQDFYDSTAGKFSHPE

Q08DE6	GPNGTAGIFATYPSGHLDMVNGFFDQFIGTASLIVCVLAIV
P42216	ESGTDRLVEVMHKVEADIYINLQGDEPMIRPRDVETLLQGM
Q6PE84	ILVLPCIDVFVKVDLRTVTCNIPPQEILTRDSVTTQVDGVV
Q5GJ75	KDLVHELVQRHLTPRTHGRINHVFNHFADVEFLSTLYSLDG
Q3KNI6	GPPKAKESKAIPEPRSDESENIWNPEEVPEGAEHDDIWDVR
Q2KIN3	SYCSNWRCPTRVQEDQQRTINIKCGGLREFSQRVFCHGAPP
Q1LZ96	DTDTVCYRVEEPETLVELQRNEWDPVISWAEKRYGVEIGSS
Q8IWL3	PTGVPRRRPLSCDAASQAGSNYPRCWNCGGPWGPGREDRFF
Q9QZL9	LPLAFVSSSALPIHDVDSQQNTSGFLGLQRLLQSFSRLFLK
O15194	ELSKVIIVDNSPASYIFHPENAVPVQSWFDDMTDTELLDLI
Q9UBQ5	AKENAYDLEANLAVLKLYQFNPAFFQTTVTAQILLKALTNL
Q9WTY4	AGAGILYWLAPGNARGNLAVNALSNNTTPGKAVVVELILTF
A6NJY1	CLSIVFYSGGMINNAIASLRNVCISLLAGIVLGFFVRYFPS
Q99NH8	PCQRVVSTHGVWLLAFLKKRNGSTVIADDTLAGTVTITLKN
Q1JQ93	REKKLGLGTAYIHGMKHATGNYIIIMDADLSHHPKFIPEFI
P28070	GGVVIAADMLGSYGSLARFRNISRIMRVNNSTMLGASGDYA
Q9Y624	QDGSPPSEYTWFKDGIVMPTNPKSTRAFSNSSYVLNPTTGE
Q66JT5	LEKPHFDCRSAIFELDSCNGNGKVCLVYKNGKPGLAHDTEI
P20108	IALRGLFIIDPNGVVKHLSVNDLPVGRSVEETLRLVKAFQF
Q2KIE2	RDEDEDDEEEEEEGVTWGRGNSRFEGPQSPEEFSFGFSFSP
Q9UFF9	ILNLFFPSIYDVKYLMKSCKNLKGGLQEVADQLDLQRIGRQ
P14207	LLLLVCVATMCSAQDRTDLLNVCMDAKHHKTKPGPEDKLHD
Q9H2P9	THSDLVLRATKLGIPYRVIHNASIMNAVGCCGLQLYKFGET
Q9P0W0	QNLRHLSSMSNSFPVECLRENIAFELPQEFLQYTQPMKRDI
Q8BGS0	FEDMDKLNTDSEEDQDDESSNDEEAHKAKHKGKAPLKGPLR
O75934	QLEHQAVRIENLELMSQHGCNAWKVYNENLVHMIEHAQKEL
P38573	AFRTENSDADYPWPRQCCVMNSLKEPLNLDACKLGVPGYYH
Q9ERQ8	PDGLAVVGVFLETGDEHPSMNRLTDALYMVRFKDTKAQFSC
Q91VH6	EHLDKMGMSIIEQLDPVSFSNYLKKYHNTICGRHPIGVLLN
P0DMB1	DDFSLEFHAQDGDISDMRRENVPFSPAEEGKAAPLYQQPLM
Q9WU03	EECLDKCAGVTENTTDDMARNRNGADSSVLSVPRKQSAEDL
P08865	LDFQMEQYIYKRKSDGIYIINLKRTWEKLLLAARAIVAIEN
Q71SS4	SFWFGTRFKACYLPWVILGFNYIIGGSVINELIGNLVGHLY
P29020	VLEKKRARDPEMGGKQQRRRNPQEGVYNALQKDKMAEAYSE
P97371	HPEAQAKVDVFREDLCSKTENLLGSYFPKKISELDAFLKEP
A6H7B8	WNHYLSCTSPCAGYRPLCRLNFGLNVVENVALLVLTYVSSS
Q8VCC1	TYLGLDYMSKQNGGEGGIIINMSSLAGLMPVAQQPVYCASK
O43633	LLFGRRKTPEELLRQNQRALNRAMRELDRERQKLETQEKKI
Q9D3X5	SLTSSPEAARRARPRPSDKLNPKTINPFGEQPRAPTAFAAI
Q32L77	VHRKCRSQFTDLDGSKRVGINTWHDESGIYANSYVKRKLYS
P08217	GDPTYPPYVTRVVGGEEARPNSWPWQVSLQYSSNGKWYHTC

Q9H560	LFAINSRRQQIVEFLLKNQANLHAIDNFRRTALMLAVQHNS
A5PJN0	RIWRKYDADSSGFISAAELCNFLRDLFLHHKKAISEAKLEE
P81125	KIEEACEIYARAANMFKMAKNWSAAGSAFCQAAHVHLQLQS
P23184	REQWKCDGVFVSHIIDIKDNNINVSDSTLIWLHLENYHSDI
Q8C8M1	LKTTLKPKKVKTLSGNRMKSNQISKLQKEFKRHNSDAHSTT
P47803	LEVLAVGTVLLVEALSGLSLNILTILSFCKTPELRTPSHLL
Q56P03	CLDCQRHESYKTQYRAMFVMNCSINKEEVLRYKASENRKKR
A0PG75	LIIIHQQVELLGMILGTETSNKYEIKNSLGQRIYFAVEESI
O60248	RPMNAFMVWSSAQRRQMAQQNPKMHNSEISKRLGAQWKLLD
O43763	RAALAKALRMTDAQVKTWFQNRRTKWRRQTAEEREAERHRA
P0DML2	EDGSRRTGQILKQTYSKFDTNSHNHDALLKNYGLLYCFRKD
P0CI00	HTGERPYKCHQCGKAFIQSFNLRRHERTHLGQKCYECDKSG
Q5T6M2	AVPVPHFTTWGSLIPIDSQRNKERTRFTWMDGPPHGGLGTR
Q9WV35	HAEEAFFNTILPAFDPALKYNVTWYVSSSPCAACADRILKT
Q6UXX9	KRASYVSNPICKGCLSCSKDNGCSRCQQKLFFFLRREGMRQ
A6NKC4	LALRCHAWKDKLVYNVLYYRNGKAFKFFHWNSNLTILKTNI
P57796	SRKRTGSSGEQTGPEAPGSSNNPPSTGEGPAGAPPASPGPA
P62258	DTLSEESYKDSTLIMQLLRDNLTLWTSDMQGDGEEQNKEAL
Q9CY64	ISIHFKSGSLEEVPNVGVNKNIFLKDQDIFIQKLLGQVSAE
Q6BCY4	LQDPEAKYPLPLIEKEKISHNTRRFRFGLPSPDHVLGLPVG
O14593	AADGSDTVVLSLFPCTPEPVNPEPDASVSSPQAGSSLKHST
Q9BZM4	PYLLFDWSGTGRADAHSLWYNFTIIHLPRHGQQWCEVQSQV
Q9BUJ0	YGELDHILARESLRQLRHLPNHSVVKLRNAGHACYLHKPQD
Q3SYR2	PEDVLGLQRITGDYLCSPEENIYKIDFVRFKIRDMDSGTVL
Q8TCD6	SNGHLTVENYHTHSCNRCPKNLCKKVVLIEFVDKQLQQGVN
Q8IUW5	RCTTEAEQDIEEEKVEKIELNDSVNENSDTVGQIVHYIMKN
Q9DCH6	QETNHSQAPMLCSTGCGFYGNPRTNGMCSVCYKEHLQRQNS
Q9QXN5	LQDPRYSTELGMYQPHCGLENVLMSWGHDEYLYQMMKFNKF
Q8BFY6	SVDADHSGYISLKELKQALVNSNWSSFNDETCLMMINMFDK
P21845	NDEPLPPPYPLKQVKVPIVENSLCDRKYHTGLYTGDDFPIV
O43396	LTEDDIKEDGIVPLRYVKFQNVNSVTIFVQSNQGEEETTRI
O08547	SKANNLSSLSKKYRQDAKYLNMRSTYAKLAAVAVFFIMLIV
O14817	AATQGSFATLSSSFPSLSAANLLIITGAFVMAIGFVGCLGA
P63101	RYDDMAACMKSVTEQGAELSNEERNLLSVAYKNVVGARRSS
Q5PR19	PLETQGNPTSPRYNPRPLEGNVQLKSLTENNQTDKAQVHAV
Q9D563	SSHSGLWRTCRVQSSCTPLMNPFWQENVTVSDSSRQLLTMH
Q8BWR2	EERTDRSKFVESDADEELLFNIPFTGNVKLKGVIIMGEDDD
Q14088	SSLDQYVQIRIFKIIVIGDSNVGKTCLTFRFCGGTFPDKTE
P38447	NRSSCDFHEDDSVHAYHRATNADYRGSGFDRGHLAAAANHR
Q2KIR1	AMPPQQLMPGQMPPAQPLSENPPNHILFLTNLPEETNELML
Q4JM65	MQELSNILNLSYKQVKTWFQNQRMKCKKWQKNNWPRNSNGM

Q6P073	KLIFPREFPFKPPSIYMITPNGRFKCNTRLCLSITDFHPDT
Q5E9I1	LGCVGLSCFYLAVKSTEEERNVPLATDLIRISQYRFTVSDL
P20340	GEQSVGKTSLITRFMYDSFDNTYQATIGIDFLSKTMYLEDR
Q92629	PTRSLVMEAPKGVEINAEAGNMEATCRTELRLESKDGEIKL
P08637	PKATLKDSGSYFCRGLFGSKNVSSETVNITITQGLAVSTIS
Q6ZN79	KCYECDKSGKAFSQSSGFRGNKIIHTGEKPHACLLCGKAFS
Q8R2U4	IPPTVDGMLGGYGHISNIDLNSSRKFLQRFLREGPNKTGTS
P00642	FLTENISITRPDGRVVNLEYNSGILNRLDRLTAANYGMPIN
O60762	RELEVRSPRQNKYSVLLPTYNERENLPLIVWLLVKSFSESG
Q3T0F5	KVLLKVIILGDSGVGKTSLMNQYVNKKFSNQYKATIGADFL
Q9BRJ7	VRVPLYTQKDRVGGFPNFLSNAFVSTAKCQLLFALKVLNMM
P57776	PGGDHSELIVRITSLEVENQNLRGVVQDLQQAISKLEARLS
Q0IIC4	SSEVGQRRTRKEGNEYKMMPNRRNMYAVQNNSAGPYFNPRS
Q3TQI7	QEEQKAKPKNAEDCLYELPENIRVSSAKKTEEMLSNQMLSG
Q7L2Z9	QILASEVEEEEERVKQMHQINSSGVLSLPELSQKTLKAPTL
Q9QZM8	LRVRLLDEYENEVVKFSASPNPVLQWTERSCRQVSHVFTNF
Q810M6	SYSINHPCFAEIEDTLSSQINESLRWDGILTDPEAEKERIR
P05231	AITTPDPTTNASLLTKLQAQNQWLQDMTTHLILRSFKEFLQ
Q9TVC8	GLCYFTNGTERVRYVTRYIYNQEENVRFDSDWDEYRAVTPL
Q8IYI0	RGQESQKYALRSFQMARVIFNRDGCSVLQRHSRDTHFYPLE
Q9NQY0	AKLHQAREELRPVREDFEAKNRQLLEEMPRFYGSRLDYFQP
Q9UBY8	TCVSWMLLKAGWSESLFWKLNQWLMIHMFHCRMVLTYHMWW
Q08E20	AETGKCPVLYWLSGLTCTEQNFISKSGYHQAASEHGLVVIA
Q00286	HFGEHSKPSSQEIMRMAEELNLEKEVVRVWFCNRRQREKRV
Q3SWY9	SVVKKVSEESETQPLVALVGNKIDLEHMRTVKPEKHLRFCQ
Q9UNN8	RLHMLQISYFRDPYHVWYQGNASLGGHLTHVLEGPDTNTTI
P57052	ESVSYAIALLNGIRLYGRPINVQYRFGSSRSSEPANQSFES
P53519	FNNIVEKLKWHKNGKYLVLKNNTPYYISFSEVFFDSDKVNN
Q99623	IKLRKIRAAQNISKTIATSQNRIYLTADNLVLNLQDESFTR
P04769	KLGVIMRKEFFMNSFSSETFNKIILDLHKSTENITKAFNSC
Q920C4	PLWGAVFFISSGSLTVAAGRNPTRMLMQNSFGINIASTTIA
Q9MZ13	QLHTHVNDGTEFGGSIYQKVNEKIETSINLAWTAGSNNTRF
Q9HDB8	KEIPKESKNTEVLVWEECVANSAVILQNNEFGTIIDWAPRG
Q8NDB6	CECRYCQSHRPNLSGIPGESNRAPHPSSWETLVQGLSGLTL
Q9P126	LSRQKSNEVWKWEDGSVISENMFEFLEDGKGNMNCAYFHNG
Q9CZJ6	ARCRRPLGDSLTWVASQEDTNCILLRSVSCNVSVDKEPKLS
Q96BQ1	PTKEIQVKKYKCGLIKPCPANYFAFKICSGAANVVGPTMCF
O75360	RHRTTFSPVQLEQLESAFGRNQYPDIWARESLARDTGLSEA
Q2KIN3	YSAAFQIDGHWMHYDGLRNVNLILLNKPPEFLLLSSLVYIR
Q9JHE6	VHTFVSTCQAIDATVSAELLNHLLESMPLREGSSFQDLLGD
Q3SZ18	CVLKGGYKFFADLLDYIKALNRNSDKSIPMTVDFIRLKSYC

P28651	VEWGLVFPDANGEYQSPINLNSREARYDPSLLDVRLSPNYV
Q00187	YWGPIEHEGKGFEQLKAYTANKGRVSAIIQIPALKEETYGR
P59539	SNWPATSLSIFYLLKIANFSNLIFLRLKRRVKSVILVVLLG
Q06323	VHELDEAEYRDIRLMVMEIRNAYAVLYDIILKNFEKLKKPR
Q9ERQ8	YEACMSLSITNNGHSVQVDFNDSDDRTVVSGGPLEGPYRLK
P27792	AFAELRKLLPTHPPDRKLSKNEVLRLAMKYIGFLVRLLRDQ
A0PJX2	RWRYTRLPSQVEDTLSGEEGNEEEEEEEAAPDPAAAPEDPT
P46926	GSSLVSRTRVKTLAMDTILANARFFDGELTKVPTMALTVGV
Q96CS2	DSAVALETKDTSLASFIPAVNDLTSDLFRTKSKSEEIKIEL
Q8SPU8	RERLVATAVKLHGGVDILISNAAVSPFFGSLMDVPEEVWDK
Q14201	CEVCCRYGEKNNAFIVASFENKDENKDEISRKVTRALDKVT
P18121	PENREEVHETKTEDLLKAMINVSISWKEPLKHLVSALTALP
Q58DS9	NWVKELQRQASPNIVIALAGNKADLASKRAVEFQEAQAYAE
Q61451	KLNTLVAEGLNDSIQHYHSDNSTMKAWDFIQTQLQCCGVNG
C9JJ37	VKELRLALLAPAELSALEEQNRQEPLIPVEQIVEAWKCHAL
Q9Y277	ETKYKVCNYGLTFTQKWNTDNTLGTEISWENKLAEGLKLTL
Q96EY4	LNASNLKTFREWDFDLKKLPNIKMRKICANDAIPKTCKRKT
P63046	YRFLPSDLHNGDSKVIYMARNPKDLVVSYYQFHRSLRTMSY
A5PJT0	RSLPEYLYSSGLLRDRAARDNAACRPLLAVATHVHFDHSGG
Q3YBM2	LAVCVLKVIVSLVSLGVGLRNLCGQSSQPLNEEGSEKRLLG
Q32LM6	PGGVAERHGGLKRGDQLLSVNGVSVEGEHHEKAVELLKAAK
Q3MHP2	RWLKELRDHADSNIVIMLVGNKSDLRHLRAVPTDEARAFAE
O00233	MSRKLGQSESQGPPRAFAKVNSISPGSPASIAGLQVDDEIV
Q7Z5A4	CISSRFHYSVKMGDRSVYNENTSVVVSVQRAFVHPKFSTVT
Q9UBS3	FTSGKGQRGSGSSFEQSFNFNFDDLFKDFGFFGQNQNTGSK
P31268	LAKGACDKTDEGALHGAAEANFRIYPWMRSSGPDRKRGRQT
O14893	TVRQNVNKHRSHWKSQQLDSNVTMPKSEDEEGWKKFCLGEK
O95571	TLNPRLTLSCEEFVKIMGNLNLPKPQQIDFAVPANMRCGVQ
Q8BYP3	PSVFEKYTASVTVGNKEVTLNLYDTAGQEDYDRLRPLSYQN
Q9JI59	GTSLLGNPKGGTHNNSSYTMNTKSGILQFNMISKMDSGEYY
P81126	YAAQLEQYQKAIEIFEQIGANTMDNPLLKYSAKDYFFKAAL
Q80WR1	TDVFSATWNSVMITFGCCGVNGPEDFKLASVFRLLTLDTEE
Q9WTN0	YRYLLQLPGKQVRSKLSQAFNHWLKVPEDKLQIIIEVTEML
Q9CZX7	PCNCLLICKDTSRRIGCPRPNCRRIINLGPVMLISEEQPAQ
P07628	TKWQKPDDLQCVFLKLLPIKNCIENHNVKVTDVMLCAGEMS
Q2TBU3	RMFETMAIEIEQLLARLTGINDKMAEYTSSAGVPSLNAALM
Q6PHW0	HNHYPEKEMVKRSQEFYELLNKRRSVRFISNEQVPMEVIDN
Q47427	NSELIAIQAGNIIVYNYDGGNREYISASTEYLAVGVGIPAN
P49888	LVDRIIHHTSFQEMKNNPSTNYTTLPDEIMNQKLSPFMRKG
Q9H0U4	QESYANVKQWLQEIDRYASENVNKLLVGNKSDLTTKKVVDN
P33783	LRHMQDGQPVYTGVYDDLNANGLPGESTAMKTSDIPGTLQT

Q9NSE7	SPLIMKQIIIFCEHSSDFGWNGYGYAVALLVVVFLQTLILQ
Q8BYP3	RLRPLSYQNTHLVLICYDVMNPTSYDNVLIKWFPEVTHFCR
O08602	ATEVGECLTQPLKDLCQKLRNKVSNTKVDTHKTNGYPHLQV
P42574	PVEADFLYAYSTAPGYYSWRNSKDGSWFIQSLCAMLKQYAD
Q9QZM8	LYALAQRCPADADADADADGNRHDEFPFCALARFCLRAPFG
Q3T0Y3	SPRVVKTHLPIDLIPKSFWENNCKIIYLARNAKDVAVSFYH
P50615	RCIRVNKFQRVDPDVLKACENSCILYSDLGLPKELTLWVDP
Q8NAJ2	PCLGPDCLLCTQGCELHEGRNHMAVHSCVARAWPGDPQEVR
P12970	EAIRTNYNDRYDEIRRHWGGNVLGPKSVARIAKLEKAKAKE
Q58CY8	RGNLTREFFTKELTKHYQGSNDTDVFSATWNSVMITFGCCG
Q8N565	QKEADSLLSVTKLSTISDSKNTRKAREMLLKLAEETNIFPT
Q5SSG5	VAVLGARGVGKSAIVRQFLYNEFSEVCVPTTTRRLYLPAVV
Q9QWV4	HDRGHVIRKSKNNKTGDEEVNQEFINMNESDAHAFDDEWQN
Q2KJ84	ARDRNIQESNFDRVNFWSMVNLVVMVVVSAIQVYMLKSLFE
Q6UXP7	GIKLDFKSLAVVEPSMMLLENVKRHLKRPVWINADILPGPN
Q9NQ60	NGTESEISVRATTDLNFALKNDKTVNATTYEKSTIEEETTT
Q9D9F8	DEAIAHVKHMEMSLKMLENINPKENDMTLQELLDRINNADT
Q8NGN8	YPLLKLACTDTHVIGLLVVANGGLSCTIAFLLLLISYGVIL
Q9D8N6	SNTYVIKLFDRSVDLAQFSENTPLYPICRAWMRNSPTVRER
Q50H32	LARTASEKAFGTRRVNILSKNGTVRGVKYKVSAGQALFNNL
A3KN24	LTGRLMFMGKIILILLQGDRNDIKEYLILQKTCKVDVDSSG
Q04323	SLIEMGFPRGRAEKALALTGNQGIEAAMDWLMEHEDDPDVD
Q3ZCK9	LNEDMACSVAGITSDANVLTNELRLIAQRYLLQYQEPIPCE
Q0P565	VSDHMYRMAVMALVTKDEHLNKDRCVRLALVHDMAECIVGD
Q9HC57	RQIIRALCLLLLLLHAGSAKNIWKRALPARLAEKSRAEEAG
Q9CWT3	NALLVQLPELPSKNLFFNMNNRQHVDQRRQGLEDFLRKVLQ
Q14749	KNIYYKSDLTKDVTTSVLIVNNKAHMVTLDYTVQVPGAGQD
P31273	RIEVSHALGLTERQVKIWFQNRRMKWKKENNKDKLPGARDE
Q9NVT9	EEPDALSVVNQLRDLAADPLNRRAIVQDQGCLPGLILFMDH
Q8C3X2	VKQQLTNETSRIRADNKLDINLERSRVTDMFTDQEKQLIEA
Q3I5F7	ITKSGFLTFMDTWSNPLEEHNHQSLVPLEKAQVPFLFIVGM
B2RXH8	PPPPPIALAVVPSKRQRISGNTSRRGKSGFNSKSGKRGSSK
Q9BSN7	GLVTFYRIGPYTNLSWSCYLNIGACLLATLAAAMLIWNILH
Q3SZR3	MDLLSGKWFYIGSAFRNPEYNKSARAIQAAFFYLEPRHAED
Q8BQN6	GKSNLTEHEKIHIGEKPFKCNECGTAFGQKKYLIKHQNIHT
Q9R1P1	LWEPNMDPEHLFETISQAMLNAVDRDAVSGMGVIVHVIEKD
Q9D3J8	PLFPGAIPPSGQAGTKPDVQNGVLPTRQAGAKAVNQGTTPG
Q3SZJ9	VEKDGYKTIYFFGDKTMPGGNDHEIFTDPRTVGYTVAAPED
Q9H4Y5	PYSHRTRLVLKAKDIRHEVVNINLRNKPEWYYTKHPFGHIP
Q9BXU9	FMTILGPKLVSSEGRDGFLGNTIDSIFWQFDMQRITLEELK
Q78PG9	FHRQKDVKIVTVEKKVNEILNRLEKTKLEKFPDLAAEKEGR

Q8IVQ6	HHCSRCGHCVRRMDHHCPWINNCVGEDNHWLFLQLCFYTEL
P97428	KERSFSEDVLGWRESFDLLLNSKNGVAAFHAFLKTEFSEEN
P07992	SNSIIVSPRQRGNPVLKFVRNVPWEFGDVIPDYVLGQSTCA
Q2KI30	VLIFGTLYPAYSSYKAVKTKNVKEYVKWMMYWIVFAFFTTA
Q5E993	YINEHDYAKAAFCLEELMMTNPYNHLYCQQYAEVKYTQGGL
Q9BUN5	SFLTEVTDVYEMEGGLLNLLNDFHSGRLQAFGKECSFEQLE
O95073	NKHSVIVEKNRCWDIIAVNYNAIGVDRPPRTAQGLRTLYKR
P68002	KLTRNNFAVGYRTGDFQLHTNVNDGTEFGGSIYQKVCEDLD
Q5SSH8	TKGMEANEQEQREKQKFPPCNSEWSSAKGSRLWCSQKSGGV
Q2TBV3	AEKEKVDLVLLGKQAIDDDCNQTGQMTAGFLDWPQGTFASQ
P68510	LETVCNDVLALLDKFLIKNCNDFQYESKVFYLKMKGDYYRY
Q9BG79	AKPVTELVCSGQCGPARLLPNAIGRGKWWRPSGPDFRCIPD
P15327	GEGQWNKENRFCSWVDQKLNNDGLEEARNCGRQLKALNFEF
Q6ZUS5	SASRYAEQISVLKTALLQKENIQSSLKRKLQAMRDIAILKE
Q60652	TGVKHWFCYGTKCFYFIMSKNTWSGCKQTCQHYSLPLVKIE
Q9D1B9	PESQQGLWGGEGLILGYRYANNDKLSKRVKKVWKPQLFTRE
P06342	EGGNSERHFVAQLKGECYFTNGTQRIRSVNRYIYNREEWVR
Q3ZCC4	EFASESSRSLTEEEQQRIERNKQLALERRQAKLLSNSQSLG
O55135	NRHGLLVPNNTTDQELQHIRNSLPDSVQIRRVEERLSALGN
Q14761	LWASPPGRWLQARAELGSTDNDLERQEDEQDTDYDHVADGG
P00766	QKLKIAKVFKNSKYNSLTINNDITLLKLSTAASFSQTVSAV
Q9NWX6	PSNQTLKDYLSWRQADCHINNLYNTVFWALIQQSGLTPVQA
P70677	KNFHKSTGMSSRSGTDVDAANLRETFMGLKYQVRNKNDLTR
Q8WUZ0	ERRGRGASPRGGGPLILLDLNDENSNQSFHSEGSLQKGTEP
Q9H1Y0	SQVINEMQKKDHKQLWMGLQNDRFDQFWAINRKLMEYPAEE
Q5JUQ0	TETCTIVGPTKRDSKFIISMNDNFYPSVTWAVPVSESNVAK
Q8VBV7	PVAVMADNAFSFRKLLDQCENQELEAPGGIATPPVYGQLLA
P62995	RHSHSHSPMSTRRRHVGNRANPDPNCCLGVFGLSLYTTERD
Q96DG6	PVDAGKAVIVIQDIFGWQLPNTRYIADMISGNGYTTIVPDF
Q5E997	IHAPPGEFNEVFNDVRLLLNNDNLLREGAAHAFAQYNLDQF
Q8N5J4	GKRKGNRKTMTYQKMARALRNYGRSGEITKIRRKLTYQFSE
Q8WWZ3	LLDVIRIKLDPCHPTVKNWRNFASKWGMSYDELCFLEQRPQ
Q8K3V1	YIYQMRLPLVPRNTVSKSIKNNIRLSELLSNPNVLSNELKI
P97299	KSVLWLKDSLQCTCEEMNDINAPYLVMGQKQGGELVITSVK
Q8CGF5	FQILFHFVSYWFSARVSSGYNSLSIDKKIEWNSRVVSTCHS
P52907	TVSNEAQTAKEFIKIIENAENEYQTAISENYQTMSDTTFKA
Q9JK39	EGMELRWYRDKPSSVVHVYKNGEDVYDEQMVEYKGRTSFNG
Q2TA29	VVLIGDSGVGKSNLLSRFTRNEFNLESKSTIGVEFATRSIQ
P04095	TIKNVPDIIISKATDIKKKINAVRNGVNALMSTMLQNGDEE
P04390	IGYVYTRVATRKSSLKTYNINELNEIPKPYKGVKVFLQDKW
Q9ULZ2	MLQKNPSLGNMILRPGSDSRNYSITIRQEIDIPRIKHYKVM

P08884	RYCGGFLVQDDFVLTAAHCRNRTMTVTLGAHNIKAKEETQQ
Q9NXJ5	VVHVGVSGMATTVTLEKCGHNKGYKGLDNCRFCPGSQCCVE
Q9CZ92	VFPKLDLLTKVPERALGLDKNRVIETAPLSFRSLLGVLGIE
Q9D035	AALQRQIFDFLGYQWAPILANFLHIMAVILGIFGTVQYRSR
Q9BPW5	QPSSTPEKRRTSLIPRPKSPNMQDLKRRFKQALSAKVRTVT
Q8BHI7	YFKAFLGPRDTRVKGWFLLDNYIPTFVCSVIYLLIVWLGPK
A2AKM2	RVCQSISLLELLHIYIGIESNQLFPRFLQLTERVIILFGVI
P31041	ALCVIWTNSRRNRLLQSDYMNMTPRRPGLTRKPYQPYAPAR
O08603	LWYEAKCFVGEILILHLSNINKTMTSGDPGETANATEVKKC
P30405	FTNHNGTGGKSIYGSRFPDENFTLKHVGPGVLSMANAGPNT
Q92871	LLSKQTIQNHLGEELLQDLINFCLSYMALLRLPKKRGTFIE
Q99LD8	RPGLPGATPFLLHRGGGDLPNSQEALQKLSDVTLVPVSCSE
Q8R3S2	KKFSCCGGISYRDWSQNMYFNCSEDNPSRERCSVPYSCCLP
P05837	LWQINPFPDYPFFALNQSGSNTNTSDKLTIWNDLSPGTLVV
Q99JY3	KAPKFFQEVMHEFQNRYCLFNNRASGAEKEEQKMQLLTLVQ
Q5E9T4	CGLIPVGQPCNQVTTTGLKWNLTHQMLGFGTLVSTSNTYDG
P55258	EKLALDYGIKFMETSAKANINVENAFFTLARDIKAKMDKKL
Q8N8R7	LSSNDMLLLQLRTGMTLSGNNTICFHHVKIYIDRFEDLQKS
Q12999	QFVISCSCLAINRSKQTDVINASWWVMSNKTRDELERSFDC
P59539	CSVELRTTAYNIWAVTGHFSNWPATSLSIFYLLKIANFSNL
Q9JJU9	EFPGYRGRQYVFERGEFRHWNEWDANQPQLQSVRRIRDQKW
Q99623	IRARPRKISSPTGSKDLQMVNISLRVLSRPNAQELPSMYQR
Q8IY49	ELLCYVVMGFFPALVILSMPNTEGIWELVTGGVFYCLGMVF
Q9EST5	GGLDRLAEELPSLTHLNLSGNNLKDISTLEPLKRLDCLKSL
Q6P6J4	WVPVLERVRDRVCEVTGQTFNFVLVNRYKDGCDHIGEHRDD
P0C0E4	QAYAERLGVTFFEVSPLCNFNIIESFTELARIVLLRHRLNW
P11456	SNGKETVVGRFNETQIFNGSNWIMLIYKGGDEYDNHCGREQ
Q3TAS6	GPETAAFIERLEMEQAQKAKNPQEQKSFFAKYWMYIIPVVL
Q71SV0	DHYHFSSSELGGDFEFMDDANMCIAIAISVLMILICAMATY
Q14493	DFETDESVLMRRQKQINYGKNTIAYDRYIKEVPRHLRQPGI
Q9DAZ2	GERDIATYSAWSGLASLQSSNEDVRCFSFYTMIRCLLRDSR
Q61335	PKRWQKVFKSRLVELVVTYGNTFFVVLIVILVLLVIDAVRE
Q3SX44	DGVRKALQDLGLRIVEMGDENATLDGTDVLFTGREFFVGLS
A5D7P8	SEPNPDYRGQQNKGAHNEQKNNSMNNSNNVGTGTFGPMGNG
P10284	QIKIWFQNRRMKWKKDHKLPNTKIRSGGTAGAAGGPPGRPN
Q6ZP29	VIGSISSVLYLLSRLPQIRTNFLRKSTQGISYSLFALVMLG
Q9D338	FLGICIKRSGNGLGATFTLRNTIEGQGVEICFELYNPRIQE
Q9D0I8	NSKLKDIRNAWKHSRMFFGKNKVMMVALGRSPSDEYKDNLH
Q91VJ5	DRSHEKPDRSHEKADRNHEKNDRERERNYDKVDRERDRDRE
Q8NCL8	AFVFSSLIPLLLMTPVFCLGNTSECFQNFSQSHKCILMHSP
O15537	VQYRTDERLNWIYYKDQTGNNRVFYGNSDRTSTVQNLLRPP

O76038	QIWRKYDADSSGFISAAELRNFLRDLFLHHKKAISEAKLEE
Q2T9T3	EEPKVGIKTIKVYCQRMQEENITRALIVVQQGMTPSAKQSL
Q925U0	TTWPNDTYEFIYRTYSCGIANKVLCDVTLLKTQLTYISKNA
Q32KV0	WTILDGTDQMWLPVVRTWRLNERHYGGLTGLNKAETAAKHG
Q8K479	SESRVPPPADTPLPFDRVLLNEQGHYDPTTGKFTCQVPGVY
P54920	IAHYEQSADYYKGEESNSSANKCLLKVAGYAALLEQYQKAI
P59024	SKHEVKVYLQKEFEKHGAVVNESHHDALVEDIFDKEDEDKD
P01138	KCRDPNPVDSGCRGIDSKHWNSYCTTTHTFVKALTMDGKQA
Q3SZ72	SVGSGLLSVSLGLVALLASRNLFRPRLHWALLALALVNLLL
Q5VWZ2	FALSSFLNKASAVYQALQKSNGVLPELFQCHGTADELVLHS
P47741	GYKLGVDCVPCPPGHFSPGNNQACKPWTNCTLSGKQTRHPA
Q9N0T5	PGCDYSLFKDGIEPMWEDEKNKRGGRWLITLNKQQRRSDLD
Q148E0	PGNNIHFIHHDRNRLDHKPINSVLQLVGVQHVDFTSRPSVI
Q32L79	SSSSQDISPYIHEIIEFQKKNTNKIKTLSNLFWGNHPQRKR
Q9NYK6	VCKLGTDKETLSFCHICFELNIEGVPKSDLLHTKSLRGHKD
Q2MH31	ERLNAYEREVVVNMLNSLSRNRTLPQIVPRCGCVDPLPGRL
Q5E988	REAAFRNIKTIAECLADELINAAKGSSNSYAIKKKDELERV
Q8R1L4	ETITTHYLFFLGLYRLLYLANWIRRYQTENFYDQISVVSGV
Q08DJ0	GQPLDINMAGEPKPYRPKPGNKRPISALYRLESKEPFLSVG
P04394	VLPVLDLAQRQNGWLPISAMNKVAEILQVPPMRVYEVATFY
Q15286	FSGSYITTIGVDFKIRTVEINGEKVKLQIWDTAGQERFRTI
Q9NP95	TYSSNIYKHGDTGRRYFVALNKDGTPRDGARSKRHQKFTHF
Q9JJR9	LGSSKDTQPHNILQRRLMETNLSKLRSTRVPWASKTNKFNQ
P57784	GLPTDKKKGGPSAGDVEAIKNAIANASTLAEVERLKGLLQS
Q8CGF5	RFGFVIQSVWIVTCVILDVMNIMWMIKITKGCIKVISLIRQ
Q8C5C9	QSPSLSRFFTSADILDFSVENATVTYHLQFGVPSEDDDFMK
O43759	EGYLNSASEGEEFCIYNRNPNACSYGVAVGVLAFLTCLLYL
Q9QWZ1	LLTQYNEEEYEQYCLVASLDNVRNLSTVLKAIHFREHATCF
P04393	NRDDVVYCDPPYIGRHVDYFNSWGERDERLLFETLSSLNAT
P61247	KVVDPFSKKDWYDVKAPAMFNIRNIGKTLVTRTQGTKIASD
P58321	KKFLEESVSMSPEERAKYLENYDAIRVTHETSAHEGQTEAP
P08962	MSKNRVPDSCCINVTVGCGINFNEKAIHKEGCVEKIGGWLR
Q91XQ6	WYLIINAVVLLILLSALADPNQYHFSGSELGGEFEFMDDAN
Q86UA6	RDRLLNRYRQAGSSGPGNSQNSFLVQEVMEEEWNALQSVEN
Q96AX2	AWLTEIHEYAQRDVVIMLLGNKADMSSERVIRSEDGETLAR
Q96SZ5	ASDRDAASGPEAPMQPGFPENLSKLKSLLTQLRAEDLNIAP
Q9NWZ8	AMAWMQSHHNAYRKAVESCFNLPWYLPSALLPQSSYDNEAA
Q3T0S3	VVVPNIKKEYGSQKDFTQVWNSTMAGLKCCGFTNYTDFEGS
Q9BQQ7	MREVKPWHRWTLRPDKGLLPNVLKPGWMQYQQWTFARFQCS
Q8K177	GHVNLLVGAVLHGTVLRHVANPRGAVTPEYTTANVISVGSG
P20040	FYNGTQRVRFLKRYFYNLEENLRFDSDVGEFRAVTELGRPD

Q9ES30	GPPGPPGPPGIPGNHGNNGNNGATGHEGAKGEKGDKGDLGP
P00847	LGLPLVTLIVLFPSLLFPTSNRLVSNRFVTLQQWMLQLVSK
Q7M729	AINGSSILLPCTFSSCYGFENLYFKWSYNNSETSRILIDGI
A6NDR6	SEEQKKQLAQDTGLTILQVNNWFINARRRIVQPMIDQSNRT
Q8NC06	CLVPRPGFWDPIGRYKWDAWNSLGKMSREEAMSAYITEMKL
O75663	QHGSGFGIEFNATDALRCVNNYQGMLKVACAEEWQESRTEG
Q92904	PGAEVVPNECSVHEATPPSGNGPQKKSVDRSIQTVVSCLFN
P14436	GGLQNIAVVKHNLEILTKRSNFTPAANEAPQATVFPKSPVL
Q6NVG5	VSGNNPYSSFGATLERDDEKNLWSMPHDVSHTEADDDRILY
P05531	TYIKDSFKDSNVKLEQLWKTNKQERKKINNKFCEQYITTFQ
Q9CQ07	ELDLSRNMIRKIPDSIAKFQNLRWLDLHSNYIDKLPESIGQ
P55212	HDVPVIPLDVVDNQTEKLDTNITEVDAASVYTLPAGADFLM
Q9NZC4	NLQHLKWNGQCSSDLFQSTHNVIVKTEQTEPSIMNTWKDEN
P11352	LMTDPKYIIWSPVCRNDIAWNFEKFLVGPDGVPVRRYSRRF
Q1LZH0	KKESGQLRFGGRDRPFRKPINLPVVKNDQFREGKRERRERS
Q4VBF2	GIFAEACSNATYVEVWNDFMNRSGEEQERVLRYLEDESQGK
Q6ZP80	HHEGFFWRCWFNGIVEENDSNIWKFWYTNQPPSKNCTHAYL
Q13268	LSVNVKSPALLLSQLLPYMENRRGAVILVSSIAAYNPVVAL
Q8BVG8	REDEKPLVLEMLKAGVKDTENRVALHALTRPPALLLLAAAS
Q9NX63	TFEADENENITVVKGIRLSENVIDRMKESSPSGSKSQRYSG
Q96LR2	MELAYLRAIDVKILQQLVTLNEGIEAVRWLLEERGTLTSHC
Q3KPI0	QASSTTVTEKGSVVLTCHTNNTGTSFQWIFNNQRLQVTKRM
P35625	KYQYLLTGRVYDGKMYTGLCNFVERWDQLTLSQRKGLNYRY
Q58DS9	AQAAIVVYDITNTDTFARAKNWVKELQRQASPNIVIALAGN
O95456	KTSESTGSLPSPFLRALKTQNFKDSACCPLLEQPNIVHDLP
P63139	EEAFTNQLPAGLITADGQQQNVMVYTTSYQQISGVQQIQFS
P50295	LKTLTEILQHQIRAIPFENLNIHCGESMELSLEAIFDQIVR
Q0IIB1	FEKGVFDKQGNFLVPPEKSINKIGHALHALDPIFRCVTHSH
Q9D7F7	LRNMGFAAKAMKAVHDNMDLNKIDDLMQDITEQQDIAQEIS
Q96PP4	LILSDDPTSKREQWFRFSTDNDFKSEGKYSKVYALRTQKKM
Q8N6I1	LLGEPVDEEGPEGRPRSRHGNGGLAALPYLRLRHPLSVLGI
O43291	GPCRASFPRWYFDVERNSCNNFIYGGCRGNKNSYRSEEACM
Q5VTT2	KLSQMCLNTEWVEMKSKALLNEETVSSGIIERVTGLPATGF
P61982	TELNEPLSNEERNLLSVAYKNVVGARRSSWRVISSIEQKTS
Q6QRN8	FIIFKAYLINCVWNCYKYINNRNMPEIAVYPAFEAPPQYVL
A6NM15	EHLNMFIQNLLWEKNVRNKDNHCMEVIRLKGLVSIKDKSQQ
Q8K386	QGQQLAKEYGMDFYETSACTNLNIKESFTRLTELVLQAHRK
Q6P047	QRQIQNTFAILDLKLQKKTLNLNAPTPIPPPITSHAGQEEA
Q8WTT0	LGLSDPGGRRHWQWVDQTPYNENVTFWHSGEPNNLDERCAI
Q9EP73	SRTEGMLLNVTSSLRVNATANDVFYCTFWRSQPGQNHTAEL
Q3T166	QSFVGEIWDVSLWDHVVSLKNLCFTCYTSNILNWKALIYQA

Q08DY6	PSLLVDPAEETVRRRCRDPINVEGLLPSKIRINLEDNVQYV
Q8WUU8	GATVVQYIPPPYGSPEPMGINTSYLQSVVSPCGLITSGGAA
P53026	VLCLAVAVGHVKMTDDELVYNIHLAVNFLVSLLKKNWQNVR
P00639	YLNQDDPNTYHYVVSEPLGRNSYKERYLFLFRPNKVSVLDT
Q99KR3	ILGEGTTIFEDLYDYMNSLNNLLKIKANIIYPGHGPVIHNA
Q7Z692	MLLRRAQPTDSGTYQVAITINSEWTMKAKTEVQVAEKNKEL
A6NJI9	LKGMLNLKILSLYQNPLCQYNLYRLYIIYHLPGVELLDRNQ
Q9P2Z0	VSCTRPRAGKQAAASQITCENELVQTQPHADNPSNTVTSVP
Q9BXN2	DGSKRQCWQLGSNLLKIDSSNELGFIVKQVSSQPDNSFWIG
A8MUP6	AGLLGMVAHMMYTTIFQITVNLGPEDWKPQTWDYGWSYCLA
Q3T110	SLLIPLALWSIIVNILLYFPNGQASYASSNKLTNYVWYFEG
Q15006	YLEQFVGDQEAWHELAELYINEHDYAKAAFCLEELMMTNPH
Q2TBH7	KDLDTDREVTFLEASRFAQENELMFLETSALTGENVEEAFV
P19404	SAALRARAAGLTAHWGRHVRNLHKTVMQNGAGGALFVHRDT
Q8VC04	TFEDAHVQLNTTNVLNIFNSNFYPITVTQLTAEVLHQASVV
Q9R1A9	SEPPRAETFVFLDLEATGLPNMDPEIAEISLFAVHRSSLEN
O70435	DGVVFGVEKLVLSKLYEEGSNKRLFNVDRHVGMAVAGLLAD
P15177	IQANVLKADKMDLRESQGWLNRGYEGAAVGAALGAGITGYN
P20339	EESFARAKNWVKELQRQASPNIVIALSGNKADLANKRAVDF
Q00977	MHVAYRRHEKKRKFMKGEIKNEFKDIEEIKTQKVRIEGSLW
P09629	RIEIAHTLCLTERQIKIWFQNRRMKWKKENKTAGPGTTGQD
P31273	FQHASHHVQDFFHHGTSGISNSGYQQNPCSLSCHGDASKFY
Q9UPY8	KGKFQDNFEFIQWFKKFFDANYDGKDYNPLLARQGQDVAPP
P51908	YEINWGGRHSVWRHTSQNTSNHVEVNFLEKFTTERYFRPNT
Q9NQR4	SFIREAATQGAKIVSLPECFNSPYGAKYFPEYAEKIPGEST
Q8BHL8	LNLDDYIDAEDLSDFHRTYKNSEELRSQIRSGIITPIHEQW
Q96KN8	IIQRTKKMVNKIVQYSLIEGNCEHFVNGLRYGVPRSQQVEH
Q3U0B3	ILSMFSAVRSQHSGVDICINNAGMARPDTLLSGSTSGWKDM
P39687	EEEGEEEDVSGEEEEDEEGYNDGEVDDEEDEEELGEEERGQ
Q32KM6	FKCPQKSEPLWKEWDQKAQKNGLRHQVFAVNGDHYVGEWKD
Q8VHX2	KYPIQDTGLPKAKECDTVNSNCPPNSDDQPQGEENDFPDST
Q2T9Y6	RKKCPSTHSEELRDCIQKTLNEWSSQISPDLIREFPDVLEC
Q56A07	SKYDVSVTLKNVQLEDEGIYNCYITNPPDRHRGHGKIYLQV
Q9EQ06	VYTADLFATQDPQIEKTFEVNVLAHFWTTKAFLPVMMKNNH
Q8VHW3	MQEEDRRRTAVGRRRAQEQQNLGLTPEREGKIKLGLLVAIV
Q03255	QQTTLAFRKDSSLCTDSHSANDQCERGEHVRESSQDHVKRP
Q9NYP7	TLLSLYMFCELVTGVWEGKYNFFCQGTRTAGESDMKIIRVL
Q8R1J3	PFAKCFVCGEMGHLSRSCPDNTKGVYADGGSCKLCGSVEHF
Q9H3V2	FLIAVKRKTTETLIILSRIMNFLSALGAIAGIILLTFGFIL
P16563	ENLYMSTDPTLWSTVIQSWYNENEDFVYGVGAKPNSAVGHY
Q8WV92	EGIDLLLQVLKGTKDNTKRCNLREKISKYMDRAENIKKYLD

Q9Z2U0	AWKANAIGRGAKSVREFLEKNYTDDAIETDDLTIKLVIKAL
P78417	AERTRLVLKAKGIRHEVININLKNKPEWFFKKNPFGLVPVL
P10861	VDVVKTRFVNSSPGQNTSVPNCAMMMLTREGPSAFFKGFVP
Q16854	LLTKTYPEWHVATEPVATWQNIQAAGTQKACTAQSLGNLLD
P05531	APELDVIEEHNPVTRDDENANPEEVVGDTRSPVQNILGKFE
P43320	LSSLRPIKVDSQEHKIILYENPNFTGKKMEIIDDDVPSFHA
Q9DC16	INKVPGNFHVSTHSATAQPQNPDMTHTIHKLSFGDTLQVQN
Q0VCM2	AEAPVTSLPAFLAMPFNSLVNMAYVFLGVYWLRSQARAPGG
Q5JBG6	ASFKRLIQICNMMTMADLKKNLIAKVHDESYLNYYYYYNKP
Q9H7T3	SRRLPKAEGDKSGSAGAPSKNSSRLGGRPCMCTAGRRPNRA
Q9GZP9	QAFTIMLVYVWSRRNPYVRMNFFGLLNFQAPFLPWVLMGFS
Q4JM65	QQNSPVCDLEATLGTAGENYNVIQQTVKYFNSQQQITDLFP
Q9CQE6	VLITCTYRGQEFIRVGYYVNNEYTETELRENPPVKPDFSKL
P19404	RKPVGKYHIQVCTTTPCMLRNSDSILEAIQKKLGIKVGETT
Q99MS4	AGLGSDVALLQLAVSVQSFPNVKPVKLPSESLEVTKKDVCW
Q9H3S4	PLLSTGNLKYCLVILNQPLDNYFRHLWNKALLRACADGGAN
Q5EA90	HSLLLHIGPLASEQMFYAVTNRINDENTYKICTWLEIKVHH
Q9D7L8	IVDLKNGNKINISSVCVSPINESDNGVRFTCKLQRDQTVSV
Q96HA8	DGPVIWDYHVVLLHVSSGGQNFIYDLDTVLPFPCLFDTYVE
A6H7I7	ARRQEPALRGGPGPLTPHTCNELGPPPSPRTPRPARRGSGR
P48755	EEERRRVRRERNKLAAAKCRNRRKELTDFLQAETDKLEDEK
P20851	LGTYVCIKGYHLVGKKTLFCNASKEWDNTTTECRLGHCPDP
Q28153	TLNSYVQLGVLPQSGTILANNTPCYITGWGRTKTNGQLAQT
Q9JKK1	LRSIEWDLEDLDETISIVEANPRKFNLDATELSIRKAFITS
O18879	QQVPMVEIDGMKLVQTRAILNYIATKYNLYGKDMKERALID
Q9BZG1	RFKCIASTYYRGAQAIIIVFNLNDVASLEHTKQWLADALKE
Q0V7M7	LKELFESLEEDYKDVEHLKENIPPHLPQVTVTQNFVNGSDL
Q9CRA5	LMEEVLLLGLKDREGYTSFWNDCISSGLRGCMLIELALRGR
Q9Y6H3	PERLAQGNPQQGFFSSFFTSNQKCQLRLLKTLETNPYVKLL
P42574	NLEPKIIHGSESMDSGISLDNSYKMDYPEMGLCIIINNKNF
Q60753	GAAARGPGPEPVTVATLFTANSTAGIFSAKVLGFHVCGLYG
P49788	TTQVSHYYLAQLTSVRQWKTNDDTIDFDYTVLLHELSTQEI
P56966	DLKPLLDTLGLFFQIRDDYANLHSKEYSENKSFCEDLTEGK
P39905	FRYCSGSCDAAETTYDKILKNLSRNRRLVSDKVGQACCRPI
A6QPH1	PITASRPEGGEPALSKTAKRNLKRKEKRRQQQEKGEAEALS
A0JN69	LFTIYLFWTLVSFRYHCRLYNEWRRTNQRVILLIPKSVNIP
P70377	GWYLGLNKEGEIMKGNHVKKNKPAAHFLPKPLKVAMYKEPS
P98086	LFIKSSSGGQPRDSLSFSNTNNKGLFQVLAGGTVLQLRRGD
O75636	SLSLHSGRPFTTYDADHDSSNSNCAVIVHGAWWYASCYRSN
P97299	LGSARGLFLFGQPDFSYKRSNCKPIPANLQLCHGIEYQNMR
A6ND01	RMSYTCKSNWRGGWDWSQGKNRCPKGAQCLPFSHYFPTPAD

A1E959	GFLGATLSAPLIPQRLMSASNSNELLLNLNNGQLLPLQLQG
Q6ICI0	SSMYYGGKCRLGWGYMTAILNAVLASLLPIISWPHTTKVQG
Q96IL0	EFLSKNFQKHMYYNRDWYKRNFAITFFMGKVALERIWNKLK
Q8N1B3	FQVSFQHPHKYLLHYLVSLQNWLNRHSWQRTPVAVTAWALL
Q96CE8	ICFSGIMMLIVTTVLLVLENNNNYKCCQSENCSKKYVTLLS
Q8WWG9	QTVSLAPAVEAASMLKMEPLNSTHPGTAASSSPLESRAAGG
P31946	TEQGHELSNEERNLLSVAYKNVVGARRSSWRVISSIEQKTE
P61106	AFLEAAKKIYQNIQDGSLDLNAAESGVQHKPSAPQGGRLTS
Q47456	KTGRYLQQGLVEEGYQADLFNNGRDGLGAASKGQYDLIILD
Q56A07	TRLPCTFNSCYTVNHKQFSLNWTYQECNNCTEEMFLQFRMK
Q8BGI3	LGLGITIAFLATLITQFLVYNGVYQYTSPDFLYIRSWLPCI
Q6NXY9	EDGEEDAEQEDYDEEEQEEENDYINSYFDNGDDFGVDSDDN
P08218	GVSTYAPDMSRMLGGEEARPNSWPWQVSLQYSSNGQWYHTC
O75934	VREAAAALVEEETRRYRPTKNYLSYLTAPDYSAFETDIMRN
Q9CR68	KPEWVILIGVCTHLGCVPIANAGDFGGYYCPCHGSHYDASG
Q6PKH6	VSKTALLGLNNTLAIELAPRNIRVNCLHLDLSRLASAGCSG
Q2TBL9	GVDVNGKYTADGKEVLEYLGNPANYPVSIRFGRPRLTSNEK
Q8WUN7	AAHAFESNDHELAQAIIDGANITLPHGALTECYDELGNRYQ
P57087	TRSDAGKYRCEVSAPSEQGQNLEEDTVTLEVLVAPAVPSCE
P31275	DPPSCQSLESDSSSSLLNEGNKGAGAGDPGSLVSPLNPGGG
O43610	GTCLCCVKGLFYHCSTDDEDNCADEPCSCGPSSCFVRWAAM
Q9Y3E7	KEMIRSRKAVSKLYASKAHMNSVLMGMKNQLAVLRVAGSLQ
Q8NH89	TAVTIFYGTLSYMYLQPQSNNSQENMKVASIFYGTVIPMLN
Q3SYP2	RLWTNGPIAEVLQQGLQPIVNHTTCSRLDWWFIKVRETMVC
P32850	LEITGRTTTSEELEDMLESGNPAIFASGIIMDSSISKQALS
Q6IR41	KTGLHSSENFLSLLFDRVFVNTDGHFDMATGSFVAPLRGLY
Q9BVG4	VSGELVSVAHALSLPAESYGNDPDIEMAWAMRAMQHAEVYY
Q8K3A2	AVFIDGPLALTDGIPFFCSANGVILTPGNAEGFLLPKYFKE
Q9H8T0	DPTSGELDVKRAFAKWRRNHNHIWQVLMYARRVFYKIDTAS
Q96D96	QFEALGLLILLRLWRVARIINGIIISVKTRSERQLLRLKQM
Q9BQ51	VTSVLRLKPPPGRNFSCVFWNTHVRELTLASIDLQSQMEPR
Q9NWC5	LFLTICFCWHYAVTIVIVGMNYAFITWLVKSRLKRLCSSEV
A1A4Q8	PQLKESLQTLMKVAAQNLIQNTNIDNGQKSSDGPIQRFDKC
Q8N6M8	ESKAEAKTPVLVETQTVDNANEKSEKPPENQKKLSDKDTVA
Q3T160	TVSLGAGAKDELHVVEAEAMNYEGSPIKVTLATLKMSVQPT
Q2KIG1	VVLITCTYRGQEFIRVGYYVNNEYTETELRENPPVKPDFSK
Q2KIN3	HHYSAAFQIDGHWMHYDGLRNVNLILLNKPPEFLLLSSLVY
Q8WXI8	FQSNCYFPLTDNKTWAESERNCSGMGAHLMTISTEAEQNFI
Q9DCT5	VSAFGEEGEGDYLDDWTVLCNGPYWVRDGEVRFKHSSTDVL
Q96PB8	NLKEIPRDLPPETVLLYLDSNQITSIPNEIFKDLHQLRVLN
Q8BX10	VWDTNWDRREPLSLINLKKRNVESGEDELTSRLDHYKAKAT

O35723	SCGGGAAGNYKSVSTSTEIINGKKITTKRIVENGQERVEVE
P0CW71	SAIDDLQFKFLQVDDKDDHFNLPVIAETFRGLSPKMKPLKG
P0DMB1	PAEEGKAAPLYQQPLMIPQANHMAGISPSFLVTPLCIPRCR
Q14493	TVPADFETDESVLMRRQKQINYGKNTIAYDRYIKEVPRHLR
Q61199	IHNTESVDWEDRTVPETLVGNLFHSRITSPLRLFVKQPPDP
A6NCD4	TAEEIIDKHLQKDLDAEENQNVAKTLRGKVREKLKISKINK
Q9D1D1	AAEITASVDRLQQDFKCCGSNSSADWQHSAYILSQEALGRQ
Q61885	PIRALVGDEAELPCRISPGKNATGMEVGWYRSPFSRVVHLY
Q9XSK2	EKILAVTNKVPDSCCVNITHNCGINFVVKDIHTEGCVEKIA
Q8BG84	LSALLFCFLRHRQKKQGLPNNKRQQQRPEERLNLATNGLEM
Q32KY8	QTRQSNILKILLQYGILERENNPINIVLTILLYPSRVRIMV
P97461	RRVNQAIWLLCTGAREAAFRNIKNIAECLADELINAAKGSS
Q02738	EERERKHRLKHGPNAPALYSNLSKKRGGLWWTYLLSLIFKA
Q9NQ48	ITSSNKKPILDVTKPKLAPLNEGGTAELLNKEILRLQEENE
Q60631	KVLRDGAGKYFLWVVKFNSLNELVDYHRSTSVSRNQQIFLR
Q9H4A5	ATEPTETVQTWIELLTGETWNPFKLQYQLRNVRERIAKNLV
A6NGC4	TAVLSGHYVGFSMVSLLLELNSACLHLRKLLLLSRQAPSLA
P08311	CGGFLVREDFVLTAAHCWGSNINVTLGAHNIQRRENTQQHI
Q8N4E7	PAAGPSRVRQNFHPDSEAAINRQINLELYASYVYLSMAYYF
Q14919	HMDGDKGARRGRKPGSGGRKNGGMGTKSKDKKLSGTDSEQE
Q58DM4	AGLAEEEGNSKKNPRRAAPGNGVDSAGLTWGRIRAEGLNCD
Q28132	CIYLQLLLFNPLVHTQGICSNRVTDDVKDVTKLVANLPKDY
P18181	NELKITLEVFDPVPKPSIEINKTEASTDSCHLRLSCEVKDQ
Q9CRB9	TFEADENENITVVKGIRLSENVIDRMKESSPSGSKSQRYSS
Q3SX30	LNLNDYIDSEHLVDFHRVYKNSEELRSRIVSGIITPIHEQW
Q5VXU3	PSVLTGKVAPEEFKTSIGRVNACLKKALPVNVKWLLCGCLC
P97765	FMMPFYLMKDCEIKQPVFGANFIKGIVKAEAGGGWEGSASY
A6QLP7	TSSQNDVDMSWIPQETLNQINKASPRRLPRKRAQKRSVGSD
P35288	FTKDYKKTIGVDFLERQIQVNDEDVRLMLWDTAGQEEFDAI
P13761	LLVCSVSGFYPGSIEVRWFRNGQEEKAGVVSTGLIQNGDWT
P49721	YKMRNGYELSPTAAANFTRRNLADCLRSRTPYHVNLLLAGY
Q8WTZ3	IKIIHTEEKPDKCEECNKVFNWSSTHTKYKRIHTEDKFYKY
Q5E943	RQIMRFAMKSRRGPHVPVGHNAPKDLKEEIDIRLSKVQDIK
Q9Z1Y9	SDGWAVPGDSSLSFEVKKHRNQEDRCAAAHQLLERFSLPPQ
Q9H082	SMVQHYYRNVHAVVFVYDMTNMASFHSLPSWIEECKQHLLA
Q8NCR9	KGTTHSYGYSFWLILLVILLNIVTVTIIIFYQKARYQRKQE
Q9NP55	SGGLLGGLLGKVTSVIPGLNNIIDIKVTDPQLLELGLVQSP
Q3T0N1	GTPASFGKTFAQKSGYFLCLNPLGSLENPQENVVVDIQILV
Q920L5	ITLSQITQMLMGCVINYLVFNWMQHDNDQCYSHFQNIFWSS
Q60654	TTKYNIRDGLCMSLSKTRLDNGDCDKSYICICGKRLDKFPH
Q9Y272	AMRRLSILTGDVFILVFSLDNRDSFEEVQRLRQQILDTKSC

Q5E948	DNLEKEMLPHYEPIPFSASLNESTPTGITDHIAQGPDPNTE
O60258	KSKDCVFTEIVLENNYTAFQNARHEGWFMAFTRQGRPRQAS
P04390	LGSDTKVLSTIFELFSRPIINKIAEKHGYIVEEPKQQNHYP
Q9BTE7	TWPLFSVFYQYLEQSKYRVMNKDQWYNVLEFSRTVHADLSN
P48230	GCARCLGGTLIPLAFFGFLANILLFFPGGKVIDDNDHLSQE
Q0V881	KAGDAGRYWCALLGQRYRYQNWRVYDVSVLRGSQFSARAAD
Q5VWW1	YHVLMRGGDGTSMWADLCKNNQVRASAIAQDADQNYDYASN
P49406	ELKVKMKPKPWSKRWERPNFNIKGIRFDLCLTEQQMKEAQK
P46926	ESYHSFMWNNFFKHIDIHPENTHILDGNAVDLQAECDAFEE
P04483	DRHHTHFCPLEGESWQDFLRNNAKSFRCALLSHRDGAKVHL
Q9NYP7	ITVLHVYHHASMLNIWWFVMNWVPCGHSYFGATLNSFIHVL
A2VDR2	VASREQLLYLYARYKQVKVGNCNTPKPSFFDFEGKQKWEAW
Q8R035	PLDRLSISYCRSSGPGGQNVNKVNSKAEVRFHLASADWIEE
Q7L0L9	ARFIVVPAAYALALGLGLPANVAALAMFIRSGGRLGQALLL
P09926	IVPSTKNRHQLFCKLTLRHINKSPEHVLRHTQGRRYQRALH
P0DMR3	SKENLLALNTHSRRQKGKRENKVCVSTWQKSRGDRTYSSMA
P09012	ILFLTNLPEETNELMLSMLFNQFPGFKEVRLVPGRHDIAFV
Q61999	GSCVKIESPCYPCTSPCNPCNPCSPCSPCAPCACGPCGPCG
P17433	HYWDFSAHHVHNNEFENFPENHFTELQSVQPPQLQQLYRHM
P13661	TWMQFSVVWVSQEITQKIGLNKIKNYLKDFDYGNQDFSGDK
Q17QN8	HDQPIPWKVQFNLGNSSRPSNQCRNSIQGKHLITDELGYVC
Q5E995	VAADALGEEWKGYVVRISGGNDKQGFPMKQGVLTHGRVRLL
P50540	AKAHIKKLEEAERKSQHQLENLEREQRFLKRRLEQLQGPQE
Q9DCS2	WEWEQWGGIPPRSLDLLLCINMIHISPLNCTEGLFRAAGHL
Q32PI9	KDRVSWAGDLDKKDASINIENMQFIHNGTYICDVKNPPDIV
Q92527	DCATILLNFGADPDLRDIRYNTVLHYAVCGQSLSLVEKLLE
P28063	LPRGMQPTAFLRSFGGDQERNVQIEMAHGTTTLAFKFQHGV
P08218	VSLTDKIQLACLPPAGTILPNNYPCYVTGWGRLQTNGALPD
O35671	AIEKLAVSEGKSLEGPLDLINYIDVAQQDGKLPFVPLEEEF
Q99LS3	RSIVEHVAAKLNIPTTNVFANRLKFYFNGEYAGFDEMQPTA
Q8K2M3	PTVFYMPHCGTALYNNLLWSNWSADALSRVLIIGNSFRGLE
O54709	VSSREGYCGPCPNNWICHRNNCYQFFNEEKTWNQSQASCLS
Q3ZBY3	TEFMEAWRKYDTDRSGYIEANELKGFLSDLLKKANRPYDEP
P48758	LSRILARKLNEQRRGDKILLNACCPGWVRTDMAGPKATKSP
A5PKI3	NVLMSGVKNNVGRGINVALVNGKTGELIDTRFFDMWGGNVA
Q92874	VYLDVIDKWGTDDMLFLGDFNADCSYVRAQDWAAIRLRSSE
Q7L2Z9	VIMTILSNSIKEKEEIQYHLNFLKKRLLQQCETLKVPPKKM
O00560	GFIFKNGKITSIVKDSSAARNGLLTEHNICEINGQNVIGLK
Q9D5K1	NLKFVKRLYFGLRVLPPKLVNLNFQQSLTEDQKLIDKGWEV
O43513	QQVSALPPPPMQYIKEYTDENIQEGLAPKPPPPIKDSYMMF
Q6MZM9	RNLPPPLYYRPVNTVPSYPGNTYTDTGLPSYPWILTSPGFP

Q9WTQ8	MNGLRLGLKETQSMAWSKPRNVQILNMVTRQGALWANTLGS
Q9JKX8	LVRVGNNGTCFWDPNFQGLCNPPLTAATEYRFKYVLVNMST
P70331	PEEAAPSMPHDSGLRAQQALNSIDLDVPTEAVTCQPQGNPQ
P11900	VFEGLVNPGRFTDQNIAYKWNGLSKAEMAGYVEKLMPGQSA
Q32KP7	ADQEDSLPKLCTAWGLRSHLNGMKERLSKLQAPGHPARLLK
Q9D6Y1	EMLCDQAWGSMLEVPAGSRLNLTGLGYFSCHSHTVVQDYSY
P07477	DRKTLNNDIMLIKLSSRAVINARVSTISLPTAPPATGTKCL
P47754	ESWRTSVETALRAYVKEHYPNGVCTVYGKKVDGQQTIIACI
Q9CPX5	AVVKYSRLEDVLHGCSWKINNKLDGTYLPLPVDKIMQRTKN
P13765	SDVGMFVALTKLGQPDAEQWNSRLDLLERSRQAVDGVCRHN
Q96BD8	CDEFNGVPSYMKSRLTYNQINDVIKEINKAVISKYKILHQP
Q8R191	QTLLRVVSWVFSIAVFGPIVNEGYVNSDSGPELRCVFNGNA
A6NJ46	GLSSQGVYYSPQVGNFSKAGNEYPTRTRNCWADTGQDWRGG
P41317	SFEDLTGNRVRYTNWNDGEPNNTGDGEDCVVILGNGKWNDV
Q29438	RTTNRILASSCCSSNILGSVNVCGFEPDQVKVRVKDGKVCV
P31275	PPHDPPSCQSLESDSSSSLLNEGNKGAGAGDPGSLVSPLNP
Q505H4	CEPSNEVHCVVADCAVPECVNPIYEPEQCCPVCKNGPNCFA
O54831	ELFEYTQSILNSIYGTTTTGNVEYTVFSGLEDLKSSDEEFS
Q60930	AIEDQICQGLKLTFDTTFSPNTGKKSGKIKSAYKRECINLG
Q9H1E3	DEDFLMEDDDDSDYGSSKKKNKKMVKKSKPERKEKKMPKPR
Q2TBH5	TSWVNSFKPSQVYLKEEEEKNEKRQKLVRKKQQEAQGEKVS
Q8WXK3	ANVNAAKLHETALHHAAKVKNVDLIEMLIEFGGNIYARDNR
Q2KJD3	PSHTKIKYRQTTQDAPEEVRNRDFRRELEERERAAAREKNR
O75954	AIGTIVMVTGFLGCLGAIKENKCLLLSFFIVLLVILLAELI
P07478	VVSNGELQGIVSWGYGCAQKNRPGVYTKVYNYVDWIKDTIA
Q3SZ18	PDFVGFEIPDKFVVGYALDYNEYFRDLNHVCVISETGKAKY
Q1LZC5	RRLGPTGKEVHALKRLRDSANANDVETVQQLLEEGTDPCAA
Q9D0I8	IEELRKCVDTYKYLFIFSVANMRNSKLKDIRNAWKHSRMFF
Q06186	LLPTGGDRAQGVQDLEGTDLNLFKVAFSSKPQGLATPSKER
P47878	SESKRETEYGPCRREMEDTLNHLKFLNVLSPRGVHIPNCDK
Q92535	ERQPFPDNYVDRRFLEELRKNIHARKYQYWAVVFESSVVIQ
O95237	LLISNFTLFSSGAAGEDKGRNSFYETSSFHRGDVLEVPRTH
Q9NPC3	VLGFPLGNNSKFPLDNTPVRNRGDGDGDFQFRPFFAGSPTA
P01583	KKRRLSLSQSITDDDLEAIANDSEEEIIKPRSAPFSFLSNV
Q9H741	DELGYVCERKDLLVNGCCNVNVPSTKQYCCDGCWPNGCCSA
Q86VG3	KDVLDKFLNCHEQTYDEEFLNTFTHLSQEDHVSKRGVFGTD
Q8QZV2	GARAPPTRSQTNCCLPEGTMNNVYVNMPTNFSVLNCQQATQ
Q8BGC9	EKIQGLPFGSCLAISDGPVHNSTGIPFFYMTAKDPAVADLV
O95407	PCPPRHYTQFWNYLERCRYCNVLCGEREEEARACHATHNRA
P06342	RWFRNGQEETVGVSSTQLIRNGDWTFQVLVMLEMTPHQGEV
Q9BY71	AVAVFCSLRGLQEVPEDIPANTVLLKLDANKISHLPDGAFQ

Q96FW1	MDLIEQVEKQTSVADLLASFNDQSTSDYLVVYLRLLTSGYL
Q8BNL5	FDLLRKEADFYQIEPLIQCLNDPRPLYPMDTFEEVVELSST
Q8BGF6	NLTEMAYSLLKSEALKLHLYNFVPGVPTMEHFHQFYCYLVY
Q9ERQ8	LTDALYMVRFKDTKAQFSCFNPKCLLPTSRHYWTYPGSLTT
A5PKE4	NAQKLLSEALELEMDHLLVENIERETFHLCSRLINGPYRRT
Q9Y657	TEDGSKDEWRGMVLARAPVMNTWFYITYEKDPVLYMYQLLD
Q9H7X0	HISTTAQDHCKAIYLHVLTTNNTAINFYENRDFKQHHYLPY
Q9H400	RALPAAAATAGCAGLEATYSNVGLAALPGVSLAASPVVAEY
Q9NVK5	EIDEQQGCKEQERIFQLEQENKGLREILQITRESFLNLRKD
P22676	AEFMEAWRKYDTDRSGYIEANELKGFLSDLLKKANRPYDEP
P26892	LDYLQNEYEGNQENVRDLRKNIRTLIQILKQKIADLITTPA
P53026	AFLASESLIKQIPRILGPGLNKAGKFPSLLTHNENMVAKVD
P97348	MVFAKGAFPESYSPTVFERYNATLQMKGKPVHLQIWDTAGQ
P61982	ARRSSWRVISSIEQKTSADGNEKKIEMVRAYREKIEKELEA
Q75VT8	AKNLQKQRERESCWAQINFTNTDMSFDNSLFAISTKMTQED
Q2HJF3	LNSNIGIRDLAVQFSCTEAVNLASKILQSYESSLPQTQQVD
Q91ZT8	TIDWRTPLFNACVSGSQDCVNLLLQHGATPHPETELASPIH
Q2HJ54	KEDFLIKIETWHKPDLGTLENVHKLEPEAWKHVEVIYIDIA
Q3SZC2	YQLELHGMELEEPPLVLAAANVVRNITYKYREDLSAHLMVA
Q28110	VTLTCQGTSFSAGNLTTWFHNGSSIHTQKQPSYSFRAGSND
Q9D4V7	HDLTNKKSSQNLYRWSLEVLNRDAVPTGVLVTNGDYDREQF
Q91XD3	GCARCLGGTLIPLAVFGLLANILLFFPGGKVVNDKSHLSDE
P54821	DQLNSEEKKKRKQRRNRTTFNSSQLQALERVFERTHYPDAF
Q8N4E4	EDVWVIIHLYRSSIPMCLLVNQHLSLLARKFPETKFVKAIV
P14435	VFPKSPVLLGQPNTLICFVDNIFPPVINITWLRNSKSVTDG
P08294	SIVGRAVVVHAGEDDLGRGGNQASVENGNAGRRLACCVVGV
Q9NPE2	GSGALPSGQKLEELKAEEPDNFSSKVVQRGREFFDSNGNFL
Q9Y255	QSVLRSSWDQVFAAFWQRYPNPYSKHVLTEDIVHREVTPDQ
Q5H9L2	EGKREDEGEPGDEGQLEDEGNQEKQGKSEGEDKPQSEGKPA
P05231	QIRYILDGISALRKETCNKSNMCESSKEALAENNLNLPKMA
P00435	RGLVVLGFPCNQFGHQENAKNEEILNCLKYVRPGGGFEPNF
Q9H560	ALMLAVQHNSSSIVSLLLQQNINIFSQDLFGQTAEDYAVCY
C9J442	AVVGPFHGPEWEPVQGLLSQNHSCRDPQCCGNLLVLCLFLV
Q3MHY7	LLRREIAAVFRDNRMIAVCQNVAMSAEDKLLMRHQLRKHKI
P00403	IILVLIALPSLRILYMTDEVNDPSLTIKSIGHQWYWTYEYT
A6NNA5	SVATLRMKAREHSEAVLQSANLLPSTSSSPGPVAKPAPPDG
P14439	EPLRKHWEFEEKTLLPETKENVVCALGLFVGLVGIVVGIIL
P41272	FLIFVLGAILFFHQRRNHGPNEDRQAVPEEPCPYSCPREEE
Q9D6N1	DSSLRPLSIKYDPASAKIISNSGHSFNVDFDDTEDKSVLRG
P48230	FFGFLANILLFFPGGKVIDDNDHLSQEIWFFGGILGSGVLM
Q3UA16	QELKEEYARKRETISTANKANEERLKGLQKSADLYRDYLGL

Q28030	IGDPTHYGYSTGQPCVFIKMNRVISFYAGANQSMNVTCVGK
Q5T0J7	LHHCGTCCEKCLLCALKNNYNRGNIPSEASGLYKGGEEPVT
O70435	STFSPDGRVFQVEYAMKAVENSSTAIGIRCKDGVVFGVEKL
Q8K3A2	LQLPQFHSFSIEDVQLVVNTNEKQRFTLQPGEPSTGLLIRA
Q9EQ06	GRLTAYEFAKLNTKLVLWDINKNGIEETAAKCRKLGAQAHP
Q8WXI8	WVDQTPFNPRRVFWHKNEPDNSQGENCVVLVYNQDKWAWND
P14190	RTKLFGSLSRNDILGQIQRVNANITSLVDVAGSYRENMEYT
Q9NPJ6	YPTDLEMRSGLLGQMNNPSTNGVNGHLPGDALAAGRLPDVL
Q2KJ84	RLSKSGHIQTLLRAFEARDRNIQESNFDRVNFWSMVNLVVM
Q5E9K3	KDGFRFFTNFESRKGKELDSNPFASLVFYWEPLHRQVRVEG
P50296	IEDFWSISTYYQVSRTSVMTNTSLCSLHTKDGVHGLMGTIL
Q14002	TRQFYVFSEPPKPSITSNNFNPVENKDIVVLTCQPETQNTT
Q9D3G5	ARAFQQAMYEYNQAEMKQRDNCKIRIQRQLEIMGKDMSGEQ
P27512	PWTSCEDKNLEVLQKGTSQTNVICGLKSRMRALLVIPVVMG
Q91WB7	FEGRKEIWDALKAAAYAAEANDHELAQAILDGASITLPHGT
A7MAZ5	GVSLAALKKALAAAGYDVEKNNSRIKLGLKSLVSKGTLVQT
P98066	GIIDYGIRLNRSERWDAYCYNPHAKECGGVFTDPKQIFKSP
Q8NBR9	TMYPWGLPPSHLGSSSPFSANMEQWDYYKSQTRFAPFLPES
Q9NQ35	METNLSKLRSGPRVPWASKTNKLNQAKSEGLKKSEEDDMIL
Q96HZ4	VHTFVSTCQAIDATVAAELLNHLLESMPLREGSSFQDLLGD
Q3ZCA1	LVVQNACGHLGLKSEEICWTNSESFAAWCRFGKREFKAGGE
P31213	PGEISYRIPQGGLFTYVSGANFLGEIIEWIGYALATWSLPA
Q32KU2	PYLVYLKSHFNPCVGVLIKSNWVLAPAHCYLPNLKVMLGNL
A5PJA8	NTSDPDMRREMEQSMNMLNSNHELPDVSEFMTRLFSSKSSD
P27144	ETLKDRLSRRWIHPPSGRVYNLDFNPPHVHGIDDVTGEPLV
Q00059	ERFQEAKGDSPQEKLKTVKENWKNLSDSEKELYIQHAKEDE
P20645	QDLGNLVADGCDFVCRSKPRNVPAAYRGVGDDQLGEESEER
Q96SJ8	FRENLTREFFTKELTKHYQGNNDTDVFSATWNSVMITFGCC
O00338	TQLLPPSFWENNCKFLYVARNAKDCMVSYYHFQRMNHMLPD
Q9BRP0	DTFDLKRHVRTHTGIRPYKCNVCNKAFTQRCSLESHLKKIH
Q3SZY9	SGSVLDYFSERSNPFYDRTCNNEVVKMQRLTLEHLNQMVGV
Q99J59	DGTSFLKVFGSLSSSAMQFVNVGYFLIAAGAVLFILGFLGC
P01584	LSTFFPFIFEEEPIFFDTWDNEAYVHDAPVRSLNCTLRDSQ
Q9D1E6	LTDFKPGYWVGVRYDEPLGKNDGSVNGKRYFECQAKYGAFV
Q9CY52	LRRCLRLGVAMAKSKFEYVRNFEVQDTCLPHCWVVVRLDGR
Q86Y82	SAEERQREEQLVSFDSHEEWNQMQSQEDEVAITEQDLELIK
Q29460	RVVVLGLLPRGQHPNPLREKNRRVNELVRAALAGHPRAHFL
A3KMZ6	CQTTGLGGSAVAGHASDQIENMVPVKDRIIKITFNADVHAS
Q8IZ81	KTDFRGMGILGLINLVYFSENYTSEAHQILSRSNHPKLGYS
Q86WH2	FVLREHEIGEWEAFSLPELQNFLRILDKEEDEQLQNLKRRY
P00920	PALQPLLISYDKAASKSIVNNGHSFNVEFDDSQDNAVLKGG

O88792	SSPRVEWKFVQGSTTALVCYNSQITAPYADRVTFSSSGITF
Q8WUB2	PDLTTGQKRYLCSIAKIYNANYLKMLMKRQYMHVLQHSSQK
Q96EC8	MRDLKAVGKKFMHVLYPRKSNTLLRDWDLWGPLILCVTLAL
Q99M03	GQRKFPILEEKAFSVHGARGNHMDFGQLYQFLNARGCGDVF
Q0VCA9	AIEESTRKVHYNIFHMWRHYNFARMRKTADFFLLQSNYNYV
Q5SQ13	AFPQDLSPAWPQKGAGTHPANAGPGPLSRRSSQLRTPLEAG
Q58DS5	AMGIILVYDITDEKSFENIQNWMKSIKENASAGVERLLLGN
Q8WV48	EEIGDSQAWEEPTNWSTETWNLATSWEVGRGLRRRCSQAVA
P14138	CHLDIIWINTPEQTVPYGLSNYRGSFRGKRSAGPLPGNLQL
Q9NPA1	PCLQVFVNLSHPGQKALLHYNEEAVQINPKCFYTPKCHQDR
Q3ZBP0	EEQIMIGDGQKAGSSFQGDLNRAGKVKVDNFLDIEDLDLDE
P12034	ASAKFTDDCKFRERFQENSYNTYASAIHRTEKTGREWYVAL
P62956	TATPFPMVSLFLVFTAFVISNIGHIRPQRTILAFVSGIFFI
Q9D5U5	PNGVEAAIHCGMQVVMVPHENLSADLTRKATLVLSSLHDFK
Q8VHZ7	SRLKMFAKELKLVFPGAQRMNRGRHEVGALVRACKANGVTD
Q6ICL3	TTDVDSLSYLKKVSMEGHLYNGFNLIAADLSTAKGDVICYY
P05813	SGSNAYHIERLMSFRPICSANHKESKMTIFEKENFIGRQWE
P18917	HQWTYNLVRGSCLLLLLVMSNLLLCQGILCPSLCPDGDDVC
Q5SS90	PVGAPPASQIPGLSDLRDSPNVNLPRARRYWIKETDSEYVK
P21311	KIAADCEAFISRARVLFEIANREVAYYNIRQEFNYSTEITD
Q6QRN6	CARHLKLFGYQPTIYYPKRPNKPLFTALVTQCQKMDIPFLG
Q8BSU7	QLPPGEDLNDWVAVHVVDFFNRINLIYGTISDGCTEQSCPV
Q8VHW4	RYTAEDMYRPHPGFYRPRLSNCSDYSGQFLHPDAWIRGRSP
Q5E9C4	ILVFVAVEILLTFKIKVMALNSAEKITKFLENIPQDSSDAI
Q9D3N2	TFGMTDDMIMDRVFRGFDKDNDGCISVSEWIHGLSLFLRGT
P25393	NFNQILMQLRMSKAALLLLENSYQISQISNMIGISSASYFI
Q91WB2	DGARERRQSQQLPEEDCMQLNPSFKGIAFNSLLAIDICMSK
P01915	RRVEPTVTVYPTKTQPLEHHNLLVCSVSDFYPGNIEVRWFR
O43704	PMLEMTLPGLRTSGIEQLEKNPSPRIVKTHLPTDLLPKSFW
Q4KUS1	FDSQKHQCRAFLYSGCRGNANNFLTKTDCRNACMFVEKKGQ
Q8N5J4	SAADFYFEGNIHQSLQNITENQLVQPTLLQQKGGKGRKKLR
Q9BXN2	KIDSSNELGFIVKQVSSQPDNSFWIGLSRPQTEVPWLWEDG
Q8QZV2	LPEGTMNNVYVNMPTNFSVLNCQQATQIVPHQGQYLHTPYV
A7E2S9	CVHQQLLEYKQKISKNSQNSNPEGTSEGTPDEAAPLAERTP
Q5E9C0	TLPRGFGSLPALEVLDLTYNNLNENSLPGNFFYLTTLRALY
O95816	HSIQNSQDMRQISDGEREELNLTANRLMGRTLTVEVSVETI
P15947	WQVAVYRFTKYQCGGILLNANWVLTAAHCHNDKYQVWLGKN
P70280	TEQILAKIPSENNKLTYSHGNYLFHYICQDRIVYLCITDDD
Q10738	IIIGFARRDHGDSFPFDGPGNTLGHAFAPGPGLGGDAHFDK
Q3T189	FRRSCREGICGSCAMNINGGNTLACTRRIDTNLSKVSKIYP
P32243	LFAKTRYPDIFMREEVALKINLPESRVQVWFKNRRAKCRQQ

Q91V08	GLHRESSEHPWKWTDNTEYNNTIPIRGEERFAYLNNNGISS
P40293	RKRWQNMKFGADIQDDYEDENLYEGLNLDDCSMYEDISRGL
O88452	SQQKGRLSLQNTAEIQHCLVNAGDVGCGVFECFENNSCEIQ
Q9D9V4	LDSLEYDIPRGSWSIQMERGNALVVLRSLLWPGLTFYHAPR
Q6GTX8	PDSPDTEPGSSAGPTQRPSDNSHNEHAPASQGLKAEHLYIL
Q9D7K5	EEWIRPNNRSHFLAEIQKFQNVPVEAVDASGCAINYQGLSN
Q3SZ27	KEKTKLGSADETETPAETTHNLKHSVPLPAELSSETKTKST
Q91X52	PHKAKAMLDRIPLGKFAEVENVVDTILFLLSNRSGMTTGST
Q08DG5	EPGQRCEAISSSPPPPPCAQNPLHPSPSHSACKEPVVYPWM
P68510	KELETVCNDVLALLDKFLIKNCNDFQYESKVFYLKMKGDYY
Q5VXU1	QYRPRYITGYAVWLVLWVTWNVFVICFYLEAGDLSKETDLI
Q497Q6	IIEKVHSHKNIKKRRRQELDNFLKHSNKKGNAFIEHYDPKE
Q78PG9	ANSIQGCKMNNVNVVYTPWSNLKKTADMDVGQIGFHRQKDV
Q17QR7	HTGERPYECTECGRTFSDISNFGAHQRTHRGEKPYRCTACG
Q96S21	DRWIKEIDEHAPGVPRILVGNRLHLAFKRQVPTEQARAYAE
Q9D7W5	FNPGDTNALVAAVAFGKGLSNWRPSGSSGPGQPGQPGAGTI
Q28071	NQTDIYFCKLEVLYPPPYIDNEKSNGTIIHVKEKHLCPSPR
Q8CJC5	WAALLPEGFVRAGNVVCFWVNRRGWLFAKVNAGRPLLLRKD
Q0VD00	SPVSPLAAQGIPLPAQLTKSNAPVHIDVGGHMYTSSLATLT
Q3UF25	RIWFLDTSKQAIGMLFIHFANVYLADLTEEDPCSLYLINFL
P05305	SPRSKRALENLLPTKATDRENRCQCASQKDKKCWNFCQAGK
Q9JIQ3	FGMTLCAVPIAQKSEPQSLSNEALMRRAVSLVTDSTSTFLS
Q7M3E1	VCAGGDGVISACNGDSGGPLNCQAENGNWDVRGIVSFGSGL
Q3URF8	IFSTSCLFLKLLGSKLLYCSNGNLSSITSHLQDPNHLTLDW
Q9NVL8	LNRGPGNSKNTEFLKHQAVNNYCPWKIGKMETWLHEQEAQG
Q2KHY5	MEASCLIIQLVQEQTREPSKNPFLRKKRALVSEPALLRSVQ
Q8R2M0	LKKKMPSREVLRSTRIGHAVNKMRRHSDPEVAGLAKEVYTE
Q99627	IWSVGQRIWQRDFPGIYTTINAHQWSETVQPIMEALRDATR
Q3SWZ3	PDDEFFQKVRTIRQTIVKLENKVRELEKQQVTILATPLPEE
P17918	ARICRDLSHIGDAVVISCAKNGVKFSASGELGNGNIKLSQT
Q9Y5K2	PLYHPSMFCAGGGHDQKDSCNGDSGGPLICNGYLQGLVSFG
Q91WL6	LEEVITVRVQDPRVQNEGSWNSYVDYKIFLHTNSKAFTAKT
Q8R2N0	RGSVRKGQGFAFRRKLKIQQNYKKLLWKVKEAPASQESQFT
P53004	ETEKKSPLSWIEEKGPGLKRNRYLSFHFKSGSLENVPNVGV
P49675	MERLYEELVERMEAMGEWNPNVKEIKVLQKIGKDTFITHEL
Q3UV31	VGLSKPAEPTLPVPVQDPAPNPGWLARCWARFRDWRRVALV
Q6UX52	PANFSFLPSQTSDWFWCQAANNANVQHSALTVVPPGGDQKM
Q03358	KLKMAAKPMLPSGFSLPFPINSPLQAASIYGASYPFHRPVL
Q9D504	DTDLAHKDIYGFTAEEYASFNGFTMYHHITANNENKKKTEQ
Q24JQ0	SCCASGFLGFFLMFSTVKLKNLLAPGQCAAWIFFAKIITAG
Q0II87	RDGTSQVKLKAINENWKNLSNSQKQVYIQLAKDDKIRYYNE

Q4VAE3	IELSIGIILGISTMAAAALGNLVSDLAGLGLAGYVEALASR
Q96LT6	EELKGLGFLTFGLHILEIGENSLIVSPEHVCQHLEQVLLGT
P19652	TNATLDRITGKWFYIASAFRNEEYNKSVQEIQATFFYFTPN
P48758	AFKVNDDTPFHIQAEVTMKTNFFGTRDVCKELLPLIKPQGR
A5PLL7	HRIHHVSPHETYFCITTGWLNYPLEKIGFWRRLEDLIQGLT
Q8R1Q0	KTFIVRQLEVAGKEVSEEEVNDMLHHGKWEVFNESLLTETS
P30404	KGSTFHRVIPSFMCQAGDFTNHNGTGGKSIYGSRFPDENFK
P08294	VHAGEDDLGRGGNQASVENGNAGRRLACCVVGVCGPGLWER
Q29974	RWFLNGQEEKAGMVSTGLIQNGDWTFQTLVMLETVPRSGEV
P28585	QQKLAELERQSGGRLGVALINTADNSQILYRADERFAMCST
Q12846	VSDEELEQMLDSGQSEVFVSNILKDTQVTRQALNEISARHS
Q9Y5N6	LNSNIGIRDLAVQFSCIEAVNMASKILKSYESSLPQTQQVD
P62696	LSSLRPIKVDSQEHKIILYENPNFTGKKMEIVDDDVPSFHA
O35166	IEQIFSHLERLEILSSKEPLNRRQNAKLRVDQLKYDVQHLQ
Q8BL95	LWWAAKELRRTKKLSDYVGKNEKTKIIVKIQQRGQGAPARE
Q13907	ILIDENDNKIGAETKKNCHLNENIEKGLLHRAFSVFLFNTE
A9UHW6	REQLRARSLQGWVCYVTFICNIFDYLRVNNMPMMALVNPVY
O08992	PSLEDLKVDKVIQAQTAYSANPASQAFVLVDASAALPPDGN
Q9D883	KYGEVEEMNVCDNLGDHLVGNVYVKFRREEDAEKAVIDLNN
Q6P575	PPHPVVFLPGTGYPKGYFVQNTDFDFFSYAGLLWSLLLYTT
Q8BIX3	HVKIYIDRFEDLQKSCCDPFNIHKKLAKKNLHVIDLDDATF
Q96HV5	ILNIPIVQFFFSVLIGLIPYNFICVQTGSILSTLTSLDALF
O77512	EMADDFDHYTNTYQIYSKDLNNCQESLATSDVINWKQHLQI
Q8N7R0	TQGCLVNPTGNLPMWSNQTWNNSTWSNQTQNIQSWSNHSWN
Q91WG1	NVVIRGVVLFFIGVFLALVLNLLQIQRNVTLFPPDVITSIF
Q99801	ETLAETEPERHLGSYLLDSENTSGALPRLPQTPKQPQKRSR
O95983	GSMDLSTFDFRTGKMLMSKMNKSRQRVRYDSSNQVKGKPDL
P25731	NTGNVRVGIKDAFFCKKTSINNDDCIKKTYNKNIYPGSSFD
P16562	SLKYYYVCQYCPAGNNMNRKNTPYQQGTPCAGCPDDCDKGL
G3X9C2	HVFRQYGPGVRFVYFQHKAKNRMEPGGLRRTRVTDSSVSVQ
Q2M2T2	WKYTPMGRDAAGQLWYTGLTNSDSREAWYTLPRAPDSPYRE
Q59I47	HRSYVTRYCQGPKQVLFLGMNPGPFGMAQTGVPFGEVSVVR
P27348	TEQGAELSNEERNLLSVAYKNVVGGRRSAWRVISSIEQKTD
Q8MJ87	LVYMGFDAVAAKAALRVFRDNVQLAAQTLVHNGGRLPPDLQ
Q6PEB6	KECPAIDYTRHTLDGAACLLNSNKYFPSRVSIKESSVAKLG
Q9D241	IALALLMSTLFCCHPQSQNSNWLCPRTLFCREQKQTQITSI
Q3T0Z7	RARNWWVASIDVQENEEASANVVVKMTDSFTEQADQVTAEV
O95843	MQEKMEQKLKWYFKLYDADGNGSIDKNELLDMFMAVQALNG
Q8N1D0	EHAHKGPELTTPDSGLPRPPNPALAGFRALAQHSPPLGTST
O35164	VRKRECTQQKIKVEKYILPPNYNVSSKFNDIVLLKLKKQAN
Q6PHZ8	SISAQLEEASSTGGFLYAQNNTKRSIKERLMKLLPCSAAKT

Q2QD12	MLDSGADYLHLDVMDGHFVPNITFGHPVVESLRKQLGQDPF
Q924T2	PYIFGNRLGQDIIDLDQTALNLQLALNFTAHVAYRKGIILF
Q9D1N2	VVKAAHPQSDGANLPKSGKANQPQGSGAGYPSGWTKFGNIA
Q86UA6	EKSLQFDEKCLSIMLAEWEANPLICPVCTKYNLRITSGVVV
Q8NBQ5	IYSSAKKVKAEIGDVSILVNNAGVVYTSDLFATQDPQIEKT
Q9JKT3	LIHSLRRHIQKMQRNRTSFWNPQTEAHMGAMRLMICFLVLY
O95407	LEHASCPPGAGVIAPGTPSQNTQCQPCPPGTFSASSSSSEQ
Q9QYN3	TDGCEQRRMATESFPHPDFNNSLPNKDHRNDIMLVKMSSPV
O00584	EVQTIGQIELCLTKQDQQLQNCTEPGEQPSPKQEVWLANGA
P24592	AEENPKESKPQAGTARPQDVNRRDQQRNPGTSTTPSQPNSA
Q3TDE8	HTGERPYECTECGRTFSDISNFGAHQRTHRGEKPYRCTLCG
A8MVS5	RDRESCWAQINFDSTDMSFDNSLFTVSAKTMPEEDPATLDD
P40617	RISENQGVPVLIVANKQDLRNSLSLSEIEKLLAMGELSSST
Q2HJF5	EVEGISVGAILSDYQRVRVENVCKRLNLQPLAYLWQRNQED
Q14681	GNEDQAEFLCVVSRELNNSTNGIVIEPSEKAKILQERGSRM
Q96FV3	LGALFLAIGLWAWGEKGVLSNISALTDLGGLDPVWLFVVVG
P60487	APELLQRLARAGKNTLFVSNNSRRARPELALRFARLGFAGL
P39942	AFSLAEAKFTAGDFSTTVIQNVNKAQVKIRAKKDNVAGVTL
Q6BDI9	QKLKEYLGFEYPPSKLCPAANTLNEIFLIHFITFCQEKGVD
Q9BXY4	EANNHTMECVSIVHCEVSEWNPWSPCTKKGKTCGFKRGTET
Q6BCY4	TRRFRFGLPSPDHVLGLPVGNYVQLLAKIDNELVVRAYTPV
Q8WUE5	GPSDSGTRVLIGCVTSINEDNIYISNSIYFSIAIVSEDFVP
P06748	TVSLGAGAKDELHIVEAEAMNYEGSPIKVTLATLKMSVQPT
P55327	SQVLAAKEKHLAEIKRKLGINSLQELKQNIAKGWQDVTATS
P10415	VNREMSPLVDNIALWMTEYLNRHLHTWIQDNGGWDAFVELY
Q9CQ37	RPSLNIATVLTSIQLLMAEPNPDDPLMADISSEFKYNKIAF
Q3SZR9	VFVTEVVVVVLGYVYRAKVENEVDRSIQKVYKTYNGTNPDA
Q64008	LKVAQEIKAEYWAVSSLTGENVREFFFRVAALTFEANVLAD
Q32L49	QRHLLSGEFDQLRDFRIFESNFVQVTRLGEVANKVTMGVAA
Q9Y3Y2	PSVQAALKLKQSLKQRLGKSNIQARLGRPIGALARGAIGGR
Q8WXF0	RSKSLPRRSTSARQSRTPRRNFGSRGRSRSKSLQKRSKSIG
P12961	NKKLLYEKMKGGQRRKRRSVNPYLQGKRLDNVVAKKSVPHF
Q13868	VKRQKTHFHDLPCGASVILGNNGFIWIYPTPEHKEEEAGGF
Q8JZX9	PRLHLESPQPSPQPSPQGAGNVDVWRIPEAGSPHNGMSPEP
P47741	ALYLLRKAWRLPNTPKPCWGNSFRTPIQEEHTDAHFTLAKI
P70452	ELHEIFTFLATEVEMQGEMINRIEKNILSSADYVERGQEHV
B7ZW38	KIAGCSVHKGFAFVQYDKEKNARAAVAGEDGRMIASQVVDI
O35166	QVREQQERQRDELLSRTFTTNDSDTTIPMDESLQFNSSLHN
Q9D4F2	LDLLLVAVIKGLVRRRRPAHNQKDMFFTLSVDRYSFPSGHA
Q08D83	VDSLKLAVFMWLMTYVGAVFNGITLLILAELLVFSIPIVYE
Q9Y5N6	NKETYQSCLKSFECLLGLNSNIGIRDLAVQFSCIEAVNMAS

A6H759	LHGITFLTRNYCLAELYLNNNAIFDIEGLHYLPSLHILLLH
Q3UPR9	CSGDGLDSDGNQTLRWQAIGNPRCQGTWKKVRRVEQCSCPD
O88822	SAADYYFFTPYVYPATWPEDNIIRQTISLLIVTNLGAYILY
Q3KNV8	TVTECLHTFCRSCLVKYLEENNTCPTCRIVIHQSHPLQYIG
I3L3R5	EERKRGPMETFEDLWQRHLENGGDLQKRVAEKASDKETAQF
P06346	CRHNYEGVETPTSLRRLEQPNVVISLSRTEALNHHNTLVCS
Q925G2	LFFVLKHPSYHSFPPEGVFTNTLGLLILVFGALIFWIVTRP
P09056	AVSLQVKLNATIDVMRGLLSNVLCRLCNKYRVGHVDVPPVP
Q8N5I3	LNPYLLQPRPALVEVHFLSRNTQAFFRVFGSCSKTIEMLTG
A5PKE4	VIEDIKRWKTMLELPDQSKENLVEALRELKKKIPSREVLKS
Q8R3S2	ASKVIYTNGCIDKLVNWIHSNLFLLGGVALGLAIPQLVGIL
Q07699	VVWNGSRGTKDLQDLSIFITNVTYNHSGDYECHVYRLLFFE
Q6PCP5	GVPNASVIMQVPERIVVTGNNEDISFSRPADLDLIQSTPFK
P08218	GTILPNNYPCYVTGWGRLQTNGALPDDLKQGQLLVVDYATC
Q58DM8	RNSNVGLIQLNRPKALNALCNGLIVELNQALQAFEEDPAVG
Q9D504	NGRDKRSRTPLHLACANGYTNIVSLLIENQCKINVQDSENR
Q8K3A0	HCRALQPPDPTRDYFSLMNCNRSFRVDVTKLQHRYQQLQRL
Q9GZV9	IFGSHYFDPENCRFQHQTLENGYDVYHSPQYHFLVSLGRAK
Q2YFS1	FIYNSSMPFIHEHFKGRLILNWTQGQTSGVLRILNFKESDQ
A6H7B8	CILWRLTKKHTVSQEDRKSYNWKQRLFIINFVSFFTALAVY
Q9D7A8	RAIVQDQGCLPGLILFMDHPNPPVVHSALLALRYLAECRAN
Q148G4	ALMDLGLYIGFNGCSLKTEANLEVLKSIPSEKLMIETDAPW
Q8N966	FSPALLHGALFLFLSANALGNYVLVIQNSPDDLGACQGASA
Q96NL8	VVSYDDYMWDKSCDYLFFRNNMPEFHKLKAKLIKKKGTRAY
Q8BHL8	SQIRSGIITPIHEQWEKARANSPPREFPPATAREVDPLQIS
Q8NG11	WDESIFTKGCIQALESWLPRNIYIVAGVFIAISLLQIFGIF
Q5E9C8	RFGGWFAIRGVVLLRGTEVPNLPPTKPVDCVPTRADRISLL
Q8K396	YYWAFPSKALHARKRKLEALNSQLSEGSQKHADLQKSIEKA
P0DMR1	PPPPPIALAVVPSKRQRISGNTSRRGKSGFNSKSGKRGSSK
P14190	KLFGSLSRNDILGQIQRVNANITSLVDVAGSYRENMEYTDG
P56966	RRGFPVAHSIYGIPSVINSANYVYFLGLEKVLTLNHPDAVK
Q61754	LNPNWVLTAAHCYGNATSQYNVWLGKNKLFQREPSAQHRWV
Q9NSI2	LPELLGLEAGSRRQARSRESNKPRPSELSRMSAAQRQQLLE
Q3KNI6	KDPSTACCQELVVKIKLPNTNPSEIQIDIQEMLLDLRTPRK
B6A8C7	ILESPKPLDSTEGAAEFHLNNLKVRNAGEYTCEYYRKASPH
Q9GZX9	LSHHENLVSFLETVNQPHHQNVSVPSNNVHAPYSSDKEHMC
Q0VCF3	YKPHILLSQENTQIRDLQQENRELWVSLEEHQDALELIMSK
O08337	VTRLDRWETELNEALPGDARNTTTPASMAATLRKLLTSQRL
P62753	PRVLQHKRRRIALKKQRTKKNKEEAAEYAKLLAKRMKEAKE
Q02105	LYYFVYYTSHTANLCVHLNLNLARVASFCDHMFNSKQVSSG
Q9P0N9	LNTKYRDSLPQLPKAFEQYLNLEDGRLLTHLRMCSAAPKLP

Q8BGZ2	EFQFLHSAYATLLMKQAWPQNSSSCGTEGTFHLPVDTGTEN
Q9BZX4	ELAQMWKVVNLPTDLFNSVMNVGRFTEEIEWLKFLALACSA
P09021	APPAQPQIYPWMRKLHISHDNIGGPEGKRARTAYTRYQTLE
Q5SVD0	ERAPVAGTLEPDAAVTPIVPNPASLTHSLAAICSPRLCPLS
Q5EA33	DSKDTLELLLMNRYIKPGLKNSLEETAFDIARRTGIYHYLF
P41976	HINAQIMQLHHSKHHAAYVNNLNVAEEKYREALEKGDVTAQ
O00584	LYRELDLNSVLLKLGIKPSINYYQVADFKDALARVYGVIPK
A6NIK2	ERLPDGLCRLPRLTRLYLGGNRLLALPADFAQLQSLRCLWI
P36369	GIDAAPPLQSRVVGGFNCEKNSQPWQVAVYYQKEHICGGVL
P20160	QLDREANLTSSVTILPLPLQNATVEAGTRCQVAGWGSQRSG
Q9NVV9	KYSSICSEHFTPDCFKRECNNKLLKENAVPTIFLCTEPHDK
O15400	LGMMIHEQGDVIDSIEANVENAEVHVQQANQQLSRAADYQR
Q6NZE7	LSPSLQMFVSSSGMPPSPVLNPRHFSRRSQSPVKCIRPSVL
Q99750	PLLPNDSGHPSELGGTRRAGNGALGGPKAHRKLQTHPSLAS
Q3ZCD8	TLSERPLDFLDLERPAPTPQNEEIRAVGRLKRERSMSENAV
Q96EI5	KPEVTCTLEDKKLENEGKTENKGKTGDEEMLKDKGKPESEG
P02763	TNATLDQITGKWFYIASAFRNEEYNKSVQEIQATFFYFTPN
Q9BXU9	MKDIENIIINEEESLNETSGNCQTEFEGVHSQKQNRQTCVR
Q8IW03	HLRHHAHPHHLHHQEAGLHANPVTPCLCMCPLFSCQWEGRL
Q86V81	GTADVHFERKADALKAMKQYNGVPLDGRPMNIQLVTSQIDA
Q96GG9	KMEQELKEPGRFKDFYQFTFNFAKNPGQKGLDLEMAIAYWN
Q9Y6H3	KHSGCAVNKDRHFSCEDCNGNVSGGFDASTSQIVLCQNNIH
Q15773	MSRMLSGGFGYSPFLSITDGNMPGTRPASRRMQQAGAVSPF
P10628	RSRTAYTRQQVLELEKEFHFNRYLTRRRRIEIAHTLCLSER
A2VDH3	FESLESLQVLELNDNNLRSLNVAALDALPALRTVRLDGNPW
P41317	TLTEGVQNSCPVVTCSSPGLNGFPGKDGRDGAKGEKGEPGQ
Q9WUJ8	NKMMYQSCLKSFECLLGLNSNVGIRDLAVQFSCTEAVNLAA
Q9EQC7	CRAAPCPVPSNPGQELCGNNNVTYISSCHLRQATCFLGRSI
Q6UWV2	VGNVYKGDASISISNPTIKDNGTFSCAVKNPPDVHHNIPMT
O70552	GHWHPDCPSKGQAFRCIRINNNENKDPVLERACAESNVNFF
Q6IPT2	QRYLQSGEFDQFRDFPIFESNFVQFCPDIYPAPTSDLWPQV
P50553	VKLVNLGFATLREHVPNGAANKKMSKVETLRSAVEYIRALQ
Q8R023	CSVRAEVRHLRRVLCHRLMLNPQHVQLLFDNEVLPDHMTMK
Q9Y277	SQNNFALGYKAADFQLHTHVNDGTEFGGSIYQKVNEKIETS
Q3MHR7	SKAYIHTRMRAKTSDFLKVLNRARPDAEKKEMKTITGKTFS
Q5T0U0	VAKQQQSQASEIEKNKKVLFNLKNELHELEKEIAAISAETK
Q2KHY8	HIPSMRRNESEGDRVPCSCYNSSATNDSTIFDKISPQFSRL
Q3UFY7	MDFSEDGFLKGFKGQLIHTYNKNSSVCENSSYFQQLQNKTN
O70378	NSYVIAGYYQANERVKDASPNQVAEKVASRIAEGFGDAALI
P97436	ERKFSHQKYLSAPERAHLAKNLKLTETQVKIWFQNRRYKTK
Q8BSU7	PGEDLNDWVAVHVVDFFNRINLIYGTISDGCTEQSCPVMSG

Q5HZK2	VCSHGTSVEESTWYGFDFYPNLFYNDWLGTTTLPYNPERIP
P20783	NSPVKQYFYETRCKEARPVKNGCRGIDDKHWNSQCKTSQTY
Q7Z3S9	ETDVNECDIPGHCQHGGTCLNLPGSYQCQCLQGFTGQYCDS
Q923D2	ADVDKTVAGQEAVIVLLGTGNDLSPTTVMSEGTRNIVTAMK
Q15102	LENGELEHIRPKIVVVWVGTNNHGHTAEQVTGGIKAIVQLV
Q96EQ8	VRTRCGHVFCRSCIATSLKNNKWTCPYCRAYLPSEGVPATD
Q3T112	YYVDENGTRLSGNMFSTGSGNSHAYGVMDSGYRPDLSIEEA
Q3T113	EELLWYREDGRVDLKSGNKINSSSVCVSGISEDDNGITFTC
Q9BRU9	PHFKNAVSGSECLLSMVEEGNPHHYFVATQDQNLSVKVKKK
Q8K386	QQLAKEYGMDFYETSACTNLNIKESFTRLTELVLQAHRKEL
Q9CRC9	RLVILDNYDLASEWAAKYICNRIIKFKPGQDRYFSLGLPTG
Q8BZI6	YQGHFIVLRGYNRATGCIFYNNPAYADRMCSTSISNFEEAR
Q9DBU2	HISTTAQDHCKAIYLHVLTTNNTAINFYENRDFRQHHYLPY
Q077R2	TKISNQIHFMETEKVFFSYSNYYIEKDFVITGVFSSPPEIN
P19256	TSIYFKMENDLPQKIQCTLSNPLFNTTSSIILTTCIPSSGH
O43399	RHCGELKRRLGLSTLGELKQNLSRSWHDVQVSSAYVKTSEK
Q8VCR9	ASRRGRRGPLARLSGPEATCNSRPAARGRQRAAAPRMPAPE
P61087	LAAAEPDDPQDAVVANQYKQNPEMFKQTARLWAHVYAGAPV
Q3ZBS1	TPCSPMRRTVSGYQILHMDSNYLVGFTTGEELLKLAQKCTG
Q8WXH6	LPLPSTLRSHLKSFSMAKGLNARMMRGLSYSLTTSSTHKSS
P97371	EEKKKGDEDDKGPPCGPVNCNEKIVVLLQRLKPEIKDVTEQ
Q9Z2H6	TCCPVSWRAFQSNCYFPLNDNQTWHESERNCSGMSSHLVTI
Q9DCY0	NSLRKHLPESLKVYGTVFHINQGNPFKLKTLVDKWPDFNTV
Q925G2	IVTRPQWKRPREPGSVPLQLNGGNAECRMEGAIAISSAHSM
P37141	IDGEEYIPFKQYAGKYILFVNVASYUGLTGQYVELNALQEE
A6H789	ANNPWHCDCTLQQVLRSMVSNHETAHNVICKTSVLDEHAGR
O35975	SPERKKSNFNCFYNGSAQTVNIDFSISGSRESCVSLFLVVL
Q8R3T5	EGSTSFVRRSQWMLEQLRQVNGIDPNRDSAEFDLLFENAFD
Q8N5S3	KPSCGIVPLASPGTSAELQNNFIEYISFIHQYDARKTPNEP
P20108	VAVSVDSHFSHLAWINTPRKNGGLGHMNITLLSDITKQISR
Q8NEG2	VRAVSMPDYMVHEEFNPDQANGSYASRRGPFDFDMKTVWQR
A2VE67	EVWGDEQADFVCNTLQPGCKNVCYDHYFPISHIRLWALQLI
Q96MV1	FSAKVSPGFNSLSFKKKIEWNSRVVSTCHSLVVGIFGLYIF
Q58DF9	SDLRYFFLFCIQVEILAGEFNDSAASEMFIFHNGGVQILCK
Q13326	EIHSRVDSSLLLQSTQNVTVNARNSEGEVTGRLKVGPKMVE
P00915	PATAKEIINVGHSFHVNFEDNDNRSVLKGGPFSDSYRLFQF
Q2YDG1	QQQATPRLFSGGLQDLLLPQNQFAMFLYCFIFIHIIYVTKE
Q969E2	TLGVNLIACLAWWIGGGSGTNFGLAFVWLLLFTPCGYVCWF
Q0P5H5	FLAVQHECGPMDKGAGTGPKNEEKREKMKRTLLKDWKSRLS
Q8BR70	GGNISFFQSLCVLGYCILPLNIAMLICRLLLLAGQGPINFM
O18870	NENESQISTDESENSSRSPLNKPNNIRSRAAPWNSFLPPPP

Q2HJH8	SESSGEWLTLGTEVKKIEAINVPCTQLSMSFFNRLYDEAIV
Q92506	RGSIINISSIVGKVGNVGQTNYAASKAGVIGLTQTAARELG
Q9DAE8	ELQGLGFLEPGLHILEIGENNFIVSPEYACQHLEQTLLGTV
Q6PZD9	SPLLKAPSPAGPSHKGKKAVNKDSLEYRLRRERNNIAVRKS
Q8WW43	MFRFAYYKLLKKASEGLKSINPGETAPSMRLLAYVSGLGFG
Q9UBD9	TYDLTRYLEHQLRSLAGTYLNYLGPPFNEPDFNPPRLGAET
Q5VST6	YADIEAAWLALRTRYGIRPENVIIYGQSIGTVPSVDLAARY
Q9D939	EDMKRNPKHEIQKVMQFMGKNLDEDVVDKIVLETSFEKMKE
Q9BXJ2	GKFICAFPGIYYFSYDITLANKHLAIGLVHNGQYRIKTFDA
Q99388	LTIVIIAIVSQSLQLRKECFNPFVYGLHNRDLQSKLYPWLC
Q96E09	VRMHSSRLHQIKQEEGMDLINRETVHEREVQTAMQISHSWE
Q9CY50	EMGTSSQNDVDMSWIPQETLNQINKASPRRQPRKRAQKRSV
O60499	AVQEMKDHMVSPTAVAFLERNNREILAGKPAAQKSPSDLLD
Q07699	VTYNHSGDYECHVYRLLFFENYEHNTSVVKKIHIEVVDKAN
O09101	VPCLCLLGPNLKAWLRVFSRNGVTSIWENSLQITTISSFTG
O88456	SFLKGGGGGGGGGGLGGGLGNVLGGLISGAAGGGGGGGGGG
Q2TBU3	DYTHEFHKTKANFVAIRERENLMGSVRKDIESYKSGSGVNN
P54149	PQEALPGRKEPLVVAAKHHVNGNRTVEPFPEGTQMAVFGMG
A7MBB3	AWICILRYYQLRDWGVRKWHNQVILWTGLLCALGTSIVGNF
Q9BQI9	IHGQESRRKTSRTEIPALLVNCKCQDQLLRVAVDTGTQYNR
Q96EC8	VMFAWSIVASTAFLADSQPPNRRALAVYPVFLFYFVISWMI
Q9JJE4	TGYRPASSGSGCLRSLFYLHNELGNIYTHGLALLGFLVLVP
Q8K1I3	VYDYDPSSLQEALSASVAKVNSQSLSPYLFRATRSSLKRVN
Q6UX65	LNKAGLVLGILSCLGLSIVANFQKTTLFAAHVSGAVLTFGM
Q9NYP7	TFICSVIYLLIVWLGPKYMRNKQPFSCRGILVVYNLGLTLL
Q96A35	KQGKVVQVIRQRNWVVVGGLNTHYRYIGKTMDYRGTMIPSE
A6NLU5	PRELLHELALSVPGARSKVTNKDATKISTVRVQGNDISHRL
Q9JKY0	QEIVNIYPSINPPTLTAHQSNRVCNALALLQCVASHPETRS
Q8TCT1	AAPGQRLPESLRATYREGFYNEYMQRVFKYLGEQGVRPRDL
P31098	NSVSSEETDDNKQNTLPSKSNEDPEQTDDLDDDDDNSQDVN
Q6UX52	LIGKDGQVHLQQRPCHRQPANFSFLPSQTSDWFWCQAANNA
Q9BQY4	IWFENRRAKWRRHQRALMARNMLPFMAVGQPVMVTAAEAIT
P36369	LSLGGIDAAPPLQSRVVGGFNCEKNSQPWQVAVYYQKEHIC
Q14CZ0	RTIRREDLISFLCGKVPPPRNSRAPPRLTVVSPNRATSTET
Q08DY9	RSSFICVLLSHGEEGIIFGTNGPVNLKKLASFFRGDYCRSL
Q9Z0F1	ATALLWLSCSIALLRALASSNARAQQRAAQRRSFLNAHHRS
P49772	WIEQLKTVAGSKMQTLLEDVNTEIHFVTSCTFQPLPECLRF
Q07444	WEESLQACASKNSSSLLCIDNEEEMKFLASILPSSWIGVFR
Q96BH3	ALVPGFPCHFPFNYKNKNYFNCTNEGSKENLVWCATSYNYD
Q58DS9	SLAPMYYRGAQAAIVVYDITNTDTFARAKNWVKELQRQASP
Q8N9N7	GALPPQLCSLRHLDVMDLSKNQIRSIPDSVGELQVIELNLN

Q3SZ90	GRKVVVVRCEGINISGNFYRNKLKYLAFLRKRMNTNPSRGP
Q61806	LSNMFMEQQKFIHESLTLQKNRMEEFKSLCEKYLEKLEVLR
P01882	EMRNGNYTMVLQVTVLASELNLNHTCTINKPKRKEKPFKFP
Q9Y5K2	QMVEASLSVRHPEYNRPLLANDLMLIKLDESVSESDTIRSI
A6NI73	RLVKEGSPEPWDTQNPLEPKNKARFSIPSMTEHHAGRYRCY
Q3V2Q8	SRANRNQGRNSEPSSGSGYWNTYTELPNRRANGLYSNEGYR
Q8VHQ4	LPLPVTIKSHLKSFSMANGMNAVMMHGRSYSLASGAGGSGS
P0C646	IIPHSYCEHIARLACGNINVNIIYGLTVALLSTGLDIVLII
Q3T116	ADWIAEPVRQKMALTHKNKINRAGELILTSEYSRYQFRNLA
Q58DH1	SIPVPESNICCHYCNQMIPGNKYFHHLDRCRRVSGAVTPSP
Q5UBV8	SYPEPARLLTGSKSVCEISNNWFQSLYLGAMFSLEEGDRLM
Q96L08	ALSLSGSSSSPQAQVMVHMANPRQPLPASGLATGMPQQPAA
P09926	PELQEYTRGKKYQRLSSSFSNFDYAAFEPHIVPSTKNRHQL
Q3MHP2	IGDSGVGKSNLLSRFTRNEFNLESKSTIGVEFATRSIQVDG
Q91ZW8	VDGSPLSPSFTRYWNRGEPNNVGDEDCAEFSGDGWNDLSCD
Q62276	ETLLQSYNKRLKDDIKSIMDNFTEIIKTAKIEDETQVSRAT
Q62189	GFKEVRLVPGRHDIAFVEFDNEVQAGAARDALQGFKITQNN
Q64374	CCFGGKDYSEMYVTCARDGLNAEGLLRQPDAGNIFKITGLG
Q3TLP5	RGMEVDIASGMAIEQMCYAQNIPTQDRLEGMAAFREKRAPK
P30041	PVCTTELGRAAKLAPEFAKRNVKLIALSIDSVEDHLAWSKD
Q9JHL0	LLLGATAWLCVHCSRPGVKRNEKIYEQRNRQENAQSSAAAQ
Q9CR58	CLLQTWKNEGFFALYKGFWPNWLRLGPWNIIFFLTYEQLKK
Q8N4S7	PASSGSGCLRSLFYLHNELGNIYTHGLALLGFLVLVPMTMP
Q1JQA4	KTIDKERLSVQNVIYVRGCTNAVLMWFTDNYTIMAGVLLGI
Q8HZJ5	ENPDIVLIGNKADLPDQREVNERQARDLAEKYSIPYFETSA
Q8WUK0	TVLLGALPLRSLTRQLVQDENVRGVITMNEEYETRFLCNSS
O60762	ADLSHHPKFIPEFIRKQKEGNFDIVSGTRYKGNGGVYGWDL
Q9XT98	FAFFSAGFLVVATWTDCWMVNADDSLEVSTKCRGLWWECVT
Q3TQI7	SNQMLSGIPEVDLGIDAKIKNIISTEDAKARLLAEQQNKKK
Q5JQF8	KNLGKTIDNKALYNIFSAFGNILSCKVACDEKGPKGYGFVH
Q9NP55	GLLDSLTGILNKVLPELVQGNVCPLVNEVLRGLDITLVHDI
P30281	WMLEVCEEQRCEEEVFPLAMNYLDRYLSCVPTRKAQLQLLG
Q9NRX1	GKEETRKIPVPANRYTPLKENWMKIFTPIVEHLGLQIRFNL
Q91VT1	QSQTNFICPITQLEMKKPVKNKMCGHTYEEEAIVRMIESKH
P30048	LALRGLFIIDPNGVIKHLSVNDLPVGRSVEETLRLVKAFQY
Q96S19	AAGHLLKPRALLITYGPYAINGKISPQSNVDFDLMLRCRNP
Q9D9G2	ALDGALYLLVMVDPDAPSRSNPVMKYWRHWLVSNITGADMK
Q62276	LKQFLILNDFPSVNEAIDQRNQQLRALQEECDRKLITLRDE
Q8TED1	IVLCTVTLFLLQLKFLKPKINSFYAFEVKDAKGRTVSLEKY
Q5E9L0	TATYWANLWKTCVTDSTGVSNCKDFPSMLALDGYIQACRGL
P50289	FEGGKLQFMVQGCENMCPSMNLFSHGTRMQIMCCRNEPLCN

Q9DCZ9	VSGLGFGIMSGVFSFVNTLSNSLGPGTVGIHGDSPQFFLNS
Q2MH31	TGMESVRGMPLEYPPKQERLNAYEREVVVNMLNSLSRNRTL
O54830	RINLSEGTPGIDDDILPIYKNIETKIAELLEDSKSILSQAY
P32043	ACSAAAAPGHALGRDEAAPLNPGMYSQKAARPALEERAKSS
P51164	EEGKPCFIIKMNRIVKFLPSNGSAPRVDCAFLDQPRELGQP
Q9UKK9	AVCMDPGLSNCTIHIVTVTINGDDAENARPKPKPGDGEFVE
Q3T123	EPMDTDDSNNCIGQNEQQRENSGHRRDQIIEKDAALCVLID
Q9JJW0	THQMHSFLAAFIGGLLLFGENNNINSQINMYLTSRVLYALC
Q9CQ58	LNVMQDVPETILSKVRQIEENKRKLLEDLRWILTKVYPTAE
O55023	VYSCVEDKMYTGRKGKGAFCNGQKLQVSQQEDITKSLLVTE
P20645	INKSNGKETVVGRLNETHIFNGSNWIMLIYKGGDEYDNHCG
A5D7F5	TIGIDYGVTKVQVRDREIKVNIFDMAGDPFFYEVRNEFYKD
P0C7V0	SSALSHQGWKNTRCATRGLVNTLVNTGHFLYLQPPAPLIMP
Q3T0F5	KVIILGDSGVGKTSLMNQYVNKKFSNQYKATIGADFLTKEV
Q91VT1	EKVPDLKLLVEKKFLALQDKNSDADFKENEKFVQFKQQLRE
Q8NG11	YYRYSNAKVSCWYKYLLFSYNIIFWLAGVVFLGVGLWAWSE
Q8MJ87	ELDQIKKEEREKKKRRLENINHLKGMGYSMRAARQALHQAA
Q2KI56	ELANVQELAEGANAAYLQLLNLFAYGTYPDYIANKESLPEL
Q9CZ82	MGDKNRHALVRNCVDIATSENLTDFLMEMGFRMDHEFVAKG
Q80W35	SPEVIKIKGDLRYQRPTLPTNQQSVSTQKRQQYVNEACTYI
P0DMD0	YTRLGLTGYLLSRGVKKREINDIETVDDLAIACDSQRPSVV
P33784	VSGEVTPEAEKQVKRQLWQVNGYEWTPDYAGLTARPDAFIS
Q17QQ3	REQMAISGGFIRRVTNDARENEMDENLEQVSGIIGNLRHMA
Q810N5	NRFVELFNLTMKTQILPMNLNEESCIKDFFEQMIRNFKEMQ
Q7Z309	IHGLSDLSQVFQPYTLRTRRNSTTIMSRHSLEEGLDMVNRE
P00766	IQKLKIAKVFKNSKYNSLTINNDITLLKLSTAASFSQTVSA
Q3UTB7	GRLWSRPPLHYFHLIALALRNSPPCGLSVQQIYSFTREHFP
O75608	ESGIKQAAENIKALIDQEVKNGIPSNRIILGGFSQGGALSL
P19157	VEDLRGKYVTLIYTNYENGKNDYVKALPGHLKPFETLLSQN
Q6NXS1	EREKKRQFEMRRKLHYNEGLNIKLARQLISKDLHDDDEDEE
Q9ESJ7	NAYHMEAEVCITFTEFGKMQNICNFLVEKLDSSVVISPPEF
Q9JKY0	RLTSLGVIGALVKTDEQEVINFLLTTEIIPLCLRIMESGSE
Q9D3G5	DVHELFLQMAVLVEKQEDTLNVIELNVQKTLDYTGEAKAQV
Q3ZCC4	ERRQAKLLSNSQSLGNDLSVNTPSTQTSEAGSTGEEQKEEE
P97447	VAYEGQSWHDYCFHCKKCSVNLANKRFVFHNEQVYCPDCAK
Q08DC7	YVSILSPKEVSLDSRVREGINKKMQEPSAHTFDDAQLQIYT
Q9HB71	ERSFDLLVKNLNGKSYSMIVNNLLKPISVEGSSKKVKTDTV
Q9R0Q4	AKKNVDAILEEYANCKKSQGNVDNKEYAVNEVVGGIKEYFN
P08637	APRWVFKEEDPIHLRCHSWKNTALHKVTYLQNGKGRKYFHH
P01582	SLNQTIYQDVDKHYLSTTWLNDLQQEVKFDMYAYSSGGDDS
Q969T7	DFDMTLSRFAYNGKRCPSSYNILDNSKIISEECRKELTALL

Q99P58	DLTSQQSFLNVRNWMSQLQANAYCENPDIVLIGNKADLPDQ
Q8WW62	FGVFYEGPETDHKQKERKQLNDTLDAIEDGTQKVQNNIFHM
P61328	MHPDGTIDGTKDENSDYTLFNLIPVGLRVVAIQGVKASLYV
Q9NW97	AMWNLVPGFSAAEKPTAQGSNKTEVGGGILKSKTFSVAYVL
A5D7F5	WAESKGFLYFETSAQTGEGINEMFQTFYLSIVDLCENGGKR
P23888	GAGKSTLLRIIGGIDRPDSGNIITEHKISWPVGLAGGFQGS
Q3T169	VEVRVTPTRTEIIILATRTQNVLGEKGRRIRELTAVVQKRF
Q9D1N9	VVHFASHQWKVTAEDLILIENELDIKCGERIRLEKVLLVGA
Q9DB32	TLGTLPPETKVFCGHEHTLSNLEFAQKVEPCNEHVQAKLSW
P04925	KPGGWNTGGSRYPGQGSPGGNRYPPQGGTWGQPHGGGWGQP
O00299	IEEFLEAVLCPPRYPKLAALNPESNTAGLDIFAKFSAYIKN
Q5QGZ9	YDYWLGLSPEEDSTRGMRVDNIINSSAWVIRNAPDLNNMYC
Q8R1J3	GTGICYRCGSTEHEMSKCRANVDPALGEFPFAKCFVCGEMG
A6H7F6	TWNPFKLQYQLRNVRERIAKNLVEKGILTTEKQNFLLFDMT
P17931	TILGTVKPNANRIALDFQRGNDVAFHFNPRFNENNRRVIVC
Q6XJV4	CHWEQNNVSVVSCLVSHSTGNQSLSIELSQGTMTTPRSLLT
O15194	TNPKEDEGRLPGAGEKASQCNVSLKKQRSRSILSSFFCCFR
P55083	FANLNGFYLGGSHLSYANGINWAQWKGFYYSLKRTEMKIRR
Q9NZQ0	ALDAWLAEMKQELGPHGNMENIIFVVCANKIDCTKHRCVDE
P13661	ISKSGHKYVFVSALTGNLGSNLTSSIKAKKNAITILNTLNL
Q8C996	GLEDRGLLESSTRLKPHEAQNYRKKALWVSWLSIIVTLALA
P28231	LWHGFTMPRLVQCASIVPCPNTVDCYIARPTEKKVFTYFMV
Q3T095	GMYLEHYLDSIENLPFELQRNFQLMRDLDQRTEDLKAEIDK
P21926	LWLRFDSQTKSIFEQETNNNNSSFYTGVYILIGAGALMMLV
A4FV42	ETAEMGLQRFHKPLATFSFANHTIQIRQDWKQLGVAAVVWD
Q96BX8	EAQINNEDLFPTNVGTPFPKNFLQTVRKILSRLFRVFVHVY
P07361	HGAFMLAFDLKDEKKRGLSLNAKRPDITPELREVFQKAVTH
Q17QL1	DEYVGLPRNHPESYHSYMWNNFFKHIDIDPNNAHILDGNAT
Q8BJ25	SYALSDLDTLKKKLFLTLKENKRLRKRLKAQRLLLRRTCGR
Q9D099	WLRGLGYTSLTVFLLGFLLWNIDNIFCDSLRNFRKRVPPVL
Q5T1C6	RSFSSEEVILKDCSVPNPSWNKDLRLLFDQFMKKCEDGSWK
A6H7F6	LMEEVLLLGLKDKEGYTSFWNDCISSGLRGGILIELAMRGR
A6H793	RIPNGAFQHLHQLRELDLSQNAIETIGPAAFSGLAGGLRLL
Q6ZMS7	VLGTLLQEYGLLQRRLENVENLLRNRNFWILRLPPGSKGEA
Q96M98	PVHQPHSLVSEGFTVKAMMKNSVVRGPPAAGAFKERPTKPT
P20489	NHLQMNSTTKWIHNGTVSEVNSSHLVIVSATVQDSGKYICQ
Q9D1R1	LNIHGTLVFGTSSSLSGIMANLVFRNSFKVKYEALKTYASL
Q3ZCK5	QKDLKFLSEEEYWKLQIFFTNVIQALGEHLKLRQQVIATAT
Q99JL1	MHNYVPANSLQQKLSNWGHLNRKVLNKLNFSVPDDVMRKIA
O95833	YRESNTAGNDVFHKFSAFIKNPVPAQDEALYQQLLRALARL
Q9ULC0	PVTIPENTSQSQVIGTEGGKNASTSATSRSYSSIILPVVIA

Q2MKA7	RRVPCPEGQKRRKGGQGRRENANRNLARKESKEAGAGSRRR
P24539	VMVYGIKKYGPFVADFADKLNEQKLAQLEEAKQASIQHIQN
P48060	QFCPKVSGFDALSNGAHFICNYGPGGNYPTWPYKRGATCSA
Q9D902	KKAKVEHGGSSGSKQNSDHNNGSFNLKALSGSSGYKFGVLA
Q6UX98	PPLGPLARALQLALAAFQLLNLLGNVGLFLRSDPSIRGVML
Q765P1	VVGGVFFILGGLLGFIPVAWNLHGILRDFYSPLVPDSMKFE
Q96DX5	LLKHGAQVNGVTADWHTPLFNACVSGSWDCVNLLLQHGASV
Q9Z2H6	FQSNCYFPLNDNQTWHESERNCSGMSSHLVTINTEAEQNFV
Q9H5J8	SDSSLFKTQCIPYSPKGEKRNPIRKFVRTPESVHASDSSSD
Q3URF8	LTLDWVANVEGLPEEEYTKQNLKRLWVVPANKQINSFQVFV
P03038	WHFRNKRALLDALAEAMLAENHTHSVPRADDDWRSFLIGNA
Q61334	LITQLAKEIANKGVLKIQAENTNKAAKKFMEENEKLKLGLR
Q17RH7	STSSPSSSSSSSSSSPSSSNSSSSSSSSSPSSSSSSSSSS
Q56K12	FNVQSRLTEAKHINWRAVLSNSCLYVEIPGGALPEGSKDSF
Q5SSG5	NGHVHDLQILDFPPISAFPVNTLQEWADACCRGLRSVHAYI
A7MBI7	LQFFGRAFIVWNEFIMKPIRNLLMGSSKEQRILQHVLQHAV
Q9BSU3	NAQPDDLMNMQHCNLLCLPENYQMKYYLYHGLSWPQLSYIA
P0C7V9	PYFCRMYKECLSCWLESGIPNLGVWPKRIHTTAEKYREYEA
Q29RZ5	ERGRELPRDALVAQLLAVLSNDEAQLPDPAIEAQGREYVRP
Q92567	GYPTAYPAAAPAYNPSLYPTNSPSYAPEFQFLHSAYATLLM
P0C672	FLVLGLLFMGFGAWLLLDRNNFLTAFDENNHFIVPISQILI
A5D9C6	TARSHEFSREPSLSWVVNAVNCSLFSAVREDFKALKPQLWN
Q60931	QLHTHVNDGTEFGGSIYQKVNERIETSINLAWTAGSNNTRF
P51504	LGTGDGLHSQVLQEQVSTGDNLHECDSQGPSKDTLVREGKT
O70338	NGRKRARAGIGVYWGPGHPLNVGIRLPGRQTNQRAEIHAAC
Q3MHP2	YRIVSQKQIADRAAHDESPGNNVVDISVPPTTDGQKPNKLQ
O95750	SEKHRLPVSLSSAKQRQLYKNRGFLPLSHFLPMLPMVPEEP
O95633	AECCASGNIDTAWSNLTHPGNKINLLGFLGLVHCLPCKDSC
Q8BW00	RHSVGMAVLGQIARRLGVAENWTRDSRCAADLALAPLGDAQ
Q15116	AVLQLGWRPGWFLDSPDRPWNPPTFSPALLVVTEGDNATFT
A2ADA5	RGNPRAVGVLNFLEEAAKRLNSVDPVRFTISSRTDAGVHAL
O70255	TALCPTEAVEIYTSGALEAVNGTDVRLKCTFSSFAPVGDAL
P37237	EDRPFGQRSRAGKNFTNPAPNYPEEGSKEQRDSVLPKVTQR
Q9D720	HCYQVHDRNATVDKLEDFINNINSVLESLYIEIKKGVTEDD
Q60931	HAYTDTGKASGNLETKYKVCNYGLTFTQKWNTDNTLGTEIS
Q5JUR7	VPNKSLTYGIIILTHGASGDMNLPHLMSLASHLASHGFFCLR
O95416	EVPHTLATGALPYASTLGYQNGAFGSLSCPSQHTHTHPSPT
P41217	VFWKVPRSGIENSTVTLSHPNGTTSVTSILHIKDPKNQVGK
O43763	QDALPRPLRPPLPPDPLCLHNSSLFALQNLQPWAEDNKVAS
Q9WVL0	KYCVGDEVSMADVCLVPQVANAERFKVDLSPYPTISHINKE
O54836	KMVATRRNMNPVQSNSGPYFNARSRQRIPRDLAMCVTPSGQ

P0C7W6	AIKKQMIEEEDKFIKEITDFNNDYEITKKRELLMKENVKIE
Q2KI39	KEGDEPITQWKGTVLDQVPINPSLYLVKYDGIDCVYGLELH
P06748	KMQASIEKGGSLPKVEAKFINYVKNCFRMTDQEAIQDLWQW
P46782	GRYAAKRFRKAQCPIVERLTNSMMMHGRNNGKKLMTVRIVK
Q8NGY7	HTVVGHFFCDILPVMKLSCINTTINEIINFVVRLFVILVPM
Q0VB26	PILNESQYHDEYTWKLRSKENMVKTGTSRGVWNHKTHPGQE
Q58DS3	LGTYGLAEAGGYLHTAEGTLNPVRSAAAGALAGVMGAYLGS
Q9H8W4	EGVLTKLCRKKPKARQFFLFNDILVYGNIVIQKKKYNKQHI
Q9DB29	NTRWAKIILPRLIRKGPGMENPVAVTIFFGANDSSLKDENP
Q3ZBV0	CACLALNQEQQAQLLEVGWNNTASARDDIQRNLNCCGFRSF
P81269	RKILKDLSSEDTRGRKGEGENPSISAITSMSVPAPIYQTSS
P60153	IEPGMPLNHIEYCNHEIMGKNVYYKHRWVAEHYFLLMQYDE
Q96C19	MELKLMMEKLGAPQTHLGLKNMIKEVDEDFDSKLSFREFLL
Q077R2	IDKEDYALWLNILSKGIKARNTNLVDTYYRVHAGSVSANKF
Q9R0M5	GLKWNLTNDVLGFGTLVSTSNTYDGSGLVTVETDHPLLWTM
P79124	ISGLERGLGTFDSTIEIIFQNLKTELESRCLNDVVEETQQT
Q9GZN2	FINARRRLLPDMLRKDGKDPNQFTISRRGGKASDVALPRGS
P00639	RDSHLVAVGKLLDYLNQDDPNTYHYVVSEPLGRNSYKERYL
Q8N4C7	REPVAERHLHEIQKLQESINNLADNVQKFGQQQKSLVASMR
Q8NDY6	NYILMQAQALDEMRRLVAFLNQGQGLAAPVNAAPLTPFGQA
Q3URK1	KGVDPSKDVSVPCHDQELSTNKTEDSGVSSQDGERGSAPAN
Q8R0F8	TQSCTMASTKPLSRFWEWGKNIVCVGRNYADHVKEMRSTVL
Q8R0A6	DTKPRKNASSVVPSSVHNSANQRMHSTSSPQAVAKIPKQSP
Q9BQT8	LEFWRKFGIGLLSGTIASVINIPFDVAKSRIQGPQPVPGEI
Q3TT38	EQDQDRLSQSSVNLSPSSPANNLSVVTYSKGLHGPYPFGQS
Q9Z0S7	GILVLIPVCWTAHAIIQDFYNPLVAEALKRELGASLYLGWA
Q60595	LSRKLTSDAYKLKKQAETLSNMFMEQQKFIHETLTLQKNRM
A6QNY1	TEVPNGSPSPCDIMDDCPDHNKNLLNCGPQSCPEGELCCPL
Q9H7E9	ADSRAHPLGDEGGTASKKQKNKKKTRNRASVANGGEKASEK
Q32KP7	VSLFSELEAKQLRKLYKYTKNNQTTKFLMAFCPLDAPESSL
P25393	ADAFNVSEITIRKRLESENTNFNQILMQLRMSKAALLLLEN
Q8K0Z7	SIRLAVKEGGPNPENNSSLANILELCRSKNMPKSTIESALK
Q3SZX8	KMERFPDLEAEKECRDHEERNEKKAQIQEMKRREKEEMKKK
Q8IUA0	KKCMDPFQEPCMLPVRHGNCNHEAQRWHFDFKNYRCTPFKY
Q8WVE0	LQEFYAEQKQQIEPGEDDKYNIGIIEENWQLSQFWYSQETA
Q8K1A0	GAGLCVGFDIDEDALEIFNKNVEEFELTNVDMIQCDVYSLS
Q61200	ESSKDLSISRLLSQTFRGKENDTDLDLRYDTPEPYSEQDLW
P27144	AVILGPPGSGKGTVCQRIAQNFGLQHLSSGHFLRENIKAST
Q92637	PTPVWFHVLFYLAVGIMFLVNTVLWVTIRKELKRKKKWNLE
P56966	ILRQRTENIDIKKYCVHYLENVGSFEYTRNTLKELESKAYK
Q9ZIS7	CTAERKARDRLAMERHLGIANEIKDYGYYSVIYRTKLRKFI

P18021	VPDLRRKMLLATLWNTGARINEALALTRGDFSLTPPYPFVQ
P35452	EFLVNEFINRQKRKELSNRLNLSDQQVKIWFQNRRMKKKRV
P30279	SIKPQELLEWELVVLGKLKWNLAAVTPHDFIEHILRKLPQQ
Q96HA9	MEAQMQSEALSLLSNLADLANAVHWLPRGVLWAGRFPPWLV
P07203	LMTDPKLITWSPVCRNDVAWNFEKFLVGPDGVPLRRYSRRF
P01904	GALANIAVDKANLDVMKERSNNTPDANVAPEVTVLSRSPVN
A8E4L3	EYFYYVDHQGQLFLDDSRMKNFTTCFKDPQFLVMFFSRLRP
P53811	IETWHKPDLGTLENVHGLDPNTWKTVEIVHIDIADRSQVEP
Q8K136	LKTESKTEAPLLTQTRIITQNALHRAPHYNSCCRRKHRPEA
A0PK11	LVVPHWLSGKILCQTGVDLVNATDRELVKFIGDIYYGLFRG
Q6ZVK1	NGHDFRGRCLLFTEGMWLSANLTVQERERFTVQEWGPPAAC
Q3T0F5	KTLDSWRDEFLIQASPRDPENFPFVVLGNKIDLENRQVATK
Q9QZN4	PVEDWKVFYILCSLQRNLLRNPCAEENLSSWRIDSNGGDRW
Q60817	EEVDETGVEVKDIELVMSQANVSRAKAVRALKNNSNDIVNA
Q86VZ6	PVPGCKKRYKNVNGIKYHAKNGHRTQIRVRKPFKCRCGKSY
Q93LM8	AFLRQIGDNVTRLDRWETELNEALPGDARDTTTPASMAATL
P56404	LGIEIILTMLLVLAVCMGAVNEKTMGPLAPFSIGFSVIVDI
P18468	RWFRNGKEEKTGIVSTGLVRNGDWTFQTLVMLETVPQSGEV
Q96MV1	GVLAYIGNFRLLAELSSPFVNQRWFFEALKYPKFSKAIVIN
Q9ET43	LSFLCGIASVAGLFAGTLLPNWRKLRLITFNRNEKNLTIYT
Q8VBV8	LRGSLEHKLKWTFKIYDKDRNGCIDRLELLDIVEAIYKLKK
P80217	LRLSEEELLDKLEIFFGKTRNGGGDVDVRELLPGSVMLGFA
O54917	IELVEKKSKNHIRWIGSDLNNFGAAPQQKKLQAELSDLSAM
Q5E9M6	VDHGNGTFSVYFRHNSTGQGNVSVSLVPPTKIVEFDLAQQT
O35381	DLSTIEPLKKLENLKSLDLFNCEVTNLNAYRENVFKLLPQV
O14972	FPLHLKGNKVLYETYHGVFVNIQYTLRCDMKRSLLAKDLTK
P62242	WGSECCTRKTRIIDVVYNASNNELVRTKTLVKNCIVLIDST
Q15475	ETSYCFKEKSRGVLREWYAHNPYPSPREKRELAEATGLTTT
Q9CQ29	RLPCSHIFCKKCIFRWLARQNTCPCCRKEVTRRKMVEVNKL
P19387	LDVRCNEDQTRHVTSRDLISNSPRVIPVTSRNRDNDPNDYV
Q505G4	PGASAQMDDVVDTLTSLRLTNSALRREASTLRAEKANLTNM
Q9BY08	VCELYGCWMTFLPEWLTRSPNLNTSNWLYCWLYLFFFNGVW
Q0IIG7	AKTSMNVNEIFMAIAKKLPKNEPQNPGANSTRGRGVDLTEP
P46638	DEYDYLFKVVLIGDSGVGKSNLLSRFTRNEFNLESKSTIGV
Q9CZJ0	VKQDYYRFPSVSKLKPEDFDNVQSLLTNSIYLQDSEVTVKG
Q17QN4	YENEVLQLEEDERFEGRVVWNGSRGTKDLQDLSIFITNVTY
P24071	QLMIIKNSTYREIGRRLKFWNETDPEFVIDHMDANKAGRYQ
P82923	TVGVVDTNCNPCLITYPVPGNDDSPPAVQLFCQLFQTAVTR
Q9D9W1	GMYQKVDTLSEKHKKSFAPFNILMPRFRSAAKGDSYPGPGT
Q86UF1	MCGQGMQAFDYLEASKVIYTNGCIDKLVNWIHSNLFLLGGV
P14191	AYFVLPMKNAEGTKVGSVKVNASYAGALGRGGVTSADGELM

Q9NP90	YRGADCCLLTFSVDDRQSFENLGNWQKEFIYYADVKDPEHF
Q56P03	MFVMNCSINKEEVLRYKASENRKKRRVHKKMRSNREDAAEK
Q17R16	LAFVLGNRQTDLLQEELKQENKDFVGSTVSSVLRPGGDVSG
P08883	KLESKAKRTKAVRPLKLPRPNARVKPGHVCSVAGWGRTSIN
Q01362	GAGEELKGNKVPEDRVYEELNIYSATYSELEDPGEMSPPID
Q29S11	TKKDDSHSAEDSEDEKEDHKNVRQQRQAASKAASKQREMLM
Q6PHN9	LLIIGDSGVGKSSLLLRFADNTFSGSYITTIGVDFKIRTVE
Q9NZD2	RFIQVFLQSICDGERDENHPNLIRVNATKAYEMALKKYHGW
Q7TSP5	APRWFPQPTVAWASQVDQGANFSEVSNTSFELNSENVTMKV
Q9BSM1	LNLCLERLSSGKDKNKSVLQNKYVRCSVRAEVRHLRRVLCH
Q9H2W2	RERLAALTLLPESRIQVWFQNRRAKSRRQSGKSFQPLARPE
Q8WU03	LYKSLEKSIPESIKVYGAIFNIKDKNPFNMEVLVDAWPDYQ
P54368	LHSRGGSSSESSRVSLHCCSNPGPGPRWCSDAPHPPLKIPG
A6NJ46	GAGAGAGGDRAPSENEDDEYNKPLDPDSDDEKIRLLLRKHR
P43275	GVSLAALKKSLAAAGYDVEKNNSRIKLGLKSLVNKGTLVQT
O55135	IPVVHASIAGCRIIGRMCVGNRHGLLVPNNTTDQELQHIRN
P53026	CDERKAVDIPHMDIEALKKLNKNKKLVKKLAKKYDAFLASE
Q9QZE7	EAVSFQHFIKTRSLISMEEINKQLTFTAEDSGKESKTPPAE
P23560	KCNPMGYTKEGCRGIDKRHWNSQCRTTQSYVRALTMDSKKR
Q9JL16	LPVSAEQLAGPLLCGRCQGHNGALQNLSATQSPARAALPWD
P07738	NERIAPEVLRGKTILISAHGNSSRALLKHLEGISDEDIINI
Q9D8M4	ELILKRGQAKINNKTVPLTDNTVIEEHLGRFGVICLEDLIH
Q2TJ95	SSSSDSKGLESSIETPDQQENKERQQQQKRRARDKQQKSVS
P11456	VCREAGNHSSGAGLVQINKSNGKETVVGRFNETQIFNGSNW
P01131	IVLPIALLILLAFGTFLLWKNWRLKSINSINFDNPVYQKTT
P13284	YKTGNLYLRGPLKKSNAPLVNVTLYYEALCGGCRAFLIREL
Q2YDL1	FQILHHTCQRYLTDRKPEFINCQSKIMGGNSILHSAADSVT
Q29RK9	SLNSGVDLKAAVQLPSGEDQNDWVAVHVVDFFNRINLIYGT
P11843	IGRQWEICDDYPSLQAMGWPNNEVGSMKIQCGAWVCYQYPG
Q32L00	WHPVLLMLKNQIEENTGHSFNSLLCNLYRNEKDSVDWHSDD
Q5TGL8	GPLRQGLVAIKEAHDIETRLNEVEKLLKTIISMPCKYSRSE
Q53RY4	SVGSGLLSVSVGLVALLASRNLLRPPLHWVLLALALVNLLL
Q9D1F4	SVPVWAFKEKRTEARSSDEENGPPSSPDLDRIAASMRALVL
Q14331	GPREQWEPVFQNGKMALLASNSCFIRCNEAGDIEAKSKTAG
Q9DCK3	VGLTNAWSIIQTDFRCCGVSNYTDWFEVYNATRVPDSCCLE
P41439	RWWEDCRTSYTCKSNWHKGWNWTSGINECPAGALCSTFESY
P17981	VAYTGNIHRSLNEATREFKINPGNFHYKAFHYYLTRALQLL
P20033	CKTRTVIYEIPRSQVDPTSANFLIWPPCVEVKRCTGCCNTS
Q99623	AQNISKTIATSQNRIYLTADNLVLNLQDESFTRGSDSLIKG
Q96KN8	AVVKYSRLEDVLHGCSWKVNNKLDGTYLPLPVDKIIQRTKK
Q9H400	DLAYQTLPLRALDVDSGPLENVYESIRELGDPAGRSSTCGA

P53811	SKNETGGGEGIEVLKNEPYENDGEKGQYTHKIYHLKSKVPA
P81126	KLHMQLQSKHDSATSFVDAGNAYKKADPQEAINCLNAAIDI
P04390	VYTRVATRKSSLKTYNINELNEIPKPYKGVKVFLQDKWVIA
Q96FV3	VVGGVMSVLGFAGCIGALRENTFLLKFFSVFLGLIFFLELA
P23185	METGSMYQNAVLVADQIGLKNRVWAGYTDSYVAKTMNLDQR
Q5T681	HFSRLSEEKLALDNNASASGNATQTESGSEEVSSTVHIETF
O95297	AGDLDKKDASINIENMQFIHNGTYICDVKNPPDIVVQPGHI
Q9ERI2	QSFLNVRNWISQLQMHAYCENPDIVLCGNKSDLEDQRAVKE
Q16740	IAQLLFLQSESNKKPIHMYINSPGGVVTAGLAIYDTMQYIL
P53519	SIRGCIKLIYRPASVPSPVFNNIVEKLKWHKNGKYLVLKNN
Q9JM71	HPDYNMSLLNDHIPHPEDKSNDLMLLRLSKPADITDAVKPI
A6NJ46	RARLAYSLGMTESQVKVWFQNRRTKWRKKSALEPSSSTPRA
Q8WU03	EIPFYFHVADNNEKSLQALNNLGFKICPCGWHQWKCTPKKY
P43464	GIIVADADDSVLDTFKSIDHNDDSRITSDLIYLISKIENNR
Q148M8	VPGIVYLGHIPPRFRPLHVRNLLSAYGEVGRVFFQAEDGFV
P62079	EYWQCCGAFGADDWNLNIYFNCTDSNASRERCGVPFSCCTK
P00766	RYTNANTPDRLQQASLPLLSNTNCKKYWGTKIKDAMICAGA
O95156	VHATEGLDWEDKDAPGTLVGNVVHSRIISPLRLFVKQSPVP
P40261	KEPEAFDWSPVVTYVCDLEGNRVKGPEKEEKLRQAVKQVLK
Q8TAA3	VTVEYITRFIATLKQKYTQSNGRRPFGISALIVGFDDDGIS
P06344	RWFRNGQEETVGVSSTQLIRNGDWTFQVLVMLEMTPRRGEV
Q3ZCD0	ETLNCCGSNTLMTLTTSVLKNSLCPSSGNVITNLFKEDCHG
P17931	PLPGGVVPRMLITILGTVKPNANRIALDFQRGNDVAFHFNP
Q99990	PNQWRYSSPWTKPQPEVPVTNRAANCNLHVPGPMAVNQFSP
P50538	EHGYASMLPYSKDRDAFKRRNKPKKNSTSSRSTHNEMEKNR
Q92520	VFCGGKGIKTKSPFEQHIKNNKDTNKYEGWPEVVEMEGCIP
F7BWT7	MELIGGIVALIFRNQTIDFLNDNIRRGIENYYDDLDFKNIM
P41976	LQPALKFNGGGHINHSIFWTNLSPNGGGEPQGELLEAIKRD
P05839	FFLFCALGFTEMLCSDRYFGNKVEDYVVTFGSWFLLLAPGI
Q9UMY1	SQTNIKKSPGKVKEVNLQKKNEDCEKGNDSKKVKVQKVQSV
Q924T2	LLFGPSVRLPDLIIFLHTLNNVFEPHVAVRDAAKMNIPTVG
O60637	VFVTEVVVVVLGYVYRAKVENEVDRSIQKVYKTYNGTNPDA
Q08DZ3	KTDFRGMGILGLINLVYFSENYTSEAHQILSRSNHPKLGYS
Q3UP87	VPRRQAGICFGDSGGPLVCNNLVQGIDSFIRGGCGSGLYPD
Q9ULC3	KIGVFNTSGGSHSGQNSGTLNGGDVINLRPNKQRTKKNRNP
O77512	KTVVIRPQEQEMADDFDHYTNTYQIYSKDLNNCQESLATSD
P41217	LSHPNGTTSVTSILHIKDPKNQVGKEVICQVLHLGTVTDFK
P07903	LRYHNLHPDYIHERLQSLGKNFALRVLLVQVDVKDPQQALK
Q3ZBP0	GKVKVDNFLDIEDLDLDEEINPQLSKDVLLLPGQVEQEVSL
O35526	PDEKTKEELEELMSDIKKTANKVRSKLKSIEQSIEQEEGLN
P14148	NKASINMLRIVEPYIAWGYPNLKSVNELIYKRGYGKINKKR

Q14406	EDGSHLTGQTLKQTYSKFDTNSHNHDALLKNYGLLHCFRKD
O43930	RFLYMLMEYVPGGELFSYLRNRGHFSSTTGLFYSAEIICAI
Q0VBU9	RTDKTEIKEAFLAVSLALALNGVCTNTIKLIVGRPRPDFFY
P05820	NITLKTEGTLTISANGSWTYNGVVRSYDDKYDFNASTHRGI
Q6P6J4	VEVVRLQLAHGSLLMMNPPTNTHWYHSLPIRKRVLAPRVNL
P04224	AVDKANLDVMKERSNNTPDANVAPEVTVLSRSPVNLGEPNI
Q3ZBE1	DSFNEMNSRRRKAQFFLGTTNKRAKTVVLHIDGLDDTSRRN
Q8VC88	SGQIPGMQMQMGQPMPGAGPNMFSGGYPGYLGYSDSYSPAD
Q3SYV5	AGVLGFVFSDKVRGKVSEIINNAIVHYRDDLDLQNLIDFGQ
Q6NXT1	DPCAADDKGRTALHFASCNGNDQIVQLLLDHGADPNQRDGL
Q32L31	RPPSGFFLFCSEFRPKIKSANPGISIGDVAKKLGEMWNNLS
P06345	RLEQPNVVISLSRTEALNHHNTLVCSVTDFYPAKIKVRWFR
P20719	SYFVNSFCGRYPNGPDYQLHNYGDHSSVSEQFRDSASMHSG
Q9H008	SNPNCVVIADAGESFSYQNMNNAFQVLMELEKPVLISLGKG
P62491	YRIVSQKQMSDRRENDMSPSNNVVPIHVPPTTENKPKVQCC
P45379	EEEENRRKAEDEARKKKALSNMMHFGGYIQKQAQTERKSGK
Q3LUH2	QQLNKTFSYFLGLSDPQGNGNWQWIDQTPYKENVRFWHQNE
Q9GZU8	SQENKKEVEKKLTVKPIETKNKFSQAKLLAGAVKHKSSESG
P05538	NNYKDFLEQERAAVDKVCRHNYEAELRTTLQRQVEPTVTIS
Q9WU03	WWYNITDGSCQPFVYGGCEGNGNNYQSKEECLDKCAGVTEN
P13972	VDVFSIPACVRNTNGDLSATNEKFSKEFIGSLDIKEWFYSL
Q32KY9	EFAMMDRELNHYLKAVQSTINHVKEERSEKIPDLKLLVEKK
Q96DX5	HEAARRGHVECVNSLIAYGGNIDHKISHLGTPLYLACENQQ
P06717	GGLMPRGHNEYFDRGTQMNINLYDHARGTQTGFVRYDDGYV
Q6P3D7	ANYYGMSPTENPLYDWRGVTNGSADLYLEGGFHQSVQNIAE
P01920	NSQKEVLERTRAELDTVCRHNYQLELRTTLQRRVEPTVTIS
Q14002	TLVLLSATKNDIGPYECEIQNPVGASRSDPVTLNVRYESVQ
Q8CD60	DSVDGPEAPRPGTGPDYQLMNGGLPIPNGPRVETPDSSSEE
P41439	NACCTASTSQELHKDTSRLYNFNWDHCGKMEPTCKRHFIQD
Q8N6D2	DNNILVNLTCGGKGKKCLPENPTELLLTPKRLASLVSPSHT
Q9D287	WQECVNNSMAQLEHQAVRIENLELMSQHGCNAWKVYNENLV
Q9BQD7	AGSVCYRRKDLWKVSLRDCRNVSVFLAPSVLPLLEDKLRTE
Q6UWN0	KFQAGFLNTTFLLMGCAREHNQLLADFHHIGSIKVTEVLNI
Q9QY33	VFVTEVVVVVLGYVYRAKVENEVDRSIQKVYKTYNGTNSDA
Q9XSK0	LFAKTQYPDVYAREEVALKINLPESRVQVWFKNRRAKCRQQ
Q8C5C9	NSAEDPSPAQVGKESPWSPCNKSVVGKCKLWMVIVTIFLCF
Q3SZX8	FHRQKDVKIVTVEKKVNEILNRLEKTKMERFPDLEAEKECR
Q9HBJ8	WLLFFLVTAIHAELCQPGAENAFKVRLSIRTALGDKAYAWD
A4FV54	AMGIMLVYDITNEKSFDNIRNWIRNIEEHASADVEKMILGN
A5PJK7	ENVLGGHAPSPTAVAAAENGNREPRPSLPFQCPKDDAGSWG
Q6UXN2	SGFYWCGIYNASENIITVLRNISLVVSPAPTTSPMWTLPWL

P13949	YRNSPVPVCVRNKNRKILYANGAFIELFSREDKPLSGESYI
Q9H446	AEEAEKQLFHGTPVTIENFLNWKAKFDAELLEIKKKRMKEE
Q148K5	VLLLARVLRGHPPLLLDVVRNACDLFIPLDKLGLWRCGPGI
Q01081	EVFTEMEEKYGEVEEMNVCDNLGDHLVGNVYVKFRREEDAE
Q9JL62	AALYAAPYKSDFLKALSKGQNVTEEECLEKIRLFLVNYTAT
P07738	NRFCSWVDQKLNSEGMEEARNCGKQLKALNFEFDLVFTSVL
Q08DH5	ESAHKSQRALDDCKMLVQEFNTQVALYRELVISIGDVSVSC
Q9BT09	TETICKRLLDYSLHKERTGSNRFAKGMSETFETLHNLVHKG
Q9H7V2	QKSMLVHSKISDAGKRNGLINTRNLMAESRDGLVSVYPAPQ
Q5EA90	LILPPNPAFGSKAASYSSMGNSRPFFSCVPCERAAGAGFVT
Q9CPX5	DGTYLPLPVDKIMQRTKNMINKIVQYSLIEGNCEHFVNDLR
Q96ET8	ALYWKPAPRLSSPPLRAAPGNCQQMAPARLFLSLRLWAWRG
P07743	QNLNLDVELLQQATSWPLAKNSILETLNTADLGNLKSFTSL
Q8NGA4	HLKFRTTRKWNGCMQCYLQFNLENETAQMWTQEVFGRQMAV
Q9CR57	KFPHSARQKYVRKAWEKADINTKWAATRWAKKIDARERKAK
Q3ZBP0	DEEFDYDAVVLTPKFTPAEMNAIKELSKQKTKSADLEDPHD
Q8R1J3	DGGSCKLCGSVEHFKKDCRENQNSDRIITVGRWAKGMSADY
Q32PI9	TMILAVIYRRRNSKRDYAGCNTSENVSPVKQVSRKSPSDTE
Q8N9E0	KQLENKKTGSKALAEFEEKMNENWKKELEKSREKLLSGNES
Q3SZ16	PPPLCEAAWAQECLQQGCKLNRLNSVVGEYARACLQVAQDC
O88396	LEKEVQDLTLRYQRAVADCENIRRRTQRCVEDAKIFGIQSF
P46412	ENSEILPSLKYVRPGGGFVPNFQLFEKGDVNGEKEQKFYTF
Q9CZQ6	PWCDVKCSVDEITILHLNNINKTMTSGDPGKMANATGKCLT
Q9NWU2	GQIQEAIALINSLHPELLDTNRYLYFHLQQQHLIELIRQRE
Q6GTX8	PDTEPGSSAGPTQRPSDNSHNEHAPASQGLKAEHLYILIGV
P62609	KAGNKSLENTMVPPQGKVTVNIPGGYTGGDITYKTINDYGA
Q5E9C8	TKPVDCVPTRADRISLLERFNFHWRDWTYRDAVTPQERYSE
Q53TN4	VAAILAIISVVAVFENHNVNNIANMYSLHSWVGLIAVICYL
Q9Y277	QKVNEKIETSINLAWTAGSNNTRFGIAAKYMLDCRTSLSAK
Q0VC58	GGGILSCVLLAEPPLRFLANNTNILLASSIWYIAFFCPCDL
Q8WY22	VVLFSMSCVYILHKYEGEPENAVLPLCFVVAVYFMTGPMGF
Q2KIA1	NFSKPEAAARARIELHETDFNGRKLKLYFAQVQMSGETRDK
Q3T040	RKQFEKYHAASAEEQATVERNPYTIFHQALKNCEPVIGLVP
Q6P047	VKEVFQLQRPQGRERLRRLLNWEEFDEQRDSRRSILLDTLY
P58499	SLLFMVTYDDGSTRLNNDAKNAIEALGSKEIRNMKFRSSWV
O14618	LHVHQYGDLTNNCNSCGNHFNPDGASHGGPQDSDRHRGDLG
Q00169	SVAEASKNETGGGEGVEVLVNEPYEKDGEKGQYTHKIYHLQ
Q9EQ00	HKQCSHKQYVELADLEKKWKNLCLPVEKLRTILELDPCEDK
A0JN86	TMEFIYMTRWRLRGENFRCLNCSASQVCSQDGPLYQSYPMV
Q0VC58	GWIVMIAVGWARGAGGSIITNFEQLVKGCWKPEAEEWLKMS
O00560	EVILCKDQDGKIGLRLKSIDNGIFVQLVQANSPASLVGLRF

P15173	KRRLKKVNEAFEALKRSTLLNPNQRLPKVEILRSAIQYIER
Q9NX20	AVSRGTLEKMRKDQEERERNNQNPWTFERIATANMLGIRKV
Q5U4E0	LAIIGILNALILSFLAFVLGNRQTDLLQEELKQENKDFVGT
Q9CZD0	VTEKFPEVHMEVIADYEVHPNRRPKILAQTAAHVAGAAYYY
Q2KHU3	VTINEETALAEVNLKKKSCLNIRTHPVATSFAVFDDTLLIV
Q9BZD6	AGVFLVIFGLLGYYLCITKCNRLQHPCSSAVYERGRHTPSI
Q80SV1	AGCALYPLGWNSPEVMQTCGNVSNQFQLGTCRLGWAYYCAG
P62593	HLTDGMTVRELCSAAITMSDNTAANLLLTTIGGPKELTAFL
P09024	AGQSAAGVYAAGYGLEPSSFNMHCAPFEQNLSGVCPGDPAK
P00918	LSVSYDQATSLRILNNGHAFNVEFDDSQDKAVLKGGPLDGT
Q9D0L6	AQNHSGPAMPTLECCHEDMCNYRGLHDVLSPSKSEASGQGN
O54907	QDGAQAGVDGTVSGWEETKINSSSPLRYDRQIGEFTVIRAG
Q8WV22	KALELIIDSETGFASSTNILNLVDQLKGKKMRKKEAEQVLQ
Q7YRZ7	RRYMALIKGLKLCAGALIKENWVLTAAHCDLKGNPQVILGA
Q0V7M7	HLKENIPPHLPQVTVTQNFVNGSDLDPEEPVKVEEPAPTKK
P53516	ATETNARVFSLHLGATRVVYNPASSGETLTVINDQDYPMLV
P07516	LKAVQRIAESHLQSISNLGENQASEEEDELGELRELGYPRE
P13726	ARTLLLGWVFAQVAGASGTTNTVAAYNLTWKSTNFKTILEW
P50591	KGFYYIYSQTYFRFQEEIKENTKNDKQMVQYIYKYTSYPDP
Q3TT38	EAWPPGVCLKYVGGDQFGHVNMVMVRSLEPQEIADVSVQMC
Q3SZW3	KSCQKPVDKYIEYDPVIILINAILCKAQAYRHILFNTKINM
Q9D6D8	KKRELKGETWVYSSYVMCFVNDQLLGNAFDLKKWAQKVWDV
O54901	SSITFWNTTLEDEGCYMCLFNTFGSQKVSGTACLTLYVQPI
O75845	TLSYYFVFDHALMKHPQFLKNQVRREIKFTVQALPWISILT
Q9Y255	INHARLMVVEERCVYCVNSDNSGWTEIRREAWVSSSLFGVS
P00757	SGWGSTTPIKFKYPDDLQCVNLKLLPNEDCDKAHEMKVTDA
Q4U5R3	DEKKKGEDEDKGPPCGPVGCNEKIVVLLQRVKPEIKDVIEK
Q9H8U3	KCFADFQKKQPDDDSAPSTSNSQSDLFSEETTSDNNNTSIT
Q9D172	VPQMHVIDHTKGEPSERESRNVLAESARIARGKITSLAQLN
Q8N1Q1	AAHEPDGLAVLGVFLQIGEPNSQLQKITDTLDSIKEKGKQT
Q2TBT8	GAYHRCSLGCGATLGPVERANHNCYRELREAWCQRQQRSRT
O94903	GHGQRTFGENYVQELLEKASNPKILSLCPEIKWHFIGHLQK
Q6PFX2	LTGAKSSTSRDKVVKPAMNQNEVQEIIGVTKQVFPSADDVS
P63101	TELRDICNDVLSLLEKFLIPNASQPESKVFYLKMKGDYYRY
A6H789	TLQTLDLSDNRIQSVHKNAFNNLKARARIANNPWHCDCTLQ
P46412	IDGEEYIPFKQYAGKYILFVNVASYUGLTDQYLELNALQEE
Q47036	ITTRPRRREPLWAVTDETMRNWLKQAVRRAEADGVHFSISV
Q9DCX7	SGAAAAAPSPPQYYTIYPQDNAAFVESEGFSPFVGTGYDRP
Q8VCZ2	GLWWSVKYPLKYFHHKGLKNNRLSRQQERIEIIEGAVKTLF
A6NH52	FSLKLKWLALVVAGISLQAANLYGYILCKMGGNSDIGKVTA
P97382	PGLQGSLDRLQLEYVDIVFANRSDPNSPMEEIVRAMTYVIN

Q9D5H4	ETHYLHEQVKSIKELGDHVHNLVTMGAPAAGLAEYLFDKHT
Q62283	FVFRHEIKDTFLRTYTDAMQNYNGNDERSRAVDHVQRSLSC
Q13868	KRQKTHFHDLPCGASVILGNNGFIWIYPTPEHKEEEAGGFI
Q16623	IRKTQHSTLSRKFVEVMSEYNATQSDYRERCKGRIQRQLEI
Q17QW4	RRFVEACRAREAAFDAEYQRNPQRMDFDILTFSITLTASEI
Q9CWU6	VCLGSEQTVGADTAQNRKYHNTSKLLTTQDFPQPVEEKVGP
Q3TZ65	PRPLEDTPGPGTHNPEQVTVNRARAPAYTMGIRHSKRASTM
Q9Y399	ALNFTAHMAYRKGIILFISRNRQFSYLIENMARDCGEYAHT
P41047	PSQPLPLPPLTPLKKKDHNTNLWLPVVFFMVLVALVGMGLG
P49888	PFLECRKENLMNGVKQLDEMNSPRIVKTHLPPELLPASFWE
Q9NZQ0	WAESKGFLYFETSAQTGEGINEMFQTFYISIVDLCENGGKR
Q9DB26	APSDSGPGTSFLGSDPAWASNLFVPLPVRRGGLVLIHGEVV
P47753	DEEKVRIAAKFITHAPPGEFNEVFNDVRLLLNNDNLLREGA
P11842	GRKGELSDDYPSLQAMGWDGNEVGSFHVHSGAWVCSQFPGY
Q9BQ50	VRTLQAFLSRQAGPICLVAHNGFDYDFPLLCAELRRLGARL
Q6F5E0	LLVRWGRPRGLQCDLLLFSTNAHGRAFFAAAFHRVGPPLLI
Q6UWM5	PPYVRGESCSLCSKEEKCVKNLCRTPQLIIPNQNPFLKPTG
Q3SZD1	FTSCLKETLSGLAKNATDLQNSGMSEEELTKAMEGLGMDEG
Q8WXX5	VDSLKAAIQSRQKDRQKEMDNFLAQMEAKYCKSSKGGGKKS
O88958	GSSLVSRTRVKTLAMDTILANARFFDGDLAKVPTMALTVGV
Q3SZ18	QSTGDIKVIGGDDLSTLTGKNVLIVEDIIDTGKTMQTLLAL
Q7L0L9	CSRQALGHLSGRGPSPRSEMNSSVGDLGVGGCSLWDDPARF
Q60590	YRQAIQTMQSEFFYLTTNLINDTIELRESQTIGDQCVYNST
Q16594	LIGSKNILITTNMMSSQNTANESSNALKRKREDDDDDDDDD
Q9BQ15	ASQPTTGPSAASPASENQNGNGLSAPPGPGGGPHPPHTPSH
P12319	NFFEVSSTKWFHNGSLSEETNSSLNIVNAKFEDSGEYKCQH
Q9ES30	YGFRGYQGPPGPPGPPGIPGNHGNNGNNGATGHEGAKGEKG
Q32KX8	DVDQRCLDSILATTQAHGLPNVKAPMYLDVTWDWKQWGGIL
Q1LZ83	GDICKSINNDTTYCVKKLPRNPERKEIIGNGEQQYVYVKDG
O14893	LSIVSRMNQATVTSVLEYLSNWFGERDFTPELGRWLYALLA
Q9NS28	HGSGKEETSKEAKIRAKEKRNRLSLLVQKPEFHEDTRSSRS
Q32KY3	KKSKNNKTGDEEVNQEFINMNECDAHAFDDEWQNEILKYQP
Q01081	GDRCSRLHNKPTFSQTIALLNIYRNPQNSSQSADGLRCAVS
Q91WD9	KNKDGRLDLNDLARILALQENFLLQFKMDASSTEERKRDFE
O14972	NVKERALLPKFLLRGHLNSTNCVITQPLTGELVVESSEAAI
Q3MQ24	DHKQLWMGLQNDRFDQFWAINRKLMEYPAEENGFRYIPFRI
Q8BNX1	LSQHTRGRGYWLGLRAVRHLNKIQGYRWVDGASLNFSHWNS
P09016	SGTALKQPAVVYPWMKKVHVNSVNPNYTGGEPKRSRTAYTR
Q6PHZ8	VLKEDAPRQHVETFFQKMDKNKDGVVTIDEFIESCQKDENI
Q96BH3	DTRISALVPGFPCHFPFNYKNKNYFNCTNEGSKENLVWCAT
Q9BZM6	HGRGSWQFLFNGQKFLLFDSNNRKWTALHPGAKKMTEKWEK

Q9NRX1	VTRTRIVLADVKVHILGSFQNIKMARTAICNLILGNPPSKV
Q5JUQ0	QEGKIQAISDSDGVNYPWYGNTTETCTIVGPTKRDSKFIIS
P20934	AEIKSQGETFKKEVCEENTSNTAMLICLIVIAVLFLICTFL
Q8K3I6	AESRLPGVNLDLLPLGYHLPNVSLTFQAWHHLSDSERLCFL
Q9D0V7	QDMPFIHQSSELGDLDTWQENSNAWEEEEDAAWQAEEVLRQ
P11911	FFPEVNKNHRGLYWCQVIENNILKRSCGTYLRVRNPVPRPF
Q8TCF1	CHRWSIGKAIDFAASLARLKNDNNKFTAKKLRLCHITSGEA
P19075	NGKQVYKETCISFIKDFLAKNLIIVIGISFGLAVIEILGLV
Q5SRD1	SHADLAGVPLTGMNPLSPYLNVDPRYLVQDTDEFILPTGAN
P46952	PVCNKLMHQEQLKVMFIGGPNTRKDYHIEEGEEVFYQLEGD
Q8CCA0	NKDQWCNVLEFSRTISLDLSNYDEDGAWPVLLDEFVEWYKD
Q05B78	FYDRQLKLLLSGATFLVTFENSEKPDTMVCRLSSNQRFLLL
Q96EU6	QRQLALAEKFKELKRSKKLENFLSRKRRRNAGKDRRHLPLS
P20336	TITTAYYRGAMGFILMYDITNEESFNAVQDWSTQIKTYSWD
Q92688	RIHLELRNRTPAAVRELVLDNCKSNDGKIEGLTAEFVNLEF
Q9D968	ANAHIDYTSRPAIVFRISAKNEKGYGPATQIRWLQGNSKKA
Q9BZE7	TYTPGTCELLRVMMKESKLTNIQQRHIMDIMKRGDALPLQC
Q8R3G9	PEYNRILNETLYENAKLLSDNTDEAKDFQKAMIVFQSEFKC
Q9BPZ2	KEGDEPITQWKGTVLDQVPINPSLYLVKYDGIDCVYGLELH
P01909	QFRFDPQFALTNIAVLKHNLNSLIKRSNSTAATNEVPEVTV
Q3TQI7	VNYVQHNRFYHEELNAPIRRNKEEPKARPLRVGDTEKPEPE
P10144	GDPEIKKTSFKGDSGGPLVCNKVAQGIVSYGRNNGMPPRAC
Q3SZR5	AFSFGSPYGTTTLKYFQDQRNAALGRSSTAFSRGGHFPTIF
Q9BW66	KIQVKMEKLSSTTKGICELENYHYGEESKRPPLFHTWPTTH
Q91V41	YYRGAAGALMVYDITRRSTYNHLSSWLTDARNLTNPNTVII
P01882	FFPENIHLMWLGVHSKMKSTNFVTANPTAQPGGTFQTWSVL
Q58DI5	VLVVLWAFILGLSRVMLGRHNVTDVAFGFFLGYMQYSIVDY
A6NIH7	CEHIYEFPQLSEDVIRLMIENPYETRSDSFYFVDNKLIMHN
Q16611	HCIARWIAQRGGWVAALNLGNGPILNVLVVLGVVLLGQFVV
P78345	KQQVSGWTPAHVRKQLAIGVNEVTRALERRELLLVLVCKSV
Q9CPQ5	LTSEVEEEEQEVKQVFHIDSNKVLALPELSQKSLKAPILQE
P31213	NRGRPYPAILILRGTAFCTGNGVLQGYYLIYCAEYPDGWYT
O43543	SVNLQESTLRKCSQCLEKLVNDYRLVLFATTQTIMQKASSS
O09131	PFAQRTLMVLKAKGIRHEVININLKNKPEWFFEKNPLGLVP
P21266	PDYDRSQWLDVKFKLDLDFPNLPYLLDGKNKITQSNAILRY
Q8BG94	KWKQNASTLAQWAMGQTLMVNQLIDMEWRFGVTSGSSELEK
Q1WG82	LEISSATSPASLQRRPRKQLNPRMGIEKVDPRFKGVTLEFQ
Q2YDG2	EVYFSGKKRLLPRPTDMSFYNWDSHIAIWNSTPNYQVIADN
Q865R3	SDDQWGEYSCVFLPEPMGTANIQLHGPPRVKAVKSSEHINE
A0JNC4	TWWFGVKFAAGGLGTFHAFLNTAVHVVMYSYYGLCALGPDY
Q9D1H0	FGSPARTRPAQPDFDPPWVQNCNRSRGVGPGPPKGSLAKRD

Q8C5C9	REELELSMLDGPQEELTPLNNDLRIQPNSAEDPSPAQVGKE
Q9CRA7	TLCKCHYIEDNCLQRLSQLENLRKSLLELEIIACGNVTDNG
P00915	GSEHTVDGVKYSAELHVAHWNSAKYSSLAEAASKADGLAVI
Q8N7B6	NRATGNYDQRTSSSTQLKHRNAVQGSKSSLSTSSPESARKL
Q6QLQ4	SWYGSKRHCSQLGAHLLKIDNSKEFEFIESQTSSHRINAFW
P97766	QLIGLGYSYQSEGDGAREVSNILSPVIPGTTLDRTLSNSSR
Q495A1	TTAQVTQVNWEQQDQLLAICNADLGWHISPSFKDRVAPGPG
O55183	VVRCLNSALQVGCGAFACLENSTCDTDGMYDICKSFLYSAA
Q8TCZ2	RAPANTLGNDFDLADALDDRNDRDDGRRKPIAGGGGFSDKD
P41732	DERSRAVDHVQRSLSCCGVQNYTNWSTSPYFLEHGIPPSCC
Q9Y2Y6	SELELDDVVITNPHIEAILENEDWIEDASGLMSHCIAILKI
P26892	LLEYQIYLDYLQNEYEGNQENVRDLRKNIRTLIQILKQKIA
P19397	DNSTKAAWDSIQSFLQCCGINGTSDWTSGPPASCPSDRKVE
Q6P5X7	NAESVITSSSSHIISQPPGGNSHSLSLQSQLTASERFQENS
O75712	EQKDFDCNTKQPGCTNVCYDNYFPISNIRLWALQLIFVTCP
Q9H0T7	WLKDLEEELHPGEVLVMLVGNKTDLSQEREVTFQEGKEFAD
Q9Y5Z4	STSVESMDWDSAIQTGFTKLNSYIQGKNEKEMKIKMTAPVT
Q9HAV5	ESLFPVPPSKETSAESQVSENIFQTQPLNPILEDDCSSTSG
Q99618	SELDLPLGTQLSVEEQMPPWNQTEFPSKQVFSKEEARQPTE
Q3KPI0	HFQNVTLEDTGYYTLQVTYRNSQIEQASHHLRVYESVAQPS
Q28071	KEFRASLYKGADSAVEVCAVNGNHSHPLQSTNKEFNCTVKV
Q8BH50	LASTESDKPTTGRVYESDSSNHCMLSPSSSGHLADSDTLSS
P56750	VGTLATTLLPQWRVSAFVGSNIIVFERLWEGLWMNCIRQAR
P97769	PSFNLHPTPHLMASMQLQKLNSQYQGAAATAAAALTGAGLP
Q9BXS1	AKSDRIWGEHEICYLLLVRKNVTLNPDPSETKSILYLSQEE
O60812	LQAIKQELTQIKQKVDSLLENLEKIEKEQSKQEVEVKNAKS
P09021	VSEQFRDSASMHSGRYGYGYNGMDLSVGRSGSGHFGSGERA
P47876	AAQQKAGDEIYKFYLPNCNKNGFYHSKQCETSLDGEAGLCW
Q8C7B6	EDWLKSALQRSICSQHESLVNDFLLQVCNRCPNLTSVTLSG
Q91ZT8	VSDWSPLHDAAIHGCLLTLRNLISQGWPVNIITADHVSPLH
Q9GZT6	SETSNKIDAEIASLKTLMESNKLETIRYLAASVFTCLAIAL
P50992	YYGKKAQPHYSNPLVAAKLLNVPKNMQVSIVCKILADHVTF
A1A546	LFHFTHYPDIHVRSQLASRINLPEARVQIWFQNQRAKWRKQ
Q05685	TCKRDWHKGWDWSSGINKCPNTAPCHTFEYYFPTPASLCEG
Q96E17	DWSTQIKTYSWDNAQVILVGNKCDMEDERVISTERGQHLGE
Q9MZ06	NPTSCLELHKNNAYWKQIGRNLRSQKVICGDAKSVLKTRVC
Q9CQ52	MVCAGGDIQSGCNGDSGGPLNCPADNGTWQVHGVTSFVSSL
Q92737	QRIAETRPAGAPEAPILVVGNKRDRQRLRFGPRRALAALVR
Q3MHF7	LLARHGRQHTIMPSKVNYQANIWALKEEGCTHVIVTTACGS
Q9Z260	SAFIESNIVVFENRWEGLWMNCMRHANIRMQCKVYDSLLAL
Q9BV86	IPPTVDGMLGGYGHISSIDINSSRKFLQRFLREGPNKTGTS

Q9D6K7	LGEIVLAIRSFQIALHIYPMNPELWKEDLSWARKLQEQQKV
P56537	NSLPDTVQIRRVEERLSALGNVTTCNDYVALVHPDLDRETE
Q80X91	VRRQEPALRGGPGPLTPHPCNELGASASPRTPGPARRGSGR
O70273	ESPDMKKEQDHPVKSHTKKHNPRGTHLWEFIRDILLSPDKN
P14509	LRWLAGHISVPSVVSFVRTPNQAWLLTTAIHGKTAYQVLKS
Q9Y478	EEIKAPEKEEFLAWQHDLEVNDKAPAQARPTVFRWTGGGKE
O43731	FLGLYRALYLANWIRRYQTENFYDQIAVVSGVVQTIFYCDF
P15328	PNLGPWIQQVDQSWRKERVLNVPLCKEDCEQWWEDCRTSYT
B1ANY3	GLSLPSGGPVLPYVKESARRNPASAATPSAAVGLFPAPTEY
Q14964	SITRSYYRNSVGGFLVFDITNRRSFEHVKDWLEEAKMYVQP
P54709	KLHVGYLQPLVAVQVSFAPNNTGKEVTVECKIDGSANLKSQ
Q9ESG4	CIVTVAIALLVLSGIRQRRRNNKGPPGVEDAEDKCENIITI
Q32KW0	DEAFTNQLPAGLITADGQQQNVMVYTTSYQQISGVQQIQFS
Q9NYP8	LLLISTLGVFALNCFTKGQKNSTLIFTRENTIRNCSCSADI
Q9NPF0	LRNATTMGPPVTLESVPSVGNATSSSAGDQSGSPTAYGVIA
A3KN25	PSSNYSPTSQSAKRCKAPTDNFPVVEIRERPGEAGERGSEV
P27811	YNSFWIGLRYTLPDMNWKWINGSTLNSDVLKITDDTENDSC
Q8IUI4	SDHSSVNIMSAFESPFGPNSNGSQSSNSWKIDSLSLNREFG
O43657	KIQNTLHCCGVTDYRDWTDTNYYSEKGFPKSCCKLEDCTPQ
Q7Z6A9	SWKEEKNISFFILHFEPVLPNDNGSYRCSANFQSNLIESHS
Q8N5P1	SPKSSLHRKSRSKDYDVYSDNDICSQESEDNFAKELQQYIQ
P19075	KQFQEAIIVFQEEFKCCGLVNGAADWGNNFQHYPELCACLD
A5D989	KIWFDKFKYDDAERKFYEQMNGPVAGSSRQENGASVILRDI
P01911	SDVGEFRAVTELGRPDAEYWNSQKDILEQARAAVDTYCRHN
Q9JL59	LNAHNDVWDEPCCTEHEVSVNRGSRVVMACNISNNLRDVTI
Q3ZCH5	YYKDREGSHTFQGAFGCELRNNESSGAFWGYAYDGQDFIKF
Q9X2V8	HLNIFKSDCLTYSYALKRILNSRNIDAHLVIGVRTQPFYSH
P19217	VTYPKSGTTWLSEIICMIYNNGDVEKCKEDVIFNRVPYLEC
Q8N144	SVAELGHLLWKGRPRAGERDNRCNRAHEEAQKLLPPPPPPP
Q9R1Q6	SCLLLLACIATAAAATVLGVNSLIRQTSVPYYVEIFSTCNP
P10279	GWGQPHGGGGWGQGGTHGQWNKPSKPKTNMKHVAGAAAAGA
P70190	PFLTQVTGRIFVQAHFFRCVNLHIEVSKDLKESLEVVNQAQ
Q96PM5	ENCGICRIGPKEDFFHCLKCNLCLAMNLQGRHKCIENVSRQ
P09237	LYAATHELGHSLGMGHSSDPNAVMYPTYGNGDPQNFKLSQD
P68401	NHENTAEEVAGGIEAIVQLINTRQPQAKIIVLGLLPRGEKP
P08865	NLKRTWEKLLLAARAIVAIENPADVSVISSRNTGQRAVLKF
P61267	PDEKTKQELEDLTTDIKKTANKVRSKLKAIEQSIEQEEGLN
Q3KNM2	LVFPTIATIVGKLMFSSVNSNLQRTILGGIAFVAIKGAFKV
P41238	CLLYEIKWGMSRKIWRSSGKNTTNHVEVNFIKKFTSERDFH
Q9EP73	YGGADYKRITLKVNAPYRKINQRISVDPATSEHELICQAEG
Q9CR00	KQARDMAEAREEAMNRRLASNSPVLPQAFARVNSISPGSPA

Q08624	RIEIAHSLCLSERQIKIWFQNRRMKWKKDHRLPNTKVRSAP
Q9D3G2	VLSVSLGLGDKDVAFTCIASNPVSWDMTTVTPWESCHHEAA
Q99N09	CLQSKELRPTEYHYYQFLDRNECFAAKSVLAGVFSLMLIST
Q5SX19	GLARLEQQVSLLCFSSGNYYNQGEIRKKELLQSCAVLGIPP
Q9CXW3	IETELKNKMQQKSQKKPELDNEKPAAVVAPLTTGYTVKISN
P40259	QSPRFIARKRGFTVKMHCYMNSASGNVSWLWKQEMDENPQQ
H3BS89	HRDRCKAFNTRRVDPGFIYNNNNNLPLRASRSRLDRWEGKL
Q8BGN8	NQGRYNQESYGSSGGYSQQANLGPTSDEFGQQPSGPTSFNN
Q9NVV0	EVTRTWKIVGGVTHANSYYKNGWIVMIAIGWARGAGGTIIT
Q8C8C1	GTARLDESSMLEENIESLVDNLITSLREQCYGERGGHYRIH
Q91WG1	VLFFIGVFLALVLNLLQIQRNVTLFPPDVITSIFSSAWWVP
O00161	TMLDEQKEQLNRIEEGLDQINKDMRETEKTLTELNKCCGLC
Q15475	AAEAKERENTENNNSSSNKQNQLSPLEGGKPLMSSSEEEFS
Q3TEW6	LLTARISALEVHTPKEIFVVNGTQGKLTCTFDSPNTTGWLT
Q9D9F8	RELGNEVPSEDLELPGRKTSNFQVLQPCRDEGASAECSIVE
Q9HAT0	ALSRGETPPVRERSERVALCNRAELTPELLKILHSQVAGRL
Q9D6Y1	NCSQGQQLTTFSSDWEVQEDNRLMCSSVQKALFEEEDHVKK
Q96SN7	FALLISTCILPNVEAVSNIHNLNSISESPHERMHPYIELAW
O95848	SVGRCAASPYLRPLTLHYRQNGAQKSWDFMKTHDSVTVLLF
Q2HJ54	YHLQSKVPTFVRMLAPEGALNIHEKAWNAYPYCRTVITNEY
Q9Y5U4	MRCVAVFVGINHASAKVDFDNNIQLSLTLAALSIGLWWTFD
Q92600	NIYPSINPPTLTAHQSNRVCNALALLQCVASHPETRSAFLA
Q6ZQT7	WLSAAFPGPAFDFWRPLQAQNLPSSGPLQARPRPRPHSGLS
P02763	LPLLEAQIPLCANLVPVPITNATLDQITGKWFYIASAFRNE
Q80ZA7	DFFFPTACIIRDNQVVVACNNQPYLSESECLKSKCCSSTSG
P70280	WCGGNFLEVTEQILAKIPSENNKLTYSHGNYLFHYICQDRI
Q9P0W0	WKERHLKQIQIGLDQQAEYLNQCLEEDKNENEDMKEMKENE
Q7Z5L0	LVAFSLRVEAPTTLGDNTAANNVRFRCSDGEELQGPGLSWG
Q99LQ7	PFREHHIDPTAITRHDFIETNGDNCLVTLLPLLNMAYKFRT
Q7TQB9	MIVASLASSRFCLHGIAILANLLASFDFCYQANLIGILWDF
P49406	DALPEYSTFDVNMKPVVQEPNQKVPVNELKVKMKPKPWSKR
Q9CQJ2	HAELDAKGQGCTAYDVAVNSNFYLRMQNSDFLRELVVTIAR
A5D7F5	SFDALDTWLAEMKQDLGPHGNMENIVFAVCANKIDCTKHRC
Q96DX8	LKTGNSSPGIGAVYLANQAKNQSAEAKEAKGSGYEKLGPSR
Q8WVD5	LVRESGSLTYEEFLGRVAELNDVTAKVASGQEKHLLFEVQP
A8MXD5	LPVVFIDGHYLGGAEKILSMNESGELQDILTKIERVQHPHE
Q29463	ASAGTECLISGWGNTLSSGVNYPDLLQCLVAPLLSHADCEA
Q9P0W0	IGLDQQAEYLNQCLEEDKNENEDMKEMKENEMKPSEARVPQ
P26441	MPVASTDQWSELTEAERLQENLQAYRTFHVLLARLLEDQQV
Q9D112	TTDPDSNSLNTEQKGSWDSENFWLDPSSKGQLETNEEEDGL
Q5E9K1	VSSLQGEDCREAVQHLRAGANLPEEQLGALIAGTHTLLQQA

Q5JPI9	GSIIEKEGLSNIKLKVEDFLNLSTQLSGFHICIDKGTFDAI
Q8N4C9	IRELQMQETHTETKRTTFIQNRTIATLQCLGSDSKVKVNLV
Q9Y3B6	VWGAQAGLVVAGYYHANAAVNDQSPGPLALKIAGRIAEFFP
P51809	LDKVMETQAQVDELKGIMVRNIDLVAQRGERLELLIDKTEN
Q99614	RMKQDKKEMAINDCSKAIQLNPSYIRAILRRAELYEKTDKL
Q9NYZ1	FITCMVTIILLLSCDFWAVKNVTGRLMVGLRWWNHIDEDGK
P43487	THADFADECPKPELLAIRFLNAENAQKFKTKFEECRKEIEE
Q08E12	AIPEGTKLELPLWLAKGLFDNKRRILSVELPKIYQEGWRTV
P18128	RLMCADLCQTGFCLSIIENRNFPESSILYLASKGVFSFICL
Q5E9B7	NPESNTAGLDIFAKFSAYIKNSNPALNDNLEKGLLKALKVL
Q30167	FECHFFNGTERVRLLERRVHNQEEYARYDSDVGEYRAVTEL
P50553	GYSLPQQQPAAVARRNERERNRVKLVNLGFATLREHVPNGA
Q32L47	ESNNLRLVTEYNAITAIGLFNSMIPIHLLLIMNKASSEFEE
Q9NP55	LDGLGPLPIQGLLDSLTGILNKVLPELVQGNVCPLVNEVLR
Q9Z0H7	PFFSMASSLNLPVLEVGRTENSSFSSATLPRPGDPGAPPLP
P24863	LMAPTCVFLASKVEEFGVVSNTRLIAAATSVLKTRFSYAFP
Q9JHB3	EPWEDLSLVQRESLNHHYHQNCGCQITTCYAVPCTISAPNE
Q9CQ56	QGLQEKLAEEMLGLARSLKTNTLAAQSVIKKDNQTLSHSLK
P50289	TPLPSTSAAITINCHTCAYMNDDAKCLRGEGVCTTQNSQQC
P05141	LSFWRGNLANVIRYFPTQALNFAFKDKYKQIFLGGVDKRTQ
Q14002	YVFSEPPKPSITSNNFNPVENKDIVVLTCQPETQNTTYLWW
Q99627	STTRMVLPRKPVAGALDVSFNKFIPLSEPAPVPPIPNEQQL
A6QLC6	IPVRSRVREFDSSTLNESVQNTIMRDLKAVGKKFMHVLYPR
Q9UF72	SPSIGTVPPPAVGHTSLLPVNPPAISTTTSDLSAREDATPS
Q505H4	NCFAGTTIIPAGIEVKVDDCNICHCHNGDWWKPAQCSKREC
P56966	GLFFQIRDDYANLHSKEYSENKSFCEDLTEGKFSFPTIHAI
Q8N144	LVLATVGGAVFEDEQEEFVCNTLQPGCRQTCYDRAFPVSHY
P32326	SHRCGFGSNAYFCDVFKRKYNMTPSQFRLQSRQSNDPNFIT
Q8HXY9	TSEEDEDYVPSGGEYSEDDINELVKEDEVDGEEETQKTKGT
Q96EC8	FVIVWFGAVTITLNSKLLGGNISFFQSLCVLGYCILPLTVA
A6NCJ1	RASDYVPSLSAPQRPPGTTQNYREWVLEPYCPSTCQRSPPS
P28068	GSLTNRTRPPSVQVAKTTPFNTREPVMLACYVWGFYPAEVT
P58417	IVEFDLAQQTVIDAKDSKSFNCRIEYEKVDKATKNTLCNYD
Q9CQD1	AKTSMNVNEIFMAIAKKLPKNEPQNPGANSARGRGVDLTEP
Q9HB31	CLPEAKVQVWFQKRWAKIIKNRKSGILSPGSECPQSSCSLP
P16152	ELQQKFRSETITEEELVGLMNKFVEDTKKGVHQKEGWPSSA
P10144	EQEPTQQFIPVKRPIPHPAYNPKNFSNDIMLLQLERKAKRT
P70379	GYYLQMHPDGALDGTKDDSTNSTLFNLIPVGLRVVAIQGVK
Q921D4	GSVLDYFSERSNPFYDRTCNNEVVKMQRLTLEHLNQMVGIE
Q7M721	FGISLNCVLLLIYSLSKHLKNMKFYGKGCQDQSTMVHIKAL
Q24K06	PVHVCSFQELVKLYLSDNRLNSLPPELGQLQNLQILALDFN

A5PJN0	SIRGVDLDKFREILLRHCDVNKDGKIQKSELALCLGLKINP
P0C8J7	MYASAPVLDFSRLENIRQWVNLPLVLHGASGLSTKDIQQTI
Q0VCT4	KGPSEMEDSEDKCENVITIENGIPCDPLDMKGGHINDAFVT
Q9Y287	LNLDKCYVIPLNTSIVMPPRNLLELLINIKAGTYLPQSYLI
Q6ZMS7	QEYGLLQRRLENVENLLRNRNFWILRLPPGSKGEAPKVPVT
O35723	VSTSTEIINGKKITTKRIVENGQERVEVEEDGELKSLIING
Q5XFZ0	GSCPGQARYLLWAYSSTHEDNSTFQETCPHCFQLLVLDNSR
P61266	RDRCKDRIQRQLEITGRTTTNEELEDMLESGKLAIFTDDIK
Q99JZ0	GLSLSSQEVQKNLTQIPDSDNMVVTSPGPGQVVAPVSGNNL
O43423	RIHSELRNRAPSDVKELALDNSRSNEGKLEALTDEFEELEF
Q8N9P0	LFPFIHFCWSPRAAAPGVAMNGWMPARWDHQVRRDVAGARG
Q0VD07	FMLAWLGVTAFTSLPVYMYFNLWTICRNTTLVEGANLCLDL
Q15771	LFLDLACRLISEARQNTLVNNVSSPLPGEGKSISYLTCCNF
A6H707	EQSYAGRVLPSDLDLLLHMNNARYLREADVARIAHLTRCGV
Q6L8G5	GCGSCGGSKGGCGSCGCSQCNCCKPCCCSSGCGSCCQSSCC
Q9NQT4	TSVLAGVYGPAEVKVSKEIFNKATLEVILRPKIGLPGVAEK
O08849	EKEAPKEINIDFQTKSLIAQNIQEATSGCFTTAQKRVYSLM
Q9CZ92	ELRALEAEVAALQRECRMLQNAGEKASGAWKSFQKISQSDS
Q9BXL8	QSLLDMSLVKLQLCHMLVEPNLCRSVLIANTVRQIQEEMTQ
Q9BPX1	GRMGQPAEVGAAAVFLASEANFCTGIELLVTGGAELGYGCK
Q0V7M7	VVSKYKILHQPKKSMSSVARNLYHRFIDEETKETKGHYFVV
Q3T040	SSVFEDPVISKFTNMMMKGGNKILARSLMTQTLEAVKRKQF
P47962	IVCAAYAHELPKYGVKVGLTNYAAAYCTGLLLARRLLNRFG
O35621	AEQLGEGDEVIEKFDYVFAENGTVQYKHGRLLSKQTIQNHL
P48787	SDAAGEPQPAPAPVRRRSSANYRAYATEPHAKKKSKISASR
Q15404	NSLPGNFFYLTTLRALYLSDNDFEILPPDIGKLTKLQILSL
Q02878	VKKGNLKAKKPKKGKPHCSRNPVLVRGIGRYSRSAMYSRKA
Q16637	LLSPICEVANNIEQNAQENENESQVSTDESENSRSPGNKSD
Q8WXJ9	TRQSNIFKILLQYGILEREKNPINIVLTIVLYPSRVRVMVD
Q15848	PLPKGACTGWMAGIPGHPGHNGAPGRDGRDGTPGEKGEKGD
Q9BX51	TINLYFGSKVRSPVSGILLNNEMDDFSSTSITNEFGVPPSP
P26717	NKSSISGTEQEIFQVELNLQNPSLNHQGIDKIYDCQGLLPP
Q9BXN2	PCPPNWIIYEKSCYLFSMSLNSWDGSKRQCWQLGSNLLKID
Q8NAJ2	AVHSCVARAWPGDPQEVRHLNPLLCDPGSQVEPSWPWHPGL
P97351	QQVRQIRKKMMEIMTREVQTNDLKEVVNKLIPDSIGKDIEK
Q14847	TCKMTLNMKNYKGYEKKPYCNAHYPKQSFTMVADTPENLRL
Q5JUQ0	TCTIVGPTKRDSKFIISMNDNFYPSVTWAVPVSESNVAKLT
P00493	PSVVISDDEPGYDLDLFCIPNHYAEDLEKVFIPHGLIMDRT
A1YQ93	APEIAAMPAEVSPYLQKEMINFQHTNAGIFIPSTSQKPSTT
Q9UHQ4	FWLVLRRLVTLITQLAKELSNKGVLKTQAENTNKAAKKFME
O88574	KSARHLYICDYHKNLIQSVRNRRKRKGSDDDGGDSPVQDID

Q8QZV2	CCCRCLRPKQDPQQSRAPGANRLMETIPMIPSASTSRGSSS
Q9EQF4	SPERRNSGRCLQKWFEPTLSNPNVEVALHFAGSALAPQLSY
Q14847	KFWHKACFHCETCKMTLNMKNYKGYEKKPYCNAHYPKQSFT
O35257	DEYGEMSIYDLLDHVTILSHNVSELTAEMHRIFMEDVRYKP
Q8N6D2	DTAESQASDELECKICYNRYNLKQRKPKVLECCHRVCAKCL
Q8BJU2	STTPLWRTGCYEKVKLWFDDNKHVLGTVGMCILIMQILGMA
Q3T0X5	KHGLRALRETLPAEQDLTTKNVSIGIVGKDLEFTIYDDDDV
P47757	HIANIGRLVEDMENKIRSTLNEIYFGKTKDIVNGLRSLDAI
Q02878	VVFLKQLASGLLLVTGPLVLNRVPLRRTHQKFVIATSTKID
Q68D42	GLRGKPPPQSQGEVSVASSINSPTPTEEGECQSLVQNGHQE
Q78JT3	PVCNKLMHQEQLKIMFVGGPNTRKDYHIEEGEEVFYQLEGD
Q05B50	YPSNQTLKDYLSWRQADCHINNLYNTVFWALVQQSGLTPLQ
Q8NGU1	GTLELALMGVMAVDRYVAVCNPLRYNIIMNSSTFIWVIIVS
Q4KXC9	LRILNECPNIRWDNLSECLLNYRIHNKSTQKSALAYYECAS
C9J442	THSEPIFCTTSISNTCLLPQNSSWKAWQVPWCLHDGQTRPA
H7C241	YPPGIACVGIFRVCIYRRRTNSTTTKFCYRYSYQDTFLPFE
Q3UPR9	PPAMNSVSLRCSGDGLDSDGNQTLRWQAIGNPRCQGTWKKV
P55075	DTFGSRVRVRGAETGLYICMNKKGKLIAKSNGKGKDCVFTE
Q6S5G4	SAWSDEQFEFHCNTQQPGCENVCYDHAFPISHVRLWVLQVI
P23560	FEHVIEELLDEDQKVRPNEENNKDADLYTSRVMLSSQVPLE
Q15493	TKFCALNWKEQSAVVLATVDNDKKNNRFNDGKVDPAGRYFA
Q497H0	KAPSLPPRCPCGFWGSSKTMNLCSKCFADFQKKQPDDDSTP
Q64317	EDPGADSEKSTVVEGGEVRFNGKGKKIRKPRTIYSSLQLQA
Q2HJJ1	PESQLGLWGGEGWLKGHRYVNNDKFSKRVKKVWKPQLFQRE
Q7Z7D3	IGFGISGRHSITVTTVASAGNIGEDGILSCTFEPDIKLSDI
Q14331	KREEDEETQLDIVGIWWTVTNFGEISGTIAIEMDKGTYIHA
O15217	LELLIMHPFLKPDDQQKEVVNMAQKAIIRYFPVFEKILRGH
Q92637	RNGKAFKFFHWNSNLTILKTNISHNGTYHCSGMGKHRYTSA
Q0VCT9	PGTVNGGHPPSALAPAARFNNSQFMGPPVASQGGSLPASMQ
Q9CQ28	MQCIAEGHQIVALANLRPDENQVESDELDSYMYQTVGHHAI
P20826	LLLFNPLVKTKEICGNPVTDNVKDITKLVANLPNDYMITLN
Q00169	CPYMCAYKLVTVKFKWWGLQNKVENFIHKQERRLFTNFHRQ
P02830	SGYGPGAGAFASTVPGLYNVNSPLYQSPFASGYGLGADAYN
Q8K3W2	IRYFPSLAHLTNLKLVIYDHNPCRNAPKVGKGVRRVGRWAE
P82348	LESPTRSLSMDAPRGVHVKANAGKLEALSQMDIILQSSEGV
Q05717	ASLQELKASPRMVPRAVYLPNCDRKGFYKRKQCKPSRGRKR
Q9P0I2	LRENGKYIPKQSFLTRKYYFNNPEDGFFKKTKRKVVPPSPM
Q0II87	YVRFSKEQLPIFKAQNPDAKNSELIKKIAKLWRELPDSEKK
Q9CZE3	STFDAVLKWKNDLDSKVHLPNGSPIPAVLLANKCDQKKDNS
Q3TEW6	PPFKDRVTWAGDLDKKDASINIENIQAVHNGTYICDVKNPP
Q8TDQ7	DEYVGLPRNHPESYHSYMWNNFFKHIDIDPNNAHILDGNAA

Q8BV13	CEAVLLGIEQQVLRANQYKENHHRTQQQVEAEVSNIKKTLK
P23409	QQEKMQELGVDPFSYRPKQENLEGADFLRTCSSQWPSVSDH
P80219	GMMCSVAGWGRLGVNMPSTDNLQEVDLEVQSEEKCIARFKN
P18128	AGRRCRVGIRPPCRASVPCGNPPTLPGAGAVWKFAHLHDKN
Q8BVH9	SCREYEGQKLTLLEAGCGVGNCLFPLLEEDLNLFAYACDFS
P22615	NLPENHLNISSLPWVSFDGFNLNITGNDDYFAPVFTMAKFQ
Q8N614	QDITGEFKMCSSCLVCESKGNMDFISQEQTSKVLIRRGSME
P15246	ATISAPHMHAYALELLFDQLNEGAKALDVGSGSGILTACFA
Q16635	TVLIGKPFSALPVLERLRAENKSAVEMRKALTDFIQEEFQH
P60880	NLEQVSGIIGNLRHMALDMGNEIDTQNRQIDRIMEKADSNK
P36369	TPTRWQKSDDLQCVFITLLPNENCAKVYLQKVTDVMLCAGE
Q8R1J3	ALKKDSRREGRRLKRQAAKKNAMVCFHCRQPGHGIADCPAV
Q9WV35	KEAGCKLRIMKPQDFEYIWQNFVEQEEGESKAFEPWEDIQE
A1YQ93	LGATMSAPLIPQHLMSASNSNELLLNLNNAQLRPLQLQGPF
Q60932	SPNTGKKNAKIKTGYKREHINLGCDVDFDIAGPSIRGALVL
Q3T168	AESVQVNFTERSFDLLVKNLNGKSYSMIVNNLLKPISVEGS
Q61168	ILTLCSSYMEVPTYLNFKSMNHMNYLPSQEGVPHSQFINMM
P48230	NSTWGYPFHDGDYLNDEALWNKCREPLNVVPWNLTLFSILL
P02746	KVPGLYYFTYHASSRGNLCVNLMRGRERAQKVVTFCDYAYN
Q6P073	VVEEIKQKQKAQDELSNRPQNLPLPDVVPDGELHRGQHGIQ
Q96B26	LKENCRPDGRELGEFRTTTVNIGSISTADGSALVKLGNTTV
Q9CQ29	PLAHRKEHQDSCPFELMACPNEGCTVQVLRGVLDEHRQHCQ
Q8BXV2	RGSPGGLCSPSVEEKLEHLENQVRLLNIRLNRVLENLDRSK
Q9H7V2	EEEEFQELESDYSSDTESEDNFLMMPPRDHLGLSVFSMLCC
P62993	DELSFKRGDILKVLNEECDQNWYKAELNGKDGFIPKNYIEM
Q9NPI5	GGMTNGGKTTLTNSLLRALPNCCVIHQDDFFKPQDQIAVGE
Q92979	VVLEGASLETVKVGKTYELLNCDKHKSILLKNGRDPGEARP
O08542	AALLGSRLPGCSSLRHPIYWNSSNPRLLRGDAVVELGFNDY
Q8WV35	VLQFLQLRQLSLSLLPELTDNGLVAVARGCPSLEHLALSHC
Q8TD07	IKSLSRPGQPWCEAQVFLNKNLFLQYNSDNNMVKPLGLLGK
Q86UD3	LYNEWRRTNQRVILLIPKSVNVPSNQPSLLGLHSVKRNSKE
Q8IVV8	QYRLRYVMVYTLWAAVWVTWNVFIICFYLEVGGLLKDSELL
Q8BQP9	HRKGKEASGGAKNLDSKIEENAETPALEDSLSSPLESQQQC
Q9NPB3	ETADMIGVRELRDAFREFDTNGDGRISVGELRAALKALLGE
P16152	SIRALRDFLRKEYGGLDVLVNNAGIAFKVADPTPFHIQAEV
Q4U5R3	GSYFPKKISELDAFLKEPDLNEANLSNLKAPLDIPVPDPVK
Q9UFN0	IFYEFRSYYLKPSKMNEFLENFEKNAHLRTAHSELVGYWSV
P48230	PKCLMANSTWGYPFHDGDYLNDEALWNKCREPLNVVPWNLT
P97382	NRSDPNSPMEEIVRAMTYVINQGLALYWGTSRWSAAEIMEA
Q1HCM0	RQSVRSPSSQPEKKPLLVKSNSGGRKAGSDPVPEPPAAAGF
P57746	AFSLAEAKFTAGDFSTTVIQNVNKAQVKIRAKKDNVAGVTL

Q8BVA2	DNCHSHVALALNLMRYNNSTNWNMVTLCCFCLIYGKYVSVG
Q9NZ43	LEDMLQALKVHASKPASEVINEYSWKVDFLKGMLQAEKLTS
P24158	NYDAENKLNDVLLIQLSSPANLSASVATVQLPQQDQPVPHG
Q92482	GLEAFTVGLVVLVIGTSMGFNSGYAVNPARDFGPRLFTALA
Q9CRA4	QKDKPETFEGQWKCLKKILFNHFFIQLPLICGTYYFTEFFN
P10629	SRGPYDYGSNSFYQEKDMLSNCRQNTLGHNTQTSIAQDFSS
Q9H0Z9	GYGFVTMADRAAAERACKDPNPIIDGRKANVNLAYLGAKPR
Q2YDP6	DPLHQCDSCFEKCLLCTPQNNYDRGKLPYHAWASFSPLASG
Q810M1	VSGYIDYAHRLKSEDFEVYFNGKRRLLPRTTDMSFYNWDSH
Q9NP72	VILVYDVTRRDTFVKLDNWLNELETYCTRNDIVNMLVGNKI
A4IFL0	LLYSAFGVIIEKTRGAEDDLNTVAAGTMTGMLYKCTGGLRG
Q8TAZ6	KHEVGTRRGCRRYRWELKDSNKEFWLLGHAEIKIRSLGCLI
P53674	EQSNFRGEMFILEKGEYPRWNTWSSSYRSDRLMSFRPIKMD
Q9JHL0	ERKNKMLFSHLEGPESPRYQNFYKGSNQEPDAAYVDPIPTN
P20774	IKPAPPTQQDSRIIYDYGTDNFEESIFSQDYEDKYLDGKNI
P01189	LECIRACKPDLSAETPMFPGNGDEQPLTENPRKYVMGHFRW
Q08DA8	GTTILQYAQTSDGQQILVPSNQVVVQTASGDMQTYQIRTTP
Q08DY6	YLTRKFMDLVRSAPGGILDLNKVATKLGVRKRRVYDITNVL
Q8VCI7	AYLAALLLDTAAFFSHRLGANGSCGLVVALETQKEAEADAP
Q9NP55	PLNVNPALPLSPTGLAGSLTNALSNGLLSGGLLGILENLPL
Q9ESJ7	YHTPGSVENLRRQACLVAVENAWRKAQEVCDLVGQTLGKPL
A6NN92	LVIVLVPGALFHLYAACKSINQECILQKPIYTIIYILSVLL
A4FV75	LEAYICPVLFGSASGGDHHHNNHGGSQGGSGPGSPHSPLPS
P20783	SSAIDIRGHQVTVLGEIKTGNSPVKQYFYETRCKEARPVKN
Q8N567	GTSQNLPKRKQLEANRLSLKNDAPQAKHKKNKKKKEYLNED
O35975	VLIPGYEVYHKVTAQNDNGYNEIFLDSPERKKSNFNCFYNG
Q9UMS0	SVFFGPDFITVTKENEELDWNLLKPDIYATIMDFFASGLPL
P04187	QVIPMVKCIPHPDYNPKTFSNDIMLLKLKSKAKRTRAVRPL
Q9D1G5	LLKSLSLNNNKLTVLPDELCNLKKLETLSLNNNHLRELPST
Q9NYV7	YVLCNLTITWEFFNILTFWLNSLLTVFYCIKVSSFTHHIFL
P08882	LTVQKDQVCESQFQSSYNRANEICVGDSKIKGASFEEDSGG
Q810N6	NFRGEKARDVAARYSQVECVNFLDWADARLILKKIITKSSL
Q9D099	VFLLGFLLWNIDNIFCDSLRNFRKRVPPVLGVTTQFHAWWH
Q9D8C8	DHELEAPALQSSLALSLELQNARAAVASGQFDASKAVEEQL
O60671	YGYPLMLFLEEGGVVTVCKINTQEPEETLDFDFCSTNVINK
Q6P1B3	WAIVWGPTVSREDGGDPNSVNPGFLPLDYGFAAPHGLATPH
Q9BQ51	TPEGLYQVTSVLRLKPPPGRNFSCVFWNTHVRELTLASIDL
Q9JIM5	QSPSVDTFDEEPPLLEELGINFDHIWQKTLTVLNPMKPADG
P14148	PKTLKKKVPAVPETLKKKRRNFAELKVKRLRKKFALKTLRK
Q96MC9	GTSQAAERQRIAHTRALSVGNCPRSHFQCEFSSISKWSEGT
P46638	DLRHLRAVPTDEARAFAEKNNLSFIETSALDSTNVEEAFKN

P32971	ILKRAPFKKSWAYLQVAKHLNKTKLSWNKDGILHGVRYQDG
Q8C432	PLEEPAESQASDELECKICYNRYNLKQRKPKVLECCHRVCA
P49888	EIMNQKLSPFMRKGITGDWKNHFTVALNEKFDKHYEQQMKE
P14231	KFHVNYTQPLVAVKFLNVTPNVEVNVECRINAANIATDDER
Q9R061	CGPSIPHMLRAQGKAVHQCDNGWVPVFVDQEQSISLMSVGF
P10949	GQKDSSDQNFDYMFKLLIIGNSSVGKTSFLFRYADDSFTSA
Q32KQ5	HSPWICCTPLWPSDGLEVIRNILIVVLSLSFMHNLLLGFEF
O75822	RLKKLQEESDLELAKETFGVNNAVYGIDAMNPSSRDDFTEF
Q8N4E4	KQKFGELREISGNQYVNEVTNAEEDVWVIIHLYRSSIPMCL
Q08624	PPRPSYPERQYSCTSLQGPGNSRAHGPAQAGHHHPEKSQPL
Q9NRN9	NRMSKSFDTVIMNPPFGTKNNKGTDMAFLKTALEMARTAVY
Q9QY36	NARPEDLMNMQHCNLLCLPENYQMKYYFYHGLSWPQLSYIA
P70331	TEAVTCQPQGNPQGCTPLLPNGSSHDHLSEPGSAGHAGNGA
Q0VCQ4	IINLEEVDEEMKSVIEALKDNFNKTVNIRTSPGALDNITVV
Q969F1	DTLGTCVIFEENVEHADTEGNNKTVLKYKCHTMKKLSMTRT
Q9XT56	PPSEYKWFKDGVEMPLEPKSNRAFSNSSYTLNQKTGELIFD
Q9CQ07	ASIAKLPKLKKLNIKRNPFPNADESEMFVDSIKRLENLYLV
Q0Z7W6	ILMSSMSALFQLSMWIRTCHNYFIEDLGLPIWGSYTVFALA
Q96S19	RALLITYGPYAINGKISPQSNVDFDLMLRCRNPEWGLRDTA
Q2KIB6	PGGVADRHGGLKRGDQLLSVNGVSVEGEQHEKAVELLKAAQ
Q9JJV3	LLVAFILSSGGLLTFIILLKNQINLLGFTLMFWCEFTASFL
Q8IWZ4	PILTQCFECIKTIQQRNLKTNIRLKKMASLARKASLWLFLS
Q91UZ4	KIALEYIVPCLHEVGFCYLDNFLGEVVGDCVLERVKQLHYN
P04224	NNTPDANVAPEVTVLSRSPVNLGEPNILICFIDKFSPPVVN
Q3ZC48	YVRYKQVHALNPEENRIIRLNKAGLVLGLLSCLGLSLVANF
P98173	GLPQPCPEEHLAFRVVSGAANVIGPKICLEDKMLMSSVKDN
P61087	SLQALLAAAEPDDPQDAVVANQYKQNPEMFKQTARLWAHVY
Q4JM65	RFQRQKYLSLQQMQELSNILNLSYKQVKTWFQNQRMKCKKW
Q7RTU5	PKPGPFGGGLALGPAPRGTMNNNFCRALVDRRPLGPPSCMQ
Q8NHS0	VMSSTEMINGHKVTTKRIVENGQERVEVEEDGQLKSVTVNG
Q3LI81	TFEDRLCLPSSFHSRTCFLDNFQETCNETTSCQMTNCEQDL
P39039	WKKDEPNNHGSGEDCVIILDNGLWNDISCQASFKAVCEFPA
Q8CBW7	PVICLNKRTRRSIQKLLEWENNRLYHKLALHWKLTKRKCET
Q719I0	WGQGNPHWIVEEREDGTNVNNWRWTERDATSLSKGKFQELL
Q9D0U9	INAILCKTQAYRHILFNTKINIHGKLCMFCLLCEAYLRWWQ
A8C927	YKLAINDQNTFYIGIDPVKENLFDISKKIIKKPSKGGLSNV
Q2KIR7	LPEVINWKQHLQIQSTQSSLNEVIQNLAATKSFKVKRSKNI
Q9D6E4	SREPTDTNVLRYIQLTQLKMNRCSLQREDSDGGTGGPARNV
O95685	VCLERVTCSDLGISGTVRVCNVAFEKQVAVRYTFSGWRSTH
Q3T0M9	ELHKITRISENQGVPVLIVANKQDLRNSLSLSEIEKLLAMG
P57076	LWWAAKELRRTKKLSDYVGKNEKTKIIAKIQQRGQGAPARE

Q9CWQ3	DTNPYVKLLLDAMKHSGCAVNRGRHFSCEVCDGNVSGGFDA
A4QPB2	KANRDVIIDQLPDLMGLKAVNVDKVVGTNPHADRNGGAATC
Q3U4G0	SFGDECSHHAYLHGQFWSYWNRFGPGLVIYWYGFIQELDCN
O43247	QQDLDPWKAAYSSLDTSKFKNQGLSSPQPLPLGASAQGSSL
P61086	VSSPEYTKKIENLCAMGFDRNAVIVALSSKSWDVETATELL
F1MIW6	CCPEVAKSYGHKLLTLPSPYNYLNPLDSAWSSLKWFIINNR
P0C866	TGSRVLNKNYTLLVSMAPLTNEIRPVSSCTPQHIGPAIPEV
Q9CYX7	STVSKKDFISVLRGMDGTSRNSPAGKSPKARQTEVKSEESP
Q9Y3B9	GDAEPCDKENENDGESSVGTNMGWADAMAKVLNKKTPESKP
Q6NTF7	RLRRPYYPRKALLCYQLTPQNGSTPTRGYFENKKKCHAEIC
Q91WK7	RRLGPTGKEVHALKRLRDSANANDVETVQQLLEDGADPCAA
P52823	THEAEQNDSVSPRKSRVAAQNSAEVVRCLNSALQVGCGAFA
Q9H5V9	TIDEEEEEIEAREVADSYAQNAKVIEKQLERKGMSKRRLQE
Q5E9C0	PRGFGSLPALEVLDLTYNNLNENSLPGNFFYLTTLRALYLS
Q9D0A3	GDGPFIDSLAKLELGTVTKCNFAGDGKTGASWTDNIMAQKS
P27512	EFSAQWNREIRCHQHRHCEPNQGLRVKKEGTAESDTVCTCK
Q8TAG5	DANYGELQEHKAQAYLKVNANSHARRMQAFEASPMWLQDMK
Q6ZTW0	RLGRALWHLRLAHHSQRAAFNNNVSVAYECLSAGGRRKRPG
P20339	MNVNEIFMAIAKKLPKNEPQNPGANSARGRGVDLTEPTQPT
A1E960	LISQRLLSASNSHELLLNLNNGQLLPLQFQGAFNSWIPPFP
Q03402	ENLFMSSYLVPWSSVIQGWYNESKGLIFGVGPKQNVSVVGH
Q30154	RWFRNSQEEKAGVVSTGLIQNGDWTFQTLVMLETVPRSGEV
P20490	RIGQELESKKVPDDRLYEELNVYSPIYSELEDKGETSSPVD
Q86W47	DEHQLLTNPKCSYIPPCKRENQKNLESVMNWQQYWKDEIGS
Q91X91	TLKARFPSVSVEASGGVTLDNLTQFCGTHIDVISLGMLTQA
Q3TQI7	KDKVSEEEDLHLGTSFSAETNRRDEDADMMKYIETELKKRK
A8MWL6	YGEGYSNTHKSKQMYCVFNHNEDACRYGSAIGVLAFLASAF
Q1JQA8	QIKRANKLFTNDCIFLKKTLNIPVISEKPLLFNGLNSVDSP
O09044	ASGGYIKRITNDAREDEMEENLTQVGSILGNLKNMALDMGN
Q8CDS7	DSAKRRQCLQYLNALRSLQHNGYKTVYFGETEIPETLVTGE
P14847	SFSVFSYATKKNSNDILIFWNKDKQYTFGVGGAEVRFMVSE
P13789	EKFDLQEKFKQQKYEINVLRNRINDNQKVSKTRGKAKVTGR
Q60930	GWLAGYQMTFDSAKSKLTRSNFAVGYRTGDFQLHTNVNNGT
Q8QZW2	ALYKLPAGLAPLPFPGLSKLNLMAASAGVAPAGFSYWPGPN
Q96CS2	ESEAKYLQDLLMESVNFSPANLSSTGSRYLNALVDSAVALE
Q5E995	RPRRTGERKRKSVRGCIVDANLSVLNLVIVKKGEKDIPGLT
Q8R2Y9	APQATTGPPAASPASENQNGNGLSTQLGPVGGPHPSHTPSH
Q5U3C3	LACPPCRGAIVVFKLQMHMLNGALLALLFPVVNTRLLPFEL
Q8IUI4	ERSSMLPTMAAGPNSILFAINIDNKDLNGQSKFAPTVSDLL
P79391	CPQDWLWHEENCYQFSSGSFNWEKSQENCLSLDAHLLKINS
Q99N10	AKSWEPEQERLTWQPGTVSMNTVTSPGPMANSVYVVAPPNS

Q96HF1	CEACKNKNDDDNDIMETLCKNDFALKIKVKEITYINRDTKI
P62701	TDITYPAGFMDVISIDKTGENFRLIYDTKGRFAVHRITPEE
Q5E9C0	LPPELGNLDLTGQKQIFKAENNPWVTPIADQFQLGVSHVFE
Q16878	HGSSIHDHTNSHCFLKMLQGNLKETLFAWPDKKSNEMVKKS
Q32KN9	SSLFGVSRAVQEFGLARFKSNVTKTMKGFEYILAKLQGEAP
Q8BGA2	DWLIGKAKTRSGSGDEQAGMNSEPHYLGILCIRTPAMQQVS
Q92564	VFHQFLEQSKYKVINKDQWCNVLEFSRTINLDLSNYDEDGA
Q8WWC4	EKPQQHQKTKMIVLGFSNPINWVRTRIKAFLIWAYFDKEFS
P35428	PADIMEKNSSSPVAATPASVNTTPDKPKTASEHRKSSKPIM
Q9BQ24	TFLVTFGNSEKPETMTCRLSNNQRYLFLDGDSHYEIEIVHI
Q92913	TYSSMIYRQQQSGRGWYLGLNKEGEIMKGNHVKKNKPAAHF
P55918	SCHFANLNGFYLGGSHLSYANGINWAQWKGFYYSLKRTEMK
Q9CZ04	LAAKVKCIPYAVLLEALALRNVRQLEDLVIEAVYADVLRGS
P07451	PSLQPWSVSYDGGSAKTILNNGKTCRVVFDDTYDRSMLRGG
Q96H79	CLLYNKGEALYGYCNLKDKCNKFHVCKSFVKGECKLQTCKR
P55041	HCRRSWSSDSTDSVISSESGNTYYRVVLIGEQGVGKSTLAN
Q8K5B8	FRASGAQLPGLPSLSYPRRDNVCSLPWPSAEPCNGYPQPYL
P35428	FLIPNGAFAHSGPVIPVYTSNSGTSVGPNAVSPSSGSSLTS
Q8R2N1	DQFIGTAALIVCVLAIVDPYNNPVPRGLEAFTVGLVVLVIG
O60487	SILLWKLQFDDNGTYTCQVKNPPDVDGVIGEIRLSVVHTVR
P09661	EIRKLDGFPLLRRLKTLLVNNNRICRIGEGLDQALPCLTEL
Q6IMB1	NKGDLLHARQVQTHEGLQLANELGSLFLEISTSENYEDVCD
Q02399	NVVPKLNATGRDLLQNLLKCNPVQRISAEEALQHPYFSDFC
P13349	SPVWSRKSSTFDSIYCPDVSNVYATDKNSLSSLDCLSNIVD
Q91XF0	KGKELDSNPFASLVFYWEPLNRQVRVEGPVKKLPEKEAENY
Q9UKK9	ARPKPKPGDGEFVEVISLPKNDLLQRLDALVAEEHLTVDAR
Q9UIW0	RTELARQLNLSETQVKVWFQNRRTKQKKDQSRDLEKRASSS
Q504P2	TWQESVMACSARNASLLKVKNKDVLEFIKYKKLRYFWLALL
Q9D4G1	SLYKGWQTCMFISFLDVALFNGDSSLKSYSIDNISSLASDF
Q922H7	LQLASMLGCSFYEVSVSENYNDVYNAFHVLCKEVSPKQQPS
B7ZAP0	TSKIALRNDLDQAEDKADVLNKELLLTKQRLVETEEEKRKQ
P33681	GGFPEPHLSWLENGEELNAINTTVSQDPETELYAVSSKLDF
P62820	KEFADSLGIPFLETSAKNATNVEQSFMTMAAEIKKRMGPGA
Q8TBZ9	EQIIKYLCKSEEFMDPATFINMYNRPIHITLDSEVVWPAPS
P49721	LLLCVGEAGDTVQFAEYIQKNVQLYKMRNGYELSPTAAANF
Q3T0I4	ADALKAMKQYNGVPLDGRPMNIQLVTSQIDTQRRPAQSVNR
Q9CQJ2	LNPEWRMLKYRSFLGSISQQNIRSQQRPRIQELGTLDASGS
P53994	EAFAREHGLIFMETSAKTASNVEEAFINTAKEIYEKIQEGV
Q13571	FLLITMLFIISLSLLIGVVKNREKYLLPFLSLQIMDYLLCL
Q6IRU5	KKDLEEWNQRQSEQVEKNKINNRIADKAFYQQPDADTIGYV
Q80XD9	QSSCIALDAHLALFDSLEELNFLKRYKGASDHWIGLHRESS

Q0IIL5	LQSNALLVQLPELPSKNLFFNMNNRQHVDQRRQGLEDFLRK
Q924N9	QRVPLQPPYQLHEVKIPIQDNKSCKRAYRKKSSDEHKAVAI
Q0IIG8	FEELVEKIIQTPGLWESENQNKGVKLTHREEGQGGGACGGY
Q58D63	ERSCPIMSGGPKYEYRWQDENKFRRPTALSAPRYMDLLMDW
P15946	LSLGGIDAAPPVQSRIVGGFNCEKNSQPWHVAVYRYNKYIC
P31955	KNGKGRRNKKKKNPCTAKFQNFCIHGECRYIENLEVVTCNC
Q9Y294	QRNILASNPRVTRFHINWEDNTEKLEDAESSNPNLQSLLST
P61006	TITTAYYRGAMGIMLVYDITNEKSFDNIRNWIRNIEEHASA
P14901	MASLYHIYTALEEEIERNKQNPVYAPLYFPEELHRRAALEQ
P10628	QIKIWFQNRRMKWKKDHKLPNTKGRSSSSSSCSSSAAPGQH
A1L157	VYYQRLSDELKQHLNRTLAENYGQPGATQITASVDRLQQDF
P35270	LGALRELPRPKGLQRLLLINNAGSLGDVSKGFVDLSDSTQV
P14483	GECYFTNGTQRIRYVTRYIYNREEYVRYDSDVGEHRAVTEL
Q8K1E6	WTLKSRIEENTSHTFNSLLCNFYRDEKDSVDWHSDDEPSLG
O15266	SEARVQVWFQNRRAKCRKQENQMHKGVILGTANHLDACRVA
Q8BRX9	CTGTLGTIHRSCLEHWLSSSNTSYCELCHFRFAVERKPRPL
O89116	MGKLETDFKRSRIAYSDEVRNELLGDAGNSSENQRAHLLDN
A5D989	AERKFYEQMNGPVAGSSRQENGASVILRDIARARENIQKSL
P0C7Q3	FQVSFQHPHKYLLYYLVSLKNWLNCHSWQRTPVAVTAWALL
Q6PK04	IPFRLREIMRSRQEMKNPISNKKRKKAAQVTFRKTLEKEAK
O88452	IVEMIHFKDLLLHEPYVDLVNLLLTCGEDVKEAVTRSVQAQ
Q64695	QIYLTQFESLVKLVYRERKENVFFPLTVSCSLGCELPEEEE
Q9BQ15	YSEVPNFSEPNPEYSTQQAPNKAVQNDSNPSASQPTTGPSA
Q9UBX7	WLLTAAHCLKPRYIVHLGQHNLQKEEGCEQTRTATESFPHP
P68509	QACLLAKQAFDDAIAELDTLNEDSYKDSTLIMQLLRDNLTL
O95992	LATQYMSVWELFSLGFFDMMNVTLLGCHPLTTLTFHVVNIW
Q17R31	AKRLNLPVNVHSRSAGRPTINLLQEQGAEKVLLHAFDGRPS
Q2KIR7	ILYMASETIKELTPSLLDVKNLPVGDGKPKAIDPEMFKLSS
P28845	AEQFVAQAGKLMGGLDMLILNHITNTSLNLFHDDIHHVRKS
Q9P0L0	VLDPPTDLKFKGPFTDVVTTNLKLRNPSDRKVCFKVKTTAP
Q6P9H5	EEQEAQLRELMEKVEAIMWENEGDYYSNKAYQYTQQNFRLK
Q99LU8	KSALFEVSEVIPVMTNNYEENILKGVRDSSYSLESSLELLQ
Q80SV1	YWLFGSQLGKPVSFSTFRRCNYPVRGDGHNLIMVEECGRYA
Q9UEU0	AGTEEKKKLIRDFDEKQQEANETLAEMEEELRYAPLSFRNP
P07478	CYKSRIQVRLGEHNIEVLEGNEQFINAAKIIRHPKYNSRTL
P62702	DANGNSFATRLSNIFVIGKGNKPWISLPRGKGIRLTIAEER
P35232	QKKAAIISAEGDSKAAELIANSLATAGDGLIELRKLEAAED
Q0VCB1	FQSIQEYDSGTYRCDVQLLKNLRLVKRLYFGLRVLPPNLVN
Q16740	SPGGVVTAGLAIYDTMQYILNPICTWCVGQAASMGSLLLAA
A6QP24	RLKQELMSMKEVGDGLQDQMNCMMGALQELKLLQVQTALEQ
P21337	SISHFTLGAVLEEQATNQIENNHVIDAAPPLLQEAFNIQAR

Q8VI33	GSKNILITTNMVSQNTAESANALKRKREDDDDDDDDDDDDD
A4FV27	RVLLMLFHTVTRIMAEQEVENLSGLSTNPEKDIFVVRENGT
Q2T9T5	LVGLGCLGDCLGLEWLDLSGNALTQLGPLASLRQLAVLNVA
O43731	ETITTHYLFFLGLYRALYLANWIRRYQTENFYDQIAVVSGV
P10649	AYDILDQYRMFEPKCLDAFPNLRDFLARFEGLKKISAYMKS
P21237	FLTMVISYFGCMKAAPMKEVNVHGQGNLAYPGVRTHGTLES
P62826	SRVTYKNVPNWHRDLVRVCENIPIVLCGNKVDIKDRKVKAK
Q96K30	GGLHSSRPLKRGLSHSLTHLNVPSTGHPATSAPHTNGPQDL
Q9CR02	LDSNTLQTFREWDFDLKKLPNIKMRKLCADDAVPKKRKQKN
Q5T0U0	DFEEHMIKYNAYYAKIKAHKNSLGEVESKWSFMTELHEKRD
Q9H1R2	RIPVADTPEVPIKKHFKECINFIHCCRLNGGNCLVHCFAGI
Q9H2C2	SCQYRCIECNQEAKELYRDYNHGVLKITICKSCQKPVDKYI
O95473	TFFSILVWIFQAYLAFQDLRNDAPVPYKRFLDEGGMVLTTL
A6NC51	LWTGLLCALGTSVVGNFQEKNQRPTHLAGAFLAFILGNVYF
P45880	GTLETKYKWCEYGLTFTEKWNTDNTLGTEIAIEDQICQGLK
Q96S79	NGHVHDLQILDFPPISAFPVNTLQEWADTCCRGLRSVHAYI
Q9D0M0	HTDILVGVKAEMGTPKLEKPNEGYLEFFVDCSANATPEFEG
P09012	PQQLMPGQMPPAQPLSENPPNHILFLTNLPEETNELMLSML
P56857	IISGICAIIGVSVFANMLVTNFWMSTANMYSGMGGMGGMVQ
Q9NQY0	QIQKTVIEPLKKFGSVFPSLNMAVKRREQALQDYRRLQAKV
P19075	FQHYPELCACLDKQRPCQSYNGKQVYKETCISFIKDFLAKN
Q969J5	QSTHESLKPQRVQFQSRNFHNILQWQPGRALTGNSSVYFVQ
Q6ZUJ4	ENWRKENLRKDMERDLKADSNMPLNNSSQEVTKDLLDMIDH
Q96MW1	EKDDSGATTMNIGSDKLLFRNTNVEDVLNARKLERDSLRDE
A5D7A0	SRRLQMEGEGGGEAPEQPGLNGAAAAAAAAGAPDETAEALG
A4QPB2	PEMGLTYWIDWGENPEIKRANLDRQELRVLVNASLGWPNGL
Q01730	PPELGNLDLTGQKQVFKAENNPWVTPIADQFQLGVSHVFEY
P62079	QQIVIYTKGCVPQFEKWLQDNLTIVAGIFIGIALLQIFGIC
P19437	RLELIQTSKPYVDIYDCEPSNSSEKNSPSTQYCNSIQSVFL
Q9JL59	EHEVSVNRGSRVVMACNISNNLRDVTIELVTSEKTSIIFNH
Q9CPY3	VPDVHTVRRSPRISFILEKENNPPLKVPTKEDLFKTCSVPG
Q0P5E2	CEALNRSVQVVNLDPAAEHFNYSVMADIRELIEVDDVMEDS
P40337	EPSQVIFCNRSPRVVLPVWLNFDGEPQPYPTLPPGTGRRIH
Q9NVX0	FVNFTRLQQITNIQAEIYQKNLEIELLKLEKDTADVVHPFF
Q6ZSR6	LPKCWDYRHEPPRLAPLLIFNPHPSTVLSCNCEYNSFFEFC
Q8VC04	VRPVGLNSSTVTFEDAHVQLNTTNVLNIFNSNFYPITVTQL
O95926	EQRLRKFRELHLMRNEARKLNHQEVVEEDKRLKLPANWEAK
Q86UD7	RAGAAVDLGHEQVDVRKYTNNLGIVHEMELPHVSALEVKQR
Q1JQA7	QEAPSSCLESSLAEIFPLGKNCASTFNSDSGTPGLAASVLV
Q3T0Y9	TLTAGQPREAGEEEQDAVTTNRAAARRLAAHLLLLVLLIIL
Q08DM6	QYQSCRVVGSVASAGPDGARNEPLQQLLDPSTLQQSVESRY

P58417	ESSKDLSISRLLSQTFRGKENDTDLDLRYDTPEPYSEQDLW
Q9H2W6	TAERTLATLSENNMEAKFLGNAPCGHYTFKFPQAMRTESNL
E1BC52	RLGTVERPRGGGGGFFGHDQNRAEAKENLRSAETFEKEDDL
Q9P2W1	GRAEAAAGAAGILLRYLQEQNRPYSSQDVFGNLQREHGLGK
P49427	RTILLSVISLLNEPNTFSPANVDASVMYRKWKESKGKDREY
P55918	TYHSGQKFSTFDRDQDLFVQNCAALSSGAFWFRSCHFANLN
P09038	LLASKCVTDECFFFERLESNNYNTYRSRKYTSWYVALKRTG
Q5JBG6	IPFSTFMESDLIGVIHPGYKNRISILYPWERRKNATCYLGY
Q2TA03	EFPFKPPSIYMITPNGRFKCNTRLCLSITDFHPDTWNPAWS
Q8TAE8	AECMAKMPQMIVNWQQQQRENWEKAQADKERRARLQAEAQE
P0C0E4	QAQAYAERLGVTFFEVSPLCNFNIIESFTELARIVLLRHRL
Q1LZC0	RLGKPKGGISRSSSQGKVYENQRKTGRQRQRWGMTVRFDSS
Q6PZD9	KGKKAVNKDSLEYRLRRERNNIAVRKSRDKAKRRIMETQQK
P15945	AQHRLVSKAIPHPDFNMSLLNEHTPQPEDDYSNDLMLLRLK
P30041	VFGPDKKLKLSILYPATTGRNFDEILRVVISLQLTAEKRVA
Q0P5H5	SSKAKKIYTDFIEKEAPKEINIDFQTKSLIAQNIQEATSGC
P51164	GTFSLHYFPYYGKKAQPHYSNPLVAAKLLNIPRNAEVAIVC
Q3UBZ5	REQLRACSLQGWVCYVTFICNIFDYLRVNNMPMMALVNPVY
P02720	KECSGETVVYWQVKYPFPMSNRDYVYVRQRQELDFEGQKVH
Q9CQT2	SPHADQLGFSPSAQPHGHTFNQSSSSQWRQDALSSQRKRQN
Q96FV3	LQNLIDFAQEYWSCCGARGPNDWNLNIYFNCTDLNPSRERC
Q16637	SDLLSPICEVANNIEQNAQENENESQVSTDESENSRSPGNK
Q8IZS5	RTLAAHQQKFRLPASAEPKGNEYGRNYFDPLMDEEINPRQC
Q8BXX9	HRTNNERRSYIAEMTQVSGSNQVSKRQQMDPLPRMKESPVK
Q92876	GDSGGPLVCGDHLRGLVSWGNIPCGSKEKPGVYTNVCRYTN
Q8VI64	SVPESTQEVESAAPDQDDVCNEGADTSQEGADTSQEGADTS
Q8NI29	PDPIPQWNNNACLHVTHVFSNIKMGVRFVSFEHRGQDTQFW
Q8N755	TWSLSSYTCATRIITTLMTTNDFTILLRFVIMLALNIWVTV
Q9DB05	VEKAIAHYEQSADYYKGEESNSSANKCLLKVAGYAAQLEQY
Q03401	KCTFSHSPIELRTTNLRCGENLFMSSYLASWSSAIQGWYNE
Q92730	AFTSEKSIHSIFRTASMLCLNKPSPLPQKSPVRSLSKRLLH
Q6PHN7	MFFYHFSFLQILGLVTEVNLNNMLCPAISDPFYGPWYRIWA
Q9UBX7	ATESFPHPGFNNSLPNKDHRNDIMLVKMASPVSITWAVRPL
P52943	SGPPKGPSRASSVTTFTGEPNTCPRCSKKVYFAEKVTSLGK
Q9DAK2	LLPYYRQILPILNIFKNMNVNSGDGIDYSQQKRENIGDLIQ
A4IFA7	LSCDVAKEHDVQNTFEEIEKNLGRVNFLVNAAGINRDNLLV
Q99M71	CQAPQQWEGRQVLYQQSSGHNNRALVSYDGLNQRVRVLDER
A2AUU0	HPVAPLGSRILTDPAKVFEHNMWDHMQWSKEEEDAARKKVE
Q96L16	ISRLEPVTKTKPWREPLWERNWQGQHLDSRGYLAGPGREDG
P04768	SHNISIEVSELFNEFEKHYSNVSGLRDKSPMRCNTSFLPTP
Q96DN0	GVSFGISTDSEVLTHYNITGNTICLFRLVDNEQLNLEDEDI

Q7L4S7	CNSFLQQAGLNLLISMTVINNMLAKSASDLKFPLISEGSGC
P52840	LSLLPQSLLDQKIKVIYVARNAKDVVVSYYNFYKMAKLHPD
P60033	TLDCCGSSTLTALTTSVLKNNLCPSGSNIISNLFKEDCHQK
P79125	TGVRIRNVQVPDITFEATSENSANVLIPITADVTVSLPFLG
Q9BYN8	VLAERKALKDAAEHRELMAWNQAENRRLHELRIARLRQEER
Q8N5S3	HHSKSHVGRGRIYYAKFINTNARTYNEPFPYIDPKKGPEIQ
P30405	ENFTLKHVGPGVLSMANAGPNTNGSQFFICTIKTDWLDGKH
P30041	NAYNCEEPTEKLPFPIIDDRNRELAILLGMLDPAEKDEKGM
P23889	IFSSISKRSIGAIEANQGLFNYRPVKPIDTIIARALLETLI
Q17QN8	LLQVQDHDQPIPWKVQFNLGNSSRPSNQCRNSIQGKHLITD
Q5SZD4	QGLQSELYDVSKAVANSKQLNIKLTSFKAVHFSPVSSLPDT
Q8WUN7	HDSSGSLNENSEGTGVALGRNQPLKKEKPKWKSDYPMTDGQ
Q96PB8	NQITSIPNEIFKDLHQLRVLNLSKNGIEFIDEHAFKGVAET
Q9DAT2	SPEVEVCLFHAMLGHKPVGVNRHFHMICIRDKFSQNIGRQV
P52803	NFDGYSACDHTSKGFKRWECNRPHSPNGPLKFSEKFQLFTP
O15520	GSKEFNNDCKLKERIEENGYNTYASFNWQHNGRQMYVALNG
Q9UGN4	GPVGGSLSVQCPYEKEHRTLNKYWCRPPQIFLCDKIVETKG
Q8IUH2	TGIPFFYMTAKDPVVADLMKNPMASLMLPESEGEFCRKNIV
Q9D819	IPQTWEDPGHSDKHTGCCGDNDPIDVCEIGSKVCARGEIIR
Q96BS2	LHRRFKQLSGDQPTIRKENFNNVPDLELNPIRSKIVRAFFD
Q2KIP7	PELNQYIQDTLHCVKPLLEKNDVEKVVVVILDKEHRPVEKF
Q4KUS1	CPRQRLECRNESLSSCKTDFNCKAHFKCCQFACGRKCMDPY
Q3SWY4	RAELKGLNPGGAYVVCVVAANDAGESRAPGPGAEGLDSADG
P60191	QASHESIEDSMNSYGSEGNLNYGGVCLASDAQFSDFLGSMG
P17981	MSYPAGFHDFHGTALVAYTGNIHRSLNEATREFKINPGNFH
Q9S169	HLADGMTVGELCAAAITMSDNSAANLLLATVGGPAGLTAFL
Q5NCY3	MDQTLEENGIRDEEEEFDYLNMDGKLHTPAILLYFNDDLTE
Q99LD8	DGTDVLFTGREFFVGLSKWTNHRGAEIVADTFRDFAVSTVP
P05839	YINLPMVPVPSWCAHPETASNTMHAMASRGYDYPVDFPRLI
Q3SZ48	QSLINLDGKLHQHVVKCTVCNEATPIKNPPAGKKYVRCPCN
Q9Y5X0	RIQKEDFWHSYIDYEICIHTNSMCFTMKTSCVRRRYREFVW
Q13907	AQSDGIWGEHEIDYILLVRKNVTLNPDPNEIKSYCYVSKEE
Q28151	QKQYVIKHQNTHTGEKLLECNECGKSFSQKENLLTHQKIHT
Q9UBP6	IRALRAAPAGGFQNIACLRSNAMKHLPNFFYKGQLTKMFFL
P35282	NWVKELRKMLGNEICLCIVGNKIDLEKERHVSIQEAESYAE
P25401	KGAEEGISLQSRPDGRTMLVNTTPYIFAIGSLLDGNGKKIA
Q9D7L8	ENYILDTQHGVQASLECAVQNHTEDEELLWYREDGIVDLKN
P24071	LVLVALLAILVENWHSHTALNKEASADVAEPSWSQQMCQPG
Q9D9B0	TFWKKYRTFWKEDKAFWREDNALWERDRNLLQEDKALWEEE
Q96DX5	TASEELACLLMDFGADTQAKNAEGKRPVELVPPESPLAQLF
Q95JH2	ILFLAVLAIPVCAVRGRNVENMKILRLMLLHIKYLYGIRVE

Q3ZBP0	NNFTSIKFTQRNEPNDCRLSNKAIFLHTSSQCSEEEQIMIG
Q2NL23	DAGQVGPAAAEPRRQSPPSKNQRERARSGALPLGALYTAAA
Q0V7M7	DVEHLKENIPPHLPQVTVTQNFVNGSDLDPEEPVKVEEPAP
O14753	HMKCHNDVKRHLCTYCGKGFNDTFDLKRHVRTHTGVRPYKC
Q2NL11	SITEAVTILGNKLRDYQGQFNTTHLLALCDYFHNTFIRHYK
P45379	RKRMEKDLNELQALIEAHFENRKKEEEELVSLKDRIERRRA
Q4G0S7	DQSSEGCMKKISSVNLDKLINDFSQIEKKMVETNGKNNILD
P60033	LAGGVILGVALWLRHDPQTTNLLYLELGDKPAPNTFYVGIY
Q28030	RYYEQPDNGVLNYPKRACQFNRTQLGDCSGIGDPTHYGYST
Q96FF9	VPAVQSPRRSPRISFFLEKENEPPGRELTKEDLFKTHSVPA
Q9NV56	SPEVEVCLFHAMLGHKPVGVNRHFHMICIRDKFSQNIGRQV
Q9D1G1	TTAKEFADSLGVPFLETSAKNATNVEQAFMTMAAEIKKRMG
P25322	ISNPPSMVAAGSVVAAMQGLNLGSPNNFLSCYRTTHFLSRV
Q2HJJ1	ESQLGLWGGEGWLKGHRYVNNDKFSKRVKKVWKPQLFQREL
P13760	NSQKDLLEQKRAAVDTYCRHNYGVGESFTVQRRVYPEVTVY
Q3SZC6	NRICGGLDMLAEKLPNLTHLNLSGNKLKDISTLEPLKKLEC
Q9BQ24	FYDKQLKVLLSGATFLVTFGNSEKPETMTCRLSNNQRYLFL
Q8CAK3	NRREPHVPGTSCAHPKSRKQNHLPKVLHPSNPHISSGSTVA
Q9NY30	PKEMTIWVDPFEVCCRYGEKNHPFTVASFKGRWEEWELYQQ
Q96S90	SIFLKKTLYIPILTEPRDLFNGLDSEEEKDGEEKVHPSNSE
Q3SZX8	ANSIQGCKMNNVNVVYTPWSNLKKTADMDVGQIGFHRQKDV
Q2TA24	HRNYHAGYSMFGAGLTVGLSNLFCGVCVGIVGSGAALADAQ
Q99714	TIAPGLFGTPLLTSLPEKVCNFLASQVPFPSRLGDPAEYAH
O15247	QVDPEIELFVKAGSDGESIGNCPFCQRLFMILWLKGVKFNV
P22615	IYLISRAVNQFPEFRMALKDNELIYWDQSDPVFTVFHKETE
P19071	WRKTAPKYGFGRTELMPVRSNSEGIGRYVGKYISKGIESRT
P79391	ETLAHKLDEKSKKLMELHRQNLNLQEVLKEAANYSGPCPQD
Q3SZW3	VTMTSNTDASASCQYRCIECNQEAKELYRDYNHGVLKITIC
P28069	HFGEQNKPSSQEIMRMAEELNLEKEVVRVWFCNRRQREKRV
Q58DS9	TDTFARAKNWVKELQRQASPNIVIALAGNKADLASKRAVEF
P23025	EFDYVICEECGKEFMDSYLMNHFDLPTCDNCRDADDKHKLI
Q8BMB3	SDFHLFKEGIKPMWEDDANKNGGKWIIRLRKGLASRCWENL
Q2HJ38	QVQSTLLALASMAKTKGNKVNVGVKYAEKQERRFEPEKLRE
Q9QZ28	KRELAQATGLTPTQVGNWFKNRRQRDRAAAAKNRLQQQVLS
P63011	GQKESSDQNFDYMFKILIIGNSSVGKTSFLFRYADDSFTPA
P29084	QHLDIGLKQKQWLMTEALVNNPKIEVIDGKYAFKPKYNVRD
Q8BK84	PGAFELERLFWKGSPQYTHVNEVWPRLHIGDEATALDRYGL
P62906	GNQRKRRKFLETVELQISLKNYDPQKDKRFSGTVRLKSTPR
Q1JQA7	LSRSELTSVSPNTGMTVFILNFEKGGKGRPTAQCVCVNLQG
O14925	LLYSAFGVIIEKTRGAEDDLNTVAAGTMTGMLYKCTGGLRG
P04481	YAHALYQLTCPVPVDSKLERNTLTALLNVASWLKRKPGTPE

Q3UZZ6	ISTYPKSGTTWVSEILDLIYNNGDAEKCKRDAIYKRVPFME
Q5TA50	EGLRTSPEDARTSALCADSYNASLAAYHPWVVRRAVTVAFC
P32972	TLKSTPSKKSWAYLQVSKHLNNTKLSWNEDGTIHGLIYQDG
Q9DCH6	NRCFMCRKKVGLTGFECRCGNVYCGVHRYSDVHNCSYNYKA
A6NFK2	YGGRDQHDRPLVEAESTLPQNRYTQEGDIPEDSCFHCRGSG
P25444	KQTRAGQRTRFKAFVAIGDYNGHVGLGVKCSKEVATAIRGA
Q96NT3	KGYKNQSFYRKHFDTEETRVNQLFAQAKACKVLVEKCTVSV
O15305	KVQEQLGNDVVEKYDYVFPENGLVAYKDGKLLCRQNIQSHL
Q3V2J0	RLRDKADQLVKQLIDFANTENPELRATIRDFAEDLAKVQDY
O43708	KQVGEEMQLTWAQNAITCGFNALEQILQSTAGIYCVGDEVT
Q9Z2F7	ENGNGKNGGLEHVPSSSSIHNGDMEKILLDAQHESGQSSSR
Q3SZU4	HLYSPPFDTCHAFDQRTGHKNKVIMTFHSKFGIKTPFTTSG
Q32LN0	SMSLQEFTRAAGTAGQLLYSNLQHLKWNGQCSSDLFQSTHN
Q9BRK0	RDKSYETMMRVGKRGLNLAANAAVTAAAKGVLSEKLRSFSM
Q9DCC4	NLPCVVQEGAMVMARGHHAGNDDAELLQNLLEACGQCIEVP
O18870	DFKRETCVVVYTGYGNREEQNLSDLLSPTSEVANIEQNAQE
P62577	HDDFRQFLHIYSQDVACYGENLAYFPKGFIENMFFVSANPW
Q9P299	EIVDGGVILESDPQQVIQKVNFRADDGGLTEQSVAQVLQSA
P18124	EYRQMYRTEIRMARMARKAGNFYVPAEPKLAFVIRIRGING
P56856	SGIMFIVSGLCAIAGVSVFANMLVTNFWMSTANMYTGMGGM
P68250	RYDDMAAAMKAVTEQGHELSNEERNLLSVAYKNVVGARRSS
Q7TSP5	IGFGISGKHFITVTTFTSAGNIGEDGTLSCTFEPDIKLNGI
P45879	FGIAAKYQIDPDACFSAKVNNSSLIGLGYTQTLKPGIKLTL
Q3V038	SSRLSEEQSKTVEAIEIDCYNSLAACLLQAELVNYERVKEY
P51150	SPRDPENFPFVVLGNKIDLENRQVATKRAQAWCYSKNNIPY
P79107	TADPSKAVVLLDPQWNHVLTNDRVTLKCQGDYPVEDNSTKW
P26717	YYIGKERRTWEESLLACTSKNSSLLSIDNEEEMKFLASILP
Q8N4E7	PSRVRQNFHPDSEAAINRQINLELYASYVYLSMAYYFSRDD
Q1ZYL8	PGYFPNELRNIFREQVHLIQNAIIESRIDCQHRCGIFQYET
Q5EBJ4	DEQPVFGKKGDIARNSYSRYNTISYRKIRKGNTKQRIDEFE
Q8C4B4	GPGGLVAGKEEKKKAGGGVLNRLKARRQGPPHTPDDGSGAA
Q9JM71	IGGFKCKKNSQPWHVAVLRSNKYICGGVLLDPNWVLTAAHC
A6QLZ5	VNASGAAAASAFRESAQQMSNERGFENVELGVIGKKKKVPR
Q3SZM1	LFREWHMPFKRPSYPAVKRYNQNRSLVQKLRMEERFKKKEK
Q8K3B1	DDQSWGWNLVDNNLLHNGEVNGSFPQCNNAPKYQIGERIRV
P30932	LSVGLWLRFDSQTKSIFEQENNDSSFYTGVYILIGAGALMM
Q9BRN9	VKPSVTCVDQDFKSQKNFIINMTCRFCWQLPETDYECTNST
Q9D142	GVQKSWDFMKTHDSVTILMFNSSRRSLVLVKQFRPAVYAGE
Q6P8Y1	EEAEELFQRFDRDGSGTIDFNEFLLTLRPPMSRARKEVIMK
Q32L00	RLTAWYGELPYTYSRITMEPNPHWHPVLLMLKNQIEENTGH
Q9D8B3	QLAQIDGTLSTIEFQREALENANTNTEVLKNMGYAAKAMKA

A5PJT0	ERLIELVDRGLVEKVLPGHFNTFGAERLFRLASNYISKAGI
A6QQ24	GLARLRHQLFLLCFSAGNYYNQGEIRKKELLQSCDVLGIPP
P04228	LLGQPNTLICFVDNIFPPVINITWLRNSKSVTDGVYETSFL
Q92527	GQSLSLVEKLLEYEADLEAKNKDGYTPLLVAVINNNPKMVK
P08760	SRITKHFELKHLSSGDLLRDNMLRGTEIGVLAKTFIDQGKL
Q9NY12	PCEDDIVCKCTTDENKVPYFNAPVYLENKEQIGKVDEIFGQ
Q9BRQ6	SFGVDEEERVRVLQGVRLSENVVNRMKEPSSPPPAPTSSTF
Q1RML7	LGNKQFCSFKIRTKTQSFCRNEYSLTGLCNRSSCPLANSQY
Q12918	IGLNFSLSEKNWKWINGSFLNSNDLEIRGDAKENSCISISQ
Q3UPL6	THFGCQATRAQTNASVIFLPNAFGTDFNIPSPAVSPPPAYD
Q6PEY0	LVYMVAAEHVWKDEQKEFECNSRQPGCKNVCFDDFFPISQV
Q7Z7J7	LAFVLGNRQTDLLQEELKPENKDFVGSTVSSVLRPGGDVSG
Q92564	LQEWLKGMTSLQCDTTEKLRNTLDYLRSFLNDSTNFKLIYR
Q9Z0S4	GLLMLVPLSWVTHNIIHGFFNPLLGFSKKVQMGSSLSLAWT
Q6NXY9	EDAEQEDYDEEEQEEENDYINSYFDNGDDFGVDSDDNMDEA
Q9BXJ5	AIGLVHNGQYRIRTFDANTGNHDVASGSTILALKQGDEVWL
P47753	THAPPGEFNEVFNDVRLLLNNDNLLREGAAHAFAQYNMDQF
P20456	LLVTELGSSRTPETVRIILSNIERLLCLPIHGIRGVGTAAL
P23774	AEEMHISEISVRKRLEQECLNFNQLILDVRMNQAAKFIIRS
O75828	IRALRDFLRKEYGGLNVLVNNAAVAFKSDDPMPFDIKAEMT
Q9D7D7	LSGVVFFVAGLFSLIPVSWYNHFLSDPDVLAAPSSPVTVQV
P47756	QPLKIARDKVVGKDYLLCDYNRDGDSYRSPWSNKYDPPLED
P20764	VDGVPVTQGVETTQPSKQTNNKYMVSSYLTLISDQWMPHSR
Q6UX27	AEFLSLLCLGLCLGYEDEKKNEKPPKPSLHAWPSSVVEAES
Q2T9Y1	ALSKKVKPPKECFLIQPKETNEDATKTRKRKKKKITDILAK
Q3ZBM6	EGNQESEEDDTGPPARKLRRNQDDDDDDDDEGFFGPALPPG
Q60651	HELKEFLKHHNNCSIMQSDINLKDELLKNKSIECNLLESLN
Q8BWP5	KIAAVLTDSFPLKVRGIHLINEPVIFHAVFSMIKPFLTEKI
P24668	QDLGNLVADGCDFVCRSKPRNVPAAYRGVGDDQLGEESEER
Q2NL33	MTYATLTFQDSVAAGNNQDRNNLRKRGYPAPSSIWRQAALG
Q96EU6	FDDLSGEYNPEVFDKTYQFLNDIRAKEKELVKKQLKKHLSG
Q8TAK6	QQQQLRRKINSRERKRMQDLNLAMDALREVILPYSAAHCQG
Q3KNV8	PEEDNDYHRSDEQVSICLECNSSKLRGLKRKWIRCSAQATV
Q8TDN7	LGSGYSIWMPRCYFPSFLGGNRSQFIRLVFITTVVSTLLSF
Q8IWP9	KRRSPKSFSAHCTQVVNAKKNAIPVSKSTGFSNPASQSTSQ
P14206	TIALCNTDSPLRYVDIAIPCNNKGAHSVGLMWWMLAREVLR
Q08331	SGYIEANELKGFLSDLLKKANRPYDEPKLQEYTQTILRMFD
Q00356	SGGPLLCAGVAHGIVSSGRGNAKPPAIFTRISPHVPWINRV
Q0IIC4	RKEGNEYKMMPNRRNMYAVQNNSAGPYFNPRSRQRIPRDLA
P00918	LKEPISVSSEQVLKFRKLNFNGEGEPEELMVDNWRPAQPLK
Q0VB26	IRPKSTRRLGFTYSIGDPILNESQYHDEYTWKLRSKENMVK

P41247	LYVNIAKQDIMLSLANLVRLNQALFPPSKRKMESLYQCGFD
P11672	QPDFRSDQFRGRWYVVGLAGNAVQKKTEGSFTMYSTIYELQ
Q8IUA0	LPVRHGNCNHEAQRWHFDFKNYRCTPFKYRGCEGNANNFLN
Q62189	ATIATMPVPETRANHTIYINNLNEKIKKDELKKSLYAIFSQ
P20489	DSGKYICQKQGLFKSKPVYLNVTQDWLLLQTSADMVLVHGS
O14966	SMTRLYYRDASACVIMFDVTNATTFSNSQRWKQDLDSKLTL
Q80ZQ3	VWRHEQQVAELSTAEVEKRANVLMLKETLRMEQAQMLQKAF
Q9BTT0	KINLELRNRSPEEVTELVLDNCLCVNGEIEGLNDTFKELEF
Q4VAA2	VDYSGLRVQAMQISEKEDDDNEKREDPGDNWEEGGGGSGAE
A2VE33	NETFHFESQCCLGEPCNQTSNTTASPHQVGSGNMECPACYG
Q9NVP2	LNPELRENPPMKPDFSQLQRNILASNPRVTRFHINWDNNMD
Q86UD1	AVRQAEEVRGLEHLHMDVAVNFSQGALLSPHLHNVCAEAVD
Q91ZW7	QLKSEVDRLCRLCPWDWTFFNGNCYFFSKSQRDWHDSMTAC
Q8BHD0	QLWDTAGQERFRSITRSYYRNSVGGFLVFDITNRRSFEHVK
P54107	RVVPPASNMLKMSWSEEAAQNARIFSKYCDMTESNPLERRL
O88992	TYHVLMRGGDGTSMWADLCKNGQVRASAIAQDADQNYDYAS
P70447	GAKTAETVQRIKKTRRLKANNRERNRMHNLNAALDALREVL
Q59I47	LRLNDELRQLQFSELVGIVYNPVEYAWEPHRSYVTRYCQGP
O14668	STRLSNCDPPPTYEEATGQVNLQRSETEPHLDPPPEYEDIV
Q86W74	KLETMQTAESESAMESHSLLNPNLQQGEGVLSSFRTTWQEF
Q9WTP7	PRTLPQAEALDKVYQIDTVINLNVPFEVIKQRLTARWIHPA
Q32L35	EELRCRDAFYKEQLGRLERQNLEAYRLSSQQFHEAATKIEG
Q8R5M0	ITDNGAGYAFIKRIKEGSIINRIEAVCVGDSIEAINDHSIV
O15217	LSLADVILLQTILALEEKIPNILSAFPFLQEYTVKLSNIPT
A5D7K7	QFGYVYGMSAIGCLGIHALLNLMSSSGVSYGCVASVLGYCL
P04183	LVIKYAKDTRYSSSFCTHDRNTMEALPACLLRDVAQEALGV
P31944	FLKGEDGEMVKLENLFEALNNKNCQALRAKPKVYIIQACRG
Q64704	IRKSQHSVLSRKFVEVMTKYNEAQVDFRERSKGRIQRQLEI
Q99P30	DAKARLRKSDVGTRYSHLSSNKFSVLVPLLARGGKLYLMFT
Q2KI22	SIRPDELLHMELVLVNKLKWNLAAMTPHDFIEHFLSKMPVA
Q99811	TVPPYSPGSSGPATPGVNMANSIASLRLKAKEFSLHHSQVP
Q9Y3B8	LDIEKDQIIEMACLITDSDLNILAEGPNLIIKQPDELLDSM
Q9D5L7	GVATADAPLHSVGYTTLVGNNHESWGWDLGRNRLYHDGKNQ
Q8N5M1	QDTIKYYTMHLTTLCNTSLDNPTQRNKDQLIRAAVKFLDTD
Q9CRA7	KKIPWSCDSRYFWEWLNTVFNKVDYERLRDVGPDRAASEWL
Q8K201	SENPPNNATVSSPVVVTAPGNHTSPSVSQISTTLSPASAEK
O75832	RDEIVKALLGKGAQVNAVNQNGCTPLHYAASKNRHEIAVML
O08738	ADRDNLTRRFSDLGFEVKCFNDLRAEELLLKIHEVSTSSHI
Q9CQS9	FIEKFETHLETVKNSPHLDANLKQMSKALAKMDILVNKTEE
Q0VCV7	AKTPARPVGTSEPKSANLCGNRTYGKALMPPVARISVKAPT
P15483	SLKTLCVRGIPPKQGDLWANNEKEFVGMKLNVSINTCIKLI

O95976	CLDGCKSEADKFTVREALKENQVSLTVNRVTSNDSAIYICG
Q0P5F2	KTSESTSSLHTPFLKALKTQNFKEPPFCSLLEQPNIVHDLP
Q13253	RRKLQMWLWSQTFCPVLYAWNDLGSRFWPRYVKVGSCFSKR
Q7Z7J7	RNSRAIGVLWAIFTICFAIINVVVFIQPYWVGDSVSTPKPG
Q6S5G4	LLLGTAIESAWSDEQFEFHCNTQQPGCENVCYDHAFPISHV
Q86YB7	PDQGITEILMNRPSARNALGNVFVSELLETLAQLREDRQVR
Q9H560	AVHCQEEACAIILLEHGANPNIKDIYSNTALHYAVYNKGTS
Q70YC4	ESVSTSQSRNISLLTLGQLQNCVIGKLTIIDLLTEHLLGVR
Q9EQ48	YCSVYIRNAFFSEIGIGISANSCLLLFHTFMFIRGHRPRLT
P63162	DKHMNLILCDCDEFRKIKPKNAKQPEREEKRVLGLVLLRGE
P14106	VRGRDRDSMQKVVTFCDYAQNTFQVTTGGVVLKLEQEEVVH
P05531	LKLSKCSQSQTLEAIKDMHENYMEGLMNLETNNYNMLFDVD
Q3SZ72	GHVNLLLGAVLHGTVLRHVANPRGAVTPEYTTANVISVGSG
O35885	VPREHFSCAAPELVAGAQGLNASLMDGGALPRLMPTSSGVA
P70202	STEDTWYKMLKIQTVKQVQRNDDFIELDYTILLHDIASQEI
P22005	YGGFMKKMDELYPMEPEEEANGGEILAKRYGGFMKKDADEG
Q9NPC6	FKVPDFELLLLTDPRFMSFVNPLSGRRSFNRTPKGWISENI
Q148M8	LRSVERGQRFLAADGDSTRPNGSWAFAQRPTEQEMRARKAA
O70552	SREAQIIPKVNNPKSVYQVENFKQSLQPWFCLPRRKHLADG
Q0III6	EVLSDAKKRDIYDRYGKEGLNGGGGGGSHFDSPFEFGFTFR
P30932	DCCGLTGVPEQFLTDTCPPKNLIDSLKTRPCPEAIDEIFRS
P49427	SILHPPVDDPQSGELPSERWNPTQNVRTILLSVISLLNEPN
Q9HBM1	EIRKIYGEKLQFIFTNIDPKNPESPFMFSLHLNEARDYEVS
Q3U1J1	IVLEDDGSQGTDVPTPGNAENEPPEKEGLSPSQRTTATLDP
Q5KR48	EKLEQAEKKATDAEADVASLNRRIQLVEEELDRAQERLATA
Q9DAS2	GCFPTIFSPNSPLVLTDNSQNWDRWLHQPTYSRSNQDNGRT
Q96BH3	TNEYGGNSLRKPCIFPSIYRNNVVSDCMEDESNKLWCPTTE
Q9WU03	SCGVSKVVGKCRASIPRWWYNITDGSCQPFVYGGCEGNGNN
Q8BGS0	KVIERAAFPRRLWERVRLSKNYEKALEQIDENLIYWPRFIR
P82979	VKITSEIPQTERMQKRAERFNVPVSLESKKAARAARFGISS
Q9BZM6	GRGSWQFLFNGQKFLLFDSNNRKWTALHPGAKKMTEKWEKN
Q9QVN7	GKEEEIARIARRLDKMVTRKNAEGAMDLLRELKNMPITLHL
Q86T03	AADGERSPLLSEPIDGGAGGNGLVGPGGSGAGPGGGLTPSA
Q9H2R5	WVLSAAHCQSRFMRVRLGEHNLRKRDGPEQLRTTSRVIPHP
Q6PZD9	AVKEEPRGPEGNRGTSRGSYNPLQYQVAHCGQTAVHLPPTL
Q3T0Z5	GGTAAPEEQPEEVDERRRRYNSQFNPQLARRPRRQDPPVAP
P07738	DQLPRSESLKDVLERLLPYWNERIAPEVLRGKTILISAHGN
Q6UX82	VAAVESLSCVQCNSWEKSCVNSIASECPSHANTSCISSSAS
O95857	GQLLEVGWNNTASARNDIQRNLNCCGFRSVNPNDTCLASCV
P29965	GSALFAVYLHRRLDKIEDERNLHEDFVFMKTIQRCNTGERS
Q9DC07	VCKMALNMNNYKGYEKKPYCNAHYPKQSFTTVADTPENLRL

Q8K015	QTFSFSARLLYEDPTAALPTNVTVTRPGVEASSPPWEEHRA
Q9JJJ3	IGLLIIVISCSLGLNSGCAMNPARDLSPRLFTALAGWGFEV
P52797	YWNSSNQHLRREGYTVQVNVNDYLDIYCPHYNSSGVGPGAG
A8E4L3	GGGEALAVPFEPARLLPLATNGRLYHPAPERAGGVGLVRSA
P57729	DPETVVRLQLWDIAGQERFGNMTRVYYREAMGAFIVFDVTR
Q32L15	DKWSDRHRNCPICRLQMTGANESWVVSDAPTEDDMANYILN
P10747	TGNKILVKQSPMLVAYDNAVNLSCKYSYNLFSREFRASLHK
P51157	KQYKQTIGLDFFLRRITLPGNLNVTLQIWDIGGQTIGGKML
P58417	KDSKSFNCRIEYEKVDKATKNTLCNYDPSKTCYQEQTQSHV
Q32KY8	TISIREIEMQLSLGRRPIISNWLDYIPSTRYKDPCELLHLC
O43557	QLIQERRSHEVNPAAHLTGANSSLTGSGGPLLWETQLGLAF
P31041	KEFRASLYKGVNSDVEVCVGNGNFTYQPQFRSNAEFNCDGD
O60487	TALWPIAAVEIYTSRVLEAVNGTDARLKCTFSSFAPVGDAL
Q9NY30	HSDCPSKGQAFRCIRINNNQNKDPILERACVESNVDFSHLG
P09631	ACGSPPVDREKQPSEGAFSENNAENESGGDKPPIDPNNPAA
Q61754	ICDGILHGITSWGPVPCGKPNAPAIYTKLIKFASWIKDTMA
Q9NX53	TNGPPWNSLGAPWNTNGPPWNRHGAPPQNSLDAPWNSLGAP
Q9BYG3	GIDYDFPSLILQKTESISKTNRQTSTKGQVLRKKKKKVSGT
Q9D8N6	DLAQFSENTPLYPICRAWMRNSPTVRERERSPGSPLPPLPE
Q8R173	RCIRKMDHHCPWVNNCVGENNQKYFVLFTMYIALISLHALI
Q8TDC0	QEFVSYRDYQSDGRSHTPSPNDYRNFNKTPVPFGGPLVGGT
Q9H0X6	RKGSSELGFPRVAPEDEVIVNQYVIRPGPSASAASSAAAGE
Q07817	FVELYGNNAAAESRKGQERFNRWFLTGMTVAGVVLLGSLFS
Q80WR5	EEVRASVLQMDSSIFLDDDSNQPMPVSRFFGNVELMQDLPP
P19404	ETTPDKLFTLIEVECLGACVNAPMVQINDNYYEDLTAKDIE
P02741	DSFGGNFEGSQSLVGDIGNVNMWDFVLSPDEINTIYLGGPF
Q3Y5Z3	SVLLYLEKGDQVWLQVYEGENHNGVYADNVNDSTFTGFLLY
Q9QY76	HELKFRGPFTDVVTTNLKLGNPTDRNVCFKVKTTVPRRYCV
Q925N4	IGSVWYAVDVYVERSSLVLHNIFLGIQYKFGWSCWLGMAGS
P49888	KEDVIFNRIPFLECRKENLMNGVKQLDEMNSPRIVKTHLPP
Q9NQT5	VGGSEPASLSYLSFEGATKRNRPNVQVGDLIYGQFVVANKD
Q80X85	ALRAPLRWSGLALGVRCAVWNLPGLTQVRGSRYAPEFREPL
Q9CZB6	QEGLAPKPPPPIKDSYMMFGNQFQCDDLIIRPLESQGIERL
Q0ZUP1	LKYEEVDKVWKWMNGSILNTNLLQITGKDEENSCALISQTE
Q9ESK4	CVESLPHDMQRNVSVLRELDNKYQETLKEIDDVYEKYKKED
Q8WU03	KEIPFYFHVADNNEKSLQALNNLGFKICPCGWHQWKCTPKK
P18124	ELIYKRGYGKINKKRIALTDNALIARSLGKYGIICMEDLIH
Q2KHY8	SRLGSLAQPRHNVEVCSVPANSYIYQQGCERNLSNWLTNNL
Q15773	MLGMSGGFMDMFGMMNDMIGNMEHMTAGGNCQTFSSSTVIS
Q8BG31	TKTPSRPVGISEPKTSNLCGNRAYGKSLIPPVARISVKAPA
Q9Y624	RNGYGTPMTSNAVRMEAVERNVGVIVAAVLVTLILLGILVF

P51159	FRDAMGFLLLFDLTNEQSFLNVRNWISQLQMHAYCENPDIV
Q5HZI9	QLYGIKTRDAVLQLRKDGFRNLYRGILPPLMQKTTTLALMF
Q9CX92	LVIVLLPGAIFHLYAACKSINQDCILQKPVYTVIYVLSVLL
Q2PT27	LSGTEFIGCQFYDRESQKGCNFSRANLKDAIFKSCDLSMAD
Q9CRA8	SITVVLQVVSDAGSLLACCLNAACMALVDAGVPMRALFCGV
Q6ZVX7	LELPQRLTWKLLLLRRPLYRNLLRSPNPEGINIYEPAPPTG
A5PJA8	PMVMMMVLPLLIFVLLPKVVNTSDPDMRREMEQSMNMLNSN
Q91ZR1	AWLTDARTLASPNIVVILCGNKKDLDPEREVTFLEASRFAQ
Q95106	RAQGSLAYPGVRTHGTLESMNGPKVGSRGLTSSSSLADTFE
Q80XM9	RRTQNADGFSTHVCLVLLVANILRILFWFGRHFESPLLWQS
O00212	EMARSVGAVAYLECSARLHDNVHAVFQEAAEVALSSRGRNF
Q9BXY4	SGFYLHLGKCLDNCPEGLEANNHTMECVSIVHCEVSEWNPW
Q924N9	CWKSNKWIQVGVVSKGIDCSNNLPSIFSRVQSSLAWIHQHI
B1ATL7	PPALIVGGTKVNNGGTERGSNNARLHVALPQGKGFFPPRGP
P07203	LLIENVASLUGTTVRDYTQMNELQRRLGPRGLVVLGFPCNQ
O75496	ALYEALKENEKLHKEIEQKDNEIARLKKENKELAEVAEHVQ
O15247	RLFLDGDQLTLADCSLLPKLNIIKVAAKKYRDFDIPAEFSG
Q60961	VLVFFVVFIIFKAYLINCVWNCYKYINNRNVPEIAVYPAFE
Q61759	VGGFNCEKNSQPWHVAVFRYNKYICGGVLLNPNWVLTAAHC
P51151	YRGSDCCLLTFSVDDSQSFQNLSNWKKEFIYYADVKEPESF
Q9NVT9	LASEIYDILQSSNMADGDSFNEMNSRRRKAQFFLGTTNKRA
O89094	KGEDEKMVRLEDLFEVLNNKNCKALRGKPKVYIIQACRGEH
Q9QWV4	FSEPLGRDLLSISDGRGRTHNRRERDDGEDSLTHADVNPFQ
Q0VCY7	GGGGGGAPRGRYGPPSRRSENRVVVSGLPPSGSWQDLKDHM
P59095	SDFLYQTGDRITWDKSLQVYNMVHRIDSDTFICHTITQSFA
Q9CQS9	AVLKEKHAIRQRLLRPMCQENLPLEAVYHRYVVHMLDLAVT
Q9WUZ7	GDEDNRKWMRENVPGEKKPQNGIPLPPQIFNEEQYCGDFDS
Q93015	LTLEPVHRRPELLDACADLINDQWPRSRTSRLHSLGQSSDA
Q5E943	GRHPHSLMGKNFRSYLLDLRNTSTPFKGVRKALIDTLLDGY
Q9CY28	NNLKRTFLLVDSVVGITKLDNIAIEMCEEFALPYVMILTKI
Q3MHR2	GQSLELDGIIYLRATPEKCLNRIYLRGRNEEQGIPLEYLEK
Q9NNZ3	MLAGMGLHYIAFRKVKQMHLNFMDEKDRIITAFYNEARARA
P42125	VNSLSLECLTEFTISLEKLENDKSIRGVILTSECPGIFSAG
Q8N7C4	AQIFRGLVIYIVWIFFYETANVVIQILTNNDFDIKEVRIMR
Q9UIJ7	PVLEYYQKKGVLETFSGTETNKIWPYVYAFLQTKVPQRSQK
Q8N1A0	LPSAIINEISFTTKVPQKYENENVETVTKQAILNGSIVKES
P15173	RLKKVNEAFEALKRSTLLNPNQRLPKVEILRSAIQYIERLQ
Q8BGU2	SEMSNRTMTIYFDQVLVNIGNHFDLASSIFVAPRKGIYSFS
A6H6X4	TTFLEITDNHDRSQGLRIFCNAPDFISKIKSRIVLVSKSRL
Q91VH6	MSIIEQLDPVSFSNYLKKYHNTICGRHPIGVLLNAITELQK
P08884	DSGGPLVCDNKAYGLLAYAKNRTISSGVFTKIVHFLPWISR

Q9WUK2	QRPRLQLKPRTVATPLNQVANPNSAIFGGARPREEVVQKEQ
Q9Y508	STDTKSVVCPICASMPWGDPNYRSANFREHIQRRHRFSYDT
P14148	CMEDLIHEIYTVGKRFKEANNFLWPFKLSSPRGGMKKKTTH
O55125	DQAVHLWRFSGGYPALMDCMNKLKNNKEYLEFRKERSKMLL
P55064	IGLSVTLGHLVGIYFTGCSMNPARSFGPAVVMNRFSPAHWV
Q9JKY0	KEPSARLLKHVVRCYLRLSDNPRAREALRQCLPDQLKDTTF
O43704	SPFMRKGTAGDWKNYFTVAQNEKFDAIYETEMSKTALQFRT
Q96CX3	TKAILVQHLRIHTGEKPYKCNECGKAFCQSPSLIKHQRIHT
P41220	EKEAPKEINIDFQTKTLIAQNIQEATSGCFTTAQKRVYSLM
Q15526	LESRVLAEPVPLPADPMELKNLEYRPVKVRGCFDHSKELYM
P56402	FGLGIGTLVQALGHVSGAHINPAVTVACLVGCHVSFLRAAF
Q9BRV3	TATLLGVLLLGYGYFWLLVPNPEARLQQLGLFCSVFTISMY
P31098	SERCMSTKKTSRLTDHSKETNRCELSKELMPKAKDKNKHSN
Q96EF6	RDRSAEGRALYAVAQRCLPSNEDKEEFPLCALARYCLRAPF
Q96GV9	SSRLYKTRSRYYQPYEIPAVNGRRRRRMPSSGDKCTKSLPY
Q3T0C6	PRIECTGKSESTAFLSTYPSNGTIDLKYFPYYGKKLHGNYL
O75937	TDEEIKKRFRQLSILVHPDKNQDDADRAQKAFEAVDKAYKL
Q8IVU9	IGPTHSQKSEDWNIFDVKQANAIIDYLKISLFQHYKLYEFM
P28033	DKAKMRNLETQHKVLELTAENERLQKKVEQLSRELSTLRNL
Q3SZW3	LIKVFVLTSNFQAIRVTLNINRKLAFLAILSGLLVESTMVY
P41227	KEKGRHVVLGAIENKVESKGNSPPSSGEACREEKGLAAEDS
Q8R4X1	LASGYSVWLPRCYFPKFVKGNRFYFSCLVTITTIISTFLTF
A2AFE9	MALMIGGPRVGSRVLERSGNNSKPYIPVPRSQGFFPPRGSQ
P01906	VFSKFPVTLGQPNTLICLVDNIFPPVVNITWLSNGHSVTEG
A9UHW6	EEVDCLVLQLHRVGEQLEKMNGQRMDELFVLIRDGFLLPTG
O43240	SSITILSPKECEVFYPGVVTNNMICAGLDRGQDPCQSDSGG
Q9HBH0	SACEQIRAALYLECSAKFRENVEDVFREAAKVALSALKKAQ
Q04917	THPIRLGLALNFSVFYYEIQNAPEQACLLAKQAFDDAIAEL
Q9ER80	SPLPKSSSPSKSCPPPPQTRNTDFGNKTFQDFGNRTFQGCR
O15537	SVQYRTDERLNWIYYKDQTGNNRVFYGNSDRTSTVQNLLRP
Q9NQT5	GMNGRIWVKAKTIQQTLILANILEACEHMTSDQRKQIFSRL
Q9BVM2	KDVYSVSVDQKERCIIVRTTNKKYYKKFSIPDLDRHQLPLD
Q9CWJ3	AYLVRIEDLGVVVDCLPVLTNSLQEEKQYISLGCCVDLLPL
Q5E9E3	SGPMGPAGLPGLKGTKGSPGNIKDQPRPAFSAVGPNSVSRD
P33792	SESGTVDVSKVSEKNKYIIKNNTNKVMDVWADYCGSYNNNK
Q6UYE1	QTLQLLQQEWGWGDGPVAPGNPRDPDHVSTAPARRSGPPRA
Q99KJ0	KCLSPTEQKKQVDVNIKLWKNGFTVNDDFRSYSDGASQQFL
P19388	LVDMAPKYILEQFLQQELLINITEHELVPEHVVMTKEEVTE
A5D7R8	CRLCYFKKASGQPAVLDSEENKENCPMTLGKPAEAPGVRKL
Q17QQ2	PRANIGKFDRIWDRGALVAVNPSDRKRYSDVMLSLTRPGFR
A6QNL6	KETDLILTFNISMHRSWWMENGPGCTVTSVTPAPDWAPEDH

P20774	YEDKYLDGKNIKEKETVIIPNEKSLQLQKDEAITPLPPKKE
P0CG22	FRPLPGFSPYNVSKTALLGLNKTLAIELAPRNIRVNCLAPG
P18669	WRRSYDVPPPPMEPDHPFYSNISKDRRYADLTEDQLPSCES
Q86W47	MNWQQYWKDEIGSQPFTCYFNQHQRPDDVLLHRTHDEIVLL
Q3TR08	LLLVSCDLGFVRADRPPSPVNVTVTHLRANSATVSWDVPEG
P61296	PGAGPPGLGGPRPVKRRGTANRKERRRTQSINSAFAELREC
A6NCD4	KSSEQRPVNRSYPKCFSLGVNLQNVAESEEEEFMKEFILTD
P40630	QEAKDDSAQGKLKLVNEAWKNLSPEEKQAYIQLAKDDRIRY
O54831	LAKNIETKLAELFEYTQSILNSIYGTTTTGNVEYTVFSGLE
Q5NE16	MNAFGEMTSEEFRQVVNGFQNQKHRKGKVLQEPLLHDIRKS
Q3T062	QRIATRGVVQLFNAVQKHQKNVDEKVKEAGGSIRKRAKLIS
Q5E9A1	SKLGLRQVTGVTRVTIRKSKNILFVITKPDVYKSPASDTYI
Q8K3A2	HIHLASGLPGDPGVISGIRPNCEVAVFIDGPLALTDGIPFF
Q9CX99	HPWYSGRISRQLAEETLMKRNHLGAFLIRESESSPGEFSVS
Q05685	YSRGSGRCIQMWFDSTQGNPNEDVVKFYASFMTSGTVPHAA
Q2TBU2	TIDEELERDKRVTWIVEFFANWSSDCQSFAPIYADLSLKYN
Q15527	RASPAFDYAEFEPHIVPSTKNPHQLFCKLTLRHINKCPEHV
P30681	AFFVQTCREEHKKKHPDSSVNFAEFSKKCSERWKTMSAKEK
P12319	NPPWNRIFKGENVTLTCNGNNFFEVSSTKWFHNGSLSEETN
Q9BYD2	YWCEVTVNGLDTVRVPMSVVNFEKPKTKRYKYWLAQQAAKA
Q8N6S5	EFSAGAWSEPRKRSVLPPDGNGSPVLPDKRNGIFPAAAGSR
P60879	ISGGFIRRVTNDARENEMDENLEQVSGIIGNLRHMALDMGN
Q9JHW2	KSDNLTRACSLVREAAKQGANIVSLPECFNSPYGTTYFPDY
Q96HV5	LFCGAYLYKQGFAIPGSSFLNVLAGALFGPWLGLLLCCVLT
P20718	GILVRKDFVLTAAHCQGSSINVTLGAHNIKEQERTQQFIPV
Q9D270	FVVDPHGWCCMGLIVFVWLYNIVIIPKIVLFPHYEEGHIPG
Q9Z2X2	AGRDEIVKALLVKGAHVNAVNQNGCTPLHYAASKNRHEIAV
Q9H2P9	SLENLIKGRKIYEPPRYMSVNQAAQQLLEIVQNQRIRGEEP
Q9JL59	TIELVTSEKTSIIFNHTPPGNYSKDSWQLHIQGVQAQLVIT
Q96L15	QNGIAIMVYTNSSNTLYWELNQAVRTGGGSRELYMRHFPFK
P51945	GRDLTFWQELVSKCLTEYSSNKCSKPNGQKLKWIVSGRTAR
P57776	AERRFYEQMNGPVTSGSRQENGASVILRDIARARENIQKSL
P54116	VLTKDSVTISVDGVVYYRVQNATLAVANITNADSATRLLAQ
Q99P30	VPYVFDNDALVTPVVGFLDHNFQAQPNADEVKEVFFVPLDY
Q8CDN6	PQAVFLEVDVHQCQGTAATNNISATPTFLFFRNKVRIDQYQ
P79125	KSGLERGLDTFDSTIEIIFQNLKTELESRCSDEVVEQQETE
Q9WUI0	RERLAALTLLPESRIQVWFQNRRAKSRRQSGKSFQPLSSRR
Q3SZZ2	DRKKARMSELEQQVVDLEEENQKLLLENQLLREKTHGLVVE
A5D7U1	PNSTDITTVKINIYTVNSLRNAELDSITLPEVGTVTVKHVS
Q9EQ48	LSIFYTSISSQILIAAIPTQNITFVNLIYITNSCSFLPLSS
P13805	EKFDLMAKLKQQKYEINVLYNRISHAQKFRKGAGKGRVGGR

Q3TQI7	KKDSETSFVPTNMAVNYVQHNRFYHEELNAPIRRNKEEPKA
Q6UXN8	CALQMKYCQAFMQNSLSSAHNSSPCPNNWIQNRESCYYVSE
Q14749	NIYYKSDLTKDVTTSVLIVNNKAHMVTLDYTVQVPGAGQDG
Q99KL7	SVVKTVSEESETQPLVALVGNKIDLEHMRTVKADKHLRFCQ
Q8BQM7	EVNRLHQQLLDCLSDSFQVTNKLTGVLNTHLGCRLAFIEMK
Q8VHX2	DELCFLEQRPQSPTLEFLFRNSQRTVGQLMELCRLYHRADV
A8MUZ8	LLTHSGKKPYVSKQCGKSLRNLLSTEPHKQIHTKGKSYQCN
Q60819	TCPPPVSIEHADIRVKNYSVNSRERYVCNSGFKRKAGTSTL
Q8VDP6	SFYLLSGLLDAFDGHAARALNQGTRFGAMLDMLTDRCATMC
O69395	AALEKSSGGRLGVALIDTADNTQVLYRGDERFPMCSTSKVM
O43396	PFINKAGCECLNESDEHGFDNCLRKDTTFLESDCDEQLLIT
Q921J4	PASPPKGYFLTKIFHPNVGPNGEICVNVLKRDWTAELGIRH
Q8BTQ0	IRCSAQATVLHLKKFIAKKLNLSSFNELDILCNEEILGKDH
P52743	GKSSLFYHQAIHGVGKLCKCNDCHKVFSNATTIANHWRIHN
Q6UWN0	TSASCSCPTCVGEHMKDCLPNFVTTNSCPLAASTCYSSTLK
Q0VCT9	PQHAFNALMGEHIHYGASNMNASSGIRHAMGPGTVNGGHPP
P07146	NSWTLDNDIMLIKLASPVTLNARVASVPLPSSCAPAGTQCL
O88587	HLGWGLVAIGWFEFVQQPVHNLLMGGTKEQRILRHVQQHAK
Q8NA92	SQRRTRSTQKPVSPPPPLQKNTPLPQSPAIPVSGPVRLVVL
A6NC86	CQSDGCNSAFLSVPLTNLTENGLMCPACTASFRDKCMGPMT
P28676	DRDHTGKMGFNAFKELWAALNAWKENFMTVDQDGSGTVEHH
Q8BHI7	ITVLHVYHHATMLNIWWFVMNWVPCGHSYFGATLNSFIHVL
B6A8R8	QLTATSPKTPGTMTEGYTVDNLIRVGVAAAILLIVGGFLVE
P62823	EESFNAVQDWSTQIKTYSWDNAQVILAGNKCDMEDERVVST
A2VE67	TEKTVFTVFMIAVSGICILLNVTELCYLLIRFCSGKSKKPV
Q9Z2X2	HYAASKNRHEIAVMLLEGGANPDAKDHYDATAMHRAAAKGN
Q6IMH0	YAVRYLKPEVTQNWRYCLNQNPSLDRYGQKPLPFDSLNAFR
Q9JM71	TPTKYQIPNDLQCVFIKLLPNENCAKAYVHKVTDVMLCVGE
Q2HJF3	AASCMKCPLDRAYLIKLSGLNKKMYQSCLKSFECLLGLNSN
Q96A61	LQLANMVQIIRQMCPTPYRGNRSNDQGMCFKHQEALKLFCE
Q32LD7	SIQVSIEKNLEISKARDIVINRLLQYGSTEISTQSLHISQY
O35387	PRGGMRFHGNFGFDDLVRDFNSIFSEMGAWTLPSHSPELPG
Q8NC96	KLDLGFKEGQTIKLCIGNITNKKGGASKPRTARGGGLSLLP
Q5E9Q4	NGRRAFIGIGFGDRGDAFDFNVALQDHFKWVKQQCEFAKQA
Q9BRK0	VLIFGTLYPAYSSYKAVKTKNVKEYVKWMMYWIVFAFFTTA
P51572	IDAVREIRKYDDVTEKVNLQNNPGAMEHFHMKLFRAQRNLY
P42125	LVETEGPAGVAVMKLRNPPVNSLSLECLTEFTISLEKLEND
Q71SV0	ILLFISIILAFKGYLISCVWNCYRYINGRNSSDVLVYVTSN
P0C6B7	VLQPGYLEVDYGSDAVTMECNFSTVGCPPVPPKSLWFRCGT
Q8BL95	VMIVYPMGLPPYDPIRMEFENKEDLSGTQAALEVIQESEAQ
P52803	PHYEDSVPEDKTERYVLYMVNFDGYSACDHTSKGFKRWECN

Q8BHK2	QDVSITVLNVTLNDSGLYTCNVSREFEFEAHRPFVKTTRLI
Q04743	DPIRPAALSYANSSPINPFLNGFHSAAAAAAGRGVYSNPDL
P53519	KSKLIIILLTLVPFSSFSTGNNFEINKTRVIYSDSTPSVQIS
P55258	TITTAYYRGAMGIMLVYDITNEKSFDNIRNWIRNIEEHASA
Q9D287	QLEHQAVRIENLELMSQHGCNAWKVYNENLVHMIEHAQKEL
P07766	THWRVLGLCLLSVGVWGQDGNEEMGGITQTPYKVSISGTTV
Q7M729	LPMYLSLEVSVGKATTIYAINGSSILLPCTFSSCYGFENLY
Q8CCI5	AFKCSICDVRKGTSTRKPRINSQLVAQQVAQQYATPPPPKK
P06717	RSGGLMPRGHNEYFDRGTQMNINLYDHARGTQTGFVRYDDG
Q96H79	ECQACDQLHFCRRHMLGKCPNRDCWSTCTLSHDIHTPVNMQ
Q077R2	YRVHAGSVSANKFKELIRQSNVLKSIGIKAHHRIICLFYYA
Q3T075	RQNLLDDLVTRERLLLASFKNEGAEPDLIRSSLMTGGAKRG
Q64152	RTATADDKKLQFSLKKLGVNNISGIEEVNMFTNQGTVIHFN
Q2TBX6	SASAMLQPLLDNQVGFKNMQNVEHVPLSLDRAMRLVKDVFI
Q8R2N0	RTGGFAARGEGISQVRFGNQNGKRRTWRPNPQQAFRGSVRK
Q12981	MLSNQASWRKANLTCKIAIDNLEKAELLQGGDLLRQRKTTK
P41439	MEPTCKRHFIQDSCLYECSPNLGPWIRQVNQSWRKERILNV
Q3SZX4	QLLLDALDKIKTKGKEAPFNNFNPSCLFPACRDYWTYHGSF
Q9DCX7	ERVPDSDQTSGGQSFVFTGINQPITFHGATVVQYIPPPYGS
Q9BT67	ESAAYFDYKDESGFPKPPSYNVATTLPSYDEAERTKAEATI
Q8VBV8	NEEASQYVESMFRAFDKNGDNTIDFLEYVAALNLVLRGSLE
O75712	LVYVVAAERVWGDEQKDFDCNTKQPGCTNVCYDNYFPISNI
B7ZW38	PPPPPIALAVVPSKRQRISGNTSRRGKSGFNSKSGKRGSSK
P15946	PDYNMSLLIIHNPEPEDDESNDLMLLRLSEPADITDAVKPI
Q96PU9	FRGAPMLLAENCSPGPRYNVNPKILRTGKDLGPAYSILGRY
P0DMN0	WWELSRTHPVLYLFYEDMKENPKREIQKILEFVGRSLPEET
Q86W74	CGHVDTIQFLVSNGLKIDICNHQGATPLVLAKRRGVNKDVI
Q96FA7	HWKQVLVYKVKEIRVSEYSLNSPSPLQSPRGLCVDPTRVAK
Q8IXA5	YFTSGFNAAALDYEADGSTNNGIFQINSRRWCSNLTPNVPN
Q86V81	GRGGGAQAAARVNRGGGPIRNRPAIARGAAGGGGRNRPAPY
Q8N699	LFLVDIMANNTTSLGSPWPENFWEDLIMSFTVSMAIGLVLG
Q64ET8	TAFSHSSEKHIQRQGSEPNPNKENSEETKLKAGNSTAGSEP
Q01362	FWGAIFFSISGMLSIISERRNATYLVRGSLGANTASSIAGG
Q9QXV3	AITQADKPNNKRSRRQRNNENRENASNNHDHDDITSGTPKE
Q96BD6	PPVSYDVQLLHSWNNNDRSLNVFVKEDDKLIFHRHPVAQST
Q3V3F6	RSGYKKSSIEVSDDTDQEDSNDCMTRLCVRCAECLDSDYEE
Q75WM6	KAARKAREVWRRNARAKAKANARARRTRRARPRAKEPPCAR
Q9JIN6	DQHQLLTNPKCSYIPPCKRENQKNSESVMNWQQYWKDEIGS
Q00322	AGKRGPDRGSPEYRQRRERNNIAVRKSRDKAKRRNQEMQQK
Q8C8M1	SPCYSNQSDEGSDTEMASSSNRTPVFSFLDLTYWKRQKICC
Q9QXT1	AAEALLLSNGRDGSYLLRDSNEQTGLYSLSVRAKDSVKHFH

Q86TL2	RIWFLDTSKQAIGMLFIHFANVYLADLTEEDPCSLYLINFL
Q9UJQ1	YDSSEKTHFKDAVSAGKHTANSHHLSALVTPAGKSYECQAQ
Q9D710	DFDWREVEILMFLSAIVMMKNRRSITVEQHVGNIFMFSKVA
Q9H0R3	TAVHDASEEYKHRMHNLCCDNCHSHVALALNLMRYNNSTNW
Q2TBU3	HSSARDGRRDRYSSDTTPLLNGSSQDRMFETMAIEIEQLLA
P22061	KTDKVFEVMLATDRSHYAKCNPYMDSPQSIGFQATISAPHM
P30084	EKRGKNNTVGLIQLNRPKALNALCDGLIDELNQALKTFEED
P53994	TAKEIYEKIQEGVFDINNEANGIKIGPQHAATNASHGSNQG
Q80XC2	LPQVYNVRTVSLPLPDLGANNLETNMGSDASPFRSGTPMKE
Q9D5W8	FRILLDYVAHEKSETEFMLQNMKIVVASHNIPTRLFMQWHI
Q9BYE0	NRDGPKMLKPLVEKRRRDRINRSLEELRLLLLERTRDQNLR
P47754	DQVLITEHGDLGNGKFLDPKNRICFKFDHLRKEATDPRPYE
Q9CQC5	NYELLPGNQEKAHSGQFPGHNDFFRANSTSDSMFTETPSPV
P55253	TVFAYHVNDPERYGVVEFDNNGTAISLEEKPLEPKSNYAVT
P50283	QEPTVDRTFSGRINFSGSQKNLTITISSLQLADTGDYTCEA
Q08DD7	TSCVRRRYREFVWLRKQLQRNAGLVPVPELPGKSTFFGSSD
Q8BGN8	WHSSGQRYLSDPMEKHSSSYNQGRYNQESYGSSGGYSQQAN
Q86U90	HAFMQDLAQMFEGPLALTSANLSSQASSLNVEEFQDLWPQL
Q6UWN5	PVTLVRKGCWTGPPAGQTQSNADALPPDYSVVRGCTTDKCN
Q9JM90	PVTLPNLFSVIDYFVKETRGNLRPFIHSADDNFGQDPNIED
Q8N6M0	KEMEQKHREELEQLKLTTKENKIDSVAVNISNLVLENQPPR
O35565	GDVRWRRLFSFTKYFLTIEKNGKVSGTKNEDCPYSVLEITS
Q9DC07	CLDKYWHKGCFHCEVCKMALNMNNYKGYEKKPYCNAHYPKQ
P38572	EVVFPWPPLCCRRTGNFIPVNEEGCRLGHLDYLFTKGCFEH
P26998	AGKSHGDLGGSYKVILYELENFQGKRCELSAECPSLTDSLL
Q9JM14	PGFFLNLEPIPGALDALREMNDMKDTEVFICTTPLLKYDHC
Q8TBE3	DLGQREEDLQGLPLVEMPRKNSRDGAELDPEANQDAPDAGA
Q6ICI0	TPTFSFGILTYCSWPQGNSWNQSCVTFSSLEDIPDFAWKVS
P46425	GRSLGLYGKNQREAAQVDMVNDGVEDLRGKYGTMIYRNYEN
Q9CPZ1	NKIAQSKPRSNGYDILTILPNENINRDPGNPQDEEFLDCHT
G3X9C2	NIYQPAPPTGPTRKPLKELGNFRGWYITTQNLQGPLSWTVK
Q5EA33	EKVNDEKPDPENSLDFSEHFNQLELLETHGHLIPTGTQSLW
Q9UGB7	EAVDLLDGLVDESDPDVDFPNSFHAFQTAEGIRKAHPDKDW
Q7YRA3	IQARMGVFAQADGSAYIEQGNTKALAVVYGPHEIRGSRARA
P20334	VGAVQNSCDNCQPGTFCRKYNPVCKSCPPSTFSSIGGQPNC
Q9BXN2	AVVLGTMAIWRSNSGSNTLENGYFLSRNKENHSQPTQSSLE
Q32LD7	YWRITSIKEKNSLQMQKPISNAVLNEYLEQKLVELYKQYFM
Q06599	LRWWDSYANALMANGVKLEDNQLVVPADGLYLIYSQVLFRG
Q99N05	GAIQVMIAFINFSLGIIIILNRVSERFMSVLLLAPFWGSIM
Q15744	DKAKRRILETQQKVLEYMAENERLRSRVEQLTQELDTLRNL
P00484	SVMERYKSDTKLFPQGVTPENHLNISALPWVNFDSFNLNVA

P0DML3	LKQTYSKFDTNSHNHDALLKNYGLLYCFRKDMDKVETFLRM
Q9HB20	HSSPSAENMNEASSLLSATCNTFITTLEECVKIANAKFKPE
P00551	CIDVGRVGIADRYQDLAILWNCLGEFSPSLQKRLFQKYGID
O55126	EVLPKIHEGKQYPCTLVGTWNTWYGEQDQAVHLWRYEGGYP
Q96L46	RQQFTQLAGPDMEVGATDLMNILNKVLSKHKDLKTDGFSLD
Q2TA29	IGDSGVGKSNLLSRFTRNEFNLESKSTIGVEFATRSIQVDG
Q8R2Y9	EPNPEYNTQQAPNKSVQNNDNSPTAPQATTGPPAASPASEN
O14798	NSPEMCRKCSRCPSGEVQVSNCTSWDDIQCVEEFGANATVE
Q8N567	ALKKDSRREGRRLKRQAAKKNAMVCFHCRKPGHGIADCPAA
Q28132	KSSKSPEPRQFTPEKFFGIFNKSIDAFKDLEIVASKMSECV
Q92930	EKLAIDYGIKFLETSAKSSANVEEAFFTLARDIMTKLNRKM
Q9NP50	SPCYSNQSDDGSDTEMASGSNRTPVFSFLDLTYWKRQKICC
P97361	GQLLPLAQGLPLAVSPALPSNPTDLLAGKFTDALSGGLLSG
O08602	DPLWYEAKCLVDEILILHLSNINKTMTSGDPGETANATEVG
Q9UPY8	KNMQTSGRLSNVAPPCILRKNPPSARNGGHETDAQILELNQ
Q8N8J0	YCSSDQVEIFSSLLQRSMSLNIGGAKGSMNRHVAAIGPRFK
Q5E9I1	WQELVSKCLAEYSSNKCAKPNVQKLKWIVSGRTARQLRHSY
Q9GZN7	GSQRLEVSHVHKVECVIPWLNDALVYFTVSLQLCQQLKDKI
Q80ZA7	NRDDTLALSMYQGPPSADQGNNMADAPRFGFWTSVSQCLQY
P59024	HYEGYLEKDGSLFHSTHKHNNGQPVWFTLGILEVLKGWDQG
Q32L00	GAWAGPAKSQATAQPAPTAENNLQQRPGKAWMNKEQHLSDR
Q96CG8	LPIEAIIYLDQGSPEMNSTINIHRTSSVEGLCEGIGAGLVD
Q28110	LNKITFYQDRKSKIFSYQRTNFSIPRANLSHSGQYHCTAFI
Q32KV0	SLRGIVKHLEGMSDQAIMELNLPTGIPIVYELDQALKPTKP
Q8IXM7	FGMSSCPQVPMEERISNLRLNPTLASCQYYFEKIHPPGERR
Q969T7	NKNSSACENSGYFQQLEGKTNVILLGDSIGDLTMADGVPGV
Q9JLJ4	PFGCLIFQSSYMMTLVILFLNFYIQTYRKKPVKKELQEKEV
Q3SYT7	PNLSKCGEELGRLKLVLLELNFLPTTGTKLTKQQLILARDI
Q68D91	YQFDRVAVHHAEAEALARGDNFETVTWLSDSEVVRTPSPGW
Q6ZN79	DASTSMTMENSLILEDPFECNDSGEDCTHSSTITQRLLTHS
O18870	LSDLLSPTSEVANIEQNAQENENESQISTDESENSSRSPLN
P01921	SLRRLEQPNVAISLSRTEALNHHNTLVCSVTDFYPAKIKVR
Q8BX32	YLLYCQNPRHRGRVYVGFTVNPARRVRQHNAGRKKGGAWRT
P00586	GHPVTSEPSRPEPAIFKATLNRSLLKTYEQVLENLESKRFQ
Q6PCP5	DGVPNASVIMQVPERIVVTGNNEDISFSRPADLDLIQSTPF
Q9BR10	QARGCPGGTSWETLRKEYSRNCHKFPHVRQLESLGWDNGYS
Q8CG50	WDTAGQERFRTITQSYYRSANGAILAYDISKRSTFLSVPHW
P35270	VSKGFVDLSDSTQVNNYWALNLTSMLCLTSSVLKAFPDSPG
P20490	IVSSIAAGTGIAMLILNLTNNFAYMNNCKNVTEDDGCFVAS
Q9QY80	SFYTFSLLDHLPHFIKWARYNLFIILYPVGVAGELLTIYAA
P07361	MILVMVSLLPLLEAQNPEHVNITIGDPITNETLSWLSDKWF

Q9BXY4	YPDINKCTKCKADCDTCFNKNFCTKCKSGFYLHLGKCLDNC
Q9Z2X2	KMVHILLFYKASTNIQDTEGNTPLHLACDEERVEEAKFLVT
Q5QGZ9	LKIEMKKMNKLQNISEELQRNISLQLMSNMNISNKIRNLST
Q80X85	LADRRRRFLAMKWMITECRENKPRRTLMPEKLSHELLEAFH
Q96E22	MAVGISYISVYDHQGIFKRNNSRLMDEILKQQQELLGLDCS
O43513	GMRVKTEPMDADDSNNCTGQNEHQRENSGHRRDQIIEKDAA
Q99LZ3	SPEEFVFAKEYMDHTETHFKNVALKHMPPNLQKVDLLRAVP
Q8CB49	GDGLRIFWGSTEGWSLLSRWNPWSTEVPYATFTEHPMKQTS
Q9UKD2	NSKLKDIRNAWKHSRMFFGKNKVMMVALGRSPSDEYKDNLH
Q9NVX0	AVLKEKRSLRQRLLKPMCQENLPIEAVYHRYMVHLLELAVT
A7MAZ5	VSLAALKKALAAAGYDVEKNNSRIKLGLKSLVSKGTLVQTK
O95997	KSSVPASDDAYPEIEKFFPFNPLDFESFDLPEEHQIAHLPL
Q6ZMS7	KWAVLGTLLQEYGLLQRRLENVENLLRNRNFWILRLPPGSK
O95873	KPMRPDPPYPEPRRVDSSSENSGSDWDSAPETMEDVGHPKT
Q9Z2M7	KLQEQLGNDVVEKYDYVFPENGLVAYKDGKLLCKQNIQGHL
Q8BFR6	CLRWSIGKVVDFAASLANLRNENNKLTAKKLRLCHVPSGEA
Q32LN5	DEYGVSSIIPNFQISQDSVGNSNRSETSASDNIETYQENTG
A6NJY1	HFIEWTKWSQEKMKVQKIITNVWDIFQPLLFGLVGAEVSVS
Q16763	RHVLLTIKCLLIHPNPESALNEEAGRLLLENYEEYAARARL
P10103	KGEHPGLSIGDVAKKLGEMWNNTAADDKQPYEKKAAKLKEK
Q3LI81	CHSLRSFHNAPPLSAITHGTNPITFEDRLCLPSSFHSRTCF
Q9JJ57	VLKEDTPRQHVDVFFQKMDKNKDGIVTLDEFLESCQEDDNI
P60410	ACCTSSPCQQACCVPVCCKSNCCKPVCCVSICSGASSPCCQ
O15551	AGVLFLLAALLTLVPVSWSANTIIRDFYNPVVPEAQKREMG
Q47066	RVEIKKSDLVNYNPIAEKHVNGTMTLAELGAAALQYSDNTA
Q9NTI7	RGRNRQPLVLGDNVFADLVGNWLDLPELEKGGEKGETGGAR
P61087	TPYEGGRYQLEIKIPETYPFNPPKVRFITKIWHPNISSVTG
Q6UWM5	DFDSLSCSRVCGHYTQLVWANSFYVGCAVAMCPNLGGASTA
Q0P5N1	KKQCDVLVEEFEEVIEDWYRNHQEEDLTQFLCANHVLKGKD
P16035	VIRAKAVSEKEVDSGNDIYGNPIKRIQYEIKQIKMFKGPEK
P0AC11	LDSYQPATQAYALSRGVAYLNDIRGFPDAAFYPQLAKSSAK
Q91UZ4	FVADVEPIFDRLLFFWSDRRNPHEVQPSYATRYAMTVWYFD
Q2KI06	LLNFIRKNKNKFDCIIISDSNSVFIDWVLEATNFHDVFDKV
Q5TGJ6	KFGKPNKRRGFSAGLWEIENNPTVQASDCPLASEKGSGDGP
Q3SZ16	DSPVAVTIFFGANDSALKDENPKQHVPLEEFVANLRSMVRY
A6NDR6	GCREDFEDYPASCPSLPDQNNIWIRDHEDSGSVHLGTPGPS
Q9CQG3	PVLNVTRYYNANGDVVEEEENSCTYYECHYPPCTVIEKQLR
P82918	KVAGNPCPICRDQKLHVDFRNVKLLKQFVCAHTGIIFHAPY
Q7YS81	CSSHSASCSPQWGSALEFGPNPGDHLLPADPTDAHNLHSLT
P11900	TSYSGFHNWDDLSHSNYTSANKASYLSYGSGVSAGSTLVMN
Q58DM4	QEQTGGGPAGLAEEEGNSKKNPRRAAPGNGVDSAGLTWGRI

Q58D78	LYLLHTLWYGFGMPRLVQCANVAPCPNTVDCYIARPTEKKL
Q0VCP1	QNFGLQHLSSGHFLRENIKANTEVGDMAKQYIEKGLLVPDH
Q2KIG1	FSKLQRNILASNPRVTRFHINWEDNTEKLEDAESSNPNLPS
Q9NYZ1	IDEDGKSHWVFESRKESSQENKTVSEAESRIFWLGLIACPV
Q8R0J7	QTVQLNKEMTLASNRSLAEGNLLYQPQLDAQKARLTQKYQE
P27707	IIYLQATPETCLHRIYLRGRNEEQGIPLEYLEKLHYKHESW
Q08E08	LPCTFSSCFGFENLHFWWSYNSSDTYKILIDGTVKNEKSDP
P49070	GSGSEEENQTKSKPQDSDKLNSLSIPSVSKRVVLGDSVDGG
Q47036	SALLFYMPDQRHHMLFATLWNTGMRIGEARIVTPESFDLDG
P00405	AYPFQLGLQDATSPIMEELMNFHDHTLMIVFLISSLVLYII
Q99619	LEELLSAPPPDLGAQRRHGWNPKDCSENIEVKEGGLYFERR
Q9NPL8	RTAVFVTIFNTVNTSLNVYRNKDALSHFVIAGAVTGSLFRI
P25322	RACQEQIEALLESSLRQAQQNVDPKATEEEGEVEEEAGLAC
Q9ULY5	QWVDGTPLTKSLSFWDVGEPNNIATLEDCATMRDSSNPRQN
Q4U5R3	EKLNLVTTWLQLQIPRIEDGNNFGVAVQEKVFELMTALHTK
Q9R1P4	ARSQSARTYLERHMSEFMECNLDELVKHGLRALRETLPAEQ
Q2HJF5	VGAILSDYQRVRVENVCKRLNLQPLAYLWQRNQEDLLQEMI
Q8BHE8	LMCFWYLTSANIPSESLSGANVFQVKLGDQSVETKQLLSAS
Q15493	EQIPDGMCIDAEGKLWVACYNGGRVIRLDPVTGKRLQTVKL
Q12918	AILFWIGLNFSLSEKNWKWINGSFLNSNDLEIRGDAKENSC
P04116	GCGHEALTGTEKLIETYFSKNYQDYEYLINVIHAFQYVIYG
A5D7U1	GSWENPSDHCEGRAEYYLKGNFSTLFKAKWMSPNSTDITTV
Q9DB26	LSSGDKIRFFFEKGVFDEKGNFLVPPEKSINKIGHALHAHD
O35164	QKIKVEKYILPPNYNVSSKFNDIVLLKLKKQANLTSAVDVV
O14925	MTGAAFGAMNGLRLGLKETQNMAWSKPRNVQILNMVTRQGA
A6ND01	LELWTVMPTWAGDELLNICMNAKHHKRVPSPEDKLYEECIP
Q9H1U9	HEKRPPILTSSKQDISPHITNVGEMKHYLCGCCAAFNNVAI
Q9ER80	PPPPQTRNTDFGNKTFQDFGNRTFQGCREPPQREIEPPLFL
Q99KJ0	LSAVSLNNLEPITRIQIWLANGERTVQRFNVSHRVSHIKDF
Q02539	VSKGTLVQTKGTGASGSFKLNKKASSVETKPGASKVATKTK
P08505	VLWEIVEMRKELCNGNSDCMNNDDALAENNLKLPEIQRNDG
Q9EQK7	RHPSYVGWFYWSIGTQVMLCNPICGVVYALTVWRFFRDRTE
Q9ESN5	NIQDSNAQLITLNEILEMLINRMFDVPHDPYVKIRDSFWPP
P34057	QKLEWAFSLYDVDGNGTISKNEVLEIVMAIFKMIKPEDVKL
Q6PII5	FCGHEHTLSNLEFAQKVEPCNDHVRAKLSWAKARPLSRRGK
Q8K572	GEMRRDVFHHFCMEGASQEFNEICLELMSEHLARAVKNAGN
A7YY49	DYIDTTWNCGYLLASSFVFLNLLGQLTGCILVLSRNFVQYA
P27105	VDGVVYYRVQNATLAVANITNADSATRLLAQTTLRNVLGTK
P04233	TAYFLYQQQGRLDKLTVTSQNLQLENLRMKLPKPPKPVSKM
Q96L46	EQLYQMIVRRYANEDGDMDFNNFISCLVRLDAMFRAFKSLD
Q3U497	DTYWCGIEKFGTDRGTRVKVNVYSVGKDTMSTSNQLPWPTV

Q9CQT2	ASVSYPQHHVGNLSPTSTSPNSYERTVGNVSPTAQMVQRSF
P78380	FNWEKSQEKCLSLDAKLLKINSTADLDFIQQAISYSSFPFW
Q9CXX9	ETPRQAEKLEEESRPPAAPGNTLDEAAAAEELPGVDVLLEV
Q8R1L4	FLGLYRLLYLANWIRRYQTENFYDQISVVSGVVQTIFYCDF
Q673H1	ARLREENARLRLENRRLRRENRSLFRQALRLPGDSGEREAA
Q07424	ASNFTAAPVYPHYVGYPHMSNMDPHGPSLGAWSSPYSPPRE
Q2NL24	QLRSTPFRYLLTPSMQKSVQNKIQSLNWEEMEKTPCIPEID
Q9H082	KFADTHSMPLFETSAKNPNDNDHVEAIFMTLAHKLKSHKPL
Q9JI59	GGTHNNSSYTMNTKSGILQFNMISKMDSGEYYCEARNSVGH
P01138	RRARSAPAAAIAARVAGQTRNITVDPRLFKKRRLRSPRVLF
P27814	ECPQDWLLHRDKCFHVSQVSNTWEEGQADCGRKGATLLLIQ
Q2KIE2	PEEFSFGFSFSPGGGMRFHDNFGFDDLVRDFNNIFSEMGAW
P20352	NLTWISTDFKTILEWQPKPTNYTYTVQISDRSRNWKNKCFS
P43120	RQVKTWFQNRRAKWRRLKQENPQSNKKDALDSLDTSCEQGQ
P15945	SGWGSITPVIYEPADDLQCVNFKLLPNEDCVKAHIEKVTDV
Q3ZBL4	ENTVAALKSEFQKTLNDQTENQKSLEENLATAKHDLLRVQE
Q86UA6	SIMLAEWEANPLICPVCTKYNLRITSGVVVCQCGLSIPSHS
A6QR46	TGYNVKQLFRRVASALPGMENVQEKSKEGMIDIKLDKPQEP
Q8TAB5	PGAEPEKMGGAGTVCSPLEDNGYASSSLSIDSRSSSPEPAC
Q9DAY2	EMTEEFPHWEYLSFLKSSDKNNKFLAMFNLSYCIDHDSKYI
P17931	HDALSGSGNPNPQGWPGAWGNQPAGAGGYPGASYPGAYPGQ
Q9CX11	PHFKSPVSGSECLLSMVDEGNPHHYFVATQDQNLSVKVKRT
Q91W82	SPDYPFKPPKVTFRTRIYHCNINSQGVICLDILKDNWSPAL
Q9NRE1	HEDGWPFDGPGGILGHAFLPNSGNPGVVHFDKNEHWSASDT
P25325	RGTEPEPRDGIEPGHIPGTVNIPFTDFLSQEGLEKSPEEIR
Q9D8U3	NVTSNSICLFRLVDDQQLHLNAEDIENLDAAKLSRFIHVNN
Q80XM9	MVFGGVVPYIPQYRDIRRTQNADGFSTHVCLVLLVANILRI
Q9H825	NLDSEKHKKGPMETGLFPGSNATFRILEVGCGAGNSVFPIL
Q96GG9	MAIAYWNLVLNGRFKFLDLWNKFLLEHHKRSIPKDTWNLLL
Q99MS4	KWPWQVSLRIYRYYWAFWVHNCGGSIIHPQWVLTAAHCIRE
O43822	LAELFYLKGLPRLRVLWLAENPCCGTSPHRYRMTVLRTLPR
Q8K4T5	EATFTTALSLVKEARPSICPNPGFMEQLRTYQVGKESNGGD
Q17QH6	AIGEMDNQVTQLTAELKFIKNAVAGVRETEQKMYLLVKEEK
Q16637	YGNREEQNLSDLLSPICEVANNIEQNAQENENESQVSTDES
P13598	HDTVLQCHFTCSGKQESMNSNVSVYQPPRQVILTLQPTLVA
Q0X0E2	RMESHSEEEDRPGAGGGLGWNGRSPRTQSLGACSAEAMLAR
Q8K3A0	EAAMEEIEATVRAKQKEFTDNINSAFEQGDFEKAKELLTKM
Q9UKR3	NIQLRSDEECRQVYPGKITDNMLCAGTKEGGKDSCEGDSGG
Q969X5	NELYVDDPDKDSGGKIDVSLNISLPNLHCELVGLDIQDEMG
P51911	DGIILCEFINKLQPGSVKKINESTQNWHQLENIGNFIKAIT
Q3T0C2	FRKVVDHFGKLDILVNNAGVNNEKNWEKTLQINLVSVISGT

Q9H2P9	QNGMHTLCLLDIKVKEQSLENLIKGRKIYEPPRYMSVNQAA
Q61199	SFNCHIEYEKTDRAKKTALCNFDPSKICYQEQTQSHVSWLC
Q9BRX8	FIRLGVWYNFFRAWNGGFSGNLEGEGFILGGVFVVGSGKQG
P46425	NDGVEDLRGKYGTMIYRNYENGKNDYVKALPGHLKPFETLL
Q969T7	FNEDGFLQGFKGQLIHTYNKNSSACENSGYFQQLEGKTNVI
Q96E22	SAAVLAPLGFTLRKPPAVGRNRRHHRHPRGGSCLAAAHHRM
Q3UY34	EAGAADKQAPTPQPSRRHEVNQHEEDGNDLRTTPEFRAHVA
Q06600	KPAAHLVGDPSTQDSLRWRANTDRAFLRHGFSLSNNSLLVP
Q99KR3	VTHWHSDHSGGIVDICKNINNDTTYCIKKLRRNPQREEIIG
Q9D241	EVRLCPQRLVGSAASATRPANWTGEEEQDTVSVNRVAARRL
P59542	SMWLAASLSIFCLLKIANFSNLISLHLKKRIKSVVLVILLG
Q9DAF8	RMSPCMQPKKPGFELLMSYRNRGKALLKRLQRQWDYESKLG
Q3SWZ6	LKEKMKIERAHMRLRFILPVNEGKKLKEKLKPLIKVIESED
Q9QZN4	VFYILCSLQRNLLRNPCAEENLSSWRIDSNGGDRWKVETLP
Q58DH1	KSTRQAPRGTNKTTNLSLKSNGKLRASSPVEDETAYDILRR
Q96AH0	EQKNNSMNSNMGTGTFGPVGNGVHTGPESREHQFSHAGRSN
P70190	VEEQENKFQAAVIEELQELSNEALRKILRRDLPVTRTLIDW
Q8K2U2	RQVFNAPKPKWTQLSGRKLQNWGGLPHPRGMVPERLPPWLQ
Q5JBG6	VIHPGYKNRISILYPWERRKNATCYLGYLKKGIYYQGCFNG
P59279	SWLEDARQHSSSNMVIMLIGNKSDLESRRDVKREEGEAFAR
Q00188	SGFAQLASQFPAEALFVVWLNPYWGPIEHEGKSFEQMKAYT
Q64471	ELYLDLLSQPCRAIYIFAKKNNIPFQMHTVELRKGEHLSDA
P24390	HDTFRVEFLVVPTAILAFLVNHDFTPLEILWTFSIYLESVA
Q14AM7	EAAAAAQPEHKQQKLWHLFQNSATAVAQLYKDRVCQQPGLS
Q2MKA7	CSEVNGCLKCSPKLFILLERNDIRQVGVCLPSCPPGYFDAR
Q00286	ASAALPLRMHHSAAECLPASNHATNVMSTATGLHYSVPSCH
Q6URK8	TTYCRKDEFDNAHFTLLGVPNKPLQCLDITATGQKLRNRYH
Q5T1C6	VVMINSQLDKVEGRKFFVSCNVQSVDEKTLYSEATSLFIKL
Q7TQI3	AAEEPQQQKQEPLGSDSEGVNCLAYDEAIMAQQDRIQQEIA
P22692	EIEAIQESLQPSDKDEGDHPNNSFSPCSAHDRRCLQKHFAK
P39687	LEFLSTINVGLTSIANLPKLNKLKKLELSDNRVSGGLEVLA
P09611	LFERATLVASNNYRLAREMFNEFNKQFGEGKNFTSKVINSC
P52743	QQSNLAQHQRVYTGEKPYKCNEWGKALSGKSSLFYHQAIHG
H3BQB6	QGGKPLKRKKSKCDATLIDRNESDESFGVVESDMSYNQADD
Q8BGZ2	PYKVPPTQSNTAPPPYSPSPNPYQTAMYPIRSAYPQQNLYA
P05142	QIQIPNQRPPPSGFQPRPPVNGSQQGPPPPGGPQPRPPQGP
O43692	LLCEASTVVLLNSTDSSPPTNNFTDIEAALKAQLDSADIPK
P51908	ISSGVTIQIMTEQEYCYCWRNFVNYPPSNEAYWPRYPHLWV
P41976	LKFNGGGHINHSIFWTNLSPNGGGEPQGELLEAIKRDFGSF
Q8CAK3	SCAHPKSRKQNHLPKVLHPSNPHISSGSTVATCLSQGGLVD
Q9NXJ0	SQPGQGNIQMINPSVGTAVMNFKEEAKALGVIQIMVGLMHI

Q9NZ63	VPTNMAVNYVQHNRFYHEELNAPIRRNKEEPKARPLRVGDT
Q8K3K7	DLMVKENLKVWIYPEGTRNDNGDLLPFKKGAFYLAIQAQVP
Q96LR5	ELAEITLDPPPNCSAGPKGDNIYEWRSTILGPPGSVYEGGV
Q17QJ0	VLVGPVPAGRHMFVFQADAPNPSLIPETDAVGVTVVLITCT
O43763	RLTAALSPFSGTRRIGHPYQNRTPPKRKKPRTSFSRSQVLE
Q9JL62	SKGQNVTEEECLEKIRLFLVNYTATIDAIYDMYTKMNAELD
Q5Y7A7	SECHFFNGTERVRFLDRYFHNQEENVRFDSDVGEFRAVTEL
Q3SZI4	FDISKKEMQPTHPIRLGLALNFSVFYYEILNNPELACTLAK
O00560	SSAARNGLLTEHNICEINGQNVIGLKDSQIADILSTSGTVV
Q14135	PGERQQNRPSVITCASAGARNCNLSHCPIAHSGCAAPGPAS
Q15526	IPVTAFGLGTWQVQRRKWKLNLIAELESRVLAEPVPLPADP
Q8N2G6	LAFGKGRPEQLGSPLHSSYLNSFFQLQRGEALSNSVYKGAS
Q9UNI1	TLNSYVQLGVLPQEGAILANNSPCYITGWGKTKTNGQLAQT
Q9UF11	RRIWVRVYSPYQDYYEVVPPNAHEATYVRSYYGPPYAGPGV
Q5E9L3	APYPAPAGSYPTPGLYPTPNNPFQVPSGPSGAPPMPGGPHS
O95749	LAVGLMQLFSDYKEDLKPLLNTLGLFFQIRDDYANLHSKEY
Q8IZJ6	GPFIYSDILDYKNLREIVVNNRITWLFHYSALLSAFGEANV
P53701	ARIRSWMGYELPFDRHDWIINRCGTEVRYVIDYYDGGEVNK
Q8WW43	VLLTHLLVSAQTFISSYYGINLASAFIIILVLMGTWAFLAAG
Q8N5S3	SIHHSKSHVGRGRIYYAKFINTNARTYNEPFPYIDPKKGPE
Q9D7T1	IFGVPGEPKAIRGVVLESVRNGMMESQLPGRKAVEQFVLET
P56749	GTLLPNWRKLRLITFNRNEKNLTVYTGLWVKCARYDGSSDC
Q9D7I0	FGEDNSIPVFCPDFCCGSCSNQYCCSDVLRKIQWNEEMCPE
Q8N7R0	KQVKTWFQNQRMKSKRWQKNNWLKNSNGVTQGCLVNPTGNL
Q9D1G5	TEFPSELQKLTSNLRTIDLSNNKIDSLPPLIIGKFTLLKSL
O14593	YTDIVGLLLERDVDINIYDWNGGTPLLYAVRGNHVKCVEAL
Q3ZCD8	EGVSNASVIMQVPERIVVAGNNEDIPFSRPADLDLIQSTPF
Q6QRN6	SSAVKYLSQEEAQAVDQELFNEYQFSVDQLMELAGLSCATA
Q8NHV1	AEKESQVQELVELIEKMVQCNEGAYFSDDIYKDTEERLKQR
Q9Z120	KPEEMDWSELYPEFFAPLIQNKSHDDPKDEKEKHSGAQVEF
Q6P1N9	GEFEKNNPDLYLKELLNLAENNKGKVVAIGECGLDFDRLQF
P53701	ERAYEYVECPIRGTAAENKENLDPSNLMPPPNQTPAPDQPF
Q96LD8	NSNQAAGGTHWSLLVYLQDKNSFFHYDSHSRSNSVHAKQVA
Q99426	PGYWIGVRYDEPLGKNDGSVNGKRYFECQAKYGAFVKPAVV
Q8BJ83	FDCTVKPSVTCVDQDLKPQRNFVINMTCRFCWQLPETDYEC
Q64695	ELPEEEEEGSEPHVFFDVAVNGSAFVSFRPKTAVWVSGSQE
F1MN90	VARVTFDLYKVNPQDFIGCLNVKATLYGTYSVSYDLHCSGA
Q3T014	RLNERHYGALISLNREQMALNHGEEQVRLWRRSYNVTPPPI
P12544	DPATREGDLKLLQLMEKAKINKYVTILHLPKKGDDVKPGTM
Q14CZ0	SVDTHQRSFDIGIQIGYQRRNKDVLAWVKKRRRTIRREDLI
O00161	ETEKTLTELNKCCGLCVCPCNRTKNFESGKAYKTTWGDGGE

Q5EA91	GCQPEQVTQRPEEGKESLSKNLLLVALCLTFGVEVGFKFAT
P97361	TGILTKVLPELIQGKVCPLVNGILSGLDVTLVHNIAELLIH
P15177	VSSTLALSGCGAMSTAIKKRNLEVKTQMSETIWLEPASERT
P20160	VAGWGSQRSGGRLSRFPRFVNVTVTPEDQCRPNNVCTGVLT
Q9P0N8	CTGTLGAVHKSCLEKWLSSSNTSYCELCHTEFAVEKRPRPL
P50172	QTSLSLFHDDIHSVRRVMEVNFLSYVVMSTAALPMLKQSNG
A6QQC0	KWGQGDPRWIVEEREDGTNVNNWHWTERDATSWSKGRLREL
P07628	KPDDLQCVFLKLLPIKNCIENHNVKVTDVMLCAGEMSGGKN
Q8R5B6	KLYPVVSAVWGHCEVTMRYINGLDPEPLPLMDLCRRSIRSA
P40337	ELGAEEEMEAGRPRPVLRSVNSREPSQVIFCNRSPRVVLPV
P10738	TTDVPDKYWKLYTYFVSKWVNREYRQLFTKNQFHQAMKHAK
Q6UW56	ICQGQKNLCNNTGDPEMCPENGSCVPDGPGLLQCVCADGFH
Q64302	KCARYIGYSLVWAAVFCIVANALLYFPNGETKYATEDHLSR
O75192	TVKNLCDILNPLDQLGIYKSNPGIIGLGGLVSSIAGMITVA
Q99KR7	ENFTLKHVGPGVLSMANAGPNTNGSQFFICTIKTDWLDGKH
O14990	ESSILAAHRATYRDYDLMKANEPGTSYMSVQDNGEDSVRDV
Q3T0B6	KKIQKYKSLPKMSGGWELEVNGTEAKLVRKVAGEKITVTFN
Q3SZD7	AFKTADTTPFHIQAEVTMKTNFFGTRDVCTELLPLIKPQGR
P50539	SSGSSNTSTANRSTHNELEKNRRAHLRLCLERLKVLIPLGP
Q62193	NEFTAHILEVVNSHMMLSKPNSQASAGRPSMSNPGMSESFN
P11672	MCLGLALLGVLQSQAQDSTQNLIPAPSLLTVPLQPDFRSDQ
Q8BKT2	RINRSLEELRLLLLERTRDQNLRNPKLEKAEILEFAVGYLR
O70338	VYWGPGHPLNVGIRLPGRQTNQRAEIHAACKAIMQAKAQNI
P01882	STHPEMSSWLLCEVSGFFPENIHLMWLGVHSKMKSTNFVTA
Q99KF1	SLFAGGMLRVHLDIQVGEHANDYAEIAAKDKLSELQLRVRQ
Q9NUM4	SYDVQKRTIYLNITNTLNITNNNYYSVEVENITAQVQFSKT
P19437	IYDCEPSNSSEKNSPSTQYCNSIQSVFLGILSAMLISAFFQ
Q01730	NNQIEELPTQISSLQKLKHLNLGMNRLNTLPRGFGSSRLLE
Q497H0	STPSTSNSQSDLFSEETTSDNNNTSVTTPTLSPSQQSLPTE
P36369	NWVLTAAHCYVDQYEVWLGKNKLFQEEPSAQHRLVSKSFPH
Q86XI6	ESESFVLDFSQPSADYLDFRNRLQADHVCLENCVLKDKAIA
Q2KIV0	ADELDRLVTAKTESVQYDHPNHTVTVTTISDLDLSGARLLG
Q2T9L9	EVGKLRIAKNQGRTEVSFTLNEDLANIHDIGGKPASVSAPR
Q8N1B3	KVEEQHLRTRDIINVSNRYFNPSGEPLELDSRFWELRDSIV
O35381	GDLEVLAEKCPNLKHLNLSGNKIKDLSTIEPLKKLENLKSL
Q3T112	SRASAGNYIATLKVNKVIEINPYLLGTMSGCAADCLYWERL
Q3T0X5	LLIAGYDDMGPHIFQTCPSANYFDCRAMSIGARSQSARTYL
P08505	SSGLLEYHSYLEYMKNNLKDNKKDKARVLQRDTETLIHIFN
O55144	LPASFAGLGAPFEDAGSYSVNLSLAPAGVIRVPAHRPLPGA
P56749	LPLSMLIAMGALLLCLIGMCNTAFRSSVPNIKLAKCLVNSA
Q28153	LLVFTSLVLYGHSTQDFPETNARVVGGTAVSKNSWPSQISL

O75461	YLTRKFMDLVRSAPGGILDLNKVATKLGVRKRRVYDITNVL
P62242	NFSWGSECCTRKTRIIDVVYNASNNELVRTKTLVKNCIVLI
A4FV75	EAYICPVLFGSASGGDHHHNNHGGSQGGSGPGSPHSPLPSK
Q6IMH0	AIEDWSKFVSRSEEFKLPCANKRVEGFSGYAVRYLKPEVTQ
Q9XT98	IGSVWYAVDVYVERSSLVLHNIFLGIQYKFGWSCWLGMAGS
Q9CQX0	QTPNDLLAAGFEEHKFRNFFNAFYSVVELVEKDSSVSSLLK
Q32KY9	LVEKKFLALQNKNSDADFQNNEKFVQFKQQLKELKKQYGLQ
Q8NC06	MPRPPETFLRRVTGWKEQVVNGDVGAVSEPPCLPKEPAPPS
Q9Z120	EDPIVEHLGSSTEEGKKVLRNGGKNFPAVFRRIQDPLLQAV
A4QPB2	VARSLYWTHTGTEHIEVTCLNSTSHKILVSEDMDEPRAIAL
P35242	IAAPRNPEENEAIASITKKYNTYPYLGVIEGQTPGDFHYLD
Q8NDC0	WGSMSSGPWAPGMGGQYPTPNMPYPSPGPYPAPPPPQAPGA
Q1RML1	GKDFPASPPKGYFLTKIFHPNVGANGEICVNVLKRDWTAEL
Q9D8T4	IDEDGKSHWVFESRKSTPQDNKTISEAESRIFWLGLIACPV
P51159	IALAEKYGIPYFETSAANGTNISQAIEMLLDLIMKRMERCV
P49406	LDDSLLYLRDALPEYSTFDVNMKPVVQEPNQKVPVNELKVK
Q9D650	VIHLHQGEIRQDSLYQAGAANVGRVVNSWYRYRPLVAELVV
P15530	SCGTELLVLGFSTLDQLKRRNTLKDGIILIQTLLIILFIIV
Q504P2	YSQLNQYGTWQESVMACSARNASLLKVKNKDVLEFIKYKKL
Q0P5E2	IRELIEVDDVMEDSTLQFGPNGGLVFCMEYFANNFDWLENC
Q2KHU0	EHPPHLTPGIRELVSRLQERNVQVFLISGGFRSIVEHVASK
Q925U0	ISLQCADHWFHLRIRPTIFHNIFMEPDEVFLGIGCPVTTTW
P14231	CVFIKMNRVINFYAGANQSMNVTCVGKRDEDAENLGHFVMF
Q8N1N2	HGKYILNVEHSENQPPITHPNDQEAHSSICWCLPSNDITSD
Q3ZBX1	ANTKEVRWQKVLYERQPFPDNYVDRRFLEELRKNIYARKYQ
P22676	MSKSDNFGEKMKEFMQKYDKNSDGKIEMAELAQILPTEENF
P51151	AWCRDNGDYPYFETSAKDATNVAAAFEEAVRRVLATEDRSD
P12544	ICAGALIAKDWVLTAAHCNLNKRSQVILGAHSITREEPTKQ
Q3ZCC4	QTSEAGSTGEEQKEEESNGFNKDLLDSPHNAGAASTVNEEE
Q99390	PEGNIKHPLIILCHGFCGIRNVLLPCFANAFTEAGFATITF
A7E2S9	SGADPNIVDVYGNTAVHYAVNSENLSVVAKLLSCGADIEVK
A4D1T9	EAPVMSDRECQKTEQGKSHRNSLCVKFVKVFSRIFGEVAVA
Q53H82	TGEPAIPEYISCLKQALTEFNTAIQEIVVTHWHRDHSGGIG
Q5BU09	EDEDKTVTKKKKKKQHRIPTNDELLYDPEKDNRDQAWVDAK
P04179	DVTAQIALQPALKFNGGGHINHSIFWTNLSPNGGGEPKGEL
Q8TDU5	SANHTLGFILFSWVLNMFITNNLLLFIVPTPNRIGASLLFV
Q9D4V7	RHEVLIRTAFLAEDFNAEEINLDCTNPRSSAAGSSNAVKLS
P62261	DTLSEESYKDSTLIMQLLRDNLTLWTSDMQGDGEEQNKEAL
Q9QVN7	PSTPRITTFPQVPITCDAVRNKCREMLTLALQTDHDHVAVG
P49863	LREVTVTVLSRKLCNSQSYYNGDPFITKDMVCAGDAKGQKD
Q9WUJ8	FLRLSKVKCVSLSARSSETSNAVICLDLAASCRKCPLDRAY

Q9P016	SGKRTKTENSGEALAKVEDSNPQKTSATKNCLKNLSSHWLM
P48307	LSSMTCEKFFSGGCHRNRIENRFPDEATCMGFCAPKKIPSF
Q02013	GCGINPARSFGSAVLTRNFSNHWIFWVGPFIGGALAVLIYD
Q3KRB8	SLRSSENKMDSSNLAVIFAPNLLQTSEGHEKMSSNAEKKGV
P32972	LVVAVAIILVLVVQKKDSTPNTTEKAPLKGGNCSEDLFCTL
O60248	PRRKAKSSGAGPSRCGQGRGNLASGGPLWGPGYATTQPSRG
Q8NEA5	QGAASRSTAASPTNPMKFLRNKAIIRHRPALVKVILISSVA
Q9CWE0	VAADAASASLTPDFFSSGSSNVSSPLPCFGSSLHSTTSFVI
Q8N6I1	RHPLSVLGINYQQFLRHYLENYPIAPGRIQELEERRRRFVE
Q28151	QKKYLIKHQNIHTGEKPYECNECGKAFSQRTSLIVHVRIHS
Q96MF7	LVEKKFLALQSKNSDADFQNNEKFVQFKQQLKELKKQCGLQ
Q27956	SSANSLFILYYTAQGEPFPNNLDKLCSPNVTDFPPFHANGT
Q08DN6	TRTATLCGRELPDGPSPLLKNAISLPVIGGPQALTLPAAQA
O35622	YGWGKITRLQYLYSAGPYVSNCFLRIRSDGSVDCEEDQNER
Q6PFX2	TLLKSGGAVSTPASTLWRATNNSSPDSFASLCSNSNSTSSS
Q8BX65	QEPGKEETGKIKNGGHTRMSNGNGIPHGAKHVSVENHKISA
O43711	LPASFAGLGAPFEDAGSYSVNLSLAPAGVIRVPAHRPLPGA
P09025	FQHASHHVQDFFHHGTSGISNSGYQQNPCSLSCHGDASKFY
A4FUA8	TIDGQQTIIACIESHQFQPKNFWNGRWRSEWKFTITPPTAQ
Q9H2A3	GAKTAETVQRIKKTRRLKANNRERNRMHNLNAALDALREVL
Q30154	NSQKDFLEDRRAAVDTYCRHNYGVGESFTVQRRVEPKVTVY
P97428	ASRAHHIFDEYIRSEAPKEVNIDHETRELTKTNLQAATTSC
P07288	EPEEFLTPKKLQCVDLHVISNDVCAQVHPQKVTKFMLCAGR
Q96KN4	LVVQNACGHLGLKSEEICWTNSESFAAWCRFGKREFKAGGE
P97466	LRAAPAGGQHYLHIRPAPSDNLPLVDLIEHPDPIFDPKEKD
Q8BVN0	LYEKRDLIKNLKTMKEDLMENLQDSQGNCTIQIQEDISEIK
Q9P1T7	VPSGEEIGKIKNGHTGLSNGNGIHHGAKHGSADNRKLSAPV
Q96E11	VNEEMKSVIEALKDNFNKTLNIRTSPGSLDKIAVVTADGKL
Q9CQL0	ESAAIGLQKFHKPLATFSFANHTIQIRQDWRQLGVAAVVWD
Q9TVC8	TWQRQVEPTVTIFLSRTEALNHHNLLVCSVTDFYPGQIKVR
Q9D3F7	LVLRLDSRLWPKIQGLLSSANSSLVPGYSQSLTLSTGFRVI
Q9NP50	NSDAHSTTSSASPAQSPCYSNQSDDGSDTEMASGSNRTPVF
O35963	KFADTHSMPLFETSAKNPNDNDHVEAIFMTLAHKLKSHKPL
Q2NL33	WEDSSRPSPSLFSAYEYAQINESKGCAYFQNGNIYISRCSA
Q61160	RSLSERVRESLKVWKNAEKKNASVAGLVKALRTCRLNLVAD
Q2HJI0	TFILRNTIEGQGVEICFELYNPRIQEIQVVKLEKRLDDSLL
Q8BX65	LPQLQTSAQEPGKEETGKIKNGGHTRMSNGNGIPHGAKHVS
Q9CVB6	CFASVFEKYFQFQEEGKEGENRAVIHYRDDETMYVESKKDR
O35658	KEVSFQATGDSEWRDTNYTLNTDSLDWALYDHLMDFLADRG
Q96GG9	RQFMIFTQSSEKTAVSCLSQNDWKLDVATDNFFQNPELYIR
Q61200	DLRYDTPEPYSEQDLWDWLRNSTDLQEPRPRAKRRPIVKTG

Q8CCI5	PPSEANSIQSANATTKTSETNHTSRPRLKNVDRSTAQQLAV
Q96LD8	FLGRKGDKLAFVEEKAPAQQNSYDCGMYVICNTEALCQNFF
Q3MHM8	ACLLAITYLIVKELHAENLKNEDDVNTGLLGFWSLLIISLT
Q9NVX0	ASAPNGAGLVLGHFIASGMVNQEMLNMSKKTVSCFVNFTRL
P17931	DVAFHFNPRFNENNRRVIVCNTKLDNNWGREERQSVFPFES
Q99KI3	PPSPMTDPTMLTDMMKGNVTNVLPMILIGGWINMTFSGFVT
Q8BWY2	NRKLIETLQQVAVKLCRELYNKSGGHRCSPCPEKWKWYGDK
Q8MJ50	GYADIVQLLLEKGARTDLRNNEKKLALDMATNAACASLLKK
Q78HU3	QKAGYFLCLSTLGIPENPQDNVVVDMQIVMDKGPLPSGFSA
P29020	LYLRAKFSRSAETAANLQDPNQLYNELNLGRREEYDVLEKK
Q8BVA2	KHRMHNLCCDNCHSHVALALNLMRYNNSTNWNMVTLCCFCL
Q9CY52	VRLDGRNFHRFAEEHNFAKPNDSRALHLMTKCAQTVMEELE
P43528	YNDGYVSTTTTLRQAHFLGQNMLGGYNEYYIYVVAAAPNLF
P25732	EQNISLNISRNAKKNIIIYNNGNVRAGVKDIYFCKSSNIDD
O75822	LKKLQEESDLELAKETFGVNNAVYGIDAMNPSSRDDFTEFG
Q14990	CTSPCSPCSPCSPCNPCSPCNPCSPYDPCNPCYPCGSRFSC
Q80WB5	WREPPPEYPCIETGDSKMNLNDFISMDPAVGWGAVYTLPEF
Q8BXX9	VVQLSTLEIRHKIAELEANLNGDLAGSEWKTRYETQLELND
O18870	ESQISTDESENSSRSPLNKPNNIRSRAAPWNSFLPPPPHMP
Q9JIQ3	WVTRSVCSLFRYRQRFPVLANSKKRCFSELIKPWHKTVLTG
Q8N131	AAMAASANIENSGLPHNSSANSTETLQHVPSDHTNETSNST
Q9BQP9	NIQLDCGGIQISFHKEWFSANISLEFDLELRPSFDNNIVKM
Q8VD53	VVCVLSAITTCLAFIKPAINNISLMILGLPCTALLVAELKR
Q6UXV1	ARAGAVLMGMEGPFFRDYALNVFVGKVETNQLDLVASFVKN
A6QP81	PGDSNEIEASLRSLQKFVPTNYASYTQEYYRFVGKKIVIQE
Q9S169	ADKTGAGERGARGIVALLGPNNKAERIVVIYLRDTPASMAE
P28585	AELERQSGGRLGVALINTADNSQILYRADERFAMCSTSKVM
P16152	GTRDVCTELLPLIKPQGRVVNVSSIMSVRALKSCSPELQQK
Q9DB75	PPYEPPGQPVPQPGFVPPHMNADGTYMPAGFYPPPGPHPPM
P33783	TPGEDFSGELKLEGAVTSTRNPWVWKVGQGNESLEVKQSRG
A6QQ85	QLRVLRVGNDTRCSPRTRGCNRPLPGPGPYRVKFLVMSDRG
O96011	KQIRQLESHLSLGRKLLRLGNSADALESAKRAVHLSDVVLR
P97328	DVSQVTWQSQGDTPCSCCIVNNSNGSRTIILYDTNLPDVSA
Q9EST5	RIHLELRNRTPAAVRELVLDNCKAMDGKIEGLTDEFVNLEF
Q9JKY0	HSFGTIAALLQEIVNIYPSINPPTLTAHQSNRVCNALALLQ
Q5E9I1	GRDLTFWQELVSKCLAEYSSNKCAKPNVQKLKWIVSGRTAR
Q2TA42	KAREEEERAKREELERILEENNRKIAEAQAKLAEEQLRIVE
Q7YRA3	YGPHEIRGSRARALPDRALVNCQYSSATFSTGERKRRPHGD
Q9JM71	ICGGVLLDPNWVLTAAHCYGNDTSQHNVWLGKNKLFQREPS
Q3T123	PHSEAGMRVKTEPMDTDDSNNCIGQNEQQRENSGHRRDQII
Q13158	EDRYPRNLTERVRESLRIWKNTEKENATVAHLVGALRSCQM

P63013	LSWGTASPYSAMATYSATCANNSPAQGINMANSIANLRLKA
Q91VN4	MDEEERVRVLQGIRLSESVVNRMKDCSQPSAGEQLVPGFGP
P51122	DLSTIEPLKKLENLKSLDLFNCEVTNLNDYRENVFKLLPQL
O09044	NGQPQQTTGAASGGYIKRITNDAREDEMEENLTQVGSILGN
Q8BYH0	SCCVPDPMGLSFLPTYGCQSNRTASVAALRMKAREHSEAVL
Q9EQC7	VVCRAAPCPVPSNPGQELCGNNNVTYISSCHLRQATCFLGR
P00847	IIETISLFIQPMALAVRLTANITAGHLLIHLIGGATLALMS
O75871	SLLFQNITLEDAGSYTLRTINASYDSDQATGQLHVHQNNVP
P09493	AELSEGKCAELEEELKTVTNNLKSLEAQAEKYSQKEDRYEE
O00338	LYVARNAKDCMVSYYHFQRMNHMLPDPGTWEEYFETFINGK
Q3T046	CVCPGTVDTPSLQERIQARPNPEEALSDFLKRQKTGRFATA
Q96MU5	GPVSSPLLGDHRCLVPFRDLNPSSEVNTANLLESPSSLLLT
Q92564	NKDQWCNVLEFSRTINLDLSNYDEDGAWPVLLDEFVEWYKD
A5PKD8	WVLINPLRKEDEGVYQCHSANAVGEAQSHGTVTVVDRSQYR
Q17QQ9	SIQTSKTEAETVRVSTEKLKNRKEKRSRDVASKKEERKRRK
O08899	VVTCQRVSQEEVKKWAESLENLIHHECGLAAFKAFLKSEYS
Q9BUV0	GTRNPNEKPTQQRSIAFSSNNSVAKPIQKSAKAATEEASSR
P0CI00	EEWDMMDTSKRKLYRDVMLENISHLVSLGYQISKSYIILQL
Q8K4T5	ECFEFIEQAKLKDGVVLVHCNAGVSRAAAIVIGFLMSSEEA
Q8C4B4	RERLLKTFDFDFGFCIPSSRNTCEHIYEFPQLSEDVIRLMI
P0C8J7	VPAFNIHNLETMQVVVETAANLHAPVIIAGTPGTFTHAGTE
A2A8T7	NVAINTGPPPAVTKTEPEDQNSDSLWELDLSEGRNFVVQDS
Q3T0Q3	HVTSRDLISNSPRVIPVTSRNRDNDPNDYVEQDDILIVKLR
Q8NAU1	LFMWAGVIALFCRQYDIIKDNEPNNNKEKTKSASETSTPEH
Q9ESN5	TEEELIKECEEMWKDMEDCQNKLSLIGTETLTNADAQLSLL
A6QQL5	QHSKPCVGRGRVYYAKFINTNVRTCNEPVPYIDVKKEPENQ
Q9Y508	RKNFFLSKIRSHVATCSKYQNYIMEGVKATIKDASLQPRNV
Q9ESP1	GGASKASAGLVTCGSVLKLLNTHHKVRLHSHDIKYGSGSGQ
Q86TL2	FANVYLADLTEEDPCSLYLINFLLDATVGMLLIYVGVRAVS
Q2KIJ1	TKEEDDGLRKSLDKFYEVFGNPQPASGNSLSTSVCQCLSQK
Q91VT4	NRDSLLVRTKTEDMISQLHTNLLGSMLTCKAAMKTMIQQGG
Q9CPQ5	LNYLKDVSKMLKMEKAQERANEESLASLQEEIDKIVETTES
Q8IZU3	LKTIKQLYEQFIKSMEELEKNHDNLLTGAQNEFKKEMAMLQ
P70190	RRDLPVTRTLIDWQRILSDLNLVMYPKLGYVIYSRSVLCNW
Q9H3L0	NRARLVSYLPGFCSLVKRVVNPKAFSTAGSSGSDESHVAAA
Q8BVD7	SAFSVGITTSYPEERLPIIFNKVLFNEGEHYNPATGKFICA
O95567	GLPTEGYQALYHAVVEPMLWNPSGTPKRYSLELGKAIKQKL
Q9CY28	YGYRAPEDFVDMVETYLKERNNLKRTFLLVDSVVGITKLDN
Q3KRB8	AVIFAPNLLQTSEGHEKMSSNAEKKGVYQTLSWKRYQPCWV
Q7SIB3	FSTMPFLYCNPGDVCYYASRNDKSYWLSTTAPLPMMPVAEE
Q96ES7	LHQLIKQTQEERSRSEHNLVNIQKTHERMQTENKISPYYRT

Q5E951	LTDFKPGYWIGIRYDEPLGKNDGSVNGKRYFECQAKYGAFV
Q6IPR3	MVTEEYIDFLLNVANQKMEENKKRIERFYNCLQHALERETM
P11019	SDADVQKQIKHMMAFIEQEANEKAEEIDAKAEEEFNIEKGR
A6NM15	IVDSKYGLKHLTEEKPDGLINEATRQVALADIILINKTDLV
O35565	SSSSSSFSSPSSAGRHVRSYNHLQGDVRWRRLFSFTKYFLT
P20774	NQLLKLPVLPPKLTLFNAKYNKIKSRGIKANAFKKLNNLTF
P09564	MHRLQLSDTGTYTCQAITEVNVYGSGTLVLVTEEQSQGWHR
Q2TBN9	EEVQEEDFVLQVAAPPSIQVNRVMTYRDLDNDLMKYSAFQT
Q5T0U0	KRDFVKKLKTMKEELMQDLQNPGGNRITQVQEDITNLKDKI
P81126	AEAEKRVKASHSFLRGLFGGNTRIEEACEMYTRAANMFKMA
Q3SZC2	FDGGVVVGSDSRVSAGEAVVNRVFDKLSPLHQHIYCALSGS
A6NN92	NEALHFICDPDKREVNLFCYNQFRPITPQVSFSALQLVIVL
A4FV84	LRKCVDTYKYLFIFSVANMRNSKLKDIRNAWKHSRMFFGKN
O95150	ALHWEHELGLAFTKNRMNYTNKFLLIPESGDYFIYSQVTFR
Q9D0B0	PFAFVRFEDPRDAEDAIYGRNGYDYGQCRLRVEFPRTYGGR
Q8BPB0	QEEAHLNTSFKHFIFFVQEFNLIDRRELAPLQELIEKLTSK
Q2TBU3	ENLMGSVRKDIESYKSGSGVNNRRTELFLKEHDHLRNSDRL
Q864V4	QLPCEDPADGSGRAPVLRVGNDAGCLADLHQPRYCNAPLPG
Q8JZV9	VHHGTILDCEEKDWDFSMNLNVRSMFLMIKAFLPKMLAQKS
Q6ZWI9	VGEVKSWSLGVCKEPADRKSNDLFPEHGFWISMKAGAIHAN
H3BNL8	TNSYLLLKFWTCYVLSVMTCNNLSCVKELKDHSALKYHLQM
Q9UBS3	SFNFNFDDLFKDFGFFGQNQNTGSKKRFENHFQTRQDGGSS
Q8K201	ESRRFRGFQDSVEVVKLPPPNREDSHFFFHLLIFAFCAAVV
Q0P5C7	ITSQEEVQEKYVVCVLFIFRNLLDMVRYTYSMLSVIGISYA
Q9WU03	NITDGSCQPFVYGGCEGNGNNYQSKEECLDKCAGVTENTTD
Q9ERS4	SALHRLPQRRRRTYSDTDSCNDVPPEDPERPLHCSGNTLNG
Q96E22	QQQELLGLDCSKYSPEFANSNDKDDQVLNCHLAVKVLSPED
Q9Y399	YRKGIILFISRNRQFSYLIENMARDCGEYAHTRYFRGGMLT
P30931	EWEPKPINHVYTVQISPRLGNWKNKCFYTTNTECDVTDEIV
P30280	SVKPQELLEWELVVLGKLKWNLAAVTPHDFIEHILRKLPQQ
P49895	EAHASDGWAFKNNMDIRNHQNLQDRLQAAHLLLARSPQCPV
Q9JKX8	EILNAYLVRVGNNGTCFWDPNFQGLCNPPLTAATEYRFKYV
Q6NZA9	NPSMIGPKNILITTSMVSSQNTATDSNPLKRKHDDDDDNDT
Q9CWQ0	FWTDTWRPESFFDKVKRNRANGMHTLCLLDIKVKEQSLENL
A6NJI9	PKSPFKQKPAQRVPSDFAFANNVDKTVLDDPEDAVFVRSMK
Q9Z211	TVKNFCDILIPLNQLGIYKSNLGVVGLGGLISSLAGLLTVV
Q9H902	LVQAKDRSYDALVHFGKRGLNVAATAAVMAASKGQGALSER
Q1LZ95	DDLDERQVQLMAEMCILVDENDRRIGAETKKNCHLNENIER
Q9WTL2	CMFAENNGLLFLETSALDSTNVELAFQTVLKEIFAKVSKQK
Q15014	AKKNVDAILEEYANCKKSQGNVDNKEYAVNEVVAGIKEYFN
Q86V35	IPEDELEEIREAFKVFDRDGNGFISKQELGTAMRSLGYMPN

Q6PHW0	EDADEWQESEENVEHIPFSHNHYPEKEMVKRSQEFYELLNK
Q3T040	AEEQATVERNPYTIFHQALKNCEPVIGLVPILKGGHFYQVP
Q3ZBL4	IDSMKPKLAPLHEGGAAELLNKEIIRLQEENEKLKSRLKTI
Q9BXY4	EAIPDSKSLESSKEIPEQRENKQQQKKRKVQDKQKSVSVST
Q08DT6	GEEKVKSGASWTCQQLRNKSNEDLHKLWYVLLKERNMLLTL
O55100	VSWVFSIVVFGSIVNEGYLNNPEEEEEFCIYNRNPNACSYG
A2VDU1	LTILPIDQMKTSHVENDYIDNPGLAPPSGPKRTRGGAPELA
A6NDL7	QVKELSWGVALDTNFPRSSNNFDYILAADVVYAHPFLEELL
Q1LZB9	VVCRAAPCPAPSSPGQELCGNNNVTYLSSCHLRQATCFLGR
Q8N2K1	DYLRIKKDPVPYICAEPLPSNILEWHYVVRGPEMTPYEGGY
Q6URK8	GVPNKPLQCLDITATGQKLRNRYHEGKLAPIAPGINRVDWP
Q6ZTC4	PPTLVPGGSPGTEAQPVAPANALGSRSKLNLTQPSCLSSGS
P10649	RKHHLDGETEEERIRADIVENQVMDTRMQLIMLCYNPDFEK
Q8N7P3	CLTNYLPHWKNLNLDLNEMENWTMGLWQTCVIQEEVGMQCK
Q96AH0	SEPNPDYRGQQNKGAQSEQKNNSMNSNMGTGTFGPVGNGVH
Q921C1	YVAHLGVRLALEGAALGVQYNLYGFKMSSTFICREDPCIGS
Q9Z1B5	VQKYGLTLLTTTDPELIKYLNNVVEQLKEWLYKCSVQKLVV
Q71SS4	WVILGFNYIIGGSVINELIGNLVGHLYFFLMFRYPMDLGGR
Q2PT27	GIEIRHCRAQGSDFRGASFMNMITTRTWFCSAYITNTNLSY
Q0VCQ4	LAALTRPVSEVTLQTVRGRQNDHGQCMAYAAVPVRHFATKK
Q9P0N8	LWTLVSFRYHCQLYSEWRKTNQKVRLKIREADSPEGPQHSP
Q91VJ5	DRSHEKADRNHEKNDRERERNYDKVDRERDRDRERERAFDK
P62955	FFILSGLSLVVGLVLYISSINDEVMNRPSSSEQYFHYRYGW
A4FV75	GVTLMSNLEQLLRGVWKPETNEILHMSFPTKASLYGAILFT
Q13007	FSTLANNFVLIVSQLQPSQENEMFSIRDSAHRRFLLFRRAF
O35566	IIAGILAYVYYQQLNTELKENLKDTMVKRYHQSGHEGVSSA
Q8VI88	ILCYLNHKNYWSLIMSSTTINTACSSLGPESLVSPSQTPSS
Q9WTY4	VGAIAGAGILYWLAPGNARGNLAVNALSNNTTPGKAVVVEL
Q62393	KNYLYLSPSGNTSPPGSPTQNVGLLKTEPVAEEGEDAVTML
O35621	ALLRLPKKRGTFIEFRNGMLNVSPIGRSCTLEERIEFSELD
Q9JHQ5	RSEFQKTLNDKTENQKSLEENLAAAKHDLLRVQEQLSMAEK
Q5E9J3	IEGETLAIQVQDTPGIQVHENGLSCTEQLNRCIRWADAVVI
Q9BQP9	EWFSANISLEFDLELRPSFDNNIVKMCAHMSIVVEFWLEKD
Q6ZUJ4	HTSIRTIEELAGKIEFENELNHMCGHCQDSPFKEEAWALLM
Q64669	GWFERVLVAGFAYTYAAMYDNGPFQNKKTLLSITTGGSGSM
Q58D49	VCRRAERRVVPLVQTGETDANVVKFLNRLSDYLFTLARYTA
Q9H8T0	ARLFDQPKIEDPYAISFSPWNPSVHDEAREKMLTQKKPEEQ
Q8BWP5	VFRVSLITSELIVQEVETQRNGVKAIFDLEGWQVSHAFQIT
P18469	RWFRNGKEEKTGIVSTGLVRNGDWTFQTLVMLETVPQSGEV
Q8VC52	NGIRFDPENPQTLRLEFAKANTKMAKSKLIATPNPTSVHPA
P52797	STSHSGEKPVPTLPQFTMGPNVKINVLEDFEGENPQVPKLE

Q8BX65	HTRMSNGNGIPHGAKHVSVENHKISAPVSQKMHRKIQSSLS
Q3ZBL4	DLSDLENTVAALKSEFQKTLNDQTENQKSLEENLATAKHDL
Q9Y6N1	VKDRIIKISFNADVHASLQWNFRPQQTEIYVVPGETALAFY
Q8K354	DTLTEVDLVDLMKKFVEDTKNEVHEREGWPDSAYGVSKLGV
Q8SQ24	LGKKISVPRDVMLEELSLLTNRGSKMFKLRQLRVEKFIYEN
P00492	CVLKGGYKFFADLLDYIKALNRNSDRSIPMTVDFIRLKSYC
Q8NFX7	RQVNGIDPNGDSAEFDLLFENAFDQWVASTASEKCTFFQIL
Q9D387	EVFWVDHAYTLRMLFVKESHNTSKGPEATWNLNKVHFVYDS
Q0IIL5	RIQKEDFWHSYIDYEICIHTNSMCFTMKTSCVRRRYREFVW
O55100	DIGVSAFWAFFWFVGFCFLANQWQVSKPKDNPLNEGTDAAR
Q3UG98	PYRTRKAEPVTATLSEQKSWNCPLPRPDGCMGDTSAVSSVC
P01881	KEPDMFLLSECKAPEENEKINLGCLVIGSQPLKISWEPKKS
O88452	FVTLALVFATLDPAQGTDSTNPPEGPQDRSSQQKGRLSLQN
Q3UFS4	RNRQKSVKEEERERRDIGLKNALGCENKGFALLQKMGYKSG
Q9P0W0	TQPMKRDIKKAFYEMSLQAFNIFSQHTFKYWKERHLKQIQI
Q7TNV1	VCLGKILIQYKQQHTLLHKVNGALMLLSFLCCRVLLFPYLY
Q99463	LRRRNAKVDKKKENEGRLNENKRINTMLISIVVTFGACWLP
Q8WUB2	DGYKVNSHIAKLQELWKTPQNQTIHLSKSMMEASFFKHPDL
Q3T054	YIQAQCAIIMFDVTSRVTYKNVPNWHRDLVRVCENIPIVLC
Q5E948	ITDHIAQGPDPNTEESPKRKNITYEELRNKNRESYEVTLTH
Q9D176	GEDLETVQAAYLGLKGHNHNNSSSVGGGNGGPSGGGGKPGI
O09164	VVVHAGEDDLGKGGNQASLQNGNAGRRLACCVVGTSSSAAW
O02659	YIRLATSERATLQSELNQIKNWLIFSLGKRVGKKAFFTNGK
Q91ZT8	GEQRGRSDRPGGSPHLPFLSNPLMGDVVSDWSPLHDAAIHG
Q5TBK1	RALIFSTDDFFFREDGAYEFNPDFLEEAHEWNQKRARKAMR
Q9ER80	FQELMQEEKPGAKWSLHLDKNIVPDGAALGWRQHQQTVLGR
Q9QZ28	CFKERTRHLLREWYLQDPYPNPSKKRELAQATGLTPTQVGN
P13621	QIYGIEGRYATALYSAASKQNKLEQVEKELLRVGQILKEPK
P09056	LLLVLHWKHGAGSPLPITPVNATCAIRHPCHGNLMNQIKNQ
Q08DG5	SACKEPVVYPWMRKVHVSTVNPNYAGGEPKRSRTAYTRQQV
Q5U4N7	ERALISLKNRSRFLVRPNTRNRAFSWTCRAARSKPRPRRST
Q9JMG4	TALERQVFDFLGYQWAPILANFTHIIVVILGLFGTIQYRPR
Q9H3R5	IRQNLQMEIKITTVIQHVFQNLILGSKVNWAEDPALKEIVL
Q0IIL2	LPLSILIAMGALLLCLIGMCNTAFRSSVPNIKLAKCLVNSA
Q5VVY1	LYLLEKIPLVKLYALTSQVINGEMQFYARAKLFYQEVPATE
Q9Z0G4	GPSGRGAVCLFEEDDGSCEVNGRRYLDGETFKPNCRVLCRC
P97930	ETGTFQKQVLLCFQQLMEEKNLNWKVVDASKSIEEVHKEIR
Q7Z5L3	KAQPPGPSTAALEVMQDLSANPPPPFIQGPKGDPGRPGKPG
Q08DP3	VNENSDTVGQIVQYIMKNEANVDILAAMVEDNNVCDPESPV
Q922M7	EFLLLTLEQKNIAVENEVRVNKDNLTDLYVQHAIPLPQRDL
Q9P015	PIPKRMLPPEELVPYYTDAKNRGYLADPAKFPEARLELARK

Q1LZE2	DIQTPESPGQGEPIPSELRENSLPSCSLHTSTPKSPEPGPV
O35426	TSWTLSCFSNVLPQSLLVWRNSQRSTQKDLVPYQPPFLCQW
Q9NY12	LGEFLHPCEDDIVCKCTTDENKVPYFNAPVYLENKEQIGKV
Q3MHP2	NLSFIETSALDSTNVEEAFKNILTEIYRIVSQKQIADRAAH
Q9NPE2	GHSVSGSLLMPGHEASSKDPNHSTALKVIESDTHRTNTPRR
Q8R0A6	EQGTEAAGSQVELLPDRDPDNDGTKISTVKVQGNDISHKLQ
O88696	QATDIAIQAEEIMKLKKQLYNIYAKHTKQSLQVIESAMERD
Q8CG50	SLAEHYDILCAIETSAKDSSNVEEAFTRVATELIMRHGGPM
Q91V64	VSERCKLRLVPLQIQLTTLGNLTPPSTVFFCCDMQERFRPA
Q8VBT3	ASYPQPWLGAHPAAVVTPGINVTLTCRAPQSAWRFALFKSG
P49406	VNELKVKMKPKPWSKRWERPNFNIKGIRFDLCLTEQQMKEA
Q9Y272	DLHRKVSVQYCDVLHKKALRNKKLLRAGSGGGGGDPGDAFG
O60238	HNNNNNCEENEQSLPPPAGLNSSWVELPMNSSNGNDNGNGK
P12342	VKQVTPAPEEHREKKHTDAQNQTQPPEEADLPGHCEEPPPW
Q9QUN5	LWVYVTATRYDRKSNEEIYDNLLSSSRHIHRVAKKMYKILD
Q9NZD2	RGLRFIQVFLQSICDGERDENHPNLIRVNATKAYEMALKKY
P09936	IQAAHDAVAQEGQCRVDDKVNFHFILFNNVDGHLYELDGRM
Q9BXS1	RIWGEHEICYLLLVRKNVTLNPDPSETKSILYLSQEELWEL
Q5VZY2	EIKEAFLAVSLALALNGVCTNTIKLIVGRPRPDFFYRCFPD
Q91W82	DYPFKPPKVTFRTRIYHCNINSQGVICLDILKDNWSPALTI
P50296	LDLQTLTEILQHQIRAIPFENLNIHCGKTMELSLEDTFHQI
Q99612	PPEDTLISPSFCYNLETNSLNSDVSSESSDSSEELSPTAKF
A0JN69	LCHFRFAVERKPRPLVEWLRNPGPQHEKRTLFGDMVCFLFI
Q8NBA8	ARTLQEPVARPSGASSSQTPNDKERREGGAVPAAAALGAEA
Q9EQF4	LGLWAVLPTLAGDKLLSVCMNSKRHKQEPGPEDELYQECRP
Q9QXN5	EAVGMLDDLVDESDPDVDFPNSFHAFQTAEGIRKAHPDKDW
Q08DH5	SKLRETMPLPLKNQDDSSLLNLTPYPLVRRRKRRFFGLCCL
Q3T189	SKIYPLPHMYVIKDLVPDLSNFYAQYKSIEPYLKKKDESQG
Q32L19	RKRKLEVLDSQLSEGNQKYANLQKSIEKAKVGRHETEERTM
Q28151	HTGEKLLECNECGKSFSQKENLLTHQKIHTGEKPFECKDCG
P10301	LFTQILRVKDRDDFPVVLVGNKADLESQRQVPRSEASAFGA
Q28030	HVQKLNKFLEPYNDSIQAQKNDVCRPGRYYEQPDNGVLNYP
P50194	SGTSVKAVDFIHYDEKNNEWNLLQVKNRDNTENSSSSKIRD
P32972	VQFPGLYFIVCQLQFLVQCSNHSVDLTLQLLINSKIKKQTL
Q9NZ63	EQQNKKKDSETSFVPTNMAVNYVQHNRFYHEELNAPIRRNK
Q9EQC7	CGPGKACRMLGGRPHCECVPNCEGLPAGFQVCGSDGATYRD
Q99J99	AEFSAQLDPSFIKTHEDILENLDARRFQVVDARAAGRFQGT
Q00188	DTDPVNATFEGYKALNVRRLNIMAGDEINSRNFDTLVELIA
Q9H1Y0	WHYPIGLLFDLLASSSALPWNITVHFKSFPEKDLLHCPSKD
Q92520	WVFCGGKGIKTKSPFEQHIKNNKDTNKYEGWPEVVEMEGCI
Q3MHR7	ADELLKRVYGSYLVNPESGYNVSLLYDLENLPASKDSIVHQ

P30041	KLIALSIDSVEDHLAWSKDINAYNCEEPTEKLPFPIIDDRN
Q15286	YKFAGQMGIQLFETSAKENVNVEEMFNCITELVLRAKKDNL
Q3ZBM4	AIEKLKLSEGKSLEGPLDLINYIDVAQQDGKLPFVPLEEEF
Q76JU9	LVLCHLGLALGLETSGSWLDNSTSSLGINIPVNGSPVCLEA
Q9CRA5	VLTTEKQNFLLFDMTTHPLTNNNIKQRLIKKVQEAVLDKWV
Q5VT99	VHEDAFETLESLQVLELNDNNLRSLSVAALAALPALRSLRL
Q9ULY5	QLPENFTELSCYNYGSGSVKNCCPLNWEYFQSSCYFFSTDT
A8MXV6	KSIKTRLGRRVPAAPPALRRNLLLQAWKCVCNWASRLFAPN
Q7TS99	TPVSHKVIEKRRRDRINRCLNELGKTVPMALAKQSSGKLEK
P00915	DIKTSETKHDTSLKPISVSYNPATAKEIINVGHSFHVNFED
Q91ZD6	ECILIINHDTGECRLEKLSSNITVKKTRVEGSSRIQYRLEQ
P45877	MTVVHSIELQATDGHDRPLTNCSIINSGKIDVKTPFVVEIA
Q17R06	DEDSFQKVKNWVKELRKMLGNEICLCIVGNKVDLEKERHVS
P32856	VEEIRNSIDKITQYVEEVKKNHSIILSAPNPEGKIKEELED
O14494	LGETLSVYCNLLHSNSFIRNNYIATIYKAIGTFLFGAAASQ
Q80XD9	NTEYNNLVLTRGGGECAYLSNRGIYNSSGDIHKKWICNKPN
P00435	LGFPCNQFGHQENAKNEEILNCLKYVRPGGGFEPNFMLFEK
Q3ZBW4	ARLVQGSILKKVLEALKDLINEACWDISSSGVNLQSMDSSH
O94903	HGLPPSETIAIVEHINAKCPNLEFVGLMTIGSFGHDLSQGP
Q9DBJ1	SLRGIVKHLEGLSEEAIMELNLPTGIPIVYELDKNLKPIKP
P56177	LANGRALSAGSPPVPPGWNPNSSSGKGSGGNAGSYIPSYTS
Q8C0Y1	LHRYNAYPSEQEKLSLSGQTNLSVLQICNWFINARRRLLPD
Q5QGZ9	RVDNIINSSAWVIRNAPDLNNMYCGYINRLYVQYYHCTYKK
Q96DC9	SLLGKSREIFKFKERVLQTPNDLLAAGFEEHKFRNFFNAFY
Q3ZBD9	EKERDFYFGKLRNIELICQENEGENNPVLQRIVDILYATDE
Q17QJ0	QRNILASNPRVTRFHINWDNNMDRLEAIENQDSSLGCGLPL
Q2KIV9	GESGDYKATQKIAFSASRTINHHQRQGQPIRFDHVITNANE
Q9CWJ3	PCYKTKQSPKSGGCDMANKENELACAGHLPENLRHDSRTFV
Q3TT38	LSSFETEFNTQPHRKVEGNFNPFASPQKNRQSDENNLTDPG
Q9D8U4	GKFVCSVPGIYYFTYDITLANKHLAIGLVHNGQYRIRTFDA
P47756	SLTRQMEKDETVSDCSPHIANIGRLVEDMENKIRSTLNEIY
Q99JV5	GRKIKLEGLSDVASISTKLQNTLIQYHSIKEDEWRVAKKVK
O88441	WPENATLYQQLRGEQILLSDNAASLAVQAFLQMCNLPVKVV
P46777	IVCAAYAHELPKYGVKVGLTNYAAAYCTGLLLARRLLNRFG
Q5D525	RLEKVEQRLQEARETWDSPGNCGLKTELEELEGQSQRSPEA
P21266	WFAGEKLTFVDFLTYDILDQNRIFDPKCLDEFPNLKAFMCR
Q9BTL4	AEGAFPNLARVLQRRFSGLLNCSPAAPPTAPPACEAKPACR
Q96L16	MLKSERDALFDIDRERQGHQNRMRPLPKRVFLAVQKNKPIS
O75494	RRRSRSRSFDYNYRRSYSPRNSRPTGRPRRSRSHSDNDRFK
P43528	RSGGLIPRGQDEAYERGTPININLYDHARGTATGNTRYNDG
Q99N05	MLEFCIAVSVSAFGCKASCCNSSEVLVVLPSNSAVTVTAPP

Q5VWW1	GEKGEPGRQGLPGPPGAPGLNAAGAISAATYSTVPKIAFYA
P36542	FNKFRSVISYKTEEKPIFSLNTVASADSMSIYDDIDADVLQ
Q95IE3	RWFRNGQEEKTGVVSTGLIHNGDWTFQTLVMLETVPRSGEV
P30280	VDPVRRAVPDRNLLEDRVLQNLLTIEERYLPQCSYFKCVQK
Q2YDG7	QDPSLAREGEGEPEGDEEPENDSETEKEPQAEAEDDSEGIG
Q3T0B6	KEVSFQATGESDWKDTNYTLNTDSLDWGLYDHLMDFLADRG
Q9D753	VKAEFAAPPVDAPDRGYVVPNVDLPPLCSSRFRTGPPGEEA
Q9JKK0	SPPASPPVGWKQSEDAMPVINYDLLCAVSKLGPGEKYELHA
Q8NCW5	STVVKYLSQEEAQAVDQELFNEYQFSVDQLMELAGLSCATA
Q8BGF6	KTDFRGMGILGLINLVYFSENYTSEAHQILSRSNHPKLGYS
P56856	VSIFALKCIRIGSMEDSAKANMTLTSGIMFIVSGLCAIAGV
Q9D7L8	NHTEDEELLWYREDGIVDLKNGNKINISSVCVSPINESDNG
Q9NVK5	ASRHILEAPQHGLERRHLEANQNELQAHVDQITEMAAVMRK
Q58CX6	RVEPHQLAIDRPSQKLLKFLNKHYNLETTVPQVNNFVIFEG
P16015	PMTVSSDQMAKLRSLFSSAENEPPVPLVGNWRPPQPVKGRV
Q9H008	DTSNPNCVVIADAGESFSYQNMNNAFQVLMELEKPVLISLG
O14893	WKKFCLGEKLCADGAVGPATNESPGIDYVQIGFPPLLSIVS
Q15014	NVDAILEEYANCKKSQGNVDNKEYAVNEVVAGIKEYFNVML
P0C7V0	SHQGWKNTRCATRGLVNTLVNTGHFLYLQPPAPLIMPYLDD
Q9D8B3	TIEFQREALENANTNTEVLKNMGYAAKAMKAAHDNMDIDKV
P12980	AFAELRKLLPTHPPDRKLSKNEVLRLAMKYIGFLVRLLRDQ
Q6P8M1	IETDAPWCGVKSTHAGSKYINTSFPTKKKWENGHCLKDRNE
P09586	KLSLSQPCSFSGALLLLAVSNLLVWEKVTSLPNYRLPTESL
Q8BGU2	GAVLALLLLLLPACCPVRAQNDTEPIVLEGKCLVVCDSSPS
Q9DCH6	GNPRTNGMCSVCYKEHLQRQNSSNGRISPPAASVSSLSESL
O43692	MIAILDYHNQVRGKVFPPAANMEYMVWDENLAKSAEAWAAT
P57776	SGPGGDHSELIVRITSLEVENQNLRGVVQDLQQAISKLEAR
P97805	EIPDSTLVLVASYDDPGTKMNDKIKTLFSNLGSSYAKQLGF
Q8N8I0	EGIKLQIEGEGVESQSIKNKNFQKVPDQKGTPKRLQAEAET
A4IFN5	LGFLTFIWVWMFLCTAVYFHNLSQKVFGTLFGLLGWYGTYG
Q9CQE8	LAVRLEYGDNAEKYKDLVPDNRKNTDNAAKNAEPLINLDVN
Q7Z7J5	CSRKRKAVTKRARLQRSYEMNERAEETNTVEVITSAPGAML
Q9BQ13	IFGEQVSRKQFLLQVPGYSENLELMVRLARAEAITARKSSV
Q05433	ADQTRYIFRGDKDALTITVTNNDKERTFGGQAWVDNIVEKD
Q9NUN7	WCEENYSVTWYIAEFWNTVSNLIMIIPPMFGAVQSVRDGLE
Q9Z2X2	AASAGRDEIVKALLVKGAHVNAVNQNGCTPLHYAASKNRHE
O43482	HFCLSSDKMVCYLLKTKAIVNASEMDIQNVPLSEKIAELKE
Q96IL0	RFCPPRKSCHDWIGPPDKYSNLRPVHFYIPENESPLEQKLR
Q13268	PGIIKTDFSKVFHGNESLWKNFKEHHQLQRIGESEDCAGIV
Q8N7C4	QKHLGNGSCTEITPKYRGASNIINNFIICWSFKIVLFLSFI
P08311	FVLTAAHCWGSNINVTLGAHNIQRRENTQQHITARRAIRHP

P09023	RIEIAHALCLTERQIKIWFQNRRMKWKKESKLLSASQLSAE
B1ATL7	CRGKLAHVSFPLRGPCHPMHNWPRPIPLSSSTPGLPSCSTV
Q8VDV8	SSGLEEIKQSLGSHGVVLEINYSSSIHDREIRFNNGWMIKI
Q6P7N7	LVKNLRLVKRLYFGLRVLPPNLVNLNFHQSLTEDQKLIDEG
Q32L47	SMIPIHLLLIMNKASSEFEENLHRFQEAAKLFQGRRQNHEA
A5D7C9	LVALCLASFGMTLLGNFQLTNDEEIHNVGTSLTFGFGTLTC
Q91WE2	IEKQRREEELEELKEYRSNLNKVGISAENKEVEKKLAVKPI
Q9DAP7	RVTRFHINWDNNPDSLEAIENQDPNVDFSLSLSCTPVKSLG
Q9CQE1	LRTAHSEMIGYWTVEFGGRTNRVFHIWKYDNFAHRTAVRKA
Q645M6	HSVSAGLSSSDLTQLKFLCQNHISKRKLELAQSGLDLFTVL
Q9WV55	YDPNEKSKHKFMVQTIFAPPNISDMEAVWKEAKPDELMDSK
Q8BS03	YTQMVWATSNRIGCAIHTCQNMNVWGSVWRRAVYLVCNYAP
Q8BWR2	NIPQMSFDDTEREPEQTFSLNRDITGELEYATKISRFSNVY
O95865	DGVRKALQDLGLRIVEIGDENATLDGTDVLFTGREFFVGLS
P10629	YDYGSNSFYQEKDMLSNCRQNTLGHNTQTSIAQDFSSEQGR
Q9WTL2	RWLKELYDHAEATIVVMLVGNKSDLSQAREVPTEEACMFAE
P61588	ALQSENSVRDIFHVATLACVNKTNKNVKRNKSQRATKRISH
A2AFE9	RHPLTLTPAIEEESLATAEINSSEGLESQSQKGHDSINMSQ
D3Z690	INTWHKQDEKMRTTPAFLSQNIKCLEPQHLVNLTLEKVSEC
Q08DD7	CVQGRSPVSVSDAILRYAMSNCGWAQEERRGSSHLAEGDQP
O35164	KRECTQQKIKVEKYILPPNYNVSSKFNDIVLLKLKKQANLT
Q28035	DIHLVELLYYVEELDPSLLANFPLLKALKARVSNIPAVKKF
Q9JJT2	RCLRVYAGLIGTVVTPNYLDNVSARVAPWCGCAASGNRREE
Q08DC7	ADQPPSMSSHDAAPPAPPRRNPCCLCWCCCCSCSWNEERRR
O35949	IFVHWYHHSTVLLFTSFGYKNKVPSGGWFMTMNFGVHSVMY
P62753	DTTVPRRLGPKRASRIRKLFNLSKEDDVRQYVVRKPLNKEG
Q9P126	VGLVALGIWSVMQRNYLQGENENRTGTLQQLAKRFCQYVVK
Q64368	ESQINFHGKKLKLGPAIRKQNLCTYHVQPRPLIFNPPPPPQ
Q13156	RDTTVESVPVSPSEVNDAGDNDESHRNFIQDEVLRLIHECP
Q86UF1	CGGISYKDWSQNMYFNCSEDNPSRERCSVPYSCCLPTPDQA
Q9NUM4	ENITAQVQFSKTVIGKARLNNITIIGPLDMKQIDYTVPTVI
P20339	AKTSMNVNEIFMAIAKKLPKNEPQNPGANSARGRGVDLTEP
P63032	RKVYNIHGDMYQLDILDTSGNHPFPAMRRLSILTGDVFILV
Q2NL33	LFSAYEYAQINESKGCAYFQNGNIYISRCSAEISWICEKTA
Q921K9	FPSDASANSSLLLEFQDENSNQSSVSDVYQLKVDSSTNSSP
Q9NX20	LHWGHFEMMRLTINRSMDPKNMFAIWRVPAPFKPITRKSVG
O00212	FADGAFPESYTPTVFERYMVNLQVKGKPVHLHIWDTAGQDD
Q9XSK7	YPHWPARIDEMPEAAVKSTANKYQVFFFGTHETAFLGPKDL
A5PJG7	YVQCPITGAKAANKENLDPSNLMPPPNQTPAPDQPFPLSTV
Q14201	FPPLPMWHPLPRKKPGMYRGNGHQNHYPPPVPFGYPNQGRK
Q3LUH2	QWIDQTPYKENVRFWHQNEPNFSAEECASVVFWDGRGWGWN

A4FV54	IEEHASADVEKMILGNKCDVNDKRQVSKERGEKLALDYGIK
Q9D0E3	DEIGSSSGRRKNRGSGSGRPNGTGLPPHQETSTPSHDLSAS
Q2LGB5	GALGTVGRLSVTVVQAKLAKNYGMTRMDPYCRLRLGYAVYE
Q00169	WGLQNKVENFIHKQERRLFTNFHRQLFCWLDKWVDLTMDDI
Q9GZN7	VYINLNKLCLTVYQLHALQPNSTKNFRPAGGAVLHSPGAMF
Q8BS03	KARRKRYISQNDMIAILDYHNQVRGKVFPPAANMEYMVWDE
Q8CGZ9	PLKSPKTEKEAKRISLPDFMNMILSILRAWDNPLHHMETEL
A6NLW8	REELAKEIGVPESRVQIWFQNRRSRLLLQRKREPVASLEQE
Q1JQA4	VQNVIYVRGCTNAVLMWFTDNYTIMAGVLLGILLPQFLGVL
A6PWV3	KDEFYRLGNETWKGVFIIQGNLLEIRQNLEKKLTQIIKNFS
Q9QZ28	LGRFLWSLPVAPAACEALNKNESVLRARAIVAFHGGNYREL
P25402	VQEIPPAPKGDGGSLSLAINNRVKLIYRPIALKNGRDEAEN
O08584	ESELKISSSPPEDSLISSSFNYNLETNSLNSDVSSESSDSS
Q9R0P9	SVPSPACALLLLFPLTAQHENFRKKQIEELKGQEVSPKVYF
Q92522	YSIKALVQNDTLLQVKGTGANGSFKLNRKKLEGGGERRGAP
Q9MZ13	FGIAAKYKLDCRTSLSAKVNNASLIGLGYTQTLRPGVKLTL
Q56P03	LRYKASENRKKRRVHKKMRSNREDAAEKAETDVEEIYHPVM
Q99967	PQHAFNALMGEHIHYGAGNMNATSGIRHAMGPGTVNGGHPP
Q8K015	AAAHLQTDVQRFLFRLWEYLNAYAGRKYQADQLESDFCDVL
Q8ND25	DSERAPGGGGSASDSTYAHGNGYQETGGGHHRDGMLYLGSR
P50135	VVEPSAEQIAKYKELVAKTSNLENVKFAWHKETSSEYQSRM
P51157	IDLEHMRTIKPEKHLRFCQENGFSSHFVSAKTGDSVFLCFQ
Q9NRH1	GDVFDEEADESLLAQREWQSNMQRRVKEGYRDGIDAGKAVT
Q13145	AGGLILVLLIMLALRMLRSENKRLQDQRQQMLSRLHYSFHG
O35326	KDFMRQAGEVTFADAHRPKLNEGVVEFASYGDLKNAIEKLS
P43528	PNSCLPNNKASSDTTCASLTNKLSQHDLADFKKYIKRKFTL
Q9BRV8	YKPHILLSQENTQIRDLQQENRELWISLEEHQDALELIMSK
Q6UWN0	HNQLLADFHHIGSIKVTEVLNILEKSQIVGAASSRQDPAWG
Q8WU03	DNKEGNFSNMFLDASHAGLVNEHWAFGKNERSLKYIERCLQ
Q96BX8	TALSAPRYMDLLMDWIEAQINNEDLFPTNVGTPFPKNFLQT
Q9WTP6	ATMDAGKLVSDEMVVELIEKNLETPSCKNGFLLDGFPRTVR
Q9CY45	IFEYDRRFAIYGDEFIFYDYNHPLELPERIAAHSFDLVVAD
Q2YFS2	GVLRILNLKESDQAQYFSRVNLQSTEGMKLWQSIPGTQLNV
Q99436	KNLVSEAIAAGIFNDLGSGSNIDLCVISKNKLDFLRPYTVP
Q9D1C9	HRVRQGTQSTWPPDRTLFILNVPPYCTQESLSRCLSCCGTI
P31955	QNFCIHGECRYIENLEVVTCNCHQDYFGERCGEKSMKTHSE
O95744	TVSVKQLFSTLPVRHKEFQRNIKKKRACFPFAFCRDCQFLE
Q9CPX5	SKKRTELIPTSNSEIESTQKNQAVEGNPRPRPGDLIEIFRI
O75323	PNIYELRSYQLRPGTMIEWGNYWARAIRFRQDGNEAVGGFF
P43430	GNPSENKATGKGDSGGPFVCNGVVQGIVSCRLCTGTLPRVF
Q03255	PRRRAKRPQKSLPADSSILCNPMHVETLHPFTYRDGCAKTT

P09611	PGNCRIPLQSLFERATLVASNNYRLAREMFNEFNKQFGEGK
P16562	AFTALLTTQLQVQREIVNKHNELRKAVSPPASNMLKMEWSR
P31947	TNPIRLGLALNFSVFHYEIANSPEEAISLAKTTFDEAMADL
P04973	PPGIPDAVDGVTNGDYYQESNGPTDSYAAISQVDRLQSEPE
P70677	VDAANLRETFMGLKYQVRNKNDLTREDILELMDSVSKEDHS
Q8BSA9	QHALKRETISNSHSKIKERNNPLCTHKNRRSQGKAQGPSTT
Q92520	LLTFYVISQVFEIKMDASLGNLFARSALDTAARSTKPPRYK
Q9JII2	QPHQSGTLLLLLLSNLLMWENVASVPRCIMEDGGCQKVLNY
P01246	LPWTQVVGAFPAMSLSGLFANAVLRAQHLHQLAADTFKEFE
Q14847	YDYSAADEDEVSFQDGDTIVNVQQIDDGWMYGTVERTGDTG
Q5VWC8	RLCQSVSLLELLHIYVGIESNHLLPRFLQLTERIIILFVVI
Q1ZZU3	VSEGYSVDELEDHITQLHEYNDIKDVGQMLMGKLAVIRGVT
Q96B77	QEDPKRWVGSSSPISKEKLPNAETEKFWMFYRFDAIRTFGF
Q3UC65	SEKQTEDATKNTSEKSSTQRNIAFSSNNSVAKPLQKTTKAA
O35565	KLYGSKEFNNDCKLKERIEENGYNTYASFNWQHNGRQMYVA
Q9BYD2	SVENVGVRGDLVSVKKSLGRNRLLPQGLAVYASPENKKLFE
Q8BIG7	GKRWPPWGSRRQERLLPPEDNPLWQYLLSRSMREHPALRSL
Q9Z260	NIVVFENRWEGLWMNCMRHANIRMQCKVYDSLLALSPDLQA
Q3ZBR0	PLLTQTRLITQSALHRAPHYNSCCRRKYRPEAPRPSVSRPP
Q9UI95	IFQKRKKYNVPVQMSCHPELNQYIQDTLHCVKPLLEKNDVE
Q9D0B0	EKDLEDLFYKYGRIREIELKNRHGLVPFAFVRFEDPRDAED
Q3T0C2	VGFTRSAAMAANLMNSGVRLNAICPGFVDTPILKSIEKEEN
P0C7Q3	YLAGKVEEQPLWAHDIISVSNRYFNPSSEPLGLDSRLWELR
Q9ES30	RGYQGPPGPPGPPGIPGNHGNNGNNGATGHEGAKGEKGDKG
Q50H32	AIDGLSDSEGQQNGHIGSEDNEQEKDQDNLLVLARTASEKA
Q9D7I0	CCGSCSNQYCCSDVLRKIQWNEEMCPEPESRFSTPAEETPE
Q7SIB2	GTAGSCLRKFSTMPFLFCNINNVCNFASRNDYSYWLSTPEP
Q2HJI8	AMGIMLVYDITNEKSFDNIKNWIRNIEEHASSDVERMILGN
Q2KIR7	RKSLPMSLKVYGTVMHMNHGNPFNLKALVDKWPDFQTVVIR
Q3LI81	KSKNFETLERASSQCQCQSQNPESSSCRPLVNVAPEPQLLE
Q04744	RKQLAHSLSLTETQVKVWFQNRRTKFKRQKLEEEGSDSQQK
P43528	PININLYDHARGTATGNTRYNDGYVSTTTTLRQAHFLGQNM
Q8HZJ5	FRDAMGFLLMFDLTSQQSFLNVRNWMSQLQANAYCENPDIV
Q14AM7	GLHDVDLNTFITEEMALHLDNGGTRKRTSAQCGDVITDSPT
Q6ZWV3	FKFPGRQKIHISKKWGFTKFNADEFEDMVAEKRLIPDGCGV
Q9BUV0	DGTRNPNEKPTQQRSIAFSSNNSVAKPIQKSAKAATEEASS
P30042	GFGAAKNLSTFAVDGKDCKVNKEVERVLKEFHQAGKPIGLC
Q8BHL8	LRYESKDGARKLLLKAVSVENGMIINVLELGTQQVADLTLN
Q9Z2U0	SGGKNIELAVMRRDQPLKILNPEEIEKYVAEIEKEKEENEK
P10628	KQPAVVYPWMKKVHVNSVNPNYTGGEPKRSRTAYTRQQVLE
Q64362	FIRHGLYQDGVFKFTVYIPDNYPDGDCPRLLFDIPVFHPLV

Q9BQ24	FLVTFGNSEKPETMTCRLSNNQRYLFLDGDSHYEIEIVHIS
A2A5I3	GHTVGALRMPNTTLVQGRPKNTAVWPLSGLGVPRHRRKRHI
Q9D112	SLSSLESHPFLSSSTTDPDSNSLNTEQKGSWDSENFWLDPS
Q9NVZ3	TAVESVTDSSRYFVIRIEDGNGRRAFIGIGFGDRGDAFDFN
Q9Y696	ADCNLLPKLHIVKVVAKKYRNFDIPKEMTGIWRYLTNAYSR
Q8N0W7	RAMRVAHLELATYELAATESNPESSHPGYEAAMADRPQPGW
Q5U649	LTELFSRSMNTQILLMAVKNNSYIKDFFEQMLKIFKEMQSV
Q9H2P9	VLRATKLGIPYRVIHNASIMNAVGCCGLQLYKFGETVSIVF
Q1JQE1	NGTVTGKGRGSHIVGLLTTLNFGDGPDRNKTQTFQAQVQGS
O70514	VCRKRFPESNLKLVNPNARGNTKPRKEKAEVSAREHNKVQE
O14813	SPLRPAFPAAGPPCPALGSSNCALGALRDHQPAPYSAVPYK
Q9D5L7	NNHESWGWDLGRNRLYHDGKNQPSKTYPAFLEPDETFIVPD
Q8BKE5	MIPQIASFGLIRDRSHIQSANLDETAQAGTSSLQVMVDHHP
Q1LZ83	GHGPVIHNAEAKILQYISHRNIREQQILTVFHENFEKSFTA
Q5E9B7	LCPGGQLPFLLYGTEVHTDTNKIEEFLEAVLCPPRYPKLAA
O43704	SPRIVKTHLPTDLLPKSFWENNCKMIYLARNAKDVSVSYYH
P01582	HETCTDQFVSLRTSETSKMSNFTFKESRVTVSATSSNGKIL
Q99N03	VSRWQNLGPAQPAQKVAQPQNLVPDGHLEKALEGSDLLQKL
P22794	IIAVLFLICTFLFLSTVVLANKVSSLRRSKQVGKRQPRSNG
P84751	WFRQPPGKALEWLGLIRNKGNGYTMEYSASLKGRFTISRDN
O75629	PEMKTWPSSHNWFFAKLNITNIWVLDYFGGPKIVTPEEYYN
Q8C7V8	AFFGDVVLRFPKIVHHYFDHNSNWNLLIRWGISFCNQTGVF
Q3SZM1	REWHMPFKRPSYPAVKRYNQNRSLVQKLRMEERFKKKEKLL
Q9D0V8	KIQMKMEKLSSTTKGICELENYHYREESSRPPLFHTWPTAF
P17981	SPKRKKSNFNCFYSGSTQAANVSSLGSRESCVSLFLVVLLG
Q5JUR7	FRLKEPVLFVSGSADEMCEKNLLEKVAQKMQAPHKIHWIEK
Q8CHQ9	TLYSFLFLLCLWLIFWISCRNYVAKSLQADLADITKSYLNA
Q32LD7	MEATAAASLSYSSVDETQVQNLYVSCKSSGKVISSVYSRES
P00847	VTLIVLFPSLLFPTSNRLVSNRFVTLQQWMLQLVSKQMMSI
Q9BR61	LYHEETIREEDKNIFDYCRENNIDHITKAIKSKNVDVNVKD
Q9H467	PRGTIGDMMQKLSGQLSDARNKENLQPQSSGVQGQVPISPE
P22692	ERLAASQSRTHEDLYIIPIPNCDRNGNFHPKQCHPALDGQR
P11226	TCEDAQKTCPAVIACSSPGINGFPGKDGRDGTKGEKGEPGQ
Q9UPY8	KMGVDKIIPVEKLVKGKFQDNFEFIQWFKKFFDANYDGKDY
Q96CJ1	ECILIINHDTGECRLEKLSSNITVKKTRVEGSSKIQYRKEQ
P26838	MAGNQGHRYDWVSSFPFFYMNEEPAFAKSVDAFQRAGDTVI
P33782	SAVDNGQVITQGVLADGRVMNLAAAYASAVSDFELRLPAEG
Q58DM8	GEKVFAAGADIKEMQSLTFQNCYSGGFLSHWDQLTRVKKPV
P0C646	GLHTWISIPFSFMYIVAVAGNIFLIFLIMTERSLHEPMYLF
Q3KQJ0	TVRLGAGRIDILSLKTCLLQNFSSLPPRTWLSPSFQVCMRK
Q6RXL1	VEGPTAGDFYYLDGAPVNYTNWYPGEPRGRGKEKCVEIYTD

Q13268	AVILVSSIAAYNPVVALGVYNVSKTALLGLTRTLALELAPK
Q6P1S2	KESYYEKLKRTYEIWKDNMNNCSLILKFRELISRINFRRKG
A8MXK1	KPGTQANISQSHKDRVCTFDNGSIQLFSVGVRDSGYYVITV
Q8BVF2	LARKFPDVKFIKAISTTCIPNYPDRNLPTVFVYREGDIKAQ
Q86YI8	IECNECHTWIHLSCAKIRKSNVPEVFVCQKCRDSKFDIRRS
Q5E943	YEPQLLADDDARLLQLETQGNHNCYNYLYRMKALDAIRASE
O08734	ETQGAAAPANPEMDNLPLEPNSILGQVGRQLALIGDDINRR
Q08331	GSGYIDENELDALLKDLYEKNKKEMNIQQLTTYRKSVMSLA
P52803	AIPDNGRRSCLKLKVFVRPTNSCMKTIGVHDRVFDVNDKVE
Q9D270	GRLPENPKIPHAERELWELCNKCNLMRPKRSHHCSRCGHCV
Q9JHL0	RQVWPGPQMDTAPNKSFERKNKMLFSHLEGPESPRYQNFYK
P27701	FAFLLLILIAQVTAGALFYFNMGKLKQEMGGIVTELIRDYN
Q9Y6N3	LKSSLVTLNNNGYDGIVIAINPSVPEDEKLIQNIKEMVTEA
Q6PZD9	DKAKRRIMETQQKVLEYMAENERLRNRVDQLTQELDTLRNL
Q3ZBE1	SESGEEMLVPFQDTPVEVEQNTELPDYLPEDESPTKEQDKA
Q2NL01	MVLCTVMLFLLQLKFLKPKINSFYTFEVKDANGRVVSLEKF
Q9BTE7	VFYQYLEQSKYRVMNKDQWYNVLEFSRTVHADLSNYDEDGA
Q9QY31	HTGEKPYTCSHCSRAFADRSNLRAHLQTHVGTKKYRCAVCP
P20160	QSRQTFSISSMSENGYDPQQNLNDLMLLQLDREANLTSSVT
A6H7I7	LPRPDSLIFYRQKRDCKASVNKENAKGQGLVRRLFLGSPRD
Q2TBQ5	GKKVAPAPAVVKKQEAKKVVNPLFEKRPKNFGIGQDIQPKR
O14798	KSSCTMTRDTVCQCKEGTFRNENSPEMCRKCSRCPSGEVQV
P06730	IEPMWEDEKNKRGGRWLITLNKQQRRSDLDRFWLETLLCLI
Q58CY6	ALGKPVREVAAKAKAVGIQGNLSGDLLQSGGLLVVAKGGDK
Q2KIR7	LFGGNERSLRFIERCIQSFPNFCLLGTEGTPVSWSLMDQTG
Q9QZL9	DFRDLPRNFHQEENQEHRMGNHTLSSHLQIDKVTDNQTGEV
P43464	RMSKAALLLLDNSYQISQISNMIGFSSTSYFIRLFVKHFGI
P30301	EIFLTLQFVLCIFATYDERRNGQLGSVALAVGFSLALGHLF
Q6F5E0	AHGGATDAPGLAGTPPNASANASFTNEHSTPRLLASAASAP
Q9ET43	LPLSIVIAMGALLLCLIGMCNTAFNSSVPNIKLAKCLVNSA
Q9NWZ8	SCFNLPWYLPSALLPQSSYDNEAAYPQSFYDHHVAWQDYPC
Q9HD33	LRNKSNEDLHKLWYVLLKERNMLLTLEQEAKRQRLPMPSPE
Q9DB98	SGPVDLFRELLEERKGVPRGNKEHEEEKRREKERQEKALGI
Q6ZUL3	LLWGCLMPRAQPLLHVTAYENTGHWERLASVVSSKTQQPTV
P08831	ANNTEEEIIKPRSAHYSFQSNVKYNFMRVIHQECILNDALN
Q9UPY8	KSKKLIGTAVPQRTSPTGPKNMQTSGRLSNVAPPCILRKNP
Q9CY21	LITTQATRAGFTGGVVVDFPNSAKAKKFYLCLFSGPSTSLP
P45879	IYQKVNKKLETAVNLAWTAGNSNTRFGIAAKYQIDPDACFS
Q4JM65	NQTWNNPTWSNQSWNSQSWSNHSWNSQAWCPQAWNNQPWNN
P24855	LVQEVRDSHLTAVGKLLDNLNQDAPDTYHYVVSEPLGRNSY
P97361	PLPLGQGPPLPLNQGPPLPLNQGQLLPLAQGLPLAVSPALP

P05305	CSSLMDKECVYFCHLDIIWVNTPEHVVPYGLGSPRSKRALE
Q9P013	RNRDFRRELEERERAAAREKNRDRPTREHTTSSSVSKKPRL
O88513	DLAEVAEHVQYMAEVIERLSNEPLDNFESPDSQEFDSEEEA
Q4VC05	DTSLRIYKWVPVTEPKVDDKNKNKKKGKDEKCGSEVTTPEN
Q8ND94	GLEGADIPAFGPCSRLAVPPNPRTLVHAAVGVGTALALLSC
Q9QZ06	VAPQPRCNEEDLKAIQDMFPNMDREVIRSVLEAQRGNKDAA
Q8K1A0	NRMSKLFDTVIMNPPFGTKNNKGTDMAFLKTALGMARTAVY
P02747	KFTCKVPGLYYFVYHASHTANLCVLLYRSGVKVVTFCGHTS
P62820	QESFNNVKQWLQEIDRYASENVNKLLVGNKCDLTTKKVVDY
Q9QZZ6	GYGYPYQQYQDYGDDGWVNLNRQGFSYQCPHGQVVVAVRSI
Q16635	ASSVVMGLVGTYSCFWTKYMNHLTVHNREVLYELIEKRGPA
Q7RTU3	DLQQLRLKINGRERKRMHDLNLAMDGLREVMPYAHGPSVRK
Q1LZC5	ADPNQRDGLGNTPLHLAACTNHAPVITTLLRGGARVDALDR
Q3UA16	ILEYKNQLCKQNKLIQEKKENVLKMIAEVKGKEQESEELTA
P52803	SQDPGSKAVADRYAVYWNSSNPRFQRGDYHIDVCINDYLDV
Q9BVC5	LSNSSSSVSPLILSSNLPVNNKTEHNNNDAKQNHDLTHRKS
P25325	AFGHHAVSLLDGGLRHWLRQNLPLSSGKSQPAPAEFRAQLD
Q3UPL6	PFWGGACFIISGSLSVAAERNHTSCLLKSSLGTNILSAMAA
Q9D7S0	LNCTQCYTYNSTCDGQATECNEQSFSCVESSINSTLGGFLH
Q15363	TAVKHEQEYMEVRERIHRAINDNTNSRVVLWSFFEALVLVA
Q60651	HHNNCSIMQSDINLKDELLKNKSIECNLLESLNRDQNILCD
Q16611	NRRYDSEFQTMLQHLQPTAENAYEYFTKIATSLFESGINWG
Q9JJE4	GGSPVYTRLLALDMCGVCLVNTLGALPIIHCTLACRPWLRP
P09067	SYFVNSFSGRYPNGPDYQLLNYGSGSSLSGSYRDPAAMHTG
P56880	SGVLTATLLPNWKVNVDVDSNIITAIVQLHGLWMDCTWYST
O95567	INVRRDPSIPIYGLRQSILLNTRLQDCYVDSPALTNIWMAR
A1E959	SAPLIPQRLMSASNSNELLLNLNNGQLLPLQLQGPLNSWIP
Q9NQY0	TDADLAMSKSAVKISLDLLSNPLCEQDQDLLNMVTALDTAM
P41976	NAQIMQLHHSKHHAAYVNNLNVAEEKYREALEKGDVTAQIA
P10746	GLIFTSPRAVEAAELCLEQNNKTEVWERSLKEKWNAKSVYV
P35242	LQGSMLSVGDKVFSTNGQSVNFDTIREMCTRAGGHIAAPRN
Q99JZ0	DNMVVTSPGPGQVVAPVSGNNLGILRAEIKPGVREIHLCKD
Q1RML1	GYFLTKIFHPNVGANGEICVNVLKRDWTAELGIRHVLLTIK
A0JN87	GLTEAIVVNPFEVVKVGLQANRNRFTEQPSTMSYARHIIKK
P08861	INSGDLFVHPLWNRSCVACGNDIALIKLSRSAQLGDAVQLA
Q58D45	VCPHCQQAITTKISYEIGLMNFVLGFFCCFMGCDLGCCLIP
Q4JM65	NLPMWGNQTWNNPTWSNQSWNSQSWSNHSWNSQAWCPQAWN
Q96A37	QMANCPKFVPVVPTSQPIPSNIPNRSTFACPYCGARNLDQQ
P29972	TTLFVFISIGSALGFKYPVGNNQTAVQDNVKVSLAFGLSIA
Q9CPY6	AFYQYAKSFNSDDFDYEELKNGDYVFMRWKEQFLVPDHTIK
P56966	AIWSRPESTQVQNILRQRTENIDIKKYCVHYLENVGSFEYT

Q92914	PVGLRVVTIQSAKLGHYMAMNAEGLLYSSPHFTAECRFKEC
Q04743	RIRTAFSPSQLLRLEHAFEKNHYVVGAERKQLAHSLSLTET
P10833	GAMREQYMRAGNGFLLVFAINDRQSFNEVGKLFTQILRVKD
Q96LL9	TQAQIKAAYYRQCFLYHPDRNSGSAEAAERFTRISQAYVVL
Q3TZ65	EVTPGPGAYSPEKAPPVRQRNAPAFTLGSRLRQKPPDTSVP
Q0P5C7	QLCQSISLLELLHIYVGIESNHLLPRILQLTERIIVLFMVI
Q15560	SAQIEECIFRDVGNTDMKYKNRVRSRISNLKDAKNPDLRRN
P55064	AGAGILYGVAPLNARGNLAVNALNNNTTQGQAMVVELILTF
Q5KR47	RAELAESKCSELEEELKNVTNNLKSLEAQAEKYSQKEDKYE
A6QPN6	EQKIAFLTIVCLEEMDDMEQNLKPCLQIYAPKVSADSIMEC
Q8BPB0	AHIYHQHFDPVIQLQEEAHLNTSFKHFIFFVQEFNLIDRRE
Q96HS1	WKPEAVQYYEDGARIEAAFRNYIHRADARQEEDSYEIFICH
Q9ESJ7	VCRRLDYITQSLQQQGFQAENVTVTKNIRRVENAYHMEAEV
A2VDP0	EFLLLTLEQKNITVENDMRVNKDSLTDLYVQHAIPLPQRDL
P39905	IKRLKRSPDKQMAVLPRRERNRQAAAANPENSRGKGRRGQR
Q6P1S2	KKESYYEKLKRTYEIWKDNMNNCSLILKFRELISRINFRRK
Q3TBL6	GEQSEGEPGTAAGPHVFSSKNLALQAQKKILSKIASKTVAN
Q62193	NSQASAGRPSMSNPGMSESFNFSGNNFMPANRLTVVQNQVL
Q9BQI4	YNLLPHGVNFQDAIFPDTQENRRMFSSLFQFSNCSQGQQLA
P98066	VFTDPKQIFKSPGFPNEYEDNQICYWHIRLKYGQRIHLSFL
Q96PP4	SFMLKVTQYDQDKTLLIMTNNPPPCSITQQDKESASKYFSK
Q2KIV9	KPGTPGTKGEKGLPGLVSHLNENGEKGDPGFPGMPGKVGPK
P26373	TIGISVDPRRRNKSTESLQANVQRLKEYRSKLILFPRKPSA
Q8K3J1	GHLNGQSLHSSAVAATYKYVNKKEQESEVDMKSATDNAARI
O55101	LVSMVLALIVFSCIFGEGYTNIHTSDQLYCVFNQNEDACRY
Q2KJF9	EGLPNSQKAVKALGDQILFVNRPDKKKILFFNDKSCQFSVD
Q9CPR7	QVDDNQFCKVQERLAQLELENKELRELLSISSESLQVGKES
Q9CQS3	EESYGKYLRRESHQIGDAYSNSDKSLTELESKFKQGQEQDS
Q0VD59	GSSVASAPVSSFPRTSVTPSNQDICRICHCEGDDESPLITP
O70552	ASFKGRWENWELAQHVSCAVNRATGDCSSGTSSDEESCSRE
A1L3X0	TWWFGVKFAAGGLGTFHALLNTAVHVVMYSYYGLSALGPAY
Q8K4T5	NAFLSEFTYKTISILDVPETNILSYFPECFEFIEQAKLKDG
P57746	STTVIQNVNKAQVKIRAKKDNVAGVTLPVFEHYHEGTDSYE
O00625	YLLEGGSMAHEDFCGHTGKMNPGDLQWMTAGRGILHAEMPC
P12004	MSKILKCAGNEDIITLRAEDNADTLALVFEAPNQEKVSDYE
Q3T0L1	STALKKHLEKVDIKTSVLMDNMKQILNLNKLIMKSQQETWD
Q9Y2D1	QRKRAEGEALEGECQGLEARNRELKERAESVEREIQYVKDL
Q6BBL6	GVVFLLAGLLVMVPVSWTANNVIRDFYNPLVASGQKREMGA
Q3ZCD8	ASLRRQIIKLNRRLQLLEEENKERAKREMVMYSITVAFWLL
Q9BY27	VADQQSTLEKAGVAGFYVTTNPQELMLQMNLLELIRKLQQR
Q8N7N1	LAACREHKRAPEVYVAFTVRNPETCQLFTTELGRDGIRWEA

Q5HYI8	DVGGSVGSASSVKSTRAVFYNSVNGIIFVHDLTNKKSSQNL
Q9CQI7	MEQAAAAANKKPGQGTPNAANTQGTAAPNPQVPDYPPNYIL
P42574	RSSFVCVLLSHGEEGIIFGTNGPVDLKKITNFFRGDRCRSL
Q14002	YRVCIPWQGLLLTASLLTFWNLPNSAQTNIDVVPFNVAEGK
P20336	EESFNAVQDWSTQIKTYSWDNAQVLLVGNKCDMEDERVVSS
Q5E9C4	LNSKYRDTLPQLPKAFEQYLNLEDSRLLSHLKACCAVSTLP
Q96DN0	AAKLFQGKILFILVDSGMKENGKVISFFKLKESQLPALAIY
Q9D387	VENLSGLSTNPEKDIFVVRENGTTCLMAEFAAKFIVPYDVW
O14668	IFGLFIILLVIFLIWRCFLRNKTRRQTVTEGHIPFPQHLNI
Q96PU9	YSFRGAPMLLAENCSPGPRYNVNPKILRTGKDLGPAYSILG
Q9UKW6	SLQSSHLWEFVRDLLLSPEENCGILEWEDREQGIFRVVKSE
A1XBS5	IFAAYVRKTARLRDKADLLVNEINAYAATETPHLKLGLMNF
P61588	PETLDSVLKKWKGEIQEFCPNTKMLLVGCKSDLRTDVSTLV
Q60809	VWACNLDEEMKKIRQVIRKYNYVAMDTEFPGVVARPIGEFR
Q60932	RVTQSNFAVGYKTDEFQLHTNVNDGTEFGGSIYQKVNKKLE
A7Z070	RNKTRRQTVTESHIPFPQHLNIITTPPPPDEVFDNSGLSPG
Q6PID6	LEGCAEKSKQLKDEGASLAENKRYREAIQKWDEALQLTPND
Q2HJC9	LPYYWNVDTDLVSWLSPHDPNSVVTKSAKKLRSSNADAEEK
Q9D305	KLKSRNLLKTNNSFPPTGPCNLKLNGSQQVLLEHSYAFRNP
P59279	TAKEIYRKIQQGLFDVHNEANGIKIGPQQSITSSVGPCSPQ
Q3ZBW4	AVVISCAKDGVKFSASGELGNGNIKLSQTSNVDKEEEAVAI
Q8K305	LALRDAQWTFESAVQENVSFNGQAWEEAKEHGLMDSDIKVL
Q9CXV9	LQCDCTEKLQSRFDFLRSQLNDISSFKNIYRYAFDFARDKD
Q8N1Q1	LSIKYDPSSAKIISNSGHSFNVDFDDTENKSVLRGGPLTGS
P79124	CPNNPESISLTVLHRRPGLLNDVVDFGVNLVRQLVSSVVQH
P16114	EKIIESIYISSVSFFSDKVRNLIEKDLSRKWTLGIIADAFN
Q3UK78	SDPQIAICLDCLRNNGQSGDNVVKGLMKKFIRCSTRVTVGT
O60725	WYMCSLSLFHYSEYLVTAVNNPKSLSLDSFLLNHSLEYTVA
Q9DD18	KGNKPDFHLAMPTEQAESFYNSFLEQLRKSYRPELIRDGKF
Q2HJF2	QLNRECLLHLFSFLDKDSRKNLARTCPQLQDVFEDPALWPL
Q9H560	ANLHAIDNFRRTALMLAVQHNSSSIVSLLLQQNINIFSQDL
P0CI32	HAATGLIRQLAYELAPKVRVNGVGPCGMASDLRGPQALGQS
O35739	GDTWKDYCTLVTIAKSLLDLNKYRPIQTPSVCSDSLESPDE
Q3SWZ3	QQFVELINKCNLMQSEYREKNVERIRRQLKITNAGMVSDKE
P14415	HVQKLNKFLEPYNDSIQAQKNDVCRPGRYYEQPDNGVLNYP
Q3MHR2	YLEKLHYKHESWLLHRTLKTNFDYLQEVPILTLDVNEDFKD
P97436	TPTEPESDAHFETYLLDCEHNPGDLASAPQVTKQPQKRSRA
Q969T7	IRQMKVFHPNIHIVSNYMDFNEDGFLQGFKGQLIHTYNKNS
Q8TCQ1	NTRTPEISGDLADASQTSTLNEKSPGRSASRSSNISKASSP
Q9BTT0	GGLEVLAEKCPNLTYLNLSGNKIKDLSTVEALQNLKNLKSL
Q9BY14	PMFVREACPHQLLTQPRKTENGATCLPIPVWGLQLLLPLLL

Q91WG1	FLYVRSWLPCIFFAGGITMGNIGRQLAMYECKVIAEKSHQE
P23888	TPTGRHYVFKNLNIIFPKGYNIALIGQNGAGKSTLLRIIGG
P29692	KPATPAEDDEDDDIDLFGSDNEEEDKEAAQLREERLRQYAE
P35232	CRSRPRNVPVITGSKDLQNVNITLRILFRPVASQLPRIFTS
P01131	NEAIFSANRLTGSDISLMAENLLSPEDIVLFHNLTQPRGVN
Q96BD8	VINELLNKLELEIQYQEQTNNSLKELCESLEEDYKDIEHLK
P01589	MQPVDQASLPGHCREPPPWENEATERIYHFVVGQMVYYQCV
Q8R2N0	EGISQVRFGNQNGKRRTWRPNPQQAFRGSVRKGQGFAFRRK
Q5EAA4	ESLINDEDVFPTRVGVPFPKNFQQVCTKILTRLFRVFVHVY
Q9UBX3	LLGSVSGLAGGFVGTPADLVNVRMQNDVKLPQGQRRNYAHA
Q9QW08	SLACLAVEYPMVKFCKIKASNTGAGDRFSTDVLPTLLVYKG
Q9Z1P5	IDKIFQEENATTTRISTTMENETSFRNVTFTSAGDSSRNPS
O95661	TLLHKWASGNFRHEYLPTIENTYCQLLGCSHGVLSLHITDS
Q8K2W6	KRTIVRQGKQVVFRDEDSTGNDEDIMVDSDDDSWDLVTCFC
Q8BSU7	RKFEPGTQRFELHKRAQASLNAGLDLRLAVQLPPGEDLNDW
Q9NVV0	GWIVMIAIGWARGAGGTIITNFERLVKGDWKPEGDEWLKMS
Q8R107	QSVLRSSWDQVFAAFWQRYPNPYSKHVLTEDIVHREVTPDQ
P33781	KDITARSVKPYDFFIVPVSGNVTPGAPVTRDTSANINSVNA
Q5TFG8	QCMLAIKEGRPLPPPPPPSLNPDYIQRPYCMRRFNESAAER
Q06323	DEKKKGEDEDKGPPCGPVNCNEKIVVLLQRLKPEIKDVIEQ
O43657	FRHEIKNSFKNNYEKALKQYNSTGDYRSHAVDKIQNTLHCC
Q9H063	LDSDPFGEDGSLWSFNYFFYNKRLKRIVFFSCRSISGSTYT
O09044	TMLDEQGEQLNRIEEGMDQINKDMREAEKTLTELNKCCGLC
Q91WB4	MNHINKCVTDLLSKSGKTPSNEHAAVWVPDSEATVCMRCQK
Q9Z261	ACSWIGHQIVTDFYNPLTPMNVKYEFGPAIFIGWAGSALVL
O43759	VVSWLFSIVVFGSIVNEGYLNSASEGEEFCIYNRNPNACSY
P56371	AAGALLVYDITSRETYNALTNWLTDARMLASQNIVLILCGN
Q3TP92	TVIQYQTVRYDILPLSPLSRNRLAQVKRKILVLDLDETLIH
Q3ZBW7	RIGTNLPLKPCPRASFETLPNISDLCLRDVPPVPTLADIAW
P08962	FKCCGAANYTDWEKIPSMSKNRVPDSCCINVTVGCGINFNE
Q8CBG9	LVAVVVSLALTATLLYALLRNVQQNIHPENQELVRVLREQF
Q5FWC3	IVAASLTIVELSHFRSVSYRNYGQAKLGREVSRVLLCSYPL
P18468	LDEKRAAVDTYCRHNYEIFDNFLVPRRVEPTVTVYPTKTQP
Q3T165	IGKFGLALAVAGGVVNSALYNVDAGHRAVIFDRFRGVQDIV
Q7RTY3	HLQTPVTFSEYVQPICLPEPNFNLKVGTQCWVTGWSQVKQR
Q2TBT5	FAKMEEDGDFVGWALDVLSPNLISTSMLGRVKYNLNALSHD
O75841	FTPNLFLKQMLERYQNNSPPNNDDQWKNNGVTKTWDRLMLQ
P18468	RRVEPTVTVYPTKTQPLEHHNLLVCSVSDFYPGNIEVRWFR
Q96PG2	GLPSWQVLSPVQPWQTSAPQNTTQPKLLAPHQHEKSQKKSS
P41976	GWGWLGFNKEQGRLQIAACSNQDPLQGTTGLIPLLGIDVWE
P35278	AKTAMNVNEIFMAIAKKLPKNEPQNAAGAPGRTRGVDLQES

Q9NXJ5	LYQSHGRSAFVHVPPLGKPYNADQLGRALRAIIEEMLDLLE
O43482	QNVPLSEKIAELKEKIVLTHNRLKSLMKILSEVTPDQSKPE
P61006	RGEKLALDYGIKFMETSAKANINVENAFFTLARDIKAKMDK
P70396	HHPHAHPPTSNQSPASSYLENSASWYPSAASSINSHLPPPG
Q9BVX2	RDSITCLTCQGTGYIPTEQVNELVALIPHSDQRLRPQRTKQ
P11456	VVGAKGMEQFPHLAFWQDLGNLVADGCDFVCRSKPRNVPAA
Q3UZZ6	STYPKSGTTWVSEILDLIYNNGDAEKCKRDAIYKRVPFMEL
Q92637	VYNVLYYRNGKAFKFFHWNSNLTILKTNISHNGTYHCSGMG
Q8BJ83	CPRQRYFANCTVRDHIHCLGNRTFPKLLYCNWTGGYKWSTA
Q32LN3	FPETKFVKAIANSCIEHYHDNCLPTIFVYKNGQIEGKFIGI
Q3T0S3	KDFTQVWNSTMAGLKCCGFTNYTDFEGSPYVRKNGTFPPYC
A5D7U1	AEYYLKGNFSTLFKAKWMSPNSTDITTVKINIYTVNSLRNA
Q0VC80	LLPGPVTLVMERSEELNKDLNPFTPLVGIRIPDHAFMQDLA
Q9DCH6	TTQQPSEEQSKSLEKPKQKKNRCFMCRKKVGLTGFECRCGN
A6NDY0	TQAWLQTVSSDPEAQGWGAWNETKEILGPEGGEGKEEKEEE
O35723	GFGGGFASLDTGFTSFGSPGNSGLSSFSMSCGGGAAGNYKS
Q96KN4	IIHLHQGEIRQDSLYEAGAANVGRVVNSWYRYRPLVAELVV
P60191	QVLLFPESPQGKVLQVIVWGNYGRMERKQFMGVARVLLEEL
Q9MYP6	LLGTYTLTKLALPHLRKSRGNVINISSLVGAIGQSQAVPYV
P26884	TLDEGPPKYTKSVLKKGDKTNFPKKGDVVHCWYTGTLQDGT
Q9CY57	VVLKSTTKMSLNERFTNMLKNKQPMPVNIRASMQQQQQLAS
Q9DAU1	TETICKRLLDYSLHKERTGSNRFAKGMSETFETLHNLVHKG
P13972	QVATSFLREELDAMSLPSSMNKIQSVAIGDKLWLVQFIPLI
Q9UL40	KIVLNSIEQYQAHVSGFKHKNQSPKTVASSLGQIPMQRQPI
Q9CQN4	ILYSHVVKPVPAHPSSNSTLNQARNGGRHFSSTGLDRNSRV
Q5SSH8	TGPPSDQQDNPRHSNHGDLDNPNLEEYTGCPPLATTCSFPL
Q969G5	TTSNTLAQLLAKAERVSSHANAAQERAVRRAAQVQRLEANH
P51749	ASSKTTSVLQWAPKGYYTLSNNLVTLENGKQLAVKRQGFYY
Q9CQJ2	GQGCTAYDVAVNSNFYLRMQNSDFLRELVVTIAREGLEDKY
Q6PDY0	REEAARLAALVQRGRLMQEVNRQLQGHLGEIRELKQLNRRL
P63011	EESFNAVQDWSTQIKTYSWDNAQVLLVGNKCDMEDERVVSS
Q9BQ83	YLLYCLNPRYRGRVYVGFTVNTARRVQQHNGGRKKGGAWRT
Q60653	FLLLITVAVLAVKIFQYGQHNQEIHETLNYHHNCSNMQSDF
O89051	NKKLTAYLDLNLDKCYVIPLNTSIVMPPKNLLELLINIKAG
Q9D780	SIERAGMDVTYIWLSSQDSTNTSHEGSVLSTSWRPGDKAPS
P70280	CGGNFLEVTEQILAKIPSENNKLTYSHGNYLFHYICQDRIV
Q46668	GDSNSFGELRNWQIMPGTRPNTIQFRNVDVGTCMTSFPGFK
Q0VD86	SGAASEPVVLGEEGCGFPSTNEYPDLEEERATYPQEEDRFL
Q86WA6	VLLLPGMLGSGETDFGPQLKNLNKKLFTVVAWDPRGYGHSR
Q3T0D3	CVYNSCYCEENIWKLCEYIKNHDQYPLEECYAVFISNERKM
Q9D061	SPSQAMQEYIAAVKKLDPGWNPQVSEKKGKEGSSGFGGPVV

Q9NPC6	LYPKLFKPEGKAELPDYRSFNRVATPFGGFEKASRMVKFKV
P61981	THPIRLGLALNYSVFYYEIQNAPEQACHLAKTAFDDAIAEL
Q9H560	RRQQIVEFLLKNQANLHAIDNFRRTALMLAVQHNSSSIVSL
P09488	YEEKKYTMGDAPDYDRSQWLNEKFKLGLDFPNLPYLIDGAH
Q91VX5	LVTGKIVDHGNGTFSVHFRHNATGQGNISISLVPPSKAVEF
Q8QZV2	PTRSQTNCCLPEGTMNNVYVNMPTNFSVLNCQQATQIVPHQ
O70552	HPDCPSKGQAFRCIRINNNENKDPVLERACAESNVNFFHLG
Q922H7	QPSSTPEKRRTSLIPRPKSPNMQDLKRRFKQALSAKVRTVT
A5PLL7	RHDFIETNGDNCLVTLLPLLNMAYKFRTHSPEALEQLYPWE
P62917	AYHKYKAKRNCWPRVRGVAMNPVEHPFGGGNHQHIGKPSTI
P05529	PLLEINSLSFSYKVNLPPVFNNLSLKIEQGELIGLLGENPA
O35425	CTVLLRLGDELEQIRPSVYRNVARQLHIPLQSEPVVTDAFL
Q9JKD3	RGSGGSFSKAQEEWTTGAWKNPHVQQAAQNAAMGAAQGAMN
Q8BR90	LVDGINYLLQMLNYRCPVQLNEGVSFQDLDTAKLLSTGVFS
Q9GZV9	GFVVITGVMSRRYLCMDFRGNIFGSHYFDPENCRFQHQTLE
Q04741	RIRTAFSPSQLLRLERAFEKNHYVVGAERKQLAGSLSLSET
O88552	FSLVAGVILCFSCSPQGNRTNYYDGYQAQPLATRSSPRSAQ
Q6PKH6	ALLGLNNTLAIELAPRNIRVNCLHLDLSRLASAGCSGWTRK
Q96JQ5	LMSLSMGITMMCMASNTYGSNPISVYIGYTIWGSVMFIISG
Q17QI5	LDKVMETQAQVDELKGIMVRNIDLVAQRGERLELLIDKTEN
O14494	PDWSKINCSDGYIEYYICRGNAERVKEGRLSFYSGHSSFSM
Q13277	LHDMFMDIAMLVENQGEMLDNIELNVMHTVDHVEKARDETK
Q8BW11	YLPCTGVLIHPLWVITAAHCNLPNLQVILGITNPADPMERD
Q3ZBK2	YGPQRRKMMFMGFVRLGVWQNFFRAWNGGFSGNLDGEGFIL
Q9DB15	GINLVQAKKLVESLPQEIKANVAKAEAEKIKAALEAVGGTV
Q5T0T0	PETSKKNIFEKSPLTEPNFENKHGYGICHSDTNSSCCTEPE
O94778	HGLALGLVIATLGNISGGHFNPAVSLAAMLIGGLNLVMLLP
P57052	SDLYQMTAPLPNSASVSSSLNHVPDLEAGPSSYKWTHQQPS
Q9CRA4	IRLLETIDVHSGYDIPLNPLNLVPFYTGARHHDFHHMNFIG
P62080	QLYFFINNNIRAYRDDIDLQNLIDFTQEYWQCCGAFGADDW
Q9Y5U5	PWTDCTQFGFLTVFPGNKTHNAVCVPGSPPAEPLGWLTVVL
Q9CWU6	DEGILSDDHGLAAALWRTFFNQKCEDPRQLELLVEYVRKQM
Q9CQ58	MQTAGTLRTLADQFYVELYHNQFSSGQFLIFNSNLIRRDET
Q99JT6	MMMKINNAQDLLLYKVNKYINLVMYFLFRLAPQAYLTKFFL
Q6Q7D1	AAPAGTRVRSSQSTRWLMQMNFLSYVQLTSSALPSLTDSKG
Q5E9J3	VQDTPGIQVHENGLSCTEQLNRCIRWADAVVIVFSITDYKS
Q3SZI4	MATCMKAVTEQGAELSNEERNLLSVAYKNVVGGRRSAWRVI
P14191	GSITADDYRQKWEWKVGTGLNGFGNVLNDLTNGGTKLTITV
P23709	KTYTDELTPIESSVPVYKAANWYEREIWDMFGVFFANHPDL
Q5T0T0	KVYVQLWKRLKAYNRVIYVQNCPETSKKNIFEKSPLTEPNF
Q8NEB5	DSRQACLAASLALALNGVFTNTIKLIVGRPRPDFFYRCFPD

Q9HBM6	DQSFTSPPPRDFLLDIARQKNQTPLPLIKPYAGPRLPPDRY
Q6UX82	FLSGENKTLGGVIFRKFECANVNSLTPTSAPTTSHNVGSKA
A6QL48	LTRSDECAITGFLRDKLQYRNRLQYMKHYFPINYRVSVPYE
Q13145	KKGQVAKLDLECMVPVSGHENCCLTCDKMRQADLSNDKILS
Q9CQA5	GRLPDVLAPQYPWQSNDMSVNMLPPNHSSDFLLEPPGHNKE
A6NNP5	NDELEKQIVYLKEKVEKIHGNSSDRLSSIRVYERMPVESLN
P13378	VQYPDCKSSSGNIGEDPDHLNQSSSPSQMFPWMRPQAAPGR
Q6UJB9	PALLRRLYLWVEHRTTLTSWNLAYLVTWTTCLASHLLQAAF
Q3MHM8	RKDPVLQPDGDGSPVLPDKRNGIFSADAGGKALARRWPVQV
Q969T9	VPSTPAAEAKAAEAAASAYYNPGNPHNVYMPTSQPPPPPYY
Q99P51	EKVHKYNSAVTDKLKMTLNSNGIYTGFIKVQMELCKPAQPS
Q6ZWI9	KVPALEEWQVSVLTLMTKQHNSRLEQSLHVREELRHFREDV
Q9CQX3	FRHGLLSGGILSFLEHIPLLNYVRPTGSNAGGLVGVLGKVI
Q969E2	QTEWNTGTWRNPPSREAQYNNFSGNSLPEYPTVPSYPGSGQ
Q15475	KNRRQRDRAAEAKERENTENNNSSSNKQNQLSPLEGGKPLM
Q8BX10	RATEPPAWTGARAGRGVWDTNWDRREPLSLINLKKRNVESG
P0C7W6	SKVQQFFEKSFFLQLLKAHENALEKQYSEITNHRNMLLQTF
Q32L08	LLHLCNCRKLKKLNLKSSKENRISITSKGIKAVASSCSYLH
P00642	GKRGDQDLMAAGNAIERSHKNISEIANFMLSESHFPYVLFL
Q96PB8	VTMFGWFTMVISYVVYYVRQNQEDARRHLEYLKSLPSRQKK
Q16878	VLRENQCAYINDSIGLHRVENISHTEPAVSLHLYSPPFDTC
Q96CE8	VTTVLLVLENNNNYKCCQSENCSKKYVTLLSIIFSSLGIAF
Q9QZ28	ERLGRFLWSLPVAPAACEALNKNESVLRARAIVAFHGGNYR
Q0VD50	AKVLRHHQEKLEASICDPQQNSPVLEKPGRKRKWAEQKQDI
Q5SZJ8	HSLTGAKSSTSRDKAVKPAMNQNEVQEIIGVTKQLFPNTDD
Q2TA24	YITGSSIIGGGVKAPRIKTKNLVSIIFCEAVAIYGIIMAIV
Q9ER00	ELIKERETAIRQLEADILDVNQIFKDLAMMIHDQGDLIDSI
P48307	LLLFLTEAALGDAAQEPTGNNAEICLLPLDYGPCRALLLRY
Q0VCT4	PSRNHTLPAVEVQSAIRMNRNRINNAFFLNDQTLEFLRIPS
Q9DAS2	ASSFGSSYGNTTFKYFQDLRNTGLEKSYALLSGGCFPTIFS
Q2HJH2	NVNKLLVGNKSDLTTKKVVDNTTAKEFADSLGIPFLETSAK
O75508	VVGFVTSFVGWIGVIVTTSTNDWVVTCGYTIPTCRKLDELG
Q9D753	LKENCRPDGRELGEFRATTVNIGSISTADGSALVKLGNTTV
Q924T2	VHLGHKAGCRHRFMEPYIFGNRLGQDIIDLDQTALNLQLAL
Q60660	TLLKTNDEDELKFLKSQLQRNTYWISLTHHKSKEESQQIGD
Q3T0X7	YALVNLATTSVSKMASDFAENELDLFRKALELIIDSDTGFA
Q9WTJ2	QSRDQGFLRTKMKVTLGDSPNGDLFTCHICQKSFTHQRMLN
Q91VU0	NVLMSGVKNNVGRGINIALVNGKTGEVIDTKFFDMWGGDVA
Q9BUA6	RDGFIDKEDLRDTFAALGRINVKNEELEAMVKEAPGPINFT
Q3KNM2	ILGKMIRWEDYVLRLWRKYSNKLQILNSIFPGIGCPVPRIP
Q077R2	KEDYALWLNILSKGIKARNTNLVDTYYRVHAGSVSANKFKE

Q5M956	LSGDKSLIEEVFPEIGDVMCNSVNAGWNHDSTHVIRFPLNG
Q96MV1	DKFFIMHHCASLYAYYLVLKNGVLAYIGNFRLLAELSSPFV
Q9JKT3	FFLLCWKFLDANSLWLVTILNSLYCVKITNFQHPVFLLLKR
Q9D9S3	ITLQIEEPKVTIDLQVTMPHNYPYVALQLFGRSPELDRQQQ
Q6ZMS7	LEWGKLEDWQKELYKHVMRGNYETLVSLDYAISKPDILTRI
Q9CWG1	WDPKLAQIAKAWTKSCEFKHNPQLHSRIHPNFTALGENIWL
O35660	VYDVLDQHRMFEPTCLDAFPNLKDFMARFEGLRKISAYMKT
P13634	QKVLDALNSVKTKGKRAPFTNFDPSSLLPSSLDYWTYFGSL
Q9BSU3	ENQETQGSTLSDSEEACQQKNPATEESGSDSKEPKESVEST
Q8K0E1	LSGEKALIEEVFPETGDVMCNSVNAGWNQDPTHVIRFPLNG
P43320	IIFEQENFQGHSHELNGPCPNLKETGVEKAGSVLVQAGPWV
P58753	LGKMADWFRQTLLKKPKKRPNSPESTSSDASQPTSQDSPLP
O08859	LCVLLWEEAHGWGFKNGIFHNSIWLEQAAGVYHREARAGRY
Q2KJD3	ENILSGNPLLNLTGPSQPQANFKVKRRWDDDVVFKNCAKGV
Q8WUU8	PQDNSAFVVDEGCLSFTDGGNHRPNPDVDQLEETQLEEEAC
Q8TAL6	SFFCHLCQGYFDGPLYPEMSNGTLHHYFVPDGDYEENDDPE
Q8TBG9	KVATDPKEVLLLMSACKQPSNKCMAIHSPVMSSLNTSVVFG
P15483	PIKLIFAALSLFPCSNIYANNITTQKFEAILGATRVIYHLD
Q0VCH8	QEKLEAQPPTPGQLRYVFIHNAIPFIGFGFLDNAIMIVAGT
P97299	DLKKSVLWLKDSLQCTCEEMNDINAPYLVMGQKQGGELVIT
Q8R1M8	WDVSLWDHVVPLEKVSDSCNNGNLINWQALNYEDNGYVVIK
Q0P5A4	QYNFDHPDAFDNDLMHRTLKNIVEGKTVEVPTYDFVTHSRL
Q9CQ61	QLFFINHVILTDTFIGYLVGNTLWLIAVGYYIYVTFLGYSA
Q2TBH7	SRFAQENELMFLETSALTGENVEEAFVQCARKILNKIESGE
Q8C1T8	SDHWIGLHRASTQHPWIWTDNTEYSNLVLTRGGGECGFLSD
Q96HJ5	CSSGTLSVVAGIKPTRTWIQNSFGMNIASATIALVGTAFLS
Q5E9D3	TFEADENENITVVKGIRLSENVIDRMKETSPSGPKSQRYSG
Q96E22	ALLCLHRTLTSWLRVRFGTWNWIWRRCCRAASAAVLAPLGF
O14966	YRDASACVIMFDVTNATTFSNSQRWKQDLDSKLTLPNGEPV
Q8BLR7	TEEQRVELSLWDTSGSPYYDNVRPLCYSDSDAVLLCFDISR
Q9BSU3	ELRRQMDLKKGGYVVLGSRENQETQGSTLSDSEEACQQKNP
Q86VX2	LTEPKEVERFLAQLSEFATTNQISLGSLRSIVKSLLLVPNG
Q3SZY9	DIRDNLLGISWVDSSWIPILNSGSVLDYFSERSNPFYDRTC
Q9P0G3	RWEATQQVLRVVRQVTHPNYNSRTHDNDLMLLQLQQPARIG
Q9Y294	RENPPVKPDFSKLQRNILASNPRVTRFHINWEDNTEKLEDA
Q8VE10	AANRLGDPLEAFPVFKKYDRNGLNVSIECKRVSGLEPATVD
Q9UGN4	HSELSQNPKQAATQSELHYANLELLMWPLQEKPAPPREVEV
Q8BGB7	VEASAAEEADVHVAVVVRPGNAGLTDDEKTYYNLITSFSEL
P28063	MGSMICGWDKKGPGLYYVDDNGTRLSGQMFSTGSGNTYAYG
P62080	VVGGVMFILGFAGCIGALRENTFLLKFFSVFLGIIFFLELT
H3BQB6	KTKEEEIRKRLRSDRLLPSANHSDSAELDGAEVAFAKGLQR

Q3MHR7	YITFVLFPRHTNASARDNTINLIHTFRDYLHYHIKCSKAYI
Q9BQ75	VHLGVGTPGRIKELVKQGGLNLSPLKFLVFDWNWRDQKLRR
Q9DCT2	HIAANWYEREVWDMFGVFFFNHPDLRRILTDYGFEGHPFRK
Q6ICC9	TGEAEKWAIPHMQPDSPLRNNYQGFLAELRRTYKSPLRHAR
P51674	LAGAGAAVIAMVHYLMVLSANWAYVKDACRMQKYEDIKSKE
Q969X5	PTVYEDKSGKQRYSYQYTVANKEYVAYSHTGRIIPAIWFRY
Q9CPR8	QGTPISGLLMIVLGLIFMKGNTITETEVWDFLRRLGVYPTK
Q9BYJ0	EYRGQPSMCQAFAADPKPYWNQALQELRRLHHACQGAPVLR
Q9D8X5	DDAKYCGRLYGLGTGVAQKQNEDVDCAQEKMSILAMINNMQ
Q9JKF4	LSDPQGNGKWQWIDDTPFSQNVRFWHPHEPNLPEERCVSIV
Q8VHW4	YISSINDEMLNRTKDAETYFNYKYGWSFAFAAISFLLTESA
Q32LJ6	YNSVNGIILVHDLTNKKSSQNLYRWSLEALNRDLVPTGVLV
Q9D1J1	NPDEGPKLDLGFKDGQTIKINIANMRKKEGAAGTPRARPTS
P0DMB1	VEQGPSTGAKKPSISGAPHLNSYQSLELPQNQQDSGTEELM
Q9CQ01	QFWKHEWVKHGTCAAQVDALNSEKKYFGKSLDLYKQIDLNS
Q2KI95	NPISGLGGTKYISFEERQWHNDCFNCKKCSLSLVGRGFLTE
Q9D305	HRFPLDPKRRKEWVRLVRRKNFVPGKHTFLCSKHFEASCFD
P61296	FAAAAAAAAAAAASRCSHEENPYFHGWLIGHPEMSPPDYSM
Q5TFG8	QSKSPPVRKSNWRQQHEDFINAIRSAKQCMLAIKEGRPLPP
Q8N4E7	AAASSRDPTGPAAGPSRVRQNFHPDSEAAINRQINLELYAS
O95407	RRDSPTTCGPCPPRHYTQFWNYLERCRYCNVLCGEREEEAR
P68401	LKNGELENIKPKVIVVWVGTNNHENTAEEVAGGIEAIVQLI
P17976	ISLSDLRFFMPSLTAEELRGNRSQWLYAVDVLIETQGEVCL
P09629	RGRQTYTRYQTLELEKEFHYNRYLTRRRRIEIAHTLCLTER
P15946	DDLQCVSIKLLPNEVCVKNHNQKVTDVMLCAGEMGGGKDTC
Q7Z7H5	LFAGGKLRVHLDIQVGEHANNYPEIAAKDKLTELQLRARQL
Q07021	IFSIREVSFQSTGESEWKDTNYTLNTDSLDWALYDHLMDFL
Q9ESK4	SQELGDEKIQIVTQMLELVENRARQMELHSQCFQDPAESER
Q61334	SKENLKTELKKASDALLKAQNDVMTMKIQSERLSKEYDRLL
Q9CR48	YVRYKQVHALNPEENLIIKLNKAGLVLGILSCLGLSLVANF
P14206	QMKEEDVLKFLAAGTHLGGTNLDFQMEQYIYKRKSDGIYII
Q5E9L3	EFSLADALPENSPAKTSAVSNTKPGQPPQGWPGSNPWNNPS
Q8CFP6	TIGIDYGVTKVQVRDREIKVNIFDMAGHPFFFEVRNEFYKD
P57076	QKQLMLYYHRRQEELKRLEENDDDAYLNSPWADNTALKRHF
Q01105	EIDRLNEQASEEILKVEQKYNKLRQPFFQKRSELIAKIPNF
P15319	EGEFETVMLSPRSEQTVKSANYNTPYLSYINDYGGRPVLSF
Q1LZ83	SSRVIRVLGCNPGPMTLQGTNTYLVGTGPRRILIDTGEPSI
P61019	AFINTAKEIYEKIQEGVFDINNEANGIKIGPQHAATNATHA
Q8N7P3	SLSLLGWVLSCLTNYLPHWKNLNLDLNEMENWTMGLWQTCV
P18021	SALLHYVPDLRRKMLLATLWNTGARINEALALTRGDFSLTP
Q99N84	KVAGNPCPICRDHKLHVDFRNVKLLEQFVCAHTGIIFHAPY

O43423	GGLEVLAEKCPNLTHLYLSGNKIKDLSTIEPLKQLENLKSL
Q9D5K1	KFVKRLYFGLRVLPPKLVNLNFQQSLTEDQKLIDKGWEVNL
Q80WB5	ADSYLKHFASDRSHMKDSSGNWREPPPEYPCIETGDSKMNL
P15927	ISNPGMSEAGNFGGNSFMPANGLTVAQNQVLNLIKACPRPE
Q9H2A3	SPASTWSCTNSPAPSSSVSSNSTSPYSCTLSPASPAGSDMD
P0C7M4	GGGEEKDGGGAGVPGHLWEGNLEGTSGSDGNVEDSDQSEKE
Q58DU5	TLYSAVRPFGCSFMLGSYSVNDGAQLYMIDPSGVSYGYWGC
P51911	PKLGTDQPLDQATISLQMGTNKGASQAGMTAPGTKRQIFEP
Q3UZZ6	EEMDHSVSPFMRKGISGDWKNQFTVAQYEKFEEDYVKKMED
O60930	YTDSMFTINGITNWVQGWKKNGWKTSAGKEVINKEDFVALE
P09629	DSAKAAGAKEQRDSDLAAESNFRIYPWMRSSGTDRKRGRQT
Q6ZTZ1	KLFEMTGERRLGEEIKIKITNMTFQYRKLKCMTDSESAPPD
Q8BX35	TCITCAVINRVQKANCTNTSNAICGDCLPRFYRKTRIGGLQ
Q9QZ73	RQFMIFTQSSEKTAVSCLSQNDWKLDVATDNFFQNPELYIR
Q9XSK2	AVTNKVPDSCCVNITHNCGINFVVKDIHTEGCVEKIAAWLR
P14231	VAVKFLNVTPNVEVNVECRINAANIATDDERDKFAGRVAFK
P28907	FNTSKINYQSCPDWRKDCSNNPVSVFWKTVSRRFAEAACDV
Q9P1T7	GICTSEACCCCCGDEMGDDCNCPCDMDCGIMDACCESSDCL
Q7YRQ8	ICRLKVNKKQCGELREQYFFNLSSMTCKKFISGGCHSNENR
P50295	IRREQYVPNQEFINSDLLEKNKYRKIYSFTLEPRTIEDFES
Q8CBW7	VICLNKRTRRSIQKLLEWENNRLYHKLALHWKLTKRKCETS
Q8R2Y9	TCKVADKTGSINISVWDDVGNLIQPGDIIRLTKGYASVFKG
Q9CQF3	LFAVPKNYKLVAAPLFELYDNAPGYGPIISSLPQLLSRFNF
Q8NC96	PPPPGGKVTIPPPSSSVAISNHVTPPPIPKSNHGGSDADIL
O15120	LGERMVRENLKVWIYPEGTRNDNGDLLPFKKGAFYLAVQAQ
O15217	VEFDEEFLETKEQLYKLQDGNHLLFQQVPMVEIDGMKLVQT
P33946	ACSFTTVWMIYSKFKATYDGNHDTFRVEFLVIPTAILAFLV
P05452	TPQTGSENDALYEYLRQSVGNEAEIWLGLNDMAAEGTWVDM
Q6Y1H2	AALPFVRQAGLYSISLPNKYNFSFDYYAFLILIMISYIPIF
Q8K4Z3	GAAVKYLSQEEAQAVDQELFNEYQFSVDQLMELAGLSCATA
Q9DB20	KSLNDITKREKFSPLTANLMNLLAENGRLGNTQGIISAFST
Q6P9G0	HRYLPYNSHAASYTWKYEGKNLNMDFTLEENGIRDEEEEFD
Q765N9	AGVLFLLAALLTLVPVSWSANTIIRDFYNPLVPEAQKREMG
P97952	VVWNGSRGTKDLQDLSIFITNVTYNHSGDYECHVYRLLFFD
Q96SI1	GWNQDPTHVIRFPLNGYCRLNSVQVLERLFQRGFSVAASCG
P52801	LRARNEDPARANADRYAVYWNRSNPRFQVSAVGDGGGYTVE
Q5SVD0	STTIVYPKYPKTVYTTTLDYNCHKKLRRFLSSVELEATEFL
P22615	LAETGYKFYPLMIYLISRAVNQFPEFRMALKDNELIYWDQS
P43307	LESRKRKRPIQKVEMGTSSQNDVDMSWIPQETLNQINKASP
Q6UWV2	SISISNPTIKDNGTFSCAVKNPPDVHHNIPMTELTVTERGF
P20334	GQELTKQGCKTCSLGTFNDQNGTGVCRPWTNCSLDGRSVLK

P35509	NFRVGKKIGCGNFGELRLGKNLYTNEYVAIKLEPMKSRAPQ
Q99JY3	IPQLRIVLLGKTGAGKSSTGNSILGEKVFNSGICAKSITKV
P23812	LENEFLVNEFINRQKRKELSNRLNLSDQQVKIWFQNRRMKK
Q923D2	VLLGTGNDLSPTTVMSEGTRNIVTAMKAHGVDKVVACTSAF
Q91VR2	VSSDRGLCGAIHSSVAKQMKNEVAALTAAGKEVMIVGVGEK
Q80VP8	LLASGLVFFFLFPHSVLVDDNGIKVTKVTFNEQDSLVVLDV
P62577	CTYNQTVQLDITAFLKTVKKNKHKFYPAFIHILARLMNAHP
Q9D504	DPNLVDVYSNTALHYAVCGQNISLANKLLQYKANLEAKNKD
Q3T0C6	TKTDKKSFNQSLAEWKLFIYNRTTGEFLGRTAKSWGLILLF
P97299	RSNCKPIPANLQLCHGIEYQNMRLPNLLGHETMKEVLEQAG
Q96S21	QARAYAEKNCMTFFEVSPLCNFNVIESFTELSRIVLMRHGM
O35075	NVKERASLPKFFIRGHLNSTNCAITQPLTGELVVEHSDAAI
Q9QYI7	SFGGSGSSGFKSVMSSTEMVNGRKVTTKRIIENGQERVEVE
Q9QZC7	AGVYGQQPANQVIIRERYRDNDSDLALGMLAGAATGMALGS
P23184	QWKCDGVFVSHIIDIKDNNINVSDSTLIWLHLENYHSDIVK
P12980	QPQKVARRVFTNSRERWRQQNVNGAFAELRKLLPTHPPDRK
Q99967	GGSSTPGGSGSSSGGGAGSSNSGGGSGSGNMPASVAHVPAA
P04156	KPGGWNTGGSRYPGQGSPGGNRYPPQGGGGWGQPHGGGWGQ
P62258	DYHRYLAEFATGNDRKEAAENSLVAYKAASDIAMTELPPTH
Q1JQA4	GPLACGVPYTCCFRNTTEVVNTMCGYKTIDKERLSVQNVIY
O55144	ASAQTPHPHEPISFGIDQILNSPDQDSAPAPRGPDGASYLG
Q9H4A6	RMDRRLLALIYLAHASDVLENAFAPLLDEQYDLATKRVRQL
Q8K201	EEEADSNEDPSMEEEDLLALNSSPATGKDTLDNGDYGEPDY
B6A8C7	GSLPKPSLSAWPSSVVPANSNVTLRCWTPARGVSFVLRKGG
Q9WU03	SSVLSVPRKQSAEDLSAEIFNYEEYCVPKAVTGPCRAAFPR
P99026	SPRAIHSWLTRAMYSRRSKMNPLWNTMVIGGYADGESFLGY
P21583	EGICRNRVTNNVKDVTKLVANLPKDYMITLKYVPGMDVLPS
O88456	AAQYNPEPPPPRSHYSNIEANESEEVRQFRKLFVQLAGDDM
Q5U4N7	FRKIRSSAPSSPSFSRALMSNTYLCFLTTGPRSSRENTQKS
Q0VCY1	TDLKFKGPFTDVVTTNLKLRNPSDRKVCFKVKTTAPRRYCV
Q16611	WIAQRGGWVAALNLGNGPILNVLVVLGVVLLGQFVVRRFFK
Q6P9H5	TNNQALAWLDVTLARRHCGFNNRAQGEEQEAQLRELMEKVE
P63073	AVVNVRAKGDKIAIWTTECENRDAVTHIGRVYKERLGLPPK
P26715	IYSDLNLPPNPKRQQRKPKGNKNSILATEQEITYAELNLQK
Q1ZYL8	YNCESSAQWKSAVQGLLNYINNWHKQDTSMRPRSSAFSWPG
Q9WVS0	PYFCRVFVFCFLIRLLTGEINGSADHRMFSFHNGGVQISCK
Q9DAK2	GGNKILPVIPQLIIPIKNALNLRNRQIICVTLKVLQHLVVS
Q56JV9	KIASDGLKGRVFEVSLADLQNDEVAFRKFKLITEDVQGKNC
A7MBB3	MCLAFWAIAGIWTVFSLAVVNKAVNLTDGFPYISVCGNVPP
Q08091	QREQELREWIEGVTGRRIGNNFMDGLKDGIILCEFINKLQP
Q9BQR3	RQLVQPGPHAMYARVRQVESNPLYQGTASSADVALVELEAP

Q08D83	GHPFKAYLDVDITLSSEAFHNYVNAAMVHINRALKLIIRLF
Q00977	MILVVAAKEVWGDEQADFVCNTLQPGCKNVCYDHHFPISHI
Q29S19	RRLFPQSPETRPILGLDVGCNSGDLSVALYKHFLSLHDGET
O54830	SDSYDQFMLEFLGQQELLTKNLTYCHKYSIKVPEDIEEAQN
Q02878	PLRRTHQKFVIATSTKIDISNVKIPKHLTDAYFKKKKLRKP
O35975	SVRFGQFSSSSLTKRVALSSNFFSNHGTLFIIRTCLGVNIK
A1XBS5	VNEINAYAATETPHLKLGLMNFADEFAKLQDYRQAEVERLE
Q9JLK7	MRTMGYMPTEMELIELSQQINMNLGGHVDFDDFVELMGPKL
A1E959	ELLLNLNNGQLLPLQLQGPLNSWIPPFSGILQQQQQAQIPG
Q504M8	KSKGGSEPATSTLPAAAAATNGPRLAHPRTVRPGPEAPPNG
Q96L14	KQLQAINAMIDPDGTLEALNNMGFPSAMLPSPPKQKSSPVN
P04394	PETPFDFTPENYKRIEAIVKNYPEGHKAAAVLPVLDLAQRQ
Q497K7	GDGPGLESVELPLPLETEHRNAMELEKVRMEFELTLLKYLH
Q5VST6	SGMRVAFPDTKKTYCFDAFPNIDKISKITSPVLIIHGTEDE
Q3SX30	HWEVVTHGYYGLGAGDQPGPNDKKSELLPVEWNSNKDLYVL
Q8R088	KSSEKKIESEEDTNQERSPDNEDPGDSKDIRLTLMEEVLLL
Q62283	DERSRAVDHVQRSLSCCGVQNYTNWSSSPYFLDHGIPPSCC
Q2YDD6	SLLTDGYQNKTDNSELHCVLNSNSTACSIAVGAGLLAFLSS
P52803	LGFEFRPGREYFYISSAIPDNGRRSCLKLKVFVRPTNSCMK
A5D7C9	LVVAVLRFIQLKPKVLNPWLNISGLVALCLASFGMTLLGNF
Q9WU03	CLDKCAGVTENTTDDMARNRNGADSSVLSVPRKQSAEDLSA
Q8R4V2	GLGWQEVLEAIKASRPIANPNPGFRQQLEEFGWANSQKLRR
Q9CWY9	HIPVHSTDLTEQKLRACLEENVNEHSVHCPHTPVFSVTGGT
Q9BUT1	AVIGLTKSVAADFIQQGIRCNCVCPGTVDTPSLQERIQARG
Q921H9	VDYLEGIQKNFDEAAKVLKFNCEKYGHGDSCYKLGAYYVTG
Q9NVV5	FFGSTTILMNFLYLLGEVLNNYIWDTQKKPPSWQDMKIKFM
Q9CRD2	KRKIAIRKAQGKTVEAIRELNEYLEQFVGDQEAWHELAELY
Q3T0A3	PLVCGSVAEGVVTSGSRICGNHKKPGIYTRLASYVAWIDGV
A5D7A0	EALGSADEELSAKLLRRADLNQGIGEPQSPSRRVFNPYTEF
Q3UME2	IYAGSKALSRLAVPVFFILHNVAEVLTCGYQKCVWKEKTSL
O35565	SVEIGVVAVKAINSNYYLAMNKKGKLYGSKEFNNDCKLKER
Q9TTJ5	VALRQSGGYVATVGTKFCALNWEDQSAVVLATVDKEKKNNR
Q8K015	PGRKCKGGVLAHLERLEAQTNISNRKSEEPAVRKKESSLRT
P97930	VTLVLDRYAFSGVAFTGAKENFSLDWCKQPDVGLPKPDLIL
Q8NDD1	KNTKQANPSVLERDVDTQEFNLEKARLEVHRFGITGYGKGK
Q2HJH7	NYLKKYHNTICGRHPIGVLLNAITELQKNGMNMSFSFFQQA
Q9P0W0	EARVPQLSSLELRRYFHRIDNFLKEKKYSDCAWEIVRVEIR
Q8BHC4	RINAQLPLKDKARMANHVLDNSGEWSLTRRQAILLHAKLER
O35740	GAGNMNATSGIRHAMGPGTVNGGHPPSALAPAARFNNSQFM
Q8N8R7	CHKTYYTRHTGFKTLQELSSNDMLLLQLRTGMTLSGNNTIC
Q96F25	VVAGSGGHTTEILRLLGSLSNAYSPRHYVIADTDEMSANKI

Q96AQ7	VARVTFDLYKLNPQDFIGCLNVKATFYDTYSLSYDLHCCGA
P20489	TSADMVLVHGSFDIRCHGWKNWNVRKVIYYRNDHAFNYSYE
Q8R3G9	VFQSEFKCCGLENGAADWGNNFVEAKESCQCTGTDCATYQG
P24042	ISDILYADIESKAKELTVNSNNTVQPVALMRLGVFVPKPSK
P06870	DDENTAQFVHVSESFPHPGFNMSLLENHTRQADEDYSHDLM
Q9Z1L2	LTDGYQNRTESPQLRCVLNSNHMACSFAVGAGFLSFLSCLV
Q6P8I6	QQTEIYVVPGETALAFYKAKNPTDKPVIGISTYNVVPFEAG
Q9ESJ7	QQQGFQAENVTVTKNIRRVENAYHMEAEVCITFTEFGKMQN
P00846	TKGRTWSLMLVSLIIFIATTNLLGLLPHSFTPTTQLSMNLA
Q8VCC1	LRDTFRKVVDHFGRLDILVNNAGVNNEKNWEQTLQINLVSV
O00124	RILLLLALLTLIISVTTSWLNSFKSPQVYLKEEEEKNEKRQ
Q5SRE7	RAPVGSAPGTSFLGSEPARDNSLFVPTPVQRGALVLIHGEV
Q9D7J9	TCSEVMMLIRNHPVPILAMVNGLATAAGCQLVASCDIAVAS
Q8R088	TRRTEVSKSSEKKIESEEDTNQERSPDNEDPGDSKDIRLTL
P43274	VSKGTLVQTKGTGASGSFKLNKKAASGEAKPKAKRAGAAKA
P49069	IKTTVLTAALLLSGIPAEVINRSMDTYSKMGEVFTDLCVYF
Q8BVD7	SGSTVIYLQPEDEVWLEIFFNDQNGLFSDPGWADSLFSGFL
Q62447	QKDLKFLSEEEYWKLQIFFTNVIQALGEHLKLRQQVIATAT
Q6PER3	FEFIQWFKKFFDANYDGKDYNPLLARQGQDVAPPPNPGDQI
P50194	FIHYDEKNNEWNLLQVKNRDNTENSSSSKIRDNTTIKKWFR
P23946	FVLTAAHCAGRSITVTLGAHNITEEEDTWQKLEVIKQFRHP
Q64317	PESLNSPVSGKAVFMEFGPPNQQMSPSPMSHGHYSMHCLHS
P70195	IDLCVISKSKLDFLRPFSVPNKKGTRLGRYRCEKGTTAVLT
Q9Y5K2	GGGHDQKDSCNGDSGGPLICNGYLQGLVSFGKAPCGQVGVP
Q149M0	CNPCPKTWQWYGNSCYYFSINEEKSWSDSRKDCIDKNATLV
Q8JZS9	KAKVELRAINVGTDYEYGVLNIHLTAYDMSLAESYAQYVHR
Q58CY6	VAGLRRFGCMVCRWIARDLSNLKGLLDQHGVRLVGVGPEAL
A1XBS5	TYGTIVKMKRDDLKATLTARNREAKQLTQLERTRQRNPSDR
Q3SZY3	KSSVPASDDTYPEIEKFFPFNPLDFENFDLPEEHQIARLPL
Q0VCD4	GSVLDHSLESLIHRLRGLCDNMEPETFVDHEMVFLLKGQQA
A5D7U1	IISVVLEARDQNKSVIGSWENPSDHCEGRAEYYLKGNFSTL
Q96BQ1	NVVGPTMCFEDRMIMSPVKNNVGRGLNIALVNGTTGAVLGQ
Q9NUS5	SDEQVPLHEAPRGAFRLAAENPFQEPRTVVWLGVLSLGFAL
Q2WG77	YWPKSHSFDYLYSAGEILLNNFPVQATINLYEDSDSADNEE
Q91XE0	NWKQHLQIQSSQSHLNKTIQNLASIQSFQIKHSENILYVSS
P35695	NISGGIDKFWLEGQLRISAVNQVEFLESLFLNKLSASKENQ
Q9DB52	VRMHSSRLHQIKQEEGMDLINRETVHEREVQTAMQISHSWE
Q13868	KRWKVETNSRLDSVLLLSSMNLPGGELRRRSAEDELAMRGF
Q3SY17	LRGPIKEHLPTATTHSAHLVNDFIGGGLLGAMLGFLCFPIN
Q2QD12	MHMMVSKPEQWVKPMAVAEANQYTFHLEATENPGTLIKDIR
Q9D8K3	QALMAMLVYVWSRRSPHVRVNFFGLLNFQAPFLPWALMGFS

Q3SX42	ALEKQEKQLELEIKKMAKIGNKEACRVLAKQLVHLRKQKTR
Q29RT9	SKVGSCPCDHTVSSHSPPNENIMRHLRKYLKAYQRCFSYVR
O14593	VDINIYDWNGGTPLLYAVRGNHVKCVEALLARGADLTTEAD
Q5IBH6	QYRPIHFFLALYLANDMEEDNQAPKQDIFYFLYGKSYAQRP
Q2HJH2	QESYANVKQWLQEIDRYASENVNKLLVGNKSDLTTKKVVDN
Q9NZC2	PCQRVVSTHNLWLLSFLRRWNGSTAITDDTLGGTLTITLRN
Q9D198	LAAQKREQRLRKFRELHLKRNEARKLNHQEVVEEDKRLKLP
O95857	SARNDIQRNLNCCGFRSVNPNDTCLASCVKSDHSCSPCAPI
P26441	ELMILLEYKIPRNEADGMPINVGDGGLFEKKLWGLKVLQEL
Q62276	LMKLVSDLKQFLILNDFPSVNEAIDQRNQQLRALQEECDRK
O95471	WAGSALVILGGALLSCSCPGNESKAGYRVPRSYPKSNSSKE
P04184	IGIDEGQFFPDIVDFCEMMANEGKTVIVAALDGTFQRKAFG
Q9JJG7	MKDIENIIINEEESLNETSGNCQTEFEGVHSQKQNRQTCVR
Q9H765	DEACVEVLLEYGANPNALDGNRDTPLHWAAFKNNAECVRAL
Q8BH93	AAPYPGPAGSYPTPAPHPALNNPYQVPSGPAGAPPMPGGPH
Q9UKQ9	KWEGPEQLFRVTDFFPHPGFNKDLSANDHNDDIMLIRLPRQ
Q7Z4H3	VSDHMYRMAVMAMVIKDDRLNKDRCVRLALVHDMAECIVGD
Q9ES81	YALNDRVYIGKKHHYDIRLPNYYHMSTPDLSRSPLTEQFRN
P06340	AAIKAHLDILVERSNRSRAINVPPRVTVLPKSRVELGQPNI
P37141	FGLVILGFPCNQFGKQEPGENSEILATLKYVRPGGGFTPNF
Q8WWF3	AWDPSQTMKKPKQNQLTPVTNSEVALVNAYPEQRRARRQSQ
Q8WU03	KTSSNDKMELFEVDDDNKEGNFSNMFLDASHAGLVNEHWAF
Q8C4A0	FNLATATAVQISWKTSGAHANPAVTLAYLVGSHISLPRAMA
Q9KIP8	FKHIALEEIYALCSAYSTTYNMPLALHLDHHESLDDIRRKV
A6QLZ5	QADSVVQSDQPETLASSSFVNLNFEMEGDCEVITESKQNPV
Q06520	VDYVVDKAQLLRKGVSGDWKNHFTVAQAEDFDKLFQEKMAD
Q8WUE5	VKPIRCIHTEEVCITSVHGRNGVIDYTIFFTLDSVKLPDGY
A6NJY1	AITGFNTCLSIVFYSGGMINNAIASLRNVCISLLAGIVLGF
O15537	YHKPLGFESGEVTPDQITCSNPEQYVGWYSSWTANKARLNS
A5D7J5	LCAEEIKATSYIECSALTQKNLKEVFDAAIVAGIQYSDSQQ
P62258	MVESMKKVAGMDVELTVEERNLLSVAYKNVIGARRASWRII
Q9H8T0	WQVLMYARRVFYKIDTASPLNPEAAVLYEKDIQLFKSKVVD
Q0VD27	HKVESESYQKIASSIGCSTNNILFLTDVSREASAAEEAGVH
A5PJS2	RGGKPMPLYAFLLAFIFCTYNGYLQSRYLSQYAVYADDWLS
O15520	GDVRWRKLFSFTKYFLKIEKNGKVSGTKKENCPYSILEITS
Q8N9P6	QRWMLPLRRGARLLPLASSKNPRARSPGLDPLGSSETLWSH
Q6Q6R3	NGRPYCHKPCYGALFGPRGVNIGGVGCYLYNLPTPPPASRI
P58418	LVMILFASEVKIHHLSEKIANYKEGTYVYKTQSEKYTTSFW
Q15181	KDPLNPIKQDVKKGKLRYVANLFPYKGYIWNYGAIPQTWED
Q58DW6	LAFETVLKEIFAKVSKQRQNNARTNAVTLGSGPAGQELGPG
Q8BX07	ICVVIDLDETLVHSSFKPINNADFIVPVEIEGTTHQVYVLK

A3KN05	STEEQPQQRQKTKMIILGFSNPINWVRTRIYSFLIWAYFDQ
Q30167	LLVCSVNGFYPGSIEVRWFRNGQEEKTGVVSTGLIQNGDWT
Q0VCU8	LKKLQEESDLELAKETFGVNNTVYGIDAMNPSSRDDFTEFG
Q6UWM5	SSKIPSITDPHFIDNCIEAHNEWRGKVNPPAADMKYMIWDK
P04769	TFNKIILDLHKSTENITKAFNSCHTVPINVPETVEDVRKTS
A6NJY1	FQPLLFGLVGAEVSVSLLESNIVGISVSTLSLALCVRILNI
Q3TC33	VISEHRRAVAQLSLELEKEQNRTSSFREALISQGRKLAEEK
P62491	YRGAVGALLVYDIAKHLTYENVERWLKELRDHADSNIVIML
Q7Z2X4	KPVIELWKKHTLAREDVFPANALLEIRPFQVWLHHLDHKGE
O69395	SKVMAAAAVLKQSETQKQLLNQPVEIKPADLVNYNPIAEKH
Q5HZI9	LRGPIKESLPTATTYSAHLVNDFICGGVLGAVLGFLSFPIN
Q8R0K4	IPFRLREIMRSRQEMKKTLSNKKRKKEAQVAFKKTLEKEAK
Q00623	EQMRESLAQRLAELKSNPTLNEYHTRAKTHLKTLGEKARPA
Q53RY4	ARGPRRLMRVGLALILVGHVNLLLGAVLHGTVLRHVANPRG
P11912	IIQNVNKSHGGIYVCRVQEGNESYQQSCGTYLRVRQPPPRP
Q86XT2	QGLQLEREACLASNYALAKENLALRPRLEMGRAALAIKYQE
Q96LD8	QQNSYDCGMYVICNTEALCQNFFRQQTESLLQLLTPAYITK
Q8NGY7	CDILPVMKLSCINTTINEIINFVVRLFVILVPMGLVFISYV
P58499	NLLMGEQLGNVARGINIAIVNYVTGNVTATRCFDMYEGDNS
Q80ZS3	RKAQQAITEHRELMAWNRDENRRMQELRIARLQLEAQAQEV
Q8C6C7	KCPSGIPLNIWNKFQELHKKNSEQKNSTPRFRQKKRKRSKK
A4IFN5	FCALMIVEEMAVLHEVKTDRNHCLHAAITTLVVALGFLTFI
Q6ZN79	HMISMHPITRKDASTSMTMENSLILEDPFECNDSGEDCTHS
P07203	MNELQRRLGPRGLVVLGFPCNQFGHQENAKNEEILNSLKYV
Q3TB82	GTVCGASSGDDDDSDEDREGNGDGDWPTQVEFYASGVSWSA
O95073	TKKVMEMIPQISSFCLVRDRNHIQSANLDEEAQAGTSSLQV
Q6IRU2	RAEVSELKCGDLEEELKNVTNNLKSLEAASEKYSEKEDKYE
Q9JM14	LRPDLAEKVASVYESPGFFLNLEPIPGALDALREMNDMKDT
Q2KIR7	MKDDLDHYTNTYHVYSEDLKNCQEFLDLPEVINWKQHLQIQ
P09326	HMTVVSGSNVTLNISESLPENYKQLTWFYTFDQKIVEWDSR
Q9D3W4	TYCSTMVQHCEALNRSVQVVNLDPAAEHFNYPVMADIRELI
A2VE58	VNEGYVNADSGPELRCVFNGNAGACRFGVALGLGAFLACSC
Q96PB8	VICKTSVLDEHAGRPFLNAANDADLCNLPKKTTDYAMLVTM
P01213	DCLSRCSLCAVKTQDGPKPINPLICSLQCQAALLPSEEWER
A1A4L1	QLFTRGFEDGLGFEYVIFKNNDEKRTVCLFQGGPYLQGVPG
P16110	SGKPFKIQVLVEADHFKVAVNDAHLLQYNHRMKNLREISQL
A6NGA9	FAGIISLLNYLTSRSPACDENVTVIPTERSRLGVGPVTTVS
Q32KW0	DYIGGSHYVIQPHDDTEDSMNDHEDTNGSKESFREQDIYLP
Q9UKR8	SGIILVGLGIGGKCGGASLTNVLGLSSAYLLHVGNLCLVMG
Q504P2	LQRAKEELQENVFLQLKHNLNSSKKIKNLSAMLQSTATQLC
P14207	YSRGSGRCIQMWFDSAQGNPNEEVARFYAAAMHVNAGEMLH

Q9D7S9	DAELVKYKDQIKKMREGPAKNMVKQKALRVLKQKRMYEQQR
P48060	RDQVKRYYSVVYPGWPIYPRNRYTSLFLIVNSVILILSVII
Q9BUE0	LVELSVVAPAGQDMVSDDMKNFAEQLKPLVHLEKIDPKRLM
Q7YRQ8	EFMYGGCEGNANNFETLEACNEACWKIEKVPKICRLKVNKK
Q9NYV7	GISRFCLQWASMLNNFCSYFNLNYVLCNLTITWEFFNILTF
P0DMM9	LALLPQTLLDQKVKVVYVARNPKDVAVSYYHFHRMEKAHPE
P15428	VYCASKHGIVGFTRSAALAANLMNSGVRLNAICPGFVNTAI
Q5EAE5	QRLSPNTMVTPHKKSMLGNGNYDVNVIMAALQTKGYEAVWW
Q3TBW2	LLRQDIVAVFRDNRMIAVCQNVALSAEDKLLLRHQLRKHKI
Q8BTG6	REQPIPWKVQFNLGNSSRPSNQCRNSVQGKHLLTDELGYVC
Q60720	ALLFKLGSKYSTFEPEGVLANVLGLLLVCFGVVVLYILAQA
Q5E995	PRVLQHKRRRIALKKQRTKKNKEEAAEYAKLLAKRMKEAKE
Q8NGU1	FIIIGSLIPTIVSYTYIISTNLKIPSASGWRKSFSTCASHF
Q96BX8	VHVYIHHFDRIAQMGSEAHVNTCYKHFYYFVKEFGLIDTKE
F6YCR7	LFEETQYPDLLTRGELARTLNVPEVKVKVWFTNRRAKQRKI
A6NCN2	ATVIRHGETLRRTKEEINELNRMIQRLTAEVENAKCQNSKL
Q9BPW8	AVLPKLHLDEDYPCSLVGNWNTWYGEQDQAVHLWRFSGGYP
P57735	EDYNFVFKVVLIGESGVGKTNLLSRFTRNEFSHDSRTTIGV
Q58DW6	VVLIGESGVGKTNLLSRFTRNEFSHDSRTTIGVEFSTRTVM
Q8BWY2	ISLYSGTSTVTRRAEPRHSENGTPSSVWRPVALTLLTLCLV
Q96DX5	GMDGSKPAGPRDFPGIRLLSNPLMGDAVSDWSPMHEAAIHG
Q92527	AVINNNPKMVKFLLEKGADVNASDNYQRTALILAVSGEPPC
Q60653	SDFNLKEEMLTNRSIDSRPGNELLESLNREQNRGYSETKTD
P53516	PDDVAGKVEWQRAGNRLKGVNPTPFYINLSTLTVGGKEVKE
Q7L4S7	IKQRPFPYEHKNTWSAQNCKNGSCVLDLSKCLFIQGKLLFA
P49069	EEAMKLRKQLISEKPSQEDGNTTEEFDSFRIFRLVGCALLA
P23946	LKEKASLTLAVGTLPFPSQFNFVPPGRMCRVAGWGRTGVLK
Q9NUN7	IFLLGFLFWNIDNIFCESLRNFRKKVPPIIGITTQFHAWWH
Q9D9B4	TPSLEYLSLLGNVACPNELVNLEKDEEDYKRYRCFVLHKLP
Q17QU3	RKFDPKGNEIEPNFSATRKVNTGFLMSSYKVEAKGDSDRLT
Q9CPR5	ARGCGTSLDLLRSLPRVSLANLKPSPNSRKRERRPRDRRRG
Q9CQE6	QRNILASNPRVTRFHINWEDNTEKLEDAESSNPNLQSLLST
P80311	EDIGRVVIGLFGKTVPKTVDNFVALATGEKGFGYKDSKFHR
Q8TAF8	CFSVLVMALFIQPYWIGDSVNTPQAGYFGLFSYCVGNVLSS
P62259	VIGARRASWRIISSIEQKEENKGGEDKLKMIREYRQMVETE
Q8BH49	PSVAPQRPPPLPPRRRASAANGPLASFAQLHARYGLEVQAL
P20718	EQERTQQFIPVKRPIPHPAYNPKNFSNDIMLLQLERKAKWT
Q15014	ENPPSGSVRKTRKNKQKTPGNGDGGSTSEAPQPPRKKRARA
O95249	EIEQLLARLTGVNDKMAEYTNSAGVPSLNAALMHTLQRHRD
P47741	YDTCKQCTQCNHRSGSELKQNCTPTQDTVCRCRPGTQPRQD
Q0VCI6	EPHAELDAKGQGCTAYDVAVNSDFFRRMQNSDFLRELVITI

Q96PM5	EVAQTPMPSEYQNMTVDILCNDCNGRSTVQFHILGMKCKIC
O60762	FIRKQKEGNFDIVSGTRYKGNGGVYGWDLKRKIISRGANFL
P21337	KHHTRSAPLPTESWQQFLQENALSFRKALLVHRDGARLHIG
Q3KRB8	IHTEGLFRKSGSVIRLKALKNKVDHGEGCLSSAPPCDIAGL
Q8K2U2	LLHGISATRVDELDATSLPPNATACKSALPGAHLVRGTRVS
Q8BVA2	LCCDNCHSHVALALNLMRYNNSTNWNMVTLCCFCLIYGKYV
P08883	DPKKIQSTYSGDSGGPLVCNNKAYGVLTYGLNRTIGPGVFT
O15260	KTKLAALTLVVWLFAINVYFNAFWTIPVYKPMHDFLKYDFF
O88822	TLSYYFVYDHSLMKHPQFLKNQVSREIVFTVKSLPWISIPT
Q99967	GPGTVNGGHPPSALAPAARFNNSQFMGPPVASQGGSLPASM
P31213	VLLGLFCVHYFHRTFVYSLLNRGRPYPAILILRGTAFCTGN
Q6NS38	VAVVRLPLAHGSLLMMNHPTNTHWYHSLPVRKKVLAPRVNL
Q9CXK4	NNLSWLSYGVLKGDGTLIIVNSVGAVLQTLYILAYLHYSPQ
Q8BWP5	FSMIKPFLTEKIKDRIHLHGNNYKSSMLQHFPDILPREYGG
Q8TCD6	LFNFIRKNKDKFDCIIISDSNSVFIDWVLEAASFHDIFDKV
Q9D0M0	VDGRGCEDYRCVEVETDVVSNTSGSARVKLGHTDILVGVKA
P48230	IFPALVFLGLKNNDCCGCCGNEGCGKRFAMFTSTIFAVVGF
Q9D2R4	RHDFLESAPGLGMFVTVTTYNDEVLLSKLYGAQGTFYFTSH
Q9WVG7	EITAGGSGAALKTKPRFDFANLALAATQEDPTKLGRGEGPG
Q91VT1	RENDLTEGVDEDMIVTQSQTNFICPITQLEMKKPVKNKMCG
Q17QK6	GPEDERDHWQQFYFLSKRRRNLLRNPCGEEDLEGWCDVEHG
P10738	IHQDILQFQFPNKQRYKIVGNIPYHLSTQIIKKVVFESHAS
Q8BPA8	MAEEYDEKTSELLVRKWRVKNALGALGQWQLEVGEPVPSGA
P60334	FAGDEVNVEEVQAVLEAYESNPAEWALYAKFDQYRYTRNLV
Q9QVN7	AIREHQMARTGGTQTDLFTCNKCRKKNCTYTQVQTRSSDEP
Q9HBM1	KEEERMVEMFLEYQNQISRQNKLIQEKKDNLLKLIAEVKGK
Q6FIF0	TAQQPSEEQSKSLEKPKQKKNRCFMCRKKVGLTGFECRCGN
P02702	PVKAACHRFDFYFPTPAALCNEIWSHSYKVSNYSRGSGRCI
Q6PHW0	DSSDLHQAEEDADEWQESEENVEHIPFSHNHYPEKEMVKRS
P20615	KQGTPEYSLETSAGREAVLSNQRAGYGDNKICEGSEDKERP
Q3SZ22	QPGETLRQTAERTLATLSENNLEAKFLGNAPCGHYKFKFPQ
Q5TBK1	RYEHDVTFHSVLHAEKPSRMNRNQDRNNALPSNNARYWNSY
P61982	KELEAVCQDVLSLLDNYLIKNCSETQYESKVFYLKMKGDYY
P15949	GIDAAPPVHSRIVGGFKCEKNSQPWHVAVYRYNEYICGGVL
Q9NP72	WLNELETYCTRNDIVNMLVGNKIDKENREVDRNEGLKFARK
P01246	EDGTPRAGQILKQTYDKFDTNMRSDDALLKNYGLLSCFRKD
Q99LB2	SVVIVGSVAGFTRFPSLGPYNVSKTALLGLTKNFAAELAPK
Q9HAN9	KKKGLIPAYHRVIMAELATKNSKWVEVDTWESLQKEWKETL
Q53H82	RVLYTPGHTDDHMALLLEEENAIFSGDCILGEGTTVFEDLY
Q5JQC4	DSDIGPATEEEEEEEGNEAANFDLAVVARRYPASGIHFVLL
P08218	SLQYSSNGQWYHTCGGSLIANSWVLTAAHCISSSGIYRVML

P61092	QHKSITTLQGEDIVFLATDINLPGAVDWVMMQSCFGFHFML
Q5JWF8	VCHATPIYAGHSWHQATFRLNVAGSTLSRYLRDLLVAANPD
Q8WU68	SFFEEVFTELQEKYGEIEEMNVCDNLGDHLVGNVYVKFRRE
Q80WR1	KELTKHYQGDNDTDVFSATWNSVMITFGCCGVNGPEDFKLA
Q08331	EIWKHFDADGNGYIEGKELENFFQELEKARKGSGMMSKSDN
Q9JLK7	ETADMIGVKELRDAFREFDTNGDGEISTSELREAMRKLLGH
Q32KN7	QCTLAIKEGRPLPPPPPPTVNPDYIQCPYCKRRFNETAASR
Q60930	GTLETKYKWCEYGLTFTEKWNTDNTLGTEIAIEDQICQGLK
Q3ZBR0	HLSSLQLFYGKKPPGKGTEQNPRQHNELRREETTAEAPLLT
Q8BJK1	TDSEGRYFIDRDGTHFGDVLNFLRSGDLPPREHVRAVHKEA
Q9UBV8	DSDHSGYISMKELKQALVNCNWSSFNDETCLMMINMFDKTK
Q8IZJ6	HNLQLFVPSTIGAFGPTSPRNPTPDLCIQRPRTIYGVSKVH
P09497	ESKEETPGTEWEKVAQLCDFNPKSSKQCKDVSRLRSVLMSL
Q8N4C7	ILKSQHAAMFRHFQQIMFIYNDTIAAKQEKCKTFILRQLEV
Q7L8S5	FSNPETSDSFGYDDFMIYCDNIVRTTAWGGQLELRALSHVL
Q8CB49	LKQEEEDQPSENDHLSTKEGNSGKDPGSRRISRQQSITKAT
P04233	PLKVYPPLKGSFPENLRHLKNTMETIDWKVFESWMHHWLLF
Q8BVZ5	YFRKEPTKRYSLKSGTKHEENFSAYPRDSRKRSLLGSIQAF
Q9CR35	TFGCGVPAIQPVLTGLSRIVNGEDAIPGSWPWQVSLQDRTG
Q3T166	VSLWDHVVSLKNLCFTCYTSNILNWKALIYQAKGYVVVKPK
P59796	HVLFVNVAAYUGLAAQYPELNALQEELKNFGVIVLAFPCNQ
A2RRY8	GKKKYDIDPRNGIPKLTPGDNPYMFPEQSKEFFKAGATLPP
P15483	ESLKTLCVRGIPPKQGDLWANNEKEFVGMKLNVSINTCIKL
Q7YRA3	QIDIYVQVLQADGGTYAACVNAATLAVLDAGIPMRDFVCAC
Q9BRQ6	KEPSSPPPAPTSSTFGLQDGNLRAPHKESTLPRSGSSGGQQ
Q3UC65	DATKNTSEKSSTQRNIAFSSNNSVAKPLQKTTKAAVEEKSS
Q92564	LIYRYAFDFAREKDQRSLDINTAKCMLGLLLGKIWPLFPVF
P12979	RLKKVNEAFEALKRSTLLNPNQRLPKVEILRSAIQYIERLQ
Q6PIL6	FEDFIKGLSILLRGTVQEKLNWAFNLYDINKDGYITKEEML
Q9QY76	DRNVCFKVKTTVPRRYCVRPNSGVIDAGASLNVSVMLQPFD
Q8TDQ1	APVTQEETSSSPTLTGHHLDNRHKLLKLSVLLPLIFTILLL
Q8MKI3	KKQTAREMKKKVLAERRKPLNIDHLSEDKLRDKAKELWDTL
Q5XG99	PVFYLVYFKIQASGETPNSLNTTVIPNGSMAMGTVPGQAPR
Q99807	GKEGAMACTVAVEESIAHHYNNQIRTLMEEDPEKYEELLQL
P53519	KNNTPYYISFSEVFFDSDKVNNAKDILYVKPYSEKKIDISN
Q8R173	HHCSVCKRCIRKMDHHCPWVNNCVGENNQKYFVLFTMYIAL
Q6ZRR5	LVLGESGTEVNAVLFGSELTNPLLQMRWFLRETGHYHSFTG
Q8VCQ3	SSTWQKFAANTGKAKDIPIPNLPPLDFPSPELPLMELSEDI
Q8BH58	VEKLADELHMPSLPEMMFGDNVLRIQHGSGFGIEFNATDAL
O43688	MIGRLRPNFLAVCDPDWSRVNCSVYVQLEKVCRGNPADVTE
Q969U7	LKFVSEGDNIPDALGLVEYLNEWLQILKPLSDDPTVSASRW

Q9BSU3	RLGLAQKLMDQASRAMIENFNAKYVSLHVRKSNRPALHLYS
Q8WUU5	GGGGFGAATFASTSATPPQSNGGGGGKQSKQEIHRRSARLR
Q8WUD1	YYRGAAGALLVYDITRRETFNHLTSWLEDARQHSSSNMVIM
Q9Z0F5	LATQYMSFWELLSLTFFDVLNVAVLRCHPLTIFTFHVINIW
Q9P0P8	GLDIGRNKVEDAFYKGELRLNEEKLWKKSRTVKVGDTLDLL
P20645	VCREAGNHTSGAGLVQINKSNGKETVVGRLNETHIFNGSNW
Q86WI0	YWLFGSQMGKPVSFSTFRRCNYPVRGEGHSLIMVEECGRYA
Q32LB5	MSPYTNGTPCSMCQGDTCENNLCRNKERDKSQRYPNWNPSG
Q8R023	YRYDEQLSLCLERLSSGKDKNKNVLQNKYVRCSVRAEVRHL
P57729	KPVSVVLLANKCDQGKDVLMNNGLKMDQFCKEHGFVGWFET
Q8BTZ5	QGNTALHLCGHVDTIQFLVSNGLKIDICNHQGATPLVLAKR
Q9H2K0	MEKANPKTGPTLRKELILSSNIGQHDLDTKTKQIQQWIKKK
O08585	FYKQPFADLIGYVAAEEAFVNDIDESSPGTEWERVARLCDF
O95721	VFGGLVNYFKSKPVETPPEQNGTLTSQPNNRLKEAISTSKE
Q2KI11	VVEWFYRPEGGKDFLIYEYRNGHQEVESPFQGRLQWNGSKD
Q9QWZ1	CFATKNGIKVTVENAKCVQANAFIQADVFQEFVIQEESVTF
Q9CY45	LQEFYAEQKQSVNPRGDDKYNVGVIEENWQLSQFWYSQDTA
Q9D1N9	KELVRVEATVIEKTESWPKINMKFRKRKNFRKKKIIVNPQT
P97952	AEMVYCYKKIAAATEAAAQENASEYLAITSESKENCTGVQV
Q9NWC5	DILCFTISSLPVSLTKLMLSNALFVEAFIFYNHTHGREMLD
Q06432	AMTAVVTDHWAVLSPHMEHHNTTCEAAHFGLWRICTKRIPM
P11049	AESHIYREGCAQGLQKWLHNNLISIVGICLGVGLLELGFMT
A5D6W6	HVNPRTIFASHGNFFNIKFVNSAWGWTCTFLGGFVLLVVFL
P68252	THPIRLGLALNYSVFYYEIQNAPEQACHLAKTAFDDAIAEL
Q9NVV0	YLVSVMAVKRQPGAAALAWKNPISSWFTAMLHCFGGGILSC
Q9NPC8	TFGFTQEQVACVCEVLQQGGNIERLGRFLWSLPACEHLHKN
Q8BFX1	RKALRGKENKGSVEIMRKDLNDARDLHGQAESAAAVWKGHV
Q9CQE6	FSKLQRNILASNPRVTRFHINWEDNTEKLEDAESSNPNLQS
Q15006	LENLELSRKYFAQALKLNNRNMRALFGLYMSASHIASNPKA
A5D9A7	VSGINEKLFFSLKNGTKTYSNLPSEAVFANCAGMLVVVFGL
Q05B67	HLITSAVWFGTFYYAAMKGVNVVPFLELIGLPDSIVNILKN
Q2HJ48	LSGDKSLIEEVFPEIGDVMCNSVNAGWNHDSTHVIRFPLNG
Q28203	KQYPVNSLCCDLCPPGQKLVNDCTEVSKTECQSCGKGEFLS
Q91WN2	INTTALITGCTNAAGLVVVGNFQVDHAKSLHYIGTGVAFTA
Q9D0Z3	LELLFDYEIEREPLLFHVFSNAGVMLYRYVLELLQTHQRFR
Q9BXJ1	CLRCCDPGTSMYPATAVPQINITILKGEKGDRGDRGLQGKY
Q9D6Y7	VQMEAALRSKEEYQKVLSKHNFGPITTDIREGQVFYYAEDY
Q8K2Y7	VNPKDAAHSGCRSSLSLLHKNTPHVTSFLQCKLLHTTLSRK
Q9DCI3	ENTLTGSQSSHASLRDIHSINPAQLMARIESYEGREKKGIS
Q7Z7J5	INKVCRDTLRDWCQQLGLSTNGKKIEVYLRLHRHAYPEQRQ
Q8N9N7	ESLPPLLIGKFTLLKSLSLNNNKLTVLPDEICNLKKLETLS

O95619	DPNERPVTLYHLLKLFQSDTNAMLGKKTVVSEFYDEMIFQD
Q9CQF4	SSHKLCASWNRYLYFSSTKLNTSNYKTLFRNIFSLRLPELL
P80724	KDKDKKAEGAGTEEEGTPKENEAQAAAETPEVKEGKEEKPE
P26717	TFSEVSLAQDPKRQQRKPKGNKSSISGTEQEIFQVELNLQN
Q9H082	MASFHSLPSWIEECKQHLLANDIPRILVGNKCDLRSAIQVP
Q9D1G1	QWLQEIDRYASENVNKLLVGNKSDLTTKKVVDNTTAKEFAD
Q8BVA2	TAVHDASEEYKHRMHNLCCDNCHSHVALALNLMRYNNSTNW
Q5TZF3	YAACMAGQSDVIRALAKYGVNLNEKTTRGYTLLHCAAAWGR
Q3UTB7	KKTNPDKDGPDIKPNLWMWVNPNMVYPPGKLEVAVKEEDQS
P63013	NLTEARVQVWFQNRRAKFRRNERAMLANKNASLLKSYSGDV
P35276	SPRDAADQNFDYMFKLLLIGNSSVGKTSFLFRYADDSFTPA
Q99LU8	YEKQFLHVLSRKDKTGIVVNNPNQSVFLFIDRQHLQTPKNK
O75636	WSSYRAGFGNQESEFWLGNENLHQLTLQGNWELRVELEDFN
Q96S90	LFNGLDSEEEKDGEEKVHPSNSEVWPHSTERKKQETGAGRA
Q76JU9	LLTLLLCLGPYNASNVASFINPDLGGSWRKLGLITGAWSVV
Q96DN0	EINSLHMVTEYNPVTVIGLFNSVIQIHLLLIMNKASPEYEE
Q8R1H8	ELLTETDLGESQKQLKKKQKNRVAAQRSRQKHTSKADALHQ
Q9D0S4	SLPDLVSLGHSWVFAITRHHNRVPREGQPEAEAAVPSGPQA
P24668	VCREASNHSSGAGLVQINKSNDKETVVGRINETHIFNGSNW
Q13643	KYIQTDSGPYCVPCYDNTFANTCAECQQLIGHDSRELFYED
Q9H765	NAECVRALLESGASVNALDYNNDTPLSWAAMKGNLESVSIL
Q8K3A2	EKQRFTLQPGEPSTGLLIRANQGHSLQVPELELTPLETPQA
Q8N6G2	LNEDIFLWTLPHCQQTGTLKNCLPWKIPASMKEVNKALSNQ
P57087	VYYQQTLQGDFKNRAEMIDFNIRIKNVTRSDAGKYRCEVSA
O88384	RLLGTAGTEEKKKLVRDFDENQQEANETLAEMEEELRYAPL
Q6P1S2	EPNVVARISQWADDHLRLVRNISTGMAIAGIMLLLRSIRLT
Q9XSA7	HPESNTAGMDIFAKFSAYIKNSRPEANEALERGLLKTLQKL
Q80WB5	YNSCYCEENIWKLCEYIKTHNQYLLEECYAVFISNEKKMVP
Q9CZ04	FRLLTVFAYGTYADYLAEARNLPPLTDAQKNKLRHLSVVTL
Q86W74	DICQLLHKFGADLLATDYQGNTALHLCGHVDTIQFLVSNGL
Q4VC05	VTTPENSSSPGMMDMHDDNSNQSSIADASPIKQENSSNSSP
P28663	AEAEKRVKASHSFLRGLFGGNTRIEEACEMYTRAANMFKMA
Q9ESL8	GILRRRQLYCRTGFHLEIFPNGTVHGTRHDHSRFGILEFIS
Q3KQJ0	LSPSFQVCMRKIQCYHVSPCNFKKQKAVLPPKKRSTITYLL
A2A5I3	VCSHYTQMVWASSSRLGCAINTCSSINVWGNTWQQAVYLVC
Q9BUN5	LQHSFLTEVTDVYEMEGGLLNLLNDFHSGRLQAFGKECSFE
Q8C7N7	WLVSLLLSSVFWFLVRVITDNRDGPVQNYLLIFGVLLSVCI
Q9GZU7	ICVVIDLDETLVHSSFKPVNNADFIIPVEIDGVVHQVYVLK
Q8IXQ9	NDIDPIAGMAITLNCELNRLNPFPILIQNILNLEQDKWDLV
Q60652	KQWAWIDNGPSKLDMKTRKMNFKPGGCIFLSKTRLEDTNCN
P13972	WLFFCKNSNVIVDYCRGLRTNITNDRMLEFKNKSTEIQWKV

P08505	VGGLITHVLWEIVEMRKELCNGNSDCMNNDDALAENNLKLP
Q9NYN1	SSVFGKPRAGSGPQSAPLEVNLAILGRRGAGKSALTVKFLT
Q91ZR1	RETYNSLAAWLTDARTLASPNIVVILCGNKKDLDPEREVTF
Q53H82	SGDCILGEGTTVFEDLYDYMNSLKELLKIKADIIYPGHGPV
Q2LGB5	VSAQPRCSEEDLKAIQDMFPNMDREVIRSVLEAQRGSRDAA
Q78HU3	MDGVPFTLHPRFEGKSCGPLNLSAFGDLTIKSLADIEKEYN
Q8R2H9	ARGLLTLRPFHTHSCSRCPANMCKHKVLSEYLRERARDGVH
Q91VE3	VCNDTLQGLVSWGTYPCGQPNDPGVYTQVCKYKRWVMETMK
Q9BYD2	KAGEATVKFLKSCRLEVGMKNNVKWELNPEIVARHFFKNLG
Q9EQQ2	QFGYVYGISAIGCLGMFCLLNLMSMTGVSFGCVASVLGYCL
Q13445	ADGVHTVEPTEAGDYKLCFDNSFSTISEKLVFFELIFDSLQ
Q9CXW3	VQVHFTERSFDLLVKNLNGKNYSMIVNNLLKPISVESSSKK
Q9D1H9	PVFCDMTTEGGKWTVFQKRFNGSVSFFRGWSDYKLGFGRAD
O75558	AAEAQHGPHSAVARISRAQYNALTLTFQRAMHDYNQAEMKQ
Q9WTN0	GLFFQIRDDYANLHSKEYSENKSFCEDLTEGKFSFPTIHAI
Q9JIY7	VVCIFFLFLFLWFLASKPWKNYVSKCLHTDMADITKSYLSV
Q9H009	SKLGLLQVTGVTRVTIWKSKNILFVITKLDVYKSPASDAYI
O75832	NPDAKDHYEATAMHRAAAKGNLKMIHILLYYKASTNIQDTE
Q2KID0	FIWVYPTPEHKEDDAGGFIANLEPVSLTDREVISRLRNCIV
P11403	KLFGVPFFTDECKFKEILLPNNYNAYESYAYPGMFMALSKN
Q47098	AELLAGAGFDWLLIDGEHAPNNVQTVLTQLQAIAPYPSQPV
Q8TDC0	SYRDYQSDGRSHTPSPNDYRNFNKTPVPFGGPLVGGTFPRP
P31269	FSENNAENESGGDKPPIDPNNPAANWLHARSTRKKRCPYTK
Q7Z7D3	GRTAVFADQVIVGNASLRLKNVQLTDAGTYKCYIITSKGKG
A6NKC4	FTEGEPLALRCHAWKDKLVYNVLYYRNGKAFKFFHWNSNLT
Q3UFF7	KETNSKLKSLGVSTTFHSLPNLNHELNKTELEKLKSWILTR
Q92826	YATLDGAKDIEGLLGAGGGRNLVAHSPLTSHPAAPTLMPAV
O95983	VRQTASIFKQPVTKITNHPSNKVKSDPQKAVDQPRQLFWEK
P0CI32	QFFPQPADFTGPYVMLTSRRNNRALSGVMINADAGLAIRGI
Q2YFS2	PFIVTSEFTTAGLEHTSDQRNPSLMNLGAMVTMLLAKVLVI
Q8QZY6	MVGVVMFTLGFAGCVGALRENICLLKFFCGAIVLIFFLELA
Q8IUI4	REFGYQKLDVKSIDDEDVDENEDDVYGNSSGRKHRGHSESP
P07451	DPSLQPWSVSYDGGSAKTILNNGKTCRVVFDDTYDRSMLRG
Q96BT1	YSPKKRPHFPALKKKKRGMENILRKSDLTVGKLQMQVDDLI
Q3U1C6	LSSICLETDSPALGPEKLTRNEPCNISIAAEFIAQVKGISV
Q9NZ43	QPVSEKQLAAELDLVLQRHQNLQEKLAEEMLGLARSLKTNT
Q91WN2	LLEQSRHSWINTTALITGCTNAAGLVVVGNFQVDHAKSLHY
Q15056	SQKELPTEPPYTAYVGNLPFNTVQGDIDAIFKDLSIRSVRL
Q00188	TPLCIDTDPVNATFEGYKALNVRRLNIMAGDEINSRNFDTL
Q4JM65	VPVKKQKIRTVFSQTQLCVLNDRFQRQKYLSLQQMQELSNI
Q8R1Q0	EHINRALGDVVKEVKKSEVENGPSSVVTRILKSQYAAMFRR

P56965	DMMKEALEKLQLNIVEMKDENATLDGGDVLFTGREFFVGLS
Q7M729	FKWSYNNSETSRILIDGIVKNDKSDPKVRVKDDDRITLEGS
P09920	LSGISPALAPTLDLLQLDVANFATTIWQQMENLGVAPTVQP
Q7L4S7	WTEPGAPGGTEDRPSGGGKANRAHPIKQRPFPYEHKNTWSA
Q9CQG3	HRGGVAPGFQVVHLNAVTVDNRLDNLQLVPWGWRPKAEETS
Q17QJ5	AGVLAFVFKDWIKDQLYFFINNNIRAYRDDIDLQNLIDFTQ
Q8CIL4	VLERERAIMLGAKPPKNTYVNYKVLQKQIKEKKIAVEEEKR
Q2TA42	AREEEERAKREELERILEENNRKIAEAQAKLAEEQLRIVEE
Q9ULW5	KKTPKSKGASTPAASTLPTANGARPARSGTALSGPDAPPNG
Q0P5E2	FISGILAALSAMISLEIPQVNVMTKMDLLSKKAKKEIEKFL
Q96A56	EATRGTDELHSPSSPRVEAQNEMGQHIHCYVAALAAHTTFL
Q6P5X7	SKSWLHGSIFGDINSSPSEDNWLKGTRRLDTDHCNGNADDL
Q3T033	PDLVFRADQRSGPRQLLTLYNPTGAVLRFRVLCTAPAKYTV
Q96GY3	SNTYVIKLFDRSVDLAQFSENTPLYPICRAWMRNSPSVRER
O14593	SPQAGSSLKHSTTLTNRQRGNEVSALPATLDSLSIHQLAAQ
P51809	TEQILAKIPSENNKLTYSHGNYLFHYICQDRIVYLCITDDD
P70661	ELALSKQRRSRRKKANDRERNRMHNLNSALDALRGVLPTFP
Q99N07	WKQSSSAFSGNVIFLSQNSKNKSSVSSESLCNPTYENILTS
B6A8R8	PSKYFILKKEGFALNSVKPYNLTEETADFHFTDLRQNDGGH
P57784	NEIRKLDGFPLLRRLKTLLVNNNRICRIGEGLDQALPCLTE
Q28030	QKNDVCRPGRYYEQPDNGVLNYPKRACQFNRTQLGDCSGIG
P22794	HIVALTSKSEQELYIPSVVSNSPSTVQSIENTSKSHGEIFK
P08861	MVCAGGDIRSGCNGDSGGPLNCPTEDGGWQVHGVTSFVSAF
Q9R0X0	EYPLSCFALFENGPCLIADTNFDVLMVKLKGFFQSAKASKI
Q6PIF2	SLDSSIDILQKRAQELIENINKSRQKDHALMTNFRNSLKTK
Q32L52	GQIQEAIALINSLHPELLDTNRYLYFHLQQQHLIELIRQRE
Q9NYN1	APLTARHGLASCTFNTLSTINLKEMPTVAQAKLVTVKSSRA
Q9Y5N5	PLVPDLLSPKGLFYLVTIKENNPEEILKIMKTKGLQGTTAL
Q3T0G5	TKPADMVIEAYSHGQRTFGENYVQELLEKASNPQILSSCPE
Q693B1	ARRGPHHYELSSVQVDTFRANLFCTDSECLGALRARFGVAS
Q00724	HWIIDTDYDTFALQYSCRLQNLDGTCADSYSFVFSRDPNGL
Q8BHH8	GLFRGTSAQELNEGLQDLDKNFEVLGILDNSSQKSLHLVVI
Q9UPY8	GRLSNVAPPCILRKNPPSARNGGHETDAQILELNQQLVDLK
O75431	TILTTQLTNDELSEKVKNYSNLLAFCRRIEQHYFEDRGKGR
Q9D676	LPATKTEQILINKTYAACPKNWIGVGNKCFYFSEYTSNWTF
Q9CQ07	RLTTNGLPVELNQLKNIRTVNLGLNHLDSVPTTLGALKELH
Q9X2V8	KKGMAWIFISNKKENRLYSLNEEHLIRKEISNLSIIFHLNI
P70379	QESGRAWFLGLNKEGQVMKGNRVKKTKPAAHFLPKPLEVAM
Q60652	NKSIDCSPGEELLESLNREQNRWYSETKTDLDSSQDTGTGV
P49716	SAGKRGPDRGSPEYRQRRERNNIAVRKSRDKAKRRNQEMQQ
P78345	PRVPSLSVPWLQDRIEDSGENLETEPLESQDRELLDTSFED

Q7TSN2	KTSSSEEVRNGRVTIRDHPDNLTFTVTYESLTLEDADTYMC
P47753	EDGNVQLVSHKDVQDSVTVSNEVQTTKEFIKIIESAENEYQ
Q9ESN5	NVLNSDVENQVVTLTESRIFNELTTKIRGIKEFKEKLLLTL
P07318	IKMDAQEHKLCLFEGANFKGNTMEIQEDDVPSLWVYGFCDR
P0CI32	YQRAVDQILTRSGKLDCFIGNAGIWDHNASLVNTPAETLET
P62258	LKLICCDILDVLDKHLIPAANTGESKVFYYKMKGDYHRYLA
Q15102	VQLMHQCEIWRELFSPLHALNFGIGGDGTQHVLWRLENGEL
A5PKI3	WVFCGGKGIKTKSPFEQHIKNNKDTNKYEGWPEVVEMEGCI
Q8BJ83	ECSNSTTCMTVACPRQRYFANCTVRDHIHCLGNRTFPKLLY
Q6QUN5	VGKYFLEDIPSEGMLQIQMANLQVEDSGLYRCVILGPSDPI
Q8BH50	LSPSSSGHLADSDTLSSVEENEPSQAETTVEGDTSGVSGAT
Q28145	KFKKMFGWGDFHSNIKTVKLNLLITGKIVDHGNGTFSVYFR
F7BWT7	NTTDVVNTMCGYKTIDKERLNAQNIIHVRGCTNAVLIWFMD
Q96NF6	RVWWRLPVIPATREAEDNRLNPEGRGCGEPRSRHCTPAWTT
P54130	ELYGSEKLTQECVFREQFEENWYNTYSSNLYKHVDTGRRYY
Q8WXB1	HLCSNHSVILLACRIRYERDNNFLAMLERQFTVRKVHYDPE
Q9R1P3	ANFTRRNLADCLRSRTPYHVNLLLAGYDEHEGPALYYMDYL
P25402	RVKLIYRPIALKNGRDEAENNIKLINSGTDSCLENTTPYYF
Q62283	SPYFLDHGIPPSCCMNETDCNPLDLHNLTVAATKVNQKGCY
Q148G4	LPMFLHCRNSHAEFLDIMRRNRDRCVGGVVHSFDGTKEAAA
Q9JJU9	LLSLRPLHIDGPDHKLHLFENPAFSGRKMEIVDDDVPSLWA
O55126	DSWLKSLFVRKVDPRKDAHSNLLAKKETSSLYKLQFHNVKP
Q3SWZ3	AQLKAIEPQKEEADENYNSVNTRMRKTQHGVLSQQFVELIN
Q64105	ATLGDVSKGFLNVNDLAEVNNYWALNLTSMLCLTSGTLNAF
Q8N402	YDICNEDAVDISDEDTVDISNEASVHDISNEAAVCDISNDA
Q1JQA8	CIFLKKTLNIPVISEKPLLFNGLNSVDSPENETVDSFSHEE
P20774	LYARFNKIKKLTAKDFADIPNLRRLDFTGNLIEDIEDGTFS
Q15404	EILPPDIGKLTKLQILSLRDNDLISLPKEIGELTQLKELHI
P01131	AIFEDKVFWTDVINEAIFSANRLTGSDISLMAENLLSPEDI
Q9DCC7	LDRGLQVHVAVDACSSQSEMNRLVALARMQQSGVFLSTSEV
P01731	KITWDEKLNSSKLFSAMRDTNNKYVLTLNKFSKENEGYYFC
Q96DN0	HYNITGNTICLFRLVDNEQLNLEDEDIESIDATKLSRFIEI
P49891	PRIVKTHLPPKLLPASFWEKNCKMIYLCRNAKDVAVSYYYF
Q2HJH7	GRHPIGVLLNAITELQKNGMNMSFSFFQQASWRRRMGAAAV
Q28203	VIPVTMGVLFAVLLVSACIRNITKKRQAKALHPTAERQDPV
Q3MHR0	ALSCWLPLRASFPQGPIGGVNRDISILQCHGDLDPLVPLMF
P15692	SERRKHLFVQDPQTCKCSCKNTDSRCKARQLELNERTCRCD
Q96E11	VPIPQVTREHREMLVKLAKQNTNKAKDSLRKVRTNSMNKLK
Q17QJ5	LGIAFLGIGLWAWNEKGVLSNISSITDLGGFDPVWLFLVVG
Q3UFF7	HLAYRSHPDVAGVFVLSGFLNKASVVYQDLQQGGRMLPELF
Q8N2U9	RRTQNADGFSTYVCLVLLVANILRILFWFGRRFESPLLWQS

Q60660	EMLRNKSSECKALNDSLHYLNREQNRCLRKTKIVLDCSQNK
P70190	RCVNLHIEVSKDLKESLEVVNQAQLALSFARLVEEQENKFQ
Q96M98	TFPYEFFARQGIHDMLEHGGNKILPVLPQLIIPIKNALNLR
Q14249	ATNADYRGSGFDRGHLAAAANHRWSQKAMDDTFYLSNVAPQ
Q1RMJ7	GYCFVEFADLATAEKCLHKINGKPLPGATPAKRFKLNYATY
Q80WY3	GATAARLLKPELQVCVFCRNNKEAVALYTTHILKGPDGRVL
Q9CQ06	SSPFYRYGMSRPGSIADKRKNPPWSRRRPVVVEPISDEDWH
Q9BY08	RSPNLNTSNWLYCWLYLFFFNGVWVLIPGLLLWQSWLELKK
Q58DV7	MRSLFTDHSRYVESFRRFLSNSTEHQCMQEFMDKKLPGIIA
Q86WA6	PRAGPAAAFGTSVTSAKVAVNGVQLHYQQTGEGDHAVLLLP
Q2NL23	ASIPFLEDFCIEEDEEEAGDNQAWEEPLNWNTGTRNLTPPR
Q2KJ84	LLRAFEARDRNIQESNFDRVNFWSMVNLVVMVVVSAIQVYM
Q96BI3	QMAYVSGLSFGIISGVFSVINILADALGPGVVGIHGDSPYY
Q6P587	LEEGDIILTGTPKGVGPVKENDEIEAGIHGLVSMTFKVEKP
Q8NH89	GSINASVHTGFTFSLSFCKSNKINHFFCDGLPILALSCSNI
O35980	IANRLRWTKKMTKTPEETRKNLEEWLPRVLWSEVNGLLVGF
P24668	HIFNGSNWIMLIYKGGDEYDNHCGKEQRRAVVMISCNRHTL
Q99714	LATAERLVGQGASAVLLDLPNSGGEAQAKKLGNNCVFAPAD
Q9HA38	RKEGNEFKMMPNRRNMYTVQNNSAGPYFNPRSRQRIPRDLA
Q2KJ37	ETITTHYLFFLGLYRALYLVNWIWRFYFEGFFDLIAVVAGV
Q9Y5K2	DQEPGSQMVEASLSVRHPEYNRPLLANDLMLIKLDESVSES
A6QQC0	IRESGAKHKGLIEIPSLSEENEVDDTEVNVSKKKGDGDILK
Q96HS1	PAEPPAWAGGARPGPGVWDPNWDRREPLSLINVRKRNVESG
Q9JL95	RCYRGNLASVHSYSFNYQIQNLARKINQSIVWIGGILRGWF
P19437	QTIKMKEEIIELSGVSSQPKNEEEIEIIPVQEEEEEEAEIN
Q9TQE0	FECHFFNGTERVRYLHRGIYNQEENVRFDSDVGEYRAVTEL
P0C8J7	GICKINVATELKNAFSQALKNYLTAHPEATDPRDYLQSAKS
P15948	GIDAAPPVQSRILGGFKCEKNSQPWQVAVYYLDEYLCGGVL
Q9BPZ2	RVLSLKILSDRVASSHISDANLANTIIGKAVEHMFEGEHGS
P41272	GTSFSPDYHTRPHCESCRHCNSGFLIRNCTVTANAECSCSK
Q3T0A9	CMISHGRKVDPWVCPDFCCGNCNDQYCCSDVLKQVMWIEED
Q3MHR2	TRIKKISIEGNIAAGKSTFVNILKQVCEDWEVVPEPVARWC
Q17QJ0	VLENPSPFHSPFRFEISFECNEALADDLEWKIIYVGSAESE
Q02739	AFYPRYTLPSMVKCHAEPCPNTVDCFIAKPSEKNIFIVFMV
Q9NQT4	SITVVLQVVSDAGSLLACCLNAACMALVDAGVPMRALFCGV
Q9UH92	KGSVVSRANSIGSTSASSVPNTDDEDSDYHQEAYKESYKDR
P46412	LFEKGDVNGEKEQKFYTFLKNSCPPTAELLGSPGRLFWEPM
Q00535	MTKLPDYKPYPMYPATTSLVNVVPKLNATGRDLLQNLLKCN
P17322	WNFCQAGKELRDQDSMEKAWNNQKRGKDCSKLGEKCLHQQL
P10948	GVKDASDQNFDYMFKLLIIGNSSVGKTSFLFRYADDTFTPA
Q9QZE7	TPFEVSQFLRQVYDGFSFIGNTGPYEVSKKLYTLKQSLAKV

P43276	VSLPALKKALAAGGYDVEKNNSRIKLGLKSLVSKGTLVQTK
Q9Y696	KGVVFSVTTVDLKRKPADLQNLAPGTHPPFITFNSEVKTDV
O60573	EEICGAVVSVRFQEDIISIWNKTASDQATTARIRDTLRRVL
Q9H063	NCSLFSAVREDFKDLKPQLWNAVDEEICLAECDIYSYNPDL
Q3T165	CRSRPRNVPVITGSKDLQNVNITLRILFRPVASQLPRIFTS
Q6GV28	VCGVLSLLECKLSTSSCTCLNIHKSDNECKESENSIEDISL
Q9H3L0	MTVWSEEVEIEREVLLEKFINGAKEICYALRAEGYWADFID
P05631	TISSAESMSIYDDIDADVLRNYQEYSLANIIYYSLKESTTS
P63104	TELRDICNDVLSLLEKFLIPNASQAESKVFYLKMKGDYYRY
Q17QI5	YAMNSEFSSVLAAQLKHHSENKGLDKVMETQAQVDELKGIM
P10966	TVLHGNSVLQQTPAYIKVQTNKMVMLSCEAKISLSNMRIYW
A6NJ46	RTRNCWADTGQDWRGGRQCSNTPDPLSDSIHKKKHTRPTFT
Q9Z1Q5	DEGISQRKFLDGNELTLADCNLLPKLHIVQVVCKKYRGFTI
Q3URK1	GVSSQDGERGSAPANETRSENASQKPRGDADVQNSPSSVDY
Q8TCZ2	QKKFCFSIQQGLNADYVKGENLEAVVCEEPQVKYSTLHTQS
Q9Y3B9	AMAKVLNKKTPESKPTILVKNKKLEKEKEKLKQERLEKIKQ
Q64267	ALEVWGSQEALEDAKEVRQENREKMKQKKFDKKVKELRRAI
Q9CQI4	CHEQTYEEFLSTFTHLSKEDNVAKWGAHGTDSSENIFSTVK
P26884	GDVVHCWYTGTLQDGTVFDTNIQTSSKKKKNAKPLSFKVGI
Q149F5	GYYIWTEDSFFCDPDGHITLNPSQTSVMYKENLVRIFRKKK
A2AQ14	MLCYLLKTNAIVNTSEMDFHNVPLPEKIAELKEKIMLMHTR
O14753	SGDLFTCRVCQKAFTYQRMLNRHMKCHNDVKRHLCTYCGKG
Q8CBW7	YAPDPVLVRGAGHITVFGLSNKFDTEFPSVLTGKVAPEEFK
Q9BQY4	AGVPGHLWEGDLEGTSGSDGNVEDSDQSEKEPGQQYSRPQG
P34927	QNFLQRHMGPLNTWIGLTDQNGPWKWVDGTDYETGFQNWRP
P08074	QVSQMVARDMINRGVPGSIVNVSSMVAHVTFPNLITYSSTK
Q8BGJ9	KYGEIEEMNVCDNLGDHLVGNVYVKFRREEDAERAVAELNN
Q8VCH9	DLPPETVLLYLDSNQITSIPNEIFKDLHQLRVLNLSKNGIE
P35509	LGHNTRGTGSSSSGVLMVGPNFRVGKKIGCGNFGELRLGKN
Q9H115	ATSFVDAGNAYKKADPQEAINCLNAAIDIYTDMGRFTIAAK
Q92813	LLERFSLPPQCRVVADRMDNNANIAYGVAFERVCIVQRQKI
Q7YRD0	LSRAIGRIAGKGGKTKFTIENVTRTRIVLADVKVHILGSFQ
Q9GZU0	KEKKKKSALFEVSEVIPVMTNNYEENILKGVRDSSYSLESS
Q6IPR3	VPLSHKGKLMVTEEYIDFLLNVANQKMEENKKRIERFYNCL
P04768	KKKINAVRNGVNALMSTMLQNGDEEKKNPAWFLQSDNEDAR
Q2PT27	RANLKDAIFKSCDLSMADFRNINALGIEIRHCRAQGSDFRG
P32243	LNQSPASLSTQGYGASSLGFNSTTDCLDYKDQTASWKLNFN
O89104	SAGATPSMGLANLSVLFGFINFFLWAGNCWFVFKETPWHGQ
P09661	DPLASLKSLTYLSILRNPVTNKKHYRLYVIYKVPQVRVLDF
Q6QRN8	VEVTHPNSMPAVNIQYEVIGNYYSSERMADNACVLFAVSVL
Q9D413	AGRRLSASSIAPALSSSVAENGSLLGQPWYSGNCDRQSVER

Q9BXY4	MEYIGSQNASRGRRQRRMHPNVSQGCQGGCATCSDYNGCLS
P24539	RLYRVYKEVKNRLDYHISVQNMMRRKEQEHMINWVEKHVVQ
Q14002	NIDVVPFNVAEGKEVLLVVHNESQNLYGYNWYKGERVHANY
P59544	ILIMVLFVLGNFANGFIALVNFIDWVKRKKISSADQILTAL
P63073	QANLRLISKFDTVEDFWALYNHIQLSSNLMPGCDYSLFKDG
Q9D8Z6	SEELDRALRKVVGEFKDALRNSGGDGLGQMSLEFYQKKKSR
Q91X58	ERSGCRQREMMKLTCDRCGRNFCIKHRHPLDHECSGEGHQT
Q86XI6	SLPEKIQSYERMEFAVYYECNGQTYWDSNRGKNYRIIRAEL
Q5T0L3	SPAQALPPQYQSIIVRQGIQNTALSPDCSLGDTQHGEKLRR
P54920	YSAKDYFFKAALCHFCIDMLNAKLAVQKYEELFPAFSDSRE
O08992	DGNLYPKLYPELSQYMGLSLNEAEICESMPMVSGAPAQGQL
Q9CXK4	QRTRSVDNIQFLPFLTTDVNNLSWLSYGVLKGDGTLIIVNS
O88796	ANLGLLFGLSENDGKAAVSTNCRAVFLHGETRKTAFGIIST
Q91ZW8	DWTFFNGSCYFFSKSQRNWHNSTTACQELGAQLVIIETDEE
Q9WVS0	QGSYYFCSLSIFDPPPFQERNLSGGYLHIYESQLCCQLKLW
Q3TTJ4	FCTKKVNLIHQRQDLRAKKSNRPKRLQLRWIAETSEVDAFN
Q0IIL5	RQHVDQRRQGLEDFLRKVLQNALLLSDSSLHLFLQSHLNSE
P57076	DPIRMEFENKEDLSGTQAGLNVIKEAEAQLWWAAKELRRTK
Q01730	ISLPKEIGELTQLKELHIQGNRLTVLPPELGNLDLTGQKQV
P50236	ELDLVLKYSSFQAMKENNMSNYSLIKEDRVTNGLKLMRKGT
Q9Y3B8	ASHRALDDISESIKELQFYRNNIFKKKIDEKKRKIIENGEN
Q5JBG6	DSDRIYFSKYLNVEVINVEKNCWPLNTLLRFSYFLKVIDKL
O14925	ELAFFTIGGCCMTGAAFGAMNGLRLGLKETQNMAWSKPRNV
P00760	QFISASKSIVHPSYNSNTLNNDIMLIKLKSAASLNSRVASI
O69395	SETQKQLLNQPVEIKPADLVNYNPIAEKHVNGTMTLAELSA
Q2KJD9	LRKNNHQITVLHVYHHASMLNIWWFVMNWVPCGHSYFGATL
Q9D7U6	DSFLALPAELQVSRVLMSLCNGLGLLGLLASGCGLDCLRLG
P62753	RPRRTGERKRKSVRGCIVDANLSVLNLVIVKKGEKDIPGLT
O08543	VRPTNSCMKTIGVHDRVFDVNDKVENSLEPADDTVHESAEP
Q6IR41	FDMATGSFVAPLRGLYFFSLNVHSWNYKETYVHIVHNEQAV
A4FV08	KKLIEYYKNGDLSFKYVKTFNMDEYVGLPRDHPESYHSFMW
P16035	CYISSPDECLWMDWVTEKNINGHQAKFFACIKRSDGSCAWY
Q07699	AEMIYCYKKIAAATETAAQENASEYLAITSESKENCTGVQV
Q96BS2	RAFFDNRNLRKGPSGLADEINFEDFLTIMSYFRPIDTTMDE
Q96CJ1	RVEGSSKIQYRKEQQQQQMWNSARTPNLVKHSPSEDKMSPA
P16562	SNMLKMEWSREVTTNAQRWANKCTLQHSDPEDRKTSTRCGE
P55258	IEEHASADVEKMILGNKCDVNDKRQVSKERGEKLALDYGIK
Q9NX40	PDPNLEESPKRKNITYEELRNKNRESYEVSLTQKTDPSVRP
Q8BK84	RYGLQKAGFTHVLNAAHGRWNVDTGPDYYRDMAIEYHGVEA
Q9UFF9	NFKFNLTEDMYSQDSIDLLANSGLQFQKHEEEGIDTLHFAE
Q8N488	RPRLKNVDRSTAQQLAVTVGNVTVIITDFKEKTRSSSTSSS

Q5E9H1	EESYGKYLRRESHQIGDAYSNSDKSLTELESKFKQGQEQDS
P56371	KKFKDDSNHTIGVEFGSKIINVGGKYVKLQIWDTAGQERFR
Q6ZUI0	DPMPRQISRQSSVTESTLYPNPYHQPYISRKYFATRPGAIE
O18879	AQIKERTTNRYFPAFEKVLKNHGQDYLVGNKLSKADIHLVE
Q5E9Z5	TEKTVFTIFMISASVICMLLNVAELCYLLLKVCFRRSKRAQ
Q58D49	RRVVPLVQTGETDANVVKFLNRLSDYLFTLARYTAMKEGNP
Q7TST3	LVYVEAFSQHAITGRALLRLNADKLQRMGLTQEAQRQEVLQ
Q9Y5N6	AASWMKCPLDRAYLIKLSGLNKETYQSCLKSFECLLGLNSN
Q9D7K5	SLAGCPRISERGLACLHHLQNLRRLDISDLPAVSHPGLTQI
Q9NQ48	LNEGGTAELLNKEILRLQEENEKLKSRLKTIEIQATNALDE
Q32LJ6	FADNQIPLLVIGTKLDQIHENKRHEVLTRTAFLAEDFNAEE
Q3UG98	AEKWQAQPRPPEESCMVGDVNLFLTDLEDPTLGEIEVMIAE
P01895	ERDRAYLEGECVEWLRRYLKNGNATLLRTDPPKAHVTHHRR
P0C002	GLPVKDLGPASLAAELHAIGNGADYVRTHAPGDLRSAITFS
Q9NYW5	DSSSVWFVTLLNILYCVKITNFQHSVFLLLKRNISPKIPRL
P09056	PITPVNATCAIRHPCHGNLMNQIKNQLAQLNGSANALFISY
Q9DAZ2	SETAKLFTEFNNQYAQGKRYNDRIPGTCHTEFFDTPVNKEQ
Q9BWV2	ALTSIIYASYAALIYLAVCVNAVLKKVKNIFQEEESIRQNR
Q8N6F8	AIPELHVTKPGGLVCLTTRTNSSNLQYKEALEATLDRLEQA
Q96CS7	YDDQTRQNIEDKVHMPMDCINIRTGQECRDTQPPDGKSKDC
Q9JKY0	AAPVPTALAQVDREKIYQWINELSSPETRENALLELSKKRE
Q9NQM4	SKKDSSTGCCSELVAKIKLPNTNPSDIQIDIQETILDLRTP
P13598	FEVHVRPKKLAVEPKGSLEVNCSTTCNQPEVGGLETSLDKI
P21266	DGKNKITQSNAILRYIARKHNMCGETEEEKIRVDIIENQVM
Q5QR91	RVTVHLVYSERRPKVKYIMKNLPVITDLPRNSTASPRCHLR
P01909	VFSKSPVTLGQPNILICLVDNIFPPVVNITWLSNGHSVTEG
P38117	KIRVKPDRTGVVTDGVKHSMNPFCEIAVEEAVRLKEKKLVK
Q8N4C7	ITKAQLSEIEQRHKELVNLENQIKDLRDLFIQISLLVEEQG
Q8VEE0	MLDSGADYLHLDVMDGHFVPNITFGHPVVESLRKQLGQDPF
Q9DCQ2	GPRTVLYEGPVRGLCPLAPRNSNTMAAAALAAPSLGFDRVI
P28072	DTQAVADAVTYQLGFHSIELNEPPLVHTAASLFKEMCYRYR
P17981	YEGCVEDMEKKAPQLLQEDFNMNEELKLEWEKAEIKWKEIK
Q32NC0	CNRTVKHHGKSRSFVSTLKSNPATPTSKLSLKTPERRTANP
Q9NYW5	TCLYITLSQASPFFPELVTTRNNTSFNISEGILSLVVSLVLS
Q9NP55	SLTNALSNGLLSGGLLGILENLPLLDILKPGGGTSGGLLGG
P04370	RTTHYGSLPQKSQHGRTQDENPVVHFFKNIVTPRTPPPSQG
Q9JMG7	LFPYKEYKDKFGKSNKRKGFNEGLWEIENNPGVKFTGYQTI
Q9CWP9	PGPSTGAEQYSPRFIWELDINMLGPFPGSPLQPLPPSPSRN
Q9MZ13	GWLAGYQMSFDTAKSKLSQNNFALGYKAADFQLHTHVNDGT
Q14B24	QTFSIAAAVSHPDFQPATQANDICLLRLNGSAVLGPAVRLL
Q3UUI3	DQFMKKCEDGSWKRMPSHRQNPTRAIQEFQTLFVDSKFKKE

P43080	KNLSPSASQYVEQMFETFDFNKDGYIDFMEYVAALSLVLKG
Q8IZ81	ATHVVQSEVDKYVDDIMKEKNINPEKDASFKICMKMCLLQI
P31783	VGTCGVLLLSLVITGICYRRNRRRVCKCPRPVVRQGGKPNL
P57729	AKENINIDEASRCLVKHILANECDLMESIEPDVVKPHLTST
Q15661	QFYTAQIGADIALLELEEPVNVSSHVHTVTLPPASETFPPG
Q80ZQ5	FGGCGLHFPTLADLIEHIEDNHIDTDPRVLEKQELQQPTYV
Q2HJF2	EDPALWPLLHFRSLTELKKDNFLLSPALRSLSICWHSSRVQ
P0DMB1	KPSISGAPHLNSYQSLELPQNQQDSGTEELMIVLEQGTEVR
Q2YDK0	LVEKPFAAKDERLLCSECYSNECSSKCFHCKKTIMPGSRKM
P14436	ANEAPQATVFPKSPVLLGQPNTLICFVDNIFPPVINITWLR
Q5HZI9	TPFERVQTLLQDHKHHDKFTNTYQAFRALRCHGIAEYYRGM
Q3T0D3	YQPASPPRDACVYNSCYCEENIWKLCEYIKNHDQYPLEECY
Q3ZC48	LCVATIYVRYKQVHALNPEENRIIRLNKAGLVLGLLSCLGL
Q3LI83	TNVSPSPSCSPSTQTNGYVCNCHIPTRNASKACQTLRNGSN
Q8VE10	KRVSGLEPATVDWAFDLTKTNMQTMYEQSEWGWKDREKREE
Q9D9S2	LSFFAGCLLFYAIIVYHHKLNKGQYVYFVNYKTKWIAFTVY
Q5M8M2	PRGPIAALYRGPGPKYKLPTNTGYKLHDPSRPRAPAFSFGS
P06717	FGVIDERLHRNREYRDRYYRNLNIAPAEDGYRLAGFPPDHQ
Q9NZH5	KATRKALGTVNRATEKSVKTNGPRKQKQPSFSAKKMTEKTV
Q2QD12	YTFHLEATENPGTLIKDIRENGMKVGLAIKPGTSVEYLAPW
O95297	NPPDIVVQPGHIRLYVVEKENLPVFPVWVVVGIVTAVVLGL
Q8TAF8	GDSVNTPQAGYFGLFSYCVGNVLSSELICKGGPLDFSSIPS
Q9BXJ1	FFSLNVHTWNQKETYLHIMKNEEEVVILFAQVGDRSIMQSQ
Q17QL1	RLVILDNYDLASEWAAKYICNRIIQFRPGQDRYFTLGLPTG
A1XBS5	ELQRAAMDASRTSRHLEETINNFERQKMKDIKTIFSEFITI
Q9BQE9	NGFPSDASANSSLLLEFQDENSNQSSVSDVYQLKVDSSTNS
Q9Y680	SCHRTTLHVLKCMYLLVLNNNTCAEGKIPPDATLIFEIELY
Q8C767	VCLENCVLKDKAIAGTVKVQNLAFEKVVKIRMTFDTWKSFT
P06730	SKFDTVEDFWALYNHIQLSSNLMPGCDYSLFKDGIEPMWED
P00766	PWQVSLQDKTGFHFCGGSLINENWVVTAAHCGVTTSDVVVA
Q8K5B8	NPGFVGPLVNIHTGDTFYFPNFRASGAQLPGLPSLSYPRRD
Q3T0L1	HLEKVDIKTSVLMDNMKQILNLNKLIMKSQQETWDLEEKLL
Q2WG77	EEEEEEEDDEEEEEDEDKDVNENEPEVCMGVSEATTHKATA
Q9D7L8	NGFQTVEENSDVSLVCNVKSNPQAQMMWYKNNSALVLEKGR
P0C6A0	GIRYKKYGTRCSSCWLVPRKNVQPKRLCGRCGVSLDPIQEG
Q3UFY0	ELLRLQGQGRPKAHKQLVAGNSTRTRSPQQPVCVADKHRPL
A2A8T7	TEPEDQNSDSLWELDLSEGRNFVVQDSSPRGEASDLLQHVL
P19652	WVLTVLSLLPLLEAQIPLCANLVPVPITNATLDRITGKWFY
Q8BJF9	QTMQNFQKENMKMEMTEEMINDTLDDIFDGSDDEEESQDIV
P01881	FPESWDSQSSKRVTPTLQAKNHSTEATKAITTKKDIEGAMA
Q9D7I5	SNPNCVVIADAGEAFSYQNMNRAFQVLMELENPVLISLGKG

A2A8T7	QWVEVMRAATFTYSPLLYWINKRRYHGMNVAINTGPPPAVT
P29020	VIITALYLRAKFSRSAETAANLQDPNQLYNELNLGRREEYD
A6NMD0	VIEVYPDTTEVNDYYLWSIFNFVYLNFCCLGFIALAYSLKV
Q9NUL5	NRREPHVPGTSCAHPKSRKQNHLPKVLHPSNPHISSGSTVA
Q9H4A6	WIELLSGETWNPLKLHYQLRNVRERLAKNLVEKGVLTTEKQ
Q9ULR0	AEKWRRQIIGEISKKVAQIQNAGLGEFRIRDLNDEINKLLR
Q1LZE2	FPQLPVVTHTRQVPSKPVDHNTITSWVSPQFDTTAESWFPG
Q9Y2W7	LIYAQFFPQGDATTYAHFLFNAFDADGNGAIHFEDFVVGLS
Q8CEG5	SFLTEVTDVYEMEGGLLNLLNDFHSGRLQAFGKECSFEQLE
P04230	RWFRNGKEEKTGIVSTGLVRNGDWTFQTLVMLETVPQSGEV
P07360	QKPQRPRRPASPISTIQPKANFDAQQFAGTWLLVAVGSACR
Q6P1N9	FMITGGNLQDSKDALHLAQTNGMFFSTVGCHPTRCGEFEKN
P01193	IDVWSWCLESSQCQDLTTESNLLACIRACKLDLSLETPVFP
Q497H0	KCFADFQKKQPDDDSTPSTSNSQSDLFSEETTSDNNNTSVT
Q3T116	PVDRLTISYCRSSGPGGQNVNKVNSKAEVRFHLASADWIAE
P51150	EFLIQASPRDPENFPFVVLGNKIDLENRQVATKRAQAWCYS
Q32PI9	KEIFVANGTQGKLTCKFKSTNTTGTLTSVSWSFQPEGTDTT
Q8BIL2	MRGLMLVWEEFFDELKQTKRNAKVYEKMASKLFEMTGERRL
Q96Q80	VWRLVTNFLFFGPLGFSFFFNMLFVFRYCRMLEEGSFRGRT
Q91YP1	QLFPPEGPEKRPILGLDVGCNSGDLSVALYKHFLSPRDGET
Q96PY0	RRGCGDSGSSGMAQRAQAGSNQSRGKCGRDGRCPPRSSPGA
A6H773	VRCSHTQLDKSEDGRLIYTGNLARTVFGVKCFSYSTSLISL
P56915	AKWRRQKRSSSEESENAEKWNKTSSSKASPEKREEEGKSDL
Q3SZD7	LSRIHARKLSEQRGGDKILLNACCPGWVRTDMGGPKASKSP
P08505	CMNNDDALAENNLKLPEIQRNDGCYQTGYNQEICLLKISSG
Q5T036	LPPKQALASAARNLCRGAGCNRQAVAGQLLPSTWSLHAHGL
Q9EQF4	LTPACRKHFIQAICFHECSPNLGPWIQPVVPNGQEEQRVWG
Q9JJ69	RRELQVLYRGFKNECPSGIVNEENFKQIYSQFFPQGDSSNY
P28067	FGPTFVSAVDGLSFQAFSYLNFTPEPSDIFSCIVTHEIDRY
O60921	NLSRALKTAQNARALKIKLTNKHFPCLTVSVELLSMSSSSR
P28069	LPLIMHHSAAECLPVSNHATNVMSTATGLHYSVPSCHYGNQ
Q9D061	LYLYARFKQVKVGNCNTPKPNFFDFEGKQKWEAWKALGDSS
Q9QXV3	SEAITQADKPNNKRSRRQRNNENRENASNNHDHDDITSGTP
O88878	GSNSPTSDSASVQRADAGLNNCEGAAGSTSEKSRNVPVAAL
Q9D8P7	TMKKLSMTRTLLTEKKEGEENIDGVEWLQMKDNDFSYRPNM
O95475	KRELAQATGLTPTQVGNWFKNRRQRDRAAAAKNRLQQQVLS
Q9TT89	ASSVFATGAFPEQTSCAFASNPQRPGYGAGSGASFAASMQG
Q8IYP2	QTVNISVISKPQCRDAYKTYNITENMLCVGIVPGRRQPCKE
O95843	QGLNQKANKHIDQVYNTFDTNKDGFVDFLEFIAAVNLIMQE
O43247	PPTAASRDSLGMDPQSRSLKNAGSRSSSRENRATSGEGAQP
Q8R5M0	EGSIINRIEAVCVGDSIEAINDHSIVGCRHYEVAKMLRELP

Q9CQE6	EFIRVGYYVNNEYTETELRENPPVKPDFSKLQRNILASNPR
Q9BGI1	KVGDAIPSVEVFEKEPGNKVNLAELFKGKKGVLFGLPGAFT
Q9Y3Q3	DSQTHYRLREAQDRARAEDLNSRVSYWSVGETIALFVVSFS
Q9CYH2	DLKTLEKEPRTFKAKELWEKNGAVIMAVRRPGCFLCRAEAA
Q9MZ06	PEKTQTMATKDPQCEEEDLKNQRKAALEYCGETWGSLCNFF
Q8N3Z3	RNLKRTFLLVDSVVGIQKTDNIAIEMCEEFALPYVIVLTKI
Q9BRK0	ITQARDKSYETMMRVGKRGLNLAANAAVTAAAKGVLSEKLR
Q70IB2	ISEDEVGGDRMLRAEVLLHSNKDYLRSDVMDRECNALMALK
Q9CWJ3	AVIKRWWSELSSTSEIISDGNIKILKQQLSGLWEQESHLTL
Q61133	ELYLDLLSQPSRAVYIFAKKNGIPFQTRTVDILKGQHMSEQ
Q1LZ75	KLEQDFVSRVTECLTTVKSVNKTDSQTLLTTFGSLEQLIAA
Q91W61	VYLAQRRGAGLRSLSLAVNANVGDTAVQELARNCPQLEHLD
Q64310	AIGFKTKLAALTLVVWLFAINVYFNAFWTIPVYKPMHDFLK
P28845	KFALDGFFSSIRKEYSVSRVNVSITLCVLGLIDTETAMKAV
Q96B36	SVPVWGFKEKRTEARSSDEENGPPSSPDLDRIAASMRALVL
Q3T0Z4	ITNTTANNWRVYNLHFNSIENQHRIIHFSVFLGLLLVGILE
Q2T9Y1	ELIKQGGLHLNPLKFLIFDWNWRDQKLRRMMDIPEIRKEVF
Q3SY17	ALKCHGIGEYYRGLVPILFRNGLSNVLFFGLRGPIKEHLPT
Q2HJI0	PQRRESRFLSPEFIPPRGRTNPLKFQIERKDMLERRKILHI
Q6ZWZ2	RTILLSVISLLNEPNTFSPANVDASVMFRKWRDSKGKDKEY
Q61206	LKNGELENIKPKVIVVWVGTNNHENTAEEVAGGIEAIVQLI
Q0P5D3	SIKPQELLEWELVVLGKLKWNLAAVTPHDFIEHILRKLPQP
Q9NQX7	SSQVRTQMELEEDVKIYLDENYERINVPVPQFGGGDPADII
Q96AZ1	DVTITDLPLALEQIQGNVQANVPAGGQAQVRALSWGIDHHV
Q969Q5	GKTSLVERYVHDRFLVGPYQNTIGAAFVAKVMSVGDRTVTL
Q3TBL6	NLALQAQKKILSKIASKTVANMLIDDTSSEIFDELYKVTEI
Q96MU5	LLGDHRCLVPFRDLNPSSEVNTANLLESPSSLLLTSCYICS
Q8NE22	DGVLIDGNDKGISKVVYRSCNGRDRLGPLKMSDSTWLTSEI
P19639	RYVMGDAPNFDRSQWLSEKFNLGLDFPNLPYLIDGSHKVTQ
Q62193	AGRPSMSNPGMSESFNFSGNNFMPANRLTVVQNQVLNLIKA
Q9TQE0	LAGDTQPRFLKQDKFECHFFNGTERVRYLHRGIYNQEENVR
Q8R092	RQIMRFAMKSRRGPHVPVGHNAPKDLKEEIDIRLSRVQDIK
Q9CR58	TVATHFLSSFTCGLVGALASNPVDVVRTRMMNQRALRDGRC
P56880	LALSGVSGVLTATLLPNWKVNVDVDSNIITAIVQLHGLWMD
P41217	VTQDEREQLYTPASLKCSLQNAQEALIVTWQKKKAVSPENM
Q9Z1P5	QSALPCSCDNISGCSDVSDKNLNCSRPPCQESELHCILDDV
Q3T0C6	QGYIDDLKKFLKPYGLEEQKNLTDCTNGTFFEQKGPEYTAC
Q9BXY0	NAIEKELLERLKQDTYGDIYNFPIHAFDKALEQQEAESDSS
Q8N4E4	LEELEWKLAEVGAIQTDLEENPRKDMVDMMVSSIRNTSIHD
Q9H5J8	SISPIEESTAEDEDATHLEDNECDIKLAGDSFIVSSEFPVR
Q9BTT0	NIISGGLEVLAEKCPNLTYLNLSGNKIKDLSTVEALQNLKN

Q3SWZ3	FTFLATEVEMQGEMINRIEKNILSSADYVERGQEHVKVALE
Q5SS90	SHQREVPVVSIPDYMVYEEFNPDQANGSYESRQGPFDFDRK
A2VDP0	LSSNLPTNNKMEHGNNDNKQNHDLTHRKSPLGPVRSPPLSP
Q91UZ4	VRHVDNPNGDGRCITCIYYLNKNWDAKLHGGVLRIFPEGKS
P11034	EVELRIMDKEACKMYKHYDYNFQVCVGSSTKLKTAYMGDSG
Q57239	TASVLVPVWRQHTLALITTKNMNPAMVAEAAKAHQALMRSL
Q9ZIS7	RSAGLTFPNDFYFLAERKIFNYASVFIMLIEYVEGVELNDM
P01584	KLEFESAQFPNWYISTSQAENMPVFLGGTKGGQDITDFTMQ
Q9CXW3	RSFDLLVKNLNGKNYSMIVNNLLKPISVESSSKKVKTDTVI
Q96GD0	APELLERLARAGKAALFVSNNSRRARPELALRFARLGFGGL
Q9JII2	IFNMTSTISENFNNLSSETLNDFDTEYDPHQKFQNRPTMTC
Q5BKY9	AMARSRGPIQSSGPTIQDYLNRPRPTWEEVKEQLEKKKKGS
Q03084	RGGIKYAISEIKAQYHFYRANYISYQDFIINIITRIFVRLL
Q2HJB9	SELELDDVVITNPHIEAILENEDWIEDASGLMSHCIAILKI
Q96PQ5	EREKKRQFEMRRTLHYNEGLNIKLARQLISKDLHDDDKVEE
P25401	VEKDTRPTFVVTPSFFKVKPNGQQTLRIIMASDHLPKDKES
Q2HJ38	RFEPEKLREGRNIIGLQMGTNKFASQQGMTAYGTRRHLYDP
P35695	EAPEYLVHSKTGFSGVGTESNPGVAWWVGWVEKGAEVYFFA
O08547	QSGRDLQQYQSQAKQLFRKLNEQSPTRCTLEAGAMTFHYII
Q7L9L4	QEEAHLNTSFKHFIFFVQEFNLIDRRELAPLQELIEKLTSK
Q49BZ4	LSRRQTEEPWLWEDGSTLLSNLFQIRSTVTEKDSSHNCAWI
Q8N699	RSNLSLASLTFQRQASLEQANSFPRKSSFRASTFHPFLQCP
Q8BV13	AAYLQLLNLFAYGTYPDYIANKESLPELSVAQQNKLKHLTI
P04370	GTSAEDTAVTDSKHTADPKNNWQGAHPADPGNRPHLIRLFS
Q9GZU7	DLRRVLILDNSPASYVFHPDNAVPVASWFDNMSDTELHDLL
Q810N6	EHPHHEHAVQLLLEEDIVGRNLLYAACMAGKSDVIKALAKY
Q8IWL2	IAVPRNPEENEAIASFVKKYNTYAYVGLTEGPSPGDFRYSD
Q9BTZ2	RERLVATAVKLHGGIDILVSNAAVNPFFGSIMDVTEEVWDK
Q9EQT3	LCAEEVKAVSYIECSALTQKNLKEVFDAAIVAGIQHSDSQL
Q3T0W0	RVGMGLLPFFLACAVYLLVRNADQPPWVNVLLKILPVLYLA
Q9Y275	ELSLVTLFRCIQNMPETLPNNSCYSAGIAKLEEGDELQLAI
Q5VXT5	SAGATPSMGLANISVLFGFINFFLWAGNCWFVFKETPWHGQ
P32043	RSRTSYTRYQTLELEKEFHFNRYLTRRRRIEIANNLCLNER
Q8BQN5	VSMNDNFYPSVTWAVPVSDSNVPLLTRIKRDQSFTTWLVAM
P06343	PFCYFTNGTQRIRLVIRYIYNREEYVRFDSDVGEYRAVTEL
A8MUP2	SPHPVDVLGVDFSPVAVAHMNSLLEGGPGQTPLCPGHPASS
P55212	TVSHADADCFVCVFLSHGEGNHIYAYDAKIEIQTLTGLFKG
P97801	DTALIKAYDKAVASFKHALKNGDICETPDKPKGTARRKPAK
O43508	AQEPAQEELVAEEDQDPSELNPQTEESQDPAPFLNRLVRPR
Q9Z1R4	ESGGAIPKLDWDPVDSGGVKNLGVSAQGRLGTIGPEALLEK
P59542	WYATVFNSALYGLEVRIVASNAWAVTNHFSMWLAASLSIFC

Q0VCI2	KELPEEEVNDMLHQGKWEVFNESLLTEISITKAQLSEIEQR
Q9QXT5	SPSADGTRCLSKEGPSPVAPNPTAGVDSMAREEVYRLQARV
P70284	KALLSQQTHLSTLQVCNWFINARRRLLPDMLRKDGKDPNQF
F7BWT7	DFVQKKFKCCGGEDYRDWSKNQYHDCSAPGPLACGVPYTCC
A4FUA8	PPTAQVVGVLKIQVHYYEDGNVQLVSHKDVQDSVTVSNEAQ
O35381	RENVFKLLPQVMYLDGYDRDNKEAPDSDVEGYVEDDDEEDE
Q8N954	SLKRKAEEKLESYRKKIHMKNQAEEKAAEQFRMRLKNKQDE
Q80YF6	SVKFLVMDAAGPPKAETKWSNPIYLHQGKNPNSIDTWPGRR
P70202	PQMKPVQHLAWVACGYVMWQNSTEDTWYKMLKIQTVKQVQR
Q4VC05	DNSNQSSIADASPIKQENSSNSSPAPEPNSAVPSDGTEAKV
A7YWN2	HVNPRTIFASHGNFFNIKFVNSAWGWTCTFLGGFVLLVVFL
Q32KQ5	GESVYYSSYKISWIIFTAYLNVLFLFISGFLSLLQYKQPID
P15119	KERCGGFLIAPQFVMTAAHCNGSEISVILGAHNINKNEPTQ
P53811	FHSVKTKRGPLGPNWKKELANTPDCPRMCAYKLVTIKFKWW
Q6QHK4	SGGYSSTENLQLVLERRRVANAKERERIKNLNRGFARLKAL
Q6ZMJ4	QQGLTDVEVSPKVESVLSLLNAPGPNLKLVRPKALLDNCFR
P13726	AGASGTTNTVAAYNLTWKSTNFKTILEWEPKPVNQVYTVQI
O60682	RLASSYIAHLRQLLQEDRYENGYVHPVNLTWPFVVSGRPDS
P00435	KNEEILNCLKYVRPGGGFEPNFMLFEKCEVNGEKAHPLFAF
Q9H4Y5	SHRTRLVLKAKDIRHEVVNINLRNKPEWYYTKHPFGHIPVL
P62917	IAAEGIHTGQFVYCGKKAQLNIGNVLPVGTMPEGTIVCCLE
P59041	QLERERRLRARREALRKKQENQANKGTSWDDTRDATFFVVL
Q865R3	GSESRFFVSSSQGRSELHIENLNMEADPGQYRCNGTSSKGS
Q0III6	DIKKAYRKLALKWHPDKNPENKEEAERKFKQVAEAYEVLSD
Q9R1P3	YKMRNGYELSPTAAANFTRRNLADCLRSRTPYHVNLLLAGY
Q9BRN9	CPRQRYPANCTVRDHVHCLGNRTFPKMLYCNWTGGYKWSTA
P14901	PQDLSEALKEATKEVHIQAENAEFMKNFQKGQVSREGFKLV
O97563	QKMIRYEIKQIKMFKGFEKVNDIQYIYTPFDSSLCGVKLEA
P04231	SDVGEFRAVTELGRPDAENWNSQPEFLEQRRAAVDTYCRHN
Q86XK3	KHIDSEFEENTNLKNTLKNLNVCESQSLDSGSCSALQNEFV
Q02739	CHAEPCPNTVDCFIAKPSEKNIFIVFMVVTAVICILLNLVE
A6PWV3	YLEKKLEELVEMIYKDDGEKNLEFGRSNSNSLTGEPTCAES
Q9JKK1	WTELLQGPSAATREEIDWTTNELRNNLRSIEWDLEDLDETI
Q9HAN9	ESDVLWKHRSNIHVVNEWIANDISSTKIRRALRRGQSIRYL
Q9CRC9	ILITGAHKAFALYKAMEEGVNHMWTVSAFQQHPRTIFVCDE
P61028	MLVYDITNEKSFDNIKNWIRNIEEHASSDVERMILGNKCDM
P97952	VTYNHSGDYECHVYRLLFFDNYEHNTSVVKKIHLEVVDKAN
Q8N2K1	EFPFKPPSIYMITPNGRFKCNTRLCLSITDFHPDTWNPAWS
Q86VU5	TLEQPQGDSMMTCEQAQLLANLARLIQAKKALDLGTFTGYS
A6NDL7	PQVKELSWGVALDTNFPRSSNNFDYILAADVVYAHPFLEEL
Q3ZBK2	KMMFMGFVRLGVWQNFFRAWNGGFSGNLDGEGFILGGVFVM

Q9NYW4	IINLLLTVQIGLTFYHPPQGNSSIRYPFESWQYLYAFQLNS
Q2KI75	ELQEVRRGIERQLQEHEMLLNTKMRLEQEIATYRRLLEKEE
P61294	GEQSVGKTSLITRFMYDSFDNTYQATIGIDFLSKTMYLEDR
Q9JHK0	LLEGVKSILIQMQNGDTEDENYPGWSGLASLKSETEDIRLF
Q6QLQ4	AFFPNSFQVRNTAPQESLLHNCVWIHGSEVYNQICNTSSYS
Q2HJF5	MQCVAAGHQIVALANLRPAENQVGSDELDSYMYQTVGHHAI
Q9H446	DAELLEIKKKRMKEEEQAGKNKLSGKQLFETDHNLDTSDIQ
O18883	LDRHLVILSKKHLETKFLKLNVEKAPFLCERLRIKVIPTLA
Q5MJ68	TSPVVTTQVELGGCSRQGGGNGFLRFRQHQEVQAFLSLLED
Q15466	FWSLELSPKEYACLKGTILFNPDVPGLQAASHIGHLQQEAH
P78369	AGASLCIIGGVIFCFSISDNNKTPRYTYNGATSVMSSRTKY
P59034	DIPADTVLLKLDANRISRVPNGAFQHLPQLRELDLSHNAIE
P98086	TLGNVVIFDKVLTNQESPYQNHTGRFICAVPGFYYFNFQVI
Q0VD01	ILRYLFPVPKDDSRRVITFANQDDYISFRHHVYKKTNHRNV
Q8NGN7	IVIYLQPNPSALLGSIIQILNNLVTPMLNPLIYSLRNKDVK
Q6QRN6	FGFSFTGEVREPFRSILSVLNGLTVPIASIDIPSGWDVEKG
Q02242	QIIQLPNRHDFHMNILDTRRNDSGIYLCGAISLHPKAKIEE
Q9DA08	YRTKLRGLYTTAKTDAEAECNILRKALDKIAEIKSLLEERR
O55125	AVLPKLHLDEDYPCSLVGNWNTWYGEQDQAVHLWRFSGGYP
Q62232	ERENSENSNSSSHNPLASSLNGSGKSVLGSSEDEKTPSGTP
Q8BGU2	HEPSEMSNRTMTIYFDQVLVNIGNHFDLASSIFVAPRKGIY
Q5E9F2	EMTPEVRQRVLDEAKTAFLLNIQLFEELQGLLTQKAKDHDP
Q2M2E3	YFIGWLVLILYFTCAILCYFNHKSFWSLILSHPSGAVSCSS
P0C8J7	GQLGGQEDDVQVNEADAFYTNPAQAREFAEATGIDSLAVAI
Q9BRX5	LVARLDEMERGLFQTGQKGLNDFQCWEKGQASQITASNLVQ
Q3T0N1	GGFPERTLSRLGSRASTLRRNDSIYEASNLYGISAMDGVPF
P05531	LSSDEMQDGNAPELDVIEEHNPVTRDDENANPEEVVGDTRS
Q8MKH6	EKFDLMAKLKQQKYEINVLYNRISHAQKFRKGAGKGRVGGR
O88983	LLLRAVSTRQITQLEGDRRQNLLDDLVTRERLLLASFKNEG
Q8BWR2	GVIIMGEDDDSHPSEMRLYKNIPQMSFDDTEREPEQTFSLN
P20334	VIVLLLVGCEKVGAVQNSCDNCQPGTFCRKYNPVCKSCPPS
P68543	VSKAKNIEVENKNNLSAVPLNNLEPITNIQIWLANGKRIVQ
Q3V1I0	DPALIAAIISRESHGGAVLQNGWDHKGQRFGLMQLDKNMYH
Q8WTZ3	KRIHTEDKFYKYEECDKSFKNISTLITHKIIYVVEKFYKCE
P11233	LVGNKSDLEDKRQVSVEEAKNRAEQWNVNYVETSAKTRANV
P33781	FNWQGVVPSAPVTQSSWAFVNGLDIPFTPGTEQLNITLDSN
Q17RH7	RRCSISGSTKCRCGSRIAGPNALGSGGSRSSSSSSRSILSS
O54709	QDFLKLVKSYHWMGLVQIPANGSWQWEDGSSLSYNQLTLVE
P63103	TEQGAELSNEERNLLSVAYKNVVGARRSSWRVVSSIEQKTE
Q28203	KCHRWTSCERKGLVEQHVGTNKTDVVCGFQSRMRTLVVIPV
Q8K072	IVQAKERSYETMLSFGKRSLNIAASAAVQAATKSQGALAGR

P54368	GPRWCSDAPHPPLKIPGGRGNSQRDHNLSANLFYSDDRLNV
Q9CY66	GSFRGGGRGGFGRGGGRGGFNKFQDQGPPERVVLLGEFMHP
P12402	TTIMFHEFDEKYAQNQPYTINATKSCHTNSLHTPQEREKAL
P49615	KLYSTSIDMWSAGCIFAELANAGRPLFPGNDVDDQLKRIFR
Q8IX05	SIEDVRNQCTDHGADMISIHNEEENAFILDTLKKQWKGPDD
Q8BP99	CQPDLQPDNNSNFVESAKDANKNWHGVPGKVDPILIRSSSE
P37980	VKKGKLRYVANLFPYKGYIWNYGAIPQTWEDPGHNDKHTGC
O95452	LLLKVCFRRSKRAQTQKNHPNHALKESKQNEMNELISDSGQ
Q5E9J3	LGLQLASMLGCSFYEVSVSENDNDVYNAFHVLCKEVSHKQQ
Q3T093	TGRSAFIGIGFSDRGDAFDFNVSLQDHFKWVKQESEISKES
P35293	AQGVILVYDVTRRDTFVKLDNWLNELETYCTRNDIVNMLVG
Q8TBG9	MSSLNTSVVFGFLNFILWAGNIWFVFKETGWHSSGQRYLSD
O88452	QHCLVNAGDVGCGVFECFENNSCEIQGLHGICMTFLHNAGK
P06344	SLRRLEQPNVVISLSRTEALNHHNTLVCSVTDFYPAKIKVR
Q5E997	PVKIEGYEDQVLITEHGDLGNGKFLDPKNRISFKFDHLRKE
Q9EST4	FYTDCLVPMVGNNPYATEEENSNELSINTEVYSLPSKKLVV
Q96AH0	NRVNDPLIFIRDIKPGLKNLNVVFIVLEIGRVTKTKDGHEV
Q9UN19	AAEALLLSNGCDGSYLLRDSNETTGLYSLSVRAKDSVKHFH
Q9D0I8	KYLFIFSVANMRNSKLKDIRNAWKHSRMFFGKNKVMMVALG
Q32KM6	IYEGQWRNDKPEGEGMLRLKNGNRYEGNWQRGVKNGSGRFF
Q9D7J7	FTQLAGPDMEVGATDLMNILNKVLSKHKELKTEGFSLDTCR
Q08DK7	EGWRVFTRGLASTLLRAFPVNAATFATVTVVLSYARGEEAR
Q8C4A0	GSVLPWPVALPSVLQIAITFNLATATAVQISWKTSGAHANP
Q5VT99	GLPSVPDPFPLDVRKLLVAGNRIQRIPEDFFIFYGDLVYLD
Q14493	DEMRTRVNKEMARYKRKLLINDFGRERKSSSGSSDSKESMS
P70447	IKKTRRLKANNRERNRMHNLNAALDALREVLPTFPEDAKLT
A6QPM6	RSKRLFRDLVSLQVPEEQVLNAALREKLALLPPQARAPPPK
Q9NXJ0	IVSSILAFIGVILLLVDMCINGVAGQDYWAVLSGKGISATL
P37980	VVEVPRWSNAKMEIATKDPLNPIKQDVKKGKLRYVANLFPY
Q9EPA7	ESDVLWRHQSNIHLVNEWITNDISSTKIRRALRRGQSIRYL
Q9CWG1	TWPYKQGATCSDCPKDDKCLNSLCINPRRDQVSRYYSVDYP
P62996	YRRRHSHSHSPMSTRRRHVGNRANPDPNCCLGVFGLSLYTT
P11023	GQKESSDQNFDYMFKILIIGNSSVGKTSFLFRYADDSFTPA
Q14469	SRHSKLEKADILEMTVKHLRNLQRAQMTAALSTDPSVLGKY
Q80VP8	TNLFSQVQYMKAVVGSYTTTNVSLIAPRSEHLVNFTVKAEV
Q47456	LQRTGEVLPRSLISSLVWNMNFDSDTNVIDVAVRRLRSKID
D3Z423	KEAQEMAASQNKELSALRKENGELKKILDILKGSPSCYYGS
A6NGY1	HSSEKHTQRQAGSDPNPNKENSEETKLKAGNSTAGSEPESS
Q8N511	ARLEKIKIQLANEEYKRITRNVTCQDTRHGGTLSDLGKQVR
P05208	AGSAAVQVSKLVVHQRWNSQNVGNGYDIALIKLASPVTLSK
Q91X79	STEDVPETDARVVGGAEARRNSWPSQISLQYQYGGSWHHTC

Q8NBR9	LLPVPLAPHPLCLSGQLYLPNIPCTVIDGCGPVISHLKLTM
P58465	KCVVIDLDETLVHSSFKPISNADFIVPVEIDGTIHQVYVLK
Q2KJA4	SYTVVGPSSVLTYASQPLITNVTTRSAAAPTVGPPLEGPEH
Q5M956	DKSLIEEVFPEIGDVMCNSVNAGWNHDSTHVIRFPLNGYCH
Q8R1H8	PSGCPIQAKQPSGQPAPLGYNGCQEQLGLFQTPGSSPRAQH
Q15907	RAFAEKNNLSFIETSALDSTNVEEAFKNILTEIYRIVSQKQ
Q9DAY2	WEYLSFLKSSDKNNKFLAMFNLSYCIDHDSKYILLQLRLLK
Q6PER3	GCVHLRKVKFQAKLEHEYIHNFKVLQAAFKKMGVDKIIPVE
Q9BTT0	TYLNLSGNKIKDLSTVEALQNLKNLKSLDLFNCEITNLEDY
Q8BGZ2	PSPNPYQTAMYPIRSAYPQQNLYAQGAYYTQPVYAAQPHVI
Q8N801	TDTPIPGTYHLKTFIEESLLNPVIATYNFKNEGRKKPPLVQ
Q6T310	GKSAMIVRFLTKRFIGDYEPNTGKLYSRLVYVEGDQLSLQI
Q3ZBE1	EEPDALSVVNQLRDLAADPLNRRAIVQDQGCLPGLILFMDH
Q6NZB0	YVEHTVKERKKQLKKEGKPTNVEEDDPELFKQAVYKQTMKL
Q9R0U0	YSPRNSRPTGRPRRSRSHSDNDRFKHRNRSFSRSKSNSRSR
Q5TA50	GGPQSEHYRSLQAMVAHELSNRLVDLERRSHHPESGCRTVL
Q9BEG9	AVRKLQESGFYWSTVTGGEANLLLSAEPAGTFLIRDSSDQR
Q58DF9	ENEDTVSIRNLNVCKFQLSNNSVSFFLYNLDSSYASYYICK
P41247	SILPPSAHELAQNRLHVSITNAKTRENHLVSTFSSREDLIK
Q14002	NVTHNDAGFYTLHVIKENLVNEEVTRQFYVFSEPPKPSITS
P57076	LQAPGSTELEELTVQVARVYNGRLKVQRLCSEMEELAEHGI
Q99M03	LAFTRLWIYSHHIYNKCKRRNILEWAKELSLTGFSMPGKPG
Q32PF7	AGPLPGRCGTESDASPAETENEPLPPRHGAPVGGESNGGCP
Q2KI14	LRRHLELKEKGRHVVLGSIENKMEGKGNSLPSSGEACREEK
Q2NL24	PMVGNNPYATAEENSAELSINAEVYALPSKKLVALQLRSIF
Q9D4V7	NAEEINLDCTNPRSSAAGSSNAVKLSRFFDKVIEKRYFFRE
Q86TA1	ALPAPQYMNLLMDWIEVQINNEEIFPTCVGVPFPKNFLQIC
Q8NC69	DSMLGAMFGGDFPTARDPQGNYFIDRDGPLFRYVLNFLRTS
O00501	GGVLYLFCGLLALVPLCWFANIVVREFYDPSVPVSQKYELG
P09210	LDSSLISSFPLLKALKTRISNLPTVKKFLQPGSPRKPPMDE
Q9D7A8	EIYDILQSSNLADGDSFNEMNSRRRKAQFFLGTTNKRAKTV
P56965	DHRYDKLTVPDDTAANCIYLNIPSKGHVLLHRTPEEYPESA
Q5XG99	DSLNKLALQYGCKVADIKKVNNFIREQDLYALKSVKIPVRN
P20618	DCLTLTKIIEARLKMYKHSNNKAMTTGAIAAMLSTILYSRR
O70255	HMDPFRPMSGRFKDRVVWDGNPERYDVSILLWKLQFDDNGT
P19879	KLTAKDFADIPNLRRLDFTGNLIEDIEDGTFSKLSLLEELT
Q969F1	TLGTCVIFEENVEHADTEGNNKTVLKYKCHTMKKLSMTRTL
P68509	TELNEPLSNEDRNLLSVAYKNVVGARRSSWRVISSIEQKTM
A0JN87	FTATLGRHGVFNMVYFGFYFNVKNIIPVNKDPTLEFLRKFG
Q99463	VPLGFILICYLKIVICLRRRNAKVDKKKENEGRLNENKRIN
Q9CQY8	GLMAIPATTMSLAARKRACCNNKTGMFLSSLFSVITVVGAV

P97428	NSKNGVAAFHAFLKTEFSEENLEFWLACEEFKKIRSATKLA
P21311	YHLASVLEYRSSEGHPVIVSNSDTSLIRSLYRNFTHHYIKA
Q9DCX1	TIVMVQGHRDCGEDWFQPKLNYRVPSRGHKLTVTLSCGRPS
Q8R191	VVSWVFSIAVFGPIVNEGYVNSDSGPELRCVFNGNAGACRF
Q3T013	EVDALKKSVDWVSDWSSRPENIPPKEFHFRHPKRSVSLSMR
Q6DKI1	FPNLKSVRELILKRGQAKVKNKTIPLTDNTVIEEHLGKFGV
Q96HA8	ADSYLKNFASDRSHMKDSSGNWREPPPPYPCIETGDSKMNL
A2AQ14	PFLVGIEGFLKSSTYNLLFCNSCGTPVGFHLYSTHAAMAAL
Q920C4	SAMWCLGNVCGLREAITSPPNSVESGILPEGSDSENLNTQP
Q9CX62	DACLEHWQGLEAEDWTAALLNRGRSRQPLVLGDNCFADLVH
Q96F24	LLIQERWKRAQREERLKAQQNTDKDAAAHLQTSHKPSAEDA
P62821	YRGAHGIIVVYDVTDQESFNNVKQWLQEIDRYASENVNKLL
P12658	DLCEKNKQELDINNITTYKKNIMALSDGGKLYRTDLALILS
P05402	SWNNPLHHLVTELQHMKELSNAFLSSATRFENMSEKLQAFI
Q9Z2X2	NPDAKDHYDATAMHRAAAKGNLKMVHILLFYKASTNIQDTE
Q8IUB2	LESWITAGEHAKEGECPPHKNPCKELCQGDELCPAEQKCCT
Q0VCQ4	TSPGALDNITVVTADGKLPLNQISQISMKSPQLILVNMASF
P08246	HNLSRREPTRQVFAVQRIFENGYDPVNLLNDIVILQLNGSA
Q7Z4H3	RLALVHDMAECIVGDIAPADNIPKEEKHRREEEAMKQITQL
A4FUA8	TVSNEAQTAKEFIKIIEHAENEYQTAISENYQTMSDTTFKA
A6NLC5	MTPVEQLPSTEIPARPREPTNTIQISVSLTEHFLKFASVFQ
Q99M02	CTGTLGAVHKSCLEKWLSSSNTSYCELCHTEFAVEKRPRPL
Q91X52	ALLQPFLEVTKEACDTSFNVNLRAVIQVSQIVAKGMIARGV
Q9H0E2	CRLRLGYAVYETPTAHNGAKNPRWNKVIHCTVPPGVDSFYL
Q61759	QPWHVAVFRYNKYICGGVLLNPNWVLTAAHCYGNQYNVWLG
Q9NPA0	VVNTSDPDMRREMEQSMNMLNSNHELPDVSEFMTRLFSSKS
Q6Q6R3	SLVVRHLDPSPIPSDLHPLANKALGDRALSFRCEHWRCGLL
B7ZW38	ASNVTNKMDPHSMNSRVFIGNLNTLVVKKSDVEAIFSKYGK
P14438	TWLRNSKSVADGVYETSFFVNRDYSFHKLSYLTFIPSDDDI
P46737	HSLTHLDSVTKIHNGSVFTKNLCSQMSAVSGPLLQWLEDRL
Q62446	FESKRFKGTETISKVSEQVKNVKLSDDKPKDSKSEETLDEG
Q8R1L4	FALVFTTRYLDLFSNFISIYNTVMKVVFLLCAYVTVYMIYW
P01732	FSGKRLGDTFVLTLSDFRRENEGYYFCSALSNSIMYFSHFV
P25393	LSERPYVAFRLNGDILRHLKNALMIIYGMSKVDTNDCRGMS
Q9D142	RGGPGGGLAEEGELIEVIHLNLDDAQAFADNPDIPKTLGVI
Q32KU6	LVAAIIGFVFRHEIKNSLKNNYEKALKQYNATGDYRSDAVD
Q58DH1	NRHRAPFLSEKSTRQAPRGTNKTTNLSLKSNGKLRASSPVE
Q96BD8	KPPKEQRSIKEMPFITCDEFNGVPSYMKSRLTYNQINDVIK
Q32LM7	KELEISELNAKLRTQEREKQNEMIKLQLEFDAKLARVQIKT
Q01730	QLKELHIQGNRLTVLPPELGNLDLTGQKQVFKAENNPWVTP
Q0VC21	PWALDLLRALPRVSLANLRPNPGSRKPERRRRGQRRGRKCG

Q15475	HLHKNESVLKAKAVVAFHRGNFRELYKILESHQFSPHNHPK
Q3ZCC4	QTCLKRIRLDLPILHEDFVSNNDEVEENNGHDVTATELDHF
O14668	NSILKRYPRANGFFEEIRQGNIERECKEEFCTFEEAREAFE
Q9Z2C6	FVGICLFCLSVLAIVGIMKSNRKILLAYFIMMFIVYGFEVA
O55023	MEKLCSIPIHGIRSVGTAAVNMCLVATGGADAYYEMGIHCW
O75841	CCGVNGPSDWQKYTSAFRTENNDADYPWPRQCCVMNNLKEP
Q9D1D6	CSGPLPIEAIIYLDQGSPELNSTINIHRTSSVEGLCEGIGA
Q6Y1H2	SGTRKKKGPGPLATAYLVIYNVVMTAGWLVIAVGLVRAYLA
Q8R2R5	LLLKLRGLGVVDLGCLGECLNLEWLDLSGNALTHLGPLASL
Q9N0T5	KTESNQEVANPEHYIKHPLQNRWALWFFKNDKSKTWQANLR
P17026	HTGKKSHKCADCGKSFFQSSNLIQHRRIHTGEKPYKCDECG
Q9BYD2	MKNNVKWELNPEIVARHFFKNLGVVVAPHTLKLPEEPITRW
O14668	IERECKEEFCTFEEAREAFENNEKTKEFWSTYTKAQQGESN
P54368	LLEFAEEQLRADHVFICFHKNREDRAALLRTFSFLGFEIVR
Q95104	ELMGDAQLNDGALEAEARDSNEEEPKEQVKRYGGFLRKYPK
P61266	PDEKTKQELEDLTADIKKTANKVRSKLKAIEQSIEQEEGLN
Q91YN9	IGCALEDQKKIKRRLETLLRNIDNSDKAIKLLEHAKGAGSK
Q7TSP5	NANLEYKTGAFSMPEINVDYNASSESLRCEAPRWFPQPTVA
P62242	PAANTKIGPRRIHTVRVRGGNKKYRALRLDVGNFSWGSECC
O88551	IGSIVSTALPQWKIYSYAGDNIVTAQAIYEGLWMSCVSQST
Q2KIX7	VFQEVLYNHQGHFDPATGVFNCSVPGVYHFGFDIELFQSAV
Q8TCT1	LLQFVAKQGACFEVILISDANTFGVESSLRAAGHHSLFRRI
P19404	AGLTAHWGRHVRNLHKTVMQNGAGGALFVHRDTPENNPDTP
Q8N4T8	GRAVAQLMARKGYRLAVIARNLEGAKAAAGDLGGDHLAFSC
Q70IA6	ELHGHLNTLYVHFILFAREFNLLDPKETAIMDDLTEVLCSG
Q9JHL0	KGSNQEPDAAYVDPIPTNYYNWGCFQKPSEDDDSNSYENVL
Q96CS7	AGLYGQQPANQVIIRERYRDNDSDLALGMLAGAATGMALGS
Q9JHL0	YYNWGCFQKPSEDDDSNSYENVLVCKPSTPESGVEDFEDYQ
Q8JZY2	AKLQSPQAVLQLGVSKEDAKNVEKVLVEFNHKELFDFYNKL
Q9NUR3	LYRRRTFVPGKGSRKTYGSINLRMRQLNGDGGQALVENEVV
Q9UHW5	PAGSGKSTYCATMVQHCEALNRSVQVVNLDPAAEHFNYSVM
Q9JHR9	LGTSKDLQPHSVIQRRLVEGNQRRLQGESPLLQALIRGHDS
Q9NX04	DCQPLLSSLSNLAEQLQAAQNLRFEDVPALRAFPDLKERLR
P28074	SRATAGAYIASQTVKKVIEINPYLLGTMAGGAADCSFWERL
Q8CCX5	KVSQKYENENMETVTKQAVVNRDVKESAEAHGTIQTEKVDE
Q8VCH2	GIYWCGITKGGPDPMFKVNVNIDQAPKSSMMTTTATVLKSI
P59190	TELVLQAHRKELEGLRMRASNELALAELEEEEGKPEGPANS
Q80ZA7	RDDTLALSMYQGPPSADQGNNMADAPRFGFWTSVSQCLQYL
Q9BRX5	SQSLLQTFIGRFRRIMDSSQNAYNEDTSALVARLDEMERGL
Q9DCM0	TAHRDAQLIKELGLKLLYAVNTHCHADHITGTGVLRSLLPG
Q13242	LRVEFPRTYGGRGGWPRGGRNGPPTRRSDFRVLVSGLPPSG

Q15631	VEDYLSGVLILASELSRLSVNSVTAGDYSRPLHISTFINEL
Q9NV56	HLSTMYDMQALHESEILPFPNPERNFVLPEEIIQEVREGKV
Q14469	LSTCEGVNTEVRTRLLGHLANCMTQINAMTYPGQPHPALQA
Q62066	SPLRPAFPAAGPPCPALGSSNCALGALRDHQPAPYSAVPYK
Q9R0Q8	AGEPNNIVLVEDCATIRDSSNSRKNWNDIPCFYSMPWICEM
Q4FZH1	ARFLEQSSCSMTPDIHELVENIKSVLKSDEGHMEEAITSAS
Q9D1G1	SYANVKQWLQEIDRYASENVNKLLVGNKSDLTTKKVVDNTT
Q9CQE0	TRNFVSHLNQRHQFDYGEFVNLQLDEETQYQTAVEESFQVN
Q9CVB6	SKAYIHTRMRAKTSDFLKVLNRARPDAEKKEMKTITGKTFS
O70302	VLEEDGTVVDTEEFFQTLRDNTHFMILEKGQKWTPGSKYVP
Q80X71	IAEEMSYMYDFCTLLSIKVHNIVLMMQVTVTTAYFGHSEQI
O00161	RTKNFESGKAYKTTWGDGGENSPCNVVSKQPGPVTNGQLQQ
P54797	PILNKYAAVWCRCASYGTRTNTIILVDANGHVTFTERSMLD
Q5TZF3	ESEGPPESESSEFFSQQEEENEEEEAQEPEETGPKNPLLQP
Q9BX73	LCSYLPDEFIECEDPVDHVGNATASQELGYGCLKFGGQAYS
Q61469	SIGESLSVYFNVLHSNSFVGNPYIATIYKAVGAFLFGVSAS
Q496A3	FYPRFSSNIHTYHVGKQCFFNGVFLGNKRSLSERTVDKCFG
Q2KIN3	VHKASLIIDLFGNEHDNFTKNLENLMSTIQESYCSNWRCPT
O95983	GKMLMSKMNKSRQRVRYDSSNQVKGKPDLNTALPVRQTASI
Q9CZ96	DTRKSKGVAFILFLDKDSALNCTRAINNKQLFGRVIKASIA
Q8TCT1	GVESSLRAAGHHSLFRRILSNPSGPDARGLLALRPFHTHSC
Q8WUU5	AAEKKVSTKGKGRRHIFKLKNPIKAPESVSTIITAESIFYK
P59544	DQILTALAVSRIGLLWALLLNWYLTVLNPAFYSVELRITSY
O08899	HHECGLAAFKAFLKSEYSEENIDFWISCEEYKKIKSPSKLS
Q9CY66	CKCTTEENKVPYFNAPVYLENKEQVGKVDEIFGQLRDFYFS
P04233	QALPMGALPQGPMQNATKYGNMTEDHVMHLLQNADPLKVYP
P61028	NWIRNIEEHASSDVERMILGNKCDMNDKRQVSKERGEKLAI
Q9Y5M8	TGLYRDTQTSITDSCAVYRVNNNRGNSLTLIDLPGHESLRL
Q8R0A0	KLSLEGIVVQRAECRPAASENYMKLKRLQIEESSKPVRLSQ
Q1LZ79	RFAEDNDSESDDCGIVCDLSNMVITDELRQYFAETEQHREE
Q99M54	PSRDPETPQSSGSKRSRRKANSKVLGRSPLTILQDDNSPGT
Q8N1Q8	KTAFLAGEGLFTLSLNIRFKNLIPVDSLVVMDVELDKIEDQ
Q28071	AENKILVKQSPMLVVNDNEVNLSCKYTYNLFSKEFRASLYK
Q2KI30	RDKSYETMMRVGKRGLNLAANAAVTAAAKGQGVLSEKLRSF
Q9UGC6	QQSQNEGTPAVSQAPGNQRPNNTCCFCWCCCCSCSCLTVRN
Q3SZW3	WEHDYTPLCLRLIKVFVLTSNFQAIRVTLNINRKLAFLAIL
P31955	NKTEGEKSTEKPKRKKKGGKNGKGRRNKKKKNPCTAKFQNF
P52743	NATTIANHWRIHNEDRSYKCNKCGKIFRHRSYLAVYQRTHT
Q32KY3	HDRAHVIKKSKNNKTGDEEVNQEFINMNECDAHAFDDEWQN
P43487	YITPMMELKPNAGSDRAWVWNTHADFADECPKPELLAIRFL
Q66JS6	LKKLQEESDLELAKETFGVNNTVYGIDAMNPSSRDDFTEFG

Q17QK8	GTLGDVSKRWVDLTDPTEVNNYWTLNLTSTLCLTSSILQAF
Q15024	ASATPEFEGRGGDDLGTEIANTLYRIFNNKSSVDLKTLCIS
A6QQL5	KTEKGNSAESKMISPGLCRQNSQELLETKTHLSETDIRVAA
Q64326	NPYCVCYMSLFQVNGMLIMCNAVIDPFIYAFRSPELRDAFK
P32972	VVQKKDSTPNTTEKAPLKGGNCSEDLFCTLKSTPSKKSWAY
Q9Y6H3	DASTSQIVLCQNNIHNQAHMNRVVTHELIHAFDHCRAHVDW
P07516	TPPSLKAVQRIAESHLQSISNLGENQASEEEDELGELRELG
Q57239	ATIGEYGAGIDGADSLNELSNLMEHGAVAALADKISQIVAK
Q8NGY7	AINNCFLLTVMGYDHYMAICNPLRYRVITSKKVCVQLVCGA
Q9BR09	TALDPASLAPVPEFSLPDLVNLGHTWVFAITRHHNRVPREG
Q6UX82	LVLKGCSNVSNATCQFLSGENKTLGGVIFRKFECANVNSLT
Q5BVD1	SLDEEVPPAEANKESPWSSCNKNVVGRCKLWMIITSIFLGV
P20934	SVTASGSSNQNGSSRHPSDNNTNLVTPAVGHKVSATDKPAS
P11440	NTFPKWKPGSLASHVKNLDENGLDLLSKMLVYDPAKRISGK
Q9D7K5	EIQKFQNVPVEAVDASGCAINYQGLSNLLPLKELRSLSLQR
Q9NQX7	VVTEHVSDKEALGSFIYHLCNGKDTYRLRRRATRRRINKRG
Q9BY27	VLRQALRQKHQEAQQACRPHNLPVVQAAQQRELEAVEHRIR
Q86V81	MNIQLVTSQIDAQRRPAQSVNRGGMTRNRGAGGFGGGGGTR
Q61200	KDSKSFNCRIEYEKVDKATKNTLCNYDPSKTCYQEQTQSHV
Q2HJI8	IEEHASSDVERMILGNKCDMNDKRQVSKERGEKLAIDYGIK
P16368	CNADIVIRAKAVNKKEVDSGNDIYGNPIKRIQYEIKQIKMF
Q4G0X4	DSMLGAMFSGKMPTKRDSQGNCFIDRDGKVFRYILNFLRTS
Q9WV68	VDQALQEFGKINILINCAAGNFLCPASALSFNAFKTVVDID
Q8BJ73	ESRKCPIKRLCPGERNPRQKNRKDRRQRKDRKLERRPHQRG
Q99KR7	FTLKHVGPGVLSMANAGPNTNGSQFFICTIKTDWLDGKHVV
Q9R0M5	ARFRHLWKKALLRACADGGANHLYDLTEGERESFLPEFVSG
Q9UJ68	FWVLKGVYSTQVGFAGGYTSNPTYKEVCSEKTGHAEVVRVV
Q08DP3	NDSVNENSDTVGQIVQYIMKNEANVDILAAMVEDNNVCDPE
Q3ZC25	YIGVKSAYVSYDVQKRTIYLNITNTLNITNNNYYSVEVENI
P06345	YYNKQYLEQTRAELDTVCRHNYEGVETHTSLRRLEQPNVVI
P06798	EPVVYPWMKKIHVSAVNSSYNGGEPKRSRTAYTRQQVLELE
Q5HZK2	LLGVAALTTGYAVPPKLELVNESKFSSMEDPVADYNQALMT
P30907	SLKEFYGEDAKKSRDYGRIINSRHFQRVMGLLEGQKVAYGG
Q9CQD7	KDCYSGFVSTTMTPSDYMVSNAHCCQSDGCNSGSVPPPLNN
P02747	PHLGLKLLLLLLLLPLRGQANTGCYGIPGMPGLPGAPGKDG
Q9UBW8	TYADYLAEARNLPPLTEAQKNKLRHLSVVTLAAKVKCIPYA
Q9NQT4	LRPKIGLPGVAEKSRERLIRNTCEAVVLGTLHPRTSITVVL
Q8K1I3	NSQSLSPYLFRATRSSLKRVNVLDEDTLVMNLEFSVQETTC
P98086	LCLFIKSSSGGQPRDSLSFSNTNNKGLFQVLAGGTVLQLRR
Q58DU5	DGVVFGVEKLVLSKLYEEGSNKRLFNVDRHVGMAVAGLLAD
Q86T03	GKMHQHVVKCGVCNEATPIKNAPPGKKYVRCPCNCLLICKV

Q2TAM9	ADLAASHLEAIRARDEWDRQNARLRQENARLRLENRRLKRE
Q9D9P2	AEHARLSDRTAVLGILHSLANTGRFNLNLSLLSHAERESCQ
A5D989	SGPSGDHSELVTRIASLEVENQSLRGVVQDLQQAVSKLEAR
A0PK84	FSPAVLHGALFLFLSANALGNYVLVIQNSPDDLGTCQGTMS
Q2M2T8	TGRFQSQEEQRKKETACIKANNGYSKDDFCLATKKVICDPS
P41236	QPRGNVDEELSKKSQKWDEMNILATYHPADKDYGLMKIDEP
Q29463	SSWTLDNDILLIKLSTPAVINARVSTLLLPSACASAGTECL
P25787	KATAMGKNYVNGKTFLEKRYNEDLELEDAIHTAILTLKESF
P0C591	RNMTLVDAIQQVAKNRCVLPNRGFLKQLRELDRQLVQQRRQ
Q8K3W2	RLLPGQLRRLRELRTIWLSGNQLADFPSVLLRMPFLEVIDV
Q91Z38	MPEEEKQKRREESAKLKEEGNERFKRGDYMEAESSYSQALQ
Q99JR6	RDHLHQTGRYQVIEGIISPVNDSYGKKDLVASHHRVAMARL
Q04743	PFLNGFHSAAAAAAGRGVYSNPDLVFAEAVSHPPNPAVPVH
Q2HJF3	LFTTAALLSACKILKLKVDRNKMAATSGVKKAIFDRLCKQL
Q8BMB3	DQATTARIRDTLRRVLNLPPNTIMEYKTHTDSIKMPGRLGP
P49795	HSPAGRSVFRAFLRTEYSEENMLFWLACEELKAEANQHVVD
P35270	AARDMLFQVLALEEPNVRVLNYAPGPLDTDMQQLARETSVD
P68543	SELQGIFDKEEVDVKVEDKKNEICLSTKPVFQPFSGQGHRL
Q28001	ECEKSSATTYFWYREALDISNSISESGGLNWKMTLCLLVAW
Q08DT6	RLLQTTLSRRGLEEFFDDPKNWGEEKVKSGASWTCQQLRNK
Q8VI63	ELHGHLNTLYVHFILFAREFNLLDPKETAVMDDLTEVLCSS
Q13126	LLARHGRQHTIMPSKVNYQANIWALKEEGCTHVIVTTACGS
A6NM10	LTLLFLLFLAHGVTLDGASANPTVSLQEFLMAEESLPGTLL
Q9EQ00	IKFLALGCSSLGRTLNTAMKNVCEILTSDPEGGPARIPFET
Q8N2G6	FGEYKCPKCKRKWMSGNSWANMGQECIKCHINVYPHKQRPL
Q9D2E1	VEAIRYCHGCGVAHRDLKCENALLQGFNLKLTDFGFAKVLP
O60258	IGKPSGKSKDCVFTEIVLENNYTAFQNARHEGWFMAFTRQG
Q32NC0	PATPTSKLSLKTPERRTANPNHDMSGSKGKSPASVFRTPTS
Q9DBJ1	WTVLDAIDQMWLPVVRTWRLNERHYGGLTGLNKAETAAKHG
P15949	WHVAVYRYNEYICGGVLLDANWVLTAAHCYYEENKVSLGKN
Q2TUM3	GSDGAAENQAGVRYISIKPDNRKLANGTNVLGLLVDTLLKE
Q9NPC3	QYQKLQGLYDSLRLRNITIANHEGTLEPSMIAQSGVLGFPL
Q9D3J9	HARASQHSRSQSRTVLQANSNPVFESPTLAAVGICRDVIRE
Q6P8I6	VDMPVFFYIDPEFAEDPRMVNVDLITLSYTFFEAKEGHKLP
Q5FW52	DGEDEATCRQGEQGPPGGPGNIATRPKSLAISSSLASDVVR
A6H7I7	GKRALCPTCSLPLSEKERFFNYCGLERALVEVLGAERFSPQ
Q9BYD2	GAGRLLRGGVQELLRPRHEGNAPDLACNFSLSQNRGTVIVE
Q96FE7	FLVSNMLLAEAYGSGGCFWDNGHLYREDQTSPAPGLRCLNW
P28845	MKKYLLPILGLFMAYYYYSANEEFRPEMLQGKKVIVTGASK
Q8N4S7	TGYRPASSGSGCLRSLFYLHNELGNIYTHGLALLGFLVLVP
Q8NHV1	EEEKEKEIKLLKLKYDEKIKNIREEAERNIFKDVFNRIWKM

Q6P0X2	DHMVRLHLYPADFRFDKPKTNRGPAMPAAADAATRAPTDDS
Q8K4Z2	LFMWAGVIALFCRQYDIIKDNEPNNNKEKTKSASETSTPEH
A6NGY1	KTAFSHSSEKHTQRQAGSDPNPNKENSEETKLKAGNSTAGS
Q96QU4	PEKECSLSLNKKSRSSTPVHNSEIQETCDAHHRGRSRACTG
Q9P0I2	QSRMMQEQMTGAAMAMPADTNKAFKTEWEALELTDHQWALD
Q5E9G3	EMETDKQEKKEVPKCGFLPGNEKVLALLALVKPEVWTLKEK
Q9H190	KGKIVSLVKGSSAARNGLLTNHYVCEVDGQNVIGLKDKKIM
Q6UWN5	TTSPWTAIDLQGSCCEGYLCNRKSMTQPFTSASATTPPRAL
Q9QY36	LRRHLELKEKGKHMVLAALENKAENKGNVLLSSGEACREEK
P97822	GGLEVLAEKCPNLTYLNLSGNKIKDLSTVEALQNLKNLKSL
Q3UX61	VSLHVRKSNRAALHLYSNTLNFQVSEVEPKYYADGEDAYAM
Q8CCM6	QGPDPRKAKEDSTKQVSIRRNQREETGVSMSQKVREAGRDV
Q92930	IGDSGVGKTCLLFRFSEDAFNTTFISTIGIDFKIRTIELDG
P04394	EILQVPPMRVYEVATFYTMYNRKPVGKYHIQVCTTTPCMLR
Q8BVP6	AFRAVSLHNWKWLLLRSMVCNQREGCEETVVFIETGTSKGV
Q5T0J7	CGTCCEKCLLCALKNNYNRGNIPSEASGLYKGGEEPVTTQP
Q8BZ09	LEFLRKFGIGFVSGTMGSVFNIPFDVAKSRIQGPQPVPGEI
Q3SXB8	GEPGIPCECSQLRKAIGEMDNQVTQLTTELKFIKNAVAGLR
Q8BGI3	VLFSFGVVLALVLNLLQIQRNVTLFPDEVIATIFSSAWWVP
Q1RMJ7	IPKASRVKPVEYSQMYSYSYNQYYQQYQNYYAQWGYDQNTG
Q9Z1L4	YHKPLGFESGEVTPDQITCSNPEQYVGWYSSWTANKARLNS
Q8VCH9	RIQSVHKNAFNNLKARARIANNPWHCDCTLQQVLRSMASNH
Q9CQ58	LSFLKSQGKHYKLLAMFNLSNCIYNETYHILFYLRELKCQI
Q6IRU5	ESKEETPGTEWEKVAQLCDFNPKSSKQCKDVSRLRSVLMSL
Q9KIP8	AFAGAVKAWFAENPQGNDPRNYMRVGMDAMKEVVRNKINVC
Q9DBS2	ALLIQAVKKAQKESPLPGQANTVLVLERPLLIETYVGLMSF
Q13432	DSGTVLFEIKKPPVSERLPINRRDLDPNAGRFVRYQFTPAF
Q9H741	GCCNVNVPSTKQYCCDGCWPNGCCSAYEYCVSCCLQPNKQL
Q9D8X2	RAAFTAFEEVQLPRLKQENPNMRLSQLKQLLKKEWLRSPDN
Q8TDU5	LLFTQAILVSSDLFESWNIQNNDLKCKIITFLNRVMRGVSI
P06880	LLWPQEASAFPAMPLSSLFSNAVLRAQHLHQLAADTYKEFE
Q92871	KRYCLDSLDQDSFDTIHFFGNETSPGGNDFEIFADPRTVGH
Q920L5	KFAMFITLSQITQMLMGCVINYLVFNWMQHDNDQCYSHFQN
P13727	DASKKDGAVESISVPDMVDKNLTCPEEEDTVKVVGIPGCQT
P14434	VFPKSPVLLGQPNTLICFVDNIFPPVINITWLRNSKSVADG
Q6PHZ8	EIYSQFFPQGDSTTYAHFLFNAFDTDHNGAVSFEDFIKGLS
Q6ZTW0	GRALWHLRLAHHSQRAAFNNNVSVAYECLSAGGRRKRPGLD
Q5T0L3	ESQLEAYDFPEVQQDEGKWDNCLSEDMAENICSSSSSPENT
Q9NQ48	QLDQGNQKDFIKAQDLSNLENTVAALKSEFQKTLNDKTENQ
Q9R0X0	TAASTLGSQGQAGKLMYVMHNSEYPLSCFALFENGPCLIAD
A6NI87	PRRPPLRRMPSLSTFYLLDHNTRQAELGLAYGAPCMRLSNQ

Q60961	VFIIFKAYLINCVWNCYKYINNRNVPEIAVYPAFETPPQYV
P21765	LNALQEDLKPFGLVILGFPCNQFGKQEPGDNLEILPGLKYV
Q99967	ASMQLQKLNNQYFNHHPYPHNHYMPDLHPAAGHQMNGTNQH
P22061	LIDQLKPGGRLILPVGPAGGNQMLEQYDKLQDGSIKMKPLM
A6NM15	AKEEHLNMFIQNLLWEKNVRNKDNHCMEVIRLKGLVSIKDK
Q8CJ26	QGQDGDREGMWVGAGGALAPNTSSLFPPEPPGASSNIIPVY
Q5FW52	ETIKTPTTHPRAAGRETKYANLSSSSSTASESQLTKPGVIR
Q1JQA4	IELIGGVVALIFRNQTIDFLNDNIRRGIENYYDDLDFKNIM
P59025	EQCYGERGGQYRIHVASRQDNRRHRGEFCEACQEGIVHWKP
P08637	CLVMVLLFAVDTGLYFSVKTNIRSSTRDWKDHKFKWRKDPQ
Q2KIU3	KLGLMKNDTQILEKESKAKDNYRHLSGNVVLQLTLGDRVWL
Q62276	VRAGESLMKLVSDLKQFLILNDFPSVNEAIDQRNQQLRALQ
Q9JJW6	FDSGCGGGEGVETGAKLLVSNLDFGVSDADIQELFAEFGTL
Q13670	FHLLCLLAMYCNGNKGPEHPNPGKPFTARGFPASATFQTTP
Q15744	KGKKAVNKDSLEYRLRRERNNIAVRKSRDKAKRRILETQQK
Q8CCX5	QCHHLQVEIESLHAVERGLENSLQASEQHYQMQLQDLESVI
Q8N4C7	EINITKAQLSEIEQRHKELVNLENQIKDLRDLFIQISLLVE
P30405	PAARACSKGSGDPSSSSSSGNPLVYLDVDANGKPLGRVVLE
Q99N10	IISGSLSVAAETQPNSPCLLNGSVGLNIFSAICSAVGIMLF
Q96EY4	RKAAQITREAHKQEKKEKLKNEKALRLNLVGEKLQWFQNHL
P28801	EDYVKALPQHLKPFETLLSQNKGGQAFIVGDQISFADYNLL
P14148	ELIYKRGYGKINKKRIALTDNSLIARSLGKFGIICMEDLIH
O94903	GEESKHGLPPSETIAIVEHINAKCPNLEFVGLMTIGSFGHD
Q8TDB4	TDAAARETTEVNPETTPEVTNAALDEAVTIDNDKDTTKNET
Q3T063	TLKAQFPSVSVEASGGVRLDNLPQFCGPHIDVISLGMLTQA
Q3UP87	HFCGATLIARNFVMSAAHCVNGLNFRSVQVVLGAHDLRRQE
Q9BY14	EAITIVQHSSPPGLIVTSYSNYCEDSFCNDKDSLSQFWEFS
Q5T013	VGRPNLQLQMDIFHWQIMDGNLTGNIREFLPIVGHVQVAQV
Q5VZ72	VKGCLECDPKFIEDVGSLLGNLIPSEVPGRTQLLERQIKEM
Q9Z0S5	LLMLGLTLSNSYWRVSTVHGNVITTNTIFENLWYSCATDSL
Q9UIY3	EMEMLFSMFPNQGEVKLEDVNALTNIKRYLEGTREALPPKI
Q01718	FTISIVGVLENLIVLLAVFKNKNLQAPMYFFICSLAISDML
O09172	AAGALLARASTLHLQTGNLLNWGRLRKKCPSTHSEELRDCI
Q8BHX1	LMESVNFSPANLSKSGSRFLNALVDSAIALEIKDTSLASFI
Q96LT6	GLQRDLSLAVSYSRLHSSDWNLCTVFGILLGYPVPYTFHLN
Q8K177	SVGSGLLSVSVGLVALLASRNLLRPRLHWALLTLALVNLLL
Q9D819	YLEATVDWFRRYKVPDGKPENEFAFNAEFKNKDFAVDIIKS
Q5U3C3	GSQPEQVTQRPEEGKESLSKNLLLVALCLTFGVEVGFKFAT
P00848	STNLLGLLPHTFTPTTQLSMNLSMAIPLWAGAVITGFRHKL
Q32L52	RIKIREMILKGQIQEAIALINSLHPELLDTNRYLYFHLQQQ
Q96H79	LRILLLQNDPCLLPEVCLLYNKGEALYGYCNLKDKCNKFHV

Q9JKT3	TGRSVYFSTFFLLCWKFLDANSLWLVTILNSLYCVKITNFQ
A6ND01	ERRNSGRCLQKWFEPAQGNPNVAVARLFASSAPSWELSYTI
P00551	DALAVFLRRLHSIPVCNCPFNSDRVFRLAQAQSRMNNGLVD
P02753	FSVDETGQMSATAKGRVRLLNNWDVCADMVGTFTDTEDPAK
Q13491	GTTGVEQICVDIRQYGIIPWNAFPGKICGSALENICNTNEF
Q9D142	RPAVYAGEVERHFPGSLTAVNQDQPQELQQALPGSAGVMVE
P52483	AAPEPEEQEERKPSATQQKKNTKLSSKTTAKLSTSAKRIQK
P55041	RWSIPADARHLMVQKDPHPCNLRNRHSTAPEEHCRRSWSSD
Q8BTF8	NITNKNDPKSINSRVFIGNLNTAIVKKVDIEAIFSKYGKIV
P20036	AFSFEAQGGLANIAILNNNLNTLIQRSNHTQATNDPPEVTV
P55327	SPFDFDAGVNKSYLYLSPSGNSSPPGSPTLQKFGLLRTDPV
Q91V37	LFCGVCVGIVGSGAALADAQNPSLFVKILIVEIFGSAIGLF
Q96CE8	SIIVNILLYFPNGQTSYASSNKLTNYVWYFEGICFSGIMML
Q9GZP4	NIPQMSFDDTEREPDQTFSLNRDLTGELEYATKISRFSNVY
Q5VXT5	IFAFGSCGSYSGETGAMVRCNNEAKDVSSIIVAFGYPFRLH
Q3T062	EWEMMCRVKPDVVKDKETERNLQRIATRGVVQLFNAVQKHQ
Q9D9B0	FWKEDKAFWREDNALWERDRNLLQEDKALWEEEKALWVEER
Q05716	QSRTHEDLYIIPIPNCDRNGNFHPKQCHPALDGQRGKCWCV
P38117	AEKEKVDLVLLGKQAIDDDCNQTGQMTAGFLDWPQGTFASQ
P09611	MFNEFNKQFGEGKNFTSKVINSCHTEFMTTPNNKEAAANTE
Q810N5	LFFFHIRDLVSFINRFVELFNLTMKTQILPMNLNEESCIKD
Q95IE3	RRVHPKVTVYPSKTQPLQHHNLLVCSVSGFYPGSIEVRWFR
Q32KM6	NDKPEGEGMLRLKNGNRYEGNWQRGVKNGSGRFFHLDHGQL
Q60819	GFKRKAGTSTLIECVINKNTNVAHWTTPSLKCIRDPSLAHY
Q505H4	HPKCTKVEHNGCCPECKEVKNFCEYHGKNYKILEEFKPSPC
Q8K1A0	DIDEDALEIFNKNVEEFELTNVDMIQCDVYSLSNRMSKLFD
Q2M2T8	RQRPSKAKIKSHPQCVFISRNFHTGRFQSQEEQRKKETACI
Q9H3N1	ELLEGDWMIEFYAPWCPACQNLQPEWESFAEWGEDLEVNIA
Q60809	ILRLFFPVIYDVKYLMKSCKNLKGGLQEVAEQLELERIGPQ
Q3T0Y3	WWKKKEGHPILFLFYEDMKENPKQEIKKVVRFLEKNLDDEI
Q810P2	GTNIPVFRAAPSYSLASTNKNWFHKENIAGGPGPAMHTRPE
Q9TR36	CAYKLVTIKFKWWGLQSKVENFIQKQEKRIFTNFHRQLFCW
P55083	PVFCDMTTEGGKWTVFQKRFNGSVSFFRGWNDYKLGFGRAD
Q9D287	NWQRKNMQLTAGSKLREMESNWVSLVSKNYEIERTIVQLEN
P16563	KKSTPYQQGTPCASCPNNCENGLCTNSCDFEDLLSNCESLK
Q6ZUS5	FLEYLTNKTEEYTEQPEKVWNSYLQKSGEIERRRQESASRY
Q9D883	NVYVKFRREEDAEKAVIDLNNRWFNGQPIHAELSPVTDFRE
Q9Y224	VWLEDQKIRHYKIEDRGNLRNIHSSDWPKFFEKYLRDVNCP
Q8BGR6	IKAVPFQNAVLNVKELGGADNIRKYWSRYYQGSQGVIFVLD
Q47108	DKIGEHLGDKYKAIAKDIADNIKNFQGKTIRSFDDAMASLN
Q9QXV3	REASPADLPIDPNEPTYCLCNQVSYGEMIGCDNDECPIEWF

Q8BL86	KVLPGHFNTFGAERLFRLASNYISKAGICHKVSTFAMRSLA
Q6PHN7	FKLQMHMLNGALLALLFPVVNTRLLPFELEIYYIQHAMLYV
O35740	SQGGSLPASMQLQKLNNQYFNHHPYPHNHYMPDLHPTAGHQ
Q2TBT3	TSTLRFPRPWKPLETRGCSSNPGAAGREIQVCALAGPNQGI
P09630	RGRQIYSRYQTLELEKEFHFNRYLTRRRRIEIANALCLTER
Q0P5D3	TNTDVDCLKACQEQIEVVLLNSLQQYRQDQGDGSKSEDELD
Q9Y680	LVSCHRTTLHVLKCMYLLVLNNNTCAEGKIPPDATLIFEIE
P55212	CEMLGKYGSSLEFTELLTLVNRKVSQRRVDFCKDPSAIGKK
P16114	NLIEKDLSRKWTLGIIADAFNASEITIRKRLESENTNFNQI
Q9D3B1	TIYAALPFVRQAGLYSISLPNKYNFSFDYHAFLILIMISYI
Q3KRA9	RGMVPERLPPWLQRYVDKVSNLSLFGGLPANHVLVNQYLPG
Q2WGJ8	GVGVGLWLVISSLPRRRLVLNHTRGVYHFSIQGRTVCQGPL
Q7YRZ7	LREANVTVIDRKICNDAQHYNFNPVIDLSMICAGGRKGEDD
Q8VEN2	HLGLFLCLALHLSPSLSASDNGSCVVLDNIYTSDILEISTM
A2VE67	MILVVAAKEVWGDEQADFVCNTLQPGCKNVCYDHYFPISHI
Q99J56	SFWFGTRFKACYLPWVILGFNYIIGGSVINELIGNLVGHLY
Q8BHX1	KASEYESEAKRLEDFLMESVNFSPANLSKSGSRFLNALVDS
Q8K3Y3	ERRPKGKNMQKRRSKGDRCYNCGGLDHHAKECKLPPQPKKC
Q06520	VLYGSWFDHIHGWMPMREEKNFLLLSYEELKQDTGRTIEKI
P40313	HCNVSPGRHFVVLGEYDRSSNAEPLQVLSVSRAITHPSWNS
Q2TBK4	IPQYEVNAWTTEILYNLSERNRIRDRDVYLVTEDLKQKAKE
P24369	PKTVDNFVALATGEKGFGYKNSKFHRVIKDFMIQGGDFTRG
Q9QXT8	ILLRGTVHEKLKWAFNLYDINKDGCITKEEMLAIMKSIYDM
Q99437	SIIFCEAVAIYGIIMAIVISNMAEPFSATDPKAIGHRNYHA
P97822	EVTELVLDNCLCVNGEIEGLNDTFKELEFLSMANVELSSLA
P09630	NSTAYDPVRHFSTYGAAVAQNRIYSTPFYSPQENVVFSSSR
Q7M759	QYSSREKDAIECFMTRTSKGNRIACMFVRCSPNAKYTLLFS
Q2PT27	NLKDAIFKSCDLSMADFRNINALGIEIRHCRAQGSDFRGAS
Q9QWG7	ALDRIVHHTSFEMMKENPLVNYTHLPTAMMDHSKSPFMRKG
Q01081	SRLHNKPTFSQTIALLNIYRNPQNSSQSADGLRCAVSDVEM
O75431	TELDALVFGHLYTILTTQLTNDELSEKVKNYSNLLAFCRRI
Q17QN8	DHFELCLAKCRTSSQSVQHENTYRDPIAKYCYGESPPELFP
Q1JPH6	EERAQRPRLQLKPRTVATPLNQVANPNSAIFGGARPREEVV
Q8IWL1	VGLTEGPSPGDFRYSDGTPVNYTNWYRGEPAGRGKEQCVEM
Q5BU09	TGESESSSEDEFEKEMEAELNSTMKTMEDQLSSLGTGSSSG
P62609	PQGLVIPPEQAPGKLEFTRENGGLTLFNPTPYYLTVTDLKA
O00194	LGDSGVGKTTFLYRYTDNKFNPKFITTVGIDFREKRVVYNA
Q9D1G0	QAVAEIDALYDVYLDVIDKWNTDDMLFLGDFNADCKYVKAH
Q05685	PEDKLHDQCSPWKKNACCSVNTSQELHKADSRLYFNWDHCG
Q68FG0	EKMAHVEELRLIHADINVMENTIKQSENDLNKLLESTRRLH
Q9ES30	GNTVFSMYSYETKGKSDTSSNHAVLKLAKGDEVWLRMGNGA

Q1JQA8	IALKYGVSMEQIKRANKLFTNDCIFLKKTLNIPVISEKPLL
A5PKI3	LLTFYVISQVFEIKMDASLGNLFARSALDAVVRSTKPPRYK
A2AQ14	VVLLEPFLVGIEGFLKSSTYNLLFCNSCGTPVGFHLYSTHA
Q9D9M5	AVTSLPFTSGMIELLSFLRMNKDRFDCIIISDSNSIFIDWV
Q32KN9	TWNINHARLMVVEERCVYRVNSDNSGWTEIRREAWVSSSLF
Q8K015	YQADQLESDFCDVLTGPLQRNALCNLLSFTYKVEQRCQTFS
P53518	QISNNKAYPLIVQSNIWDENNNKNHDFIATPPIFKMESESR
Q05B50	RLQGTLAADKNEILFSEFNINYNNEPLMYRKGTVLIWQKVE
Q9CQA5	ALEDLEVLSRELIEMLAISRNQKLLQLEEENQVLELLIHRD
P09211	GRTLGLYGKDQQEAALVDMVNDGVEDLRCKYISLIYTNYEA
Q3SZY3	SDDTYPEIEKFFPFNPLDFENFDLPEEHQIARLPLSGVPLM
Q9UHV2	CLLDDGLEGLFEDIDTSMYDNELWAPASEGLKPGPEDGPGK
Q9H8H3	KSIAENRHLQFERFVVAAGENMHQVADGSVDVVVCTLVLCS
Q9DCX7	TFILIAVCKFKMLSCQLCSDNEERVPDSDQTSGGQSFVFTG
Q32LN0	HLLDTNQLDASCIPFQEFDVNGEHLCSMSLQEFTRAAGTAG
Q96H12	CEGTSQPEPSCSAVRITANKNYRSKTSQEGALKKMHEEEHH
Q9NTU7	SEMSNKTRIIYFDQILVNVGNFFTLESVFVAPRKGIYSFSF
Q64205	GYYSAPQHDYYSGQPYGQTVNPYTYHHQFNLNGLAGTGAYS
Q3T075	GLDALSSIISRQKQMGQEIGNELDEQNEIIDDLANLVENTD
Q9UHY7	REASAAEEADVHVAVVVRPGNAGLTDDEKTYYSLITSFSEL
Q3KPI0	VYLPENLYSYGWYKGKTVEPNQLIAAYVIDTHVRTPGPAYS
Q3SZX4	DPSLKPWTASYDPGSAKTILNNGKTCRVVFDDTYDRSMLRG
P13675	RALVGRAVQHKFERKDGSEVNWRGVVLAQVPIMKDLFYITY
Q9D9W1	LMPRFRSAAKGDSYPGPGTYNPEMKSVPKVTWPMKFGSPDW
O70456	HTLSEDSYKDSTLIMQLLRDNLTLWTADSAGEEGGEAPEEP
Q8BSU2	VLSLLAIVFFLTAAMAYVLCNRRATQQNSAGLQLWYTPVEP
P70202	TFYQSLMSLKRPLEAQDIPDNFGNVSPQMKPVQHLAWVACG
B0YJ81	LSPTSPRCAATMASSDEDGTNGGASEAGEDREAPGERRRLG
Q5HYI8	VLVLGDSGVGKSSLVHLLCQNQVLGNPSWTVGCSVDVRVHD
Q9BYQ8	SCCVSSCCKPQCCQSVCCQPNCCRPSCSISSCCRPSCCESS
Q3LI81	CLPSSFHSRTCFLDNFQETCNETTSCQMTNCEQDLFTDDSC
Q3SZU4	AKFDQYRYTRNLVDQGNGKFNLMILCWGEGHGSSIHDHTDS
Q14469	FLIPNGAFAHSGPVIPVYTSNSGTSVGPNAVSPSSGPSLTA
Q9HBV2	LTIGVIICVFIIFLLIFIIINWAAVKAFWGAKASTPEVQSE
Q9NX53	LGAPWNTNGPPWNRHGAPPQNSLDAPWNSLGAPWNSLGAPW
Q9BX46	GYGFVTMADRAAAERACKDPNPIIDGRKANVNLAYLGAKPR
A0PJX2	GETFLFSFSPQLKVFKWTGSNSFFVKGDLDSLMMGSGSGRF
H3BS89	CSDHWYETDARKHRDRCKAFNTRRVDPGFIYNNNNNLPLRA
Q99426	YDQRQDTVRSFLKRSKLGRYNEEERAQQEAEAAQRLAEEKA
P19879	EKSFQLQKDENITPLPPKKENDEMPTCLLCVCLSGSVYCEE
Q9BSG5	CRSALGHALPVAAPGARHCFNISISAVPRPRPGRRGREAPS

Q8N614	FSVAPLVDHLEEYNTTCHLKNHTGRSTIMEDEPSKEKSINY
P54709	GMNDPDFGYSQGNPCILVKMNRIIGLKPEGVPRIDCVSKNE
Q9CQ56	QSVIKKDNQTLSHSLKMADQNLEKLKLESERLEQHAQKSVN
P01138	FYTLITAFLIGIQAEPHSESNVPAGHTIPQAHWTKLQHSLD
P97822	DLSTVEALQNLKNLKSLDLFNCEITNLEDYRESIFELLQQI
P29084	TQHLDIGLKQKQWLMTEALVNNPKIEVIDGKYAFKPKYNVR
Q6UX52	GTKNIKVAKKVVKTHEPASFNLNVTLKSSPDLLTYFCWASS
Q3SX42	DTLDDIFDGSDDEEESQDIVNQVLDEIGIEISGKMAKAPSA
Q8VI33	INPSLIGSKNILITTNMVSQNTAESANALKRKREDDDDDDD
O08585	PESIRKWREEQTERLEALDANSRKQEAEWKEKAIKELEEWY
Q96DZ9	ITLAFLFLYATQYYQRFDRINWPCLLQGHGQSGGPHPLDLL
P14231	AGANQSMNVTCVGKRDEDAENLGHFVMFPANGSIDLMYFPY
A6QLK2	YSEVPNFSEPNPEYSAQQAPNKTVQNDSGPAAPQPPTGPPA
Q3SZX8	VLMDCAHLVKANSIQGCKMNNVNVVYTPWSNLKKTADMDVG
Q9DC70	LSHLPRSRAEYVVTKLDDLINWARRSSLWPMTFGLACCAVE
Q3SZA1	PSVFEKYTASVTVGSKEVTLNLYDTAGQEDYDRLRPLSYQN
Q32L31	KSANPGISIGDVAKKLGEMWNNLSDSEKQPYINKAAKLKEK
Q56K12	LLEFAEEQLHVDHVFICFHKNRDDRAALLRTFSFLGFEIVR
P25731	VYTKKVLNPGTKEEYEVDTPNWEGGLVTTPSKVILPGGGSK
P49638	LLRFLRARDFDLDLAWRLLKNYYKWRAECPEISADLHPRSI
P05531	RDDENANPEEVVGDTRSPVQNILGKFEGDINKRLHIKRKRM
Q9P126	WEDGSVISENMFEFLEDGKGNMNCAYFHNGKMHPTFCENKH
Q9Z2R6	EIKKPPVSERLPINRRDLDPNAGRFVRYQFTPAFLRLRQVG
Q9QXJ4	SVRLPTKNFEVDLLEIGGSQNLRFYWKEFVNEVDVLVFMVD
P04393	KEPLDFLFLNRSCFNGMIRFNSKGGFNVPFCKKPNRFAQAY
P30931	ARVLSYPADTSSSTVEPPFTNSPEFTPYLETNLGQPTIQSF
Q9UBU2	ANRSAGMYQGLAFGGSKKGKNLGQAYPCSSDKECEVGRYCH
P55041	IPADARHLMVQKDPHPCNLRNRHSTAPEEHCRRSWSSDSTD
Q9Y275	GLKIFEPPAPGEGNSSQNSRNKRAVQGPEETVTQDCLQLIA
Q8CEZ0	MSDGWKFEQLISIGSSYNYGNQDQAEFLCVVSRELNNSTNG
P99026	GGVVIAADMLGSYGSLARFRNISRIMRVNDSTMLGASGDYA
Q28071	ALSNCWMKNKRNRMLQSDYMNMTPRRPGPTRRHYQPYAPAR
Q5SRD1	MTGAAFGAMNGLRLGLKETQNMAWSKPRNVQILNMVTRQGA
P10144	SGGPLVCNKVAQGIVSYGRNNGMPPRACTKVSSFVHWIKKT
Q921J4	RHVLLTIKCLLIHPNPESALNEEAGRLLLENYEEYAARARL
O00142	VSSVRLMERSIHSARYIFVENLYRSGKMPEVDYVVLSEWFD
Q9ES56	VNGKYTADGKEVLEYLGNPANYPVSIRFGRPRLTSNEKLML
P28069	KLKAILSKWLEEAEQVGALYNEKVGANERKRKRRTTISIAA
Q765P1	FSLIAGIFLCFSCSPQGNRSNYYDAYQAQPLATRSSPRPGQ
Q9NPI7	TCFRKMKGDYLETCGYKGEVNSRPTYRMFDQRLPSETIQTY
P15947	YQCGGILLNANWVLTAAHCHNDKYQVWLGKNNFLEDEPSAQ

P35509	ISRMEYVHSKNLIYRDVKPENFLIGRPGNKTQQVIHIIDFG
Q9D711	VLIAGEPLREPVVQHGPFVMNTNEEISQAILDFRNAKNGFE
Q14249	QKAMDDTFYLSNVAPQVPHLNQNAWNNLEKYSRSLTRSYQN
Q9DAC0	LLTMELSICAFFFFLYSLAINRYIPFVFWPMMDLMNDLACS
Q9Y624	SSPRVEWKFDQGDTTRLVCYNNKITASYEDRVTFLPTGITF
A7MBB3	PYISVCGNVPPQSCIFSQVLNIGAASAAWICILRYYQLRDW
Q17QJ5	DFTQEYWQCCGAFGADDWNLNIYFNCTDSNASRERCGVPFS
Q9ULY5	FTELSCYNYGSGSVKNCCPLNWEYFQSSCYFFSTDTISWAL
Q49A33	KNFTNVKNAAKPLLNPHPLINKRIHTGEKPYTCEECGKAFY
Q5EA90	IGPLASEQMFYAVTNRINDENTYKICTWLEIKVHHVLLYIQ
Q5E982	ILRATKLGIPYRVIHNASIMNAVGCCGLQLYKFGETVSIVF
Q5FW57	LGTPDVINWKQHLQIQSSQSNLNEAIMDLAAGKMVKVKRTQ
P41217	IVTWQKKKAVSPENMVTFSENHGVVIQPAYKDKINITQLGL
A2AUU0	QVKFESDANKYWDIFYQTHKNKFFKNRNWLLREFPEILPVN
Q9XT56	VEMPLEPKSNRAFSNSSYTLNQKTGELIFDPVSASDTGDFT
Q96MV1	IFLFDEATKADPLWGGPSLANVNIAIASGYLISDLSIIILY
Q7TN89	LQQIWSTWTPHHKQPLVLVGNKCDLVTTAGDAHAAAALLAH
Q3KRB8	VHVLRYFFNFLRNVSLRSSENKMDSSNLAVIFAPNLLQTSE
Q8VEJ3	TVCVNDVCTAVEDTRPVMDRNTDGQDGAYAEGTTKWPAEEN
P68254	FDISKKEMQPTHPIRLGLALNFSVFYYEILNNPELACTLAK
Q6BBL6	LYVGWAAAGLLILGGALLCFNCPPRNDKPYSAKYSAARSAP
P18468	LVRDSRPWFLEYCKSECHFYNGTQRVRFLKRYFYNLEENLR
O43513	QGIERLHPMQFDHKKELRKLNMSILINFLDLLDILIRSPGS
O00194	QSFLNVRNWMSQLQANAYCENPDIVLIGNKADLPDQREVNE
Q9CWQ0	ANGMHTLCLLDIKVKEQSLENLIRGRKIYEPPRYMSVNQAA
A6NDL7	VVWPSALVLCYFLETNAKQYNMVDKNVIEIGAGTGLVSIVA
Q3T0Y3	IYLARNAKDVAVSFYHFDLMNNLQPLPGTWGEYLEKFLTGN
Q15560	PSTPRITTFPPVPVTCDAVRNKCREMLTAALQTDHDHVAIG
P04768	LVNSSLLWKNVASFPMCAMRNGRCFMSFEDTFELAGSLSHN
Q99KI3	KVVPPSPMTDPTMLTDMMKGNVTNVLPMILIGGWINMTFSG
Q9CQK7	EENLGMVMIFTLVTAVQEKLNEIVDQIKTRREEEKKQKEKE
Q9D309	VNYETGKVIATKYFDMYEGDNSGPMAKFIQSTPSKSLLFMV
O95843	NGMAKDQDLLEIVYKSFDFSNVLRVICNGKQPDMETDSSKS
Q9CQ37	GRICLDILKLPPKGAWRPSLNIATVLTSIQLLMAEPNPDDP
Q0VCT9	SGGGAGSSNSGGGSGGGSSSNMPASVAHVPAAVLPPNVIDT
Q91UZ4	PRAGVSKRHLRGDQITWIGGNEEGCEAINFLLSLIDRLVLY
O09101	LFAVVLSTFTTVPCLCLLGPNLKAWLRVFSRNGVTSIWENS
P20618	AGGSASAMLQPLLDNQVGFKNMQNVEHVPLSLDRAMRLVKD
P29965	VLQWAEKGYYTMSNNLVTLENGKQLTVKRQGLYYIYAQVTF
Q712K3	PPVDDPQSGELPSERWNPTQNVRTILLSVISLLNEPNTFSP
Q9NX53	NGPPWNRHGAPPQNSLDAPWNSLGAPWNSLGAPWNSLVLLE

Q9DAM2	HRNTLNDVLFFHFLQHVTNLNKSQIGMIFDLLDWTAVGEIG
Q3ZCC4	EEESGRGAPVPVPPKRTVKRNIPKLNAERLISERGLPALRH
Q9H019	RIGTNLPLKPCARASFETLPNISDLCLRDVPPVPTLADIAW
P80219	GFLVREDFVLTAAHCLGSSINVTLGAHNIMERERTQQVIPV
Q9NQR4	ECSIYLIGGSIPEEDAGKLYNTCAVFGPDGTLLAKYRKIHL
Q9CPV4	DDHFVAELTYNYGIGDYKLGNDFMGITLASSQAVSNARKLE
Q9NYP8	NTIRNCSCSADIRDCDYSLANLMCNCKTVLPLAVERTSYNG
Q5M956	DSLKQHYFIDRDGQMFRYILNFLRTSKLLIPDDFKDYTLLY
O94760	DHRYDKLTVPDDIAANCIYLNIPNKGHVLLHRTPEEYPESA
Q3T0C6	GVDDPTFGYQEGKPCILVKMNRIIGLKPQGEPRIECTGKSE
Q3MHZ1	REDEKPLVLEMLKAGVKDTENRVALHALTRPPALLLLAAS
Q14088	DLREQIQVPSNLALKFADAHNMLLFETSAKDPKESQNVESI
Q4G0X4	LSSITSHLQDPNHLTLDWVANVEGLPEEEYTKQNLKRLWVV
P52843	VLFGSWFEHVRGWLSMREWDNFLVLYYEDIKKDTKGTIKKI
P01190	TENPRKYVMGHFRWDRFGRRNGSSSSGVGGAAQKREEEVAV
Q921I9	QIDIYVQVLQADGGTYAACVNAATLAVMDAGIPMRDFVCAC
Q91WB7	GRQRRERPAAPGHPRKRAGRNEPLKKERLKWKSDYPMTDGQ
P04925	IHFGNDWEDRYYRENMYRYPNQVYYRPVDQYSNQNNFVHDC
Q8BJU2	LKEGLLLYNTENNVGLKNAWNIIQAEMRCCGVTDYTDWYPV
Q8R1M8	FTLCMKAFTDLTRPYSIFSYNTRTKDNEILLFVENIGEYMF
Q8C8S3	PYISFAGDDPPASCVFSQVMNMAAFLALVVAVLRFIQLKPK
Q8CGF5	NQRWFFEALKYPKFSKANVINGILMTVVFFIVRIISIPPMY
Q8CDS7	ILHAGGSQGWVPWKYRMFLRNDLCIKKEDSLFLEFCDVVKR
O70401	KIQSTLHCCGVTNYGDWKGTNYYSETGFPKSCCKLEGCYPQ
Q9D6D8	PHNKRGVLGMVKRAITPMGHNSTSHYRQLPTWIKNMWLLGN
Q6PGD0	LQTLILPQDVPCPGGSNAWDNVTSFKDKQICQGQRDLCNST
Q08624	QIKIWFQNRRMKWKKDHRLPNTKVRSAPPAGAAPSTLSAAT
Q9D979	TAKFLAALCPLDAPERSLLANQEDCLPRLCSAWGLHGNISG
Q53TN4	IVTRPQWKRPKEPNSTILHPNGGTEQGARGSMPAYSGNNMD
O18870	KNKSQRKNTTSPSKQWKVGDNCCAIWSEDGCIYPATIASID
P97370	YRACQFPVSLLEECSGVTDANFGYSKGQPCILVKMNRIIDL
Q2T9N3	SSIDINSSRKFLQRFLREGQNKTGTSYALDCGAGIGRITKR
Q86VG3	NIFTSAKVTHKNEADDYHLRNKTIFLRTSSQCLEEQVDNFL
Q96FV3	ILAFVFKDWIRDQLNLFINNNVKAYRDDIDLQNLIDFAQEY
Q6PEB6	EGQDEGVWKYEHLRQFCLELNGLAVKLQSECHPDTCTQMTA
Q92913	AAIASSLIRQKRQAREREKSNACKCVSSPSKGKTSCDKNKL
Q6QUN5	ILGPSDPIILFHPVRLVVTKNSLGTPASDEYPCQVSVQNPT
Q96F25	LSNAYSPRHYVIADTDEMSANKINSFELDRADRDPSNMYTK
P0C7X4	ATGCLPLLAPPGGTSMFPFQNILFIFLLSVLPLLAIKLSVL
Q7L4S7	QGKLLFAEPKDAGFPFSQDINSHLASLSMARNTSPTPDPTV
Q0VCT9	GSSSNMPASVAHVPAAVLPPNVIDTDFIDEEVLMSLVIEMG

Q8VE95	FITCFKDLQFLVTFFSRLRPNHSGRYEASFPFLSLCGRERN
Q8BS03	QMVWATSNRIGCAIHTCQNMNVWGSVWRRAVYLVCNYAPKG
P05452	FSLLTQVTTEPPTQKPKKIVNAKKDVVNTKMFEELKSRLDT
Q9D676	FDYWIGLHRASSKHPWMWTDNTEYNNMFVYHMNAQCLKKPE
Q3TB92	IRCWLDKLLLWALTISITLQNAAVDCTRVENNELPSPNLNS
A4D1T9	KLAKPAMLNPKVQPLTLATTNVRPGTVCLLSGLDWSQENSG
Q8K0Z7	LCRSKNMPKSTIESALKTEKNKGIYLLYEGRGPGGSSLLIE
Q9BRJ7	LSNAFVSTAKCQLLFALKVLNMMPEEKLVEALAAATEKQKK
Q9D7S0	LGGFLHVYQNKFCSASNCTENSTEVAFTVHLFDDQRYHFAS
Q3UWA4	ISHYKERLNRRSRKLRKDLGNLQQLKAQEKKMLQALQVDCE
P0C7V0	SRRGVRRTARPAGADTRSSANQLPQPSGACANADSAPPADV
P35428	AGFSECMNEVTRFLSTCEGVNTEVRTRLLGHLANCMTQINA
P57052	KWTHQQPSDSDLYQMTAPLPNSASVSSSLNHVPDLEAGPSS
Q9D7G9	PAQYPTPEASYPSPGLQPSPNNPYPLPPGPSAASPGPGSLH
P24586	AQLFKERHKESSFLMVSHSLNSLKEFCDVAIVFKNSYIIGY
P13761	LAGDTQPRFLWQGKYKCHFFNGTERVQFLERLFYNQEEFVR
Q9CXV9	VFYQYLEQSKYRVMNKDQWYNVLEFSRTVHADLSNYDEDGA
Q8BGT7	QEREDQKVKWQQFNNRAYSKNKKGQVKRSIFASPESVTGKV
Q60654	LLAITIFQHSQQKHELQETLNCHDNCSPTQSDVNLKDELLR
Q9NVK5	LNEVARHRPRSTLVMGIQQENRQIRELQQENKELRTSLEEH
O08585	AAGGPDAVDGVMNGEYYQESNGPTDSYAAISEVDRLQSEPE
Q9QZ49	FENSSQASFETINGEAARRQNLPKFSTEISPAARPLLRKEV
Q8R3T5	FQILHHTCQRYLTDRKPEFINCQSKIMGGNSILHSAADSVT
P47758	TGQYRDTQTSITDSSAIYKVNNNRGNSLTLIDLPGHESLRF
A6NJI9	KSPFKQKPAQRVPSDFAFANNVDKTVLDDPEDAVFVRSMKR
A8MTZ7	DVDIGSDNTDSRANRLLNGDNLWIDKLPKERTKLSVGKLNN
Q9Y581	ARKLCGRYLVKEIEKLCGHANWSQFRFEEETPFSRLIAQAS
P15018	LYYTAQGEPFPNNLDKLCGPNVTDFPPFHANGTEKAKLVEL
P29368	MQTEDIISVINWAEKQECIDNQRIGLWGTSLGGGHVFSARA
P08579	GNSTPNPQVPDYPPNYILFLNNLPEETNEMMLSMLFNQFPG
Q9UD71	LKAVQRIAESHLQSISNLNENQASEEEDELGELRELGYPRE
Q3UFF7	TNSKLKSLGVSTTFHSLPNLNHELNKTELEKLKSWILTRLP
Q99LS1	NRARLVSYLPGFCSLVKRVINPRAFSTAGSSGSDESHVATA
Q3SYS0	LPPNYRYGMSRPGSLADKKKNPPGTRRRRVAVEPIPEEDWH
P56748	TVMPQWRVSAFIENNIVVFENFWEGLWMNCVRQANIRMQCK
Q921C1	PCIGSTTCFQSHPSEKTIFLNIMFGISGACFLFIFLELALL
O70378	QRISASLLDSRSYETLVDFDNHLDDIRSDWTNPEINKAVLH
O88513	NENKDVGDLTQEAFDLISKENPSSQYWKEVAEQRRKALYEA
Q9CY52	GTLTADKNEILFSEFHINYNNEPHMYRKGTVLVWQKVEEVR
P50294	GTWYLDQIRREQYVPNEEFVNSDLLEKNKYRKIYSFTLEPR
P50592	PRGGRPQKVAAHITGITRRSNSALIPISKDGKTLGQKIESW

Q8BFY6	DADHSGYISLKELKQALVNSNWSSFNDETCLMMINMFDKTK
P20339	AQAAIVVYDITNEESFARAKNWVKELQRQASPNIVIALSGN
Q9JKM7	ETLAREYGVPFMETSAKTGMNVELAFLAIAKELKYRAGRQP
Q9BV99	ALTHLGPLASLRQLAVLNVSNNRLTGLEPLATCENLQSLNA
Q9JKX8	VNMSTGLVQDQTLWSDPIWTNRPIPYSAIDTWPGRRSGGMI
P70190	GECAGHQHCQKYCVPLCIDGNPVLLSHHNVMGDFRFFDYQS
P05142	VVLFTVALLALSSAQGPREENQNQIQIPNQRPPPSGFQPRP
P31273	HVQDFFHHGTSGISNSGYQQNPCSLSCHGDASKFYGYEALP
Q60932	IYQKVNKKLETAVNLAWTAGNSNTRFGIAAKYQVDPDACFS
Q8BXK9	KKYRNYDIPAEMTGLWRYLKNAYARDEFTNTCAADSEIELA
Q9GZU8	QELIEKQRREEELKELKEYRNNLKKVGISQENKKEVEKKLT
Q5E9L3	PAPYPAPAGSYPTPGLYPTPNNPFQVPSGPSGAPPMPGGPH
Q6UWI4	CCCRCLRPKQDPQQSRAPGGNRLMETIPMIPSASTSRGSSS
Q62422	AAKRGNLSWLRECLDNRVGVNGLDKAGSTALYWACHGGHKD
Q92904	QKKSVDRSIQTVVSCLFNPENRLRNSVVTQDDYFKDKRVHH
P62609	NPTPYYLTVTDLKAGNKSLENTMVPPQGKVTVNIPGGYTGG
Q6ZVX7	NIYEPAPPTGPTQRPLETLGNFRGWYIRTEKLQQNQSWTVK
P51151	EFIYYADVKEPESFPFVILGNKIDISERQVSTEEAQAWCRD
Q96L46	SVMDSDTTGKLGFEEFKYLWNNIKKWQCVYKQYDRDHSGSL
O95476	DLSSIVILDNSPGAYRSHPDNAIPIKSWFSDPSDTALLNLL
P08311	GFLVREDFVLTAAHCWGSNINVTLGAHNIQRRENTQQHITA
Q7TQI3	MDLIEQVEKQTSVADLLASFNDQSTSDYLVVYLRLLTSGYL
Q922H7	ALVVRFLTKRFIGDYERNAGNLYTRQVHIEGETLAIQVQDT
Q8NCR6	WLSQEEADKCSPNYLGSDWYNTWRMEPYNSSCCNKYTTYLP
Q96BT1	FKRKGIERWHRAVSTNLLKQNVLVPKEESSSDSDMGFHESQ
Q9WTY4	FGLAIGTLAQALGPVSGGHINPAITLALLIGNQISLLRAIF
P81269	MSVPAPIYQTSSGQYIAIAPNGALQLASPSTDGVQALQTLT
P51674	EKKICTVSENFLRMCESTELNMTFHLFIVALAGAGAAVIAM
Q3T160	APVKKSVRDTPAKNAQKSNQNGKDSKPSTPRSKGQESFKKQ
P00639	VAEINSLYDVYLDVQQKWHLNDVMLMGDFNADCSYVTSSQW
Q7Z5A4	EGGENPEASPAPGPEAGPPLNLFTSFPGDSLLCGRTPLRIV
P20181	SEFQPMIATDTELLRQQRRYNSPRVLLSDSTPLEPPPLYLM
Q8N488	DKEKPEKDKEISPSVTKKNTNKKTKPKSDILKDPPSEANSI
Q9NWX6	QGRLQGTLAADKNEILFSEFNINYNNELPMYRKGTVLIWQK
P35846	QWWEDCQSSFTCKSNWHKGWNWSSGHNECPVGASCHPFTFY
Q8CCA0	NSHLSTLASIHKIYHTLNKLNLTEDVGQDDHQTGSLRSCSS
Q8WXI8	VVFILLLSVCFIASCLVTHHNFSRCKRGTGVHKLEHHAKLK
Q9NWM8	KGKIPPESTLIFNIDLLEIRNGPRSHESFQEMDLNDDWKLS
Q47066	AFARSLGDETFRLDRTEPTLNTAIPGDPRDTTTPLAMAQTL
Q61599	TEKDAQPQLEEADDDLDSKLNYKPPPQKSLKELQEMDKDDE
Q1ZYL8	NCESSAQWKSAVQGLLNYINNWHKQDTSMRPRSSAFSWPGT

P47985	KPEWVILIGVCTHLGCVPIANAGDFGGYYCPCHGSHYDASG
Q6UXN8	GLLPAERSQSANQVCGYVKSNSLLSSNCSTWKYFICEKYAL
Q58DM4	PGNGVDSAGLTWGRIRAEGLNCDYTILFGKAEADEIFQELE
Q9CQQ4	PGIDYVQVGFPPLLSIVSRMNQTTITSVLEYLSNWFGERDF
Q8N567	LKNDAPQAKHKKNKKKKEYLNEDVNGFMEYLRQNSQMVHNG
Q9CR57	RKAKMTDFDRFKVMKAKKMRNRIIKTEVKKLQRAAILKASP
Q9DC16	DDPDKDSGGKIDVSLNISLPNLHCELVGLDIQDEMGRHEVG
P06748	DEEAEEKAPVKKSIRDTPAKNAQKSNQNGKDSKPSSTPRSK
P17751	GGNWKMNGRKKCLGELICTLNAANVPAGTEVVCAPPTAYID
Q9Y5U4	ESPGPKKCGPYISSVTSQSVNLMIRGVVLFFIGVFLALVLN
P23812	PYTKQQIAELENEFLVNEFINRQKRKELSNRLNLSDQQVKI
Q5SZD4	RLVALTLARKLQSRGFPSQGNVLDDNTASISLLKSLHAEFL
Q8BIX3	KFGRQLVPGWKLCPKCTQIINGSVDVDSDDRQRRKPDSDGR
Q3B7M5	YDYSAADEDEVSFQDGDTIVNVQQIDDGWMYGTVERTGDTG
P17322	KATVHRKRCQCASQTDKKCWNFCQAGKELRDQDSMEKAWNN
P52907	TIDGQQTIIACIESHQFQPKNFWNGRWRSEWKFTITPPTAQ
P01911	RECHFFNGTERVRFLDRYFYNQEESVRFDSDVGEFRAVTEL
P27105	ILTKDSVTISVDGVVYYRVQNATLAVANITNADSATRLLAQ
P17481	TERQVKIWFQNRRMKWKKENNKDKFPSSKCEQEELEKQKLE
O75461	LDELIKDCAQQLFELTDDKENERLAYVTYQDIHSIQAFHEQ
P53347	KTPAFPKLDSARHNILGIRNNVFCMARLLNHSLEIPEPTQT
Q8MJ87	KILLHNPQLWWLNDSAPESNNRQQSPSQEKIDQLVYMGFDA
Q8BIG7	GVLAVLRVLWRGEVLQPQPRNKTVECVRNLNERILRDARVY
Q6P8M1	STHAGSKYINTSFPTKKKWENGHCLKDRNEPCHIIQILEIM
Q32LD7	EGYLSGLAYRNDIQWSYPSSNEQVAEEKEEEMEATAAASLS
Q2TBK4	FGDHPIPQYEVNAWTTEILYNLSERNRIRDRDVYLVTEDLK
Q9CPV4	VLGMQVLRHEEFEEGCKAACNGPYDGKWSKTMVGFGPEDDH
P01882	TEATKAITTKKDIEGAMAPSNLTVNILTTSTHPEMSSWLLC
Q2T9R6	ASPARDEKASYVAWGHSTVVNPWGEVLAKAGTEETIVYADI
Q9DBJ6	QDSNAFTRETLQEIFQRLSPNTMVTPHKKSMLGNGNYDVNV
Q3T110	KCGSCLSSLLIPLALWSIIVNILLYFPNGQASYASSNKLTN
Q9UKR8	DDYSTQWNLVMEKLKCCGVNNYTDFSGSSFEMTTGHTYPRS
P13988	DIAYDLELGKTLSEIRALTANGFIKFNIYKSFRANKCADLC
Q9JM93	RSGRRGSEKRSKRSSKDASRNCSASRSQGHKAGSASGVEER
Q24JQ0	IYAGSRALSRLAIPVFLTLHNVAEVIICGYQKCFQKEKTSP
P12342	FIVVPGCVTEACHDDPPSLRNAMFKVFRYEVGTMINCDCKT
P47963	GMILKPHFHKDWQQRVDTWFNQPARKIRRRKARQAKARRIA
P27548	VEEEVNLHEDFVFIKKLKRCNKGEGSLSLLNCEEMRRQFED
Q96PN8	GKICLVMELAEGGDVFDCVLNGGPLPESRAKALFRQMVEAI
Q8NI29	DANQTVLDKFSAVPDPIPQWNNNACLHVTHVFSNIKMGVRF
Q96BZ8	SGPVDLFRELLEEGKGVIRGNKEYEEEKRQEKERQEKALGI

Q9BUT1	VDTPSLQERIQARGNPEEARNDFLKRQKTGRFATAEEIAML
Q99KX1	MLGMSGGFMDMFGMMNDMIGNMEHMAAGGNCQTFSSSTVIS
P14190	GNVLNDLTNGGTKLTITVTGNKPILLGRTKEAFATPVTGGV
Q60930	KLDVKTKSCSGVEFSTSGSSNTDTGKVSGTLETKYKWCEYG
Q60651	KDWAWIDNRPSKLALNTTKYNIRDGGCMFLSKTRLDNNYCD
Q66JS6	WEGEDEDEDVKDNWDDDDDENKEEAEVKPEVKISEKKKIAE
Q9QUK3	FHITTATGFFFFENVAVHLSNLFFRTFDLFLVVHHLFAFLG
Q8R4V2	RPIANPNPGFRQQLEEFGWANSQKLRRQLEERFGEIPFRDE
P25786	ARSQSARTYLERHMSEFMECNLNELVKHGLRALRETLPAEQ
Q9Y3Y2	LRGGRATRTLLRGGMSLRGQNLLRGGRAVAPRMGLRRGGVR
Q9H3H9	HEGKPEVACILEDKKLENEGNTENTGKRVEEPLKDKEKPES
P51557	TKLTWLLSIDLKGWLPKTIINQVLSQTQIEFANHLRKRLEA
P04925	VYYRPVDQYSNQNNFVHDCVNITIKQHTVTTTTKGENFTET
Q13595	YTTERDLREVFSRYGPLSGVNVVYDQRTGRSRGFAFVYFER
Q9D1J1	TAVESVTDSSRYFVIRIEDGNGRRAFIGLGFGDRGDAFDFN
Q2LGB7	MNSVNRQHKYNSVIPMRPLNNPLPRERTPFALRTINALEEE
Q9DCK4	PFPLLEPFTSAPTGPFLPYLNPAPFGLSPPRLRPFLAATPG
P10284	RSRTAYTRQQVLELEKEFHYNRYLTRRRRVEIAHALCLSER
P57784	RIGEGLDQALPCLTELILTNNSLVELGDLDPLASLKSLTYL
P10629	YGAAVAQNRIYSTPFYSPQENVVFSSSRGPYDYGSNSFYQE
P21107	EKLELAEKKAADAEAEVASLNRRIQLVEEELDRAQERLATA
O95096	REHLASLIRLTPTQVKIWFQNHRYKMKRARAEKGMEVTPLP
P15949	GGVLLDANWVLTAAHCYYEENKVSLGKNNLYEEEPSAQHRL
P02662	LARPKHPIKHQGLPQEVLNENLLRFFVAPFPEVFGKEKVNE
Q8MJI9	SQPSTGSSFARPYQSEDWYPNLHPTPAGHLACPPPPPMLPL
Q9UMY1	KKRKLLPDTILEKLTTASQTNIKKSPGKVKEVNLQKKNEDC
Q9CQS3	PEMSNGTLHHYFVPDGDYEENDDPEKCQLLFRVSDRRRCSQ
Q3TQR0	YCEAGVYTIFAILEYTVVLTNMAFHMTAWWDFGNKELLITS
P05937	GNGYIDENELDALLKDLCEKNKQDLDINNITTYKKNIMALS
P60201	LTVVWLLVFACSAVPVYIYFNTWTTCQSIAFPSKTSASIGS
Q28071	PSIQVAENKILVKQSPMLVVNDNEVNLSCKYTYNLFSKEFR
Q9EP72	LPKVVNTSDPDMRREMEQSMNMLNSNHELPDVSEFMTRLFS
Q9UIY3	FEELLLEAHGDYGLRNDYHMNLGQFLEFLKKHKSEHVFQIL
Q9ESP1	RGLPVRCGQAVRLTHVLTGKNLHTHHFPSPLSNNQEVSAFG
Q8CDY7	CEQIHTRVRELNLPRYKLVCNVVLGPREGQGVHVVSRALWD
Q2HJ98	LEEGDIILTGTPKGVGPVKENDEIQAGIHGVLSMKFKVERP
Q9Z1L4	VQYRTDERLNWIYYKDQTGNNRVFYGNSDRSSTVQNLLRPP
O15266	IASLAESASAAAVVAAAAKSNSKNSSIADLRLKARKHAEAL
A5D7C9	ASFGMTLLGNFQLTNDEEIHNVGTSLTFGFGTLTCWIQAAL
Q7SIB3	DLGLAGSCLARFSTMPFLYCNPGDVCYYASRNDKSYWLSTT
O14645	KCEATEKRESERRQVEEKKHNEEIQFLKRTNQQLKAQLEGI

Q12981	QLAKEQDKESEKQLLLQEVENHKKQMLSNQASWRKANLTCK
O77512	MADDFDHYTNTYQIYSKDLNNCQESLATSDVINWKQHLQIQ
P00551	LYGKPDAPELFLKHGKGSVANDVTDEMVRLNWLTEFMPLPT
Q61142	YMYQLLDDYKEGDLRIMPDSNDSPPAEREPGEVVDSLVGKQ
P04224	ANVAPEVTVLSRSPVNLGEPNILICFIDKFSPPVVNVTWLR
P82918	IPVSPYEDEPWKYLDSEEYHNRNGSRPVWADYRRNHKGGIP
Q5SS90	SVSSKSTEDSKSNQDTETPENPETPEGSEKTPDAEVSSPSE
E9Q9D5	FSLPLYFVSAADGTNVVKLFNDAIRLAVAYKESSQDFMDEV
Q32L00	NQIEENTGHSFNSLLCNLYRNEKDSVDWHSDDEPSLGRCPI
Q8VI88	HSMSTGLLYICVSKSCPSSDNGEDNFKMWTIHPVFGVAKIS
O14595	AQHVGQSSSSTELAAYKEEANTIAKSDLLQCLQYQFYQIPG
A6QQA5	GLQRDRSLAVSCSRLCSSAWNLCTVFGILLGYPVPYTFHEN
A6H7B8	NVALLVLTYVSSSEDFTIHENAFIVFIASSLSHMLLTCILW
Q9QXN5	SLVYRPDVDPEMAKSKDSFRNYTSGPLLDRVFTTYKLMHTH
Q9JKM7	FKDGAFLSGTFIATVGIDSRNKVVTVDGARVKLQIWDTAGQ
Q8TDU5	ANHTLGFILFSWVLNMFITNNLLLFIVPTPNRIGASLLFVT
P08831	KFMSLDTSETSKTSKLSFKENVVMVAASGKILKKRRLSLNQ
Q9CQY7	MNHRAPANGRYKPTCYEHAANCYTHAFLIVPAIVGSALLHR
Q8BFR1	CPHRDCWSTCSLSHDIHTPINVQVLKSRGLFGLNEGQLRIL
Q32L49	RDFRIFESNFVQVTRLGEVANKVTMGVAASSPALELPDLLL
Q3U4G0	HHTPEAIESYYQRYLNGVGKNGAAPVLLELANEVDYAPSLM
Q2YDK0	KDLCYKGHHWHEGCFNCTKCNHSLVEKPFAAKDERLLCSEC
Q8C552	TNIYRDQSFTTWLVATNTSTNDMIILQTLHWRMQLSIEVNP
O14595	LVLTKQGLVSKSSPKKPRGRNIFKALFCCFRAQHVGQSSSS
Q99943	LPTLWFCSPSAKYFFKMAFYNGWILFLAVLAIPVCAVRGRN
Q8WTV1	KHNAVPTVFAFQDPTQQVRENTDPASERGNASSSQKEKVLP
Q6UXP7	QLLWLLKKSNRYSLTIWTGKNDNYSVEDLLYIRDHFDKKQV
Q5QGZ9	QTWQESKMACAAQNASLLKINNKNALEFIKSQSRSYDYWLG
P04394	FTLIEVECLGACVNAPMVQINDNYYEDLTPKDIEEIIDELK
Q5SX19	LVGFLCVAVAVLTWGFLRVWNSAERMRSPEQAGLPGAGSRA
Q6ZQI3	KLPILRSTPEDQILYQTERYNEETFGYEVPVKEEGDYVLVL
Q96G25	NLLEKISKEERESESGGLRPNKQTFNPTDTNALVAAVAFGK
Q6PER3	LLARQGQDVAPPPNPGDQIFNKSKKLIGTAVPQRTSPTGPK
Q9H446	FETDHNLDTSDIQFLEDAGNNVEVDESLFQEMDDLELEDDE
Q8WV92	KYLDQEKEDGKYHKQIKIEENATGFSYESLFREYLNETVTE
A6QNY1	LSNATVTAIEDWDSVQSGNRNVYGIIAAVAVLSISLAAGIL
P28907	SRDLCQDPTIKELESIISKRNIQFSCKNIYRPDKFLQCVKN
Q64ET8	QGSEPNPNKENSEETKLKAGNSTAGSEPESSSYRENCRKRK
Q8BMB3	EEICGAVVSVRFQEDIISIWNKTASDQATTARIRDTLRRVL
P85094	GRSQLQSVLLCGIETQACILNTALDLLHRGLQVHVVVDACS
P06345	RWFRNGQEETVGVSSTQLISNGDWTFQVLVMLEMTPRRGEV

Q3T169	AVQISKKRKFVADGIFKAELNEFLTRELAEDGYSGVEVRVT
P60334	ESNPAEWALYAKFDQYRYTRNLVDQGNGKFNLMILCWGEGH
Q6PFX2	FTIERWQIARCNKSKPQKFINDLMQVLYTNEYMATHSLTGA
Q9D7V2	LKKTLSIPILSEKPLLFNGLNSIDSPESETVDSSFCQEEEP
Q8CG80	SRTDAENLLRLCKEASYLVRNSETSKNDFSLSLKSSQGFMH
Q9UBX3	SGLAGGFVGTPADLVNVRMQNDVKLPQGQRRNYAHALDGLY
A6H759	LKGMLNLKTLTLYQNPLCQYNLYRLYIIYHLPGVELLDRNQ
Q9QYM8	LRLRAQTKQQLLEYKSMIDTNEEKTPEQIMQEKQIEVKIEE
Q4VAA2	SRSGDGGSAGPAGKAITKDENEWKEFEQREVDYSGLRVQAM
Q99JV5	LSCGVSVEWSETRPEFVRGYNHPCGWFCVPLKDSPSQSLLT
Q9NVZ3	NPDQGPKLDLGFKEGQTIKLNIANMKKKEGAAGNPRVRPAS
Q6PFG8	HAANAVHPVHPILGGALSSGNASSPLSATSLPTIGTIRPPH
Q8VI33	IPATSTVQNVLINPSLIGSKNILITTNMVSQNTAESANALK
Q2T9Y6	KTQLEQLYQWAQVKPNSNQVNLASCCVMPPDLTAFAKQFDI
P59796	YVCPGSGFVPSFQLFEKGDVNGEKEQKVFTFLKNSCPPTSD
P20774	AKYNKIKSRGIKANAFKKLNNLTFLYLDHNALESVPLNLPE
G3N131	VSLAALKKALAAAGYDVEKNNSRIKLGLKSLVGKGTLVQTK
Q5PR19	GPSFPARRYGAPRRLCFLPQNTGTPLRVLPSVFWSPPSRKK
Q9UNN8	ADTQVTSGVVTFTLQQLNAYNRTRYELREFLEDTCVQYVQK
Q8C407	VSSADAEDLSGSIAAPDVKLNLGVSGDFIKESTATTFLRQR
P24699	LKRCTTTNPNQRLPKVEILRNAIRYIESLQELLREQVENYY
P31268	QRSGYGAGAGAFASTVPGLYNVNSPLYQSPFASGYGLGADA
O94903	KILSLCPEIKWHFIGHLQKQNVNKLMAVPNLFMLETVDSVK
Q8NF86	QSGSWVLVGVVSWGKGCALPNRPGVYTSVATYSPWIQARVS
Q8BHC4	KHTVVVYCDRDTQLARLMKRNNLNREDAEARINAQLPLKDK
P53519	NNAKDILYVKPYSEKKIDISNRIIKKIKWAMIDDAGAKTKL
A0JLT2	HDNSSLRSLIEKPPILSSSFNPITGTMLAGFRLHTGPLPEQ
Q9NRG1	EAPDYGRGVVIMDDWPGYDLNLFTYPQHYYGDLEYVLIPHG
P08166	RRITGRLIHPQSGRSYHEEFNPPKEPMKDDITGEPLIRRSD
Q9NW81	AAVAPEGNQKKKRTILQFLTNYFYDVEALRDYLLQREMYKV
Q9TR36	ADEDPALFQSVKTKRGPLGPNWKKELANNPDCPQMCAYKLV
Q8N983	FVEREVIDFARRNPGVVIYVNSRPCCVPRVVAEYLNGAVRE
Q8TDQ7	PESYHSYMWNNFFKHIDIDPNNAHILDGNAADLQAECDAFE
P11911	RKRWQNEKFGVDMPDDYEDENLYEGLNLDDCSMYEDISRGL
Q2M2E3	HVMSMGLLHFYKSRSCSDLENGKVTFIFSTLMLFPINIWIF
Q06985	RLTWEATPRSIHEGIATAIMNSDCLVFDTSIAQLFAENGNL
P49862	HCGGVLVNERWVLTAAHCKMNEYTVHLGSDTLGDRRAQRIK
O75792	LFAKMEDTDFVGWALDVLSPNLISTSMLGRVKYNLNSLSHD
Q9D2Z4	FLGSKGDKLVFVEEKAPAQENSYDCGMYVICNTEALCQSLF
P26715	PKGNKNSILATEQEITYAELNLQKASQDFQGNDKTYHCKDL
P51163	SATASLVNKIGLDAEGAGSGNAEKLAEYICSKPSSELPLLF

Q99020	EYFGQFGEIEAIELPIDPKLNKRRGFVFITFKEEDPVKKVL
Q9Y3B6	RVPPVIVLENQGLRWVPKDKNLVMWRDWEESRQMVGALLED
Q9BS40	QRLKSMKEPLEAQNIPDNFGNVSPEMTLVLHLAWVACGYII
C9JJ37	LGLVVHGKAEPFSAALRSLVNNPRYSDVCFVVGQERQEVFA
P58340	FSEPFGRDLLSISDGRGRAHNRRGHNDGEDSLTHTDVSSFQ
Q13103	SYSSEEMIFGDMLGSHKWRNNYLFGLISDESISEQFYDRSL
P59041	TQAQIKAAYYRQSFLYHPDRNPGSAEAAERFTRVSEAYLVL
A5PJK7	TGLGSRTTAHCFIPPRPPSFNPFLAMPMAFAPPPIFGPPLP
O08989	FHQLILRVKDRESFPMILVANKVDLMHLRKVTRDQGKEMAT
Q3ZC27	ARTVDENFDYLFKIILIGDSNVGKTCVVQHFKSGVYMEAQQ
Q8R088	TWNPFKLQYQLRNVRERIAKNLVEKGILTTEKQNFLLFDMT
A6NDU8	QKVKELISFLSEPEILVKENNMHPKHCNLLGDELLECLSWR
P14415	LDVIVNVSDTESWDQHVQKLNKFLEPYNDSIQAQKNDVCRP
P23946	DPQACSHFRDFDHNLQLCVGNPRKTKSAFKGDSGGPLLCAG
Q9HB71	CKEKEKPSYDTETDPSEGLMNVLKKIYEDGDDDMKRTINKA
Q9BQI4	KKVKRSLRQARKKGRHLELANQKLSEKLAAGALPHINARGP
Q9GZN2	SEQEKLSLSGQTNLSVLQICNWFINARRRLLPDMLRKDGKD
Q9H2C2	LIKVFVLTSNFQAIRVTLNINRKLSFLAVLSGLLLESIMVY
Q8NGU1	VMAVDRYVAVCNPLRYNIIMNSSTFIWVIIVSWVLGFLSEI
Q8IWD4	ELVLWKPLPELLSDKPKPSSNTKNYTGESQAKHVAAGTAFP
A6NC51	VFLAVWAISGVWIVFAIAVTNRTVDLSKGFPYISICGSFPP
P59034	RVPNGAFQHLPQLRELDLSHNAIEAIGPAAFSGLAGGLRLL
O70338	TNQRAEIHAACKAIMQAKAQNISKLVLYTDSMFTINGITNW
Q3MHX1	LTEPKEVERFLAQLSEFAATNQISLGPLRSIVKSLLLVPNG
Q9P031	RPGGIEARGEGVSTVGYRNKNVRQKTWRPNHPQAFVGSVRE
P50135	EIDLQILSKVQAQYPGVCINNEVVEPSAEQIAKYKELVAKT
Q86XI6	LALTMVKVFSEFDDPLDMPFNITELLDNIVSLTTAESESFV
O70250	QVKIWRRSFDTPPPPMDEKHNYYTSISKDRRYAGLKPEELP
Q9TS87	LMALGSLAVTKNDGHYRGDPNWFMKKAQEHKREFTESQLQE
Q5T0L3	TALSPDCSLGDTQHGEKLRRNCTIYRPWFSPYSYFVCADKE
Q3UK78	CPRCGNQVHETNPLEMLRLDNTLEEIIFKLVPGLREQELQR
A3KN05	DEKGRKFVNILMCFWYLTSANIPSEAISGARVFQVKLGDQN
P50553	ERNRVKLVNLGFATLREHVPNGAANKKMSKVETLRSAVEYI
P63321	DLEDKRQVSVEEAKNRADQWNVNYVETSAKTRANVDKVFFD
Q2HJ68	TEEQRVELSLWDTSGSPYYDNVRPLCYSDSDAVLLCFDISR
Q5BU09	FGLQRPRQKQQPVPNSDAVLNCPACMTTLCLDCQRHESYKT
Q9JME9	DTQEHKAQALLRVLSRFAPPNMQAAEAVSHIQSSGPRRHGA
O54836	QRRTRKEGSEFKMVATRRNMNPVQSNSGPYFNARSRQRIPR
Q99N84	GGVPPQRTRKTCIRNNKVAGNPCPICRDHKLHVDFRNVKLL
Q9UEE9	VRVTKEVDATSKEAKSFFKQNEKEKPQANVPSALPSLPAGS
P16402	VSKGTLVQTKGTGASGSFKLNKKAASGEGKPKAKKAGAAKP

Q8BRJ4	GGTLLFALRYRVTGREFWDNNGGRDYALLGPEHPAGAGAAE
O75489	LVDLTAVDVPTRQNRFEIVYNLLSLRFNSRIRVKTYTDELT
Q96BH3	NVVSDCMEDESNKLWCPTTENMDKDGKWSFCADTRISALVP
Q8NHY5	LQFLEGQQIHPTTALCNIWDNTLLQLVLVQEDVSLQYFIPA
Q2TA29	SDLRHLRAVPTDEARAFAEKNGLSFIETSALDYTNVEAAFQ
P81125	IAHYEQSADYYKGEESNSSANKCLLKVAGYAAQLEQYQKAI
Q8VDK3	GIFRVVKSEALAKMWGQRKKNDRMTYEKLSRALRYYYKTGI
Q8MJ87	LKILLHNPQLWWLNDSAPESNNRQQSPSQEKIDQLVYMGFD
Q99935	PLYRPRWVPPSPPPPYDSRLNSPLSLPFVPGRVPPSSFSRF
P17667	LKRCTTTNPNQRLPKVEILRNAIRYIESLQELLREQVENYY
O43709	KPGTFDGCISISAVQWLCNANKKSENPAKRLYCFFASLFSV
A2VDR2	ACDRGHKELVTVLLQYRADINCQDNEGQTALHYAAACEFLD
P50518	VQIDQEAYLPEEIAGGVEIYNGDRKIKVSNTLESRLDLIAQ
Q6IB77	TPFLLKSKILSPNGGKPKAINQEMFKLSSMDVTHAHLVNKF
P11456	DEYDNHCGREQRRAVVMISCNRHTLADNFNPVSEERGKVQD
Q149M0	TYATLMLQDSARVRGNQDGNNLRKEGHPAQSSLWRGAALSL
P0C7M9	FTRREPWIGLRRVGDDFHWVNGDPFDPDTFTISGMGECVFV
Q9GZN2	LHRYNAYPSEQEKLSLSGQTNLSVLQICNWFINARRRLLPD
O60725	EYLVTAVNNPKSLSLDSFLLNHSLEYTVAALSSWLEFTLEN
Q8WTV1	QLTFHRFPFSRPELLKEWVLNIGRGNFKPKQHTVICSEHFR
Q8BXK9	DRDPEIELFVKAGIDGESIGNCPFSQRLFMILWLKGVVFNV
Q6PE84	TTQVDGVVYYRIYSAVSAVANVNDVHQATFLLAQTTLRNVL
P17483	RSRTAYTRQQVLELEKEFHYNRYLTRRRRVEIAHALCLSER
Q9D4V7	DSGVGKSSLVHLLCHNQVLGNPSWTVGCSVDIRVHDYKEGT
A2VE40	TVPKPDLDAYVFLRVKERQENILVEPENDEQRDYVIDLEEG
P04632	SGTICSSELPGAFEAAGFHLNEHLYNMIIRRYSDESGNMDF
Q6UW01	RQTVQVSLMLNTWPVISAFANDPDVTREAATSSVLLPLDPG
Q91XE0	AIDQDKFKLSSLDVVHAALVNKFWLFGGNERSQRFIERCIK
Q9CY45	LSSHTLAALQEFYAEQKQSVNPRGDDKYNVGVIEENWQLSQ
Q9CR36	ENSFAATRLFSKKSCIVHRMNKDAMPSLQDLDTMVKEQKGK
P11456	GREQRRAVVMISCNRHTLADNFNPVSEERGKVQDCFYLFEM
Q96BQ1	WVFIGAKDLRGKSPFEQFLKNSPDTNKYEGWPELLEMEGCM
Q60969	KSKLPILQKHGITHIICIRQNIEANFIKPNFQQLFRYLVLD
Q9D9R0	RTRCGHVFCRSCIATSIKNNNKWTCPYCRAYLPSEGVPATD
Q04742	HSFFSAQHRDPLHFYPWVLRNRFFGHRFQASDVPQDGLLLH
Q3SZX5	EVLLEAQDMAVRDHNVEFRSNLYIAESTSGRGQYLKRIRYH
Q2TA29	TILTEIYRIVSQKQMSDRRENDMSPSNNVVPIHVPPTTENK
P32885	IQANVLKADKMDLRESQGWLNRGYEGAAVGAALGAGITGYN
Q6P5C5	FRHCFVHNLCPLLFLAPSGRNLTPAELPAKQREQLLSICDA
P14415	YYGKKFHVNYTQPLVAVKFLNVTPNVEVNVECRINAANIAT
Q17QR4	LGKQRAAPRPGPRSKEKKKVNCKPKNQDEQEIPFRLREIMR

Q8N511	KPPRNPELVARLEKIKIQLANEEYKRITRNVTCQDTRHGGT
P29020	SRSAETAANLQDPNQLYNELNLGRREEYDVLEKKRARDPEM
Q9Y508	SVVCPICASMPWGDPNYRSANFREHIQRRHRFSYDTFVDYD
P97461	KHAFEIIHLLTGENPLQVLVNAIINSGPREDSTRIGRAGTV
O14931	SQPPEIRTLEGSSAFLPCSFNASQGRLAIGSVTWFRDEVVP
P52793	MVEHQEYVACQPQSKDQVRWNCNRPSAKHGPEKLSEKFQRF
Q9D7J9	KQLPQDLRTAYFLASQAMVDNLALQDGQEGIEAFIQKRKPI
Q9JL95	LASVHSYSFNYQIQNLARKINQSIVWIGGILRGWFWKKFCW
P08218	GGEEARPNSWPWQVSLQYSSNGQWYHTCGGSLIANSWVLTA
Q9D3N2	LFLRGTLDEKMKYCFEVFDLNGDGFISKEEMFHMLKNSLLK
Q3T040	KPRRMLMPEKLSQELLEAFCNRGPVIKRKHDMHKMAEANRA
Q9ESX4	RRRRSFQDYTGQKITLEAVLNTTCKKCGCKGHFAKDCFMQP
Q96MN5	DHLLVENIERETFHLCSRLINGPYRRTVRALVFTLKHRAEI
Q9DCK4	TESFPSMQELVAHGKLHYKPNRYFKCENCLLRFRTHRSLFK
Q9BX73	LGGCPVSYLLLCGQAALLLGNLLLLHCVSRSHSQNATAEPE
Q2KJ29	VTECLHTFCRSCLVKYLEENNTCPTCRIVIHQSHPLQYIGH
Q5EA91	FFYHFSILQILGLVTEVNLNNMLCPAISDPFYGPWYRIWAS
P62918	IVCCLEEKPGDRGKLARASGNYATVISHNPETKKTRVKLPS
Q29438	VKIESPCYPCTSPCNPCNPCNPCSPCSPCNPCNPCSPCSPC
Q1RML4	QHSTGDFQYSSDYKNYLAFINHRSHIRGNSNSYGVQPAEEP
P10279	GGWGQGGTHGQWNKPSKPKTNMKHVAGAAAAGAVVGGLGGY
Q80X71	TAQVQFSKTVIGKARLNNITNIGPLDMKQIDYTVPTVIAEE
Q9BQR3	VGQSWLQAGVISWGEGCARQNRPGVYIRVTAHHNWIHRIIP
P09025	LYGAQQEASVVQYPDCKSSANTNSSEGQGHLNQNSSPSLMF
Q8BZJ7	ELKDPAKFKDLYQFTFTFAKNPGQKGLDLEMAVAYWKLVLS
Q9CRA8	TSVLAGVYGPAEVKVSKEIFNKATLEVILRPKIGLPGVAEK
Q9UBY8	DLFLVIHHLFAFLGFLGCLVNLQAGHYLAMTTLLLEMSTPF
Q6QHK4	QSYSNNSSESHTSSARQLSRNITQHISCAFGLKNEEEGPWA
P11226	QFVDLTGNRLTYTNWNEGEPNNAGSDEDCVLLLKNGQWNDV
Q8BG17	GRNKKKKKRDGDDRRPRLVLNFDEEKRREYLTGFHKRKVER
Q9CWY9	LQLELPLDLAVLQDIEQELCNEEKSIISEYEEDLEFDESCL
B9EJG8	LTFGFGTLTCWIQAALTLKVNIKNEGRRVGIPRVILSASIT
O75494	YSPRNSRPTGRPRRSRSHSDNDRFKHRNRSFSRSKSNSRSR
Q9JKK1	QGPSAATREEIDWTTNELRNNLRSIEWDLEDLDETISIVEA
Q3UBG2	ATVHMDTFQVARIAYCTADHNVSPNIFAWVYREINDDLSYQ
P97348	DMARSVGAVAYLECSARLHDNVEAVFQEAAEVALSSRRHNF
Q969W9	PPYQGPCTLQLRDPEQQLELNRESVRAPPNRTIFDSDLMDS
Q9JJR9	DNEKSLSLGLQTLRSLKCIINLDKHRLIVGKTDKEEIPFVE
Q5T036	EFWSGALSQPSSVPTRPRTPNRDSWRRAWAARGLHPRPSIL
Q99KL7	KMLDKYIYGAQGILLVYDITNYQSFENLEDWYSVVKTVSEE
Q8BJN4	ISWSPSEPCLEDYYHIMYRPNWNSIFSGYLRYNFHHEEKVP

F1MIW6	PYNYLNPLDSAWSSLKWFIINNRKEFCLQSVDGVYSYQYIL
Q3UGC7	LKKLQEESDLELAKETFGVNNTVYGIDAMNPSSRDDFTEFG
O35417	DCLSLCSLCAVRIQDGPRPINPLICSLECQDLVPPSEEWET
Q3V0I2	FCSFLRRRLKRRQEERLREQNLRALELEPLELEGSLAGSPP
Q8NG50	NGCSKRIIKLQELSDLEERENEDSMVPLPKQSLKFFCALEV
Q9D819	VLGILAMIDEGETDWKVIAINVDDPDAANYKDISDVERLKP
Q9UF02	GLSLVVGLVLYISSINDEMLNRTKDAETYFNYKYGWSFAFA
Q99P58	QSFLNVRNWMSQLQANAYCENPDIVLIGNKADLPDQREVNE
Q0VCB1	LLKNLRLVKRLYFGLRVLPPNLVNLNFHQSLTEDQKLVDEG
Q8C4A0	LLYGVTPGGIRETLGVNVVHNSTSTGQAVAVELVLTLQLVL
A6NJY1	SNIVGISVSTLSLALCVRILNIYLLMCFAGFSFKEKIFIAL
Q5SXG7	GTSFLVAFCLRVEPFTFPGDNTGVNNVRFRCSDGVELEGPG
Q9EST4	PAVSVGNVGQLAIDLIISTLNMCKIGYFYTDCLVPMVGNNP
Q5TBK1	QKRARKAMRNGISPIIIDNTNLHAWEMKPYAVMALENNYEV
Q17QR7	EVDEKPFVCVQCGKTFNNTSNLRTHQRIHTGEKPYQCSECG
P27814	REKCCVFIQENLNKTTDCSVNLECPQDWLLHRDKCFHVSQV
Q8IVN8	PPAMNSVSLRCSGDGLDSDGNQTLHWQAIGNPRCQGTWKKV
Q91YQ1	YRDASACVIMFDVTNATTFSNSQRWKQDLDSKLTLPSGEPV
Q5ND56	PFMYWSYGQQKGLSLLQVPFNIPLHCNMANAVLISPQLYWF
Q8IUW5	VNENSDTVGQIVHYIMKNEANADVLKAMVADNSLYDPESPV
Q9UIV1	QLLRCNVDLLKIIQLGLTFMNEQGEYPPGTSTWQFNFKFNL
P26892	TAGLLEYQIYLDYLQNEYEGNQENVRDLRKNIRTLIQILKQ
Q3SX00	QGNTALHLCGHVDTIQFLVSNGLKIDICNHQGATPLVLAKR
Q5E997	SFKFDHLRKEATDPRPYEAENAIESWRTSVETALRAYVKEH
Q3SYZ9	DALAAGRLPDVLAPQYPWQSNDMAMNMLPPNHSHDFLLEPP
Q17QK8	AGTLGDVSKRWVDLTDPTEVNNYWTLNLTSTLCLTSSILQA
Q8R3G9	LQVAAGILGAAFKPEYNRILNETLYENAKLLSDNTDEAKDF
Q567V2	PAAQFVNFLFVPPQFRVTYINGLTLGWDTYLSYLKYRSPVP
O43482	VVLEAPFLVGIEGSLKGSTYNLLFCGSCGIPVGFHLYSTHA
O08738	QHDVPVVPLDMVDHQTDKLDNVTQVDAASVYTLPAGADFLM
A3KN05	AKETLHVLKEKVTSLPDNHKNALAADIDEIVYTSTGDISIY
P19639	LLEYTDSSYEEKRYVMGDAPNFDRSQWLSEKFNLGLDFPNL
Q810N5	LPMNLNEESCIKDFFEQMIRNFKEMQLMVDGKHKQMQKEPL
Q9R0Q4	RWGGRSAENPPSGSVRKTRKNKQKAPGNGDGGSTSEVPQPP
Q9CQG0	QRKQLNDTLDAIKDSTQRVENQVFHMWRFYNYARMRKVADF
Q8N2C3	CLSSNDISCLKGVHLCQVLMNHKVFEPVGMKKLFKKEKELE
Q03401	VLFFLAAVLPPSLLQDSSQENRLEKLSTTKMSVQEEIVSKH
Q9QYI5	VSTSTTFVQGRRITTRRIMENGQERVEVEEDGQLKSVSING
Q9D1D6	ENPKVKQKALIRQREVVDLYNGMCLQGPAGVPGRDGSPGAN
Q9UPY8	KNPPSARNGGHETDAQILELNQQLVDLKLTVDGLEKERDFY
Q9NYV7	SRFCLQWASMLNNFCSYFNLNYVLCNLTITWEFFNILTFWL

E9Q9D5	GTWYAELREFRPEIPCILVANKIDADIQMTQKNFSFAKKFS
Q2HXU8	STKELDQINGSKGCAYFQKGNIYISRCSAEIFWICEKTAAP
A6NJW4	GLSAVPSGIPNDTRKLYLDANQLASVPAGAFQHLPVLEELD
Q8K2W6	VPDRCFSHLQPSLLQRAKPSNYLLDRKTTDKLKKKKRRKRR
P47741	SGYKLGVDCVPCPPGHFSPGNNQACKPWTNCTLSGKQTRHP
Q9BUJ0	LQLLSQRGYRAVALDLPGFGNSAPSKEASTEAGRAALLERA
Q5SPV6	QHPKPCVDRRRVNYAKFIHTNARTYNEPVPYIDNKGPEKQR
P35625	GDWGAEACTCSPSHPQDAFCNSDIVIRAKVVGKKLVKEGPF
P61294	EQRAKELSVMFIETSAKTGYNVKQLFRRVASALPGMENVQE
Q3LI83	YVCNCHIPTRNASKACQTLRNGSNCFGQLNCLSKSFQTLNH
Q02738	EVWDDDQKDFICNTKQPGCPNVCYDEFFPVSHVRLWALQLI
Q9NZ63	KKDSETSFVPTNMAVNYVQHNRFYHEELNAPIRRNKEEPKA
Q58DU5	VAGLLADARSLADIAREEASNFRSNFGYNIPLKHLADRVAM
P62258	VIGARRASWRIISSIEQKEENKGGEDKLKMIREYRQMVETE
Q91XQ6	ILLTLKGYLISCVWSCYRYINGRNSSDVLVYVTSNDTTVLL
Q3T0X7	YIEIKKGVTEDDGRPIYALVNLATTSVSKMASDFAENELDL
P25942	TEFTETECLPCGESEFLDTWNRETHCHQHKYCDPNLGLRVQ
Q13795	AFEKVVTSEALCGVPVLVLANKQDVETCLSIPDIKTAFSDC
Q9ESG4	NYTLPAAEVQSAIRKNRNRINSAFFLDDHTLEFLKIPSTLA
P11403	VRSGAGDYLLGLKRLRRLYCNVGIGFHLQVLPDGRIGGVHA
P28067	RYTAIAYWVPRNALPSDLLENVLCGVAFGLGVLGIIVGIVL
P0C672	LIGMGSSTVLFCLLGYIGIHNEIRWLLIVYAVLITWTFAVQ
Q6QRN6	PPTSLSRSPPTVLVICGPGNNGGDGLVCARHLKLFGYQPTI
Q62283	FRHEIKDTFLRTYTDAMQNYNGNDERSRAVDHVQRSLSCCG
Q8N144	ELGHLLWKGRPRAGERDNRCNRAHEEAQKLLPPPPPPPPPP
P41732	HEIKDTFLRTYTDAMQTYNGNDERSRAVDHVQRSLSCCGVQ
P01193	TENPRKYVMGHFRWDRFGPRNSSSAGSAAQRRAEEEAVWGD
Q9D1C8	AMERIKEDRPITIKDDKGNLNRCIADVVSLFITVMDKLRLE
A5PJN1	GRTGGLGTRGRVSPVEFRNKNVKRKTWRPNHLQAFAGSVRE
Q60817	QLAAAEKFKVQGEAVSNIQENTQTPTVQEESEEEEVDETGV
P53516	GVLSVAGLLSAGAYAAGGEGNMSASATETNARVFSLHLGAT
P06753	EKLELAEKKAADAEAEVASLNRRIQLVEEELDRAQERLATA
P19075	FLFWLCGILILALAIWVRVSNDSQAIFGSEDVGSSSYVAVD
Q5JQF8	RPVRIMWSQRDPSLRKSGVGNVFIKNLGKTIDNKALYNIFS
P02253	VSKGTLVQTKGTGASGSFKLNKKAATGEAKPKAKKAGAAKP
Q16890	KKFGDMSYSIRHSISMPAMRNSPTFKSFEERVETTVTSLKT
F6YCR7	RGELARTLNVPEVKVKVWFTNRRAKQRKIERREMLRNIPPG
Q9P2Z0	AAASQITCENELVQTQPHADNPSNTVTSVPTHCEEGPVHKS
Q9BZX2	EEFCLPTKKYADVIIPRGADNLVAINLIVQHIQDILNGGPS
Q99LV7	RERNLTEAVMLSESFNIEAPNYLSNESAVLIYARQDAQCID
Q61334	EKLKLGLRNDNAEEHLLEAENKKLIESKENLKTELKKASDA

Q5SYL1	GAVSCRQGQHTRVSAPQKGDNIQGHWVQGWKSLWCGMGTIR
O75792	DVLSPNLISTSMLGRVKYNLNSLSHDTATGLIQYALDQGVN
Q9MZ13	LTFTQKWNTDNTLGTEISWENKLAEGLKLTLDTIFVPNTGK
Q8C0Y1	GNLPKESVKILRDWLYLHRYNAYPSEQEKLSLSGQTNLSVL
Q9CQY7	HMCDRMVIYFFIAASYAPWLNLRELGPLASHMRWFIWLMAA
Q6UXN8	NRESCYYVSEIWSIWHTSQENCLKEGSTLLQIESKEEMDFI
A7E3W5	TFLWFVGFCFLTNQWAATKKNDVHVEADSARAAITFSFFSI
Q96BD6	VGVATADAPLHSVGYTTLVGNNHESWGWDLGRNRLYHDGKN
P08831	LKNCYSENEDYSSEIDHLSLNQKSFYDASYEPLREDQMNKF
Q9D110	LSTGGLDLIFLPGLGFDKDGNRLGRGKGYYDTYLKRCVQHQ
Q8N1N2	AHSRCLQSESCNTQVKEYCRNDWSMWKVFLACLLACVIMTA
Q3SZ45	TVCERGTPIRCGQPIRLTHVNTGRNLHSHHFTSPLSGNQEV
Q8VE70	NPGLTQDIIMKILEKKSVEVNFTESLLRMAADDVEEYMIER
Q96PU9	FRVDSTPGPAAYMLPMVMGPNTVGKASQPSFSIKGRSKLGG
Q6ZWZ2	PIDYPYSPPTFRFLTKMWHPNIYENGDVCISILHPPVDDPQ
Q9NYN1	RALPHQAPLTARHGLASCTFNTLSTINLKEMPTVAQAKLVT
P13725	DLERSGLNIEDLEKLQMARPNILGLRNNIYCMAQLLDNSDT
Q9BXY0	LGNKQFCSFKIRTKTQSFCRNEYSLTGLCNRSSCPLANSQY
Q8R1J3	LRNDSPQAKRKKNKKKKEYLNEDVNGFMEYLKQNSQVLHNG
Q28105	IQQLQPLQEPDSWALTKIYLNRYLEEFVGLVQVVHQERGVT
P62242	ALPLGRKKGAKLTPEEEEILNKKRSKKIQKKYDERKKNAKI
Q6UXN2	TSSKPWTAVQKSHYTIWDKPNAGFFNITMIQLTQNDSGFYW
Q9ERE7	SILKMTKKGKTLMMFVTVSGNPTEKETEEITSLWQGSLFNA
P0DMS9	MDEKVKRSFVLDTASAICNYNAHYKNHPKYWCRGYFRDYCN
P13634	GVLMKVGPANPSLQKVLDALNSVKTKGKRAPFTNFDPSSLL
Q9NPI7	EYICGSHGVEHRVYKHFSSDNSTSTHQASHKQIHQKRKRHP
Q0V882	LVVMCGFVLFDTQLIIEKAENGDKDYIWHCVDLFLDFVTLF
O75628	RACAVVFDCKFIETSATLQHNVAELFEGVVRQLRLRRRDSA
Q32PI6	YRRWGDSVLFVDLEHEDMPQNVVAATSGLKTFNLIPAIGLN
Q7Z3H0	LDLERRDQRGLTALMKAAMRNRCECVATLLMAGADLTAVDP
B6A8R8	AKAPYRASGPSNLLEISVIDNHLPQDLAASTFPPQLTATSP
Q6UWM5	LGGASTAIFVCNYGPAGNFANMPPYVRGESCSLCSKEEKCV
Q13162	INTEVVACSVDSQFTHLAWINTPRRQGGLGPIRIPLLSDLT
Q9CYX7	AAESSAGTNSGWADAMAKILNKKTPKSKATILTKNKELEKE
P17041	ERLHTGQKPYECAICQRSFRNQSNLAVHRRVHSGEKPYRCD
Q2KJF9	QHDQRGLGGILLEDIEEGLPNSQKAVKALGDQILFVNRPDK
H3BS89	PLRASRSRLDRWEGKLLRARNRRQLFAMSPADECSRQYNST
P15428	RKVVDHFGRLDILVNNAGVNNEKNWEKTLQINLVSVISGTY
P49798	VVICQRVSQEEVKKWAESLENLISHECGLAAFKAFLKSEYS
Q9D1R1	AKFRSPIISEIIEKKFEHYRNDKTLNIHGTLVFGTSSSLSG
Q3UHX8	LAAGTPHGITDILSRPVATPNSSLLSGYPHVAGFGGLSSQG

Q8BFR6	LDHTLERWITKEECPLYNGGNVILEYLNDEEQFLKNVDSYL
Q99811	REELARRVNLSEARVQVWFQNRRAKFRRNERAMLASRSASL
Q5UCC4	EHSFEIDDSANFRKRGSLLWNQQDGTLSLSQRQLSEEERGR
Q9JJV3	AFILSSGGLLTFIILLKNQINLLGFTLMFWCEFTASFLFFL
Q6P050	EDWLKSAFQRSICSRHESLVNDFLLRVCDRLSAVRSPRRRE
Q99895	VCAGGDGVISACNGDSGGPLNCQLENGSWEVFGIVSFGSRR
Q9H019	SDTRPLRHTWKPSPLIVMQRNASVPNLRGSEERLLALKKPA
P52907	PVKIEGYEDQVLITEHGDLGNSRFLDPRNKISFKFDHLRKE
P08263	AGVEFEEKFIKSAEDLDKLRNDGYLMFQQVPMVEIDGMKLV
A6NCQ9	TLAVPVGLPSVPPLDSLGHTNPLAASSPAWRPPPGQARPPG
Q86TN4	RKQRFALQLGDPSTGLLIRANQGHSLQVPKLELMPLETPQA
Q5UBV8	ALHWEHDLGMAFTKNGMKYINKSLVIPESGDYFIYSQITFR
P60202	TSASIGSLCADARMYGVLPWNAFPGKVCGSNLLSICKTAEF
Q9H741	TFKQEERAVRDRNLLQVHDHNQPIPWKVQFNLGNSSRPSNQ
P59025	FYEKMEEAKPADSWDLIIDPNLKHNVLSPGWKQYLELHASG
Q9UGN4	YSTVASPREELHYASVVFDSNTNRIAAQRPREEEPDSDYSV
P07992	QALKPGAKSNSIIVSPRQRGNPVLKFVRNVPWEFGDVIPDY
Q96HF1	KSVLWLKDSLQCTCEEMNDINAPYLVMGQKQGGELVITSVK
Q3T0U1	ADVTLEGQVYPGKGGGSKEKNQTKQEKGKKKKERDLKPRAS
O88822	IYPFVFPLHKVVYLGLYVLVNVWTISIHDGDFRVPQILRPF
Q3UWA4	SGILSCPVCRKPCSEGVLGDNYICHTHQKRVRRFCEASGHL
Q5T9Z0	SRFMYPKEWQHLTMFILLTLNGCVDFMSKNVLPQRCVGLEK
Q6P1S2	KTVLVKGLKYDSKIYWTVHRNLLKAELTALKKGEGIWKEDS
Q9CZE3	FETSAKDNINIDEATRFLVENMLANQQSFPSEEIDLDRIKL
Q969T7	LQGFKGQLIHTYNKNSSACENSGYFQQLEGKTNVILLGDSI
Q17QQ4	DQSFTSPPPRDFLLDIARQRNQTPLPLIKPYSGPRLPPDRY
Q8NBL3	RRHKESCERSRAGADPPDQKNRLMPLSHLPLRDSPPLGRRL
O95456	LSSFVMNSGVWEEVGCAKLWNEWCRTTDTTHLSSTEAFCVF
Q52LC2	YNKLSIQSWFSLRRVEIISNNSIQAVFNPTGVYAPSGYSYR
Q64317	SALANGRALSAGSPPVPPGWNPNSSSGKGSGSSAGSYVPSY
P13727	FSQAWFTCRRCYRGNLVSIHNFNINYRIQCSVSALNQGQVW
Q86XK3	VESEENDQTFSEKPASSTEENCLEFQESFKHIDSEFEENTN
P09428	FVQGEERDNKIPVALGIKDKNLYLSCVKKGDTPTLQLEEVD
Q9P0I2	RENGKYIPKQSFLTRKYYFNNPEDGFFKKTKRKVVPPSPMT
P55061	LFLGGILMSALSLLLLSSLGNVFFGSIWLFQANLYVGLVVM
Q9D1J3	KQDLINRLQAYLEDHAEEEANEEDVLGDETEEEEPKPIELP
Q9QVN7	DKMVTRKNAEGAMDLLRELKNMPITLHLLQSTRVGMSVNAL
P51150	IDLENRQVATKRAQAWCYSKNNIPYFETSAKEAINVEQAFQ
Q0VD50	AGNDAQKFIYESDVLWKHQNNIHLVNEWITNDISSTKIRRA
O00142	FDWILRNMDVSVDLIVYLRTNPETCYQRLKKRCREEEKVIP
Q9NY35	ALFRYNGTVGLWRRCITIPKNMHWYSPPERTESFDVVTKCV

Q8N1Q1	QKITDTLDSIKEKGKQTRFTNFDLLSLLPPSWDYWTYPGSL
Q5SYH2	TLSDLGKQVRSVKALVVTIFNFIITVAAAFVCTYLGSQYVF
Q9DCS2	KAVLITYGPYAVNGKISPQSNVDFDLTLRCRNPEWGLRDTV
P32850	NKVRSKLKSIEQSIEQEEGLNRSSADLRIRKTQHSTLSRKF
Q8C4B4	LLALDTIRPEHVLRLNRVTENYLCKPEDNVYSIDFTRFKIR
Q9CRA0	SGSPKGDLINLRSAGNMSTYNCQLLKACSKKCAPAPVVIGC
Q8VIM0	TDERNVTYQKSSRYQLKGDLNKGDVSLIIKNVTLDDHGTYC
Q07011	DICRQCKGVFRTRKECSSTSNAECDCTPGFHCLGAGCSMCE
Q5TFG8	EPTVTSAVGALLQNRVLVATNEVPTKSGLAMDPASGAKLRQ
Q9NXF7	PPFQKPLTSPSRLSRDHATLNGALQFATKQLSRTLSRATPI
A6QPL2	WAAVIGLFCRQYDIIKDNDSNNNPKEKGKGPEQSPQGRPVG
Q96GD0	GRQALVVGKPSPYMFECITENFSIDPARTLMVGDRLETDIL
Q6BDI9	KEYLGFEYPPSKLCPAANTLNEIFLIHFITFCQEKGVDEWL
P52483	SSDYPFKPPKVTFRTRIYHCNINSQGVICLDILKDNWSPAL
Q9D309	RDKYAKICFEDEVLIGEKTGNVARGINIAVVNYETGKVIAT
Q9H7E9	VSSARNPSTVCLCPEQPTCSNADSRAHPLGDEGGTASKKQK
O14818	DGTPRLYQTDPSGTYHAWKANAIGRGAKSVREFLEKNYTDE
Q9Y399	AVRDAAKMNIPTVGIVDTNCNPCLITYPVPGNDDSPLAVHL
Q91ZX1	VDGSPLTLSFMKYWSKGEPNNLGEEDCAEFRDDGWNDTKCT
P05837	YRNSPVPVCVRNKNRKILYANGAFIELFSREDKPLSGESYI
P08962	TDWEKIPSMSKNRVPDSCCINVTVGCGINFNEKAIHKEGCV
Q9D7X1	RHVLNFLRNGELLLPEGFRENQLLAQEAEFFQLKGLAEEVK
Q8IXF9	LTLLFLLFLAHGVTLDGASANPTVSLQEFLMAEQSLPGTLL
Q9NP50	SGNRIKSNQISKLQKEFKRHNSDAHSTTSSASPAQSPCYSN
Q9ULN7	MKALMNEKAQAALVEFVEDVNHAAIPREIPGKDGVWRVLWK
Q92956	GGTESQDTLCQNCPPGTFSPNGTLEECQHQTKCSWLVTKAG
Q9BQT8	LTATLGRHGVFNMVYFGFYYNVKNMIPVNKDPILEFWRKFG
Q9QZ23	RIEGVKSVFFGPDFITVTKENEELDWNLLKPDIYATIMDFF
Q5E9V3	SGSQQAPFTPPNTPDPRSPPNPENIAPGYSGPLKEIPPERF
O88456	SGTIGSHELPGAFEAAGFHLNEHLYSMIIRRYADESGNMDF
O43736	MPKSTIYRGEMCFFDSEDPANSLRGGEPNFLPVTEEADIRE
Q16740	QATDIAIQAEEIMKLKKQLYNIYAKHTKQSLQVIESAMERD
Q9BXJ4	YSFRGYQGPPGPPGPPGIPGNHGNNGNNGATGHEGAKGEKG
A1E960	GLIGASSSAPLISQRLLSASNSHELLLNLNNGQLLPLQFQG
P02702	CKEDCQSWWEDCRTSYTCKSNWHKGWNWTSGYNQCPVKAAC
Q8WWA1	TDFHKQDGKAGLFSQEQYERNKSSSSSSSSSSSSSSSSSSS
Q14406	FCFSDSIPTSSNMEETQQKSNLELLHISLLLIESRLEPVRF
Q6UE05	VCQKSHCFRLGRQLSKALQVNCVVRKLLVQLRRLYWWVETM
Q9NQ60	EKNGNYYKDIKQYVFTTQNPNGTESEISVRATTDLNFALKN
Q60948	FDGDFARKKTKTAGLVRKGPNNRSSHNELEKHRRAKLRLYL
Q62422	KGRTGLIPSNYVAEQAESIDNPLHEAAKRGNLSWLRECLDN

Q3T0C2	MGPIKDMMKYYGILDPSMIANGLITLIEDDALNGAIMKITT
Q01105	KEQQEAIEHIDEVQNEIDRLNEQASEEILKVEQKYNKLRQP
Q9ZIS7	KASMEKLHALNMLSGDPHRGNFIVSKDGVRIIDLSGKSCTA
Q2KI22	SMVAAGSVAAAAQGLHLGSANGFLSYHRLTRFLSKVIRCDP
Q2KIV9	GTPGTKGEKGLPGLVSHLNENGEKGDPGFPGMPGKVGPKGP
Q3T186	GSGSTIVHAVQRIAERVEQENLKLVCIPTSFQARQLILQYG
Q91XR9	WKWMKVQPKGRGMLGNAIKWNFTKFLIDKNGCEVKRYGPME
Q3SZW3	VIILINAILCKAQAYRHILFNTKINMHGKLCVFCLLCEAYL
Q3KNM2	ILNSIFPGIGCPVPRIPAEANPLADHVSATRILCGALVFPT
O00560	SPASLVGLRFGDQVLQINGENCAGWSSDKAHKVLKQAFGEK
Q8BQM7	QLLDCLSDSFQVTNKLTGVLNTHLGCRLAFIEMKSDGTIKE
P06798	QVKIWFQNRRMKWKKDHKLPNTKMRSSNTASAPAGPPGKAQ
E9Q7Y3	AAGPARELAGSVESLPSSPWNSPRVTPKTALSSQAGSRRAG
P63013	QVWFQNRRAKFRRNERAMLANKNASLLKSYSGDVTAVEQPI
Q5E988	KAQCPIVERLTNSMMMHGRNNGKKLMTVRIVKHAFEIIHLL
Q8TF63	RKSLKSSDPWHPPSLSPNSWNRQAGFRAWSSHLISLSLTCS
P20160	GYDPQQNLNDLMLLQLDREANLTSSVTILPLPLQNATVEAG
P14190	VDGIPHIAFTDYEGASVVLRNPDGETNKKGLAYFVLPMKNA
Q8N5P1	PEESTKKEGVKDTPQAAKQKNKNLKAGHKNGKQKKMKRKWP
P61026	MLVYDITNGKSFENISKWLRNIDEHANEDVERMLLGNKCDM
P59539	AVSRVGLLWVLLLNWYSTVLNPAFCSVELRTTAYNIWAVTG
A8MT33	IKLQKEGSLEPQIEDLISRINDLQQAKKKSSEELRETHSLW
Q8TBZ9	KGSKRPLPLEPLLFLQVPRSNYLHFQEEKQRLHLKKFLLDR
Q86XT9	VPGILPSSQPPISCSEEGAGNATLSPRMGEECVSVWSHEGL
Q8C3M9	KKATGQNGDPVGKGTHTPATNISPTPVLSSAQPFHSSTVMW
Q9Z205	ADASASSLQGSFLKHSTTLTNRQRGNEVSALPATLDSLSIH
Q5BKY9	EQLEKKKKGSKALAEFEEKMNENWKKELEKHREKLLSGSES
P53519	MPPIEKSTKINRKEGRTDSINISIRGCIKLIYRPASVPSPV
Q6PFX2	NQVLRKRKRKRTETANSENANSALEKAQRDPYSGNAFLPGE
P00403	SYMLPPLFLEPGDLRLLDVDNRVVLPIEAPIRMMITSQDVL
Q9NRH1	KKIDEAKDERLCENNAEFNKNCSKSHSGIDCSYVECCRTQE
Q13126	KYVDTPFGKPSDALILGKIKNVDCVLLARHGRQHTIMPSKV
Q3ZCC4	RGAPVPVPPKRTVKRNIPKLNAERLISERGLPALRHVFEKA
P37980	IPIYADKEVFHMVVEVPRWSNAKMEIATKDPLNPIKQDVKK
Q9JL10	SMYDSELWLPASEGLKPGPENGPAKEEPPELDEAELDYLMD
Q14201	MYRGNGHQNHYPPPVPFGYPNQGRKNKPYRPIPVTWVPPPG
Q17R16	LLSMVLILGCITCFALFFFCNTATVYKICAWMQLLAALCLV
P52623	EQKAKALKGQYNFDHPDAFDNDLMHKTLKNIVEGKTVEVPT
O75832	EERVEEAKLLVSQGASIYIENKEEKTPLQVAKGGLGLILKR
Q58DF6	CGYVCWFRPAYKAFRSDSSFNFMAFFFIFGAQFILTIIQAV
P02970	IAFTDYEGASVKLRNTDGETNKGLAYFVLPMKNAEGTKVGS

Q5E982	EPPRYMSVNQAAQQLLEIVQNQRIRGEEPAVTEETLCVGLA
Q2KIX7	GSMNIAGFWILAQFVLLLVANVKSSADSELCGPRGARGPPG
Q96BD8	EINKAVISKYKILHQPKKSMNSVTRNLYHRFIDEETKDTKG
O95416	RAQHMKEHPDYKYRPRRKPKNLLKKDRYVFPLPYLGDTDPL
Q61200	SFNCRIEYEKVDKATKNTLCNYDPSKTCYQEQTQSHVSWLC
O15260	SFVFLNLLGQLTGCVLVLSRNFVQYACFGLFGIIALQTIAY
Q9D176	RGEDLETVQAAYLGLKGHNHNNSSSVGGGNGGPSGGGGKPG
A0PG75	TLRSCTLRITDNSGREVITVNRPLRCNSCWCPCYLQELEIQ
Q3ZBY3	SGYIEANELKGFLSDLLKKANRPYDEPKLQEYTQTILRMFD
Q8R1L4	GKSQILFALVFTTRYLDLFSNFISIYNTVMKVVFLLCAYVT
Q2YDK0	IESGSKDLCYKGHHWHEGCFNCTKCNHSLVEKPFAAKDERL
P01139	VNINNSVFRQYFFETKCRASNPVESGCRGIDSKHWNSYCTT
Q2HJB9	GAMETQSEPSELELDDVVITNPHIEAILENEDWIEDASGLM
A7E3W5	CIFGEGYSNTHDSQQQYCVFNRNEDACRYGSAIGVLAFLAS
Q9EP73	PQHSNFRGRASLPKDQLLKGNAALQITDVKLQDAGVYCCII
P40293	MKFGADIQDDYEDENLYEGLNLDDCSMYEDISRGLQGTYQD
P35288	HSSSNKIGVFNASVGSHLGQNSSSLNGGDVINLRPNKQRTK
Q4G0S7	HNIIKGLQQTIEYQQNLKGENEQLKISADLIKEKLKSHEQE
Q9P2Z0	VICSDHFAPACFDVSSVIQKNLRFSQRLRLVAGAVPTLHRV
P61587	SENSVRDIFHVATLACVNKTNKNVKRNKSQRATKRISHMPS
Q53TN4	HPNGGTEQGARGSMPAYSGNNMDKSDSELNSEVAARKRNLA
Q1LZ83	VTHWHRDHTGGIGDICKSINNDTTYCVKKLPRNPERKEIIG
Q17R06	SNGAILVYDITDEDSFQKVKNWVKELRKMLGNEICLCIVGN
Q5SXG7	GHAQIQVHVEPRYASIVDVTNGGTWGDWAWPEMCPDGYFAS
Q02013	GITSSLVDNSLGRNDLAHGVNSGQGLGIEIIGTLQLVLCVL
Q8MJ50	KGYADIVQLLLEKGARTDLRNNEKKLALDMATNAACASLLK
P28161	PDYDRSQWLNEKFKLGLDFPNLPYLIDGTHKITQSNAILRY
O60238	DNGNGKNGGLEHVPSSSSIHNGDMEKILLDAQHESGQSSSR
P46638	RWLKELRDHADSNIVIMLVGNKSDLRHLRAVPTDEARAFAE
P56537	IEDQDELSSLLQVPLVAGTVNRGSEVIAAGMVVNDWCAFCG
P28845	NTSLNLFHDDIHHVRKSMEVNFLSYVVLTVAALPMLKQSNG
P09417	LLGEEKVDAILCVAGGWAGGNAKSKSLFKNCDLMWKQSIWT
Q9QZB0	YISILSPKEVSLDSRVREVINRSLLDPSPHMYEDAQLQIYT
Q8N2C3	PARELMAVLLTPRFRRLVSQNELPGPGLNGPSSRNRRDGFC
P50171	TRDEFLLHMSEEDWDRVIAVNLKGTFLVTQAAAQALVSSGG
Q16878	AWPDKKSNEMVKKSERVLRENQCAYINDSIGLHRVENISHT
Q8BR26	KKTMTNPTTVIEVYPDTTEVNDYYLWSIFNFVYLNFCCLGF
Q6NZB0	QREEEIEAQEKAKREREWQKNFEESRDGRVDSWRNFQANTK
Q9JHS9	ENILSGNPLLNLTGPSQPQANFKVKRRWDDDVVFKNCAKGI
Q8N5J4	GKEIFYSQCVQPDQEYLSLNNWNANYNYTYANYHELNHHDC
Q9JKL5	NVPDLELNPIRSKIVRAFFDNRNLRKGSSGLADEINFEDFL

P36543	SDADVQKQIKHMMAFIEQEANEKAEEIDAKAEEEFNIEKGR
Q9H3N1	HCKDGEFRRYQGPRTKKDFINFISDKEWKSIEPVSSWFGPG
P21796	PSIRGALVLGYEGWLAGYQMNFETAKSRVTQSNFAVGYKTD
A6NMD0	PDTTEVNDYYLWSIFNFVYLNFCCLGFIALAYSLKVRDKKL
P09210	IDGMKLVQTRAILNYIASKYNLYGKDIKEKALIDMYIEGIA
Q5E9M6	WYVLLLLQPTVYLVTCANLTNGGKSELLKSGGSKSTLKHIW
P61289	CEEAFQGTKVFVMPNGMLKSNQQLVDIIEKVKPEIRLLIEK
Q3SYS0	VGKTVDFRGTMVPSEAPLLHNQVKLVDPMDRKPTEVEWRFT
Q61096	HDLLSSEPEQQKFTISQVFQNNYNPEENLNDVLLLQLNRTA
P0A9Z7	DKTGASERGARGIVALLGPNNKAERIVVIYLRDTPASMAER
Q9Y680	IETFKQIDMDNDRQLSKAEINLYLQREFEKDEKPRDKSYQD
P31041	CRYSYNLLAKEFRASLYKGVNSDVEVCVGNGNFTYQPQFRS
Q6ZTI6	PASEMRPRMLPVFFGESIKVNPEPTHEIRCNSEVKYASEKH
Q3ZBK3	KLSVKLKEEERMVEMFLEYQNQICRQNKLIQEKKENLLKLI
Q9NQ88	LSRSELMSVTPNTGMSLFIINFEEGREVKPTVQCICMNLQD
Q9CR50	QTPMPSEYQNVTVDILCNDCNGRSTVQFHILGMKCKLCDSY
Q8N983	VVIYVNSRPCCVPRVVAEYLNGAVREESIHCKSVEEISTLV
Q8CC84	NNFIREQDLYALKSIKIPVRNHGILTETHQELMPLGASSSE
Q3T144	MGGPYSYVYFFCTLPYIGVHNVVVFVRTSVKISYIGHVTQS
P43431	LCLGSIYEDLKMYQTEFQAINAALQNHNHQQIILDKGMLVA
A0JN69	LYNEWRRTNQRVILLIPKSVNIPSNQQSLLGLHSAKRNSKE
B0FP48	QDIPAPANFSQRGYYLTLRANRVLYQTRGQLHVLRVGNDTH
Q2KIB9	TKHVFKIITKEDRLKVIPKLNCVFTVEIDGFISYIYGSKFQ
Q3T108	MLGSYGSLARFRNISRIMRVNNSTMLGASGDYADFQYLKQV
Q8CDN6	CQGTAATNNISATPTFLFFRNKVRIDQYQGADAVGLEEKIK
Q32L79	ARRLCGQHLLHEIVKLCGDVNWSHVEKEKPFTQLLSQASEK
P35293	RDTFVKLDNWLNELETYCTRNDIVNMLVGNKIDKENREVDR
Q9R088	PPDKDRENDKEKKAVVCIEGNIASGKTTCLEFFSNTTDVEV
P14190	WEWKVGTGLNGFGNVLNDLTNGGTKLTITVTGNKPILLGRT
P97370	KKLVEDLESFLKPYSVEEQKNLTSCPDGAPFIQHGPDYRAC
Q8BUY5	PAFIYAKKRYIEQSQAEIYHNRFDAVQSAHRAATRGFIRYG
Q8BG50	SFVVSFYYGTITWYNIFLVYNEERTFWHKISCCPCLILFYP
P19437	WKRMCTRSKSNVVLLSAGEKNEQTIKMKEEIIELSGVSSQP
Q8N6G2	TTMRTAFTPKTGAVPALIRQNGIRRLGYTYSLSDPILNQTQ
P56537	NRHGLLVPNNTTDQELQHIRNSLPDTVQIRRVEERLSALGN
Q07133	KGAKGVQQRKSPAKARAANPNSGKAKMVMQKTDLRKAAGRK
Q05717	AVKKDRRKKLTQSKFVGGAENTAHPRVISAPEMRQKSEQGP
Q86Z23	ADLMKNGQVRASAIAQDADQNYDYASNSVILHLDVGDEVFI
P22676	LNGDGKLGLSEMSRLLPVQENFLLKFQGMKLTSEEFNAIFT
P41317	WNDGEPNNTGDGEDCVVILGNGKWNDVPCSDSFLAICEFSD
A6H759	LTEVIDLSRFKKLKYLWLHHNKLHGITFLTRNYCLAELYLN

Q32LD7	ASELIMTNVDQISIQVSIEKNLEISKARDIVINRLLQYGST
Q8N6Q1	RNIISLNMDLERDTQRIDEANQKLLLKIQEREDKIQRLESE
P35737	MLACYVWGFYPADVTITWMKNGQLVPSHSNKEKTAQPNGDW
Q2KIJ1	LSTSVCQCLSQKINELKDQENQTYTLRSFQMARVIFNQNGC
Q2HJ54	CAYKLVTVKFKWWGLQNKVENFIHKQERRLFTNFHRQLFCW
Q8IUB2	KRCITDETCPGVKKCCTLGCNKSCVVPISKQKLAEFGGECP
Q1JPA6	NTDLVEFKTLNEKEIEENLKNIFNISLEKKLTPKHGDKFFT
O08756	YHQKKNKIHTLEDFQRVINVNLIGTFNVIRLVAGEMGQNEP
O35718	DSSDQRHFFTLSVKTQSGTKNLRIQCEGGSFSLQSDPRSTQ
P20615	KKRCPYTKYQTLELEKEFLFNMYLTRDRRHEVARLLNLSER
Q86SM5	PLLNFLLPVFSPLATLLACVNSSSKPLIYSGLGRQPGKREP
Q3T133	ELQLRVRQLVEQVEQIQKEQNYQRWREERFRQTSESTNQRV
Q5E9Y0	LLKELNHPNIVKLLDVIHTENKLYLVFEFLHQDLKKFMDAS
P50194	TVPDEVVSIVLNISFNIQPENLERIKEEHRLSMAAENIVGD
Q9UBY8	FHITTATGFFCFENVAVHLSNLIFRTFDLFLVIHHLFAFLG
Q9JIN6	NNSESNSRALLHSDQHQLLTNPKCSYIPPCKRENQKNSESV
Q8WVE0	IFEYDKRFAMYGEEFIFYDYNNPLDLPERIAAHSFDIVIAD
O54998	GQTKEESTEEVKIEVLHRPENCSKTSRKGDLLNAHYDGYLA
Q8VE10	LQLMANSTQMKKVMLTVFKHNHGAYQFFREALQFEIDDSSP
Q8VI33	MPASQSPAVKASIPATSTVQNVLINPSLIGSKNILITTNMV
Q8R2H9	APGAPRFLLTFDFDETIVDENSDDSIVRAAPGQQLPESLRA
P21237	MLSSQVPLEPPLLFLLEEYKNYLDAANMSMRVRRHSDPARR
P00639	LIQEVRDSHLVAVGKLLDYLNQDDPNTYHYVVSEPLGRNSY
Q8VE42	ELLKTHGHLIPTGTQSLWVGNSDEDEEQEEKNEEWYQLQEK
Q9QXV3	KRSKAKAEREASPADLPIDPNEPTYCLCNQVSYGEMIGCDN
Q3ZBV9	VARALVQQGLKVVGCARTVGNIEELAAECKSAGYPGTLIPY
Q5E9E6	ILGPGLNKAGKFPSLLTHNENMVAKVDEVKSTIKFQMKKVL
Q99M54	KQVFTKEEAKQSAETIAASQNSDKPSRDPETPQSSGSKRSR
P14148	GNFYVPAEPKLAFVIRIRGINGVSPKVRKVLQLLRLRQIFN
Q8R0J7	ELQVLFEAYQIKKTKLDKQSNNASLETLLALLQAEGAKIEE
Q3SYW9	LRGGRATRTLLRGGMSLRGQNLLRGGRAVAPRMGLRRGGVR
Q8BSA9	THKGKLMVTEEYIEFLLTIANQKMEENKRRIGRFYNYLQHA
Q8K572	ERMANVGSHVLVEANLNGRMNLTVETDRVTIKSYFKNLGNP
Q9BS92	FWSVEFGGRTNKVFHIWKYDNFAHRAEVRKALANCKEWQEQ
Q2LGB7	LQNLDDAVNGSAWTILLLTENFLRDTWCKFQFYSSLMNSVN
Q9UI43	KTWAGSQSRRLQRRLTEEFQNVRIIKPEASRKESSEVYFLA
Q8N5I3	PQRPSYHDLVFQCGSDSTTDNQTGVRYVSIKPDNRKLANGT
Q9JKT4	NHLTVWFATSLSIFYFLKIANFSDCVFLWLKRRTDKAFVFL
Q9GZY6	YYNWGRFSKPPEDDDANSYENVLICKQKTTETGAQQEGIGG
O08602	DLCQKLRNKVSNTKVDTHKTNGYPHLQVTMIYLQSQGQIPS
Q8IZS5	SKSAEFLMVKEDREATEGTGNPAFNMSSPDLSACQTAEKKV

Q17QR4	DEQEIPFRLREIMRSRQEMKNPISNKKRKKEAQAAFSKTLE
Q2TBU2	DFDWREVEILMFLSAIVMMKNRRSITVEQHVGNIFMFSKVA
P52843	GPDELDLVLKYSSFQAMKENNMSNFSLIKEDQVTNGLKLMR
Q2NL24	GDSVPDLAGCTLLMPAVSVGNVGQLAIDLIISTLNMHKIGY
Q9D504	HYAVCGQNISLANKLLQYKANLEAKNKDGHTPLLLAVAENN
Q8VBX0	ACASGSIECVKLLLSYGAKVNPPLYTASPLHEACMSGSSEC
Q9BTT0	PDSEEEDDEDGDEDDEEEEENEAGPPEGYEEEEEEEEEDE
Q9ULC0	ILFLLPSICSSNSTGVLEAANNSLVVTTTKPSITTPNTESL
A4IFK7	TDPNRPRFTLQELRDVLQERNKLKSQLLVVQEELQCYKSGL
P33782	LVSGQSVTDVQSGSLPQGLKNRLSALLLMNKGFGNGMSAVD
Q6IED9	LSLPIRNQKRLVKSALELGENELFQQFPNPQSSWVQRTQEA
Q13268	LPYMENRRGAVILVSSIAAYNPVVALGVYNVSKTALLGLTR
Q91ZD6	TSCEGNLEVGKGEQVTITLPNIEGSTPPVTVFKGSKRPYLK
Q17QH7	IVLEDEGSQGTDAPTPGNAENEPPDKEGLSPPRRTPAPPEP
Q64329	RAFNLKEEMLTNKSIDCRPSNETLEYIKREQDRWDSKTKTV
P19652	YQTRQNQCFYNSSYLNVQRENGTVSRYEGGREHVAHLLFLR
P25322	MVAAGSVVAAMQGLNLGSPNNFLSCYRTTHFLSRVIKCDPD
Q96A61	EAVGAMDGWDGSIREVLYRGNADEELFQDQDDDELWLGDSG
Q8IZR5	GVLLIMFSLNLHMRIPQINWNLTDLVNTGLSAFLFFIASIV
P10861	VRLQAQSHLHGPKPRYTGTYNAYRIIATTEGLTGLWKGTSP
P26436	GSIDHQTSVQQLPGEFFSLENPSDAEALYETSSGLNTLSEH
Q3ZCD8	YTEGISQRMRVPEKLKVAPPNADLEQGFQEGVSNASVIMQV
Q3ZBQ1	DGASITLPHGTLCECYDELGNRYQLPIYCLSPPVNLLLEHT
Q9Z2Y8	AVPNLSMLETVDSVKLADKVNSSWQKKGPTEPLKVMVQINT
P22794	YLHLAFLMTTVFSLSPGTKANYTRLWANSTSSWDSVIQNKT
Q9BXS1	IVVDENDKVIGADTKRNCHLNENIEKGLLHRAFSVVLFNTK
Q0VCT4	FSMRKVPNRETTEISHVLLCNVTQRVSFWFVVTDPSRNHTL
Q8BJF9	KTMQAVNKKMDPQKTLQTMQNFQKENMKMEMTEEMINDTLD
Q5JTZ5	KLVGSDQRLPPEGDTHLFETNQMTQQGTGIPEAAQLPCQVG
Q5XG99	YFKIQASGETPNSLNTTVIPNGSMAMGTVPGQAPRLAVAVP
P53701	RGTAAENKENLDPSNLMPPPNQTPAPDQPFALSTVREESSI
Q60654	KDWVWIDNGPSKLALNTTKYNIRDGLCMSLSKTRLDNGDCD
Q6IPU0	KNQLGHLESELSFLSTLTGINIRNHSKQTEDLTSTEMTEKS
Q3SYT7	VSLEQYLMEGSYNKVFLAKGNIPAESYTFFIDILLDTIRDE
Q9Y2V0	KSIINSMLRDPSQIPDGVLANQVYQCIVNDCCYGPLVDCIK
Q8TDQ7	ESYHSYMWNNFFKHIDIDPNNAHILDGNAADLQAECDAFEN
Q00322	SMAAVPTLELCHDELFADLFNSNHKAAGAGGLELLQGGPTR
Q5H913	CLRTTEETRRNVTIPIIGLNNSGKTVLVEAFQKLLPSKTDH
Q4U5R4	RQPRSVPNRYTFPCPYCPEKNFDQEGLVEHCKLSHSTDTKS
Q9DA17	NSFMLKVTEYDQDMTLLLMTNNPPPCSISQEKDGAPIYFPP
O95567	NPLIASSFSLVKLVLRRQLKNKCCPPPCKFGEGKLSKRLKH

O08543	ACDHTSKGFKRWECNRPHSPNGPLKFSEKFQLFTPFSLGFE
P62823	TITTAYYRGAMGFILMYDITNEESFNAVQDWSTQIKTYSWD
Q9HDB8	KNTEVLVWEECVANSAVILQNNEFGTIIDWAPRGQFYHNCS
P35705	HFSHLAWINTPRKNGGLGHMNIALLSDLTKQISRDYGVLLE
Q0P5F2	KALKTQNFKEPPFCSLLEQPNIVHDLPAAVLSYCQVWRIPA
Q00356	EEIIGGVESEPHSRPYMAYVNTFRRKGYVAICGGFLITPQF
P08074	RDTVKALHASGAKVVAVTRTNSDLVSLAKECPGIEPVCVDL
P43430	KKKNTQLIPVSEAIPHESFDNETLVNDIMLLKLERKAQLNS
Q9H8W3	KTEIIDFSTDEPKTETESNVNAYEECPSGIPIDMWNKFQEL
O43736	VAVEEIRDVSNLGIFIYQLCNNRKSFRLRRRDLLLGFNKRA
Q91YN9	TANRLMGRTLTVEVSVETIRNPQQEESLKHATRIIDEVVSK
Q2TBX6	IAAMLSTILYSRRFFPYYVYNIIGGLDEEGKGAVYSFDPVG
O09101	CICSASVTTVNLLSYLVVKPNVSSKRSSLSHKVTRALKCCV
Q9DAF8	AADGKGRLPDIASPSRDSPLNIKHKVAHQIWGSEVPCPTFL
Q08E00	RAVHHQAPLTARHGLASCTFNTLSTTSLKEMPAVAQAKLVT
Q9CQD1	NWVKELQRQASPNIVIALSGNKADLANKRAVDFQEAQSYAD
P06624	VGFSLTLGHLFGMYYTGAGMNPARSFAPAILTRNFTNHWVY
O60812	VPSKRQRLSGNTSRRGKSGFNSKSGKRGSSKSGKLKGDDLQ
A6NM10	TGMVLAVLLHQGRLPHLFQRNLFYGQKNKYRAPRGKPAPAS
P28063	LGRRAIAYATHRDNYSGGVVNMYHMKEDGWVKVESSDVSDL
Q29463	AVAFPSDDDDKIVGGYTCAENSVPYQVSLNAGYHFCGGSLI
P62956	FAGREKGRCVASEYFLEPEINLVTENTENILKTVRTATPFP
A6NI73	GFYNKPTLSALPSPVVTSGENVTLQCGSRLRFDRFILTEEG
P15864	VSLAALKKALAAAGYDVEKNNSRIKLGLKSLVSKGILVQTK
P41272	YHTRPHCESCRHCNSGFLIRNCTVTANAECSCSKNWQCRDQ
Q9WV93	SSSDSELDETIEVEKESADENGNLSSALCSMSPTTSSQVLA
Q6GTX8	QQRPDLAVDVLERTADKATVNGLPEKDRETDTSALAAGSSQ
Q9NZ63	VNYVQHNRFYHEELNAPIRRNKEEPKARPLRVGDTEKPEPE
Q3TBW2	HFQRQKLMAITEYIPPKPAINPRCLPPPPKPPKEESGLVRL
P35762	RHDPQTTSLLYLELGNKPAPNTFYVGIYILIAVGAVMMFVG
Q9CR48	LGIATMYVRYKQVHALNPEENLIIKLNKAGLVLGILSCLGL
Q6ICB4	ALQRRSSWKSVASRCKPQAPNHRAAGLENGHCLSKDSSPVG
P10738	VTSIELDSHLFNLSSEKLKSNTRVTLIHQDILQFQFPNKQR
Q3T135	APIPLHPSLQLTNYSFLQAVNTFPAAVDHLQGLYGLSAVQT
O14494	IIIPFSIIVIILGETLSVYCNLLHSNSFIRNNYIATIYKAI
Q16772	IDGMKLVQTRAILNYIASKYNLYGKDIKERALIDMYTEGMA
Q8VCQ3	ADLKRHVEFLVAENERLRKENKQLKAEKARLLKGTAEKELD
Q3ZCH9	MLLVDDRALPDFKGIQTSDPNAVVIGLAPEHFHYQILNQAF
Q14192	CRKQLSGQRFTARDDFAYCLNCFCDLYAKKCAGCTNPISGL
A6NJW4	LASVPVEAFVGLQIQVNLSANPWHCDCALQEVLRQVRLVPG
Q9Z0F5	HLLHHKVPWLYRTFHKVHHQNSSSFALATQYMSFWELLSLT

Q60994	GLETRVTVPNVPIRFTKIFYNQQNHYDGSTGKFYCNIPGLY
Q64329	TGRGVKYWFCYSTKCYYFIMNKTTWSGCKANCQHYSVPILK
Q9JHQ5	ALDEKSKLERVLQDLQLDQENQQDLLKAQDLDDLENTVATL
Q9CXN7	VCLLERTLDEDAHQDIAREMNLSETAFVRKLQPTDDFTQSS
Q3ZBR9	RQKERISEQPLEEREDEELENTALQATEIEWLGFQKSSQVD
Q9CRA7	ENLRKSLLELEIIACGNVTDNGVIALRHFRNLKYLFLSDLP
P19404	LIEVECLGACVNAPMVQINDNYYEDLTAKDIEEIIDELKAG
Q8N614	KNHTGRSTIMEDEPSKEKSINYTCRIMEYPNDCIHISLHLE
Q8IX29	PPWRSSDKHPTDIIRFNYLDNRDPMETVQQGRRKRNQMTPD
O09044	SILGNLKNMALDMGNEIDAQNQQIQKITEKADTNKNRIDIA
Q64368	YPPYPSSPVQVITGYQLPVYNYQMPPQWPAGEQRSYVIPPA
Q3MHN0	FDGGVVLGADSRTTTGSYIANRVTDKLTPIHDRIFCCRSGS
Q96K30	SPAGTRPTPPDFDPPWVEKANRTRGVGKEASKALGAKGSCE
Q9CQS9	MLDLAVTFIEKFETHLETVKNSPHLDANLKQMSKALAKMDI
P35695	IAREVGEVRMQKYLKKFSYGNQNISGGIDKFWLEGQLRISA
Q9D7M1	GQIQEAIALINSLHPELLDTNRYLYFHLQQQHLIELIRQRE
Q9QUK3	LSSSLNATYRSLAAKEKVFWNLAATRAVFGVQSTTAGLWAL
P15514	IPGYIVDDSVRVEQVVKPPQNKTESENTSDKPKRKKKGGKN
O75323	LQRAAPCSLLPRLRTWTSSSNRSREDSWLKSLFVRKVDPRK
Q8BVN8	PVRRELYSQCFDELIREVTINCAERGLLLLRVRDEIHMTIA
Q9BR09	RLGVLFCPRPDGTADMHIIINGEDMGPSARGLPAAQPLYAV
P49615	EGVPSSALREICLLKELKHKNIVRLHDVLHSDKKLTLVFEF
Q8IVN8	WMQYLREGYTVCVDCQPPAMNSVSLRCSGDGLDSDGNQTLH
P23185	IGLKNRVWAGYTDSYVAKTMNLDQRTVAPLIVQFFGDVNDD
Q5U4E0	YSLGDCSVRWAYILAIIGILNALILSFLAFVLGNRQTDLLQ
O70552	CRYGEKKHPFTIASFKGRWENWELAQHVSCAVNRATGDCSS
Q9EST4	FRYLLTPCLQKSVQNKIKSLNWLEMEKSRCIPEMSDSEFCI
Q0IIC4	DTALEELCKPLYCKLCNVTLNSAQQAQAHYQGKNHGKKLRN
Q6ZRP0	RSMFSPYREGPIRGPCALAPNPSSERRSPRPIFDLEFHLLE
Q3SZG6	LQRATMDATRTTRHLEETIDNFEKQKIKDIKTIFSEFITIE
Q9BQ15	PHPPHTPSHPPSTRITRSQPNHTPAGPPGPSSNPVSNGKET
A6NCD4	EQRPVNRSYPKCFSLGVNLQNVAESEEEEFMKEFILTDILK
Q9NZ63	KDKISEEEDLHLGTSFSAETNRRDEDADMMKYIETELKKRK
Q1JQ93	KGNGGVYGWDLKRKIISRVANFITQILLRPGASDLTGSFRL
P63013	WFQNRRAKFRRNERAMLANKNASLLKSYSGDVTAVEQPIVP
Q9QZM3	NEPDFNPPRLGAETLPRATVNLEVWRSLNDRLRLTQNYEAY
Q96BX8	ALSAPRYMDLLMDWIEAQINNEDLFPTNVGTPFPKNFLQTV
Q4VA44	MRVHTGEKPYVCSECGRGFSNSSNLCMHQRVHTGEKPFKCE
Q9CPZ1	FFALNRNLEEESSFTRLQDQNRTREGQLPPLRETWYGRLPA
Q9CR78	PEEEPEYHQDAAAQESFAVSNRELCEDEKEFGPFPVCEGTS
Q8N0Y7	SLQGIAKHVEGLSEEAIMELNLPTGIPIVYELDKNLKPIKP

Q96E40	SGTPVTNNLLEKCKTLVSQSNDLSSLRAKYPHDVVNHLSCD
Q96EH8	LTGDLVRFWVDRRGCLFAKVNAGCRLLLREGVPVGAPLWAV
Q58DT0	GIFRVVKSEALAKMWGQRKKNDRMTYEKLSRALRYYYKTGI
Q80VY2	EPKGEKGQSRELGRKFALTANIFRKFLRSVRPDRDRLLKEK
Q07133	MSLAALKKALAAAGYDVEKNNSRIKLALKRLVNKGVLVQTK
Q17QQ2	HSVVGVEISELGIRDFFTEQNLSYSEEPIMEIPGAKIFKSS
Q8QZY6	REKCGVPFSCCVPDPAQKVVNTQCGYDVRIQLKSKWDEFIF
A0JLT2	YNLEQAYNKFCGKKVKEKLSNFLPDLPGMIDLPGSHDNSSL
P11352	LTGGEPVSLGSLRGKVLLIENVASLUGTTIRDYTEMNDLQK
Q9CZJ0	KVTITVDEYSSNPTQAFTHYNINQSRFQPPHVHMVDPIPYD
Q3MHQ4	ERLQSKLSDPHAIEAEKFQCNLCNKTYSTFSGLGKHKQLHC
Q8WZ71	HFCCLDFSLEELQGEPGWRLNRKPIESTLVACFMTLVIVVW
Q9D8T0	GLPQPCPEEHLSFRIVSGAANVIGPKICLEDKMLMSSVKDN
Q8VCZ2	FVPDGPHLHLYHENQWVKLMNWQHSTMYLFFGVSGLMDMIT
Q99944	CHVDVDECRTSITLCSHHCFNTAGSFTCGCPHDLVLGVDGR
Q9NVV0	GILSCLLLAEPPLKFLANHTNILLASSIWYITFFCPHDLVS
Q92914	ANPDGSIQGTPEDTSSFTHFNLIPVGLRVVTIQSAKLGHYM
Q8R173	HCSVCKRCIRKMDHHCPWVNNCVGENNQKYFVLFTMYIALI
P63101	FEISKKEMQPTHPIRLGLALNFSVFYYEILNSPEKACSLAK
O15520	YNTYASFNWQHNGRQMYVALNGKGAPRRGQKTRRKNTSAHF
P19437	LSAMLISAFFQKLVTAGIVENEWKRMCTRSKSNVVLLSAGE
Q64704	FMDIAMLVENQGEMLDNIELNVMHTVDHVEKARDETKRAMK
Q9NYW5	IASVILNFVGIIMNLFITVVNCKTWVKSHRISSSDRILFSL
P30301	VGFSLALGHLFGMYYTGAGMNPARSFAPAILTGNFTNHWVY
Q96B33	LSGVVLFVAGLLGLIPVSWYNHFLGDRDVLPAPASPVTVQV
Q3ZBP0	NCHEQTYEEFLRTFTHLSKDNVTKREAFGTNSSENNFTSIK
P20160	DQCRPNNVCTGVLTRRGGICNGDGGTPLVCEGLAHGVASFS
P42125	NMMRKATADNLIKQREADIQNFTSFISKDSIQKSLHMYLEK
Q9JJW0	QMHSFLAAFIGGLLLFGENNNINSQINMYLTSRVLYALCRL
Q2TJ95	IGVCLSSCPSGYYGTRYPDINKCTKCKVDCDTCFNKNFCTK
Q96G25	YGRLTWPSVLDSFALLSGQLNTLNKVLKHEKTPLFRNQVII
Q96EY5	GGLLERTASRLGSRASTLRRNDSIYEASSLYGISAMDGVPF
P17041	HTGQKPYECAICQRSFRNQSNLAVHRRVHSGEKPYRCDQCG
O35943	TVNAGATRHAHLNLHYLQILNIKKQSVCVVHLRNLGTLDNP
O88838	GVATALAPLQADHYAALLGSNSESWGWDIGRGKLYHQSKGL
P19632	FSRKMSVQEYELIHKDKEDENCLRKYRRQCMQDMHQKLSFG
Q2M2E3	FIFSTLMLFPINIWIFELERNVSIPIGWSYFIGWLVLILYF
Q9D9M5	RIVYIGDGGNDVCPVTFLKKNDVAMPREGYTLHRTLAKMSQ
Q58DN3	LQNLIDFAQEYWSCCGARGPNDWNLNIYFNCTDLNPSRERC
Q16667	EPIEDEQTPIHISWLSLSRVNCSQFLGLCALPGCKFKDVRR
Q80ZQ3	NTFIRHYRLYQYVLSQDQEVNLTVAHEQICAPPQPLPLTDG

Q9D8C4	NLEDCRLRVQVQPLELPVVTNIQVSSQPDNHRVLVSGFPAG
Q6NZQ8	CVVWSLYVLIDRTAEEIKQGNDNGVLEWPFWTKLVVVAIGF
P51572	PKRWQKIFKSRLVELLVSYGNTFFVVLIVILVLLVIDAVRE
Q8BS03	MIAILDYHNQVRGKVFPPAANMEYMVWDENLAKSAEAWAAT
Q9BVC5	NAFRKLSNSSSSVSPLILSSNLPVNNKTEHNNNDAKQNHDL
Q8BG84	QKKQGLPNNKRQQQRPEERLNLATNGLEMTPDIVADDRLPE
P50225	SPFMRKGMAGDWKTTFTVAQNERFDADYAEKMAGCSLSFRS
P39905	PDKQMAVLPRRERNRQAAAANPENSRGKGRRGQRGKNRGCV
Q3U4G0	QRYLNGVGKNGAAPVLLELANEVDYAPSLMARIILERFLQG
P04975	ASGASEDMGATVNGDVFQEANGPADGYAAIAQADRLTQEPE
O70514	LSFLLLATQAFSEKVRKRAKNAPHSTAEEGVEGSAPSLGKA
Q8BLR7	MLQVLAKDCYPETYVPTVFENYTACLETEEQRVELSLWDTS
Q99LU8	NNPNQSVFLFIDRQHLQTPKNKATIFKLCSICLYLPQEQLT
P30084	ICPVETLVEEAIQCAEKIASNSKIVVAMAKESVNAAFEMTL
P04095	QSDNEDARIHSLYGMISCLDNDFKKVDIYLNVLKCYMLKID
Q3MHR7	ELQAHGADELLKRVYGSYLVNPESGYNVSLLYDLENLPASK
Q91V08	IGLHRESSEHPWKWTDNTEYNNTIPIRGEERFAYLNNNGIS
O94811	FRRFAVHGDARATGREMHGKNWSKLCKDCQVIDGRNVTVTD
O95997	KTFDAPPALPKATRKALGTVNRATEKSVKTKGPLKQKQPSF
Q99LS3	DGATDMEACPPADAFIGFGGNVIRQQVKDNAKWYITDFVEL
P09428	KGDTPTLQLEEVDPKVYPKRNMEKRFVFYKTEIKNTVEFES
Q0ZUP1	LVGFLVQKPLIEKCSVAVQENRTEPTGRSATLECPRDWHPH
Q3T013	PPPAGLNSSWVELPMNSSNGNDNGNGKNGGLEHVPSSSSIH
Q17QF9	PAGEIGNGTSGAIYFDQVLVNEGGGFDRTSGSFVAPVRGVY
P19217	LVDKIIKHTSFQEMKNNPSTNYTTLPDEVMNQKVSPFMRKG
Q2KJ37	ACSYATVYLIYMKFKATYDGNHDTFRVEFLVVPVGGLSFLV
Q9D3J9	CRDVIRETYLVPPSCKSICKNYNDLHIAGGQVMAINSVMAN
Q93LM8	DKTGASERGARGIVALLGPNNKAERIVVIYLRDTPASMAER
P13980	LSSVLVLSGCGAMSTAIKKRNLEVKTQMSETIWLEPSSQKT
A4FUA8	LLLNNDNLLREGAAHAFAQYNMDQFTPVKIEGYEDQVLITE
Q9CY21	EHSGPPELFYDQNEARKYVRNSRMIDIQTKMTERALELLCL
P35695	LASSITENTFWNKEFSAEAVNGVFVLCKSSSKLACATNNLA
P09038	ASKCVTDECFFFERLESNNYNTYRSRKYTSWYVALKRTGQY
O14798	SFKGEECPAGSHRSEHTGACNPCTEGVDYTNASNNEPSCFP
Q9UMR7	SSTFWHPREPSDPNERCVVLNFRKSPKRWGWNDVNCLGPQR
Q9D1D6	RESFEESWTPNYKQCSWSSLNYGIDLGKIAECTFTKMRSNS
P30048	VNCEVVAVSVDSHFSHLAWINTPRKNGGLGHMNIALLSDLT
Q9NS61	ALREEAPREHVESFFQKMDRNKDGVVTIEEFIESCQKDENI
Q58DM8	GFLSHWDQLTRVKKPVIAAVNGYALGGGCELAMMCDIIYAG
P07992	IPDYVLGQSTCALFLSLRYHNLHPDYIHGRLQSLGKNFALR
Q8TBF5	ALENEDICQWNKMKYKSVYKNVILQVPVGLTVHTSLVCSVT

Q5E971	TGRIPDQLVILDMKHGVEAKNYEEIAKVEKLKPLEVELRRL
P05529	VFNNLSLKIEQGELIGLLGENPAGKTTLFNLIRGGVSNYEG
P53674	FDRVRSIIVSAGPWVAFEQSNFRGEMFILEKGEYPRWNTWS
Q99N84	YSWQQPPERELSRLRRLYQGNLLEESGPPPESMPEMPTTPP
P07146	GFLEGGKDSCQGDSGGPVVCNGELQGIVSWGYGCAQPDAPG
Q3TQI7	VPTNMAVNYVQHNRFYHEELNAPIRRNKEEPKARPLRVGDT
P10279	HDCVNITVKEHTVTTTTKGENFTETDIKMMERVVEQMCITQ
Q3SX42	QTMQNFQKENMKMEMTEEMINDTLDDIFDGSDDEEESQDIV
Q8CJ61	VTGVLLILFSLNLHMRIPQINWNLTDLVNTGLSTFFFFIAS
Q9R0Q9	LLALLSPLTPLAVVTLLQASNVPAVVVGKLLQAATNYRNGH
P20851	ICKSRDCDPPGNPVHGYFEGNNFTLGSTISYYCEDRYYLVG
Q9NZQ0	MKQELGPHGNMENIIFVVCANKIDCTKHRCVDESEGRLWAE
Q8N614	SFVTFLQPVRETQIIMRIFLNPSNFRNFTRTCQDITGEFKM
Q9Y3A4	AASLRVLERERRKRSRKELLNFYAWQHRESKMEHLAQLRKK
P47754	APPGEFNEVFNDVRLLLNNDNLLREGAAHAFAQYNLDQFTP
Q9DCS2	AAGHLLKTKAVLITYGPYAVNGKISPQSNVDFDLTLRCRNP
P59024	VHYEGYLEKDGSLFHSTHKHNNGQPVWFTLGILEVLKGWDQ
Q80YT9	FGSSRSSEPANQSFESCAKINSHSFRNDEMAGRPSFPVPFF
Q2KIH8	HTPGQSRPSIRTAALSIAWPNALATRWTTHLGLAPAPTMSS
Q96B26	VTINEETALAEVNLKKKSYLNIRTHPVATSFAVFDDTLLIV
Q32PI9	SINIENMQFIHNGTYICDVKNPPDIVVQPGHIRLYVVEKEI
Q9JJJ3	FATAVVMALYATFGVSGGHINPAVSFAMCTFGRMEWFKFPF
Q9CRD2	MERYDDAIQLYDRILQEDPTNTAARKRKIAIRKAQGKTVEA
Q08708	NGRVSIRDSPANLSFTVTLENLTEEDAGTYWCGVDTPWLRD
Q9JIK9	VRPRLIAELARRVRALREQRNQPRDSQLYALDYETLTRPHS
Q9DAP7	RENPPPKPDFSQLQRNILASNPRVTRFHINWDNNPDSLEAI
Q9CQ52	ADNGTWQVHGVTSFVSSLGCNTLRKPTVFTRVSAFIDWIEE
Q9UF11	LGYYHDETAQDEEDRVLIHFNVRDIKIGPECHDVQPPEGRS
P82347	PTRSLVMEAPKGVEINAEAGNMEAICRSELRLESKDGEIKL
Q9BXU9	ELKHILYHAFRDHLTMKDIENIIINEEESLNETSGNCQTEF
Q8BPA8	EPVPSGAGSLGSELIKESNANPIFMRKDTKTSFQWRIRNLP
Q8BZI6	VSVQDIQVHLAQGHVAIVLVNSGVLHCDLCSSPVKYCCFTP
O94903	TKPADMVIEAYGHGQRTFGENYVQELLEKASNPKILSLCPE
Q10738	YLRKFYPHDSKTKKVNSLVDNLKEMQKFFGLPMTGKLSPYI
Q05718	SVLQQLQTEVFRGAHTLYVPNCDHRGFYRKRQCRSSQGQRR
P14847	DFVLSPEQISTVYVGGTLSPNVLNWRALNYKAQGDVFIKPQ
Q3ZCA1	IIHLCQGEIRQDSLYEAGAANVGRVVNSWYRYRPLVAELVV
Q14331	VKLSDSRIALKSGYGKYLGINSDGLVVGRSDAIGPREQWEP
Q17QF2	MCKFIPEHTRTLGNEFRCFVNYNSGLDCNLSVQLPVQEGPK
O75841	KQMLERYQNNSPPNNDDQWKNNGVTKTWDRLMLQDNCCGVN
Q8N1A0	IQTEKVDEVIKEWEGSFFKDNPRLRKKSVSLRFDLHLAATD

Q9H1U9	PHITNVGEMKHYLCGCCAAFNNVAITFPIQKVLFRQQLYGI
P39687	EPLKKLENLKSLDLFNCEVTNLNDYRENVFKLLPQLTYLDG
O55126	KNQLLLEFSFWNEPVPRPGPNIYELRSYQLRPGTMIEWGNY
O08604	LWYEAKCLVDEILILHLSNINKTMTSGDPGETANATEVGEC
O35906	FQFSSKHKELLARRWGQQKGNRKRMTYQKLARALRNYAKTG
P62826	KSIVFHRKKNLQYYDISAKSNYNFEKPFLWLARKLIGDPNL
Q91ZD6	AFHTVRYDFKPASIDTSCEGNLEVGKGEQVTITLPNIEGST
P47876	LAAAQQKAGDEIYKFYLPNCNKNGFYHSKQCETSLDGEAGL
Q70YC4	IQGFSQIWVLFPFCGGTFHHNEKDVLGLQDFERESVSTSQS
P26452	NLKRTWEKLLLAARAIVAIENPADVSVISSRNTGQRAVLKF
A6H773	TYKAITYSVVLSEKSTVFHQNDVKIPNSTHVFTTFYAKTKS
A2RRY8	SKVSLPEIPKEKYPEEFSLLNSQTEDGQRPEWTFYPRFSSN
Q2YDG7	LTIGVIICIFVIFVLIFIIINWTAVKDFWAKASTTEIQSEL
P35270	AGSLGDVSKGFVDLSDSTQVNNYWALNLTSMLCLTSSVLKA
Q9NYK6	LHTKSLRGHKDCFEKYHLIANQGCPRSKLSKSTYEEVKTIL
Q2KIJ1	QENQTYTLRSFQMARVIFNQNGCSILQKHSRDAHFYPVREG
Q8NBA8	NRCLSTLECAAVALSILEKNNYIQETLLRPLQALCSFQLQH
Q9BUA6	RINVKNEELEAMVKEAPGPINFTVFLTMFGEKLKGTDPEET
Q3ZBD9	PITTHRTTATPKAGPGVVRKNPGVGNGDDEAAELMQQVNVL
P28676	ETCRIMIAMLDRDHTGKMGFNAFKELWAALNAWKENFMTVD
P79103	TDITYPAGFMDVISIDKTGENFRLIYDTKGRFAVHRITPEE
Q9BQ65	PEDDSTKHGGRVRTFPHERGNWATHVYVPYEAKEEFLDLLD
Q9UNK0	RQNLLDDLVTRERLLLASFKNEGAEPDLIRSSLMSEEAKRG
Q9Y3B3	HKTVYFDFQVGEDPPLFPSENRVSALTQMESACVSIHEALK
P07306	LSCQMAALQGNGSERTCCPVNWVEHERSCYWFSRSGKAWAD
P33681	SGDMNIWPEYKNRTIFDITNNLSIVILALRPSDEGTYECVV
Q8N4T8	GLVGFSRALAKEVARKKIRVNVVAPGFVHTDMTKDLKEEHL
Q8R3G9	ILGAAFKPEYNRILNETLYENAKLLSDNTDEAKDFQKAMIV
Q9D8X2	EQRAEPVEKAKSHLELPLEENLNRRLQEEGSVEARTVEDAI
P00760	DKIVGGYTCGANTVPYQVSLNSGYHFCGGSLINSQWVVSAA
P68252	REKIEKELEAVCQDVLSLLDNYLIKNCSETQIESKVFYLKM
Q3ZC21	FICNIFDYLRVNNMPMMALVNPVYDCLFRLAQPDSLSKEEE
Q9CXK4	MQRTRSVDNIQFLPFLTTDVNNLSWLSYGVLKGDGTLIIVN
Q96L14	RQSIDKTAGKIRILFKDKDRNWDDIESKLRAESEVPIVKTS
Q99N05	FRQFRSQPAIASLDVLMTILNMLEFCIAVSVSAFGCKASCC
Q923D3	TTSPATSLSESLSASVTSSHNSTVANIQPTEAPMAPASPTE
Q8QZW2	LTDPDEVARRWGERKSKPNMNYDKLSRALRYYYDKNIMSKV
P18351	QQKNLAELKRSFLDPALKQINEKTPLLAKYSIDDSGKFLFS
Q9NPJ6	PRRPYPTDLEMRSGLLGQMNNPSTNGVNGHLPGDALAAGRL
Q9Y2B4	VQGRQPLTEGPRVIFIKPYRNRTPMGHMKQLDVADQWIWFE
Q8VBV3	AGSVERVNKLICVKALKTRYNGEVGDIVVGRITEVQQKRWK

Q9QWG7	VWRKDLKMIHGYPMIYAFALNWERIEEFQSTPGDIVITTYP
P46004	IAIALSVFTFAHAQSFENVENNAKVFSLHLGATRMIYKPNS
Q9NYN1	ELLALHAKETQRSIPALLLGNKLDMAQYRQVTKAEGVALAG
Q8CDN6	VPLRYVKFQNVNSVTLFVQSNQGEEETTRISYFTFIGTPVQ
P61289	KCNTVKMWVQLLIPRIEDGNNFGVSIQEETVAELRTVESEA
P23888	KISWPVGLAGGFQGSLTGRENVKFVARLYAKRDELNERVDF
Q9Y287	VIPLNTSIVMPPRNLLELLINIKAGTYLPQSYLIHEHMVIT
Q00535	LKGLGFCHSRNVLHRDLKPQNLLINRNGELKLADFGLARAF
P12979	LEFGPNPGDHLLAADPTDAHNLHSLTSIVDSITVEDMSVAF
Q96MP8	TQAGHALPLLPQEFPEVVPLNIGGAHFTTRLSTLRCYEDTM
P97361	SLLQLAVKLNITAEVLAVKDNQGRIHLVLGDCTHSPGSLKI
P33783	KKVTLFLFVVSLLPSTVLAWNTPGEDFSGELKLEGAVTSTR
Q9NP98	LGIDLLAYGAKAELPKYKSFNRTAMPYGGYEKASKRMTFQM
P10598	ILPKHKQMPLLNSETVLRLINSQIPSLASLANLHLPQSLVQ
P62754	PRVLQHKRRRIALKKQRTKKNKEEAAEYAKLLAKRMKEAKE
P35293	WLNELETYCTRNDIVNMLVGNKIDKENREVDRNEGLKFARK
Q61955	SPQENFPNTLNCAEVKIYSQNKCERAYPGKITEGMVCAGSS
Q9NS61	FEDFVAGLSVILRGTVDDRLNWAFNLYDLNKDGCITKEEML
P61982	MAAAMKNVTELNEPLSNEERNLLSVAYKNVVGARRSSWRVI
Q96FZ7	EFTQIEMKVMEGLQFGNECLNKMHQVMSIEEVERILDETQE
P56402	ILHEITPVEIRGDLAVNALHNNATAGQAVTVELFLTMQLVL
Q96G21	SRLKMFAKELKLVFPGAQRMNRGRHEVGALVRACKANGVTD
O15217	KIPNILSAFPFLQEYTVKLSNIPTIKRFLEPGSKKKPPPDE
Q9D9P2	SHLKPLEQKDKMGGKRFVPWNCHTTRERTSQDVVTEIPQEK
E9PI22	KPSISGAPHLNSYQSLELPQNQQDSGTEELMIVLEQGTEVR
Q60682	ESYWIGLSYDKKKKEWAWIHNGQSKLDMKIKKMNFTSRGCV
Q9X2V8	KVFYYSWNIEKKGMAWIFISNKKENRLYSLNEEHLIRKEIS
Q9D9M4	AAASDGILQPGDVLISVGHANVLGYTLREFLKLLQNITIGT
Q8K3I6	LLSEVQGYVHSFAESRLPGVNLDLLPLGYHLPNVSLTFQAW
Q9NVS9	KDGFRFFTNFESRKGKELDSNPFASLVFYWEPLNRQVRVEG
Q5E9E3	SRDNVVVFGKVITNQENVYQNNTGRFRCSVPGYYYFTFQVV
Q9UBT3	QLDEQDGTHAEGTTGHPVQENQPKRKPSIKKSQGRKGQEGE
P07478	ISLPTAPPAAGTESLISGWGNTLSSGADYPDELQCLDAPVL
O70552	SGTSSDEESCSREAQIIPKVNNPKSVYQVENFKQSLQPWFC
Q6GV28	TSSCTCLNIHKSDNECKESENSIEDISLPECTAMPRSIVRA
P27814	YNSFWIGLRFTLPDMNWKWINGTTFNSDVLKITGVTENGSC
Q08AT1	GKSALTVKFLTKRFISEYDPNLEDTYSSEETVDHQPVHLRV
Q9HBJ8	FKVRLSIRTALGDKAYAWDTNEEYLFKAMVAFSMRKVPNRE
Q9Y651	RPMNAFMVWSRAQRRKMAQENPKMHNSEISKRLGAEWKLLT
Q9CWG1	WMASSVSSSSFTASTLPDITNEDFIKECVQVHNQLRSKVSP
Q8IUX1	PMVIEIIEKNFDYLRKEMTQNIYQMATFGTTAGFSGIFSNF

Q3SZJ9	LSYIAKIKLPKKRGTFIEFRNGMLNVSPIGRSCSQEERIEF
P61247	VAFRKFKLITEDVQGKNCLTNFHGMDLTRDKMCSMVKKWQT
F8VTS6	PQLHRVGIFLDVGMRSIAFYNVSDGCHINTFIEIPVCEPWR
P53519	PASVPSPVFNNIVEKLKWHKNGKYLVLKNNTPYYISFSEVF
P62821	YYRGAHGIIVVYDVTDQESFNNVKQWLQEIDRYASENVNKL
P51908	HPYVTLFIYIARLYHHTDQRNRQGLRDLISSGVTIQIMTEQ
P46065	TECPSGQLTLYEFRQFFGLKNLSPWASQYVEQMFETFDFNK
Q5SWZ9	IVDKKVLITGSLNWTTQAIQNNRENVLIMEDTEYVRLFLEE
Q9TR36	IETWHKPDLGTLENVHGLDPNTWKTVEIVHIDIADRSQVEP
Q9NZH4	KQKQPSFSAKKMTEKTVKAKNSVPASDDGYPEIEKLFPFNP
Q3LI81	SCQPASLKGNSCPPKTSKSKNFETLERASSQCQCQSQNPES
Q9D711	YLLEGGSMAHEDFCGHVGKMNPGDLQWMTAGRGILHAEMPC
Q00188	YKALNVRRLNIMAGDEINSRNFDTLVELIAPTKDDVVIDNG
Q9UEE9	TSEEDEDYVPSGGEYSEDDVNELVKEDEVDGEEQTQKTQGK
Q9D309	EVLIGEKTGNVARGINIAVVNYETGKVIATKYFDMYEGDNS
P09488	LPEKLKLYSEFLGKRPWFAGNKITFVDFLVYDVLDLHRIFE
P04632	VMDSDTTGKLGFEEFKYLWNNIKRWQAIYKQFDTDRSGTIC
Q9TT89	RIEIAHALCLTERQIKIWFQNRRMKWKKENKTSGPGTTGQD
Q9D902	KKKAKVEHGGSSGSKQNSDHNNGSFNLKALSGSSGYKFGVL
P61313	SLQSVAEERAGRHCGALRVLNSYWVGEDSTYKFFEVILIDP
O94760	MQQMSDHRYDKLTVPDDIAANCIYLNIPNKGHVLLHRTPEE
Q9Y287	LQPDDVYYCGIKYIKDDVILNEPSADAPAALYQTIEENIKI
P05833	PISFERLRERLSLISQVKEQNRIIKKAITKLIDIGNLDASM
P24668	GKEQRRAVVMISCNRHTLAANFNPVSEERGKVQDCFYLFEM
Q8C552	QEGKIEAISDSDGVNYPWYGNTTETCTIVGPTKRDSKFIIS
P15692	MRCGGCCNDEGLECVPTEESNITMQIMRIKPHQGQHIGEMS
Q2KI19	KNRQKSIKEEEQERRDMGLKNALGCENKGFALLQKMGYKSG
P29459	LCLSSIYEDLKMYQVEFKTMNAKLLMDPKRQIFLDQNMLAV
Q8N9P6	PGPICSANICLSGVRFLTCLNRVREHVVGPSPSPAAPICFF
Q9DAS2	LSGGCFPTIFSPNSPLVLTDNSQNWDRWLHQPTYSRSNQDN
Q9QYB1	ADCNLLPKLHIVKVVAKKYRNFDIPKGMTGIWRYLTNAYSR
Q8BJG4	AKLSAPRYMALLMDWIEGLINDEDVFPTRVGVPFPKNFQQV
Q9UBT3	AVLLGLSWLCSPLGALVLDFNNIRSSADLHGARKGSQCLSD
Q96D70	GIFAEACNNATYVEVWNDFMNRSGEEQERVLRYLEDEGRSK
Q91X52	GVEPVCVDLADWEATEQALSNVGPVDLLVNNAAVALLQPFL
P31041	NCDGDFDNETVTFRLWNLHVNHTDIYFCKIEFMYPPPYLDN
P47753	KEFIKIIESAENEYQTAISENYQTMSDTTFKALRRQLPVTR
Q9WTP9	TPGLPGLPASHRGTSLVDPRNSSPRLNPMPSASASSPLPPP
P53347	ANLTGNTESLLEPYIRLQNLNTPDLRAACTQHSVAFPSEDT
O55135	EVFRQTVADQVLVGSYCVFSNQGGLVHPKTSIEDQDELSSL
Q06599	LRSSSQASSNKPVAHVVADINSPGQLRWWDSYANALMANGV

O54831	MEYLVKALTCCHNYSIKTPENLDEAQQIPFNEFPKLILSRM
P00757	NWVLTAAHCYNDKYQVWLGKNNFLEDEPSDQHRLVSKAIPH
Q9D2R4	KEQDYQRDREENFRITSEDTNRNVLWWAFAQILIFISVGIF
Q8HZJ5	QSFLNVRNWMSQLQANAYCENPDIVLIGNKADLPDQREVNE
P10833	LDTAGQEEFGAMREQYMRAGNGFLLVFAINDRQSFNEVGKL
Q9CZ04	ATSQDPEQHLTELREPASGTNQRQPSKKASKGKGLRGSAKI
Q9HB71	RKRVRDALTAEKSKIETEIKNKMQQKSQKKAELLDNEKPAA
Q8VDP2	MRPRDRSRVIDAAKHAHKFCNTEDEETTYLRRPEGIERQYR
Q9QZE7	LLGVADLTGELMRMCINSVGNGDIDTPFEVSQFLRQVYDGF
O95073	EKLDLLKLVKPYVKILEEHTNKHSVIVEKNRCWDIIAVNYN
Q8BGB7	EGTTTPIAFVKDVLFPYIKENVKEYLQTHWEEEECQQDVSL
P24158	HNVRTQEPTQQHFSVAQVFLNNYDAENKLNDVLLIQLSSPA
O54830	TRFLALCKLSYCLHVDIHTANFYLQFLRCVALVNSDSCLSS
Q8N2C3	HLPFLDNILEPPVKTQNLQLNKEEDLVITNTCLDRELIPSL
P21912	TLTFRRSCREGICGSCAMNINGGNTLACTRRIDTNLNKVSK
P20718	QFIPVKRPIPHPAYNPKNFSNDIMLLQLERKAKWTTAVRPL
Q08DY6	STKGPIDVYLCEVEQGSHSSNKTSDNVGTSSSKSKPLEHPQ
Q9UI43	VKAAKVESYRCRSAFKLLEVNERHQILRPGLRVLDCGAAPG
P51858	EKFGKPNKRKGFSEGLWEIENNPTVKASGYQSSQKKSCVEE
A6NJV1	LMERASTRDRWLHKPSYTRFNLDSHRSTELTNFYQMVQQHR
A3KN05	ELVAKETLHVLKEKVTSLPDNHKNALAADIDEIVYTSTGDI
Q9QZM3	LRLTQNYEAYSHLLCYLRGLNRQAATAELRRSLAHFCTSLQ
Q8IVV7	AFYQYAKSFNSDDFDYEELKNGDYVFMRWKEQFLVPDHTIK
O55233	RTVPFNQTIAHEDCQKVVVQNNLCFGKCSSIRFPGEGADAH
Q9ZIS7	IIPENVKAEIKASMEKLHALNMLSGDPHRGNFIVSKDGVRI
Q1WG82	LGSCWELMVIGMSDHLSMARNPRGTQCPNLEISSATSPASL
Q61581	KVKRDHSGVQRTELLPGDRENLAIQTRGGPEKHEVTGWVLV
Q3TR08	LLMWAAVIGLFCRQYDIIKDNDSNNNPKEKGKGPEQSPQGR
Q3TTJ4	TTSSEEQDEQSELSLLQKDENKLSEMWINHLKSKEIHSERS
Q3SX00	DPNIRDSRGRTGLHLAAARGNVDICQLLHKFGADLLATDYQ
P61026	TNGKSFENISKWLRNIDEHANEDVERMLLGNKCDMDDKRVV
P21583	CIYLQLLLFNPLVKTEGICRNRVTNNVKDVTKLVANLPKDY
P34741	KEKVHLSDSERKMDPAEEDTNVYTEKHSDSLFKRTEVLAAV
Q8VE10	RLGDPLEAFPVFKKYDRNGLNVSIECKRVSGLEPATVDWAF
Q8N0W5	VSHFTMISRTPCPQDKSETINPKTCSPKEYLETFIFPVLLP
P00642	NIRNGLLVGKRGDQDLMAAGNAIERSHKNISEIANFMLSES
Q2HJF2	LTAVAAHGRALQTLHVDFCRNVSAAGLRRLRAACPRLTLRA
A7YY49	SFVFLNLLGQLTGCILVLSRNFVQYACFGLFGIIALQTIAY
Q64152	PSLPLGGSRGRESQMKETIMNQEKLAKLQAQVRIGGKGTAR
Q96BT1	EESSSDSDMGFHESQQNQKSNLKTKVKTAFGRMLSYKYRSK
A6QLP7	FEYSFIPAEPMGGRPFGLVINLNYKDLNGNVFQDAVFNQTV

Q6GTX8	CRGPVGVQTFRLERESRSTYNDTEDVSQASPSESEARFRID
Q9D701	SNAAQNFTAPRKVEDRHAPANFDRNGYYLTLRANRVHYKGG
Q9CQT6	PCVKAFQERTQRYKEDQQGLNCSVANTPPLKPICSEDTVLW
P40313	SWGCGIPAIKPALSFSQRIVNGENAVLGSWPWQVSLQDSSG
Q8VI63	QYSTISEFCTGETCQTMAVCNTQYYWYDERGKKVKCTAPQY
Q9DAE8	FGILLGYPVSYTFDLNREDDNCLTMTPLRVFTARISWLPGQ
Q5EBN2	ASCPICLDYLKDPVTISCGHNFCLSCIIMSWKDLHDSFPCP
P12544	SDTLREVNITIIDRKVCNDRNHYNFNPVIGMNMVCAGSLRG
Q99LX5	QDQFNWEDVKTDKQRENYLGNSLMAPVGRWQKGRDLTWYAK
Q01105	EDIKSGYRIDFYFDENPYFENKVLSKEFHLNESGDPSSKST
Q2TBK2	KAGEATVKFLRSCHLEVGMKNNVKWELNPEIVARHFLRNLG
Q9H741	EYCVSCCLQPNKQLLLERFLNRAAVAFQNLFMAVEDHFELC
Q9JIW9	REQILRVKSEEDKIPLLVVGNKSDLEERRQVPVDEARGKAE
Q920C4	ILTLLELSVTISISAMWCLGNVCGLREAITSPPNSVESGIL
Q8BVN0	FEEQMIKYNAYYVKIKAYKDNLGEIKSQCPFMTELYEKRDL
P01881	STHPEMSSWLLCEVSGFFPENIHLMWLGVHSKMKSTNFVTA
Q2HJH7	VVCREASHAGSWYTASGPQLNAQLEGWLSQVQSTKRPARAI
Q9CRD0	GELRRSSPPGHYTQKPKFDSNVSGQSSFGTSPAADNIEKEA
Q9CPV4	SKTMVGFGPEDDHFVAELTYNYGIGDYKLGNDFMGITLASS
A6NFK2	RDFVRKILQKEEEAEEESLMNKEESYGGRDQHDRPLVEAES
O43657	VFLVELVAAIVGFVFRHEIKNSFKNNYEKALKQYNSTGDYR
P35846	YFPTSAALCEEIWSHSYKLSNYSRGSGRCIQMWFDPAQGNP
Q15040	KGYEAVWWDKRRDVGVIALTNVMGFIMNLPSSLCWGPLKLP
A6QNY1	VPNGSPSPCDIMDDCPDHNKNLLNCGPQSCPEGELCCPLDG
O95992	HLLHHKVPWLYRTFHKVHHQNSSSFALATQYMSVWELFSLG
P43431	IILDKGMLVAIDELMQSLNHNGETLRQKPPVGEADPYRVKM
P11911	ILLFCAVVPGTLLLFRKRWQNEKFGVDMPDDYEDENLYEGL
Q96KX2	FKYDLLQNQLKDIQSHGIIQNEAEYLRVVLLCALKLYVNDH
P56915	CRRKRRHRTIFTDEQLEALENLFQETKYPDVGTREQLARKV
Q46999	AISNTSPNLFTVPGDRIIAVNSLDGALTNNEQTASGGVVVA
Q9CRA5	NNIKQRLIKKVQEAVLDKWVNDPHRMDKRLLALIYLAHASD
Q9QXP7	FFSLNVHTWNQKETYLHIMKNEEEVVILYAQVSDRSIMQSQ
O15520	NSSSSSFSSPSSAGRHVRSYNHLQGDVRWRKLFSFTKYFLK
Q9BUT1	YLMIKAFLPKMLAQKSGNIINMSSVASSVKGVVNRCVYSTT
A1YIY0	CESMAEAQELFPNPELTEFTNSETMDVILKCTIKVDPKNPT
Q9NQR4	FNLTTGPAHWELLQRSRAVDNQVYVATASPARDDKASYVAW
Q8TBG9	MAIHSPVMSSLNTSVVFGFLNFILWAGNIWFVFKETGWHSS
Q96BX8	PGEDLNDWVAVHVVDFFNRVNLIYGTISDGCTEQSCPVMSG
Q3T0U1	SILKMTKKGKTLMMFVTVSGNPTEKETEEITSLWQGSLFNA
Q5RKV6	TRLEEERAAAGLTVALMPVLNQVAGLLGSGEGGLTESWAEA
Q8WVN6	GARDSHAGLYMWHLVGHQRNNRQVTLEVSGAEPQSAPDTGF

O89116	DANLGKSSRILTGMLRRIIQNRILLVILGIIVVIAILTAIA
Q9D6J6	VLPVLDLAQRQNGWLPISAMNKVAEVLQVPPMRVYEVATFY
O09164	LGPGSRLEAYFSLEGFPAEQNASNRAIHVHEFGDLSQGCDS
P27348	LACTLAKTAFDEAIAELDTLNEDSYKDSTLIMQLLRDNLTL
P68510	AEQAERYDDMASAMKAVTELNEPLSNEDRNLLSVAYKNVVG
Q9BTP7	ADLVAGNGYRKRLVRVRNSNNLKGIVVVEKTRMSEQYFPAL
Q7L0Q8	LVVSYTTNGYPTEYIPTAFDNFSAVVSVDGRPVRLQLCDTA
Q96CX3	KQSNLTQHLRIHTGEKPYKCNECEKAFQTKAILVQHLRIHT
Q64329	ILCFLLLVTVAVLAVKIFQYNQHKQEINETLNHHHNCSNMQ
Q96PB8	IQSVHKNAFNNLKARARIANNPWHCDCTLQQVLRSMASNHE
Q9NYK6	NWIVQYAQNKDLDSDSECSKNPQHHLFNFRHKPEEKLLPQF
Q5NE16	MIEQHNQEYREGKHSFTMAMNAFGEMTSEEFRQVVNGFQNQ
P04975	ASKVMEQEWREKAKKDLEEWNQRQSEQVEKNKINNRIADKA
Q9D0L6	YMCKSELSACFSRLLDPQNTNSPLTHGCLDSLASTADICRA
Q91VR2	ETMSIYDDIDADVLQNYQEYNLANLIYYSLKESTTSEQSAR
Q5BIS9	KDTGISCDPALLPEPNHVMLNHLYALSIKDGVMVLSATHRY
A6NN92	LFFGSIRIFFLGVLGFAVYGNEALHFICDPDKREVNLFCYN
Q99990	LTPSSQSEGVMLKNDDSMSPNQWRYSSPWTKPQPEVPVTNR
O00322	AAAAEAEKGSPVVVGLLVVGNIIILLSGLSLFAETIWVTAD
Q2T9X7	AAPEPEEQEERKPSATQQKKNTKLSSKTTAKLSTSAKRIQK
Q6ZUV0	VTMKVMDEVAGILAARHCKTNLVTASMEAINFDNKIRKGCI
Q96DN0	QKFPGVSFGISTDSEVLTHYNITGNTICLFRLVDNEQLNLE
Q9D270	HCVRRMDHHCPWINNCVGEDNHWLFLQLCFYTELLTCYALM
Q8WVF5	EKEKEYEGKHNSLEDTDQGKNCKSTLMTLNVGGYLYITQKQ
Q9UKD2	KRDKKVSLTKTAKKGLELKQNLIEELRKCVDTYKYLFIFSV
Q99811	NLSEARVQVWFQNRRAKFRRNERAMLASRSASLLKSYSQEA
Q32L31	GKMSAYAFFVQTCREEHKKKNPEVPVNFAEFSKKCSERWKT
Q96LD8	LLVYLQDKNSFFHYDSHSRSNSVHAKQVAEKLEAFLGRKGD
Q7Z692	TWMATTEKPELGPAHDAGDNNIYEVMPSPVLLVSPISDTRS
Q9CY24	AIFLILVSACILRFGTNSFCNSIISLNLTISCSEAQKTSWT
P04085	CKTRTVIYEIPRSQVDPTSANFLIWPPCVEVKRCTGCCNTS
Q9UBV8	PPSSYGAQQPGLYGQGGAPPNVDPEAYSWFQSVDSDHSGYI
P14206	ADHQPLTEASYVNLPTIALCNTDSPLRYVDIAIPCNNKGAH
Q17QD9	LIYSQFFPQGDATTYAHFLFNAFDADGNGAIRFEDFVVGLS
P01903	TKRSNYTPITNVPPEVTVLTNSPVELREPNVLICFIDKFTP
Q15546	MNHRAPANGRYKPTCYEHAANCYTHAFLIVPAIVGSALLHR
P04095	LVNSSLLWKNVASFPMCAMRNGRCFMSFEDTFELAGSLSHN
A6NCD4	CWFGESGEIMSLPTPIKQSWNFRLNVRKEPLNPLLKTIYRK
Q9UK85	SHLQIDKMTDNKTGEVLISENVVASIQPAEGSFEGDLKVPR
Q96JQ5	RGSLGMNITSSVLAASGILINTFSLAFYSFHHPYCNYYGNS
Q32KP1	VIAVGVFLLLIAVAGLVGAVNHHQVLLFFYMIILGLVFIFQ

P19473	VKCRMIYFFHDPNFLVSIPVNPKEQMECRCENADEEVAMEE
Q8BS40	SSEDARTSTLCSEAYNATLANYHSWIVRQAVTVAFCALPSR
Q8BGR6	ESPENVVSTTGFSIKAVPFQNAVLNVKELGGADNIRKYWSR
O95760	DSSITGISPITEYLASLSTYNDQSITFALEDESYEIYVEDL
Q3UZZ6	KRDAIYKRVPFMELIIPGITNGVEMLNNMPSPRIVKTHLPV
Q3UPR9	SQSQDCGHSFVPAFITSSVFNKKRIIQAVSPQWSTHTKDAG
Q8JZV9	FLMIKAFLPKMLAQKSGNIINMSSVASSIKGVENRCVYSAT
Q9Y337	LLGVTEHVLANNDVSCDHPSNTVPSGSNQDLGAGAGEDARS
O35622	VSNCFLRIRSDGSVDCEEDQNERNLLEFRAVALKTIAIKDV
Q9NQX7	LVFASVYIYRYFFLAQLARDNFFRCGVLYEDSLSSQVRTQM
Q5Y7A7	RWFRNGQEEKTGVVSTGLIHNGDWTFQTLVMLETVPRSGEV
Q3TWL2	GKMHQHVVKCGVCNEATPIKNAPPGKKYVRCPCNCLLICKV
P04925	RENMYRYPNQVYYRPVDQYSNQNNFVHDCVNITIKQHTVTT
Q6ZWZ2	NPTQNVRTILLSVISLLNEPNTFSPANVDASVMFRKWRDSK
Q8K2H2	QLKQLTFKDSKIDSVAVNISNLVLENQPPRISKAQKRREKK
P04467	NELDALLKDLCEKNKQDLDINNIPTYKKSIMALSDGGKLYR
Q9WTY4	IYFTGCSMNPARSFGPAVVMNRFSPSHWVFWVGPIVGAVLA
O75828	PMPFDIKAEMTLKTNFFATRNMCNELLPIMKPHGRVVNISS
Q3ZBH3	HEGVTSAVDKLQQEFHCCGSNNSRDWQDSEWIHSGEAGGRV
Q9CXT7	NRAKPLPDVLEEEKIYAYPSNTASETGFRTVSSLEEIVEKQ
Q5UCC4	LSQRQLSEEERGRLRDVAALNGLYRVRIPRRPGALDGLEAG
P32243	LPGPGATLSPMGTNAVTSHLNQSPASLSTQGYGASSLGFNS
Q9D3G2	AVPKPEVQVFTAAAEETQPLNTCQVFLSCWAPNISDITYSW
Q01730	SLAHITQLVLSHNKLTTVPPNVAELKNLEVLNFFNNQIEEL
Q07424	TSPVFGSPDYSTLGPTSGASNGGSLPDAASESLVSLDSGTS
Q9D7G4	GVKTIVSKKINWIVQYAQNKNLDLESECSKTSQHPLLNFRH
P68402	KNGELENIKPKVIVVWVGTNNHENTAEEVAGGIEAIVQLIN
Q8BR90	KSNLLQKYLVDGINYLLQMLNYRCPVQLNEGVSFQDLDTAK
Q8VHZ7	AQRMNRGRHEVGALVRACKANGVTDLLVVHEHRGTPVGLIV
P50592	LACFSKTDEDFWDSTDGEILNRPCLQVKRQLYQLIEEVTLR
Q9JJ69	ALREEAPREHVESFFQKMDRNKDGVVTIEEFIESCQQDENI
Q505H4	AVPECVNPIYEPEQCCPVCKNGPNCFAGTTIIPAGIEVKVD
Q16594	MPTSQSPAVKASIPATSAVQNVLINPSLIGSKNILITTNMM
P11032	KGDDVKPGTRCRVAGWGRFGNKSAPSETLREVNITVIDRKI
P10938	GLAAVSSAYQRFEPRAYLRNNYAPPRGDLSCPDGVGPWKLR
Q9CQJ2	AEVDLPKLDGAQGLALEIGENRLVIGGPQQLYHLDATVPLR
Q1RMU1	GFYLHLGKCLDNCPEGLEANNHTMECVSIVHCEASEWSPWS
Q91WN1	EEAKEAELSRKELGLEEGVDNLKALIQSRQKDRQKEMDSFL
Q32L00	YSRITMEPNPHWHPVLLMLKNQIEENTGHSFNSLLCNLYRN
A1YIY0	LLASVKAEWSTKNYSCEAKNNISREISELKKFPLVVSGTAW
Q96L21	FKFPGRQKIHISKKWGFTKFNADEFEDMVAKKCLIPDGCGV

Q9CR29	DDAGASTANVSSDRTLFRNTNVEDVLNARKLERDSLRDESQ
Q3TYX2	LKPGVAYVLCVVAANDAGESNVPGAEVEGPENWTGPSFGPC
O88992	AISTATYTTVPRVAFYAGLKNPHEGYEVLKFDDVVTNLGNN
P48739	SLVFHEKAWNAYPYCRTIVTNEYMKDDFFIKIETWHKPDLG
Q8JZU6	SRSEVVLTFFERSPLDQVLKNDNVHKIQPSFQSPVKISEIM
Q9NS61	QIYSQFFPQGDSSTYATFLFNAFDTNHDGSVSFEDFVAGLS
Q7RTY3	QTPVTFSEYVQPICLPEPNFNLKVGTQCWVTGWSQVKQRFS
P0DML3	SLLLIESWLEPVRFLRSMFANNLVYDTSDSDDYHLLKDLEE
Q99598	LLGVADLTGELMRMCINSVGNGDIDTPFEVSQFLRQVYDGF
Q9BQ13	TPLPQSPRPRRPTMSTVVELNVGGEFHTTTLGTLRKFPGSK
Q99N08	AVTVLALGIILASVPPVPYFNSVFSVLLKSGYPFIGALFFI
Q99PG4	ILKAKAIYEKFIQNDAPKEVNIDFHTKEVIAKSIAQPTLHS
P58340	NMRNYMQKLERNFGQLSVDPNGHSFCSSSVMTYSKIGDEPP
Q8N402	RVKREPYSTTMLQVTSLSPINEILRRYSLYTNQHWRHHGFW
Q5VTT2	PCQDDYSIVHRKCRSQFTDLNGSKRFGINTWHDESGIYANS
Q9CPS7	VHVHILGSFQNIKMARTAICNLILGNPPSKVYGNIRAVASR
Q96BD8	FITCDEFNGVPSYMKSRLTYNQINDVIKEINKAVISKYKIL
O08583	SQIDTQRRPAQSINRGGMTRNRGSGGFGGGGTRRGTRGGSR
P53994	TRRDTFNHLTTWLEDARQHSNSNMVIMLIGNKSDLESRREV
O88445	LRREVEIQAHLQHRNILRLYNYFYDDTRIYLILEYAPGGEL
Q58DI5	LGTLYCLSRSDSWAGREVLMNLLFALLLDLLLVSLIKGLVR
Q3MHR7	CFASVFEKYFQFQEEGKEGENRAVIHYRDDETMYVESKKDR
F1MM41	DAERAPGSGGSASDSTYAHGNGYQETGGGHHRDGMLYLGSR
Q9CPZ1	LPQAITSERLLCHQEGRTKSNTKQENEKKIQLPMYTSGKRQ
P52744	ITTSKIFQCNKYVKVMHKFSNSNRHKIRHTENKHFRCKECD
Q29461	GVPTYPPQLSRVVGGEDARPNSWPWQVSLQYSSSGQWRHTC
Q8N6M0	LLRRHRKEKKELQAKIQGMKNAVPKNDKKRRKQLTEDVAKL
Q9H3S4	ANRLYDITEGERESFLPEFINGDFDSIRPEVREYYATKGCE
O95452	MILVVAAQEVWGDEQEDFVCNTLQPGCKNVCYDHFFPVSHI
Q9D9C7	GASCAPESENPAFRTHHIPVNSKLQQPLYPKRKPLTSKENV
Q9H4A6	LMEEVLLLGLKDREGYTSFWNDCISSGLRGCMLIELALRGR
P26884	DSFLAEHKLLGNIKNVAKTANKDHLVTAYNHLFESKRFKGT
Q3ZBK8	YQADRLQSDFAAFLVGPLQRNSLCNLLSFTYKVKPEGQSFP
P41272	SGFLIRNCTVTANAECSCSKNWQCRDQECTECDPPLNPALT
Q91VF2	ECYDLVSTMDITDCFIDGNENGDLLWDFLTETCNFSKTAPL
Q9ER00	GTKQDSSKLQENLQQLQHSTNQLAKETNELLKELGSLPLPL
Q9BSB4	VEVMNRHEYLPKMPTQSEVDNVFDTGLRDVQPYLYKISFQI
Q15907	DEYDYLFKVVLIGDSGVGKSNLLSRFTRNEFNLESKSTIGV
Q6UXZ0	DQSVSVSVVLNVTFPPLLSGNDFQTVEEGSNVKLVCNVKAN
Q8VHX2	QDTGLPKAKECDTVNSNCPPNSDDQPQGEENDFPDSTKDPL
Q60660	YYFIMDDKKWNGCKQICQDYNLTLLKTNDEDELKFLKSQLQ

Q96Q83	RLTAWYGELPYTYSRITMEPNPHWHPVLRTLKNRIEENTGH
Q9H8W3	SNQLATRELGVKIAKAVACHNFVKAKKEVENSQAARKKKKL
Q9HD33	GQEKVKSGAAWTCQQLRNKSNEDLHKLWYVLLKERNMLLTL
O95816	NSQDMRQISDGEREELNLTANRLMGRTLTVEVSVETIRNPQ
P50053	ATDFEKVDLTQFKWIHIEGRNASEQVKMLQRIDAHNTRQPP
Q01892	LELAPSLEAPGPGLPAYPTENFASQTLVPPAYAPYPSPVLS
Q9CQG0	FGVFYEGPEVDHKQSQRKQLNDTLDAIKDSTQRVENQVFHM
P47757	PPLEDGAMPSARLRKLEVEANNAFDQYRDLYFEGGVSSVYL
Q02844	ASETFPSGTLCWVTGWGNIDNGVNLPPPFPLKEVQVPIIEN
Q9CQ52	GDIQSGCNGDSGGPLNCPADNGTWQVHGVTSFVSSLGCNTL
Q9CQY8	LMAIPATTMSLAARKRACCNNKTGMFLSSLFSVITVVGAVY
O14595	RICVVIDLDETLVHSSFKPINNADFIVPIEIEGTTHQVYVL
P97805	EIIMSPVRNNVGRGLNVALVNGSTGQVMKKDSFDMYSGDPQ
Q9CZ04	ANQHKEQQLGLKQQIESEVANLKKTIKVTTAAAAAATSQDP
O75352	LQSVMLELVALTGTMVYSITNNFPFSSWGEALFLMLQTITI
Q0VCS6	NRKLAETLHHVAEKLCRELYNKTGEHRCSPCPEKWKWHGDK
Q64374	ALNWENQSVFVLAMVDEDKKNNRFNDGKVDPAGRYFAGTMA
O75940	LASYKAQLQQVEAALSGNGENEDLLKLKKDLQEVIELTKDL
O18870	YGNREEQNLSDLLSPTSEVANIEQNAQENENESQISTDESE
P20774	EKSLQLQKDEAITPLPPKKENDEMPTCLLCVCLSGSVYCEE
P13949	CALDRRERPLNSQSVNKYILNVQNIYRNSPVPVCVRNKNRK
Q96B21	TGILAEQFVPDGPHLHLYHENHWIKLMNWQHSTMYLFFAVS
Q9D7W5	ARIGADAAQKQIQSLNKMCSNLLEKISKEERESESGGLRPN
Q8IZ81	HVVQSEVDKYVDDIMKEKNINPEKDASFKICMKMCLLQITG
P57052	YRNEEMLVGRSSFPMQYFPINNTSLPQEYFLFQKMQWHVYN
Q719I0	VKESGVKHKGLIEIPNLSEENEVDDTEVSLSKKKGDGVILK
O35403	GHRNRTENIETIDRAPFFEYNIHKLDYAKMPSPRIFSSHIP
Q8SQH5	CWRKIARDEGAKAFFKGAWSNVLRGMGGAFVLVLYDEIKKF
Q6S5G4	VVSFVLDIIELFYVLFKAIKNRMRKAEDEVYCDELPCPSHV
P29965	IEDERNLHEDFVFMKTIQRCNTGERSLSLLNCEEIKSQFEG
A6PWV3	QGNLLEIRQNLEKKLTQIIKNFSEVACSEDCIVIEGPVLDC
Q9BZ81	GPRAYTETSKMKVLEYLAKVNDIAPGAFSSQYEEALQDEEE
Q6P6M5	IRTLGYCCQLIGGVLVEQCPNRSEVGRRLLVVSAQFNHCRT
Q1RMT9	ILVSNMLLAEAYGSGGCFWDNGHLYRADQPSPAPGHSCLNW
Q91YN9	AATAVEQEKEILLEMIHSIQNSQDMRQISDGEREELNLTAN
Q9CQX5	IGTDFWYEYRSPIQENSSDSNKIAWEDFLGDEADEKTYNDV
Q9WVS0	GCILIIWFSKKKYGSSVHDPNSEYMFMAAVNTNKKSRLAGV
Q8N131	SVASDSSNTTVTTMKPTAASNTTTPGMVSTNMTSTTLKSTP
O43752	VRDMKDQMSTSSVQALAERKNRQALLGDSGSQNWSTGTTDK
Q9H8W3	HSEPSSNETQWKELTQYFGVNDRFDPPVKRKKVEKSGLEKR
Q9JJT2	NYLDNVSARVAPWCGCAASGNRREECEAFRKLFTRNPCLDG

Q8N699	ILVFLSVFLLFLLFLVDIMANNTTSLGSPWPENFWEDLIMS
Q6V702	SAGKDLQDNFLTALAMREEDNRSGKLSSVIFIRDRNSHGQE
O88445	SDALTYCHEKKVIHRDIKPENLLLGLNGEVKISDFGWSVHT
Q9D7J7	RQQFTQLAGPDMEVGATDLMNILNKVLSKHKELKTEGFSLD
Q05066	RPMNAFIVWSRDQRRKMALENPRMRNSEISKQLGYQWKMLT
Q8R2R5	SGNALTHLGPLASLRQLAVLNVSNNRLTGLEPLAACENLQS
Q8CEZ0	KDETGAYLIDRDPTYFGPILNYLRHGKLIITKELGEEGVLE
Q9CQL0	VIYLEDTFTDLLQTLGHLCSNNSVILLACRIRYERDSNFLT
Q8N1B3	SSIYLAGKVEEQHLRTRDIINVSNRYFNPSGEPLELDSRFW
Q15006	MERYDDAIQLYDRILQEDPTNTAARKRKIAIRKAQGKNVEA
Q8CCA0	LIYRYAFDFAREKDQRSLDINTAKCMLGLLLGKIWPLFPVF
A2VE33	GEPCNQTSNTTASPHQVGSGNMECPACYGNNETSCNETRKC
Q8C996	FAFDAILDVLSSAIVLWRYSNAAAVHSANREYIACVILGVI
Q9BZX2	DSFYRVLTSEQKAKALKGQFNFDHPDAFDNELILKTLKEIT
P17433	GSDGESHSDHYWDFSAHHVHNNEFENFPENHFTELQSVQPP
Q60590	SQTIGDQCVYNSTHLGFQRENGTFSKYEGGVETFAHLIVLR
Q9JI59	GLLMLLLLHYLIVALDYHKANGFSASKDHRQEVTVIEFQEA
Q8BYP3	EQLRKLRAAQLEPITYTQGLNACEQMRGALYLECSAKFREN
Q8CC84	YFKIQATGEPSNGLNATVVPNGSMTLSPVPGQAPRLAIPVP
Q9BV99	LTHLGPLASLRQLAVLNVSNNRLTGLEPLATCENLQSLNAA
Q96LT6	FGILLGYPVPYTFHLNQGDDNCLALTPLRVFTARISWLLGQ
Q6PEB6	IYRIFSHAYFHHRQIFDEYENETFLCHRFTKFVMKYNLMSK
Q9QXV3	SQELGDEKIQIVSQMVELVENRSRQVDSHVELFEAHQDISD
Q9BXJ1	FNMFTGKFYCYVPGLYFFSLNVHTWNQKETYLHIMKNEEEV
Q3T127	NVYVKFRREEDAERAVVELNNRWFNGQAVHAELSPVTDFRE
Q91XQ6	YQIFDFALNTLVAITVLVYPNSIQEYIRQLPPSFPYRDDIM
Q6QUN5	TLAEGQTLKVSCPTNTNIYSNSQKAWQRLKDNGEVQTLAIT
O08709	KGRDLAILLGMLDPVEKDDNNMPVTARVVFIFGPDKKLKLS
Q86UV7	VIEALRLPGDPEPKVCVHHRNPLSLFCEKDQELICGLCGLL
P46777	GLTNYAAAYCTGLLLARRLLNRFGMDKIYEGQVEVTGDEYN
Q32LB6	SGSSLFKTQCVPVPPKRRQRNTIRKFVHIPKNTQATESSSD
A6NM15	PGNAKEEHLNMFIQNLLWEKNVRNKDNHCMEVIRLKGLVSI
Q96G30	VIFMFFVLTLLTKTGAPHQDNAESSEKRFRMNSFVSDFGRP
Q9D305	RRATRRWIKATCFVKSLEASNMLPKGISEQILPTALSNLPL
Q0P5E4	VWRLITNFLFFGPLGFSFFFNMLFVFRYCRMLEEGSFRGRT
O35047	MQKEIQELKKECAQYTERLKNIKAATNHVTPEEKEKVYRDR
Q6IRU2	AEERAEVSELKCGDLEEELKNVTNNLKSLEAASEKYSEKED
Q3T0I4	RGRAGSQGGRGGGAQAAARVNRGGGPIRNRPAIARGAAGGG
Q29RZ1	AEGVSAQSFLHCFTLASAAFNLQVATPGGKTMDFVDVNESN
Q9CPQ5	EIDKIVETTESMTESIQSLKNKIQILTSEVEEEEQEVKQVF
P08508	HNGRSIRSQVQASYTFKATVNDSGEYRCQMEQTRLSDPVDL

O08859	KECGGVFTDPKRIFKSPGFPNEYDDNQVCYWHIRLKYGQRI
Q3T144	RNSNFYSVAVTSLSSEVQYMNTVVGSYTTTNISLIPPRSEH
P30301	YYTGAGMNPARSFAPAILTGNFTNHWVYWVGPIIGGGLGSL
Q15116	CSFSNTSESFVLNWYRMSPSNQTDKLAAFPEDRSQPGQDCR
Q9JKK1	SWNAGVADRYGRLDRELQLANSHFIEEQQAQQQLIVEQQDE
Q9BPW5	LHQHVQQLHLGTRLPVVVVANKADLLHIKQVDPQLGLQLAS
P28068	LRNGLQNCATHTQPFWGSLTNRTRPPSVQVAKTTPFNTREP
P28067	LPHSWAVPEAPTPMWPDDLQNHTFLHTVYCQDGSPSVGLSE
Q6NTF7	FPKFADCWENFVDHEKPLSFNPYKMLEELDKNSRAIKRRLE
Q923S9	KLFLDLACRLISEARQNTLVNNVSSPLPGEGKSISYLTCCN
P30931	YTLYYWKASSTGKKKATTNTNGFLIDVDKGENYCFHVQAVI
Q08E08	DFQHQATIFLQVVDKLEEVDNTVTLIILGVVGGVIGLLIFI
Q3T123	QQVSALPPPPMQYIKEYTDENIQEGLAPKPPPPIKDSYMMF
O75871	RTINASYDSDQATGQLHVHQNNVPGLPVGAVAGIVTGVLVG
Q96CX3	HQRSHSGDKPFKCNECGKAFNQSACLMQHQRIHSGEKPYTC
Q46669	LGIRIGNDAFFTAHAIAMRNNDAPALVEEVYNFFRDSRDPV
Q9NQM4	KDSSTGCCSELVAKIKLPNTNPSDIQIDIQETILDLRTPQK
Q8N2A8	VDKRVLITGSLNWTTQAIQNNRENVLITEDDEYVRLFLEEF
Q61205	LGLLPRGQHPNPLREKNRQVNELVRAALAGYPRAHFLDADP
Q3T168	GSSKKVKTDTVLILCRKKAENTRWDYLTQVEKECKEKEKPS
Q60660	LRQEYQVMKNDSSLMEEMLRNKSSECKALNDSLHYLNREQN
Q3T123	KREEKLEDLKLLFVHVHHLINEYRPHQARETLRVMMEVQKR
Q86YB7	ALAGPDQGITEILMNRPSARNALGNVFVSELLETLAQLRED
Q96G21	EEAQRSAQERKERLRRALEENRLIPTELRREALALQGSLEF
P08311	RRENTQQHITARRAIRHPQYNQRTIQNDIMLLQLSRRVRRN
Q53H82	VTHWHRDHSGGIGDICKSINNDTTYCIKKLPRNPQREEIIG
Q8NDZ9	GPGRTPAEPAAAGPSRRELRNGSYLSTVPTARLWAADRAGG
Q8BGY7	HLITSGIWFGTFYYATIKGVNVIPFLEVIGLPDSIVDILKN
Q8BVP6	SPPGVSIASYSRVCRSYLCNNLTNLEPFVRLKASQPMSTLP
Q9QYB1	KGMTGIWRYLTNAYSRDEFTNTCPSDKEVEIAYSDVAKRLT
P23463	RIEVSHTLALTERQVKIWFQNRRMKWKKENNKDKFPASRPE
Q9D7N2	YGCRPSGCVTYGPQTIHIVSNSLRPLQPVCGGCQPSIPVFG
P14207	HTSHTCKSNWHRGWDWTSGVNKCPAGALCRTFESYFPTPAA
Q921Y0	AHIYHQHFDSVMQLQEEAHLNTSFKHFIFFVQEFNLIDRRE
Q9BRN9	TVRDHVHCLGNRTFPKMLYCNWTGGYKWSTALALSITLGGF
Q14681	SYNYGNEDQAEFLCVVSRELNNSTNGIVIEPSEKAKILQER
P02817	QPMMPVPGQHSMTPTQHHQPNLPLPAQQPFQPQSIQPQPHQ
Q92876	CGGVLIHPLWVLTAAHCKKPNLQVFLGKHNLRQRESSQEQS
Q96KN4	GRVVNSWYRYRPLVAELVVQNACGHLGLKSEEICWTNSESF
Q9R1P0	QVEYAMEAIGHAGTCLGILANDGVLLAAERRNIHKLLDEVF
O60238	SSHLVEPPPPLHNNNNNCEENEQSLPPPAGLNSSWVELPMN

Q8BU85	TRASAAAFASAGDTTAMSAFNLLHLVTKSQPVAPRACGLPS
P10746	LAEYICSRESSALPLLFPCGNLKREILPKALKDKGIAMESI
Q9QYI7	RVFPSGFGEFPAFMEALSSFNTLGHGGGSRSTFSSASFGGS
Q8TAP8	QLRKSFQIRCGLEESVSEGLNVPRSKRLFRDLVSLQVPEEQ
Q80WR5	LDPKSDISSLPKVAPVAPCENKFAEDSAEAAVSVPESREPP
P36368	TPTRWQKPDDLQCVFITLLPNENCAKVYLQKVTDVMLCAGE
Q9DCH6	HSQAPMLCSTGCGFYGNPRTNGMCSVCYKEHLQRQNSSNGR
Q91XE0	KFWLFGGNERSQRFIERCIKNFPSSCVLGPEGTPASWTLMD
Q9NPC8	KRELAEATGLTTTQVSNWFKNRRQRDRAAEAKERENNENSN
Q924N9	VSKRFDLALMQLTALLVTSTNVSPVSLPKDSSTFDSTDQCW
Q96AX2	YRDAQALLLLYDITNKSSFDNIRAWLTEIHEYAQRDVVIML
Q6PIV2	PKLPLEKKPNPDKDGPDYEPNLWMWVNPNIVYPPGKLEVSG
Q9Z2F7	GLNSSWVELPMNSSNGNENGNGKNGGLEHVPSSSSIHNGDM
Q9BSJ6	ALGSLCRQFQRRLPLRAVNLNLRAGPSWKRLETPEPGQQGL
P17677	TRKKLKGEKKDDVQAAEAEANKKDEAPVADGVEKKGEGTTT
Q8NDY6	EQRSLRLSINARERRRMHDLNDALDGLRAVIPYAHSPSVRK
Q96L14	DESLNFQKIPPLVHSKTPEGNNGRSGDPRPQAAEPPDHLTI
Q8BFQ8	NLQVATPGGKAIDFVDVTESNARWVQDFRLKAYASPAKLES
P04769	AIEAKNKDLLEYIIRIISKVNPAIKENEDYPTWSDLDSLKS
Q32KU6	KIQSMLHCCGVTNYRDWKDTNYYSEKGFPESCCKLEDCSPQ
Q8R0A6	RRMQAFEASPMWLQDTKPRKNASSVVPSSVHNSANQRMHST
Q95106	FEHVIEELLDEDQKVRPSEENNKDADMYTSRVMLSSQVPLE
Q6IPR3	DQFFTTSSCAGRILLLDRGINGFEVQKQNCCWLLVTHKLCV
O08899	SVQATKEVNLDSCTREETSRNMLQPTITCFDEAQKKIFNLM
Q32L52	RTLALLAFDNPEDSPFGDLLNMMQRQKVWSEVNQAVLDYEN
P19157	SQNQGGKAFIVGDQISFADYNLLDLLLIHQVLAPGCLDNFP
Q9D287	VREAAAALVEEETRRYRPTKNYLSYLTAPDYSAFETDIMRN
O08602	GECLTQPLKDLCQKLRNKVSNTKVDTHKTNGYPHLQVTMIY
P14106	SPLRPNQVIRFEKVITNANENYEPRNGKFTCKVPGLYYFTY
Q6PGD0	LMQARCCLNQKGTILGLDLQNCSLKDPGPNFLQAYTAIIID
Q2TBQ5	ARLGRLVHRKTCTTVAFTQVNSEDKSALAKLVEAIRTNYND
P13810	EFKGGVCISATNVLSKYDLMNFKKLLKRRLALTFFMSEDDF
Q96B21	RLVMAVAVFMEGFLFYYHVHNRPPLDQHIHSLLLYALFGGC
Q8NAJ2	ALSDKPHLSGEVGEEACSSWNPPLFSPRPLPRLWPLPGTPF
Q3SZ19	SIAGLQVDDEILEFGSVNTQNFQSLQNIGSVVQHSEGKPLN
P11836	LSIMDILNIKISHFLKMESLNFIRAHTPYINIYNCEPANPS
Q6PIL6	EIYSQFFPQGDSTTYAHFLFNAFDTDHNGAVSFEDFIKGLS
Q504P2	LQENVFLQLKHNLNSSKKIKNLSAMLQSTATQLCRELYSKE
P70122	LKEKMKIERAHMRLRFILPVNEGKKLKEKLKPLMKVVESED
Q9R1A9	VRTLQGFLSRQEGPICLVAHNGFDYDFPLLCTELQRLGAHL
Q2TBR5	SSMVPGYTGFVPQAQFIFAKNCSQVWAEALNGFTQRNGGQG

P09067	LSGSYRDPAAMHTGSYGYNYNGMDLSVNRSSASSSHFGAVG
A1YQ93	AMPAEVSPYLQKEMINFQHTNAGIFIPSTSQKPSTTIFFTS
Q495Y7	AGLPSWQYQCIHFFLALYLANDMEEDDEDPKQNIFYFLYGK
Q3SWZ3	DIRAQLKAIEPQKEEADENYNSVNTRMRKTQHGVLSQQFVE
Q9BSD3	IQTPESSSVKEELIPQDQKENSLLSCTLHTGTPNSPEPGPV
Q9Z1L2	SLEEGSVVLNTLSPSSTSPSNPPITGPNSLSYTSSALSPYM
Q9NXI6	DLAAGHPSLVGEDGQDEVSANHVAARRLAAHLLLLALLIIL
Q9EQQ2	IWQKTLTVLHPLRAADGSIMNETDLAGPVVFCLAFGATLLL
Q8N7B6	LSTSSPESARKLHPRPSDKLNPKTINPFGEQSRVPSAFAAI
Q28030	VAVKFLNVTPNVEVNVECRINAANIATDDERDKFAPRVAFK
O75558	ISRAQYNALTLTFQRAMHDYNQAEMKQRDNCKIRIQRQLEI
Q58CX6	ASAKAQHLPAPITSASRMQSNRHVMYILKDTSARPAGKGAI
P51153	DKLAREHGIRFFETSAKSSMNVDEAFSSLARDILLKSGGRR
Q9D035	ICFYLEVGQLSQDRDFIMTFNTSLHRSWWMENGPGCLVTPV
Q8WWZ3	DELCFLEQRPQSPTLEFLLRNSQRTVGQLMELCRLYHRADV
Q3ZCH9	EPEETVMIGDDCRDDVGGAQNAGMRGILVKTGKYRAADEDK
P13378	RIEVSHALALTERQVKIWFQNRRMKWKKENNKDKFPVSRQE
P56965	DGGDVLFTGREFFVGLSKRTNQRGAEILADTFKDYAVSTVP
P81126	IEKAIAHYEQSADYYKGEESNSSANKCLLKVAAYAAQLEQY
Q8TEA8	QQQRKEKTRAKGPSESSKERNTPRKEDRSASSGAEGDVSSE
Q9CY66	RGGFNRGGGGGGFNRGGGSNNHFRGGGGGGGGSFRGGGGGG
Q8BW74	PYDGDTFQLEYMDLEEFLSENGIPPSPSQHDHSPHPPGLQP
Q1JPH6	SQKELPTEPPYTAYVGNLPFNTVQGDIDAIFKDLSIRSVRL
Q5QGZ9	MRVDNIINSSAWVIRNAPDLNNMYCGYINRLYVQYYHCTYK
Q5XFR0	VRQLLSPETVDCFFSRTSKENVEADHRSVFVGNVDYGGSAA
Q9P0L0	DRKVCFKVKTTAPRRYCVRPNSGIIDPGSTVTVSVMLQPFD
Q9NVL8	INKQSPRDHKAKKTLQSTPRNDDHDLLTMLPDEILNRGPGN
Q5QGZ9	TWQESKMACAAQNASLLKINNKNALEFIKSQSRSYDYWLGL
Q9H160	KRSKAKQEREASPVEFAIDPNEPTYCLCNQVSYGEMIGCDN
Q9NYW7	FLFAVLLLIFSLGRHTRQMRNTVAGSRVPGRGAPISALLSI
Q32L47	KGEDFESMDATKLSRFIESNNLRLVTEYNAITAIGLFNSMI
Q92915	MHPDGALDGTKDDSTNSTLFNLIPVGLRVVAIQGVKTGLYI
Q8MKH6	KRQTGREMKLRILSERKKPLNIDHMGEEQLREKAQELSDWI
P52946	LHHHLPAQLGLAHPPPGPFPNGTEPGGLEEPNRVQLPFPWM
O75489	TNAQFKSLVDLTAVDVPTRQNRFEIVYNLLSLRFNSRIRVK
P02663	QFYQKFPQYLQYLYQGPIVLNPWDQVKRNAVPITPTLNREQ
Q148F6	GLTRMFAPTLPSDLLVNVYINLNKLCLTVYQLHTLQPNSTK
Q1JP75	DWEATEQALGGVGPVDLLVNNAAVAFLQPFLEVTKEAYDMS
P51148	ADLASKRAVEFQEAQAYADDNSLLFMETSAKTAMNVNEIFM
Q9BQP9	GLIKHNAESRIQNIHFGDRLNASAQVAPGLVGWLISGRKHQ
Q8N9P0	GWGRGMTRICPLCPGAPEYINSAVSSQTLSQSCTPPQSPQP

Q924N9	LPKDSSTFDSTDQCWLVGWGNLLQRVPLQPPYQLHEVKIPI
Q2HJ38	PKLGTDQPLDQATISLQMGTNKGASQAGMTAPGTKRQIFEP
P09671	GWGWLGFNKEQGRLQIAACSNQDPLQGTTGLIPLLGIDVWE
Q6Q8B3	AQISWIPEGSILATKQEYWGNGTVTVKSTCPWEGHKSTVTC
Q9CRA8	LRPKIGLPGVAEKSRERLVRNTCEAVVLGALHPRTSITVVL
P52801	TPPNLVDRPCLRLKVYVRPTNETLYEAPEPIFTSNSSCSGL
P26717	IVLMATVLKTIVLIPFLEQNNSSPNTRTQKARHCGHCPEEW
P15927	VLNLIKACPRPEGLNFQDLKNQLKHMSVSSIKQAVDFLSNE
Q2TBV3	KIRVKPDKTGVVTDGVKHSMNPFCEIAVEEAVRLKEKKLVK
Q9BX74	QYICKDPKINDATQEPVNCTNYTAHVSCFPAPNITCKDSSG
Q9Z1R4	KSTKADLPAPEPRWVDSSPENSGSDWDSAPETMGDVGPLKT
Q9BQR3	FCGGSLIAEQWVLTAAHCFRNTSETSLYQVLLGARQLVQPG
Q9D8Z6	EQERQICREKVGEKLCEKIINIVEVMSRHEYLPKMPTQSEV
Q0VCM2	YEYYAEAPVTSLPAFLAMPFNSLVNMAYVFLGVYWLRSQAR
Q96S21	LHLAFKRQVPTEQARAYAEKNCMTFFEVSPLCNFNVIESFT
Q9Y320	DFDWREVEILMFLSAIVMMKNRRSITVEQHIGNIFMFSKVA
Q5U649	HQSAKEVFKSAHTPVIISVLNSSNILGSLESSLSHLMKFPI
P97469	QPKLSDPHAIEAEKFQCNLCNKTYSTFSGLAKHKQLHCDAQ
A6H759	NKLHGITFLTRNYCLAELYLNNNAIFDIEGLHYLPSLHILL
P35283	KLVDDILKKMPLDVLRNELSNSILSLQPEPEIPPELPPPRP
P43166	LTDALYMVRFKGTKAQFSCFNPKCLLPASRHYWTYPGSLTT
Q3ZBG4	VNTEVRTRLLGHLANCMTQINAMTYPGQPHPALQAPPPPPP
P97950	VPKVLVGNKCDLREQIQVPSNLALKFADAHNMLLFETSAKD
P0CI32	AAKVASLRQRFGEHILAVEGNVTCYADYQRAVDQILTRSGK
P15328	CKEDCEQWWEDCRTSYTCKSNWHKGWNWTSGFNKCAVGAAC
Q9H8W3	RKKVEKSGLEKRIDQAVEEWNIEKAEELSNQLATRELGVKI
P28063	LYYLRNGERISVSAASKLLSNMMLQYRGMGLSMGSMICGWD
Q8NBI2	FSLKNTTRPYHSLPSEAVFANSTGMLVVAFGLLVLYILLAS
O43513	QEGLAPKPPPPIKDSYMMFGNQFQCDDLIIRPLESQGIERL
P08074	MLTKAMAMELGPHKIRVNSVNPTVVLTDMGKKVSADPEFAR
Q8BNI4	VDLLGIAVGHIYFFLEDIFPNQPGGIRILKTPSILRTIFDT
Q5E948	YFAGKLSYVKTCQEKFKNLENSPLGEALRSGQARRSSPTGH
Q32LJ6	RHEVLTRTAFLAEDFNAEEINLDCTNPRYLAAGSSNAVKLS
A7MBB3	FWAIAGIWTVFSLAVVNKAVNLTDGFPYISVCGNVPPQSCI
Q9R0Q9	ASNVPAVVVGKLLQAATNYRNGHTGQLSAITVFMLFGGSLA
A6QP66	AEQSLEVEMMFIRIIRDILTNFKLKEKRRYSGSKSMAKLIN
Q8SQ24	NRGSKMFKLRQLRVEKFIYENHPDVFSDSSMDRFQKFIPTV
P54797	VWCRCASYGTRTNTIILVDANGHVTFTERSMLDKDTSRWET
Q96PB8	CPKGCLCSSSGGLNVTCSNANLKEIPRDLPPETVLLYLDSN
Q9D7U6	SLLGWVLSCLTNYLPHWKNLNLELNEMENWTMGLWKSCVIQ
O43482	HLAWDLSRSLGAVVFSRVTNNVVLEAPFLVGIEGSLKGSTY

Q60994	PPPKGTCAGWMAGIPGHPGHNGTPGRDGRDGTPGEKGEKGD
Q9NWU2	ENRESTPKLAKLLKLLLWAQNELDQKKVKYPKMTDLSKGVI
P10415	PFTARGRFATVVEELFRDGVNWGRIVAFFEFGGVMCVESVN
Q6GV28	LWALILYHNKLKQGQSMHFSNYRITWIMYTAYLNVFFLSVC
P36368	PGFNMSLLMLQTIPPGADFSNDLMLLRLSKPADITDVVKPI
Q9Z2G9	DSWASGYAVPVVTVVRAMLNNLVSPSSGQMELLENKAILHL
Q0VCS6	YQFYKESKSWQGCEYFCIAENSTMLKINTQEVLEFAMPQSY
P32043	SITFPPPAPSNSLHGVDMAANPRAHPDRPACSAAAAPGHAL
Q2YDI9	AAASSRRPADGAGAPSRVRQNFHPDSEAAINRQINLELYAS
P46412	FGLVILGFPSNQFGKQEPGENSEILPSLKYVRPGGGFVPNF
Q8WW14	APKLKPVNSEETVLQALHQYNLQYHPLILECKYVKKPLQEP
Q9NUL5	MKQDRDIQAVATSLLPLTEANLRMFQRAQDDLIPAVDRQFA
Q99N09	QIMCAVMVLSLGIILASVPSNLHFTSVFSVLLKSGYPFIGA
Q9UEU0	LGEQRDQLERTKSRLVNTSENLSKSRKILRSMSRKVTTNKL
Q6SP97	AAMKRLSDEEREAVLQGLKKNWEEVHKEFQSLSVFIDSVPK
O95813	RTVPFSQTITHEGCEKVVVQNNLCFGKCGSVHFPGAAQHSH
P23463	YKAAAAAAAAAAAAAAGEAINPTYYDCHFAPEVSGRHAAAL
Q61200	KFKKMFGWGDFHSNIKTVKLNLLITGKIVDHGNGTFSVYFR
P11245	LQTPEGVYCLVGFILTYRKFNYKDNTDLVEFKTLTEEEVEE
Q9HBV2	NDSETAENYAPPETEDVSNRNVVKEVEFGMCTVTCGIGVRE
Q5E958	HTVRVRGGNKKYRALRLDVGNFSWGSECCTRKTRIIDVVYN
Q2M2D7	QRFGVKQQELCDILVAYSAYNPVSIPGQRYSWYLCPYSQAW
Q7L2Z9	EILALIPNQNALLKDLDILHNSSQMKSMSTFIEEAYKKLDA
P61587	STLVELSNHRQTPVSYDQGANMAKQIGAATYIECSALQSEN
Q8NHM4	CYKPHIQVRLGEHNIEVLEGNEQFINAAKIIRHPKYNRIIL
Q61199	LLVVPGLLQLLFCDSEEVIHNTESVDWEDRTVPETLVGNLF
P35232	IGKFGLALAVAGGVVNSALYNVDAGHRAVIFDRFRGVQDIV
Q2TAA2	LCETAWEEQCIIQGCKLNRLNSVVGEYANACLQVAQDCGTD
Q8VEJ3	VTLLGLSWFCSPLAALVLDFNNIKSSADVQGAGKGSLCASD
Q2HJ98	AFVPKEKIPDPHNLKLWLKVNGELRQEGETSSMIFSIPYII
Q9DBD2	LFMYVQCKVYLQLWKRLKAYNRVIYVQNCPETSKKNIFEKS
Q3ZCB8	PPSPVTDPTMLTDMMKGNVTNVLPMILIGGWINMTFSGFVT
Q17QF9	SFVAPVRGVYSFRFHVVKVYNRQTVQVSLMLNTWPVVSAFA
Q8CF98	GVNDLEREGQYVFTDNTPLQNYSNWKEEEPSDPSGHEDCVE
Q61199	VDHGNGTFSVYFRHNSTGLGNVSVSLVPPSKVVEFEISPQS
P0C1Z6	IVLEDEGSQGTDAPTPGNAENEPPEKETLSPPRRTPAPPEP
P97805	WVFVGAKDLKSKSPYEQFLKNNPETNKYDGWPELLELEGCV
Q9H867	DISGFETCIICCYEQRTMGKNPEIEKKYFELLQLDFDFEKI
Q04917	LETVCNDVLSLLDKFLIKNCNDFQYESKVFYLKMKGDYYRY
Q96KX2	SKEYLIACIEDHNYETGECWNGLWKSKWIFQVNPFLTQVTG
A8MXD5	LGDSDGQQNGHIESEGDENENDQDSLLVLARAASEKGFGTR

O14972	TLQNVKERALLPKFLLRGHLNSTNCVITQPLTGELVVESSE
Q14331	PVFQNGKMALLASNSCFIRCNEAGDIEAKSKTAGEEEMIKI
Q969T4	ELAEITLDPPPNCSAGPKGDNIYEWRSTILGPPGSVYEGGV
P17751	TAYIDFARQKLDPKIAVAAQNCYKVTNGAFTGEISPGMIKD
Q9JIK9	VPKHEEEAFTAFTAKPEDRLNSVPYPPLLRAMILAERQKNG
Q3T075	STYDSTCQIAQEIAEKIQQRNQYERNGENTTKLTVTIRALL
P21266	DVKFKLDLDFPNLPYLLDGKNKITQSNAILRYIARKHNMCG
Q9D8B1	VFFGICVLTDLSSLLTRGSGNQEQERQLRKLISLRDWTLAV
Q5E9Q4	ESVLCVKPDVHVYRIPPRATNRGYRAAEWQLDQPSWSGRLR
Q0VC21	AKVNIEVQLASELAIAAIEKNGGVVTTAFYDPRSLEILCKP
Q91XR9	LVDLHARYAECGLRILAFPCNQFGRQEPGSNQEIKEFAAGY
Q9CR68	CRESLSGQAAARPLVATVGLNVPASVRFSHTDVKVPDFSDY
Q9XT56	THGDIRGLVCYNNKITASYENRVTFSDTGITFHSVTRKDTG
P97950	DLREQIQVPSNLALKFADAHNMLLFETSAKDPKESQNVESI
A6H6X4	SRWEKEQLTPRETTFLEITDNHDRSQGLRIFCNAPDFISKI
O88456	TAMRILGGVISAISEAAAQYNPEPPPPRSHYSNIEANESEE
Q6P1K2	FIAQLQTSIREEISDIKEEGNLEAVLNALDKIVEEGKVRKE
Q15771	CEESFRCLPEWLREIEQYASNKVITVLVGNKIDLAERREVS
Q5TZF3	WGRLETLKALVELDVDIEALNFREERARDVAARYSQTECVE
Q9BU20	LADGRTLDGRAGLADVAHILNGLAEQLWHQDQVAAGLLPNP
Q0III0	YNSQPSVSLRDFKQLKKCWENIKARTKKIMAHERREKVKRS
Q7TMY4	RRKQFHVLLSTIHELQQTLENDDKLSEVDEAQESTMEADPK
Q2HJ67	GSDSHHGGKNSLGNSSGASANAGSTHISSREGVGTASGAEE
Q9D9R0	VRTRCGHVFCRSCIATSIKNNNKWTCPYCRAYLPSEGVPAT
O08583	ADALKAMKQYNGVPLDGRPMNIQLVTSQIDTQRRPAQSINR
P20339	YADDNSLLFMETSAKTSMNVNEIFMAIAKKLPKNEPQNPGA
Q12918	IYWQQLREKCLLFSHTVNPWNNSLADCSTKESSLLLIRDKD
Q8NC54	QEEADNNEDPSIEEEDLLMLNSSPSTAKDTLDNGDYGEPDY
Q8CFI2	VTLVDEGDLYNWEVAIFGPPNTYYEGGYFKARLKFPIDYPY
Q27956	AHGLHSKLSTTADVLRGLLSNVLCRLCSKYHVSHVDVTYGP
A4D161	IRCRCKHFADQHSAAPGFTCNTCSKCSGFHSCFTCACGQPA
P67870	RQALDMILDLEPDEELEDNPNQSDLIEQAAEMLYGLIHARY
P09067	RIEIAHALCLSERQIKIWFQNRRMKWKKDNKLKSMSLATAG
A2VDN0	DTYRLRRRATRRRINKREAKNCNAIRHFENTFVVETLICGV
P22794	RLWANSTSSWDSVIQNKTGRNQNENINTNPITPEVDYKGNS
H3BNL8	PSAETYFHEEKIVVLGQVLMNESLPIEKRAQAAQKIGLLAF
P34022	FEECRKEIEEREKKGPGKNDNAEKVAEKLEALSVREAREEA
Q5E9Q4	NPDQGPKLDLSFKEGQTIKLNIASMKKKDGAAGTPRARPTS
A6QLP7	SFRYPQDYQFYIQNFTALPLNTVVPPQRQATFEYSFIPAEP
Q9TU47	EVFRQTVADQVLVGSYCVFSNQGGLVHPKTSIEDQDELSSL
Q9JL25	HNKAENIYKAFVHSDAVKQINIDFHTRESTAKKIKTPTPTS

Q6UX65	CSSVLHSGNFGTDLEQKLHWNPEDKGYVLHMITTAAEWSMS
Q9P0I2	GIELLTLDASWVSSASWYFLNVFGLRSIYSLILGQDNAADQ
Q8NBL3	RCTAIKYHFSQPIRLRNIPFNLTKTIQQDEWHLLHLRRITA
Q7TS73	PVFFGESIEVDPEPAHEIRCNSEITYASERYFRDKIFYAPV
Q96NF6	VTTQVCIMPARLSLGSGISRNLLRLSVCHFTLLLPFRSLRP
Q1RML4	FQFVSKNKEKLAQLWGKRKGNRKTMTYQKMARALRNYGRTG
Q08091	RFEPEKLREGRNIIGLQMGTNKFASQQGMTAYGTRRHLYDP
P35802	FMLAWLGVTAFTSLPVYMYFNVWTICRNTTLVEGANLCLDL
Q9DAZ2	KEQSLVRTPETLLTLVHSLLNSWTNALNHLVNEMSTMQGDT
Q15116	RVTQLPNGRDFHMSVVRARRNDSGTYLCGAISLAPKAQIKE
Q9JI59	YIWFKDGTSLLGNPKGGTHNNSSYTMNTKSGILQFNMISKM
Q9D5K1	WRLARGIISTNDELFRPFRANSPFIGFKPAYEYNAGTYRCD
P00492	EDIIDTGKTMQTLLSLVRQYNPKMVKVASLLVKRTPRSVGY
Q0VCV7	GPHPAKTPARPVGTSEPKSANLCGNRTYGKALMPPVARISV
Q62189	IATMPVPETRANHTIYINNLNEKIKKDELKKSLYAIFSQFG
Q9BUV0	DRMELLEIAKTNAAKALGTTNIDLPASLRTVPSAKETSRGI
Q2KHU3	LKNVQLPEVTINEETALAEVNLKKKSCLNIRTHPVATSFAV
P41220	EKMKRTLLKDWKTRLSYFLQNSSTPGKPKTGKKSKQQAFIK
P11352	KNEEILNSLKYVRPGGGFEPNFTLFEKCEVNGEKAHPLFTF
P50591	ALGRKINSWESSRSGHSFLSNLHLRNGELVIHEKGFYYIYS
O19137	QQTQPPTKRTPQVIGVMQSQNSSAGSRGPRPLEQVTCYKCG
A2VE33	IQCNSLKDSCVAKNATECPSNATTSCTSFSTNFYHGEHPTW
Q6PID6	SKVTSQQFEAEAADEKDVVDNDEGNWLHAIKRRKEILLEGC
Q62348	LHISTFINELDSGFRLLNLKNDSLRKRYDGLKYDVKKVEEV
Q96A37	PIPSNIPNRSTFACPYCGARNLDQQELVKHCVESHRSDPNR
A4IFA7	GLVGFSRALAKEVAKKKIRVNVVAPGFIHTDMTKDLNEELL
P00642	LTAANYGMPINSNLCINKFVNHKDKSIMLQAASIYTQGDGR
Q14AM7	ASISVRSSTPGSPTHVSSGPNASRRRNGLHDVDLNTFITEE
P49862	VYKDLLENSMLCAGIPDSKKNACNGDSGGPLVCRGTLQGLV
Q6ZN17	PEPAEEESQVLRGTGHCKWFNVRMGFGFISMINREGSPLDI
Q56JV9	VAFRKFKLITEDVQGKNCLTNFHGMDLTRDKMCSMVKKWQT
A5PKE4	LKKKIPSREVLKSTKIGHTVNKMRQHSDSEVACLAREVYTE
P12235	IIDCVVRIPKEQGFLSFWRGNLANVIRYFPTQALNFAFKDK
P59034	IGPAAFSGLAGGLRLLDLSHNRIRRIPKDALGKLSAKIRLS
P33681	HLRVNQTFNWNTTKQEHFPDNLLPSWAITLISVNGIFVICC
Q86XR7	MRPLNNPLPRERTPFALQTINALEEESRGFPTQVERIFQES
P0AC11	HIAAFFDARIAALTGAGIKRNRLVLDPGMGFFLGAAPETSL
O75828	ETLTEGDLVDLMKKFVEDTKNEVHEREGWPNSPYGVSKLGV
P0DMC8	LEQIEWVNVVGEFEDSTALINNLPKLDAHVLITDLSMPGDK
P09056	IRHPCHGNLMNQIKNQLAQLNGSANALFISYYTAQGEPFPN
Q96L15	GLTLPPGFKAQNGIAIMVYTNSSNTLYWELNQAVRTGGGSR

Q9D3G5	RQNVRFLTSMRRLSSIKRDTNSIAKAIKTRGEGIHQKLRSM
Q9CZ96	VAFILFLDKDSALNCTRAINNKQLFGRVIKASIAIDNGRAA
Q5SQ64	GRPAPDPGKPGRESRLRLLGNYSLWLEGSKEEDAGRYWCAV
Q0VD35	MGELRGLRRNYRCTTCSVSLNSIEQYHAHLKGSKHQTNLKN
Q8TDC0	LGKKLSVPQDLMMEELSLRNNRGSLLFQKRQRRVQKFTFEL
Q9WUL5	VTSVLRLKPQPSRNFSCMFWNAHMKELTSAIIDPLSRMEPK
P12235	GDCIIKIFKSDGLRGLYQGFNVSVQGIIIYRAAYFGVYDTA
Q14002	EVTRQFYVFSEPPKPSITSNNFNPVENKDIVVLTCQPETQN
Q3T0X7	LKGKKMRKKEAEHVLQKFVQNKWLIEKEGEFTLHGRAILEM
Q17QI5	WCGGNFLEVTEQILAKIPSENNKLTYSHGNYLFHYICQDRI
Q9GZN7	TDQVKGVLTLQGDALSQADVNLKMPRNNQLLHFAFREDKQW
P51149	GDSGVGKTSLMNQYVNKKFSNQYKATIGADFLTKEVMVDDR
Q3SWY4	RVEADMGRAEVHWCAPSSPVNQYWLLLWEGGGAPQKGPSFN
Q15005	KWKIDDKPVKIDKWDGSAVKNSLDDSAKKVLLEKYKYVENF
Q8R092	LADDDTRLLQLETQGNQSCYNYLYRMKALDAIRASEIPFHA
P47985	SRESLSGQAVRRPLVASVGLNVPASVCYSHTDIKVPDFSEY
Q6PDY0	QGHLGEIRELKQLNRRLQAENRELRDLCCFLDSERQRGRRA
Q9H853	RLISQIVSSITASLRFDGALNVDLTEFQTNLVSYLTSTSPW
P04179	EALAKGDVTAQIALQPALKFNGGGHINHSIFWTNLSPNGGG
P15119	AAHCNGSEISVILGAHNINKNEPTQQIIKTEKTFVHPKFQY
Q3SZG6	AYGTIVKMKRDDLKATLTARNREAKQLTQLERTRQRNPSDR
Q86UA6	LVGSPPWKEAFRQRCLERMRNSRDRLLNRYRQAGSSGPGNS
Q99614	ECFHDCSASFEEEPGADKVENKSNEDVNSSELDEEYLIELE
O43760	AVCLVFALIVFSCIYGEGYSNAHESKQMYCVFNRNEDACRY
O54709	DTSQEQQKQRLALTTSQPGENGIIRGRYPIEKLKISPMFVV
Q9TQE0	SDVGEYRAVTELGRPVAESWNSQKDFLERRRAEVDTVCRHN
Q15560	MARTGGTQTDLFTCGKCRKKNCTYTQVQTRSSDEPMTTFVV
Q3UME2	VRRSLLGLTFCTCYLASHLTNKYVLSVLKFTYPTLFQGWQT
O75841	AFRTENNDADYPWPRQCCVMNNLKEPLNLEACKLGVPGFYH
O95377	CHADPCPNIVDCFISKPSEKNIFTLFMVATAAICILLNLVE
Q9CWQ0	ILRATKLGIPYQVIHNASIMNAVGCCGLQLYRFGETVSIVF
Q9Z1L4	ERLNWIYYKDQTGNNRVFYGNSDRSSTVQNLLRPPIISRFI
Q9CQE8	AEKYKDLVPDNRKNTDNAAKNAEPLINLDVNNPDFKAGVMA
Q9CR59	SYRAPPPPRRRPGPHSPDPENLLTPRWQLTPRYVAKQFGRH
P49720	GLAGLATDVQTVAQRLKFRLNLYELKEGRQIKPYTLMSMVA
B9EJG8	LVVAVLRFIQLKPKVLNPWLNISGLVALCLASFGMTLLGNF
Q60651	YDNKKKDWAWIDNRPSKLALNTTKYNIRDGGCMFLSKTRLD
P53810	PTFVRMLAPEGALNIHEKAWNAYPYCRTVITNEYMKEDFLI
P55859	LGGLVNKLTQAQTFDYSEIPNFPESTVPGHAGRLVFGILNG
Q8BXP5	CYHGFGSIVALLSCCPFLDVNQQDKDGNTALMLAAQAGHMS
P05208	VCAGGDGVTSSCNGDSGGPLNCRASNGQWQVHGIVSFGSSL

Q8N6Q1	IEEELEALFLEREVSKLVSMNPVEKEHTSQNNEGTPTQKTA
P30711	ELYLDLLSQPCRAVYIFAKKNDIPFELRIVDLIKGQHLSDA
Q6ZS82	DVADLRELEREVLQVGEMIDNMEMKVNVPRWTVQARQAAGA
Q9H190	KMPALPVQATAISPPPVLYPNLAELENYMGLSLSSQEVQES
P08831	EEEIIKPRSAHYSFQSNVKYNFMRVIHQECILNDALNQSII
P70190	EYLRSVVMCALKLYVNDHYPNGNCNVLRKTVKSKEFLIACI
Q9BYG3	PTPVCTPTFLERRKSQVAELNDDDKDDEIVFKQPISCVKEE
Q96SN7	VHLFALLISTCILPNVEAVSNIHNLNSISESPHERMHPYIE
P47865	VATAILSGITSSLPDNSLGLNALAPGVNSGQGLGIEIIGTL
P09093	GDILPNKTPCYITGWGRLYTNGPLPDKLQQARLPVVDYKHC
Q96PB8	IDEHAFKGVAETLQTLDLSDNRIQSVHKNAFNNLKARARIA
A5D9C6	EGPLSDKCSRKTLFYLIATLNESFRPDYDFSTARSHEFSRE
A6QR46	IPSYIRDSTVAVVVYDITNLNSFQQTSKWIDDVRTERGSDV
P21796	ETKYRWTEYGLTFTEKWNTDNTLGTEITVEDQLARGLKLTF
Q2KI95	ICHRCQQPIGTKSFIPKDSENFCVPCYERQYALQCVQCKKP
P59542	DERVWTKEYEGNVTWKIKLRNAIHLSSLTVTTLANLIPFTL
O75629	PGAGSGVPYFYLSPLQLSVSNLQENPYATLTMTLAQTNFCK
P41227	RLGLAQKLMDQASRAMIENFNAKYVSLHVRKSNRAALHLYS
Q96JC9	PMTVFKGNKRPYQKDCVLIINHDTGEYVLEKLSSSIQVKKT
Q7Z692	STWMATTEKPELGPAHDAGDNNIYEVMPSPVLLVSPISDTR
Q1LZB9	AECCASGNIDTAWSNFTHPGNKISLLGFLGLVHCLPCKDSC
P78380	ASQESENELKEMIETLARKLNEKSKEQMELHHQNLNLQETL
Q6PH85	MAVAYWKLVLSGRFKFLDLWNTFLMEHHKRSIPRDTWNLLL
Q8IZ81	NLTEMAYSLLKSEALKFHLYNLVPGIPTMEHFHQFYCYLVY
A2AFE9	PMALMIGGPRVGSRVLERSGNNSKPYIPVPRSQGFFPPRGS
Q8R092	GRHPCSLMGKNFRSYLLDLRNTSTPFKGVGKALIDTLLDGY
Q9BVC5	SVSPLILSSNLPVNNKTEHNNNDAKQNHDLTHRKSPSGPVK
O95639	QNSSPNQQRTPQVIGVMQSQNSSAGNRGPRPLEQVTCYKCG
P40240	AIGLWLRFDSQTKSIFEQENNHSSFYTGVYILIGAGALMML
P15945	ICDGVLHGITSWGPSPCGKPNVPGIYTKLIKFNSWIKDTIA
Q5JUR7	LASHLASHGFFCLRFTCKGLNIVHRIKAYKSVLNYLKTSGE
Q2TBT5	YPVVSAASICAKVARDQAVKNWKFVEKLQDLDTDYGSGYPN
O89051	VILNEPSADAPAARYQTIEENIKIFEEDAVEFISVPVPEFA
Q9EQ06	IYSAAKKVKEEVGDVSILVNNAGVVYTADLFATQDPQIEKT
P54369	GNSQRDHSLSASILYSDERLNVTEEPTSNDKTRVLSIQSTL
P17667	LRNAIRYIESLQELLREQVENYYSLPGQSCSEPTSPTSSCS
Q9NUI1	AVDAMTRHLAVEWGPQNIRVNSLAPGPISGTEGLRRLGGPQ
Q9Y2B2	TVLGLARLRHWVYLLCFSAGNYYNQGETRKKELLQSCDVLG
Q9CQE0	FKISQDSVRSSNRSETSASDNTETYQEDTSSSGHPTFKCPL
Q9D9J8	KEWFSANITLEFDIEFKLPFNSNIIKTHACMGLTAESWLEK
O35943	LHYLQILNIKKQSVCVVHLRNLGTLDNPSSLDETAYERLAE

Q5VVH5	KTPPTRVFVELVPWADRSRENNLASGRETLPGLRHPLSSTQ
Q64329	YSTKCYYFIMNKTTWSGCKANCQHYSVPILKIEDEDELKFL
Q8CJC5	GEPVPESEVISGEECVICFHNTANTRLMPCGHSHFCGSCAW
P70280	IVYLCITDDDFERSRAFSFLNEVKKRFQTTYGSRAQTALPY
Q8BXV2	EVLGLDVSNLSQYFSPASVSNSPTRALVLVGVVLLAYWFLS
Q14197	KINRLGELILTSESSRYQFRNLADCLQKIRDMITEASQTPK
P02647	KDSGRDYVSQFEGSALGKQLNLKLLDNWDSVTSTFSKLREQ
P52803	VRPTNSCMKTIGVHDRVFDVNDKVENSLEPADDTVHESAEP
Q8VCH9	CPKGCLCSSSGGLNVTCSNANLKEIPRDLPPETVLLYLDSN
Q9D4C5	PMTVFKGNKRPYQKDCVLIINHDTGEYVLEKLSSSIQVKKT
Q9HBJ8	TDPSKNHTLPAVEVQSAIRMNKNRINNAFFLNDQTLEFLKI
P10747	VCVVYGNYSQQLQVYSKTGFNCDGKLGNESVTFYLQNLYVN
Q7L4S7	NTSPTPDPTVREALCAPDNLNASIESQGQIKMYINEVCRET
Q2YDI9	RLQNQRGGLICLQDIKKPDQNDWKSGLNAMECALLLEKNVN
O08547	IQKTKKLYIDSRARRNLGSINTELQDVQRIMVANIEEVLQR
P67936	RELDGERERREKAEGDVAALNRRIQLVEEELDRAQERLATA
O35536	YDRDQQKCRRFNYGGCLGNANNFHSRDLCQQTCGSIEKVPP
Q9H2J4	VVKTYEDMTLEELEDHEDEFNEEDERAIEMYRRRRLAEWKA
Q53TN4	RLPWTWKCSKLLMKSIHAGLNAVAAILAIISVVAVFENHNV
Q91ZW8	AGLLLIILILVSKVPSSEVQNKIYQELMQLKAEVHDGLCQP
Q8R023	YDEQLSLCLERLSSGKDKNKNVLQNKYVRCSVRAEVRHLRR
Q9UKY7	MEKSFEVVRHKNRGRDEVSKNQALKLQLDNQYAVLENQKSS
Q8N966	AARLFSPALLHGALFLFLSANALGNYVLVIQNSPDDLGACQ
P0DMB1	SQTEPQNDNEGETSLATTQMNPPKRRQVEQGPSTGAKKPSI
Q8TED1	LKELHKEFGPSHFSVLAFPCNQFGESEPRPSKEVESFARKN
Q9JHH9	ETVDGGVILESDPQQVIQKVNFRTDDSGLTEQSVAQVLQSA
Q3T116	YPESRGADTAWRVPGDAKQGNDDIPVDRLTISYCRSSGPGG
Q9NP99	TVTQAPPKSTADVSTPDSEINLTNVTDIIRVPVFNIVILLA
Q8TAG6	SKVSSPARRRAKSSQHLLTKNVVIESDLYTHQPLELLPHRG
Q6NZB0	LTSKNQIERLTRPGSSYFNLNPFEVLQIDPEVTDEEIKKRF
Q92567	VQPNSIPSAIYPAPVAAPRTNGVAMGMVAGTTMAMSAGTLL
A3KN24	QAETLLLEPMVHELVLWIQENLRHILKCPEAGGGSEKCSSA
Q96AQ2	CLLALLVLVKQLMSSAVQDMNCIRQAHHVALLRSGGGADAL
P11900	SVITPSFTSNTAMDIAVKVKNSGDNTELGTLSVPLSFGAAV
P63158	REMKTYIPPKGETKKKFKDPNAPKRPPSAFFLFCSEYRPKI
Q9CRA0	KALPESMTPVHAVAIVVFTLNLNVSSDLAKAMARAAGSPGQ
Q1LZB9	ATCSLVLKTDVSQAECCASGNIDTAWSNFTHPGNKISLLGF
P32972	NSKIKKQTLVTVCESGVQSKNIYQNLSQFLLHYLQVNSTIS
P10629	YSPQENVVFSSSRGPYDYGSNSFYQEKDMLSNCRQNTLGHN
P81126	EMYTRAANMFKMAKNWSAAGNAFCQAAKLHMQLQSKHDSAT
Q9BZM5	LHYDCGNKTVTPVSPLGKKLNVTTAWKAQNPVLREVVDILT

Q80W35	SVLLLACTSPLNLVQFLVNNNGLELKAGLWTLCYHELCWSH
Q9UNN8	LGCELPPEGSRAHVFFEVAVNGSSFVSFRPERALWQADTQV
P28667	GDVTAEEAAGASPAKANGQENGHVRSNGDLTPKGEGESPPV
P25401	PYIFAIGSLLDGNGKKIATDNGTTQKLLMFMPGDEVQVKGN
Q32KP7	SLFSELEAKQLRKLYKYTKNNQTTKFLMAFCPLDAPESSLL
Q9BQI4	FEEEDHVKKLQQKVATLEKRNRQLRERVKKVKRSLRQARKK
Q8VC04	GKAVSQLTSRKDEDKPILPDNPAMASQAANYFSTGSSKPAH
Q6P4H8	HGSAKFYISDLWKVTFSQYSNVVIFGVPQMMLQLEKKLERE
Q9EPQ7	NATHVEHPLCPPKPGFVRGFNHPCGCFCEPLPGDPNKTNLV
Q8WW62	FYQLCLSNQHNHFGSVQVYLNFGVFYEGPETDHKQKERKQL
Q9N0V4	GLDFPNLPYLIDGTHKLTQSNAILRYIARKHNLCGETEEEM
Q96KN8	TGPKDQPPALRRSAVPHSGLNSISPLELEESVGFAALVQLP
Q8CCI5	SANATTKTSETNHTSRPRLKNVDRSTAQQLAVTVGNVTVII
Q9NRD1	SADYFVLASFEPPPVTIQQWNNATWTEVSYTFSDYPRGVRY
P04230	SDVGEFHAVTELGRPDAENWNSQPEFLEQKRAEVDTVCRHN
E1ANH6	AIPGDPRDTTTPLAMAQTLKNLTLGKALAETQRAQLVTWLK
P23888	VFKNLNIIFPKGYNIALIGQNGAGKSTLLRIIGGIDRPDSG
Q2HXU8	MLLIGLVTLGMMFLQISNDINSDSEKLSQLQKTIQQQQDNL
P22027	KKLPWSRDSRYFWGWLNAVFNKVDHDRIRDVGPDRAASEWL
Q2T9W7	KRIKEHLTESLFFEQSFQPENRTVSSGPSLLTVFLAVVCIT
Q9NPA0	PMVMMMVLPLLIFVLLPKVVNTSDPDMRREMEQSMNMLNSN
Q9JJW5	GGSQQGPSTPPNTPDPRSPPNPENIAPGYSGPLKEIPPERF
Q61334	TQLAKEIANKGVLKIQAENTNKAAKKFMEENEKLKLGLRND
P09016	KQPAVVYPWMKKVHVNSVNPNYTGGEPKRSRTAYTRQQVLE
A5PJS2	GSALWLIGMLINIHSDHVLRNLRKPGETGYKIPRGGLFEYI
Q78HU3	VVDMQIVMDKGPLPSGFSAVNDPQDIKASVSKKKRMCVKLM
P48739	CAYKLVTIKFKWWGLQSKVENFIQKQEKRIFTNFHRQLFCW
Q2YFS1	KDPQMSIAWRWKDFFGHFIYNSSMPFIHEHFKGRLILNWTQ
Q30134	RWFRNGQEEKTGVVSTGLIHNGDWTFQTLVMLETVPRSGEV
P04228	TNEAPQATVFPKSPVLLGQPNTLICFVDNIFPPVINITWLR
P17931	FNPRFNENNRRVIVCNTKLDNNWGREERQSVFPFESGKPFK
Q8K5B8	LNEGNKSASAGDPGSLVSPLNPGGGLSASGAPWYPIHSRSR
Q8N755	VLVSSWFILALQKWIIDLAMNLCTFISAASKFAQLQCLWKT
Q5U680	AGGFRGIYAGVPSAAVGSFPNAAAFFLTYEYVKSLLHTDST
Q2YDG2	NWDSHIAIWNSTPNYQVIADNPEGLLFKYKRDRKILNVDPK
P10036	EALAAVHGSEFSQTTICRFENLQLSFKNACKLKAILSKWLE
Q1LZ96	VEQAVLLSRLEEEYQIQKWGNIEWAHDYELQELRARTAAGT
Q3ZBS0	RIKAQLPLKDKARMARHVLDNSGEWSVTKRQVVLLHAELEH
P23946	LEIVTSNGPSKFCGGFLIRRNFVLTAAHCAGRSITVTLGAH
Q96BS2	HRRFKQLSGDQPTIRKENFNNVPDLELNPIRSKIVRAFFDN
Q8K3A2	APGGRRKEGRRTHRPREQDRNVQLSKALSYALRHGALKLGL

A2ANU3	VQQLLDPNTLQQSVESHYRPNIILYSDGVLRSWGDGVATDC
Q9DBJ1	LPVVRTWRLNERHYGGLTGLNKAETAAKHGEAQVKIWRRSY
Q9CQ61	AVGYYIYVTFLGYSALPFLKNTVILLYPFAPLMVLYGLSLA
Q8C4N4	SILCFAASTFPQYIKACKTGNMDQALSLWFLLGWIGGDSCN
P60879	EAEKNLTDLGKFCGLCVCPCNKLKSSDAYKKAWGNNQDGVV
A5D9C6	LDSDPFGEDGSLWSFNYFFYNKRLKRIVFFSCRSISGSTYT
Q8N129	KRKRHVPYSVSETRLEEALENLCERILDYSVHAERKGSLRY
Q9NPC3	MERNRQYQKLQGLYDSLRLRNITIANHEGTLEPSMIAQSGV
Q9Z2C6	CGVNGPSDWQKYTSAFRVENNDADYPWPRQCCVMDKLKEPL
A2VDZ9	RGPFTDVVTTNLKLGNPTDRNVCFKVKTTAPRRYCVRPNSG
O35075	PRLFTCPTLETTNFKVEFEVNVVVLLHADHLITENFPLKLC
P59041	RERRLRARREALRKKQENQANKGTSWDDTRDATFFVVLFLI
Q6PIL6	IKGLSILLRGTVQEKLNWAFNLYDINKDGYITKEEMLDIMK
P18669	EAIMELNLPTGIPIVYELDKNLKPIKPMQFLGDEETVRKAM
Q1WG82	SLRKSRPPALEPGALRCLTPNIRSLWPTCQDSVSTALPFLQ
Q9D8U2	SLFFFLLFLRLFPMTPNWFLNLSAPILNIPIVQFFFSVLIG
Q92979	GEQAQDWDALPPKRPRLGAGNKIGGRRLIVVLEGASLETVK
Q91VE3	GIPDSKTNTCNGDSGGPLVCNDTLQGLVSWGTYPCGQPNDP
B2RXH8	LLENLEKIEKEQSKQEVEVKNAKSEEEQSSSSMKKDETHVK
Q9D777	RAVLTQKHKKKHSVLHLVPVNITSKADSDVTEVMWQPVLRR
Q13242	EKDLEDLFYKYGRIREIELKNRHGLVPFAFVRFEDPRDAED
O35658	EEPSQGQKAEEQEPERTSTPNFVVEVTKTDGKKTLVLDCHY
Q99LS1	WPDETMGPFGPQDQRFQLPGNIGFDCHLNGTASQKKSQAHK
Q9BTT0	EVTELVLDNCLCVNGEIEGLNDTFKELEFLSMANVELSSLA
Q9D3B1	SGARKKKGPGPVATAYLVIYNVVMTAGWLVIAVGLVRAYLA
Q9CRC9	EAGGIDLFVGGIGPDGHIAFNEPGSSLVSRTRLKTLAMDTI
P01139	TTATDIKGKEVTVLAEVNINNSVFRQYFFETKCRASNPVES
P97299	MSAFGFPWPDMLECDRFPQDNDLCIPLASSDHLLPATEEAP
P0DMD0	ETVDDLAIACDSQRPSVVFINEDCFIHDASNSQHIKHIINQ
Q14493	RSRCSDWASAVEEDEMRTRVNKEMARYKRKLLINDFGRERK
Q13637	AKDNINIEEAARFLVEKILVNHQSFPNEENDVDKIKLDQET
Q9JKF4	FGSSCYLISTKENFWSTSEQNCVQMGAHLVVINTEAEQNFI
P41247	SAGSLVASVLLTAPEKIEECNQFTYKFAEEIRRQSFGAVTP
Q96M98	VAEIIVNSGDGIDYSQQKRENIGDLIQETLEAFERYGGENA
Q99581	KGSGQIQLWQFLLELLADRANAGCIAWEGGHGEFKLTDPDE
P30408	HSLVGLALLCIAANILLYFPNGETKYASENHLSRFVWFFSG
P40237	LFFILGAVILGFGVWILADKNSFISVLQTSSSSLQVGAYVF
Q8WY41	SGAAAARLLKPELQVCVFCRNNKEAMALYTTHILKGPDGRV
Q077R2	KNIEIIICDDSSTDDTWDIINKIKDSRIICIKNNYCKGAAG
P09326	KRPFPKELQNSVLETTLMPHNYSRCYTCQVSNSVSSKNGTV
Q9D3G5	FETDNILESLYRVIQDIQDENQLLLIDVRRLGRQNVRFLTS

P02970	WEWKVGTGLNGFGNVLNDLTNGGTKLTITVTGNKPILLGRT
Q3ZBN4	KIHRPMAPKEAPKKLIRYIDNQIVSTKGERFKDVRNPEAEE
Q0VCU8	RLKKLQEESDLELAKETFGVNNTVYGIDAMNPSSRDDFTEF
P32971	LVFTVATIMVLVVQRTDSIPNSPDNVPLKGGNCSEDLLCIL
Q0VD35	SPYQRRDSDRYCGLCAAWFNNPLMAQQHYDGKKHKKNAARV
Q6P3A1	PRKGWEEGSKADVRVTSSKENCSPQTEAAWPKHTIDNAKSL
Q8C5T4	IYEGQWQNDKPEGEGMLRLKNGNRYEGIWERGMKNGHGRFF
Q14135	DLDCDNDHVSKMSRIFNPHLNKTANGDCRRDPRERSRSPIE
O89116	IAYSDEVRNELLGDAGNSSENQRAHLLDNTERLERSSRRLE
Q9Y2W7	ILLRGTVHEKLKWAFNLYDINKDGYITKEEMLAIMKSIYDM
Q9CWD3	ISQESQEQLQARIQVNPNEVNAFMWLGPDVAAAVVATEDGT
P31947	NVVGGQRAAWRVLSSIEQKSNEEGSEEKGPEVREYREKVET
Q80VP8	ADRDQEEAIAQFPYVEFTGRNSITCHTCQGAGYIPAEQVNE
P50135	SMRSLFSDHGKYVESFRRFLNHSTEHQCMQEFMDKKLPGII
Q9BR01	TSPRLIKSHLPYRFLPSDLHNGDSKVIYMARNPKDLVVSYY
P20231	QFYTAQIGADIALLELEEPVNVSSHVHTVTLPPASETFPPG
Q8TD47	TVQIDLGTGKITSFIKFDTGNVCMVIAGANLGRVGVITNRE
P0CI00	DTSTSMTMENSLILEDPFEYNDSGEDCTHSSTITQCLLTHS
Q96PB8	RIQSVHKNAFNNLKARARIANNPWHCDCTLQQVLRSMASNH
P02722	LSFWRGNLANVIRYFPTQALNFAFKDKYKQIFLGGVDRHKQ
Q6IC83	LENIGPTEDVQASAHGGVEENMTSDIEIPEAKHDHRPTEDV
P47753	GQQTIIACIESHQFQPKNFWNGRWRSEWKFTITPPSAQVVG
Q9EP52	ELHEPIPSLFRALTEGDTQLNWNIVSFPVAEELSHHENLVS
Q8R3S2	EDNPSRERCSVPYSCCLPTPNQAVINTMCGQGMQALDYLEA
Q3KP22	KEKPNKDCRRLWPLISLMSRNKILSGDTACQGELSHPCSTT
Q61599	EFLTPVEEAPKGMLARGTYHNKSFFTDDDKQDHLTWEWNLA
Q9UIY3	IGTFDPGELCVCAAIQWLQDNSASYFLNRKLVYEPSTQAKP
Q8CD60	LTTRRLGPTLAPEQTRRVTRNSSTQTVSDKSTQTVLPYTAT
Q99KR3	RNPQREEIIGNGEQQFIYIENGDVVKTEGATLRVLYTPGHT
Q8BW11	KDPDSLQTVNVTVISKAECRNAYKAFDITENMICVGIVPGR
P51148	NWVKELQRQASPNIVIALAGNKADLASKRAVEFQEAQAYAD
A8MWP4	SLHLQDSRNASSLPHKGWRCNFPLQGPAGLTHKSACVGRMG
Q9H0W7	HRFPLDPKRRKEWVRLVRRKNFVPGKHTFLCSKHFEASCFD
Q8NGN8	DIIYSSSISPRLISDLFFGNNSISFQSFMAQLFIEHLFGGS
Q0VCP2	LLLLFHSLKRHPPLFLDTVKNFCDILNPLDQLGIYKSNPGI
Q5VWZ2	FLHGSGDSGQGLRMWIKQVLNQDLTFQHIKIIYPTAPPRSY
Q9D711	EQSEGVGARVRRSIGRPELKNLDPFLLFDEFKGGKPGGFPD
A6NDR6	AFAKQVRSERPFFSSNPELDNLMIQAIQVLRFHLLELEKGK
Q64695	SFRPKTAVWVSGSQEPSKAANFTLKQLNAYNRTRYELQEFL
Q8CI43	QAPAKSQEPPVDLSKVVIEFNKDQLEEFREAFELFDRVGDG
Q9HC24	PALILLFALGSLGLIFALILNRHKYPLNLYLLFGFTLLEAL

Q0VD59	RLKAYNRVIYVQNCPETSKRNIFEKPALPEPNFESKDGRGV
Q9D968	ALVSQFYLLPKGKQSMSKVGNADVPDYSLLKKQDLVPGTVY
P12319	LNPPWNRIFKGENVTLTCNGNNFFEVSSTKWFHNGSLSEET
Q8IUA0	WTVRTEGGHFPLHSPTFSWRNVAFLLLLSLALEWTSAMLTK
Q3ZCD8	ENAVRQNGQLVKTDSMYGISNIDAMIEGTSEDMTVVDAASL
Q9CXV9	NKDQWYNVLEFSRTVHADLSNYDEDGAWPVLLDEFVEWQKI
Q15040	EWIGGESELRKFLKHHLRGKNCELLLVVPEEVEAHQSWRTD
P15927	PCTISQLLSATLVDEVFRIGNVEISQVTIVGIIRHAEKAPT
O09044	RTKNFESGKNYKATWGDGGDNSPSNVVSKQPSRITNGQPQQ
Q9BUA6	RLERNGMISAHCNLCLTGSSNSPASASQAFTIMDQNRDGFI
P41273	DDPAGLLDLRQGMFAQLVAQNVLLIDGPLSWYSDPGLAGVS
Q8CCX5	KKEEKPTKSKVGFLLPSAIINEISFSTKVSQKYENENMETV
P54130	TYSSNLYKHVDTGRRYYVALNKDGTPREGTRTKRHQKFTHF
P23409	RRRLKKINEAFEALKRRTVANPNQRLPKVEILRSAISYIER
Q9Z2U1	LKEAIKSSLIIILKQVMEEKLNATNIELATVQPGQNFHMFTK
Q9CY24	ISATAIFLILVSACILRFGTNSFCNSIISLNLTISCSEAQK
P68265	AALYAAPYKSDFLKALSKGQNVTEEECLEKVRLFLVNYTAT
Q27956	PITPVNATCATRHPCPSNLMNQIRNQLGQLNSSANSLFILY
P01193	DLSLETPVFPGNGDEQPLTENPRKYVMGHFRWDRFGPRNSS
P01033	IRFVYTPAMESVCGYFHRSHNRSEEFLIAGKLQDGLLHITT
O35257	IITELSYMEQAPDEIISRARNIEEKIIVLIEALRGILSKIQ
P21583	KPFMLPPVAASSLRNDSSSSNRKAKNPPGDSSLHWAAMALP
Q147U7	LPDLVRGLPTLASVLRRKVKNKRVRVVWESILEECGLQEGD
Q9P0V8	AQLHSNLSLELGPLESGDSGNFSVLMVDTRGQPWTQTLQLK
Q99390	KCIVSQLAFADGDVLVTGEMNESERASFLSTLNKMAEKKKN
P31098	DPEQTDDLDDDDDNSQDVNSNDSDDAETTDDPDHSDESHHS
Q3ZBR5	PEDDMNPSELDEEYLMELEKNMPDEEKKRRREESSRLKEEG
Q8BX35	RCQTCITCAVINRVQKANCTNTSNAICGDCLPRFYRKTRIG
Q864V4	SAADTVWLVVAFSNASRVFQNPQTLAEIPASPRLLTDGHYM
P97382	EAYSMARQFNLIPPVCEQAENHFFQREKVEMQLPELYHKIG
A0PJW6	AVAAVSRPPVPVQPLDAEVPNRGPFDLRSALWRYGLAVGCG
Q9CQX5	CMSFTLNEQFMEKYVDPGNHNSGIDLLRTYLWRCQFLLPFV
A6NGA9	VQFLVIKNGLELYAGLWTLCNHELCWSHTPKPPYYLQYSRA
P52955	APGEERRRSPLDHLPPPANSNKPLTPFSIEDILNKPSVRRS
P83626	CSRTADRVCQCKQGTYCDSENCLERCHTCSSCPDGRVVRKC
Q3T113	LLSGNDFQTAEEGSDVKLVCNVKSNPQAQMMWYKNNGILNL
Q9D413	VPLPRTSGLPKSVAGYQEARNGAMDGALKAGRRLSASSIAP
P62080	EYWQCCGAFGADDWNLNIYFNCTDSNASRERCGVPFSCCTK
Q6ZRC1	ELHRHGLGNLLLSELAQNMLNDVALAEYTATFLAPGVPETS
P20490	GIAMLILNLTNNFAYMNNCKNVTEDDGCFVASFTTELVLMM
Q6P3D7	SENRNYMAIINPYPHVRGNANYYGMSPTENPLYDWRGVTNG

Q9CY57	LRGGRATRTLLRGGMSLRGQNLLRGGRAVAPRMGLRRGGVR
A0JN86	DKDYHRSDPQIAICLDCLRNNGQSGDNVVKGLMKKFIRCST
Q91XQ6	TLKGYLISCVWSCYRYINGRNSSDVLVYVTSNDTTVLLPPY
P70195	DGIVLGADTRATEGMVVADKNCSKIHFISPNIYCCGAGTAA
Q3T0S6	ETKKTRVKLPSGSKKVISSANRAVVGVVAGGGRIDKPILKA
Q9CQE1	EGVYELATFQMKPGGPALWGNAFKRAVNAHVELGYSTLVGV
O43186	LFAKTQYPDVYAREEVALKINLPESRVQVWFKNRRAKCRQQ
P01910	TWLRNSKSVTDGVYETSFFVNRDYSFHKLSYLTFIPSDDDI
Q91WR8	CGLTATYPELNTLQEELKPFNVTVLGFPCNQFGKQEPGKNS
B2RPK0	GEHPGLSIGDVAKKLGEMWNNTAADDKQPGEKKAAKLKEKY
Q04744	SRSEDPIRPAALSYANSSPINPFLNGFHSAAAAAAAGRGVY
Q8WTZ3	DYKHEPLSPVECGKVFNKLSNHTGEKLYKPKRHDSALENTL
P0C2W7	LYLVHSLLRRLYHNDHIQIANRHLSRLMVGPHAAVPNLWDN
Q02878	TKEKKPEAKKVDAGGKVKKGNLKAKKPKKGKPHCSRNPVLV
Q9NVA1	WEDVQQRGRVMGVNPYILKKNMILMTNHFYAAILGYDEGIL
O02769	SMAPRIPEWLFVASYYMGYFNSCLNAIIYGLLNQNFRQEYR
Q8BYH0	GRTVGPTGPFFPSCLPGTLLNTATYAQALSHVASLKGGPLC
P30042	VPQMHVIDHTKGQPSEGESRNVLTESARIARGKITDLANLS
P46738	LGATRVVYNPASSGETLTVINDQDYPMLVQSEVLSEDQKSP
Q8C3X2	SIRKDMVILEKSEFANLRAENEKMKIELDQVKQQLTNETSR
Q8WXJ9	DPCELLHLCRLTIRNQLLTNNMLPDGIFSLLIPARLQNYLN
Q3LI81	SSQCQCQSQNPESSSCRPLVNVAPEPQLLESSPGVEPTCCV
Q32KV0	LPVVRTWRLNERHYGGLTGLNKAETAAKHGEEQVKIWRRSF
P97765	IEFGQRMLQVASQASRGEVPNGAYGYPYMPSGAYVFPPPVA
Q61999	YKLYCLRPSLRSLERLRRTTNRILASSCCSSNILGSVNVCG
D3Z3K2	VFGFPPGNNSKFSLDHIPVGNQGGGDEDVQFRPFFVCSPTA
Q5XG99	TFQGPAVVCGTPTSHVYMFKNGSGDSGDSSEEESHRVVLRP
O60671	INTQEPEETLDFDFCSTNVINKIILQSEGLREAFSELDMTS
Q0III0	CEGTSQPEPSCSAVRITANKNYRSKTSQEGALKKMHEEEHH
Q3V1I0	YGDIMTMETFGAPCDINNLMNCGIHGSEMFAEMDLKAIKPY
P18440	LQTPDGVHCLVGFTLTHRRFNYKDNTDLIEFKTLSEEEIEK
Q9H9Z2	AARAADEPQLLHGAGICKWFNVRMGFGFLSMTARAGVALDP
Q91YJ3	LKAQPKQTACWDGVRNYQARNFLRAMKLEDEAFFYHSNCKQ
P62917	RIDKPILKAGRAYHKYKAKRNCWPRVRGVAMNPVEHPFGGG
P27348	DTLNEDSYKDSTLIMQLLRDNLTLWTSDSAGEECDAAEGAE
P07203	LGFPCNQFGHQENAKNEEILNSLKYVRPGGGFEPNFMLFEK
O15260	AIGFKTKLAALTLVVWLFAINVYFNAFWTIPVYKPMHDFLK
P39039	VKSLCTELQGTVAIPRNAEENKAIQEVATGIAFLGITDEAT
Q9Y224	NFIVWLEDQKIRHYKIEDRGNLRNIHSSDWPKFFEKYLRDV
A2VDR2	SPSQAMQEYIAVVKKLDPSWNPQSPEKKGKEANTGFGGPVV
O14669	FLGPPEAQSFLSSHTRIPRANHWDLELLTPGNLERECLEER

Q9Z260	TIMPQWRVSAFIESNIVVFENRWEGLWMNCMRHANIRMQCK
Q9D9J2	SRSPEQRTVPLSKKDSVIPENIRHKFGSKMVDQLISEDQAR
Q8IUK8	IFVAPRKGIYSFSFHVVKVYNRQTIQVSLMQNGYPVISAFA
P11912	YLRVRQPPPRPFLDMGEGTKNRIITAEGIILLFCAVVPGTL
P68543	EVENKNNLSAVPLNNLEPITNIQIWLANGKRIVQKFNITHR
Q7L2Z9	ETLKVPPKKMEDLTNVSSLLNMERARDKANEEGLALLQEEI
Q99JL1	DGVLVAELIKFYFPKMVEMHNYVPANSLQQKLSNWGHLNRK
P62821	TTAKEFADSLGIPFLETSAKNATNVEQSFMTMAAEIKKRMG
O95372	AGIVALSCWLPLHRAFPQAANGSAKDLAILQCHGELDPMVP
Q9DA08	AVEGDEQWILAEVVSYSHATNKYEVDDIDEEGKERHTLSRR
Q497N7	KMPKNYAQPDVLNHTFDLLSNLHKLLPNHLVEVLHSYRSEE
P01915	LLVCSVSDFYPGNIEVRWFRNGKEEETGIVSTGLVRNGDWT
P51164	GLADPNFGFEEGKPCFIIKMNRIVKFLPSNGSAPRVDCAFL
P20959	TEYGPCRREMEDTLNHLKFLNMLSPRGIHIPNCDKKGFYKK
P55064	IYFTGCSMNPARSFGPAVVMNRFSPAHWVFWVGPIVGAVLA
Q9CQY8	STVTCEFSLKNLSKFDPESFNLLWFFNGTCVSPTDFKNPTI
P51122	GGLEVLAEKCPNLTHLNLSGNKIKDLSTIEPLKKLENLKSL
P40617	DVERMEEAKTELHKITRISENQGVPVLIVANKQDLRNSLSL
Q6IR37	PLARALQLALAAYQLLNLLGNVVLFLRSDPSIRGVMLAGRG
Q1RMT9	ARSEAAAVQPVIGISQRVRVNSKEKKDLGTLGYVLGITMMV
P51153	EAFSSLARDILLKSGGRRSGNGNKPPSTDLKTCDKKNTNKC
Q9C004	LTILPIDQVKTSHVENDYIDNPSLALTTGPKRTRGGAPELA
Q3U6N9	PLRSSVQPACDSAAGTHPVGNTVAMKQKKKKTPNRVSGTNG
A6NLW8	KQKLALEINTEESRIQIWFQNRRARHGFQKRPEAETLESSQ
P56966	YKYLLQLPGKQVRTKLSQAFNHWLKVPEDKLQIIIEVTEML
Q02013	IGLSVALGHLLAIDYTGCGINPARSFGSAVLTRNFSNHWIF
Q9WVS0	KNPMLCLYHLSNNSVSFFLNNPDSSQGSYYFCSLSIFDPPP
P50221	RSLQPPAPVWGCLRNPHSEGNGASGLPHYPPTPFSFHQKPD
Q32KM6	WKEWDQKAQKNGLRHQVFAVNGDHYVGEWKDNVKHGKGTQV
O18968	MVLVVAAESVWGDEKSSFICNTLQPGCNSVCYDHFFPISHV
Q92988	PLPSLWDLPKAGTLPTSGYGNSFGAWYQHHSSDVLASPQMM
Q8K3K7	AIQAQVPIIPVVYSSFSSFYNVKTKLFTSGTIKVQVLDAVP
Q3ZBK8	RLQSDFAAFLVGPLQRNSLCNLLSFTYKVKPEGQSFPFCAR
Q2TUM3	AENQAGVRYISIKPDNRKLANGTNVLGLLVDTLLKEGFHLV
O89104	VCHGEEAVCSAGATPSMGLANLSVLFGFINFFLWAGNCWFV
Q03287	EELAIRHKNLDIVFVESGGDNLSATFSPELADLTIYVIDVA
Q13588	LGAFLIRESESSPGEFSVSVNYGDQVQHFKVLREASGKYFL
Q9P126	DGSVISENMFEFLEDGKGNMNCAYFHNGKMHPTFCENKHYL
Q9BRX8	YGPQRRKMMFMGFIRLGVWYNFFRAWNGGFSGNLEGEGFIL
A6NDR6	KKQLAQDTGLTILQVNNWFINARRRIVQPMIDQSNRTGQGA
Q9NYK6	DSQVPKYSAKWIDGSAGGISNCTQRILEQRENTDFGLSMLQ

Q9NQ35	VSCQCAGKDVKALVDTGCLYNLISLACVDRLGLKEHVKSHK
P97376	EISGTIAIEMDKGAYIHALDNGLFTLGAPHREVDEGPSPPE
Q8R0W6	ALAALAAVEPACGSGYQQLQNEEEPGEPEQTAGDAPPPYSS
P61247	QQVRQIRKKMMEIMTREVQTNDLKEVVNKLIPDSIGKDIEK
P40617	IVILGLDCAGKTTVLYRLQFNEFVNTVPTKGFNTEKIKVTL
Q2KI11	QDVSITVLNVTLNDSGLYTCNVSREFEFEAHRPFVKTTRLI
Q3MHY7	LMKVFPNQILKPFLEDSKYQNLLPLFVGHNLLLVSEEPKVK
Q9UK85	LLRGIDSLFSAPMDFRGLPGNYHKEENQEHQLGNNTLSSHL
Q8N9W6	ESDLRKFFSQYGSVKEVKIVNDRAGVSKGYGFVTFETQEDA
Q60931	GNLETKYKVCNYGLTFTQKWNTDNTLGTEISWENKLAEGLK
Q9Y277	YQKVNEKIETSINLAWTAGSNNTRFGIAAKYMLDCRTSLSA
Q6NVG5	DVSHTEADDDRILYNLIVIRNQQTKDSEEWQRLNYDIYTLR
Q497N7	QSMSGGSMFQCSWQHRFMAKNKTWSRKCTSKVHLEKRESTV
P39905	SGSCDAAETTYDKILKNLSRNRRLVSDKVGQACCRPIAFDD
Q9QY36	KYYFYHGLSWPQLSYIAEDENGKIVGYVLAKMEEDPDDVPH
Q14696	GSPTEKETEEITSLWQGSLFNANYDVQRFIVGSDRAIFMLR
P04390	DFVEGKGIFDSEDEFLDYWRNYERTSQLRNDKYNNISEYRN
Q9JHW2	ELAQIYAQRGCQLLVYPGAFNLTTGPAHWELLQRARAVDNQ
O02691	DVQAALTLAREKFGRVDVAVNCAGIAVASKTYNLKKSQAHT
Q3SZG6	LRRSLENRDAQTRQLQDAVTNVEKHFGELCQIFAAYVRKTA
Q58DS5	YRGAMGIILVYDITDEKSFENIQNWMKSIKENASAGVERLL
Q9EP52	ECMLCLGALWDECCDCVGMCNPRNYSDTPPTSKSTVEELHE
Q5ND56	VSLARIMIQLKQQHTLLYKVNGILTVTTFLFCRILLFPFMY
P13760	LAGDTRPRFLEQVKHECHFFNGTERVRFLDRYFYHQEEYVR
Q6I9Y2	CEFSMGKTLLVYDMNLREMENYEKIYKEIECSIAGAHEKIA
Q8VI88	RRSLEHRRNSLLPFQWKATNNSRWMAQVVASEFSLVAFLLL
Q00688	GDVVHCWYTGTLQDGTVFDTNIQTSAKKKKNAKPLSFKVGV
Q9H0R3	LCCDNCHSHVALALNLMRYNNSTNWNMVTLCFFCLLYGKYV
P23889	LIPFFIFSSISKRSIGAIEANQGLFNYRPVKPIDTIIARAL
P07361	TNETLSWLSDKWFFIGAAVLNPDYRQEIQKTQMVFFNLTPN
Q9N1R0	TMGYMPTEMELIELSQQINMNLGGHVDFDDFVELMGPKLLA
P70677	YLDSSYKMDYPEMGICIIINNKNFHKSTGMSSRSGTDVDAA
Q0VD07	LAGAGAAVIAMVHYLMVLSANWAYVKDACRMQKYEDIKSKE
Q9NXJ5	EQPRKAVVVTGFGPFGEHTVNASWIAVQELEKLGLGDSVDL
Q3ULM0	FEEAPHLDSQILYRLSPSRRNVEEPPEGASPTLALMSSVKA
P07766	ELEQSGYYVCYPRGSKPEDANFYLYLRARVCENCMEMDVMS
Q96Q83	WHPVLRTLKNRIEENTGHTFNSLLCNLYRNEKDSVDWHSDD
Q8N7R0	SNGVTQGCLVNPTGNLPMWSNQTWNNSTWSNQTQNIQSWSN
O43633	FVLMRANIQAVSLKIQTLKSNNSMAQAMKGVTKAMGTMNRQ
O60930	DTFSYMGDFVVVYTDGCCSSNGRRRPRAGIGVYWGPGHPLN
Q03402	VIQGWYNESKGLIFGVGPKQNVSVVGHHTQVVWKSNLQVAC

Q8BQN6	LSGGSPFACKVCGKLFSHKSNLTEHEHFHSREKPFECNECG
Q6IMH0	STYFSKDDFNKAHFTLLGVPNKPLQCLDFTATGQKLCHKYR
Q9NP50	RAGPSLKTTLKPKKVKTLSGNRIKSNQISKLQKEFKRHNSD
Q5E9C8	VRESLPELQIEVIADYEVHPNRRPKILAQTAAHVAGAAYYY
P20334	PVCKSCPPSTFSSIGGQPNCNICRVCAGYFRFKKFCSSTHN
Q6PFR5	YTTERDLREVFSRYGPLSGVNVVYDQRTGRSRGFAFVYFER
Q9NPA1	EEAVQINPKCFYTPKCHQDRNDLLNSALDIKEFFDHKNGTP
Q47427	KLTQAETTQLNSVLDYIDELNAMDLTTAPDLNWPEKQLSTA
O18870	TIASIDFKRETCVVVYTGYGNREEQNLSDLLSPTSEVANIE
Q9CR00	SPASIAGLQVDDEIVEFGSVNTQNFQSVQNVGTVVQHSEGK
O75323	GTMIEWGNYWARAIRFRQDGNEAVGGFFSQIGQLYMVHHLW
Q58DC7	QVKELSWGVALDKNFPRASINFDYILAADVVYAHPFLEELL
P33783	KLEGAVTSTRNPWVWKVGQGNESLEVKQSRGVRDGEQGIPV
P50221	EAEFAHHNYLTRLRRYEIAVNLDLSERQVKVWFQNRRMKWK
O35943	KQIWLSSPSSGPKRYDWTGKNWVYSHDGVSLHELLARELTK
P20615	EFLFNMYLTRDRRHEVARLLNLSERQVKIWFQNRRMKMKKM
Q3B7M5	CLDKFWHKACFHCETCKMTLNMKNYKGYEKKPYCNAHYPKQ
Q32KL2	ADCSFWERLLARQCRIYELRNKERISVAAASKLLANMVYQY
Q969J5	SCSMKSSHQKPSGCWQHISCNFPGCRTLAKYGQRQWKNKED
Q6Q8B3	SSCMGGKQMTQNYSTIFAEGNISQPVLMDINAVLCCPPIAL
Q8VHX2	DQDLLDTIRIKLDPCHPTVKNWRNFASKWGMPYDELCFLEQ
Q9Y337	NLMLIKLNRRIRPTKDVRPINVSSHCPSAGTKCLVSGWGTT
Q1LZB9	KTDVSQAECCASGNIDTAWSNFTHPGNKISLLGFLGLVHCL
Q810P2	RVMDPNPAPNQYQLPVTLGTNIPVFRAAPSYSLASTNKNWF
Q6P5C5	GFFRTLCGQPQVFFRHCFVHNLCPLLFLAPSGRNLTPAELP
P23356	AQEGQCRVDDKVNFHFILFNNVDGHLYELDGRMPFPVNHGT
P16562	YTQLVWYSTYQVGCGIAYCPNQDSLKYYYVCQYCPAGNNMN
P12544	KGDDVKPGTMCQVAGWGRTHNSASWSDTLREVNITIIDRKV
Q3UP87	PSVLQELNVTVVTNMCRRRVNVCTLVPRRQAGICFGDSGGP
O08992	MPMVSGAPAQGQLVARPSSVNYMVAPVTGNDAGIRRAEIKQ
Q60948	RKKTKTAGLVRKGPNNRSSHNELEKHRRAKLRLYLEQLKQL
P10648	SLLTPFPLLKAFKSRISSLPNVKKFLHPGSQRKPPLDAKQI
Q01081	KFRREEDAEKAVIDLNNRWFNGQPIHAELSPVTDFREACCR
P51164	EDSINCTSEQYFFQESFRAPNHTKFSCKFTADMLQNCSGLA
O02659	PFNEVKTLCAQFQGRVATPMNAEENRALKDLVTEEAFLGIT
Q969U7	PAVSVGNVGQLAMDLIISTLNMSKIGYFYTDCLVPMVGNNP
P17918	MSKILKCAGNEDIITLRAEDNADTLALVFEAPNQEKVSDYE
Q8CCA0	IWPLFPVFHQFLEQSKYKVINKDQWCNVLEFSRTISLDLSN
P58417	KFKKMFGWGDFHSNIKTVKLNLLITGKIVDHGNGTFSVYFR
O89051	TYLPQSYLIHEHMVITDRIENVDNLGFFIYRLCHDKETYKL
Q3T024	GLDSDIPESDTEAYLSSLKENAAARKNGMIGLSDFFVLKRK

P54709	FYGFLAALFSFTMWVMLQTLNDEVPKYRDQIPSPGLMVFPK
Q3UTB7	REHFPFFRTAPEAWKNTVRHNLSFRDSFEKVPASRQGGAST
A8WH74	SIEDVRNQCTNHGADMISIHNEEENAFILDTLKKQWKDPAD
Q7Z5W3	QRLRLLPPELLRQLFPESPENGPILGLDVGCNSGDLSVALY
Q6XJV6	IKPRGQPSCIMAYKVETKETNETCLGRNITWASTPDHIPDL
P60880	NDARENEMDENLEQVSGIIGNLRHMALDMGNEIDTQNRQID
P27144	DRLSRRWIHPPSGRVYNLDFNPPHVHGIDDVTGEPLVQQED
Q2NL24	LKFVSEGDNIPDALGLVEYLNEWLQIIKPHCEDPTASSLPW
A6NC51	PNQLILWTGLLCALGTSVVGNFQEKNQRPTHLAGAFLAFIL
P39687	ELRNRTPSDVKELVLDNSRSNEGKLEGLTDEFEELEFLSTI
O55239	QLLSACESFTEIIVSDYTDQNLWELQKWLKKEPGAFDWSPV
Q3T0P5	HDVPVIPLDVVDHRTDTPDANLTQVDAASVYTLPAGADFLM
Q9JI58	NISDSYFFTFYTEIMSWRSANDESGVIMNKWKDDGEFVKQL
Q6P1N9	NGMFFSTVGCHPTRCGEFEKNNPDLYLKELLNLAENNKGKV
Q2KIS7	IAYKNWETEITAQPDGGKVENCATLSGAANGKWFDKRCRDK
Q96BF3	AIVWGAWFWGRRSCQQRDSGNSPGNAFYSNVLYRPRGAPKK
Q9Y224	DRQEAIDWLLGLAVRLEYGDNAEKYKDLVPDNSKTADNATK
Q8BVI4	RCVQAFRARNWWVASIDVVENEEASASVVVKMTDSFTEQAD
P0A9Z7	ADKTGASERGARGIVALLGPNNKAERIVVIYLRDTPASMAE
P18181	SGPFPKKSPGYVLDLIVTPQNKSTFYTCQVSNPVSSKNDTV
P53518	FNNIVEKLKWHKNGKYLVLKNNTPYYISFSEVFFDSDKVNN
O75431	QILLSDNAASLAVQAFLQMCNLPIKVVCRANAEYMSPSGKV
Q3T013	SLPPPAGLNSSWVELPMNSSNGNDNGNGKNGGLEHVPSSSS
O43561	NVPESGESAEASLDGSREYVNVSQELHPGAAKTEPAALSSQ
O89104	LYQVQYEMPLCDQDSTSKTMNLMGDFSAPAEFFVTLGIFSF
P0CG22	ALLGLNKTLAIELAPRNIRVNCLAPGLIKTSFSRMLWMDKE
Q9D7B7	LRELHKEFGPYHFNVLAFPCNQFGESEPKSSKEVESFARQN
Q0P5E7	FPLEELHEHFVPGSRPDLHLNIFHPSLEDIARAESFFTASA
Q6PAM0	FKSPPILPPHLLQVILNKDTNISCDPALLPEPNHVMLNHLY
Q6PF18	RSGWGRMYYSNGDIYEGQWENDKPNGEGMLRLKNGNRYEGC
P47753	ITHAPPGEFNEVFNDVRLLLNNDNLLREGAAHAFAQYNMDQ
Q8BGB7	HKVDSESYRKIADSIGCSTNNILFLTDVTVEASAAEEADVH
P20160	DIVGGRKARPRQFPFLASIQNQGRHFCGGALIHARFVMTAA
Q3T0Y3	DVLRKNLKLIHGCPITYAFANNWEKIEQFQSRPDDIMIVTY
Q8NDY6	GSAADGRRRPREQRSLRLSINARERRRMHDLNDALDGLRAV
Q9Y337	MFQGVKSIPHPGYSHPGHSNNLMLIKLNRRIRPTKDVRPIN
Q8NEG0	FYLQLCPPSDASEDLFVHWENLVYILRPPVEAYSDTRAILA
Q7M3E1	AENGNWDVRGIVSFGSGLSCNTFKKPTVFTRVSAYIDWINQ
Q9HCN8	THVLTGKNLHTHHFPSPLSNNQEVSAFGEDGEGDDLDLWTV
Q32KY9	QSQTNFICPITQLEMKKPVKNKVCGHTYEEEAIVRMIESKH
P82970	AVAAEVKNEEEDQKEDEEDQNEEKGEAGKEDKDEKGEEDGK

Q9H063	DFSTARSHEFSREPSLSWVVNAVNCSLFSAVREDFKDLKPQ
P0C866	VQSTGEREAQRYFEHALTLRNTTLFLRHNKDLVVQTAQPDQ
Q9H7J1	IGGALLFALRYRVTGHEFWDNNGGRDYALRGPEHPGSGGAP
P33792	IIKNNTNKVMDVWADYCGSYNNNKCRVQLITRPYSEKKIEI
Q9NXJ0	TAHFANQANTTTNMSVLVIPNMYESNPVTPASSSAPPRCNN
Q9XT56	SSPRVEWKFTHGDIRGLVCYNNKITASYENRVTFSDTGITF
Q3UZZ6	IPEEILNKILYHSSFSVMKENPSANYTTMMKEEMDHSVSPF
Q96B33	PKPKPKVGFPMPRPRPKAYTNSVDVLDGEGWESQDAPSCST
Q91V08	EQIPVNKTYAACPQNWIGVENKCFYFSEYPSNWTFAQAFCM
Q9JJZ6	RRDGKDSASLFVVARILADLNQQAPAPAPAERREGAAARKA
O95749	DIKKYCVHYLEDVGSFEYTRNTLKELEAKAYKQIDARGGNP
Q9WV93	AFGHHPHIAHPLLLPQNGHGNAGTAASPTEPHHQGRLASAH
Q5SX19	TMLGLARLEQQVSLLCFSSGNYYNQGEIRKKELLQSCAVLG
A2RRY8	NSQTEDGQRPEWTFYPRFSSNIHTYHIGKQCFFNGVFRGNR
Q96S21	LPLPVTIKSHLKSFSMANGMNAVMMHGRSYSLASGAGGGGS
P70280	LFAVVARGTTILAKHAWCGGNFLEVTEQILAKIPSENNKLT
P53805	SPPASPPVGWKQVEDATPVINYDLLYAISKLGPGEKYELHA
C9J442	EPVQGLLSQNHSCRDPQCCGNLLVLCLFLVWQVRHCWHQVT
Q00322	DKAKRRNQEMQQKLVELSAENEKLHQRVEQLTRDLAGLRQF
P62079	DFTQEYWQCCGAFGADDWNLNIYFNCTDSNASRERCGVPFS
Q9JL95	QRVCRRCYRGNLASVHSYSFNYQIQNLARKINQSIVWIGGI
Q29461	GTILPNNYVCYVTGWGRLQSNGALPDILQQGKLLVVDYATC
Q9H6Z9	KIALEYIVPCLHEVGFCYLDNFLGEVVGDCVLERVKQLHCT
Q5I3B2	DAPNSSVVHVSSPEGGDTSGNGAQEKTVDGTECHLLDFASP
Q99KL7	IKLNKAEIEQSQRVVKADIVNYNQEPLSRTVNPPRSSMCAV
Q9HD33	RLLHTTLSRKGLEEFFDDPKNWGQEKVKSGAAWTCQQLRNK
Q8N128	AAAAAFGESAGQMSNERGFENVELGVIGKKKKVPRRVIHFV
Q3TB92	VDCTRVENNELPSPNLNSSMNVVRMGQNVSLSCSTKNTSVD
P97801	DICETPDKPKGTARRKPAKKNKSQKKNATTPLKQWKVGDKC
Q9NY30	SHWHSDCPSKGQAFRCIRINNNQNKDPILERACVESNVDFS
Q8NFX7	EGSTSFVRRSQWMLEQLRQVNGIDPNGDSAEFDLLFENAFD
Q8BK26	PVMIQQKSDAKWSEVSHTFSNYPPGVRYIWFQHGGVDTHYW
Q9NZQ0	SFDALDAWLAEMKQELGPHGNMENIIFVVCANKIDCTKHRC
Q0VCS6	KSWQGCEYFCIAENSTMLKINTQEVLEFAMPQSYSEFFYSY
Q47456	TGRYLQQGLVEEGYQADLFNNGRDGLGAASKGQYDLIILDV
Q9Z2B3	LDYSRGDRNFISFLFTMAFFNFLVPLFITHTSYRFMEQKFS
Q9GZV9	PQYHFLVSLGRAKRAFLPGMNPPPYSQFLSRRNEIPLIHFN
P47962	NRFGMDKIYEGQVEVNGGEYNVESIDGQPGAFTCYLDAGLA
P27701	NSLSVRKGFCEAPGNRTQSGNHPEDWPVYQEGCMEKVQAWL
Q7Z5L3	KSPHEGYEVLKFDDVVTNLGNHYDPTTGKFSCQVRGIYFFT
Q2YDP3	FDFLKDLVASVPDMQGDGEDNHMDGDKGPRRGRKSGSSGRK

Q9NXV2	RDPTYFGPVLNYLRHGKLVINKDLAEEGVLEEAEFYNITSL
Q99LU8	KQFLHVLSRKDKTGIVVNNPNQSVFLFIDRQHLQTPKNKAT
Q5E9Q4	ALQDHFKWVKQQCEFAKQAQNPDQGPKLDLSFKEGQTIKLN
Q8WW62	FARMRKMADFFLIQSNYNYVNWWSTAQSLVIILSGILQLYF
Q6PZD9	PGSYDPRAVAVKEEPRGPEGNRGTSRGSYNPLQYQVAHCGQ
Q9UKR0	VLGLSQAATPKIFNGTECGRNSQPWQVGLFEGTSLRCGGVL
Q45KJ6	ERRPKGKTLQKRKPKGDRCYNCGGLDHHAKECSLPPQPKKC
Q2KID0	GDIVVGRITEVQQKRWKVETNSRLDSVLLLSSMNLPGGELR
Q91WK7	ADPNQQDGLGNTPLHLAACTNHVPVITTLLRGGARVDALDR
Q9BXJ2	VTSFAICASGQPRGNQLKGENYSPRYICSIPGLPGPPGPPG
O15305	KIKIGVVGGSDFEKVQEQLGNDVVEKYDYVFPENGLVAYKD
Q61955	SRDQPEQEIQVAQSIQHPCYNNSNPEDHSHDIMLIRLQNSA
Q8VI88	EEESERIRTSRNRRSLEHRRNSLLPFQWKATNNSRWMAQVV
O54830	IAELLEDSKSILSQAYGATENVADYTLWSGLEDLQSSDEET
O43402	NTKFTMDCVAPTIHVYEHHENRWRCRDPHHDYCEDWPEAQR
Q9TVC8	RQVEPTVTIFLSRTEALNHHNLLVCSVTDFYPGQIKVRWFR
P78346	KALRGLVETAAHLGYSVVAINHIVDFKEKKQEIEKPVAVSE
Q9Y2B4	RNRLRTVLKNLSLLKLLKSSNRRIQELHKLAKRCWHSLLSV
Q96HS1	YIVCRALQFPPEGWLRLSLNNGSITHLVIRPNGRVALRTLG
Q8BGB8	RLRTQSLQVLFSELIPWAIQNGRRAPCVLNIYYEQRWEQPL
A8MUZ8	LQLEQGKELWREGRVFLQDQNPNRESALKKTHMISMHPITR
P54846	LRGCGRDEALRLKQRRRTLKNRGYAQACRSKRLQQRRGLEA
Q96DX8	FQELIQEAKPRATWTLKLDGNLQLDCLAQGWKQYQQRAFGW
Q3T112	WDKKGPGLYYVDENGTRLSGNMFSTGSGNSHAYGVMDSGYR
Q8BPB0	MLPEGEDLNEWVAVNTVDFFNQINMLYGTITDFCTEESCPV
Q28145	VHATEGLDWEDKDATGTLVGNVVHSRIINPLRLFVKQSPVP
Q9CZQ6	ILHLNNINKTMTSGDPGKMANATGKCLTQPLNDLCQELRDK
Q6ZUJ4	SPQKATDADPGSLKQAFDDHNIVETVLDLEEDYNVMTSFKY
Q6UWN5	NADALPPDYSVVRGCTTDKCNAHLMTHDALPNLSQAPDPPT
Q9H741	CWPNGCCSAYEYCVSCCLQPNKQLLLERFLNRAAVAFQNLF
Q5SYH2	LHELLEGSDIYFPEIVKPPRNPELVARLEKIKIQLANEEYK
Q86Y82	PGPSGPQLRDFSSIIQTCSGNIQRISQATAQIKNLMSQLGT
Q96G27	AFRHRRAKLRLQQQQRQREINLLAYHGACHGAGPFPTGSLL
Q9D7W4	CGARGPNDWNLNIYFNCTDLNPSRERCGVPFSCCVRDPAED
Q91V37	LGFRFDVAWFLTETSPFMWSNLGIGLAISLSVVGAAWGIYI
Q9D198	ARKLNHQEVVEEDKRLKLPANWEAKKARLEWELQEEEKKKE
Q62447	QYVQDMGQEDVLLPLAWRIVNDTYRTDLCLLYPPFMIALAC
P51177	VPDNEEATQYVEAMFRAFDTNGDNTIDFLEYVAALNLVLRG
Q2HJF4	QDVRYSPDTKKLLVRLSDTYNRSFLESLTVNTENLLQVETT
Q8IUI4	DVYGNSSGRKHRGHSESPEKNGAHSVTQAGVQWHDLSSLQP
Q96LD8	CSDHVSFISPEVTQFIKCTSNPAEIAMFLEPLDLPNKRVVF

Q91XB7	YSSQLGGYPAPGADVAFSVNNLLGDPVANMAMAYGTSIASQ
Q8VCH9	VICKTSVLDEHAGRPFLNAANDADLCNLPKKTTDYAMLVTM
P28063	SRATAGSYISSLRMNKVIEINPYLLGTMSGCAADCQYWERL
Q9CQC3	TLRSHHKKYSEPVLVYSWHRNREAFPKDYDIESLETVKNLC
Q99075	ADLDLLRVTLSSKPQALATPNKEEHGKRKKKGKGLGKKRDP
Q96MH2	EEMFAKGQPVAPYNTTQFLMNDRDPEEPNLDVPHGISHPGS
P42270	CHNIDPETQRPRKVFDTISDNAANSGVILGGRPIKPDELDL
P12544	SVVVSLLLIPEDVCEKIIGGNEVTPHSRPYMVLLSLDRKTI
Q7YS81	EILRSAIQYIERLQALLSSLNQEERDLRYRGGGGPQAAVPS
Q9QYB1	KKYRNFDIPKGMTGIWRYLTNAYSRDEFTNTCPSDKEVEIA
P11911	LTVNLGEEARLTCENNGRNPNITWWFSLQSNITWPPVPLGP
Q9P0N9	TFKIKVMALNSAEKITKFLENIPQDSSDAIVSKAIDLWHKH
Q04741	QKLEEEGPESEQKKKGSHHINRWRIATKQANGEDIDVTSND
Q7Z6A9	CLRRHQGKQNELSDTAGREINLVDAHLKSEQTEASTRQNSQ
P08833	LAKAQETSGEEISKFYLPNCNKNGFYHSRQCETSMDGEAGL
Q9CRA9	TALSKRVEAMKQYQEEIQELNEVARHRPRSTLVMGIQQENR
Q75VT8	KAKAKNLQKQRERESCWAQINFTNTDMSFDNSLFAISTKMT
Q9Z1Y9	AYKQVKLGEDAPNSSVVHVSNPESGNNYASEKTADGAECHL
O89104	MGLANLSVLFGFINFFLWAGNCWFVFKETPWHGQGQDQGQG
P06837	PSEEKAGSAETESAAKATTDNSPSSKAEDGPAKEEPKQADV
Q9ULW5	ANGARPARSGTALSGPDAPPNGPLQPGRPSLGGGVDFYDVA
Q8N4T8	MIQQQGGSIVNVGSIVGLKGNSGQSVYSASKGGLVGFSRAL
P02662	VALARPKHPIKHQGLPQEVLNENLLRFFVAPFPEVFGKEKV
P01906	AVGKHTLEFMMRQSNSTAATNEVPEVTVFSKFPVTLGQPNT
Q3U1J1	KYQALGRRCREIEQVNERVLNRLHQVQRITRRLQQERRFLM
O95456	TDTTHLSSTEAFCVFYHLKSNPSVFLCQCSCYVAEDQQYQW
Q9UBS3	FKDFGFFGQNQNTGSKKRFENHFQTRQDGGSSRQRHHFQEF
P61087	AVQRIKREFKEVLKSEETSKNQIKVDLVDENFTELRGEIAG
Q58CY8	TDVFSATWNSVMITFGCCGVNGPEDFKYASVFRLLTLDSDE
P09237	LEAKLKEMQKFFGLPITGMLNSRVIEIMQKPRCGVPDVAEY
A5PKI3	GISKACPEKHFAFKMASGAANVVGPKICLEDNVLMSGVKNN
Q9CZD0	PSEHPSTTSELPLSLLTKPQNSRRARSWLSPSVSPPVSPGP
A4QPB2	DQLPDLMGLKAVNVDKVVGTNPHADRNGGAATCASSRPTQP
O14818	VTVEYITRYIASLKQRYTQSNGRRPFGISALIVGFDFDGTP
Q96EY5	KSFAQKSGYFLCLSSLGSLENPQENVVADIQIVVDKSPLPL
P12658	VDDTKLAEYTDLMLKLFDSNNDGKLELTEMARLLPVQENFL
Q16637	GNREEQNLSDLLSPICEVANNIEQNAQENENESQVSTDESE
Q86UP9	AAAAAAAMLPAQEAAKLYHTNYVRNSRAIGVLWAIFTICFA
Q9H741	ITDELGYVCERKDLLVNGCCNVNVPSTKQYCCDGCWPNGCC
Q8IXQ8	LLQHITIGTVLQIKVYRDFINIPEEWQEIYDLIPEAKFPVT
Q9WV68	EVMTAVDQALQEFGKINILINCAAGNFLCPASALSFNAFKT

F1MK05	VSLALIATLVYALFSRNAHQNIHPENQELVRVLREQLQTEQ
P21237	SYFGCMKAAPMKEVNVHGQGNLAYPGVRTHGTLESVNGPRA
P30793	AQPADGWKGERPRSEEDNELNLPNLAAAYSSILSSLGENPQ
P19652	LPLLEAQIPLCANLVPVPITNATLDRITGKWFYIASAFRNE
Q15014	KQLFQLPAKKNVDAILEEYANCKKSQGNVDNKEYAVNEVVA
A0JNI1	DEVRPHSAERKKRERGLGHANGEPLPTAGQEPARHDLSASD
Q80W21	RKHNLCGETEEERIRVDILENQLMDNRMVLARLCYNADFEK
P50236	AIYLMRNPRDILVSGYFFWGNTNLVKNPGSLGTYFEWFLQG
Q16763	VYKEVTTLTADPPDGIKVFPNEEDLTDLQVTIEGPEGTPYA
Q9NRD1	LPENILLELFTHVPARQLLLNCRLVCSLWRDLIDLMTLWKR
P09661	PVIENLGATLDQFDAIDFSDNEIRKLDGFPLLRRLKTLLVN
Q32KU6	DYRSDAVDKIQSMLHCCGVTNYRDWKDTNYYSEKGFPESCC
Q9H2W2	CAPGTETKCLKPQLPLEVDVNCLPEPNGVGGGISDSSSQGQ
Q9N0V4	YEERQYSVGDAPDYDRSQWLNEKFKLGLDFPNLPYLIDGTH
Q9R1P4	LLIAGYDDMGPHIFQTCPSANYFDCRAMSIGARSQSARTYL
P21658	RLYTTPSFHDECKFRETLLPNNYNAYESDLYRGTYIALSKY
Q62000	TLAENQLLRLPVLPPKLTLLNAKHNKIKSKGIKANTFKKLN
Q2M1V0	IFHFTHYPDVHIRSQLAARINLPEARVQIWFQNQRAKWRKQ
O95983	TMPITGQLSAAVEKNPGVWLNTTQPLCKAFMVTDEDIRKQE
Q8IWZ4	KLLKKMQSLWEKACENQRNLNVETTRISHWKAFGDILYRSE
P70378	ANPDGSIQGTPEDTSSFTHFNLIPVGLRVVTIQSAKLGHYM
Q66JS6	GGTAGGDRWEGEDEDEDVKDNWDDDDDENKEEAEVKPEVKI
P32243	LTPMHHQLPGPGATLSPMGTNAVTSHLNQSPASLSTQGYGA
Q86TL2	VDNFLMRKGKTKAKLEERGANQDSRNGSKVRYRRAASHEES
P0DMS9	PKYWCRGYFRDYCNIIAFSPNSTNHVALRDTGNQLIVTMSC
Q9D720	NHCYQVHDRNATVDKLEDFINNINSVLESLYIEIKKGVTED
Q80XC2	VLPQVYNVRTVSLPLPDLGANNLETNMGSDASPFRSGTPMK
Q9JJV3	LLGFTLMFWCEFTASFLFFLNAASGLHINSLTQPWDPPAGT
Q5TZF3	GSGKLLKEDKNTILSACRAKNEWLETHTEASINELFEQRQQ
P20181	LSDSTPLEPPPLYLMEDYVGNPVVANRTSPRRKRYAEHKSH
Q9D7W4	VVGGVMSVLGFAGCIGALRENTFLLKFFSVFLGLIFFLELA
P12544	ASWSDTLREVNITIIDRKVCNDRNHYNFNPVIGMNMVCAGS
Q08DJ0	NVTNKNDPKSINSRVFIGNLNTAIVKKVDIEAIFSKYGKIV
Q9DAK2	EALLPYYRQILPILNIFKNMNVNSGDGIDYSQQKRENIGDL
Q9R0P3	KANYRMYSYVTEELPQLINANFPVDPQRMSIFGHSMGGHGA
Q9WU63	DRAEMTVFVRSFDGFSSGQKNQEQLLTLANILREEGKVFNE
Q3ZBE1	ECRANREKMKGELGMMLSLQNVIQKSTTPGETKLLASEIYD
Q5UKY4	RIETTDGIFQERHSIQVPGENRTVVCEAIASKPAMQILWTP
Q3T0Z4	GVMAIPATTMSLAARKRACCNNKTGMFLSSLLNAITVIGAA
Q13162	HTLRGLFIIDDKGILRQITLNDLPVGRSVDETLRLVQAFQY
P28293	DSGGPLVCSNVAQGIVSYGSNNGNPPAVFTKIQSFMPWIKR

Q8IZR5	VNTGLSAFLFFIASIVLAALNHRAGAEIAAVIFGFLATAAY
O70255	DGNPERYDVSILLWKLQFDDNGTYTCQVKNPPDVDGLVGTI
P78369	GGVIFCFSISDNNKTPRYTYNGATSVMSSRTKYHGGEDFKT
Q96A05	AVNAAGGVEVYSGNQRIKVSNTLESRLDLSAKQKMPEIRMA
Q9NZQ7	PVEKQLDLAALIVYWEMEDKNIIQFVHGEEDLKVQHSSYRQ
Q8WVD3	PVTCPICVSLPWGDPSQITRNFVSHLNQRHQFDYGEFVNLQ
Q14002	GLLLTASLLTFWNLPNSAQTNIDVVPFNVAEGKEVLLVVHN
Q8TDQ7	WNNFFKHIDIDPNNAHILDGNAADLQAECDAFENKIKEAGG
Q9Y5K8	STTVIQNVNKAQVKIRAKKDNVAGVTLPVFEHYHEGTDSYE
P53518	SIRGCIKLIYQPASVPSPVFNNIVEKLKWHKNGKYLVLKNN
P49720	LYELKEGRQIKPYTLMSMVANLLYEKRFGPYYTEPVIAGLD
Q9CPZ3	PLKKKVLAYLSSISLEEWPGNTVSNTFCSEQKTDSLKELLV
Q0P5A4	RGVDNMVAINLIVQHIQDILNGDICKWHRAGANGRSHKRTF
Q8TCF1	SFKDCAERELVAVICPYCEKNFCLRHRHQSDHECEKLEIPK
Q9CR10	PCSLPPELEPPTNCCMSGCPNCVWVDYAEALLRLYQDGGEK
Q3T093	PPSSSVAISNHVTPPPIPKSNHGGSDADILLDLDSPAPITT
Q8WXB1	LCSNHSVILLACRIRYERDNNFLAMLERQFTVRKVHYDPEK
Q9D9S3	EMEMLFSMFPNQGEVKLEDVNALTNIKRYLEGTREALPPNI
Q3TT38	QSDENNLTDPGGSEFDSISKNTWAPVPEQSEQDQDRLSQSS
Q99MS4	RLQQVQVKIIDNSLCEEMYHNATRHRNRGQKLILKDMLCAG
P09237	EIMQKPRCGVPDVAEYSLFPNSPKWTSKVVTYRIVSYTRDL
Q3TR08	AVIGLFCRQYDIIKDNDSNNNPKEKGKGPEQSPQGRPVGTT
Q28918	HVRSMKGLQQQAVLAIGQELNRRALGGPAPAAWINQVRRRG
Q96BF3	GAWFWGRRSCQQRDSGNSPGNAFYSNVLYRPRGAPKKSEDC
P16563	DFTSLLTNQLQVQREIVNKHNELRRSVNPTGSDILKMEWSI
Q9H8M1	VVTLVKPHLVKASCTDGRLFNHLETIWRFSPGLPGYPRTCT
Q9CQ79	LDRHLAILAKKHLETKFLKLNVEKAPFLCERLRIKVIPTLA
Q8CGR5	SGWGTIASPIARYPTALQCVNVNIMSEQACHRAYPGIITSG
Q9ESN5	NHFPLPEASTPKKRKNIQDSNAQLITLNEILEMLINRMFDV
Q16560	YVPNKGVIGDPLLTLFVARLNLQTKEDKLKEVFSRYGDIRR
Q810N9	REMKSVERNRGQLWELQKLKNELLQELFTLQKKLKVLKDEE
P68252	ARRSSWRVISSIEQKTSADGNEKKIEMVRAYREKIEKELEA
Q8BXP5	NTALMLAAQAGHMSLVTLLLNYFAGLDLERRDQRGLTALMK
Q8IXQ9	GASRILANDIDPIAGMAITLNCELNRLNPFPILIQNILNLE
Q3URF8	VREAPQIYSLSSSSMEVFNANIFSTSCLFLKLLGSKLLYCS
P41317	QGLRGLQGPPGKVGPTGPPGNPGLKGAVGPKGDRGDRAEFD
Q5BVD1	PVDELELSVLERQPEENTPLNGADKVFPSLDEEVPPAEANK
Q2HXU8	LQKTIQQQQDNLSQQLGNSNNLSMEEEFLKSQISSVLKRQE
Q8BFR6	SFKGCTDVELVAVICPYCEKNFCLRHRHQSDHDCEKLEVAK
Q9TU47	GCRIIGRMCVGNRHGLLVPNNTTDQELQHIRNCLPDSVQIR
Q96LL9	PRTPPPTSRTHDGSRASPGANRTMFNFDAFYQAHYGEQLER

Q969U7	LNMSKIGYFYTDCLVPMVGNNPYATTEGNSTELSINAEVYS
Q62422	CHGGHKDIVEVLFTQPNVELNQQNKLGDTALHAAAWKGYAD
Q9NP50	LKTTLKPKKVKTLSGNRIKSNQISKLQKEFKRHNSDAHSTT
Q9GZN2	VALPRGSSPSVLAVSVPAPTNVLSLSVCSMPLHSGQGEKPA
Q16629	GELERAFSYYGPLRTVWIARNPPGFAFVEFEDPRDAEDAVR
P62826	SNYNFEKPFLWLARKLIGDPNLEFVAMPALAPPEVVMDPAL
Q8R1F5	VPQGADRAAVKGEMETVFVENLKHAAGVLAQENLVGLLEPI
P48755	RPCEQISPEEEERRRVRRERNKLAAAKCRNRRKELTDFLQA
Q6URK8	DGSTHVYASPAILLPMERQRNQLAGVKQQLYHPALPTLRHM
Q8WWF8	RIMDDDNNRTLDFKEFMKGLNDYAVVMEKEEVEELFRRFDK
Q9R0Q4	SQPKNLDPALPGRWGGRSAENPPSGSVRKTRKNKQKAPGNG
Q5HYI8	VKLSRFFDKVIEKRYFLREGNQIPGFPDRKRFGAGTLKSLH
Q8K2J7	NTGSHTLHSRAETTPSSPTNNPGNGHPEYIAYVLVPVFFVM
Q32KX8	WDWKQWGGILPQSLDLLVCINMSHISPLSCTEGLFRAAGHL
Q17QI5	IVYLCITDDDFERSRAFNFLNEIKKRFQTTYGSRAQTALPY
Q9CQQ4	RCSEVRLLVGSKDDERVPALNLLICLVSRYFDQRDLADEPS
Q3UPR9	AGQCQPTTRVRRRSVRQEPLNGGAPCPPLEERAGCLEYSSS
P14191	ENMAYTDGSVVSVAYALGIANGQTIEATFNQAVTTSTQWSA
Q8K2Y7	KNWGEEKVKSGASWTCQQLRNKSNEDLHKLWYVLLKERNML
Q8TAG5	LSSQAKFTEFPRNVTATEGQNVEMSCAFQSGSASVYLEIQW
Q3SYT7	LQAAAGMYEQLKGEWNRKSPNLSKCGEELGRLKLVLLELNF
Q8BFU0	ALIILNCMDYSQCQGNRWRRNKRASYVSNPICKGCLSCSKD
Q4G0X4	IFSTSCLFLKLLGSKLFYCSNGNLSSITSHLQDPNHLTLDW
O14613	TRTATVCGRELPDGPSPLLKNAISLPVIGGPQALTLPTAQA
F2Z333	VLLQWRLAPAAARRVRAFALNCSWRGAYTRFPCERVLLGAS
Q3T0X5	VDNHIGISIAGLTADARLLCNFMRQECLDSRFVFDRPLPVS
P51945	LGCVGLSCFYLAVKATEEERNVPLATDLIRISQYRFTVSDL
Q3T113	GITFTCKLQRNQSVSISVVLNVTFPPLLSGNDFQTAEEGSD
Q2HJH8	INPYLETTKLIYKDLVSVRKNPQTKKIQITSSIFKVTAYDS
Q17QW4	PGLAALPYLRLRHPLGVLGINYQQFLRHYLEHYPIAPGRIQ
Q9GZP4	GIIIMGEDDDSHPSEMRLYKNIPQMSFDDTEREPDQTFSLN
Q2HJ22	GGALYALCGLLALVPLCWFANIVVREFYDPTVPMSQKYELG
Q13277	IRKSQHSVLSRKFVEVMTKYNEAQVDFRERSKGRIQRQLEI
Q6PCP5	GGSAAATSNPHHDNVRYGISNLDAAIEGASDDMTVVDAASL
P01906	TLICLVDNIFPPVVNITWLSNGHSVTEGVSETSFLSKSDHS
Q08DU1	LYGAKLIAQKCQVRNCPHFKNAVSGSECLLSMVEDGNPHHY
Q2TJ95	FKRGTETRVRDILQHPSAKGNLCPPTSETRTCIVQRKKCSK
Q08E62	EGLGRCGSLESSDCESLDSSNSGFGPEEDSAYLDGVSLPDF
P09630	NSFYQEKDMLSNCRQNTLGHNTQTSIAQDFSSEQGRTAPQD
P62956	KGRCVASEYFLEPEINLVTENTENILKTVRTATPFPMVSLF
Q9H1R2	EVPIKKHFKECINFIHCCRLNGGNCLVHCFAGISRSTTIVT

P01189	MEVRGWCLESSQCQDLTTESNLLECIRACKPDLSAETPMFP
A0PK84	GQTRYQVRKGMAVRARPWRKNLQEVFGKRWLLGLLVPMFNV
A5PJN0	DAFFYHMLTKLGVDDAVKEENVQKMKQQFMAPHDVSKDGCI
Q3TT38	GTTDKDVLISEFQRLLGFQLNPAGCAFFLDMTNWNLQAAIG
Q9UKL6	YYFDNPGGQIPSWLINWAAKNGVPNFLKDMARACQNYLKKT
Q8IWL1	IQEACARAGGRIAVPRNPEENEAIASFVKKYNTYAYVGLTE
Q8N3J9	MRVHTGEKPYVCSECGRGFSNSSNLCMHQRVHTGEKPFKCE
P43346	TRIKKISIEGNIAAGKSTFVNILKQASEDWEVVPEPVARWC
Q9D1X0	EPELEAEATEGDEPDLEQEMNPEQEPEPEPEPEPEPEPEPE
Q8N801	IATYNFKNEGRKKPPLVQRNNPVLNDLPQYMPPDFLDLLKK
P56537	TVQIRRVEERLSALGNVTTCNDYVALVHPDLDRETEEILAD
Q32L77	CLNTKLAKIKSKALLNEETMNSGIIERDTGLPATGFGALFT
Q9D7V2	DTLQGIALKYGVTMEQIKRANKLFTNDCIFLKKTLSIPILS
Q9DCI3	DLLFVTLLWIIELNVNGGIENTLKKEVIHYDYYSSYFDIFL
Q96EL1	RGRSRQPLVLGDNCFADLVHNWMELPETGSEGGDGGGHRAR
Q9ULZ2	IRQEIDIPRIKHYKVMSVGQNYTIELEKPVTLPNLFSVIDY
Q8BVH9	LNVYKVLKPGRSVLFRDYGLNDHAMLRFKAGSKLGENFYVR
Q3UP87	RQTFSVQRIFENGFDPSQLLNDIVIIQLNGSATINANVQVA
Q9BZ81	LLYKFKMKQRILKEDMLKIVNPRYQNQFAEIHRRASEHIEV
P0CI00	HMISMHPIIRKDTSTSMTMENSLILEDPFEYNDSGEDCTHS
O43291	MVLILFLGASMVYLIRVARRNQERALRTVWSSGDDKEQLVK
Q96NT3	YQGHFIVLRGYNRATGCIFYNNPAYADPGMCSTSISNFEEA
Q8BHH8	NAESNHLSDKLSQKLYPDTTNKRTTHTYGYSFWLTLHVIFL
G3N131	VGKGTLVQTKGTGASGSFKLNKKVASVDAKPTATKVATKTK
Q9BXJ2	TVIYLQPEDEVWLEIFFTDQNGLFSDPGWADSLFSGFLLYV
Q3ZCC4	KQLALERRQAKLLSNSQSLGNDLSVNTPSTQTSEAGSTGEE
Q91W78	PEDDSAKHGGRIRTFPHERGNWATHIYIPYEAKEDFRDLLD
Q27956	EKARLVELYRIIAYLGASLGNITRDQKVLNPYAHGLHSKLS
Q64124	REPRQRHTANARERDRTNSVNTAFTALRTLIPTEPADRKLS
Q6PIL6	ESISAQLEEASSTGGFLYAQNSTKRSIKERLMKLLPCSAAK
Q3T024	HKQCSHKEYVDLADLEQKWKNLCLPVEKFRALLQLDPCEDK
Q86UD3	DCTSSAAPVVKTVEDCGSLVNGQPQYVMQVSAKDGQLLSTV
Q8IXM7	KITRFGMSSCPQVPMEERISNLRLNPTLASCQYYFEKIHPP
P51911	TKYGVKPHDIFEANDLFENTNHTQVQSTLLALASMAKTKGN
Q9JHL0	CSRPGVKRNEKIYEQRNRQENAQSSAAAQTYSLARQVWPGP
P62491	IGDSGVGKSNLLSRFTRNEFNLESKSTIGVEFATRSIQVDG
O35083	ILFLAILAIPVCAVRGRNVENMKILRLLLLHAKYLYGIRVE
A6H6X4	YITQKQTLTKYPDTFLEGIVNGKILCPFDADGHYFIDRDGL
Q6IB77	KSLRKSLPASLKVYGTVFHINHGNPFNLKAVVDKWPDFNTV
O60939	SCYTVNHKQFSLNWTYQECNNCSEEMFLQFRMKIINLKLER
Q9NWS0	AEVDLPKLDGALGLSLEIGENRLVMGGPQQLYHLDAYIPLQ

P68254	SELRSICTTVLELLDKYLIANATNPESKVFYLKMKGDYFRY
Q5BVD1	ITVIIIGLCLAAVTYVDEDENEILELSSNKTFFIMLKIPEE
Q61205	RVVVLGLLPRGQHPNPLREKNRQVNELVRAALAGYPRAHFL
Q7TQD2	EMHGKNWSKLCKDCHVIDGKNVTVTDVDIVFSKIKGKSCRT
A6NJW9	TVLHGNSVLQQTPAYIKVQTNKMVMLSCEAKISLSNMCIYW
Q5SRD1	GAGGAGYSHADLAGVPLTGMNPLSPYLNVDPRYLVQDTDEF
Q16854	KTTKLHFEALMNIPVLVLDVNDDFSEEVTKQEDLMREVNTF
Q15771	EEFSEAQDMYYLETSAKESDNVEKLFLDLACRLISEARQNT
P00642	LSESHFPYVLFLEGSNFLTENISITRPDGRVVNLEYNSGIL
O14627	PREDWSVYPGPSSTMGTVPVNDVTSSPAAFCSTDYSNLGPV
O14931	HDASIYVCRVEVLGLGVGTGNGTRLVVEKEHPQLGAGTVLL
P00642	EGSNFLTENISITRPDGRVVNLEYNSGILNRLDRLTAANYG
P37237	IAKSNGKGKDCVFTEIVLENNYTALQNAKYEGWYMAFTRKG
Q3ZCH5	YKDREGSHTFQGAFGCELRNNESSGAFWGYAYDGQDFIKFD
P31269	AFSENNAENESGGDKPPIDPNNPAANWLHARSTRKKRCPYT
O54836	RGEPDSALEELCKPLFCKLCNVTLNSAQQAQAHYQGKNHGK
Q91V79	LYHAWLAAVVIFGPLLQFHVNSRTIFASHGNFFNIKFVNSA
P61296	VKEEKRKKELNEILKSTVSSNDKKTKGRTGWPQHVWALELK
Q99865	RVLSLKILSDRVASSHISDANLANTIIGKAVEHMFEGEHGS
Q9Z0G4	AWGPCSTTCGLGIATRVSNQNRFCQLEIQRRLCLSRPCLAS
P70447	RGAKTAETVQRIKKTRRLKANNRERNRMHNLNAALDALREV
P41047	LGMYQLFHLQKELAELREFTNQSLKVSSFEKQIANPSTPSE
Q96S96	LVTAALLLGLMMVVTGDEDENSPCAHEALLDEDTLFCQGLE
Q8C432	EPAESQASDELECKICYNRYNLKQRKPKVLECCHRVCAKCL
Q9D7J9	SEPRLTSTRQQDGIRNIVLSNPRRRNALSLAMLKSLRSDIL
Q9Z132	TPCPEGQKRRKGGQGRRENANRHPARKNSKEPRSNSRRHKG
P00757	VQSQVDCENSQPWHVAVYRFNKYQCGGVLLDRNWVLTAAHC
Q9NTQ9	VMHVAYREERERKHHLKHGPNAPSLYDNLSKKRGGLWWTYL
P22227	GEKPYQCTFEGCGKRFSLDFNLRTHIRIHTGERRFVCPFDG
Q02067	EHDAVSAAFQAGVLSPTISPNYSNDLNSMAGSPVSSYSSDE
Q9BW66	RKPVLSVSARKIKDNAADWHNLILKWETLNDAGFTTANNIA
Q96LD1	LYVKEIHSRKDSPLVLQSDRNVTVNARNHMGQLTGQLTIGA
P57759	ASSEELLVAEVGISDYGDKLNMELSEKYKLDKESYPVFYLF
Q61166	LKLTVEDLEKERDFYFGKLRNIELICQENEGENDPVLQRIV
Q78JT3	RVRVKSWVEENRASFQPPVCNKLMHQEQLKIMFVGGPNTRK
Q8N8J0	GPRFKLLTLGLSLLHADVVPNATIRNVLREKIYSTAFDYFS
P46777	EGKTDYYARKRLVIQDKNKYNTPKYRMIVRVTNRDIICQIA
Q2T9N3	VPATVDGMLGGYGHISSIDINSSRKFLQRFLREGQNKTGTS
Q05B67	NVVPFLELIGLPDSIVNILKNSQSGNALTAYALFKIATPAR
Q9UBK7	GTNVVKLFNDAIRLAVSYKQNSQDFMDEIFQELENFSLEQE
Q9GZN7	IAASGLTRMFAPALPSDLLVNVYINLNKLCLTVYQLHALQP

O00161	MEENLTQVGSILGNLKDMALNIGNEIDAQNPQIKRITDKAD
Q7Z7H8	QEEIGLIRLLRREIAAVFQDNRMIAVCQNVALSAEDKLLMR
Q9H825	ASKYWDTFYKIHKNKFFKDRNWLLREFPEILPVDQKPEEKA
P00484	ERYKSDTKLFPQGVTPENHLNISALPWVNFDSFNLNVANFT
Q8TAY7	GKRALCPTCSLPLSEKERFFNYCGLERALVEVLGAERFSPQ
Q6ZS82	KTTACYHHLVLTVGGSADSQNLRQELQKTRQKAQELAVSTC
P11086	FLEVNRQELGRWLQEEPGAFNWSMYSQHACLIEGKGECWQD
Q9BY19	SHFGCQLVCCQSSNVSVIYPNIYAANPVITPEPVTSPPSYS
Q9CPQ5	QLKKRLLQQCATLKVPPRKLNYLKDVSKMLKMEKAQERANE
P09632	RIEVSHALGLTERQVKIWFQNRRMKWKKENNKDKFPSSKCE
P05231	RKETCNKSNMCESSKEALAENNLNLPKMAEKDGCFQSGFNE
Q9WV68	AAKAAVDAMTRHLAVEWGPQNIRVNSLAPGAISGTEGLRRL
Q8VCR7	APGSQLRGFVPVAPICTDKINAVDYASVKTPALIVYGDQDP
P01921	RWFRNGQEETVGVSSTQLIRNGDWTFQVLVMLEMTPHQGEV
P59279	EAFAREHGLIFMETSAKTACNVEEAYINTAKEIYRKIQQGL
Q9R0M5	IPVGQPCNQVTTTGLKWNLTNDVLGFGTLVSTSNTYDGSGL
Q3MHX1	RLHCTQDPVPEAVGGDMQQLNQLSAQQFSALTEVLFHFLTE
Q8BZI6	QGHFIVLRGYNRATGCIFYNNPAYADRMCSTSISNFEEART
Q9TR36	PCSVQEYQVGQLYSVAEASKNETGGGEGIEVLKNEPYEKDG
Q9DCX1	KEFYELDLSRLAPFGVDQGLNTAACLRRLFRAIFLADPFSE
O55100	FWFVGFCFLANQWQVSKPKDNPLNEGTDAARAAIAFSFFSI
Q9UPY8	LLARQGQDVAPPPNPGDQIFNKSKKLIGTAVPQRTSPTGPK
Q96A25	REDENKSILSSKPAIGSKAVNYSSTGSSKSFCSCVPCEGTA
P04975	EKAKKDLEEWNQRQSEQVEKNKINNRIADKAFYQQPDADII
P63158	GEHPGLSIGDVAKKLGEMWNNTAADDKQPYEKKAAKLKEKY
Q5TAG4	RGRRSKKKRRRGRKEGEEDQNPPCPRLNSVLMEVEEPEVLQ
Q15006	ELYDVTWEEMRDKMRKWREENSRNSEQIVEVGEELINEYAS
Q9H898	ASPYQRRDSDRYCGLCAAWFNNPLMAQQHYDGKKHKKNAAR
Q75VT8	AVLFSGLVAITVLVRKAKAKNLQKQRERESCWAQINFTNTD
Q9CPS7	ENWMKIFTPIVEHLGLQIRFNLKSRNVEIRTCKDTKDVSAL
Q3T0S3	LKCCGFTNYTDFEGSPYVRKNGTFPPYCCYDSVNNSFMEPC
O75792	NSLSHDTATGLIQYALDQGVNVTQVFVDTVGMPETYQARLQ
O75845	IYPFIFPLHKVVYLSLYILVNIWTISIHDGDFRVPQILQPF
Q9D819	HDYWKALVTKKTDGKGISCMNTTVSESPFKCDPDAAKAIVD
Q5E9C8	IEDTLCPFGFEVYPFQVAWYNALLPPAFHLPLPGPTLAFLV
Q9BRV3	TSASWCLYGFRLRDPYIMVSNFPGIVTSFIRFWLFWKYPQE
O15144	VNNRIIEETLALKFENAAAGNKPEAVEVTFADFDGVLYHIS
Q9CY50	LVINLNYKDLNGNVFQDAVFNQTVTVIEREDGLDGETIFMY
O00299	EEQPQVELFVKAGSDGAKIGNCPFSQRLFMVLWLKGVTFNV
Q9Y2Q3	SPYSWLGFEILCRYQNIWNINLQLRPSLITGIMKDSGNKPP
Q8K2H2	PIEILQADAPPIIVGEEYPRNPLVLVYMRHAYGLGEHYNSV

Q3UBG2	YCTADHNVSPNIFAWVYREINDDLSYQMDCHAVQCESKLEA
Q645M6	ARHLGVSDVKIEAIEEKYPRNLAEQVRELLRVWKNSTRENA
Q9D9J8	WFSANITLEFDIEFKLPFNSNIIKTHACMGLTAESWLEKDE
Q9JIW9	RGKAEEWGVQYVETSAKTRANVDKVFFDLMREIRAKKMSEN
P41047	ELREFTNQSLKVSSFEKQIANPSTPSEKKEPRSVAHLTGNP
P46926	KLIILEHYSQASEWAAKYIRNRIIQFNPGPEKYFTLGLPTG
Q5JBG6	KNATCYLGYLKKGIYYQGCFNGGKTASFKRLIQICNMMTMA
Q80UW2	YSGRTDAGSLYELTVRLLSENEDVLAEFATGQVAVPEDGSW
Q6P3D7	IRRKLTYQFSEAVLQRLAPANYLGKDLFYPQYGQPDQGYLS
Q0V7M7	IVVNELLNKLELEIQYQEQTNSSLKELFESLEEDYKDVEHL
Q6UX82	TSCISSSASSSLETPVRLYQNMFCSAENCSEETHITAFTVH
Q9DBD2	KTKDKEQNEKTLGHSMSHPSNISKAGSSPPSTTAPVSAFSR
Q14CZ0	GKVPPPRNSRAPPRLTVVSPNRATSTETSSSVETDLQPFRE
O00194	ENPDIVLIGNKADLPDQREVNERQARELADKYGIPYFETSA
Q14002	EEVTRQFYVFSEPPKPSITSNNFNPVENKDIVVLTCQPETQ
Q9D8C2	TASARNDIQRNLNCCGFRSYNPNDTCPASCAKSTQKCSSCA
Q9D9J2	NDKEGQQDMNSFRANHSSLDNSKFKYHARLSQSPLGSSLGQ
Q6NZQ8	VFMYVQCKVYVQLWRRLKAYNRVIFVQNCPDTANKLEKNFP
P80217	PLVFRGHTQQDPEVPKSLVSNLRIHCPLLAGSALITFDDPK
Q16635	FILEKLNHGDWVHIFPEGKVNMSSEFLRFKWGIGRLIAECH
Q8WZ82	EEPAVCRGLEESLGMVAQALNRLGPFDGLLGFSQGAALAAL
Q8BHH2	KVDKEDRQVTTEEAQAWCMENGNYPYLETSAKDDTNVTVAF
Q3SZ21	FELLYSPAIKDSTMRRYTISNALNLMQVCKGKNVIISSAAE
P11049	AQLERSLRDVVEKTIQKYGTNPEETAAEESWDYVQFQLRCC
P09936	EELKGQEVSPKVYFMKQTIGNSCGTIGLIHAVANNQDKLGF
Q5E9C0	NTLPRGFGSLPALEVLDLTYNNLNENSLPGNFFYLTTLRAL
P48758	AYGVTKIGVTVLSRILARKLNEQRRGDKILLNACCPGWVRT
A2VE58	QTLLRVASWVFSIAVFGPIVNEGYVNADSGPELRCVFNGNA
A3KMZ6	QQTEIYVVPGETALAFYKAKNPTDKPVIGISTYNVVPFEAG
Q5EA33	DVHAVTVDGWTPLHSACKWNNARVASFLLQHDADVNAQTKG
Q3ZCB8	GIELLTLDASWVSSASWYFLNVFGLRSIYSLILGQDNAADQ
Q08E43	YSLSERLIRTIAAIRSFPHDNVEDLIRGGADVNCTHGTLKP
P17861	IQSLISCWAFWTTWTQSCSSNALPQSLPAWRSSQRSTQKDP
Q5E9C0	HNKLTTVPPNIAELKNLEVLNFFNNQIEELPTQISSLQKLK
Q9D7S0	TTVGEFVFKSVECTQPTEYTNSTTTIPPITNTSLTSVTRPG
Q96BX8	QLPPGEDLNDWVAVHVVDFFNRVNLIYGTISDGCTEQSCPV
P00921	LALVYGEATSRRMVNNGHSFNVEYDDSQDKAVLKDGPLTGT
Q9CY24	CSEAQKTSWTPSGTAVQFYSNLHTAETSSWVNLILWCLALL
O08992	SSAARNGLLTDHHICEINGQNVIGLKDAQIADILSTAGTVV
P70377	YLYTSEHFTPECKFKESVFENYYVTYSSMIYRQQQSGRGWY
Q32KZ1	IHAKAFSTEDTQDEMTKKKKNETAFSSVGRKINERIIHVLD

P39942	PSRMAQTIMKARLKGAQTGRNLLKKKSDALTLRFRQILKKI
P05831	ISSEKGFTQVPLCLRKSKLSNLREYQNRIADIAKRSKAVLG
Q0VC18	SLLYRLKFKEFVQSIPTKGFNTEKIRVPLGGSRGITFQVWD
Q9CQ52	LVALASGCGQPSHNPSSRVVNGEEAVPHSWPWQVSLQYEKD
Q91ZX1	ATACHNVGAQLVVIKSDEEQNFLQQTSKKRGYTWMGLIDMS
Q9BS40	STEDTWYKMVKIQTVKQVQRNDDFIELDYTILLHNIASQEI
Q9MZ13	SGVEFSTSGHAYTDTGKASGNLETKYKICNYGLTFTQKWNT
Q99M71	TWIGVYTAKDCYPVQETFIRNYTVVMSTRFFDVQLGIKDPS
P05402	SEKLQAFIERQFSKIIVPVLNTMIQARSSWTGLPSLMSSDE
Q9Z2F7	PPPAGLNSSWVELPMNSSNGNENGNGKNGGLEHVPSSSSIH
P19473	PNFLVSIPVNPKEQMECRCENADEEVAMEEEEEEEEEEEEE
A4D1S5	RSTFESIPHWIHEIEKYGAANVVIMLIGNKCDLWEKRHVLF
P48739	KDDFFIKIETWHKPDLGTLENVHGLDPNTWKTVEIVHIDIA
Q9UL40	QPVGREEVEHMIQKNQCLFTNTQCKVCCALLISESQKLAHY
Q9UGN4	PPQIFLCDKIVETKGSAGKRNGRVSIRDSPANLSFTVTLEN
O70401	FRHEIKNSFKSNYENALKEYNSTGDYRSEAVDKIQSTLHCC
Q8R218	AIAGIWIVFAIAVVNGSVDLNEGFPFISICGSYAPQSCIFG
Q8NBQ5	DIYSSAKKVKAEIGDVSILVNNAGVVYTSDLFATQDPQIEK
Q0V7M8	SGEEEPSLLRTRALRPHPPPNGRANQDDGHPPTGKLEGFES
Q9HBU1	RIDLAESLGLSQLQVKTWYQNRRMKWKKIVLQGGGLESPTK
Q99627	PVAVMAESAFSFKKLLDQCENQELEAPGGIATPPVYGQLLA
Q32KY8	DILLSDYIAFVEKSGCHLEVNFNLEFTEICVNTILYWVFAR
P18111	EKEFHYSRYITIRRKSELAANLGLTERQVKIWFQNRRAKER
Q9EQI8	WEQAFLQFRPGARETEADKKNDRTSLHRKLDRNLVLLVREK
P04393	ISKGNYTFLCQSFEKTIGMVNRDDVVYCDPPYIGRHVDYFN
Q91V79	PAGAPLRLVFLLNVLLLGLWNFLLLCTVIYFHQYTHKVVGA
P35282	FLDLCKRMIETAQVDERAKGNGSSQAGAARRGVQIIDDEPQ
Q0P5B4	KQHTVICSEHFRPECFSAFGNRKNLKHNAVPTVFAFQGPPQ
Q29RR0	ANGPRPVRPGTARPGPEAPPNGPPQPGRSSVGGGGDFYDVA
Q9H2W1	LSVKQATLNPASLQCELDKNNIPTRSYVSYFYHDSLYTTDC
Q8NCR6	KCSPNYLGSDWYNTWRMEPYNSSCCNKYTTYLPRLPKEARM
O70584	PGLLRRVVVPVLVHDRPPSNNGRGEGTSAVPQDKCSARLAT
Q96E22	DCSKYSPEFANSNDKDDQVLNCHLAVKVLSPEDGKADIVRA
Q924T2	LHTLNNVFEPHVAVRDAAKMNIPTVGIVDTNCNPCLITYPI
P63073	EPETTPTTNPPPAEEEKTESNQEVANPEHYIKHPLQNRWAL
Q8BPA8	VGEPVPSGAGSLGSELIKESNANPIFMRKDTKTSFQWRIRN
P12246	LTPQDILFVYRDSPVNPNILNWQALNYEINGYVVIRPRVWD
Q9Z1L2	IFGGVFSLVIFSSLLTDGYQNRTESPQLRCVLNSNHMACSF
Q9D819	VKKGKLRYVANLFPYKGYIWNYGAIPQTWEDPGHSDKHTGC
Q8CCI5	TRSSSTSSSTVTSSAGSEQQNQSSSGSESTDKGSSRSSTPK
Q9NYP7	WYYFSKLIEFMDTFFFILRKNNHQITVLHVYHHASMLNIWW

Q32LM7	KLINDFSQIEKKMIESSGKNNILDMQLEKANCLLRVMQTKE
Q9DBU2	PYYYSIRGVLKDGFTYVLYINGGHPPWTILDYIQHLGSALA
Q00724	EDPAKFKMKYWGVASFLQRGNDDHWIIDTDYDTFALQYSCR
Q96PM5	RYWRQLDDEVAQTPMPSEYQNMTVDILCNDCNGRSTVQFHI
Q96G30	TKTGAPHQDNAESSEKRFRMNSFVSDFGRPLEPDKVFSRQG
Q0VCV7	VARISVKAPTVLEAAAPGSENVAVLTRGSRHLKKMTEEFPT
P07743	SAVNNLKILNPPSEAVPQNLNLDVELLQQATSWPLAKNSIL
P25402	SLSLAINNRVKLIYRPIALKNGRDEAENNIKLINSGTDSCL
Q9BSU3	HRRLGLAQKLMDQASRAMIENFNAKYVSLHVRKSNRPALHL
O08542	VDWSGYEACTAEGANAFQRWNCSMPFAPFSPVRFSEKIQRY
Q8N6G2	KNCLPWKIPASMKEVNKALSNQFISLTKRDFVDRSKAQKIK
P11672	VQKKTEGSFTMYSTIYELQENNSYNVTSILVRDQDQGCRYW
Q6P2H8	ASAELDYTIEIPDQPCWSQKNSPSPGGKEAETRQPVVILLG
Q53R12	SNDTMASGWRASSFHFDSEENKHRLIHFSVFLGLLLVGILE
P48507	AAKALLARARTLHLQTGNLLNWGRLRKKCPSTHSEELHDCI
P13349	DVSNVYATDKNSLSSLDCLSNIVDRITSSEQPGLPLQDLAS
O09172	LVREFPDVLECTMSHAVEKINPDEREEMKVSAKLFIVGSNS
Q969M3	QPFYGNNFEDEPPLLEELGINFDHIWQKTLTVLHPLKVADG
Q8N3J9	GEKVYKCDDCGKDFSTTTKLNRHKKIHTVEKPYKCYECGKA
P12319	WDVYKVIYYKDGEALKYWYENHNISITNATVEDSGTYYCTG
Q8BH95	IQLNRPKALNALCNGLIEELNQALETFEQDPAVGAIVLTGG
P41227	SRAMIENFNAKYVSLHVRKSNRAALHLYSNTLNFQISEVEP
P25732	PFVANANFMIYPISKDLKNGNSELVRVYSKSKEIQYIKIYT
P0CI32	SLVNTPAETLETGFHELFNVNVLGYLLGAKACAPALIASEG
Q5SS90	WQREAEELENVKRKVKLPAINSKNPSKTGTPVSHKEPERSR
Q8MJ50	YVAEQAESIDNPLHEAAKRGNLSWLRECLDNRVGVNGLDKA
Q3KNV8	IRCSAQATVLHLKKFIAKKLNLSSFNELDILCNEEILGKDH
Q96D70	ALASSQVKRLSASRRKQHFINQAVRNSDLVPKAKGRKSLQR
Q80X71	SYDAEKRTIYLNITNTLNITNNNYYSVEVENITAQVQFSKT
Q9NRE1	TSISPGRCKWNKHTLTYRIINYPHDMKPSAVKDSIYNAVSI
P12979	EILRSAIQYIERLQALLSSLNQEERDLRYRGGGGPQPMVPS
P20290	KLQFSLKKLGVNNISGIEEVNMFTNQGTVIHFNNPKVQASL
Q8VCD6	RDKSYETMMRVGKRGLNLAANAAVTAAAKGQGVLSEKLRSF
Q91WR8	QKSKVDCNKGVTGTVYEYGANTIDGGEFVNFQQYAGKHILF
Q15907	DLRHLRAVPTDEARAFAEKNNLSFIETSALDSTNVEEAFKN
Q9Z1L2	AFCFLASQWQHSKSKHFLLGNSSAKAAIALSFFSVPVWILQ
P20489	ADMVLVHGSFDIRCHGWKNWNVRKVIYYRNDHAFNYSYESP
Q5KR49	AELSEGKCAELEEELKTVTNNLKSLEAQAEKYSQKEDKYEE
Q1RML4	KGGKGRKKLRLFEYLHESLCNPEMASCIQWIDQTKGIFQFV
P15946	VGGFNCEKNSQPWHVAVYRYNKYICGGVLLDRNWVLTAAHC
Q9CY28	VRLDHAPSLQQPEVCFIGRSNVGKSSLIKALFSLAPDVEVR

Q5VZV1	QVTATDLPDVLGNLQYNLLKNTLQCTAHLPEVKELVWGEDL
Q969F1	QIKDNDFSYRPNMICNFLHENEDEEVVASAPDKSLELEEEE
O35949	MVITSLQILQMVLGTIFGILNYIWRQEKGCHTTTEHFFWSF
A2VDN0	TRMELEEDVKIYLEENYERINVPVPQFGGGDPADIIHDFQR
P10300	AALWSSSALIQTPSSLLVQTNHTAKMSCEVKSISKLTSIYW
Q8R1Q0	ETSITKAQLSEIEQRHKELVNLENQVKDLRDLFIQISLLVE
Q9Z210	KRAVHLSDVVLRFCITVSHLNRALYFACDNVLWAGKSGLAP
P62079	LGITFLGIGLWAWNEKGVLSNISSITDLGGFDPVWLFLVVG
Q9UIY3	RLFHSFEELLLEAHGDYGLRNDYHMNLGQFLEFLKKHKSEH
Q9UQN3	ALEKQEKQLELEIKKMAKIGNKEACKVLAKQLVHLRKQKTR
Q9D172	GFGAAKNLSTFAVDGKDCKVNKEVERVLKEFHGAKKPIGLC
Q9DA17	LDDPTFKGQQWFRFSTDNDFNIEGKYSEIYALRKQKKMYPN
P51858	KFGKPNKRKGFSEGLWEIENNPTVKASGYQSSQKKSCVEEP
Q8IWT1	FRWTYNSSDAFKILIEGTVKNEKSDPKVTLKDDDRITLVGS
A6H7F9	IQNQTVAILQCLGSGSKVKVNLVHSEKRQKVKHILKNLRVM
Q2KIS7	ALYEYLRQSVGSEAEVWLGFNDMASEGSWVDMTGGHIAYKN
Q9BQB4	CLLVHTAFRVVEGQGWQAFKNDATEIIPELGEYPEPPPELE
P01881	WEPKKSSIVEHVFPSEMRNGNYTMVLQVTVLASELNLNHTC
Q6UXB4	DASAHLVIVGGLDEQGFLTRNTRGRGYWLGLRAVRHLGKVQ
Q9DB05	IAHYEQSADYYKGEESNSSANKCLLKVAGYAAQLEQYQKAI
P50914	RKAKMTDFDRFKVMKAKKMRNRIIKNEVKKLQKAALLKASP
Q3U1C6	EFIAQVKGISVEEVREVTTRNAFRLFPKLQSLLQKELQSHP
Q3T0B6	NGTEAKLVRKVAGEKITVTFNINNSIPPAFGGEEEEPSQGQ
Q2HXU8	IIHTSDHRCNPCPKMWQWYQNSCYYFTTNEEKTWANSRKDC
P05305	WNFCQAGKELRAEDIMEKDWNNHKKGKDCSKLGKKCIYQQL
Q32L50	WMQYLREGYTVCVDCQPPAMNSVSLRCSGDGLDSDGNQTLH
P63075	TSGKHVQVTGRRISATAEDGNKFAKLIVETDTFGSRVRIKG
Q2PT27	TNLSYANFSKVVLEKCELWENRWMGTQVLGATFSGSDLSGG
Q5EA91	LTFGVEVGFKFATKTVIYLLNPCHLVTMMHLPFELEIYYIQ
Q61335	GAAEDGDKLDIGNTEMKLEENKSLKNDLRKLKDELASTKKK
Q925K9	YKGTVAIKVVDRRRAPPDFVNKFLPRELSILRGVRHPHIVH
Q9D7L8	DQTVSVTVVLNVTFPPLLSGNGFQTVEENSDVSLVCNVKSN
Q9CY57	GRGRGRGALTRPVLTKEQLDNQLDAYMSKTKGHLDAELDAY
O76080	NPRTNGMCSVCYKEHLQRQQNSGRMSPMGTASGSNSPTSDS
Q9UKI2	IHIGKEGQHDVFGDISFLQGNYELLPGNQEKAHLGQFPGHN
P13600	TTATDIKGKEVMVLGEVNINNSVFKQYFFETKCRDPNPVDS
Q99LS3	QVFLISGGFRSIVEHVAAKLNIPTTNVFANRLKFYFNGEYA
A6QNP3	SLPEKIQSYERMEFAVCYECNGQTYWDSNKGKNYRIIRAEL
Q9BS16	NIQESSVNLITLHEMLEILINRLFDVPHDPYVKISDSFWPP
Q9HAN9	LKVLRHHQEKLEASDCDHQQNSPTLERPGRKRKWTETQDSS
O60636	AFIGKGVAIRHVQTMYEEAYNDYLKDRGKGNGTLITFHSTF

Q8WVD3	CVSLPWGDPSQITRNFVSHLNQRHQFDYGEFVNLQLDEETQ
Q4JM65	YKQVKTWFQNQRMKCKKWQKNNWPRNSNGMPQGPAMAEYPG
A1YIY0	APSGRAASLLASVKAEWSTKNYSCEAKNNISREISELKKFP
Q99P51	FVLREHEIGEWEAFSLPELQNFLRILDKEEDEQLQSLKRRY
P23184	AREQWKCDGVFVSHIIDIKDNNINVSDSTLIWLHLENYHSD
O95292	SLEPQHELKFRGPFTDVVTTNLKLGNPTDRNVCFKVKTTAP
Q9ET26	SVVCPICASMPWGDPSYRSANFMEHIQRRHRFSYDTFVDYD
P57729	LVIGDLGVGKTSIIKRYVHQNFSSHYRATIGVDFALKVLHW
Q8C552	TNDMIILQTLHWRMQLSIEVNPNRPLGQRARLREPIAQDQP
Q62231	HLHKNESVLKAKAVVAFHRGNFRELYKILESHQFSPHNHPK
Q9BZG1	KCIASTYYRGAQAIIIVFNLNDVASLEHTKQWLADALKEND
Q96S90	DTLAGLALKYGVTMEQIKRANRLYTNDSIFLKKTLYIPILT
P58321	IKSQGQDVTSSVYFMKQTISNACGTIGTIGLIHAIANNKDK
Q9QZE7	GPYEVSKKLYTLKQSLAKVENACYALKVRGSEIPKHMLADV
Q9JHS9	QKAEEERIRMENILSGNPLLNLTGPSQPQANFKVKRRWDDD
O75431	SDGLEEVQKAEMKAYMELVNNMLLTAELYLQWCDEATVGEI
P15319	LDRTRAVFDGSEKSMTLDISNDNKQLPYLAQAWIENENQEK
Q64329	HHHNCSNMQRAFNLKEEMLTNKSIDCRPSNETLEYIKREQD
Q9Y508	IMEGVKATIKDASLQPRNVPNRYTFPCPYCPEKNFDQEGLV
P50294	IRREQYVPNEEFVNSDLLEKNKYRKIYSFTLEPRVIEDFEY
Q3T013	GEQSLPPPAGLNSSWVELPMNSSNGNDNGNGKNGGLEHVPS
Q6ZRN7	GSPRAAQGLGFRGSGQRARHNSFTSPSPPGAHHPLGTHTRA
Q9BQY4	QYMTSLLSPAVDDEKELQDMNAMVLSLTEEVKEEEEDAQPE
Q12846	MRKTQHGVLSQQFVELINKCNSMQSEYREKNVERIRRQLKI
P61085	SLQALLAAAEPDDPQDAVVANQYKQNPEMFKQTARLWAHVY
Q8R138	HEEGLDTSHQLQADILAATQNLRSPARALPGNGEGAKPVKG
A6NMN3	DLLECCLQELREPPDWLVTTNYGVRCVACCRVLPSLDALLE
P03039	TRRLAERLGVQQPALYWHFKNKRALLDALAEAMLTINHTHS
Q91VR2	TIATAETMSIYDDIDADVLQNYQEYNLANLIYYSLKESTTS
Q96G30	DKVFSRQGNEESRSLFHCYINEVERLDRAKACHQTTALDSD
Q9D176	NGGPSGGGGKPGIQHSQAHDNHSFTTDPGDIREQAGVTHSV
P10284	CKEPVVYPWMRKVHVSTVNPNYAGGEPKRSRTAYTRQQVLE
Q9Y3Y2	MQQQQQLASARNRRLAQQMENRPSVQAALKLKQSLKQRLGK
Q9BQI9	VNCKCQDQLLRVAVDTGTQYNRISAGCLSRLGLEKRVLKAS
Q96GG9	NPGQKGLDLEMAIAYWNLVLNGRFKFLDLWNKFLLEHHKRS
Q8VEN2	SPVGTWSGTYEKCNDSSVYYNLTSQSQSVFQTNWTVPTSED
A2ADA5	ALNTHLKHPAIRVLKAKFRVPNDFHARHAATSRTYQYRLATG
Q9D7Z7	VQCHQDQSACFQGNGRMNIGNFSVPVYIRTCHRPSCTTMGT
Q9BTT0	LSSLARLPSLNKLRKLELSDNIISGGLEVLAEKCPNLTYLN
Q765P1	SSLFSLIAGIFLCFSCSPQGNRSNYYDAYQAQPLATRSSPR
Q80UU9	ATFCLDKDALRDEYDDLSDLNAVQMESVREWEMQFKEKYDY

Q15072	FKCSECGTAFGQKKYLIKHQNIHTGEKPYECNECGKAFSQR
Q7M757	SQMSAVCGPLLQWLEDRLEQNQQRLQELEQEKEDLMEELSS
P31240	NANFLVWPPCVEVQRCSGCCNNRNVQCRASQVQMRPVQVRK
Q8K4Z2	WAGVIALFCRQYDIIKDNEPNNNKEKTKSASETSTPEHQGG
Q6UWY2	EELPPGLMEAKVRVLDPDVCNSSWKGHLTLTMLCTRSGDSH
P17026	HLRQHMKVHKEEKPRKTRGKNIRVKTHLPSWKAGTGRKSVA
Q96KX2	VVLLCALKLYVNDHYPKGNCNMLRKTVKSKEYLIACIEDHN
Q8C996	VLKFMLGKVLTSRALITDGFNSLVGGVMGFSILLSAEVFKH
Q32L09	SSGSTVATCLSQGGLLEDLDNLILEDLKEEEEEEEEEEEEG
Q96HF1	PIPANLQLCHGIEYQNMRLPNLLGHETMKEVLEQAGAWIPL
Q96E40	YKLFQQQQLTFTAALEHCRENAHDKIRPISSIGQVQSYMEH
Q9UHW5	IDDYSMVRFLPYDQSDEESMNIVLQHIDFAIQYGEDLEFKE
F1MIW6	ATRPKEGKETEAQAHVPSVVNLTSVTCCPEVAKSYGHKLLT
Q9UMY1	AAQAFIHNSLYGPGTNRTTVNKFLSLANKRLPVKRAAVQFL
Q29438	PCNPCNPCNPCSPCSPCNPCNPCSPCSPCSPCNPCDPCNPC
Q68CL5	RHMISSWEQKNNCVMPEDVKNFYLMTNGFHMTWSVKLDEHI
Q6NZA9	DQSFTSPPPRDFLLDIARQKNQTPLPLIKPYAGPRLPPDRY
O60479	PYSASPSYLDDPTNSWYHAQNLSGPHLQQQPPQPATLHHAS
O89051	ISVPVPEFADSDPANIVHDFNKKLTAYLDLNLDKCYVIPLN
Q9CPR5	AKINIEVQLASELAIAAIEKNGGVVTTAFYDPRSLEILCKP
Q9CY24	FLCGALAAATLTRTQGSFGGNCPLYGVAALNGSSLALLGPS
Q8C7B6	QRSICSQHESLVNDFLLQVCNRCPNLTSVTLSGCGHVTDDC
P49771	VKIRELSDYLLQDYPVTVASNLQDEELCGGLWRLVLAQRWM
P04394	YPEGHKAAAVLPVLDLAQRQNGWLPISAMNKVAEILQVPPM
P62424	IRLQRQRAILYKRLKVPPAINQFTQALDRQTATQLLKLAHK
Q01730	HNKLTTVPPNVAELKNLEVLNFFNNQIEELPTQISSLQKLK
Q14197	TPKEPTKEDVKLHRIRIENMNRERLRQKRIHSAVKTSRRVD
Q08DY6	RRRCRDPINVEGLLPSKIRINLEDNVQYVSMRKALKVKRPR
Q0IIL5	SNALLVQLPELPSKNLFFNMNNRQHVDQRRQGLEDFLRKVL
P70689	LCYLLLKLCFRRSKRTQAQRNHPNHALKESKQNEMNELISD
P56597	VAKETHPDSLRAIYGTDDLRNALHGSNDFAAAEREIRFMFP
Q29RR0	FKDGAFLAGTFISTVGIDFRNKVVDVDGMKVKLQIWDTAGQ
Q3SZC1	LCTAAKQKNNGQNLEEDAGQNEQKTDLPSTEKTLMEEKVKL
Q99KJ0	TEQKKQVDVNIKLWKNGFTVNDDFRSYSDGASQQFLNSIKK
Q8IU54	ALEESLKLKNWSCSSPVFPGNWDLRLLQVRERPVALEAELA
Q60898	QKTFDWKRVSRLQEGSEGAENPLRVDESLFSYGLRESIASY
P30412	TFPDENFKLKHYGIGWVSMANAGPDTNGSQFFITLTKPTWL
Q96ND0	NVVPFLELIGLPDSVVSILKNSQSGNALTAYALFKIATPAR
A6NDR6	VNNWFINARRRIVQPMIDQSNRTGQGAAFSPEGQPIGGYTE
A1YQ93	QGPFNSWFPPFPGILQQQQQNQVPGLSPFSLSTREWFAGLV
P41731	LDKLQKENNCCGASNYTDWENIPGMAKDRVPDSCCINITVG

Q8BHC1	YRNSVGGLLLFDITNRRSFQNVHEWLEETKVHVQPYQIVFV
Q5VXT5	FAFGSCGSYSGETGAMVRCNNEAKDVSSIIVAFGYPFRLHR
Q2HXU8	WEDGSVPSPSLFSTKELDQINGSKGCAYFQKGNIYISRCSA
A6NJI9	LHGITFLTRNYCLTELYLNNNAIFEIEGLHYLPSLHILLLH
P61006	IEEHASADVEKMILGNKCDVNDKRQVSKERGEKLALDYGIK
P04095	EFEKHYSNVSGLRDKSPMRCNTSFLPTPENKEQARLTHYSA
Q9NQ48	DLSNLENTVAALKSEFQKTLNDKTENQKSLEENLATAKHDL
Q8N8R7	HVKIYIDRFEDLQKSCCDPFNIHKKLAKKNLHVIDLDDATF
Q92520	ARRLIADLGSTSITNLGFRDNWVFCGGKGIKTKSPFEQHIK
Q2KHU0	DGATDMEACPPADAFIGFGGNVIRQQVKDNAEWYITDFVEL
Q9DBJ6	APPQIYHEKQRRELCALHALNNVFQDSNAFTRETLQEIFQR
Q925G2	LNAVAAILAIISVVAVFEYHNVQKVPHMYSLHSWVGLTALI
P25393	KEMIKINNIMIHKYTVLYTSNCIMDIYSEEEKITCFSNRLV
Q8VHN8	VLGLVRVPLYTQKDRVGGFPNFLSNAFVSTAKYQLLFALKV
B1ATL7	RGPSHIPTLRSGIVMEVPPGNTRIACRGKLAHVSFPLRGPC
P10966	GEEVEQEKIAVFRDASRFILNLTSVKPEDSGIYFCMIVGSP
P62826	YIQAQCAIIMFDVTSRVTYKNVPNWHRDLVRVCENIPIVLC
Q99M71	QAPQQWEGRQVLYQQSSGHNNRALVSYDGLNQRVRVLDERK
Q86UF1	ASGEEFSFVSPLVKYLLFFFNMLFWVISMVMVAVGVYARLM
P61588	DSQCGKTALLHVFAKDCFPENYVPTVFENYTASFEIDTQRI
Q8BIX3	EDLQKSCCDPFNIHKKLAKKNLHVIDLDDATFLSAKFGRQL
Q8BJK1	RAVHKEAQYYAIGPLLEQLENMQPLKGEKVRQAFLGLMPYY
Q9P1T7	AQVPSGEEIGKIKNGHTGLSNGNGIHHGAKHGSADNRKLSA
A6NGY1	QENCRKRKISSKDICQDRAGNCPEEECNLTLNKKSRSSTAV
P25393	SKVDTNDCRGMSRKIMTTEVNKTLLDELKNINSHDDSAFIS
P05833	LVISDVLFGNTEEKQKPLTVNELNTIQPVAFMRLGLFVPKP
P18121	KAADIKGRNLVILEGLQTIYNRSQANIEENENFDYPAWSGL
P28067	GKPNTLVCFVSNLFPPMLTVNWHDHSVPVEGFGPTFVSAVD
Q15040	KLPLKRQHWICVREVGGAYYNLDSKLKMPEWIGGESELRKF
Q8VCH9	NLKEIPRDLPPETVLLYLDSNQITSIPNEIFKDLHQLRVLN
P04187	EVELTVQKDRECESYFKNRYNKTNQICAGDPKTKRASFRGD
Q9D3G5	MSGEQIEDMFEQGKWDVFSENLLADLKGARAALNEIESRHR
Q0VC80	HAFMQDLAQMFGGPLALTSANLSSQSSSLNVEEFQDLWPHL
Q8WUK0	LGVEQLRLSTVDMTGIPTLDNLQKGVQFALKYQSLGQCVYV
Q9CRA5	TWNPLKLHYQLRNVRERLAKNLVEKGVLTTEKQNFLLFDMT
P0CG29	WVQVLGPLIGVQVPKEKVERNRTAMDQALQWLEDKFLGDRP
Q3V2Q8	PNLLEEAHEWNQRRARKAMRNGISPIIIDNTNLHAWEMKPY
Q9D176	TLRGRARPRWRAGNTTPVPVNQTGTCAQLHPPPQGTLQVVR
P0A1V9	GVNRGFAGHNQDQDLRSAMRNSTVWVYELFAKEIGDDKARR
Q9H9H4	VQKMEETQNVQLNKEMTLASNRSLAEGNLLYQPQLDTLKAR
Q9UL40	THAKNLKLKQQSTKVEALHQNREMIDPDKFCSLCHATFNDP

Q08E62	LVLRLDSRLWPKIQGLFSSANSPFVPGFSQSLTLSTGFRVI
Q61754	LTAAHCYGNATSQYNVWLGKNKLFQREPSAQHRWVSKSFPH
Q3LI83	LSSSDLSPTFGHCLPSSYQGNLWLLDYCQESYGEAPTCKSP
Q15102	ERQPQARVVVLGLLPRGQHPNPLREKNRQVNELVRAALAGH
Q8C8M1	SGNRMKSNQISKLQKEFKRHNSDAHSTTSSASPAQSPCYSN
Q3T013	SSWVELPMNSSNGNDNGNGKNGGLEHVPSSSSIHNGDMEKI
Q148M8	ARPGGRERARLANAQDQARSNRGLLAKIFGAPTPSESRGNS
P20959	SESKRETEYGPCRREMEDTLNHLKFLNMLSPRGIHIPNCDK
O43482	MVCYLLKTKAIVNASEMDIQNVPLSEKIAELKEKIVLTHNR
Q8K3V1	RRYKSRGSKEKAAQPLSKSDNDGNARKEIHVKQSGNPCENT
Q3T0T1	QELLVEAITAGILGDLGSGGNVDACVITAAGAKMLRALSSP
Q9UIV1	VAMDTEFPGVVARPIGEFRSNADYQYQLLRCNVDLLKIIQL
Q80V23	HTGQKPFECTQCGKSFRAKGNLVTHQRIHTGEKPYQCKECG
Q9HB20	TIQEFVHHDENHSSPSAENMNEASSLLSATCNTFITTLEEC
O60939	TVPATLNVLNGSDARLPCTFNSCYTVNHKQFSLNWTYQECN
P21812	PTSDTLREVKLRIMDKEACKNYWHYDYNLQVCVGSPRKKRS
Q8BGI3	DLVQRSLVLFSFGVVLALVLNLLQIQRNVTLFPDEVIATIF
P22710	EDRAERLALLACLLAVIPALNLASSDAAAVAGLVMTVLTVG
H3BS89	ARNRRQLFAMSPADECSRQYNSTNMGLWRKCHRQGFDPEIA
Q2KJ29	VPGDIKGETCSAKQHLDPHRNGETKADDSSNKEAAEEKQEE
O75558	FLQMAVLVEKQADTLNVIELNVQKTVDYTGQAKAQVRKAVQ
Q99895	VLTAAHCISNTRTYRVAVGKNNLEVEDEEGSLFVGVDTIHV
Q3SZ21	VIISSAAERPLEIRGPYDVANLGLLFGLSESDAKAAVSTNC
Q9QVN7	LKNMPITLHLLQSTRVGMSVNALRKQSSDEELIALAKSLIK
P51908	CLLYEINWGGRHSVWRHTSQNTSNHVEVNFLEKFTTERYFR
P02722	GNCITKIFKSDGLRGLYQGFNVSVQGIIIYRAAYFGVYDTA
Q9CY94	SVELPKMYQEGWRTVFSADANVVDLHKMGPHFYGFGSQLLH
Q15475	SFGFTQEQVACVCEVLQQGGNLERLGRFLWSLPACDHLHKN
Q9JJW0	NKLLRQRRYHAALAVIKGFRNGAVYGVKIRAPHALVMTFLF
O35955	TLFRYQGHVGASLVVGGVDLNGPQLYEVHPHGSYSRLPFTA
Q9R0X0	LEFLQSFLGSHAPGAPTVFGNRHDAVYGPADTMIQYMELFN
P0CG37	TVSLALQIINLGNSYQREKHNGGREEVTKVATQKHRQSPLN
Q17R31	PEDQRSVTLKDLDVALPIIENYKDRLLAIGEVGLDFSPRFA
P20033	KLKEVQVRLEEHLECACATSNLNPDHREEETGRRRESGKNR
Q99KX1	DMFGMMNDMIGNMEHMAAGGNCQTFSSSTVISYSNTGDGAP
Q8K1T1	VLPGAFFDKLEKLQTLIVTHNQLDSVDRSLALRCDLELKAD
Q8R191	VNEGYVNSDSGPELRCVFNGNAGACRFGVVLGLGAFIACVA
Q29RZ5	AANRDEFYHRPARAADFWGSNNEVLSGLDMEEGKEGGTWLG
Q3URF8	VQPLIEALQEKEVELSKAEKNAMLNITLKQRVQTVHFTVRE
Q0II65	AFWKKYRIFWKEDRAFWKEDNALWERDRNLLQEDKALWEEE
Q9XSK0	REEVALKINLPESRVQVWFKNRRAKCRQQRQQQKQQQQPPG

Q9BQT8	GVFNMVYFGFYYNVKNMIPVNKDPILEFWRKFGIGLLSGTI
P0DMD0	IKHIINQHPNTLFIVFMAIANVHFDEYLLVRKNLLISSKSI
O60637	YRAKVENEVDRSIQKVYKTYNGTNPDAASRAIDYVQRQLHC
Q9DB05	QLHLQLQSKHDAATCFVDAGNAFKKADPQEAINCLMRAIEI
Q64279	ERRRTESINSAFAELRECIPNVPADTKLSKIKTLRLATSYI
O35943	NPRWREPIVTCGRRGLHVTVNAGATRHAHLNLHYLQILNIK
Q99M03	VCVEGPQSACEEFWSRLRKLNWKRILIRHREDIPLDGTAGG
P13949	RPLNSQSVNKYILNVQNIYRNSPVPVCVRNKNRKILYANGA
Q99020	LKDYFTKFGEVVDCTIKMDPNTGRSRGFGFILFKDSSSVEK
P08034	TEKTVFTVFMLAASGICIILNVAEVVYLIIRACARRAQRRS
Q58DS3	DGLAALQRGLAPALLYQFLMNGIRLGTYGLAEAGGYLHTAE
P17936	HAKDSQRYKVDYESQSTDTQNFSSESKRETEYGPCRREMED
O95760	SNCVSFECKTDPGVFIGVKDNHLALIKVDSSENLCTENILF
Q9TR36	WGLQSKVENFIQKQEKRIFTNFHRQLFCWIDKWIDLTMEDI
Q6UWM5	KEEKCVKNLCRTPQLIIPNQNPFLKPTGRAPQQTAFNPFSL
Q6ZMS7	LLQEYGLLQRRLENVENLLRNRNFWILRLPPGSKGEAPKVP
Q6ZUS5	QEQWYLESLIQARQRLQGSHNQCLNRQDVPKTTPSLPQGTK
P45879	QKVNKKLETAVNLAWTAGNSNTRFGIAAKYQIDPDACFSAK
Q9H560	GANPNIKDIYSNTALHYAVYNKGTSLAEKLLSHHANIEALN
Q8CFI2	SILHPPVDDPQSGELPSERWNPTQNVRTILLSVISLLNEPN
P57052	NNTSLPQEYFLFQKMQWHVYNPVLQLPYYEMTAPLPNSASV
Q00262	FMDMAMFVETQGEMVNNIERNVVNSVDYVEHAKEETKKAIK
Q32KU2	SHTAPQDDLMLIRLAKPAILNEKVQPIALATSTVKPGTICM
Q6UX82	GKECSNTSDALDPPLKNVSSNAECPACYESNGTSCRGKPWK
Q3SYZ9	VLAPQYPWQSNDMAMNMLPPNHSHDFLLEPPGHNKENEDDV
Q3T0Y3	ILDKIIYHTSFEMMKDNPLVNYTHLPSEVMDHSKSSFMRKG
O09116	MVPGPSPSSTSEPSSEPCSINVREPGYMNASEPTHAKVPDQ
Q80WY3	RVLCPVLRRYTCPLCGASGDNAHTIKYCPLSKVPPPTVRPP
Q4JM65	NNQFNNYMEEFLQPGIQLQQNSPVCDLEATLGTAGENYNVI
Q58DN3	QQGFIHTKGCVGQFEKWLQDNLIVVAGVFVGIALLQIFGIC
Q8R0W6	YWLWWVFLVLGFLLFLRGFINYAKVRKMPETFSNLPRTRVL
Q3T106	TGMPRRSRFDRPPARRPFDPNDRCYECGEKGHYAYDCHRYS
Q8WTR2	LVHCNAGVSRAAAIVIGFLMNSEQTSFTSAFSLVKNARPSI
P05408	LTGDNIPKDFSEDQGYPDPPNPCPVGKTADDGCLENTPDTA
P25393	ILRHLKNALMIIYGMSKVDTNDCRGMSRKIMTTEVNKTLLD
P68250	AELQDICNDVLQLLDKYLIPNATQPESKVFYLKMKGDYFRY
Q2T9L9	PVRLSQQLDKVVTTNYKPVANHQYNIEYERKKKEDGKRARA
Q2HJ98	KPSTAYAPEGSPVLVPAYTRNLHHELELAVVMGKRCRAVSE
Q53HV7	FSEPVGIIYNPVEYAWEPHRNYVTRYCQGPKEVLFLGMNPG
Q8VI88	YFIGWLVLILYFTCGILCYLNHKNYWSLIMSSTTINTACSS
O60259	WQAALFQGQQLLCGGVLVGGNWVLTAAHCKKPKYTVRLGDH

Q8BUY5	KDAMSHFAIAGAVTGGLFRINLGVRGLVAGSIIGALLGAPM
Q3SZW6	QHYPCTTWRHQLEREDLGPPNTAAASAPEMIQHSLWRPVRN
P51180	LGAVAGAAVLYSVTPPAVRGNLALNTLHAGVSVGQATTVEI
Q9UKR8	PDDYSTQWNLVMEKLKCCGVNNYTDFSGSSFEMTTGHTYPR
Q3U1C6	EQRQVLIRQVQLAKRLNVPLNVHSRSAGRPTISLLREQGAE
P14231	TGQPCVFIKMNRVINFYAGANQSMNVTCVGKRDEDAENLGH
P04179	LQPALKFNGGGHINHSIFWTNLSPNGGGEPKGELLEAIKRD
Q9WV55	DRKVCFKVKTTAPRRYCVRPNSGIIDPGSIVTVSVMLQPFD
P16110	VLDFRRGNDVAFHFNPRFNENNRRVIVCNTKQDNNWGKEER
Q99967	GPPVASQGGSLPASMQLQKLNNQYFNHHPYPHNHYMPDLHP
Q9WTN0	WLKVPEDKLQIIIEVTEMLHNASLLIDDIEDSSKLRRGFPV
P10522	VGDPHRKDGSIVIHNLDYGDNGTFTCDVKNPPDIVGKTSQV
Q9CQL0	QVTITDRKVALEFLKSNVEANLPPHIQPKAVVKELTWGQNL
Q505H4	PVCDQPECPKIHPKCTKVEHNGCCPECKEVKNFCEYHGKNY
Q91WD9	FISAAELCNFLRDLFLHHKKNISEAELEEYTSTMMKIFDKN
P11911	CLGPGCQALRVEGGPPSLTVNLGEEARLTCENNGRNPNITW
P07992	GSEPLAGETPNQALKPGAKSNSIIVSPRQRGNPVLKFVRNV
Q5JUR7	RFTCKGLNIVHRIKAYKSVLNYLKTSGEYKLAGVFLGGRSM
Q8NBQ5	TAAKCKGLGAKVHTFVVDCSNREDIYSSAKKVKAEIGDVSI
Q96H12	YNSQPSVSLRDFKQLKKCWENIKARTKKIMAHERREKVKRS
Q8C3M9	LVKQEVKRLLGEYIGIRLRENEFDPKGRGQLTFLDDMVNQV
P70331	LPNGSSHDHLSEPGSAGHAGNGALGGSKAHRKLQTHPSLGS
Q9QY36	HRRLGLAQKLMDQASRAMIENFNAKYVSLHVRKSNRAALHL
O88630	RMFETMAIEIEQLLARLTGVNDKMAEYTHSAGVPSLNAALM
Q3ZBW4	VISCAKDGVKFSASGELGNGNIKLSQTSNVDKEEEAVAIEM
Q58DN3	LIDFAQEYWSCCGARGPNDWNLNIYFNCTDLNPSRERCGVP
P82348	GWRKRCLYLFVLLLLAILVVNLALTIWILKVMWFSPIGMGH
P27573	SIVIHNLDYSDNGTFTCDVKNPPDIVGKTSQVTLYVFEKVP
Q8N8L6	LSEAMSMGELQRELGLQAIDNQREVFLLAASIAPAGPTFEE
P36980	QTHLEGDTVQIICNTGYRLQNNENNISCVERGWSTPPKCRS
Q3T046	GFVHHGTILDCEETDWDFSMNLNVRSMYLMIKAFLPKMMAQ
Q96FX7	ALAARGFSELSTLEVLPQVYNVRTVSLPPPDLGTGTDGPAG
Q9QZ06	CRLRLGYAVYETPTAHNGAKNPRWNKVIQCTVPPGVDSFYL
P53810	CPYMCAYKLVTVKFKWWGLQNKVENFIHKQEKRLFTNFHRQ
Q8TCD6	FDKVFTNPAAFNSNGHLTVENYHTHSCNRCPKNLCKKVVLI
O35257	QPCFSGTLLMLLASNFLLWKNVAPVPMYASLDEYGEMSIYD
Q969T4	SPALTISKVLLSICSLLTDCNPADPLVGSIATQYLTNRAEH
Q8N4C7	DLRDLFIQISLLVEEQGESINNIEMTVNSTKEYVNNTKEKF
Q91XF0	GAVVSRQSSVIPDREYLRKKNEELGQLYQDQEVPKPEYWGG
Q04743	RYLGHRFQGNDTSPESFLLHNALARKPKRIRTAFSPSQLLR
Q3T0M7	YITPMMELKPNAGSDRAWVWNTHADFADECPKQELLAIRFL

P62956	CVASEYFLEPEINLVTENTENILKTVRTATPFPMVSLFLVF
Q61820	KVDVKDMKVKAKPILFHRKKNLQYYDISARSNYNFEKPFFW
P19639	RKHNLCGETEEERIRVDTLENQVMDTRIQLMIVCCSPDFEK
P61106	AKTGENVEDAFLEAAKKIYQNIQDGSLDLNAAESGVQHKPS
Q9EST4	QLRSTPFRYLLTPCLQKSVQNKIKSLNWLEMEKSRCIPEMS
Q8C8C1	KQLCYECGTARLDESSMLEENIESLVDNLITSLREQCYGER
P00756	WHVAVYRYTQYLCGGVLLDPNWVLTAAHCYDDNYKVWLGKN
P11019	YEKKEKQIEQQKKIQMSNLMNQARLKVLRARDDLITDLLNE
P10648	EFEEKFIQSPEDLEKLKKDGNLMFDQVPMVEIDGMKLVQTR
A5D7U1	TVSTQVSHGIISVVLEARDQNKSVIGSWENPSDHCEGRAEY
Q7TMJ8	QRITLPLSAFTNPTCETVDENTIIVHSNQTPADVQEGSTLL
P09024	RGRQTYTRYQTLELEKEFHYNRYLTRRRRIEIAHTLCLTER
Q2HJF4	QKIAKEMNLSETAFIRKLHPNDNFTQSSCFGLRWFTPQNEV
P13726	NLTWKSTNFKTILEWEPKPVNQVYTVQISTKSGDWKSKCFY
Q8WV92	DNTKRCNLREKISKYMDRAENIKKYLDQEKEDGKYHKQIKI
Q6UW56	LANTFRGFTQLQTLILPQHVNCPGGINAWNTITSYIDNQIC
Q29RS4	KLLKVSRDKSFLLDRLLQYENVDEDSSDSDATASSDNSETE
P32326	RKYNMTPSQFRLQSRQSNDPNFITNLSLRSNPIEFDKEIDE
Q8WUJ1	KEPRCVCVRTTGPPSGQMPDNPPHRNRGDLDHPNLAEYTGC
Q9UBT3	VLLGLSWLCSPLGALVLDFNNIRSSADLHGARKGSQCLSDT
P29692	SLRGVVQELQQAISKLEARLNVLEKSSPGHRATAPQTQHVS
P24699	SPVWSRKNSSFDSIYCPDVSNACAADKSSVSSLDCLSSIVD
A8MWP4	RAQCQPLTQRPHSLHLQDSRNASSLPHKGWRCNFPLQGPAG
Q8N488	RQAKPAADEGFWDCSVCTFRNSAEAFKCSICDVRKGTSTRK
A7E369	QPPLPPDSPRYCMISDLFIDNYQVKCINGKMCYVQKQPAPH
Q9NX70	SLQTLMKVAAQNLIQNTNIDNGQKSSDGPIQRFDKCLEEFY
A6H773	YSVVLSEKSTVFHQNDVKIPNSTHVFTTFYAKTKSLLVNPA
P15018	RIVVYLGTSLGNITRDQKILNPSALSLHSKLNATADILRGL
A6NCN2	EMKATVIRHGETLRRTKEEINELNRMIQRLTAEVENAKCQN
O95865	DGTDVLFTGREFFVGLSKWTNHRGAEIVADTFRDFAVSTVP
Q86TG1	ALICLLRYGQLLEQSRHSWVNTTALITGCTNAAGLLVVGNF
Q9BQY9	ADVFLPCEDPPPTPQSSGMDNHLEELSLPVPTSDRTTSRTS
Q8BVN0	IKNLKTMKEDLMENLQDSQGNCTIQIQEDISEIKNKIMTVK
P19217	VKLLPVSFWEKNCKIIYLSRNAKDVVVSYYFLILMVTAIPD
P01241	PVQFLRSVFANSLVYGASDSNVYDLLKDLEEGIQTLMGRLE
P52823	IAKRNPEAITEVVQLPNHFSNRYYNRLVRSLLECDEDTVST
Q96L14	EKQLQAINAMIDPDGTLEALNNMGFPSAMLPSPPKQKSSPV
Q10738	ALPLSQEAGDVSAHQWEQAQNYLRKFYPHDSKTKKVNSLVD
Q99836	SSLPLAALNMRVRRRLSLFLNVRTQVAADWTALAEEMDFEY
P30039	PDTQKLLVRLSDVYNRSFLENLKVNTENLLQVENTGKVKGL
Q2KID0	KRWKVETNSRLDSVLLLSSMNLPGGELRRRSAEDELAMRGF

Q9NUP1	KAEAELGTFPRAFKKLLHTMNVPSLFSKSAPSRPQQAGYEA
Q5SZD4	FLKGPSPRLTYLSVANADLLNRTWSRGGNEQCLRYIANLIS
P33681	TTVSQDPETELYAVSSKLDFNMTTNHSFMCLIKYGHLRVNQ
Q3MHR7	EAVEVTFADFDGVLYHISNPNGDKTKVMVSISLKFYKELQA
A6NI73	LMVLLCLGLSLGPRTHVQAGNLSKATLWAEPGSVISRGNSV
P29218	MDYAVTLARQAGEVVCEAIKNEMNVMLKSSPVDLVTATDQK
Q9H190	VQATAISPPPVLYPNLAELENYMGLSLSSQEVQESLLQIPE
Q2HXU8	CNPCPKMWQWYQNSCYYFTTNEEKTWANSRKDCIDKNSTLV
Q8CEZ0	SYNYGNQDQAEFLCVVSRELNNSTNGIVIEPSEKPRFFRKR
P28161	YEEKKYTMGDAPDYDRSQWLNEKFKLGLDFPNLPYLIDGTH
Q8N0Y7	DQLPSYESPKDTIARALPFWNEEIVPQIKEGKRVLIAAHGN
Q99MH5	PDSLRAIYGTDELRNALHGSNDFAASEREIRFMFPAVIIEP
B6A8C7	SPAGKEIDFSLVDVTAGDAGNYSCMYYQTKSPFWASEPSDQ
Q3ZBG5	IGCALEDQKKIKRRLETLLRNIENADKAIKLLEHSKGAASK
Q8IYP2	EAELNDYVKLANLPYQTISENTMCSVSTWSYNVCDIYKEPD
Q9JHL0	LCVHCSRPGVKRNEKIYEQRNRQENAQSSAAAQTYSLARQV
Q14002	PGPAHNGRETIYPNGTLLIQNVTHNDAGFYTLHVIKENLVN
Q9BGL2	VASIRVDTVEDFAYGAEILVNHLDRSLKKKALLNEEVAQRA
A5PKE4	DHLLVENIERETFHLCSRLINGPYRRTVRALVFTLKHRAEI
O43921	LLLLPLPPPPFARAEDAARANSDRYAVYWNRSNPRFHAGAG
Q9JKV5	QVLVKRIYRLWMFYCATLGVNLVACLAWWIAGGAGANFGLA
Q0P5F2	TDTAHLSPTEAFCVFYHLKSNPSVMLCQCSCYVAEDQQYQW
Q96SJ8	AMGGLLFLLGFLGCCGAVRENKCLLLFFFLFILIIFLAELS
Q56A07	SVTLKNVQLEDEGIYNCYITNPPDRHRGHGKIYLQVLLEVP
P37980	VLGILAMIDEGETDWKVIAINVEDPDAANYNDINDVKRLKP
Q02105	KFTCEVPGLYYFVYYTSHTANLCVHLNLNLARVASFCDHMF
P18121	LSGSAGMQLLLLVSSLLLWENVSSKPTAMVPTEDLYTRLAE
Q8BQP9	ESAHKTQRALDDCKMLVQEFNTQVALYRELVISIGDVSVSC
Q9JK39	GRTSFNGSHVARGEAAVKIHNVTVFDNGTYHCVFKEYTSHS
H3BNL1	AVFLQRCQEPAQRHFFSKHDNRTSFDKGPYCLLQGIGRRKD
Q2YDD6	LTDGYQNKTDNSELHCVLNSNSTACSIAVGAGLLAFLSSLA
Q8TDB4	GKDASLRRMSSNRFPGSSGSNMIYYLVVGVTVSAGGYYAYK
P09632	SQIQEFYHGPSSLSTAPYQQNPCAVACHGDPGNFYGYDPLQ
Q92600	QEIVNIYPSINPPTLTAHQSNRVCNALALLQCVASHPETRS
B0FP48	IGCNHPLPGPGPYRVKFLVMNDEGPVAETKWSSDTRLQQAQ
Q8TBE9	EDVKAMLTELRKEVRLLLLTNGDRQTQREKIEACACQSYFD
Q9D0U6	SYNPDLDSDPFGEDGSLWSFNYFFYNKRLKRIVFFSCRSIS
Q5E9S8	QRYLNGVRKNGAAPVLLELANEVDYAPSLMARIILERFLQK
Q9ULC0	LFLLPSICSSNSTGVLEAANNSLVVTTTKPSITTPNTESLQ
Q92629	EGDSEFLQPLYAKEIQSRPGNALYFKSARNVTVNILNDQTK
Q96EU6	VPISKKVARDPRFDDLSGEYNPEVFDKTYQFLNDIRAKEKE

Q5E948	PDPNTEESPKRKNITYEELRNKNRESYEVTLTHKTDPSVRP
P61019	TRRDTFNHLTTWLEDARQHSNSNMVIMLIGNKSDLESRREV
Q8VC90	RCVRRYDHHCPWMENCVGERNHPLFVAYLALQLVVLLWGLC
Q32KL5	QRISASLLDSRSYETLVDFDNHLDDIRNDWTNPEINKAVLH
P0DMN0	LALLPQTLLDQKVKVVYVARNPKDVAVSYYHFHRMEKAHPE
Q80WB5	YQPVCPTRDACVYNSCYCEENIWKLCEYIKTHNQYLLEECY
Q6PHN7	LACPPCPGATVIFKLQMHMLNGALLALLFPVVNTRLLPFEL
Q7Z4W1	MLTKVMALELGPHKIRVNAVNPTVVMTSMGQATWSDPHKAK
Q9CR95	SVAISNHVTPPPIPKSNHGGNDSDILLDLDSPAPVSTSAPA
Q9CZQ6	NISDSYFFTFYTENMSWRSANDESGVIMNKWKDDGDLVQQL
O09172	IEDGVNLSLEHLQPYWEELENLVQSKKIVAIGTSDLDKTQL
A4IFJ1	AFYFSQGTSKAISKGDFRLANTTSRRALFLATLSIAVGAGL
Q32LM6	VELPKTDEGLGFNVMGGKEQNSPIYISRIIPGGVAERHGGL
Q921I9	YGPHEIRGSRSRALPDRALVNCQYSSATFSTGERKRRPHGD
P35292	KSLLFMETSAKLNYQVSEIFNTVAQELLQRAGDTGSSRPQE
Q9R1P3	LIGIQGPDYVLVASDRVAASNIVQMKDDHDKMFKMSEKILL
P20827	APLLGLCCSLAAADRHTVFWNSSNPKFRNEDYTIHVQLNDY
Q9HAT0	QVAGRLIIRAEELAQMWKVVNLPTDLFNSVMNVGRFTEEIE
Q96BQ1	MCFEDRMIMSPVKNNVGRGLNIALVNGTTGAVLGQKAFDMY
P70447	QRIKKTRRLKANNRERNRMHNLNAALDALREVLPTFPEDAK
Q9BXS1	DWVDRRQLQRLEEMLIVVDENDKVIGADTKRNCHLNENIEK
Q91VU0	LLTFYVISQVFEIKMDASLGNLFARSALDSAIRSTKPPRYK
Q8K3B1	LCNLPSYKAKVRAFQHAFSTNDCSRNVYIKKNGFTLHRNPI
Q58DU5	LADARSLADIAREEASNFRSNFGYNIPLKHLADRVAMYVHA
P48230	FSILLVVGGIQMVLCAIQVVNGLLGTLCGDCQCCGCCGGDG
Q9NVP2	EFIRVGYYVNNEYLNPELRENPPMKPDFSQLQRNILASNPR
Q28110	IITWFRLRRKPISAGLTDAENDAARTEAENTVTYSLLSHPD
O88992	GQVRASAIAQDADQNYDYASNSVILHLDAGDEVFIKLDGGK
P61085	AVQRIKREFKEVLKSEETSKNQIKVDLVDENFTELRGEIAG
A4IFN5	AGSLLKELSPLPESYLSNKRNVLNVYFVKVAWAWTFCLLLP
Q8IUW5	PDNGSSRTLHSRTETTPSPSNDTGNGHPEYIAYALVPVFFI
Q9NXJ0	TLMIFSLLEFFVACATAHFANQANTTTNMSVLVIPNMYESN
P56965	STVPVVDALHLKSFCSMAGPNLIAIGSSESAQKALKIMQQM
Q9CZE3	DSRTLVRLQLWDIAGQERFGNMTRVYYKEALGAFVVFDISR
Q5EA92	LSSNDMLLLQLRTGMTLSGNNTICFHHAKVYIDRFEDLQKS
O43315	DQVVATMILLIIVFAIFDSRNLGAPRGLEPIAIGLLIIVIA
Q3LI83	RNGSNCFGQLNCLSKSFQTLNHCRLSTLGYKSYQNPCFIPS
Q9R1P0	LYQSDPSGNYGGWKATCIGNNSAAAVSMLKQDYKEGEMTLK
Q1W209	LGPSPLAASPAFLGQGQVPLNPFSFTLSGKSRFSGAGASTP
Q17R31	LTSICLETDSPALGPEKQVRNEPWNISISAEYIAQVKGISV
Q3T0C2	VDHFGKLDILVNNAGVNNEKNWEKTLQINLVSVISGTYLGL

P52743	GKLCKCNDCHKVFSNATTIANHWRIHNEDRSYKCNKCGKIF
P29084	LVNNPKIEVIDGKYAFKPKYNVRDKKALLRLLDQHDQRGLG
P35270	GSLGDVSKGFVDLSDSTQVNNYWALNLTSMLCLTSSVLKAF
Q3SYW9	KMSLNERFTNMLKNKQPMPVNIRASMQQQQQLASARNRRLA
A6H789	LQTLDLSDNRIQSVHKNAFNNLKARARIANNPWHCDCTLQQ
P35705	FVCPTEIIAFSDKASEFHDVNCEVVAVSVDSHFSHLAWINT
Q9Z2Y8	GHGQRTFGENYVQELLEKASNPKILSSCPEIKWHFIGHLQK
Q96CS2	KASEYESEAKYLQDLLMESVNFSPANLSSTGSRYLNALVDS
P06343	RWFRNGQEETVGVSSTQLIRNGDWTFQVLVMLEMTPRRGEV
Q60682	KEWAWIHNGQSKLDMKIKKMNFTSRGCVFLSKARIEDTDCN
P47962	GLTNYAAAYCTGLLLARRLLNRFGMDKIYEGQVEVNGGEYN
P46777	YDSESKEFNAEVHRKHIMGQNVADYMRYLMEEDEDAYKKQF
Q86TA1	EVQINNEEIFPTCVGVPFPKNFLQICKKILCRLFRVFVHVY
P62492	RIVSQKQMSDRRENDMSPSNNVVPIHVPPTTENKPKVQCCQ
Q91V79	HVNSRTIFASHGNFFNIKFVNSAWGWTCTFLGGFVLLVVFL
Q9D9M5	DVAMPREGYTLHRTLAKMSQNLEPMESSIVVWSSGVEIISH
O60635	TDFEDSPYFKENSAFPPFCCNDNVTNTANETCTKQKAHDQK
B2RXH8	LQAIKQELTQIKQKVDSLLENLEKIEKEQSKQEVEVKNAKS
Q9Z210	VLRFCITVSHLNRALYFACDNVLWAGKSGLAPRVDQEKWAQ
Q8TBE3	WCVRCHEPRWSYRAGHMEEANGLVRWPEEAPDLGQREEDLQ
Q99218	FAMPLPPHPGHPGYINFSYENSHSQAINVDRIALVLTPLKW
Q9Y235	LKELIELPPFEIVTGERLPANFFKFQFRNVEYSSGRNKTFL
O18979	LATSSTRAQQRAAAQRRTFLNAHHRSAAQVFPEPPESDHED
B9EJG8	ASFGMTLLGNFQLTNDEEIHNVGTSLTFGFGTLTCWIQAAL
Q8BGN6	NTIKFWQTYSIKGPTTGSDVNKEKIDVMSLLTGLIVAGVFL
P01139	GDKTTATDIKGKEVTVLAEVNINNSVFRQYFFETKCRASNP
Q02242	CSLSNWSEDLMLNWNRLSPSNQTEKQAAFCNGLSQPVQDAR
P12970	VNSEDKGALAKLVEAIRTNYNDRYDEIRRHWGGNVLGPKSV
Q6Q7D1	LGSHLVLTAHTEALLQQVVGNCRKLGAPKVFYIAADMASPE
Q02399	TTSLVNVVPKLNATGRDLLQNLLKCNPVQRISAEEALQHPY
Q2TBL9	GQRDGIRVGHAVLAINGVDVNGKYTADGKEVLEYLGNPANY
P10649	ADIVENQVMDTRMQLIMLCYNPDFEKQKPEFLKTIPEKMKL
Q8VI64	SQEGADTSQEGADTTKEEADNSKEAEGTTTEDPRSISEESA
Q3UX61	MKRDLSQMTDELRRQLVLKKNRYVVLGSEETQGGTLPDAGE
A6NI79	ETVKEKNLILEEKITTLQQENEDLHVRSRNQVVLSRQLSED
Q3SX11	EKIQMKKTIKMHEKRNTKQKNDEKTPQGAVPAYLLDREGQS
P49788	SLLQEGEGRLGKCSARVFFKNQKPRPTINVTCTRLIEKKKR
Q9D9M4	LGLGLVVIQNGPYLQISHLINKGAAASDGILQPGDVLISVG
Q6P073	LHRGQHGIQLLNGHAPAAGPNLAGLPQANRHHGLLGGALAN
E9PW74	LHLALLVQRADVPFFGQEASNAVQLMQELLGDSCKNYMAVL
Q3ZBG4	PADIMEKNSSSPVAATPASVNTTPDKPKTASEHRKSSKPIM

Q9Z2H6	QTWHESERNCSGMSSHLVTINTEAEQNFVTQLLDKRFSYFL
Q58DF9	VEILAGEFNDSAASEMFIFHNGGVQILCKYPDTVRQFKMQL
P11456	GAGLVQINKSNGKETVVGRFNETQIFNGSNWIMLIYKGGDE
A6NLW8	KTNHRRCRTKFTEEQLKILINTFNQKPYPGYATKQKLALEI
P11234	REQILRVKAEEDKIPLLVVGNKSDLEERRQVPVEEARSKAE
P14415	KFHVNYTQPLVAVKFLNVTPNVEVNVECRINAANIATDDER
Q9CR48	CSSILYSSDFGPDVVQKLHWNPEDKGYVLHLVTTAAEWSMS
P22352	YVLFVNVASYUGLTGQYIELNALQEELAPFGLVILGFPCNQ
Q3T0Y9	VLERLARPGLEVPLRPQGLANPATLTAGQPREAGEEEQDAV
Q6PL45	PKPRLQTLRMTLPSPHMPRPNQTILVDVARNAATITVTPPQ
O14618	LLEGTGRQAVLKGMGSGQLQNLGAAVAILGGPGTVQGVVRF
Q8BPA8	NANPIFMRKDTKTSFQWRIRNLPYPKDVYSVSVAQKERCVI
Q96L33	HNPQAPVLLVGTQADLRDDVNVLIQLDQGGREGPVPQPQAQ
Q96K30	SLTHLNVPSTGHPATSAPHTNGPQDLRPSTSGVTFRSPLVT
Q58DH1	APFLSEKSTRQAPRGTNKTTNLSLKSNGKLRASSPVEDETA
P18181	VLDLIVTPQNKSTFYTCQVSNPVSSKNDTVYFTLPCDLARS
Q9H444	EFLEKKIEQELTAAKKHGTKNKRAALQALKRKKRYEKQLAQ
P21812	NLHDIMLLKLQKKAKETPSVNVIPLPRPSDFIKPGKMCRAA
P27105	AEAEASREARAKVIAAEGEMNASRALKEASMVITESPAALQ
Q9EP72	SGPPSYFIKRESWGWTDFLMNPMVMMMVLPLLIFVLLPKVV
Q5T7N8	QPKTHTHTGMHTQTHRERERNTQRLRDRERRENGRHTHRHT
Q17QI8	VQPSSFQNITEKWLPEIRTHNPQAPVLLVGTQADLRDDVNV
Q9UFN0	LVGVFHTEYGALNRVHVLWWNESADSRAAGRHKSHEDPRVV
P43431	QQIILDKGMLVAIDELMQSLNHNGETLRQKPPVGEADPYRV
Q08AT1	ELLALHAKETQRGYPALLLGNKLDMAQYRQVTKAEGAALAG
P01374	SLLWRANTDRAFLQDGFSLSNNSLLVPTSGIYFVYSQVVFS
P35330	TVPQEATATFNSTALKKDGLNFSCQAELDLRPHGGYIIRSI
Q4PNJ2	QYRPRYVTGYAVWLVLWVTWNVFVICFYLEAGDLSKETDLI
Q86SE9	FCKTCIVQHFEDSNDCPRCGNQVHETNPLEMLRLDNTLEEI
Q9JM90	VGKLDIIDLVCLTGQHSTEKNCAKFTLVLPKEEVHVKTENT
A1A4M2	RGSLLWNQQDGTLSLSQRQLNEEERGRLRDVAALNGLYRVR
P48060	WMVSFVSNYSHTANILPDIENEDFIKDCVRIHNKFRSEVKP
O70273	GIFRFLKSEAVAQLWGKKKNNSSMTYEKLSRAMRYYYKREI
Q9JIM5	APPEMFLPSDYGGQLFQPASNLDYYSQSPSVDTFDEEPPLL
Q96C74	KEKFKALLQLDPCENKIKWINFLALGCSMLGGSLNTALKHL
Q6Q8B3	RGQPSCTKAYKKETNETKETNCTVERITWVSRPDQNSDLQI
Q15800	LLPENPLQEPFKNAWNYMLNNYTKFQIATWGSLIVHEALYF
P35282	DEDSFQKVKNWVKELRKMLGNEICLCIVGNKIDLEKERHVS
Q6GPI1	QVLKIAKVFKNPKFSILTVNNDITLLKLATPARFSQTVSAV
Q8TDN7	SEVDWCESNFQYSELVAEFYNTFSNIPFFIFGPLMMLLMHP
Q9CWU6	RQLELLVEYVRKQMQYLDSMNGEDLLLTGEVRWRPLVEKNP

Q9HBM6	DRYCLTAPNYRLKSLIKKGPNQGRLVPRLSVGAVSSKPTTP
P20941	DSIPPSKKEILRQMSSPQSRNGKDSKERVSRKMSIQEYELI
Q3T004	LTLCLRAYSDLSRGYSLFSYNIHSKDNELLVFKNGIGEYSL
Q3ZC21	EEVDCLVLQLHRVGEQLEKMNGQRMDELFVLIRDGFLLPAG
P16563	SVNPTGSDILKMEWSIQATTNAQKWANKCILEHSSKDDRKI
Q3ZCI8	IVQAKERSYETVLSFGKRGLNIAASAAVQAATKSQGALAGK
Q7RTU4	RRMAANVRERKRILDYNEAFNALRRALRHDLGGKRLSKIAT
Q2KJA5	DFCLSILTLCSSYMEVPTYLNFKSMNHMNYLPSQDGMTHNQ
Q58DM3	CPKGSAVKGCYIQAKQWFHSNFLYIGITTICVCVIQVLGMS
Q06348	NLSEARVQVWFQNRRAKFRRNERAMLATRSASLLKSYGQEA
Q9EQU5	KEQQEAIEHIDEVQNEIDRLNEQASEEILKVEQKYNKLRQP
Q9D8S4	LDIEKDQIIEMACLITDSDLNILAEGPNLIIKQPDELLDSM
Q7TQB0	RLLLGSLIIGGLSAISSATGNTIALQMAACENYTIYYKMMA
Q9D902	YAFKPKYNLKDKKALLRLLDNHDQRGLGGILLEDIEEGLPN
Q8IUA0	WHFDFKNYRCTPFKYRGCEGNANNFLNEDACRTACMLIVKD
P36369	TRWQKSDDLQCVFITLLPNENCAKVYLQKVTDVMLCAGEMG
B5L3F2	YNAEHTISASISSVLKQTYANWELLVCDDDSSDNTRFKVLE
Q6GUQ1	WKKPHPGALTCDAICSKPCLNGGVCTGPDRCECAPGWGGKH
O60762	VRSPRQNKYSVLLPTYNERENLPLIVWLLVKSFSESGINYE
P58466	NYVKDLSRLGRDLRRVLILDNSPASYVFHPDNAVPVASWFD
Q99801	RAHLAKNLKLTETQVKIWFQNRRYKTKRKQLSSELGDLEKH
Q99JL1	PKMVEMHNYVPANSLQQKLSNWGHLNRKVLNKLNFSVPDDV
Q921N7	FSYSTSVVSLAFLPYLLSQNNMMFGSLPLQVLFYGVMGSFT
P39905	KGRRGQRGKNRGCVLTAIHLNVTDLGLGYETKEELIFRYCS
P06798	RIEIAHTLCLSERQVKIWFQNRRMKWKKDHKLPNTKMRSSN
Q2YDF1	IRALRAAPGGGFQNIACLRSNAMKHLPNFFHKGQLTKMFFL
Q96H79	IHAASLKLLQDQGLNIPSVVNFQIISTYKHMKLHKMLENTD
O14966	SKENGFTGWTETSVKENKNINEAMRVLIEKMMRNSTEDIMS
Q1LZ83	DTTYCVKKLPRNPERKEIIGNGEQQYVYVKDGDIIKTEGAT
P35737	WMKNGQLVPSHSNKEKTAQPNGDWTYQTVSYLALTPSYGDV
P70331	GKAPAVRIHRQTASPTCCLRNAQLSGTALRSLRLESQGHRE
P20290	TNQGTVIHFNNPKVQASLAANTFTITGHAETKQLTEMLPSI
P48540	IKRLKRSPDKQAAALPRRERNRQAAAASPENSRGKGRRGQR
P61019	FINTAKEIYEKIQEGVFDINNEANGIKIGPQHAATNATHAG
P97461	GRYAAKRFRKAQCPIVERLTNSMMMHGRNNGKKLMTVRIVK
P52823	NVCSIAKRNPEAITEVVQLPNHFSNRYYNRLVRSLLECDED
Q13491	LGVAWLGVFGFSAVPVFMFYNIWSTCEVIKSPQTNGTTGVE
Q9QYI6	FTNGKGQRGNGSPFEQSFNFNFDDLFKDFNFFGQNQNTRSK
Q32L26	VFDLNGDSFISKEEMFHMLKNSLLKQPSEEDPDEGIKDLVE
Q9R0M5	CGLIPVGQPCNQVTTTGLKWNLTNDVLGFGTLVSTSNTYDG
P46782	RKAQCPIVERLTNSMMMHGRNNGKKLMTVRIVKHAFEIIHL

Q9BUB7	LRRPGRAQIPVYWEGYVRFLNTPSDKSEDGRLIYTGNMARA
A1XBS5	EMLFHGKALEVYTAAYQNIQNIDEDEDLEVFRNSLYAPDYS
Q9BX51	TAHLSVVAEDGSAVSATSTINLYFGSKVRSPVSGILLNNEM
P35293	PELAATIGVDFKVKTISVDGNKAKLAIWDTAGQERFRTLTP
P15947	NWVLTAAHCHNDKYQVWLGKNNFLEDEPSAQHRLVSKAIPH
Q9BTE7	KEEWLKGMTSLQCDCTEKLQNKFDFLRSQLNDISSFKNIYR
Q8SPU8	SIVIVSSIAAYSPFPSLGPYNVSKTALLGLTKNLALELAES
P43025	TPQSELENEALFEYARHSVGNDANIWLGLNDMAAEGAWVDM
Q5FW52	GIAPQQKHGLALDEPARTESNSKASVLDLPVEHSSDSPSRP
Q9ES30	SSNHAVLKLAKGDEVWLRMGNGALHGDHQRFSTFAGFLLFE
O95999	PFFSTNSSLNLPVLEVGRTENTIFSSTTLPRPGDPGAPPLP
Q6IRU5	EKAKKDLEEWNQRQSEQVEKNKINNRIADKAFYQQPDADTI
P34927	VCVITSQNSQLREDLLALRQNFSNLTVSTEDQVKALSTQGS
Q6PB51	LWKPLPELLPEKPKPSSSPKNYRRESQAKHAAPGTAFPQRT
Q8NG50	QALALNSSKCQELANYYFGFNGCSKRIIKLQELSDLEEREN
Q9JHB3	KQYLLTGQILSDGKVFIHLCNYIEPWEDLSLVQRESLNHHY
Q9CPT3	QPGDCVMVGDTLETDIQGGLNAGLKATVWINKSGRVPLTSS
Q2YDD6	FCFLANQWHRSPPRYFLLGSNSAKAAITFSFFSILVWIFQA
Q91ZT9	ANPNALDGNRDTPLHWAAFKNNAECVRALLESGASVNALDY
O43240	PEAYGSPCARGSQPWQVSLFNGLSFHCAGVLVDQSWVLTAA
Q3ZBG0	KSVAKLQDERTVRKICALDDNVCMAFAGLTADARIVINRAR
Q61759	ICGGVLLNPNWVLTAAHCYGNQYNVWLGKNKLFQHESSAQH
Q9H2R5	QLRTTSRVIPHPRYEARSHRNDIMLLRLVQPARLNPQVRPA
Q96ET8	FITCMVTIILLLSCDFWAVKNVTGRLMVGLRWWNHIDEDGK
Q9CXS4	KAQHTFCKRCGVQSFYTPRSNPGGFGIAPHCLDEGTVRSVV
P52744	DKSLCMLSRLTQHKKIHTRENFYKCEECGKTFNWSTNLSKP
Q9UKR3	LELQSPVQLTGYIQTLPLSHNNRLTPGTTCRVSGWGTTTSP
P55041	ERTLVVDGESATIILLDMWENKGENEWLHDHCMQVGDAYLI
P19159	SCHTNSFHTPEERDKAQQMNNEDLSKWTLVLLYSWNNPLYY
Q9D6N1	QKITDILDSIKEKGKQTRFTNFDPLCLLPSSWDYWTYPGSL
Q3T0M9	FVNTVPTKGFNTEKIKVTLGNSKTVTFHFWDVGGQEKLRPL
P19159	ELRNMKNLSEAVISSAMEIENMSEKLQAFIESQFRKIIVPV
Q8TAY7	ARRQEPALRGSPGPLTPHPCNELGPPASPRTPRPVRRGSGR
Q4PNJ2	CVLERQIFDFLGYQWAPILANFVHIIIVILGLFGTIQYRPR
Q8N966	LRHDHHCFFTGNCIGSRNMRNFVLFCLYTSLACLYSMVAGV
O14798	SCTMTRDTVCQCKEGTFRNENSPEMCRKCSRCPSGEVQVSN
Q2YDD6	LDEGGVVLTSLSPPSAASPVNTPTTGPHGPSYASSSLSPYL
O35326	SRGGRGRGRYSDRFSSRRPRNDRRNAPPVRTENRLIVENLS
Q9CZJ6	ESYTLGSSEKQIVSEDKELFNLESRVEIEKSIKQMEEVLTA
Q8N6G2	GPRAPDPSLCHHNLQPTDDPNWDSYATTMRTAFTPKTGAVP
Q9D2Z4	NSNQAAGGTHWSLLVYLQDKNSFFHYDSHSRSNSIHAKQVA

Q16637	DFKRETCVVVYTGYGNREEQNLSDLLSPICEVANNIEQNAQ
P52793	EHQEYVACQPQSKDQVRWNCNRPSAKHGPEKLSEKFQRFTP
P27548	RRQFEDLVKDITLNKEEKKENSFEMQRGDEDPQIAAHVVSE
Q8IYI0	DLPSACDIRDYVLQGPSQEANSEAFSSLEFHSFPYSSDVDP
Q80X71	DAEKRTIYLNITNTLNITNNNYYSVEVENITAQVQFSKTVI
P14415	TGQPCVFIKMNRVINFYAGANQSMNVTCAGKRDEDAENLGN
Q5VWZ2	RILIGGFSMGGCMAIHLAYRNHQDVAGVFALSSFLNKASAV
P15374	FMKQTISNACGTIGLIHAIANNKDKMHFESGSTLKKFLEES
Q5SZD4	RYIANLISCFPSVCVRDEKGNPVSWSITDQFATMCHGYTLP
Q8BZJ7	SVDQKKLEQLYSRYKDPQDENKIGIDGIQQFCDDLSLDPAS
P10417	PFTARGRFATVVEELFRDGVNWGRIVAFFEFGGVMCVESVN
P01213	GSFLKELEKSKFLPSISTKENTLSKSLEEKLRGLSDGFREG
P29218	VYSCVEGKMYTARKGKGAFCNGQKLQVSQQEDITKSLLVTE
P15949	WVLTAAHCYYEENKVSLGKNNLYEEEPSAQHRLVSKSFLHP
P51572	EAFKKQAESASEAAKKYMEENDQLKKGAAVDGGKLDVGNAE
Q9R1Q6	YSDDSDWKTERCREYLNMMMNLFLAFCIMLTVVCILEIVVS
P52793	CLKLKVTVNGKITHNPQAHVNPQEKRLQADDPEVQVLHSIG
Q8CDN6	EDDIKEDGIVPLRYVKFQNVNSVTLFVQSNQGEEETTRISY
P00756	NWVLTAAHCYDDNYKVWLGKNNLFKDEPSAQHRFVSKAIPH
Q64669	LETVWEETPLYFAPSSLFDLNFQAGFLMKKEVQEEQKKNKF
O75489	KTYTDELTPIESAVSVFKAANWYEREIWDMFGVFFANHPDL
Q9Y478	RPTVFRWTGGGKEVYLSGSFNNWSKLPLTRSHNNFVAILDL
Q9QWZ1	LLKPSTKALALSCKVSIRTDNRGFLSLQYMIRNEDGQICFV
Q3KNK3	ENMKIGDTILFRGPTGRLFYNEPGTLLIKANKTSEPEKKLV
O95750	LLQYSEEDCAFEEEIRPDGYNVYRSEKHRLPVSLSSAKQRQ
Q2KIN3	YKSEVHKASLIIDLFGNEHDNFTKNLENLMSTIQESYCSNW
P07306	IGSQNSQLQEELRGLRETFSNFTASTEAQVKGLSTQGGNVG
O35658	SLDWALYDHLMDFLADRGVDNTFADELVELSTALEHQEYIT
Q8BHC1	SITRAYYRNSVGGLLLFDITNRRSFQNVHEWLEETKVHVQP
Q91VE3	IDGYKCKEGSHPWQVALLKGNQLHCGGVLVDKYWVLTAAHC
Q07507	ECWWEEINRAGMEWYQTCSNNGLVAGFQSRYFESVLDREWQ
O35723	SAEAIRKAYRKLALKWHPDKNPEHKEEAERRFKQVAQAYEV
P09586	ALLLLAVSNLLVWEKVTSLPNYRLPTESLYQRVIVVSHNAH
P53674	IKMDAQEHKISLFEGANFKGNTIEIQGDDAPSLWVYGFSDR
Q9UPY8	DGKDYNPLLARQGQDVAPPPNPGDQIFNKSKKLIGTAVPQR
Q0VD01	AQRMNRGRHEVGALVRACKANGVTDLLVVHEHRGTPVGLIV
P49863	TSDPQSNDIMLVKLQTAAKLNKHVKMLHIRSKTSLRSGTKC
Q8R2Y9	PTAPQATTGPPAASPASENQNGNGLSTQLGPVGGPHPSHTP
P52843	LGPDELDLVLKYSSFQAMKENNMSNFSLIKEDQVTNGLKLM
Q78IS1	LLLLRAEPLRSAELTFELPDNAKQCFHEEVEQGVKFSLDYQ
Q99390	MQTEDIISVINWAEKQECIDNQRIGLWGTSLGGGHVFSAAA

Q47098	GSALARASRWNRIPDYLQKANDQMCVLVQIETREAMKNLPQ
Q9DCT5	TVCERGTPIKCGQPIRLTHINTGRNLHSHHFTSPLSGNQEV
Q8NDB6	QEGPAPSQPSYSEQPMMGLSNLSPGPGPSQAVPLPEGLLRQ
Q9UKR8	PRSCCKSIGSVSCDGRDVSPNVIHQKGCFHKLLKITKTQSF
Q9CPY6	DVDRKHWGKFLAFYQYAKSFNSDDFDYEELKNGDYVFMRWK
A1E959	PLIPQRLMSASNSNELLLNLNNGQLLPLQLQGPLNSWIPPF
O35403	YYLVPKGLKDKKAKILYMYRNPKDVLISYFHFSNLMLIFQN
Q9Y4Z2	QRRSRRKKANDRERNRMHNLNSALDALRGVLPTFPDDAKLT
Q3UV48	NIAESIQCLASLQIFIAESNNIHSFPRSLCLVTSLELLNLN
Q8R4X1	RAKMSSIFAYQSSEVDWCESNFQHSELVAEFYNTFSNVFFL
Q8BV13	RNQLLEVDFCIGRDIRKKDINNIVKTLHEWCDGCEAVLLGI
Q9UKR3	SGWGTTTSPQVNYPKTLQCANIQLRSDEECRQVYPGKITDN
Q96CF2	KKMTNIRLPNVPSSSLPAQPNRKPGMSSTARRSRAASSQRA
Q7Z4M0	PGPCLEAGTAPCPTWKVFDSNEESGYLVLTIVISGHFFIFQ
Q8NH89	SNKINHFFCDGLPILALSCSNIDINIILDVVFVGFDLMFTE
A5PJG7	TGAKAANKENLDPSNLMPPPNQTPAPDQPFPLSTVREESSI
P53004	RYLSFHFKSGSLENVPNVGVNKNIFLKDQNIFVQKLLGQFS
Q5SX19	HANGTDLVVTFDAEGVSGHSNHIALYKAVRALHSGGKLPKG
P82970	EAKITEAPASEKEIVEVKEENIEDATEKGGEKKEAVAAEVK
Q9CQD1	MNVNEIFMAIAKKLPKNEPQNPGANSARGRGVDLTEPAQPA
Q53HL2	VEIRIKQIESDRQNLLKEVDNLYNIEILRLPKALREMNWLD
O75323	EVLPKIHEDKHYPCTLVGTWNTWYGEQDQAVHLWRYEGGYP
Q8TBZ9	HVKDETVFFIQQMKIIVISHNIPMRLFMQWHVSVIKVPVMY
Q5TGL8	SEVVLTFFERSPLDQVLKNDNVHKIQPSFQSPVKISEIMRS
A5D7N9	ASQEDTNCILLRCVTCNVSVNEEQILSKRKNENGCILETLY
Q91X79	MVNGQYAVHGVTSFVSSMGCNVARKPTVFTRVSAYISWMNN
P0CG30	ELFLDLVSQPSRAVYIFAKKNGIPLELRTVDLVKGQHKSKE
Q7L2Z9	QKTLKAPTLQKEILALIPNQNALLKDLDILHNSSQMKSMST
Q9TT89	AAGYGLEPSSFNMHCAPFEQNLSGVCPGDSAKAAGAKEQRD
Q58CY6	GELYLDESKQFYKELGFKRYNSLSILPAALGKPVREVAAKA
A6QPM6	EPGLDLSLSPRSESPGRGRPNCSPGRRKGRADRRGGARKGR
P47756	KLTSTVMLWLQTNKSGSGTMNLGGSLTRQMEKDETVSDCSP
Q58DS5	AFSSLARDILLKSGGRRLKNNNKPPSTDLKTCDKKNTNKCS
Q3UMZ3	DSKQACLAASLALALNGVFTNIIKLIVGRPRPDFFYRCFPD
Q9QWZ1	QITVSPDKPYFRLSTFGNAGNSHLDYPKDSDLVEAFHCDKT
P35288	EGLAKRLKLRFYRTSVKEDLNVSEVFKYLAEKHLQKLKQQI
P14207	IQDTCLYECSPNLGPWIQQVNQSWRKERFLDVPLCKEDCQR
Q99865	TKKKVSQKKQRGRPSSQPRRNIVGCRISHGWKEGDEPITQW
Q3SZ45	VSAFGEEGEGDYLDDWTVLCNGPYWVRDGEVRFKHSSTEVL
Q91WK7	QIVQLLLDHGADPNQQDGLGNTPLHLAACTNHVPVITTLLR
Q3ZCA1	GRVVNSWYRYRPLVAELVVQNACGHLGLKSEEICWTNSESF

Q8BZ09	LGFQGLNKGLTATLGRHGIFNMVYFGFYHNVKNIIPSSKDP
Q9D853	DYSPSAIKLSASILEKEGLSNINLKVEDFLNPSTKLSGFHV
Q9ESN4	TYHVLMRGGDGTSMWADLCKNNQVRASAIAQDADQNYDYAS
P70245	CWFAVCTFIHLVIEGWFSLYNGILLEDQAFLSQLWKEYSKG
Q9D0M0	ANATPEFEGRGGDDLGTEIANTLYRIFNNKSSVDLRSLCIS
Q5JTZ5	LPKGGNTPAPAESMVNAVWINKERRSSLSLEEADSEVEGRL
P21796	KLDLKTKSENGLEFTSSGSANTETTKVTGSLETKYRWTEYG
P05631	RAGVAGLSAWTVQPQWIQVRNMATLKDITRRLKSIKNIQKI
Q9UBY8	LYLPHLTLFLVGLALLTLIINPYWTHKKTQQLLNPVDWNFA
Q8IUE1	SEEEKQMLSEKTNLSLLQISNWFINARRRILPDMLQQRRND
Q96S44	TVRDYIQSTMETEKTPQGLSNLAKTIGQVLARMHDEDLIHG
Q96CS7	QAYAVPYQYPYAGLYGQQPANQVIIRERYRDNDSDLALGML
P29368	DGERGRLVPAMQTEDIISVINWAEKQECIDNQRIGLWGTSL
Q7Z2W9	VVLPDPVEETRHHAEVVKKVNEMIVTGQYGRLFAVVHFASR
Q8BRJ4	VGGTLLFALRYRVTGREFWDNNGGRDYALLGPEHPAGAGAA
Q29438	PCSPCNPCNPCSPCSPCSPCNPCDPCNPCYPCGSRFSCRKM
P15947	AQHRLVSKAIPHPDFNMSLLNEHTPQPEDDYSNDLMLLRLK
Q32PI9	PPFKDRVSWAGDLDKKDASINIENMQFIHNGTYICDVKNPP
Q05433	KGAEEGISLQSRPDGRTMLVNTTPYIFAIGSLLDGNGKRIA
Q9HAE3	DDMIMDRVFRGFDKDNDGCVNVLEWIHGLSLFLRGSLEEKM
P31268	SPLYQSPFASGYGLGADAYGNLPCASYDQNIPGLCSDLAKG
P48758	ELQQKFRSETITEEELVGLMNKFVEDTKKGVHAEEGWPNSA
Q969X5	LFILFLFLSELTGFITTEVVNELYVDDPDKDSGGKIDVSLN
Q9JKT4	GLGVLGNTFIALVNCMDWAKNNKLSMTGFLLIGLATSRIFI
Q9P299	SSYENELMLMSVLTCLFESLNHMLRKNVEKRWLLENMDGAF
Q6ZTZ1	AAEGPGYLVSPQAEKHRRARNWTDAEMRGLMLVWEEFFDEL
Q6PHZ8	ESISAQLEEASSTGGFLYAQNNTKRSIKERLMKLLPCSAAK
Q6PEX7	PDVLWDLDIPEGRSHADQDSNPKAEAPAPLQPALQLAPQQP
P00551	QAQSRMNNGLVDASDFDDERNGWPVEQVWKEMHKLLPFSPD
P81623	ASSDDLLVAEVGISDYGDKLNMELSEKYKLDKENYPIFYLF
Q68D42	VFLVFGFMFTVSGMKGETLGNIPLLAIGPAICLPGIAAIAL
Q9Y294	VLITCTYRGQEFIRVGYYVNNEYTETELRENPPVKPDFSKL
Q8N2U9	TLGFLAVLTEAMLGVPQLYRNHRHQSTEGMSIKMVLMWTSG
P61267	DIKMDSQMTKQALNEIETRHNEIIKLETSIRELHDMFVDMA
P62070	FQRQILRVKDRDEFPMILIGNKADLDHQRQVTQEEGQQLAR
Q9D676	EQILINKTYAACPKNWIGVGNKCFYFSEYTSNWTFAQTFCM
O43423	LRKLELRVSGGLEVLAEKCPNLTHLYLSGNKIKDLSTIEPL
P46638	YRGAVGALLVYDIAKHLTYENVERWLKELRDHADSNIVIML
Q9DCT2	LADLTAVDVPTRQNRFEIVYNLLSLRFNSRIRVKTYADELT
Q9H160	SSRRPRRQRTSESRDLCHMANGIEDCDDQPPKEKKSKSAKK
Q9NQ35	NLSKLRSGPRVPWASKTNKLNQAKSEGLKKSEEDDMILVSC

Q9H6H4	IVQAKERSYETVLSFGKRGLNIAASAAVQAATKSQGALAGR
Q9ESN5	IRGIKEFKEKLLLTLGAFLDNHFPLPEASTPKKRKNIQDSN
P20108	YGVLLESAGIALRGLFIIDPNGVVKHLSVNDLPVGRSVEET
Q0VCT9	GMGQFPSPHHHQQQQPQHAFNALMGEHIHYGASNMNASSGI
Q8IWL3	IPERTDYEMDRQFLIEIMEINEKLAEAESEAAMKEIESIVK
P18917	DEQYAQGKEYFINVSDKCHTNSLHLPEDMQHNPTDEQKGLI
Q9H160	QETLKEIDDVYEKYKKEDDLNQKKRLQQLLQRALINSQELG
Q9NP95	GSEKLTSECIFREQFEENWYNTYSSNIYKHGDTGRRYFVAL
A6QL48	LDVKQGLGGVEVSPQVEAVLNLLSAPGSLKLVRPKALLDNC
Q99807	ISRAAVDRIIRVDHAGEYGANRIYAGQMAVLGRTSVGPVIQ
O54917	LVDPAQETVRRRCRDPINVENLLPSKIRINLEENVQYVSMR
Q3T0E2	LFQQWDIDMQKAEEQEEKLANLFRQQQKVFQQSRIVQSQRL
Q68D42	ASSINSPTPTEEGECQSLVQNGHQEETSRYLDGYCPSGSSL
Q3T054	FEKKYVATLGVEVHPLVFHTNRGPIKFNVWDTAGQEKFGGL
Q15181	YKGYIWNYGAIPQTWEDPGHNDKHTGCCGDNDPIDVCEIGS
P25731	KPEKQDGLSKNKSLKTDLSVNIIYAALIRVLPKDGKSDMRA
P68252	QKARLAEQAERYDDMAAAMKNVTELNEPLSNEERNLLSVAY
A6NCJ1	AWEAWYNLPRAPASPFREAYNRWHSCYQHRECSMPSAYTQH
A7E1Z1	PGIAAIALARKTEGCTKWPENELLWVRKLPCFRKPKDKEVV
P32442	GTIANETEKKSSRRKKERSDNQENGGGKPEGSSKARKERTA
B4E2M5	DWDAKKRELELSLPSLNQNMNKKNKKSRGPTRPSNTKGRRV
Q92915	AREQHWDRPSASRRRSSPSKNRGLCNGNLVDIFSKVRIFGL
Q2T9X7	ELAEITLDPPPNCSAGPKGDNIYEWRSTILGPPGSVYEGGV
Q9H3H3	AWLAKYPPSQVTRYGDPGSPNSEPVGWIAVYGQGYSPNSGD
Q8BFR6	MFFCLRWSIGKVVDFAASLANLRNENNKLTAKKLRLCHVPS
Q92564	KYKVINKDQWCNVLEFSRTINLDLSNYDEDGAWPVLLDEFV
Q91XB7	PAPGADVAFSVNNLLGDPVANMAMAYGTSIASQGKDIVHKE
P22090	TGNLCMVIGGANLGRVGVITNRERHPGSFDVVHVKDANGNS
P59529	NLPKNNSLILRLQQFEWYFSNPLKMIGFGIPFFVFLASIIL
Q96LT6	NLCTVFGILLGYPVPYTFHLNQGDDNCLALTPLRVFTARIS
C9J302	PPNYGKYCVRPKKPAQEALINYSRRGKGVLKHLHGRCDSES
P15814	LFPPSSEELQANKATLVCLMNDFYPGILTVTWKADGTPITQ
P0CH99	KCYECDKSGKAFSQSSGFRGNKIIHIGEKPHACLLCGKAFS
Q5E9E6	AVGHVKMTDDELVYNIHLAVNFLVSLLKKNWQNVRALYIKS
Q9HBM6	NPSMIGPKNILITTNMVSSQNTANEANPLKRKHEDDDDNDI
Q9NVS9	PSARMLLLKGFGKDGFRFFTNFESRKGKELDSNPFASLVFY
Q32LD3	GLSIASYSRVCRTYLCNNLTNMNAILHLKARTPKTLKSSSH
Q3T0Z7	GLPPGAAAVALLPVTLDTPVNRKSMPEADFSSWTPLEFLVE
Q86VX2	SELEKVGSIFLQLKLVVKKGNQTENVYIELTLPQFYSFLHE
Q9BR61	VKKLDPGWNPQIPEKKGKEANTGFGGPVISSLYHEETIREE
O08545	GGGAEQYVLYMVNLSGYRTCNASQGSKRWECNRQHASHSPI

Q9Y6H3	AMKHSGCAVNKDRHFSCEDCNGNVSGGFDASTSQIVLCQNN
O00299	LCPGGQLPFLLYGTEVHTDTNKIEEFLEAVLCPPRYPKLAA
Q8K2Q2	KSKPDWYYTKHPFGQIPVLENSQCQLVYESVIACEYLDDVY
O35129	PSMYQRLGLDYEERVLPSIVNEVLKSVVAKFNASQLITQRA
Q9BYD2	EDTKHRPKENLELILTQSVENVGVRGDLVSVKKSLGRNRLL
Q9DAS4	KKKEIFPSWELLSFLKSSSRNSKFLAMFNLSHCLEYDTQFF
O15318	VLKKMEELEKRGDGEKSDEENEEKEGSKEKSKEGDDDDDDD
Q8VE04	RKFEPGTQRFELHKRAQASLNSGVDLRAAVQLPNGEDQNDW
Q91YJ3	VGLMKIVKEAYPDHTQFEKSNPHYDPSSKEDDPKWSMVDVQ
Q00059	KPVSSYLRFSKEQLPIFKAQNPDAKTTELIRRIAQRWRELP
Q86UD7	LAVRGRAWSLLLDIDRIKSQNPGKYKVMKEKGKRSSRIIHC
P25393	EKIIESIYISSVSFFSDKVRNVIEKDLSRKWTLGIIADAFN
Q64362	PKNALPIAKPTSPAPAAQSTNGTHASYGPFYLEYSLLAEFT
Q15404	GFGSLPALEVLDLTYNNLSENSLPGNFFYLTTLRALYLSDN
Q7RTV5	FDFQGDPAQQGGTLILGPGNNIHFIHRDRNRLDHKPINSVL
Q1RMR0	VERYSDKYQMSGPIDNAIDWNPDWRRLPRELKIRVRKLQKE
P82970	DTSAQAVAETKQEAVVEEDYNENAKNGEAKITEAPASEKEI
O43704	TVMDHSKSPFMRKGTAGDWKNYFTVAQNEKFDAIYETEMSK
O95157	HSPPPSAKVKKIFGWGDFYSNIKTVALNLLVTGKIVDHGNG
Q9BS92	FHIWKYDNFAHRAEVRKALANCKEWQEQSIIPNLARIDKQE
Q9ZIS7	IFNYASVFIMLIEYVEGVELNDMPIIPENVKAEIKASMEKL
P32243	REEVALKINLPESRVQVWFKNRRAKCRQQQQQQQNGGQNKV
Q9P013	QKAEEERIRMENILSGNPLLNLTGPSQPQANFKVKRRWDDD
P11226	IKKWLTFSLGKQVGNKFFLTNGEIMTFEKVKALCVKFQASV
Q5E982	QNGMHTLCLLDIKVKEQSLENLIKGRKIYEPPRYMSVNQAA
Q8N4L2	QSLINLDGKLHQHVVKCTVCNEATPIKNPPTGKKYVRCPCN
A6H789	SMCPKGCLCSSSGGLNVTCSNANLKEIPRDLPPETVLLYLD
P49755	LLLFLLGPRLVLAISFHLPINSRKCLREEIHKDLLVTGAYE
Q8BHE1	RCEEEELESDSDDEVECDLSNMEITEELRQYFAQTERHREE
P11843	TIYDQENFQGKRMEFTSSCPNVSERNFDNVRSLKVECGAWV
Q3MHX1	FMTLGLSEEKAIYFSEKWKQNAPTLARWAVGQTLMINQLVD
Q91YY4	MGPSIPTQTREVLTSHLSSYNMWALQGIEFVVAQLKSMLLT
P49638	FSMIKPFLTEKIKERIHMHGNNYKQSLLQHFPDILPLEYGG
Q8TED1	DAKGRTVSLEKYKGKVSLVVNVASDCQLTDRNYLGLKELHK
Q8WVL7	TGTQSLWVGNSDEDEEQDDKNEEWYRLQEKKMEKDPSRLLL
Q80ZQ3	RDYQKQFNVTHLLALCDYSHNTFIRHYRLYQYVLSQDQEVN
A4QPB2	DEVWAIRRAYLDGSGAQTLINTKINDPDDIAVNWVARSLYW
Q0VC33	YKVEQDDWLTVYLKYLLFIFNFFFWVGGAAVMAVGVWTLVE
Q969E2	CGYVCWFRPVYKAFRADSSFNFMAFFFIFGAQFVLTVIQAI
Q9Y6N1	FEAGQYFNKIQCFCFEEQRLNPQEEVDMPVFFYIDPEFAED
P17483	EPGQRCEAVSSSPPPPPCAQNPLHPSPSHSACKEPVVYPWM

P04229	LLVCSVSGFYPGSIEVRWFRNGQEEKAGVVSTGLIQNGDWT
P0C7T8	ELRRAPRLWKVRAMMIFNTFNLILGFIVVVVEVMKTALGPA
Q17QL1	PESYHSYMWNNFFKHIDIDPNNAHILDGNATDLQAECDAFE
Q8QZZ8	LVIGDLGVGKTSIIKRYVHQNFSSHYRATIGVDFALKVLHW
P18021	WYVSQLQTMVATLKIPMERRNKRTGRTEKARIWEVTDRTVR
Q2KIS7	FSLLTQVTAETPTPKAKKAANAKKDAVSPKMLEELKTQLDS
Q9D7L8	QLLLVVLSLPQGRTSSVLTVNGRTENYILDTQHGVQASLEC
P01033	AGKLQDGLLHITTCSFVAPWNSLSLAQRRGFTKTYTVGCEE
O35969	YDTYPLSEEAWHTHQFNFIKNHAFRLLKTGGVLTYCNLTSW
Q96G30	EEELNRLMKFDIPNFVNTDQNYFGEDDLLISEPPIVLETKP
P48023	VYFRGQSCNNLPLSHKVYMRNSKYPQDLVMMEGKMMSYCTT
P32972	LKSTPSKKSWAYLQVSKHLNNTKLSWNEDGTIHGLIYQDGN
Q9H6K1	IGEGESIPPDTQFVKTWRIQNSGAEAWPPGVCLKYVGGDQF
P09496	PGPQPHGEPPGGPDAVDGVMNGEYYQESNGPTDSYAAISQV
Q8K1I3	ESNSSEEMMFGDMARSHRRRNDYLLGFLSDESRSEQFRDRS
Q29438	ELAKLRRTTNRILASSCCSSNILGSVNVCGFEPDQVKVRVK
Q80V24	ISPSKRKFSMEPGDKDLDCENDHVSKMSRIFSPHLNKTVNG
Q2HJI8	YRGAMGIMLVYDITNEKSFDNIKNWIRNIEEHASSDVERMI
Q8R0F8	LEEGDLILTGTPKGVGPIKENDEIEAGIDGVVSMRFKVKRS
Q80ZS3	EQAWVQLKEQEVLKLQEEAKNFITRENLEARIEEALDSPKS
D3Z1Q2	TKTGAPHQDNAESSERRFRMNSFVSDFGKPLESDKVFSRQG
Q8CEZ1	EHYAANVRRMYIKDVSFMITNMVKNQALQDGLLRAVQIIEK
A6QPA3	PGLVVHGEAAPFSTALRSLVNNPLYSDVRFVVGQERQEVFA
Q9NW81	EVPVEAVDAGDCDINYEGLDNLLRLKELQSLSLQRCCHVDD
Q0II87	KPMTSYVRFSKEQLPIFKAQNPDAKNSELIKKIAKLWRELP
Q9GZW8	SFTPKGITIPQREKPGHMYQNEDYLQNGLPTETTVLGTVQI
Q9D0Z3	EAGKQQPVVILLGWGGCRDKNLAKYSAIYHKRGCIVIRYTA
P62593	AFLHNMGDHVTRLDRWEPELNEAIPNDERDTTMPAAMATTL
Q14249	FLPRTEADGKSYVKYQVIGKNHVAVPTHFFKVLILEAAGGQ
P04227	TWLRNSKSVTDGVYETSFLVNRDHSFHKLSYLTFIPSDDDI
Q2TBT8	CPNEGCMLRVPRGALDEHRQNCQHGAYHRCSLGCGATLGPV
Q8NHR7	IRTPVVEKQMYFPLQNYPVNNMVTGYISIDAMKKFLGELHD
Q2KIS9	ELQEELKCCGLVNGASDWGSNFQHYYRTCECPSESDSSCTK
Q3SZY7	HSQVPMLCSTGCGFYGNPRTNGMCSVCYKEHLQRQNSSNGR
Q9DCY0	QQIQTLEKLGFPMYAHVDKANFTVQRMVGLLGQILLPCTWN
Q8WTT0	WVDQTPYNENVTFWHSGEPNNLDERCAIINFRSSEEWGWND
P23150	GQLTSFDPQGGLQEIATGKYNLEILIKDSNFTPAANEAPQA
Q58DC7	IVASLLGAHVTATDLPELLGNLQYNISRNTKTKAKHLPQVK
Q32KN7	TDIPTVGKAPQSKPQPVRKSNWRQHHEDFINAIQSAKQCTL
Q12918	KCSVDIQQSRNKTTERPGLLNCPIYWQQLREKCLLFSHTVN
Q32KP7	PQKKKLKYMKQSLVVLGDHINTFLQHYCRAWEIKHWKKMLW

P97823	IKSPHIKYICPHAPVMPVTLNMNMAMPSWFDIVGLSPDSQE
P48060	WPYKRGATCSACPNNDKCLDNLCVNRQRDQVKRYYSVVYPG
Q810M6	YRLMALKCFHSDPCVEESVENLPYLSDKDCSPCSKQPSSKG
O15492	LGCDTGSTGKFEWGSKHSKENRNFSEDVLGWRESFDLLLSS
P53811	SVAEASKNETGGGEGIEVLKNEPYENDGEKGQYTHKIYHLK
P20181	KHWNSQCKTSQTYVRALTSENNKLVGWRWIRIDTSCVCALS
P28033	MRRERNNIAVRKSRDKAKMRNLETQHKVLELTAENERLQKK
Q99NH8	NGSTVIADDTLAGTVTITLKNLQAGDAGLYQCQSLRGREAE
P15246	LIDQLKPGGRLILPVGPAGGNQMLEQYDKLQDGSVKMKPLM
P58340	GRDLLSISDGRGRAHNRRGHNDGEDSLTHTDVSSFQTMDQM
Q9WV93	IIEGLDASDPLLVRLVSHLNNYASQREAASGAHGGLGHIPW
P14483	SLRRLEQPNVVISLSRTEALNHHNTLVCSVTDFYPAKIKVR
O60238	EVEALKKSADWVSDWSSRPENIPPKEFHFRHPKRSVSLSMR
Q5EA92	GKQTEFAPETGKREKRRLTKNATAGSDRQVIPAKSKVYDSQ
Q8BHH9	NGHDFRGRCLLFTEGMWLSANLTMQGRERFTVQEWGPPAAC
Q9CR78	QIERECEMAEEEHRIKMEVLNKKKMYWERKLQTFTKEWPVS
A7MAZ5	VSKGTLVQTKGTGASGSFKLNKKAATGEAKPKGKKAGAAKP
Q3MHP2	RAFAEKNNLSFIETSALDSTNVEEAFKNILTEIYRIVSQKQ
B9EJG8	LVALCLASFGMTLLGNFQLTNDEEIHNVGTSLTFGFGTLTC
Q9BW60	FLMSGWLSTYTWRCDPVDYSNSPEALRMVRVAWLFLFSKFI
Q9UKR3	ELQSPVQLTGYIQTLPLSHNNRLTPGTTCRVSGWGTTTSPQ
Q61133	YPADLQARAQVHEYLGWHADNIRGTFGVLLWTKVLGPLIGV
Q5ND56	YGQQKGLSLLQVPFNIPLHCNMANAVLISPQLYWFSLLCKK
Q8IUX1	QNIYQMATFGTTAGFSGIFSNFLFRRCFKVKHDALKTYASL
O43711	ASAQTPHPHEPISFGIDQILNSPDQDSAPAPRGPDGASYLG
Q17QQ4	MPTSQSPAVKASIPATSAVQNVLINPSLIGSKNILITTNMV
P17677	SSEEKAGSAETESATKASTDNSPSSKAEDAPAKEEPKQADV
P70377	QQSGRGWYLGLNKEGEIMKGNHVKKNKPAAHFLPKPLKVAM
Q9ES30	SLATHFSNQNSGIIFSSVETNIGNFFDVMTGRFGAPVSGVY
Q3ZC64	CLRLKVMIAGKITHSPQAHPNAQEKRLPADDPEVQVLHSIG
Q8BGX2	VDFPGRILDVGFVGRWWILQNRMHDCDINDDEFLHLPAHLR
Q6FIF0	GFECRCGNVYCGVHRYSDVHNCSYNYKADAAEKIRKENPVV
A6H789	IDEHAFKGVAETLQTLDLSDNRIQSVHKNAFNNLKARARIA
B6A8C7	KPLDSTEGAAEFHLNNLKVRNAGEYTCEYYRKASPHILSQR
P0C7V9	ERGSSLRKDGPLDIRMDGGRNISSLCYLYTERLTTAIYLYC
P19427	TECWWEEINRAGMEWYQTCSNNGLVAGFQSRYFESVLDREW
P29692	KIWFDKFKYDDAERRFYEQMNGPVAGASRQENGASVILRDI
O54908	LCSLPLLGASATLNSVLINSNAIKNLPPPLGGAGGQPGSAV
Q08D99	LFCSAYLYKQSFAIPGSSFLNVLAGALFGPWLGLLLCCVLT
Q5NCY3	RTPRGRFVHIPPPLPRSDWANDFGVPWWKGANYQVGRLSAR
Q9NXJ0	NQANTTTNMSVLVIPNMYESNPVTPASSSAPPRCNNYSANA

Q9JKF4	FWHPHEPNLPEERCVSIVYWNPSKWGWNDVFCDSKHNSICE
Q9BZM6	LHYDCVNHKAKAFASLGKKVNVTKTWEEQTETLRDVVDFLK
Q92913	QQSGRGWYLGLNKEGEIMKGNHVKKNKPAAHFLPKPLKVAM
Q96KN4	TPCPESPSRHHHHLLHQLVLNETQFSAFRGQECIFSKVSGG
Q9BPX1	KALALDESPYGVRVNCISPGNIWTPLWEELAALMPDPRATI
Q2T9Q7	GQDLWEESSWSNQRWSRTAPNPRGARARSLARGRSEASPEN
P08962	EVAAAIAGYVFRDKVMSEFNNNFRQQMENYPKNNHTASILD
P97372	EMETDKQEKKEVPKCGYLPGNEKLLALLALVKPEVWTLKEK
P01193	EHFRWGKPVGKKRRPVKVYPNVAENESAEAFPLEFKRELEG
Q9NRE1	HDMKPSAVKDSIYNAVSIWSNVTPLIFQQVQNGDADIKVSF
Q8IUQ4	NCRPKLTCCPTCRGPLGSIRNLAMEKVANSVLFPCKYASSG
Q9BGI2	HLSKAKISKPAPYWEGTAVINGEFKELKLTDYRGKYLVFFF
P04179	EKLTAASVGVQGSGWGWLGFNKERGHLQIAACPNQDPLQGT
P20826	CIYLQLLLFNPLVKTKEICGNPVTDNVKDITKLVANLPNDY
P13366	NARVKPGDVCSVAGWGKTSINATKASARLREAQLIIQEDEE
Q92904	VKIITDRTGVSKGYGFVSFFNDVDVQKIVESQINFHGKKLK
Q8VCC1	RKVVDHFGRLDILVNNAGVNNEKNWEQTLQINLVSVISGTY
Q9HD15	QAEVEMAELYVKPGNKERGWNDPPQFSYGLQTQAGGPRRSL
Q24K21	DTKMSFQWRIRNLPYPKDVYNVFVDQKERCVVVRTTNKKYY
Q14CZ0	CQQPGLSLWVPFQNAATAVTNLYKESVDTHQRSFDIGIQIG
P28907	NGSRSKIFDKNSTFGSVEVHNLQPEKVQTLEAWVIHGGRED
P39687	LELSDNRVSGGLEVLAEKCPNLTHLNLSGNKIKDLSTIEPL
Q9HAV7	LVMTEVQIQKVFTKHGLLKLNPVGAKFDPYEHEALFHTPVE
Q9BS16	EEFQKLRQDLEMVLSTKESKNEKLKEDLEREQRWLDEQQQI
Q99LJ8	QQQELLGQDCSKYSAEFANSNDKDDQDLNCPSAVKVLSPED
P09326	MPHNYSRCYTCQVSNSVSSKNGTVCLSPPCTLARSFGVEWI
Q0VD18	DTSNPNCVVIADAGEGFSYQNMNKAFQVLMELENPVLFSLG
O18870	FLPPPPHMPRSGLGPGKSGLNFSGPPPPPPPPPHFLSRWLP
Q8JZV9	QKSGNIINMSSVASSIKGVENRCVYSATKAAVIGLTKSVAA
Q99LZ3	SEEMVLTPAELIEKLEQAWMNEKFAPELLESKAEIVECVME
Q9JIW9	EVQIDILDTAGQEDYAAIRDNYFRSGEGFLLVFSITEHESF
Q8BN57	KEDSALFCYLLVNKGGYFNVNLNEEILRRGLGKTVLVKGLN
Q9HB71	GSSKKVKTDTVLILCRKKVENTRWDYLTQVEKECKEKEKPS
Q6UWP2	ILSMFSAIRSQHSGVDICINNAGLARPDTLLSGSTSGWKDM
Q12962	PGPAPPVSAPAALPSSTAAENKASPAGTAGGPGAGAAAGGT
P47804	SISPKLQMVPALIAKMVPTINAINYALGNEMVCRGIWQCLS
Q46668	VPSPDEPGLPLPGPGPALPTNGAIPIPEPGTAPAVSLMNMD
Q4G0S7	LLKVMQAKEVSIKEECATLHNIIKGLQQTIEYQQNLKGENE
P17081	KCVVVGDGAVGKTCLLMSYANDAFPEEYVPTVFDHYAVSVT
Q8C8S3	SAGLWIVYFIAVEDDKILPLNSAARKSGAKHAPYISFAGDD
Q9ESK4	SSRRPRRQRTSESRDLCHMTNGIDDCDDQPPKEKRSKSAKK

Q96FV3	GILAFVFKDWIRDQLNLFINNNVKAYRDDIDLQNLIDFAQE
Q61133	TVDILKGQHMSEQFSQVNCLNKVPVLKDGSFVLTESTAILI
Q64329	KIFQYNQHKQEINETLNHHHNCSNMQRAFNLKEEMLTNKSI
Q14696	PTEKETEEITSLWQGSLFNANYDVQRFIVGSDRAIFMLRDG
P18669	DQLPSCESLKDTIARALPFWNEEIVPQIKEGKRVLIAAHGN
Q9Y3A2	KKIERLKSELHLLDFQGKQQNKHVFFFDTKKEVEQFDVATH
Q8N9N7	SMLPQSILSDSQICLLAVEGNLFEIKKLRELEGYDKYMERF
P49888	EKCKEDVIFNRIPFLECRKENLMNGVKQLDEMNSPRIVKTH
Q2TBL9	LGNPANYPVSIRFGRPRLTSNEKLMLASMFHSLFAIGSQLS
P02663	INPSKENLCSTFCKEVVRNANEEEYSIGSSSEESAEVATEE
Q80YT9	YGRPINVQYRFGSSRSSEPANQSFESCAKINSHSFRNDEMA
P97823	DPLVPLMFGSLTVERLKALINPANVTFKIYEGMMHSSCQQE
P00848	NITAGHLLMHLIGGATLVLMNISPPTATITFIILLLLTILE
Q9DC58	FNLVSLALGLVGCIGMGIVANFQELAVPVVHDGGALLAFVC
O43396	ITVAFNQPVKLYSMKFQGPDNGQGPKYVKIFINLPRSMDFE
P15319	EFETVMLSPRSEQTVKSANYNTPYLSYINDYGGRPVLSFIC
Q32KU2	TSTVKPGTICMLSGLDWSQNNNGRHPDLRQNLEAPVMSDTA
Q9BXY0	KVIERAAFPRRLWERVRLSKNYEKALEQIDENLIYWPRFIR
Q9Y235	ASRGYLEDEHAAAHAEEAFFNTILPAFDPALRYNVTWYVSS
P40198	YSGRETIYTNASLLIQNVTQNDIGFYTLQVIKSDLVNEEAT
Q3SX30	MIINVLEHGSQQVSDLTLNLNDYIDSEHLVDFHRVYKNSEE
Q96FE7	DQKVCEREMQRITLPLSAFTNPTCEIVDEKTVVVHTSQTPV
Q9Y3B3	DYQTHFRLREAQGRSRAEDLNTRVAYWSVGEALILLVVSIG
Q8C767	SLPEKIQSYERMEFAVCYECNGQAYWDSNKGKNYRITRAEL
O95073	DPQILQMLKEEHQIILENQKNFGLYVQEKRDGLKRRQQLEE
P32850	ISKQALSEIETRHSEIIKLENSIRELHDMFMDMAMLVESQG
P55789	PDTRTRACFTQWLCHLHNEVNRKLGKPDFDCSKVDERWRDG
Q47066	QQQLEALEKSSGGRLGVALINTADNSQILYRADERFAMCST
Q3KPI0	IQASSTTVTEKGSVVLTCHTNNTGTSFQWIFNNQRLQVTKR
P08057	ETLDLRAHLKQVKKEDTEKENREVGDWRKNIDALSGMEGRK
P14435	TLICFVDNIFPPVINITWLRNSKSVTDGVYETSFLVNRDHS
Q8IXQ8	RDDKKEVRAPSPYWIMVKQDNESSSSSSTSSTSDAFWLEDCA
Q15102	ENGELEHIRPKIVVVWVGTNNHGHTAEQVTGGIKAIVQLVN
P08294	VVVHAGEDDLGRGGNQASVENGNAGRRLACCVVGVCGPGLW
Q9Y5K2	INGEDCSPHSQPWQAALVMENELFCSGVLVHPQWVLSAAHC
Q91Z38	RMKQDKKETAITDCSKAIQLNPTYIRAILRRAELYEKTDKL
Q56P03	EDRAVQVTKKKKKKQHKIPTNDELLYDPEKDNRDQAWVDAQ
P41731	MQNYLKDNKTATILDKLQKENNCCGASNYTDWENIPGMAKD
Q8NCL8	KLHMALYVLQALTATSQGLLNCGVYGWTQHKFHQLKQEARR
Q9H2A3	SAALSSSGDSPSPASTWSCTNSPAPSSSVSSNSTSPYSCTL
P13745	EFEEKFIQSPEDLEKLKKDGNLMFDQVPMVEIDGMKLAQTR

P40630	LVKRRELILLGKPKRPRSAYNIYVSESFQEAKDDSAQGKLK
Q62283	LAVGVWGKLTLGTYISLIAENSTNAPYVLIGTGTTIVVFGL
Q2KI30	SPYTKGSSVLYRKFVHPTLSNKEKEIDEYITQARDKSYETM
Q29463	DKIVGGYTCAENSVPYQVSLNAGYHFCGGSLINDQWVVSAA
Q3T071	RKAHKQEKKEELKNEKALRLNLIGEKLQWFQNHLDPKKVGY
A6QLC6	FVIVWFGAVTITLNSKLLGGNISFFQSLCVLGYCILPLTMA
Q8IYJ0	WAIVWGPTVSREDGGDPNSANPGFLDYGFAAPHGLATPHPN
Q4U5R3	FPKKISELDAFLKEPDLNEANLSNLKAPLDIPVPDPVKEKE
Q9CZB6	HPMQFDHKKELRKLNMSILINFLDLLDILIRSPGSIKREEK
Q9WV68	FNVSSVLYKKFFRDHGGVIVNITATLSMRGQVLQLHAGAAK
Q3SWZ6	PYTVILIERAMKDIHYSVKPNKSTKQQALEVIKQLKEKMKI
Q14681	KLIITKELAEEGVLEEAEFYNIASLVRLVKERIRDNENRTS
A4D1S5	LMAKELIARNSLHLYGESALNGLPLDSSPVLMAQGPSEKTH
Q02067	QQPAAVARRNERERNRVKLVNLGFATLREHVPNGAANKKMS
P59542	ILVVFAFVLGNVANGFIALVNVIDWVNTRKISSAEQILTAL
P13765	LLHCSVTGFYPGDIKIKWFLNGQEERAGVMSTGPIRNGDWT
Q17QB7	DNVEKLFLDLACRLISEARQNTLVNNVSSPLPGEGKSISYL
Q6UXX9	MECVEGCEVGHWSEWGTCSRNNRTCGFKWGLETRTRQIVKK
Q9Y235	HAEEAFFNTILPAFDPALRYNVTWYVSSSPCAACADRIIKT
Q9BQQ7	QCSSCSRNWASAQVLVLFHMNWSEEKSRGQVKMRVFTQRCK
A2AUU0	FFKNRNWLLREFPEILPVNQNTKEKVGESSWDQVGSSISRT
Q9H213	TTPEEASSTAQAQKPSVPRSNFQGTKKSLLMSILALIFIMG
Q9GZN7	TGAEVLKLMDAVMLQLTRARNRLTTPATLTLPEIAASGLTR
O15503	RRGPPRASAAGLAAKVGEMINVSVSGPSLLAAHGAPDADPA
O08807	INTEVVACSVDSQFTHLAWINTPRRQGGLGPIRIPLLSDLN
P18181	YNSTKTIFESEFKGRVYLEENNGALHISNVRKEDKGTYYMR
P11023	YYRGAMGFILMYDITNEESFNAVQDWSTQIKTYSWDNAQVL
P30039	KLLVRLSDVYNRSFLENLKVNTENLLQVENTGKVKGLILTL
Q8TCD6	TQIVYIGDGGNDVCPVTFLKNDDVAMPRKGYTLQKTLSRMS
Q6PKN7	SLPPQNRRPMPQPYGDAFWENLSQRSSSNWMVEQYIPPILR
P13378	PLYSKYKAAAAAAAAAGEAINPTYYDCHFAPEVGGRHAAAA
Q9D1J1	NGRRAFIGLGFGDRGDAFDFNVALQDHFKWVKQQCEFAKQA
A1A4K8	EVFTEMEEKYGEVEEMNVCDNLGDHLVGNVYVKFRREEDAE
P62955	TATPFPMVSLFLVFTAFVISNIGHIRPQRTILAFVSGIFFI
A5PJA8	VVNTSDPDMRREMEQSMNMLNSNHELPDVSEFMTRLFSSKS
Q9WTL7	KAAENIKALIEHEMKNGIPANRIVLGGFSQGGALSLYTALT
P59539	GHFSNWPATSLSIFYLLKIANFSNLIFLRLKRRVKSVILVV
Q8BHC1	QIWDTAGQERFRSITRAYYRNSVGGLLLFDITNRRSFQNVH
P49891	EMMNQKVSPFMRKGIIGDWKNHFPEALRERFDEHYKQQMKD
A6NJV1	KSHTPFSQGGHFPTIFSTNPNLLLMERASTRDRWLHKPSYT
Q0VCT9	GPGTVNGGHPPSALAPAARFNNSQFMGPPVASQGGSLPASM

Q99766	EIISCGNITDKGIIALRHLRNLKYLLLSDLPGVREKENLVQ
Q96LJ8	AIPYELPSSQKPGACAPKSPNQGASDEIPELQQQVPTGASS
Q61039	VKEEKRKKELNEILKSTVSSNDKKTKGRTGWPQHVWALELK
Q9D0V8	KKELLLKHTIGAELAHTADRNLSLTYLSMWLHQPYIESNSK
E9Q0B3	DSANATAVRCCKLPLHAFLDNVGWFVRKLSGLLILLVLFAI
Q28035	AGVEFEEKFIEKPEDLDKLKNDGSLMFQQVPMVEIDGMKLV
Q2YDG2	RLAERTQKKTLTSAGKDLHDNFLKALAVREEDNRSGKSVIF
Q6ZN79	LQLEQGKELWREGREFLQDQNPDRESALKKKHMISMHPITR
P59025	YTFSRAPSPTKSQDQTGSGWNFCSIPWCLFWATVLLLIIYL
P39039	PFSKVKSLCTELQGTVAIPRNAEENKAIQEVATGIAFLGIT
Q9UMX2	CKRCRPSVYSLSYIKRGKTRNYLYPIWSPYAYYLYCYKYRI
Q0X0E2	RRLSQAEEEMRRLRQLRGCTNWRPCYQVEELAAEVERLRTE
Q8IY45	EASLKRCCNLTDEGVVALALNCQLLKIIDLGGCLSITDVSL
Q8N7C4	GNGSCTEITPKYRGASNIINNFIICWSFKIVLFLSFITILI
A6NI73	LQVWSEPSDLLEIPVSGAADNLSPSQNKSDSGTASHLQDYA
Q9NNZ3	SQFHSVRPQGPQLRQQQHKQNKQVLGYCLLLMLAGMGLHYI
Q148F6	LNKLCLTVYQLHTLQPNSTKNFRPAGGAVLHSPGAMFEWGT
Q28145	FVKQSPVPKPGHLAYADSMENFWDWLANITEVQEPLARTKR
P30115	QQVPMVEIDGMKLVQTKAILNYIASKYNLYGKDMKERAIID
Q61199	PPDPKPSYADNTKNFWDWLANITEIQEQLARTKRRPIVKTG
Q2T9X7	DYPFKPPKVTFRTRIYHCNINSQGVICLDILKDNWSPALTI
Q80ZM3	AFSLCWKAPRSPWSFLQAVNNGSPLFLWRTVGSCLDPKMKA
Q6DIA9	ARHDSGCKYRLFVTLLDAHQNVIDKFSAVPDPIEQWNNDIY
P07738	EDIINITLPTGVPILLELDENLRAVGPHQFLGDQEAIQAAI
Q3ZCK9	RRNIHKLLDEVFFSEKIYKLNEDMACSVAGITSDANVLTNE
Q9D8T0	LLGGPGVGLPRIQQFFTSPENSVTAEPRARKYKCGLPQPCP
Q99627	NDMNNARYLWKRIPPAIKSANSELGGIWSVGQRIWQRDFPG
P52823	VSPRKSRVAAQNSAEVVRCLNSALQVGCGAFACLENSTCDT
B1AXV0	GFSSDKKMGGDDVMACVHDDNGRVRIQHFYNVGQWAKEVQR
P10767	TVERGVVSLFGVRSALFVAMNSKGRLYATPSFQEECKFRET
Q9QZB0	DEVLSWSQNFDKMMKTPAGRNLFREFLRTEYSEENLLFWLA
Q9QZM8	LIHNSCGEQGFRGWEVEHGGNGWAVEKNLTLVPGAPSQTCF
Q05B60	AVGRCAASPYLVPLTLHYRQNGAQKSWDFMKTHDSVTILMF
Q0VD35	LYYMLHPRDGGCPAKRLRSENGNDADMVDKNKCCTLCNMSF
P45379	KEAEDGPMEESKPKPRSFMPNLVPPKIPDGERVDFDDIHRK
P55083	GKWTVFQKRFNGSVSFFRGWNDYKLGFGRADGEYWLGLQNM
P33681	HSFMCLIKYGHLRVNQTFNWNTTKQEHFPDNLLPSWAITLI
P29965	QQSIHLGGVFELQPGASVFVNVTDPSQVSHGTGFTSFGLLK
O35566	VPDSCCKTMVAGCGKRDHASNIYKVEGGCITKLETFIQEHL
P70447	GAALGASGDSPSPPSSWSCTNSPASSSNSTSPYSCTLSPAS
Q96E11	GKGQSQTRVNINAALVEDIINLEEVNEEMKSVIEALKDNFN

P01904	AKFASFEAQGALANIAVDKANLDVMKERSNNTPDANVAPEV
Q9QYI7	KKRSVYDRAGCDRWRAGGGANVPHSSPFGAGYPFRNPEDIF
Q8IZ81	DTYVGAQRTHRIENSLTYSKNKVLQKATHVVQSEVDKYVDD
Q9EP72	PMVMMMVLPLLIFVLLPKVVNTSDPDMRREMEQSMNMLNSN
O55125	LYVVHHLWAYKDLQSREETRNAAWRKRGWDENVYYTVPLVR
Q32KU2	FLKVFSRIFGELAVATVICKNKLQGIEVGHFMGGDVGIYTN
Q8QZR4	LHMDIAVNFSQGGLLSPHLHNVCAEATDAIYTRQEDVQFWT
O75391	KYSHLIGKGAAKDAAHMLQANKTYGCVPVANKRDTRSIEEA
Q99N84	YRRNHKGGVPPQRTRKTCIRNNKVAGNPCPICRDHKLHVDF
Q8R527	PASFQNVKEEWVPELKEYAPNVPFLLIGTQIDLRDDPKTLA
Q46669	ASATTESKWNINVRQLISGENAVDILAVQEAGSPPSTAVDT
Q810M6	RAEPRDKEENRQTLPYSYSINHPCFAEIEDTLSSQINESLR
Q9CQM0	VVDVTFPNIAPFELQEIMFKNYYTAFLSIRVRQQSSMHTAA
Q6PER3	VPQRTSPTGPKNMQTSGRLSNVAPPCILRKNPPSARNGGHE
O00287	SETTSQVAKQRKPWMCKKHRNKMYKDKYKKKKSDQALNCGG
Q9D7G4	VCKLGTDKDTLSFCHICFELNLEGVPKSNLLHTKSVRGHKD
Q9CQA5	RKGAISSEEIIKYAHRISASNAVCAPLTWVPGDPRRPYPTD
O94903	LEFVGLMTIGSFGHDLSQGPNPDFQLLLSLREELCKKLNIP
Q8N8F6	QSNFTIDNQEQSGNDSNAYGNLYGSRKQQAGEQPQPASFVP
Q9BVM2	VGDPAPLGAGNLGPELIKESNANPIFMRKDTKMSFQWRIRN
Q3ZCD0	TLTTSVLKNSLCPSSGNVITNLFKEDCHGKIDELFSGKLYL
Q8IUA0	FDFKNYRCTPFKYRGCEGNANNFLNEDACRTACMLIVKDGQ
A0JNC3	VLFSVGVVLALVLNLLQVQRNVTLFPDEVIATIFSSAWWVP
Q8N699	APISQWSSSRRSRSSYTHGLNRTGFYRHSGCERRSNLSLAS
Q7Z7J5	PQKARCKIPALPLPTILPPINKVCRDTLRDWCQQLGLSTNG
Q3SZR5	LGRSSTAFSRGGHFPTIFSLNPTQVLRNRALTRDRWLHTPS
O08989	DLMHLRKVTRDQGKEMATKYNIPYIETSAKDPPLNVDKTFH
Q0III0	SDARTIALKQRTWQALAHEYNSQPSVSLRDFKQLKKCWENI
Q9D8S4	YEFLSFVRQQTPPGLCPLAGNSVHADKKFLDKHMPQFMKHL
O75391	AKDAAHMLQANKTYGCVPVANKRDTRSIEEAMNEIRAKKRL
A4FV84	KRDKKVSLTKTAKKGLELKQNLIEELRKCVDTYKYLFIFSV
Q9CRB5	GMEENTDYPLWTDLASLQATNKERQFFALYKLFYCLRVDTF
A6NDL7	HLCKETTIILWAMKFRLEKENKFVDRFKELFDLEEISSFPS
Q9JIZ9	NMYELRSGTGQQLGQAAEESNCCARLCCGARRPFRIRLADP
Q8TBF4	VIKASIAIDNGRAAEFIRRRNYFDKSKCYECGESGHLSYAC
D3Z4I3	AAERACKDPNPIIDGRKANVNLAYLGAKPRIMQPGFAFGVQ
Q1JPA6	LYWALTMIGFETTILGGYVYNTFNDKYSSAMIHLLLKVTID
P04393	LLFETLSSLNATFITSTWHHNDYRENKYVRDLWSSFRILTK
Q96LD1	EIHSRKDSPLVLQSDRNVTVNARNHMGQLTGQLTIGADAVE
P83714	GAFLALTQHLQLVGDDQSYLNPGSPILLAQLGAARLRAQGL
Q7L0Q8	LCAEEIKAASYIECSALTQKNLKEVFDAAIVAGIQYSDTQQ

Q5E9V3	RFNTTAVPKYYQSPWEQAISNDPELLEALYPKFFKPEGKAE
Q9QXT1	EPCIYESVRVHTAMQTGRTENDLVPTAPSLGTKEGYLTKQG
Q9DCG2	VRKQCTDHGADMVSIHNEEENAFILDTLQKRWKGPDDLLLG
Q3T127	HNKPTFSQTIVLLNLYRNPQNTAQTADGSHCHVSDVEVQEH
Q9Z0H7	REKTQSFLIQKITDEVLKLRNIKLEHLKGLKCSSCEPFAAG
Q32L00	AQPAPTAENNLQQRPGKAWMNKEQHLSDRQFVFKEPQEGVR
Q2KIX7	YHFGFDIELFQSAVKVGLMRNGIQIRDKRAEAGDSHEQASG
Q9NYW5	FFVLCFMFLDSSSVWFVTLLNILYCVKITNFQHSVFLLLKR
P16114	MLRHLKDALMIIYGMSKIDTNACRSMSRKIMTTEVNKTLLD
P35428	AHSGPVIPVYTSNSGTSVGPNAVSPSSGSSLTSDSMWRPWR
Q7SIB3	SLVSPGSCLEDFRATPFIECNGARGTCHYYANKYSFWLTTI
P01903	EPLLKHWEFDAPSPLPETTENVVCALGLTVGLVGIIIGTIF
Q0VBU9	EIKEAFLAVSLALALNGVCTNTIKLIVGRPRPDFFYRCFPD
P05208	NCGGSLVANNWVLTAAHCLSNYQTYRVLLGAHSLSNPGAGS
A8MVS1	LQLEQGKELWREGRVFLQDQNPDRESALKKKHMISMHPIIR
Q3UJP5	SQRACDRLRCVACDFRIVSYNDYMWDKSCDYLFFRNNMPEF
Q08DE6	FLGAGIIFGLYYDAIWAFANNQLIVSGPNGTAGIFATYPSG
Q2KHU0	RSIVEHVASKLNIPSTNVFANRLKFYFNGEYAGFDETQPTA
P63075	QNQREAHFIKRLYQGQLPFPNHAERQKQFEFVGSAPTRRTK
Q9JMF7	FKRELHTISFLGGLALNQGVNWLIKHVIQEPRPCGGPHTAV
P43430	SKDWVKPGQVCTVAGWGKLANCTLSDTLQEVNLEVQKGQKC
O15492	CDTGSTGKFEWGSKHSKENRNFSEDVLGWRESFDLLLSSKN
Q96FW1	AYDEAIMAQQDRIQQEIAVQNPLVSERLELSVLYKEYAEDD
P70660	LRRSRRVKANDRERNRMHNLNAALDALRSVLPSFPDDTKLT
Q9BUN5	GLPEEQKKTMADRNLDQLLSNLEDLSNSIQKLHLAENAEPE
Q9X2V8	YSWNIEKKGMAWIFISNKKENRLYSLNEEHLIRKEISNLSI
Q9D7U6	LLSLLGWVLSCLTNYLPHWKNLNLELNEMENWTMGLWKSCV
O02659	KFVDLTGKGVTYQNWNDGEPNNASPGEHCVTLLSDGTWNDI
Q922M7	PLIVFDGSSTSTSIKVKRTENGADDRLKPLAQIGSTSDAFW
Q96A61	PGQFTCPQCRKSFTRRSFRPNLQLANMVQIIRQMCPTPYRG
P09012	GFKEVRLVPGRHDIAFVEFDNEVQAGAARDALQGFKITQNN
Q5E9B7	ESNTAGLDIFAKFSAYIKNSNPALNDNLEKGLLKALKVLDN
P38447	TFYLSNVAPQVPHLNQNAWNNLEKYSRSLTRTYQNVYVCTG
Q8K2U2	YYVPDFISKEEEEYLLRQVFNAPKPKWTQLSGRKLQNWGGL
P52907	DEEKVRIAAKFITHAPPGEFNEVFNDVRLLLNNDNLLREGA
O35326	AHRPKLNEGVVEFASYGDLKNAIEKLSGKEINGRKIKLIEG
P30048	WGISATAALRPAACGRTSLTNLLCSGSSQAKLFSTSSSCHA
Q9ER00	FKDLAMMIHDQGDLIDSIEANVESSEVHVERATDQLQRAAY
O02691	FGRVDVAVNCAGIAVASKTYNLKKSQAHTLEDFQRVINVNL
O95999	EHLKGLKCSSCEPFPDGATNNLSRSNSDESNFSEKLRASTV
Q9P299	LMLMSVLTCLFESLNHMLRKNVEKRWLLENMDGAFLVLDEI

Q6XJV6	SAVALQHEGNYLCEITTPEGNFHKVYDLQVLVPPEVTYFLG
Q68CL5	TIIEKPPAERHMISSWEQKNNCVMPEDVKNFYLMTNGFHMT
P45379	REKKKKILAERRKVLAIDHLNEDQLREKAKELWQSIYNLEA
A6NLU0	RALVSIIKELEPKLKSVLTMNPRMRKFQVDMTFDVDTANNY
O95237	GDVLEVPRTHLTHYGIYLGDNRVAHMMPDILLALTDDMGRT
Q9CPZ1	TPLQSQETETPSTGPVFFALNRNLEEESSFTRLQDQNRTRE
Q99JZ0	KTGLRLQAVDKGLFVQLVQANTPASLVGLRFGDQILQIDGC
A3KMZ6	ASDQIENMVPVKDRIIKITFNADVHASLQWNFRPQQTEIYV
P11226	VKFQASVATPRNAAENGAIQNLIKEEAFLGITDEKTEGQFV
Q9H7V2	TVPASLDSSRSEPMQQLLDPNTLQQSVESRYRPNIILYSEG
P97469	RSNLRAHLQTHSDVKKYQCKNCSKTFSRMSLLHKHEESGCC
Q5T0J7	GKTHREPQLRPKKMDGASGVNGAPCALHKKTMAPQKTKQGS
Q91WG1	HKFKREWSSVMRCVAVFVGINHASAKVDFDNNFQFSLTLAA
Q59I47	FRHCFVHNLCPLLLLAPSGRNITPAELPAKQREQLLGVCDA
A6H759	DATVKELKGMLNLKTLTLYQNPLCQYNLYRLYIIYHLPGVE
P08034	NPPSRKGSGFGHRLSPEYKQNEINKLLSEQDGSLKDILRRS
P56177	SPPVPPGWNPNSSSGKGSGGNAGSYIPSYTSWYPSAHQEAM
Q5E9D3	SENQKRLKQSKELDAEKAFANEQLTRAILRERISNEEERAK
P97822	LELSDNIISGGLEVLAEKCPNLTYLNLSGNKIKDLSTVEAL
Q9JKT3	DGILTLVASLVLNSLLQFMLNVTFASLLIHSLRRHIQKMQR
Q9H6K1	PDTWAPAPDQTEQDQNRLSQNSVNLSPSSHANNLSVVTYSK
P15018	GSPLPITPVNATCAIRHPCHNNLMNQIRSQLAQLNGSANAL
P19397	KLNEYVAKGLTDSIHRYHSDNSTKAAWDSIQSFLQCCGING
P26842	FLVFTLAGALFLHQRRKYRSNKGESPVEPAEPCHYSCPREE
Q9UIY3	ICVEGFKEHCEEFWHTIRYPNWKHISCKHAESVETEGNGED
P06624	EIFLTLQFVLCIFATYDERRNGRLGSVALAVGFSLTLGHLF
P01138	TTATDIKGKEVMVLGEVNINNSVFKQYFFETKCRDPNPVDS
P14190	GSITADDYRQKWEWKVGTGLNGFGNVLNDLTNGGTKLTITV
Q8R0F8	STKPLSRFWEWGKNIVCVGRNYADHVKEMRSTVLSEPVLFL
Q9BS92	VVGVFHTEYGELNRVHVLWWNESADSRAAGRHKSHEDPRVV
A6H789	RIQSVHKNAFNNLKARARIANNPWHCDCTLQQVLRSMVSNH
P16368	PVHPQQAFCNADIVIRAKAVNKKEVDSGNDIYGNPIKRIQY
Q6EIG7	QWIDKTPYEKNVRFWHLGEPNHSAEQCASIVFWKPTGWGWN
Q96L15	FLVTRFSQDGAQSLVTLWSYNQTCSHFNCAYLGGEKRRGCV
Q9CY52	DIVIAYGQSDEYSFVFRKKSNWFKRRASKFMTLVASQFASS
Q9NSI2	PEATPPPASAAGKDWAFINTNIFARTKIDPSALVQKLELDV
Q8N8I6	GRAEGRQARKSACKCPRKGPNPGPWTRAAAWWGRLEGAKAS
Q9BX74	FPAPNITCKDSSGNETHFTGNEVGFFKPISCRNVNGYSYKV
Q8VE04	VHVYIHHFDRVIVMGAEAHVNTCYKHFYYFVTEMNLIDRKE
A6NLU0	WEVDVGTSQVWDVGVCKESVNRQGKIVLSSEHGFLTVGCRE
Q3MHQ4	HTGEKPFSCSHCSRAFADRSNLRAHLQTHSDVKKYQCKSCS

P19387	NQPTVRITELTDENVKFIIENTDLAVANSIRRVFIAEVPII
Q6IE21	YYMRKHIDDFLPFFTEPEAGNFYTREDFLRYCDDIVHNASW
P31946	MAAAMKAVTEQGHELSNEERNLLSVAYKNVVGARRSSWRVI
Q8TAV4	AEAEATREARAKVLAAEGEMNASKSLKSASMVLAESPIALQ
Q9NVP2	LQRNILASNPRVTRFHINWDNNMDRLEAIETQDPSLGCGLP
P23774	LTSLRRVIEPSVKFAAESYTNKRSFKERIFKVKSCSIVIDL
Q32KN7	GRRFAADVLERHGPICRKLFNKKRKPFNSLKQRLRGTDIPT
Q9D902	VEHGGSSGSKQNSDHNNGSFNLKALSGSSGYKFGVLAKIVN
Q58DP0	LITTQATRAGFTGGVVVDYPNSAKAKKFYLCLFSGPSTSLP
A2A5I3	VWGNTWQQAVYLVCNYAIKGNWIGEAPYKAGKPCSACPPSY
Q91WN1	IESKEIPAYSAFVKESKQKMNARKRRAQEEAKEAELSRKEL
P53808	SRVFMYYFDNPGGQIPSWLINWAAKNGVPNFLKDMVKACQN
Q9EP73	VNATANDVFYCTFWRSQPGQNHTAELIIPELPATHPPQNRT
Q91ZM8	ELKRLLYDTFCEHLSMKDIENIIMTEEESHLGTAEECPVDV
Q9TR36	PAFVRMIAPEGSLVFHEKAWNAYPYCRTIVTNEYMKDDFFI
O60939	ARLPCTFNSCYTVNHKQFSLNWTYQECNNCSEEMFLQFRMK
Q8BGS0	LWERVRLSKNYEKALEQIDENLIYWPRFIRHKCKQRFTKIT
Q86VG3	HLSQEDHVSKRGVFGTDSSENIFTSAKVTHKNEADDYHLRN
Q92813	VCIVQRQKIAYLGGKGPFSYNLQEVRHWLEKNFSKRUKKTR
Q32LB5	HNSCSSKSFKCHPTFQYAGENLWLGPLTISAAKFAINMWYD
Q9QUN5	LTHLDSDDGMMTRAVELNYGNKVVLEGAKALLSRIQPGIEE
Q6UJB9	LTWKLMQKAHHFSLGWLPSQNSVLLEAPALLRRLYLWVEHR
Q9BPW8	RNQLLLEFSFWNEPQPRMGPNIYELRTYKLKPGTMIEWGNN
O75934	AQKELQKLRKHIQDLNWQRKNMQLTAGSKLREMESNWVSLV
O08542	YWNSSNPRLLRGDAVVELGFNDYLDIFCPHYESPGPPEGPE
Q8CC84	LLPITQKPVADGADCGIQWWNAVFLMLLIGIVLPVFYLVYF
Q2TBG9	QPKEEEGDDFIDYPMDDGVWNVPVDSRYVTLTGTITRGKKK
Q17QJ3	SPVRVRGALEEPPAVQHPFLNVFELERLLYTGKTACNHADE
Q9JIN6	FIFGFCWLSPALQDLQATAANCTVLSVQQIGEVFECTFTCG
P19879	IKPAPPSQQDSRIIYDYGTDNLEETFFSQDYEDKYLDGKST
Q9D8U3	NAEDIENLDAAKLSRFIHVNNLHWVTEYSPMIAAGLFNTMV
A2A7V7	NGRVSIRDHPDNLTFTVTLENLTLEDAGTYMCMVDIGFFYD
P35705	LALRGLFIIDPNGVIKHLSVNDLPVGRSVEETLRLVKAFQF
P21926	GYSHKDEVIKEVQEFYKDTYNKLKTKDEPQRETLKAIHYAL
Q8NGA4	FRTTRKWNGCMQCYLQFNLENETAQMWTQEVFGRQMAVIMA
Q6PI78	IEMSIGIILGISTMAAAALGNLVSDLAGLGLAGYVEALASR
P31955	RSVLLLLVLGSGHYAAALELNDPSSGKGESLSGDHSAGGLE
Q9JJZ6	ICEKRFMRSDHLTKHARRHANFHPGMLQRRGGGSRTGSLSD
Q8BJ83	QAIKDPGPTRTFSVVPRAAENQLFSHLTESTEIPPYMTKCP
Q8R035	YIPLDRLSISYCRSSGPGGQNVNKVNSKAEVRFHLASADWI
Q99935	AASTENTTQILANRPHTVLLNATVQVTTSNQTILSSPAFKS

Q5SSH8	NGLSSSEILTLHNWLSFYEKNYVFVGRLVGRFYRKDGLPTS
O94907	VAMVAAALGGHPLLGVSATLNSVLNSNAIKNLPPPLGGAAG
Q2KIP8	SSSRRKKGPGPLTTAYLVIYNVVMTAGWLVIAVGLVRAYLA
Q9H446	EEEQAGKNKLSGKQLFETDHNLDTSDIQFLEDAGNNVEVDE
Q17QU3	IRPSRIHRRKFDPKGNEIEPNFSATRKVNTGFLMSSYKVEA
Q8BL97	RFERGFWLWGGDSETKVYVGNLGTGAGKGELERAFSYYGPL
P41976	RQKHSLPDLPYDYGALEPHINAQIMQLHHSKHHAAYVNNLN
A0PG75	VGPCVTCGCFGDVDFEVKTINEKLTIGKISKYWSGFVNDVF
P30408	CIAANILLYFPNGETKYASENHLSRFVWFFSGIVGGGLLML
Q6UX53	TWKHIGDGCCLTRETWKDLENAQFSEIQMERQPPPLKWLPV
P17918	RNLAMGVNLTSMSKILKCAGNEDIITLRAEDNADTLALVFE
P97348	GVPIIVVGCKIDLRKDKVLVNNLRKKRLEPVTYHRGHDMAR
P32972	KKQTLVTVCESGVQSKNIYQNLSQFLLHYLQVNSTISVRVD
Q91XE0	LPESLKVYGTVYHMIHGNPFNLKALVDKWPDFNTVVVRPQE
P56749	LLFFLAGTVSLSPSIWVIFYNIHLNKKFEPVFSFDYAVYVT
Q92623	PGRLSEEQSKTVEAIEIDCYNSLAACLLQAELVNYERVKEY
Q8BGP5	RHYPTEEEEQALELLSEMEENDPYPAEYEVINQFQPPPAYT
Q8MJ50	LYWACHGGHRDIVEMLFTQPNIELNQQNKLGDTALHAAAWK
Q3KNV8	VPGDIKGETCSAKQHLDSHRNGETKADDSSNKEAAEEKPEE
P61328	PVGLRVVAIQGVKASLYVAMNGEGYLYSSDVFTPECKFKES
P61026	KGEQIAREHGIRFFETSAKANINIEKAFLTLAEDILRKTPV
Q2WG77	QEGARNERAAYLWRPWLSSINDQPRQARSLVDWADNRATAA
P33681	EKKMVLTMMSGDMNIWPEYKNRTIFDITNNLSIVILALRPS
Q5VVH5	VCRRLDYITQSLQQQGVQAENITVTKDFRRVENAYHMEAEV
P51908	GGRHSVWRHTSQNTSNHVEVNFLEKFTTERYFRPNTRCSIT
P04227	TNEAPQATVFPKSPVLLGQPNTLICFVDNIFPPVINITWLR
Q9Y478	VYLSGSFNNWSKLPLTRSHNNFVAILDLPEGEHQYKFFVDG
Q9DCU6	LDIVHQVAIWQRNFRRISYANTKTRAEVSGGGRKPWQQKGS
Q92564	TTEKLRNTLDYLRSFLNDSTNFKLIYRYAFDFAREKDQRSL
B3SHH9	WRTCRVQSPCTPLMNPFRLENVTVSESSRQLLTMHGTFVIL
P61087	PETYPFNPPKVRFITKIWHPNISSVTGAICLDILKDQWAAA
Q14B98	RAVASCSKSQAPDHRAPDPENGHFLPRDRSSIGTVEERGIR
Q9BRU9	LLSMVEEGNPHHYFVATQDQNLSVKVKKKPGVPLMFIIQNT
Q8SPU5	SLTGALSNGLLSEGLLGILENLPLLDILKTRGNAPSGLLGS
P09417	LVYGGRGALGSRCVQAFRARNWWVASVDVVENEEASASIIV
Q7L8S5	ILRRHQRERQELQAQIRSLKNSVPKTDKTKRKQLLQDVARM
Q7YRQ8	CSANVTRYYFNPRHKACEAFNYTGCGGNDNNFVNLKDCKRT
P16114	ADAFNASEITIRKRLESENTNFNQILMQLRMSKAALLLLEN
Q6UWM5	CPNLGGASTAIFVCNYGPAGNFANMPPYVRGESCSLCSKEE
Q8K3W2	FKALPRVVCTLKQLCILYLGNNKLCDLPDELSLLQNLRTLW
Q5E9M6	KDSKSFNCRIEYEKVDKATKNTLCNYDPSKTCYQEQTQSHV

P82923	TARSCGEYAHTRYFKGGLLTNAPLLLGARVRLPDLIIFLHT
Q9TQE0	LLVCSVSGFYPGSIEVRWFRNGQEEKAGVVSTGLIQNGDWT
Q9UJ68	CQLLLLHSLFPVPRMGNSASNIVSPQEALPGRKEQTPVAAK
Q9NW97	KPLKVRRIKSEKLHLKDFRINLPDKNVPPPSIEPLTPPPQY
Q32KU6	FRHEIKNSLKNNYEKALKQYNATGDYRSDAVDKIQSMLHCC
Q3T197	MRPRDRSRVIDGAKHAHKFCNTEDEETIYLRRPEGIERQYR
Q80XC2	ALAAHGFTELSTLEVLPQVYNVRTVSLPLPDLGANNLETNM
P46638	NLSFIETSALDSTNVEEAFKNILTEIYRIVSQKQIADRAAH
Q32LD7	CKSSGKVISSVYSRESQHSRNPRITVLQTNPNPVYESPNLA
Q92623	LVNYERVKEYCLKVLKKEGENFKALYRSGVAFYHLGDYDKA
A6XGL0	QCEKMDIPFLSYLPTEVQLINEAYGLVVDAVLGPGVEPGEV
Q9UBW8	ANQHKEQQLGLKQQIESEVANLKKTIKVTTAAAAAATSQDP
Q6PF18	WKGWDRKAQRNGLRSQVYAVNGDYYVGEWKDNVKHGKGTQV
Q9UKW6	LQFCCDQYKLDTNCISFCNFNISGLQLCSMTQEEFVEAAGL
P62261	DYHRYLAEFATGNDRKEAAENSLVAYKAASDIAMTELPPTH
P16563	AQKWANKCILEHSSKDDRKINIRCGENLYMSTDPTLWSTVI
Q6IR37	VCILRRDHHCRLLGCCVGFHNYRPFLCLLLHSAGVLLHISV
Q96FW1	PLVSERLELSVLYKEYAEDDNIYQQKIKDLHKKYSYIRKTR
P21912	YEVDLNKCGPMVLDALIKIKNEVDSTLTFRRSCREGICGSC
Q15006	EHDYAKAAFCLEELMMTNPHNHLYCQQYAEVKYTQGGLENL
P13765	ADCYFTNGTEKVQFVVRFIFNLEEYVRFDSDVGMFVALTKL
Q96CS7	YGYGPYGGAYPPGTQVVYAANGQAYAVPYQYPYAGLYGQQP
Q9WVF9	IGLHRESSEHPWKWTNNTEYNNMNPILGVGRYAYLSSDRIS
P08861	AQLGDAVQLASLPPAGDILPNETPCYITGWGRLYTNGPLPD
P70122	PYTVILIERAMKDIHYSVKPNKSTKQQALEVIKQLKEKMKI
Q9CR11	ITETGWGEFEIIIKIFFIDPNERPVTLYHLLKLFQSDTNAM
P35330	KINCSTNCAAPDMGGLETPTNKIMLEEHPQGKWKQFLVSNV
Q5E9V3	NTMVKQRKQQASAIMKEIHGNDVDVMHLGKKVSIPRDIMLE
P04071	DECEVWLGKNQLFQEEPSAQNRLVSKSFPHPGFNMTLLTFE
Q9NP99	VGRIILEDYHDHGLLRVRMVNLQVEDSGLYQCVIYQPPKEP
P59024	KGKIPPESTLIFNIDLLEIRNGPRSHESFQEMDLNDDWRLS
Q8NAU1	LVQADSPSAPVNVTVRHLKANSAVVSWDVLEDEVVIGFAIS
Q92535	IYAMSVFMLLGHLIFFDYGANAAIVSSTLSLNMAIFASVCL
Q9JHH9	LMLMSVLACLFDSLSHILRKNVEKRWLLENMDGAFLVLDET
P61213	RISENQGVPVLIVANKQDLRNSLSLSEIEKLLAMGELSSST
Q9D7Z3	AAKHFIHSCLYGSDSKRTTVNKFLSLNNKRSPVKKAAAQFL
Q9CYH2	YGPERRKMMFMGLIRLGVWYNSFRAWNGGFSGNLEGEGFIL
P61328	IYSSTLYRQQESGRAWFLGLNKEGQIMKGNRVKKTKPSSHF
Q2NL00	ELYLDLLSQPCRAIYIFAKKNRIPFELRTVDLRKGQHLSDA
Q32LN3	LEELEWKLAEVGAIQTDLEENPKKAIVDVMVSSIRNTSIYG
Q9BQY4	QYSRPQGAVGGLEPGNAQQPNVHAFTPLQLQELERIFQREQ

Q0VC21	YLIDLGRVDPTQPIDLTQLVNGRGVTIQPSKRDYGVQLVEE
A0PK11	CGLGGRQSQFTIFPHLVKELNAGLHVMILLLLFLALALALV
P50194	WIWCSGTSVKAVDFIHYDEKNNEWNLLQVKNRDNTENSSSS
Q9BGL2	EVAQRAEKLLGITPYSLLWNNCEHFVTYCRYGTPISPQADK
O43247	LGAQDQRSTPTNQKGSIIPNNIRHKFGSNVVDQLVSEEQAQ
Q9NPL8	RWGWRTAVFVTIFNTVNTSLNVYRNKDALSHFVIAGAVTGS
B2RVY9	CWFSGVVEENNSNIWKFWYTNQPPSKNCTHAYLSPYPFMRG
Q5E9E3	IKDQPRPAFSAVGPNSVSRDNVVVFGKVITNQENVYQNNTG
Q03287	GEKIPRKGGPGITHSDLLVINKIDLAPYVGASLEVMEADTA
P58465	NYVKDLSRLGRELSKVIIVDNSPASYIFHPENAVPVQSWFD
Q8WVD3	QENTSSSGHPTFKCPLCQESNFTRQRLLDHCNSNHLFQIVP
Q6NVE3	KEGDEPITQWKGTVLDQVPINPSLYLVKYDGIDCVYGLELH
Q08E08	FWWSYNSSDTYKILIDGTVKNEKSDPKVKLKDDDRITLEGS
P27812	ECPQDWLSHRDKCFRVFQVSNTWEEGQADCGRKGATLLLIQ
Q6UW01	GRVAFAAVRSHHHEPAGETGNGTSGAIYFDQVLVNEGGGFD
Q9HDB8	NTEVLVWEECVANSAVILQNNEFGTIIDWAPRGQFYHNCSG
Q9Y5X0	LQSNALLVQLPELPSKNLFFNMNNRQHVDQRRQGLEDFLRK
Q5TA31	RKALRGKENKGSVEIMRKDLNDARDLHGQAESAAAVWKGHV
O08602	PSATWEFNISDSYFFTFYTENMSWRSANDESGVIMNKWKDD
O75608	IIGLSPDSQEDESGIKQAAENIKALIDQEVKNGIPSNRIIL
Q9NZC4	LFQSTHNVIVKTEQTEPSIMNTWKDENYLYDTNYGSTVDLL
O97563	QILSDGKVFVHLCNYIEPWENLSFLQRESLNHHYHLNCGCQ
Q9BTT0	DLSTVEALQNLKNLKSLDLFNCEITNLEDYRESIFELLQQI
O00560	ANPANPAILSEASAPIPHDGNLYPRLYPELSQYMGLSLNEE
Q95104	GLSGRLGEDAESELMGDAQLNDGALEAEARDSNEEEPKEQV
Q1RMU1	MEYIGSQNASRGRRQRRMHPNVSQGCQGGCATCSDYNGCLS
P09661	NEIRKLDGFPLLRRLKTLLVNNNRICRIGEGLDQALPCLTE
P18121	TALPGASESMGKKAADIKGRNLVILEGLQTIYNRSQANIEE
O70578	KSCEHVTPSGEKNCSYFRHFNPGESSEIFEFTTQKEYSISA
Q920L5	QEYEFEKQFNENEAIQWMQENWKKSFLFSALYAAFIFGGRH
Q9Y5Z4	DRAEMTVFVRSFDGFSSAQKNQEQLLTLASILREDGKVFDE
Q9H7E9	PGTPCASRGARLPGPVSSARNPSTVCLCPEQPTCSNADSRA
Q9QYI7	DIKKAYRKLALRWHPDKNPDNKEEAEKKFKQVSEAYEVLSD
P01246	SLLLIQSWLGPLQFLSRVFTNSLVFGTSDRVYEKLKDLEEG
P04441	VSHIPAVYPGAFRPKCDENGNYLPLQCHGSTGYCWCVFPNG
Q86WH2	LPELQNFLRILDKEEDEQLQNLKRRYTAYRQKLEEALREVW
Q9BZX2	EQKAKALKGQFNFDHPDAFDNELILKTLKEITEGKTVQIPV
Q9CYN2	KNSLDDSAKKVLLEKYKYVENFGLIDGRLTICTISCFFAIV
Q86XT2	VRLSRKFQGLQLEREACLASNYALAKENLALRPRLEMGRAA
Q8BGN8	VAVFAFLYSLAATVVYIFFQNKYRENNRGPLIDFIVTVVFS
Q91ZT8	HVVARMSSVELVHLLMDFGANAQAKNADGKRPVDLVPLESP

P68254	TEQGAELSNEERNLLSVAYKNVVGGRRSAWRVISSIEQKTD
Q2KI14	NARPEDLMNMQHCNLLCLPENYQMKYYFYHGLSWPQLSYIA
Q8CGZ9	ILSQHINSLAIETRRIFLSNNFSSDMFITFTLQFNRHDEFV
Q8R218	VILFLWAIAGIWIVFAIAVVNGSVDLNEGFPFISICGSYAP
Q3SYR2	FTVGDKPVNNFRMIERHYFRNQLLKSFDFHFGFCIPSSKNT
P11245	EGVYCLVGFILTYRKFNYKDNTDLVEFKTLTEEEVEEVLKN
Q9Z2U0	DLTIKLVIKALLEVVQSGGKNIELAVMRRDQPLKILNPEEI
P97370	FYGFLAALFTFTMWAMLQTLNDEVPKYRDQIPSPGLMVFPK
P61019	GLIFMETSAKTASNVEEAFINTAKEIYEKIQEGVFDINNEA
Q96EL1	DACLEHWQGLEAEDWTAALLNRGRSRQPLVLGDNCFADLVH
Q96KN4	VPAGTQPPQQQYYLKVHLGENKVHTARFHSLEDLIREKRRI
Q60774	MAFVLYPPCGSATWNLSDIQNKMFLSMCFCWHYASILILIG
Q15072	QKKYLIKHQNIHTGEKPYECNECGKAFSQRTSLIVHVRIHS
Q91XE0	NTVVVRPQEQEMTDDLDFYINTYQVYSKDPQNCQEFLESSE
Q9BUB7	FSYSTSLIGLTFLPYIFTQNNAISESVPLPIQIIFYGIMGS
Q719H9	SPASPLNNQGIPTPAQLTKSNAPVHIDVGGHMYTSSLATLT
P13598	APQEATATFNSTADREDGHRNFSCLAVLDLMSRGGNIFHKH
Q60654	HELQETLNCHDNCSPTQSDVNLKDELLRNKSIECRPGNDLL
P97760	QELRLRAYVKKRSGKEHAKWNPTAGVAFEYDPDNGLRHTVY
Q8N3Z3	VRIDHAPDLPRPEVCFIGRSNVGKSSLIKALFSLAPEVEVR
P01921	NSQPEILERTRAEVDTACRHNYEGPETSTSLRRLEQPNVAI
Q9CR59	RQAREQRIAECMAKMPQMIENWRKQKRERWEKIQADKERRA
P51124	GRLSRVLRELDLQVLDTRMCNNSRFWNGSLSPSMVCLAADS
Q96IY1	HPVVHPLDLKYDPDPAPHMENLKCRGETVAKEISEAMKSLP
Q96GR4	RCVRRYDHHCPWMENCVGERNHPLFVVYLALQLVVLLWGLY
P57784	IRKLDGFPLLRRLKTLLVNNNRICRIGEGLDQALPCLTELI
P01895	LPEPLTLRWGKEEPPSSTKTNTVIIAVPVVLGAVVILGAVM
O09116	PGNTVVLEPDYTTMPGPCSTNITEPDYTTIPGPCSTNITEP
Q3URK1	NKTEDSGVSSQDGERGSAPANETRSENASQKPRGDADVQNS
Q1LZA1	TAKEIVNVGHSFHVNFEDSDNRSVLKGGPLSESYRLRQFHF
Q9Y3A6	KSDGVHTVETEVGDYMFCFDNTFSTISEKVIFFELILDNMG
B2RVL6	KKRCFGEYKCPKCKRKWMSGNSWANMGQECIKCHINVYPHK
Q5E9E8	QIFQPTQTYTPTTPQPFYGNNFEDEPPLLEELGINFDHIWQ
P97466	RRKLQMWLWSQTFCPVLYAWNDLGSRFWPRYVKVGSCFSKR
Q9H765	ANPNALDGNRDTPLHWAAFKNNAECVRALLESGASVNALDY
Q96HF1	DLKKSVLWLKDSLQCTCEEMNDINAPYLVMGQKQGGELVIT
Q6PIF2	AQELIENINKSRQKDHALMTNFRNSLKTKVSDLTEKLEERI
P19217	IICMIYNNGDVEKCKEDVIFNRVPYLECSTEHVMKGVKQLN
Q60819	VCNSGFKRKAGTSTLIECVINKNTNVAHWTTPSLKCIRDPS
A6QLZ1	VTDLKEKTKAELQPPQGEKENLVGAEEASLEAPEVSSTEAS
Q13595	RRSRSHSPMSNRRRHTGSRANPDPNTCLGVFGLSLYTTERD

P25787	SDPSGAYFAWKATAMGKNYVNGKTFLEKRYNEDLELEDAIH
Q13588	QHFKVLREASGKYFLWEEKFNSLNELVDFYRTTTIAKKRQI
A6NLC5	QPPLPPDSPRYCMISDLFIDNYQVKCINGKMCYVQKQPAPH
Q3T0M9	IVILGLDCAGKTTVLYRLQFNEFVNTVPTKGFNTEKIKVTL
A4FUA8	THAPPGEFNEVFNDVRLLLNNDNLLREGAAHAFAQYNMDQF
Q8N8F6	QFGYVYGMSAIGCLVIHALLNLMSSSGVSYGCVASVLGYCL
Q8R088	ILTTEKQNFLLFDMTTHPVTNTTEKQRLMKKLQDSVLERWV
Q92600	RLTSLGVIGALVKTDEQEVINFLLTTEIIPLCLRIMESGSE
Q2YDL1	RYLTDRKPEFINCQSKIMGGNSILHSAADSVTSAVQKASQA
Q9NWX6	RLQGTLAADKNEILFSEFNINYNNELPMYRKGTVLIWQKVD
P0DMS9	ARDDMDFTELIVTDDKGTLANDFWSGKDLSGNKTRSCKAPK
P12401	WSRFSSMTFSDEDRSISEYYNLFYCLRRDSRKVDMYIKILT
P25311	VHWTRAGEVQEPELRGDVLHNGNGTYQSWVVVAVPPQDTAP
P02970	AYFVLPMKNAEGTKVGSVKVNASYAGVFGKGGVTSADGELF
Q9JHL0	LFSHLEGPESPRYQNFYKGSNQEPDAAYVDPIPTNYYNWGC
Q3SZ19	VSDVQELIRRKEEIEAQIKANYEVLESQKGIGMNEPLVDCE
Q99578	FKELIFQVRHTYEIPLVLVGNKIDLEQFRQVSTEEGLSLAQ
Q6NZQ8	ASQTSTLNEKSPGRSASRSSNISKASSPTTGTAPRSQSRLS
Q96D70	ERLLRFFSVSPQAVYTAMLDNSFERLLLHAVCQYMDLISAS
Q8K1A5	HLINYIIFLRITPFLPNWFINITSPVINVPLKVFFIGTFLG
P39039	EKLANMEAEIRILKSKLQLTNKLHAFSMGKKSGKKLFVTNH
P0DMC8	LIKYIKRHFPSLSIIVLTMNNNPAILSAVLDLDIEGIVLKQ
Q9BUP3	FSKVTLIGRRKLTFDEEAYKNVNQEVVDFEKLDDYASAFQG
P19437	LVTAGIVENEWKRMCTRSKSNVVLLSAGEKNEQTIKMKEEI
Q32KM6	RSGWGRMYYSNGDIYEGQWRNDKPEGEGMLRLKNGNRYEGN
Q9CPZ1	SNGYDILTILPNENINRDPGNPQDEEFLDCHTENDYYVRKI
Q86UF1	VVGVLMFLLTFCGCIGSLRENICLLQTFSLCLTAVFLLQLA
Q9P013	EHTTSSSVSKKPRLDQIPAANLDADDPLTDEEDEDFEEESD
Q9CR35	SDEENVQVLKIAQVFKNPKFNSFTVRNDITLLKLATPAQFS
Q7YS81	RLKKVNEAFEALKRSTLLNPNQRLPKVEILRSAIQYIERLQ
Q0IIG8	VKLDNWLNELETYCTRNDIVNMLVGNKIDKENREVDRNEGL
P15530	RGSQQPQELVSEEGRIVQTQNGSVYTLTIQNIQYEDNGIYF
P53518	NNAKDILYVKPYSEKKIDISNRIIKKIKWAMIDDAGAKTKL
A5D7C3	YVIFSLEIKADAHVRGYVGENIKLRCTFKSSSSITDKLTID
Q8N1N2	ACLLACVIMTAIGVLIICLVNNKGSANSSIVIQLSTNDGEC
Q86TN4	ALADGIPFFRSANGVILTPGNTDGFLLPKYFKEALQLRPTR
Q99KJ5	GAPQRPLSTLSSAPKATLILNSIGSLSKLQAQPLTFSRGGG
Q9NWU5	EVLLEAQDMAVRDHNVEFRSNLYIAESTSGRGQCLKRIRYH
Q8BGZ2	SSAAFRYTAGTPYKVPPTQSNTAPPPYSPSPNPYQTAMYPI
Q61335	LLVIDAVREILKYDDVTEKVNLQNNPGAMEHFHMKLFRAQR
Q9BRQ5	VHLFALMVSTCLLPHIEAVSNIHNLNSVHQSPHQRLHRYVE

Q3T178	PELYEEVKLYKNAREREKYDNMAELFAVVKTMQALEKAYIK
Q15691	VYSTSVTSDNLSRHDMLAWINESLQLNLTKIEQLCSGAAYC
Q60753	QTDSSISFLPHLEAKIRQTHNLARLLTKYAEQLLEEYVQQQ
Q99J99	AFFDIDRCSDHTSPYDHMLPNATHFADYAGSLGVSAATHVV
Q9BS92	FRTYYLKPSNMNAFMENLKKNIHLRTSYSELVGFWSVEFGG
P15947	EDEPSAQHRLVSKAIPHPDFNMSLLNEHTPQPEDDYSNDLM
P59542	VTSFLMLFAIYFLCIITSTWNLRTQQSKLVLLLCQTVAIMY
Q5TBK1	HAEKPSRMNRNQDRNNALPSNNARYWNSYTEFPNRRAHGGF
P50053	KEDSEIRCLSQRWQRGGNASNSCTVLSLLGAPCAFMGSMAP
O95661	ELICKIKGNNLHKFPIVLVGNKSDDTHREVALNDGATCAME
Q9UH92	VTALKIMKVNYEQIVKAHQDNPHEGEDQVSDQVKFNVFQGI
O75431	HLYTILTTQLTNDELSEKVKNYSNLLAFCRRIEQHYFEDRG
O95297	LLTAGVSALEVYTPKEIFVANGTQGKLTCKFKSTSTTGGLT
Q9R0U0	PTGRPRRSRSHSDNDRFKHRNRSFSRSKSNSRSRSKSQPKK
Q6NZB0	MTFYSEVKQIEKRDSVLTSKNQIERLTRPGSSYFNLNPFEV
P48739	WGLQSKVENFIQKQEKRIFTNFHRQLFCWIDKWIDLTMEDI
Q9H560	SLAEKLLSHHANIEALNEEGNTPLLFAINSRRQQIVEFLLK
Q9UM22	CQAPQQWEGRQVMYQQSSGRNSRALLSYDGLNQRVRVLDER
P09936	LFNNVDGHLYELDGRMPFPVNHGASSEDTLLKDAAKVCREF
Q8MJ50	NPLHEAAKRGNLSWLRECLDNRVGVNGLDKAGSTALYWACH
P51157	IYGAQGVLLVYDITNYQSFENLEDWYTVVKKVSEESETQPL
P56371	GTGKSCLLHQFIEKKFKDDSNHTIGVEFGSKIINVGGKYVK
P61213	FVNTVPTKGFNTEKIKVTLGNSKTVTFHFWDVGGQEKLRPL
Q8WU68	MGECTRGGFCNFMHLRPISQNLQRQLYGRGPRRRSPPRFHT
Q865R3	PPVTDWAWYKITDSEDKALMNGSESRFFVSSSQGRSELHIE
Q8K201	FFFHLLIFAFCAAVVYVTYHNKRKIFLLVQSRKWRDGLCSK
P11900	IPKIEMASYDGSVITPSFTSNTAMDIAVKVKNSGDNTELGT
Q06186	CKCLPGYHGHRCHGLTLPVENPLYTYDHTTVLAVVAVVLSS
P13789	REAEDGPVEEFKPKPRPFMPNLVPPKIPDGERVDFDDIHRK
Q2T9Y1	LGVGTPGRIKELIKQGGLHLNPLKFLIFDWNWRDQKLRRMM
O35943	GRAAVGLLPRTASRASAWVGNPRWREPIVTCGRRGLHVTVN
P20764	TILGQPKSDPLVTLFLPSLKNLQANKATLVCLVSEFYPGTL
A2AIP0	RGRAHKDPKPLSPLPRPTFQNLGLLPHYGGYVPGYKFQFGG
P13810	YNDGYVSTTVTLRQAHLIGQNILGSYNEYYIYVVAPAPNLF
Q0IIC4	MMPNRRNMYAVQNNSAGPYFNPRSRQRIPRDLAMCVTPSGQ
Q96E09	TGGSPAEGGGSGGGGGLRRSNSAPLIHGLSDTSPVFQAEAP
Q9NY12	CKCTTDENKVPYFNAPVYLENKEQIGKVDEIFGQLRDFYFS
A1A4M6	ICREGNGVSVSWRPSVEFPGNLYKGEGIVNGTPEQVWDCVK
Q3T0E1	VVANKDMEPEMVCIDSCGRANGMGVIGQDGLLFKVTLGLIR
Q9KIP8	KVHAGVRSAMIDGSHFPFAENVKLVKSVVDFCHSQDCSVEA
A0PK84	LRHDHHCFFTGNCIGSRNMRNFILFCLYTSLACLYSMVAGV

Q9Y6H3	AHVDWFTNIRHLACSEVRAANLSGDCSLVNEIFRLHFGLKQ
Q9H6K1	VEGNFNPFASPQKNRQSDENNLKDPGGSEFDSISKNTWAPA
P27814	LVRVLVQKPSREKCCVFIQENLNKTTDCSVNLECPQDWLLH
Q9CQ52	EGQEQVIPINAGDLFVHPKWNSMCVSCGNDIALVKLSRSAQ
Q8NC54	GASVVPHPSPTPLSQEEADNNEDPSIEEEDLLMLNSSPSTA
O15520	NDCKLKERIEENGYNTYASFNWQHNGRQMYVALNGKGAPRR
Q99JB4	LLIQDQEELRFLLDSIKEKYNSFWIGLSYTLTDMNWKWING
Q32NC0	PASVFRTPTSGQSVSTCSSKNTSKTKKHFSQLKMLLSQNES
Q9HA47	EQKAKALKGQYNFDHPDAFDNDLMHRTLKNIVEGKTVEVPT
Q9DAM2	KKQELRDLFHDFDITGDRLLNYKEFKLYTIFCTDKSIDRKK
Q96DN0	LEDEDIESIDATKLSRFIEINSLHMVTEYNPVTVIGLFNSV
Q9EQI8	TAERILATLSENNMEAKFLGNAPCGHYKFKFPKAIQTESDL
Q99M03	EQAEAQLSELDLLASMFPSENELIVNDQLALAELKDCIEKR
P70339	TRTGDDDDPHRLLQQLVLSGNLIKEAVRRLHSRQLQLHAKL
Q80ZI2	EQHASGRFHCSWCWHTWQSANVVILFHMHLDRAQRVGSVRM
Q8N9E0	AMARWRGPTQSVGPTIQDYLNRPRPTWEEVKKQLENKKTGS
Q8IWD4	QEIEDRIIDEDEEVEADRNVNHLPSLVLSDTMKTGLKREFD
Q9R1A9	ASEITGLSSESLMHCGKAGFNGAVVRTLQGFLSRQEGPICL
P62906	ILGPGLNKAGKFPSLLTHNENMVAKVDEVKSTIKFQMKKVL
Q810P2	NIAGGPGPAMHTRPEPSVYQNRSPLFSMAKRFGCPLDHTHR
Q86W74	QGNTALHLCGHVDTIQFLVSNGLKIDICNHQGATPLVLAKR
Q5T700	VTILGLPSCTPGAQACITLTNRTGFLCHDQRSCIPASGVCD
Q3TDK6	TLTLQGDALSQADVNLKMPRNNQLLHLAFREDKQWKLQQIQ
Q6ZNR8	ARPGLQGLLARPVLALRGEENQRGRGRAWRRASFCRLVGDL
Q8K2J7	RCTTEAEQEVEEEKVEKIELNDSINENSDTVGQIVQYIMKN
Q9H2R5	DECAPHSQPWQVALYERGRFNCGASLISPHWVLSAAHCQSR
Q9H3S4	DVIVTLGGLAGRFDQIMASVNTLFQATHITPFPIIIIQEES
O70401	ILLAVGIWGKVSLENYFSLLNEKATNVPFVLIGTGTVIILL
O75631	IGDVSKASQILNAYLVRVGANGTCLWDPNFQGLCNAPLSAA
P43431	IYEDLKMYQTEFQAINAALQNHNHQQIILDKGMLVAIDELM
P0A1V9	EGSLAISAQEQIAFLRKLYRNELPFRVEHQRLVKDLMIVEA
Q9H115	AEAEKRVKASHSFLRGLFGGNTRIEEACEMYTRAANMFKMA
Q8TCB7	SCREFEDQKLTMLEAGCGVGNCLFPLLEEDPNIFAYACDFS
O43423	ELRNRAPSDVKELALDNSRSNEGKLEALTDEFEELEFLSKI
Q9UNI1	DNVAAGYDIALLRLAQSVTLNSYVQLGVLPQEGAILANNSP
P13661	EGFIISKSGHKYVFVSALTGNLGSNLTSSIKAKKNAITILN
Q6P8I6	KPVIGISTYNVVPFEAGQYFNKIQCFCFEEQRLNPQEEVDM
Q15631	SVNSVTAGDYSRPLHISTFINELDSGFRLLNLKNDSLRKRY
Q2M2T8	EKVFSYLTTIPLEQCTENVLNMTLRDDQKDDNLKEIFTQRN
Q9BSN7	TVQRLVAAAVLVALVSLILNNVAAFTSNWVCQTLEDGRRRS
Q9R0X0	KLMYVMHNSEYPLSCFALFENGPCLIADTNFDVLMVKLKGF

Q8IUE1	KHRFKAYPSEEEKQMLSEKTNLSLLQISNWFINARRRILPD
Q8NE22	DEDVLGTLLKVFQALFLNDFNKQSEILSMLPESVKSKYQDL
Q91WE2	ELIEKQRREEELEELKEYRSNLNKVGISAENKEVEKKLAVK
Q8N9W6	PRYGTVIPNRIFVGGIDFKTNESDLRKFFSQYGSVKEVKIV
Q9CQG0	AIKDSTQRVENQVFHMWRFYNYARMRKVADFFLLQSNYTYV
Q9Y3A3	DEYENETFLCHRFTKFVMKYNLMSKDNLIVPILEEEVQNSV
Q17QQ2	EIPGAKIFKSSSGNISLYCCNLFDLPRANIGKFDRIWDRGA
Q91ZT9	LHYAAERDEACVEVLLEYGANPNALDGNRDTPLHWAAFKNN
P02722	KQIFLGGVDRHKQFWRYFAGNLASGGAAGATSLCFVYPLDF
Q2TBU3	NLMGSVRKDIESYKSGSGVNNRRTELFLKEHDHLRNSDRLI
Q9Y2W7	LQAQTKFTKKELQSLYRGFKNECPTGLVDEDTFKLIYAQFF
Q9R9D6	ITEDLTPTISLEDYCADWATNPPDVRVKRMLIKRVATMVRD
Q9HAV5	SCITCAVINRVQKVNCTATSNAVCGDCLPRFYRKTRIGGLQ
Q8CEZ0	EAEFYNIASLVRLVKERIRDNENRTSQGPVKHVYRVLQCQE
P50194	IWCSGTSVKAVDFIHYDEKNNEWNLLQVKNRDNTENSSSSK
O35417	DLYKRYGGFLRRIRPKLKWDNQKRYGGFLRRQFKVVTRSQE
Q2TBG8	MKQTISNACGTIGLIHAIANNKDKMHFESGSTLKKFLEESA
P20336	GQKESSDQNFDYMFKILIIGNSSVGKTSFLFRYADDSFTPA
Q0IIN9	LPTCSLLTSSPPLEGCCHRLNEEAEVQRGFRPIAVELEFEN
Q5VWC8	NAYLFIYYLIQFCGHSWIFTNMTVRFFSFGKDSMVDTFYAI
Q8N755	LEYPILIAQDVILLLCIFHFNGNVKQATPYIAVLVSSWFIL
Q2YDE4	MTGMTADSRSQVQRARYEAANWKYKYGYEIPVDMLCKRIAD
Q9NZQ0	NMPKRKEPGRSLRIKVISMGNAEVGKSCIIKRYCEKRFVSK
O95843	DLLEIVYKSFDFSNVLRVICNGKQPDMETDSSKSPDKAGLG
P15946	GVLLDRNWVLTAAHCHVSQYNVWLGKTKLFQREPSAQHRMV
Q9JII2	VQQSKRRVQLFGLHSLFFCLNNDAQKVSDFISILRDQIVPN
A4FV84	GKNKVMMVALGRSPSDEYKDNLHQVSKKLRGEVGLLFTNRT
Q96MF7	EFATLDRQLNHYVKAVQSTINHVKEERPEKIPDLKLLVEKK
O75832	AASAGRDEIVKALLGKGAQVNAVNQNGCTPLHYAASKNRHE
Q9D504	NENMVKFLLKKGADVNASDKNHRTAIMIALIVEPTSSVKLL
Q00724	SVDEKGHMSATAKGRVRLLSNWEVCADMVGTFTDTEDPAKF
Q6P0X2	CCQVFAAIEDYQHHYHMMHGNTCSFCNRAFPSGHLLDVHIL
A6QPN6	NAQLTDALRPPHKYVPWVVVNGEHMKDAEHLLLHLVCRLYQG
O95843	YPSGLQTLHEFKTLLGLQGLNQKANKHIDQVYNTFDTNKDG
Q9ET43	LLFFLAGTVSLSPSIWAIFYNSHLNRKFEPVFTFDYAVFVT
Q9BY27	KAGVAGFYVTTNPQELMLQMNLLELIRKLQQRGCRAGNAAL
Q8VEJ3	NTDGQDGAYAEGTTKWPAEENRPQGKPSTKKSQSSKGQEGE
O60725	NHSLEYTVAALSSWLEFTLENIFWPELKQITWLSVTGLLMV
P52556	VLLGTRNDLSPTTVMSEGAQNIVAAMKAHGVDKVVACTSAF
Q9QXP7	CYRCCDPSTPVYQTIPPPQINITILKGEKGDRGDRGLQGKY
P01881	SSSLDTYTCVVEHEASKTKLNASKSLAISGCYHLLPESDGP

Q9NVP2	VLITCTYHGQEFIRVGYYVNNEYLNPELRENPPMKPDFSQL
A2VDH3	QLGAGAFRSAGRLVKLSLANNHLAGVHEAAFESLESLQVLE
P01375	DKPVAHVVANPQAEGQLQWLNRRANALLANGVELRDNQLVV
P68252	KELEAVCQDVLSLLDNYLIKNCSETQIESKVFYLKMKGDYY
Q2KIN3	NTCTIDGFLMLLYVILNENENFPRELSLHLGREFVDCFLYL
Q99598	EAVSFQHFIKTRSLISMDEINKQLIFTTEDNGKENKTPSSD
Q12981	ESEKQLLLQEVENHKKQMLSNQASWRKANLTCKIAIDNLEK
Q32KL9	INQAAKKFMEENERLKRLLKNYGKEEEHILEAENKKLEEDK
Q3LUH2	LSDPQGNGNWQWIDQTPYKENVRFWHQNEPNFSAEECASVV
Q8BGJ9	QTADGSHCHVSDVEVQEHYDNFFEEVFTELQEKYGEIEEMN
O70401	GIWGKVSLENYFSLLNEKATNVPFVLIGTGTVIILLGTFGC
P19141	EFPGYRGRQYVFERGEYRHWNEWDANQPQLQSVRRIRDQKW
Q9BPW5	ALVVRFLTKRFIGDYERNAGNLYTRQVQIEGETLALQVQDT
Q9Y4Z2	ELALSKQRRSRRKKANDRERNRMHNLNSALDALRGVLPTFP
Q62276	KTAKIEDETQVSRATQGEQDNYEMHVRAANIVRAGESLMKL
P61027	YRGAMGIMLVYDITNGKSFENISKWLRNIDEHANEDVERML
Q504M8	FKDGAFLAGTFISTVGIDFRNKVLDVDGMKVKLQIWDTAGQ
Q96CF2	TIEFQREALENSHTNTEVLRNMGFAAKAMKSVHENMDLNKI
P53518	NNIVEKLKWHKNGKYLVLKNNTPYYISFSEVFFDSDKVNNA
Q96PB8	SMCPKGCLCSSSGGLNVTCSNANLKEIPRDLPPETVLLYLD
Q9D676	QTFCMAQEAQLARFDNEKELNFLMRYKANFDSWIGLHRESS
Q6NZB0	TDEEIKKRFRQLSILVHPDKNQDDADRAQKAFEAVDKAYKL
Q8WUJ1	TRGLEANKLQLQEKQTFPPCNAEWSSARGSRLWCSQKSGGV
Q8N1L9	NGLLTQTDPKEQQRQLKKQKNRAAAQRSRQKHTDKADALHQ
Q96S44	LRCRRAGISAPVVFFVDYASNCLYMEEIEGSVTVRDYIQST
O88630	GMLKSIHSKMNTLANRFPAVNSLIQRINLRKRRDSLILGGV
Q9GZP9	SLVFLGQAFTIMLVYVWSRRNPYVRMNFFGLLNFQAPFLPW
Q5VWC8	ITSQEEVQEKYVVCVLFVFWNLLDMVRYTYSMLSVIGISYA
Q5VY80	RWCAVQGQVDEKTFLHYDCGNKTVTPVSPLGKKLNVTMAWK
P79124	VSIETDAETGDSRVVVGECPNNPESISLTVLHRRPGLLNDV
Q62000	KLNKLSFLYLDHNDLESVPPNLPESLRVIHLQFNSISSLTD
Q4KXC9	LINENGEFIKELKYPNHNKINKILPFKNCFAHPTLMFKKDV
P15018	GSANALFILYYTAQGEPFPNNLDKLCGPNVTDFPPFHANGT
O60635	LVVPAIKKDYGSQEDFTQVWNTTMKGLKCCGFTNYTDFEDS
P32850	IRKTQHSTLSRKFVEVMSEYNATQSDYRERCKGRIQRQLEI
Q92637	KLVYNVLYYRNGKAFKFFHWNSNLTILKTNISHNGTYHCSG
Q2HJI8	VEEAFFTLARDIMTKLNRKMNDSNSSGAGGPVKITENRSKK
Q6P926	KENEIKELQQVISQQKQNFRNHISDFRIQKQQETYMAQVLD
P04973	KELDEWYARQDEQLQKTKANNRVADEAFYKQPFADVIGYVT
Q0VD00	LSGEKALIEEVFPETGDVMCNSVNAGWNQDPTHVIRFPLNG
P0CW71	KRLAQFKAASVKDVVSDNVVNMLRGFHEGLATEYQATHDLW

P0CL83	HDKHREVRVKCVKALKGLYGNRDLTARLELFTGRFKDWMVS
Q8BVD7	YYFSYDITLANKHLAIGLVHNGQYRIRTFDANTGNHDVASG
P30042	NLSAANHDAAIFPGGFGAAKNLSTFAVDGKDCKVNKEVERV
Q96NL8	KETLRSTETFKKEDDLDSLINEILEEPNLDKKPSKLKSKSS
Q5JQF8	MWSQRDPSLRKSGVGNVFIKNLGKTIDNKALYNIFSAFGNI
Q3SZC6	RIHLELRNRTPAAVRELVLDNCKSNDGKIEGLTAEFVNLEF
P11900	PSFTSNTAMDIAVKVKNSGDNTELGTLSVPLSFGAAVATIF
Q6QRN8	LLFIVLVFFALFIIFKAYLINCVWNCYKYINNRNMPEIAVY
P23185	SSSHNFSRERLPSGINFCDKNKLSIRTIEKLLVNAFSSPDP
Q9H2K0	TAEDTQNEGKKTKKNKTAFSNVGRKISQRVIHLFDEKGNDL
O00584	LQNCTEPGEQPSPKQEVWLANGAAESRGLRVCEDGPVFYPP
Q9D0L6	NYRGLHDVLSPSKSEASGQGNRYQHDSSRNLITKMQELTSS
P05538	LLVCSVTDFYPAQIKVRWFRNDQEETAGVVSTSLIRNGDWT
O18968	VAEVVYLIFRACARRAQRRSNPPSRKGSGGFGHRLSPEYKQ
Q8R0I4	PSGAAHLEGPAASSWEYSDPNSPVILCSYLPDEFVDCDAPV
Q96BH3	NEYGGNSLRKPCIFPSIYRNNVVSDCMEDESNKLWCPTTEN
Q99944	SLRESQGVCSKQTLVVPLHYNESYSQPVYKPYLTLCAGRRI
Q61955	PKVGQKCIISGWGTVTSPQENFPNTLNCAEVKIYSQNKCER
Q9EQI8	RETEADKKNDRTSLHRKLDRNLVLLVREKLGDQDVWMLPQV
P06730	QANLRLISKFDTVEDFWALYNHIQLSSNLMPGCDYSLFKDG
Q2YDG7	NRFKYIWRLLRPDQQAIILANDSAILEVHRDTHPKAFECET
Q6SP97	CKRNEDVKKAQEEYDNYIQENLKKAAMKRLSDEEREAVLQG
Q02591	CRRKRRHRTIFTDEQLEALENLFQETKYPDVGTREQLARKV
A0JN86	SDPQIAICLDCLRNNGQSGDNVVKGLMKKFIRCSTRVTVGT
Q28145	VVEFEVSPQSTLETKESKSFNCRIEYEKTDRAKKTALCNFD
Q8BH49	RLVVRELEQQLAAMREGSPANALPANPSPVLTQRPKENGWV
Q9JL25	DILSAEEVMQWSQSLEKLLANQTGQNVFGRFLKSEFSEENI
Q8WW43	LLAYVSGLGFGIMSGVFSFVNTLSDSLGPGTVGIHGDSPQF
P16114	NSHDNSAFISSLIYLISKLENNEKIIESIYISSVSFFSDKV
P00766	GASGVSSCMGDSGGPLVCKKNGAWTLVGIVSWGSSTCSTST
Q9H8W3	TESNVNAYEECPSGIPIDMWNKFQELHKKHSEQKSTTSRFR
Q9UKW6	CGEYLYFILQNIRTQGYSFFNDAEESKATIKDYADSNCLKT
Q61039	RPVKRRGTANRKERRRTQSINSAFAELRECIPNVPADTKLS
Q9R9D6	EIIKNLLSLRMPVLGADREWNAIHRLRDVGVDTMYGVAFGE
D3Z3K2	KAENHLKQMEKMYMQQIQSKNIELTSMKGEVISMKKVLEEY
Q9NY72	VVEWFYRPEGGKDFLIYEYRNGHQEVESPFQGRLQWNGSKD
Q7L4S7	CAPDNLNASIESQGQIKMYINEVCRETVSRCCNSFLQQAGL
Q0VCT4	WLLFFLVTAIHADLCRPDAENAFKVRLSIRTALGDKAYAWD
P10749	SLVLSDPYELKALHLNGQNINQQVIFSMSFVQGEPSNDKIP
Q99JB4	KWINGTAFNSDVLKITGVTENGSCAAISGEKVTSEGCSSDN
O89101	VGKPDGTSKECVFIEKVLENNYTALMSAKYSGWYVGFTKKG

Q9D7L8	VLSLPQGRTSSVLTVNGRTENYILDTQHGVQASLECAVQNH
Q9BXY0	FCRNEYSLTGLCNRSSCPLANSQYATIKEEKGQCYLYMKVI
Q8IVN8	PQGQDCGHTYVPAFITTSAFNKERTRQATSPHWSTHTEDAG
Q9WTP6	RRITGRLIHPKSGRSYHEEFNPPKEPMKDDITGEPLIRRSD
Q0VCQ4	VKLAKQNTNKAKDSLRKVRTNAINKVKKSKDKASEDTIRLI
Q9WU03	KAVTGPCRAAFPRWYYDTEKNSCISFIYGGCRGNKNSYLSQ
Q99943	VAQTLLTQDVRVWVFPEGTRNHNGSMLPFKRGAFHLAVQAQ
Q8VIM0	TLTTERNGSETQTLVTLHNNNGTKISTWADEIKDSGETIRT
O43692	KAQLDSADIPKARRKRYISQNDMIAILDYHNQVRGKVFPPA
P17981	TQSVERYIQISLDSPKRKKSNFNCFYSGSTQAANVSSLGSR
Q8IUX1	SPSLEDAKLRRPMVIEIIEKNFDYLRKEMTQNIYQMATFGT
Q9H3Y0	PWRCDGPTCSHYTQMVWASSNRLGCAIHTCSSISVWGNTWH
O94760	YDKLTVPDDIAANCIYLNIPNKGHVLLHRTPEEYPESAKVY
Q8N9N7	IESLPPLLIGKFTLLKSLSLNNNKLTVLPDEICNLKKLETL
Q9BUT1	QKSGNIINMSSVASSVKGVVNRCVYSTTKAAVIGLTKSVAA
Q8TB68	FCSFLRRRLKRRQEERLREQNLRALELEPLELEGSLAGSPP
Q99N94	KSVGRNKLLSQGLAVYASPENRKLFEEEKSLRREGKLEKIQ
A5D7F5	MFQTFYLSIVDLCENGGKRPNTNSSASFTKEQADTIRRIRS
Q9CZJ6	SADALGLEKERPEEKAAAAENPLVFLCARCRRPLGDSLTWV
Q6PI78	QEKLEAPPPTPGQLRYVFIHNAIPFIGFGFLDNAIMIVAGT
P46738	PDDVAGKVEWQRAGNRLKGVNPTPFYINLSTLTVGGKEVKE
P06493	FHGDSEIDQLFRIFRALGTPNNEVWPEVESLQDYKNTFPKW
Q1RMQ5	RSPSSPLPPLPEDEEGSEVTNSKSRDVYKLPPPTAPGPPGD
P01127	NANFLVWPPCVEVQRCSGCCNNRNVQCRPTQVQLRPVQVRK
Q924T2	TAQACGEYAHTRYFKGGLLTNAQLLFGPSVRLPDLIIFLHT
Q0VCC1	MVFGGVVPYIPQYRDIRRTQNAEGFSTYVCLVLLVANILRI
Q8BHX3	GGNKQALEEAAKADRDITEINNLTAEAIQTPLKSVKKRKVI
Q9D1J1	ESVLCVKPEVHVYRIPPRATNRGYRASEWQLDQPSWSGRLR
Q9NYK6	MLQDSGATLCRNSVLWPHSHNQAQKKEETISSPEANVQTQH
Q9D2R4	DIRVGEHDLDAAIVQAKDKVNEVTFKLQHLIEQVEQILKEQ
A1E959	PEIAVMSTGEEIPYLQKEAINFRHDSAGVFMPSTSPKPSTT
Q9BXJ2	GITTSYPEERLPIIFNKVLFNEGEHYNPATGKFICAFPGIY
Q3TLP5	RRLNGAQARELGLVNHAVAQNEEGNAAYHRALALAQEILPQ
A8MXK1	LLSVEYSCHGVPTIEWTYSSNWGTQKIVEWKPGTQANISQS
A2VDH3	SLNVAALDALPALRTVRLDGNPWLCDCDFAHLFSWIQENTS
Q2T9Y6	VEDGVNLSLEHLQPYWEELQNLVQSKKIVAIGTSDLDKTQL
Q92482	FGFAVTLGILIAGQVSGAHLNPAVTFAMCFLAREPWIKLPI
Q8BXL7	GLYKYMFQKDEYCILILGLDNAGKTTFLEQSKTRFNKNYKG
Q46669	IVFLSFYAQADLTDFRVATWNLQGASATTESKWNINVRQLI
Q92882	MSDTNWWKGTSKGRTGLIPSNYVAEQAESIDNPLHEAAKRG
O43731	FALVFTTRYLDLFTNFISIYNTVMKVVFLLCAYVTVYMIYG

Q8WWC4	STEEKPQQHQKTKMIVLGFSNPINWVRTRIKAFLIWAYFDK
P79099	AGAALLHEITPPAIRGDLAVNALNNNSTAGQAVTVELFLTL
A6NKC4	PTPVWFHVLFYLAVGIMFLVNTVLWVTIRKELKRKKKWNLE
Q6P2H8	EAETRQPVVILLGWGGCKDKNLAKYSAIYHKRGCIVIRYTA
P05231	KETCNKSNMCESSKEALAENNLNLPKMAEKDGCFQSGFNEE
Q00731	EIEYIFKPSCVPLMRCAGCCNDEALECVPTSESNITMQIMR
P43487	KFEECRKEIEEREKKAGSGKNDHAEKVAEKLEALSVKEETK
Q2HJ38	EPGLGMEHCDTLNVSLQMGSNKGASQRGMTVYGLPRQVYDP
P12402	VIRTIFKARIYWSGLASLVSNDEDVRHSAFYKLFMCLYRDS
Q9JKK0	SQSDLCSSDQEEEEEMVFGENEDGLEEMMDLSDLPTSLFAC
Q9BRQ6	VDEEERVRVLQGVRLSENVVNRMKEPSSPPPAPTSSTFGLQ
Q8IWZ4	EEHWEKLLKKMQSLWEKACENQRNLNVETTRISHWKAFGDI
Q9NYK6	YEEVKTILSKKINWIVQYAQNKDLDSDSECSKNPQHHLFNF
P24668	EKDKESKNEVALLERLRPLFNKSFESTVGQGSDTYSYIFRV
Q5EBJ4	PVDILHSKCDEEEEEEEEVWNEEINEEDVDECAEEEDEVRV
P05531	SNVKLEQLWKTNKQERKKINNKFCEQYITTFQKFDMDVQKF
Q8TDQ7	MDEYVGLPRNHPESYHSYMWNNFFKHIDIDPNNAHILDGNA
Q9ERI2	LGDSGVGKTSVLYQYTDGKFNSKFITTVGIDFREKRVVYRA
Q00187	DVIIDNGASSFVPLSHYLISNQVPALLQDMGHELVVHTVVT
P20036	GQAFSFEAQGGLANIAILNNNLNTLIQRSNHTQATNDPPEV
Q8VCR7	PVRFSVLLLHGIRFSSETWQNLGTLQRLAEAGYRAVAIDLP
B1AXV0	DDVMACVHDDNGRVRIQHFYNVGQWAKEVQRNPARDEEGVF
D3Z4I3	RAAAERACKDPNPIIDGRKANVNLAYLGAKPRIMQPGFAFG
Q6IPU0	SFQAIHQFNLEGWKSSKDLKNQLGHLESELSFLSTLTGINI
O55234	SRATAGAYIASQTVKKVIEINPYLLGTMAGGAADCSFWERL
Q9Y272	SFEEVQRLRQQILDTKSCLKNKTKENVDVPLVICGNKGDRD
Q15907	YRGAVGALLVYDIAKHLTYENVERWLKELRDHADSNIVIML
Q810I0	LEMGRTALAIKYQELREVAENCADKLQRLEKSMHRWSPQCA
P0CW71	ATHDLWQPAMFNALATDKLFNYWSPAWDNLDGIGGEVKSYL
P58417	SNIKTVKLNLLITGKIVDHGNGTFSVYFRHNSTGQGNVSVS
A6NJR5	FFLALYLANDMEEDDEDPKQNIFYFLYGKTRSRIPLIALFQ
Q0VD59	KVYVQLWRRLKAYNRVIYVQNCPETSKRNIFEKPALPEPNF
Q5BKY1	KLYLSDNHLNSLPPELGQLQNLQILALDFNNFKALPQVVCT
P59796	KVDCNKGVTGTIYEYGALTLNGEEYIQFKQFAGKHVLFVNV
P63011	DWSTQIKTYSWDNAQVLLVGNKCDMEDERVVSSERGRQLAD
Q9HBJ8	FSMRKVPNREATEISHVLLCNVTQRVSFWFVVTDPSKNHTL
P41439	YFPTPAALCEGLWSHSFKVSNYSRGSGRCIQMWFDSAQGNP
P04116	LTVVWLLVFACSAVPVYIYFNTWTTCQSIAAPSKTSASIGT
P52803	NSLEPADDTVHESAEPSRGENAAQTPRIPSRLLAILLFLLA
P00551	TAIPGKTAFQVLEEYPDSGENIVDALAVFLRRLHSIPVCNC
Q60654	DISWIGLSYDNKKKDWVWIDNGPSKLALNTTKYNIRDGLCM

Q9BTZ2	VSKTALLGLTKTLAIELAPRNIRVNCLAPGLIKTSFSRMLW
Q96HS1	VWDPNWDRREPLSLINVRKRNVESGEEELASKLDHYKAKAT
Q7YRD0	ENWLKIFTPIVEHLGLQIRFNLKSRNVEIRTCKETKDVSAL
Q8VCH9	VTMFGWFTMVISYVVYYVRQNQEDARRHLEYLKSLPSRQKK
Q3UTA9	SGCDYALFKEGILPMWEDNRNKQGGRWLLSIDKQLRHFELD
O15400	RISSNIQKITQCSVEIQRTLNQLGTPQDSPELRQQLQQKQQ
Q148F6	LTLQGDALSQADVNLKMPRNNQLLHFAFREDKQWKLQQIQD
Q9UF02	RYTAEDMYRPHPGFYRPRLSNCSDYSGQFLHPDAWVRGRSP
Q96M11	DGEVLVTDESIISESESGTENDQDLWDLRQRLMNVQFQEDK
Q3ZBG9	QIELLEVLIGFETNNKYEIKNSLGQRIYFAAEDTDCCTRNC
Q3SYR2	EIKKPPASERLPINPRDLDPNAGRFVRYQFTPAFLRLRQVG
P20356	EERQAVADGLLIQPLIGSLPNIMAPEWFDGLKRAAEGRRLM
O60258	LQLLILCCQTQGENHPSPNFNQYVRDQGAMTDQLSRRQIRE
Q5TGJ6	AAAAAATAVDEESPFLVAVENGSAPSEPGLVCEPPQPEEEE
Q5JQC4	ILIENRQLSRLMVGPHAAARNLWGNLPPLLLPQRLGAGAAA
Q9NPC8	ETSYCFKEKSRSVLREWYAHNPYPSPREKRELAEATGLTTT
Q9BVV7	YTEKLHRSSAKRLLLPYIVLNKACLKTEPSLRCGLQYQKKT
Q9WTN0	AIWSRPESTQVQNILRQRTENIDIKKYCVQYLEDVGSFAYT
Q8IWT1	VTLKDDDRITLVGSTKEKMNNISIVLRDLEFSDTGKYTCHV
Q3SYR3	TWYVSSSPCAACADRIVKTLNKTKNLRLLILVGRLFMWEEP
P67778	FDCRSRPRNVPVITGSKDLQNVNITLRILFRPVASQLPRIY
P0C2W1	ELRSCALVCKHWYRCLHGDENSEVWRSLCARSLAEEALRTD
A2A5I3	TCSSINVWGNTWQQAVYLVCNYAIKGNWIGEAPYKAGKPCS
Q9NVL8	LQNKEVETPSAGRVDFAFNQNLEEKTSYSLARLQDQNKALE
O95661	SDDTHREVALNDGATCAMEWNCAFMEISAKTDVNVQELFHM
Q5TD97	DLCEEQFMSRDDYPFCVDCYNHLYANKCVACSKPISGLTGA
A6NDL7	LLGAHVTATDLPELLGNLQYNISRNTKMKSKHLPQVKELSW
Q9X2V8	LNEEHLIRKEISNLSIIFHLNIFKSDCLTYSYALKRILNSR
Q0VD27	PAASGNGADDPQWMIQAVVDNVYWQMSLDRKTTALKQLQGH
P04095	KKKINAVRNGVNALMSTMLQNGDEEKKNPAWFLQSDNEDAR
P55918	PVFCDMTTEGGKWTVFQKRFNGSVSFFRGWNDYKLGFGRAD
Q7TQB0	MIMACLAASRFCLHGIAVLNNFLASAMFWTIKNYFSILWDF
O55239	KEPGAFDWSPVVTYVCDLEGNRMKGPEKEEKLRRAIKQVLK
Q9CQE1	YWTVEFGGRTNRVFHIWKYDNFAHRTAVRKALAKDKEWQER
P68543	FSGQGHRLGSATPKIVSKAKNIEVENKNNLSAVPLNNLEPI
Q91ZT9	ADCVELLLEKGAEVNALDGYNRTALHYAAERDEACVEVLLE
Q14390	VRSPVSEILFNDEMDDFSSPNITNEFGVPPSPANFIQPGKQ
Q86UP9	GLSMMLIIACIICFTLFFFCNTATVYKICAWMQLTSAACLV
O95744	KDYGMDLIEVSGNGCGVEEENFEGLSLSALKHHTSKIREFA
A0JLT2	KLSNFLPDLPGMIDLPGSHDNSSLRSLIEKPPILSSSFNPI
P40617	GLDCAGKTTVLYRLQFNEFVNTVPTKGFNTEKIKVTLGNSK

Q9D338	VPEDERRFLSPEFIPPRGRTNPLKFKIERKDMLDRRKVLPI
P32885	TETGNQHKYQTRVVSNANKVNLKFEEAKPVLEDQLAKSIAN
Q3UFY7	HTYNKNSSVCENSSYFQQLQNKTNIILLGDSIGDLTMADGV
Q07817	HLEPWIQENGGWDTFVELYGNNAAAESRKGQERFNRWFLTG
O88696	ASMGSLLLAAGSPGMRHSLPNSRIMIHQPSGGARGQATDIA
Q8BH93	GPWGPMSSGPWAPGIAGQHPNMPYRSPGPYPTVPPPVSGAP
P53516	VEWQRAGNRLKGVNPTPFYINLSTLTVGGKEVKEREYIAPF
A2VDP0	ELLSQEFLLLTLEQKNITVENDMRVNKDSLTDLYVQHAIPL
Q32L08	CDISDTALLHLCNCRKLKKLNLKSSKENRISITSKGIKAVA
Q9ERS4	LLSATCNTFITTLEECVKIANAKFKPEMFQLPHPDPLVSPV
Q64008	ASLEHTKQWLTDALKENDPSNVLLFLVGSKKDLSTPAQYSL
Q3SZ62	NEEIVPQIKEGKRVLIAAHGNSLRGIVKHLEGLSEEAIMEL
Q9UNI1	GTEQYVSVQKIVVHPYWNSDNVAAGYDIALLRLAQSVTLNS
P70661	PGGGSNGDWGSIYSPVSQAGNLSPTASLEEFPGLQVPSSPS
P04233	RSCREDQKPVMDDQRDLISNNEQLPMLGRRPGAPESKCSRG
P50876	LETAISCPDAACPKQGHLQENEIECMVAAEIMQRYKKLQFE
Q3SZZ2	KRQRLTHLSPEEKALRRKLKNRVAAQTARDRKKARMSELEQ
A8MTZ7	LSIFLAWDVDIGSDNTDSRANRLLNGDNLWIDKLPKERTKL
Q3T0S3	DGPSFVKIFGPMSSSAMQFVNVGYFLIAAGAVLFALGFLGC
Q9BQI4	CHSHTVVQDYSYFFFLRMDENYNLLPHGVNFQDAIFPDTQE
Q9GZP9	LVYVWSRRNPYVRMNFFGLLNFQAPFLPWVLMGFSLLLGNS
P20036	FGQAFSFEAQGGLANIAILNNNLNTLIQRSNHTQATNDPPE
Q9CRA4	NPLNLVPFYTGARHHDFHHMNFIGNYASTFTWWDKLFGTDA
P49788	EKKKRQQEDYLLYKQMKQLKNPLEIVSIPDNHGHIDPSLRL
Q62000	VIHLQFNSISSLTDDTFCKANDTRYIRERIEEIRLEGNPIA
Q8HZJ5	AMGFLLMFDLTSQQSFLNVRNWMSQLQANAYCENPDIVLIG
P05837	DESVVLWQINPFPDYPFFALNQSGSNTNTSDKLTIWNDLSP
P25789	TMDVSKLSAEKVEIATLTRENGKTVIRVLKQKEVEQLIKKH
Q9D710	LSAIVMMKNRRSITVEQHVGNIFMFSKVANAILFFRLDIRM
A2AFE9	LRSGIMMEVTPGNARMANRGNMAHVSFPLGSPRHPMDNWQQ
Q969W9	IFDSDLMDSARLGGPCPPSSNSGISATCYGSGGRMEGPPPT
O60828	GLPPSWYKVFDPSCGLPYYWNADTDLVSWLSPHDPNSVVTK
Q3SZ45	THVNTGRNLHSHHFTSPLSGNQEVSAFGEEGEGDYLDDWTV
Q60930	YQKVCEDFDTSVNLAWTSGTNCTRFGIAAKYQLDPTASISA
Q1LZA1	GYDGENGPEHWGKLYPIANGNNQSPIDIKTSETKRDPSLKP
Q8K2Q2	KQDPAVCALHTDKSVFLGFLNLYFQNNPCAFDFGLCNPIIR
Q9CWJ3	LACAGHLPENLRHDSRTFVINTSDSGSSQTESPSSKYSGFF
Q8BWY2	DGTPYSFELFEIIIDPTNLRNRDCMTIFNGKAYSKDCKELR
Q96CJ1	PVTVFKGSKKPYLKECILIINHDTGECRLEKLSSNITVKKT
Q8C1T8	CPSGWIGFGSKCFYFSEDMGNWTFSQSSCVASNSHLALFHS
Q6PK04	PAPWPGLRSKEKKKVNCKPKNQDEQEIPFRLREIMRSRQEM

Q62189	PQQLMPGQMPPAQPLSENPPNHILFLTNLPEETNELMLSML
A6NEH6	GQAGDGPKPAELPPTPGTERNPEMELEKVRMEFELTRLKYL
P13634	LYPIANGNNQSPIDIKTSEANHDSSLKPLSISYNPATAKEI
Q9ESL9	TSECIFREQFEENWYNTYSSNIYKHGDTGRRYFVALNKDGT
Q9D3F7	GVSLPDFELLSDPEDEHLCANLMQLLQESLSQARLGSRRPA
Q8C1T8	GLHRASTQHPWIWTDNTEYSNLVLTRGGGECGFLSDNGISS
P28651	EGVEWGYEEGVEWGLVFPDANGEYQSPINLNSREARYDPSL
P16563	YQQGTPCASCPNNCENGLCTNSCDFEDLLSNCESLKTSAGC
P46926	DEYVGLPRDHPESYHSFMWNNFFKHIDIHPENTHILDGNAV
P35695	EAVNGVFVLCKSSSKLACATNNLARASKEYLPASTFKIPNA
Q497H0	TPSTSNSQSDLFSEETTSDNNNTSVTTPTLSPSQQSLPTEL
Q9D8B3	QEELDKNLLEISGPETVPLPNVPSVALPSKPAKKKEEEDDD
O35075	EGTVNLQLSAKSVGVFEAFYNSVKPIQIINSTIDVLKPGKI
A2VDN0	VIELNTTIVLPPRNFWELLMNVKRGTYLPQTYIIQEEMVVT
Q56JV9	VKTTDGYLLRLFCVGFTKKRNNQIRKTSYAQHQQVRQIRKK
Q3TQI7	KIKNIISTEDAKARLLAEQQNKKKDSETSFVPTNMAVNYVQ
Q9NW81	MWNGRIRGIHRLGAAVAPEGNQKKKRTILQFLTNYFYDVEA
P0C2W1	GYVALLGSDDQSWGWNLVDNNLLHNGEVNGSFPQCNNAPKY
Q6ZS02	SHSFMHSMDPQLERQMETTQNLVDSYMAIVNKTVWDLMVGA
Q58CY8	LGIGIWVMVDPTGFREIVAANPLLITGAYILLAMGGLLFLL
O43291	CLKKCATVTENATGDLATSRNAADSSVPSAPRRQDSEDHSS
Q2HJ97	GEALSKNPGYIKLRKIRAAQNISKTIATSQNRIYLTADNLV
Q9Z0G9	ALLTLVPVSWSANTIIRDFYNPLVPEAQKREMGAGLYVGWA
Q9ULW3	SLHNTPMGARRRSPFRYDLWNLKYLHRFTWSHLSEHLAFER
O35740	PQHAFNALMGEHIHYGAGNMNATSGIRHAMGPGTVNGGHPP
Q06186	LFKVAFSSKPQGLATPSKERNGKKKKKGKGLGKKRDPCLRK
P35762	DVKQFYDQALQQAVMDDDANNAKAVVKTFHETLNCCGSNAL
P29972	IGLSVALGHLLAIDYTGCGINPARSFGSAVITHNFSNHWIF
A2RRY8	WEKEKANNLKNEIKEVEELDNWQVPMPFLHGFFSTGASNFS
Q9GZU7	KICVVIDLDETLVHSSFKPVNNADFIIPVEIDGVVHQVYVL
Q17R00	QKLEEEGSDSQQKKKGTHHINRWRIATKQASPEEIDVTSDD
Q99619	GVATALAPLQTDHYAALLGSNSESWGWDIGRGKLYHQSKGP
Q9R061	PVFVDQEQSISLMSVGFLLENPDEAVVWRGPKKHALIKQFV
Q9WUJ8	GLNKMMYQSCLKSFECLLGLNSNVGIRDLAVQFSCTEAVNL
Q1RMR4	LLLIGDSGVGKTCLLCRFTDNEFHSSHISTIGVDFKMKTIE
Q3SXB8	NDLEKEGAFVYSDRSPMQTFNKWRSGEPNNAYDEEDCVEMV
Q9DCX8	NKRRSVRFISSEHVPMEVIENVIKAAGTAPSGAHTEPWTFV
O43760	LAFLASAFFLVVDAYFPQISNATDRKYLVIGDLLFSALWTF
Q60898	YSLSVRHRQVKHYRIFRLPNNWYYISPRLTFQCLEDLVTHY
Q99PM9	NLIVQHIQDILNGGLSKRQTNGYLNGYTPSRKRQASESSSR
O75841	NNGVTKTWDRLMLQDNCCGVNGPSDWQKYTSAFRTENNDAD

P33781	KQLTLSTAVEAAKGEVAITLNGQALKVGSASPTVVTVASNK
Q32L50	PPAMNSVSLRCSGDGLDSDGNQTLHWQAIGNPRCQGTWKKV
P62823	QRLGEQLGFEFFETSAKDNINVKQTFERLVDIICDKMSESL
Q99LP6	LEVADILEKATQSVPKEEISNNNPHLKSLYEGLVMTEVQIQ
Q3T0S3	TAHNMSVQGCFKQLLYDIRTNAVTVGGVAAGIGGLELAAMI
Q99581	ALYKLPAGLAPLPFPGLSKLNLMAASAGVAPAGFSYWPGPG
P54709	GPVYVACQFPISLLQACSGMNDPDFGYSQGNPCILVKMNRI
Q60595	LSNMFMEQQKFIHETLTLQKNRMEEFKSLCEKYLEKLEVLR
O75636	NSNCAVIVHGAWWYASCYRSNLNGRYAVSEAAAHKYGIDWA
P04390	PSEPNKKIAIDIKTTYTNKENEKIKFTLGGYTSFIRNNTKN
Q2TA29	RWLKELRDHADSNIVIMLVGNKSDLRHLRAVPTDEARAFAE
Q2KHU4	IDDSVASLVIAQLLFLQSESNKKPIHMYINSPGGVVTSGLA
P28065	LHGIELEEPPLVLAAANVVRNISYKYREDLSAHLMVAGWDQ
Q7Z692	MLQGSQAALYIQKIPEQPQKNQDLLLSVQGVPDTFQDFNWY
O43736	LLLGNCYLMPLNTSIVMPPKNLVELFGKLASGRYLPQTYVV
P60174	LGSSAMAPSRKFFVGGNWKMNGRKQSLGELIGTLNAAKVPA
P11912	MVSLGEDAHFQCPHNSSNNANVTWWRVLHGNYTWPPEFLGP
P43166	SPSLQPLELSYEACMSLSITNNGHSVQVDFNDSDDRTVVTG
Q5VST6	QYSSREKDAIECFMTRTSKGNRIACMFVRCSPNAKYTLLFS
Q3ZCD8	PQNEEIRAVGRLKRERSMSENAVRQNGQLVKTDSMYGISNI
Q5SRD1	MNGLRLGLKETQNMAWSKPRNVQILNMVTRQGALWANTLGS
Q08DT3	LPEDSLNSLIIKLIQADILKNKLSKQMVDVKENYQSTLPKA
Q8NAU1	PASEPVLFKTPREAEKMASKNKDEVTMKEMGRNQQLRTGEV
Q9CZQ6	TKRHHFMIQKLLILLSYGYTNGLDDAHSLRCNLTIKDPTSA
Q9TT92	KPWSKCTSATITEFLDDGHGNCLLDLPRKQIPGPEELPGQT
Q9CQX3	LPYYGVTPVVSTYPSDHLDRNFRDAFRHGLLSGGILSFLEH
Q8N5S3	HVGRGRIYYAKFINTNARTYNEPFPYIDPKKGPEIQGDWWS
P52840	VPVKGIPLIKYFAETMEQLQNFTAWPDDVLISTYPKSGTNW
P35330	FEVYIWSEKQIVEATESWKINCSTNCAAPDMGGLETPTNKI
Q61599	KDTFVLKEGIEYRVKINFKVNKDIVSGLKYVQHTYRTGMRV
Q16878	PRTLADLIRILHQLFAGDEVNVEEVQAIMEAYESDPTEWAM
Q9CQS9	SGIVTKEMLDVSKKMAPCFVNFSRLQQISDIQAEIYQNNLE
Q9Z1Q5	EVDETSAEDEGISQRKFLDGNELTLADCNLLPKLHIVQVVC
P15428	QLRDTFRKVVDHFGRLDILVNNAGVNNEKNWEKTLQINLVS
Q9D8T0	PLHEGTLVFVASYDDPATKMNEETRKLFSELGSRNAKDLAF
Q3ZCU0	MAEELNLDKETVRLILKENLNMRKISAKVISGVLKETEPHY
P63032	LLVSGDENCAYFEVSAKKNTNVNEMFYVLFSMAKLPHEMSP
Q15040	PPQIYHEKQRRELCALHALNNVFQDSNAFTRDTLQEIFQRL
P22387	KCIYQQLVEGRKLRRLEAISNSIKASFRVAKLKAELYRDQK
Q9JM90	MLEKNPSWGNMILRPGSDSKNYSITIRQEIEMPRIKHFKVT
Q9JM90	FYAVSRKEATAMLEKNPSWGNMILRPGSDSKNYSITIRQEI

O00322	YIFECASCITSYTHRDYMVSNPSLITKQMLTFYSADTDQGQ
Q9Y535	WQFERKLNDSIAEELNKKLANKVVYNVGLCICLFDITKLED
Q9Z2Y8	TKPADMVIEAYGHGQRTFGENYVQELLEKASNPKILSSCPE
Q99463	IIIIFKKQRKAQNFTSILIANLSLSDTLVCVMCIHFTIIYT
Q17QJ5	CGAFGADDWNLNIYFNCTDSNASRERCGVPFSCCTKDPAED
Q6UXN2	WTAVQKSHYTIWDKPNAGFFNITMIQLTQNDSGFYWCGIYN
Q61937	SIEKGGSLPKVEAKFINYVKNCFRMTDQEAIQDLWQWRKSL
Q86WA6	ALIAAAKYPSYIHKMVIWGANAYVTDEDSMIYEGIRDVSKW
P46638	SDLRHLRAVPTDEARAFAEKNNLSFIETSALDSTNVEEAFK
P02763	HFAHLLILRDTKTYMLAFDVNDEKNWGLSVYADKPETTKEQ
Q8BL95	HKQLMLFYHRRQEELKKLEENDDDSCLNSPWADNTALKRHF
Q923S9	DNVEKLFLDLACRLISEARQNTLVNNVSSPLPGEGKSISYL
A6H6X4	GHYFIDRDGLLFRHVLNFLRNGELLLPEGFRENQLLAQEAE
Q9BXJ4	SLATHFSNQNSGIIFSSVETNIGNFFDVMTGRFGAPVSGVY
Q53H96	SLSTLEELLPPNTRVLRVLPNLPCVVQEGAIVMARGRHVGS
Q47456	LLLQRTGEVLPRSLISSLVWNMNFDSDTNVIDVAVRRLRSK
P40673	KGKSEAGKKGPGRPTGSKKKNEPEDEEEEEEEEDEDEEEED
Q3ZCC4	QRIERNKQLALERRQAKLLSNSQSLGNDLSVNTPSTQTSEA
D3Z690	PGYFPNELRAIFREQVRLIQNAIIESRIDCQRHCGIYQYET
Q3T0A6	NGFPSDASANSSLLLEFQDENSNQSSVSDVYQLKVDSSTNS
Q8K1A0	LLEQYPTRPHIAACMLYTIHNTYDDIENKAVADLGCGCGVL
Q6PHW0	HYYNEISVSIACGILLAALQNAGLVTVTTTPLNCGPRLRVL
Q14002	PYECEIQNPVGASRSDPVTLNVRYESVQASSPDLSAGTAVS
P80219	DIMLLKLTRKADITDKVSPINLPRSLAEVKPGMMCSVAGWG
Q28110	QDRKSKIFSYQRTNFSIPRANLSHSGQYHCTAFIGKMLHSS
P14436	LLGQPNTLICFVDNIFPPVINITWLRNSKSVTDGVYETSFL
P09326	TSIQGHLVHMTVVSGSNVTLNISESLPENYKQLTWFYTFDQ
P62827	PNWHRDLVRVCENIPIVLCGNKVDIKDRKVKAKSIVFHRKK
Q3KNJ2	QQVWHEQVDTSVVSQRAKELNKRLTAPPAALLCHLDEALRP
P14190	AYFVLPMKNAEGTKVGSVKVNASYAGVLGRGGVTSADGELL
Q2KI06	DEMKRAMISMPFTPGMVELLNFIRKNKNKFDCIIISDSNSV
Q3T021	LRAISHRLLKEVNAPRQPLYNIQVRKGSLFEIISFPAKTAL
Q9JI58	ILHLSNINKTMTSGDPGETANATEVGECLTQPLKDLCQKLR
Q6SJQ5	YWKSCEILVETDASEQLVKENRVSIRDDQTDFIFTVTMEDL
P02522	SLRPIKVDSQEHKITLYENPNFTGKKMEVIDDDVPSFHAHG
Q99990	LLQQDRCLARPQESAARENGNPGQIAGSTGLLFNLPPGSVH
P23150	TWLRNSKSVTDGVYETSFLVNRDHSFHKLSYLTFIPSDDDI
Q99614	LGKLKDLGNLVLRPFGLSTENFQIKQDSSTGSYSINFVQNP
Q60692	FNGGVVLGADSRTTTGSYIANRVTDKLTPIHDHIFCCRSGS
P13972	AIGDKLWLVQFIPLIYGEVVNVLWLFFCKNSNVIVDYCRGL
Q9D8U2	FLRLFPMTPNWFLNLSAPILNIPIVQFFFSVLIGLIPYNFI

Q8N2K1	KLIFPREFPFKPPSIYMITPNGRFKCNTRLCLSITDFHPDT
Q9GZX9	ECMLCLGALWDECCDCVGMCNPRNYSDTPPTSKSTVEELHE
Q61599	TKYKKTLLGDVPVVADPTVPNVTVTRLSLVCDSAPGPITMD
Q9JJJ3	GLEPIVIGLLIIVISCSLGLNSGCAMNPARDLSPRLFTALA
P04230	LVRDSRPWFLEYCKSECHFYNGTQRVRLLERYFYNLEENLR
Q8BR70	LMFAWSVVASTAFLADSQPPNRKALAVYPVFLFYFVISWMI
Q5HZI9	HGIAEYYRGMVPILFRNGFGNVLFFGLRGPIKESLPTATTY
Q9BUB7	VFTTFYAKTKSLLVNPVLFPNREDYIHLMGYDKEEFILYME
Q1RMU1	DAIPDNKGLEPSRETPEQRENKQQQKKRKVQDKQQKSVSVS
Q2KI69	ESISAQLEEASSTGGFLYTQNSTKRSIKERLMKLLPCSAAK
Q46668	GELRNWQIMPGTRPNTIQFRNVDVGTCMTSFPGFKGGVQLS
Q9NPI5	MKSREELFREVLEDIQNSLLNRSQESAPSPARPARTQGPGR
O95050	KEEKLRAAVKRVLKCDVHLGNPLAPAVLPLADCVLTLLAME
Q969E2	AQTEWNTGTWRNPPSREAQYNNFSGNSLPEYPTVPSYPGSG
Q9CQD7	NAHCCQSDGCNSGSVPPPLNNRTENGLMCPSCIAPFQETCP
A1XBS5	LTARNREAKQLTQLERTRQRNPSDRHVISQAETELQRAAMD
Q99JY3	GKTGAGKSSTGNSILGEKVFNSGICAKSITKVCEKRVSTWD
Q9CR48	FGFFLTYIRDFQKITLRVEANLHGLTLYDTVPCPVNNERTP
P33781	AMATVSGSVLAAVTNGQLTFNWQGVVPSAPVTQSSWAFVNG
Q17QJ2	GLGLFCTVADRLLQFPIIQQNAWLRALSDNSVHCVIGMWSW
P08883	DSGGPLVCNNKAYGVLTYGLNRTIGPGVFTKVVHYLPWISR
P70122	HTQLEQMFRDIATIVADKCVNPETKRPYTVILIERAMKDIH
Q6PHN7	YVVPVYLLWKGGAYTPEPLCNFQWALLSTGLMFFYHFSFLQ
Q96DR5	QKAQEAEKLLNNVISKLLPTNTDIFGLKISNSLILDVKAEP
Q9H6K1	EAWPPGVCLKYVGGDQFGHVNMVMVRSLEPQEIADVSVQMC
Q9UEU0	ANETLAEMEEELRYAPLSFRNPMMSKLRNYRKDLAKLHREV
P08246	FCGATLIAPNFVMSAAHCVANVNVRAVRVVLGAHNLSRREP
O08604	DLCQKLRDKVSNTKVDTHKTNGYPHLQVTMIYPQSQGQTPS
Q9Y691	SVWTEESQCTLLNASITETFNCSFSCGPDCWKLSQYPCLQV
P53811	PAFVRMIAPEGSLVFHEKAWNAYPYCRTIVTNEYMKDDFFI
Q9ZIS7	PKTKRNERFLKSFVKGDYYQNLIVETDRVRSAGLTFPNDFY
Q02878	EKKKKEKVLATVTKPVGGDKNGGTRVVKLRKMPRYYPTEDV
P97930	GHRAELLRFPERSTEIGKLLNSYLEKKTELEDHSVHLLFSA
Q7RTU5	KPGPFGGGLALGPAPRGTMNNNFCRALVDRRPLGPPSCMQL
P78417	YAGLKEEFRKEFTKLEEVLTNKKTTFFGGNSISMIDYLIWP
O18968	LLYPGYAMVRLVKCDAYPCPNTVDCFVSRPTEKTIFTVFML
Q9WVS0	IRLLTGEINGSADHRMFSFHNGGVQISCKYPETVQQLKMRL
Q8IUQ4	QHKSITTLQGEDIVFLATDINLPGAVDWVMMQSCFGFHFML
P97440	RYIKEVPRHLRQPGIHPRTPNKFKKYSRRSWDQQIKLWKVA
P57735	SDLSQAREVPTEEARMFAENNGLLFLETSALDSTNVELAFE
Q9BZD7	FIIWRCQLQKATRHHPSYAQNRYLASRAGHTLPRVMVYRGT

Q9H3N1	VLMSSMSALFQLSMWIRTCHNYFIEDLGLPVWGSYTVFALA
P24071	LPQHQSGEHPANFSLGPVDLNVSGIYRCYGWYNRSPYLWSF
Q9D7S0	IAAFAITVVDSLNCTQCYTYNSTCDGQATECNEQSFSCVES
P51959	DLLSLTQFFGFDTETFSLAVNLLDRFLSKMKVQPKHLGCVG
Q28153	NNVAAGYDIAVLRLAQSATLNSYVQLGVLPQSGTILANNTP
A6QNL6	QYRPRYITGYAVWLVLWVTWNVFVICFYLEAGDLSKETDLI
Q6P9G0	CTPRGRFVHVPPQLPCSDWANDFGKPWWQGSYYEVGRLSAK
Q9HCN8	LTHVLTGKNLHTHHFPSPLSNNQEVSAFGEDGEGDDLDLWT
Q6P9F5	VTQLRSLVIDLERTAKELDTNTLKNAGDLLNRSAPQKLEVI
Q8BFY6	PPGGPYGTQPGHYGQGGVPPNVDPEAYSWFQSVDADHSGYI
P26373	GMVLKPHFHKDWQRRVATWFNQPARKIRRRKARQAKARRIA
Q9WTL2	EDYNFVFKVVLIGESGVGKTNLLSRFTRNEFSHDSRTTIGV
Q9CQY8	NGTCVSPTDFKNPTINNMVSNWKIPNSNSEEDRHRIFHFSV
P07743	ELLQQATSWPLAKNSILETLNTADLGNLKSFTSLNGLLLKI
P58466	ICVVIDLDETLVHSSFKPVNNADFIIPVEIDGVVHQVYVLK
Q61754	TPTKWQKPNDLQCVFIKLLPNENCTKPYLHKVTDVMLCAGE
Q1JQA4	LLGVVMFIVSFIGVLASLRDNLCLLQAFMYILGICLIIELI
Q9BUE0	GSVLDHSLESLIHRLRGLCDNMEPETFLDHEMVFLLKGQQA
P08831	DEPVLLKEMPETPKIIKDETNLLFFWEKHGSMDYFKSVAHP
Q9JL99	KNGINLSGNTEENMNCAYLHNGKIHPASCKERHYLICERNA
Q8NDD1	ILEQERAIMLGAKPPKKSYVNYKVLQEQIKEKKAAKEEEKR
Q3ZCA2	TQFGNKYIQQTKPLTLERTINLYPLTNYTFGTKEPLYEKDS
B1ATL7	ENVLGGHAPSPLVVSVDKNGNQELHHDMPLQCLSSKPEDDA
Q8WXK3	HHAAKVKNVDLIEMLIEFGGNIYARDNRGKKPSDYTWSSSA
P18405	PGDTGYKIPRGGLFEYVTAANYFGEIMEWCGYALASWSVQG
P61289	QLVDIIEKVKPEIRLLIEKCNTVKMWVQLLIPRIEDGNNFG
Q0VD00	EKALIEEVFPETGDVMCNSVNAGWNQDPTHVIRFPLNGYCR
Q8N6N2	DYARALRYLQEARSREPTDTNVLRYIQLTQLKMNRCSLQRE
Q8JZU6	SFQSPVKISEIMRSNGFCLANTETIVIDHSIPNGKDQLLDA
Q8HZJ5	ITTVGIDFREKRVAYNTQGPNGPTGKAFKVHLQLWDTAGQE
P13762	LAGDTQPRFLEQAKCECHFLNGTERVWNLIRYIYNQEEYAR
P97458	RHRTTFNPAQLEQLESAFGRNQYPDIWAREGLAQDTGLSEA
B2RXH8	NVTNKMDPHSVNSRVFIGNLNTLVVKKSDVEAIFSKYGKIA
A4IFK7	PPREGPGGRREKEALFPRGSNANSNKEEKTIIRKLFSFRSG
P63075	DTFGSRVRIKGAESEKYICMNKRGKLIGKPSGKSKDCVFTE
Q8N8J0	LLTLGLSLLHADVVPNATIRNVLREKIYSTAFDYFSCPPKF
Q9GZU7	NYVKDLSRLGRDLRRVLILDNSPASYVFHPDNAVPVASWFD
Q28153	HCVDSQMTFRVVLGDHNLSQNDGTEQYISVQKIVVHPSWNS
Q8BVH9	AYACDFSPRAVDYVKQHPLYNAERCKVFQCDLTRDDLLDHV
Q5EA33	PLHLAAGNRDSKDTLELLLMNRYIKPGLKNSLEETAFDIAR
Q9D8C2	NREQQGQLLEVGWNNTASARNDIQRNLNCCGFRSYNPNDTC

Q9D6K7	EDLSWARKLQEQQKVAQRIENKEMPPEGPDLSPGSIPDYDF
Q9H3R5	STALKKNLEKISRQSSVLMDNMKHLLELNKLIMKSQQESWD
Q8TAM6	ADEMTFREGHQWEKIPLSGSNQEIRRQKERITEQPLKEEED
Q5EAE3	FEGRKEIWDALKAAAHAFESNDHELAQAIIDGANITLPHGA
Q9NWB6	SRSRDKERVRKRSKSRESKRNRRRESRSRSRSTNTAVSRRE
P41272	SCSKNWQCRDQECTECDPPLNPALTRQPSETPSPQPPPTHL
A8C927	HIDLGTGDGRNIYKLAINDQNTFYIGIDPVKENLFDISKKI
Q9DCC4	LIQAGKVEAKQVLASAPTDNNLCHFRALGCQTTHSNHEVLQ
Q96PB8	SIPNEIFKDLHQLRVLNLSKNGIEFIDEHAFKGVAETLQTL
Q2KIL7	ERLLRFFSVSPQAVYTAMLDNSFERLLLHAICQYMDLISAS
P80206	LNQSPASLSTQGYGASSLGFNSTTDCLDYKDQTASWKLNFN
Q96JC9	PASPPQPSHQQPYNSRPAVANGTSRPQGSNQLMNTLRNDLQ
Q9D7X1	GHYFIDRDGLLFRHVLNFLRNGELLLPEGFRENQLLAQEAE
Q56A07	YQECNNCTEEMFLQFRMKIINLKLERFGDRVEFSGNPSKYD
Q3SX24	FQHGGKDTQFWAGWYGPRVTNSSVVISHRVTRNPPHAMAQP
Q8NH89	LTAVTIFYGTLSYMYLQPQSNNSQENMKVASIFYGTVIPML
O43633	KSNNSMAQAMKGVTKAMGTMNRQLKLPQIQKIMMEFERQAE
Q9QZ49	RIFLLLALLTLVISVTTSWFNSLKPSQGHLKEGEKENEKRR
Q03014	EDILGRGPAAPTPAPTLPSPNSSFTSLVSPYRTPVYEPTPI
Q2YDF9	QTSKYCPMCNIKIHETQPLLNHKLDRVMQDIVYKLVPGLQD
Q7Z692	YEVMPSPVLLVSPISDTRSINPARPLPTPPHLQAEPENHQY
Q9UKJ0	HFHGQSFYSTRPPSIHKDYVNRLFLNWTEGQESGFLRISNL
Q8VCZ2	MFITMCFCWHYLVALCIVAINYSLVYCFLTRVKRRAEGEII
A5PKI3	NVVGPKICLEDNVLMSGVKNNVGRGINVALVNGKTGELIDT
Q91VK4	DTYRLRRRSTRRRINKRGGKNCNAIRHFENTFVVETLICGV
Q9DCK4	QELVAHGKLHYKPNRYFKCENCLLRFRTHRSLFKHLHVCID
Q8N2G6	RGEALSNSVYKGASPYGSLNNIADGLSSLTEHFSDLTLTSE
P97299	PATEEAPKVCEACKTKNEDDNDIMETLCKNDFALKIKVKEI
P33782	SDTSVQRFRASVPVRDPETGNVSGQLSFTLNQGMAVSTGKQ
Q32LB5	DTCENNLCRNKERDKSQRYPNWNPSGTRQLIACNPLYLISV
P32007	LSFWRGNLANVIRYFPTQALNFAFKDKYKQIFLGGVDKRTQ
P40855	QLQDLGHPPKELAGEMPPGLNFDLDALNLSGPPGASGEQCL
Q8IUI4	DNSDHSSVNIMSAFESPFGPNSNGSQSSNSWKIDSLSLNRE
Q9BVG4	KGVNNGGEKRADSGEEENTKNGGEKGADSGEEKEEGINRED
Q5EAB0	DGNPERYDVSILLWKLQFDDNGTYTCQVKNPPDVDGLIGEI
Q3T0V3	ATLERYVETQAKENAYDLEANLAVLKLYQFNPAFFQTTVTA
P21237	HGQGNLAYPGVRTHGTLESVNGPRAGSRGLTTTSLADTFEH
Q32LJ6	TRTAFLAEDFNAEEINLDCTNPRYLAAGSSNAVKLSRFFDK
O43315	CGCVAQAILSRGRFGGVITINVGFSMAVAMAIYVAGGVSGG
P55075	GAETGLYICMNKKGKLIAKSNGKGKDCVFTEIVLENNYTAL
P37141	LNALQEELEPFGLVILGFPCNQFGKQEPGENSEILATLKYV

P56857	IIGVSVFANMLVTNFWMSTANMYSGMGGMGGMVQTVQTRYT
Q149M0	YFSINEEKSWSDSRKDCIDKNATLVKIDSTEERDLLQSQLS
Q5E958	WGSECCTRKTRIIDVVYNASNNELVRTKTLVKNCIVLIDST
Q2KJ15	CKTRTVIYEIPRSQVDPTSANFLIWPPCVEVKRCTGCCNTS
Q5SSG5	FSEVCVPTTTRRLYLPAVVMNGHVHDLQILDFPPISAFPVN
Q61199	SNIKTVKLNLLITGKIVDHGNGTFSVYFRHNSTGLGNVSVS
A6QPL2	FVRADRPPSPVNVTVTHLRANSATVSWDVPEGNIVIGYSIS
Q5VXT5	FFYTMAALVIYLRFHNLYTENKRFPLVDFCVTVSFTFFWLV
Q5E9A0	KECQCKRWHDMEVYSFSGLQNVPPLAPERRSTLEDYSQSLH
Q8BJU2	YMDKVNENAKQDLKEGLLLYNTENNVGLKNAWNIIQAEMRC
Q99JH8	ACSFTTVWMIYSKFKATYDGNHDTFRVEFLVVPTAILAFLV
Q8WV48	SALSGVLGDRANPDLRAHPGNAAHPGSGATEPRRRPPLKDQ
Q86SE9	VQHFEDSNDCPRCGNQVHETNPLEMLRLDNTLEEIIFKLVP
Q9NW97	YALTAIGLGMLVLGVIMAMWNLVPGFSAAEKPTAQGSNKTE
Q61470	AKAHIYREGCAQSLQKWLHNNIISIVGICLGVGLLELGFMT
Q86WN2	DLKLIIFQQRQVNQESLKLLNKLQTLSIQQCLPHRKNFLLP
Q2KJD9	TLLSLYMFCELVTGVWEGQYNFFCQGTRSGGEADMKIIRVL
Q9DCS2	DVDQRCLDSIAATTRAQGLSNVKAPLYLDVTWEWEQWGGIP
P28230	MVLVVAAESVWGDEKSSFICNTLQPGCNSVCYDHFFPISHV
Q9CQV8	RYDDMAAAMKAVTEQGHELSNEERNLLSVAYKNVVGARRSS
Q91X79	DGTEQYVNVQKIVSHPYWNKNNVVAGYDIALLRLAKSVTLN
Q9BT09	LHNLVHKGVKVVMDIPYELWNETSAEVADLKKQCDVLVEEF
Q9BPW8	PGPRAGDVASAAAARFYSKDNEGSWFRSLFVHKVDPRKDAH
Q9CWU6	WEDVEQRGRVMGVNSYILKKNMALMTNNFYAAILGYDEGIL
Q3TDE8	FSSPETDEKLFICAQCGKTFNNTSNLRTHQRIHTGEKPYKC
Q8MJ87	LKGMGYSMRAARQALHQAAGNLEEALKILLHNPQLWWLNDS
A8MTL3	QEAQQALVSQDKELSVLRKENGELKKFLAILKESPSRYQGS
Q5EA90	GETFSQLASQKDENKLILPPNPAFGSKAASYSSMGNSRPFF
Q8BGJ9	NFFEEVFTELQEKYGEIEEMNVCDNLGDHLVGNVYVKFRRE
Q8BJQ4	IVFAEMPCGRLHLQNLDDAVNGSAWTILLLTENFLRDTWCN
Q16594	KNILITTNMMSSQNTANESSNALKRKREDDDDDDDDDDDYD
P45379	RRAERAEQQRIRNEREKERQNRLAEERARREEEENRRKAED
P07146	SSCAPAGTQCLISGWGNTLSNGVNNPDLLQCVDAPVLPQAD
P14190	IAFTDYEGASVVLRNPDGETNKKGLAYFVLPMKNAEGTKVG
Q5T0T0	RLKAYNRVIYVQNCPETSKKNIFEKSPLTEPNFENKHGYGI
Q3UF25	VDNFLMRKGKTKAKLEERGANQDSRNGSKVRYRRAASHEES
P50225	LALLPQTLLDQKVKVVYVARNAKDVAVSYYHFYHMAKVHPE
Q05685	HKTKPGPEDKLHDQCSPWKKNACCSVNTSQELHKADSRLYF
Q2TBH0	FRGAPMLLAENCSPGPRYSVNPKILRTGKDLGPAYSILGRY
Q9CR00	VSDIQDLMRRKEEIEAEIKANYDVLESQKGIGMNEPLVDCE
Q9CRA4	VGVFSSASLAVEYVDSLLPENPLQEPFKNAWVYMLDNYTKF

Q8BHI7	QIGYMISLIALFTNFYIQTYNKKGASRRKDHLKGHQNGSVA
O15194	LKKQRSRSILSSFFCCFRDYNVEAPPPSSPSVLPPLVEENG
Q91WR8	HILFVNVASFCGLTATYPELNTLQEELKPFNVTVLGFPCNQ
Q2HJ61	AAVSQSLTDLAKYMTGRLRPNFLAVCDPDWSRVNCSAYVQV
Q91WR8	LFMAALAQETLNPQKSKVDCNKGVTGTVYEYGANTIDGGEF
P05833	ALPRLEVAQALYTFLASLPSNPAPISFERLRERLSLISQVK
O15266	KGVILGTANHLDACRVAPYVNMGALRMPFQQVQAQLQLEGV
Q15040	QDSNAFTRDTLQEIFQRLSPNTMVTPHKKSMLGNGNYDVNV
P43489	GPGFYNDVVSSKPCKPCTWCNLRSGSERKQLCTATQDTVCR
P56177	SALANGRALSAGSPPVPPGWNPNSSSGKGSGGNAGSYIPSY
Q9D6S7	FATKKAKAKGKGQPQARVTVNRALVEDIISLEEVDEDMKSV
Q3UTB7	KQRRSTVPLPLAPGRRAPLENPWRLPQAISPEGRLWSRPPL
P05529	EGTLKRNFSGGELVSLPQVINLSGTLRNEEVLDLICCFNKL
Q99LS3	EHPPHLTPGIRELVSRLQERNVQVFLISGGFRSIVEHVAAK
Q3UC65	DRMELLEIAKANAAKALGTANFDLPASLRAKEASQGTAVSS
Q3U1J1	RERDEEEEAARGRRRRQRELNRRKYQALGRRCREIEQVNER
Q96G25	LLSQVADLKNSLGSFICKLENEYGRLTWPSVLDSFALLSGQ
Q9H6K1	TWAPAPDTWAPAPDQTEQDQNRLSQNSVNLSPSSHANNLSV
P31096	AVATWLKPDPSQKQTFLAPQNSVSSEETDDNKQNTLPSKSN
Q08E20	KTNYRMYSYVTKELPQLVNDNFPVDPQRMSVFGHSMGGHGA
H3BPM6	TQGTFLREYDGRSDLHVGITNTNGVVYNYSAHGVQRDGEGW
P49788	RLGKCSARVFFKNQKPRPTINVTCTRLIEKKKRQQEDYLLY
Q8IY95	HLVFVVLAFLTGVLCSYPNPNEDKCPGNYTNPLKVQTVIIL
P31098	NRCELSKELMPKAKDKNKHSNLIESQENSKLSQEFHSLEDK
Q6IR41	MLPLVPGDRVWVRLFKRERENGIYSDDVDTYITFSGHLIKA
Q9CVB6	LENLPASKDSIVHQAGMLKRNCFASVFEKYFQFQEEGKEGE
P46777	NRFGMDKIYEGQVEVTGDEYNVESIDGQPGAFTCYLDAGLA
Q9UF02	SATPFPLVSLFFMFIGFILNNIGHIRPHRTILAFVSGIFFI
Q9BQB4	ATEIIPELGEYPEPPPELENNKTMNRAENGGRPPHHPFETK
Q9NQ88	VDEPLSETGFKQAAAAGIFLNNVKFTHAFSSDLMRTKQTMH
P11034	VSKSESTQQRIKVEKQIIHKNYNVSFNLYDIMLLKLEEKAE
P08217	SLQYSSNGKWYHTCGGSLIANSWVLTAAHCISSSRTYRVGL
P10412	VSLAALKKALAAAGYDVEKNNSRIKLGLKSLVSKGTLVQTK
Q8CHJ2	ASANPTVALQEFLMVEASLPNTLLKLSAQVLGAQAACALTQ
P11233	DLEDKRQVSVEEAKNRAEQWNVNYVETSAKTRANVDKVFFD
A1A4M4	EQRQVLIRQVQLAKRLNLPLNVHSRSAGRPTISLLNEQGAD
Q3T062	DEAEPREDDNDSAAEATAGANAGWADAMARILSKKIPESKP
Q8MKI3	APKIPEGEKVDFDDIQKKRQNKDLMELQALIDSHFEARKKE
A2VE33	TTASPHQVGSGNMECPACYGNNETSCNETRKCYGERCVSII
Q2KI56	EWCDGCEAVLLGIEQQVLRANQYKENHSRTQQQVEAEVTNI
P68002	GTEFGGSIYQKVCEDLDTSVNLAWTSGTNCTRFGIAAKYQL

P97805	TPKDIQTPKSKCGLSKICPNNFFAFKISSGAANVVGPSMCF
P40429	ISGNFYRNKLKYLAFLRKRMNTNPSRGPYHFRAPSRIFWRT
A0PG75	KLTIGKISKYWSGFVNDVFTNADNFGIHVPADLDVTVKAAM
Q5QJU3	VVSVLSAVTTCLAFVKPAINNISLMTLGVPCTALLIAELKR
Q32KY3	NMRNSMQELQRNFGHLSMDPNGHSFSSSSVMTYSKVGDEPP
Q9D7W4	LELAAGILAFVFKDWIRDQLNLFINNNVKAYRDDLDLQNLI
P58499	WVFIAAKGLELPSEIQREKINHSDAKNNRYSGWPAEIQIEG
Q3UV48	HSFPRSLCLVTSLELLNLNNNDIQTLPDELYLLCRLGRIAW
P78330	SGGFRSIVEHVASKLNIPATNVFANRLKFYFNGEYAGFDET
Q5E9Q4	TAVESVTDSSRYFVIRIEDGNGRRAFIGIGFGDRGDAFDFN
P10036	LVEEPIDMDSPEIRELEKFANEFKVRRIKLGYTQTNVGEAL
O95292	APRRYCVRPNSGIIDAGASINVSVMLQPFDYDPNEKSKHKF
P31096	QTFLAPQNSVSSEETDDNKQNTLPSKSNESPEQTDDLDDDD
Q8C7N7	LFRLAYYKLLKKASEGLKSINPEETAPSMRLLAYVSGLGFG
Q8CDZ2	RVIATDLPELLGNLQYNISRNTKMKCKHLPQVKELSWGVAL
Q8BHL8	NGMIINVLELGTQQVADLTLNLDDYIDAEDLSDFHRTYKNS
A1A4K8	KYGEVEEMNVCDNLGDHLVGNVYVKFRREEDAEKAVIDLNN
Q8N3J9	HTGEKPYVCSECGRGFSNSSNLCMHQRVHTGEKPFKCEECG
Q9DCL8	ILATYHPADKDYGLMKIDEPNTPYHNMIGDDEDAYSDSEGN
Q9NPC6	LGKKVSIPRDIMLEELSHLSNRGARLFKMRQRRSDKYTFEN
Q05718	CAEAGGCLRREGQQCGVYTPNCAPGLQCQPPEKEDLPLRAL
P13598	LHYETFGKAAPAPQEATATFNSTADREDGHRNFSCLAVLDL
Q9BU76	QDQFNWEDVKTDKQRENYLGNSLMAPVGRWQKGRDLTWYAK
P21844	HFTSFRHNSQLCVGNPKKMQNVYKGDSGGPLLCAGIAQGIA
Q66JT5	VYSLPNAPTLADLEDDTQEGNEDHQLEKPHFDCRSAIFELD
Q08DY6	IDVYLCEVEQGSHSSNKTSDNVGTSSSKSKPLEHPQPEKEE
A6QLI0	TKQCSKITLTEPWDPLDIPQNSTFEDQYSIGGPQEQITVQE
Q9H063	TARSHEFSREPSLSWVVNAVNCSLFSAVREDFKDLKPQLWN
Q3T0Z4	FSLLVLLLLGVTLNAIPLILNFVDEDQFFENPISCFEWWFP
Q8N6Q1	EREVSKLVSMNPVEKEHTSQNNEGTPTQKTARLFSKKIFCC
P47758	YQVLIDSMALKNSPSLLIACNKQDIAMAKSAKLIQQQLEKE
Q3SYR2	FLRLRQVGATVEFTVGDKPVNNFRMIERHYFRNQLLKSFDF
Q9JKX6	AVCMDPGLSNCTTHVVTVTINGDDAGNVRPKPKPGDGEFME
Q2TBI8	DEVNVPPLTLSQPLLLGIACNETSAGRASAEFYVQCSLTSE
P02746	PGLAGDHGEFGEKGDPGIPGNPGKVGPKGPMGPKGGPGAPG
P07146	DKIVGGYTCRESSVPYQVSLNAGYHFCGGSLINDQWVVSAA
P15927	MTAAPMDVRQWVDTDDTSSENTVVPPETYVKVAGHLRSFQN
Q8N2M4	ELAAGSGALFFIISDLTIALNKFCFPVPYSRALIMSTYYVA
Q9D1C9	PGFQVAYVVFQKPSGVSAALNLKGPLLVSTESHLVKSGIHK
O75954	FWLCGCGLLGVGIWLSVSQGNFATFSPSFPSLSAANLVIAI
P53346	QQDLPEAAHQQAEMNVRGFGNNIHCMAQLLRGSSDPKAAEP

Q0VC58	EVTRTWKIVGGVTHANSYYKNGWIVMIAVGWARGAGGSIIT
Q969Q5	LEEDRRRRRVDFHDVQDYADNIKAQLFETSSKTGQSVDELF
A1A4M2	LSQRQLNEEERGRLRDVAALNGLYRVRVPRRPGAPDGPEAG
Q60932	FGIAAKYQVDPDACFSAKVNNSSLIGLGYTQTLKPGIKLTL
Q9NV56	FSSSGSLGKASEKSSKDKEKNSSDLGCKEGADKRKRSRVTD
Q99N20	LKIRIQVAGIYKGFCLDVIRNKYECELQGAKQHLESEKMLL
O43399	WHDVQVSSAYVKTSEKLGEWNEKVTQSDLYKKTQETLSQAG
P51557	ELSYIQQGEVAMQKALGILNNQEGWKKESQQENGDEVLSKM
Q9D0U6	ESPLSDKCSRKTLFYLIATLNESFRPDYDFSTARSHEFSRE
Q8BH10	TTVLVAVHLFALLISTCILPNVEAVSNIHNLNSISESPHER
Q8N5S3	KEDKHQQHKIEDAAITYVSENEEIKHEEKPGKSIHHSKSHV
Q99727	KQYLLTGQVLSDGKVFIHLCNYIEPWEDLSLVQRESLNHHY
Q6QD59	QSAREQDKESEKQLLLQEVENHKKQMLSNQTSWRKANLTCK
Q6P5C5	EPLPCTRSLAEGFLEEELRLNAELSQLQFPEPVGVIYNPVD
Q4VA44	HTGEKPYVCSECGRGFSNSSNLCMHQRVHTGEKPFKCEECG
P14106	RTINSPLRPNQVIRFEKVITNANENYEPRNGKFTCKVPGLY
Q9D902	ALGDQILFVSRPDKKKILFFNDKSCQFSVDEEFQKLWRSVT
P09326	RVRLDPQSGALYISKVQKEDNSTYIMRVLKKTGNEQEWKIK
Q9NPL8	PAFIHAKQQYIEQSQAEIYHNRFDAVQSAHRAATRGFIRYG
P40198	PSHSSAFSMSPLSTAQAPLPNPRTAASIYEELLKHDTNIYC
Q13007	CYLVHTLLEFYLKTVFKNYHNRTVEVRTLKSFSTLANNFVL
P47963	TIGISVDPRRRNKSTESLQANVQRLKEYRSKLILFPRKPSA
Q9R0Q3	TAVKHEQEYMEVRERIHRAINDNTNSRVVLWSFFEALVLVA
Q9Y3A0	LVSELIPWAVQNGRRAPCVLNLYYERRWEQSLRALREELGI
P54107	QEEIVNIHNALRRRVVPPASNMLKMSWSEEAAQNARIFSKY
A5D7P8	EPNPDYRGQQNKGAHNEQKNNSMNNSNNVGTGTFGPMGNGV
Q8NC96	ARPKLDLGFKEGQTIKLCIGNITNKKGGASKPRTARGGGLS
P30048	YPLDFTFVCPTEIVAFSDKANEFHDVNCEVVAVSVDSHFSH
P56597	RSGFTIVQRRKLRLSPEQCSNFYVEKYGKMFFPNLTAYMSS
O35740	HPYPHNHYMPDLHPTAGHQMNGTNQHFRDCNPKHSGGSSTP
P97760	CTCEEFCPECSVEFTLDVRCNEDQTRHVTSRDLISNSPRVI
Q9D6J6	EVLQVPPMRVYEVATFYTMYNRKPVGKYHIQVCTTTPCMLR
P51157	YKQTIGLDFFLRRITLPGNLNVTLQIWDIGGQTIGGKMLDK
Q969F1	TEKKEGEENIGGVEWLQIKDNDFSYRPNMICNFLHENEDEE
Q3ZBW4	RNLAMGVNLTSMSKILKCAGNEDIITLRAEDNADTLALVFE
A2RU54	LASSLQLTETQVKTWFQNRRNKWKRQLSAELEAANMAHASA
P54107	RIFSKYCDMTESNPLERRLPNTFCGENMHMTSYPVSWSSVI
O75015	APRWVFKEEDPIHLRCHSWKNTALHKVTYLQNGKDRKYFHH
Q15181	VLGILAMIDEGETDWKVIAINVDDPDAANYNDINDVKRLKP
Q8IV03	HSSALERLETKLHLLRQEMVNLRATDVRLMRQLLVINESIE
P10767	VFLGILVGMVVPSPAGTRANNTLLDSRGWGTLLSRSRAGLA

Q91WR8	LFGSPEHLFWDPMKVHDIRWNFEKFLVGPDGVPVMRWFHHT
P26892	LLLVMTSAFPTPGPLGEDFKNDTTPGRLLLTTPEKTEALIK
P59190	QQLAKEYGMDFYETSACTNLNIKESFTRLTELVLQAHRKEL
O77834	ALSIDSVEDHLAWSKDINAYNGEEPTEKLPFPIIDDKNRDL
Q32L52	ENRESTPKLAKLLKLLLWAQNELDQKKVKYPKMTDLSKGVI
Q8N682	FNLVSLVLGLVGCFGMGIVANFQELAVPVVHDGGALLAFVC
Q8CDW1	SPRERQRASARLMQSKPGGRNRYKGASSEKPVFTLKSHLPK
Q9QWV4	RTHNRRERDDGEDSLTHADVNPFQTMDRMMANMRSGIQELQ
Q8BK26	PVADWKIFYFLRSLQRNLLHNPCAEEGFEFWSLDVNGGDEW
Q8BJ25	AFGNRKNLKHNAVPTVFAFQNPTEVCPEVGAGGDSSGRNMD
Q8N699	LQCPPLPVETESQLVTLPSSNISPTISTSHSLSRPDYWSSN
Q9NWZ8	VYARYWQHYHQAMAWMQSHHNAYRKAVESCFNLPWYLPSAL
Q12829	LPLPIALRSHLKSFSMANGLNARMMHGGSYSLTTSSTHKRS
Q9H560	HYAVYNKGTSLAEKLLSHHANIEALNEEGNTPLLFAINSRR
P15947	ICDGVLQGITSWGPSPCGKPNVPGIYTRVLNFNTWIRETMA
Q8TAF8	ALGMFLIIGSIICFSLFFICNTATVYKICAWMQLAAATGLM
Q9D9B4	LGDDLVLPGLPHLHTLTLNKNQITDLEYLLDHLAEVTPSLE
H7C241	MSMRMFEEDTYNSFVVSGILNIAAGVFNLIAVLQNYDAVIN
Q6PIL6	KKELQILYRGFKNECPSGVVNEETFKEIYSQFFPQGDSTTY
Q17QB7	WDTAGQERFRSITQSYYRSANALILTYDITCEESFRCLPEW
P04467	KEFNKAFELYDQDGNGYIDENELDALLKDLCEKNKQDLDIN
Q9H6D8	SPPGPRVHFRTLKGSDRLPSNSSSPGDITVEGLDGERPLQT
Q13520	VGAALLYGVMPGDIRETLGINVVRNSVSTGQAVAVELLLTL
P49615	TTSLVNVVPKLNATGRDLLQNLLKCNPVQRISAEEALQHPY
Q0IIG7	QRQASPNIVIALSGNKADLANKRAVDFQEAQSYADDNSLLF
P59542	NSALYGLEVRIVASNAWAVTNHFSMWLAASLSIFCLLKIAN
Q3T046	TKKKQIDQFANDIERLDVLFNVAGFVHHGTILDCEETDWDF
Q8CCI5	RQAKPAADEGFWDCSVCTFRNSAEAFKCSICDVRKGTSTRK
O08609	GCREDSSHPACAKVEYAYSDNSLDPGLFVESTHKGSVVSRA
A6NGC4	GIGLVTVGIMSIILGIRILVNDVLQSRPHPPSPGHEKTRGT
Q3T021	KIFQEEESIRQNREESENFRNAFSEPVLSEPLFPEGEIKAK
Q7RTY3	KVGTQCWVTGWSQVKQRFSANSMLTPELQEAEVFIMDNKRC
P39687	EGKLEGLTDEFEELEFLSTINVGLTSIANLPKLNKLKKLEL
Q9QYN3	ATESFPHPDFNNSLPNKDHRNDIMLVKMSSPVFFTRAVQPL
Q9TTJ5	ECVLRENCHCGESPVWEEASNSLLFVDIPAKKVCRWDSLSK
Q9D8T4	QDSNDDTEDVSLFDAEEETTNRPRKSKIRHPVASFFHLFFR
Q28071	FRASLYKGADSAVEVCAVNGNHSHPLQSTNKEFNCTVKVGN
Q91VT4	GRAVAQLMAQKGYRLAIVSRNLEVAKVTAGELGGNHLAFRC
Q8R2H9	AAPGQQLPESLRATYREGYYNEYMQRVFKYLGEQGVRPRDL
O75629	SGVPYFYLSPLQLSVSNLQENPYATLTMTLAQTNFCKKHGF
O88513	QYWKEVAEQRRKALYEALKENEKLHKEIEQKDSEIARLRKE

P04394	ETTPDKLFTLIEVECLGACVNAPMVQINDNYYEDLTPKDIE
P30793	AKSAQPADGWKGERPRSEEDNELNLPNLAAAYSSILSSLGE
Q922M7	PVNHKMEHNNNDTQQNHDLMNRKSPSGPVKSPPLSPVGTTP
Q0VCJ2	PIIENTPEEKDLKERMARAVNDYPDSCAVLVRRHGVYVWGE
Q969T7	RESNAMLREGYKTFFNTLYHNNIPLFIFSAGIGDILEEIIR
Q6MZT1	SSSSPVDSQQHSWQVSTDIENTERDMREMKNLLSKLRETMP
A2A9Q0	TPGCETLASAGGSLRAHVLRNRSVLLQWRLAPAEARRVRVF
O75888	RQETLFRCIRSMPSHPDRAYNSCYSAGVFHLHQGDILSVII
O09061	GDCLTLTKIIEARLKMYKHSNNKAMTTGAIAAMLSTILYSR
Q8BRX9	AKDGQLLSTVVRTLATQSPFNDRPMCRICHEGSSQEDLLSP
Q9Y3A4	HGVRQGTKSTWPQKRTLFVLNVPPYCTEESLSRLLSTCGLV
O14817	QQDLKKGLHLYGTQGNVGLTNAWSIIQTDFRCCGVSNYTDW
Q66JT5	MYKPITYNTSLLTEESDNFANKLDPSKVFKSKNKILIPKKK
P50540	LKVLIPLGPDCTRHTTLGLLNKAKAHIKKLEEAERKSQHQL
Q9CR67	SLLQPFIYYRFLTLRYSSRRNPYCRNLFNELRIVVEHIIMK
Q9JMF7	TLIIFKRELHTISFLGGLALNQGVNWLIKHVIQEPRPCGGP
Q6IB77	NWKQHLQIQSSQPSLNEAIQNLAAIKSFKVKQTQRILYMAA
Q0VD50	QKFIYESDVLWKHQNNIHLVNEWITNDISSTKIRRALRRGQ
P79121	WDQLTLSQRKGLNYRYHLGCNCKIKSCYYLPCFVTSKNECL
Q9EP73	GQNHTAELIIPELPATHPPQNRTHWVLLGSILLFLIVVSTV
Q8NI29	HGATGRALLHLARSCQSPARNARPCPLGRFCARRPIGRNLI
Q03401	YDVGPKQPDSVVGHYTQVVWNSTFQVACGVAECPKNPLRYY
P30048	HFSHLAWINTPRKNGGLGHMNIALLSDLTKQISRDYGVLLE
Q9Y2Y8	NYRIQCCTSTVNQAQVWIGGNLRGWFLWKRFCWTDGSHWNF
Q924N9	WKSNKWIQVGVVSKGIDCSNNLPSIFSRVQSSLAWIHQHIQ
Q9BZD6	FFIHRRLLYNRFDLELFTPGNLERECNEELCNYEEAREIFV
Q9D3J9	YWRITSIKEKSSLQMQKPISNAVLNEYLEQKVVELYKQYIM
P09326	SLLVTSIQGHLVHMTVVSGSNVTLNISESLPENYKQLTWFY
Q9TU03	EALKKETFVLKEGVEYRVKINFKVNKDIVSGLKYVQHTYRT
Q86SE5	NVTNKNDPKSINSRVFIGNLNTAIVKKVDIEAIFSKYGKIV
P05837	QINPFPDYPFFALNQSGSNTNTSDKLTIWNDLSPGTLVVFS
Q60930	RSNFAVGYRTGDFQLHTNVNNGTEFGGSIYQKVCEDFDTSV
Q8R0A6	WLQDTKPRKNASSVVPSSVHNSANQRMHSTSSPQAVAKIPK
Q9UKY3	IQYRLGIWGFFSTGDEHSPGNWGHLDQLAALHWVQDNIASF
P70122	NVAVVRMKRGGKRFEIACYKNKVVGWRSGVEKDLDEVLQTH
P54797	MDSLSYLKKVSTEGHLYNGFNIIAADLSTSKGDVVCYYGNR
Q6PHZ8	IKGLSILLRGTVQEKLNWAFNLYDINKDGYITKEEMLDIMK
Q8CI43	GKILYSQCGDLMRALGQNPTNAEVLKVLGNPKNEELKSRRV
Q8BNL5	RDPQGNYFIDRDGPLFRYVLNFLRTSELTLPLDFKEFDLLR
P56857	RGLTPDDSNFKAVSYHASGQNVAYRPGGFKASTGFGSNTRN
Q2YFS1	SEFTTAGLEHTRDKRNPSLMNLGAMVTMLLAKVVVIILVYG

O08337	HLADGMTVGELCAAAITMSDNSAANLLLATVGGPAGLTAFL
Q9NRH1	YGRLRGTLSALLSWCHLHNNNSTLINKINNLLDAVGQCEEY
A8C927	ADSISILFPWGTLLEYVIKPNRDILSNVADLAKKEAHFEFV
P13949	DESVVLWQINPFPDYPFFALNQSGSNTNTSDKLTIWNDLSP
P00642	SNEYPQLSFRYRDSIKKTEINEALKKIDPDLGGTLFVSNSS
Q9Y6N3	MVTEASTHLFHATKQRAYFRNVSILIPMTYKSKSEYLIPKQ
Q96D70	GLEDGDLAPPASPGIFAEACNNATYVEVWNDFMNRSGEEQE
P05631	IQVRNMATLKDITRRLKSIKNIQKITKSMKMVAAAKYARAE
Q9JKB1	KKFLEESVSMSPEERAKFLENYDAIRVTHETSAHEGQTEAP
Q8R2N1	QFIGTAALIVCVLAIVDPYNNPVPRGLEAFTVGLVVLVIGT
Q8N2C3	VWKEQTLLCLLQLIHLPFLDNILEPPVKTQNLQLNKEEDLV
P49891	NAKDVAVSYYYFLLMITSYPNPKSFSEFVEKFMQGQVPYGS
P16110	TIMGTVKPNANRIVLDFRRGNDVAFHFNPRFNENNRRVIVC
Q9QZ73	SLDRKKLEQLYTRYKDPQDENKIGIDGIQQFCDDLALDPAS
Q80YF6	CVFDEHVSTKDTIWLVVAFSNASRDFQNPQTAAKIPTFPQL
Q8K015	LSGKLTSRGVCMCISTAFEGNLLDSYFVDLVIEKPLRIHHH
Q9H560	ACAIILLEHGANPNIKDIYSNTALHYAVYNKGTSLAEKLLS
P21796	RFGIAAKYQIDPDACFSAKVNNSSLIGLGYTQTLKPGIKLT
Q765N9	ALLTLVPVSWSANTIIRDFYNPLVPEAQKREMGAALYVGWA
P31947	MDISKKEMPPTNPIRLGLALNFSVFHYEIANSPEEAISLAK
O43315	IGLLIIVIASSLGLNSGCAMNPARDLSPRLFTALAGWGFEV
Q6Q8B3	RPVDTTHDGYYRGIVVTPDGNFHRGYHLQVLVTPEVNLFQS
P23184	LHLENYHSDIVKRIYSKFESNPGVAFIQSYYLKESFRIDGV
Q14681	VSTMSDGWKFEQLISIGSSYNYGNEDQAEFLCVVSRELNNS
P61092	IVQLIGTRKQAENFAYRLELNGHRRRLTWEATPRSIHEGIA
Q9Y316	VVCREASHAGSWYTASGPQLNAQLEGWLSQVQSTKRPARAI
Q3SZX2	DLNDEEEDEEMSETAAGESMNMEESSQGSATSDQLQNKSQS
A6NH52	AGISLQAANLYGYILCKMGGNSDIGKVTASFLSQTVFQTAC
Q8NC69	MDTFEEVVELSSTRKLSKYSNPVAVIITQLTITTKVHSLLE
P15530	LLLLFSGEPVPAMTSSDLPLNFQGSPCSQIWQHPRFAAKKR
Q8IXQ9	TLNCELNRLNPFPILIQNILNLEQDKWDLVVLGDMFYDEDL
A6QLM0	PFREHHIDPTAITRHDFIETNGDNCLLTLLPLLNMAYKFRT
Q86XK3	GSCSALQNEFVSEKLPKQRLNAEKAKLVKQVQEKEDLLRRL
Q9NYZ1	CDFWAVKNVTGRLMVGLRWWNHIDEDGKSHWVFESRKESSQ
P41732	HGIPPSCCMNETDCNPQDLHNLTVAATKVNQKGCYDLVTSF
Q9CZM9	FSCGPDCWKLSQYPCLQVYVNLTSSGERLLLYHTEETMKIN
Q9Z0S6	AGASLCIIGGVIFCFSISDNNKTPRMGYTYNGPTSAMSSRT
O95237	EVARRAEKLLGFTPYSLLWNNCEHFVTYCRYGTPISPQSDK
P52651	PMGDGDKDSGTRAGGVEQEQNEPVAEGTESQENGNPGGRQM
O02659	GTPGIPGIPGPIGQKGDPGENMGDYIRLATSERATLQSELN
P15374	KKFLEESVSMSPEERARYLENYDAIRVTHETSAHEGQTEAP

Q9Z262	WVNALVSCALPMWKVTAFIGNSIVVAQMVWEGLWMSCVVQS
P0A1V9	VGWVEWPTGSVFFALNIDTPNRMDDLFKREAIVRAILRSIE
Q1HCM0	GRKAGSDPVPEPPAAAGFQPNGLDQNAELTETYCTEKWHSL
Q6P1X6	EYFYYVDHQGQLFLDDSKMKNFITCFKDPQFLVTFFSRLRP
Q17QQ9	FTRDCFSGKPVPPNLSQHESNCSSYSVESGVYRHLSSENNT
Q497H0	PSTSNSQSDLFSEETTSDNNNTSVTTPTLSPSQQSLPTELN
Q99N94	SVDEIGVRGDLVSVKKSVGRNKLLSQGLAVYASPENRKLFE
Q96A61	EELFQDQDDDELWLGDSGITNWDNVDYMWDEEEEEEEEDQD
Q9D2C7	GFAFLTGVGLGPALELCIAVNPSILPTAFMGTAMIFTCFSL
P20645	EKGKESEKELALVKRLKPLFNKSFESTVGQGSDTYIYIFRV
P48962	LSFWRGNLANVIRYFPTQALNFAFKDKYKQIFLGGVDRHKQ
Q3UFY0	VPISKKVARDPRFDDLSGDYNPEVFDKTYQFLNDIRAKEKQ
Q8WV35	AIGQACRQLRVLDVATCPGINMAAVRRFQAQLPQVSCVQSR
P61588	IDTQRIELSLWDTSGSPYYDNVRPLSYPDSDAVLICFDISR
Q9D0M0	EGRGGDDLGTEIANTLYRIFNNKSSVDLRSLCISPREHCWV
P48299	CFTYKDKECVYYCHLDIIWINTPEQTVPYGLSNYRESLRGK
P49638	KIAAVLTDSFPLKVRGIHLINEPVIFHAVFSMIKPFLTEKI
Q8TAX0	EITAGGSVPALKTKPRFDFANLALAATQEDPAKLGRGEGPG
Q80W21	YEEKRYTMGDAPDYDQSQWLNEKFKLGLDFPNLPYLIDGSH
P27144	TLGQAEALDKICEVDLVISLNIPFETLKDRLSRRWIHPPSG
P63139	ISFITSEASERCHQEKRKTINGEDILFAMSTLGFDSYVEPL
Q6PHN7	GLMFFYHFSFLQILGLVTEVNLNNMLCPAISDPFYGPWYRI
Q9R0U0	RRSRSRSYERRRSRSRSFDYNYRRSYSPRNSRPTGRPRRSR
Q5E9V3	QRRSDKYTFENFQYETKAQINHNIAMQNEKLDGINLESGSQ
P22794	NTSKSHGEIFKKDVCAENNNNMAMLICLIIIAVLFLICTFL
P35509	SSGVLMVGPNFRVGKKIGCGNFGELRLGKNLYTNEYVAIKL
P02666	NVPGEIVESLSSSEESITRINKKIEKFQSEEQQQTEDELQD
P10746	GIAMESITVYQTVAHPGIQGNLNSYYSQQGVPASITFFSPS
P04393	KDALLCDTNPHLISFYNALKNKDITGDLVKDFLYREGEKLL
P52623	RGVDNMVAINLIVQHIQDILNGDLCKRHRGGPNGRNHKRTF
P02253	VSLAALKKALAAAGYDVEKNNSRIKLGLKSLVSKGTLVQTK
Q64695	LCNSDGSQSLHMLQISYFQDNHHVRHQGNASLGKLLTHTLE
Q86UF1	FDYLEASKVIYTNGCIDKLVNWIHSNLFLLGGVALGLAIPQ
Q8IXM7	RVMDLNPAPNQYQMPLLLGPNTPVSRAAPCYSLASRDKNWF
Q3V2Q8	EHNVTFHSVLHAEKPSRANRNQGRNSEPSSGSGYWNTYTEL
Q6PER3	KNPPSARNGGHEADAQILELNQQLLDLKLTVDGLEKERDFY
Q24K06	FKALPQVVCTLKQLCILYLGNNKLCDLPRELSLLQNLRTLW
Q3T0C2	ILVNNAGVNNEKNWEKTLQINLVSVISGTYLGLDYMSKQNG
Q9D8C4	VTFEDPKVVDRLLQQKEHRVNLEDCRLRVQVQPLELPVVTN
P51159	EEAIALAEKYGIPYFETSAANGTNISQAIEMLLDLIMKRME
Q29461	IAVSKSVIHEKWNSNQLAQGNDIALLKLASSVPLTDKIQLG

Q3SYT7	CIEKAYEKILFTEATRILFFNTPKKMTDYAKKRGWVLGINN
Q3T0Z4	DSCVPHTDSNNPSITNTTANNWRVYNLHFNSIENQHRIIHF
Q8R0W6	ESAAYFDYKDESGFPKPPSYNVATTLPSYDEAERTKTEATI
Q9NRY7	PGRQHDYLVPPAGTAGIPVQNQPGRPEGVPWMPAPPPPLNC
P28293	GFLVREDFVLTAAHCLGSSINVTLGAHNIQMRERTQQLITV
Q9H0U4	SYANVKQWLQEIDRYASENVNKLLVGNKSDLTTKKVVDNTT
Q2TBH5	ESQTSFETSNREAAKRRNLPNSVTNISPPAEQPTKKEVLDL
P10923	PQNAVSSEEKDDFKQETLPSNSNESHDHMDDDDDDDDDDGD
P68250	VGARRSSWRVISSIEQKTERNEKKQQMGKEYREKIEAELQD
Q0VCN1	ADLGPVVLSLLKTPEEYVGRNIGLSTCRHTVEEYAALLTKH
Q9TTJ5	EQIPDGMCIDVEGKLWVACYNGGRVIRLDPETGKRLQTVKL
Q61133	WTKVLGPLIGVQVPQEKVERNRDRMVLVLQQLEDKFLRDRA
Q17QD9	ILREDAPLEHVERFFQKMDRNQDGVVTIDEFLETCQKDENI
Q9P0N9	AMEEMVEDSVDCYWITRRFVNQLNTKYRDSLPQLPKAFEQY
P79103	PHLVTHDARTIRYPDPLIKVNDTIQIDLETGKITDFIKFDT
P11403	GVPFFTDECKFKEILLPNNYNAYESYAYPGMFMALSKNGRT
Q969T7	ILLGDSIGDLTMADGVPGVQNILKIGFLNDKVEERRERYMD
Q32LB6	QITEKQFKDKRPGFPFLESENGRKPLPWRKILTFEQAVARG
Q9Y3V2	QAESLLSEPMVHELVLWIQQNLRHILSQPETGSGSEKCTFS
Q9BTT0	LELSDNIISGGLEVLAEKCPNLTYLNLSGNKIKDLSTVEAL
Q15012	WYMVVNLLMAILLTVEVTHPNSMPAVNIQYEVIGNYYSSER
Q96HV5	SLFFFLLFLRLFPMTPNWFLNLSAPILNIPIVQFFFSVLIG
Q16534	DARRLKENQIAIRASFLEKENSALRQEVADLRKELGKCKNI
Q9CZJ0	PWFNGWGFNLPRGQSLLDKWNLIPEGIDILMTHGPPLGFRD
P41047	DTYGTALISGVKYKKGGLVINETGLYFVYSKVYFRGQSCNN
Q2PT27	FIGCQFYDRESQKGCNFSRANLKDAIFKSCDLSMADFRNIN
Q9CQI4	HKVGSSFQGDMTRAGKVKVDNFLDLEDVDMEEESKPQMNKD
Q5E9D3	ELKRRVAEELALEQAKKESENQKRLKQSKELDAEKAFANEQ
P00492	QSTGDIKVIGGDDLSTLTGKNVLIVEDIIDTGKTMQTLLSL
Q96S96	LLDEDTLFCQGLEVFYPELGNIGCKVVPDCNNYRQKITSWM
Q9QXV3	KSEAITQADKPNNKRSRRQRNNENRENASNNHDHDDITSGT
Q2KI06	DCIIISDSNSVFIDWVLEATNFHDVFDKVFTNPAAFDSNGH
Q2KI39	RKKKISQKKQRGRPSSQTRRNIVGCRISHGWKEGDEPITQW
Q13637	QLFSQHYRATIGVDFALKVLNWDSRTLVRLQLWDIAGQERF
P61289	ANFFPKKLLELDSFLKEPILNIHDLTQIHSDMNLPVPDPIL
Q9H019	PIWQNKPHGAARSVVRRIGTNLPLKPCARASFETLPNISDL
P50296	DGVHGLMGTILAYKKFNYKDNIDLVEFKTLKEEEIEEVLKS
Q9H3L0	GYWADFIDPSSGLAFFGPYTNNTLFETDERYRHLGFSVDDL
P50553	AVSAAFQAGVLSPTISPNYSNDLNSMAGSPVSSYSSDEGSY
Q1LZ95	ILVDENDRRIGAETKKNCHLNENIERGLLHRAFSVFLFNTE
P08246	ATINANVQVAQLPAQGRRLGNGVQCLAMGWGLLGRNRGIAS

Q96EC8	EGEITIPMRSRIREFDSSTLNESVRNTIMRDLKAVGKKFMH
Q9NPC6	QQAPLTPPNTPDPRSPPNPDNIAPGYSGPLKEIPPEKFNTT
P04390	LYKPSEPNKKIAIDIKTTYTNKENEKIKFTLGGYTSFIRNN
P39942	VELASLQTSFVTLDEAIKITNRRVNRIEHVIIPRIERTLAY
Q9DAA4	TLSGLDWSQENNGRHPDLRQNLEAPVMTDKDCQKTQQGSSH
Q2NL11	RDYQGQFNTTHLLALCDYFHNTFIRHYKLYQYVLGQDQDVN
Q3UK78	KLVPGLREQELQRELEFWKKNKPQENGQDDISKVDKSKADE
Q2KI56	ISQVLEAPGVYVFGELLELANVQELAEGANAAYLQLLNLFA
Q3ZBK3	QDLKEEYARKKETISTANKANEERLKRLQKSADLYKDRLGL
Q99N09	GLAVLTAMLWWKQSHSNIPGNVMFLPHSSNNDSNMESKVLC
P43276	VSKGTLVQTKGTGASGSFKLNKKAASGEAKPKAKKTGAAKA
A0JNC4	YYFSKFIELLDTIFFILRKKNSQVTFLHVFHHTIMPWTWWF
P30932	SVGLWLRFDSQTKSIFEQENNDSSFYTGVYILIGAGALMML
Q32KU2	MLSGLDWSQNNNGRHPDLRQNLEAPVMSDTACQETEQGKSH
Q96CT7	REAPDTAEKAKSHLEVPLEENVNRRVLEEGSVEARTIEDAI
Q3ZBY3	EEFNAIFTFYDKDGSGYIDENELDALLKDLYEKNKKEMNIQ
A5PJN0	EYTGTMMKIFDKNKDGRLDLNDLARILALQENFLLQFKMDA
Q64105	AATLGDVSKGFLNVNDLAEVNNYWALNLTSMLCLTSGTLNA
Q9Z0F5	VLNVAVLRCHPLTIFTFHVINIWLSVEDHSGYDFPWSTHRL
P49427	PIDYPYSPPAFRFLTKMWHPNIYETGDVCISILHPPVDDPQ
P46782	VAETPDIKLFGKWSTDDVQINDISLQDYIAVKEKYAKYLPH
Q80ZQ3	SITEAVTILGKKLRDYQKQFNVTHLLALCDYSHNTFIRHYR
O88384	APGGRGDLKYGTYTLENEHLNRLQSQRALLLQGTESLNRAT
P0C1Z6	KYQALGRRCREIEQVNERVLNRLHQVQRITRRLQQERRFLM
Q8BGI3	HKFKREWASVMRCIAVFVGINHASAKLDFANNVQLSLTLAA
Q86W74	VNKDVIRLLESLEEQEVKGFNRGTHSKLETMQTAESESAME
E1BC52	VESYDDYRWDKSCDYLFFRNNMPEFHKLKTKLVKKKGTRAY
P20181	RCKEARPVKNGCRGIDDKHWNSQCKTSQTYVRALTSENNKL
Q8NHS0	DIKKAYRKLALRWHPDKNPDNKEEAEKKFKLVSEAYEVLSD
O95484	WLGTLVSCALPLWKVTAFIGNSIVVAQVVWEGLWMSCVVQS
O08604	GECLTQPVNDLCQKLRDKVSNTKVDTHKTNGYPHLQVTMIY
Q9DCW4	KIRVKPDKSGVVTDGVKHSMNPFCEIAVEEAVRLKEKKLVK
Q6ZUJ4	AAPFAAVPCAPENENPAFATNHAPVNAKPHALCPERKPLTS
Q2TBK2	KFLRSCHLEVGMKNNVKWELNPEIVARHFLRNLGVVVAPHA
Q01081	HNKPTFSQTIALLNIYRNPQNSSQSADGLRCAVSDVEMQEH
Q6QLQ4	GHNSGRNPEEKDNFLSRNKENHKPTESSLDEKVAPSKASQT
Q91VU0	NVVGPKICLEDNVLMSGVKNNVGRGINIALVNGKTGEVIDT
P20826	SRVSVTKPFMLPPVAASSLRNDSSSSNRKAAKAPEDSGLQW
Q47066	SKVMAAAAVLKQSESDKHLLNQRVEIKKSDLVNYNPIAEKH
Q9WVJ5	QYPGYRGYQYLLEPGDFRHWNEWGAFQPQMQAVRRLRDRQW
Q96EF9	HLGRHMEALEIAANLENKATNTDHLTTVLYLQLAICSSLQN

Q3ZBL4	GLQAVVHSEVESELINTAYTNVLLLRQLFSQAEKWYLKLQT
B1ANY3	TEGPWPADAPSWMNKPAVDGNSQSEALSLEMAGLSLPSGGP
A6NLC5	SPRYCMISDLFIDNYQVKCINGKMCYVQKQPAPHSHRMSPE
Q9NQZ6	EKMAHVEELRLIHADINVMENTIKQSENDLNKLLESTRRLH
Q8QZV2	CALRYCCSSAEARLDQGGCDNDRQQGVGEPGRTDREGPDSS
Q5T0T0	LFMYVQCKVYVQLWKRLKAYNRVIYVQNCPETSKKNIFEKS
P56748	SFLAFMMAILGMKCTRCTGDNEKVKAHILLTAGIIFIITGM
Q3UF25	AIVMLIVPFFVNAFMFWVVDNFLMRKGKTKAKLEERGANQD
Q9UL46	EMETDKQEKKEVHKCGFLPGNEKVLSLLALVKPEVWTLKEK
Q9HBV1	YALNDRVYIGKRYHYDIRLPNFYQMSTPEIRRSPLTQHFQN
P21237	PLEPPLLFLLEEYKNYLDAANMSMRVRRHSDPARRGELSVC
P09497	EKAKKDLEEWNQRQSEQVEKNKINNRIADKAFYQQPDADII
Q8TF63	GGETPEFPGCHSPAPPENFGNELLPLSAPLQGLSEGLYPPG
P01881	FFPENIHLMWLGVHSKMKSTNFVTANPTAQPGGTFQTWSVL
Q62225	QPFVRRSSARSLQHLCRLVINRLVADVDCLPLPRRMADYLR
Q3ZBL4	SEVLNGLQAVVHSEVESELINTAYTNVLLLRQLFSQAEKWY
B2RUY7	CEPSNEVHCVVADCAVPECVNPVYEPEQCCPVCKNGPNCFA
Q7Z6A9	LNGTTCVKLEDRQTSWKEEKNISFFILHFEPVLPNDNGSYR
Q15528	IDLYELEEEYYSSSSSLCEANDLPLCEAYGRLDLDTDSADG
Q9Y2W7	VVGLSILLRGTVHEKLKWAFNLYDINKDGYITKEEMLAIMK
P14207	HKTKPGPEDKLHDQCSPWKKNACCTASTSQELHKDTSRLYN
P22090	SFDVVHVKDANGNSFATRLSNIFVIGNGNKPWISLPRGKGI
Q9QZ28	NPSKKRELAQATGLTPTQVGNWFKNRRQRDRAAAAKNRLQQ
Q8K1T1	TCMVHPSRVDWTQTFNGTCLNFSGLGLSLPRSPLQASHAQV
Q92530	VGDQPGPNDKKSELLPAGWNNNKDLYVLRYEYKDGSRKLLV
Q61096	AMGWGRLGTQAPTPRVLQELNVTVVTFLCREHNVCTLVPRR
Q2TBP0	DGIVLGADTRATEGMVVADKNCSKIHFISPNIYCCGAGTAA
P01909	LRQFRFDPQFALTNIAVLKHNLNSLIKRSNSTAATNEVPEV
Q3SZ27	VKNGIMDTVQFGKDAYVYLKNPPRDFLPKIGVITVSGLAGF
Q9D504	ACANGYTNIVSLLIENQCKINVQDSENRTPLIKAVECQQES
P37980	HDYWRALVTKKTDGKGISCMNTTVSESPFQCDPDAAKAIVD
Q9JJE4	AFLTGPRLLDWASSPPHLQFNKFVLTGYRPASSGSGCLRSL
Q8NEG0	VYILRPPVEAYSDTRAILAGNTLDSSVLEEVQRSPVGYAMK
Q1LZ95	DAKITFPGCFTNTCCSHPLSNPSELEENDAIGVRRAAQRRL
Q9DCY0	QTAKKLAPSLMDAKNLVVSRNKLTPLDQQLFKFASLDVTHA
Q9CXE2	KEHPGAEDASEEQNSQSSMENSVNSSEKAERQPSAESGLAA
Q3TC33	LLEQERAQIKQEKSRLQPLRNVYLSCLQEEDDWQRRAQHVL
Q91VK4	ISLDKCYVIELNTTIVLPPRNFWELLMNVKRGTYLPQTYII
Q8TBE9	AEIICDKVQVKLSKECFHPYNTCITDLRTSHWEEAIQETKG
Q86UP9	RNSRAIGVLWAIFTICFAIVNVVCFIQPYWIGDGVDTPQAG
P12980	STLGTAPPTLALHYHPHPFLNSVYIGPAGPFSIFPSSRLKR

Q05066	SSSFLCTESCNSKYQCETGENSKGNVQDRVKRPMNAFIVWS
Q6P9H5	RELMEKVEAIMWENEGDYYSNKAYQYTQQNFRLKELQERQV
Q3T0V3	YLGDLLETCHFQAFWQALDENMDLLEGITGFEDSVRKFICH
Q8WWZ3	KENCTCSSCLLRAPTISDLLNDQDLLDVIRIKLDPCHPTVK
Q9WTJ2	HMKCHNDVKRHLCTYCGKGFNDTFDLKRHVRTHTGVRPYKC
Q6ZUI0	KFLEICKLSGFMSKLVPAIQNAHKNSTGSGRGKKLMVLTEP
P01911	RWFLNGQEEKAGMVSTGLIQNGDWTFQTLVMLETVPRSGEV
O60671	LSTILKAIHFREHATCFATKNGIKVTVENAKCVQANAFIQA
O75631	RFGSAVNLQPQLASVTFATNNPTLTTVALEKPLCMFDSKEA
A6QQ85	QFSHPSISDSDAIWLVVAHSNATQKFTAPQKVEDTPVPADF
Q14353	YDTYPLSEETWHTHQFNFIKNHAFRLLKPGGVLTYCNLTSW
P51153	DITDEKSFENIQNWMKSIKENASAGVERLLLGNKCDMEAKR
Q8HZJ5	LLALGDSGVGKTTFLYRYTDNKFNPKFITTVGIDFREKRVA
P43528	GGLIPRGQDEAYERGTPININLYDHARGTATGNTRYNDGYV
P79099	VLAEFLATLLFVFFGLGSALNWPQALPSVLQIAMAFGLAIG
Q8VD53	LFFILPPICMCLFRQYATCFNSGIYLIWTLLVVVGIGSVYF
Q96CN7	VSERCKVRLVPLQIQLTTLGNLTPSSTVFFCCDMQERFRPA
Q91WB4	FFLFNDILVYGNIVIQKKKYNKQHIIPLENVTIDSIKDEGE
Q96AT9	IEVDGGVGPDTVHKCAEAGANMIVSGSAIMRSEDPRSVINL
Q6I9Y2	EVIRKRLLIDGDGAGDDRRINLLVKSFIKWCNSGSQEEGYS
O88346	KRQTGREMKLRILSERKKPLNIDYMGEDQLREKAQELSEWI
Q9CQV8	THPIRLGLALNFSVFYYEILNSPEKACSLAKTAFDEAIAEL
Q8VC04	FSLTIFFLYPRPIAVRPVGLNSSTVTFEDAHVQLNTTNVLN
O89116	LEQMDLEVREIPPQSRGMYSNRMRSYKQEMGKLETDFKRSR
O08899	SRNMLQPTITCFDEAQKKIFNLMERDSYRRFLKSRFYLDLT
O54917	PSLLVDPAQETVRRRCRDPINVENLLPSKIRINLEENVQYV
P17482	LETSAGREAVLSNQRPGYGDNKICEGSEDKERPDQTNPSAN
Q29RZ1	AAFNLQVATPGGKTMDFVDVNESNARWVQDFRLKAYASPAK
Q8N983	ASSRGAREFVEREVIDFARRNPGVVIYVNSRPCCVPRVVAE
P58774	EKLEQAEKKATDAEADVASLNRRIQLVEEELDRAQERLATA
Q13007	VPQKLWEAFWAVKDTMQAQDNITSARLLQQEVLQNVSDAES
P70377	KSNACKCVSSPSKGKTSCDKNKLNVFSRVKLFGSKKRRRRR
Q8R0J7	LQVLFEAYQIKKTKLDKQSNNASLETLLALLQAEGAKIEED
P07146	PAGTQCLISGWGNTLSNGVNNPDLLQCVDAPVLPQADCEAS
Q9QZ23	LQGSCTSCPSSIITLKSGIQNMLQFYIPEVEGVEQVMDDDE
Q8BW74	SVMDLSSRATAPLHPGIPSPNCMQSPIRPGQLLPANRNTPS
Q9D720	YALVNLATTSVSKMATDFAENELDLFRKALELIVDSETGFA
Q00262	NRIRGKLKSIEQSCDQDENGNRTSVDLRIRRTQHSVLSRKF
Q9DAZ2	LVRTPETLLTLVHSLLNSWTNALNHLVNEMSTMQGDTSSLI
Q5BKY1	SLPPELGQLQNLQILALDFNNFKALPQVVCTLKQLCILYLG
P15428	ILVNNAGVNNEKNWEKTLQINLVSVISGTYLGLDYMSKQNG

P09428	KVYPKRNMEKRFVFYKTEIKNTVEFESVLYPNWYISTSQIE
P62906	AVGHVKMTDDELVYNIHLAVNFLVSLLKKNWQNVRALYIKS
Q8WV22	HLPCVAKYFQSNAEPRCPHCNDYWPHEIPKVFDPEKERESG
Q3UUI3	TIIDVTTGTCAISEGVAMTANLNITYKKPIPLLSVVVVNSQ
Q9H1Y0	DHKQLWMGLQNDRFDQFWAINRKLMEYPAEENGFRYIPFRI
Q8WVF5	PEEFSISSNIIQFKYFIKSENGTRLVLKEDNTFVCTLETLK
Q8WUK0	DENVRGVITMNEEYETRFLCNSSQEWKRLGVEQLRLSTVDM
P20040	FDSDVGEFRAVTELGRPDAENWNSQPEILEQKRAAVDTYCR
P40293	LQCTHNGSNTNVTWWHVLQSNSSWPPVMYRGDVGAGGELII
Q925N4	ADDSLEVSTKCRGLWWECVTNAFDGIRTCDEYDSIYAEHPL
P09611	NLLLCQGVEDYAPYCKNQPGNCRIPLQSLFERATLVASNNY
Q9Z2Y8	LSSCPEIKWHFIGHLQKQNVNKLMAVPNLSMLETVDSVKLA
Q9ESG4	LLCNITQRVSFWFVVTDPSNNYTLPAAEVQSAIRKNRNRIN
O14618	IDGLEPGLHGLHVHQYGDLTNNCNSCGNHFNPDGASHGGPQ
Q8IYS5	PGPVVGPGANVSLRCAGRLRNMSFVLYREGVAAPLQYRHSA
P00405	LHSWAVPSLGLKTDAIPGRLNQATVTSNRPGLFYGQCSEIC
Q9UBK7	FNFAKKFSLPLYFVSAADGTNVVKLFNDAIRLAVSYKQNSQ
Q3MHK4	VVGFVTSFVGWIGIIVTTSTNDWVVTCGYTIPTCRKLDELG
Q00688	DSFLAEHKLLGNIKNVAKTANKDHLVTAYNHLFETKRFKGT
Q64267	LLKDCDLEKREPALRFLVKKNPRHSQWGDMKLYLKLQVVKR
P36980	EEGWSPTPKCLRLCFFPFVENGHSESSGQTHLEGDTVQIIC
Q8CDW1	VQPLQQRITDKITSYRRPGKNQVKYEHCLKQTNKPTKVSSS
Q16890	SFEERVETTVTSLKTKVGGTNPNGGSFEEVLSSTAHASAQS
Q8BFZ6	KQDQIIGADTKKNCHLMENINKGLLHRAFSVVLFNMKNELL
Q8VC04	IGPLASEQMPYEVASRILDENTYKICTWPKIRVHHILLNIQ
Q8VCE6	DITGAEPHPSWEHILFTSCHNYHLQLQPPRRRLHSWADDWK
Q8VCG9	RETTSQVAKQRKPWMCKKHRNKMYKDKYKKKKSDQALGSGG
P06340	INVPPRVTVLPKSRVELGQPNILICIVDNIFPPVINITWLR
A9UHW6	FICNIFDYLRVNNMPMMALVNPVYDCLFRLAQPDSLSKEEE
Q8N9E0	LENKKTGSKALAEFEEKMNENWKKELEKSREKLLSGNESSS
P08865	ADHQPLTEASYVNLPTIALCNTDSPLRYVDIAIPCNNKGAH
P23463	TERQVKIWFQNRRMKWKKENNKDKFPASRPEAKDGDPKKEV
P23709	YKAANWYEREIWDMFGVFFANHPDLRRILTDYGFEGHPFRK
E1BE10	KVLITGSLNWTTQAIQNNRENVLIVEDEEYVRLFLEEFERI
Q01081	KYGEVEEMNVCDNLGDHLVGNVYVKFRREEDAEKAVIDLNN
Q0II86	RTEKMVDKMEQDLKTSQKHINSIKSVFGGLVNYFRSKPAET
P52744	HTRENFYKCEECGKTFNWSTNLSKPKKIHTGEKPYKCEVCG
P41732	GVWGKLTLGTYISLIAENSTNAPYVLIGTGTTIVVFGLFGC
A2AQ14	HLAWDLSRSLGALAFSKVTNNVVLLEPFLVGIEGFLKSSTY
P01912	SDVGEFRAVTELGRPDAEYWNSQKDLLEQKRGRVDNYCRHN
Q9NPB1	DITGAEPTPSWEHVLFTACHNQHLQLQPPRRRLHSWADDWK

Q9UNK0	DLANLVENTDEKLRNETRRVNMVDRKSASCGMIMVILLLLV
Q8C436	TLGADVIVTDLEELQDLLKMNIDMNKHLVTGSVQAKVLKWG
Q9CWH6	VEYAQEAVKKGSTAVGIRGTNIVVLGVEKKSVAKLQDERTV
Q2KI69	PQGDSTTYAHFLFNAFDTDHNGAVSFEDFIKGLSILLRGTV
Q8IX29	NLAELDQLWMLKCLRFNWYINFSPTPFEQGIWKKHYIQMVK
P33681	LSVKADFPTPSISDFEIPTSNIRRIICSTSGGFPEPHLSWL
Q1RMX6	PEDVLTLSSQGGQKHRALQANKASALTTNTTQGRSKTKSQM
Q5E9B7	AGLDIFAKFSAYIKNSNPALNDNLEKGLLKALKVLDNYLTS
Q9D701	QNFTAPRKVEDRHAPANFDRNGYYLTLRANRVHYKGGQPDS
Q2HJ38	VKYAEKQERRFEPEKLREGRNIIGLQMGTNKFASQQGMTAY
Q3ZC52	LEKVAQKMQAPHKIHWIEKANHSMAVKGRSTNDVFKEINTQ
Q58CX6	QKLLKFLNKHYNLETTVPQVNNFVIFEGFFAHQHPPARKLP
P30084	RGPLRPPVRCPAWRPFASGANFEYIIAEKRGKNNTVGLIQL
O35526	LEITGRTTTSEELEDMLESGNPAIFASGIIMDSSISKQALS
Q2TBH5	LDSESQTSFETSNREAAKRRNLPNSVTNISPPAEQPTKKEV
O88668	FDPQSPLCVHIMMSGTVTKVNKTEEDYARDSLFVRHPEMKH
Q78PG9	GQIGFHRQKDVKIVTVEKKVNEILNRLEKTKLEKFPDLAAE
P59542	VVSRIGLLWVMLFLWYATVFNSALYGLEVRIVASNAWAVTN
Q9CR10	DCCQSLHRHRAPVQVTDSRRNLGMDHREEGFQVGTEDDREP
Q9D198	EQRLRKFRELHLKRNEARKLNHQEVVEEDKRLKLPANWEAK
Q9BU76	ATSSPTSPERPRHHHHDSDSNSPCCKRRKRGHSGDRRSPSR
Q16772	QQVPMVEIDGMKLVQTRAILNYIASKYNLYGKDIKERALID
Q60809	QLLRCNVDLLKIIQLGLTFMNEQGEYPPGTSTWQFNFKFNL
P62261	AGMDVELTVEERNLLSVAYKNVIGARRASWRIISSIEQKEE
Q5TAG4	KRRRGRKEGEEDQNPPCPRLNSVLMEVEEPEVLQDSLDRCY
Q9D3J9	DVIRETYLVPPSCKSICKNYNDLHIAGGQVMAINSVMANFP
Q9P013	KEQEQKAEEERIRMENILSGNPLLNLTGPSQPQANFKVKRR
Q3T014	SYNVTPPPIEESHPYYHEIYNDRKYKVCDVPLDQLPRSESL
P31268	YTAGASLFQNAEPTSCSFAPNSQRSGYGAGAGAFASTVPGL
P0C8J7	DIQQTIKLGICKINVATELKNAFSQALKNYLTAHPEATDPR
P14415	THYGYSTGQPCVFIKMNRVINFYAGANQSMNVTCAGKRDED
O35943	LNIKKQSVCVVHLRNLGTLDNPSSLDETAYERLAEETLDSL
Q8TDN7	HVLISITFPYGMVTMALVDANYEMPGETLKVRYWPRDSWPV
Q9QXS8	KDYVAQCLQTDMADITRSYLNAHGSFWVAESGGLVVGTVGG
Q9ERB0	LRRLQDAELDSVPKEPSSTVNTEVYPKNSTLRTYHQKIDSN
Q6X4U4	ILYSHVVKPVPAHPSSNSTLNQARNGGRHFSNTGLDRNTRV
Q9JJR5	RRKPPEMHMYSDTGHSQEQRNCRGETSVGQESIYQTSEHSQ
Q80YT9	SEPANQSFESCAKINSHSFRNDEMAGRPSFPVPFFPITSAA
Q16653	GLIFLCLQYRLRGKLRAEIENLHRTFDPHFLRVPCWKITLF
O95475	NPSKKRELAQATGLTPTQVGNWFKNRRQRDRAAAAKNRLQQ
Q3T0G5	SHGQRTFGENYVQELLEKASNPQILSSCPEIKWHFIGHLQK

Q9QWZ1	ITTQEPEETLDFDFCSTNVMNKIILQSEGLREAFSELDMTG
Q13637	IEEAARFLVEKILVNHQSFPNEENDVDKIKLDQETLRAENK
Q8R191	PIVNEGYVNSDSGPELRCVFNGNAGACRFGVVLGLGAFIAC
Q9QWZ1	DVLQITVSPDKPYFRLSTFGNAGNSHLDYPKDSDLVEAFHC
Q28110	KPISAGLTDAENDAARTEAENTVTYSLLSHPDVAEEDSESD
Q9D1C9	AASLRVLEKEKRKRARKELLNFYAWQHRETKMEHLAQLRKK
P35270	YCAGKAARDMLFQVLALEEPNVRVLNYAPGPLDTDMQQLAR
Q3URF8	FTVREAPQIYSLSSSSMEVFNANIFSTSCLFLKLLGSKLLY
Q29461	AANRQWQVHGIVSFGSSLGCNYYRKPSVFTRVSNYNDWISS
Q0IIG7	EESFARAKNWVKELQRQASPNIVIALSGNKADLANKRAVDF
O95452	SIFFRIIFEAAFMYVFYFLYNGYHLPWVLKCGIDPCPNLVD
Q8BJN4	WCLRCHEPRWSYRAGQMEEANGLVRWPEETPALGQREEDLQ
Q9QWV4	ERMAVGHHIHDRGHVIRKSKNNKTGDEEVNQEFINMNESDA
Q9BZJ3	ASETFPPGMPCWVTGWGDVDNNVHLPPPYPLKEVEVPVVEN
Q9EQU5	GLPKGEKEQQEAIEHIDEVQNEIDRLNEQASEEILKVEQKY
Q7M759	SGMRVAFPDTKKTYCFDAFPNIDKISKITSPVLIIHGTEDE
Q9BX73	AALLLGNLLLLHCVSRSHSQNATAEPELTSAGAAQPEGPGG
O75954	LKEGLLLYHTENNVGLKNAWNIIQAEMRCCGVTDYTDWYPV
P41236	DLHDDDEDEEMLETADGESMNTEESNQGSTPSDQQQNKLRS
Q3TZ65	TLENGSGPGLYVLPSTVGYVNHDCTKAASPAYSLARRPSEA
Q9H426	GICIAKKKTKVARKSLDPLYNQVLLFPESPQGKVLQVIVWG
O70338	YTDSMFTINGITNWVQGWKKNGWRTSTGKDVINKEDFMELD
O70273	EGIFRFLKSEAVAQLWGKKKNNSSMTYEKLSRAMRYYYKRE
Q2HJE0	CREKVGEKLCEKIINIVEVMNRHEYLPKMPTQSEVDNVFDT
Q3SZ19	SPASIAGLQVDDEILEFGSVNTQNFQSLQNIGSVVQHSEGK
P20490	AAGTGIAMLILNLTNNFAYMNNCKNVTEDDGCFVASFTTEL
Q96BQ1	AKDLRGKSPFEQFLKNSPDTNKYEGWPELLEMEGCMPPKPF
Q3SYR2	RNQLLKSFDFHFGFCIPSSKNTCEHIYDFPALSEELINEMI
P0CI32	FFPQPADFTGPYVMLTSRRNNRALSGVMINADAGLAIRGIR
Q9DA16	NAFVGKVVVDQLEKVATSFKNQAQYIKANSKTDVPLLEELV
Q16890	LSAKERHLVEIKQKLGMNLMNELKQNFSKSWHDMQTTTAYK
Q6NS38	PSRRVAVVRLPLAHGSLLMMNHPTNTHWYHSLPVRKKVLAP
Q8BQN6	QSSSLTVHVRSHTGEKPYGCNECGKAFSQFSTLALHLRIHT
Q8WXK3	AKCFEYYEKTPLTLSQLCRVNLRKATGVRGLEKIAKLNIPP
Q3T028	PKYFSNELLLKESHQHKPTENFFLPLMPQKKNLRSGLKPVF
P70396	MKNGEMPPEHSPSSSDPMACNSPQSPAVWEPQGSSRSLSHH
P54368	PPLKIPGGRGNSQRDHNLSANLFYSDDRLNVTEELTSNDKT
P28907	WKTVSRRFAEAACDVVHVMLNGSRSKIFDKNSTFGSVEVHN
Q3SZ21	FADLDLRAGSDLKALRGLVENAAHLGYSVVAINHVVEFKEK
Q6PFG8	EMYLRDHHHRHHHHHQESRLNSVSSTQGDMVQKMPGESLSR
Q921H9	GKARDYYSRACDGGYAASCFNLSAMFLQGAPGFPKDMGLAC

Q80UW8	LVDMAPKYVLEQFLQQELLINITEHELVPEHVVMTKEEVTE
Q9BZM6	QWCEVQGLVDERPFLHYDCVNHKAKAFASLGKKVNVTKTWE
Q9BY19	YPNSQPQVHLVPGNPPSLVSNVNGQPVQKALKEGKTLGAIQ
O46471	GSRAHRIFEEFICSEAPKEVNIDHETRELTRTNLQAATAVC
Q0VCS6	FYQLSNTQQDSILQKEEKLGNLSRQLQSLRTQNRKLAETLH
Q8R2M0	VIEDIKRWKTMLELPDQTKENLVAALQELKKKMPSREVLRS
Q9EQC7	DQTGSAHCVVCRAAPCPVPSNPGQELCGNNNVTYISSCHLR
Q92813	ERFSLPPQCRVVADRMDNNANIAYGVAFERVCIVQRQKIAY
Q16635	RLIAECHLNPIILPLWHVGMNDVLPNSPPYFPRFGQKITVL
O35740	GPGTVNGGHPPSALAPAARFNNSQFMGPPVASQGGSLPASM
P05142	LFTVALLALSSAQGPREENQNQIQIPNQRPPPSGFQPRPPV
A0JLT2	MRELPGSTELTGSTNLITHYNLEQAYNKFCGKKVKEKLSNF
Q9DAX2	WSQVNCSGYVQLEVCRGSPANVTEARLSFYSGHSSFGMYCM
Q3UMZ3	TELLPPFQRRIQPEELWLYRNPYVEAEYFPTGRMFVIAFLT
Q5T700	AHCGDPASWIYSDQKCDGTNNCGDCSDELSPVTVCPPCGPG
Q0VD27	EGTTTPIAFVKDILFPYVKENVEEYLQAHWEEEECQQDVRL
Q2KI39	KLKILPDKVPFSRVRDVHLANTIIGKAVEHMFEGEHGSKDG
Q8BHH2	RYVTNKFDSQAFHTIGVEFLNRDLEVDGRFVTLQIWDTAGQ
Q6UX52	KNIKVAKKVVKTHEPASFNLNVTLKSSPDLLTYFCWASSTS
Q9JIM5	QSNYIIDNQEPSCNDSNAYGNVYGYREPQATEQPSSPAPPE
B1AQL3	SYSHLAFVEAARTYGVTHVQNVHISFGWSLALAWASCASEV
Q8TAG5	DMKPRKNVSAAIPSSIHGSANQRTHSTSSPQVVAKIPKQSP
Q9JM71	LTAAHCYGNDTSQHNVWLGKNKLFQREPSAQHRWVSKSFPH
Q8IY95	AFLTGVLCSYPNPNEDKCPGNYTNPLKVQTVIILGKVILWI
P04481	KPGTPELSLERPLFDTEVYVNGEKKYVLPDFIVTARAPDGK
Q0IIL5	NALLVQLPELPSKNLFFNMNNRQHVDQRRQGLEDFLRKVLQ
Q9JHH2	VVVIHFGDHFTVIGHASLERNPYETLRYWAFYVGISLAGLL
Q2YFS1	KGRLILNWTQGQTSGVLRILNFKESDQTWYFCRVFLQTTEG
Q75VT8	SSHEDPIYISCTAPGDILGANFTLFRGGEVVQLLQAPSDRP
A6NNA5	SCCVPDPMGLSFLPTYGCQSNRTASVATLRMKAREHSEAVL
Q9HAV5	LDLQKFSSSASYTGAETLGGNTVESTGDRLELNVPFEVPSP
Q8C1T8	QSSCVASNSHLALFHSLEELNFLKRYKGTSDHWIGLHRAST
Q9D7N2	ASYRPVSYVSSSCRPVSPFMNNCRPVSCVSGGYRPLPCGSN
Q2TBP0	KKLVSEAIAAGIFNDLGSGSNIDLCVISKSKLDFLRPYSVP
Q3T0C2	ASKHGIVGFTRSAAMAANLMNSGVRLNAICPGFVDTPILKS
P00915	QKVLDALQAIKTKGKRAPFTNFDPSTLLPSSLDFWTYPGSL
O02691	KTYNLKKSQAHTLEDFQRVINVNLIGTFNVIRLVAGEMGQN
Q96BQ1	RMIMSPVKNNVGRGLNIALVNGTTGAVLGQKAFDMYSGDVM
O08600	ATNADYRGSGFDRGHLAAAANHRWSQRAMDDTFYLSNVAPQ
A4D1S5	TLAEKYGLLAVLETSAKESKNIEEVFVLMAKELIARNSLHL
Q3TBL6	LNQTAMTMVSFYEVEYTFDTNVLSKLLHECKDLVHELVQRH

P20774	CKANDTSYIRDRIEEIRLEGNPIVLGKHPNSFICLKRLPIG
O75631	VNMSTGLVEDQTLWSDPIRTNQLTPYSTIDTWPGRRSGGMI
P06717	GQSILSGYSTYYIYVIATAPNMFNVNDVLGVYSPHPYEQEV
P14438	VFPKSPVLLGQPNTLICFVDNIFPPVINITWLRNSKSVADG
Q62082	SMFDQSQIQEFKEAFTIMDQNRDGFIDKEDLRDTFAALGRI
Q5E9E3	TGRFRCSVPGYYYFTFQVVSNWDICLSIRSSRRDQIQPLGF
Q9D8C8	QLRKSFRTRCALEETVAEGLNVPRSKRLYRDLVSLQVPEEQ
Q9CY28	IDKSSKGYLLKQVLQIQKFVNTQTQGCFPQLFPISAVTNSG
P08034	ESVWGDEKSSFICNTLQPGCNSVCYDQFFPISHVRLWSLQL
O08989	FVPDYDPTIEDSYLKHTEIDNQWAILDVLDTAGQEEFSAMR
Q9NYW5	LFYFSAIIASVILNFVGIIMNLFITVVNCKTWVKSHRISSS
Q8BW74	GIPSPNCMQSPIRPGQLLPANRNTPSPIDPDTIQVPVGYEP
Q5HZI9	ALRCHGIAEYYRGMVPILFRNGFGNVLFFGLRGPIKESLPT
P10746	FFSPSGLTYSLKHIQELSGDNIDQIKFAAIGPTTARALAAQ
O94778	LVAEIILTTLLALAVCMGAINEKTKGPLAPFSIGFAVTVDI
Q92688	NRIFGGLDMLAEKLPNLTHLNLSGNKLKDISTLEPLKKLEC
Q2KIP7	EQLLRAFILKISVCDAVLDHNPPGCTFTVLVHTREAATRNM
A6NLU0	KDLEEAVQLKCGYACCLQCLNSLQKEPDGEGLLCRFCSVVS
Q8CD60	APRPGTGPDYQLMNGGLPIPNGPRVETPDSSSEEAFSAGPA
Q8N0W7	SYYLCSGSSYFVLANGHILPNSENAHGQSLEEDSALEALLN
Q8VHW4	DAWIRGRSPSDISSDASLQMNSNYPALLKCPDYDQMSSSPC
Q9UBU2	RAKLNSIKSSLGGETPGQAANRSAGMYQGLAFGGSKKGKNL
P19803	PTAEQLAQIAAENEEDEHSVNYKPPAQKSIQEIQELDKDDE
Q5KR48	KLEEAEKAADESERGMKVIENRAMKDEEKMELQEMQLKEAK
P43307	FVKGEDFPANNIVKFLVGFTNKGTEDFIVESLDASFRYPQD
Q00286	LVEEPIDMDSPEIRELEQFANEFKVRRIKLGYTQTNVGEAL
Q99PT1	PTAEQLAQIAAENEEDEHSVNYKPPAQKSIQEIQELDKDDE
Q8VE95	NHSGRYEASFPFLSLCGRERNFLRCEDRPVVFTHLLASDSE
Q8BH58	MMFGDNVLRIQHGSGFGIEFNATDALRCVNNYQGMLKVACA
Q8C552	DQPKILSKNEPIPPSALVKPNANDAQVLMWRPKYGQPLVVI
Q6PB51	GAVTDEWALGAHQGREGHGVNTCPSSLSMPSMLDVVCEEMD
Q9BXJ2	AIGLVHNGQYRIKTFDANTGNHDVASGSTVIYLQPEDEVWL
Q00688	RFKGTESISKVSEQVKNVKLNEDKPKETKSEETLDEGPPKY
Q08DY6	DLNKVATKLGVRKRRVYDITNVLDGIDLVEKKSKNHIRWIG
Q8MJI9	GYPAAETVTSSVDMNTQPYRNLSGVRVGRPKLSLQGVQRGR
P04769	KDLLEYIIRIISKVNPAIKENEDYPTWSDLDSLKSADKETQ
Q99618	PPSPLVKQLSEVFETEDSKSNLPPEPVLPPEAPLSSELDLP
Q03402	KCTFSHSPIELRTTNLKCGENLFMSSYLVPWSSVIQGWYNE
O54957	ENQEPACKNVDADEDEDDYPNGYLVVLPDSSPAAVPVVSSA
O95229	AFEQLQAKKQMAMEKRRAVQNQWQLQQEKHLQHLAEVSAEV
Q0V7M7	IKRTLSLRNCGQEPTLKTILNKIGDEIIVVNELLNKLELEI

Q9N0T5	LCLIGESFDDYSDDVCGAVVNVRAKGDKIAIWTTECENREA
A6NDU8	MYSGEMCYWGSKYCADQQPENHEVDTSVSGAGCTTYKEPLD
Q8K354	DDPQSIRALRDFLRKEYGGLNVLVNNAGIAFRMDDPTPFDI
Q46669	TAHAIAMRNNDAPALVEEVYNFFRDSRDPVHQALNWMILGD
P09017	QIKIWFQNRRMKWKKDHRLPNTKVRSAPPAGAAPSTLSAAT
O09061	DCLTLTKIIEARLKMYKHSNNKAMTTGAIAAMLSTILYSRR
P28230	SRKGSGFGHRLSPEYKQNEINKLLSEQDGSLKDILRRSPGT
Q8N456	NGLPVELKQLKNIRAVNLGLNHLDSVPTTLGALKELHEVGL
Q9NWG9	LKVMHFVARVRNRCSKDWPCNYDWDSDDDAEVEAILNSGAR
Q3UGR5	QDPNAVVIGLAPEHFHYQLLNQAFRLLLDGAPLIAIHKARY
Q60932	GWLAGYQMNFETSKSRVTQSNFAVGYKTDEFQLHTNVNDGT
P97328	QVTWQSQGDTPCSCCIVNNSNGSRTIILYDTNLPDVSAKDF
P38573	QMLERYQNNSPPNNDDQWKNNGVTKTWDRLMLQDNCCGVNG
P53805	EMKLYFAQTLHIGSSHLAPPNPDKQFLISPPASPPVGWKQV
Q7Z3S9	EGFLGEYCQHRDPCEKNRCQNGGTCVAQAMLGKATCRCASG
P86176	QAAFLATAIGATAGTIDTKRNISAEEGGSVILQCHFSSDTA
Q8BVA2	ICTSAGVIRDFAGPYFVSEDNMAFGKPAKFWKLDPGQVYAS
Q58DT1	NKASINMLRIVEPYIAWGYPNLKSVNELIYKRGYGKINKKR
Q9BU20	VPNWHESAEGKEYLACILRKNRRRVFGLLERPVLLPPVSID
O75954	ATTPLWRTGCYEKVKMWFDDNKHVLGTVGMCILIMQILGMA
O15266	DGQTKLKQRRSRTNFTLEQLNELERLFDETHYPDAFMREEL
Q9DBU2	LCGDWFPIEYPDSWYRDITSNKKFFSLAATYRGAIVGMIVA
Q8N9N7	SKNQIRSIPDSVGELQVIELNLNQNQISQISVKISCCPRLK
O35718	YYIYSGGEKIPLVLSRPLSSNVATLQHLCRKTVNGHLDSYE
Q8R2N0	TEQTSSEEPPGGHQPQPEELNTGSSVTFPKNKKKKTSNQKA
Q15800	NPLNLIPFYAGSRHHDFHHMNFIGNYASTFTWWDRIFGTDS
Q9D676	KHPWMWTDNTEYNNMFVYHMNAQCLKKPEEGESSPGTGGVH
Q9D9S3	VNALTNIKRYLEGTREALPPNIEFVITLQIEEPKVTIDLQV
Q9BPW8	ERSQMLLSRRNQLLLEFSFWNEPQPRMGPNIYELRTYKLKP
Q6PGD0	QAYTAIIIDLQANPLKDDLANTFRGFTQLQTLILPQDVPCP
Q86V81	GGTRRGTRGGARGRGRGAGRNSKQQLSAEELDAQLDAYNAR
P50294	LDLATLTEVLQHQMRAVPFENLNMHCGEAMHLDLQDIFDHI
P20039	NSQKDFLEDRRAAVDTYCRHNYGVGESFTVQRRVHPKVTVY
F7BWT7	KTIDKERLNAQNIIHVRGCTNAVLIWFMDNYTIMAGLLLGI
P13366	AKEETQQIIPVAKAIPHPAFNRKHGTNDIMLLKLESKAKRT
Q9D9X8	RTLASNGPNLCRIYCTDLLNNDLKDSIVCAMKIVQEPLGLG
Q3SZC6	ELRNRTPAAVRELVLDNCKSNDGKIEGLTAEFVNLEFLSLI
Q6PHN9	DVTSAESFVNVKRWLHEINQNCDDVCRILVGNKNDDPERKV
Q9H2J4	RAIEMYRRRRLAEWKATKLKNKFGEVLEISGKDYVQEVTKA
Q9QYI6	PDAEAKFREIAEAYETLSDANSRKEYDTIGHSAFTNGKGQR
P35295	NHPQSLFELEDRFLGLTETANNDCLFAIVGNKVDLTSERDT

Q3SWZ6	NVAVVRMKRAGKRFEIACYKNKVVGWRSGVEKDLDEVLQTH
A4FUA8	EDGNVQLVSHKDVQDSVTVSNEAQTAKEFIKIIEHAENEYQ
Q9NWS0	QTTRPESTQIQPQPGFCIKTNSSEGKVFINICHSPSIPPPA
Q3SX64	TEGQIPETGLRKSCGTATLENGSGPGLYVLPSTVGFINHDC
Q8BTQ0	EVPGDIKGEACSAKQHLDPRNGETKADDNSNKETAEEKQEE
P54369	LLEFAEEQLQADHVFICFPKNREDRAALLRTFSFLGFEIVR
P62241	IDVVYNASNNELVRTKTLVKNCIVLIDSTPYRQWYESHYAL
Q9CRB5	MFRKIVLDFLKDHKHMIETLNSCHTFSLSVPETLEEARKIS
Q07326	VPCLCLLGPNLKAWLRVFSRNGVTSIWENSLQITTISSFVG
P13600	VNINNSVFKQYFFETKCRDPNPVDSGCRGIDAKHWNSYCTT
Q3TB82	EGVLTKECRKKAKPRIFFLFNDILVYGSIVLSKRKYRSQHI
P45379	NLEAEKFDLQEKFKQQKYEINVLRNRINDNQKVSKTRGKAK
O43315	GENATAHIFATYPAPYLSLANAFADQVVATMILLIIVFAIF
Q96AJ9	LSHDREKIQRARERLRETDANLGKSSRILTGMLRRIIQNRI
Q5E9K3	VKQFAAWFEEAVQCPDIMEANAMCLATCTRDGKPSARMVLL
Q07133	GMSLAALKKALAAAGYDVEKNNSRIKLALKRLVNKGVLVQT
Q08DN6	MISPPLGDFRHTIHIGSGGGNDTFGDISFLQGKFHLLPGTA
A7Z070	PFPQHLNIITTPPPPDEVFDNSGLSPGFLEYVVGRSDSVST
Q8BHE8	ELVAKEVLQILKEKVTSLSDNHKNALAADIDDIVYTSTGDI
P0C866	HTKHIAFLFDSTLTAFLMMGNLSPVQSTGEREAQRYFEHAL
Q9P299	YYDDTFPSMKEQMVFEKNVFNKTSRTESEIAFFGGMTIVYK
P97371	VHELDEAEYQEIRLMVMEIRNAYAVLYDIILKNFEKLKKPR
Q9UKJ0	VNRLFLNWTEGQESGFLRISNLRKEDQSVYFCRVELDTRRS
Q14B98	PGSPPTPSGTGRRYWFVLKGNLLFSFETRESRVPLSLVVLE
P09024	AAGYGLEPSSFNMHCAPFEQNLSGVCPGDPAKAAGAKEQRD
Q9HAV5	RYKSSWGHHRCQSCITCAVINRVQKVNCTATSNAVCGDCLP
Q8WVD3	CPLCQESNFTRQRLLDHCNSNHLFQIVPVTCPICVSLPWGD
P10746	NATASLVSKIGLDTEGETCGNAEKLAEYICSRESSALPLLF
Q8BQY8	MMKSVVEKMRNISNQLVIEANLKGELNLKIETELVCVTTHF
Q9BPX1	LLGTYTLTKLALPYLRKSQGNVINISSLVGAIGQAQAVPYV
P26452	TIALCNTDSPLRYVDIAIPCNNKGAHSVGLMWWMLAREVLR
Q9UNN8	LWQADTQVTSGVVTFTLQQLNAYNRTRYELREFLEDTCVQY
A8C927	LFPWGTLLEYVIKPNRDILSNVADLAKKEAHFEFVTTYSDS
Q9EP52	VSFLETVNQLHHQNVSVPSNNVHAPFPSDKERMCTVVYFDD
P11836	QTIEIKEEVVGLTETSSQPKNEEDIEIIPIQEEEEEETETN
Q4KL25	GDSVSTPQAGYFGLFSYCVGNVLSSELICKGGPLDFSSIPS
A0JLT2	KKKEEDPDRKRKKEKKKKNRHSPDHPGMGSSQASSSSSL
Q9CR36	KEQKGKGPGGAPPKDLMYSVNPTRVEDLNTFGPKIAGMCRG
Q13795	ILGLDNAGKTTFLEQSKTRFNKNYKGMSLSKITTTVGLNIG
Q99J59	LHCCGFNNYTDFNASRFVKENKVFPPPCCANPGNHTVEPCT
Q9D7W5	LLNQVADLKNSLGSFIYKLENEYDRLTWPSVLDSFALLSGQ

P27814	IGLRFTLPDMNWKWINGTTFNSDVLKITGVTENGSCASILG
Q9CVB6	YITFVLFPRHTNATARDNTINLIHTFRDYLHYHIKCSKAYI
P07477	ISLPTAPPATGTKCLISGWGNTASSGADYPDELQCLDAPVL
P28230	LLYPGYAMVRLVKCEAFPCPNTVDCFVSRPTEKTVFTVFML
Q2T9T3	HVVMTKEEVTELLARYKLRENQLPRIQAGDPVARYFGIKRG
Q96MC5	PLRRYGAVEETAWKTERLGRNQLDIISMAETTMMPEEIELE
P50553	NFSGFGYSLPQQQPAAVARRNERERNRVKLVNLGFATLREH
Q2HJ38	QREQELREWIEGVTGRRIGNNFMDGLKDGIILCEFINKLQP
Q2TJ95	SGFYLHLGKCLDSCPEGLEANNHTMECVSIVHCEASEWSPW
Q921D4	SGSVLDYFSERSNPFYDRTCNNEVVKMQRLTLEHLNQMVGI
P09056	NGSANALFISYYTAQGEPFPNNVEKLCAPNMTDFPSFHGNG
P04467	SNNDGKLELTEMARLLPVQENFLLKFQGVKMCGKEFNKAFE
P26842	HHTRPHCESCRHCNSGLLVRNCTITANAECACRNGWQCRDK
P20783	RCKEARPVKNGCRGIDDKHWNSQCKTSQTYVRALTSENNKL
P10897	ALLFELGTKYSMFEPEGVLANVLGLLLATFATVILYILTRA
Q5E9F2	KLVMASLYHIYVALEEEIERNKENPVYTPLYFPEELHRRAS
Q9CXE7	KSDGVHTIETEDGDYMFCFDNTFSTISEKVIFFELILDNMG
P54107	SEEAAQNARIFSKYCDMTESNPLERRLPNTFCGENMHMTSY
P08311	IQNDIMLLQLSRRVRRNRNVNPVALPRAQEGLRPGTLCTVA
A6NNA5	VFAQTHYPDVFTREELAMKINLTEARVQVWFQNRRAKWRKT
Q077R2	NYYIEKDFVITGVFSSPPEINYGAMLKYCNIACSTVILDRT
Q9JMF7	RIAAWPISEFFLIRDTSLIPNVLWFEYTVTRAEARNRQRKL
Q9JL99	WEDSSVLRKNGINLSGNTEENMNCAYLHNGKIHPASCKERH
Q8VE42	SRDTLELLLMNRYIKPELKNNSQETASDIARRTSIYHYLFE
P58044	GIWGEHEVDYILFLRKNVTLNPDPNEIKSYCYVSKEEVREI
Q08DY9	VDAANLRETFMNLKYEVRIKNDLTCKEMLELMSNVSKEDHS
Q32LB6	KPLPWRKILTFEQAVARGFFNYLEKLKYEYYLKESLKQMNV
Q56P03	STMKTMEDKLSSLGTGSSSGNGKVATAPTRYYDDIYFDSDS
P25789	RRNIHKLLDEVFFSEKIYKLNEDMACSVAGITSDANVLTNE
P35293	VILVYDVTRRDTFVKLDNWLNELETYCTRNDIVNMLVGNKI
P51557	HMRNMKGLRHQAVLAIGQELNWRALGDSSPGWMGQVRRRSS
P54369	GPRWCSDVPHPPLKIPGGRGNSQRDHSLSASILYSDERLNV
P02522	IIFEQENFQGHSHELNGPCPNLKETGVEKAGSVLVQAGPWV
Q99N94	MKNNVKWELNPEIVARHFFKNLGVVVAPHALRLPEEPITRW
P13727	WFTCRRCYRGNLVSIHNFNINYRIQCSVSALNQGQVWIGGR
Q8NDX9	LSDSQIQWFYQALNLSLPLPNFHAGTEPDGLDPMVTLSLNL
P50876	CLESLDDDFLLIHYDKGPCRNKLGHSRASVIWHRTQVVGIF
Q9D7M1	HMMQRQKVWSEVNQAVLDYENRESTPKLAKLLKLLLWAQNE
P48507	LGVAQLDSVIIASPPIEDGVNLSLEHLQPYWEELENLVQSK
P79103	TNRERHPGSFDVVHVKDANGNSFATRLSNIFVIGKGNKPWI
Q91ZR1	SRFAQENELMFLETSALTGENVEEAFLKCARTILNKIDSGE

Q9GZW8	ITIPQREKPGHMYQNEDYLQNGLPTETTVLGTVQILCCLLI
P01909	FALTNIAVLKHNLNSLIKRSNSTAATNEVPEVTVFSKSPVT
Q8BGC9	TGIPFFYMTAKDPAVADLVKNPTASLVLPESEGEFCRKNIV
Q96L15	LPPGFKAQNGIAIMVYTNSSNTLYWELNQAVRTGGGSRELY
Q9BR61	IFDYCRENNIDHITKAIKSKNVDVNVKDEEGRALLHWACDR
P43307	FEYSFIPAEPMGGRPFGLVINLNYKDLNGNVFQDAVFNQTV
Q9H825	ATFRILEVGCGAGNSVFPILNTLENSPESFLYCCDFASGAV
P01131	ERTALRNGGCQYLCLPAPQINPRSPKFTCACPDGMLLAKDM
P41217	MVTFSENHGVVIQPAYKDKINITQLGLQNSTITFWNITLED
Q8BHX1	LKKAELQLSAEKAKVDSRLQNMDFLKAKAAEFRFGIKAAEE
Q9DBD2	KVYLQLWKRLKAYNRVIYVQNCPETSKKNIFEKSALTEPTL
Q9Y2Q3	FYDVLSPYSWLGFEILCRYQNIWNINLQLRPSLITGIMKDS
Q91ZT9	ACMVSDADCVELLLEKGAEVNALDGYNRTALHYAAERDEAC
P54821	PQQDNDQLNSEEKKKRKQRRNRTTFNSSQLQALERVFERTH
P0DMD0	FIVFMAIANVHFDEYLLVRKNLLISSKSIKPESLDDILGDI
Q9Z1Q5	LDIFAKFSAYIKNSNPALNDNLEKGLLKALKVLDNYLTSPL
P48023	ARSSYLGAVFNLTSADHLYVNVSELSLVNFEESQTFFGLYK
Q3SZ22	RITDADVKNDRSSLHRKLDRNLILLVKDKLGDQDVWMLPQA
P28667	EAAGASPAKANGQENGHVRSNGDLTPKGEGESPPVNGTDEA
Q05195	HGYASMLPYNNKDRDALKRRNKSKKNNSSSRSTHNEMEKNR
Q9CR10	PRGNPTGPPCSLPPELEPPTNCCMSGCPNCVWVDYAEALLR
Q9GZV9	RYLCMDFRGNIFGSHYFDPENCRFQHQTLENGYDVYHSPQY
O43761	QTLLRVASWVFSIAVFGPIVNEGYVNTDSGPELRCVFNGNA
Q9D7W4	RERCGVPFSCCVRDPAEDVLNTQCGYDIRLKLELEQEGSIY
P50226	SPFMRKGMAGDWKTTFTVAQNERFDADYAKKMAGCSLSFRS
Q8VCQ3	EEQATKIADLKRHVEFLVAENERLRKENKQLKAEKARLLKG
O88441	LAVQAFLQMCNLPVKVVCRANAEYMSPSGKVPFIHVGNQVV
Q9ULC3	EALAKRLKLRFYRTSVKEDLNVNEVFKYLAEKYLQKLKQQI
Q8N5I3	QTGVRYVSIKPDNRKLANGTNVLGLLIDTLLKEGFHLVSTR
P97760	DEDESQAPYDPNGKPERFYYNVESCGSLRPETIGLSALSGL
Q2TAM9	DEWDRQNARLRQENARLRLENRRLKRENRSLFRQALRLPGE
Q9D8X5	TNEKGEYPSGINTWQFNFKFNLTEDMYSQDSIDLLANSGLQ
P15946	TPDDLQCVSIKLLPNEVCVKNHNQKVTDVMLCAGEMGGGKD
Q9Y6N3	PYTLQYGQCGDKGQYIHFTPNFLLTNNLATYGPRGKVFVHG
Q96MU5	EKRCHSLKRLRYSVCCKVCPNFCACGKENVSGTGQVCTGVH
O95661	FMEISAKTDVNVQELFHMLLNYKKKPTTGLQEPEKKSQMPN
Q9CXM0	TSGACPDLYQWSDGSSSQFRNWYTDEPSCGSEKCVVMYHQP
Q9BWT6	VEEIRQANKVAKEAANRWTDNIFAIKSWAKRKFGFEENKID
Q7L2Z9	LALLQEEIDKMVETTELMTGNIQSLKNKIQILASEVEEEEE
P09601	FTFPNIASATKFKQLYRSRMNSLEMTPAVRQRVIEEAKTAF
Q8R4V1	QHRPGMENHTRDCMVKLSQANTSATGIYYFIVEGEETYQSD

Q3LUH2	IIQQLNKTFSYFLGLSDPQGNGNWQWIDQTPYKENVRFWHQ
P33784	GLTARPDAFISGAESLLSQENGSQHIAGAWVTSLSDVRVNF
A1YIY0	DIQFWTPVSHPVLTLQHEATNLAVGDKVEFLCEAHQGSLPI
P14439	GALANIAVDKANLDVMKKRSNNTPDANVAPEVTVLSRSPVN
Q8K2Q2	DLKVALRQELCNMEEILEYQNTTFFGGDCISMIDYLVWPWF
Q8VEE0	MHMMVSRPEQWVKPMAVAGANQYTFHLEATENPGALIKDIR
P29965	ASSKTTSVLQWAEKGYYTMSNNLVTLENGKQLTVKRQGLYY
Q3T113	ENYILDTEPGLQESLKCAVQNHIRDEELLWYREDGRVDLKS
Q2KJA5	LCSSYMEVPTYLNFKSMNHMNYLPSQDGMTHNQFIKIMIIF
Q99623	AYGVRESVFTVEGGHRAIFFNRIGGVQQDTILAEGLHFRIP
A2RU48	QLLDCLSDSFDVTNKLTEVLNMHLGCRLASIEMKRDGTIKE
Q8K572	NEICLELMSEHLARAVKNAGNASSLKLQLTNKQRPCLTLVV
Q8IZJ6	NNRITWLFHYSALLSAFGEANVSLARAVNITGLHNILDVAA
Q9DAM2	DLDGEMNIGAANFQNYRFLFNIKKQELRDLFHDFDITGDRL
Q7Z4H3	DLEHKPGRLQDFYDSTAGKFNHPEIVQLVSELEAERSTNIA
Q9CQY8	LLWFFNGTCVSPTDFKNPTINNMVSNWKIPNSNSEEDRHRI
P46777	SVTPDMMEEMYKKAHAAIRENPVYEKKPKKEVKKKRWNRPK
Q01362	LSIISERRNATYLVRGSLGANTASSIAGGTGITILIINLKK
Q5XFR0	FFSRTSKENVEADHRSVFVGNVDYGGSAAELEAYFSPCGEI
Q95IE3	LLVCSVSGFYPGSIEVRWFRNGQEEKTGVVSTGLIHNGDWT
Q3T0P5	LLTLVNRKVSQRRVDFCRDPNAIGKKQVPCFASMLTKKLHF
Q59I47	EPQPSPRSLAEGFLQEELRLNDELRQLQFSELVGIVYNPVE
P05528	NYCLYLVGRRESGFIATFVHNMDGRLLFIRSQLIASLIMSI
Q9UKR0	CATAGTECHVSGWGITNHPRNPFPDLLQCLNLSIVSHATCH
Q3SZX5	GAQIIKEVLLEAQDMAVRDHNVEFRSNLYIAESTSGRGQYL
P97805	NVVGPSMCFEDEIIMSPVRNNVGRGLNVALVNGSTGQVMKK
Q9CWD3	EILECGFRELWEECGLQLPKNQFSCVLLGLWESAYPPRLSW
Q7Z5P4	IYRSLNQVKKEVGDVTIVVNNAGTVYPADLLSTKDEEITKT
Q96GY3	RRKKRREMDDGLAEGGPQRSNTYVIKLFDRSVDLAQFSENT
O43423	DLSTIEPLKQLENLKSLDLFNCEVTNLNDYGENVFKLLLQL
Q9EQ06	ELAALGRTGVRTSCLCPNFINTGFIKNPSTNLGPTLEPEEV
Q9GZT6	MAHLDAIRKDMVILEKSEFANLRAENEKMKIELDQVKQQLM
Q3T110	GICFSGIMMLVVAAVLLVLENDNNYKCCQSENCSKKYMTVL
Q6ZMJ4	GIFLGVALGNEPLEMWPLTQNEECTVTGFLRDKLQYRSRLQ
P19071	FRDHVTDPKEAQRRFNSLKTNILAKRYRAYIRVMEPMKSGR
Q9UFN0	YWSVEFGGRMNTVFHIWKYDNFAHRTEVRKALAKDKEWQEQ
Q9WTY4	IGLSVTLGHLVGIYFTGCSMNPARSFGPAVVMNRFSPSHWV
A5D7P8	KNNSMNNSNNVGTGTFGPMGNGVQTGAEARGCQFSYAGRSN
Q32LN5	QENTGSSGHPTFKCPLCQESNFTRQRLLDHCNSNHLFQIVP
Q58DV7	PSAEQITKYKELVAKTSNLENIKFAWHKETSSEYQNRMMEK
P14437	GGLQNIATGKYTLGILTKRSNSTPATNEAPQATVFPKSPVL

P14191	GNVLNDLTNGGTKLTITVTGNKPILLGRTKEAFATPVTSGV
A6NKC4	LNGTATQTSTPSYRITSASVNDSGEYRCQRGLSGRSDPIQL
Q12829	QAQAYAERLGVTFFEVSPLCNFNITESFTELARIVLLRHGM
Q9D9R0	LPSEGVPATDIAKRMKSEYQNCAECGTLVCLSDMRAHIRTC
P31267	LEKEFHFNRYLTRRRRIEIANALCLTERQIKIWFQNRRMKW
P02702	VAAAWGAQAPRTPRARTDLLNVCMDAKHHKAEPGPEDSLHE
Q9ER00	KLQENLQQLQHSTNQLAKETNELLKELGSLPLPLSASEQRQ
P23184	DESLIDKDKGKEFLITTGVINKTKTSPLWVNSVIISDVPHL
Q9JJ69	FEDFVAGLSVILRGTIDDRLNWAFNLYDLNKDGCITKEEML
Q8TDC0	KVTGTAESGTVANANGPEGPNYRSELHIFPASPGASLGGPE
Q80WR1	REHLTREFFTKELTKHYQGDNDTDVFSATWNSVMITFGCCG
Q9HB31	TFSKGQLLELERAFAAWPYPNISTHEHLAWVTCLPEAKVQV
Q3ZBG6	VFIDCVGVGLLISTLMWFISNKYLVKRQSRDYDVEWGYAFD
Q9P016	SGEALAKVEDSNPQKTSATKNCLKNLSSHWLMKSEPESRLE
P51149	QAFQTIARNALKQETEVELYNEFPEPIKLDKNDRAKASAES
P06804	TLFCLLNFGVIGPQRDEKFPNGLPLISSMAQTLTLRSSSQN
Q9BXJ0	RPLLVLLLLGLAAGSPPLDDNKIPSLCPGHPGLPGTPGHHG
Q02013	VATAILSGITSSLVDNSLGRNDLAHGVNSGQGLGIEIIGTL
Q96DC9	YSVVELVEKDGSVSSLLKVFNDQSASDHIVQFLRLLTSAFI
Q8BH93	VTNTKAGHSSQGWPGSSPWSNPSAPPAMPSGLPPSSAAPST
P61026	YRGAMGIMLVYDITNGKSFENISKWLRNIDEHANEDVERML
Q9D9S2	NISIYLNLIIGLQFSYMISQNKCVHLLVGFLSFFAGCLLFY
Q15125	NAGPLHPYWPQHLRLDNFVPNDRPTWHILAGLFSVTGVLVV
O55125	AHSTLLSKKETSNLYKIQFHNVKPECLDAYNSLTEAVLPKL
Q68D91	KVLPGHFNTFGAERLFRLASNYISKAGICHKVSTFAMRSLA
Q8R2H9	LLQFIAKQGSCFEVILISDANTFGVESALRAAGHHSLFRRI
Q9JL95	VRTPETFDKAQRVCRRCYRGNLASVHSYSFNYQIQNLARKI
Q9Y691	TLLRSYMQSVWTEESQCTLLNASITETFNCSFSCGPDCWKL
Q96MH2	QRASRVREEMFAKGQPVAPYNTTQFLMNDRDPEEPNLDVPH
Q1LZA1	KVGQANPNLQKVLDALKAVKNKNKKAPFTNFDPSVLLPPSL
P02745	AIRRNPPMGGNVVIFDTVITNQEEPYQNHSGRFVCTVPGYY
Q80WR1	AMGGLLFLLGFLGCCGAVRENRCLLLFFFLFILIIFLVELS
P60880	GIIGNLRHMALDMGNEIDTQNRQIDRIMEKADSNKTRIDEA
Q9QWG7	WWEKREEHPLLYLYYEELKQNPKKEIKKIASFLDKTLDEEA
Q9D593	KIQLSTMRNQARITVLRARDNLILELLKDAKMRLSRIVSDE
P05813	IGRQWEISDDYPSLQAMGWFNNEVGSMKIQSGAWVCYQYPG
P00766	FDQGSSSEKIQKLKIAKVFKNSKYNSLTINNDITLLKLSTA
P43307	EMGTSSQNDVDMSWIPQETLNQINKASPRRLPRKRAQKRSV
Q9CQ07	RKIPDSIAKFQNLRWLDLHSNYIDKLPESIGQMTSLLFLNV
Q6NUI1	SIQQLVPEYKEKQTPESLPQNNNPAAPSQAEGGEGGVACGT
Q3UWA4	LCEECLQSPEHQSHTELSIENAISHYKERLNRRSRKLRKDL

P56746	TNTIFENLWFSCATDSLGVYNCWEFPSMLALSGYIQACRAL
Q3T0P5	ERFFWHLTLPNRPGTSADRDNLRRRFSDLGFEVKCFDDLRA
Q96LD1	LLLVTMIVNLAMTIWILKVMNFTVDGMGNLRVTKKGIRLEG
O70514	PHSTAEEGVEGSAPSLGKAQNKQRSRTSKSLTHGKFVTKDQ
Q8K201	VLALLLVPVLCSDRSENPPNNATVSSPVVVTAPGNHTSPSV
P23025	CGKEFMDSYLMNHFDLPTCDNCRDADDKHKLITKTEAKQEY
Q9UNT1	GTNVVKLFNDAIRLAVSYKQNSQDFMDEIFQELENFSLEQE
Q3KNW1	KTHSSHRVPNYRRLETQREINGACSACGGLVVPLLPRDKEA
A5D9C6	DFSTARSHEFSREPSLSWVVNAVNCSLFSAVREDFKALKPQ
Q8N9N7	TVLPDEICNLKKLETLSLNNNHLRELPSTFGQLSALKTLSL
Q4JM65	ATLGTAGENYNVIQQTVKYFNSQQQITDLFPNYPLNIQPED
Q5E9V3	FKVPDFDLLLLTDPRFMAFANPLSGRRSFNRTPKGWISENI
Q925U0	GWTEDMRARRDEQTVPMVQPNLSTSSEDHHVSTEPWASETS
A2A8T7	FLGLCSVLIGSCILFLHWKKNLQREERAQQWVEVMRAATFT
P16110	DVAFHFNPRFNENNRRVIVCNTKQDNNWGKEERQSAFPFES
P28585	SESEPNLLNQRVEIKKSDLVNYNPIAEKHVDGTMSLAELSA
Q61142	TEDGSKDEWRGMVLARAPVMNTWFYITYEKDPVLYMYQLLD
Q9CQF3	YIQQTKPLTLERTINLYPLTNYTFGTKEPLYEKDSSVAARF
Q15006	GGLENLELSRKYFAQALKLNNRNMRALFGLYMSASHIASNP
O95858	LIGGVVALTFRNQTIDFLNDNIRRGIENYYDDLDFKNIMDF
P10746	AMESITVYQTVAHPGIQGNLNSYYSQQGVPASITFFSPSGL
Q0VD07	LYAGVALFCGCGHEALSGTVNILQTYFEMARTAGDTLDVFT
Q9R0H2	INSLTTPKHEVGTTTEGPLRNESSTMKITVPNTPTSNANST
Q91VX5	VDHGNGTFSVHFRHNATGQGNISISLVPPSKAVEFHQEQQI
P35282	VVLLGEGCVGKTSLVLRYCENKFNDKHITTLQASFLTKKLN
P50195	SSHEEWRNSELARQQQKTIQNHVGTFHQKILGHVEGWRDMG
P30044	APIKVGDAIPAVEVFEGEPGNKVNLAELFKGKKGVLFGVPG
P10628	PGTALKQPAVVYPWMKKVHVNSVNPNYTGGEPKRSRTAYTR
Q9DCK3	QQDLKKGLHLYGTQGNVGLTNAWSIIQTDFRCCGVSNYTDW
P09630	RTAPQDQKASIQIYPWMQRMNSHSGVGYGADRRRGRQIYSR
Q3MHP2	RIVSQKQIADRAAHDESPGNNVVDISVPPTTDGQKPNKLQC
Q08DT6	QTLASTGCRFSLSLLTKNTPNVTSFHQCRLLQTTLSRRGLE
Q3ZC52	VPNKSLTYGVILTHGASGDMNLPHLTSLASHLASHGFFCLR
P50518	IEQEANEKAEEIDAKAEEEFNIEKGRLVQTQRLKIMEYYEK
Q61166	RMGVDKIIPVDKLVKGKFQDNFEFVQWFKKFFDANYDGKEY
Q80W21	DGSHKITQSNAILRYLGRKHNLCGETEEERIRVDILENQLM
P53811	CAYKLVTIKFKWWGLQSKVENFIQKQEKRIFTNLHRQLFCW
Q719H9	DKSLIEEVFPEIGDVMCNSVNAGWNHDSTHVIRFPLNGYCH
Q3SZ22	TAERTLATLSENNLEAKFLGNAPCGHYKFKFPQAVRAEGSL
A5D989	SSRQENGASVILRDIARARENIQKSLAGSSGPGASSGPSGD
Q58DS5	LLIGDSGVGKTCLIIRFAEDNFNNTYISTIGIDFRIRTVDI

Q2KJD9	YFRAWLGPRDTRVEGWFLLDNYVPTLVCSILYLLIVWLGPK
O95976	ARRIFQEIAQELYHKRHVETNQQSEKDNNTYENRRVLSNYE
Q28151	HTGEKPFECKDCGKAFIQKSNLIRHQRTHTGEKPFICKECG
Q49BZ4	VMLVITVVLSTSGIWRSSSGNNLLKSDSFPSRNKDNQSQPT
Q99L85	CVTLCQALHALSQQNGDPGDNSLVEQVRVLGLLHEELHGPG
Q9GZZ7	RLRGPRGVPAGTAVTPNYVDNVSARVAPWCDCGASGNRRED
Q9CX63	PANFSLPLSQTTGWFQCEAENDVGVDSSARIPLPRAEARAK
Q9DBZ5	ATLERYVETQAKENAYDLEANLAVLKLYQFNPAFFQTTVTA
Q3ZBM4	LALKTTDANNQEYSLWVYQCNSLEQAQAICKVLSTAFDSVL
Q9BUN8	PITATFYFPVGPGTGFLYLVNLYFLYQYSTRLETGAFDGRP
P15483	DINSEGQIEWGIRDGNLVAKNKTPYYFTIVNASFNGKALKT
Q9D4G1	LLLLQALGIVALGHFTKAQNNTLIFTKGNTIRNCSCPVDIR
P97447	TREDSPRCKGCFKAIVAGDQNVEYKGTVWHKDCFTCSNCKQ
Q9WV55	SDLKFKGPFTDVVTTNLKLQNPSDRKVCFKVKTTAPRRYCV
Q1LZ75	PGGAEATRPVGPEPLAEETPNQAPKPGAKSNSIIVSPRQRG
Q92813	AEGATCHLLDFASPERPLVVNFGSATUPPFTSQLPAFRKLV
Q01151	RMETPQEDHLRGQHYHQKGQNGSFDAPNERPYSLKIRNTTS
Q3T033	YVKPQSCIDIVIRHVAPHPRNYDVQDRFRIELSEEGTEGRV
A8C927	DRVHIDLGTGDGRNIYKLAINDQNTFYIGIDPVKENLFDIS
Q9BY71	IGSATFAGLAGGLRLLDLSYNRIQRIPKDALGKLSAKIRLS
Q9NRD1	FQHGGRDTQYWAGWYGPRVTNSSIVVSPKMTRNQASSEAQP
Q9NYV7	CSYFNLNYVLCNLTITWEFFNILTFWLNSLLTVFYCIKVSS
Q9ULC3	LAKRLKLRFYRTSVKEDLNVNEVFKYLAEKYLQKLKQQIAE
Q3T0P7	VIPLNTSIVMPPKNLLELLINIKAGTYLPQSYLIHEHMVIT
A5D7P8	AEARGCQFSYAGRSNGRGPINPQLPGTANNQTVMTTISNGR
Q8N0Y7	WRRSYDVPPPPMEPDHPFYSNISKDRRYADLTEDQLPSYES
Q8NF67	SLKKLTMLESPLEGISHYHINLDETQVPKKKLFQVESQFDD
P70452	AQLKAIEPQKEEADENYNSVNTRMKKTQHGVLSQQFVELIN
Q5VZ72	AVQQVVKLRTWLKNEFYKLGNETWKGVFIYQGKLLDVCQNL
Q91VF2	KYECYDLVSTMDITDCFIDGNENGDLLWDFLTETCNFSKTA
Q9DB29	DSSLKDENPKQHVPLDEYSANLRDMVQYLRSVDVPRERVIL
Q9UKL6	SLAIESDGKKGSKVFMYYFDNPGGQIPSWLINWAAKNGVPN
Q9NPC3	GTLEPSMIAQSGVLGFPLGNNSKFPLDNTPVRNRGDGDGDF
Q0IIN9	PIAVELEFENQPLGAETRLRNGRRAGVKRSEGRGQVRPGQV
P11032	DEYTREGDLQLVRLKKKATVNRNVAILHLPKKGDDVKPGTR
P40306	TALGSGQDAALAVLEDRFQPNMTLEAAQGLLVEAVTAGILG
Q3UX61	HRRLGLAQKLMDQASRAMIENFGAKYVSLHVRKSNRAALHL
P51749	VLQWAPKGYYTLSNNLVTLENGKQLAVKRQGFYYIYTQVTF
P35846	PEDNLHDQCSPWKTNSCCSTNTSQEAHKDISYLYRFNWNHC
O60248	FMVWSSAQRRQMAQQNPKMHNSEISKRLGAQWKLLDEDEKR
Q8VHP9	CPSPLSGITPLLYVAQTRQSNILKILLQYGILEREKNPINI

O88346	QLESEKFDLMEKLKQQKYEINVLYNRISHAQKFRKGAGKGR
Q8CDN6	DQYQGADAVGLEEKIKQHLENDPGSNEDADIPKGYMDLMPF
Q8BL86	RQFRVQAVQPTLILQDGDVINLGDRQLTVMHMPGHSRGSIC
P03038	TADAQLRFLCEAGFSAGDAVNALMTISYFTVGAVLEEQAGD
Q9Y5X0	CVSGQTKYSVEEAIHKFALMNRRFPEEDEEGKKENDIDYDS
P10738	YRQLFTKNQFHQAMKHAKVNNLSTVTYEQVLSIFNSYLLFN
O00233	SPASIAGLQVDDEIVEFGSVNTQNFQSLHNIGSVVQHSEGK
O35566	GVVVMVTGVLGCCATFKERRNLLRLYFILLLIIFLLEIIAG
O70578	AIFSLGFIIVGSICAFLSFGNKRDYLLRPASMFYAFAGLCL
Q3SZ19	IEAQIKANYEVLESQKGIGMNEPLVDCEGYPRADVDLYQVR
Q58DS3	TRMQLQGELRAPGTYQRHYRNVFHAFITIGKVDGLAALQRG
P61981	TELNEPLSNEERNLLSVAYKNVVGARRSSWRVISSIEQKTS
Q15072	HTGEKLFECNECGKSFSQKENLLTHQKIHTGEKPFECKDCG
Q9CQJ0	QEFLEKTKSGGWIKLPSFKSNRDHIQGLKLPFGLETASDKQ
P46926	KKLIEYYKNGDLSFKYVKTFNMDEYVGLPRDHPESYHSFMW
P47755	CFKFDHLRKEATDPRPCEVENAVESWRTSVETALRAYVKEH
Q8VDV8	KYLDQEKEDGKYHKQIKIEENATGFSYESLFREYLHETVTE
Q9Z1P5	ALPCSCDNISGCSDVSDKNLNCSRPPCQESELHCILDDVCI
Q9DBZ5	ITYQHIDRWLLAEMLGDLTDNQLKVWMSKYGWSADESGQVF
P31275	VSPLNPGGGLSASGAPWYPINSRSRKKRKPYSKLQLAELEG
O54831	LSINGLFHNAMRLTWNIKNLNMELRKTYTVNQVSEKLYENY
Q8SPU5	APSGLLGSLLGKVTSLTPLLNNIIELKITNPQLLELGLVQS
Q6XJV6	HKVYDLQVLVPPEVTYFLGENRTAVCEAMAGKPAAQISWTP
P09211	DMVNDGVEDLRCKYISLIYTNYEAGKDDYVKALPGQLKPFE
Q8VC33	RNNSDQDEVETEAELSRRLENRLVLLFFGAGACPQCQAFAP
A0PK84	GALFLFLSANALGNYVLVIQNSPDDLGTCQGTMSQRPQCPP
P61006	EKLALDYGIKFMETSAKANINVENAFFTLARDIKAKMDKKL
Q03255	RPMNAFIVWSRERRRKVALENPKMKNSDISKQLGYEWKRLT
P47754	EDGNVQLVSHKDIQDSLTVSNEVQTAKEFIKIVEAAENEYQ
Q96DR4	LENFEENCCVMRYTTAGQLWNIISPREFVDFSYTVGYKEGL
P56750	GIFVLIPVSWTANIIIRDFYNPAIHIGQKRELGAALFLGWA
Q6UXN2	TIWDKPNAGFFNITMIQLTQNDSGFYWCGIYNASENIITVL
P21107	RAELAESKCSELEEELKNVTNNLKSLEAQAEKYSQKEDKYE
P33792	NSKEREYIYVTLSELISEKNNRRDEIFYNADNVPLWPISAE
P20934	SPTAEIKSQGETFKKEVCEENTSNTAMLICLIVIAVLFLIC
Q8BGT7	QRIKELEQEREDQKVKWQQFNNRAYSKNKKGQVKRSIFASP
O70439	GTPQDSPELRQLLQQKQQYTNQLAKETDKYIKEFGSLPTTP
Q61199	VVEFEISPQSTLETKESKSFNCHIEYEKTDRAKKTALCNFD
Q0IIG7	AQAAIVVYDITNEESFARAKNWVKELQRQASPNIVIALSGN
Q96PP4	NGKSKTSENSSAKREKGMVVNSKEISDAVGQSKFVLENLRH
P47758	GQYRDTQTSITDSSAIYKVNNNRGNSLTLIDLPGHESLRFQ

P07738	RLNERHYGALIGLNREQMALNHGEEQVRLWRRSYNVTPPPI
Q1W209	QPLSPFNLGATLQSLPSLNFNSFHSLVETKETCFIREPKTP
P52946	RTRTAYTRAQLLELEKEFLFNKYISRPRRVELAVMLNLTER
A6NLU5	VSHIQSSGPRRHGPASAANANNAGAASRTTSEPGRGDKSPP
Q9D6Y1	RSLRQARKNSRHLELVNQKLNEKLGASSAQQHINALGREPV
P70195	ATEGMVVADKNCSKIHFISPNIYCCGAGTAADTDMTTQLIS
Q9UBV8	GPAPGGPYGPPAGGGPYGHPNPGMFPSGTPGGPYGGAAPGG
Q8C767	LALTMVKVFSEFDDPLDIPFNITELLDNIVSLTTAESESFV
Q8TBG9	CGGYSGGLRLSVDCVNKTESNLSIDIAFAYPFRLHQVTFEV
Q4JM65	HQGCLVNSPGNLPMWGNQTWNNPTWSNQSWNSQSWSNHSWN
Q29S19	EKTAAEEKPRVLEPGAAPFGNFPHYSRFHPPEQRLRLLPPE
Q13261	TTEISSHESSHGTPSQTTAKNWELTASASHQPPGVYPQGHS
P28161	RKHNLCGESEKEQIREDILENQFMDSRMQLAKLCYDPDFEK
P52945	RVELAVMLNLTERHIKIWFQNRRMKWKKEEDKKRGGGTAVG
B1ATL7	NVSWEVSGGPPALIVGGTKVNNGGTERGSNNARLHVALPQG
P35219	SLLDVRLSPNYVVCRDCEVTNDGHTIQVILKSKSVLSGGPL
Q9D1G5	GALPPQLCCLRHLDVVDLSKNQIRSIPDTVGELQAIELNLN
Q0P5A4	EQKAKALKGQYNFDHPDAFDNDLMHRTLKNIVEGKTVEVPT
Q8BVN8	DMERKITELETEKRDLERQVNEQKAKCEATEKRESERRQVE
Q3V009	ARDRNLQEDNLERVNFWSAANVAVLLLVAVLQVCTLKRFFH
P20231	ASETFPPGMPCWVTGWGDVDNDERLPPPFPLKQVKVPIMEN
Q8R2R5	LTHLGPLASLRQLAVLNVSNNRLTGLEPLAACENLQSLNAA
B6A8R8	GHYTCEYYSKWPHDTPSHPSNALFLLVTGYLPQPSFQAHHR
Q8NHM4	GFLEGGKDSCQGDSGGPVVCNGQLQGIVSWGYGCAQKRRPG
Q30167	RWFRNGQEEKTGVVSTGLIQNGDWTFQTLVMLETVPQSGEV
Q6GPI1	DFPAGTLCATTGWGKTKYNANKTPDKLQQAALPLLSNAECK
Q9D780	NLSLEIPSNEEIKHIDWLFQNNIAIVKPGKKGQPAVIMAVD
Q8N131	PHNSSANSTETLQHVPSDHTNETSNSTVKPPTSVASDSSNT
O88551	ATAWYGNRIVQEFYDPLTPINARYEFGQALFTGWAAASLCL
P19217	EVMNQKVSPFMRKGDVGDWKNHFTVALNEKFDMHYEQQMKG
Q8TD07	ATNGEKSLLFDAMNMTWTVINHEASKIKETWKKDRGLEKYF
Q58DI5	GAGSSQALPPQLPEEDRIDLNPSFLGIALRSLLAIDLWLSK
Q60809	VKWLSFHSGYDFGYLIKILTNSNLPEEELDFFEILRLFFPV
Q9CQU5	EFMRNSRKKDKLLCSQLQVVNFLQTFLAQEDTEQSPDALAS
Q6P073	RFKCNTRLCLSITDFHPDTWNPAWSVSTILTGLLSFMVEKG
Q3T0A6	LRIFKWVPVTDSKEKEKSKSNSSAAREPNGFPSDASANSSL
Q8CFP6	REIKVNIFDMAGHPFFFEVRNEFYKDTQGVILVYDVGQKDS
Q99714	QTHTLEDFQRVLDVNLMGTFNVIRLVAGEMGQNEPDQGGQR
Q96IL0	WIGPPDKYSNLRPVHFYIPENESPLEQKLRKLRQETQEWNQ
Q91VK4	VIELNTTIVLPPRNFWELLMNVKRGTYLPQTYIIQEEMVVT
Q99KK1	IVQAKERGYETMVNFGRQGLNLAAAAAVTAAVKSQGAITER

Q8BJN4	YYHIMYRPNWNSIFSGYLRYNFHHEEKVPRTITSVALEHLA
P41238	YEIKWGMSRKIWRSSGKNTTNHVEVNFIKKFTSERDFHPSM
Q3SZ18	RNSDKSIPMTVDFIRLKSYCNDQSTGDIKVIGGDDLSTLTG
Q9NX07	PVEYSQMYSYSYNQYYQQYQNYYAQWGYDQNTGSYSYSYPQ
Q4VAE3	QEKLEALPPTPGQLRYVFFHNAIPFVGFGFLDNAIMIVAGT
Q86SE9	DDKDYHRSDPQIAICLDCLRNNGQSGDNVVKGLMKKFIRCS
Q9JKF4	SLTCFSEGTMVSEKMWGCCPNHWKSFGSSCYLISTKENFWS
Q9D6I9	MELAYLRAIDVKILQQLVTLNEGIEAVRWLLEERGTLTSHC
Q9NVK5	LEQENKGLREILQITRESFLNLRKDDASESTSLSALVTNSD
P17981	RFRYTGKGSVRFGHFASSSLNRSVATSSPFFNGQGTLFIIK
Q2LGB7	IIFAEMPCGRQHLQNLDDAVNGSAWTILLLTENFLRDTWCK
Q9DB76	NQVDVWGAQAGLVVAGYYHANAVLDDQSPGPLALKIAGRIA
P52907	APPGEFNEVFNDVRLLLNNDNLLREGAAHAFAQYNMDQFTP
Q5UCC4	VTHPGGCRGHEVEDVDLELFNTSVQLQPPTTAPGPETAAFI
Q16635	CHLNPIILPLWHVGMNDVLPNSPPYFPRFGQKITVLIGKPF
Q8WVC6	PGYPAHRRIVEVFGTEVLLENGDINRKVLGDLIFNQPDRRQ
Q6DKI1	ELILKRGQAKVKNKTIPLTDNTVIEEHLGKFGVICLEDLIH
Q9H213	FQGTKKSLLMSILALIFIMGNSAKEALVWKVLGKLGMQPGR
P12319	TKWFHNGSLSEETNSSLNIVNAKFEDSGEYKCQHQQVNESE
P02970	GNVLNDLTNGGTKLTITVTGNKPILLGRTKEAFATPVSGGV
Q32L19	EVLQSLVDDGMVDCERIGTSNYYWAFPSKALHARKRKLEVL
Q9R9D6	RLRDVGVDTMYGVAFGEKGMNPLTRTSFIITEDLTPTISLE
P09601	PQDLSEALKEATKEVHTQAENAEFMRNFQKGQVTRDGFKLV
P51959	GRDLTFWQELVSKCLTEYSSNKCSKPNVQKLKWIVSGRTAR
Q5E9K2	QVSRATQGEQDNYEMHVRAANIVRAGESLMKLVSDLKQFLI
Q2TBT8	PLAHRKGHQDSCPFELMVCPNEGCMLRVPRGALDEHRQNCQ
Q8BHI7	IQTYNKKGASRRKDHLKGHQNGSVAAVNGHTNSFPSLENSV
Q9JJ69	FFPQGDSSNYATFLFNAFDTNHDGSVSFEDFVAGLSVILRG
P43431	DITRDQTSTLKTCLPLELHKNESCLATRETSSTTRGSCLPP
P04230	NSQPEFLEQKRAEVDTVCRHNYEISDKFLVRRRVEPTVTVY
P02663	EHVSSSEESIISQETYKQEKNMAINPSKENLCSTFCKEVVR
Q96DR5	LVLLCGVLTGTSESLLDNLGNDLSNVVDKLEPVLHEGLETV
Q32KW7	AAARDGTLQPGDVLISVGYANVLGYTLREFLKLLQHITIGT
Q17QF8	VGIPGWGVCGWIATISFFGTNIGSAVVMLIPTVLFTVMAVF
Q8IZS5	INPRQCATEVSREDDDRIFYNRLTKLFDESRQGEPQDESGR
Q3KRB8	ILLLSCLLADHTVHVLRYFFNFLRNVSLRSSENKMDSSNLA
Q3ZBW4	EAVAIEMNEPVQLTFALRYLNFFTKATPLSPTVTLSMSADV
Q8NHG8	NGRTRAYSGSDLPSSSSGGANGTAGGGGGARAAAAGRFPAQ
Q9QWG7	ARNGKDVAVSYYHFDLMNSINPLPGTWEEYLEKFLAGNVAY
Q8R0K4	DVAYVQRMEQEAQHVLFLSKNQAPRQPEVQAAPKKEKSERK
Q0V7M7	EADIKEFTTLKVDKRFHGILNILRHCRRLSEVRGKGLTRYV

P19388	HVVMTKEEVTELLARYKLRENQLPRIQAGDPVARYFGIKRG
Q9D287	IENLELMSQHGCNAWKVYNENLVHMIEHAQKELQKLRKHIQ
Q8BZ09	TLGRHGIFNMVYFGFYHNVKNIIPSSKDPTLEFLRKFGIGF
Q2KI22	WMLEVCEEQKCEEEVFPLAMNYLDRFLSLEPVKKSRLQLLG
Q1LZE0	QPPSKDTEEMEAEGDSAAEMNGEEEESEEERSGSQTESEEE
P08057	EERYDVEAKVTKNITEIADLNQKIFDLRGKFKRPTLRRVRI
Q9D0U6	LDSDPFGEDGSLWSFNYFFYNKRLKRIVFFSCRSISGSTYT
Q9BUT1	GQAAALAFAREGAKVIATDINESKLQELEKYPGIQTRVLDV
Q8K1A0	RPHIAACMLYTIHNTYDDIENKAVADLGCGCGVLSIGAAML
Q9D8T0	DPATKMNEETRKLFSELGSRNAKDLAFRDSWVFVGAKGVQN
Q61759	TPTKWQIPNDLQCGFIKPLPNENCAKAYIHKVTDVMLCAGE
Q6UX52	ITCCAPQPPPPITYSLCGTKNIKVAKKVVKTHEPASFNLNV
Q3UG98	MSYGVTKLGLTKFEAKIGQENEPSIRMFQKLHFKQVAMSNV
Q8BVN0	LKRLHCQMNKIQLNRRKWQWNIQQLEKTAAELKKRIREKEA
O43688	AAVSQSLTDLAKYMIGRLRPNFLAVCDPDWSRVNCSVYVQL
P04156	GWGQPHGGGWGQGGGTHSQWNKPSKPKTNMKHMAGAAAAGA
Q8K0Z7	FFDKKGVVVVGVEDREKKAVNLERALELAIEAGAEDVKEAE
Q9QXF8	KERWNRRKEPSFDNWVIEEANWLTLDKDVLSGDGFDAVICL
Q12918	LIRDKAILFWIGLNFSLSEKNWKWINGSFLNSNDLEIRGDA
Q9BYD2	LKLPEEPITRWGEYWCEVTVNGLDTVRVPMSVVNFEKPKTK
Q3T0P5	TASHVDADCFLCVFLSHGEGNHIYAYDAKIEIQTLTGLFKG
Q68FF9	GFALWLVGMVINIHSDHILRNLRKPGETGYKIPRGGLFEYV
Q32LN0	NVIVKTEQTDPSIMNTWKEENYLYDTNYGSTVDLLDSKTFC
Q8BFR6	ALPLDHTLERWITKEECPLYNGGNVILEYLNDEEQFLKNVD
P97447	NTCVDCRKPISADAKEVHYKNRYWHDNCFRCAKCLHPLASE
Q9Z2C6	NNGVTKTWDRLMLQDHCCGVNGPSDWQKYTSAFRVENNDAD
P28663	KLHMQLQSKHDSATSFVDAGNAYKKADPQEAINCLNAAIDI
P15483	SINTCIKLILRPHNLPKLDINSEGQIEWGIRDGNLVAKNKT
Q03084	GNEYLYEDYALWLKSLANGCNGDNIQQVLVDMRFSKETAKR
P0C0A3	DELLAGNFTQEDEDAILEELNAITQEQMELPEVPSEPLPDR
O95452	MHVAYYRHETTRKFRRGEKRNDFKDIEDIKKQKVRIEGSLW
Q9NYP9	CVSCNVSVDKEQKLSKREKENGCVLETLCCAGCSLNLGYVY
Q8NCL8	IPLLLMTPVFCLGNTSECFQNFSQSHKCILMHSPPSAMAEL
P82923	PLKHSDFFNVKELFSVRSLFNARVHLGHKAGCRHRFMEPYI
Q9D061	VASREQLLYLYARFKQVKVGNCNTPKPNFFDFEGKQKWEAW
Q53HI1	VLIDCVGVGLLIATLMWFISNKYLVKRQSRDYDVEWGYAFD
Q9Y6Z7	VGRMGPKGIKGELGDMGDQGNIGKTGPIGKKGDKGEKGLLG
Q2T9L9	ADKQHVLDMLFSAFEKHQYYNLKDLVDITKQPVSYLKDILK
Q645M6	AGGAAPEDRDLRAAMEIICDNVGKDWRRLARHLGVSDVKIE
Q9BS40	EIPPTNYPASRAALVAQNYINYQQGTPHRVFEVQKVKQASM
Q6IR41	CQTHYSAFSVGRKTGLHSSENFLSLLFDRVFVNTDGHFDMA

P10767	GIGFHLQVLPDGRISGTHEENPYSLLEISTVERGVVSLFGV
P00642	ISITRPDGRVVNLEYNSGILNRLDRLTAANYGMPINSNLCI
Q8BTG6	DRNLLQVQDREQPIPWKVQFNLGNSSRPSNQCRNSVQGKHL
O43760	LWTFLWFVGFCFLTNQWAVTNPKDVLVGADSVRAAITFSFF
P62827	TLGVEVHPLVFHTNRGPIKFNVWDTAGQEKFGGLRDGYYIQ
O35417	GLSSSFGNGKESELAGADRMNDEAAQGRTVHFNEEDLRKQA
Q8BHC4	PGYPAHRRIVEAFGTEVLLENGDIDRKVLGDLIFNQPDRRQ
Q6Q6R3	YGYLFGPKGGQSDPRHWTYENVWTTSLVVRHLDPSPIPSDL
P20783	KHWNSQCKTSQTYVRALTSENNKLVGWRWIRIDTSCVCALS
Q3SX42	TRTFAVSSKVTSMSTQTKVMNSQMKMAGAMSTTAKTMQAVN
Q5VVY1	PVFNSVELVDMMESFLLEAQNYLQVKGDKVESYHCYSLQEF
Q9DCA7	LISPLDVSQLQPSFSDQVVINTQTEYQLTSADQPKKFADLE
Q504P2	KCKPCPKGSEWYKDSCYSQLNQYGTWQESVMACSARNASLL
Q56K12	SNNKTRIFNVQSRLTEAKHINWRAVLSNSCLYVEIPGGALP
P22615	LNISSLPWVSFDGFNLNITGNDDYFAPVFTMAKFQQEGDRV
P41217	LTVYVQPIVSLHYKFSEDHLNITCSATARPAPMVFWKVPRS
Q96S79	PIIIVGNKRDLQRGRVIPRWNVSHLVRKTWKCGYVECSAKY
Q80ZI2	GTSRLDESSMLEENIEGLVDNLITSLREQCYDEDGGQYRIH
Q9BTT0	ESIFELLQQITYLDGFDQEDNEAPDSEEEDDEDGDEDDEEE
Q96GY3	SHMDRERLDEEAGKTPSDTHNKDCSIAATGKRPSARFPHQR
Q9TS87	RGRLGFQVWLKNGVILSKLVNSLYPDGSKPVKVPENPPSMV
Q2KHU0	ASKLNIPSTNVFANRLKFYFNGEYAGFDETQPTAESGGKGK
P62593	VLSRVDAGQEQLGRRIHYSQNDLVEYSPVTEKHLTDGMTVR
Q9BYJ0	AKPTTRPTAKPTQPGPRPGGNEEAKKKAWEHCWKPFQALCA
Q9BWP8	FVYSDHSPMRTFNKWRSGEPNNAYDEEDCVEMVASGGWNDV
Q8QZY4	CHSVLGHVLRVAAPGSSHCLNVSISSPGARRRPRAWISNVV
Q9D7G9	GPYPGPAVRVPGPTRSYVSTNVPFPELPRPNSAPTDPVGPL
Q3TQR0	SPCPGYRLLCRINFSLNVVENLALLVLTYVSSSEDFTIHEN
P81125	GLFGGSSKIEEACEIYARAANMFKMAKNWSAAGSAFCQAAH
Q8BHE1	QTERHREERRRQQQLDAERLNDYVNADHGLYFNHRRSLEPP
Q9R088	KVIPMEYLHAIHRLYEEWLVNGSLFPAAAPVLVIEADHNLE
Q14390	TAHLSVVAEDGSAVSATSTINLYFGSKVRSPVSEILFNDEM
Q99KR7	KGSTFHRVIPAFMCQAGDFTNHNGTGGRSIYGSRFPDENFT
Q9UQN3	NSQMKMAGAMSTTAKTMQAVNKKMDPQKTLQTMQNFQKENM
Q5E9S8	IKHAIGHEHEVLLRDLLLEKNLSFLDEDQLRARGYDKTPDF
Q9Y6G5	ALQQQLENIHLRQDKAEAFVNTWSSMGQETVEKFRQRILAP
Q5JQC4	GDQEAPVSQEGAQAEAAGAGNQEGGDSGPDSSDVVPAAEVV
O14653	RTFTTNDSDTTIPMDESLQFNSSLQKVHNGMDDLILDGHNI
Q8WVN6	KGARDSHAGLYMWHLVGHQRNNRQVTLEVSGAEPQSAPDTG
F2Z333	APGCVLHAPAGRSLRASVLRNRSVLLQWRLAPAAARRVRAF
Q7Z7D3	EQDEMFRGRTAVFADQVIVGNASLRLKNVQLTDAGTYKCYI

Q9QYI7	AGGGANVPHSSPFGAGYPFRNPEDIFREFFGGLDPFSFEFW
Q496A3	EKVPEKRDSGMTEVERTYSANCSDFLESKGCFANTTPSGKS
Q17QN3	DKHMNLILCDCDEFRKIKPKNAKQPEREEKRVLGLVLLRGE
Q9NV23	VKQCSPIIRADLNIVRSCTSNVPSKAVLSCDLTCFVGSEDI
O60812	MIASQVVDINLAAEPKVNRGNAGVKRSAAEMYGSSFDLDYG
Q8VBT3	DQLPRPSLVALPGPVVAPGANVSLRCAGRIPGMSFALYRVG
P52843	TNLVKNPGSLGTYFEWFLKGNVLFGSWFEHVRGWLSMREWD
Q2TUM3	QAGVRYISIKPDNRKLANGTNVLGLLVDTLLKEGFHLVSTR
B5MD39	STINLYFGSKVCSPVSGILFNNEWTTSALPAFTNEFGAPPS
Q92530	LNLDDYIDAEHLGDFHRTYKNSEELRSRIVSGIITPIHEQW
Q2KJF9	SSSKKKKAKLEHGGSSGSKQNSDHSNGSFNLKALSGSSGYK
P55253	MGRGYAWLDTGTHQSLIEASNFIATIEERQGLKVSCPEEIA
Q9P0G3	WGTISSPIARYPASLQCVNINISPDEVCQKAYPRTITPGMV
Q93LM8	HLADGMTVGELCAAAITMSDNSAANLLLATVGGPAGLTAFL
D3W0D1	TQLQAHLLVIQNLDELEFIQNSLKPGHFGWIGLYVTFQGNL
Q9CQD7	GKDACVVLVSESSTKGRKSVNTFKACMKYKDCYSGFVSTTM
Q3UTA9	PEVLSKLHPLQYRWVLWFFKNDRSRAWQDNLQLVTKFNTVE
P22692	ASQSRTHEDLYIIPIPNCDRNGNFHPKQCHPALDGQRGKCW
P54349	FQAMNAKLLMDPKRQIFLDQNMLAAIAELMQALNFDSETVP
P32043	ANSFYKQSPNIPAYNMQTCGNYGSASEVQASRYCYGGLDLS
Q2T9Y6	DSRAAGALLARASTLHLQTGNLLNWGRLRKKCPSTHSEELR
Q9CPV4	SQSDPVLKVTLAVSDLQKSLNYWSNLLGMKIYEQDEEKQRA
Q6QHK4	LLEGAKDSKKQDPDEQSYSNNSSESHTSSARQLSRNITQHI
P06880	DTYKEFERAYIPEGQRYSIQNAQAAFCFSETIPAPTGKEEA
Q4G0X4	FTVREAPQIYSLSSSSMEVFNANIFSTSCLFLKLLGSKLFY
A2VDP0	KTENGDNDRLRPQPQASATSNTFRKSSDSSSSVSPLVLSSN
P0DMR1	DDSVEEGDLLCDDDNEDQGDNQLELIKDDEKGAEEGEDDRD
Q96MI6	ELQEDDVVVMATDGLWDVLSNEQVAWLVRSFLPGNQEDPHR
Q6DIA9	DAHQNVIDKFSAVPDPIEQWNNDIYLQVTHVFSGIRRGIRF
Q91VR2	VIALELLNSGYEFDEGSIIFNQFKSVISYKTEEKPIFSLNT
O95926	ARKLNHQEVVEEDKRLKLPANWEAKKARLEWELKEEEKKKE
Q9D5K1	LFRPFRANSPFIGFKPAYEYNAGTYRCDVQLLKNLKFVKRL
Q3T0S3	SPYVRKNGTFPPYCCYDSVNNSFMEPCNNFTAHNMSVQGCF
P07766	KAKAKPVTRGAGAGGRQRGQNKERPPPVPNPDYEPIRKGQR
Q8WUE5	FVPYKGDLLEVEYSTEPGISNIKATSVKPIRCIHTEEVCIT
Q8TAX0	NYSFLQAVNGLPTVPSDHLPNLYGFSALHAVHLHQWTLGYP
P58418	SEKYTTSFWVIFFCFFVHFLNGLLIRLAGFQFPFAKSKDAE
Q8K305	QGEGFSQVLKMRPVIQLQRINQEVFSSLYRKADFKPDTCVT
Q9KIP8	KYLLQDAQANGYAVPAFNIHNAETIQAILEVCSEMRSPVIL
Q9ULC0	DVRKNDSIISNVTVTSVTLPNAVSTLQSSKPKTETQSSIKT
P56177	SKFKKLMKQGGAALEGSALANGRALSAGSPPVPPGWNPNSS

Q149M0	QLSLTFSFFWLGLSWNSSGRNWLWEDGSFPPPTLFSDKELA
P47754	TVSNEVQTAKEFIKIVEAAENEYQTAISENYQTMSDTTFKA
Q3ZCC4	PSTQTSEAGSTGEEQKEEESNGFNKDLLDSPHNAGAASTVN
H3BS89	RDRCKAFNTRRVDPGFIYNNNNNLPLRASRSRLDRWEGKLL
Q8BXK9	RKPADLHNLAPGTHPPFLTFNGDVKTDVNKIEEFLEETLTP
Q9D6S7	IISLEEVDEDMKSVVEALKDNFNKTLNIRTAPGSLDHITVV
Q9BY71	GLQEVPEDIPANTVLLKLDANKISHLPDGAFQHLHRLRELD
Q8WY41	RVLCPVLRRYTCPLCGASGDNAHTIKYCPLSKVPPPPARPP
Q8CFI2	PIDYPYSPPAFRFLTKMWHPNIYETGDVCISILHPPVDDPQ
Q9ERB0	ELDSVPKEPSSTVNTEVYPKNSTLRTYHQKIDSNLDELSVG
P14649	VGDGKILYSQCGDVMRALGQNPTNAEVLKVLGNPKSDELKS
P51749	IEDERNLHEDFVFMKTIQRCNKGEGSLSLLNCEEIRSRFED
Q4PNJ2	ICFYLEAGDLSKETDLILTFNISMHRSWWMENGPGCMVTSV
Q9NRY7	LRITDNVGREVITLERPLRCNCCCCPCCLQEIEIQAPPGVP
Q9XSK7	KFGKPNKRKGFSEGLWEIENNPTVKASGYQSSQKKSCVEEP
Q8NE22	SMYPGTVYQKYEPIFFQSIGNPFIFRCLDGVLIDGNDKGIS
P50195	IDPFGALFEAPGFSSHEEWRNSELARQQQKTIQNHVGTFHQ
P32007	KQIFLGGVDKRTQFWRYFAGNLASGGAAGATSLCFVYPLDF
Q5ND56	CRTKSQKLTRTTIIRNFLIENRLMVTHHTVILLFLVPISQK
A8MUZ8	KCYECDKSGKAFSQSSGFRGNKIIHTGEKPHACLLCGKAFS
P01241	LCFSESIPTPSNREETQQKSNLELLRISLLLIQSWLEPVQF
O43633	KIMMEFERQAEIMDMKEEMMNDAIDDAMGDEEDEEESDAVV
Q8N954	KLEGDLRRSQRACQQLDVQKNIQVPREAWYWLRLEEETEED
Q8BFZ6	EEMCIVIDKQDQIIGADTKKNCHLMENINKGLLHRAFSVVL
Q9JKL5	HRRFKQLSGDQPTIRKENFNNVPDLELNPIRSKIVRAFFDN
Q9CQE1	HVELGYSTLVGVFHTEYGALNRVHVLWWNESADSRAAGRHW
O08859	KPGPNCGFGKTGIIDYGIRLNRSERWDAYCYNPHAKECGGV
P50195	LDELVSPKHSRFKDYCAYFVNIIPRKPTRYNSPFTPSNKGS
O88456	HLYSMIIRRYADESGNMDFDNFISCLVRLDAMFRAFKSLDK
Q9BVK8	YDFIGEFMKASVDVADLIGLNLVMSRNAGKGEYKIMVAALG
Q8QZY6	IKSYRDDIDLQNLIDSLQKANQCCGAYGPEDWDLNVYFNCS
Q61096	TPRVLQELNVTVVTFLCREHNVCTLVPRRAAGICFGDSGGP
P01904	AVDKANLDVMKERSNNTPDANVAPEVTVLSRSPVNLGEPNI
Q08E43	DEACVEVLLEYGANPNALDGNRDTPLHWAAFKNNAECVRAL
Q8TF63	KHLVCSFRLYPFTVHTVSPGNSHLALYQVFKAVKLCPSETS
P24158	AAHCLRDIPQRLVNVVLGAHNVRTQEPTQQHFSVAQVFLNN
Q9BU64	AAKYLQTNIQHFLFSLCEYLNAYSGRKYQADRLQSDFAALL
Q9UBS3	EQSFNFNFDDLFKDFGFFGQNQNTGSKKRFENHFQTRQDGG
P53004	LSFHFKSGSLENVPNVGVNKNIFLKDQNIFVQKLLGQFSEK
Q56JZ1	AGIHKKVARTIGISVDPRRRNKCTESLQANVQRLKEYRSKL
P0C7M8	EDWLLYGRKCYFFSEEPRDWNTGRQYCHTHEAVLAVIQSQK

Q9JL95	SQHQDIFEDEEAMESDPDALNKDSACPKEEDTTHFQGTPGC
P21845	GGHEASESKWPWQVSLRFKLNYWIHFCGGSLIHPQWVLTAA
Q6UWD8	TLEARATAPPAPSAPNSAPSNLGPQTVLEVPARSTFWGPQP
P61018	GTGKSCLLHQFIENKFKQDSNHTIGVEFGSRVVNVGGKTVK
P54116	TISVDGVVYYRVQNATLAVANITNADSATRLLAQTTLRNAL
P20826	ESPKRPETRSFTPEEFFSIFNRSIDAFKDFMVASDTSDCVL
Q96D70	VRNSDLVPKAKGRKSLQRLENTQYLLTLLETDGGLPGLEDG
Q8BG50	PVVFVLSFVVSFYYGTITWYNIFLVYNEERTFWHKISCCPC
Q9H7E9	PLGDEGGTASKKQKNKKKTRNRASVANGGEKASEKLAPEEV
Q3T160	LFGCELKADRDYHFKVDNDENEHQLSLRTVSLGAGAKDELH
O88452	FENNSCEIQGLHGICMTFLHNAGKFDAQGKSFIKDALRCKA
Q9H444	QEELDKNLLEISGPETVPLPNVPSIALPSKPAKKKEEEDDD
Q3T0X7	KHCYKVHDCNATVEKLEDFINTINSVLESLYIEIKKGVTED
P06717	SGYSTYYIYVIATAPNMFNVNDVLGVYSPHPYEQEVSALGG
Q9NQF3	AKAWGSLQGPPVLCLHGWLDNASSFDRLIPLLPQDFYYVAM
Q9DC16	PTVYEDKSGKQRYSYQYTVANKEYVAYSHTGRIIPAIWFRY
P01375	NPQAEGQLQWLNRRANALLANGVELRDNQLVVPSEGLYLIY
Q8WY22	SYRRTVNTFSQSVSSLFGEDNVRAAQKFLARLTERFVLGVD
O15347	NPGISIGDVAKKLGEMWNNLNDSEKQPYITKAAKLKEKYEK
Q1RMX6	NNRTSHFIEEKLQAQEKESINPPKGKGSNESISLLCHNQDV
P79099	LHEITPPAIRGDLAVNALNNNSTAGQAVTVELFLTLQLVLC
A7E2S9	FKCVHQQLLEYKQKISKNSQNSNPEGTSEGTPDEAAPLAER
Q8K201	SSAAPTPTAAPSAPEEEADSNEDPSMEEEDLLALNSSPATG
Q9D7B7	MVLCTVMLFLLQLKFLKPRTNSFYSFEVKDAKGRTVSLEKF
Q3SZW6	NTAAASAPEMIQHSLWRPVRNKEGLKTGYASKTRCKSLKIF
P35279	GEQSVGKTSLITRFMYDSFDNTYQATIGIDFLSKTMYLEDR
P59190	LLLIGDSGVGKTCLLCRFTDNEFHSSHISTIGVDFKMKTIE
Q6ZVK1	CACYFLAFLFSFVVVVPLSENGHDFRGRCLLFTEGMWLSAN
Q9D853	SPSAIKLSASILEKEGLSNINLKVEDFLNPSTKLSGFHVCV
Q96GR4	TTTWEFISSHRIAYLRQRPSNPFDRGLTRNLAHFFCGWPSG
Q53HL2	VIQVDEMIVEEEEEEENERKNLQTARVKRCPPSKKRTQSIQ
Q8N8Z3	ALVSPTPPKLSPGQLSPHSVNVHWGPQGHLHLPRSGTTVLH
Q5VXU1	ICFYLEAGDLSKETDLILTFNISMHRSWWMENGPGCTVTSV
O88456	FVQLAGDDMEVSATELMNILNKVVTRHPDLKTDGFGIDTCR
P43528	ERGTPININLYDHARGTATGNTRYNDGYVSTTTTLRQAHFL
Q9NSE7	ATHGLDSKPQSPLVCPFSNPNGRISPLARAGSSSVSRGGSP
P07992	KLEQDFVSRVTECLTTVKSVNKTDSQTLLTTFGSLEQLIAA
Q13145	VSGHENCCLTCDKMRQADLSNDKILSLVHWGMYSGHGKLEF
Q3ZC52	FRIKDPVLFVSGSADEMCEKNLLEKVAQKMQAPHKIHWIEK
Q9ULN7	QAQDSGEVTGQAGSLLGAARNPRRGRRGRRNRTRRNRLTQK
P40293	PPVMYRGDVGAGGELIIKPVNKTHRGMYRCQVSDGKKIQRS

P01731	NSSKLFSAMRDTNNKYVLTLNKFSKENEGYYFCSVISNSVM
A5D7N9	SSCRRPLGDSLSWVASQEDTNCILLRCVTCNVSVNEEQILS
Q3T0S6	AYHKYKAKRNCWPRVRGVAMNPVEHPFGGGNHQHIGKPSTI
P02741	YTVGAEASIILGQEQDSFGGNFEGSQSLVGDIGNVNMWDFV
A5D7U1	LSVTTKKSSLIHISPDIYKSNTLYTVSTQVSHGIISVVLEA
Q6PDM2	PSYGRSRSRSRSRSRSRSRSNSRSRSYSPRRSRGSPRYSPR
Q8TAT2	ERASGPAAGTPPPQSAPPKENPSERKTNEGKRKAALVPNEE
H3BNL1	QHLIHFVQVQPSHTRTTYQQNFCCPSQGGHYGSYKVGPQAP
P47878	PRPAEQYPLRALLNGRGFCANASAAGSLSTYLPSQPAPGNI
Q6FIF0	GNPRTNGMCSVCYKEHLQRQNSSNGRISPPATSVSSLSESL
P09630	SYFTNPSLSCHLAGGQDVLPNVALNSTAYDPVRHFSTYGAA
Q58DH1	SWAAEDQTSKAEKDVRPKLKNRHRAPFLSEKSTRQAPRGTN
P35243	QKLEWAFSLYDVDGNGTISKNEVLEIVMAIFKMITPEDVKL
Q9WV93	PWGSAFGHHPHIAHPLLLPQNGHGNAGTAASPTEPHHQGRL
Q5BIS9	VYLSGSFNNWSKLPLTRSHNNFVAILDLPEGEHQYKFFVDG
Q9NQX7	TQMELEEDVKIYLDENYERINVPVPQFGGGDPADIIHDFQR
P63046	RSLRTMSYRGTFQEFCRRFMNDKLGYGSWFEHVQEFWEHRM
Q9QY36	KEKGKHMVLAALENKAENKGNVLLSSGEACREEKGLAAEDS
P56857	SGILFIISGICAIIGVSVFANMLVTNFWMSTANMYSGMGGM
Q3T0M7	TKFEECRKEIEEKEKKGSGKNDSTEKVVEKLEALSVQEGEQ
Q96HJ5	IASATIALVGTAFLSLNIAVNIQSLRSCHSSSESPDLCNYM
Q9WVF9	GLHRESSEHPWKWTNNTEYNNMNPILGVGRYAYLSSDRISS
Q8VHX2	SDKYPIQDTGLPKAKECDTVNSNCPPNSDDQPQGEENDFPD
Q5E9A1	SQQAQLAAAEKFKVQGEAVSNIQENTQTPTVQEESEEEEVD
Q99766	RLCKCHYIEDDCLLRLSQLENLQKTILEMEIISCGNITDKG
Q5JQC4	LDMVHSLLHRLSHNDHILIENRQLSRLMVGPHAAARNLWGN
O08807	HTLRGLFIIDDKGVLRQITLNDLPVGRSVDETLRLVQAFQY
P58771	EKLELAEKKATDAEADVASLNRRIQLVEEELDRAQERLATA
P53808	SLAIESDGKKGSRVFMYYFDNPGGQIPSWLINWAAKNGVPN
O15347	RPPSGFFLFCSEFRPKIKSTNPGISIGDVAKKLGEMWNNLN
Q13670	GCLTKCSHAFHLLCLLAMYCNGNKGPEHPNPGKPFTARGFP
P36980	THLEGDTVQIICNTGYRLQNNENNISCVERGWSTPPKCRST
Q9NP55	TMAQFGGLPVPLDQTLPLNVNPALPLSPTGLAGSLTNALSN
Q5SVS4	CGLAGALASNPVDVVRTRMMNQRVLRDGRCSGYTGTLDCLL
Q6UWD8	PTQVGTLEARATAPPAPSAPNSAPSNLGPQTVLEVPARSTF
Q8N413	CMVSSIRQEGLGVFFRGVTINSARAFPVNAVTFLSYEYLLR
Q8N1A0	QWHHLQVEIESLHAVERGLENSLHASEQHYQMQLQDLETVI
Q91W61	SLSLAVNANVGDTAVQELARNCPQLEHLDLTGCLRVGSDGV
P97371	QLNLVTTWLQLQIPRIEDGNNFGVAVQEKVFELMTNLHTKL
O54830	FPILILSRMQAWNETLKNRINLSEGTPGIDDDILPIYKNIE
O15260	IIALQTIAYSILWDLKFLMRNLALGGGLLLLLAESRSEGKS

P0C7M4	IWFENRRAKWRRHQRALMARNMLPFMAVGQPVMVTAAEAIT
Q13491	PVFMFYNIWSTCEVIKSPQTNGTTGVEQICVDIRQYGIIPW
P62955	GLSLVVGLVLYISSINDEVMNRPSSSEQYFHYRYGWSFAFA
Q6ZRC1	HHLDVKSEMTAALRAQTYLLNPEMDSVLQSSLSSESWPIPE
Q14AM7	SVDTHQRSFDIGIQIGYQRRNKDVLAWVKKRRRTIRREDLI
Q8BFU0	YGECLHSCPSGYYGHRAPDMNRCARCRIENCDSCFSKDFCT
P08579	NSANTQGNSTPNPQVPDYPPNYILFLNNLPEETNEMMLSML
Q6ZSR6	PPRLAPLLIFNPHPSTVLSCNCEYNSFFEFCDSLQQIVIPE
Q32KW0	ISFITSEASERCHQEKRKTINGEDILFAMSTLGFDSYVEPL
Q5JUQ0	WELPDLQEGKIQAISDSDGVNYPWYGNTTETCTIVGPTKRD
O14618	GNSGERLACGIIARSAGLFQNPKQICSCDGLTIWEERGRPI
Q9H2P9	EFYGRKLVVADREEVEQEADNILKDADISDVAFLVVGDPFG
P51163	KDRWNAKSVYVVGSATASLVNKIGLDAEGAGSGNAEKLAEY
Q96EF9	LISLHPFNPWNWGKLAEAYLNLGPALSAALASSQKQHSFTS
Q6P1N9	QDVIGRAVEIGVKKFMITGGNLQDSKDALHLAQTNGMFFST
B5L3F2	NKLEMQISMMLKNNISFMYGNYEIINNNSIVGKFVAPQKIT
Q0II87	QEARDGTSQVKLKAINENWKNLSNSQKQVYIQLAKDDKIRY
Q30134	SDVGEYRAVTELGRPSAEYWNSQKDFLEDRRALVDTYCRHN
E1ANH6	AFARSLGDETFRLDRTEPTLNTAIPGDPRDTTTPLAMAQTL
P07766	RGSKPEDANFYLYLRARVCENCMEMDVMSVATIVIVDICIT
O60682	AHLRQLLQEDRYENGYVHPVNLTWPFVVSGRPDSDTKEVSA
Q2KIH8	KITRFGMASCPQVPMAEHISNLPWPPWTRTGSTRICQEAPD
Q3SZD1	GMDEGDGEGTILPIMQSIMQNLLSRDVLYPSLKEITEKYPE
Q9HC57	CQAARCQADSECPRHRRCCYNGCAYACLEAVPPPPVLDWLV
Q1RMX9	NKDQWYNVLEFSRTVHADLSNYDEDGAWPVLLDEFVEWHKV
Q3T0E2	FIKSMEDLEKNHENLLTGAQNELKKEMALLQKKIMMETVSC
P52743	FLVEKHTLHVIIDFILSKVSNQQSNLAQHQRVYTGEKPYKC
P01910	ATGKHNLEILTKRSNSTPATNEAPQATVFPKSPVLLGQPNT
Q92882	LYWACHGGHKDIVEMLFTQPNIELNQQNKLGDTALHAAAWK
P31946	THPIRLGLALNFSVFYYEILNSPEKACSLAKTAFDEAIAEL
Q8CI43	RDQGTYEDYLEGLRVFDKEGNGKVMGAELRHVLTTLGEKMT
Q9Z0S5	GTLHILAGACGMVAISWYAVNITTDFFNPLYAGTKYELGPA
Q4VC05	NKNKKKGKDEKCGSEVTTPENSSSPGMMDMHDDNSNQSSIA
Q969J5	RFTPWWETKIDPPVMNITQVNGSLLVILHAPNLPYRYQKEK
Q6QD59	EEFKSMSGTIQLGRKLITKYNRRELTDKLLIFLALALFLAT
Q00169	YHLQSKVPTFVRMLAPEGALNIHEKAWNAYPYCRTVITNEY
Q9D1Z3	HGSAKFYISDLWKVTFAQYSNVVIFGVPQMMPQLEKKLELE
Q91ZT8	LLSHGAQVNGMTIDWRTPLFNACVSGSQDCVNLLLQHGATP
Q15286	YRGTHGVIVVYDVTSAESFVNVKRWLHEINQNCDDVCRILV
P35288	KIGVFNASVGSHLGQNSSSLNGGDVINLRPNKQRTKRTRNP
Q3ZBE1	NPPVVHSALLALRYLAECRANREKMKGELGMMLSLQNVIQK

Q148G4	FMITGGNLQDSKDALHLAQTNDMFFSTVGCHPTRCDEFEKN
O88822	LFLLELRGYSKLYDDIGDFPNGWIHLMVSVVSFLFFTDMLI
A6NMN3	SSFEMASNTDMRWDLEACKSNCSPEPEDIDLLECCLQELRE
Q58DW5	RYLIEEDEDAYKKQFSQYIKNNVTPDMMEEMYKKAHAAIRE
P42574	VDAANLRETFRNLKYEVRNKNDLTREEIVELMRDVSKEDHS
P68401	KNGELENIKPKVIVVWVGTNNHENTAEEVAGGIEAIVQLIN
P22794	ENTSKSHGEIFKKDVCAENNNNMAMLICLIIIAVLFLICTF
Q3SYV5	DFGQKEFSCCGGISYKDWSLNMYFNCSEDNPSRERCSVPYS
Q9D753	ACTFALLAALKNVQLPEVTINEETALAEVNLKKKSYLNVRT
Q9Y2Y8	LTEEVIQAEGEEVKASACQDNFEDEEAMESDPAALDKDFQC
Q8R2N1	GPNGTAGIFATYPSGHLDMVNGFFDQFIGTAALIVCVLAIV
Q9DBZ5	FNPAFFQTTVTAQILLKALTNLPHTDFTLCKCMIDQAHQEE
Q14749	LDKDVPQSAEGGFDAVICLGNSFAHLPDCKGDQSEHRLALK
Q7SIB2	SCLEEFRSAPFIECHGRGTCNYYANAYSFWLATIERSEMFK
Q9UNI1	MTAAHCVDYQKTFRVVAGDHNLSQNDGTEQYVSVQKIVVHP
P33792	FSVDSMIKFSGEDDFFLVNGNSKEREYIYVTLSELISEKNN
Q7Z2X4	YCTADHNVSPNIFAWVYREINDDLSYQMDCHAVECESKLEA
P06836	TRKKLKGEKKGDAPAAEAEANEKDEAAVAEGTEKKEGEGST
Q8C8M1	CNACVLLVKRWKKLPAGSKKNWNHVVDARAGPSLKTTLKPK
Q3UFY7	GGDRLQVISDFDMTLSRFAYNGQRCPSSHNILDNSKIISED
Q66JS6	EALVRDVCISLEIDDLKKITNSLTVLCSEKQKQEKQSKAKK
P30115	DIALVELLYHVEELDPGVVDNFPLLKALRSRVSNLPTVKKF
Q5TGJ6	FLSPKRLFPYKECKEKFGKPNKRRGFSAGLWEIENNPTVQA
A6NFK2	ANDHKPLPIIDFGKIIIYTNNLKIIRTPMDKRDFVRKILQK
Q9UKR0	LPNDCATAGTECHVSGWGITNHPRNPFPDLLQCLNLSIVSH
P20615	IISHESEDAPPAKFPSGQYANPRQPGHAEHLDFPSCSFQPK
Q8BH95	KFLSHWDHITRVKKPVIAAVNGYALGGGCELAMMCDIIYAG
Q62361	DLGEVGAWRPHKRQHPGRRANQDKDSWSDEGDSDWLPPSWL
Q9BPW8	NIYELRTYKLKPGTMIEWGNNWARAIKYRQENQEAVGGFFS
Q9D1N2	QPGSGWAQEDGDADPEPENYNYDDDDDEEEEEETNMIPGSR
P35278	SLAPMYYRGAQAAIVVYDITNTDTFARAKNWVKELQRQASP
P26452	QMKEEDVLKFLAAGTHLGGTNLDFQMEQYIYKRKSDGIYII
Q9H1U9	LRGPIKEHLPTATTHSAHLVNDFICGGLLGAMLGFLFFPIN
Q9NRY7	VGRISKHWSGFLREAFTDADNFGIQFPRDLDVKMKAVMIGA
Q8N699	YTHGLNRTGFYRHSGCERRSNLSLASLTFQRQASLEQANSF
Q91X91	AKSPGVLAGRPFFDAIFTQLNCQVSWFLPEGSKLVPVVKVA
Q99727	HLCNYIEPWEDLSLVQRESLNHHYHLNCGCQITTCYTVPCT
P05820	NMCLQALTQLEDYIKKHGASNPLTLQIISTNIGYFCNADRN
Q3ZBH3	IIAGALAYIYYQQLNAELKENLKDTMTRRYHQPGHEGVTSA
Q32L08	EASLKRCCNLTDEGVLALALNCRLLKIIDLGGCLGITDVSL
P14435	TWLRNSKSVTDGVYETSFLVNRDHSFHKLSYLTFIPSDDDI

Q91X52	ALDMLTKMMALELGPHKIRVNAVNPTVVMTPMGRTNWSDPH
P30404	ENFKLKHEGPGVLSMANAGPNTNGSQFFICTIKTDWLDGKH
O95999	ASTVMYHPEGESSTTPFFSTNSSLNLPVLEVGRTENTIFSS
Q99N07	TLWWKQSSSAFSGNVIFLSQNSKNKSSVSSESLCNPTYENI
Q2YDH9	SKVQQFSEKAFLLQLLKTHENALERQCNEITNQRNMLLQTF
A6NFD8	AELLAEFANYFHYGYHECMKNLVHYLTTVERMETKDTKYAR
O55242	YPGETVVHGPGEATALEWGPNTWMVEYGRGVIPSTLFFALA
P48060	GGNYPTWPYKRGATCSACPNNDKCLDNLCVNRQRDQVKRYY
Q92915	IYSSMLYRQQESGRAWFLGLNKEGQAMKGNRVKKTKPAAHF
P50195	ILGHVEGWRDMGIGGIVDLLNEERRIIAEVKNKYSTVTGGD
A6QLK6	DELAFNKGDTLKILNMEDDQNWYKAELRGAEGFVPKNYIRL
Q2TAA2	FSGYNTRWAKIILPRLIRKGNSLDIPVAVTIFFGANDSALK
O02756	DKAKRRNQEMQQKLVELSAENEKLQQRVEQLTRDLAGLRRF
Q0V882	GFAFLTGVGLGPALDLCIAINPSILPTAFMGTAMIFTCFTL
P98173	NGVSGELIEARAFDMWAGDVNDLLKFIRPLHEGTLVFVASY
P10279	RENMHRYPNQVYYRPVDQYSNQNNFVHDCVNITVKEHTVTT
Q9Y2Y6	LDARTTALLLSVSHLVLVTRNACHLTGGLDWIDQSLSAAEE
Q8WWF3	MKKPKQNQLTPVTNSEVALVNAYPEQRRARRQSQFNEVNQN
Q9D8Y0	QVLARLSEIDVSTEGVKGAKNFFEAKVQAINVSSRFEEEIK
P27701	LMNRPEVTYPCSCEVKGEEDNSLSVRKGFCEAPGNRTQSGN
P97478	KEGAMACTVAVEESIANHYNNQIRMLMEEDPEKYEELLQVI
P00848	PKGRTWTLMIVSLIMFIGSTNLLGLLPHTFTPTTQLSMNLS
Q3T123	RLETAERFQKHLERVIEMIQNCLASLPDDLPHSEAGMRVKT
Q8TBF4	RAINNKQLFGRVIKASIAIDNGRAAEFIRRRNYFDKSKCYE
Q8NGA4	IGTCAHLIRAKLLREGWVHANRPKRLLLVLVSALSAGSHLT
P47741	LAPLTVLLALYLLRKAWRLPNTPKPCWGNSFRTPIQEEHTD
Q3TBL6	DDTSSEIFDELYKVTEIHTHNKKEAHKIMKDAIKVAIKIGI
P00846	AMAIPLWAGTVIMGFRSKIKNALAHFLPQGTPTPLIPMLVI
P35452	PYTKQQIAELENEFLVNEFINRQKRKELSNRLNLSDQQVKI
Q96L21	CGRDGFHMRVRLHPFHVIRINKMLSCAGADRLQTGMRGAFG
Q9Y6N3	KCTFIPKRSQTAKESIVFMQNLDSVTEFCTEKTHNKEAPNL
Q9D9J8	GWLIGGMNFQQQQEISINITNVQLDCGGIQMAFPKEWFSAN
Q8CC84	SSEEESHQVVLRPRGKEHQKNSSQRPGAGTMVLLQRELAQE
O15305	IGRSCSQEERIEFYELDKKENIRQKFVADLRKEFAGKGLTF
Q9ULZ2	VDKLDIVDLTCLTEQNSTEKNCAKFTLVLPKEEVQLKTENT
Q9DC16	NELYVDDPDKDSGGKIDVSLNISLPNLHCELVGLDIQDEMG
Q9QXT8	ILREDAPLEHVERFFQKMDRNQDGVVTIDEFLETCQKDENI
Q8C5C9	MLDGPQEELTPLNNDLRIQPNSAEDPSPAQVGKESPWSPCN
Q8IUK8	GYPVISAFAGDQDVTREAASNGVLLLMEREDKVHLKLERGN
Q8N816	QLSAEADACPSVLYSEVLEWNKNINTYTSFHDFCLILGIFL
Q8BNX1	GYRWVDGASLNFSHWNSGEPNDSRGHEDCIMMLHSGLWNDA

Q16890	RQVLSAKERHLVEIKQKLGMNLMNELKQNFSKSWHDMQTTT
Q9CQ88	QFGISCSCLAINRNTQADVINASWSVLSNSTRHELERSFDC
Q9JK37	LGKKISVPRDVMLEELSLLTNRGSKMFKLRQMRVEKFIYEN
Q58CQ0	PAWGLEQRPRGPEKPGVDATNAKLPANQPSLMHKVDEHEQD
Q8WTT0	AVVSILLLSVCFTVSSVVPHNFMYSKTVKRLSKLREYQQYH
P22994	VVVAGHLQGELQKHVLDGFQNDFRHSVRLGGQLRQVHHARH
Q08091	QRGMTVYGLPRQVYDPKYCLNPEYPELSEPTHNHHPHNYYN
P47758	QDIAMAKSAKLIQQQLEKELNTLRVTRSAAPSTLDSSSTAP
Q96S44	LIHGDLTTSNMLLKPPLEQLNIVLIDFGLSFISALPEDKGV
Q8R4E4	QDYSSGSRSHTPIPRDYRNFNKTPVPFGGPHVREAIFHAGT
Q9H8W4	TPTKSFAVYAATATEKSEWMNHINKCVTDLLSKSGKTPSNE
P62827	KVDIKDRKVKAKSIVFHRKKNLQYYDISAKSNYNFEKPFLW
P09936	MKQTIGNSCGTIGLIHAVANNQDKLGFEDGSVLKQFLSETE
Q3TUU5	LASHLASHGFFCLRFTCKGLNIVHRIKAYKAVLNYLKTSGE
O14543	YYIYSGGEKIPLVLSRPLSSNVATLQHLCRKTVNGHLDSYE
A8MVS1	LLTHSRKKPYVSKQCGKSLRNLLSPKPRKQIHTKGKSYQCN
Q9KIP8	ISTKYLLQDAQANGYAVPAFNIHNAETIQAILEVCSEMRSP
P12961	VMEQLGIARPRVEYPAHQAMNLVGPQSIEGGAHEGLQHLGP
Q14512	QIKQKSRPGNKGKFVTKDQANCRWAATEQEEGISLKVECTQ
Q3ZBY3	LNGDGKLGLSEMSRLLPVQENFLLKFQGMKLTSEEFNAIFT
P01138	KTTATDIKGKEVMVLGEVNINNSVFKQYFFETKCRDPNPVD
Q810N6	LFSSQQEYEESQEAEETGAENPLLQPTLTGDVEGLQKIFED
Q99LP6	SFRPSPRLLCTATKQKNNGQNLDEDLGHCEPKTDPPSADKT
Q2NL33	SAYEYAQINESKGCAYFQNGNIYISRCSAEISWICEKTAAL
P63013	YSAMATYSATCANNSPAQGINMANSIANLRLKAKEYSLQRN
Q9UBW8	IHQVLEAPGVYVFGELLDMPNVRELAESDFASTFRLLTVFA
P04768	IQPCSWILLLLLVNSSLLWKNVASFPMCAMRNGRCFMSFED
P50295	LYWALTKLGFETTMLGGYVFNTPANKYSSGMIHLLVQVTIS
Q61500	PHTYVVREDLVAVEEIRDVSNLGIFIYQLCNNRKSFRLRRR
Q6ZTC4	NPHPQLQLRGKRAAGLLLRRNPWCHPQAPGGSSTWAPSLPP
Q495C1	VCPHWLLLLAFSSGRHGELTNSKTLPIYAEVQRAVLFPFQQ
P52823	ECDEDTVSTIRDSLMEKIGPNMASLFHILQTDHCAQTHPRA
Q9Y2B4	KRCWHSLLSVPKILRISSGENSACNKTKQNNEEFQEIGCSE
Q9Y320	VFLMTCKPPLYMGPEYIKYFNDKTIDEELERDKRVTWIVEF
Q9NRY7	QNQPGRPEGVPWMPAPPPPLNCPPGLEYLSQIDMILIHQQI
Q9JKT4	AAEGILLSIATVEAGLGVLGNTFIALVNCMDWAKNNKLSMT
Q9Y365	RLTVNADVGYYSWRCPKPLKNRDVITLRSWLPMGADYIIMN
Q9H3R5	KLLEIQTEKNKQKIDLDSMENSERIKIIRQNLQMEIKITTV
Q96S21	IDKLPLPVTIKSHLKSFSMANGMNAVMMHGRSYSLASGAGG
O14990	LNIKLARQLMWKELQSEDNENEETPQGTNEEKTAAEESEEA
Q9WUK2	RRAGPPMGSRFRDGPPLRGSNMDFREPTEEERAQRPRLQLK

P52803	SCMKTIGVHDRVFDVNDKVENSLEPADDTVHESAEPSRGEN
Q3ZC48	PYISDTGTVAPEKCLFGAMLNIAAVLCVATIYVRYKQVHAL
A1A4M6	YVRGFNHPCGCFCEPLPGEPNKTSLVTFFQTDLSGYLPQSV
Q8BKE5	LRCDPQVLQMLKEEHQIILENQKNFGLYVQEKRDGLRRRQR
Q96CE8	GICFSGIMMLIVTTVLLVLENNNNYKCCQSENCSKKYVTLL
O55125	LWRFSGGYPALMDCMNKLKNNKEYLEFRKERSKMLLSRRNQ
Q62447	TSVLKTRFSYAFPKEFPYRMNHILECEFYLLELMDCCLIVY
Q8R1M8	NLVAIFKAPTNLPDPVRICVNWESVSGIAEFWLNGKPLGRK
P52744	HKKIHTRENFYKCEECGKTFNWSTNLSKPKKIHTGEKPYKC
Q3Y5Z3	PLPKGACAGWMAGIPGHPGHNGTPGRDGRDGTPGEKGEKGD
Q9NYW4	WLKQRAYNLSLWCLLGYFIINLLLTVQIGLTFYHPPQGNSS
Q64ET8	RENCRKRKMSSKDSCQDTAGNCPEKECSLSLNKKSRSSTAV
O55060	PRANIGKFDRIWDRGALVAINPGDHDRYADIILSLLRKEFQ
Q8WXK3	QGQARCVQLLLAAGAQVDARNIDGSTPLCDACASGSIECVK
Q9H6K1	LSSFETEFNTQPHRKVEGNFNPFASPQKNRQSDENNLKDPG
Q9BYL1	LVYVEAFSQHAITGRALLRLNAEKLQRMGLAQEAQRQEVLQ
P63103	TELRDICNDVLSLLEKFLIPNASQAESKVFYLKMKGDYYRY
O00233	VDDEIVEFGSVNTQNFQSLHNIGSVVQHSEGKPLNVTVIRR
P05813	FRPICSANHKESKMTIFEKENFIGRQWEISDDYPSLQAMGW
Q96CG8	GFKGEKGECLRESFEESWTPNYKQCSWSSLNYGIDLGKIAE
Q9NYV7	PSAIGNYIQIQLLTMEHLPRNSTVTDKLENFHQYQFQAHTV
P45879	GWLAGYQMNFETAKSRVTQSNFAVGYKTDEFQLHTNVNDGT
Q8NE22	CYQEFDVPAVFPIELKQYLPNIAYSYDKQSPLRCVVLVALR
Q02105	VSSGGVLLRLQRGDEVWLSVNDYNGMVGIEGSNSVFSGFLL
Q5M956	GWNHDSTHVIRFPLNGYCHLNSVQVLERLQQRGFEIVGSCG
P21583	FAFGALYWKKRQPSLTRAVENIQINEEDNEISMLQEKEREF
Q5TBK1	MALENNYEVIFREPDTRWKFNVQELARRNIHGVSREKIHRM
P51177	EVVDRIFLLVDENGDGQLSLNEFVEGARRDKWVMKMLQMDL
Q3SZ21	DLVCITVTEKLPFYFKRPPINVAIDRGVGFELLYSPAIKDS
P47803	SAAVAWGRYHHFCTRSRLDWNTAVSLVFFVWLSSAFWAALP
Q9BQQ7	PRVHSIYKVEEVVKPWASGENVYSYACQNHICRNLSIFCCC
Q68FF9	RGGKPTLLFTFVLAFLFCTLNGYLQSRYLSQFAVYAEDWVT
Q9CX92	FGFQVKPIYLCDTESLGKKPNILKCMVPEHFEKTIFLIAMY
P40306	TIAGLVFQDGVILGADTRATNDSVVADKSCEKIHFIAPKIY
P28801	DMVNDGVEDLRCKYVSLIYTNYEAGKEDYVKALPQHLKPFE
P04973	ADEAFYKQPFADVIGYVTNINHPCYSLEQAAEEAFVNDIEE
Q80XF5	RFTPWWETKLDPPVVTITRVNASLRVLLRPPELPNRNQSGK
P21796	FGIAAKYQIDPDACFSAKVNNSSLIGLGYTQTLKPGIKLTL
Q99714	VAGEMGQNEPDQGGQRGVIINTASVAAFEGQVGQAAYSASK
Q1ZZU3	GPVHLRGRSPTPALDPLAPLNPLIRGPRTPGLRRWIQSLAL
Q9NVA1	RIIVHFMWEDVQQRGRVMGVNPYILKKNMILMTNHFYAAIL

Q8N954	LRQIREARRKEEKQQEANLKNRQKSLKEEEQERRDIGLKNA
P09056	RMVAYLSASLTNITRDQKVLNPTAVSLQVKLNATIDVMRGL
Q8VDK3	TFLPNASFCDPLMPWTDLFSNEDYYPAFEHQTACDSYWTSV
Q16637	PPPPPMPGPRLGPGKPGLKFNGPPPPPPPPPPHLLSCWLPP
Q86Y79	ELDKPLGRLALKLGGSARGHNGVRSCISCLNSNAMPRLRVG
Q9BPW5	LQLASMLGCSFYEVSVSENYNDVYSAFHVLCKEVSHKQQPS
Q8BG34	PDEMPELLLQQAPTRTASSLNRYPVLPSINRRSLEVGAVDT
Q9H160	NEPTYCLCNQVSYGEMIGCDNEQCPIEWFHFSCVSLTYKPK
O70552	IRINNNENKDPVLERACAESNVNFFHLGLPKEMTIWVDPYE
B5L3F2	QISMMLKNNISFMYGNYEIINNNSIVGKFVAPQKITYNKLL
P97823	QAAETVKALIDQEVKNGIPSNRIILGGFSQGGALSLYTALT
Q86XK3	EFQESFKHIDSEFEENTNLKNTLKNLNVCESQSLDSGSCSA
Q9D3G2	WAPNISDITYSWRWEGTVDFNGEVRSHFSNGQVLSVSLGLG
Q8BHX3	RTKQIESDRQTLLKEVENLYNIEILRLPKALQGMKWLDYFA
P61267	KLSEDVEQVKKQHSAILAAPNPDEKTKQELEDLTTDIKKTA
P01211	YGGFMKKMDELYPLEVEEEANGGEVLGKRYGGFMKKDAEED
P97361	IHLVLGDCTHSPGSLKISLLNGVTPVQSFLDNLTGILTKVL
A6NKC4	LVNTVLWVTIRKELKRKKKWNLEISLDSGHEKKVISSLQED
P36368	TRWQKPDDLQCVFITLLPNENCAKVYLQKVTDVMLCAGEMG
Q8CG80	QGFMHMKLSRTKEHKYVLGQNSPPFSSVPEIVHHYASRKLP
Q8SQH5	CVVRIPKEQGVLSFWRGNLANVIRYFPTQALNFAFKDKYKQ
Q3KNJ2	AHKDSGETQASSSTSPRGTDNQPEEPVSLSSTLSEPEYEPV
P60202	TEKLIETYFSKNYQDYEYLINVIHAFQYVIYGTASFFFLYG
Q5E9B7	LDIFAKFSAYIKNSNPALNDNLEKGLLKALKVLDNYLTSPL
Q8BW00	ELDKPLGKLALKLGGSARGHNGVRSCISCLNSNAMPRLLVG
Q3T144	VGSYTTTNISLIPPRSEHLVNFTAKAEMGGPYSYVYFFCTL
O54831	YMLDFIEDMEYLVKALTCCHNYSIKTPENLDEAQQIPFNEF
Q17QK6	GQPEEASPEEQQEEACAEEANGGEERPEDDGEGEAAYLDEL
Q0VD50	KKKGLISAYHRVIMAELATKNSKWVEVDTWESLQKEWTETA
P36369	QEEPSAQHRLVSKSFPHPGFNMSLLMLQTTPPGADFSNDLM
O08603	KKCLTQPLKNLCQKLRNKVSNTKVDTHKTNGYPHLQVTMIY
Q8NB15	GERRIYRHRSVSELFLKPVLNMCSVLRILGCTWAAALLILN
P50294	FTSRRFSYKDDVDLVEFKYVNEEEIEDVLKTAFGISLERKF
Q9CWY9	QVGSPHWKETFRQGCLERMRNSRHRLLNKYRQAAGSTPGTA
Q6P7N7	WRLARGVISTDDEVFKPFQANSHFVKFKYAQEYDSGTYRCD
Q3T186	SKAEEAKKLAGRAAVENHVRNNQVLGIGSGSTIVHAVQRIA
Q9Z2D8	YLGGSMDLSTFDFRTGKMLMNKMNKSRQRVRYDSSNQVKGK
Q9QYL7	SLHNTPMGARKRSPFRYDLWNLKYLHRFTWSHLSEHLAFER
Q9UKR0	LLRLRLPVRVTSSVQPLPLPNDCATAGTECHVSGWGITNHP
Q9BSJ6	VGALGSLCRQFQRRLPLRAVNLNLRAGPSWKRLETPEPGQQ
Q8R3J5	LEDREGCTWGVAYQVRGEQVNEALKYLNVREAVLGGYDTKE

Q9HAC8	FEGRKEIWDALKAAAYAAEANDHELAQAILDGASITLPHGT
Q8TBE9	QPGDCVMVGDTLETDIQGGLNAGLKATVWINKNGIVPLKSS
Q58DN3	HQHFQEPEVGCCGKYFLFGFNIVFWVLGALFLAIGLWAWSE
Q92688	DISTLEPLKKLECLKSLDLFNCEVTNLNDYRESVFKLLPQL
P05820	AKPAPSQYDYRSMNMKQMSGNVTTPIVALAHYLWGNGAERS
P41047	INETGLYFVYSKVYFRGQSCNNQPLNHKVYMRNSKYPEDLV
Q8BTZ5	DPNIRDSRGRTGLHLAAARGNVDICQLLHKFGADPLATDYQ
O54879	GKMSAYAFFVQTCREEHKKKNPEVPVNFAEFSKKCSERWKT
P47757	PLEDGAMPSARLRKLEVEANNAFDQYRDLYFEGGVSSVYLW
P01213	DLYKRYGGFLRRIRPKLKWDNQKRYGGFLRRQFKVVTRSQE
Q6PHN9	VYDVTSAESFVNVKRWLHEINQNCDDVCRILVGNKNDDPER
Q9JL59	NYSWKLHGFQAEFKNFNLTVNAADRQKTEDLPVTKVPDKPP
P19652	YFTPNKTEDTIFLREYQTRQNQCFYNSSYLNVQRENGTVSR
Q8VHZ7	PVGLIVSHLPFGPTAYFTLCNVVMRHDIPDLGTMSEAKPHL
P26892	ICEKNDECESSKETLAENKLNLPKMEEKDGCFQSGFNQAIC
Q17QN8	IPWKVQFNLGNSSRPSNQCRNSIQGKHLITDELGYVCERRD
Q8NC01	NKAGAHRCSPCTEQWKWHGDNCYQFYKDSKSWEDCKYFCLS
Q9HAV7	ALALSLRPSPRLLCTATKQKNSGQNLEEDMGQSEQKADPPA
P13972	YLDKQFFVDVFSIPACVRNTNGDLSATNEKFSKEFIGSLDI
Q8BZ09	GLTEAVVVNPFEVVKVGLQVNRNLFKEQPSTFAYARQIIKK
Q80VJ2	MRCPAGGAEVEMAELYVKPGNKERGWNDPPQFSYGLQTQTG
Q8TAG5	SVLYVQQGLSSQAKFTEFPRNVTATEGQNVEMSCAFQSGSA
Q9NWS0	RELVITIAREGLEDKYNLQLNPEWRMMKNRPFMGSISQQNI
Q16534	LPLNPTFIPPPYGVLRSLLENPLKLPLHHEDAFSKDKDKEK
Q9TU47	IEDQDELSSLLQVPLVAGTVNRGSEVIAAGMVVNDWCAFCG
Q8TDQ7	GSSLVSRTRLKTLAMDTILANAKYFDGDLSKVPTMALTVGV
P05529	YMLSKNSINRYSDMSDFIQSNNETTQKRHLLKKLWEQETDT
Q9CR36	KIAGMCRGIPTYVAEEIPGPNQPLYSKKCYTADILWILRMS
Q9CX47	SGTELLARLEGRSSLKELEPNLFADEDSPVHGDIFEFHGPE
Q2NL01	SEAEPAFRFLVDSSKKEPRWNFWKYLVNPEGQVVKTWRPEE
Q9UMX6	DEEASQYVEGMFRAFDKNGDNTIDFLEYVAALNLVLRGTLE
Q6NTF7	CGSQVPVEVMGFPKFADCWENFVDHEKPLSFNPYKMLEELD
O94778	IGFAVTVDILAGGPVSGGCMNPARAFGPAVVANHWNFHWIY
P07628	DDLQCVFLKLLPIKNCIENHNVKVTDVMLCAGEMSGGKNIC
Q8BTG6	LLQVQDREQPIPWKVQFNLGNSSRPSNQCRNSVQGKHLLTD
Q9H0X6	RPTPTLAPKRAWPSDTEIIVNQACGGDMPALEGAPHTPPLP
P0AG05	ALNETLTLWNSPPDWAGDERNVVLTLSRIWYSAVTGKIAPK
Q9NPB1	FELEPLPGAVEAVKEMASLQNTDVFICTSPIKMFKYCPYEK
P02747	YRSGVKVVTFCGHTSKTNQVNSGGVLLRLQVGEEVWLAVND
Q3SYR3	KEAGCRLRIMKPQDFEYIWQNFVEQEEGESKAFEPWEDIQE
Q68FF9	MAWPLYECIRPAAARLGNLPNRVLLAMFLIHYVQRTLVFPV

A6NJV1	LHKPSYTRFNLDSHRSTELTNFYQMVQQHRKYYQDKTGTVP
Q2YDP3	HMDGDKGPRRGRKSGSSGRKNGGMGSKGKDKKLSGTDSEQE
Q9R1Q6	SVASLGLSLRSMYGRSSQALNEEESERKLLDGHPAPASPAK
Q7RTY3	EGRWILAGVLSWEKACVKAQNPGVYTRITKYTKWIKKQMSN
Q24K06	RLLPGQLQRLRELRTIWLSGNLLTDFPPVLLHMPFLEIIDV
Q12846	MQSEYREKNVERIRRQLKITNAGMVSDEELEQMLDSGQSEV
Q8IYP2	STWSYNVCDIYKEPDSLQTVNISVISKPQCRDAYKTYNITE
Q3ZBE1	LASEIYDILQSSNMADGDSFNEMNSRRRKAQFFLGTTNKRA
P59544	NHFSMWLAANLSIFYLLKIANFSNLLFLHLKRRVRSVILVI
Q8C432	ELLLTPKRLASLVSPSHTSSNCLVITIMEVQRESSPSLSST
Q8NC54	SRTDSPSPTVLNSHISTPNVNALTHENQTKPSISQISTTLP
Q9CQQ7	EKAQQALVQKRHYLFDVQRNNIALALEVTYRERLHKAYKEV
P97328	KYPEEDTDRRCLSQRWQRGGNASNSCTVLSLLGARCAFMGS
A0JNC3	LGLGITIAFLATLITQLLVYNGVYQYTSPDFLYIRSWLPCI
P26441	IRSDLTALTESYVKHQGLNKNINLDSADGMPVASTDQWSEL
P0DMC8	IKYIKRHFPSLSIIVLTMNNNPAILSAVLDLDIEGIVLKQG
Q9TVC8	SDWDEYRAVTPLGRPDAEYFNSQKDFLEQTRAEADTVCRHN
Q0VCT4	DANEEYLFKAMVAFSMRKVPNRETTEISHVLLCNVTQRVSF
Q9Z1P5	ESCAQNGQCQPQSALPCSCDNISGCSDVSDKNLNCSRPPCQ
P15319	PRSEQTVKSANYNTPYLSYINDYGGRPVLSFICNGSRCSVK
P61027	EQIAREHGIRFFETSAKANINIEKAFLTLAEDILRKTPVKE
Q53HI1	FEFAAWQMLYLFTSPQRVYRNFHYRKQTKDQWARDDPAFLV
P43464	QYTVLYTSNCTIDVYTKEGSNTYLRNELIFLERGINISVRL
Q9JKE2	LEEERYDLVEGQTLTVKCPFNIMKYANSQKAWQRLPDGKEP
P26838	TNYFESPFKGKLLTEQVKNPNIKVGRYSYYSGYYHGHSFDD
P61266	IRKTQHSTLSRKFVEVMTEYNATQSKYRDRCKDRIQRQLEI
Q8NGA4	LGEWACKLYTGFVFLTFSTSNCLLVLISVDRCISVLYPVWA
P28067	SCIVTHEIDRYTAIAYWVPRNALPSDLLENVLCGVAFGLGV
Q5E9V3	ETKAQINHNIAMQNEKLDGINLESGSQQAPFTPPNTPDPRS
Q62000	NAKHNKIKSKGIKANTFKKLNKLSFLYLDHNDLESVPPNLP
P67868	TGFPHMLFMVHPEYRPKRPANQFVPRLYGFKIHPMAYQLQL
Q07817	RIAAWMATYLNDHLEPWIQENGGWDTFVELYGNNAAAESRK
Q9NVX0	MNQALAKMDILVTETEELAENILKWRKQQNEVSSCIPKILA
Q9JJ57	KRELQVLYRGFKNECPSGVVNEETFKQIYAQFFPHGDASTY
P21796	GSLETKYRWTEYGLTFTEKWNTDNTLGTEITVEDQLARGLK
Q9Z1Q5	SGLDIFAKFSAYIKNSNPALNDNLEKGLLKALKVLDNYLTS
Q2YDP6	MKEMQKDMDEKMDVLINIQKNSKFPLRRGLKMQQELRLIGK
O95484	GILVLIPVCWTAHAIIQDFYNPLVAEALKRELGASLYLGWA
Q6P2I7	VGNENEERGTPYASRFKDMPNFIALEKSSVLRHCCDLLIGI
Q9WU03	YDTEKNSCISFIYGGCRGNKNSYLSQEACMQHCSGKQMHPF
P15328	PEDKLHEQCRPWRKNACCSTNTSQEAHKDVSYLYRFNWNHC

P35705	YGVLLEGPGLALRGLFIIDPNGVIKHLSVNDLPVGRSVEET
Q9CQ29	GEHHNCYRELRDAWVQRHERNRTLLLGLLGRVRRVHLTTSI
P41439	RTSYTCKSNWHKGWNWTSGINECPAGALCSTFESYFPTPAA
P09564	GVACVLARTQIKKLCSWRDKNSAACVVYEDMSHSRCNTLSS
Q8BFU0	DYSQCQGNRWRRNKRASYVSNPICKGCLSCSKDNGCSRCQQ
Q9BRQ4	SDSSGQWIILGTEVKKIEAINVPCTQLSMSFFHRLYDEDIV
Q14681	MSDGWKFEQLISIGSSYNYGNEDQAEFLCVVSRELNNSTNG
Q9H2R5	EARSHRNDIMLLRLVQPARLNPQVRPAVLPTRCPHPGEACV
P50540	RAQKHSSGSSNTSTANRSTHNELEKNRRAHLRLCLERLKVL
A6NJI9	LHHNELTNIDATVKELKGMLNLKILSLYQNPLCQYNLYRLY
Q8IUW5	GEVTVLSVGRFRVTKVEHKSNQKERRSLMSVSGAETVNGEV
Q9D722	RGISTPKEFNAGENSTSLDVNHTGAAIEPLRSSVLRLPSES
Q86WI0	ALYPLGWNSPEIMQTCGNVSNQFQLGTCRLGWAYYCAGGGA
P0DMS9	KRSFVLDTASAICNYNAHYKNHPKYWCRGYFRDYCNIIAFS
A8MUP6	DKFIQRGFHVGLWQSCEESLNGEDEKCRSFRSVVPAEEQGV
Q8N7S6	KAVTCAAHLPGAAASRPLSPNKPDRVRPGQRDRIGAKRQRR
Q9NY72	LIEMIYCYRKVSKAEEAAQENASDYLAIPSENKENSAVPVE
Q8BGZ2	VQPNSIPSAIYPAPVAAPRTNGVAMGMVAGTTMAMSAGTLL
A2AWP0	RAVSIVFVPCGHFVCTECAPNLQLCPICRVPICSCVRTFLS
P61981	KELEAVCQDVLSLLDNYLIKNCSETQYESKVFYLKMKGDYY
O08756	VAGEMGQNEPDQGGQRGVIINTASVAAFEGQVGQAAYSASK
Q64124	RKLSKIETLRLASSYISHLGNVLLVGEACGDGQPCHSGPAF
Q2KJD3	EEQARKEQEQKAEEERIRMENILSGNPLLNLTGPSQPQANF
Q99990	MKKTAIRLPKGKQKPIKTEWNSRCVLFTYFQGDISSVVDEH
Q0Z7W6	CQNLQPEWESFAEWGEDLEVNVAKVDVTEQPGLSGRFIITA
P47879	ASQSRTHEDLFIIPIPNCDRNGNFHPKQCHPALDGQRGKCW
Q5UKY4	CHREKNNGHSVFCFISHLTDNWILSMEQNRGTTSILPSLLS
P09067	SSLSGSYRDPAAMHTGSYGYNYNGMDLSVNRSSASSSHFGA
Q96FW1	KSKEDLVSQGFTEFTIEDFHNTFMDLIEQVEKQTSVADLLA
Q3ZBR5	MPDEEKKRRREESSRLKEEGNEQFKKGDYIEAESSYTRALQ
Q96PG2	VTAWRGDCPSAKNDDACLVPNTPLHLKGLPVEPPPSYQSVI
Q9P2W1	ILLRYLQEQNRPYSSQDVFGNLQREHGLGKAVVVKTLEQLA
P13725	ARPNILGLRNNIYCMAQLLDNSDTAEPTKAGRGASQPPTPT
Q9R088	GKMPEVDYAILSEWFDWIVRNIDVSVDLIVYLRTTPEICYQ
Q8CHJ2	AGMILAVLLHQGHLPRLFQRNLFYRQKSKYRTPRGKLSPGS
Q5XFZ0	HHGKSRSFLWALKSNAATAANKASPKTPKRTAPGSANLGQS
Q80XM9	IVMILTMLLMLKLCTEVRVANELNIKRRSFAATDSKDEELR
Q6PAM0	QVILNKDTNISCDPALLPEPNHVMLNHLYALSIKDSVMVLS
P25402	DAKNKMGVFSPFSKVCLGNVNTSGNITVTAFNDYGVATSYT
P25402	RDEAENNIKLINSGTDSCLENTTPYYFAISDVKINGKSIDL
A6NDD5	DREPQEGPPEQPTGPGQAAENVTIQTVSYGVQEELRDQEDD

P09428	LDLGSMGDGNIQLQISHQFYNKSFRQVVSVIVAMEKLRNSA
P18469	LEDARATVDTYCRHNYEIFDNFLVPRRVEPTVTVYPTKTQP
Q3SZX2	LHYNEGLNIKLARQLISKDLNDEEEDEEMSETAAGESMNME
Q64368	PGADILPNECSVHDAAPASGNGPQKKSVDRSIQTVVSCLFN
Q8K2J7	VERDVCQRCRHKRWHFIKPTNKTKEGRPRRQGEVTVLSVGR
Q9NVZ3	NGRRAFIGIGFGDRGDAFDFNVALQDHFKWVKQQCEFAKQA
P54821	PALGGRLDSPGNLDTLQAKKNFSVSHLLDLEEAGDMVAAQA
P24158	QQHFSVAQVFLNNYDAENKLNDVLLIQLSSPANLSASVATV
Q91YQ1	SKENGFTGWTETSVKENKNINEAMRVLVEKMMNNSREDVMS
Q9Y275	EAPAVTAGLKIFEPPAPGEGNSSQNSRNKRAVQGPEETVTQ
Q6BCY4	IGETIFFRGPRGRLFYHGPGNLGIRPDQTSEPKKTLADHLG
Q8IY45	IIDLGGCLSITDVSLHALGKNCPFLQCVDFSATQVSDSGVI
O60812	DGRMIASQVVDINLAAEPKVNRGNAGVKRSAAEMYGSSFDL
Q8CGF5	FFDEATITDPLWGDPTYVNINIATASGYLISDLLIIILFNWK
P52943	DGKPFCHKPCYATLFGPKGVNIGGAGSYIYEKPLAEGPQVT
Q15848	GAYVYRSAFSVGLETYVTIPNMPIRFTKIFYNQQNHYDGST
F1MIW6	WVPWKYRMFLRDELCIKQEDNLFFEFCDVVKKAYGKCAIVV
Q99LS3	SGGFRSIVEHVAAKLNIPTTNVFANRLKFYFNGEYAGFDEM
Q8NH89	IFYGTLSYMYLQPQSNNSQENMKVASIFYGTVIPMLNPLIY
P49721	LIGIQGPDYVLVASDRVAASNIVQMKDDHDKMFKMSEKILL
Q9NZQ0	TTNSSASFTKEQADAIRRIRNSKDSWDMLGVKPGASRDEVN
P05531	RLHIKRKRMETYIKDSFKDSNVKLEQLWKTNKQERKKINNK
Q9CQL0	ANLPPHIQPKAVVKELTWGQNLESFSPGEFDLILGADVIYL
Q3T046	VHHGTILDCEETDWDFSMNLNVRSMYLMIKAFLPKMMAQKS
Q9Y6Z7	NTLIADYVAKSGFFRVFIGVNDLEREGQYMFTDNTPLQNYS
Q5E9V3	RSDKYTFENFQYETKAQINHNIAMQNEKLDGINLESGSQQA
A2VDU1	TLPSCWVCNQECLCSAQTLVNYGTCMCLVQGIFYHCTNEDD
O14818	KSVAKLQDERTVRKICALDDNVCMAFAGLTADARIVINRAR
A6NJI9	DATVKELKGMLNLKILSLYQNPLCQYNLYRLYIIYHLPGVE
A1XBS5	MRRTLENRNAQTKQLQTAVSNVEKHFGELCQIFAAYVRKTA
Q96LJ8	APPECSTVVSTAVDSLIWQPNSLNMHMIRPKSAKGRTRPSL
P13621	KELLRVGQILKEPKMAASLLNPYVKRSVKVKSLSDMTAKEK
A8MTZ7	LSEQPEKDDTPSHTQAQCCLNFGWAFSWLRQRILPSLLRRD
Q9CZE3	AFVVFDISRSSTFDAVLKWKNDLDSKVHLPNGSPIPAVLLA
Q969J5	FQSRNFHNILQWQPGRALTGNSSVYFVQYKIMFSCSMKSSH
Q969M3	QIYQPTQAYTPASPQPFYGNNFEDEPPLLEELGINFDHIWQ
Q96Q83	MEPNPHWHPVLRTLKNRIEENTGHTFNSLLCNLYRNEKDSV
Q3SZR9	CCGIHNYSDWENTDWFKETKNQSVPLSCCRETASSCNGSLA
Q6ZWI9	HFREDVTLDAATASSLLVFSNDLRSAQCKKIHHDLTKDPRL
P14415	AGANQSMNVTCAGKRDEDAENLGNFVMFPANGNIDLMYFPY
P09611	LVVSNLLLCQGVEDYAPYCKNQPGNCRIPLQSLFERATLVA

P0C591	PGAFELERLFWKGSPQYTHVNEVWPKLYIGDETTALDRYGL
Q91XQ6	QEYIRQLPPSFPYRDDIMSVNPTCLVLIILLFIGILLTLKG
Q01105	DHSDAGADELGEVIKDDIWPNPLQYYLVPDMDDEEGEGEED
Q8C8S3	ASFGMTLLGNFQLTNDEEIHNVGTSLTFGFGTLTCWIQAAL
Q91Z38	IEERNERLKEEMLGKLKDLGNLVLRPFGLSTENFQIKQDSS
P05937	PVQENFLLKFQGIKMCGKEFNKAFELYDQDGNGYIDENELD
Q9H8W3	STTSRFRGKRRKRSRKDKLKNEKELHSEPSSNETQWKELTQ
Q32KL2	SRATAGAYIASQTVKKVIEINPYLLGTMAGGAADCSFWERL
P20151	EPEDTGQRVPVSHSFPHPLYNMSLLKHQSLRPDEDSSHDLM
Q9QXF8	SFAHLPDCKGDQSEHRLALKNIASMVRPGGLLVIDHRNYDY
Q9D7P7	LEETLGPPDFPSLAPRYRESNTAGNDIFHKFSAFIKNPVPT
Q05716	EIEAIQESLQPSDKDEGDHPNNSFSPCSAHDRKCLQKHLAK
A7MBE4	EAVDMLDGLVDESDPDVDFPNSFHAFQTAEGIRKAHPDKDW
P15947	SGWGSITPVKYEYPDELQCVNLKLLPNEDCAKAHIEKVTDD
P70660	STSGLSVPARRSAPALSGASNVPGAQDEEQERRRRRGRARV
P00492	LKGGYKFFADLLDYIKALNRNSDRSIPMTVDFIRLKSYCND
Q96S79	QQILETRVIGTSETPIIIVGNKRDLQRGRVIPRWNVSHLVR
P08074	AMTMLTKAMAMELGPHKIRVNSVNPTVVLTDMGKKVSADPE
Q3KP22	IENKEVITQELEDSVRVVLGNLDNLQPFATEHFIVFPYKSK
Q8BZJ7	RQFMACTQASERTAIYCLTQNEWKLDEATDSFFQNPEAFHR
P16110	NDALAGSGNPNPQGYPGAWGNQPGAGGYPGAAYPGAYPGQA
P01732	WLFQPRGAAASPTFLLYLSQNKPKAAEGLDTQRFSGKRLGD
Q9D0U1	CSFQLQHGAQIRLSKEYLLRNGLYPKPMPKNKRKLRKMELL
Q8TC29	DKRTGDKHDLEPSGLVPKYINKKDYGVTPEYICKRNEEIKK
Q9ERI2	EEARELAEKYGIPYFETSAANGTNISHAIEMLLDLIMKRME
Q2YDE4	TVLSIDFKPSEIEVGVVTVENPKFRILTEAEIDAHLVALAE
P00755	SGWGSIIPVKFQYAKDLQCVNLKLLPNEDCDKAYVQKVTDV
