# Supplementary material for: Prediction of N-linked glycosylation sites using position relative features and statistical moments
Source: PLoS One. 2017 Aug 10;12(8):e0181966. doi: 10.1371/journal.pone.0181966 (PMC5552137; doi:10.1371/journal.pone.0181966)
Supplement: S2 File — This file contain positive n-linked sites of glycosylation along with the accession number. (PDF) [file pone.0181966.s002.pdf]

# Supplementary Data Set (S2) of Positive N-Linked Site

| Accession No | List of Sequences for Positive N-Linked Sites |
|---|---|
| P30443 | NMKAHSQTDRANLGTLRGYYNQSEDGSHTIQIMYGCDVGPD |
| P01892 | KVKAHSQTHRVDLGTLRGYYNQSEAGSHTVQRMYGCDVGSD |
| P05534 | KVKAHSQTDRENLRIALRYYNQSEAGSHTLQMMFGCDVGSD |
| P01891 | NVKAQSQTDRVDLGTLRGYYNQSEAGSHTIQMMYGCDVGSD |
| P01889 | IYKAQAQTDRESLRNLRGYYNQSEAGSHTLQSMYGCDVGPD |
| P03989 | ICKAKAQTDREDLRTLLRYYNQSEAGSHTLQNMYGCDVGPD |
| Q04826 | ISKTNTQTYRESLRNLRGYYNQSEAGSHTLQSMYGCDVGPD |
| P30501 | KYNRQAQTDRVNLRKLRGYYNQSEAGSHTLQRMYGCDLGPD |
| Q07000 | NYKRQAQTDRVNLRKLRGYYNQSEAGSHIIQRMYGCDLGPD |
| Q95604 | KYKRQAQADRVNLRKLRGYYNQSEAGSHTIQRMYGCDLGPD |
| P22282 | DVFKYLYNTHSADTYLSNIKNESFTMNIWGFGEIEMVKTKC |
| P01912 | LAGDTRPRFLEYSTSECHFFNGTERVRYLDRYFHNQEENVR |
| P01911 | LSGDTRPRFLWQPKRECHFFNGTERVRFLDRYFYNQEESVR |
| P01446 | LAYKTCPAGKNLCYKMFMVSNKTVPVKRGCIDACPKNSLLV |
| P10852 | HLEYLSTLKVKGLVLGPIHKNQKDEINETDLKQINPTLGSQ |
| P10852 | TLKVKGLVLGPIHKNQKDEINETDLKQINPTLGSQEDFKDL |
| P10852 | SSWLQDGVDGFQFRDVGKLMNAPLYLAEWQNITKNLSEDRL |
| P10852 | FQFRDVGKLMNAPLYLAEWQNITKNLSEDRLLIAGTESSDL |
| P10852 | DVGKLMNAPLYLAEWQNITKNLSEDRLLIAGTESSDLQQIV |
| P10852 | QIVNILESTSDLLLTSSYLSNSTFTGERTESLVTRFLNATG |
| P10852 | YLSNSTFTGERTESLVTRFLNATGSQWCSWSVSQAGLLADF |
| P10852 | ELGLQGALPGQPAKAPLMPWNESSIFHIPRPVSLNMTVKGQ |
| P10852 | APLMPWNESSIFHIPRPVSLNMTVKGQNEDPGSLLTQFRRL |
| P08195 | FQVRDIENLKDASSFLAEWQNITKGFSEDRLLIAGTNSSDL |
| P08195 | AEWQNITKGFSEDRLLIAGTNSSDLQQILSLLESNKDLLLT |
| P08195 | YLSDSGSTGEHTKSLVTQYLNATGNRWCSWSLSQARLLTSF |
| P08195 | APVMLWDESSFPDIPGAVSANMTVKGQSEDPGSLLSLFRRL |
| O95264 | LYHLSKQLLQKYHKEVRPVYNWTKATTVYLDLFVHAILDVD |
| O95264 | QILKTSVWYQEVWNDEFLSWNSSMFDEIREISLPLSAIWAP |
| O95264 | IIINEFVDIERYPDLPYVYVNSSGTIENYKPIQVVSACSLE |
| O95264 | PIQVVSACSLETYAFPFDVQNCSLTFKSILHTVEDVDLAFL |
| O95264 | VDLAFLRSPEDIQHDKKAFLNDSEWELLSVSSTYSILQSSA |
| O95264 | CRARIVFKTSVLVGYTVFRVNMSNQVPRSVGSTPLIGHFFT |
| P34969 | PHLLSEVTASPAPTWDAPPDNASGCGEQINYGRVEKVVIGS |
| P21589 | HTNDVHSRLEQTSEDSSKCVNASRCMGGVARLFTKVQQIRR |
| P21589 | KIEFDERGNVISSHGNPILLNSSIPEDPSIKADINKWRIKL |
| P21589 | SIPEDPSIKADINKWRIKLDNYSTQELGKTIVYLDGSSQSC |

| | |
|---|---|
| P21589 | HVSMCILNGGGIRSPIDERNNGTITWENLAAVLPFGGTFDL |
| Q61503 | HTNDVHSRLEQTSDDSTKCLNASLCVGGVARLFTKVQQIRK |
| Q61503 | KVEFDDKGNVITSYGNPILLNSSIPEDATIKADINQWRIKL |
| Q61503 | SIPEDATIKADINQWRIKLDNYSTQELGRTIVYLDGSTQTC |
| Q61503 | HVSMCIVNGGGIRSPIDEKNNGTITWENLAAVLPFGGTFDL |
| P02763 | LPLLEAQIPLCANLVPVPITNATLDQITGKWFYIASAFRNE |
| P02763 | LDQITGKWFYIASAFRNEEYNKSVQEIQATFFYFTPNKTED |
| P02763 | NEEYNKSVQEIQATFFYFTPNKTEDTIFLREYQTRQDQCIY |
| P02763 | KTEDTIFLREYQTRQDQCIYNTTYLNVQRENGTISRYVGGQ |
| P02763 | YQTRQDQCIYNTTYLNVQRENGTISRYVGGQEHFAHLLILR |
| Q60590 | TVLIILSLLPMLEAQNPEHANFTIGEPITNETLSWLSDKWF |
| Q60590 | PMLEAQNPEHANFTIGEPITNETLSWLSDKWFFMGAAFRKL |
| Q60590 | YRQAIQTMQSEFFYLTTNLINDTIELRESQTIGDQCVYNST |
| Q60590 | LINDTIELRESQTIGDQCVYNSTHLGFQRENGTFSKYEGGV |
| Q60590 | SQTIGDQCVYNSTHLGFQRENGTFSKYEGGVETFAHLIVLR |
| P19652 | LPLLEAQIPLCANLVPVPITNATLDRITGKWFYIASAFRNE |
| P19652 | LDRITGKWFYIASAFRNEEYNKSVQEIQATFFYFTPNKTED |
| P19652 | NEEYNKSVQEIQATFFYFTPNKTEDTIFLREYQTRQNQCFY |
| P19652 | KTEDTIFLREYQTRQNQCFYNSSYLNVQRENGTVSRYEGGR |
| P19652 | YQTRQNQCFYNSSYLNVQRENGTVSRYEGGREHVAHLLFLR |
| P07361 | MILVMVSLLPLLEAQNPEHVNITIGDPITNETLSWLSDKWF |
| P07361 | PLLEAQNPEHVNITIGDPITNETLSWLSDKWFFIGAAVLNP |
| P07361 | YRQEIQKTQMVFFNLTPNLINDTMELREYHTIDDHCVYNST |
| P07361 | LINDTMELREYHTIDDHCVYNSTHLGIQRENGTLSKYVGGV |
| P07361 | YHTIDDHCVYNSTHLGIQRENGTLSKYVGGVKIFADLIVLK |
| P07758 | IATNLGDFAISLYRELVHQSNTSNIFFSPVSIATAFAMLSL |
| P07758 | MLSLGSKGDTHTQILEGLQFNLTQTSEADIHKSFQHLLQTL |
| P07758 | LHVHHCSTLSSWVLLMDYAGNATAVFLLPDDGKMQHLEQTL |
| P22599 | IATNLGDFAISLYRELVHQSNTSNIFFSPVSIATAFAMLSL |
| P22599 | MLSLGSKGDTHTQILEGLQFNLTQTSEADIHKSFQHLLQTL |
| P22599 | LDVHHCSILSSWVLLMDYAGNASAVFLLPEDGKMQHLEQTL |
| Q00896 | IATNLGDFAISLYRELVHQSNTSNIFFSPVSIATAFAMLSL |
| Q00896 | MLSLGSKGDTHTQILEGLQFNLTQTSEADIHKSFQHLLQTL |
| Q00896 | LDVHHCSTLSSWVLLMDYAGNATAVFLLPDDGKMQHLEQTL |
| Q00898 | IATNLGDFAISLYRELVHQSNTSNIFFSPVSIATAFAMLSL |
| Q00898 | MLSLGSKGDTHTQILEGLQFNLTQTSEADIHNSFQHLLQTL |
| Q00898 | LDVHHCSTLSSWVLLMDYAGNATAVFLLPDDGKMQHLEQTL |
| P01009 | ITPNLAEFAFSLYRQLAHQSNSTNIFFSPVSIATAFAMLSL |
| P01009 | MLSLGTKADTHDEILEGLNFNLTEIPEAQIHEGFQELLRTL |
| P01009 | FNIQHCKKLSSWVLLMKYLGNATAIFFLPDEGKLQHLENEL |
| P04217 | FYETQPSLWAESESLLKPLANVTLTCQAHLETPDFQLFKNG |

| | |
|---|---|
| P04217 | LEVPEAQEDVEATFPVHQPGNYSCSYRTDGEGALSEPSATV |
| P04217 | GRRVHRFQSPAGTEALFELHNISVADSANYSCVYVDLKPPF |
| P04217 | SPAGTEALFELHNISVADSANYSCVYVDLKPPFGGSAPSER |
| Q19LI2 | DSGSEPKLWAEPQSLLEPWANLTLVCAVDLPTKVFELIQNG |
| Q19LI2 | QVRLETQVLSYRFSLGAITSNNSGIYRCRCGVEPPVDIHLP |
| Q19LI2 | VQKPDVQHKGTAGFLIYKPGNYSCSYLTHAAGEPSEPSDIV |
| Q19LI2 | GLRFGLQRQGKPELVVVQMLNSSGTEAVFELHNISTIDSGN |
| Q19LI2 | ELVVVQMLNSSGTEAVFELHNISTIDSGNYSCIYMEQAPPF |
| Q19LI2 | NSSGTEAVFELHNISTIDSGNYSCIYMEQAPPFSGSSSSEP |
| Q19LI2 | STRSTSAYLKLLFVGPQHAGNYSCRYTALPPFTFESGISDP |
| P02750 | PAEIPGYLPADTVHLAVEFFNLTHLPANLLQGASKLQELHL |
| P02750 | LGHLDLSGNRLRKLPPGLLANFTLLRTLDLGENQLETLPPD |
| P02750 | ARVAAGAFQGLRQLDMLDLSNNSLASVPEGLWASLGQPNWD |
| P02750 | QNLSDLYRWLQAQKDKMFSQNDTRCAGPEAVKGQTLLAVAK |
| P08697 | LILSPLSVALALSHLALGAQNHTLQRLQQVLHAGSGPCLPH |
| P08697 | LRWFLLEQPEIQVAHFPFKNNMSFVVLVPTHFEWNVSQVLA |
| P08697 | HFPFKNNMSFVVLVPTHFEWNVSQVLANLSWDTLHPPLVWE |
| P08697 | MSFVVLVPTHFEWNVSQVLANLSWDTLHPPLVWERPTKVRL |
| Q7SIH1 | VPSLLHTETPEKGCLLLSHLNETVTVSASLESVRENRSLFT |
| Q7SIH1 | LLSHLNETVTVSASLESVRENRSLFTDVVAEKDLFHCVSFT |
| Q7SIH1 | CGNGRQTVSWAVTPKSLGNVNFTVSAEAVESQELCGSEVPV |
| Q7SIH1 | GCGEQNMARFAPNIYVLDYLNETQQLTAELKSKAILYLNTG |
| Q7SIH1 | VKMVSGFIPLKPTVKMLERSNVSRTEVSNNHVLIYLDKVTN |
| Q7SIH1 | NVSRTEVSNNHVLIYLDKVTNETLTLTFTVLQDIPVRDLKP |
| P28800 | LILSPLSVALALSHLALGAQNQTLQRLKEVLHADSGPCLPH |
| P28800 | VLLLLNAIHFQGFWRSKFDPNLTQRGAFHLDEQFTVPVDMM |
| P28800 | LHWFLLEQPEIQVAHFPFKNNMSFVVLMPTRFEWNASQVLA |
| P28800 | HFPFKNNMSFVVLMPTRFEWNASQVLANLTWDILHQPSLSE |
| P28800 | MSFVVLMPTRFEWNASQVLANLTWDILHQPSLSERPTKVQL |
| P01023 | VPSLLHTETTEKGCVLLSYLNETVTVSASLESVRGNRSLFT |
| P01023 | LLSYLNETVTVSASLESVRGNRSLFTDLEAENDVLHCVAFA |
| P01023 | PKFEVQVTVPKIITILEEEMNVSVCGLYTYGKPVPGHVTVS |
| P01023 | GVPIPNKVIFIRGNEANYYSNATTDEHGLVQFSINTTNVMG |
| P01023 | EANYYSNATTDEHGLVQFSINTTNVMGTSLTVRVNYKDRSP |
| P01023 | CANGRQTVSWAVTPKSLGNVNFTVSAEALESQELCGTEVPS |
| P01023 | GCGEQNMVLFAPNIYVLDYLNETQQLTPEIKSKAIGYLNTG |
| P01023 | HVSRTEVSSNHVLIYLDKVSNQTLSLFFTVLQDVPVRDLKP |
| Q61838 | VPSEVYSGVPEKACVSLNHVNETVMLSLTLEYAMQQTKLLT |
| Q61838 | KPGQIVKFRVVSVDISFRPLNETFPVVYIETPKRNRIFQWQ |
| Q61838 | LPFSGQVLLVDEKGKPIPNKNITSVVSPLGYLSIFTTDEHG |
| Q61838 | SVVSPLGYLSIFTTDEHGLANISIDTSNFTAPFLRVVVTYK |

| | |
|---|---|
| Q61838 | YLSIFTTDEHGLANISIDTSNFTAPFLRVVVTYKQNHVCYD |
| Q61838 | EELVADAQNFEIEKCFANKVNLSFPSAQSLPASDTHLKVKA |
| Q61838 | CGNERKTVSWAVTPKSLGEVNFTATAEALQSPELCGNKLTE |
| Q61838 | IEKEQTYNTLLCPQDTELQDNWSLELPPNVVEGSARATHSV |
| Q61838 | GCGEQNMVLFVPNIYVLNYLNETQQLTEAIKSKAINYLISG |
| Q61838 | NTLPLNFDKAGDHRTFQIRINVSYTGERPSSNMVIVDVKMV |
| Q61838 | NIQRTEVNTNHVLIYIEKLTNQTLGFSFAVEQDIPVKNLKP |
| P26671 | IYNVVNIKYNPHIPYRYNFINRTLTVDELDDNVFFTHGYFL |
| P26671 | PSLIVSLSGNLKYNDIQCSVNVSCLIKNLATSTSTILTSKH |
| Q10651 | TCFSGKLDILKYCRKAYPSMNITNIVEYSHEVSISDWCREE |
| Q10651 | PCALDMFTGVEFVCCPNDQTNKTDVQKTKEDEDDDDDEDDA |
| Q10651 | ESDEKDEEEPSSQDPYFKIANWTNEHDDFKKAEMRMDEKHR |
| Q10651 | EAAAYKPTVIHRLRYIDLRINGTLAMLRDFPDLEKYVRPIA |
| P05067 | KAAQIRSQVMTHLRVIYERMNQSLSLLYNVPAVAEEIQDEV |
| P05067 | VPAVAEEIQDEVDELLQKEQNYSDDVLANMISEPRISYGND |
| P08592 | KAAQIRSQVMTHLRVIYERMNQSLSLLYNVPAVAEEIQDEV |
| P08592 | VPAVAEEIQDEVDELLQKEQNYSDDVLANMISEPRISYGND |
| P22760 | FMDSFKVVGSFDEVPPTSDENVTVTETKFNNILVRVYVPKR |
| P22760 | KAMLSRQHVPVESSHLFKFVNWSSLLPERFIKGHVYNNPNY |
| Q15758 | SALGVGLALALQPGAASAAINASVGAAGSAENAPSKEVLDS |
| Q15758 | FPSNLVSAAFRSYSTTYEERNITGTRVKVPVGQEVEGMNIL |
| P01011 | LAAGFCPAVLCHPNSPLDEENLTQENQDRGTHVDLGLASAN |
| P01011 | VIFSPLSISTALAFLSLGAHNTTLTEILKGLKFNLTETSEA |
| P01011 | FLSLGAHNTTLTEILKGLKFNLTETSEAEIHQSFQHLLRTL |
| P01011 | LTETSEAEIHQSFQHLLRTLNQSSDELQLSMGNAMFVKEQL |
| P01011 | FATDFQDSAAAKKLINDYVKNGTRGKITDLIKDLDSQTMMV |
| P01011 | IPYFRDEELSCTVVELKYTGNASALFILPDQDKMEEVEAML |
| O95477 | NNPCFRYPTPGEAPGVVGNFNKSIVARLFSDARRLLLYSQK |
| O95477 | TLQQIKKSSSNLKLQDFLVDNETFSGFLYHNLSLPKSTVDK |
| O95477 | NLKLQDFLVDNETFSGFLYHNLSLPKSTVDKMLRADVILHK |
| O95477 | DVILHKVFLQGYQLHLTSLCNGSKSEEMIQLGDQEVSELCG |
| O95477 | AAERVLRSNMDILKPILRTLNSTSPFPSKELAEATKTLLHS |
| O95477 | LFSMRSWSDMRQEVMFLTNVNSSSSSTQIYQAVSRIVCGHP |
| O95477 | LKIKSLNWYEDNNYKALFGGNGTEEDAETFYDNSTTPYCND |
| O95477 | NYKALFGGNGTEEDAETFYDNSTTPYCNDLMKNLESSPLSR |
| O95477 | VGKILYTPDTPATRQVMAEVNKTFQELAVFHDLEGMWEELS |
| O95477 | WTAQDIVAFLAKHPEDVQSSNGSVYTWREAFNETNQAIRTI |
| O95477 | KHPEDVQSSNGSVYTWREAFNETNQAIRTISRFMECVNLNK |
| O95477 | FMECVNLNKLEPIATEVWLINKSMELLDERKFWAGIVFTGI |
| O95477 | QGIGVQWDNLFESPVEEDGFNLTTSVSMMLFDTFLYGVMTW |
| O95477 | TGYYLTLVKKDVESSLSSCRNSSSTVSYLKKEDSVSQSSSD |

| | |
|---|---|
| O95477 | AFGDKQSCLRPFTEDDAADPNDSDIDPESRETDLLSGMDGK |
| O95477 | EEEWTTAPVPQTIMDLFQNGNWTMQNPSPACQCSSDKIKKM |
| O95477 | LPPPQRKQNTADILQDLTGRNISDYLVKTYVQIIAKSLKNK |
| O95477 | NLQKGENPSHYGITAFNHPLNLTKQQLSEVALMTTSVDVLV |
| O95477 | VGEWAIRKLGLVKYGEKYAGNYSGGNKRKLSTAMALIGGPP |
| O95477 | VNFAKDQSDDDHLKDLSLHKNQTVVDVAVLTSFLQDEKVKE |
| P41233 | NNPCFRYPTPGEAPGVVGNFNKSIVSRLFSDAQRLLLYSQR |
| P41233 | MLRQIKHPNSNLKLQDFLVDNETFSGFLQHNLSLPRSTVDS |
| P41233 | NLKLQDFLVDNETFSGFLQHNLSLPRSTVDSLLQANVGLQK |
| P41233 | NVGLQKVFLQGYQLHLASLCNGSKLEEIIQLGDAEVSALCG |
| P41233 | AAERVLRYNMDILKPVVTKLNSTSHLPTQHLAEATTVLLDS |
| P41233 | LFSTKSWSDMRQEVMFLTNVNSSSSSTQIYQAVSRIVCGHP |
| P41233 | LKIKSLNWYEDNNYKALFGGNNTEEDVDTFYDNSTTPYCND |
| P41233 | NYKALFGGNNTEEDVDTFYDNSTTPYCNDLMKNLESSPLSR |
| P41233 | VGKILYTPDTPATRQVMAEVNKTFQELAVFHDLEGMWEELS |
| P41233 | WTAQDIMAFLAKNPEDVQSPNGSVYTWREAFNETNQAIQTI |
| P41233 | KNPEDVQSPNGSVYTWREAFNETNQAIQTISRFMECVNLNK |
| P41233 | FMECVNLNKLEPIPTEVRLINKSMELLDERKFWAGIVFTGI |
| P41233 | QGIGVQWDNLFESPVEEDGFNLTTAVSMMLFDTFLYGVMTW |
| P41233 | TGYYLTLVKKDVESSLSSCRNSSSTVSCLKKEDSVSQSSSD |
| P41233 | AFGDKQSCLHPFTEDDAVDPNDSDIDPESRETDLLSGMDGK |
| P41233 | EEDWTISPVPQSIVDLFQNGNWTMKNPSPACQCSSDKIKKM |
| P41233 | PGAGGLPPPQRKQKTADILQNLTGRNISDYLVKTYVQIIAK |
| P41233 | LPPPQRKQKTADILQNLTGRNISDYLVKTYVQIIAKSLKNK |
| P41233 | NLQKGENPSQYGITAFNHPLNLTKQQLSEVALMTTSVDVLV |
| P41233 | VGEWAIRKLGLVKYGEKYASNYSGGNKRKLSTAMALIGGPP |
| P41233 | VNFAKDQSDDDHLKDLSLHKNQTVVDVAVLTSFLQDEKVKE |
| P78363 | NNPCFQSPTPGESPGIVSNYNNSILARVYRDFQELLMNAPE |
| P78363 | MGKILYTPDSPAARRILKNANSTFEELEHVRKLVKAWEEVG |
| P78363 | VRKLVKAWEEVGPQIWYFFDNSTQMNMIRDTLGNPTVKDFL |
| P78363 | KGPRESQADDMANFDWRDIFNITDRTLRLVNQYLECLVLDK |
| P78363 | WLPEYPCGNSTPWKTPSVSPNITQLFQKQKWTQVNPSPSCR |
| P78363 | LPPPQRTQRSTEILQDLTDRNISDFLVKTYPALIRSSLKSK |
| P78363 | VVPITGEALVGFLSDLGRIMNVSGGPITREASKEIPDFLKH |
| P78363 | SLPKDRSPEEYGITVISQPLNLTKEQLSEITVLTTSVDAVV |
| Q8N139 | LGRVDKFNSSSLMVVYTPISNLTQQIMNKTALAPLLKGTSV |
| Q8N139 | IMNKTALAPLLKGTSVIGAPNKTHMDEILLENLPYAMGIIF |
| Q8N139 | FIKSLKHQNILLEVDDFENRNGTDGLSYNGAIIVSGKQKDY |
| Q8IUA7 | LKGRTIMGWPDEKSMDELDLNYSIDAVRVIFTDTFSYHLKF |
| Q8IUA7 | EKGFVAFQAAINAAIIEIATNHSVMEQLMSVTGVHMKILPF |
| Q8IUA7 | FLHSLRRQNIAIEVDAFGTRNGTDDPSYNGAIIVSGDEKDH |

| | |
|---|---|
| O94911 | LGRVDTFNESRFSVVYTPVTNTTQQIMNKVASTPFLAGKEV |
| O94911 | DLYLFSCIISFSSFIYYASVNVTRERKRMKALMTMMGLRDS |
| O94911 | HDSFEQAPPEFQGKEAIRIRNVTKEYKGKPDKIEALKDLVF |
| O94911 | PTKGSVTIYNNKLSEMADLENLSKLTGVCPQSNVQFDFLTV |
| O94911 | FIQSVEHQNIALEVDAFGTRNGTDDPSYNGAITVCCNEKNY |
| O95342 | VFIDYDVELQELQIPGKACVNNTIVWTNSSLNQNMTNGTRC |
| O95342 | ELQELQIPGKACVNNTIVWTNSSLNQNMTNGTRCGLLNIES |
| O95342 | IPGKACVNNTIVWTNSSLNQNMTNGTRCGLLNIESEMIKFA |
| O95342 | KACVNNTIVWTNSSLNQNMTNGTRCGLLNIESEMIKFASYY |
| Q09427 | IDYWLAKWTDSALVLSPAARNCSLSQECDLDQSVYAMVFTL |
| Q09429 | IDYWLAKWTDSALVLSPAARNCSLSQECALDQSVYAMVFTV |
| Q9UNQ0 | YGFTALQHNEFLGQNFCPGLNATGNNPCNYATCTGEEYLVK |
| U6A629 | LPLLASYASAVTISVAKSGGNVTTGLQYGAMEEEINHCGEG |
| U6A629 | TYPSNLDAWSAVGGSTLSLQNLSNPLSSALPTSVRITGKGT |
| U6A629 | ANDWVQHEFTLTPKKKASSSNNTFVLTFDASKASGGSLDFN |
| U6A629 | LRFPGGNNLEGQTIEGRWKWNETIGPLTQRPGRATTWGYEE |
| U6A629 | AKYGHPKPWTIRYVEVGNEDNLSSGLSSYKSYRFQAFYDAI |
| U6A629 | KSTSWHVYNNHMTNTLPATSNDTFGPPLLCHRCETAKTNSH |
| U6A629 | LCHRCETAKTNSHIFKAAVYNSTADVPVSLTFEGVGRGTTA |
| Q8NK89 | ISPLTAGGIADASAQDTFCANTTCLITIIYDQSGNGNHLTQ |
| Q8NK89 | NAETSSTDTGAGHMEAIYLGNSTTWGYGAGDGPWIMVDMEN |
| U6BLZ9 | KRGSDGATTDIAPRHAGGVANATHQDTFCAGTTCLITIIYD |
| U6BLZ9 | PNKWAIRGGNAASGPLSTFYNGSRPNARGYNPMSKEGAIIL |
| U6BLZ9 | GSSISLRATTLCCTTRYVAHNGSTVNTQVVSSSSSAALKQQ |
| P42254 | LLSLVQNAHGISLKVSTQGGNSSSPILYGFMFEDINHSGDG |
| P42254 | STQGGNSSSPILYGFMFEDINHSGDGGIYGQMLQNPGLQGT |
| P42254 | GDGGIYGQMLQNPGLQGTAPNLTAWAAVGDATIAIDGDSPL |
| P42254 | HSSFWIKGDYSGDITVRLVGNYTGTEYGSTTITHTSTADNF |
| P42254 | GNYTGTEYGSTTITHTSTADNFTQASVKFPTTKAPDGNVLY |
| P42254 | LRFPGGNNLEGNSAENRWKWNETIGDLCDRPGREGTWTYYN |
| P42254 | GDTSTTYGAWRAANGQEEPWNLTMVEIGNEDMLGGGCESYA |
| P42254 | YHDYSTPDGLVGQFNYFDNLNRSVPYFIGEYSRWEIDWPNM |
| P42254 | FERNSDVVKMAAYAPLLQLINSTQWTPDLIGYTQSPGDIFL |
| P13689 | LMGSSSLKYPGQPQEIPFFQNTTFSIPVNDPHQVWNSDEHE |
| P11140 | VMIYDCTSAVAEATYWEIWDNGTIINPKSALVLSAESSSMG |
| P11140 | GGTLTVQTNEYLMRQGWRTGNNTSPFVTSISGYSDLCMQAQ |
| P38433 | PVGNLRFKKPKPKQPWRIPLNATTPPNSCIQSEDTYFGDFY |
| P38433 | EPRDVALARAVILYNAMKCGNMSLINPDYDRILDCFQRADA |
| P38433 | VLHGYEINFIFGEPLNQKRFNYTDEERELSNRFMRYWANFA |
| P38433 | DGSFTQDVWPKYNSVSMEYMNMTVESSYPSMKRIGHGPRRK |
| Q9BYF1 | NHEAEDLFYQSSLASWNYNTNITEENVQNMNNAGDKWSAFL |

| | |
|---|---|
| Q9BYF1 | SAFLKEQSTLAQMYPLQEIQNLTVKLQLQALQQNGSSVLSE |
| Q9BYF1 | YPLQEIQNLTVKLQLQALQQNGSSVLSEDKSKRLNTILNTM |
| Q9BYF1 | WDAQRIFKEAEKFFVSVGLPNMTQGFWENSMLTDPGNVQKA |
| Q9BYF1 | AATPKHLKSIGLLSPDFQEDNETEINFLLKQALTIVGTLPF |
| Q9BYF1 | QEALCQAAKHEGPLHKCDISNSTEAGQKLFNMLRLGKSEPW |
| Q9BYF1 | VRVANLKPRISFNFFVTAPKNVSDIIPRTEVEKAIRMSRSR |
| P07140 | QERYEYFPGFSGEEIWNPNTNVSEDCLYINVWAPAKARLRH |
| P07140 | EHPNGKQADTDHLIHNGNPQNTTNGLPILIWIYGGGFMTGS |
| P07140 | HMTSEKAVEIGKALINDCNCNASMLKTNPAHVMSCMRSVDA |
| P07140 | GEWMGVLHGDEIEYFFGQPLNNSLQYRPVERELGKRMLSAV |
| P21836 | RRRATLLARLVGCPPGGAGGNDTELIACLRTRPAQDLVDHE |
| P21836 | VVKDEGSYFLVYGVPGFSKDNESLISRAQFLAGVRIGVPQA |
| P21836 | GVPHGYEIEFIFGLPLDPSLNYTTEERIFAQRLMKYWTNFA |
| P04058 | PVGNMRFRRPEPKKPWSGVWNASTYPNNCQQYVDEQFPGFS |
| P04058 | GDHNVICPLMHFVNKYTKFGNGTYLYFFNHRASNLVWPEWM |
| P04058 | GVIHGYEIEFVFGLPLVKELNYTAEEEALSRRIMHYWATFA |
| P04058 | HQRLRVQMCVFWNQFLPKLLNATACDGELSSSGTSSSKGII |
| P12821 | LLLLLPPQPALALDPGLQPGNFSADEAGAQLFAQSYNSSAE |
| P12821 | LQPGNFSADEAGAQLFAQSYNSSAEQVLFQSVAASWAHDTN |
| P12821 | NSSAEQVLFQSVAASWAHDTNITAENARRQEEAALLSQEFA |
| P12821 | QEFAEAWGQKAKELYEPIWQNFTDPQLRRIIGAVRTLGSAN |
| P12821 | TLGSANLPLAKRQQYNALLSNMSRIYSTAKVCLPNKTATCW |
| P12821 | YNALLSNMSRIYSTAKVCLPNKTATCWSLDPDLTNILASSR |
| P12821 | VPFPDKPNLDVTSTMLQQGWNATHMFRVAEEFFTSLELSPM |
| P12821 | ALSVSTPEHLHKIGLLDRVTNDTESDINYLLKMALEKIAFL |
| P12821 | NFDWWYLRTKYQGICPPVTRNETHFDAGAKFHVPNVTPYIR |
| P12821 | DRTSQVVWNEYAEANWNYNTNITTETSKILLQKNMQIANHT |
| P12821 | NTNITTETSKILLQKNMQIANHTLKYGTQARKFDVNQLQNT |
| P12821 | ANHTLKYGTQARKFDVNQLQNTTIKRIIKKVQDLERAALPA |
| P12821 | YNKILLDMETTYSVATVCHPNGSCLQLEPDLTNVMATSRKY |
| P12821 | KEADDFFTSLGLLPVPPEFWNKSMLEKPTDGREVVCHASAW |
| P12821 | MKLGFSRPWPEAMQLITGQPNMSASAMLSYFKPLLDWLRTE |
| P09470 | LQPGNFSPDEAGAQLFAESYNSSAEVVMFQSTVASWAHDTN |
| P09470 | NSSAEVVMFQSTVASWAHDTNITEENARRQEEAALVSQEFA |
| P09470 | QEFAEVWGKKAKELYESIWQNFTDSKLRRIIGSIRTLGPAN |
| P09470 | TLGPANLPLAQRQQYNSLLSNMSRIYSTGKVCFPNKTATCW |
| P09470 | YNSLLSNMSRIYSTGKVCFPNKTATCWSLDPELTNILASSR |
| P09470 | VPFPDKPNLDVTSTMVQKGWNATHMFRVSEEFFTSLGLSPM |
| P09470 | NFDWWYLRTKYQGICPPVARNETHFDAGAKFHIPNVTPYIR |
| P09470 | DRTAQVLLNEYAEANWQYNTNITIEGSKILLEKSTEVSNHT |
| P09470 | NTNITIEGSKILLEKSTEVSNHTLKYGTRAKTFDVSNFQNS |

| | |
|---|---|
| P09470 | SNHTLKYGTRAKTFDVSNFQNSSIKRIIKKLQNLDRAVLPP |
| P09470 | YNQILLDMETTYSLSNICYTNGTCMPLEPDLTNMMATSRKY |
| P09470 | KEADNFFTSLGLLPVPPEFWNKSMLEKPTDGREVVCHPSAW |
| P09470 | MKLGYSKPWPEAMKLITGQPNMSASAMMNYFKPLTEWLVTE |
| P54244 | IRTIIILFCVISVTTTSQSLNATLKTFDPRLLNSTADRDIA |
| P54244 | VTTTSQSLNATLKTFDPRLLNSTADRDIAMKNVPLVRLTRH |
| P54244 | MIQKWRDEYLDWNPNEYGMINSTIIPFHHLWIPDTYLYNSV |
| P54244 | PAIYTITCRLNIRFFPYDRQNCTLTISSWTNSKSALDYYAD |
| Q9N587 | YNNADGNYQVTIMTKAKLTYNGTVEWAPPAIYKSMCQIDVE |
| Q27218 | GCGAGGTWLGFLVFLAVSLRNHSTCEDIDAEDRLMVDLFRG |
| Q27218 | EDRLMVDLFRGYNSLVQPVRNRSELPMIVKIGMQLVLLINV |
| Q27218 | VWLTMKWDDFQLKWDPRDYANITQIRVAPEKVWLPDIVLFN |
| P04756 | PAIFKSYCEIIVTHFPFDEQNCSMKLGTWTYDGSVVAINPE |
| P02710 | PAIFKSYCEIIVTHFPFDQQNCTMKLGIWTYDGTKVSISPE |
| P02712 | SAIYRSSCTIKVMYFPFDWQNCTMVFKSYTYDTSEVTLQHA |
| P02718 | ETLTSNVWMDHAWYDHRLTWNASEYSDISILRLPPELVWIP |
| P02718 | PAIFRSSCPINVLYFPFDWQNCSLKFTALNYDANEITMDLM |
| P02718 | GEWEIIHKPAKKNIYPDKFPNGTNYQDVTFYLIIRRKPLFY |
| Q16570 | STENSSQLDFEDVWNSSYGVNDSFPDGDYGANLEAAAPCHS |
| Q9U7D5 | NADDPRFRQTHLFQMLVKVINTSAENATKTAIATSSTSTPS |
| Q9U7D5 | RFRQTHLFQMLVKVINTSAENATKTAIATSSTSTPSFVDTY |
| Q18581 | ASNKTNKGPVFANPVAQALVNSSNYWKTDNLQAPGSIKDEE |
| Q18581 | NDRWWEIRNKYEGVRSPQPYNTSNLDALIHNSVSQVHSPAT |
| Q23534 | HTQLISVHVIFRHGARAPVLNVTSEEAKSYFYRGLGQLTDE |
| Q23534 | FLLVPKLDCDFQLGEWDNYFNLTESDKKMARKNPWFVSDKA |
| P45963 | NGFTVEGSKKEAQLYRDLLTNYSYLVRPVRNPKKALTVTMK |
| P45963 | AWLKYIWTDFRLRWNPLDYENITSVRFQGEDQIWQPDILLY |
| P08001 | PRTSPTLQEARVALIDLELCNSTRWYNGRIRSTNVCAGYPR |
| Q9GL10 | RRTSPMLQEARVDLIDLGLCNSTRWYNGRIRSTNVCAGYPE |
| O14672 | SSHVKAIDTIYQTTDFSGIRNISFMVKRIRINTTADEKDPT |
| O14672 | QTTDFSGIRNISFMVKRIRINTTADEKDPTNPFRFPNIGVE |
| O14672 | ARATSGDKLNNNKFSLCSIRNISQVLEKKRNNCFVESGQPI |
| O14672 | AREGICNGFTALCPASDPKPNFTDCNRHTQVCINGQCAGSI |
| O35598 | SSHVKAIDTIYQTTDFSGIRNISFMVKRIRINTTSDEKDPT |
| O35598 | QTTDFSGIRNISFMVKRIRINTTSDEKDPTNPFRFPNIGVE |
| O35598 | ARATSGDKLNNNKFSLCSIRNISQVLEKKRNNCFVESGQPI |
| O35598 | AKEGICNGFTALCPASDPKPNFTDCNRHTQVCINGQCAGSI |
| Q9P0K1 | HGLHGMFYDGNHTYLIEPEENDTTQEDFHFHSVYKSRLFEF |
| Q9P0K1 | MGTVCREAVNDCDIRETCSGNSSQCAPNIHKMDGYSCDGVQ |
| Q9P0K1 | LGELDGEITSTLVVQQGRTLNCSGGHVKLEEDVDLGYVEDG |
| Q9P0K1 | TPCGPQMMCLEHRCLPVASFNFSTCLSSKEGTICSGNGVCS |

| | |
|---|---|
| Q9BZ11 | APGYIETHYGPDGQPVVLAPNHTDHCHYQGRVRGFPDSWVV |
| Q9BZ11 | DSWVVLCTCSGMSGLITLSRNASYYLRPWPPRGSKDFSTHE |
| Q9BZ11 | YLELYIVADHTLFLTRHRNLNHTKQRLLEVANYVDQLLRTL |
| Q9BZ11 | ALTGLEVWTERDRSRVTQDANATLWAFLQWRRGLWAQRPHD |
| Q9BZ11 | GEECDCGPGQECRDLCCFAHNCSLRPGAQCAHGDCCVRCLL |
| O15204 | LHEIVCPKKLHILHKREIKNNQTEKHGKEERYEPEVQYQMI |
| O15204 | STDEKEHAVFTSNQEEQDPANHTCGVKSTDGKQGPIRISRS |
| O15204 | QKYIDLYLVLDNAFYKNYNENLTLIRSFVFDVMNLLNVIYN |
| Q8R2Z0 | AGARQELKKSRQLFARVDSPNITTSNREGFPGSVKPPEASG |
| Q86S40 | IGFNCNVDLIVSGVRVVDALNTTCSEGKDQETLETLADLHQ |
| Q86S40 | LVGPIGPRSQALLHPSVKRTNSTRILKDELHVILEYKQGEI |
| Q8IUX7 | LSELPEPVVARFIRIYPLTWNGSLCMRLEVLGCSVAPVYSY |
| Q8IUX7 | EQVHRGIKGVVTDEQGIPIANATISVSGINHGVKTASGGDY |
| P43652 | LFFLTESLTLPTQPRDIENFNSTQKFIEDNIEYITIIAFAQ |
| P43652 | LPNNVLQEKICAMEGLPQKHNFSHCCSKVDAQRRLCFFYNK |
| P43652 | PDLSIPELLRIVQIYKDLLRNCCNTENPPGCYRYAEDKFNE |
| P43652 | RNCCNTENPPGCYRYAEDKFNETTEKSLKMVQQECKHFQNL |
| P43652 | FACVDNLADLVFGELCGVNENRTINPAVDHCCKTNFAFRRP |
| O89020 | LLPLTESLALPTKPQDVDHFNATQKFIDENTTYLAIIAFSQ |
| O89020 | TANDAIQDMLCDMEGLPQKHNFSHCCGKAGFPRRLCFFYNK |
| O89020 | VGFLPPFPTLDPEEKCQAYKNNSESFLHLYMYEVARRNPFV |
| O89020 | EDCCSRENPAGCYRHVEDKFNETTQRSLAMVQQECKQFQEL |
| O89020 | FACVDNLADLVLGELCGVNTNRTINPAVDHCCKTDFAFRRH |
| P06280 | QLANYVHSKGLKLGIYADVGNKTCAGFPGSFGYYDIDAQTF |
| P06280 | CYCDSLENLADGYKHMSLALNRTGRSIVYSCEWPLYMWPFQ |
| P06280 | GRSIVYSCEWPLYMWPFQKPNYTEIRQYCNHWRNFADIDDS |
| P06280 | LPVKRKLGFYEWTSRLRSHINPTGTVLLQLENTMQMSLKDL |
| P06750 | SGWSFTLEDNNIFPKQYPIINFTTADATVESYTNFIRAVRS |
| P06750 | TAIQESNQGAFASPIQLQRRNGSKFNVYDVSILIPIIALMV |
| P06750 | VVIYNCSTATVGATRWQIWDNRTIINPRSGLVLAATSGNSG |
| P06750 | GTKLTVQTNIYAVSQGWLPTNNTQPFVTTIVGLYGMCLQAN |
| P56526 | EYQDSDRLNIQILPTHVDSTNASWYFLSENLVPRPKASLNA |
| P56526 | TNASWYFLSENLVPRPKASLNASVSQSDLFVSWSNEPSFNF |
| P56526 | EYNLYGLGEHITQFRLQRNANLTIYPSDDGTPIDQNLYGQH |
| P56526 | LPAMQQYNTLGFHQCRWGYNNWSDLADVVANFEKFEIPLEY |
| P56526 | ESGRYYVPIVDAALYIPNPENASDAYATYDRGAADDVFLKN |
| P56526 | GVWYDMSEVSSFCVGSCGTGNLTLNPAHPSFLLPGEPGDII |
| P56526 | PSFLLPGEPGDIIYDYPEAFNITNATEAASASAGASSQAAA |
| P56526 | LLPGEPGDIIYDYPEAFNITNATEAASASAGASSQAAATAT |
| P56526 | PYVINHDQEGHDLSVHAVSPNATHVDGVEEYDVHGLYGHQG |
| P56526 | THVDGVEEYDVHGLYGHQGLNATYQGLLEVWSHKRRPFIIG |

| | |
|---|---|
| P56526 | HGEVWYDWYTQAAVDAKPGVNTTISAPLGHIPVYVRGGNIL |
| P56526 | ALTTREARQTPWALLAALGSNGTASGQLYLDDGESIYPNAT |
| P56526 | GSNGTASGQLYLDDGESIYPNATLHVDFTASRSSLRSSAQG |
| P56526 | KEPSAVTLNGQAVFPGSVTYNSTSQVLFVGGLQNLTKGGAW |
| O60242 | STLVKGVIYGSYSVSEMFPKNFTNCTWTLENPDPTKYSIYL |
| O60242 | VKGVIYGSYSVSEMFPKNFTNCTWTLENPDPTKYSIYLKFS |
| O60242 | PDPTKYSIYLKFSKKDLSCSNFSLLAYQFDHFSHEKIKDLL |
| O60242 | LLAYQFDHFSHEKIKDLLRKNHSIMQLCNSKNAFVFLQYDK |
| O60242 | PLNEQTEGCLTQELQTTQVCNLTREAKRPPKEEFGMMGDHT |
| O60242 | TCVSPYGTHCSGPLRESRVCNNTALCPVHGVWEEWSPWSLC |
| O60242 | PVDGQWQEWSSWSQCSVTCSNGTQQRSRQCTAAAHGGSECR |
| O60242 | MSMVWKRTPAGDLAFNQCPLNATGTTSRRCSLSLHGVAFWE |
| O60242 | LTQRKNFYAGDLLMSVEILRNVTDTFKRASYIPASDGVQNF |
| O60242 | VFVLGAVLYKNLDLILPTLRNYTVINSKIIVVTIRPEPKTT |
| O60242 | IRPEPKTTDSFLEIELAHLANGTLNPYCVLWDDSKTNESLG |
| O60242 | AHLANGTLNPYCVLWDDSKTNESLGTWSTQGCKTVLTDASH |
| O60242 | FCLSIISSNILILVGQTQTHNKSICTTTTAFLHFFFLASFC |
| Q8IZF2 | VATKSPTAEEYTVNIEISFENASFLDPIKAYLNSLSFPIHG |
| Q8IZF2 | ASFLDPIKAYLNSLSFPIHGNNTDQITDILSINVTTVCRPA |
| Q8IZF2 | SLSFPIHGNNTDQITDILSINVTTVCRPAGNEIWCSCETGY |
| Q8IZF2 | EDVTLNMRVRLNVGFQEDLMNTSSALYRSYKTDLETAFRKG |
| Q8IZF2 | TTPPSLELIHKANEQVVQSLNQTYKMDYNSFQAVTINESNF |
| Q8IZF2 | VQSLNQTYKMDYNSFQAVTINESNFFVTPEIIFEGDTVSLV |
| Q8IZF2 | EIIFEGDTVSLVCEKEVLSSNVSWRYEEQQLEIQNSSRFSI |
| Q8IZF2 | KEVLSSNVSWRYEEQQLEIQNSSRFSIYTALFNNMTSVSKL |
| Q8IZF2 | EQQLEIQNSSRFSIYTALFNNMTSVSKLTIHNITPGDAGEY |
| Q8IZF2 | MKVMCDNNPVSLNCCSQGNVNWSKVEWKQEGKINIPGTPET |
| Q8IZF2 | ISAYGARGSANIKVTFISVANLTITPDPISVSEGQNFSIKC |
| Q8IZF2 | FISVANLTITPDPISVSEGQNFSIKCISDVSNYDEVYWNTS |
| Q8IZF2 | GQNFSIKCISDVSNYDEVYWNTSAGIKIYQRFYTTRRYLDG |
| Q8IZF2 | RRYLDGAESVLTVKTSTREWNGTYHCIFRYKNSYSIATKDV |
| Q8IZF2 | SSLPAAKEVNKKQVCYKHNFNASSVSWCSKTVDVCCHFTNA |
| Q8IZF2 | SSVSWCSKTVDVCCHFTNAANNSVWSPSMKLNLVPGENITC |
| Q8IZF2 | NAANNSVWSPSMKLNLVPGENITCQDPVIGVGEPGKVIQKL |
| Q8IZF2 | NVILGKPVLNTWKVLQQQWTNQSSQLLHSVERFSQALQSGD |
| Q8IZF2 | QDIQENNFAESLVMTTTVSHNTTMPFRISMTFKNNSPSGGE |
| Q8IZF2 | KNNSPSGGETKCVFWNFRLANNTGGWDSSGCYVEEGDGDNV |
| Q8IZF2 | ANNTGGWDSSGCYVEEGDGDNVTCICDHLTSFSILMSPDSP |
| O00468 | FFVNPAPPYLWPAHKNELMLNSSLMRITLRNLEEVEFCVED |
| O00468 | QQRRIRLLSRGPCGSRDPCSNVTCSFGSTCARSADGLTASC |
| O00468 | PLPPVAPLHCAQTPYGCCQDNITAARGVGLAGCPSACQCNP |

| | |
|---|---|
| O00468 | CVEESGSAHCVCPMLTCPEANATKVCGSDGVTYGNECQLKT |
| Q80TR1 | MENVQCYLPDAFKIMSQRCNNRTQCVVVAGSDAFPDPCPGT |
| Q80TR1 | ASFQCLPALGLWNPRGPDLSNCTSPWVNQVAQKIKSGENAA |
| Q80TR1 | VVETVDNLLRPEALESWKDMNATEQVHTATMLLDVLEEGAF |
| Q80TR1 | GVVKVVFILYNNLGLFLSTENATVKLAGEAGTGGPGGASLV |
| Q80TR1 | LMDPVIFTVAHLEAKNHFNANCSFWNYSERSMLGYWSTQGC |
| Q80TR1 | IFTVAHLEAKNHFNANCSFWNYSERSMLGYWSTQGCRLVES |
| Q80TR1 | YSERSMLGYWSTQGCRLVESNKTHTTCACSHLTNFAVLMAH |
| O88917 | MENVQCYLPDAFKIMSQRCNNRTQCVVVAGSDAFPDPCPGT |
| O88917 | ASFQCLPALGLWNPRGPDLSNCTSPWVNQVAQKIKSGENAA |
| O88917 | VVETVDNLLRPEALESWKDMNATEQVHTATMLLDVLEEGAF |
| O88917 | GVVKVVFILYNNLGLFLSTENATVKLAGEAGTGGPGGASLV |
| O88917 | LMDPVIFTVAHLEAKNHFNANCSFWNYSERSMLGYWSTQGC |
| O88917 | IFTVAHLEAKNHFNANCSFWNYSERSMLGYWSTQGCRLVES |
| O88917 | YSERSMLGYWSTQGCRLVESNKTHTTCACSHLTNFAVLMAH |
| O95490 | MENTDCYLPDAFKIMTQRCNNRTQCIVVTGSDVFPDPCPGT |
| O95490 | ASNAFMICGVLYVVRSVYQDNESETGKNSIDYIYNTRLNRG |
| O95490 | ASYLCMISTGTWNPKGPDLSNCTSHWVNQLAQKIRSGENAA |
| O95490 | IVDTVDNLLRPEALESWKHMNSSEQAHTATMLLDTLEEGAF |
| O95490 | GLAKLVFIIYRSLGQFLSTENATIKLGADFIGRNSTIAVNS |
| O95490 | GQFLSTENATIKLGADFIGRNSTIAVNSHVISVSINKESSR |
| O95490 | LTDPVLFTLPHIDPDNYFNANCSFWNYSERTMMGYWSTQGC |
| O95490 | LFTLPHIDPDNYFNANCSFWNYSERTMMGYWSTQGCKLVDT |
| O95490 | YSERTMMGYWSTQGCKLVDTNKTRTTCACSHLTNFAILMAH |
| Q8JZZ7 | MENTDCYLPDAFKIMTQRCNNRTQCIVVTGSDVFPDPCPGT |
| Q8JZZ7 | ASYLCMASTGTWNPKGPDLSNCTSHWVNQLAQKIRSGENAA |
| Q8JZZ7 | GLAKLVFIIYRSLGQFLSTENATIKLGADLMGRNSTIAVNS |
| Q80TS3 | MENIRCYLPDAYKIMSQRCNNRTQCAVVAGPDVFPDPCPGT |
| Q80TS3 | LRTTTWNIGRSTTASLPGRRNRSTSTPSPAVEVLDDVTTHL |
| Q80TS3 | STYLCLAPDGIWDPQGPDLSNCSSPWVNHITQKLKSGETAA |
| Q80TS3 | GEIRVAFVLYNNLGPYLSTENASMKLGTEAMSTNHSVIVNS |
| Q80TS3 | GPYLSTENASMKLGTEAMSTNHSVIVNSPVITAAINKEFSN |
| Q80TS3 | ADPVVFTVKHIKQSEENFNPNCSFWSYSKRTMTGYWSTQGC |
| Q80TS3 | YSKRTMTGYWSTQGCRLLTTNKTHTTCSCNHLTNFAVLMAH |
| Q80TS3 | IHKNLCISLFVAELLFLIGINRTDQPIACAVFAALLHFFFL |
| Q80TS3 | GAIALLCLLGLTWAFGLMYINESTVIMAYLFTIFNSLQGMF |
| Q9HBW9 | CNMGFSGNGVTICEDDNECGNLTQSCGENANCTNTEGSYYC |
| Q9HBW9 | TICEDDNECGNLTQSCGENANCTNTEGSYYCMCVPGFRSSS |
| Q9HBW9 | CIENVNANCHLDNVCIAANINKTLTKIRSIKEPVALLQEVY |
| Q9HBW9 | TDIITYIEILAESSSLLGYKNNTISAKDTLSNSTLTEFVKT |
| Q9HBW9 | ESSSLLGYKNNTISAKDTLSNSTLTEFVKTVNNFVQRDTFV |

| | |
|---|---|
| Q9HBW9 | VEQATLRISQSFQKTTEFDTNSTDIALKVFFFDSYNMKHIH |
| Q9HBW9 | KVTDRYRSLCAFWNYSPDTMNGSWSSEGCELTYSNETHTSC |
| Q9HBW9 | YSPDTMNGSWSSEGCELTYSNETHTSCRCNHLTHFAILMSS |
| P30556 | AGLASLPAIIHRNVFFIENTNITVCAFHYESQNSTLPIGLG |
| P30556 | NVFFIENTNITVCAFHYESQNSTLPIGLGLTKNILGFLFPF |
| P51635 | ASSVLLHTGQKMPLIGLGTWKSEPGQVKAAIKYALSVGYRH |
| P51635 | SVYGNETEIGEALKESVGAGKAVPREELFVTSKLWNTKHHP |
| P51635 | GAGKAVPREELFVTSKLWNTKHHPEDVEPAVRKTLADLQLE |
| P51635 | GDNPFPKNADGTVKYDSTHYKETWKALEALVAKGLVKALGL |
| P51635 | VKYDSTHYKETWKALEALVAKGLVKALGLSNFSSRQIDDVL |
| B2VPR8 | AIKSIPDDNTKRVILSFSPGNYSEKVKIGMYKHYITFYGED |
| D8VPP5 | AIKSIPDDNTKRVILSLAPGNYSEKVKIGMYKHYITFYGED |
| P02768 | AYSRGVFRRDAHKSEVAHRFKDLGEENFKALVLIAFAQYLQ |
| P02768 | LQQCPFEDHVKLVNEVTEFAKTCVADESAENCDKSLHTLFG |
| P02768 | RPEVDVMCTAFHDNEETFLKKYLYEIARRHPYFYAPELLFF |
| P02768 | IARRHPYFYAPELLFFAKRYKAAFTECCQAADKAACLLPKL |
| P02768 | LPKLDELRDEGKASSAKQRLKCASLQKFGERAFKAWAVARL |
| P02768 | KFGERAFKAWAVARLSQRFPKAEFAEVSKLVTDLTKVHTEC |
| P02768 | AWAVARLSQRFPKAEFAEVSKLVTDLTKVHTECCHGDLLEC |
| P02768 | DRADLAKYICENQDSISSKLKECCEKPLLEKSHCIAEVEND |
| P02768 | AKYICENQDSISSKLKECCEKPLLEKSHCIAEVENDEMPAD |
| P02768 | VENDEMPADLPSLAADFVESKDVCKNYAEAKDVFLGMFLYE |
| P02768 | EMPADLPSLAADFVESKDVCKNYAEAKDVFLGMFLYEYARR |
| P02768 | MPADLPSLAADFVESKDVCKNYAEAKDVFLGMFLYEYARRH |
| P02768 | PSLAADFVESKDVCKNYAEAKDVFLGMFLYEYARRHPDYSV |
| P02768 | LYEYARRHPDYSVVLLLRLAKTYETTLEKCCAAADPHECYA |
| P02768 | EKCCAAADPHECYAKVFDEFKPLVEEPQNLIKQNCELFEQL |
| P02768 | ELFEQLGEYKFQNALLVRYTKKVPQVSTPTLVEVSRNLGKV |
| P02768 | STPTLVEVSRNLGKVGSKCCKHPEAKRMPCAEDYLSVVLNQ |
| P02768 | VEVSRNLGKVGSKCCKHPEAKRMPCAEDYLSVVLNQLCVLH |
| P02768 | TKCCTESLVNRRPCFSALEVDETYVPKEFNAETFTFHADIC |
| P02768 | ETFTFHADICTLSEKERQIKKQTALVELVKHKPKATKEQLK |
| P02768 | CTLSEKERQIKKQTALVELVKHKPKATKEQLKAVMDDFAAF |
| P02768 | LSEKERQIKKQTALVELVKHKPKATKEQLKAVMDDFAAFVE |
| P02768 | KQTALVELVKHKPKATKEQLKAVMDDFAAFVEKCCKADDKE |
| Q23361 | NACAQTGVNRFLHINDAWTYNKTENLKPADLENFDFLVLGT |
| P43237 | TARLKEGDVFIMPAAHPVAINASSELHLLGFGINAENNHRI |
| P43238 | TARLKEGDVFIMPAAHPVAINASSELHLLGFGINAENNHRI |
| P19963 | NEIPTEGWAKPSLKFKLNTVNGTTRTVNPLGFFKKEALPKC |
| P81943 | GATLGEVYYXIIYARVLWVGNTTQKLEWIRSLHDYQSSFFP |
| Q01594 | QAKMTWTMKAAEEAEAVANINCSEHGRAFLDGIISEGSPKC |

| | |
|---|---|
| Q01594 | IVFGVGVTQLIHGLVISLSPNMTATPDAPESKVVAHAPFYP |
| Q01594 | QTKYFNKKGYVWAGNAANYVNVSNPEQYIEMVTSPNNPEGL |
| Q01594 | KGTMRDLNTFGFKKLRERWVNITALLDQSDRFSYQELPQSE |
| P35858 | ACVCSYDDDADELSVFCSSRNLTRLPDGVPGGTQALWLDGN |
| P35858 | LTRLPDGVPGGTQALWLDGNNLSSVPPAAFQNLSSLGFLNL |
| P35858 | TQALWLDGNNLSSVPPAAFQNLSSLGFLNLQGGQLGSLEPQ |
| P35858 | NQLQEVKAGAFLGLTNVAVMNLSGNCLRNLPEQVFRGLGKL |
| P35858 | EALPNSLLAPLGRLRYLSLRNNSLRTFTPQPPGLERLWLEG |
| P35858 | RFVQAICEGDDCQPPAYTYNNITCASPPEVVGLDLRDLSEA |
| B6KAM0 | STSGNPFQANVEMKTFMERFNLTHHHQSGIYVDLGQDKEVD |
| P00978 | SGTYEKTSADGKFLYHKAKWNITMESYVVHTNYDEYAIFLT |
| P00978 | ARRAVLTQEEEGSGAGQPVTNFSKKADSCQLDYSQGPCLGL |
| P00978 | SCQLDYSQGPCLGLFKRYFYNGTSMACETFLYGGCMGNGNN |
| P02760 | AVSAGPVPTPPDNIQVQENFNISRIYGKWYNLAIGSTCPWL |
| P02760 | SGAYEKTDTDGKFLYHKSKWNITMESYVVHTNYDEYAIFLT |
| P02760 | SCQLGYSAGPCMGMTSRYFYNGTSMACETFQYGGCMGNGNN |
| P04366 | SGAYEKTSTDGKFLYHKSKWNITMESYVVHTNYDEYAIFLT |
| P04366 | SCQLGYSQGPCLGMIKRYFYNGSSMACETFHYGGCMGNGNN |
| P83388 | AVMLFVSGTPIPPQLPAFQNNITCMFESSTPIHPFAFRTHT |
| P83388 | LMIGSGDQMSASCRFDSMDKNRTVNMGAMGVDEMCNFYMMF |
| P83388 | ILLDKKPIADPVILVISYSGNQTKLERKLGGGQFYLPHGIY |
| P14925 | FAVNGKPYFGDQEPVQGFVMNFSSGEIIDVFKPVRKHFDMP |
| Q80ZD8 | LPNVPQSLPSYTALLDLSHNNLSRLRAEWTPTRLTNLHSLL |
| Q80ZD8 | EDLYCMHSKKLHNIFSLDFFNCSEYKESAWEAHLGDTLTIR |
| Q80ZD8 | QGMTKVWVSPSNEQVLSQGSNGSVSVRNGDLFFKKVQVEDG |
| Q80ZD8 | KKVQVEDGGVYTCYAMGETFNETLSVELKVYNFTLHGHHDT |
| Q80ZD8 | TCYAMGETFNETLSVELKVYNFTLHGHHDTLNTAYTTLVGC |
| Q43077 | AIKLPLKYPPFIDSVKKRGLNLSEIVCSSFTMGWFGEEKNV |
| Q43077 | ICVFEQYGNIMWRHTENGIPNESIEESRTEVNLIVRTIVTV |
| Q43077 | DDYPQIRGAFTNYNVWVTAYNRTEKWAGGLYVDHSRGDDTL |
| P12807 | FYPKHMIEKVGAMRPEAPPINVTQPEGVSFKMTGNVMEWSN |
| Q07075 | LHVKPLLEEDTYTGTVSISINLSAPTRYLWLHLRETRITRL |
| Q07075 | LTPSSGDGLYLLTMEFAGWLNGSLVGFYRTTYTENGQVKSI |
| Q07075 | GKPLTIYVQPEQKHTAEYAANITKSVFDYFEEYFAMNYSLP |
| Q07075 | EYAANITKSVFDYFEEYFAMNYSLPKLDKIAIPDFGTGAME |
| Q07075 | VMDTWTRQMGYPVLNVNGVKNITQKRFLLDPRANPSQPPSD |
| Q07075 | SQPPSDLGYTWNIPVKWTEDNITSSVLFNRSEKEGITLNSS |
| Q07075 | YTWNIPVKWTEDNITSSVLFNRSEKEGITLNSSNPSGNAFL |
| Q07075 | EDNITSSVLFNRSEKEGITLNSSNPSGNAFLKINPDHIGFY |
| Q07075 | ITSSVLFNRSEKEGITLNSSNPSGNAFLKINPDHIGFYRVN |
| Q07075 | LIDDAFALARAQLLDYKVALNLTKYLKREENFLPWQRVISA |

| | |
|---|---|
| Q07075 | LLRSSVLGFACKMGDREALNNASSLFEQWLNGTVSLPVNLR |
| Q07075 | CKMGDREALNNASSLFEQWLNGTVSLPVNLRLLVYRYGMQN |
| Q07075 | NLRLLVYRYGMQNSGNEISWNYTLEQYQKTSLAQEKEKLLY |
| Q07075 | QKTSLAQEKEKLLYGLASVKNVTLLSRYLDLLKDTNLIKTQ |
| P15144 | TVRFTCKEATDVIIIHSKKLNYTLSQGHRVVLRGVGGSQPP |
| P15144 | AADARKSFPCFDEPAMKAEFNITLIHPKDLTALSNMLPKGP |
| P15144 | ALSNMLPKGPSTPLPEDPNWNVTEFHTTPKMSTYLLAFIVS |
| P15144 | LIRIWARPSAIAAGHGDYALNVTGPILNFFAGHYDTPYPLP |
| P15144 | FAYQNTIYLNLWDHLQEAVNNRSIQLPTTVRDIMNRWTLQM |
| P15144 | TVDTSTGTLSQEHFLLDPDSNVTRPSEFNYVWIVPITSIRD |
| P15144 | VRAQNDLFSTSGNEWVLLNLNVTGYYRVNYDEENWRKIQTQ |
| P15144 | IINDAFNLASAHKVPVTLALNNTLFLIEERQYMPWEAALSS |
| P15144 | YGPMKNYLKKQVTPLFIHFRNNTNNWREIPENLMDQYSEVN |
| P15144 | CNAIAQGGEEEWDFAWEQFRNATLVNEADKLRAALACSKEL |
| P97449 | RVILRPYLTPNNQGLYIFQGNSTVRFTCNQTTDVIIIHSKK |
| P97449 | TPNNQGLYIFQGNSTVRFTCNQTTDVIIIHSKKLNYTLKGN |
| P97449 | TVRFTCNQTTDVIIIHSKKLNYTLKGNHRVVLRTLDGTPAP |
| P97449 | AADARKSFPCFDEPAMKAMFNITLIYPNNLIALSNMLPKES |
| P97449 | FHSTPKMSTYLLAYIVSEFKNISSVSANGVQIGIWARPSAI |
| P97449 | QIGIWARPSAIDEGQGDYALNVTGPILNFFAQHYNTSYPLP |
| P97449 | QGDYALNVTGPILNFFAQHYNTSYPLPKSDQIALPDFNAGA |
| P97449 | TVNTNTGEISQKHFLLDSKSNVTRPSEFNYIWIAPIPFLKS |
| P97449 | APIPFLKSGQEDHYWLDVEKNQSAKFQTSSNEWILLNINVT |
| P97449 | EKNQSAKFQTSSNEWILLNINVTGYYLVNYDENNWKKLQNQ |
| P97449 | QTDLSVIPVINRAQIIHDSFNLASAKMIPITLALDNTLFLV |
| P97449 | YGPMKRYLKKQVTPLFFYFQNRTNNWVNRPPTLMEQYNEIN |
| P97449 | GLKECRDLVVELYSQWMKNPNNNTIHPNLRSTVYCNAIAFG |
| P97449 | LKECRDLVVELYSQWMKNPNNNTIHPNLRSTVYCNAIAFGG |
| P97449 | KECRDLVVELYSQWMKNPNNNTIHPNLRSTVYCNAIAFGGE |
| P97449 | LVVELYSQWMKNPNNNTIHPNLRSTVYCNAIAFGGEEEWNF |
| P97449 | CNAIAFGGEEEWNFAWEQFRNATLVNEADKLRSALACSKDV |
| P15145 | DQSKPWNRYRLPTTLLPDSYNVTLRPYLTPNADGLYIFKGK |
| P15145 | IVRLLCQEPTDVIIIHSKKLNYTTQGHMVVLRGVGDSQVPE |
| P15145 | STDARKSFPCFDEPAMKATFNITLIHPNNLTALSNMPPKGS |
| P15145 | PCFDEPAMKATFNITLIHPNNLTALSNMPPKGSSTPLAEDP |
| P15145 | LTALSNMPPKGSSTPLAEDPNWSVTEFETTPVMSTYLLAYI |
| P15145 | TTPVMSTYLLAYIVSEFQSVNETAQNGVLIRIWARPNAIAE |
| P15145 | LIRIWARPNAIAEGHGMYALNVTGPILNFFANHYNTSYPLP |
| P15145 | HGMYALNVTGPILNFFANHYNTSYPLPKSDQIALPDFNAGA |
| P15145 | LTEDLFKEGLASYLHAFAYQNTTYLDLWEHLQKAVDAQTSI |
| P15145 | MDRWTLQMGFPVITVDTKTGNISQKHFLLDSESNVTRSSAF |

| | |
|---|---|
| P15145 | TVDTKTGNISQKHFLLDSESNVTRSSAFDYLWIVPISSIKN |
| P15145 | SQAQNDLFKTASDDWVLLNVNVTGYFQVNYDEDNWRMIQHQ |
| P15145 | YFQVNYDEDNWRMIQHQLQTNLSVIPVINRAQVIYDSFNLA |
| P15145 | KKYLRKQVEPLFQHFETLTKNWTERPENLMDQYSEINAIST |
| P85073 | EWEKFKTKHITSQSVADFNCNRTMNDPAYTPDGQCKPINTF |
| P85073 | FIHSTTGPVKEICRRATGRVNKSSTQQFTLTTCKNPIRCKY |
| P85073 | TQQFTLTTCKNPIRCKYSQSNTTNFICITCRDNYPVHFVKT |
| P30533 | TEAEFEEPRVIDLWDLAQSANLTDKELEAFREELKHFEAKI |
| P04745 | RNMVNFRNVVDGQPFTNWYDNGSNQVAFGRGNRGFIVFNND |
| P04745 | QTGLPAGTYCDVISGDKINGNCTGIKIYVSDDGKAHFSISN |
| P0C1B3 | DTTKDVVKNEWYDWVGSLVSNYSIDGLRIDTVKHVQKDFWP |
| P69327 | ATDIVWPLVRNDLSYVAQYWNQTGYDLWEEVNGSSFFTIAV |
| P69327 | DAVKTFADGFVSIVETHAASNGSMSEQYDKSDGEQLSARDL |
| P69328 | ATDIVWPLVRNDLSYVAQYWNQTGYDLWEEVNGSSFFTIAV |
| P69328 | DAVKTFADGFVSIVETHAASNGSMSEQYDKSDGEQLSARDL |
| P04746 | QTGLPAGTYCDVISGDKINGNCTGIKIYVSDDGKAHFSISN |
| P00690 | RNMVWFRNVVDGQPFANWWANGSNQVAFGRGNRGFIVFNND |
| P54802 | VLPAFAGHVPEAVTRVFPQVNVTKMGSWGHFNCSYSCSFLL |
| P54802 | AVTRVFPQVNVTKMGSWGHFNCSYSCSFLLAPEDPIFPIIG |
| P54802 | GLFGALEAVNGGPEAARLFPNSTMVGTGMAPEGISQNEVVY |
| P54802 | YGVSHPDAGAAWRLLLRSVYNCSGEACRGHNRSPLVRRPSL |
| P54802 | GEACRGHNRSPLVRRPSLQMNTSIWYNRSDVFEAWRLLLTS |
| P54802 | HNRSPLVRRPSLQMNTSIWYNRSDVFEAWRLLLTSAPSLAT |
| P80929 | YDPSPTGHDDRYCNTMMERRNMTRPCKDTNTFIHGNSDDIR |
| Q9Y5C1 | EEKELRRTTYKLQVKNEEVKNMSLELNSKLESLLEEKILLQ |
| Q9Y5C1 | VISGSPWTLIQHRIDGSQNFNETWENYKYGFGRLDGEFWLG |
| Q9Y5C1 | DWKDNKHYIEYSFYLGNHETNYTLHLVAITGNVPNAIPENK |
| Q8NI99 | FTGAVCWSGPASTRATPEAANASELAALRMRVGRHEELLRE |
| Q8NI99 | GADLGAEPAAALALLGERVLNASAEAQRAAARFHQLDVKFR |
| P01019 | WAGLAAGDRVYIHPFHLVIHNESTCEQLAKANAGKPKDPTF |
| P01019 | ALDHTADRLQAILGVPWKDKNCTSRLDAHKVLSALQAVQGL |
| P01019 | FQGKMKGFSLLAEPQEFWVDNSTSVSVPMLSGMGTFQHWSD |
| P01019 | VSVPMLSGMGTFQHWSDIQDNFSVTQVPFTESACLLLIQPH |
| Q6IWH7 | DFLPRAYYRWTRAHDLRGFLNFTLARAPSSFAAAHNRTCRY |
| Q6IWH7 | LRGFLNFTLARAPSSFAAAHNRTCRYRAFRDDDGHYSQTYW |
| Q4KMQ2 | LLLAAVVGVACFLYGYLNQDNCTWSKEVCHPDIGGKIIMCP |
| Q4KMQ2 | IGGKIIMCPQCDRLCPFWKLNITCESSKKLCIFDSFGTLVF |
| Q4KMQ2 | IIVYRLSVFIVFSAKLPKNINGTDPIQKYLTPQTATSITAS |
| Q4KMQ2 | WSFSVPPYGDHTSYTMEGYINNTLSIFKVADFKNKSKGNPY |
| Q4KMQ2 | YTMEGYINNTLSIFKVADFKNKSKGNPYSDLGNHTTCRYRD |
| Q4KMQ2 | IFKVADFKNKSKGNPYSDLGNHTTCRYRDFRYPPGHPQEYK |

| | |
|---|---|
| Q6P9J9 | LLLAAVVGVACFLYGYLDQDNCTWSKEVCDPDIGGQILMCP |
| Q6P9J9 | IGGQILMCPQCDRLCPFWRLNITCESSKKLCIFDSFGTLIF |
| Q6P9J9 | IGIIVYRLSVFIVFSTTLPKNPNGTDPIQKYLTPQMATSIT |
| Q6P9J9 | IIVYRLSVFIVFSTTLPKNPNGTDPIQKYLTPQMATSITAS |
| Q6P9J9 | WSFSIPPYGDHTYYTMDGYINNTLSVFNITDFKNTDKENPY |
| Q6P9J9 | YGDHTYYTMDGYINNTLSVFNITDFKNTDKENPYIGLGNYT |
| Q6P9J9 | VFNITDFKNTDKENPYIGLGNYTLCRYRDFRNPPGHPQEYK |
| P18910 | LLLRGGHASDLTVAVVLPLTNTSYPWSWARVGPAVELALAR |
| P18910 | DDRPCFFIVEGLYMRVRERLNITVNHQEFVEGDPDHYPKLL |
| P18910 | DNPEYLEFLKQLKLLADKKFNFTVEDGLKNIIPASFHDGLL |
| P18910 | LYVQAVTETLAQGGTVTDGENITQRMWNRSFQGVTGYLKID |
| P18910 | ETLAQGGTVTDGENITQRMWNRSFQGVTGYLKIDRNGDRDT |
| P18910 | DFSLWDMDPETGAFRVVLNYNGTSQELMAVSEHKLYWPLGY |
| P17342 | LFSLTRVRPAIEYALRSVEGNGTGRRLLPPGTRFQVAYEDS |
| P17342 | VAHRHGMTSGDYAFFNIELFNSSSYGDGSWKRGDKHDFEAK |
| P17342 | EVLRAGYSKKDGGKIIQQTWNRTFEGIAGQVSIDANGDRYG |
| P41361 | IFLSPLSISTAFAMTKLGACNNTLKQLMEVFKFDTISEKTS |
| P41361 | TSDQIHFFFAKLNCRLYRKANKSSELVSANRLFGGKSITFN |
| P41361 | NKSSELVSANRLFGGKSITFNETYQDISEVVYGAKLQPLDF |
| P41361 | PLDFKGNAEQSRLTINQWISNKTEGRITDVIPPQAINEFTV |
| P01008 | IFLSPLSISTAFAMTKLGACNDTLQQLMEVFKFDTISEKTS |
| P01008 | TSDQIHFFFAKLNCRLYRKANKSSKLVSANRLFGDKSLTFN |
| P01008 | NKSSKLVSANRLFGDKSLTFNETYQDISELVYGAKLQPLDF |
| P01008 | PLDFKENAEQSRAAINKWVSNKTEGRITDVIPSEAINELTV |
| P32261 | IFLSPLSISTAFAMTKLGACNDTLKQLMEVFKFDTISEKTS |
| P32261 | TSDQIHFFFAKLNCRLYRKANKSSDLVSANRLFGDKSLTFN |
| P32261 | NKSSDLVSANRLFGDKSLTFNESYQDVSEVVYGAKLQPLDF |
| P32261 | PLDFKENPEQSRVTINNWVANKTEGRIKDVIPQGAINELTA |
| Q9H6X2 | IALTDGELHEDLFFYSEREANRSRDLGAIVYCVGVKDFNET |
| Q9H6X2 | EANRSRDLGAIVYCVGVKDFNETQLARIADSKDHVFPVNDG |
| Q9H6X2 | RGNGFRHARNVDRVLCSFKINDSVTLNEKPFSVEDTYLLCP |
| P19801 | ERHPVREARAVIFFGDQEHPNVTEFAVGPLPGPCYMRALSP |
| P19801 | YALLYHTLQEATKPLHQFFLNTTGFSFQDCHDRCLAFTDVA |
| P19801 | VDLDVAGTKNSFQTLQMKLENITNPWSPRHRVVQPTLEQTQ |
| Q16853 | SPPPAREALAIVFFGRQPQPNVSELVVGPLPHPSYMRDVTV |
| Q16853 | TTAPRGLQSGDRATWFGLYYNISGAGFFLHHVGLELLVNHK |
| Q16853 | LAQLEAQFEAGLVNVVLIPDNGTGGSWSLKSPVPPGPAPPL |
| Q16853 | EEQAAFLVGSATPRYLYLASNHSNKWGHPRGYRIQMLSFAG |
| Q16853 | GHPRGYRIQMLSFAGEPLPQNSSMARGFSWERYQLAVTQRK |
| Q16853 | SVFNQNDPWAPTVDFSDFINNETIAGKDLVAWVTAGFLHIP |
| Q29437 | SPPPAREALAIVFFGGQPQPNVTELVVGPLPQPSYMRDVTV |

| | |
|---|---|
| Q29437 | NSAPRGVQSGDRSTWFGIYYNITKGGPYLHPVGLELLVDHK |
| Q29437 | SVFNQNDPWTPTVDFSDFINNETIAGKDLVAWVTAGFLHIP |
| Q63HQ0 | KTEEDILRAALKYSNKKTGSNPTSASDDSNGLEWENDFVSA |
| P09465 | EKIEEGGPLRFYFRHIDCYKNCSEMEITFYVITNNQCSKTT |
| P09465 | FYVITNNQCSKTTVIGYLKGNGTYQTQFEGNNIFQPLYITS |
| P51693 | LEERRMRQINEVMREWAMADNQSKNLPKADRQALNEHFQSI |
| P51693 | KAQQMRFQVHTHLQVIEERVNQSLGLLDQNPHLAQELRPQI |
| P51693 | DAASPEKEKMNPLEQYERKVNASVPRGFPFHSSEIQRDELA |
| P10762 | AQARPDAAGHVNIAEAVQQLNHTIVNAAHELHETLGLPTPD |
| P10762 | EAEKHQGSVAEQLNRFARNLNNSIHDAATSAQPADQLNSLQ |
| Q9V496 | NYYDYSFDSILTIGASSDVPNDSDDTSLKVSGSAKIFAKGN |
| Q9V496 | STDYQIIYSQHGFLPRSSRINVTTEFFGTNYNVFEASVRQE |
| Q9V496 | LKFSTKNIGEANDKIIAFEANGSLKGELRGNGEIQGTFIFN |
| Q9V496 | EFFLDSKTSLGQYARVNTVWNGTANDGSYDFEAQTNNMESP |
| Q9V496 | DIKINSEASYVSIDEFYIIVNWSSKKLKLDGYELEARAQSK |
| Q9V496 | KNENGIIFSGTATYALKKELNKTIIDGQGKVQYQGKALSGN |
| Q9V496 | LNFAYPGNCKYILAQDSVDNNFTIIGQLTNGKLKSITLIDR |
| Q9HDC9 | CKTRDDEPVCGRPLGIRAGPNGTLFVADAYKGLFEVNPWKR |
| Q9HDC9 | NPWKREVKLLLSSETPIEGKNMSFVNDLTVTQDGRKIYFTD |
| P04114 | LPALLLLLLAGARAEEEMLENVSLVCPKDATRFKHLRKYTY |
| P04114 | LVPPETEEAKQVLFLDTVYGNCSTHFTVKTRKGNVATEIST |
| P04114 | WSVCKQVFPGLNYCTSGAYSNASSTDSASYYPLTGDTRLEL |
| P04114 | LQVPLLGVLDLSTNVYSNLYNWSASYSGGNTSTDHFSLRAR |
| P04114 | DLSTNVYSNLYNWSASYSGGNTSTDHFSLRARYHMKADSVV |
| P04114 | LSCQRDPNTGRLNGESNLRFNSSYLQGTNQITGRYEDGTLS |
| P04114 | RVNLVKTIHDLHLFIENIDFNKSGSSTASWIQNVDTKYQIR |
| P04114 | GQVYSTLVTYISDWWTLAAKNLTDFAEQYSIQDWAKRMKAL |
| P04114 | YSILKIQSPLFTLDANADIGNGTTSANEAGIAASITAKGES |
| P04114 | KINSKHLRVNQNLVYESGSLNFSKLEIQSQVDSQHVGHSVL |
| P04114 | SAQQASWQVSARFNQYKYNQNFSAGNNENIMEAHVGINGEA |
| P04114 | FDRHFEKNRNNALDFVTKSYNETKIKFDKYKAEKSHDELPR |
| P04114 | SLPDFKELCTISHIFIPAMGNITYDFSFKSSVITLNTNAEL |
| P04114 | TYDFSFKSSVITLNTNAELFNQSDIVAHLLSSSSSVIDALQ |
| P04114 | RGLKLATALSLSNKFVEGSHNSTVSLTTKNMEVSVATTTKA |
| P04114 | NGNTKSKPTVSSSMEFKYDFNSSMLYSTAKGAVDHKLSLES |
| P04114 | IVIPSFQALTARFEVDSPVYNATWSASLKNKADYVETVLDS |
| P04114 | CTMFIREVGTVLSQVYSKVHNGSEILFSYFQDLVITLPFEL |
| P04114 | IKNLLVALKDFHSEYIVSASNFTSQLSSQVEQFLHRNIQEY |
| E9Q414 | LLFLLLFLDTSVWAQDEVLENLSFSCPKDATRFKHLRKYVY |
| E9Q414 | LVPPETEEDQQELFLDTVYGNCSTQVTVNSRKGTVPTEMST |
| E9Q414 | WSTCKPLFTGMNYCTTGAYSNASSTESASYYPLTGDTRYEL |

| | |
|---|---|
| E9Q414 | LQVPLLGVLDLSTNVYSNLYNWSASYTGGNTSRDHFSLQAQ |
| E9Q414 | DLSTNVYSNLYNWSASYTGGNTSRDHFSLQAQYRMKTDSVV |
| E9Q414 | LSCERDVTTGQLSGESNMRFNSTYFQGTNQIVGMYQDGALS |
| E9Q414 | NQVYSTLVTYMSDWWTLTAKNITDFAEQYSIQNWAESIKVL |
| E9Q414 | HSILKIQSPLFILDANANIQNVTTSGNKAEIVASVTAKGES |
| E9Q414 | NINGKHLRVIQKLTYESGFLNYSKFEVESKVESQHVGSSIL |
| E9Q414 | RAQQASWQASTRFNQYKYNQNFSAINNEHNIEASIGMNGDA |
| E9Q414 | FLPDFKGFNTIDNIYIPAMGNFTYDFSFKSSVITLNTNAGL |
| E9Q414 | TYDFSFKSSVITLNTNAGLYNQSDIVAHFLSSSSFVTDALQ |
| E9Q414 | NGNTKSKPTVSSSIELNYDFNSSKLHSTATGGIDHKFSLES |
| E9Q414 | IFIPFFGELTARAGMASPLYNVTWSAGWKTKADHVETFLDS |
| P55056 | KMSRWSLVRGRMKELLETVVNRTRDGWQWFWSPSTFRGFMQ |
| P05090 | RWYEIEKIPTTFENGRCIQANYSLMENGKIKVLNQELRADG |
| P05090 | NQELRADGTVNQIEGEATPVNLTEPAKLEVKFSWFMPSAPY |
| P02649 | QVTQELRALMDETMKELKAYKSELEEQLTPVAEETRARLSK |
| Q13790 | AGCQADVWALQLQLYRQGGVNATQVLIQHLRGLQKGRSTER |
| P17690 | WPINTLKCMPRVCPFAGILENGTVRYTTFEYPNTISFSCHT |
| P17690 | CPPPPIPKFASLSVYKPLAGNNSFYGSKAVFKCLPHHAMFG |
| P17690 | NSFYGSKAVFKCLPHHAMFGNDTVTCTEHGNWTQLPECREV |
| P17690 | KCLPHHAMFGNDTVTCTEHGNWTQLPECREVRCPFPSRPDN |
| P17690 | GCHETYSLDGPEEVECSKFGNWSAQPSCKASCKLSIKRATV |
| P02749 | CPPPSIPTFATLRVYKPSAGNNSLYRDTAVFECLPQHAMFG |
| P02749 | NSLYRDTAVFECLPQHAMFGNDTITCTTHGNWTKLPECREV |
| P02749 | ECLPQHAMFGNDTITCTTHGNWTKLPECREVKCPFPSRPDN |
| P02749 | GCHDGYSLDGPEEIECTKLGNWSAMPSCKASCKVPVKKATV |
| Q01339 | PFAGILENGIVRYTSFEYPKNISFACNPGFFLNGTSSSKCT |
| Q01339 | YTSFEYPKNISFACNPGFFLNGTSSSKCTEEGKWSPDIPAC |
| Q01339 | CPPPPVPKFALLKDYRPSAGNNSLYQDTVVFKCLPHFAMIG |
| Q01339 | NSLYQDTVVFKCLPHFAMIGNDTVMCTEQGNWTRLPECLEV |
| Q01339 | KCLPHFAMIGNDTVMCTEQGNWTRLPECLEVKCPFPPRPEN |
| O14791 | HDLVIKSLDKLKEVREFLGENISNFLSLAGNTYQLTRGIGK |
| O95445 | GRPDMKTELFSSSCPGGIMLNETGQGYQRFLLYNRSPHPPE |
| P24020 | CVWTIEMKPGHKIILQILPLNLTCGKEYLEVRDQRAGPDNF |
| P29972 | TLFVFISIGSALGFKYPVGNNQTAVQDNVKVSLAFGLSIAT |
| P29972 | DYTGCGINPARSFGSAVITHNFSNHWIFWVGPFIGGALAVL |
| Q42460 | TETSFNFPNFHTDDKLILQGNATISSKGQLQLTGVGSNELP |
| Q42460 | AFYSDPIQIKDSNNVASFNTNFTFIIRAKNQSISAYGLAFA |
| Q42460 | KDSNNVASFNTNFTFIIRAKNQSISAYGLAFALVPVNSPPQ |
| P19329 | LVLLTHANSSNDASFNVETFNKTNLILQGDATVSSEGHLLL |
| P19329 | YSAPIQINDRTIDNLASFSTNFTFRINAKNIENSAYGLAFA |
| P19329 | FALVPVGSRPKLKGRYLGLFNTTNYDRDAHTVAVVFDTVSN |

| | |
|---|---|
| P15514 | PVVLSLLILGSGHYAAGLDLNDTYSGKREPFSGDHSADGFE |
| P15514 | IPGYIVDDSVRVEQVVKPPQNKTESENTSDKPKRKKKGGKN |
| P15514 | DDSVRVEQVVKPPQNKTESENTSDKPKRKKKGGKNGKNRRN |
| P15289 | HQGFHRFLGIPYSHDQGPCQNLTCFPPATPCDGGCDQGLVP |
| P15289 | PATPCDGGCDQGLVPIPLLANLSVEAQPPWLPGLEARYMAF |
| P15289 | LASSLDLLPTLAALAGAPLPNVTLDGFDLSPLLLGTGKSPR |
| P15848 | YLLGSEDYYSHERCTLIDALNVTRCALDFRDGEEVATGYKN |
| P15848 | QDKNRHHYAGMVSLMDEAVGNVTAALKSSGLWNNTVFIFST |
| P15848 | SLMDEAVGNVTAALKSSGLWNNTVFIFSTDNGGQTLAGGNN |
| P15848 | ELIHISDWLPTLVKLARGHTNGTKPLDGFDVWKTISEGSPS |
| P15848 | PRNSMAPAKDDSSLPEYSAFNTSVHAAIRHGNWKLLTGYPG |
| P15848 | WKLLTGYPGCGYWFPPPSQYNVSEIPSSDPPTKTLWLFDID |
| P51689 | PNILLIMADDLGTGDLGCYGNNTLRTPNIDQLAEEGVRLTQ |
| P51689 | DASNGYRALQWNAGSGGLPENETTFARILQQHGYATGLIGK |
| P51689 | EEMDWLIGKVLNAIEDNGLKNSTFTYFTSDHGGHLEARDGH |
| P51690 | PNILLLMADDLGIGDIGCYGNNTMRTPNIDRLAEDGVKLTQ |
| P51690 | VSSIGYRVLQWTGASGGLPTNETTFAKILKEKGYATGLIGK |
| P51690 | LASSYFVGALIVHADCFLMRNHTITEQPMCFQRTTPLILQE |
| P51690 | EEMDWMVGRILDTLDVEGLSNSTLIYFTSDHGGSLENQLGN |
| Q10723 | AAHQRPNFVVIFTDDQDGIQNSTHPRYQPKLHEHIRYPGIE |
| Q10723 | TPVCCPSRTNLWRGQFSHNTNFTDVLGPHGGYAKWKSLGID |
| Q10723 | AQISPIAPHTSTQIYFDPVANATKTFFYPPIPAPRHWELFS |
| Q10723 | EADVSDKPAWIRALPLAQQNNRTYLEEVYRLRLRSLASVDE |
| Q10723 | QGEFWGGWSDEVLHHIPRYTNNSWKAVRVYDEDNQQAWKLI |
| Q10723 | TNERELYDLKTDPGELCNIYNKTRAAVRTRLEALLAVLVVC |
| Q10723 | GESCTNPWKILHPEGSVNSWNQSLDRKYDKYYANVAPFQYR |
| Q7Z1F8 | NTNDEYYKIGKDYDIEANIDNYTNKKAVEDFLKMYRCGFLP |
| Q7Z1F8 | PEAAVEYGIVKEDNHYVYYSNYSNAITYYNEEQRLAYFTED |
| Q7Z1F8 | YFVQALQKGVFEGFGQTIYLNDSKANSFVGNYWQDNADLYG |
| Q13510 | GHLIHGRNMDFGVFLGWNINNDTWVITEQLKPLTVNLDFQR |
| Q13510 | TWVITEQLKPLTVNLDFQRNNKTVFKASSFAGYVGMLTGFK |
| Q13510 | WILGKKDVMWIGFLTRTVLENSTSYEEAKNLLTKTKILAPA |
| Q13510 | AKNLLTKTKILAPAYFILGGNQSGEGCVITRDRKESLDVYE |
| Q13510 | DRWKHPFFLDDRRTPAKMCLNRTSQENISFETMYDVLSTKP |
| Q13510 | FFLDDRRTPAKMCLNRTSQENISFETMYDVLSTKPVLNKLT |
| Q9WV54 | GHLLHGRNMDFGIFLGWNINNNTWVVTEELKPLTVNLDFQR |
| Q9WV54 | TWVVTEELKPLTVNLDFQRNNKTVFKATSFVGYVGMLTGFK |
| Q9WV54 | WMFGRKDAQWVGFITRSVLENTTSYEEAKNTLTKTKIMAPV |
| Q9WV54 | DRWKNTLFIDDRRTPAKKCLNHTTQKNLSFATIYDVLSTKP |
| Q9WV54 | LFIDDRRTPAKKCLNHTTQKNLSFATIYDVLSTKPVLNKLT |
| P07307 | EGHGGAQLQAELRSLKEAFSNFSSSTLTEVQAISTHGGSVG |

| | |
|---|---|
| P07307 | LKHFPVDLRFVACQMELLHSNGSQRTCCPVNWVEHQGSCYW |
| P08290 | VSSQSMQLQKEFWTLKETLSNFSTTTLMEFKALDSHGGSRN |
| P08290 | STTTLMEFKALDSHGGSRNDNLTSWETILEKKQKDIKADHS |
| P08290 | LKHFPLDLRTLTCQLAFFLSNGTECCPVNWVEFGGSCYWFS |
| P07306 | IGSQNSQLQEELRGLRETFSNFTASTEAQVKGLSTQGGNVG |
| P07306 | VKQFVSDLRSLSCQMAALQGNGSERTCCPVNWVEHERSCYW |
| P54074 | NVFINSFSYLNQTSQAVISGNSTFANVINFPYRLGLSFIGA |
| Q1XA76 | PALDFLVEKDNEYCVCEMPCNVTRYGKELSMVKIPSKASAK |
| Q1XA76 | ELSMVKIPSKASAKYLAKKYNKSEQYIGENILVLDIFFEAL |
| Q62962 | PALGLLAEKDSNYCLCRTPCNLTRYNKELSMVKIPSKTSAK |
| Q62962 | ELSMVKIPSKTSAKYLEKKFNKSEKYISENILVLDIFFEAL |
| Q23498 | KMSCLFCTFAVDGVQALIAQNSTDNEIAAFLVNLCDLFDVE |
| Q23498 | ICGAFIANCGHSDKPLTHMWNITIPGGKPPIKPWPKIPDNK |
| Q23498 | DFEAHDSWDYTEDLTRENMNNMTNVFLEYFPGVPVYVSIGN |
| Q23498 | IDGGYEGATYTVKDAKTYFANVTEANMKNKEPEWVLSYDTR |
| Q23498 | YQMADFSPQSWSDLSDKLWTNTTLFRDYVRLYYRNHYNNEC |
| Q92484 | DPTYHITDDHTKVCASSKGANASNPGPFGDVLCDSPYQLIL |
| Q92484 | SPPHVPVPELSTDTVINVITNMTTTIQSLFPNLQVFPALGN |
| Q92484 | NLRIISLNTNLYYGPNIMTLNKTDPANQFEWLESTLNNSQQ |
| Q92484 | IMTLNKTDPANQFEWLESTLNNSQQNKEKVYIIAHVPVGYL |
| Q92484 | NKEKVYIIAHVPVGYLPSSQNITAMREYYNEKLIDIFQKYS |
| Q92484 | RLFQYDPRDYKLLDMLQYYLNLTEANLKGESIWKLEYILTQ |
| P58242 | GVAGAQLGRFWHISDLHLDPNYTVSKDPLQVCPSAGSQPVL |
| P58242 | PVLNAGPWGDYLCDSPWALINSSLYAMKEIEPKPDFILWTG |
| P58242 | EIEPKPDFILWTGDDTPHVPNESLGEAAVLAIVERLTNLIK |
| P58242 | PAQSNRIYNQVAELWRPWLSNESYALFKRGAFYSEKLPGPS |
| P58242 | QTAGMADPGEQFRWLGDVLSNASRDGEMVYVIGHVPPGFFE |
| P17405 | GHPARLHRIVPRLRDVFGWGNLTCPICKGLFTAINLGLKKE |
| P17405 | EACGLLLGSTCGHWDIFSSWNISLPTVPKPPPKPPSPPAPG |
| P17405 | AVGNHESTPVNSFPPPFIEGNHSSRWLYEAMAKAWEPWLPA |
| P17405 | LRLISLNMNFCSRENFWLLINSTDPAGQLQWLVGELQAAED |
| P17405 | IDGNYSGSSHVVLDHETYILNLTQANIPGAIPHWQLLYRAR |
| P37064 | ADGTASISQCAINPGETFFYNFTVDNPGTFFYHGHLGMQRS |
| P37064 | KLPTSPPPQTPAWDDFDRSKNFTYRITAAMGSPKPPVKFNR |
| P37064 | QFKIGEVVDVILQNANMMKENLSETHPWHLHGHDFWVLGYG |
| P54958 | KFFDTGSAVGRGIEDSLTISNLTTSQQDIVLADELSQEVCI |
| P54958 | DCSKIPSLPDVTFVINGRNFNISSQYYIQQNGNLCYSGFQP |
| P55956 | GIPLFNGEYEVECSKIPSLPNITFNLGGQNFDLQGKDYILQ |
| P20933 | QALVRCSSPLPLVVNTWPFKNATEAAWRALASGGSALDAVE |
| P20933 | KVISRIQKHFPEFFGAVICANVTGSYGAACNKLSTFTQFSF |
| Q12797 | FLGHMRGSLLTLQRLVQLFPNDTSLKNDLGVGYLLIGDNDN |

| | |
|---|---|
| Q12797 | RLRMHLGLVIPKEGCKIRCANETKTWEEGKVLIFDDSFEHE |
| Q9BXN1 | KRYKELQRLGLGNNKITDIENGSLANIPRVREIHLENNKLK |
| Q9HD20 | VFTLSMLVAFEASLVQQQMRNMSEIRKMGNKPHMIQVYRSR |
| Q9HD20 | TTGLKPVDSGCVAYVLRTGFNTSQGKLLRTILFGVKRVTAN |
| Q93235 | QKSYKAYLEQMKTYLTKYDSNATETRECGAGDSNDDLEKNP |
| P06583 | EFNNERGERKVCRFKLEWLGNCSGINDETYGYRDGKPCVLI |
| P06583 | KPCVLIKLNRVLGFKPKPPKNESLEAYPVMKYSPYVLPVQC |
| P06583 | YYGKLLQPKYLQPLLAVQFTNLTMDTEIRIECKAYGENIGY |
| P05026 | DFNHERGERKVCRFKLEWLGNCSGLNDETYGYKEGKPCIII |
| P05026 | KPCIIIKLNRVLGFKPKPPKNESLETYPVMKYNPNVLPVQC |
| P05026 | YYGKLLQPKYLQPLLAVQFTNLTMDTEIRIECKAYGENIGY |
| P14415 | RLATPGLMIRPKTENLDVIVNVSDTESWDQHVQKLNKFLEP |
| P14415 | SDTESWDQHVQKLNKFLEPYNDSIQAQKNDVCRPGRYYEQP |
| P14415 | RYYEQPDNGVLNYPKRACQFNRTQLGNCSGIGDSTHYGYST |
| P14415 | DNGVLNYPKRACQFNRTQLGNCSGIGDSTHYGYSTGQPCVF |
| P14415 | TGQPCVFIKMNRVINFYAGANQSMNVTCAGKRDEDAENLGN |
| P14415 | CVFIKMNRVINFYAGANQSMNVTCAGKRDEDAENLGNFVMF |
| P14415 | PANGNIDLMYFPYYGKKFHVNYTQPLVAVKFLNVTPNVEVN |
| P14415 | YYGKKFHVNYTQPLVAVKFLNVTPNVEVNVECRINAANIAT |
| P14094 | DINHERGERKVCRFKLDWLGNCSGLNDDSYGYREGKPCIII |
| P14094 | RGERKVCRFKLDWLGNCSGLNDDSYGYREGKPCIIIKLNRV |
| P14094 | KPCIIIKLNRVLGFKPKPPKNESLETYPLMMKYNPNVLPVQ |
| P14094 | YYGKLLQPKYLQPLLAVQFTNLTVDTEIRVECKAYGENIGY |
| P97370 | KKLVEDLESFLKPYSVEEQKNLTSCPDGAPFIQHGPDYRAC |
| P97370 | RIIDLIPDGYPQISCLPKEENATIATYPEFGVLDLKYFPYY |
| P14231 | RLATPGLMIRPKTENLDVIVNISDTESWGQHVQKLNKFLEP |
| P14231 | SDTESWGQHVQKLNKFLEPYNDSIQAQKNDVCRPGRYYEQP |
| P14231 | RYYEQPDNGVLNYPKRACQFNRTQLGDCSGIGDPTHYGYST |
| P14231 | TGQPCVFIKMNRVINFYAGANQSMNVTCVGKRDEDAENLGH |
| P14231 | CVFIKMNRVINFYAGANQSMNVTCVGKRDEDAENLGHFVMF |
| P14231 | CVGKRDEDAENLGHFVMFPANGSIDLMYFPYYGKKFHVNYT |
| P14231 | PANGSIDLMYFPYYGKKFHVNYTQPLVAVKFLNVTPNVEVN |
| P14231 | YYGKKFHVNYTQPLVAVKFLNVTPNVEVNVECRINAANIAT |
| P54709 | AGYIEDLKKFLKPYTLEEQKNLTVCPDGALFEQKGPVYVAC |
| P54709 | KKLHVGYLQPLVAVQVSFAPNNTGKEVTVECKIDGSANLKS |
| O04846 | RRQIFYNHKLNSIHREYYFTNATLVNHVCNVAMFFGEGAGD |
| O04846 | VCNVAMFFGEGAGDVIIENKNYTLLQMHWHTPSEHHLHGVQ |
| O04846 | PFLSQMKEKLVKLKEERLKGNHTAQVEVGRIDTRHIERKTR |
| O04846 | RKTRKYYRYIGSLTTPPCSENVSWTILGKVRSMSKEQVELL |
| O04846 | RSMSKEQVELLRSPLDTSFKNNSRPCQPLNGRRVEMFHDHE |
| P18850 | MVLTEEPLLYIPPPPCQPLINTTESLRLNHELRGWVHRHEV |

| | |
|---|---|
| P18850 | DTFYVVSFRRDHLLLPATTHNKTTRPKMSIVLPAININENV |
| P18850 | TRILHIKSSSVPPYLRDQQRNQTNTFFGSPPAATEATHVVS |
| Q7Z3C6 | FTTFLVSCVDYDILFANKMVNHSLHPTEPVKVTLPDAFLPA |
| Q8N6G6 | TLQGTKGENSLSSTGTFLVDNSSVDFQKFPDKEILRMAGPL |
| Q86TH1 | VTSQERHCLQQRRKSVPGPGNRTCTGTSKRYQLCRVQECPP |
| Q86TH1 | SESAESQGLDGAGLMGFVPHNGSLYGQASSERLGLDNRLFG |
| Q86TH1 | EQAGGGACEGPPRGKGFRDRNVTGTPLTGDKDDEEVDTHFA |
| Q86TH1 | ANAISDQLLGAGSDLKDFTLNETVNSIFAQGAPRSSLAESF |
| Q86TH1 | LAESFFVDYEENEGAGPYLLNGSYLELSSDRVANSSSEAPF |
| Q86TH1 | GAGPYLLNGSYLELSSDRVANSSSEAPFPNVSTSLLTSAGN |
| Q86TH1 | SYLELSSDRVANSSSEAPFPNVSTSLLTSAGNRTHKARTRP |
| Q86TH1 | NSSSEAPFPNVSTSLLTSAGNRTHKARTRPKARKQGVSPAD |
| Q86TH1 | IRCSEDEKLCDPNTRPVGEKNCTGPPCDRQWTVSDWGPCSG |
| Q86TH1 | HPCGDKNCPAHWLAQDWERCNTTCGRGVKKRLVLCMELANG |
| Q6UY14 | RPDGCGVCGGDDSTCRLVSGNLTDRGGPLGYQKILWIPAGA |
| Q6UY14 | EFGGGGSSVPPERCGHLPRPNITQSCQLRLCGHWEVGSPWS |
| Q6ZMM2 | DSCLFVQRVFRDAGAFAGYWNVTLIPEGARHIRVEHRSRNH |
| P18434 | SPGVTLRPDVYGEKGLDISYNVSDSTTWAGLAHTLHRFLAG |
| P18434 | AHTLHRFLAGYSPAAQEGSINCTSEKYFFQESFLAPNHTKF |
| P18434 | EGSINCTSEKYFFQESFLAPNHTKFSCKFTADMLQNCSGRP |
| P18434 | SFLAPNHTKFSCKFTADMLQNCSGRPDPTFGFAEGKPCFII |
| P18434 | AEGKPCFIIKMNRIVKFLPGNSTAPRVDCAFLDQPRDGPPL |
| P18434 | CAFLDQPRDGPPLQVEYFPANGTYSLHYFPYYGKKAQPHYS |
| Q24046 | KKPAESDYWIELIDDFLRDYNHTEGRDMKHCGFGQVLEPTD |
| Q24046 | IPEVYDKEEKDMPDDLKKVINETKTEERQQVWVSCNGHLGK |
| Q24048 | PKGQVCDVDIKTWSPCTKENNYSYHKSAPCIFLKLNKIYGW |
| Q24048 | SAPCIFLKLNKIYGWIPEYYNRSNDLPANMPASLKTYIAEV |
| O75882 | SIYAPLVAAFSGLIVPERDGNETVPEVVATSGYALLHFFSD |
| O75882 | PEVVATSGYALLHFFSDAAYNLTGFNITYSFDMCPNNCSGR |
| O75882 | TSGYALLHFFSDAAYNLTGFNITYSFDMCPNNCSGRGECKI |
| O75882 | DAAYNLTGFNITYSFDMCPNNCSGRGECKISNSSDTVECEC |
| O75882 | TYSFDMCPNNCSGRGECKISNSSDTVECECSENWKGEACDI |
| O75882 | EACDIPHCTDNCGFPHRGICNSSDVRGCSCFSDWQGPGCSV |
| O75882 | RGCSCFSDWQGPGCSVPVPANQSFWTREEYSNLKLPRASHK |
| O75882 | ASHKAVVNGNIMWVVGGYMFNHSDYNMVLAYDLASREWLPL |
| O75882 | HSDYNMVLAYDLASREWLPLNRSVNNVVVRYGHSLALYKDK |
| O75882 | SLALYKDKIYMYGGKIDSTGNVTNELRVFHIHNESWVLLTP |
| O75882 | GGKIDSTGNVTNELRVFHIHNESWVLLTPKAKEQYAVVGHS |
| O75882 | YLHTAVIVSGTMLVFGGNTHNDTSMSHGAKCFSSDFMAYDI |
| O75882 | LPRPDLHHDVNRFGHSAVLHNSTMYVFGGFNSLLLSDILVF |
| O75882 | YSCTANTNDCHWCNDHCVPRNHSCSEGQISIFRYENCPKDN |

| | |
|---|---|
| O75882 | QSSQSMSKLTLTPWVGLRKINVSYWCWEDMSPFTNSLLQWM |
| O75882 | GILSEPSTRGLKAATCINPLNGSVCERPANHSAKQCRTPCA |
| O75882 | GLKAATCINPLNGSVCERPANHSAKQCRTPCALRTACGDCT |
| O75882 | ASFPFGQCMEWYTMSTCPPENCSGYCTCSHCLEQPGCGWCT |
| O75882 | GPVKMPSQAPTGNFYPQPLLNSSMCLEDSRYNWSFIHCPAC |
| O75882 | GNFYPQPLLNSSMCLEDSRYNWSFIHCPACQCNGHSKCINQ |
| O75882 | YNWSFIHCPACQCNGHSKCINQSICEKCENLTTGKHCETCI |
| O75882 | ACQCNGHSKCINQSICEKCENLTTGKHCETCISGFYGDPTN |
| O75882 | TAINFVATPDEQNRDLDMFINASKNFNLNITWAASFSAGTQ |
| O75882 | PDEQNRDLDMFINASKNFNLNITWAASFSAGTQAGEEMPVV |
| O75882 | NIKEYKDSFSNEKFDFRNHPNITFFVYVSNFTWPIKIQIAF |
| O75882 | SNEKFDFRNHPNITFFVYVSNFTWPIKIQIAFSQHSNFMDL |
| Q76LX8 | AQFRVHLVKMVILTEPEGAPNITANLTSSLLSVCGWSQTIN |
| Q76LX8 | VHLVKMVILTEPEGAPNITANLTSSLLSVCGWSQTINPEDD |
| Q76LX8 | CDGRMDSQQVWDRCQVCGGDNSTCSPRKGSFTAGRAREYVT |
| Q76LX8 | KGSFTAGRAREYVTFLTVTPNLTSVYIANHRPLFTHLAVRI |
| Q76LX8 | HLAVRIGGRYVVAGKMSISPNTTYPSLLEDGRVEYRVALTE |
| Q76LX8 | GPLQEDADIQVYRRYGEEYGNLTRPDITFTYFQPKPRQAWV |
| Q76LX8 | VWAAVRGPCSVSCGAGLRWVNYSCLDQARKELVETVQCQGS |
| Q76LX8 | WEVSEPSSCTSAGGAGLALENETCVPGADGLEAPVTEGPGS |
| Q76LX8 | VAIGRPLGEVVTLRVLESSLNCSAGDMLLLWGRLTWRKMCR |
| Q76LX8 | HARIAIHALATNMGAGTEGANASYILIRDTHSLRTTAFHGQ |
| Q9UNA0 | KQILGPEELPGQTYDATQQCNLTFGPEYSVCPGMDVCARLW |
| Q9UNA0 | CDGIIGSKLQYDKCGVCGGDNSSCTKIVGTFNKKSKGYTDV |
| Q9UNA0 | GEYLINGKYMISTSETIIDINGTVMNYSGWSHRDDFLHGMG |
| Q9UNA0 | NGKYMISTSETIIDINGTVMNYSGWSHRDDFLHGMGYSATK |
| O96910 | IILSLALVAAVFADQNCDIGNITSQCQMQHKNCEDANGCDT |
| Q7M460 | DLEFASSECQMRYQDCGEASNCTALIEECKTSLQEECDQAS |
| P02701 | PGLSARKCSLTGKWTNDLGSNMTIGAVNSRGEFTGTYITAV |
| P27038 | GRSETQECLFFNANWERDRTNQTGVEPCYGDKDKRRHCFAT |
| P27038 | GVEPCYGDKDKRRHCFATWKNISGSIEIVKQGCWLDDINCY |
| Q13705 | GEAETRECIYYNANWELERTNQSGLERCEGEQDKRLHCYAS |
| Q13705 | GLERCEGEQDKRLHCYASWRNSSGTIELVKKGCWLDDFNCY |
| P56734 | VNSRGEFTGTYLTAVADNPGNITLSPLLGIQHKRASQPTFG |
| P56734 | LLGIQHKRASQPTFGFTVHWNFSESTTVFTGQCFIDRNGKE |
| P61769 | EAIQRTPKIQVYSRHPAENGKSNFLNCYVSGFHPSDIEVDL |
| P61769 | NFLNCYVSGFHPSDIEVDLLKNGERIEKVEHSDLSFSKDWS |
| P61769 | SGFHPSDIEVDLLKNGERIEKVEHSDLSFSKDWSFYLLYYT |
| P61769 | DLLKNGERIEKVEHSDLSFSKDWSFYLLYYTEFTPTEKDEY |
| P02730 | FFIQDTYTQKLSVPDGFKVSNSSARGWVIHPLGLRSEFPIW |
| O94766 | LESSLLSHLVDPKDLEPRAANCTRVLVWHTRTEKPKMKQEE |

| | |
|---|---|
| Q9NY97 | WNREQEKLNRQYNPILSMLTNQTGEAGRLSNISHLNYCEPD |
| Q9NY97 | QYNPILSMLTNQTGEAGRLSNISHLNYCEPDLRVTSVVTGF |
| Q9NY97 | TGFNNLPDRFKDFLLYLRCRNYSLLIDQPDKCAKKPFLLLA |
| Q9NY97 | PHFARRQAIRESWGQESNAGNQTVVRVFLLGQTPPEDNHPD |
| Q9NY97 | KFESEKHQDILMWNYRDTFFNLSLKEVLFLRWVSTSCPDTE |
| Q9Z222 | LLGILMMANVFIYLIVEVSKNSSQDKNGKGGVIIPKEKFWK |
| Q9Z222 | WNREQEKLNRWYNPILNRVANQTGELATSPNTSHLSYCEPD |
| Q9Z222 | WYNPILNRVANQTGELATSPNTSHLSYCEPDSTVMTAVTDF |
| Q9Z222 | TDFNNLPDRFKDFLLYLRCRNYSLLIDQPKKCAKKPFLLLA |
| Q9Z222 | PHFARRQAIRESWGRETNVGNQTVVRVFLLGKTPPEDNHPD |
| Q9Z222 | KFESDKHQDILMWNYRDTFFNLSLKEVLFLRWVSTSCPDAE |
| Q91YY2 | LFGRDPGPTFDYSHPHDVYSNLSHLPAAPGAAGAPPAQALP |
| Q91YY2 | HPFLQRQQLAYGIYVIHQAGNGTFNRAKLLNVGVREALRDE |
| Q91YY2 | DGMNSLTYRLLARELGPLYTNITADIGTDPRGPRSPSGPRY |
| P03228 | RGFFDIHRSANTFFLVVTAANISHDGNYLCRMKLGETEVTK |
| P0CW72 | RGFFDIHRSANTFFLVVTAANISHDGNYLCRMKLGETEVTK |
| P35613 | GTVFTTVEDLGSKILLTCSLNDSATEVTGHRWLKGGVVLKE |
| P35613 | PPVTDWAWYKITDSEDKALMNGSESRFFVSSSQGRSELHIE |
| P35613 | RSELHIENLNMEADPGQYRCNGTSSKGSDQAIITLRVRSHL |
| P18572 | GTIQTSVQEVNSKTQLTCSLNSSGVDIVGHRWMRGGKVLQE |
| P18572 | PITDWFWFKTSDTGEEEAITNSTEANGKYVVVSTPEKSQLT |
| P18572 | FWFKTSDTGEEEAITNSTEANGKYVVVSTPEKSQLTISNLD |
| P18572 | KSQLTISNLDVNVDPGTYVCNATNAQGTTRETISLRVRSRM |
| P18572 | LTISNLDVNVDPGTYVCNATNAQGTTRETISLRVRSRMAAL |
| P17790 | ERPVITGQYSSSADKVVLSCNISAPPTLIKGHKWMLGDKVL |
| P17790 | VEDHSGVYECIYNTNPVAKGNVSIEVEPQVVAYKKSEHGNE |
| P17790 | HWAWYKSGQTVPLESSAGIYNISRTGNKTELRILKLNIEQD |
| P17790 | SGQTVPLESSAGIYNISRTGNKTELRILKLNIEQDMGDYSC |
| P17790 | KTELRILKLNIEQDMGDYSCNGTNMKGSGSATVNLRVRSRL |
| P50895 | YTLFRLQDEQEEVLNVNLEGNLTLEGVTRGQSGTYGCRVED |
| P50895 | RVAYLDPLELSEGKVLSLPLNSSAVVNCSVHGLPTPALRWT |
| P50895 | PLELSEGKVLSLPLNSSAVVNCSVHGLPTPALRWTKDSTPL |
| P50895 | DSTPLGDGPMLSLSSITFDSNGTYVCEASLPTVPVLSRTQN |
| P50895 | NGTYVCEASLPTVPVLSRTQNFTLLVQGSPELKTAEIEPKA |
| P23560 | PLEPPLLFLLEEYKNYLDAANMSMRVRRHSDPARRGELSVC |
| Q700S9 | LGHPGGYTSYDYGSAISESRNITREKYSELKLLGNFAKVSP |
| Q700S9 | SDYSSQASVEYKLTVPTSAGNLTIPQLGGSLTLSGRDSKIH |
| Q700S9 | GYLVRSAYLDGNDLHIQADFNATTPIEVVGAPSGAKNLVIN |
| Q700S9 | PEAKNTYDDSAWTSADHAYTNNSAHSLQTPTSLFASDYGYH |
| Q700S9 | EKTFFVQTKGGTAYGHSIWINETYVGSWAGTSINDNNNATY |
| Q700S9 | IWINETYVGSWAGTSINDNNNATYTLPTLQSGKNYVITVVI |

| | |
|---|---|
| Q700S9 | TSFDLDLPSGYDIPLYFNFGNSTSTPAAYRVQLYVNGYQYG |
| P16278 | VRILPLLLVLLLLGPTRGLRNATQRMFEIDYSRDSFLKDGQ |
| P16278 | FLKCGALQGLYTTVDFGTGSNITDAFLSQRKCEPKGPLINS |
| P16278 | YVAVDGIPQGVLERNNVITLNITGKAGATLDLLVENMGRVN |
| P16278 | ENMGRVNYGAYINDFKGLVSNLTLSSNILTDWTIFPLDTED |
| P16278 | SHLGGWGHRDSGHHDEAWAHNSSNYTLPAFYMGNFSIPSGI |
| P16278 | GGWGHRDSGHHDEAWAHNSSNYTLPAFYMGNFSIPSGIPDL |
| P16278 | HDEAWAHNSSNYTLPAFYMGNFSIPSGIPDLPQDTFIQFPG |
| P08236 | ISNLVQVGPLPSRLRITIAINNTLTPTTLPPGTIQYLTDTS |
| P08236 | GSNLFKLEVRLLDAENKVVANGTGTQGQLKVPGVSLWWPYL |
| P08236 | GIVVIDECPGVGLALPQFFNNVSLHHHMQVMEEVVRRDKNH |
| O95972 | LRYMLELYRRSADSHGHPRENRTIGATMVRLVKPLTNVARP |
| O95972 | LYQLVRATVVYRHHLQLTRFNLSCHVEPWVQKNPTNHFPSS |
| O95972 | GLELWHGTSSLDIAFLLLYFNDTHKSIRKAKFLPRGMEEFM |
| P12643 | EESLEELPETSGKTTRRFFFNLSSIPTEEFITSAELQVFRE |
| P12643 | EFITSAELQVFREQMQDALGNNSSFHHRINIYEIIKPATAN |
| P12643 | FITSAELQVFREQMQDALGNNSSFHHRINIYEIIKPATANS |
| P12643 | ATANSKFPVTRLLDTRLVNQNASRWESFDVTPAVMRWTAQG |
| P12643 | PGYHAFYCHGECPFPLADHLNSTNHAIVQTLVNSVNSKIPK |
| Q96DR5 | ISNSLILDVKAEPIDDGKGLNLSFPVTANVTVAGPIIGQII |
| Q96DR5 | VKAEPIDDGKGLNLSFPVTANVTVAGPIIGQIINLKASLDL |
| Q8N4F0 | VPRLHLKFIAGFGVRLLAAANFTFKVFRAPEPLELTLPVEL |
| Q8N4F0 | VVSISACSLFSGHANEFDGSNSTSHALLVLVQKHIKAVLSN |
| Q8N4F0 | KAGALNLDITGQLRSDDNLLNTSALGRLIPEVARQFPEPMP |
| Q8N4F0 | MPVVLKVRLGATPVAMLHTNNATLRLQPFVEVLATASNSAF |
| O22476 | SSSLLSLTGLESLFLSNSHINGSVSGFKCSASLTSLDLSRN |
| O22476 | LSGPVTTLTSLGSCSGLKFLNVSSNTLDFPGKVSGGLKLNS |
| O22476 | SGDVDVSRCVNLEFLDVSSNNFSTGIPFLGDCSALQHLDIS |
| O22476 | NKLSGDFSRAISTCTELKLLNISSNQFVGPIPPLPLKSLQY |
| O22476 | AVPPFFGSCSLLESLALSSNNFSGELPMDTLLKMRGLKVLD |
| O22476 | LKVLDLSFNEFSGELPESLTNLSASLLTLDLSSNNFSGPIL |
| O22476 | LPESLTNLSASLLTLDLSSNNFSGPILPNLCQNPKNTLQEL |
| O22476 | LQELYLQNNGFTGKIPPTLSNCSELVSLHLSFNYLSGTIPS |
| O22476 | LETLILDFNDLTGEIPSGLSNCTNLNWISLSNNRLTGEIPK |
| O22476 | TGEIPKWIGRLENLAILKLSNNSFSGNIPAELGDCRSLIWL |
| O22476 | PAELGDCRSLIWLDLNTNLFNGTIPAAMFKQSGKIAANFIA |
| O22476 | LEFQGIRSEQLNRLSTRNPCNITSRVYGGHTSPTFDNNGSM |
| O22476 | NPCNITSRVYGGHTSPTFDNNGSMMFLDMSYNMLSGYIPKE |
| O22476 | RIPQAMSALTMLTEIDLSNNNLSGPIPEMGQFETFPPAKFL |
| P14518 | KTDGVPNSAYITGYARVPRNNESSMMYAVSKQPITVAVDAN |
| Q96G97 | FYYRTDCDSSTTSLCSFPVANVSLTKGGRDRVLMYGQPYRV |

| | |
|---|---|
| Q96G97 | GLRYLLYNFPMTCAFIGVASNFTFLSVIVLFSYMQWVWGGI |
| Q9GZN4 | VVGGEDSTDSEWPWIVSIQKNGTHHCAGSLLTSRWVITAAH |
| Q10588 | IFLGRCAEYRALLSPEQRNKNCTAIWEAFKVALDKDPCSVL |
| Q10588 | KVALDKDPCSVLPSDYDLFINLSRHSIPRDKSLFWENSHLL |
| Q10588 | PLSDVLYGRVADFLSWCRQKNDSGLDYQSCPTSEDCENNPV |
| Q10588 | WKRASIQYSKDSSGVIHVMLNGSEPTGAYPIKGFFADYEIP |
| Q10589 | TIKANSEACRDGLRAVMECRNVTHLLQQELTEAQKGFQDVE |
| Q10589 | QELTEAQKGFQDVEAQAATCNHTVMALMASLDAEKAQGQKK |
| Q13410 | PEPILAVVGEDAELPCRLSPNASAEHLELRWFRKKVSPAVL |
| Q13410 | DEEGLFTVAASVIIRDTSAKNVSCYIQNLLLGQEKKVEISI |
| Q8R2Q8 | AVTANSVACRDGLRAQAECRNTTHLLQRQLTRTQDSLLQAE |
| Q8R2Q8 | LLQRQLTRTQDSLLQAETQANSCNLTVVTLQESLEKKVSQA |
| Q8R2Q8 | RQLTRTQDSLLQAETQANSCNLTVVTLQESLEKKVSQALEQ |
| P18892 | QEPILAVVGEDAELPCRLSPNVSAKGMELRWFREKVSPAVF |
| P18892 | DEEGLFTVRASVIIRDSSMKNVSCCIRNLLLGQEKEVEVSI |
| Q7KYR7 | VSAQFIVVGPTDPILATVGENTTLRCHLSPEKNAEDMEVRW |
| Q7KYR7 | GRTTFVSKDISRGSVALVIHNITAQENGTYRCYFQEGRSYD |
| Q7KYR7 | SKDISRGSVALVIHNITAQENGTYRCYFQEGRSYDEAILHL |
| O00478 | GRTSILRDGITAGKAALRIHNVTASDSGKYLCYFQDGDFYE |
| O00481 | GRTSILRDGITAGKAALRIHNVTASDSGKYLCYFQDGDFYE |
| Q9XWH8 | FIWDQVDKYEVAGFSGQITVNYTTDSEGQKTRTVKVNLTDP |
| Q9XWH8 | QITVNYTTDSEGQKTRTVKVNLTDPGGEIWYNVSRSRYLST |
| Q9XWH8 | GQKTRTVKVNLTDPGGEIWYNVSRSRYLSTYYASYNYTLEP |
| Q9XWH8 | GEIWYNVSRSRYLSTYYASYNYTLEPQKRHMEAMELDKYFA |
| Q9XWH8 | VSSCFLSFHSTYFHDLFEYDNSTSLLNIEIPVEGVSYEDLG |
| Q9XWH8 | QTPYVLGLVENHLLTNTFAWNETLMLLADKYGLMRLLGKSI |
| P43251 | TAAQKDVQIIVFPEDGIHGFNFTRTSIYPFLDFMPSPQVVR |
| P43251 | DFMPSPQVVRWNPCLEPHRFNDTEVLQRLSCMAIRGDMFLV |
| P43251 | DPRCPKDGRYQFNTNVVFSNNGTLVDRYRKHNLYFEAAFDV |
| P43251 | KSHLIIAQVAKNPVGLIGAENATGETDPSHSKFLKILSGDP |
| P43251 | EATKWNVNAPPTFHSEMMYDNFTLVPVWGKEGYLHVCSNGL |
| P43251 | DTCGQEITEATGIFEFHLWGNFSTSYIFPLFLTSGMTLEVP |
| Q8CIF4 | AAAQKGVQIIVFPEDGIHGFNFTRTSIYPFLDFMPSPKLVR |
| Q8CIF4 | DFMPSPKLVRWNPCLEPFRFNDTEVLQRLSCMAIKGGMFLV |
| Q8CIF4 | DPGCPQDGRYQFNTNVVFSDNGTLVDRYRKHNLYFEAAFDT |
| Q8CIF4 | KGHLIIAQVATNPQGLTGTGNTTSEMDPSHRKFLKILSGDP |
| Q8CIF4 | EAAKWNVNVPPTFHSEMMYDNFTLVPVWGTEGHLQVCSNSL |
| Q8CIF4 | DTCGQEITEAEGLFDFHLWGNFSTLYIFPLFLTSGMTLDTP |
| Q86VB7 | VSVICNQLGCPTAIKAPGWANSSAGSGRIWMDHVSCRGNES |
| Q86VB7 | CRGNESALWDCKHDGWGKHSNCTHQQDAGVTCSDGSNLEMR |
| Q86VB7 | SWDLSDAHVVCRQLGCGEAINATGSAHFGEGTGPIWLDEMK |

| | |
|---|---|
| Q86VB7 | DCPARRWGHSECGHKEDAAVNCTDISVQKTPQKATTGRSSR |
| Q9NR16 | FSAVVTCILLLNSCFLISSFNGTDLELRLVNGDGPCSGTVE |
| Q9NR16 | SGTVEVKFQGQWGTVCDDGWNTTASTVVCKQLGCPFSFAMF |
| Q9NR16 | FGQAVTRHGKIWLDDVSCYGNESALWECQHREWGSHNCYHG |
| Q9NR16 | EDVGVNCYGEANLGLRLVDGNNSCSGRVEVKFQERWGTICD |
| Q9NR16 | LPHLQSGSDVVWLDGVSCSGNESFLWDCRHSGTVNFDCLHQ |
| Q9NR16 | VSVICSDGADLELRLADGSNNCSGRVEVRIHEQWWTICDQN |
| Q9NR16 | RAKPSNEARDIWINSISCTGNESALWDCTYDGKAKRTCFRR |
| Q9NR16 | MTYFKEASGPIWLDDVSCIGNESNIWDCEHSGWGKHNCVHR |
| Q9NR16 | AAVVCSQLDCPSSIIGMGLGNASTGYGKIWLDDVSCDGDES |
| Q9NR16 | GKYIGERSVRVWGHRFHCLGNESLLDNCQMTVLGAPPCIHG |
| Q9NR16 | NTVSVICTGSLTQPLFPCLANVSDPYLSAVPEGSALICLED |
| Q9NR16 | GWDLSDAHVVCQKLGCGVAFNATVSAHFGEGSGPIWLDDLN |
| Q9NR16 | NATVSAHFGEGSGPIWLDDLNCTGMESHLWQCPSRGWGQHD |
| Q9NR16 | LRLYSETETESCAGRLEVFYNGTWGSVGRRNITTAIAGIVC |
| Q9NR16 | SCAGRLEVFYNGTWGSVGRRNITTAIAGIVCRQLGCGENGV |
| Q9NR16 | DASFGQGTGTIWLDDMRCKGNESFLWDCHAKPWGQSDCGHK |
| Q9NR16 | DCGHKEDAGVRCSGQSLKSLNASSGHLALILSSIFGLLLLV |
| P02745 | MGGNVVIFDTVITNQEEPYQNHSGRFVCTVPGYYYFTFQVL |
| P98086 | TLGNVVIFDKVLTNQESPYQNHTGRFICAVPGFYYFNFQVI |
| Q8CG16 | FMSQGNKMLLTFHTDFSNEENGTIMFYKGFLAYYQAVDLDE |
| Q8CG16 | PSGYVSSLEYPQPYPPDLRCNYSIRVERGLTVHLKFLDPFE |
| Q8CG16 | LELENSVTLGPELLPICLPDNETFYGQGLMGYVSGFGITED |
| P00736 | FMSQGNKMLLTFHTDFSNEENGTIMFYKGFLAYYQAVDLDE |
| P00736 | ASGYISSLEYPRSYPPDLRCNYSIRVERGLTLHLKFLEPFD |
| P00736 | DRWILTAAHTLYPKEHEAQSNASLDVFLGHTNVEELMKLGN |
| P00736 | LELENSVTLGPNLLPICLPDNDTFYDLGLMGYVSGFGVMEE |
| Q9NZP8 | EFVSSGRSLRLTFRTQPSSENKTAHLHKGFLALYQTVAVNY |
| Q9NZP8 | ENKTAHLHKGFLALYQTVAVNYSQPISEASRGSEAINAPGD |
| Q9NZP8 | GEEVLQCMPVCGRPVTPIAQNQTTLGSSRAKLGNFPWQAFT |
| Q9NZP8 | RWILTAAHTIYPKDSVSLRKNQSVNVFLGHTAIDEMLKLGN |
| Q9NZP8 | LELQHSIPLGPNVLPVCLPDNETLYRSGLLGYVSGFGMEMG |
| P09871 | YFCSCPPEYFLHDDMKNCGVNCSGDVFTALIGEIASPNYPK |
| P09871 | CEEPYYYMENGGGGEYHCAGNGSWVNEVLGPELPKCVPVCG |
| Q16581 | YKFGLSSSLDYPDFYGDPLENRSLENIVQPPGEMNDRLDPS |
| P04003 | YSCDPRFSLLGHASISCTVENETIGVWRPSPPTCEKITCRK |
| P04003 | KPELVNGRLSVDKDQYVEPENVTIQCDSGYGVVGPQSITCS |
| P04003 | TIQCDSGYGVVGPQSITCSGNRTWYPEVPKCEWETPEGCEQ |
| P20851 | LGTYVCIKGYHLVGKKTLFCNASKEWDNTTTECRLGHCPDP |
| P20851 | KGYHLVGKKTLFCNASKEWDNTTTECRLGHCPDPVLVNGEF |
| P20851 | LGHCPDPVLVNGEFSSSGPVNVSDKITFMCNDHYILKGSNR |

| | |
|---|---|
| P20851 | VNVSDKITFMCNDHYILKGSNRSQCLEDHTWAPPFPICKSR |
| P20851 | CKSRDCDPPGNPVHGYFEGNNFTLGSTISYYCEDRYYLVGV |
| P08607 | VFGKCGPPPAIPNALPASDVNRTDFESHTTLKYECLPGYGR |
| P08607 | YTCDPGFRLVGSPFIGCTVVNKTVPVWSSSPPTCEKIICSQ |
| P08607 | IVSGYKATYTHRDSVRLACLNGTVLRGRHVIECQGNGNWSS |
| P08607 | ACLNGTVLRGRHVIECQGNGNWSSLPTCEFDCDLPPAIVNG |
| P08607 | SKWKGTAPQCKALCQKPEVGNGTLSDEKDQYVESENVTIQC |
| P08607 | KPEVGNGTLSDEKDQYVESENVTIQCDSGFAMLGSQSISCS |
| P08607 | PEVPRCEQEASEDLKPALTGNKTMQYVPNSHDVKMALEIYK |
| O08532 | PNNARQLVEIAARDIEKLLSNRSKALVRLAMEAEKVQAAHQ |
| O08532 | DFASNEVVYYNAKDDLDPERNESEPGSQRIKPVFIEDANFG |
| O08532 | AAVHIPTDIYEGSTIVLNELNWTSALDEVFKRNRDEDPTLL |
| O08532 | CFQHLVQANVRNKKVLKDAVNNITAKGITDYKKGFSFAFEQ |
| O08532 | FQHLVQANVRNKKVLKDAVNNITAKGITDYKKGFSFAFEQL |
| O08532 | KGITDYKKGFSFAFEQLLNYNVSRANCNKIIMLFTDGGEER |
| O08532 | KSQDERYIDKGNRTYTWTPVNGTDYSLALVLPTYSFYYIKA |
| O08532 | WQENPETYEDSFYKRSLDNDNYVFTAPYFNKSGPGAYESGI |
| O08532 | DSFYKRSLDNDNYVFTAPYFNKSGPGAYESGIMVSKAVELY |
| O08532 | TNQIGRFFGEIDPSMMRHLVNISLYAFNKSYDYQSVCDPGA |
| Q9NY47 | KGTTGYKAGFEYAFDQLQNSNITRANCNKMIMMFTDGGEDR |
| Q9NY47 | MFTDGGEDRVQDVFEKYNWPNRTVRVFTFSVGQHNYDVTPL |
| Q9NY47 | NVYEDALGLGLVVTGTLPVFNLTQDGPGEKKNQLILGVMGI |
| Q9NY47 | LILGVMGIDVALNDIKRLTPNYTLGANGYVFAIDLNGYVLL |
| Q9NY47 | KQIRTLVKSLDERYIDEVTRNYTWVPIRSTNYSLGLVLPPY |
| Q9NY47 | VVGVKLDLEAWAEKFKVLASNRTHQDQPQKCGPNSHCEMDC |
| P55290 | QQKVFHINQPAEFIEDQSILNLTFSDCKGNDKLRYEVSSPY |
| P55290 | YEVSSPYFKVNSDGGLVALRNITAVGKTLFVHARTPHAEDM |
| P55290 | KFTKKEFQATVEEGAVGVIVNLTVEDKDDPTTGAWRAAYTI |
| P55290 | DPMMVTRQEDLSVGSVLLTVNATDPDSLQHQTIRYSVYKDP |
| P55290 | QTIRYSVYKDPAGWLNINPINGTVDTTAVLDRESPFVDNSV |
| P55290 | VNDNAPFIYPTVAEVCDDAKNLSVVILGASDKDLHPNTDPF |
| P55290 | FKFEIHKQAVPDKVWKISKINNTHALVSLLQNLNKANYNLP |
| P55290 | NKANYNLPIMVTDSGKPPMTNITDLRVQVCSCRNSKVDCNA |
| P33146 | VDEEPRNVFSIDKFTGRVYLNATLDREKTDRFRLRAFALDL |
| P33146 | HTGEIRTVQVGLDREVVAVYNLTLQVADMSGDGLTATASAI |
| P33146 | PPHGAPFHFQLNPRVPDLGRNWSVSQINVSHARLRLRHQVS |
| P33146 | HFQLNPRVPDLGRNWSVSQINVSHARLRLRHQVSEGLHRLS |
| P33146 | RLSLLLQDSGEPPQQREQTLNVTVCRCGSDGTCLPGAAALR |
| Q9VGG5 | GETNLPPVFTQTLNNIILYENVTVGTVVFRLEAYDPEGSPV |
| Q9VGG5 | SPVTYGAIGADHFSVDPVSGNITLIKPLDREEKDTLKFLVS |
| Q9VGG5 | KFRLHIIKRDATILEAAVVLNDTLNYNQRMVYHFQIEATDG |

| | |
|---|---|
| Q9VGG5 | VFDVEPKLVTGYSQVNIRVANGTLDYENPNQRKFIVLVVAE |
| Q9VGG5 | EQVESIFDIDRETGQIIIRPNATLDVTNLNSDQLIFAVEAN |
| Q9VGG5 | DQLIFAVEANDGLFTAHCGVNITVRDVNNHVPNFEQQSYSA |
| Q9VGG5 | VGTVLTTLQANDEDSSIGEFNISDNDYFAINQTSGMIYTIA |
| Q9VGG5 | NDEDSSIGEFNISDNDYFAINQTSGMIYTIARLDYEVVKEV |
| Q9VGG5 | VDIINLNDNDPKFSQSDYYFNVTENSPRGTVAGKVEAHDGD |
| Q9VGG5 | IGENNKYFSIDAYTGNVMVANSSILDREQIKELTLSVVAQD |
| Q9VGG5 | IHINILDVNDNAPVFTRDVYNSTVAENAAYQPPAALLQVQA |
| Q9VGG5 | IITVLRVNRHKPEFVIPALSNATIEIPGDIVQPDYLLLTVR |
| Q9VGG5 | EIPGDIVQPDYLLLTVRAMDNDTEENGKVSYHLQVNNRNEQ |
| Q9VGG5 | LLGNEDGAFYVDKLTGDIYTNKSLDREETDVYTLYILASIK |
| Q9VGG5 | PIYYAGVNANAKMGAAITLVNATDADQGKNAKIEFMIVASN |
| Q9VGG5 | LSRPPEEVYQEQEEIIAELRNATQHRIIVDEIRFHLDSIGR |
| P08641 | EVFVGYIEENAKPGTSVMTVNATDADDAVNTDNGIVSYSIV |
| P08641 | DPAKGIISVLGTGLDRETTPNYTLIVQATDQEGKGLSNTAT |
| P08641 | PENGIVTATQPLDRESVHAINSTYKAIILAVDNGIPDTTGT |
| P08641 | KDLPPHTYPFKAALEHGSSNNWTVEIRGQDELAMGLKKELE |
| P12830 | DTGAISTRAELDREDFEHVKNSTYTALIIATDNGSPVATGT |
| P12830 | REDFEHVKNSTYTALIIATDNGSPVATGTGTLLLILSDVND |
| P12830 | FCERNPKPQVINIIDADLPPNTSPFTAELTHGASANWTIQY |
| P12830 | ADLPPNTSPFTAELTHGASANWTIQYNDPTQESIILKPKMA |
| P19022 | ENSRGPFPQELVRIRSDRDKNLSLRYSVTGPGADQPPTGIF |
| P19022 | IVINVIDMNDNRPEFLHQVWNGTVPEGSKPGTYVMTVTAID |
| P19022 | RYRIVSQAPSTPSPNMFTINNETGDIITVAAGLDREKVQQY |
| P19022 | FTAMTFYGEVPENRVDIIVANLTVTDKDQPHTPAWNAVYRI |
| P19022 | QITTIAVLDRESPNVKNNIYNATFLASDNGIPPMSGTGTLQ |
| P19022 | DPNAGPFAFDLPLSPVTIKRNWTITRLNGDFAQLNLKIKFL |
| P19022 | EAGIYEVPIIITDSGNPPKSNISILRVKVCQCDSNGDCTDV |
| Q19319 | SLVTTSSSTEQIFEFTAPLYNLSVEENSIGSKYARSENSTK |
| Q19319 | PLYNLSVEENSIGSKYARSENSTKIGVPLPEKDANCKFRVA |
| Q19319 | RVEASDADIGINSAIYFSLVNRSHDFIVEPVTGWVRSLRHV |
| Q19319 | EPEVVGNDGTKELRWMLYAKNGSQVPKNTNVTLTIGEDYIR |
| Q19319 | TKELRWMLYAKNGSQVPKNTNVTLTIGEDYIRRSGFSISKK |
| Q19319 | GEDYIRRSGFSISKKPVIPTNETVTIQIERLAEHLIRFLDN |
| Q19319 | NPDDVSLIRYSLEYSKSDLPNATLPFAIGSKNGILRVSAKI |
| Q19319 | ATLPFAIGSKNGILRVSAKINRSERVYNFKVIASLHGINEK |
| Q19319 | AIDLGLPLSRMSALNLMFYKNGTKIPAKPKPIIIQESENKH |
| Q19319 | VHTFAIIASDEGFPMRVSVTNLTISVEDVNDNPPKCVVQHS |
| Q19319 | GCIFIHAPEYPLDYHKTPFFNLSIEVADHGDPILSTSCHLH |
| Q19319 | KLCRIFAVDADEGENARLTYNITEGDARFSIDNNGNIIASE |
| Q19319 | TGQLLLAKKVELLKRGELRLNISVTDGQAWDHSTVIIQVSR |

| | |
|---|---|
| Q19319 | TPVLVLNALHHGTVSYKLEPNCTFFEVHTLSGAVHLATWLT |
| Q19319 | STENSKFKFTRNEYHTSLIENTTLPPGSIILSVATIGDKLD |
| Q19319 | KLPSIEEEDLEFNFKLIAKDNGSVHRVEEPVKIRVVDKARP |
| Q19319 | SVDFLTGEVRLNNLKMLAETNYTFEVEAREVTRPKMIAKAQ |
| Q19319 | PESTSIGQRLLTIKATTSDENDTIEYSLSGSKDIEIHPETG |
| Q19319 | SLARPVEQDDLVNEDTKKVLNVSVTDGIIVNKSALLTVAVK |
| Q19319 | LVNEDTKKVLNVSVTDGIIVNKSALLTVAVKGGVPPLQFSL |
| Q19319 | VAVKGGVPPLQFSLAPTTSSNSSTSKEAWPVAIDRKTGRIH |
| Q19319 | SESAKEGDTVMMVSATDDDENDTIEYSLLDGSESQFFSVHP |
| Q19319 | LPSTSPILHFSIKDDDLSPNNVSQFFIPKGNEEGVFWIDSN |
| Q19319 | TKVKINLKSSKDSDIRCPEANKTVILAENSKKGTVVLGESS |
| Q19319 | LAENSKKGTVVLGESSLLGPNVTFKLSDNDGNVFVVNFRNG |
| Q19319 | DINGVVTVVKPLDSEILGFFNLTVVASDGEFEDKATILVTV |
| Q19319 | MESESIGYELAHFRASGGDQNETIEYYLKPSDVTSFVNLNA |
| Q19319 | ALLEIHVEDENDHAPTFDDCNMTALVQEGEAIGHRLLKFSV |
| Q19319 | DPNTGDIWSDHSITQGLHTFNVTVTDSKFNTVSYVEVHVTS |
| Q19319 | TNQCAKSPCEQWQLCIPSVHNSTYECVCPLGMEGDKCSVPS |
| Q19319 | TSGQCISIPRHSLESSDFICNCTGGILQSTPCAEKSDILST |
| P33151 | THRRQKRDWIWNQMHIDEEKNTSLPHHVGKIKSSVSRKNAK |
| P33151 | VFRVDAETGDVFAIERLDRENISEYHLTAVIVDKDTGENLE |
| P33151 | FTIKVHDVNDNWPVFTHRLFNASVPESSAVGTSVISVTAVD |
| P33151 | PTIDLRYMSPPAGNRAQVIINITDVDEPPIFQQPFYHFQLK |
| P33151 | TKKGDIYNEKELDREVYPWYNLTVEAKELDSTGTPTGKESI |
| P33151 | IDKDITPRNVKFKFILNTENNFTLTDNHDNTANITVKYGQF |
| P33151 | KFILNTENNFTLTDNHDNTANITVKYGQFDREHTKVHFLPV |
| P55285 | SGFPAKKRALELSGNSKNELNRSKRSWMWNQFFLLEEYTGS |
| P55285 | VIQAKDMGGQMGGLSGTTTVNITLTDVNDNPPRFPQSTYQF |
| P55285 | EPPVFSKLAYILQIREDAQINTTIGSVTAQDPDAARNPVKY |
| P55285 | VKYSVDRHTDMDRIFNIDSGNGSIFTSKLLDRETLLWHNIT |
| P55285 | SGNGSIFTSKLLDRETLLWHNITVIATEINNPKQSSRVPLY |
| P55285 | DPYSGHQFSFSLAPEAASGSNFTIQDNKDNTAGILTRKNGY |
| P33148 | EYIFSVNRRELERGRKLGKVNFSDCTTRKHGLYDVGDSRFR |
| P33148 | PGTPAWQAVYKIRVNEGGFFNITTDPESNQGILTTAKGLDF |
| P33148 | KDNGIVTGNGNLDRESEYVKNNTYTVIMLVTDDGVSVGTGT |
| P33148 | DYSIYVLLSDAQNNPQLTVVNATVCSCEGKAIKCQEKLVGG |
| Q9BY67 | LFTKDVTVIEGEVATISCQVNKSDDSVIQLLNPNRQTIYFR |
| Q9BY67 | RQTIYFRDFRPLKDSRFQLLNFSSSELKVSLTNVSISDEGR |
| Q9BY67 | KDSRFQLLNFSSSELKVSLTNVSISDEGRYFCQLYTDPPQE |
| Q9BY67 | RNLMIDIQKDTAVEGEEIEVNCTAMASKPATTIRWFKGNTE |
| Q9BY67 | VRVDDEMPQHAVLSGPNLFINNLNKTDNGTYRCEASNIVGK |
| Q9BY67 | RVDDEMPQHAVLSGPNLFINNLNKTDNGTYRCEASNIVGKA |

| | |
|---|---|
| Q9BY67 | DDEMPQHAVLSGPNLFINNLNKTDNGTYRCEASNIVGKAHS |
| Q9BY67 | PQHAVLSGPNLFINNLNKTDNGTYRCEASNIVGKAHSDYML |
| Q8R5M8 | LFTKDVTVIEGEVATISCQVNKSDDSVIQLLNPNRQTIYFR |
| Q8R5M8 | RQTIYFRDFRPLKDSRFQLLNFSSSELKVSLTNVSISDEGR |
| Q8R5M8 | KDSRFQLLNFSSSELKVSLTNVSISDEGRYFCQLYTDPPQE |
| Q8R5M8 | RNLMIDIQKDTAVEGEEIEVNCTAMASKPATTIRWFKGNKE |
| Q8R5M8 | VRVDDEMPQHAVLSGPNLFINNLNKTDNGTYRCEASNIVGK |
| Q8R5M8 | DDEMPQHAVLSGPNLFINNLNKTDNGTYRCEASNIVGKAHS |
| Q8R5M8 | PQHAVLSGPNLFINNLNKTDNGTYRCEASNIVGKAHSDYML |
| Q8BLQ9 | FYSVCGLLLQGSQGQFPLTQNVTVVEGGTAILTCRVDQNDN |
| Q8BLQ9 | NVTVVEGGTAILTCRVDQNDNTSLQWSNPAQQTLYFDDKKA |
| Q8BLQ9 | PDRMVVSGRELNILFLNKTDNGTYRCEATNTIGQSSAEYVL |
| Q8N126 | EGSVPPLKMTQESALIFPFLNKSDSGTYGCTATSNMGSYKA |
| O75493 | GTLYNTGRHVSFLPAPRPVVNVSGGPLLYSHRLSELRLLFG |
| O75493 | NHQGFSAEVQLIHFNQELYGNFSAASRGPNGLAILSLFVNV |
| O75493 | SLSTPPCSETVTWILIDRALNITSLQMHSLRLLSQNPPSQI |
| Q9WVT6 | VPPFSVRELFPQQLEQFFRYNGSLTTPPCYQSVLWTVFNRR |
| Q9ULX7 | VPPFNLRELLPKQLGQYFRYNGSLTTPPCYQSVLWTVFYRR |
| P20507 | KGSKIANGLQTQWSYPDLMSNGTSVQVINNGHTIQVQWTYN |
| P20507 | QVQWTYNYAGHATIAIPAMHNQTNRIVDVLEMRPNDAADRV |
| P20507 | QRISFGQWNRYRLAVGLKECNSTETAADAGHHHHHRRLLHN |
| Q18932 | VELRRLTVASQSINYKGQTTNLTDFQPSALLPKTSHYVTYE |
| P18915 | QSPIDLKMKKVRYNPSLRALNLTGYGLRQGEFPMTNNGHTV |
| P18915 | TVKLSKTQIEKLENSLLNHQNETIQNNYRSTQPLNHRVVEA |
| P08060 | QSPIDLQMKKVQYNPSLRALNLTGYGLWHGEFPVTNNGHTV |
| P08060 | TVKLSKTQVEKLENSLLNHQNKTIQNDYRRTQPLNHRVVEA |
| Q16790 | QYEGSLTTPPCAQGVIWTVFNQTVMLSAKQLHTLSDTLWGP |
| Q8IU99 | LDGKCFLCAFCTAVPVSALGNGSLAPGLPAPELARLLARVP |
| P52193 | KDIRCKDDEFTHLYTLIVRPNNTYEVKIDNSQVESGSLEDD |
| P27797 | GTIFDNFLITNDEAYAEEFGNETWGVTKAAEKQMKDKQDEE |
| O43852 | RQWKGHDLNEDGLVSWEEYKNATYGYVLDDPDPDDGFNYKQ |
| P34652 | PDKCGATGKVHLIFRYKNPINGTISEYHANQPTTIGSTYWD |
| P34652 | GSQSNPEPQDEEENAEQQSANSSQSSAAEEEDDEHVVPENE |
| P80015 | QLDREARLTPSVALVPLPPQNATVEAGTNCQVAGWGTQRLR |
| P80015 | VAGWGTQRLRRLFSRFPRVLNVTVTSNPCLPRDMCIGVFSR |
| P20160 | GYDPQQNLNDLMLLQLDREANLTSSVTILPLPLQNATVEAG |
| P20160 | QLDREANLTSSVTILPLPLQNATVEAGTRCQVAGWGSQRSG |
| P20160 | VAGWGSQRSGGRLSRFPRFVNVTVTPEDQCRPNNVCTGVLT |
| Q9XFX3 | SKACRAHSMYESSDSSTYKENGTFGAIIYGTGSITGFFSQD |
| Q9XFX3 | HLSTSSEELQVDCNTLSSMPNVSFTIGGKKFGLTPEQYILK |
| P09177 | VFSVYMNTNDGGGQVVFGGANNTLLGGDIQYTDVLKSRGGY |

| | |
|---|---|
| P07267 | SLACFLHSKYDHEASSSYKANGTEFAIQYGTGSLEGYISQD |
| P07267 | TLDCNTRDNLPDLIFNFNGYNFTIGPYDYTLEVSGSCISAI |
| P00799 | GSRFFDPSASSTFKATNYNLNITYGTGGANGLYFEDSIAIG |
| P00799 | LFSVYMNTNSGTGEVVFGGVNNTLLGGDIAYTDVMSRYGGY |
| P07221 | VPYWEKTFDIDLSAPQIGVVNVTDADSVWMEMDDEEDLPSA |
| P07688 | LYNSHVGCRPYSIPPCEHHVNGSRPPCTGEGDTPKCSKTCE |
| P07858 | LYESHVGCRPYSIPPCEHHVNGSRPPCTGEGDTPKCSKICE |
| A1E295 | LYDSHVGCRPYSIPPCEHHVNGSRPPCTGEGDTPKCSKICE |
| P00787 | VYNSHIGCLPYTIPPCEHHVNGSRPPCTGEGDTPKCNKMCE |
| P53634 | LAALLLLLSGDGAVRCDTPANCTYLDLLGTWVFQVGSSGSQ |
| P53634 | LDLLGTWVFQVGSSGSQRDVNCSVMGPQEKKVVVYLQKLDT |
| P53634 | YKWFAFFKYKEEGSKVTTYCNETMTGWVHDVLGRNWACFTG |
| P53634 | GSCYSFASMGMLEARIRILTNNSQTPILSPQEVVSCSQYAQ |
| P80067 | RAALLLVLLGVCTVSSDTPANCTYPDLLGTWVFQVGPRHPR |
| P80067 | PDLLGTWVFQVGPRHPRSHINCSVMEPTEEKVVIHLKKLDT |
| P80067 | GWVHDVLGRNWACFVGKKMANHSEKVYVNVAHLGGLQEKYS |
| P80067 | GSCYSFASLGMLEARIRILTNNSQTPILSPQEVVSCSPYAQ |
| P07339 | DIACWIHHKYNSDKSSTYVKNGTSFDIHYGSGSLSGYLSQD |
| P07339 | GELMLGGTDSKYYKGSLSYLNVTRKAYWQVHLDQVEVASGL |
| P18242 | DIACWVHHKYNSDKSSTYVKNGTSFDIHYGSGSLSGYLSQD |
| P18242 | GELMLGGTDSKYYHGELSYLNVTRKAYWQVHMDQLEVGNEL |
| P00795 | DIACWIHHKYNSGKSSTYVKNGTTFAIHYGSGSLSGYLSSQ |
| P00795 | GELMLGGIDSKYYKGSLDYHNVTRKAYWQIHMNQVAVGSSL |
| P14091 | LINYLDMEYFGTISIGSPPQNFTVIFDTGSSNLWVPSVYCT |
| Q9UBX1 | AFTQGSAMISSLSQNHPDNRNETFSSVISLLNEDPLSQDLP |
| Q9UBX1 | LSQDLPVKMASIFKNFVITYNRTYESKEEARWRLSVFVNNM |
| Q9UBX1 | NLGGLETEDDYSYQGHMQSCNFSAEKAKVYINDSVELSQNE |
| Q9UBX1 | SYQGHMQSCNFSAEKAKVYINDSVELSQNEQKLAAWLAKRG |
| Q9UBX1 | LRPLCSPWLIDHAVLLVGYGNRSDVPFWAIKNSWGTDWGEK |
| P08311 | GFLVREDFVLTAAHCWGSNINVTLGAHNIQRRENTQQHITA |
| P09668 | QFSDMSFAEIKHKYLWSEPQNCSATKSNYLRGTGPYPPSVD |
| P09668 | KDGYCKFQPGKAIGFVKDVANITIYDEEAMVEAVALYNPVS |
| P00786 | KNGQCKFNPEKAVAFVKNVVNITLNDEAAMVEAVALYNPVS |
| P09648 | YPYTAKDDEDCRYKAEYNAANDTGFVDIPQGHERALMKAVA |
| Q9GKL8 | SYPYEATEESCKYNPEYSVANDTGFVDIPKQEKALMKAVAT |
| P07711 | SYPYEATEESCKYNPKYSVANDTGFVDIPKQEKALMKAVAT |
| P06797 | SYPYEAKDGSCKYRAEFAVANDTGFVDIPQQEKALMKAVAT |
| P07154 | SYPYEAKDGSCKYRAEYAVANDTGFVDIPQQEKALMKAVAT |
| O60911 | SYPYVAVDEICKYRPENSVANDTGFTVVAPGKEKALMKAVA |
| O60911 | SKNLDHGVLVVGYGFEGANSNNSKYWLVKNSWGPEWGSNGY |
| Q9UBR2 | CDKFNQCGTCNEFKECHAIRNYTLWRVGDYGSLSGREKMMA |

| | |
|---|---|
| Q9UBR2 | AEIYANGPISCGIMATERLANYTGGIYAEYQDTTYINHVVS |
| Q06770 | LAMLSLSTRGSTQYLENLGFNMSKMSEAEIHQGFQYLNSLL |
| Q06770 | LTIPSKDWTKAGEQINNHVKNKTQGKIEHVVSDLDSSATLI |
| Q06770 | KGIWKLPFSPENTREEDFYVNETSTVKVPMMVQSGNISYFR |
| Q06770 | EDFYVNETSTVKVPMMVQSGNISYFRDSAIPCQMVQMNYVG |
| Q06770 | ISYFRDSAIPCQMVQMNYVGNGTTFIILPDQGQMDTVVAAL |
| Q06770 | SDTYDLQDVLADVGIKDLFTNQSDFADTTKDTPLTLTVLHK |
| P08185 | WLPTSGLWTVQAMDPNAAYVNMSNHHRGLASANVDFAFSLY |
| P08185 | MLSLGTCGHTRAQLLQGLGFNLTERSETEIHQGFQHLHQLF |
| P08185 | LAMNFQDWATASRQINSYVKNKTQGKIVDLFSGLDSPAILV |
| P08185 | ISYLHDSELPCQLVQMNYVGNGTVFFILPDKGKMNTVIAAL |
| P08185 | YDLGDVLEEMGIADLFTNQANFSRITQDAQLKSSKVVHKAV |
| P08185 | AVLQLNEEGVDTAGSTGVTLNLTSKPIILRFNQPFIIMIFD |
| Q9R171 | GVVELLLLGTAWLAGPARGQNETEPIVLEGKCLVVCDSNPT |
| Q9R171 | SAKVAFSAIRSTNHEPSEMSNRTMIIYFDQVLVNIGNNFDS |
| Q8BME9 | RALSVVPAVLLILVLPVWAQNDTEPIVLEGKCLVVCDSNPA |
| Q8BME9 | NSKVAFSAVRSTNHEPSEMSNKTRIIYFDQILVNVGNFFTL |
| Q8BGU2 | GAVLALLLLLLPACCPVRAQNDTEPIVLEGKCLVVCDSSPS |
| Q8BGU2 | SAKVAFSATRSTNHEPSEMSNRTMTIYFDQVLVNIGNHFDL |
| P07519 | KVSTMIYLDSPAGVGLSYSKNVSDYETGDLKTATDSHTFLL |
| P07519 | GLISDEIYQQASTSCHGNYWNATDGKCDTAISKIESLISGL |
| P07519 | LCTDKLYFVHDAGSMIAYHKNLTSQGYRAIIFSGDHDMCVP |
| P08818 | NKVANVLFLDSPAGVGFSYTNTSSDIYTSGDNRTAHDSYAF |
| P08818 | PAGVGFSYTNTSSDIYTSGDNRTAHDSYAFLAAWFERFPHY |
| P08818 | DVATAEQGNIDMYSLYTPVCNISSSSSSSSLSRRRTRGRYP |
| P08818 | TERYSTAYYNRRDVQTALHANVTGAMNYTWTNCSDTINTHW |
| P08818 | AYYNRRDVQTALHANVTGAMNYTWTNCSDTINTHWHDAPRS |
| P08818 | RDVQTALHANVTGAMNYTWTNCSDTINTHWHDAPRSMLPIY |
| P21529 | PGCSSELAVFYENGPFTIANNMSLVWNKFGWDKISNIIFVD |
| P08819 | NKVANVLFLDSPAGVGFSYTNTSSDIYTSGDNRTAHDSYAF |
| P08819 | PAGVGFSYTNTSSDIYTSGDNRTAHDSYAFLAKWFERFPHY |
| P08819 | DVATAEQGNIDMYSLYTPVCNITSSSSSSSSLSQQRRSRG |
| P08819 | TERYSTAYYNRRDVQMALHANVTGAMNYTWATCSDTINTHW |
| P08819 | AYYNRRDVQMALHANVTGAMNYTWATCSDTINTHWHDAPRS |
| Q9UI42 | KTRSRNPGSSCIGADPNRNWNASFAGKGASDNPCSEVYHGP |
| Q2KIG3 | QRGQVLSALPRTSRQVQILQNVTTTYKIVLWQPVAAEYIVK |
| Q2KIG3 | LWQPVAAEYIVKGYEVHFFVNASDVSNVKAHLNASRIPFRV |
| Q2KIG3 | GYEVHFFVNASDVSNVKAHLNASRIPFRVLVENVEDLIRQQ |
| Q2KIG3 | RIPFRVLVENVEDLIRQQTSNDTISPRASSSYYEQYHSLNE |
| Q2KIG3 | PVVNVDGYDYTWKKDRMWRKNRSLHEKNACVGTDLNRNFAS |
| Q96IY4 | QSGQVLAALPRTSRQVQVLQNLTTTYEIVLWQPVTADLIVK |

| | |
|---|---|
| Q96IY4 | LWQPVTADLIVKKKQVHFFVNASDVDNVKAHLNVSGIPCSV |
| Q96IY4 | KKQVHFFVNASDVDNVKAHLNVSGIPCSVLLADVEDLIQQQ |
| Q96IY4 | GIPCSVLLADVEDLIQQQISNDTVSPRASASYYEQYHSLNE |
| Q96IY4 | PVVNVDGYDYSWKKNRMWRKNRSFYANNHCIGTDLNRNFAS |
| Q9JHH6 | QSGQVLSALPRTSRQVQLLQNLTTTYEVVLWQPVTAEFIEK |
| Q9JHH6 | LWQPVTAEFIEKKKEVHFFVNASDVDSVKAHLNVSRIPFNV |
| Q9JHH6 | KKEVHFFVNASDVDSVKAHLNVSRIPFNVLMNNVEDLIEQQ |
| Q9JHH6 | RIPFNVLMNNVEDLIEQQTFNDTVSPRASASYYEQYHSLNE |
| Q9JHH6 | PVMNVDGYDYTWKKNRMWRKNRSAHKNNRCVGTDLNRNFAS |
| Q9JHH6 | IKAYISMHSYSQQILFPYSYNRSKSKDHEELSLVASEAVRA |
| P42787 | YMAQYLLGNHERISDLGQLVNSTDIYLVPTMNPDGYALSQE |
| P42787 | KQLAHTYSDNHPIMRKGNNCNDSFSGGITNGAHWYELSGGM |
| P42787 | PQKLKKQFNGFLTPTKYEHHNFTAMESYLRAISSSYPSLTR |
| P42787 | ILTKYMLERYGNDDRITKLVNGTRMHFLYSMNPDGYEISIE |
| P42787 | DQYGTDRFNKVTEPEVAAVMNWTLSLPFVLSANLHGGSLVA |
| P42787 | ANYPFDDNENDFNDPFMRLRNSSINGRKPNPTEDNALFKHL |
| P42787 | FVHSTIGTPIAGAVVRLDGANHSTYSQVFGDYWKLALPGRH |
| P42787 | HSTYSQVFGDYWKLALPGRHNLTVLGDNYAPLRMEVEVPDV |
| P42787 | VIYFLPQTSKFQNVFDMYNSNTSICDPVLGDELAERILGPE |
| P42787 | DKGQPLREAYVRLLEHDRIINVTKNVARFQLMLPHGLYGLE |
| P42787 | ESNHPVRNAKVSVVGQTQLRNFTGSMGQYRISAVPLGTITL |
| O75976 | LARELAAGYRRGDPRLVRLLNTTDVYLLPSLNPDGFERARE |
| O75976 | FGDGGPSGASGRDNSRGRDLNRSFPDQFSTGEPPALDEVPE |
| O75976 | KVHIGVKGFVKDSITGSGLENATISVAGINHNITTGRFGDF |
| O75976 | DSITGSGLENATISVAGINHNITTGRFGDFYRLLVPGTYNL |
| O75976 | HNITTGRFGDFYRLLVPGTYNLTVVLTGYMPLTVTNVVVKE |
| O75976 | HHHHFPDMEIFLRRFANEYPNITRLYSLGKSVESRELYVME |
| O75976 | MNPDGYEKSQEGDSISVIGRNNSNNFDLNRNFPDQFVQITD |
| O75976 | QVHQGVRGFVLDATDGRGILNATISVAEINHPVTTYKTGDY |
| O75976 | LVPGTYKITASARGYNPVTKNVTVKSEGAIQVNFTLVRSST |
| O75976 | RGYNPVTKNVTVKSEGAIQVNFTLVRSSTDSNNESKKGKGA |
| O75976 | KSEGAIQVNFTLVRSSTDSNNESKKGKGASSSTNDASDPTT |
| O75976 | SYKDLSEFLRGLVMNYPHITNLTNLGQSTEYRHIWSLEISN |
| O75976 | NLGQSTEYRHIWSLEISNKPNVSEPEEPKIRFVAGIHGNAP |
| O75976 | TSKIGQTNARGKDLDTDFTNNASQPETKAIIENLIQKQDFS |
| O75976 | LASLYANNHPSMHMGQPSCPNKSDENIPGGVMRGAEWHSHL |
| P83852 | MNPDGYEKSQEGDRGGTVGRNNSNNYDLNRNFPDQFFQVTD |
| P83852 | QVHRGIWGFVLDATDGRGILNATISVADINHPVTTYKDGDY |
| O89001 | LARELASGYRRGDPRLVRLLNTTDVYLLPSLNPDGFERARE |
| O89001 | LGDSGPPGTSGRDNSRGRDLNRSFPDQFSTGEPPSLDEVPE |
| O89001 | KVHIGIKGFVKDSVTGSGLENATISVAGINHNITTGRFGDF |

| | |
|---|---|
| O89001 | DSVTGSGLENATISVAGINHNITTGRFGDFHRLLVPGTYNL |
| O89001 | HNITTGRFGDFHRLLVPGTYNLTALSTGYMPLTINNIMVKE |
| O89001 | HHHHFPDMEIFLRRFANEYPNITRLYSLGKSVESRELYVME |
| O89001 | MNPDGYEKSQEGDSISVVGRNNSNNFDLNRNFPDQFVPITE |
| O89001 | QVHQGVKGFVLDATDGRGILNATLSVAEINHPVTTYKAGDY |
| O89001 | LVPGTYKITASARGYNPVTKNVTVRSEGAVQVNFTLVRSSA |
| O89001 | RGYNPVTKNVTVRSEGAVQVNFTLVRSSADANNESKKGRGH |
| O89001 | RSEGAVQVNFTLVRSSADANNESKKGRGHSTSTDDTSDPTS |
| O89001 | SYKDLSEFLRGLVMNYPHITNLTTLGQSVEYRHIWSLEISN |
| O89001 | TLGQSVEYRHIWSLEISNKPNISEPEEPKIRFVAGIHGNAP |
| O89001 | TSKTGHTNAHGKDLDTDFTSNASQPETKAIIENLIQKQDFS |
| O89001 | LASLYANNHPSMHMGQPSCPNNSDENIPGGVMRGAEWHSHL |
| P14384 | LDFNYHRQEGMEAFLKTVAQNYSSVTHLHSIGKSVKGRNLW |
| P14384 | HLIDYLVTSDGKDPEITNLINSTRIHIMPSMNPDGFEAVKK |
| P14384 | RENYNQYDLNRNFPDAFEYNNVSRQPETVAVMKWLKTETFV |
| P14384 | YRTNKYGEYYLLLLPGSYIINVTVPGHDPHITKVIIPEKSQ |
| P14384 | VTVPGHDPHITKVIIPEKSQNFSALKKDILLPFQGQLDSIP |
| Q9Y646 | ASCGDVAKAIINLAVYGKAQNRSYERLALLVDTVGPRLSGS |
| Q9Y646 | LQRRASEARGKIVVYNQPYINYSRTVQYRTQGAVEAAKVGA |
| Q9Y646 | WTAEEQGGVGAFQYYQLHKVNISNYSLVMESDAGTFLPTGL |
| Q9Y646 | EEQGGVGAFQYYQLHKVNISNYSLVMESDAGTFLPTGLQFT |
| Q9Y646 | TGSEKARAIMEEVMSLLQPLNITQVLSHGEGTDINFWIQAG |
| P00729 | ENYQLRVNKIKDPKILGIDPNVTQYTGYLDVEDEDKHFFFW |
| P00729 | GPSSIGPDLKPIGNPYSWNSNATVIFLDQPVNVGFSYSGSS |
| P00729 | AGHYIPVFASEILSHKDRNFNLTSVLIGNGLTDPLTQYNYY |
| P00729 | AWTDVLPWKYDEEFASQKVRNWTASITDEVAGEVKSYKHFT |
| O54088 | KITIGVLLIIFLLSIIFTLENVSLAQTSPQISVYKVVGSAD |
| O54088 | GSADLSNPGSAGYWSQIPWTNISLTANIPMAPTSGLTHYLL |
| O54088 | NIPMAPTSGLTHYLLVKAAWNGSWIFILEEWQAPEPAFNAW |
| O54088 | WQAPEPAFNAWSTAVAGIYPNASGPGLFRMIELTPGTTYSL |
| O54088 | GPGLFRMIELTPGTTYSLERNYTNYVSIINGKEETGRIVFN |
| O54088 | NYTNYVSIINGKEETGRIVFNYSGITLPAPNNTEITVMSNG |
| O54088 | GKEETGRIVFNYSGITLPAPNNTEITVMSNGTILLWHSPRP |
| O54088 | FNYSGITLPAPNNTEITVMSNGTILLWHSPRPVEDLLYNDG |
| O54088 | SPRPVEDLLYNDGMFYGYYVNSTWYYPDRAAIMWYLGSGVP |
| O54088 | SLAQSGGSANIWMWVSGATWNNSTYDPAFKSNIWQNESLTG |
| O54088 | LAQSGGSANIWMWVSGATWNNSTYDPAFKSNIWQNESLTGL |
| O54088 | SGATWNNSTYDPAFKSNIWQNESLTGLSYVDSGNHGFAVPL |
| O54088 | LTGLSYVDSGNHGFAVPLYTNNTNMYEVDTAGIWYTPVASE |
| O54088 | NMYEVDTAGIWYTPVASEGLNGSLFFIWTGAKYENGSWVVE |
| O54088 | VASEGLNGSLFFIWTGAKYENGSWVVEFARPLSVPLDYQPF |

| | |
|---|---|
| O54088 | WVVEFARPLSVPLDYQPFMPNITVGKTYYVAFAVWQGRLGE |
| Q9NV96 | YRRNEDKPIAPCGAIANSMFNDTLELFLIGNDSYPIPIALK |
| Q9NV96 | PCGAIANSMFNDTLELFLIGNDSYPIPIALKKKGIAWWTDK |
| Q9NV96 | RLIERKSDLHPTLPAGRYSLNVTYNYPVHYFDGRKRMILST |
| Q12127 | VDDVITQYTTWCPLTTEAPKNGTSTAAPVTSTEAPKNTTSA |
| Q12127 | EAPKNGTSTAAPVTSTEAPKNTTSAAPTHSVTSYTGAAAKA |
| Q6YHK3 | LLEHCPSQVTVKAELLKTASNLTVSVLEAEGVFEKGSFKTL |
| Q6YHK3 | ADEIYELRVTGRTQDEILFSNSTRLSFETKRISVFIQTDKA |
| Q6YHK3 | PKFEVTLQTPLYCSMNSKHLNGTITAKYTYGKPVKGDVTLT |
| Q6YHK3 | PVKGDVTLTFLPLSFWGKKKNITKTFKINGSANFSFNDEEM |
| Q6YHK3 | KQHDYIIEFFDYTTVLKPSLNFTATVKVTRADGNQLTLEER |
| Q6YHK3 | TEYWSGSNSGNQKMEAVQKINYTVPQSGTFKIEFPILEDSS |
| Q6YHK3 | LKELSYMVVSRGQLVAVGKQNSTMFSLTPENSWTPKACVIV |
| Q6YHK3 | MFMNSFAVFQECGLWVLTDANLTKDYIDGVYDNAEYAERFM |
| Q6YHK3 | IDVQESIHFLESEFSRGISDNYTLALITYALSSVGSPKAKE |
| Q6YHK3 | ETVKKVEYDHGKLNLYLDSVNETQFCVNIPAVRNFKVSNTQ |
| P08571 | VSATTPEPCELDDEDFRCVCNFSEPQPDWSEAFQCVSAVEV |
| P08571 | TMPPLPLEATGLALSSLRLRNVSWATGRSWLAELQQWLKPG |
| P08571 | RATVNPSAPRCMWSSALNSLNLSFAGLEQVPKGLPAKLRVL |
| P08571 | DLSCNRLNRAPQPDELPEVDNLTLDGNPFLVPGTALPHEGS |
| P10810 | VHASPAPPEPCELDEESCSCNFSDPKPDWSSAFNCLGAADV |
| P10810 | TAPPPLLEATGPDLNILNLRNVSWATRDAWLAELQQWLKPG |
| P10810 | LQQWLKPGLKVLSIAQAHSLNFSCEQVRVFPALSTLDLSDN |
| P10810 | SLRDAAGAPSCDWPSQLNSLNLSFTGLKQVPKGLPAKLSVL |
| P10810 | DLSYNRLDRNPSPDELPQVGNLSLKGNPFLDSESHSEKFNS |
| Q13740 | RSSTKKSVQYDDVPEYKDRLNLSENYTLSISNARISDEKRF |
| Q13740 | KKSVQYDDVPEYKDRLNLSENYTLSISNARISDEKRFVCML |
| Q13740 | ETEQLKKLGDCISEDSYPDGNITWYRNGKVLHPLEGAVVII |
| Q13740 | PTEQVTIQVLPPKNAIKEGDNITLKCLGNGNPPPEEFLFYL |
| Q13740 | PGQPEGIRSSNTYTLTDVRRNATGDYKCSLIDKKSMIASTA |
| Q13740 | EVTRQIGDALPVSCTISASRNATVVWMKDNIRLRSSPSFSS |
| Q13740 | VEGFPKPAIQWTITGSGSVINQTEESPYINGRYYSKIIISP |
| Q13740 | QWTITGSGSVINQTEESPYINGRYYSKIIISPEENVTLTCT |
| Q13740 | EESPYINGRYYSKIIISPEENVTLTCTAENQLERTVNSLNV |
| Q13740 | ENVTLTCTAENQLERTVNSLNVSAISIPEHDEADEISDENR |
| Q61490 | KKSVQYDDVPEYKDRLSLSENYTLSIANAKISDEKRFVCML |
| Q61490 | ETDQLKKLGDCISRDSYPDGNITWYRNGKVLQPVEGEVAIL |
| Q61490 | PTEQVTIQVLPPKNAIKEGDNITLQCLGNGNPPPEEFMFYL |
| Q61490 | PGQPEGIRSSNTYTLTDVRRNATGDYKCSLIDKRNMAASTT |
| Q61490 | EVTKQIGDTLPVSCTISASRNATVVWMKDNIRLRSSPSFSS |
| Q61490 | VEGFPKPAIHWTITGSGSVINQTEESPYINGRYYSKIIISP |

| | |
|---|---|
| Q61490 | HWTITGSGSVINQTEESPYINGRYYSKIIISPEENVTLTCT |
| Q61490 | EESPYINGRYYSKIIISPEENVTLTCTAENQLERTVNSLNV |
| Q61490 | SPEENVTLTCTAENQLERTVNSLNVSAISIPEHDEADDISD |
| Q61490 | ENVTLTCTAENQLERTVNSLNVSAISIPEHDEADDISDENR |
| Q61490 | LNVSAISIPEHDEADDISDENREKVNDQAKLIVGIVVGLLL |
| Q62192 | FLASCRATTSSDQKCIEKEVNKTYNCENLGLNEIPGTLPNS |
| Q62192 | VNKTYNCENLGLNEIPGTLPNSTECLEFSFNVLPTIQNTTF |
| Q62192 | TLPNSTECLEFSFNVLPTIQNTTFSRLINLTFLDLTRCQIY |
| Q62192 | LEFSFNVLPTIQNTTFSRLINLTFLDLTRCQIYWIHEDTFQ |
| Q62192 | QNNAIHYLSKEDMSSLQQATNLSLNLNGNDIAGIELGAFDS |
| Q62192 | FQSLNFGGTQNLLVIFKGLKNSTIQSLWLGTFEDMDDEDIS |
| Q62192 | FEGLCEMSVESINLQKHYFFNISSNTFHCFSGLQELDLTAT |
| Q62192 | ELDLSHDDIETSDCCNLQLRNLSHLQSLNLSYNEPLSLKTE |
| Q62192 | IETSDCCNLQLRNLSHLQSLNLSYNEPLSLKTEAFKECPQL |
| Q62192 | RLKVKDAQSPFQNLHLLKVLNLSHSLLDISSEQLFDGLPAL |
| Q99467 | FSAGCKVITSWDQMCIEKEANKTYNCENLGLSEIPDTLPNT |
| Q99467 | ANKTYNCENLGLSEIPDTLPNTTEFLEFSFNFLPTIHNRTF |
| Q99467 | TLPNTTEFLEFSFNFLPTIHNRTFSRLMNLTFLDLTRCQIN |
| Q99467 | LEFSFNFLPTIHNRTFSRLMNLTFLDLTRCQINWIHEDTFQ |
| Q99467 | QNNAIHYISREDMRSLEQAINLSLNFNGNNVKGIELGAFDS |
| Q99467 | IELGAFDSTIFQSLNFGGTPNLSVIFNGLQNSTTQSLWLGT |
| Q99467 | FQSLNFGGTPNLSVIFNGLQNSTTQSLWLGTFEDIDDEDIS |
| Q99467 | TLDLSHNDIEASDCCSLQLKNLSHLQTLNLSHNEPLGLQSQ |
| Q99467 | IEASDCCSLQLKNLSHLQTLNLSHNEPLGLQSQAFKECPQL |
| Q99467 | RLHINAPQSPFQNLHFLQVLNLTYCFLDTSNQHLLAGLPVL |
| Q99467 | NSINIISPRLLPILSQQSTINLSHNPLDCTCSNIHFLTWYK |
| P25918 | LPCLPDSSPVSSEKLAWYRGNQSTPFLELSPGSPGLGLHVG |
| P25918 | PGSPGLGLHVGSLGILLVIVNVSDHMGGFYLCQKRPPFKDI |
| P25918 | DIWQPAWTVNVEDSGEMFRWNASDVRDLDCDLRNRSSGSHR |
| P25918 | SGEMFRWNASDVRDLDCDLRNRSSGSHRSTSGSQLYVWAKD |
| P25918 | KDHPKVWGTKPVCAPRGSSLNQSLINQDLTVAPGSTLWLSC |
| P25918 | PPVPVAKGSISWTHVHPRRPNVSLLSLSLGGEHPVREMWVW |
| P25918 | LLLLPQATALDEGTYYCLRGNLTIERHVKVIARSAVWLWLL |
| P06126 | NADGLKEPLSFHVTWIASFYNHSWKQNLVSGWLSDLQTHTW |
| P06126 | WKQNLVSGWLSDLQTHTWDSNSSTIVFLCPWSRGNFSNEEW |
| P06126 | THTWDSNSSTIVFLCPWSRGNFSNEEWKELETLFRIRTIRS |
| P06126 | KVSGSFLQLAYQGSDFVSFQNNSWLPYPVAGNMAKHFCKVL |
| P29016 | SEHAFQGPTSFHVIQTSSFTNSTWAQTQGSGWLDDLQIHGW |
| P29016 | IHGWDSDSGTAIFLKPWSKGNFSDKEVAELEEIFRVYIFGF |
| P29016 | GAIVSFLRGALGGLDFLSVKNASCVPSPEGGSRAQKFCALI |
| P29016 | WMRGEQEQQGTQLGDILPNANWTWYLRATLDVADGEAAGLS |

| | |
|---|---|
| P29017 | NADASQEHVSFHVIQIFSFVNQSWARGQGSGWLDELQTHGW |
| P29017 | LDELQTHGWDSESGTIIFLHNWSKGNFSNEELSDLELLFRF |
| P29017 | THGWDSESGTIIFLHNWSKGNFSNEELSDLELLFRFYLFGL |
| P29017 | WDSESGTIIFLHNWSKGNFSNEELSDLELLFRFYLFGLTRE |
| P29017 | KSPEGFFQVAFNGLDLLSFQNTTWVPSPGCGSLAQSVCHLL |
| P11609 | PWLLLWAFLQVWGQSEAQQKNYTFRCLQMSSFANRSWSRTD |
| P11609 | QSEAQQKNYTFRCLQMSSFANRSWSRTDSVVWLGDLQTHRW |
| P11609 | SWSRTDSVVWLGDLQTHRWSNDSATISFTKPWSQGKLSNQQ |
| P11609 | PKEDYPIEIQLSAGCEMYPGNASESFLHVAFQGKYVVRFWG |
| P11609 | LPIKVLNADQGTSATVQMLLNDTCPLFVRGLLEAGKSDLEK |
| P15813 | SAEVPQRLFPLRCLQISSFANSSWTRTDGLAWLGELQTHSW |
| P15813 | SWTRTDGLAWLGELQTHSWSNDSDTVRSLKPWSQGTFSDQQ |
| P15813 | LRLSYPLELQVSAGCEVHPGNASNNFFHVAFQGKDILSFQG |
| P15813 | LAIQVLNQDKWTRETVQWLLNGTCPQFVSGLLESGKSELKK |
| Q9BZW8 | IAWKKLLPSQNGFHHILKWENGSLPSNTSNDRFSFIVKNLS |
| Q9BZW8 | LPSQNGFHHILKWENGSLPSNTSNDRFSFIVKNLSLLIKAA |
| Q9BZW8 | WENGSLPSNTSNDRFSFIVKNLSLLIKAAQQQDSGLYCLEV |
| Q9BZW8 | KILDRGRCQVALSCLVSRDGNVSYAWYRGSKLIQTAGNLTY |
| Q9BZW8 | RDGNVSYAWYRGSKLIQTAGNLTYLDEEVDINGTHTYTCNV |
| Q9BZW8 | GSKLIQTAGNLTYLDEEVDINGTHTYTCNVSNPVSWESHTL |
| Q9BZW8 | GNLTYLDEEVDINGTHTYTCNVSNPVSWESHTLNLTQDCQN |
| Q9BZW8 | GTHTYTCNVSNPVSWESHTLNLTQDCQNAHQEFRFWPFLVI |
| P24807 | ARLGLGLLLLALLLPTQIYCNQTSVAPFPGNQNISASPNPS |
| P24807 | LLPTQIYCNQTSVAPFPGNQNISASPNPSNATTRGGGSSLQ |
| P24807 | QTSVAPFPGNQNISASPNPSNATTRGGGSSLQSTAGLLALS |
| Q8VE98 | DQGSAYSNRTALFPDLLVQGNASLRLQRVRVTDEGSYTCFV |
| Q8VE98 | QGYPEAEVFWKDGQGVPLTGNVTTSQMANERGLFDVHSVLR |
| Q8VE98 | FWKDGQGVPLTGNVTTSQMANERGLFDVHSVLRVVLGANGT |
| Q8VE98 | MANERGLFDVHSVLRVVLGANGTYSCLVRNPVLQQDAHGSV |
| Q5ZPR3 | DQGSAYANRTALFPDLLAQGNASLRLQRVRVADEGSFTCFV |
| Q5ZPR3 | QGYPEAEVFWQDGQGVPLTGNVTTSQMANEQGLFDVHSILR |
| Q5ZPR3 | MANEQGLFDVHSILRVVLGANGTYSCLVRNPVLQQDAHSSV |
| Q5ZPR3 | DQGSAYANRTALFPDLLAQGNASLRLQRVRVADEGSFTCFV |
| Q5ZPR3 | RGYPEAEVFWQDGQGVPLTGNVTTSQMANEQGLFDVHSVLR |
| Q5ZPR3 | MANEQGLFDVHSVLRVVLGANGTYSCLVRNPVLQQDAHGSV |
| P26842 | HHTRPHCESCRHCNSGLLVRNCTITANAECACRNGWQCRDK |
| P10747 | TGNKILVKQSPMLVAYDNAVNLSCKYSYNLFSREFRASLHK |
| P10747 | FRASLHKGLDSAVEVCVVYGNYSQQLQVYSKTGFNCDGKLG |
| P10747 | YSQQLQVYSKTGFNCDGKLGNESVTFYLQNLYVNQTDIYFC |
| P10747 | NCDGKLGNESVTFYLQNLYVNQTDIYFCKIEVMYPPPYLDN |
| P10747 | IYFCKIEVMYPPPYLDNEKSNGTIIHVKGKHLCPSPLFPGP |

| | |
|---|---|
| P06729 | AQFRKEKETFKEKDTYKLFKNGTLKIKHLKTDDQDIYKVSI |
| P06729 | IFDLKIQERVSKPKISWTCINTTLTCEVMNGTDPELNLYQD |
| P06729 | VSKPKISWTCINTTLTCEVMNGTDPELNLYQDGKHLKLSQR |
| P08921 | ILANGDLKIKNLTRDDSGTYNVTVYSTNGTRILDKALDLRI |
| P08921 | KIKNLTRDDSGTYNVTVYSTNGTRILDKALDLRILEMVSKP |
| P08921 | ALDLRILEMVSKPMIYWECSNATLTCEVLEGTDVELKLYQG |
| P26201 | TDVYRQFWIFDVQNPDEVTVNSSKIKVKQRGPYTYRVRYLA |
| P26201 | KIKVKQRGPYTYRVRYLAKENITQDPETHTVSFLQPNGAIF |
| P26201 | TFMQGILNSFIKKSKSSMFQNRTLKELLWGYTDPFLNLVPY |
| P26201 | PFLNLVPYPITTTIGVFYPYNNTADGIYKVFNGKDDISKVA |
| P26201 | FNGKDDISKVAIIDTYKGRKNLSYWSSYCDLINGTDAASFP |
| P26201 | IDTYKGRKNLSYWSSYCDLINGTDAASFPPFVEKTRVLQFF |
| P26201 | ASPFQNPDNHCFCTEKIISKNCTLYGVLDIGKCKEGKPVYI |
| P26201 | KKIEALKNLKHNYIVPILWLNETGTIGDEKAEMFRNQVTGK |
| Q64314 | NLTSATTETSTQGISPSVPTNESVEENITSSIPGSTSHYLI |
| Q64314 | TETSTQGISPSVPTNESVEENITSSIPGSTSHYLIYQDSSK |
| Q64314 | HYLIYQDSSKTTPAISETMVNFTVTSGIPSGSGTPHTFSQP |
| Q64314 | TTSDSISTSEMTWKSSLPSINVSDYSPNNSSFEMTSPTEPY |
| Q64314 | TSEMTWKSSLPSINVSDYSPNNSSFEMTSPTEPYAYTSSSA |
| Q64314 | SEMTWKSSLPSINVSDYSPNNSSFEMTSPTEPYAYTSSSAP |
| Q64314 | CSLLLAQSEVRPECLLMVLANSTELPSKLQLMEKHQSDLRK |
| P16671 | TEVYRQFWIFDVQNPQEVMMNSSNIQVKQRGPYTYRVRFLA |
| P16671 | NIQVKQRGPYTYRVRFLAKENVTQDAEDNTVSFLQPNGAIF |
| P16671 | FLQPNGAIFEPSLSVGTEADNFTVLNLAVAAASHIYQNQFV |
| P16671 | AAASHIYQNQFVQMILNSLINKSKSSMFQVRTLRELLWGYR |
| P16671 | PFLSLVPYPVTTTVGLFYPYNNTADGVYKVFNGKDNISKVA |
| P16671 | LFYPYNNTADGVYKVFNGKDNISKVAIIDTYKGKRNLSYWE |
| P16671 | FNGKDNISKVAIIDTYKGKRNLSYWESHCDMINGTDAASFP |
| P16671 | IDTYKGKRNLSYWESHCDMINGTDAASFPPFVEKSQVLQFF |
| P16671 | ASPVENPDNYCFCTEKIISKNCTSYGVLDISKCKEGRPVYI |
| P16671 | EKIQVLKNLKRNYIVPILWLNETGTIGDEKANMFRSQVTGK |
| P28907 | VDCQSVWDAFKGAFISKHPCNITEEDYQPLMKLGTQTVPCN |
| P28907 | TLEDTLLGYLADDLTWCGEFNTSKINYQSCPDWRKDCSNNP |
| P28907 | WKTVSRRFAEAACDVVHVMLNGSRSKIFDKNSTFGSVEVHN |
| P28907 | AACDVVHVMLNGSRSKIFDKNSTFGSVEVHNLQPEKVQTLE |
| P29965 | QQSIHLGGVFELQPGASVFVNVTDPSQVSHGTGFTSFGLLK |
| P16070 | WWHAAWGLCLVPLSLAQIDLNITCRFAGVFHVEKNGRYSIS |
| P16070 | EKNGRYSISRTEAADLCKAFNSTLPTMAQMEKALSIGFETC |
| P16070 | GFIEGHVVIPRIHPNSICAANNTGVYILTSNTSQYDTYCFN |
| P16070 | RIHPNSICAANNTGVYILTSNTSQYDTYCFNASAPPEEDCT |
| P16070 | NNTGVYILTSNTSQYDTYCFNASAPPEEDCTSVTDLPNAFD |

| | |
|---|---|
| P16070 | SHSNPEVLLQTTTRMTDVDRNGTTAYEGNWNPEAHPPLIHH |
| P16070 | TTSTLTSSNRNDVTGGRRDPNHSEGSTTLLEGYTSHYPHTK |
| P16070 | AKTGSFGVTAVTVGDSNSNVNRSLSGDQDTFHPSGGSHTTH |
| P16070 | HTTHGSESDGHSHGSQEGGANTTSGPIRTPQIPEWLIILAS |
| Q08722 | PLVAALLLGSACCGSAQLLFNKTKSVEFTFCNDTVVIPCFV |
| Q08722 | CCGSAQLLFNKTKSVEFTFCNDTVVIPCFVTNMEAQNTTEV |
| Q08722 | FTFCNDTVVIPCFVTNMEAQNTTEVYVKWKFKGRDIYTFDG |
| Q08722 | EVYVKWKFKGRDIYTFDGALNKSTVPTDFSSAKIEVSQLLK |
| Q08722 | LLKGDASLKMDKSDAVSHTGNYTCEVTELTREGETIIELKY |
| Q08722 | ITVIVIVGAILFVPGEYSLKNATGLGLIVTSTGILILLHYY |
| P09326 | SLLVTSIQGHLVHMTVVSGSNVTLNISESLPENYKQLTWFY |
| P09326 | TSIQGHLVHMTVVSGSNVTLNISESLPENYKQLTWFYTFDQ |
| P09326 | RVRLDPQSGALYISKVQKEDNSTYIMRVLKKTGNEQEWKIK |
| P09326 | EDMDDNCYLKLSCVIPGESVNYTWYGDKRPFPKELQNSVLE |
| P09326 | GESVNYTWYGDKRPFPKELQNSVLETTLMPHNYSRCYTCQV |
| P09326 | KRPFPKELQNSVLETTLMPHNYSRCYTCQVSNSVSSKNGTV |
| P10252 | SLVTGFQDQSVPNVNAITGSNVTLTILKHPLASYQRLTWLH |
| P10252 | ESVFKDRVDLDKTNGALRIYNVSKEDRGDYYMRMLHETEDQ |
| P10252 | ITMEVYDLVSKPAIKIEKTKNLTDSCHLRLSCKVEDQGVDY |
| P10252 | SGPFPQRNPGYVLEITITPHNKSTFYTCQVSNPVSSENDTL |
| P10252 | TPHNKSTFYTCQVSNPVSSENDTLYFIPPCTLARSSGVHWI |
| P01730 | KKLPLHLTLPQALPQYAGSGNLTLALEAKTGKLHQEVNLVV |
| P01730 | TGKLHQEVNLVVMRATQLQKNLTCEVWGPTSPKLMLSLKLE |
| P05540 | VKDSKAFSTHSLRIQDSGIWNCTVTLNQKKHSFDMKLSVLG |
| P05540 | ETLPLTLQIPQVSLQFAGSGNLTLTLDRGILYQEVNLVVMK |
| P05540 | LSEGEEVKMDSKIQVLSKGLNQTMFLAVVLGSAFSFLVFTG |
| P31358 | LLLTISLLVMVQIQTGLSGQNDTSQTSSPSASSNISGGIFL |
| P31358 | QTGLSGQNDTSQTSSPSASSNISGGIFLFFVANAIIHLFCF |
| P13987 | GHSLQCYNCPNPTADCKTAVNCSSDFDACLITKAGLQVYNK |
| P13987 | SDFDACLITKAGLQVYNKCWKFEHCNFNDVTTRLRENELTY |
| P13987 | DACLITKAGLQVYNKCWKFEHCNFNDVTTRLRENELTYYCC |
| P41731 | VEVAVAIAGYVFRDQVKSEFNKSFQQQMQNYLKDNKTATIL |
| P41731 | QVKSEFNKSFQQQMQNYLKDNKTATILDKLQKENNCCGASN |
| P41731 | NKTATILDKLQKENNCCGASNYTDWENIPGMAKDRVPDSCC |
| P41731 | TDWENIPGMAKDRVPDSCCINITVGCGNDFKESTIHTQGCV |
| P34810 | GTTSHGPTTATHNPTTTSHGNVTVHPTSNSTATSQGPSTAT |
| P34810 | TATHNPTTTSHGNVTVHPTSNSTATSQGPSTATHSPATTSH |
| P34810 | TATSQGPSTATHSPATTSHGNATVHPTSNSTATSPGFTSSA |
| P34810 | TATHSPATTSHGNATVHPTSNSTATSPGFTSSAHPEPPPPS |
| P34810 | PPSPSPSPTSKETIGDYTWTNGSQPCVHLQAQIQIRVMYTT |
| P34810 | RVMYTTQGGGEAWGISVLNPNKTKVQGSCEGAHPHLLLSFP |

| | |
|---|---|
| P34810 | GFMQDLQQKVVYLSYMAVEYNVSFPHAAQWTFSAQNASLRD |
| P34810 | MAVEYNVSFPHAAQWTFSAQNASLRDLQAPLGQSFSCSNSS |
| P34810 | AQNASLRDLQAPLGQSFSCSNSSIILSPAVHLDLLSLRLQA |
| P08962 | KVMSEFNNNFRQQMENYPKNNHTASILDRMQADFKCCGAAN |
| P08962 | NHTASILDRMQADFKCCGAANYTDWEKIPSMSKNRVPDSCC |
| P08962 | TDWEKIPSMSKNRVPDSCCINVTVGCGINFNEKAIHKEGCV |
| P30203 | TGLLTAALSGHPSPAPPDQLNTSSAESELWEPGERLPVRLT |
| P30203 | TSSAESELWEPGERLPVRLTNGSSSCSGTVEVRLEASWEPA |
| P30203 | EAASQLAPPTPELPPPPAAGNTSVAANATLAGAPALLCSGA |
| P30203 | APPTPELPPPPAAGNTSVAANATLAGAPALLCSGAEWRLCE |
| P30203 | QALPGLHFTPGRGPIHRDQVNCSGAEAYLWDCPGLPGQHYC |
| P30203 | PHSLSGRMYYSCNGEELTLSNCSWRFNNSNLCSQSLAARVL |
| P30203 | RMYYSCNGEELTLSNCSWRFNNSNLCSQSLAARVLCSASRS |
| P30203 | NLCSQSLAARVLCSASRSLHNLSTPEVPASVQTVTIESSVT |
| P09564 | AAQEVQQSPHCTTVPVGASVNITCSTSGGLRGIYLRQLGPQ |
| P09564 | VVPTTDRRFRGRIDFSGSQDNLTITMHRLQLSDTGTYTCQA |
| Q00609 | HDKVVLSVIAGKLKVWPEYKNRTLYDNTTYSLIILGLVLSD |
| Q00609 | SVIAGKLKVWPEYKNRTLYDNTTYSLIILGLVLSDRGTYSC |
| Q00609 | YEVKHLALVKLSIKADFSTPNITESGNPSADTKRITCFASG |
| Q00609 | ALVKLSIKADFSTPNITESGNPSADTKRITCFASGGFPKPR |
| Q00609 | GGFPKPRFSWLENGRELPGINTTISQDPESELYTISSQLDF |
| Q00609 | TTISQDPESELYTISSQLDFNTTRNHTIKCLIKYGDAHVSE |
| Q00609 | QDPESELYTISSQLDFNTTRNHTIKCLIKYGDAHVSEDFTW |
| P33681 | SGVIHVTKEVKEVATLSCGHNVSVEELAQTRIYWQKEKKMV |
| P33681 | EKKMVLTMMSGDMNIWPEYKNRTIFDITNNLSIVILALRPS |
| P33681 | SGDMNIWPEYKNRTIFDITNNLSIVILALRPSDEGTYECVV |
| P33681 | GGFPEPHLSWLENGEELNAINTTVSQDPETELYAVSSKLDF |
| P33681 | TTVSQDPETELYAVSSKLDFNMTTNHSFMCLIKYGHLRVNQ |
| P33681 | QDPETELYAVSSKLDFNMTTNHSFMCLIKYGHLRVNQTFNW |
| P33681 | FNMTTNHSFMCLIKYGHLRVNQTFNWNTTKQEHFPDNLLPS |
| P33681 | HSFMCLIKYGHLRVNQTFNWNTTKQEHFPDNLLPSWAITLI |
| P27701 | NMGKLKQEMGGIVTELIRDYNSSREDSLQDAWDYVQAQVKC |
| P27701 | QDAWDYVQAQVKCCGWVSFYNWTDNAELMNRPEVTYPCSCE |
| P27701 | VKGEEDNSLSVRKGFCEAPGNRTQSGNHPEDWPVYQEGCME |
| Q01151 | RMETPQEDHLRGQHYHQKGQNGSFDAPNERPYSLKIRNTTS |
| Q01151 | DHLRGQHYHQKGQNGSFDAPNERPYSLKIRNTTSCNSGTYR |
| Q01151 | KGQNGSFDAPNERPYSLKIRNTTSCNSGTYRCTLQDPDGQR |
| Q01151 | TTSCNSGTYRCTLQDPDGQRNLSGKVILRVTGCPAQRKEET |
| P40237 | YFNADKLKKEMGNTVMDIIRNYTANATSSREEAWDYVQAQV |
| P40237 | DKLKKEMGNTVMDIIRNYTANATSSREEAWDYVQAQVKCCG |
| P40237 | EEAWDYVQAQVKCCGWVSHYNWTENEELMGFTKTTYPCSCE |

| | |
|---|---|
| P40237 | DYVQAQVKCCGWVSHYNWTENEELMGFTKTTYPCSCEKIKE |
| P40237 | EKIKEEDNQLIVKKGFCEADNSTVSENNPEDWPVNTEGCME |
| P07725 | VKLTCEVLRDTSQGCSWLFRNSSSELLQPTFIIYVSSSRSK |
| P01731 | VDLVCEVLGSVSQGCSWLFQNSSSKLPQPTFVVYMASSHNK |
| P01731 | PTFVVYMASSHNKITWDEKLNSSKLFSAMRDTNNKYVLTLN |
| P01731 | SVISNSVMYFSSVVPVLQKVNSTTTKPVLRTPSPVHPTGTS |
| P48960 | LTLPGAETQDSRGCARWCPQNSSCVNATACRCNPGFSSFSE |
| P48960 | AETQDSRGCARWCPQNSSCVNATACRCNPGFSSFSEIITTP |
| P48960 | SYDCVCSPGYEPVSGAKTFKNESENTCQDVDECQQNPRLCK |
| P48960 | SYQCRCRPGWQPIPGSPNGPNNTVCEDVDECSSGQHQCDSS |
| P48960 | TYISPSNTELTLMIQERGDKNVTMGQSSARMKLNWAVAAGA |
| P48960 | AVAAGAEDPGPAVAGILSIQNMTTLLANASLNLHSKKQAEL |
| P48960 | DPGPAVAGILSIQNMTTLLANASLNLHSKKQAELEEIYESS |
| P48960 | SIRGVQLRRLSAVNSIFLSHNNTKELNSPILFAFSHLESSD |
| P48960 | SDSDRGGHWATEGCQVLGSKNGSTTCQCSHLSSFAILMAHY |
| Q9Z0M6 | QKAESKNCAKWCPINSKCVSNRSCVCKPGFSSEKELITNPA |
| Q9Z0M6 | SYTCVCNLGYKLLSGAESFVNESENTCQASVNTGTTPVPSR |
| Q9Z0M6 | PVSTVCEDVDECSSGQHQCHNSTVCKNTVGSYKCHCRPGWK |
| Q9Z0M6 | LLRFSVEVQNLLRDFNPATVNYTIQKLIEAVDKLLEDPMET |
| Q9Z0M6 | HGQTWMELDWAVTAGAKISENGSSVAGILSSPNMEKLLGNT |
| Q9Z0M6 | TAGAKISENGSSVAGILSSPNMEKLLGNTPLNLEQRRASLE |
| Q9Z0M6 | LLSNINSVFLTNTNTEKLASNVTFKFDFTSVESIEPRHELI |
| Q9Z0M6 | CAFWKAHNGNGYWDTDGCSMNGTGFCHCNHLTSFAILMAQY |
| Q9HBB8 | MAQAQYCSVNKDIFEVEENTNVTEPLVDIHVPEGQEVTLGA |
| Q9HBB8 | TLGALSTPFAFRIQGNQLFLNVTPDYEEKSLLEAQLLCQSG |
| Q9HBB8 | NAPEFPFKTKEIRVEEDTKVNSTVIPETQLQAEDRDKDDIL |
| Q9HBB8 | VSVNRPALRLDRPLDFYERPNMTFWLLVRDTPGENVEPSHT |
| Q9HBB8 | EDGDRGINQPIIYSIFRGNVNGTFIIHPDSGNLTVARSVPS |
| Q9HBB8 | IYSIFRGNVNGTFIIHPDSGNLTVARSVPSPMTFLLLVKGQ |
| Q9HBB8 | IQAQDPEFSDLNSAITYRITNHSHFRMEGEVVLTTTTLAQA |
| Q9HBB8 | SGTTLRPPTSSTPGGPPGAENSTSHQPATPGGDTAQTPKPG |
| P13688 | IGTQQATPGPANSGRETIYPNASLLIQNVTQNDTGFYTLQV |
| P13688 | PGPANSGRETIYPNASLLIQNVTQNDTGFYTLQVIKSDLVN |
| P13688 | NSGRETIYPNASLLIQNVTQNDTGFYTLQVIKSDLVNEEAT |
| P13688 | EEATGQFHVYPELPKPSISSNNSNPVEDKDAVAFTCEPETQ |
| P13688 | AVAFTCEPETQDTTYLWWINNQSLPVSPRLQLSNGNRTLTL |
| P13688 | LWWINNQSLPVSPRLQLSNGNRTLTLLSVTRNDTGPYECEI |
| P13688 | SPRLQLSNGNRTLTLLSVTRNDTGPYECEIQNPVSANRSDP |
| P13688 | SVTRNDTGPYECEIQNPVSANRSDPVTLNVTYGPDTPTISP |
| P13688 | PYECEIQNPVSANRSDPVTLNVTYGPDTPTISPSDTYYRPG |
| P13688 | TYGPDTPTISPSDTYYRPGANLSLSCYAASNPPAQYSWLIN |

| | |
|---|---|
| P13688 | NLSLSCYAASNPPAQYSWLINGTFQQSTQELFIPNITVNNS |
| P13688 | QYSWLINGTFQQSTQELFIPNITVNNSGSYTCHANNSVTGC |
| P13688 | LINGTFQQSTQELFIPNITVNNSGSYTCHANNSVTGCNRTT |
| P13688 | QELFIPNITVNNSGSYTCHANNSVTGCNRTTVKTIIVTELS |
| P13688 | ITVNNSGSYTCHANNSVTGCNRTTVKTIIVTELSPVVAKPQ |
| P13688 | VAKPQIKASKTTVTGDKDSVNLTCSTNDTGISIRWFFKNQS |
| P13688 | KASKTTVTGDKDSVNLTCSTNDTGISIRWFFKNQSLPSSER |
| P13688 | SVNLTCSTNDTGISIRWFFKNQSLPSSERMKLSQGNTTLSI |
| P13688 | RWFFKNQSLPSSERMKLSQGNTTLSINPVKREDAGTYWCEV |
| P13688 | PVKREDAGTYWCEVFNPISKNQSDPIMLNVNYNALPQENGL |
| P16573 | WYKVTTTGLNSEIARYIRSSNTSQTEPAYSGRVTIYSNGSL |
| P16573 | RSSNTSQTEPAYSGRVTIYSNGSLFFQNVNKTDEGPYTLSV |
| P16573 | PAYSGRVTIYSNGSLFFQNVNKTDEGPYTLSVIDKQFNPIQ |
| P16573 | QFNPIQTSVQFRVYPALQKPNVTGNNSNPMEGEPFVSLMCE |
| P16573 | IQTSVQFRVYPALQKPNVTGNNSNPMEGEPFVSLMCEPYTN |
| P16573 | NNSNPMEGEPFVSLMCEPYTNNTSYLWSRNGESLSEGDRVT |
| P16573 | NSNPMEGEPFVSLMCEPYTNNTSYLWSRNGESLSEGDRVTF |
| P16573 | LWSRNGESLSEGDRVTFSEGNRTLTLLNVRRTDKGYYECEA |
| P16573 | NVRRTDKGYYECEARNPATFNRSDPFNLDVIYGPDAPVISP |
| P16573 | GPDAPVISPPDIYLHQGSNLNLSCHADSNPPAQYFWLINEK |
| P16573 | QYFWLINEKLQTSSQELFISNITTNNSGTYACFVNNTVTGL |
| P16573 | LINEKLQTSSQELFISNITTNNSGTYACFVNNTVTGLSRTT |
| P16573 | QELFISNITTNNSGTYACFVNNTVTGLSRTTVKNITVFEPV |
| P16573 | GTYACFVNNTVTGLSRTTVKNITVFEPVTQPSIQITNTTVK |
| P16573 | TTVKNITVFEPVTQPSIQITNTTVKELGSVTLTCFSKDTGV |
| P16573 | RWLFNSQSLQLTDRMTLSQDNSTLRIDPIKREDAGDYQCEI |
| Q925P2 | WHKGSTTSTNAEIVRFVTGTNKTIKGPVHSGRETLYSNGSL |
| Q925P2 | TGTNKTIKGPVHSGRETLYSNGSLLIQRVTMKDTGVYTIEM |
| Q925P2 | NYRRRVLTGQFHVHTLLLKSNITSNNSNPVEGDDSVSLTCD |
| Q925P2 | RVLTGQFHVHTLLLKSNITSNNSNPVEGDDSVSLTCDSYTD |
| Q925P2 | NPVEGDDSVSLTCDSYTDPDNITYLWSRNGESLSEGDRLKL |
| Q925P2 | LWSRNGESLSEGDRLKLSEGNRTLTLLNVTRNDTGPYVCET |
| Q925P2 | SLSEGDRLKLSEGNRTLTLLNVTRNDTGPYVCETRNPVSVN |
| Q925P2 | GDRLKLSEGNRTLTLLNVTRNDTGPYVCETRNPVSVNRSDP |
| Q925P2 | NVTRNDTGPYVCETRNPVSVNRSDPFSLNIIYGPDTPIISP |
| Q925P2 | GPDTPIISPSDIYLHPGSNLNLSCHAASNPPAQYFWLINEK |
| Q925P2 | QYFWLINEKPHASSQELFIPNITTNNSGTYTCFVNNSVTGL |
| Q925P2 | LINEKPHASSQELFIPNITTNNSGTYTCFVNNSVTGLSRTT |
| Q925P2 | QELFIPNITTNNSGTYTCFVNNSVTGLSRTTVKNITVLEPV |
| Q925P2 | GTYTCFVNNSVTGLSRTTVKNITVLEPVTQPSLQVTNTTVK |
| Q925P2 | TTVKNITVLEPVTQPSLQVTNTTVKELDSVTLTCLSKDRQA |

| | |
|---|---|
| Q925P2 | SVTLTCLSKDRQAHIHWIFNNDTLLITEKMTTSQAGLILKI |
| P31809 | VLLLVHNLPLALGAFAWYKGNTTAIDKEIARFVPNSNMNFT |
| P31809 | KGNTTAIDKEIARFVPNSNMNFTGQAYSGREIIYSNGSLLF |
| P31809 | PNSNMNFTGQAYSGREIIYSNGSLLFQMITMKDMGVYTLDM |
| P31809 | NYRRTQATVRFHVHPILLKPNITSNNSNPVEGDDSVSLTCD |
| P31809 | TQATVRFHVHPILLKPNITSNNSNPVEGDDSVSLTCDSYTD |
| P31809 | LWSRNGESLSEGDRLKLSEGNRTLTLLNVTRNDTGPYVCET |
| P31809 | SLSEGDRLKLSEGNRTLTLLNVTRNDTGPYVCETRNPVSVN |
| P31809 | GDRLKLSEGNRTLTLLNVTRNDTGPYVCETRNPVSVNRSDP |
| P31809 | NVTRNDTGPYVCETRNPVSVNRSDPFSLNIIYGPDTPIISP |
| P31809 | GPDTPIISPSDIYLHPGSNLNLSCHAASNPPAQYFWLINEK |
| P31809 | QYFWLINEKPHASSQELFIPNITTNNSGTYTCFVNNSVTGL |
| P31809 | LINEKPHASSQELFIPNITTNNSGTYTCFVNNSVTGLSRTT |
| P31809 | QELFIPNITTNNSGTYTCFVNNSVTGLSRTTVKNITVLEPV |
| P31809 | GTYTCFVNNSVTGLSRTTVKNITVLEPVTQPFLQVTNTTVK |
| P31809 | TTVKNITVLEPVTQPFLQVTNTTVKELDSVTLTCLSNDIGA |
| P31809 | IQWLFNSQSLQLTERMTLSQNNSILRIDPIKREDAGEYQCE |
| P31809 | QWLFNSQSLQLTERMTLSQNNSILRIDPIKREDAGEYQCEI |
| P06731 | IGTQQATPGPAYSGREIIYPNASLLIQNIIQNDTGFYTLHV |
| P06731 | YSGREIIYPNASLLIQNIIQNDTGFYTLHVIKSDLVNEEAT |
| P06731 | EEATGQFRVYPELPKPSISSNNSKPVEDKDAVAFTCEPETQ |
| P06731 | AVAFTCEPETQDATYLWWVNNQSLPVSPRLQLSNGNRTLTL |
| P06731 | LWWVNNQSLPVSPRLQLSNGNRTLTLFNVTRNDTASYKCET |
| P06731 | SLPVSPRLQLSNGNRTLTLFNVTRNDTASYKCETQNPVSAR |
| P06731 | SPRLQLSNGNRTLTLFNVTRNDTASYKCETQNPVSARRSDS |
| P06731 | SDSVILNVLYGPDAPTISPLNTSYRSGENLNLSCHAASNPP |
| P06731 | GPDAPTISPLNTSYRSGENLNLSCHAASNPPAQYSWFVNGT |
| P06731 | NLNLSCHAASNPPAQYSWFVNGTFQQSTQELFIPNITVNNS |
| P06731 | QYSWFVNGTFQQSTQELFIPNITVNNSGSYTCQAHNSDTGL |
| P06731 | FVNGTFQQSTQELFIPNITVNNSGSYTCQAHNSDTGLNRTT |
| P06731 | ITVNNSGSYTCQAHNSDTGLNRTTVTTITVYAEPPKPFITS |
| P06731 | RTTVTTITVYAEPPKPFITSNNSNPVEDEDAVALTCEPEIQ |
| P06731 | NSNPVEDEDAVALTCEPEIQNTTYLWWVNNQSLPVSPRLQL |
| P06731 | AVALTCEPEIQNTTYLWWVNNQSLPVSPRLQLSNDNRTLTL |
| P06731 | LWWVNNQSLPVSPRLQLSNDNRTLTLLSVTRNDVGPYECGI |
| P06731 | LYGPDDPTISPSYTYYRPGVNLSLSCHAASNPPAQYSWLID |
| P06731 | QYSWLIDGNIQQHTQELFISNITEKNSGLYTCQANNSASGH |
| P06731 | QELFISNITEKNSGLYTCQANNSASGHSRTTVKTITVSAEL |
| P06731 | RTTVKTITVSAELPKPSISSNNSKPVEDKDAVAFTCEPEAQ |
| P06731 | NSKPVEDKDAVAFTCEPEAQNTTYLWWVNGQSLPVSPRLQL |
| P06731 | LWWVNGQSLPVSPRLQLSNGNRTLTLFNVTRNDARAYVCGI |

| | |
|---|---|
| P06731 | SLPVSPRLQLSNGNRTLTLFNVTRNDARAYVCGIQNSVSAN |
| P06731 | NVTRNDARAYVCGIQNSVSANRSDPVTLDVLYGPDTPIISP |
| P06731 | GPDTPIISPPDSSYLSGANLNLSCHSASNPSPQYSWRINGI |
| P06731 | NGIPQQHTQVLFIAKITPNNNGTYACFVSNLATGRNNSIVK |
| P06731 | ITPNNNGTYACFVSNLATGRNNSIVKSITVSASGTSPGLSA |
| P40199 | IGTQQATPGPAYSGRETIYPNASLLIQNVTQNDTGFYTLQV |
| P40199 | PGPAYSGRETIYPNASLLIQNVTQNDTGFYTLQVIKSDLVN |
| P40199 | YSGRETIYPNASLLIQNVTQNDTGFYTLQVIKSDLVNEEAT |
| P40199 | EEATGQFHVYPELPKPSISSNNSNPVEDKDAVAFTCEPEVQ |
| P40199 | NSNPVEDKDAVAFTCEPEVQNTTYLWWVNGQSLPVSPRLQL |
| P40199 | LWWVNGQSLPVSPRLQLSNGNMTLTLLSVKRNDAGSYECEI |
| P40199 | SVKRNDAGSYECEIQNPASANRSDPVTLNVLYGPDGPTISP |
| P40199 | GPDGPTISPSKANYRPGENLNLSCHAASNPPAQYSWFINGT |
| P40199 | NLNLSCHAASNPPAQYSWFINGTFQQSTQELFIPNITVNNS |
| P40199 | QYSWFINGTFQQSTQELFIPNITVNNSGSYMCQAHNSATGL |
| P40199 | FINGTFQQSTQELFIPNITVNNSGSYMCQAHNSATGLNRTT |
| P40199 | ITVNNSGSYMCQAHNSATGLNRTTVTMITVSGSAPVLSAVA |
| Q61400 | LLTYWSPATTAQVTVEAVPPNVTADNNVLLLVHNLPQTLRV |
| Q61400 | WYKGNSGAGHNEIGRFVTSINRSKMGLAHSGRETIYSNGSL |
| Q61400 | TASNKIILGPAHSDREIIYNNGSLFFQGVTKNDEGAYALDM |
| P31997 | ISNQQITPGPAYSNRETIYPNASLLMRNVTRNDTGSYTLQV |
| P31997 | PGPAYSNRETIYPNASLLMRNVTRNDTGSYTLQVIKLNLMS |
| P31997 | YSNRETIYPNASLLMRNVTRNDTGSYTLQVIKLNLMSEEVT |
| P31997 | EEVTGQFSVHPETPKPSISSNNSNPVEDKDAVAFTCEPETQ |
| P31997 | NSNPVEDKDAVAFTCEPETQNTTYLWWVNGQSLPVSPRLQL |
| P31997 | LWWVNGQSLPVSPRLQLSNGNRTLTLLSVTRNDVGPYECEI |
| P31997 | SVTRNDVGPYECEIQNPASANFSDPVTLNVLYGPDAPTISP |
| P31997 | GPDAPTISPSDTYYHAGVNLNLSCHAASNPPSQYSWSVNGT |
| P31997 | NLNLSCHAASNPPSQYSWSVNGTFQQYTQKLFIPNITTKNS |
| P31997 | QYSWSVNGTFQQYTQKLFIPNITTKNSGSYACHTTNSATGR |
| P31997 | ITTKNSGSYACHTTNSATGRNRTTVRMITVSDALVQGSSPG |
| Q9NZK5 | KGAALHLHDIGIVTMDWLVRNVTYRPHCHICFTPRGIMQFR |
| Q9NZK5 | RPSEKCSKWILLEDYRKRVQNVTEFDDSLLRNFTLVTQHPE |
| Q9NZK5 | LEDYRKRVQNVTEFDDSLLRNFTLVTQHPEVIYTNQNVVWS |
| Q9NZK5 | GETDWQGTSIDRNILDALMLNTTRIGHGFALSKHPAVRTYS |
| Q9XWD6 | TVKTIVDDYELKKVIHTVVYNDTEQCLNPLTGFQCTVEKRG |
| Q9XWD6 | TGKHCDIGCSRGRFGLQCKQNCTCPGLEFSDSNASCDAKTG |
| Q9XWD6 | RFGLQCKQNCTCPGLEFSDSNASCDAKTGQCQCESGYKGPK |
| Q9XWD6 | ASECNPETGSCVCKPGRTGKNCSEPCPLDFYGPNCAHQCQC |
| P34358 | DLMKKFAERFQSAKLKLSVKNESSEEQLLTVLRNDLPMLNE |
| P34358 | KNESSEEQLLTVLRNDLPMLNETFCAINSYAAGVVFDEVDV |

| | |
|---|---|
| P34358 | TVLMYGLGAVIFGAFVASFFNNTNSAIKAILVAWGAMIGIS |
| P34358 | GAFALAVEAISDYMRRERELNLTNMFNDSSLHFSLGWALVM |
| P34358 | VEAISDYMRRERELNLTNMFNDSSLHFSLGWALVMMIVDIL |
| P34358 | TSADFSLRTLFDFEAPEDDENQTDGVTAQNTRINEQVRNRV |
| P34358 | DMKRLLSDVKLDFKENEKAVNLSGGMKRKLCVCMALIGDSE |
| P34358 | AGMDPGARQDVQKLVEREKANRTILLTTHYMDEAERLGDWV |
| P34358 | DKVDKAIASRQNSRISHNSRNASEPSLKPAGYDTQSSTKSA |
| P34358 | QVLIPIILLGLVGSLTTLKSNNTDQFSVRSLTPSGIEPSKV |
| P34358 | SVRSLTPSGIEPSKVVWRFENGTIPEEAANFEKILRKSGGF |
| P34358 | ILRKSGGFEVLNYNTKNPLPNITKSLIGEMPPATIGMTMNS |
| P34358 | NRGSLIAIGSSQELKSLYGNNYTMTLSLYEPNQRDMVVQLV |
| P34358 | MVVQLVQTRLPNSVLKTTSTNKTLNLKWQIPKEKEDCWSAK |
| P08861 | EGPEQVIPINSGDLFVHPLWNRSCVACGNDIALIKLSRSAQ |
| P30122 | QHMAIAWVKRNIEAFGGDPDNITLFGESAGGASVSLQTLSP |
| P30122 | EEDFYKLVSGLTVTKGLRGANATYEVYTEPWAQDSSQETRK |
| P19835 | QHMAIAWVKRNIAAFGGDPNNITLFGESAGGASVSLQTLSP |
| P00450 | TFHSHGITYYKEHEGAIYPDNTTDFQRADDKVYPGEQYTYM |
| P00450 | CQNLNHLKAGLQAFFQVQECNKSSSKDNIRGKHVRHYYIAA |
| P00450 | AAEEIIWNYAPSGIDIFTKENLTAPGSDSAVFFEQGTTRIG |
| P00450 | NGRQKDVDKEFYLFPTVFDENESLLLEDNIRMFTTAPDQVD |
| P00450 | EWDYSPQREWEKELHHLQEQNVSNAFLDKGEFYIGSKYKKV |
| P00450 | KVFNPRRKLEFALLFLVFDENESWYLDDNIKTYSDHPEKVN |
| Q61147 | TFHAHGVTYTKEYEGAVYPDNTTDFQRADDKVLPGQQYVYV |
| Q61147 | KEKEKNIDQEFVLMFSVVDENLSWYLEDNIKTFCSEPEKVD |
| Q61147 | AAEEVIWNYAPSGTDIFTGENLTALESDSRVFFEQGATRIG |
| Q61147 | DGRQKDVDKEFYLFPTVFDENESLLLDDNIRMFTTAPDQVD |
| Q61147 | EWDYSPSRAWEKELHHLQEQNVSNVFLDKEEFFIGSKYKKV |
| Q61147 | KVFSPKKKMEFFLLFLVFDENESWYLDDNIKTYSEHPEKVN |
| Q9D6K9 | AETLGLLWGWLWSESFWLPQNVSWADLEGPGDGYGYPRAQH |
| P11597 | SIASSQVELVEAKSIDVSIQNVSVVFKGTLKYGYTTAWWLG |
| P11597 | DPVITASYLESHHKGHFIYKNVSEDLPLPTFSPTLLGDSRM |
| P11597 | VTVHCLKMPKISCQNKGVVVNSSVMVKFLFPRPDQQHSVAY |
| P11597 | SKKKLFLSLLDFQITPKTVSNLTESSSESVQSFLQSMITAV |
| Q7Z4R8 | EEVPEEWVLLHVVQGQIGAGNYSYLRLNHEGKIVLRMRSLK |
| P00751 | HCPRPHDFENGEYWPRSPYYNVSDEISFHCYDGYTLRGSAN |
| P00751 | NVSDEISFHCYDGYTLRGSANRTCQVNGRWSGQTAICDNGA |
| P00751 | PSGSMNIYLVLDGSDSIGASNFTGAKKCLVNLIEKVASYGV |
| P00751 | IYLVLDGSDSIGASNFTGAKKCLVNLIEKVASYGVKPRYGL |
| P00751 | ALQAVYSMMSWPDDVPPEGWNRTRHVIILMTDGLHNMGGDP |
| P04186 | RCPRPQDFENGEFWPRSPFYNLSDQISFQCYDGYVLRGSAN |
| P04186 | NLSDQISFQCYDGYVLRGSANRTCQENGRWDGQTAICDDGA |

| | |
|---|---|
| P04186 | PSGSMNIYLVLDGSDSIGSSNFTGAKRCLTNLIEKVASYGV |
| P04186 | ALQAVYSMMSWAGDAPPEGWNRTRHVIIIMTDGLHNMGGNP |
| P03953 | ILGGQEAAAHARPYMASVQVNGTHVCGGTLLDEQWVLSAAH |
| P03953 | HPGSRPDSLEDDLILFKLSQNASLGPHVRPLPLQYEDKEVE |
| P03953 | VVTHAGRRPDVLHQLRVSIMNRTTCNLRTYHDGVVTINMMC |
| P32038 | ILGGQEAMAHARPYMASVQVNGTHVCGGTLVDEQWVLSAAH |
| P08603 | FWSKEKPKCVEISCKSPDVINGSPISQKIIYKENERFQYKC |
| P08603 | CDIPVFMNARTKNDFTWFKLNDTLDYECHDGYESNTGSTTG |
| P08603 | EHGWAQLSSPPYYYGDSVEFNCSESFTMIGHRSITCIHGVW |
| P08603 | RGKEGWIHTVCINGRWDPEVNCSMAQIQLCPPPPQIPNSHN |
| P08603 | NCSMAQIQLCPPPPQIPNSHNMTTTLNYRDGEKVSVLCQEN |
| P08603 | PLCVEKIPCSQPPQIEHGTINSSRSSQESYAHGTKLSYTCE |
| P08603 | YAHGTKLSYTCEGGFRISEENETTCYMGKWSSPPQCEGLPC |
| P08603 | KAGEQVTYTCATYYKMDGASNVTCINSRWTGRPTCRDTSCV |
| P08603 | YQCRSPYEMFGDEEVMCLNGNWTEPPQCKDSTGKCGPPPPI |
| P06909 | YDCKPRFLLKGPNKIQCVDGNWTTLPVCIEEERTCGDIPEL |
| P06909 | AKCSVPPYHHGDSVEFICEENFTMIGHGSVSCISGKWTQLP |
| P06909 | RVLKSTGIEAIKPKLTEFTHNSTMDYKCRDKQEYERSICIN |
| P06909 | RDKQEYERSICINGKWDPEPNCTSKTSCPPPPQIPNTQVIE |
| P06909 | KKSYRTGEQVTFRCQSPYQMNGSDTVTCVNSRWIGQPVCKD |
| P06909 | RWIGQPVCKDNSCVDPPHVPNATIVTRTKNKYLHGDRVRYE |
| P05156 | PWQRCIEGTCVCKLPYQCPKNGTAVCATNRRSFPTYCQQKS |
| P05156 | PTYCQQKSLECLHPGTKFLNNGTCTAEGKFSVSLKHGNTDS |
| P05156 | LGFQQGADTQRRFKLSDLSINSTECLHVHCRGLETSLAECT |
| P05156 | CELPRSIPACVPWSPYLFQPNDTCIVSGWGREKDNERVFSL |
| P05156 | REKDNERVFSLQWGEVKLISNCSKFYGNRFYEKEMECAGTY |
| P05156 | GSIDACKGDSGGPLVCMDANNVTYVWGVVSWGENCGKPEFP |
| Q61129 | PTYCHQKSFECLHPEIKFSHNGTCAAEGKFNVSLIYGRTKT |
| Q61129 | CLHPEIKFSHNGTCAAEGKFNVSLIYGRTKTEGLVQVKLVD |
| Q61129 | NVACVDLGFPLGVRDIQGSFNISGNLHINDTECLHVHCRGV |
| Q61129 | FPLGVRDIQGSFNISGNLHINDTECLHVHCRGVETSLAECA |
| Q61129 | NGVNDCGDQSDELCCKGCRGNASLCKSGVCIPDQYKCNGEV |
| Q61129 | RGKDNQKVYSLRWGEVDLIGNCSQFYPDRYYEKEMQCAGTR |
| Q61129 | GSIDACKGDSGGPLVCEDINNVTYVWGIVSWGENCGKPEFP |
| P81475 | PPGSRLHFQCFQGFNLRGAPNRTCGEGGRWSGVTPVCDDGS |
| P81475 | SDVAASFMASLSQSVERADSNSSHGPTEKLPRXIRVDGSAX |
| P13569 | VAASLVVLWLLGNTPLQDKGNSTHSRNNSYAVIITSTSSYY |
| P13569 | VLWLLGNTPLQDKGNSTHSRNNSYAVIITSTSSYYVFYIYV |
| P01233 | LSMGGTWASKEPLRPRCRPINATLAVEKEGCPVCITVNTTI |
| P01233 | RPINATLAVEKEGCPVCITVNTTICAGYCPTMTRVLQGVLP |
| P30922 | SCFPDAIDPFLCTHVIYSFANISNNEIDTWEWNDVTLYDTL |

| | |
|---|---|
| Q7YS85 | SCFPDAIDPFLCTHVIYSFANISNNEIDTWEWNDVTLYDTL |
| P36222 | SCFPDALDRFLCTHIIYSFANISNDHIDTWEWNDVTLYGML |
| Q8SPQ0 | SCFPDAIDPFLCTHIIYSFANISNNEIDTWEWNDVTLYDTL |
| Q29411 | SCFPDAIDPFLCTHIIYSFANISNNEIDTWEWNDVTLYDTL |
| Q6TMG6 | SCFPDAIDPFLCTHVIYSFANISNNEIDTWEWNDVTLYDTL |
| P12024 | TDLGIVHCKNVPFPALPRMVNQSKVFMLHMENTGLREIEPY |
| P12024 | TDLGIVHCKNVPFPALPRMVNQSKVFMLHMENTGLREIEPY |
| P12024 | PLKSLRTLDISHNVIWSLSGNETYEIKASTKLNLDNLHLEY |
| P12024 | PLKSLRTLDISHNVIWSLSGNETYEIKASTKLNLDNLHLEY |
| P12024 | LEYNHIEVLPPNSFKYFDTVNRTFFDGNPIHTLREDAFKPA |
| P12024 | LEYNHIEVLPPNSFKYFDTVNRTFFDGNPIHTLREDAFKPA |
| P12024 | EDAFKPARIREIYMRYCGLTNISPVAFDSLVNSLQILDLSG |
| P12024 | SPVAFDSLVNSLQILDLSGNNLTKLHHKLFNNFDVLRVISM |
| P12024 | LLKLDLSGDRNDPTNLQTLRNMTRMRNMRSLSISRLGSSSV |
| P12024 | LPNFNFDYFDQVGTLSNLNVNVSHNQIRQLMYNSSWSGRNE |
| P12024 | GTLSNLNVNVSHNQIRQLMYNSSWSGRNEHGGMYHSNIKIL |
| P12024 | GTLSNLNVNVSHNQIRQLMYNSSWSGRNEHGGMYHSNIKIL |
| P12024 | RNEHGGMYHSNIKILDLSHNNISIIHPGYFRPAEISLTHLH |
| P12024 | YFRPAEISLTHLHLGYNSLMNTTRDVFGNMPHLQWLDLSYN |
| P12024 | KVMDKSFMGLENSLIKLGLENISLSTVPEIRLKYLREFRLG |
| P12024 | LREFRLGYNELPSIPQELAHNMSNLRMLDLSNNDLTNVPLM |
| P12024 | QALPHLRRLMLSGNPITSLNNNSFDGVNEDLEMLDISNFRL |
| P12024 | TRIVKKGSGPTQEMQTLQLGNPTDLQREMEGHLPSKLTNIT |
| P12024 | LGNPTDLQREMEGHLPSKLTNITFSGPQFTNLNERILRGMR |
| P12024 | NLNERILRGMRSPYLYMQLFNTSLQALPPNFFKYMGRVRNI |
| P12024 | FNTSLQALPPNFFKYMGRVRNISLDIRYHNRNLKKIPNPNT |
| Q13231 | FNGLKKMNPKLKTLLAIGGWNFGTQKFTDMVATANNRQTFV |
| Q11174 | MPKEKIIVGMPTYGRGWTLNNASAINPGTSGSPAKITQYVQ |
| Q18143 | VHYCFVNPPPKTRLSPNLLQNITTCTHLVYGSIPIDRDTGY |
| Q18143 | GELPSNEELARVTHINPLFYNGSIPFEETIQGTVEELSKEG |
| Q18143 | FLLGNDFHRKELAEGLVKAINSTNADGLEISWTSQPMVSDF |
| Q18143 | IVVATSQQSAYSDFYDYEHLNKTASLIVLHSHRLHSDSLPF |
| Q18143 | GVGGLALHNMHQDDPSAVCDNRTAFPILNALSRAQVCQTCL |
| Q18143 | TLTLGGNVNFNDVQQEQVVKNLTSLRSKMVKCGMVLSLSCG |
| Q18143 | MVLSLSCGDSEKYLNSILGDNMTFAIGNVMNIMEKYKFSGV |
| Q18143 | NTFVKKLAQKFESGKASNGCNRTLSARFSHYTQKPSTYYSV |
| Q18143 | LSHIALRMTDKHSVDLPFFFNHTKPEFPSTEKFVNIWKNVG |
| Q18143 | GNRAVYEHDYETLTGMTKHENGTINMPMIEDFRYKIGYIQR |
| P81908 | PLGRLRFKKPQSLTKWSNIWNATKYANSCYQNTDQSFPGFL |
| P81908 | TELSEDCLYLNVWIPAPKPKNATVMIWIYGGGFQTGTSSLP |
| P81908 | ILQSGSSNAPWAVTSLYEARNRTLTLAKRMGCSRDNETEMI |

| | |
|---|---|
| P81908 | LYEARNRTLTLAKRMGCSRDNETEMIKCLRDKDPQEILLNE |
| P81908 | VNKDEGTAFLVYGAPGFSKDNNSIITRKEFQEGLKIFFPRV |
| P81908 | GVMHGYEIEFVFGLPLERRVNYTRAEEILSRSIMKRWANFA |
| P81908 | EILSRSIMKRWANFAKYGNPNGTQNNSTRWPVFKSTEQKYL |
| P81908 | SIMKRWANFAKYGNPNGTQNNSTRWPVFKSTEQKYLTLNTE |
| P06276 | KSHTEDDIIIATKNGKVRGMNLTVFGGTVTAFLGIPYAQPP |
| P06276 | PLGRLRFKKPQSLTKWSDIWNATKYANSCCQNIDQSFPGFH |
| P06276 | TDLSEDCLYLNVWIPAPKPKNATVLIWIYGGGFQTGTSSLH |
| P06276 | ILQSGSFNAPWAVTSLYEARNRTLNLAKLTGCSRENETEII |
| P06276 | LYEARNRTLNLAKLTGCSRENETEIIKCLRNKDPQEILLNE |
| P06276 | VNKDEGTAFLVYGAPGFSKDNNSIITRKEFQEGLKIFFPGV |
| P06276 | GVMHGYEIEFVFGLPLERRDNYTKAEEILSRSIVKRWANFA |
| P06276 | EILSRSIVKRWANFAKYGNPNETQNNSTSWPVFKSTEQKYL |
| P06276 | RSIVKRWANFAKYGNPNETQNNSTSWPVFKSTEQKYLTLNT |
| P06276 | SIVKRWANFAKYGNPNETQNNSTSWPVFKSTEQKYLTLNTE |
| Q9Y4C5 | LYRCDLSVFQLYSPAGSGGRNLTTLGIFGAATNKVVCSSPL |
| Q9Y4C5 | RRVYDFVGLLVSPEMEQFALNMTSGSGSSSKPFVVSARNAT |
| Q9Y4C5 | ALNMTSGSGSSSKPFVVSARNATQAANAWRTALTFQQIKQV |
| Q9NS84 | GAEARAAEEGGANQSPRFPSNLSGAVGEAVSREKQHIYVHA |
| Q9NS84 | SVLRLYAPPGDPAARAPDTANLTTAALFRWRTNKVICSPPL |
| Q9NS84 | RRLLRFSGLRALAALDAFALNMTRGAAYGADRPFHLSARDA |
| Q5VTT2 | REAFPKGYDIEGPEKVKKLCNSTYRRLGTDESPIWTSETHE |
| P35522 | VGQFFGAGLMPRETINSLFDNYTWTKTIDPRGLGNSAQWFI |
| P47001 | TTATPTSSEKISSSASKTSTNATSSSCATPSLKDSSCKNSG |
| Q5QGZ9 | LKIEMKKMNKLQNISEELQRNISLQLMSNMNISNKIRNLST |
| Q5QGZ9 | LQNISEELQRNISLQLMSNMNISNKIRNLSTTLQTIATKLC |
| Q5QGZ9 | YFLSDDVQTWQESKMACAAQNASLLKINNKNALEFIKSQSR |
| Q19970 | GLYAPEGQTHEWKWLNGNEINKTLLWSSGEPYDHSMEGRER |
| Q19970 | WQKIENLFSFFGIGGSPTPHNATIPNDYEDEVLKNETSATV |
| Q19970 | GSPTPHNATIPNDYEDEVLKNETSATVKSTVKFSDSEEETS |
| Q6UVW9 | CSGDWLGVRDKCFYFSDDTRNWTASKIFCSLQKAELAQIDT |
| Q6UVW9 | GTDMHWIGLSRKQGDSWKWTNGTTFNGWFEIIGNGSFAFLS |
| Q6UVW9 | GDSWKWTNGTTFNGWFEIIGNGSFAFLSADGVHSSRGFIDI |
| Q9XVS3 | LGTTAFEDISFPARQPPAFVNQTEKCPDGWLRFADSCYWIE |
| Q9UQC9 | NQNLISNIKEMITEASFYLFNATKRRVFFRNIKILIPATWK |
| Q9UQC9 | RGCGKEGKYIHFTPNFLLNDNLTAGYGSRGRVFVHEWAHLR |
| Q9UQC9 | CPQENCIISKLFKEGCTFIYNSTQNATASIMFMQSLSSVVE |
| Q9UQC9 | ENVKPHHQLKNTVTVDNTVGNDTMFLVTWQASGPPEIILFD |
| Q9UQC9 | IRMSKSLQNIQDDFNNAILVNTSKRNPQQAGIREIFTFSPQ |
| Q14CN2 | MVTTASTYLFEATEKRFFFKNVSILIPENWKENPQYKRPKH |
| Q14CN2 | KDRLNRMNQAAKHFLLQTVENGSWVGMVHFDSTATIVNKLI |

| | |
|---|---|
| Q14CN2 | SQKSLQLESKGLTLNSNAWMNDTVIIDSTVGKDTFFLITWN |
| Q14CN2 | TWNSLPPSISLWDPSGTIMENFTVDATSKMAYLSIPGTAKV |
| Q14CN2 | NLQAKANPETLTITVTSRAANSSVPPITVNAKMNKDVNSFP |
| Q14CN2 | PSPMIVYAEILQGYVPVLGANVTAFIESQNGHTEVLELLDN |
| Q14CN2 | IRISASILDLRDSFDDALQVNTTDLSPKEANSKESFAFKPE |
| Q14CN2 | TTDLSPKEANSKESFAFKPENISEENATHIFIAIKSIDKSN |
| Q14CN2 | PKEANSKESFAFKPENISEENATHIFIAIKSIDKSNLTSKV |
| Q14CN2 | NISEENATHIFIAIKSIDKSNLTSKVSNIAQVTLFIPQANP |
| P51797 | FVASMVLGECRQMSSSSQIGNDSFQLQVTEDVNSSIKTFFC |
| P51797 | MSSSSQIGNDSFQLQVTEDVNSSIKTFFCPNDTYNDMATLF |
| P51797 | SFQLQVTEDVNSSIKTFFCPNDTYNDMATLFFNPQESAILQ |
| Q9NY35 | YMAASIGTDFWYEYRSPVQENSSDLNKSIWDEFISDEADEK |
| Q9NY35 | DEFISDEADEKTYNDALFRYNGTVGLWRRCITIPKNMHWYS |
| Q9CQX5 | YMAASIGTDFWYEYRSPIQENSSDSNKIAWEDFLGDEADEK |
| Q9CQX5 | EDFLGDEADEKTYNDVLFRYNGSLGLWRRCITIPKNTHWYA |
| Q8R373 | LTDNEGNQKVVITYSSRHVYNNLTEEQKGRVAFASNFLAGD |
| Q8R373 | TDNEGNQKVVITYSSRHVYNNLTEEQKGRVAFASNFLAGDA |
| Q8R373 | EHLPPKSRIDYNNPGRVLLQNLTMASSGLYQCTAGNEAGKE |
| Q13286 | SYVVMLSAAHDILSHKRTSGNQSHVDPGPTPIPHNSSSRFD |
| Q13286 | HKRTSGNQSHVDPGPTPIPHNSSSRFDCNSVSTAAVLLADI |
| Q13286 | VVYFAEYFINQGLFELLFFWNTSLSHAQQYRWYQMLYQAGV |
| Q96KA5 | IDLVLNVEDFDVESKFERTVNVSVPKKTRNNGTLYAYIFLH |
| Q96KA5 | DVESKFERTVNVSVPKKTRNNGTLYAYIFLHHAGVLPWHDG |
| Q96KA5 | YLPILFIDQLSNRVKDLMVINRSTTELPLTVSYDKVSLGRL |
| O96005 | DGARSAVVAAGGGSSGQVTSNGSIGRDPPAETQPQNPPAQP |
| O96005 | KDTLMNLHVYISEHEHFTDFNATSALFWEQHDLVYGDWTSG |
| O96005 | NSDGCYEHFAELDIPQSVQQNGSIYIHVYFTKSGFHPDPRQ |
| O96005 | EMIKRAEDYGPVEVISHWHPNITINIVDDHTPWVKGSVPPP |
| O96005 | YYPIIYFNDYWNLQKDYYPINESLASLPLRVSFCPLSLWRW |
| O96005 | VRSVFFGVFQSFVVLLYILDNETNFVVQVSVFIGVLIDLWK |
| P10909 | ERKTLLSNLEEAKKKKEDALNETRESETKLKELPGVCNETM |
| P10909 | DALNETRESETKLKELPGVCNETMMALWEECKPCLKQTCMK |
| P10909 | YARVCRSGSGLVGRQLEEFLNQSSPFYFWMNGDRIDSLLEN |
| P10909 | PTEFIREGDDDRTVCREIRHNSTGCLRMKDQCDKCREILSV |
| P10909 | RMKDQCDKCREILSVDCSTNNPSQAKLRRELDESLQVAERL |
| P10909 | AERLTRKYNELLKSYQWKMLNTSSLLEQLNEQFNWVSRLAN |
| P10909 | NTSSLLEQLNEQFNWVSRLANLTQGEDQYYLRVTTVASHTS |
| Q06890 | DALEDTRDSEMKLKAFPEVCNETMMALWEECKPCLKHTCMK |
| Q06890 | YARVCRSGSGLVGQQLEEFLNQSSPFYFWMNGDRIDSLLES |
| Q06890 | DVDFLREGEDDRTVCKEIRRNSTGCLKMKGQCEKCQEILSV |
| Q06890 | ILSVDCSTNNPAQANLRQELNDSLQVAERLTEQYKELLQSF |

| | |
|---|---|
| Q06890 | AERLTEQYKELLQSFQSKMLNTSSLLEQLNDQFNWVSQLAN |
| Q06890 | NTSSLLEQLNDQFNWVSQLANLTQGEDKYYLRVSTVTTHSS |
| P23946 | FVLTAAHCAGRSITVTLGAHNITEEEDTWQKLEVIKQFRHP |
| P23946 | EEEDTWQKLEVIKQFRHPKYNTSTLHHDIMLLKLKEKASLT |
| Q96KN2 | LTEEEINTYKAIHLDLEEYRNSSRVEKFLFDTKEEILMHLW |
| Q96KN2 | KTVIPGRVIGKFSIRLVPHMNVSAVEKQVTRHLEDVFSKRN |
| Q3TWN3 | ALRVSERTRVKLRVYGQNINNETWSRIAFTEHERRRHTPSE |
| Q9BT09 | LHNLVHKGVKVVMDIPYELWNETSAEVADLKKQCDVLVEEF |
| O88507 | ADVTLPCGTASWDAAVTWRVNGTDLAPDLLNGSQLILRSLE |
| O88507 | SWDAAVTWRVNGTDLAPDLLNGSQLILRSLELGHSGLYACF |
| O88507 | PKGFYCSWHLPTPTYIPNTFNVTVLHGSKIMVCEKDPALKN |
| O88507 | HLFSTIKYKVSISVSNALGHNTTAITFDEFTIVKPDPPENV |
| Q12860 | FVSQTNGNLYIANVEASDKGNYSCFVSSPSITKSVFSKFIP |
| Q12860 | KPYPADIVVQFKDVYALMGQNVTLECFALGNPVPDIRWRKV |
| Q12860 | KDKHQARIYVQAFPEWVEHINDTEVDIGSDLYWPCVATGKP |
| Q12860 | KPKAAPKPKFSWSKGTEWLVNSSRILIWEDGSLEINNITRN |
| Q12860 | EWLVNSSRILIWEDGSLEINNITRNDGGIYTCFAENNRGKA |
| Q12860 | ITRNDGGIYTCFAENNRGKANSTGTLVITDPTRIILAPINA |
| Q12860 | ITDPTRIILAPINADITVGENATMQCAASFDPALDLTFVWS |
| Q12860 | RNAQLKHAGRYTCTAQTIVDNSSASADLVVRGPPGPPGGLR |
| Q12860 | SSVRSGSRYIITWDHVVALSNESTVTGYKVLYRPDGQHDGK |
| Q9P232 | KITLHCEARGNPSPHYRWQLNGSDIDMSMEHRYKLNGGNLV |
| Q9P232 | FVSQETGHLYISKVEPSDVGNYTCVVTSMVTNARVLGSPTP |
| Q9P232 | GAALVLEERTQIENGALTISNLSVTDSGMFQCIAENKHGLV |
| Q9P232 | VSVQEHERISLLNDGGLKIANVTKADAGTYTCMAENQFGKA |
| Q9P232 | VTKADAGTYTCMAENQFGKANGTTHLVVTEPTRITLAPSNM |
| Q9P232 | VTTWIQTVVTSPDTPRYVFRNESIVPYSPYEVKVGVYNNKG |
| Q9P232 | VRYWNGGGKEESSSKMKVAGNETSARLRGLKSNLAYYTAVR |
| Q9P232 | YYTAVRAYNSAGAGPFSATVNVTTKKTPPSQPPGNVVWNAT |
| Q9P232 | TVNVTTKKTPPSQPPGNVVWNATDTKVLLNWEQVKAMENES |
| Q9P232 | VWNATDTKVLLNWEQVKAMENESEVTGYKVFYRTSSQNNVQ |
| Q9P232 | TGYKVFYRTSSQNNVQVLNTNKTSAELVLPIKEDYIIEVKA |
| Q02246 | QVLLACRARASPPATYRWKMNGTEMKLEPGSRHQLVGGNLV |
| Q02246 | PTDGRHFVSQTTGNLYIARTNASDLGNYSCLATSHMDFSTK |
| Q02246 | FVSQTTGNLYIARTNASDLGNYSCLATSHMDFSTKSVFSKF |
| Q02246 | QPRAAPKAVVLWSKGTEILVNSSRVTVTPDGTLIIRNISRS |
| Q02246 | EILVNSSRVTVTPDGTLIIRNISRSDEGKYTCFAENFMGKA |
| Q02246 | ISRSDEGKYTCFAENFMGKANSTGILSVRDATKITLAPSSA |
| Q02246 | VRDATKITLAPSSADINLGDNLTLQCHASHDPTMDLTFTWT |
| Q02246 | EEPRVAPTKVWAKGVSSSEMNVTWEPVQQDMNGILLGYEIR |
| Q02246 | YHVTVRAYNRAGTGPASPSANATTMKPPPRRPPGNISWTFS |

| | |
|---|---|
| Q02246 | PASPSANATTMKPPPRRPPGNISWTFSSSSLSIKWDPVVPF |
| Q02246 | SWTFSSSSLSIKWDPVVPFRNESAVTGYKMLYQNDLHLTPT |
| Q8IWV2 | KVKLNCEVKGNPKPHIRWKLNGTDVDTGMDFRYSVVEGSLL |
| Q8IWV2 | DTGMDFRYSVVEGSLLINNPNKTQDAGTYQCTATNSFGTIV |
| Q8IWV2 | FVSQETGNLYIAKVEKSDVGNYTCVVTNTVTNHKVLGPPTP |
| Q8IWV2 | LKNGEPLLTRDRIQIEQGTLNITIVNLSDAGMYQCLAENKH |
| Q8IWV2 | PLLTRDRIQIEQGTLNITIVNLSDAGMYQCLAENKHGVIFS |
| Q8IWV2 | DILKENERITISEDGNLRIINVTKSDAGSYTCIATNHFGTA |
| Q8IWV2 | PSRPSEKRRTEEALPEVTPANVSGGGGSKSELVITWETVPE |
| Q8IWV2 | KMIWMLTVLASADASRYVFRNESVHPFSPFEVKVGVFNNKG |
| Q8IWV2 | VKYWRHEDKEENARKIRTVGNQTSTKITNLKGSVLYHLAVK |
| Q8IWV2 | YHLAVKAYNSAGTGPSSATVNVTTRKPPPSQPPGNIIWNSS |
| Q8IWV2 | TVNVTTRKPPPSQPPGNIIWNSSDSKIILNWDQVKALDNES |
| Q8IWV2 | IWNSSDSKIILNWDQVKALDNESEVKGYKVLYRWNRQSSTS |
| Q8IWV2 | KGYKVLYRWNRQSSTSVIETNKTSVELSLPFDEDYIIEIKP |
| Q69Z26 | KVKLSCEVKGNPKPHIRWKINGTDVDIGMDFRYSVVDGSLL |
| Q69Z26 | DIGMDFRYSVVDGSLLINNPNKTQDAGTYQCIATNSFGTIV |
| Q69Z26 | FVSQETGNLYIAKVEKSDVGNYTCVVTNTVTNHKVLGPPTP |
| Q69Z26 | LKNGDPLLTRDRIQIEQGTLNITIVNLSDAGMYQCVAENKH |
| Q69Z26 | PLLTRDRIQIEQGTLNITIVNLSDAGMYQCVAENKHGVIFS |
| Q69Z26 | EILRENERITISEDGNLRIINVTKSDAGSYTCIATNHFGTA |
| Q69Z26 | PSRPSEKRRTEEALPEVTPANVSGGGGSKSELVITWETVPE |
| Q69Z26 | KMIWMLTVLASADASRYVFRNESVRPFSPFEVKVGVFNNKG |
| Q69Z26 | VKYWRHDDKEENAKKIRTVGNQTSTKITNLKGSALYHLSVK |
| Q69Z26 | YHLSVKAYNSAGTGPSSATVNVTTRKPPPSQPPGNIIWNSS |
| Q69Z26 | TVNVTTRKPPPSQPPGNIIWNSSDSKIILNWDQVKALDNES |
| Q69Z26 | IWNSSDSKIILNWDQVKALDNESEVKGYKVLYRWNRQSSTS |
| Q69Z26 | KGYKVLYRWNRQSSTSVIETNKTSVELSLPFDEDYIIEIKP |
| P02457 | PADVAIQLTFLRLMSTEATQNVTYHCKNSVAYMDHDTGNLK |
| P02452 | PADVAIQLTFLRLMSTEASQNITYHCKNSVAYMDQQTGNLK |
| P11087 | LRVPNGETWKPEVCLICICHNGTAVCDDVQCNEELDCPNPQ |
| P11087 | PADVAIQLTFLRLMSTEASQNITYHCKNSVAYMDQQTGNLK |
| P02454 | LRVPNGETWKPDVCLICICHNGTAVCDGVLCKEDLDCPNPQ |
| P02454 | PADVAIQLTFLRLMSTEASQNITYHCKNSVAYMDQQTGNLK |
| P02459 | PNTANVQMTFLRLLSTEGSQNITYHCKNSIAYLDEAAGNLK |
| P02458 | PNTANVQMTFLRLLSTEGSQNITYHCKNSIAYLDEAAGNLK |
| P06681 | CLLFLYPGLADSAPSCPQNVNISGGTFTLSHGWAPGSLLTY |
| P06681 | PVSFENGIYTPRLGSYPVGGNVSFECEDGFILRGSPVRQCR |
| P06681 | FLIFKESASLMVDRIFSFEINVSVAIITFASEPKVLMSVLN |
| P06681 | SRDMTEVISSLENANYKDHENGTGTNTYAALNSVYLMMNNQ |
| P06681 | QVFEHMLDVSKLTDTICGVGNMSANASDQERTPWHVTIKPK |

| | |
|---|---|
| P06681 | HMLDVSKLTDTICGVGNMSANASDQERTPWHVTIKPKSQET |
| P06681 | RDHENELLNKQSVPAHFVALNGSKLNINLKMGVEWTSCAEV |
| P06681 | MGVEWTSCAEVVSQEKTMFPNLTDVREVVTDQFLCSGTQED |
| P01024 | KKLVLSSEKTVLTPATNHMGNVTFTIPANREFKSEKGRNKF |
| P01024 | HFISDGVRKSLKVVPEGIRMNKTVAVRTLDPERLGREGVQK |
| P01024 | EEKKHYLMWGLSSDFWGEKPNLSYIIGKDTWVEHWPEEDEC |
| P98093 | DQEVAKNKEIAVSVEIMTPENITIFREIVNPDKGVKSGQFK |
| P01027 | HFISDGVKKTLKVVPEGMRINKTVAIHTLDPEKLGQGGVQK |
| P01027 | QKGKKYLMWGLSSDLWGEKPNTSYIIGKDTWVEHWPEAEEC |
| P02462 | GLPGIPGQDGPPGPPGIPGCNGTKGERGPLGPPGLPGFAGN |
| P08572 | GIPGHPGQGGPRGRPGYDGCNGTQGDSGPQGPPGSEGFTGP |
| P0C0L4 | DISEPGTWKISARFSDGLESNSSTQFEVKKYVLPNFEVKIT |
| P0C0L4 | SVRRFEQLELRPVLYNYLDKNLTVSVHVSPVEGLCLAGGGG |
| P0C0L4 | ALDALSAYWIASHTTEERGLNVTLSSTGRNGFKSHALQLNN |
| P0C0L4 | GGNSKGTLKVLRTYNVLDMKNTTCQDLQIEVTVKGHVEYTM |
| P0C0L5 | DISEPGTWKISARFSDGLESNSSTQFEVKKYVLPNFEVKIT |
| P0C0L5 | SVRRFEQLELRPVLYNYLDKNLTVSVHVSPVEGLCLAGGGG |
| P0C0L5 | ALDALSAYWIASHTTEERGLNVTLSSTGRNGFKSHALQLNN |
| P0C0L5 | GGNSKGTLKVLRTYNVLDMKNTTCQDLQIEVTVKGHVEYTM |
| P08649 | DISEPGTWKISARFSDGLESNRSTHFEVKKYVLPNFEVKLT |
| P08649 | VFQTAGLAFSDGDRLTQTKENLSCPKEKKSRQKRNVNFQKA |
| P08649 | QPACREPFLSCCKFAEDLRRNQTRSQAGLARAQDMLQEEDL |
| P08649 | TLDALSAYWIASHTTEEKALNVTLSSMGRNGYKSHLLQLNN |
| P08649 | GGNSKGTLKILRTYNVLDMKNTTCQDLRIEVTVTGYVEYTR |
| P01029 | DISEPGTWKISARFSDGLESNRSTHFEVKKYVLPNFEVKIT |
| P01029 | QQACREPFLSCCKFAEDLRRNQTRSQAHLARNNHNMLQEED |
| P01029 | TLDALSAYWIASHTTEEKALNVTLSSMGRNGLKTHGLHLNN |
| P01029 | EGNSKGTLKILRTYNVLDMKNTTCQDLQIEVKVTGAVEYAW |
| P25940 | PEGHFPENFSLLITLRGQPANQSVLLSIYDERGARQLGLAL |
| P25940 | ALGPALGLLGDPFRPLPQQVNLTDGRWHRVAVSIDGEMVTL |
| P01031 | GPRCIKAFTECCVVASQLRANISHKDMQLGRLHMKTLLPVS |
| P01031 | SSSHLVTFTVLPLEIGLHNINFSLETWFGKEILVKTLRVVP |
| P01031 | QNQNSICNSLLWLVENYQLDNGSFKENSQYQPIKLQGTLPV |
| P01031 | ELVKGRQYLIMGKEALQIKYNFSFRYIYPLDSLTWIEYWPR |
| P06684 | KRSITHDTDGVAVFVLNLPSNVTVLKFEIRTDDPELPEENQ |
| P06684 | SSSHLVTFTLLPLEIGLHSINFSLETSFGKDILVKTLRVVP |
| P06684 | QDENSICNSLLWLVEKCQLENGSFKENSQYLPIKLQGTLPA |
| P06684 | NLVKGKQYLIMGKEVLQIKHNFSFKYIYPLDSSTWIEYWPT |
| P12109 | TPDHLEPRLSIIATDHTYRRNFTAADWGQSRDAEEAISQTI |
| P12109 | AGPPGDPGLMGERGEDGPAGNGTEGFPGFPGYPGNRGAPGI |
| P12109 | GTEGFPGFPGYPGNRGAPGINGTKGYPGLKGDEGEAGDPGD |

| | |
|---|---|
| P12109 | PGLSLVKENYAELLEDAFLKNVTAQICIDKKCPDYTCPITF |
| P12109 | VQYSGTGQQRPERASLQFLQNYTALASAVDAMDFINDATDV |
| P12110 | KNLQGISSFRRGTFTDCALANMTEQIRQDRSKGTVHFAVVI |
| P12110 | GEKGEFGADGRKGAPGLAGKNGTDGQKGKLGRIGPPGCKGD |
| P12110 | RCGALDVVFVIDSSESIGYTNFTLEKNFVINVVNRLGAIAK |
| P12110 | EKHESENLYSIACDKPQQVRNMTLFSDLVAEKFIDDMEDVL |
| P12110 | VALLQFGGPGEQQVAFPLSHNLTAIHEALETTQYLNSFSHV |
| P12110 | GGARRHAELSFVFLTDGVTGNDSLHESAHSMRKQNVVPTVL |
| P12111 | HTEFLLNTYRTKQEVLSHISNMSYIGGTNQTGKGLEYIMQS |
| P12111 | YRTKQEVLSHISNMSYIGGTNQTGKGLEYIMQSHLTKAAGS |
| P12111 | EGALKEIASEPLNMHMFNLENFTSLHDIVGNLVSCVHSSVS |
| P12111 | TLKDITAQDSADIIFLIDGSNNTGSVNFAVILDFLVNLLEK |
| P12111 | GYRGYPGDEGGPGERGPPGVNGTQGFQGCPGQRGVKGSRGF |
| P12111 | GERGFPGYPGPKGNPGEPGLNGTTGPKGIRGRRGNSGPPGI |
| P12111 | ITPLFLTRQEDRQLINALQINNTAVGHALVLPAGRDLTDFL |
| P12111 | ASQHFARVAVVQHAPSESVDNASMPPVKVEFSLTDYGSKEK |
| P12111 | GDQPTKNLVKFGHKQVNVPNNVTSSPTSNPVTTTKPVTTTK |
| P12111 | PYFYDLTVTSAHDQSLVLKQNLTVTDRVIGGLLAGQTYHVA |
| A6NMZ7 | ATSQFHFNLRTVRDLSMFSQNMTHIIKDVIKYKEGAVDDIF |
| A6NMZ7 | SVSALDIKENCMRVGLVAYSNETKVINSLSMGINKSEVLQH |
| A6NMZ7 | VGLVAYSNETKVINSLSMGINKSEVLQHIQNLSPRTGKAYT |
| A6NMZ7 | KVINSLSMGINKSEVLQHIQNLSPRTGKAYTGAAIKKLRKE |
| A6NMZ7 | RKNQGVPQIAVLVTHRDSEDNVTKAAVNLRREGVTIFTLGI |
| A6NMZ7 | GKAIENIRQMGGNTNTGAALNFTLSLLQKAKKQRGNKVPCH |
| A6NMZ7 | GVPQVLIVITDGESHDADKLNATAKALRDKGILVLAVGIDG |
| A6NMZ7 | FVETFGGLKGIFSDVTASVCNSSKVDCEIDKVDLVFLMDGS |
| A6NMZ7 | VKGPSLLNANLLDSLWDTFQNKSAARGKVVLLFSDGLDDDV |
| P13671 | QASHKKDSSFIRIHKVMKVLNFTTKAKDLHLSDVFLKALNH |
| P13671 | GSCQDGRQLEWGLERTRLSSNSTKKESCGYDTCYDWEKCSA |
| Q02388 | EAVSGTARTTALEGPELTIQNTTAHSLLVAWRSVPGATGYR |
| Q02388 | SVVVRTAPEPVGRVSRLQILNASSDVLRITWVGVTGATAYR |
| Q02388 | QAVQVGLLSYSHRPSPLFPLNGSHDLGIILQRIRDMPYMDP |
| P10643 | NVLSYTFQVKINNDFNYEFYNSTWSYVKHTSTEHTSSSRKR |
| P10643 | SKRILPLTVCKMHVLHCQGRNYTLTGRDSCTLPASAEKACG |
| P07357 | EDIISRVRGGSSGWSGGLAQNRSTITYRSWGRSLKYNPVVI |
| P07358 | KKRYRYAYLLQPSQFHGEPCNFSDKEVEDCVTNRPCRSQVR |
| P07358 | TQGKYEFILKEYESYSDFERNVTEKMASKSGFSFGFKIPGI |
| Q8BH35 | CLHFPGSRGGKPDFFETKAVNGSLVKSRPVRSVAEAPAPID |
| Q8BH35 | EYESYSDFERLVIEKKTHMFNFTSGFKVDGVMDLGIKVESN |
| Q8VCG4 | QRTRKPIGSTSPISTIQAQVNFSAQKFAGTWLLVAVGSSCR |
| Q8VCG4 | LYLEQGRKLSVKLYVRSLPVNDSVLDVFERRVREANLTEDQ |

| | |
|---|---|
| Q8VCG4 | RSLPVNDSVLDVFERRVREANLTEDQILFFPKYGFCETADQ |
| P02748 | ETASSISLHGKGSFRFSYSKNETYQLFLSYSSKKEKMFLHV |
| P02748 | SVGAEFNKDDCVKRGEGRAVNITSENLIDDVVSLIRGGTRK |
| P06683 | REEQEQHYPIPIDCRMSPWSNWSECDPCLKQRFRSRSILAF |
| P06683 | EVPEKGAGEVSPAEHSSKPTNISAKFKFSYFMGKNFRRLSS |
| P06683 | SVTASVNADGCIKTDNGKTVNITRDNIIDDVISFIRGGTRE |
| Q99715 | PASSTSVVLSSLKPETLYLVNVTAEYEDGFSIPLAGEETTE |
| Q99715 | TLPILTTQAPKSGPRNLQVYNATSNSLTVKWDPASGRVQKY |
| Q99715 | AQYDSGLSVPLTDQGTTLYLNVTDLKTYQIGWDTFCVKWSP |
| Q99715 | CPLIYLDGYTSPGFKMLEAYNLTEKNFASVQGVSLESGSFP |
| Q99715 | SVKIYIDCYEIIEKDIKEAGNITTDGYEILGKLLKGERKSA |
| Q05707 | QLNLQNTATKAIIQGLMPDQNYTVQIIAYNKDKESKPAQGQ |
| Q05707 | IKDLEKRKDPKPRVKVVDRGNGSRPSSPEEVKFVCQTPAIA |
| Q05707 | ITGPPTELITSEVTARSFMVNWTHAPGNVEKYRVVYYPTRG |
| Q05707 | VSETLVKVVIDCKQVGEKAMNASANITSDGVEVLGKMVRSR |
| Q05707 | LVKVVIDCKQVGEKAMNASANITSDGVEVLGKMVRSRGPGG |
| P39059 | PFEDMELSGEPVPEGTLETTNMSIIQHSSPKQGSGEILNDT |
| P39059 | TTNMSIIQHSSPKQGSGEILNDTLEGVHSVDGDPITDSGSG |
| P39059 | GVDGATGLPGMKGEKGARGPNGSVGEKGDPGNRGLPGPPGK |
| P39059 | GPPGPRGPPGHIKVLSNSLINITHGFMNFSDIPELVGPPGP |
| P39059 | PPGHIKVLSNSLINITHGFMNFSDIPELVGPPGPDGLPGLP |
| P39059 | LKGENGDKGFKGEKGEKGDINGSFLMSGPPGLPGNPGPAGQ |
| Q9UMD9 | PGQPGPQGPPGISKVFSAYSNVTADLMDFFQTYGAIQGPPG |
| P39061 | PGFPGTWVSHVAPSSGTGLSNDSALAGNGSLTSTSRCLPLP |
| P39061 | VSHVAPSSGTGLSNDSALAGNGSLTSTSRCLPLPPTLTLCS |
| P39061 | AAQVVVSLGVKLSEVRDGQQNISLLYTEPGASQTQTGASFR |
| P39061 | GPPGPPGVPGLPGEPGRFGINGSYAPGPAGLPGVPGKEGPP |
| P39060 | PQGPLPVQPTADTTTHVTPRNGSTEPATAPGSPEPPSELLE |
| P39060 | GVGAEILNVAKGIRSFVQLWNDTVPTESLARAETLVLETPV |
| P39060 | VLETPVGPLALAGPSSTPQENGTTLWPSRGIPSSPGAHTTE |
| P39060 | GPPGPPGVPGLPGEPGRFGVNSSDVPGPAGLPGVPGREGPP |
| Q8K4Q8 | CALLTITVAILGYKVVEKMDNVTDGMETSHQTYDNKLTAVE |
| Q8K4Q8 | LQANGDSLVDRQSQLKETLQNNSFLITTVNKTLQAYNGYVT |
| Q8K4Q8 | QANGDSLVDRQSQLKETLQNNSFLITTVNKTLQAYNGYVTN |
| Q8K4Q8 | DRQSQLKETLQNNSFLITTVNKTLQAYNGYVTNLQQDTSVL |
| Q8K4Q8 | AKKDTDWLKEKVQSLQTLAANNSALAKANNDTLEDMNSQLS |
| O76368 | EGTPGKRGRRGRHGISLVAPNGTINEDLKKLLKTELMPLLI |
| P06298 | SESIRKYVMTHFRWNKFGRRNSTGNDGSNTGYKREDISSYP |
| P01189 | TENPRKYVMGHFRWDRFGRRNSSSSGSSGAGQKREDVSAGE |
| P01190 | TENPRKYVMGHFRWDRFGRRNGSSSSGVGGAAQKREEEVAV |
| P06299 | SESIRKYVMTHFRWNKFGRRNNTGNDGSSGGYKREDISNYP |

| | |
|---|---|
| P49747 | ACIQTESGARCGPCPAGFTGNGSHCTDVNECNAHPCFPRVR |
| P02866 | IPSGSTGRLLGLFPDANVIRNSTTIDFNAAYNADTIVAVEL |
| P23805 | RFTYPTGEILVYSNWADGEPNNSDEGQPENCVEIFPDGKWN |
| Q9VN14 | GLDINDMERIPRGPYFVKQPNDTTFDVNKNRLINDVTLSCL |
| Q9VN14 | VFVSRDGALYFSFIETVDRANYSCTVQTLVSDTGRNGPFFP |
| Q9VN14 | VAGDEIRLECMAFGYPIPSYNWTRQGLPLQRNAYTINYGRV |
| Q9VN14 | GLPLQRNAYTINYGRVLIIQNATTNDNGEYSCTITNPRKTL |
| Q9VN14 | QRNAYTINYGRVLIIQNATTNDNGEYSCTITNPRKTLMKSI |
| Q9VN14 | IKDYNSDVTFICEAFAIPDANYTWYKNAERLDPANINRDRY |
| Q9VN14 | MKPSFKKHPLESEVYAVYNGNTTIVCDPEAAPRPKFQWKKD |
| Q9VN14 | KNNHDGTGRIIVDWNRLTVHNTSMRDAGDYECVVKSAVNEI |
| Q9VN14 | DGSHNGRAIRYYNILGRTNWNRTWVNVSTHVQAREVDRYTS |
| Q9VN14 | GRAIRYYNILGRTNWNRTWVNVSTHVQAREVDRYTSRQQAE |
| Q9VN14 | IHSAEDMPQVAPQKPIALAYNSTCFNVTWQPIDMSRENIRG |
| Q9VN14 | DMPQVAPQKPIALAYNSTCFNVTWQPIDMSRENIRGKLIGH |
| Q9VN14 | EPVEGYKVRIWESDQNMITANNTIVPIGQKLESYINNLTPG |
| Q8TAV3 | QLDGYRGRPFPLALLGWAPSNITFALLFGRRFDYRDPVFVS |
| P29602 | CTVGTHCSNGQKLSINVVAANATVSMPPPSSSPPSSVMPPP |
| Q17802 | LAFDEARVICDYVMNVPECTNGSGNDEGSADETTPESSGEM |
| P41996 | MFCPAGLKFSESTVRCDYESNVSECQETSGEESGEASGEQS |
| P41996 | LFSDGLCSATYHQCTAGQLINFTCAASNAVFSAANTECVDS |
| O16883 | PEESEAEIVGNLLESSGEKENVTEFATEKEEIDPSTLRVHD |
| O16883 | MFETMTSGLNYMCVEQKLAFNATIKCIDDEAGVVQSECDTQ |
| O16883 | SPLDVSVKTLDQLILDMYSNNKTEELNISEKNNVTSTFSEP |
| O16883 | VKTLDQLILDMYSNNKTEELNISEKNNVTSTFSEPSEKEDE |
| O16883 | LILDMYSNNKTEELNISEKNNVTSTFSEPSEKEDEASTTVI |
| O16883 | SEKEDEASTTVISVISPLHTNATDSEILEHISEKSTEESSG |
| O16883 | GEGSGEYDASGSSGDNSGEFNSSGSSGEASEEGESSGSEDQ |
| O16883 | GEGSAEYDASGSSGDNSGDFNSSGSSGEASGVGESSGSEDQ |
| O16883 | NYKKIEVIESSGDYEFSGSSNESIEQSKEGSAASIYEILQA |
| P82474 | NNGGINSEETYPYRGQDGICNSTVNAPVVSIDSYENVPSHN |
| P82474 | MDAAGRDFQLYRSGIFTGSCNISANHALTVVGYGTENDKDF |
| Q21771 | LSNIVLANSQMVLDSARLSLNETETETGTSAPATCVSSAVC |
| Q21771 | NGACNSITATCDCFAAYTKNNLTLAEALTSFCNVETCNGAE |
| P22792 | IIFVETSFTTLETRAFGSNPNLTKVVFLNTQLCQFRPDAFG |
| P22792 | DAFGGLPRLEDLEVTGSSFLNLSTNIFSNLTSLGKLTLNFN |
| P22792 | LEDLEVTGSSFLNLSTNIFSNLTSLGKLTLNFNMLEALPEG |
| P22792 | LPQGVFGKLGSLQELFLDSNNISELPPQVFSQLFCLERLWL |
| P22792 | LWLQRNAITHLPLSIFASLGNLTFLSLQWNMLRVLPAGLFA |
| P22792 | LPAGIFRDLEELVKLYLGSNNLTALHPALFQNLSKLELLSL |
| P22792 | LVKLYLGSNNLTALHPALFQNLSKLELLSLSKNQLTTLPEG |

| | |
|---|---|
| P22792 | QCRWLNVQLSPQQGSLGLQYNASQEWDLRSSCGSLRLTVSI |
| Q9DBB9 | IVFVETAFTTVRTRAFSGSPNLTKVVFLNTQVRHLEPDAFG |
| Q9DBB9 | DAFGGLPRLQDLEITGSPVSNLSAHIFSNLSSLEKLTLDFD |
| Q9DBB9 | LQDLEITGSPVSNLSAHIFSNLSSLEKLTLDFDRLAGLPED |
| Q9DBB9 | LWLQHNAICHLPVSLFSSLHNLTFLSLKDNALRTLPEGLFA |
| Q9DBB9 | LLHLSLSYNQLETIPEGAFTNLSRLVSLTLSHNAITDLPEH |
| Q9DBB9 | LPEHVFRNLEQLVKLSLDSNNLTALHPALFHNLSRLQLLNL |
| Q9DBB9 | LVKLSLDSNNLTALHPALFHNLSRLQLLNLSRNQLTTLPGG |
| Q9DBB9 | NNLTALHPALFHNLSRLQLLNLSRNQLTTLPGGIFDTNYDL |
| Q9DBB9 | RWLNIQLSSRDGSDSAAMVYNSSQEWGLRSSCGLLRVTVSI |
| Q8IYJ1 | YFFEEKQNLRFDVYNVDSKTNISKPKDFLGQAFLALGEVIG |
| P43510 | GGDPLAAWRYWVKDGIVTGSNYTANNGCKPYPFPPCEHHSK |
| P43510 | AAWRYWVKDGIVTGSNYTANNGCKPYPFPPCEHHSKKTHFD |
| P34946 | VNNGWIDSTIQEKAYIDFSYNNSYQQIIDSSTRDSLLDAYN |
| Q9H3G5 | SLVGPFPGLNMKSYAGFLTVNKTYNSNLFFWFFPAQIQPED |
| Q9H3G5 | PGGSSMFGLFVEHGPYVVTSNMTLRDRDFPWTTTLSMLYID |
| Q9H3G5 | FEILDKLLDGDLTSDPSYFQNVTGCSNYYNFLRCTEPEDQL |
| Q9H3G5 | QLYYVKFLSLPEVRQAIHVGNQTFNDGTIVEKYLREDTVQS |
| P20023 | KIRGSTPYRHGDSVTFACKTNFSMNGNKSVWCQANNMWGPT |
| P20023 | PYRHGDSVTFACKTNFSMNGNKSVWCQANNMWGPTRLPTCV |
| P20023 | IFCPSPPPILNGRHIGNSLANVSYGSIVTYTCDPDPEEGVN |
| P20023 | PHPQILRGRMVSGQKDRYTYNDTVIFACMFGFTLKGSKQIR |
| P20023 | EATGRQLLTKPQHQFVRPDVNSSCGEGYKLSGSVYQECQGT |
| P20023 | SHVHIANGYKISGKEAPYFYNDTVTFKCYSGFTLKGSSQIR |
| P20023 | CQHVRQSLQELPAGSRVELVNTSCQDGYQLTGHAYQMCQDA |
| P20023 | LRSPPVTRCPNPEVKHGYKLNKTHSAYSHNDIVYVDCNPGF |
| P20023 | HSAYSHNDIVYVDCNPGFIMNGSRVIRCHTDNTWVPGVPTC |
| P20023 | PTCIKKAFIGCPPPPKTPNGNHTGGNIARFSPGMSILYSCD |
| P20023 | LLLCTHEGTWSQPAPHCKEVNCSSPADMDGIQKGLEPRKMY |
| Q68CJ9 | TTREESPGSPGADWGFQDTANLTNSTEELDNATLVLRNATE |
| Q68CJ9 | EESPGSPGADWGFQDTANLTNSTEELDNATLVLRNATEGLG |
| Q68CJ9 | GADWGFQDTANLTNSTEELDNATLVLRNATEGLGQVALLDW |
| Q68CJ9 | DTANLTNSTEELDNATLVLRNATEGLGQVALLDWVAPGPST |
| W1I921 | IIEVPDDIQQQCDILIQRAHNCTSQRTGCSRRVEESYDRRF |
| P02522 | QGHSHELNGPCPNLKETGVEKAGSVLVQAGPWVGYEQANCK |
| P02522 | KAGSVLVQAGPWVGYEQANCKGEQFVFEKGEYPRWDSWTSS |
| P02522 | AGPWVGYEQANCKGEQFVFEKGEYPRWDSWTSSRRTDSLSS |
| P02522 | RWDSWTSSRRTDSLSSLRPIKVDSQEHKITLYENPNFTGKK |
| P02522 | SRRTDSLSSLRPIKVDSQEHKITLYENPNFTGKKMEVIDDD |
| P02522 | IKVDSQEHKITLYENPNFTGKKMEVIDDDVPSFHAHGYQEK |
| P02522 | KVDSQEHKITLYENPNFTGKKMEVIDDDVPSFHAHGYQEKV |

| | |
|---|---|
| P02522 | KKMEVIDDDVPSFHAHGYQEKVSSVRVQSGTWVGYQYPGYR |
| P02522 | SGTWVGYQYPGYRGLQYLLEKGDYKDSGDFGAPQPQVQSVR |
| P02522 | VGYQYPGYRGLQYLLEKGDYKDSGDFGAPQPQVQSVRRIRD |
| P10040 | LIYLATDVASVAVPTKEAYFNGSTYLRLTTPMPIWDHSAIS |
| P10040 | FKYEYGNLYLHVDRAASIFANSTYNSQFLTNQDIGYKDAIL |
| P10040 | LILGNSFSGCLLDGPGLQFVNNSTVQNVVFGHCPLTPGPCS |
| P10040 | ILGNSFSGCLLDGPGLQFVNNSTVQNVVFGHCPLTPGPCSD |
| P10040 | ENGGSCLENSRGDYQCFCDPNHSGQHCETEVNIHPLCQTNP |
| P10040 | YAGARCEVDTDECASQPCQNNGSCIDRINGFSCDCSGTGYT |
| P10040 | NCQTSTGDGASALALTPINCNATNGKCLNGGTCSMNGTHCY |
| P10040 | TPINCNATNGKCLNGGTCSMNGTHCYCAVGYSGDRCEKAEN |
| P10040 | GYEGENCEVDIDECGSNPCSNGSTCIDRINNFTCNCIPGMT |
| P10040 | IDECGSNPCSNGSTCIDRINNFTCNCIPGMTGRICDIDIDD |
| P10040 | DINECESNPCQYNGNCLERSNITLYQMSRITDLPKVFSQPF |
| P10040 | YQMSRITDLPKVFSQPFSFENASGYECVCVPGIIGKNCEIN |
| P10040 | TCYDQIDDYDCDCDANYGGKNCSVLLKGCDQNPCLNGGACL |
| P10040 | PCLNGGACLPYLINEVTHLYNCTCENGFQGDKCEKTTTLSM |
| P10040 | LNLHSSLLNKWEGVFIGSKLNDSNWHKVFVAINTSHLVLSA |
| P10040 | GVFIGSKLNDSNWHKVFVAINTSHLVLSANDEQAIFPVGSY |
| P10040 | LVLSANDEQAIFPVGSYETANNSQPSFPRTYLGGTIPNLKS |
| P10040 | GCMQDIMVNGKWIFPDEQDANISYTKLENVQSGCPRTEQCK |
| P10040 | LWHTFACHCPRPFFGHTCQHNMTAATFGHENTTHSAVIVET |
| P10040 | RPFFGHTCQHNMTAATFGHENTTHSAVIVETTDVARRAIRS |
| P10040 | YTVGGQKLDNGYNHLIEVVRNQTLVQVKLNGTEYFRKTLST |
| P10040 | NGYNHLIEVVRNQTLVQVKLNGTEYFRKTLSTTGLLDAQVL |
| P10040 | KEADDSRDYFKGIIQDVKVSNGSLNLIVEMYSLNVTDVQVN |
| P10040 | IQDVKVSNGSLNLIVEMYSLNVTDVQVNAKPLGAVTIDRAS |
| P10040 | VTCPGQSLCQNLDDGYECVTNTTFTGQERSPLAFFYFQEQQ |
| P10040 | ARVGDLLLPYFSMAELYSRTNVSVQQKAQFRLNATRPEEGC |
| P10040 | MAELYSRTNVSVQQKAQFRLNATRPEEGCILCFQSDCKNDG |
| P10040 | FEGDDCGTDIDECLNTECLNNGTCINQVAAFFCQCQPGFEG |
| P10040 | GQHCEQNIDECADQPCHNGGNCTDLIASYVCDCPEDYMGPQ |
| P10040 | YMGPQCDVLKQMTCENEPCRNGSTCQNGFNASTGNNFTCTC |
| P10040 | KQMTCENEPCRNGSTCQNGFNASTGNNFTCTCVPGFEGPLC |
| P10040 | NEPCRNGSTCQNGFNASTGNNFTCTCVPGFEGPLCDIPFCE |
| P10040 | FNKPGSFQCICQKPYCGAYCNFTDPCNATDLCSNGGRCVES |
| P10040 | FQCICQKPYCGAYCNFTDPCNATDLCSNGGRCVESCGAKPD |
| P10040 | ESCGAKPDYYCECPEGFAGKNCTAPITAKEDGPSTTDIAII |
| Q6WN34 | KCVEPHTPSGLRAPPKSCQHNGTMYQHGEIFSAHELFPSRL |
| Q8BGC9 | EKIQGLPFGSCLAISDGPVHNSTGIPFFYMTAKDPAVADLV |
| O75629 | FDPQSPLCVHIMLSGTVTKVNETEMDIAKHSLFIRHPEMKT |

| | |
|---|---|
| O75629 | IRHPEMKTWPSSHNWFFAKLNITNIWVLDYFGGPKIVTPEE |
| P35353 | LNPVSTSLQDQRCENLSLTSNVSGLQCNASVDLIGTCWPRS |
| P35353 | LQDQRCENLSLTSNVSGLQCNASVDLIGTCWPRSPAGQLVV |
| P35353 | SPAGQLVVRPCPAFFYGVRYNTTNNGYRECLANGSWAARVN |
| P35353 | AFFYGVRYNTTNNGYRECLANGSWAARVNYSECQEILNEEK |
| P35353 | NTTNNGYRECLANGSWAARVNYSECQEILNEEKKSKVHYHV |
| P47866 | ELLLDGWGEPPDPEGPYSYCNTTLDQIGTCWPQSAPGALVE |
| P47866 | SAPGALVERPCPEYFNGIKYNTTRNAYRECLENGTWASRVN |
| P47866 | EYFNGIKYNTTRNAYRECLENGTWASRVNYSHCEPILDDKQ |
| P47866 | NTTRNAYRECLENGTWASRVNYSHCEPILDDKQRKYDLHYR |
| Q8CII9 | CHDLETVEVTWGSGPDHHGANLSLEFRYGTGALQPCPRYFL |
| Q8CII9 | AMVFKARQRASAWLKPRPPWNVTLLWTPDGDVTVSWPAHSY |
| P06205 | TWSSWEGEATIAVDGFHCKGNATGIAVGRTLSQGGLVVLGQ |
| P06206 | TWSSWEGEATIGVDGFHCKGNATGIAMGVTLSQGGLVVLGQ |
| P06207 | TWSSWEGEATIAVDGFHCKGNATGIAVGRTLSQGGLVVLGQ |
| P48199 | WLDGKPRVRKSLQKGYIVGTNASIILGQEQDSYGGGFDANQ |
| Q27367 | DAYESWLEAPIPIYLSFYMFNWTNPEDIRNPDIKPNFVEMG |
| Q27367 | IKPNFVEMGPYTFLEKHKKENYTFYDNATVAYYERRTWFFD |
| Q27367 | EMGPYTFLEKHKKENYTFYDNATVAYYERRTWFFDPERSNG |
| Q27367 | DNATVAYYERRTWFFDPERSNGTLDDMVTAAHAITATVADE |
| Q27367 | GGKLYVTKPVGEWIFEGYQDNITDFLNLFNTTKIDIPYKRF |
| Q27367 | VGEWIFEGYQDNITDFLNLFNTTKIDIPYKRFGWLADRNES |
| Q27367 | LFNTTKIDIPYKRFGWLADRNESLTYDGLFTIHTGTDDISN |
| Q27367 | LTHWNGKSETGFYEMPCGIVNGTTGDMFPPKMNVNDEITIF |
| Q9BUF7 | FLLARWGRAWGQIQTTSANENSTVLPSSTSSSSDGNLRPEA |
| P02470 | DSGISEVRSDRDKFVIFLDVKHFSPEDLTVKVQEDFVEIHG |
| P07333 | PSVPELVVKPGATVTLRCVGNGSVEWDGPPSPHWTLYSDGS |
| P07333 | PPSPHWTLYSDGSSSILSTNNATFQNTGTYRCTEPGDPLGG |
| P07333 | VLEAGVSLVRVRGRPLMRHTNYSFSPWHGFTIHRAKFIQSQ |
| P07333 | AQIVCSASSVDVNFDVFLQHNNTKLAIPQQSDFHNNRYQKV |
| P07333 | NRYQKVLTLNLDQVDFQHAGNYSCVASNVQGKHSTSMFFRV |
| P07333 | NVQGKHSTSMFFRVVESAYLNLSSEQNLIQEVTVGEGLNLK |
| P07333 | VGEGLNLKVMVEAYPGLQGFNWTYLGPFSDHQPEPKLANAT |
| P07333 | GFNWTYLGPFSDHQPEPKLANATTKDTYRHTFTLSLPRLKP |
| P07333 | LTFELTLRYPPEVSVIWTFINGSGTLLCAASGYPQPNVTWL |
| P07333 | WTFINGSGTLLCAASGYPQPNVTWLQCSGHTDRCDEAQVLQ |
| P07333 | QEPFHKVTVQSLLTVETLEHNQTYECRAHNSVGSGSWAFIP |
| P09581 | PSGPELVVEPGETVTLRCVSNGSVEWDGPISPYWTLDPESP |
| P09581 | PISPYWTLDPESPGSTLTTRNATFKNTGTYRCTELEDPMAG |
| P09581 | NDVGTRTATMNFQVVESAYLNLTSEQSLLQEVSVGDSLILT |
| P09581 | VGDSLILTVHADAYPSIQHYNWTYLGPFFEDQRKLEFITQR |

| | |
|---|---|
| P09581 | VKASEAGQYFLMAQNKAGWNNLTFELTLRYPPEVSVTWMPV |
| P09581 | LTFELTLRYPPEVSVTWMPVNGSDVLFCDVSGYPQPSVTWM |
| P09581 | WMECRGHTDRCDEAQALQVWNDTHPEVLSQKPFDKVIIQSQ |
| P09581 | QKPFDKVIIQSQLPIGTLKHNMTYFCKTHNSVGNSSQYFRA |
| P09581 | PIGTLKHNMTYFCKTHNSVGNSSQYFRAVSLGQSKQLPDES |
| P09603 | CVRTFYETPLQLLEKVKNVFNETKNLLDKDWNIFSKNCNNS |
| P09603 | VFNETKNLLDKDWNIFSKNCNNSFAECSSQDVVTKPDCNCL |
| P15509 | DLRTVAPASSLNVRFDSRTMNLSWDCQENTTFSKCFLTDKK |
| P15509 | SSLNVRFDSRTMNLSWDCQENTTFSKCFLTDKKNRVVEPRL |
| P15509 | CSCTFREICLHEGVTFEVHVNTSQRGFQQKLLYPNSGREGT |
| P15509 | RGFQQKLLYPNSGREGTAAQNFSCFIYNADLMNCTWARGPT |
| P15509 | GREGTAAQNFSCFIYNADLMNCTWARGPTAPRDVQYFLYIR |
| P15509 | EIRCPYYIQDSGTHVGCHLDNLSGLTSRNYFLVNGTSREIG |
| P15509 | HVGCHLDNLSGLTSRNYFLVNGTSREIGIQFFDSLLDTKKI |
| P15509 | IQFFDSLLDTKKIERFNPPSNVTVRCNTTHCLVRWKQPRTY |
| P15509 | LLDTKKIERFNPPSNVTVRCNTTHCLVRWKQPRTYQKLSYL |
| P15509 | QYQLDVHRKNTQPGTENLLINVSGDLENRYNFPSSEPRAKH |
| P15509 | SSEPRAKHSVKIRAADVRILNWSSWSEAIEFGSDDGNLGSV |
| P04141 | SPSTQPWEHVNAIQEARRLLNLSRDTAAEMNETVEVISEMF |
| P04141 | NAIQEARRLLNLSRDTAAEMNETVEVISEMFDLQEPTCLQT |
| P01587 | FKKLTCVQTRLKIFEQGLRGNFTKLKGALNMTASYYQTYCP |
| P01587 | RLKIFEQGLRGNFTKLKGALNMTASYYQTYCPPTPETDCET |
| Q99062 | VSAPIVHLGDPITASCIIKQNCSHLDPEPQILWRLGAELQP |
| Q99062 | GRQQRLSDGTQESIITLPHLNHTQAFLSCCLNWGNSLQILD |
| Q99062 | SLQILDQVELRAGYPPAIPHNLSCLMNLTTSSLICQWEPGP |
| Q99062 | QVELRAGYPPAIPHNLSCLMNLTTSSLICQWEPGPETHLPT |
| Q99062 | QGYVVSWRPSGQAGAILPLCNTTELSCTFHLPSEAQEVALV |
| Q99062 | PWPQGYVIEWGLGPPSASNSNKTWRMEQNGRATGFLLKENI |
| Q99062 | LTHYTIFWTNAQNQSFSAILNASSRGFVLHGLEPASLYHIH |
| Q99062 | LEPASLYHIHLMAASQAGATNSTVLTLMTLTPEGSELHIIL |
| P40223 | ISPPVVRLGDPVLASCTISPNCSKLDQQAKILWRLQDEPIQ |
| P40223 | DRQHHLPDGTQESLITLPHLNYTQAFLFCLVPWEDSVQLLD |
| P40223 | SVQLLDQAELHAGYPPASPSNLSCLMHLTTNSLVCQWEPGP |
| P40223 | ADCQYQGDTIPDCVAKKRQNNCSIPRKNLLLYQYMAIWVQA |
| P40223 | PSEYMEQECELRYQPQLKGANWTLVFHLPSSKDQFELCGLH |
| P40223 | QGYLLSWSSPDHQGQDIHLCNTTQLSCIFLLPSEAQNVTLV |
| P40223 | IHLCNTTQLSCIFLLPSEAQNVTLVAYNKAGTSSPTTVVFL |
| P40223 | APSLLPQGYLIEWEMSSPSYNNSYKSWMIEPNGNITGILLK |
| P40223 | EMSSPSYNNSYKSWMIEPNGNITGILLKDNINPFQLYRITV |
| P40223 | LTHYTIFWADAGDHSFSVTLNISLHDFVLKHLEPASLYHVY |
| P40223 | LEPASLYHVYLMATSRAGSTNSTGLTLRTLDPSDLNIFLGI |

| | |
|---|---|
| B0R8E4 | LMVGSVIGAGVAFTGGAAAANASDLNDYQRFNENTNYTYST |
| B0R8E4 | VDTGLVVDNDSTTEIVDDYENTSISDVDYAYAIVEIDDGNG |
| B0R8E4 | YPASDTEDAPDYVNSNEELTNGSALDGVSTDDDTDFDVTQG |
| B0R8E4 | GDITLDNPTGAYVVGSEVDINGTANEGTDDVVLYARDNNDF |
| B0R8E4 | DILGLPGTYRLGIIAKSDAVNSSGGVKDNIDTSDFNQGVSS |
| B0R8E4 | DGKFGEDTANSISDLEDEVGNYTSGSPTGDQIRDRILSNTV |
| B0R8E4 | KADDNTITVELLQGDASIEINSTDEWNSDGQWSVDVPLSNV |
| B0R8E4 | EWNSDGQWSVDVPLSNVEPGNYTVEADDGDNTDRQNVEIVE |
| B0R8E4 | VEIVEELEEPDQTTVDQPENNQTMTTTMTETTTETTTEMTT |
| B0R8E4 | TTTMTETTTETTTEMTTTQENTTENGSEGTSDGESGGSIPG |
| B0R8E4 | TETTTETTTEMTTTQENTTENGSEGTSDGESGGSIPGFGVG |
| Q5V7F4 | ITVEGTEDLTFGDASETVTVNITSDQQASLNLDNDEVVQGE |
| Q5V7F4 | GEITLDSPSGAYITGSQIDVNGTANQGVDQVALYARDNNDY |
| Q5V7F4 | SDFNSGVSGATALRVTDTALNGTFTTYNGQIASDDGQIDVD |
| Q5V7F4 | QFRLTDGLTTIESVSSPVEANGTLEVQGNTNRVPDDNTITV |
| Q5V7F4 | GSDGQWSVNVDLSDVDIEPGNYTVEADDGDNTDRTSVTVVE |
| P25062 | AFTGSAAAERGNLDADSESFNKTIQSGDRVFLGEEISTDAG |
| P25062 | TENQPLGTYDVDGSGSATTPNVTLLAPRITDSEILTSSGGD |
| P25062 | TEQAKEVFRNIGDTSEVGIANSSATNTSGSSTGPTVETADI |
| P25062 | EVFRNIGDTSEVGIANSSATNTSGSSTGPTVETADIAYAVV |
| P25062 | GQFTTINGQVAPVETGTVDINGTASGANSVLVIFVDERGNV |
| P25062 | TDTVNVEVVSEREDTTTSSDNATDTTTTTDGPTETTTTAEP |
| Q50833 | KLTVKASAESDDANLKSLLTNGTNDFTELDAGKEAFVVAAA |
| Q50833 | AGKEAFVVAAADSDYSDALINATTGFANIADNVLYDQAKLA |
| P27373 | LLLIVISMSGIAGAAEVKNLNTSKTFTKIQEAIDDPSTTDG |
| P27373 | QEAIDDPSTTDGNIIIVGPGNYTENILVNKSLTLKSNGSAI |
| P27373 | TTDGNIIIVGPGNYTENILVNKSLTLKSNGSAIINAVSSEK |
| P27373 | VGPGNYTENILVNKSLTLKSNGSAIINAVSSEKSTITIKAN |
| P27373 | NNVWIEGFIIIGGKNGIYMENVTGCTITNNTIQNAFVSGWE |
| P27373 | IIIGGKNGIYMENVTGCTITNNTIQNAFVSGWEYYGGNGIC |
| P27373 | IQNAFVSGWEYYGGNGICLVNSTNNTITNNIIRNNTWNGIN |
| P27373 | AFVSGWEYYGGNGICLVNSTNNTITNNIIRNNTWNGINVCE |
| P27373 | GNGICLVNSTNNTITNNIIRNNTWNGINVCESKGNIIKNNT |
| P27373 | IRNNTWNGINVCESKGNIIKNNTIMYSGGIGIYVWGFNKFE |
| P27373 | YVWGFNKFEGNNIIENNRIINATYGGIYLFRPSNNKICRNY |
| P27373 | YGGIYLFRPSNNKICRNYIANVSSGGGGMSGAICIDVSDYN |
| P27373 | FINYETAISDPKGELVDNIWNTTEGGNYWSNYTGNNTGDGT |
| P27373 | PKGELVDNIWNTTEGGNYWSNYTGNNTGDGTGNIPYYYDNK |
| P27373 | LVDNIWNTTEGGNYWSNYTGNNTGDGTGNIPYYYDNKPLVV |
| P27373 | DPLSAGESQIVRWDKIVPEGNHTIKAEIPYSAEGYLIGTNI |
| P27373 | IRDADISNNVFSKIVQGFVQNKTFTITLTNLGKSTITIKYY |

| | |
|---|---|
| P27373 | NLGKSTITIKYYISIYTNPVNGTKVSYRELTITLKPNETKT |
| P27373 | TNPVNGTKVSYRELTITLKPNETKTIELGKYPFKYAVSGTM |
| P13611 | SGKVSLPCHFSTMPTLPPSYNTSEFLRIKWSKIEVDKNGKD |
| P13611 | VARAQCGGGLLGVRTLYRFENQTGFPPPDSRFDAYCFKPKE |
| P13611 | EDLESVSASTTVSPLIMPDNNGSSMDDWEERQTSGRITEEF |
| P13611 | EVRDMEEDFTATPGTTKYDENITTVLLAHGTLSVEAATVSK |
| P13611 | AHGTLSVEAATVSKWSWDEDNTTSKPLESTEPSASSKLPPA |
| P13611 | PPASTKFHPDINVYIIEVRENKTGRMSDLSVIGHPIDSESK |
| P13611 | IIEIDLYHSEENEEEEEECANATDVTTTPSVQYINGKHLVT |
| P13611 | KDPEAAEARRGQFESVAPSQNFSDSSESDTHPFVIAKTELS |
| P13611 | ESDTHPFVIAKTELSTAVQPNESTETTESLEVTWKPETYPE |
| P13611 | GEAEEDEDTMFTMVTDLSQRNTTDTLITLDTSRIITESFFE |
| P13611 | FSTEPTGLVLSTVMDRVVAENITQTSREIVISERLGEPNYG |
| P13611 | TTKKTYSDDKEMKEEDTSLVNMSTPDPDANGLESYTTLPEA |
| P13611 | LFSGLGSGEEVLPTLPTESVNFTEVEQINNTLYPHTSQVES |
| P13611 | EEVLPTLPTESVNFTEVEQINNTLYPHTSQVESTSSDKIED |
| P13611 | GAEGPTVAPLPFSTDIGHPQNQTVRWAEEIQTSRPQTITEQ |
| P13611 | WAEEIQTSRPQTITEQDSNKNSSTAEINETTTSSTDFLARA |
| P13611 | SRPQTITEQDSNKNSSTAEINETTTSSTDFLARAYGFEMAK |
| P13611 | AVTADGFPTVSVMLPLHSEQNKSSPDPTSTLSNTVSYERST |
| P13611 | SNDESNDDSTQVQEIYEAAVNLSLTEETFEGSADVLASYTQ |
| P13611 | EASPTELIAVEGTEILQDFQNKTDGQVSGEAIKMFPTIKTP |
| P13611 | NPVEFNTEVATPPFSLLETSNETDFLIGINEESVEGTAIYL |
| P13611 | KITCMNPSAYQRTYSMKYFKNSSSAKDNSINTSKHDHRWSR |
| Q6UVK1 | HTVVLTVVEGWATLSVDGFLNASSAVPGAPLEVPYGLFVGG |
| Q6UVK1 | DAEASRHLQEHRLGLTPEATNASLLGCMEDLSVNGQRRGLR |
| Q6UVK1 | PAMELPEPCVPEPGLPPVFANFTQLLTISPLVVAEGGTAWL |
| Q6UVK1 | IHRSTGLRLAQGSAMPILPANLSVETNAVGQDVSVLFRVTG |
| Q6UVK1 | AYDTVENLALEVQVGQEILSNLSFPVTIQRATVWMLRLEPL |
| Q6UVK1 | QHQATALLEVQASEPYLRVANGSSLVVPQGGQGTIDTAVLH |
| Q6UVK1 | QPATAFSQQDLLDGAVLYSHNGSLSPRDTMAFSVEAGPVHT |
| Q6UVK1 | LEGVLVELEVLPAAIPLEAQNFSVPEGGSLTLAPPLLRVSG |
| Q6UVK1 | LIRYVHDGSETLTDSFVLMANASEMDRQSHPVAFTVTVLPV |
| Q6UVK1 | GPQLGRLFHAQQDSTGEALVNFTQAEVYAGNILYEHEMPPE |
| Q6UVK1 | GPVTRFTQADVDSGRLAFVANGSSVAGIFQLSMSDGASPPL |
| Q6UVK1 | PTSAFSQFQIDQGEVVFAFTNFSSSHDHFRVLALARGVNAS |
| Q6UVK1 | FTNFSSSHDHFRVLALARGVNASAVVNVTVRALLHVWAGGP |
| Q6UVK1 | SHDHFRVLALARGVNASAVVNVTVRALLHVWAGGPWPQGAT |
| Q6UVK1 | WPQGATLRLDPTVLDAGELANRTGSVPRFRLLEGPRHGRVV |
| Q00657 | HTVVLTVSNSWAVLSVDGVLNTSAPIPKASHLKVPYGLFVG |
| Q00657 | DTEASRHLQEHRLGLTPGAANISLVGCIEDFSVNGRRLGLR |

| | |
|---|---|
| Q00657 | PVMDLPEPCVPEPGLPAVFANFTQLLTISPLVVAEGGTAWL |
| Q00657 | ILHNTGLRLAQGSAAAILPANLSVETNAVGQDVSVLFRVTG |
| Q00657 | TQDTVEDLTLEVQVGQETLSNLSFPVTIQRATVWMLQLEPL |
| Q00657 | QHQATAMLEVQASEPYLHVANSSSLVVPQGGQGTIDTAVLH |
| Q00657 | QSVTSFSQRDLLDGAILYSHNGSLSPQDTLALSVAAGPVHT |
| Q00657 | LEGISVELEVLPTVIPLDVQNFSVPEGGTRTLAPPLIQITG |
| Q00657 | LIRYVHDGSETQTDGFILLANASEMDRQSQPMAFTITILPV |
| Q00657 | GPQLGRLLHAQQGSAEEALVNFTQAEVNAGNILYEHEISSE |
| Q00657 | GPVTHFTQADVDAGRLAFVANGSSVAGVFQLSMSDGASPPI |
| Q00657 | PASAFSQLQVDQGDVVFAFTNFSSSQDHFKVLALARGVNAS |
| Q00657 | FTNFSSSQDHFKVLALARGVNASATVNVTVQALLHVWAGGP |
| Q00657 | SQDHFKVLALARGVNASATVNVTVQALLHVWAGGPWPQGTT |
| Q00657 | WPQGTTLRLDPTVLDASELANRTGSMPRFRLLEGPRYGRVV |
| Q8VHY0 | HTVVLTVSDSWAVLSVDGVLNTSAPIPRASHLKATYGLFVG |
| Q8VHY0 | DTEASRHLQEHRLGLAPGAANISLVGCIEDFSVNGRRQGLR |
| Q8VHY0 | PAMELPEPCIPEPGLPAVFANFTQLLTISPLVVAEGGTAWL |
| Q8VHY0 | ILHNTGLHLAQGSAAAILPANLSVETNAVGQDVSVLFRVTG |
| Q8VHY0 | TQDTVEDLILEVQVGQETLSNLSFPVTIQRATVWMLRLEPL |
| Q8VHY0 | QHQATAMLEVQASEPYLHVANSSSLVVPQGGQGTIDTAVLQ |
| Q8VHY0 | QSVTSFSQRDLLDGAILYSHNGSLSPQDTLAFSVAAGPVHT |
| Q8VHY0 | LEGISVELEVLPTVIPLDVQNFSVPEGGTRTLAPPLVQITG |
| Q8VHY0 | LIRYVHDGSETQTDAFVLLANASEMDRQSQPVAFTITILPV |
| Q8VHY0 | GPQLGRLLHAQQGSAEEVLVNFTQAEVNAGNILYEHEMSSE |
| Q8VHY0 | GPVTHFTQADVDAGRLAFVANGSSVAGVFQLSMSDGASPPI |
| Q8VHY0 | PASAFSQLQVDQGDVVFVFTNFSSSQDHFKVVALARGVNAS |
| Q8VHY0 | FTNFSSSQDHFKVVALARGVNASATVNVTVQALLHVWAGGP |
| Q8VHY0 | SQDHFKVVALARGVNASATVNVTVQALLHVWAGGPWPQGTT |
| Q8VHY0 | WPQGTTLRLDPTVLDASELANRTGSMPHFRLLAGPRYGRVV |
| Q71M36 | GSAIEAEELVRSSLAWESRANDTREEAGLPAAGEDETSWTE |
| Q71M36 | SSISLRPRPGDPGKDLASGENGTECRVGFVRHNGSCRSVCD |
| Q71M36 | GKDLASGENGTECRVGFVRHNGSCRSVCDLFPSYCHNGGQC |
| O95196 | GSAVEAEELVKGSPAWEPPANDTREEAGPPAAGEDEASWTA |
| O60676 | LSLILLTIPLALVARKDPKKNETGVLRKLKPVNASNANVKQ |
| O60676 | VARKDPKKNETGVLRKLKPVNASNANVKQCLWFAMQEYNKE |
| Q9V498 | EYLTQKEIILEKSYHGLIRENETLVEITPLIKVNEEKICNF |
| Q9V498 | HSSIALKTKHISFGCDRDISNCTSGQKVKDLLPHGAEWTKE |
| Q9V498 | NRHSNPEVIDDWPLHAAHGVNTSLAIGACYQSLENRLKHGF |
| Q9V498 | KQKPTVGRRNIEVLTTLNCKNESSLRLPPIETYIMVNEPIA |
| Q9V498 | KSTNVLPSSLSKVNFIGNTDNETSFHRNSGSVNGNDNNQPT |
| Q8IWA5 | PLARRCFPAIHAYKGVLMVGNETTYEDGHGSRKNITDLVEG |
| Q8IWA5 | KGVLMVGNETTYEDGHGSRKNITDLVEGAKKANGVLEARQL |

| | |
|---|---|
| Q8IWA5 | DDSPCPFTAKTCNPETFPSSNESRQCPNARCQFAFYGGESG |
| Q91VA1 | IIVGLVAWVYGDPRQVLYPRNSTGAYCGVGDNKDKPYVLYF |
| Q91VA1 | PRFLLPSTPALGRCFPLPNINFTLPEDLRINNTTVSNGISG |
| Q91VA1 | LGRCFPLPNINFTLPEDLRINNTTVSNGISGLLDSINARDV |
| Q91VA1 | GRCFPLPNINFTLPEDLRINNTTVSNGISGLLDSINARDVS |
| Q91VA1 | LPNINFTLPEDLRINNTTVSNGISGLLDSINARDVSVKIFE |
| Q91VA1 | QQYQVFRDKGASITQLGFTTNFSAYQSVKETWLAALIVLAV |
| Q91VA1 | AVTALYLATSGQPQYIYWASNTSTPGCENVPVNMTCDPMAP |
| Q91VA1 | PQYIYWASNTSTPGCENVPVNMTCDPMAPLNSSCPNLKCVF |
| Q91VA1 | STPGCENVPVNMTCDPMAPLNSSCPNLKCVFKGYSTTGLAQ |
| P16410 | NELTFLDDSICTGTSSGNQVNLTIQGLRAMDTGLYICKVEL |
| P16410 | GLYICKVELMYPPPYYLGIGNGTQIYVIDPEPCPDSDFLLW |
| Q8BY89 | PLAQRCFPAIHASKGVLMVGNETTYEDGHGARKNITDLVEG |
| Q8BY89 | KGVLMVGNETTYEDGHGARKNITDLVEGAKKANKILEARQL |
| Q8BY89 | DDTACPLLRKTCNPETFPLRNESLQCPTARCQFAFYGGEST |
| Q9VCR7 | NAQSSQLTVDSHDITVLLNSNETFLVFANGLLDSDVEVALG |
| Q9VCR7 | SEDHLLLDPATFVYPAGSTRNQSVVITGLKAGNVKVVADSD |
| P57757 | FIEKYESTVSLTAPPTVKLENGSSTNVDITLGHPLNSTLVI |
| P57757 | TVKLENGSSTNVDITLGHPLNSTLVITFEVTFRSKNLTIVE |
| P57757 | LGHPLNSTLVITFEVTFRSKNLTIVELPDEVIVPRGEKNAS |
| P57757 | SKNLTIVELPDEVIVPRGEKNASFQVTSQNIGQVTVFLHGN |
| P57757 | NASFQVTSQNIGQVTVFLHGNHSNQTCPRIRFLVIHSRIVS |
| P57757 | FQVTSQNIGQVTVFLHGNHSNQTCPRIRFLVIHSRIVSIIN |
| P30825 | INVLVLGFIMVSGFVKGSVKNWQLTEEDFGNTSGRLCLNND |
| P30825 | VSGFVKGSVKNWQLTEEDFGNTSGRLCLNNDTKEGKPGVGG |
| P30825 | KNWQLTEEDFGNTSGRLCLNNDTKEGKPGVGGFMPFGFSGV |
| B2DCR8 | AAPPEIHTTRPNVPEEIKRPNSTEIETPAVKQLETPSIFLL |
| B2DCR8 | KYPWYSWSIVTVKKMLANQRNSTLGNQFYEMEAVGPHGSNF |
| B2DCR8 | ILVKYISDLQKLDYILKISFNYSKKKITVDAFSRRMKAFLS |
| B2DCR8 | SKRRMRSYARYWERTSCPPLNVTHLTQTGCGALLSFEGMKV |
| B2DCR8 | KCESSWSRWSKWSACASTCGNATQSRRRRCLGQSESEKCIG |
| Q20911 | TRSRILMVDGNVWLHAGKDKNITFKTTGNGRIYVDETDVSK |
| Q20911 | TDVSKLPDIASFKQLVGRIENTSQMFTTLKSRQDTQTSAMR |
| Q20911 | QTSAMRVVLNSAKHYLVALRNMTLEVNILKKWKTDKMARDM |
| Q20911 | CPPHFTGTTCEADIDECSVYNGTTAGCQNNGTCINNRGGFE |
| Q20911 | CEADIDECSVYNGTTAGCQNNGTCINNRGGFECQCQSGYHG |
| Q20911 | GRACEEEEEHCGSHFTHSSGNYTFDLQRSNKTELSICDFVF |
| Q20911 | HCGSHFTHSSGNYTFDLQRSNKTELSICDFVFNIPAANSAV |
| Q20911 | EFDKFTQEGSGPTDCAKTDANLTLYDGPEDSSSEFATFCGD |
| Q20911 | GFVLKYKFTTPDRECGAEIDNDSNDFSFSGVIESPNYGSLY |
| Q20911 | IESPNYGSLYPPNMDCTWKINGTLSNGSYSGDMVLKLTFDE |

| | |
|---|---|
| Q20911 | YGSLYPPNMDCTWKINGTLSNGSYSGDMVLKLTFDEYDVKS |
| Q20911 | EQGKGFRIQYEMLCEKRVNGNGTIQTWNFPNGGAAGTCTYI |
| Q20911 | EVTPAKSCEYDRVEIYTSYHNETVHGELLGKFCGAMIPPSI |
| Q20911 | DKCTYHIAVHENQRILIKMNNMSLPCDKSSLMFRNGPSETS |
| Q20911 | SFSNRVTIVYKSINSEGSFFNLTYETITSGCGGRVDGLTGI |
| Q20911 | TVAVALGNKVRFALTALDDLNSSDSGGFCPLFAANRIDFFD |
| Q20911 | LKVDDTDLAEAEVTFKKTDYNITNSVNKYCDKAIPRVIRSR |
| Q20911 | PIGKQINLKIDKMDILPIGTNCTYTKDFSGFAIFMSDSNKS |
| Q20911 | GTNCTYTKDFSGFAIFMSDSNKSGTPFQTYCSSINQQNISS |
| Q20911 | SDSNKSGTPFQTYCSSINQQNISSHTNELFLFLSMKKDKLK |
| Q20911 | NELFLFLSMKKDKLKTNVFFNATVEFVDVPQSSQSDICGQV |
| Q20911 | PYYHDDRKTDKPRFMSPYTANYTCKWYLAYNVGMLSFYEGN |
| Q20911 | NYTCKWYLAYNVGMLSFYEGNSSSSSPVERLCDESDTVQTF |
| Q20911 | VYAESKPQTISLYHEGEGICNVTIKKKDPQDSEINIRLEEY |
| Q20911 | KKKDPQDSEINIRLEEYTKLNSSSTHTIDDRIDIYVGGVLK |
| Q20911 | NSNGNTVTLSILDHNLKSTPNCTDSYIEIRANNSSGKLIKR |
| Q20911 | LDHNLKSTPNCTDSYIEIRANNSSGKLIKRQCDISSIDSTE |
| Q20911 | DHNLKSTPNCTDSYIEIRANNSSGKLIKRQCDISSIDSTEY |
| Q20911 | KLEKSDKFSFTWDPVPLNYRNLTLAKDKPAKVYDCGGDLTP |
| Q20911 | EENDDVDLSSVSKHCALVRSNNTFNFSVNRALHIHFVTDRS |
| Q20911 | DVDLSSVSKHCALVRSNNTFNFSVNRALHIHFVTDRSRHGI |
| Q20911 | HSQYSKSSKYCGKLEQGERLNFTSARGRLFIKYNSGPNSRK |
| Q20911 | RRFVATYGIATCGGTVMVRENVTTHITSPSFPYPFATPVQC |
| Q20911 | SPNTHMIEAKVDHVWLFYNPNCTMEQLMIRDGNSTANPLIG |
| Q20911 | HVWLFYNPNCTMEQLMIRDGNSTANPLIGPVCVPRHAPDVF |
| Q20911 | HAPDVFTRSASNQITVQFTSNSTTTRGGRQYCSNKKCGFDV |
| Q20911 | AVVEGFPPYDGAQYSTYFCKNETQITSITDTIRIVYDDSHT |
| Q20911 | VYDDSHTREMISAIGNDQPLNSTFYAPFTIDYMAIPANSEN |
| Q20911 | DYMAIPANSENQGCTLQINKNLTTEFHLRNKVNGKGVGLDS |
| Q20911 | NPQIDLFISQLFRDQEGQQFNLTIEFQKCGGVISSPNTGDI |
| Q20911 | PLCGIEHPKDFESESNRVRLNFTTDSQTTARGFRVNWEAEC |
| Q20911 | TTVTSPAFPLPYTKDLDCVWNITTDPNRQLNIRFEEMKLEA |
| Q20911 | AFHDCSADYIEFFDSSDIMANKTLGKFCGTMDKIPQYRIVT |
| Q20911 | SHRHAFITFETDRDGTGRGFNISYSIVDSDCGGWLKATNEI |
| Q20911 | HVSNTNTIQIIVSADIMPNKNQSRPSLKFEYNILDSCNRTI |
| Q20911 | PNKNQSRPSLKFEYNILDSCNRTIDTNTIKSGRLTSPNYPQ |
| Q20911 | TNTIKSGRLTSPNYPQVYSENSTCVTNLQSSNQKMLLVFND |
| Q20911 | PNNVNKNCDYDYLMLKEGDSNGTYYCGSTLPKALMTSGKDL |
| Q20911 | FADSYELEGVISSIGYPNGYNKSYTQIFTLRPPNSHDCSII |
| O60494 | IQKNKEDIIELKGSAIGLPQNISSQIYQLNSKLVDLERKFQ |
| O60494 | QCIDTVSGYFCKCDSGWTGVNCTENINECLSNPCLNGGTCV |

| | |
|---|---|
| O60494 | WTGALCQVPQQVCGESLSGINGSFSYRSPDVGYVHDVNCFW |
| O60494 | SDQGFHITYLTSPSDLRCGGNYTDPEGELFLPELSGPFTHT |
| O60494 | THTRQCVYMMKQPQGEQIQINFTHVELQCQSDSSQNYIEVR |
| O60494 | SSQNYIEVRDGETLLGKVCGNGTISHIKSITNSVWIRFKID |
| O60494 | PGERTCRWTIHQPQSQVILLNFTVFEIGSSAHCETDYVEIG |
| O60494 | TESTGTIQSPGHPNVYPHGINCTWHILVQPNHLIHLMFETF |
| O60494 | VQPNHLIHLMFETFHLEFHYNCTNDYLEVYDTDSETSLGRY |
| O60494 | WECIYRITVRTGQLIAVHFTNFSLEEAIGNYYTDFLEIRDG |
| O60494 | IDTRSGFSAYWDGSSTGCGGNLTTSSGTFISPNYPMPYYHS |
| O60494 | SSHGSAFELEFKDFHLEHHPNCTLDYLAVYDGPSSNSHLLT |
| O60494 | QQGRGFKAEYRQTCENVVIVNQTYGILESIGYPNPYSENQH |
| O60494 | TYGILESIGYPNPYSENQHCNWTIRATTGNTVNYTFLAFDL |
| O60494 | PYSENQHCNWTIRATTGNTVNYTFLAFDLEHHINCSTDYLE |
| O60494 | ATTGNTVNYTFLAFDLEHHINCSTDYLELYDGPRQMGRYCG |
| O60494 | TGNELAIRFKTDLSINGRGFNASWQAVTGGCGGIFQAPSGE |
| O60494 | RSNTDCSWVIRVDRNHRVLLNFTDFDLEPQDSCIMAYDGLS |
| O60494 | TSSFDTVSSPRFPANYPNNQNCSWIIQAQPPLNHITLSFTH |
| O60494 | SFQLEDSQDCSRDFVEIREGNATGHLVGRYCGNSFPLNYSS |
| O60494 | REGNATGHLVGRYCGNSFPLNYSSIVGHTLWVRFISDGSGS |
| O60494 | SPFWPENYPHNSNYQWTVNVNASHVVHGRILEMDIEEIQNC |
| O60494 | TGEYMFIRFTSDSSVTRAGFNASFHKSCGGYLHADRGIITS |
| O60494 | HADRGIITSPKYPETYPSNLNCSWHVLVQSGLTIAVHFEQP |
| O60494 | PPETRIQLQFEDRFDIEVTPNCTSNYLELRDGVDSDAPILS |
| O60494 | HLQGLSGHYLTISFEDFNLQNSSGCEKDFVEIWDNHTSGNI |
| O60494 | EDFNLQNSSGCEKDFVEIWDNHTSGNILGRYCGNTIPDSID |
| O60494 | IVFNGIRSNSPQLEKLCSSVNVSNEIKSSGNTMKVIFFTDG |
| O60494 | SYTSSEDAVCGGSLPNTPEGNFTSPGYDGVRNYSRNLNCEW |
| O60494 | GSLPNTPEGNFTSPGYDGVRNYSRNLNCEWTLSNPNQGNSS |
| O60494 | VRNYSRNLNCEWTLSNPNQGNSSISIHFEDFYLESHQDCQF |
| O60494 | GFYATWNTQTLGCGGIFHSDNGTIRSPHWPQNFPENSRCSW |
| O60494 | QSQEAPAQGFSASFVSRCGSNFTGPSGYIISPNYPKQYDNN |
| O60494 | TGPSGYIISPNYPKQYDNNMNCTYVIEANPLSVVLLTFVSF |
| O60494 | QITDFGFKFSYRIISCGGVFNFSSGIITSPAYSYADYPNDM |
| O60494 | FDVVPSTSCSHDYLAIYDGANTSDPLLGKFCGSKRPPNVKS |
| O60494 | SDPLLGKFCGSKRPPNVKSSNNSMLLVFKTDSFQTAKGWKM |
| O60494 | MSFRQTLGPQQGCGGYLTGSNNTFASPDSDSNGMYDKNLNC |
| O60494 | SGNFLTVQFISDLTLEREGFNATYTIMDMPCGGTYNATWTP |
| O60494 | EREGFNATYTIMDMPCGGTYNATWTPQNISSPNSSDPDVPF |
| O60494 | TYTIMDMPCGGTYNATWTPQNISSPNSSDPDVPFSICTWVI |
| O60494 | DMPCGGTYNATWTPQNISSPNSSDPDVPFSICTWVIDSPPH |
| O60494 | LQLQDSPQGHGNSRFQFCGRNASAVPVFYSSMSTAMVIFKS |

| | |
|---|---|
| O60494 | GWPDNYDNDKDCTVTLTAPQNHTISLFFHSLGIENSVECRN |
| O60494 | FHSLGIENSVECRNDFLEVRNGSNSNSPLLGKYCGTLLPNP |
| O60494 | GTLYGDRGSFTSPGYPGTYPNNTYCEWVLVAPAGRLVTINF |
| O60494 | ISIDDPGDCVQNYLTLYDGPNASSPSSGPYCGGDTSIAPFV |
| Q9JLB4 | LGECLHQIQRNKDDIIDLKRNTTGLPQNILSQVHQLNSKLV |
| Q9JLB4 | QCIETVSSYFCKCDSGWFGQNCTENINECVSNPCLNGGTCI |
| Q9JLB4 | QAACGGILSGTQGTFAYQSPNDTYVHNVNCFWVVRTDEEKV |
| Q9JLB4 | SDKGFHITYLTTPSDLYCGGNYTDTEGELLLPPLTGPFSHS |
| Q9JLB4 | SHSRQCVYLISQPQGEQIVINFTHVELESQRGCSHTFIEVG |
| Q9JLB4 | CSHTFIEVGDHESLLRKICGNETLFPIRSISNNVWIRLRID |
| Q9JLB4 | AGRRTCRWTISQPPREVVLLNFTDFQIGSSSSCDTDYIEIG |
| Q9JLB4 | TESTGIIESPGHPNVYPSGVNCTWHIVVQRGQLIRLVFSSF |
| Q9JLB4 | IFVSDSALAHEGFSINYEAINASSVCLYDYTDNFGRLSSPN |
| Q9JLB4 | ALTARGFSAYWDASSTGCGGNLTTPTGVLTSPNYPMPYYHS |
| Q9JLB4 | ASRGSPFLLEFQDFHLEHHPNCSLDYLAVFDGPSTNSRLIN |
| Q9JLB4 | QQGRGFEINYRQTCDNVVIVNKTSGILESINYPNPYDKDQR |
| Q9JLB4 | TSGILESINYPNPYDKDQRCNWTIQATTGNTVNYTFLEFDV |
| Q9JLB4 | PYDKDQRCNWTIQATTGNTVNYTFLEFDVENYVNCSTDYLE |
| Q9JLB4 | ATTGNTVNYTFLEFDVENYVNCSTDYLELYDGPQRIGRYCG |
| Q9JLB4 | TDNELAIRFKTDSSLNGRGFNASWRAVPGGCGGIFQVSRGE |
| Q9JLB4 | RANTECSWIIQVEKYHRVLLNITDFDLEATDSCLMTYDGSS |
| Q9JLB4 | TDSSDSISSPLYPANYPNNQNCTWIIEAQPPFNHIALSFTH |
| Q9JLB4 | SFQLENSLNCNKDFVEIREGNATGHLMGRYCGNSLPGNYSS |
| Q9JLB4 | REGNATGHLMGRYCGNSLPGNYSSIEGHNLWVRFVSDGSGT |
| Q9JLB4 | SDSSKSGRGFLLEWFAVDVSNVTLPTIAPGACGGYMVTGDT |
| Q9JLB4 | TGEYMYIRFTSDGSVTGAGFNASFQKSCGGYLHADRGIITS |
| Q9JLB4 | HADRGIITSPKYPDNYLPNLNCSWHVLVQSGLTIAVHFEQP |
| Q9JLB4 | PSGRSIQLQFEDQFNIEETPNCSASYLELRDGANSNAPVLS |
| Q9JLB4 | EDFNLQSSPGCAKDFVEIWENHTSGILLGRYCGNSIPSSVD |
| Q9JLB4 | IVFNGIRNNSPRLQKLCSRVNVTNEFKSSGNTMKVIFFTDG |
| Q9JLB4 | SYTSSEDAVCGGTLPSVSGGNFSSPGYNGIRDYARNLDCEW |
| Q9JLB4 | IRDYARNLDCEWTLSNPNRENSSISIHFLGLSLESHQDCTF |
| Q9JLB4 | GFYATWNTNTLGCGGTLHSDNGTIKSPHWPQTFPENSRCSW |
| Q9JLB4 | EEMPAQGFSASFISRCGRTFNSSTGDIVSPNFPKHYDNNMN |
| Q9JLB4 | DRSAVSGTCDYDGLHIIKGHNLSSTPLVTICGSETLRPLTI |
| Q9JLB4 | VPSNLCSHDYLEVFDGPSIGNRSIGKFCGSTLPQVIKSTNN |
| Q9JLB4 | GNRSIGKFCGSTLPQVIKSTNNSLTLLFKTDSSQTARGWKV |
| Q9JLB4 | SGNFLTVQFVSDISIQMRGFNATYTFVDMPCGGTYNATSMP |
| Q9JLB4 | QMRGFNATYTFVDMPCGGTYNATSMPQNTSSPQLSNIRRPF |
| Q9JLB4 | TYTFVDMPCGGTYNATSMPQNTSSPQLSNIRRPFSTCTWVI |
| Q9JLB4 | LELQDSEQTNGNQVTQFCGANYTTLPVFYSSGSTAVVVFKS |

| | |
|---|---|
| Q9JLB4 | FYWFQLEDSRQCMNDFLEVRNGSSSSSPLLGKYCSNLLPNP |
| Q9JLB4 | GTLLGNEGILANPGFPDSYPNNTHCEWTIVAPSGRPLSVGF |
| Q39547 | RYIYSIRSPGATIFKSTTILNASAPVVVSFSSRGPNRATKD |
| Q39547 | LNYPSFGLSVSPSQTFNQYFNRTLTSVAPQASTYRAMISAP |
| Q95RU0 | SALTFDESEELIYFNDLKHRNGSIFSLKRDLIAANHVAEQT |
| Q95RU0 | LKRDLIAANHVAEQTIARTGNESVGGLAYDPLNMNLFWSDT |
| Q95RU0 | GRPDGVAVDVCRRKLYWTNSNVTHPTVERINLDGSNRTVII |
| Q95RU0 | YWTNSNVTHPTVERINLDGSNRTVIISSNIDMPRGIVVDQL |
| Q95RU0 | STDFKDPEPVAEDCPLVRVANLSEEARGIVARTGFYQRLQK |
| Q95RU0 | ENEAPSCECPAKFGGARCEQNSTEICSLFCRLLKHEPEMYV |
| Q95RU0 | EPEMYVPFGCHSICEELAQDNSTNIAIPQYQHLEVCLTPRV |
| Q6UX04 | SRLRFNRRGLVAMANAGSHDNGSQFFFTLGRADELNNKHTI |
| Q6UX04 | RLKKEKPEEEVKKLKPKGTKNFSLLSFGEEAEEEEEEVNRV |
| P97792 | DLKGRVHFTSNDVKSGDASINVTNLQLSDIGTYQCKVKKAP |
| P61073 | VYVGVWIPALLLTIPDFIFANVSEADDRYICDRFYPNDLWV |
| P04839 | SAIHTIAHLFNVEWCVNARVNNSDPYSVALSELGDRQNESY |
| P04839 | ARVNNSDPYSVALSELGDRQNESYLNFARKRIKNPEGGLYL |
| P04839 | AIHGAERIVRGQTAESLAVHNITVCEQKISEWGKIKECPIP |
| P15425 | FGKLAPKTVANFRHICLRGINGTSYVGSRFHRVVDRFLVQG |
| O76096 | IKTNDPGVLQAARYSVEKFNNCTNDMFLFKESRITRALVQI |
| O76096 | GRTTCKKNQHLRLDDCDFQTNHTLKQTLSCYSEVWVVPWLQ |
| P50488 | ESEAAEKEWANTLVSKVAQSNGTGTIKVSAPAKPTLRRMDN |
| P50488 | EAHLKAVGCLAVFGLPTQEINSTEPYLKVDKPQYKSLGCMP |
| P50488 | PQYKSLGCMPYQHADSMNCENESSCRQGRSFRGGIGMCCCS |
| P08174 | EKDSVICLKGSQWSDIEEFCNRSCEVPTRLNSASLKQPYIT |
| Q14118 | PLDTDKGVHYISVSATRLGANGSHIPQTSSVFSIEVYPEDH |
| Q14118 | NDIHKKIALVKKLAFAFGDRNCSTITLQNITRGSIVVEWTN |
| Q14118 | LVKKLAFAFGDRNCSTITLQNITRGSIVVEWTNNTLPLEPC |
| Q14118 | NCSTITLQNITRGSIVVEWTNNTLPLEPCPKEQIAGLSRRI |
| Q24114 | ESIDIKSSGTYSEKGAICGGNCCNNATELELRDKAAGMFEQ |
| Q24114 | IKSSGTYSEKGAICGGNCCNNATELELRDKAAGMFEQLLHH |
| Q24114 | LETNAKQFQSHVLELAQISENMTHSLFSKVYTRMVPSSRMM |
| Q24114 | SRMMIHQLYTEIMNHLIYTSNYTNSNGQLGRRGIGSVQSNL |
| Q63155 | LHFVSEPSDAVTMRGGNVLLNCSAESDRGVPVIKWKKDGLI |
| Q63155 | WKKDGLILALGMDDRKQQLPNGSLLIQNILHSRHHKPDEGL |
| Q63155 | VIQLRSKKYSLLGGSNLLISNVTDDDSGTYTCVVTYKNENI |
| Q63155 | SNVTDDDSGTYTCVVTYKNENISASAELTVLVPPWFLNHPS |
| Q63155 | NIQTFTVFFSREGDNRERALNTTQPGSLQLTVGNLKPEAMY |
| Q63155 | VSTDDITVVTLSDVPSAPPQNVSLEVVNSRSIKVSWLPPPS |
| Q63155 | LFTGLEKGSQYSFQVSAMTVNGTGPPSNWYTAETPENDLDE |
| P80219 | GFLVREDFVLTAAHCLGSSINVTLGAHNIMERERTQQVIPV |

| | |
|---|---|
| Q08345 | LLSYTAPVGQTMYLSEAVYLNDSTYDGHTVGGLQYGGLGQL |
| Q08345 | DFRKSQELRVWPGYDYVGWSNHSFSSGYVEMEFEFDRLRAF |
| Q08345 | FLFAGPWLLFSEISFISDVVNNSSPALGGTFPPAPWWPPGP |
| Q08345 | LFAGPWLLFSEISFISDVVNNSSPALGGTFPPAPWWPPGPP |
| Q08345 | PALGGTFPPAPWWPPGPPPTNFSSLELEPRGQQPVAKAEGS |
| Q10025 | KFLRSAAHWKSRMILGCTWPNGTSCKLSDFKAVWTTTGLCW |
| Q10025 | DACTKHFRTKTLPGLKILIYNQTDIPDSSMNGVNVPSGYSM |
| Q10025 | RYMTEVENSCHCTLRRAYTSNSTDVKMKACNVDQYFGCAQK |
| Q10025 | LIEEQEEDDEDDVEQEELDENVSFSTVSGGETFSCEDSAYL |
| Q10025 | ERYKWGWHYDTFSGVADRLSNLTCFSNFSERHRDIISILES |
| Q10025 | WHYDTFSGVADRLSNLTCFSNFSERHRDIISILESRPITSE |
| Q10025 | SEEKKANQMFFLLDETAFNRNATRYMSVGDLKSRYGDKVDD |
| P81160 | LFLLLVVLASLLGLALSQDRNDTEWIQSQKDREKWCRLNLG |
| P83553 | SSVYDVLLEDRQWEFGCRTENVTQTCSTSGYANDFGLPLSY |
| P28845 | VAQAGKLMGGLDMLILNHITNTSLNLFHDDIHHVRKSMEVN |
| P28845 | VNFLSYVVLTVAALPMLKQSNGSIVVVSSLAGKVAYPMVAA |
| P28845 | KFALDGFFSSIRKEYSVSRVNVSITLCVLGLIDTETAMKAV |
| Q01459 | GSQVTFDVAWSPKNIDRRCYNYTGIADACDFLFVMSYDEQS |
| Q01459 | SYDEQSQIWSECIAAANAPYNQTLTGYNDYIKMSINPKKLV |
| Q01459 | INPKKLVMGVPWYGYDYTCLNLSEDHVCTIAKVPFRGAPCS |
| Q01459 | APCSDAAGRQVPYKTIMKQINSSISGNLWDKDQRAPYYNYK |
| Q09165 | RRVKRAPTRNNRPEPVVVGRNGTGKCVISADRASHFCGMEE |
| Q09165 | DNLRRLIIQYYRQQGKHQPKNATFSPKSFLFVKYHCDYGYE |
| Q09165 | PPRIYIEPQGPYEVAPGGNINISCTSVAYPFPDIYWFKNQK |
| Q09165 | KEIYRNEEFTCVSDNIHGSANRTVSIVVTGPGSAPHLKSAS |
| Q09165 | NIVEGDEIRVPPMTAFEIDCNVTRADPVPVLVWLHKGRPLN |
| Q09165 | ESTQFSCVAENEAGKSTKKINVTVTGPSAPERIRYQIDGDK |
| Q09165 | KDPDAPLSDWESKTVPADTRNLTVNVDDEDTPYVVKVQART |
| Q09165 | FRCVADGRPMPSVSYSWLPINASESGDEPVPIPIHSDDSQP |
| Q09165 | GAPIDAPTDVLPSVSIDNTVNITWSPPTQPLGPIKSYTVYF |
| Q09165 | PVRFETGSGEIPPTITLDPSNSTYTVEPLGAATITCTATGV |
| Q09165 | IKTLPLAPQAITNPIIQVHPNNSVTIEFTPPDDPENPGKKV |
| Q09165 | TANKPTTISVQYEVPSIPNGNISKYIIYYTPLDDQDPDHQL |
| Q09165 | GVEGSTTIQILPGSQMTIACNATGIPLPQVKWIKAGNYEID |
| Q09165 | IDPSRVDADGNHAQFSLQVANITEDTTFNCVAQNPLGHANW |
| Q09165 | ANITEDTTFNCVAQNPLGHANWTINVNLIEGLEPNWRDDFV |
| Q09165 | TDYGVTYSDTVTTPDGIPLSNDSTGAFITEDGTVIENNEDG |
| Q09165 | VPTESTNGVPLNKDGTPLPTNDSGHFVLVLPGATVNDSKPT |
| Q09165 | TPLPTNDSGHFVLVLPGATVNDSKPTDEVIVSITNPDGTLL |
| Q09165 | KNGKASDDYSSAVKSLKAERNVTVFVVDAGDDESQQQNSEL |
| Q09165 | AIALYGLNAARQQFQLHGRENATKVVILITNGKNRGNAAAA |

| | |
|---|---|
| Q09165 | IELIPLSPSSLSVSWTCCTNNKSNYTILYTHDTSITKEKWI |
| Q09165 | IPLSPSSLSVSWTCCTNNKSNYTILYTHDTSITKEKWIRKE |
| Q09165 | NELPSDHTYTVCVMTNERVDNSTALAIDKNCDSLHIDQNTT |
| Q09165 | VDNSTALAIDKNCDSLHIDQNTTAPEDYVKPSPSSCNCQCS |
| Q9SUQ8 | PCKHFSFYFHDILYDGDNVANATSAAIVSPPGLGNFKFGKF |
| Q9SUQ8 | FYFYDMKMDFNSWFSYTLVFNSTEHKGTLNIMGADLMMEPT |
| Q96SL1 | LSYLGGACAFLVGPLVVPAPNGTSPLLAAESSRAHIKDRIE |
| Q9VNJ5 | HRRRKNKHKNRNKNKRRKEQNQSSHEHHDVAQKMMQFKKRL |
| Q9VNJ5 | NLGFDTWIGDSGVFRDYEITNDSASSSLEPTRRTEQIEYGH |
| Q9VNJ5 | DSASSSLEPTRRTEQIEYGHNTTSVDEEEHQQRVQTKKSTW |
| Q9VNJ5 | FCDSSPRKEYSHFVVQRIGPNATDSLFDLNGLLAMCQLQDQ |
| Q9VNJ5 | MLTTECCRPWSLPNYAAMLANKSSCFDLTTEDVTSLHTLLL |
| Q9VNJ5 | RLNIVFNVLNFLTDFSFIKSNDSNVYLKYAMIFIPVAQSNR |
| Q9VNJ5 | DDGDYTNPNSYGHLHYDNNFNVSSRPAQLWILDFCQSVRQQ |
| Q9VNJ5 | GPKFAEAPRLETEDYLGMSGNESAEYSTNGSFTPLLVKALV |
| Q9VNJ5 | RLETEDYLGMSGNESAEYSTNGSFTPLLVKALVIEFESNVA |
| Q9UBS4 | FRIKVVKHPIFERRGDDLYTNVTISLVESLVGFEMDITHLD |
| Q9UBP4 | EAAAKASSEVNLANLPPSYHNETNTDTKVGNNTIHVHREIH |
| Q9UBP4 | NLANLPPSYHNETNTDTKVGNNTIHVHREIHKITNNQTGQM |
| Q9UBP4 | DTKVGNNTIHVHREIHKITNNQTGQMVFSETVITSVGDEEG |
| Q9UBP4 | CCGDQLCVWGHCTKMATRGSNGTICDNQRDCQPGLCCAFQR |
| Q09163 | WDGKFCEIDVRACTSTPCANNGTCVDLEKGQYECSCTPGFS |
| Q09163 | VDDEGQASHASCLCPPGFSGNFCEIVAATNSCTPNPCENDG |
| Q09163 | ASCLCPPGFSGNFCEIVAATNSCTPNPCENDGVCTDIGGDF |
| Q09163 | HELPVQQPEQHILKVSMKELNKSTPLLTEGQAICFTILGVL |
| P80370 | WDGELCDRDVRACSSAPCANNRTCVSLDDGLYECSCAPGYS |
| P80370 | VDDEGRASHASCLCPPGFSGNFCEIVANSCTPNPCENDGVC |
| P80370 | SHASCLCPPGFSGNFCEIVANSCTPNPCENDGVCTDIGGDF |
| P28078 | LPHSWAVLEASTPVLWDDPQNHTFRHTLFCQDGIPNIGLSE |
| Q9UGM3 | NARFGQGSGPIVLDDVRCSGNESYLWSCPHNGWLSHNCGHS |
| Q9UGM3 | TITLPPSTVGSESSLTLRLVNGSDRCQGRVEVLYRGSWGTV |
| Q9UGM3 | NARFGQGSGPIVLDDVRCSGNESYLWSCPHKGWLTHNCGHH |
| Q9UGM3 | LTHNCGHHEDAGVICSATQINSTTTDWWHPTTTTTARPSSN |
| Q9UGM3 | VNSGYRINLGFSNLKLEAHHNCSFDYVEIFDGSLNSSLLLG |
| Q9UGM3 | KLEAHHNCSFDYVEIFDGSLNSSLLLGKICNDTRQIFTSSY |
| Q9UGM3 | DYVEIFDGSLNSSLLLGKICNDTRQIFTSSYNRMTIHFRSD |
| Q9UGM3 | GFLAWYNSFPSDATLRLVNLNSSYGLCAGRVEIYHGGTWGT |
| Q9UGM3 | REDAGVICSGNHLSTPAPFLNITRPNTDYSCGGFLSQPSGD |
| Q9UGM3 | ISDHSITRRGFRAEYYSSPSNDSTNLLCLPNHMQASVSRSY |
| Q9UGM3 | PNLVIFTIPYSGCGTFKQADNDTIDYSNFLTAAVSGGIIKR |
| Q9UGM3 | RIHVSCRMLQNTWVDTMYIANDTIHVANNTIQVEEVQYGNF |

| | |
|---|---|
| Q9UGM3 | MLQNTWVDTMYIANDTIHVANNTIQVEEVQYGNFDVNISFY |
| Q9UGM3 | IHVANNTIQVEEVQYGNFDVNISFYTSSSFLYPVTSRPYYV |
| P28068 | LRNGLQNCATHTQPFWGSLTNRTRPPSVQVAKTTPFNTREP |
| Q9XWC3 | ASAKTCKYDSSDFEVYWRFANNSINMQFMNTDIKNNEWTGV |
| Q9XWC2 | LVSMNWNVKNNKIQIHFEHNNLTENRWTSIAFGDGPGMNNL |
| Q9XWC2 | ITGNKLKVTVSRPLGPAGPRNFSLDQCLNWMVVPGGSLSNG |
| P00639 | GLSLKIAAFNIRTFGETKMSNATLASYIVRIVRRYDIVLIQ |
| P24855 | AVSLKIAAFNIQTFGETKMSNATLVSYIVQILSRYDIALVQ |
| P24855 | PDQVSAVDSYYYDDGCEPCGNDTFNREPAIVRFFSRFTEVR |
| P11936 | ALSLRIAAFNIRTFGETKMSNATLSNYIVRILSRYDIALIQ |
| P11936 | PDQVSVLDSYLYDDGCEPCGNDTFNREPSVVKFSSPSTQVK |
| O18998 | AGSLKIAAFNIRSFGETKMSNATLTSYIVRILQRYDIALIQ |
| O42446 | SNSLLLGAFNIKSFGDTKASNATLMNIITKIVKRYDVILIQ |
| Q8NFT8 | GNASSNSSAGGRLVSFEVPQNTSVKIRQDATASLILLWKVT |
| Q8NFT8 | KDPCANVSCLNGATCDSDGLNGTCICAPGFTGEECDIDINE |
| O00115 | ALINSPEGAVGRSLQPLYRSNTSQLAFLLYNDQPPQPSKAQ |
| O00115 | QEFPDLENVVKGHHVSQEPWNSSITLTSQAGAVFQSFAKFS |
| O00115 | ALGTNLQVQFWHKTVGILPSNCSDIWQVLNVNQIAFPGPAG |
| O00115 | IWQVLNVNQIAFPGPAGPSFNSTEDHSKWCVSPKGPWTCVG |
| P49184 | HTAAAFDFPTSFQLTEEEALNISDHYPVEVELKLSQAHSVQ |
| P15101 | GTVHLVYGFLEEPLRSLESINTSGLHTGLQRVQLLKPSIPK |
| P15101 | SFNREVLKALYGFAPISMHCNRSSAVRFQGEWNRQPLPEIV |
| P09172 | ESPLPYHIPLDPEGSLELSWNVSYTQEAIHFQLLVRRLKAG |
| P09172 | GTVHLVYGILEEPFRSLEAINGSGLQMGLQRVQLLKPNIPE |
| P20036 | GGLANIAILNNNLNTLIQRSNHTQATNDPPEVTVFPKEPVE |
| P20036 | ELGQPNTLICHIDKFFPPVLNVTWLCNGELVTEGVAESLFL |
| P16444 | NDLPWQLLDMFNNRLQDERANLTTLAGTHTNIPKLRAGFVG |
| P16444 | TDSLVMVNFYNNYISCTNKANLSQVADHLDHIKEVAGARAV |
| P16444 | PEGLEDVSKYPDLIAELLRRNWTEAEVKGALADNLLRVFEA |
| P16444 | VKGALADNLLRVFEAVEQASNLTQAPEEEPIPLDQLGGSCR |
| Q9H4A9 | DLPLVLRQVYQKGLQDVNLRNFSYGQTSLDRLRDGLVGAQF |
| Q9H4A9 | IRRMCASYSELELVTSAKALNDTQKLACLIGVEGGHSLDNS |
| Q9H4A9 | HTCNTPWAESSAKGVHSFYNNISGLTDFGEKVVAEMNRLGM |
| P22412 | NDLPWQLLNLFNNQLQDPGANLSSLAHTHTNIPKLKAGFVG |
| P22412 | TGSLVMVNFYNDYVSCSAKANLSQVADHLDHIKKVAGAAAV |
| Q18253 | FSVIFTFMQNPINLNSDNGFNQTSGNTSSLEATTLKPKFSS |
| Q18253 | TFMQNPINLNSDNGFNQTSGNTSSLEATTLKPKFSSLMTTT |
| Q18253 | KVNELWRHSAEYLYHIVKINNKTVSTEQWHVGPEENSLIQA |
| Q18253 | EQWHVGPEENSLIQAFYWNPNASSNDFVYVHNYNLYYQKDP |
| Q18253 | TDDDSYVEYFELPYPKAGVQNNTLVTQYIWDSENHKIVETA |
| Q18253 | LSAANGDYYVLTNKWITMPRNGSDLGEERLVTVWANRDQNH |

| | |
|---|---|
| Q18253 | GQPAENLQGYINTNVIPHARNVTNVKYLLAHGERDDNVHYQ |
| Q7JKY3 | AALVFFTPLFAAKSFGSWRLNVSDLRSLRYPYAEFAFTDNN |
| Q7JKY3 | ADRKYFAMMDHAPNPGMNPQNETFHLKIVNNNERLNPLLPF |
| Q7JKY3 | WWSTKGDQLAYASYDNHLTKNVSLKTYHRLEPYPIDTNFHY |
| Q7JKY3 | VQLKDSLSYHYLLAVKWLEINGTEQLVSVWTNRYQNEVALT |
| Q7JKY3 | SYSLADESRNSAYCISCSIKNCTWAQAQMDDQMKTAIVSCK |
| Q7JKY3 | QMKTAIVSCKGPAAPHTAIVNLTRMDSDKKTEHANLLYDKT |
| Q8N608 | SLEDLFRKDFVLHDPEARWINDTDVVYKSENGHVIKLNIET |
| Q8N608 | DTDVVYKSENGHVIKLNIETNATTLLLENTTFVTFKASRHS |
| Q8N608 | ENGHVIKLNIETNATTLLLENTTFVTFKASRHSVSPDLKYV |
| Q8N608 | HSHIAHWWSPDGERLAFLMINDSLVPTMVIPRFTGALYPKG |
| Q8N608 | TMVKWVSNTKTVVRWLNRAQNISILTVCETTTGACSKKYEM |
| Q8N608 | TKVHFQHSAELIKHLIKAGVNYTMQVYPDEGHNVSEKSKYH |
| Q9UHL4 | GFQERFFQQRLDHFNFERFGNKTFPQRFLVSDRFWVRGEGP |
| Q9UHL4 | RGEGPIFFYTGNEGDVWAFANNSAFVAELAAERGALLVFAE |
| Q9UHL4 | RLLSEAQRITGLRALAGLVYNASGSEHCYDIYRLYHSCADP |
| Q9UHL4 | TGCGTGPDARAWDYQACTEINLTFASNNVTDMFPDLPFTDE |
| Q9UHL4 | DARAWDYQACTEINLTFASNNVTDMFPDLPFTDELRQRYCL |
| Q9UHL4 | SNIIFSNGNLDPWAGGGIRRNLSASVIAVTIQGGAHHLDLR |
| P27487 | DHEYLYKQENNILVFNAEYGNSSVFLENSTFDEFGHSINDY |
| P27487 | QENNILVFNAEYGNSSVFLENSTFDEFGHSINDYSISPDGQ |
| P27487 | ASYDIYDLNKRQLITEERIPNNTQWVTWSPVGHKLAYVWNN |
| P27487 | TDWVYEEEVFSAYSALWWSPNGTFLAYAQFNDTEVPLIEYS |
| P27487 | SAYSALWWSPNGTFLAYAQFNDTEVPLIEYSFYSDESLQYP |
| P27487 | AVNPTVKFFVVNTDSLSSVTNATSIQITAPASMLIGDHYLC |
| P27487 | CDVTWATQERISLQWLRRIQNYSVMDICDYDESSGRWNCLV |
| P27487 | LDKMLQNVQMPSKKLDFIILNETKFWYQMILPPHFDKSKKY |
| P27487 | VYTERYMGLPTPEDNLDHYRNSTVMSRAENFKQVEYLLIHG |
| P28843 | DFEYLYKQENNILLLNAEHGNSSIFLENSTFESFGYHSVSP |
| P28843 | QENNILLLNAEHGNSSIFLENSTFESFGYHSVSPDRLFVLL |
| P28843 | SYNIYDVNKRQLITEEKIPNNTQWITWSPEGHKLAYVWKND |
| P28843 | TDWVYEEEVFGAYSALWWSPNNTFLAYAQFNDTGVPLIEYS |
| P28843 | GAYSALWWSPNNTFLAYAQFNDTGVPLIEYSFYSDESLQYP |
| P28843 | CDVVWATEERISLQWLRRIQNYSVMAICDYDKINLTWNCPS |
| P28843 | QWLRRIQNYSVMAICDYDKINLTWNCPSEQQHVEMSTTGWV |
| P28843 | LDRMLQDVQMPSKKLDFIVLNETRFWYQMILPPHFDKSKKY |
| P28843 | VYTERYMGLPIPEDNLDHYRNSTVMSRAEHFKQVEYLLIHG |
| P22411 | DHEYLYKQENNILLFNAEYGNSSIFLENSTFDELGYSTNDY |
| P22411 | QENNILLFNAEYGNSSIFLENSTFDELGYSTNDYSVSPDRQ |
| P22411 | SAYSALWWSPNGTFLAYAQFNDTEVPLIEYSFYSDESLQYP |
| P22411 | AGAENPTVKFFVVDTRTLSPNASVTSYQIVPPASVLIGDHY |

| | |
|---|---|
| P22411 | CGVTWVTEERISLQWIRRAQNYSIIDICDYDESTGRWISSV |
| P22411 | VYTERYMGLPTPEDNLDYYRNSTVMSRAENFKQVEYLLIHG |
| P14740 | DSEYLYKQENNILLFNAEHGNSSIFLENSTFEIFGDSISDY |
| P14740 | QENNILLFNAEHGNSSIFLENSTFEIFGDSISDYSVSPDRL |
| P14740 | ASYSIYDLNKRQLITEEKIPNNTQWITWSQEGHKLAYVWKN |
| P14740 | NDWVYEEEIFGAYSALWWSPNGTFLAYAQFNDTGVPLIEYS |
| P14740 | GAYSALWWSPNGTFLAYAQFNDTGVPLIEYSFYSDESLQYP |
| P14740 | CDVAWVSEDRISLQWLRRIQNYSVMAICDYDKTTLVWNCPT |
| P14740 | LDKMLQDVQMPSKKLDFIVLNETRFWYQMILPPHFDKSKKY |
| P14740 | VYTERYMGLPTPEDNLDHYRNSTVMSRAENFKQVEYLLIHG |
| P42658 | DTEFIYREQKGTVRLWNVETNTSTVLIEGKKIESLRAIRYE |
| P42658 | KTHIAHWWSPDGTRLAYAAINDSRVPIMELPTYTGSIYPTV |
| P42658 | TMVKWATSTKVAVTWLNRAQNVSILTLCDATTGVCTKKHED |
| P42658 | AIPQGGRGKFYHITVSSSQPNSSNDNIQSITSGDWDVTKIL |
| P42658 | YSANTVGNFNRQCLSCDLVENCTYFSASFSHSMDFFLLKCE |
| P42658 | SMDFFLLKCEGPGVPMVTVHNTTDKKKMFDLETNEHVKKAI |
| P42658 | EKIHFQHTAELITQLIRGKANYSLQIYPDESHYFTSSSLKQ |
| P01903 | GALANIAVDKANLEIMTKRSNYTPITNVPPEVTVLTNSPVE |
| P01903 | ELREPNVLICFIDKFTPPVVNVTWLRNGKPVTTGVSETVFL |
| P79483 | FAGDTRPRFLELRKSECHFFNGTERVRYLDRYFHNQEEFLR |
| Q30154 | LAGDTRPRFLQQDKYECHFFNGTERVRFLHRDIYNQEEDLR |
| P21918 | PWEEDFWEPDVNAENCDSSLNRTYAISSSLISFYIPVAIMI |
| P55849 | VLSAREKKVFRKRHTKEPVHNRSKRRWAPIPCSLMENSLGP |
| P55849 | ENSLGPFPQHIQQIQSDAAQNYTIFYSISGPGVDKEPYNLF |
| P55849 | DLRTVKVLDRESKFVKNNQYNISVVATDTAGRSCTGTLVVL |
| P55849 | LEPIDLDGPDNGPPFQFLLDNSSSKLWTLESQDGKRAILRQ |
| Q02487 | LCRLLLLTLAILIFASDACKNVTLHVPSKLDAEKLVGRVNL |
| Q02487 | ENSLGPFPLFLQQVQSDTAQNYTIYYSIRGPGVDQEPRNLF |
| Q02487 | EILRVTVEDKDLVNTANWRANYTILKGNENGNFKIVTDAKT |
| Q02487 | SIKVFRSLDREAETIKNGIYNITVLASDQGGRTCTGTLGII |
| Q02487 | DFSLESSTSEVQRMWRLKAINDTAARLSYQNDPPFGSYVVP |
| P55850 | ENSLGPFPLFLQQVQSDAAQNYTVFYSISGRGADQEPLNWF |
| P55850 | EILRIPVDDKDMINTANWKANFTILKGNENGWFKITTDPVT |
| P55850 | LLTISKTLDREIIAPRNDMYNITVMAIDQEGKSCTGTLAVN |
| P55850 | QFNLANTSPEVNRIWTINQVNDTAARLSYQKTADVQIYNVP |
| Q7TSF1 | VGIDQPPYGIFIINQKTGEINITSIVDREVTPFFIIYCRAL |
| Q7TSF1 | TTFLGQIEENSNANTLVMKLNATDADEPNNLNSMIAFKIIR |
| Q03763 | VGIDQPPYGIFVINQKTGEINITSIVDREVTPFFIIYCRAL |
| Q03763 | STFVGEIEENSNANTLVMVLNATDADEPNNLNSKIAFKIIR |
| Q03763 | TGTIVINLENGGWKTERPNVNGSTTSAYGLTSGGVTTNGYT |
| Q02413 | LVVVEVNSEFRIQVRDYNTKNGTIKWHSIRRQKREWIKFAA |

| | |
|---|---|
| Q02413 | VGIDQPPYGIFVINQKTGEINITSIVDREVTPFFIIYCRAL |
| Q02413 | ATFAGQIEENSNANTLVMILNATDADEPNNLNSKIAFKIIR |
| Q7TSF0 | VGIDQPPYGIFIINQKTGEINITSIVDREVTPFFIIYCRAL |
| Q7TSF0 | TTFLGQIEENSNANTLVMKLNATDADEPNNLNSMIAFKIIR |
| Q7TSF0 | KGCVFQPGSKTFIVDSRMEANHTVGEFLATDCETGQATNKF |
| O55111 | KGITEPPFGIFVFDRNTGELNITSILDREETPYFLLTGYAL |
| O55111 | EVMRIKVTDADEVGSDNWLANFTFASGNEGGYFHIETDTQT |
| O55111 | SVTSEIKLVKIPDFESRYVQNGTYTAKVVAISKEHPQKTIT |
| O55111 | DNCPVLVDSVRSVCEDEPYVNVTAEDLDGAQNSAPFSFSII |
| P32926 | VGIDQPPFGIFVVDKNTGDINITAIVDREETPSFLITCRAL |
| P32926 | QIFMGEIEENSASNSLVMILNATDADEPNHLNSKIAFKIVS |
| P32926 | SKTAEIKFVKNMNRDSTFIVNKTITAEVLAIDEYTGKTSTG |
| P32926 | TFALEDQPVKLPAVWSITTLNATSALLRAQEQIPPGVYHIS |
| Q14126 | KGITEPPFGIFVFNKDTGELNVTSILDREETPFFLLTGYAL |
| Q14126 | DVFVGSVEELSAAHTLVMKINATDADEPNTLNSKISYRIVS |
| Q14126 | EVTRIKVFDADEIGSDNWLANFTFASGNEGGYFHIETDAQT |
| Q14126 | SVTSEIKLAKLPDFESRYVQNGTYTVKIVAISEDYPRKTIT |
| Q14126 | DNCPTLIEPVQTICHDAEYVNVTAEDLDGHPNSGPFSFSVI |
| O61213 | AGSRLHRDARSYYSDGVYSVNNSLPSARELSDILFKGESGI |
| O61213 | QKIIAYDFVPGLLGEDVRLSNYTKYMPHVPPGISHAFGAAA |
| O61213 | LQKDVFFFKEGDPCPQPFQVNTTGLEPCVPFMQSTYWTDND |
| O61213 | VNTTGLEPCVPFMQSTYWTDNDTTYVFTLIGLACVPLICYG |
| Q8IVF4 | LANTVQENAKSWVISLGKLLNESAKEELYNLHEEMEHLAKN |
| P52407 | GMQGNNLPPVSEVIALYKKSNITRMRIYDPNRAVLEALRGS |
| P52407 | PEVEKHFGLFFPNKWQKYNLNFSAEKNWDISTEHNATILFL |
| Q9Z2B5 | LQRTQKTVRAVGPRSGSEKWNFSVGHFELRYIPDMETRAGF |
| Q10901 | ATFQQVQTKYIKVRPKVVKNNDSATLAALNNGSLDYVKASV |
| Q10901 | IKVRPKVVKNNDSATLAALNNGSLDYVKASVEYTSGMNVLG |
| P56564 | CFKQFKTSYEKRSFKVPIQSNETLLGAVINNVSEAMETLTR |
| P56564 | EKRSFKVPIQSNETLLGAVINNVSEAMETLTRIREEMVPVP |
| P56564 | KRSFKVPIQSNETLLGAVINNVSEAMETLTRIREEMVPVPG |
| Q21353 | QATFQQVQTEYMPIKPSRVRNATSMNMTSEVLHKQTLTYTN |
| Q21353 | QVQTEYMPIKPSRVRNATSMNMTSEVLHKQTLTYTNEMNVL |
| P42892 | WGTFSNLWEHNQAIIKHLLENSTASVSEAERKAQVYYRACM |
| P42892 | STASVSEAERKAQVYYRACMNETRIEELRAKPLMELIERLG |
| P42892 | RIEELRAKPLMELIERLGGWNITGPWAKDNFQDTLQVVTAH |
| P42892 | SNVIQVDQSGLGLPSRDYYLNKTENEKVLTGYLNYMVQLGK |
| P42892 | DEEAIRPQMQQILDFETALANITIPQEKRRDEELIYHKVTA |
| P42892 | LAPAINWLPFLNTIFYPVEINESEPIVVYDKEYLEQISTLI |
| P42892 | ESEPIVVYDKEYLEQISTLINTTDRCLLNNYMIWNLVRKTS |
| P42892 | VFNDYTAVPDLYFENAMRFFNFSWRVTADQLRKAPNRDQWS |

| | |
|---|---|
| P42892 | AFDDQGREYDKDGNLRPWWKNSSVEAFKRQTECMVEQYSNY |
| P42892 | KNSSVEAFKRQTECMVEQYSNYSVNGEPVNGRHTLGENIAD |
| Q4PZA2 | WGTFSNLWEHNQAVIKHLLENATASVSEAERKAQVYYRACM |
| Q4PZA2 | ATASVSEAERKAQVYYRACMNETRIEELRAKPLMELIEKLG |
| Q4PZA2 | RIEELRAKPLMELIEKLGGWNITGPWAKDNFQDTLQVVTAH |
| Q4PZA2 | VTAHYRTSPFFSVYVSADSKNSNSNVIQVDQSGLGLPSRDY |
| Q4PZA2 | YRTSPFFSVYVSADSKNSNSNVIQVDQSGLGLPSRDYYLNK |
| Q4PZA2 | SNVIQVDQSGLGLPSRDYYLNKTENEKVLTGYLNYMVQLGK |
| Q4PZA2 | DEDAIRPQMQQILDFETALANITIPQEKRRDEELIYHKVTA |
| Q4PZA2 | LAPAINWLPFLNTIFYPVEINESEPIVVYDKEYLRQVSTLI |
| Q4PZA2 | ESEPIVVYDKEYLRQVSTLINNTDKCLLNNYMMWNLVRKTS |
| Q4PZA2 | SEPIVVYDKEYLRQVSTLINNTDKCLLNNYMMWNLVRKTSS |
| Q4PZA2 | VFNDYTAVPDLYFENAMRFFNFSWRVTADQLRKAPNRDQWS |
| Q4PZA2 | AFDDQGREYDKDGNLRPWWKNSSVEAFKQQTECMVQQYSNY |
| Q4PZA2 | KNSSVEAFKQQTECMVQQYSNYSVNGEPVNGRHTLGENIAD |
| Q16610 | ELLALIQLEREFQRCCRQGNNHTCTWKAWEDTLDKYCDREY |
| Q16610 | HLCGNQRVLTKHKHIPGLIHNMTARCCDLPFPEQACCAEEE |
| Q61508 | QLQALTRLETEFQRCCRQGHNHTCTWKAWEGTLDGYCEREL |
| Q61508 | QLCGSGRVLSKHKQIPGLIQNMTIRCCELPYPEQACCGEEE |
| Q61508 | INCFNTNYLRNVALVAGDTGNATGLGEQGPTRGTDANPAPG |
| Q9BZQ6 | DALGKFSLTLIDSLDTLVVLNKTKEFEDAVRKVLRDVNLDN |
| Q9BZQ6 | YNDELLQMAKQLGYKLLPAFNTTSGLPYPRINLKFGIRKPE |
| Q9BZQ6 | IEDYIFTTEAHLLPLWLSTTNQSISKKNTTSEYTELDDSNF |
| Q9BZQ6 | TEAHLLPLWLSTTNQSISKKNTTSEYTELDDSNFDWTCPNT |
| Q9BZQ6 | SDKAKDRDPEMENEEQPSSENDSQNQSGEQISSSSQEVDLV |
| Q9BZQ6 | KDRDPEMENEEQPSSENDSQNQSGEQISSSSQEVDLVDQES |
| Q9BZQ6 | CTDLDNQLQEQSETEEDSNPNVSWGKKVQPIDSILADWNED |
| O43854 | INECEVEPCKNGGICTDLVANYSCECPGEFMGRNCQYKCSG |
| P20827 | APLLGLCCSLAAADRHTVFWNSSNPKFRNEDYTIHVQLNDY |
| O08545 | LPLLAQGPGGALGNRHAVYWNSSNQHLRREGYTVQVNVNDY |
| O08545 | EGYTVQVNVNDYLDIYCPHYNSSGPGGGAEQYVLYMVNLSG |
| O08545 | PHYNSSGPGGGAEQYVLYMVNLSGYRTCNASQGSKRWECNR |
| O08545 | GGGAEQYVLYMVNLSGYRTCNASQGSKRWECNRQHASHSPI |
| O08543 | CVFSQDPGSKVVADRYAVYWNSSNPRFQRGDYHIDVCINDY |
| O08543 | AIPDNGRRSCLKLKVFVRPTNSCMKTIGVHDRVFDVNDKVE |
| P52803 | CVFSQDPGSKAVADRYAVYWNSSNPRFQRGDYHIDVCINDY |
| P52800 | LMVLCRTAISRSIVLEPIYWNSSNSKFLPGQGLVLYPQIGD |
| P52800 | PNLWGLEFQKNKDYYIISTSNGSLEGLDNQEGGVCQTRAMK |
| O35393 | RGAAHSAEPGRDTIPGDPSSNATSRGAEGPLPPPSMPAVAG |
| P36368 | QEEPSAQHRLVSKSFPHPGFNMSLLMLQTIPPGADFSNDLM |
| Q9GV16 | HGTNVVVKHKPYHPSVEGYDNTSFSEVDMKILQDLGLNTIR |

| | |
|---|---|
| Q9GV16 | LNTIRLGMMLPGYVPTRGNYNETYLKIIQEIVSKAAKYGIY |
| Q9GV16 | HAWGDYYFTEAAAAAFQNFYNNTDGLLDAWADFWKKTAQGF |
| Q9GV16 | VTWDYFAAGFSKVPGGDAYRNRSVLSYHYYEPPDFNKKFQF |
| Q9GV16 | GCLTDSMHDETGHLRDIVLQNTTRTYPQAVAGHTIGYKFDR |
| P00533 | NKLTQLGTFEDHFLSLQRMFNNCEVVLGNLEITYVQRNYDL |
| P00533 | RMFNNCEVVLGNLEITYVQRNYDLSFLKTIQEVAGYVLIAL |
| P00533 | RGNMYYENSYALAVLSNYDANKTGLKELPMRNLQEILHGAV |
| P00533 | ALCNVESIQWRDIVSSDFLSNMSMDFQNHLGSCQKCDPSCP |
| P00533 | MSMDFQNHLGSCQKCDPSCPNGSCWGAGEENCQKLTKIICA |
| P00533 | PCRKVCNGIGIGEFKDSLSINATNIKHFKNCTSISGDLHIL |
| P00533 | GIGEFKDSLSINATNIKHFKNCTSISGDLHILPVAFRGDSF |
| P00533 | DILKTVKEITGFLLIQAWPENRTDLHAFENLEIIRGRTKQH |
| P00533 | EIIRGRTKQHGQFSLAVVSLNITSLGLRSLKEISDGDVIIS |
| P00533 | ALCSPEGCWGPEPRDCVSCRNVSRGRECVDKCNLLEGEPRE |
| P00533 | EFVENSECIQCHPECLPQAMNITCTGRGPDNCIQCAHYIDG |
| P00533 | AHYIDGPHCVKTCPAGVMGENNTLVWKYADAGHVCHLCHPN |
| P00533 | NNTLVWKYADAGHVCHLCHPNCTYGCTGPGLEGCPTNGPKI |
| P01133 | VSLSAPQHWSCPEGTLAGNGNSTCVGPAPFLIFSHGNSIFR |
| P01133 | NEKRIYWVDLERQLLQRVFLNGSRQERVCNIEKNVSGMAIN |
| P01133 | LLQRVFLNGSRQERVCNIEKNVSGMAINWINEEVIWSNQQE |
| P01133 | EEVIWSNQQEGIITVTDMKGNNSHILLSALKYPANVAVDPV |
| P01133 | PKAEDDTWEPEQKLCKLRKGNCSSTVCGQDLQSHLCMCAEG |
| P01133 | VGFVLLPDGKRCHQLVSCPRNVSECSHDCVLTSEGPLCFCP |
| P01133 | KSLIGRSDLNGKRSKIITKENISQPRGIAVHPMAKRLFWTD |
| P01133 | DLKNQVTPLDILSKTRVSEDNITESQHMLVAEIMVSDQDDC |
| P01133 | DGIHCLDIDECQLGEHSCGENASCTNTEGGYTCMCAGRLSE |
| Q01279 | RGNALYENTYALAILSNYGTNRTGLRELPMRNLQEILIGAV |
| Q01279 | ILCNMDTIQWRDIVQNVFMSNMSMDLQSHPSSCPKCDPSCP |
| Q01279 | MSMDLQSHPSSCPKCDPSCPNGSCWGGGEENCQKLTKIICA |
| Q01279 | PCRKVCNGIGIGEFKDTLSINATNIKHFKYCTAISGDLHIL |
| Q01279 | EILKTVKEITGFLLIQAWPDNWTDLHAFENLEIIRGRTKQH |
| Q01279 | EIIRGRTKQHGQFSLAVVGLNITSLGLRSLKEISDGDVIIS |
| Q01279 | PLCSSEGCWGPEPRDCVSCQNVSRGRECVEKCNILEGEPRE |
| Q01279 | EFVENSECIQCHPECLPQAMNITCTGRGPDNCIQCAHYIDG |
| Q01279 | AHYIDGPHCVKTCPAGIMGENNTLVWKYADANNVCHLCHAN |
| Q01279 | NNTLVWKYADANNVCHLCHANCTYGCAGPGLQGCEVWPSGP |
| P17813 | FLEFPTGPSQLELTLQASKQNGTWPREVLLVLSVNSSVFLH |
| P17813 | LQASKQNGTWPREVLLVLSVNSSVFLHLQALGIPLHLAYNS |
| P17813 | VNSSVFLHLQALGIPLHLAYNSSLVTFQEPPGVNTTELPSF |
| P17813 | IPLHLAYNSSLVTFQEPPGVNTTELPSFPKTQILEWAAERG |
| P17813 | IRGFKLPDTPQGLLGEARMLNASIVASFVELPLASIVSLHA |

| | |
|---|---|
| P08246 | AAHCVANVNVRAVRVVLGAHNLSRREPTRQVFAVQRIFENG |
| P08246 | IFENGYDPVNLLNDIVILQLNGSATINANVQVAQLPAQGRR |
| P08246 | AMGWGLLGRNRGIASVLQELNVTVVTSLCRRSNVCTLVRGR |
| Q6PCB8 | GSAPDSPFTSPPLREEIMANNFSLESHNISLTEHSSMPVEK |
| Q6PCB8 | FTSPPLREEIMANNFSLESHNISLTEHSSMPVEKNITLERP |
| Q6PCB8 | FSLESHNISLTEHSSMPVEKNITLERPSNVNLTCQFTTSGD |
| Q6PCB8 | TEHSSMPVEKNITLERPSNVNLTCQFTTSGDLNAVNVTWKK |
| Q6PCB8 | RPSNVNLTCQFTTSGDLNAVNVTWKKDGEQLENNYLVSATG |
| Q6PCB8 | ISYVGDSTVLTCKCQNCFPLNWTWYSSNGSVKVPVGVQMNK |
| Q6PCB8 | TVLTCKCQNCFPLNWTWYSSNGSVKVPVGVQMNKYVINGTY |
| Q6PCB8 | YSSNGSVKVPVGVQMNKYVINGTYANETKLKITQLLEEDGE |
| Q6PCB8 | SVKVPVGVQMNKYVINGTYANETKLKITQLLEEDGESYWCR |
| P21995 | PTDPTFTSLPVREEMMAKYSNLSLKSCNISVTEKSNVSVEE |
| P21995 | SLPVREEMMAKYSNLSLKSCNISVTEKSNVSVEENVILEKP |
| P21995 | MAKYSNLSLKSCNISVTEKSNVSVEENVILEKPSHVELKCV |
| P21995 | LSLKSCNISVTEKSNVSVEENVILEKPSHVELKCVYTATKD |
| P21995 | ILEKPSHVELKCVYTATKDLNLMNVTWKKDDEPLETTGDFN |
| P21995 | KPSHVELKCVYTATKDLNLMNVTWKKDDEPLETTGDFNTTK |
| P21995 | NLMNVTWKKDDEPLETTGDFNTTKMGNTLTSQYRFIVFNSK |
| P21995 | IAYVGDSTVLKCVCQDCLPLNWTWYMGNETAQVPIDAHSNE |
| P21995 | TVLKCVCQDCLPLNWTWYMGNETAQVPIDAHSNEKYIINGS |
| P21995 | LNWTWYMGNETAQVPIDAHSNEKYIINGSHANETRLKIKHL |
| P21995 | MGNETAQVPIDAHSNEKYIINGSHANETRLKIKHLLEEDGG |
| P21995 | AQVPIDAHSNEKYIINGSHANETRLKIKHLLEEDGGSYWCR |
| Q5UCC4 | VTHPGGCRGHEVEDVDLELFNTSVQLQPPTTAPGPETAAFI |
| Q8C7X2 | ENLIPYSPDVQVHAERFINYNQTVSRMRGIYTAPSGLESTC |
| Q8N766 | ENLIPYSPDVQIHAERFINYNQTVSRMRGIYTAPSGLESTC |
| Q9Y6C2 | APALGPASSTPRPLAQPARPNLSGSSAGSPLSGLGGEGPGE |
| Q9Y6C2 | GHPPGYTSLASRLSRLEDRFNSTLGPSEEQEESWPGAPGGL |
| Q9Y6C2 | LSHWLPAARGRLEQLGGLLANVSGELGGRLDLLEEQVAGAM |
| Q9Y6C2 | RLQDRVDAQDETAAEFTLRLNLTAARLGQLEGLLQAHGDEG |
| Q9Y6C2 | SALQALQGELSEVILSFSSLNDSLNELQTTVEGQGADLADL |
| Q9Y6C2 | GLREGLSRHVAGLWAGLRETNTTSQMQAALLEKLVGGQAGL |
| Q9Y6C2 | ALLEKLVGGQAGLGRRLGALNSSLQLLEDRLHQLSLKDLTG |
| Q9BXX0 | GYPARPSARNKNWCAYIVNKNVSCSVLEGSESFIQAQYNCA |
| Q9BXX0 | KESGMKDIKSELAEVKDTLKNKSDKLEELDGKVKGYEGQLR |
| Q9BXX0 | IVPEPDVDFDAKWNELDARINVTEKNAEEHCFYIEETLRGA |
| Q9BXX0 | VDQKIQSLEDRLGSVLLQMTNNTGAELSPPGAAALPGVSGS |
| Q9BXX0 | ALPNREDRAVRDSLHLLKSLNDTMHRKFQETEQTIQKLQQD |
| Q9BXX0 | ETEQTIQKLQQDFSFLYSQLNHTENDVTHLQKEMSNCRAGE |
| Q9BXX0 | RIKEGLNKHVSSLWNCVRQMNGTLRSHSRDISGLKNSVQQF |

| | |
|---|---|
| Q9BXX0 | ITATLTPERDAYVEAVLSVSNASVAQLHTAGYRREFLEYHR |
| P47801 | TAIMLFVSTIANVWMVADYANASVGLWKNCTGGNCDGSLSY |
| P47801 | TIANVWMVADYANASVGLWKNCTGGNCDGSLSYGNEDAIKA |
| P47801 | CILVGVSIYTHHYAHSEGNFNSSSHQGYCFILTWICFCFSF |
| O97939 | EKPKEKDPPKTETPATEPSVNTTVPETNSTQPNAPNPRGND |
| O97939 | PPKTETPATEPSVNTTVPETNSTQPNAPNPRGNDTSPTGTS |
| O97939 | VNTTVPETNSTQPNAPNPRGNDTSPTGTSGQGPNPRSNPTG |
| O97939 | TSGQGPNPRSNPTGQNGPAVNVSGQGVPRSQSPWGPRQTII |
| O97939 | TVVPTRDPSGPWRNSQDYGINKSNYKLPQPEDNMLVPNFNS |
| O97939 | SHIKYARQTVSPTSIVPGQRNSSEKILPGESQNPSPFKDDV |
| O97939 | VFGTPDKEPRPEGIPNEMQGNESERQQQRQSSILQLPCFGS |
| Q21109 | AKNGEISGMHNWVRFYVLENNRTENFDYKGFTVKRFNIMAA |
| P08110 | SRTDDEVVQREEEAIQLDGLNASQIKEIREKSEKFAFQAEV |
| P08110 | KLIINSLYKNKEIFLRELISNASDALDKIRLISLTDENALA |
| P08110 | VGFYSAFLVADRVIVTSKHNNDTQHIWESDSNEFSVIDDPR |
| P08110 | MMPKYLNFVKGVVDSDDLPLNVSRETLQQHKLLKVIRKKLV |
| P08110 | RKKLVRKTLDMIKKIAEEKYNDTFWKEFGTNVKLGVIEDHS |
| P08110 | DTFWKEFGTNVKLGVIEDHSNRTRLAKLLRFQSSHHESNLT |
| P14625 | SRTDDEVVQREEEAIQLDGLNASQIRELREKSEKFAFQAEV |
| P14625 | KLIINSLYKNKEIFLRELISNASDALDKIRLISLTDENALS |
| P14625 | VGFYSAFLVADKVIVTSKHNNDTQHIWESDSNEFSVIADPR |
| P14625 | MMPKYLNFVKGVVDSDDLPLNVSRETLQQHKLLKVIRKKLV |
| P14625 | RKKLVRKTLDMIKKIADDKYNDTFWKEFGTNIKLGVIEDHS |
| P14625 | DTFWKEFGTNIKLGVIEDHSNRTRLAKLLRFQSSHHPTDIT |
| P08113 | SRTDDEVVQREEEAIQLDGLNASQIRELREKSEKFAFQAEV |
| P08113 | KLIINSLYKNKEIFLRELISNASDALDKIRLISLTDENALA |
| P08113 | VGFYSAFLVADKVIVTSKHNNDTQHIWESDSNEFSVIADPR |
| P08113 | MMPKYLNFVKGVVDSDDLPLNVSRETLQQHKLLKVIRKKLV |
| P08113 | RKKLVRKTLDMIKKIADEKYNDTFWKEFGTNIKLGVIEDHS |
| P08113 | DTFWKEFGTNIKLGVIEDHSNRTRLAKLLRFQSSHHSTDIT |
| P90754 | VYKRKTGKKANCLFWVGCAYNNSGYAPDVAPAYNQELPFRN |
| P90754 | DYLDLKGLITAKGVVGRVYINDTTISVNDVVDKFRCKIDTV |
| P90754 | DYFNPKMHTIFYARGPSFKQNTTISPYQNIQYMNLWMNLLG |
| P90754 | NIQYMNLWMNLLGIEGAVETNGTIGFFDNILTNPPRRDNPT |
| P90754 | TNVIGECPMIAFPSVLKCSGNVSAETLNQLSVKLTNCAFSP |
| P90754 | NDARRAIIEVLSRDEASNPSNFTFLNAKYQSNCPSHIPTGS |
| P90754 | KMYVISGTATDINHDGIADSNGSVITHIYRIMLICNSTWLL |
| P90754 | GIADSNGSVITHIYRIMLICNSTWLLMNPPLCTDSDSMDTL |
| P22413 | LTRSLCACSDDCKDKGDCCINYSSVCQGEKSWVEEPCESIN |
| P22413 | TGLYPESHGIIDNKMYDPKMNASFSLKSKEKFNPEWYKGEP |
| P22413 | FWPGSDVEINGIFPDIYKMYNGSVPFEERILAVLQWLQLPK |

| | |
|---|---|
| P22413 | LRPSDVPDKYYSFNYEGIARNLSCREPNQHFKPYLKHFLPK |
| P22413 | ENIEVYNLMCDLLNLTPAPNNGTHGSLNHLLKNPVYTPKHP |
| P22413 | NLGCSCNPSILPIEDFQTQFNLTVAEEKIIKHETLPYGRPR |
| P22413 | MSGYSQDILMPLWTSYTVDRNDSFSTEDFSNCLYQDFRIPL |
| P22413 | CLYQDFRIPLSPVHKCSFYKNNTKVSYGFLSPPQLNKNSSG |
| P22413 | FYKNNTKVSYGFLSPPQLNKNSSGIYSEALLTTNIVPMYQS |
| P06802 | LSRFVCSCADDCKTHNDCCINYSSVCQDKKSWVEETCESID |
| P06802 | TGLYPESHGIIDNKMYDPKMNASFSLKSKEKFNPLWYKGQP |
| P06802 | FWPGSDVEIDGILPDIYKVYNGSVPFEERILAVLEWLQLPS |
| P06802 | LRPTDVPETYYSFNYEALAKNLSCREPNQHFRPYLKPFLPK |
| P06802 | ENIEVYNLMCDLLGLIPAPNNGSHGSLNHLLKKPIYNPSHP |
| P06802 | DLGCTCDPWIVPIKDFEKQLNLTTEDVDDIYHMTVPYGRPR |
| Q9R1E6 | IKRAEWDEGPPTVLSDSPWTNTSGSCKGRCFELQEVGPPDC |
| Q9R1E6 | GRIRPKIPNNLKYDPKAIIANLTCKKPDQHFKPYMKQHLPK |
| Q9R1E6 | ENIELYNVMCDLLGLKPAPNNGTHGSLNHLLRTNTFRPTLP |
| Q9R1E6 | GPLSVSSFILPHRPDNDESCNSSEDESKWVEELMKMHTARV |
| Q64610 | IKRAEWDEGPPTVLSDSPWTNTSGSCKGRCFELQEVGPPDC |
| Q64610 | NVDDITLVPGTLGRIRAKSINNSKYDPKTIIANLTCKKPDQ |
| Q64610 | GRIRAKSINNSKYDPKTIIANLTCKKPDQHFKPYMKQHLPK |
| Q64610 | ENIELYNVMCDLLGLKPAPNNGTHGSLNHLLRTNTFRPTMP |
| Q64610 | STEAETGKFRGSKHENKKNLNGSVEPRKERHLLYGRPAVLY |
| Q64610 | GPLSVSSFILPHRPDNDESCNSSEDESKWVEELMKMHTARV |
| Q6DYE8 | LYPESHGIIDNNMYDVHLNKNFSLSSVEKSNPAWWSGQPIW |
| Q6DYE8 | AMYQGLKAACYYWPGSDVAVNGSFPTIYRNYSNSVPYERRI |
| Q6DYE8 | CYYWPGSDVAVNGSFPTIYRNYSNSVPYERRITTLLQWLDL |
| Q6DYE8 | IRTRNIPQDFFTFNSEEIVRNLSCRKPDQHFKPYLTPDLPK |
| Q6DYE8 | ENIEVYNLLCDLLHIEPAPNNGTHGSLNHLLKTPFYKPSHA |
| Q6DYE8 | CSCPALQNTPGLEEQANQRLNLSEGEVAATVKANLPFGRPR |
| Q6DYE8 | ADVRVAPSESQKCSFYLADKNITHGFLYPAIKGTNESRYDA |
| Q6DYE8 | FYLADKNITHGFLYPAIKGTNESRYDALITSNLVPMYKEFK |
| Q6DYE8 | LDVLPFIVPHRPTNIESCSENKTEDLWVEERFQAHAARVRD |
| Q9Y6X5 | MWPGTDVPIHDTISSYFMNYNSSVSFEERLNNITMWLNNSN |
| Q9Y6X5 | TISSYFMNYNSSVSFEERLNNITMWLNNSNPPVTFATLYWE |
| Q9Y6X5 | DHSYYTLIDLSPVAAILPKINRTEVYNKLKNCSPHMNVYLK |
| Q9Y6X5 | NIVDIYPMMCHILGLKPHPNNGTFGHTKCLLVDQWCINLPE |
| Q8BTJ4 | LQENRSSAAAMWPGTDVPIHNITASYFMNYSSSVSFKERLG |
| Q8BTJ4 | ITASYFMNYSSSVSFKERLGNVTTWLSSSNPPVTFAALYWE |
| Q8BTJ4 | DRSNYSVIDLTPVAAILPKINVTEVYDKLKRCNPHMNVYLK |
| Q8BTJ4 | HSSRIQPIILVAEEGWTITLNKSSFKLGDHGYDNSLPSMHP |
| Q8BTJ4 | NTVDIYPMMCHILGLKPHPNNGTLSHTKCLLVDQWCINLPE |
| F1N5C8 | TGRHCEVHQMTGNYMWDPDTNKSFDLGINRDSRLPLWWNGS |

| | |
|---|---|
| F1N5C8 | DTNKSFDLGINRDSRLPLWWNGSEPLWVTLTKAKRKVYMYY |
| F1N5C8 | LVADEGWFITENRESLPFWMNSTVTRKPEGWQWGWHGYDNE |
| F1N5C8 | RVVDIYNLMCKVTGVTPLPNNGSWSRVMCMLKDPASSAPGA |
| Q6UWV6 | FTLVTGKYIENHGVVHNMYYNTTSKVKLPYHATLGIQRWWD |
| Q6UWV6 | TTSKVKLPYHATLGIQRWWDNGSVPIWITAQRQGLRAGSFF |
| Q6UWV6 | IWITAQRQGLRAGSFFYPGGNVTYQGVAVTRSRKEGIAHNY |
| Q6UWV6 | TYQGVAVTRSRKEGIAHNYKNETEWRANIDTVMAWFTEEDL |
| Q6UWV6 | HGMTTVDKRAGDLVEFHKFPNFTFRDIEFELLDYGPNGMLL |
| P49961 | LDAGSSHTSLYIYKWPAEKENDTGVVHQVEECRVKGPGISK |
| P49961 | ETFGALDLGGASTQVTFVPQNQTIESPDNALQFRLYGKDYN |
| P49961 | VASNEILRDPCFHPGYKKVVNVSDLYKTPCTKRFEMTLPFQ |
| P49961 | FEIQGIGNYQQCHQSILELFNTSYCPYSQCAFNGIFLPPLQ |
| P49961 | PPLQGDFGAFSAFYFVMKFLNLTSEKVSQEKVTEMMKKFCA |
| P49961 | IHFIGKIQGSDAGWTLGYMLNLTNMIPAEQPLSTPLSHSTY |
| Q9Y5L3 | LDAGSSHTSMFIYKWPADKENDTGIVGQHSSCDVPGGGISS |
| Q9Y5L3 | KERHAGTPLYLGATAGMRLLNLTNPEASTSVLMAVTHTLTQ |
| Q9Y5L3 | VLLGDVYQSPCTMAQRPQNFNSSARVSLSGSSDPHLCRDLV |
| Q9Y5L3 | SMGLPVATLQQLEAAAVNVCNQTWAQLQARVPGQRARLADY |
| Q9Y5L3 | VIFQKKAADTAVGWALGYMLNLTNLIPADPPGLRKGTDFSS |
| O55026 | LDAGSSHTSMFVYKWPADKENDTGIVGQHSSCDVRGGGISS |
| O55026 | KDRYASTPLYLGATAGMRLLNLTSPEATAKVLEAVTQTLTR |
| O55026 | VLLREVYQSPCTMGQRPQTFNSSATVSLSGTSNAALCRDLV |
| O55026 | VSLSGTSNAALCRDLVSGLFNISSCPFSQCSFNGVFQPPVA |
| O55026 | VMGLPVGTLKQLEDATETTCNQTWAELQARVPGQQTRLPDY |
| O55026 | VVFEKKAADTAVGWALGYMLNLTNLIPADLPGLRKGTHFSS |
| O75354 | VKKVFKASPFLVGDDCVSIMNGTDEGVSAWITINFLTGSLK |
| O75354 | RVEGTLQASPPGYLTALRMFNRTYKLYSYSYLGLGLMSARL |
| O75356 | QFEKTLEQTPRGYLTSFEMFNSTYKLYTHSYLGFGLKAARL |
| O75356 | GILKVEDFERKAREVCDNLENFTSGSPFLCMDLSYITALLK |
| P14351 | TISRIQWNKDIQVLGPVIDWNVTQRAVYQPLQTRRIARSLR |
| P14351 | TRRIARSLRMQHPVPKYVEVNMTSIPQGVYYEPHPEPIVVK |
| P14351 | RVLGLSQILMINSENIANNANLTQEVKKLLTEMVNEEMQSL |
| P14351 | TYNRQSIWDYYIKVESIRPANWTTKSKYGQARLGSFYIPSS |
| P14351 | SKYGQARLGSFYIPSSLRQINVSHVLFCSDQLYSKWYNIEN |
| P14351 | WYNIENTIEQNERFLLNKLNNLTSGTSVLKKRALPKDWSSQ |
| P14351 | LFREINVLDICSKPESVILLNTSYYSFSLWEGDCNFTKDMI |
| P14351 | ESVILLNTSYYSFSLWEGDCNFTKDMISQLVPECDGFYNNS |
| P14351 | DCNFTKDMISQLVPECDGFYNNSKWMHMHPYACRFWRSKKN |
| P14351 | KIASKAYGIDTVLFSLKNFLNYTGTPVNEMPNARAFVGLID |
| P14351 | MPNARAFVGLIDPKFPPSYPNVTREHYTSCNNRKRRSVDNN |
| P14351 | DCTRQDYVICDVVKIVQPCGNSSDTSDCPVWAEAVKEPFVQ |

| | |
|---|---|
| P14351 | DCPVWAEAVKEPFVQVNPLKNGSYLVLASSTDCQIPPYVPS |
| P14351 | VLASSTDCQIPPYVPSIVTVNETTSCFGLDFKRPLVAEERL |
| C1JJY3 | EPAYRNVEKTNSPDYSLAYSNTTVEKSLVDGTTVNTTGVSM |
| P03375 | VWATHACVPTDPNPQEVVLVNVTENFNMWKNDMVEQMHEDI |
| P03375 | LKPCVKLTPLCVSLKCTDLKNDTNTNSSSGRMIMEKGEIKN |
| P03375 | KLTPLCVSLKCTDLKNDTNTNSSSGRMIMEKGEIKNCSFNI |
| P03375 | NDTNTNSSSGRMIMEKGEIKNCSFNISTSIRGKVQKEYAFF |
| P03375 | TNSSSGRMIMEKGEIKNCSFNISTSIRGKVQKEYAFFYKLD |
| P03375 | RGKVQKEYAFFYKLDIIPIDNDTTSYTLTSCNTSVITQACP |
| P03375 | YKLDIIPIDNDTTSYTLTSCNTSVITQACPKVSFEPIPIHY |
| P03375 | FEPIPIHYCAPAGFAILKCNNKTFNGTGPCTNVSTVQCTHG |
| P03375 | PIHYCAPAGFAILKCNNKTFNGTGPCTNVSTVQCTHGIRPV |
| P03375 | AGFAILKCNNKTFNGTGPCTNVSTVQCTHGIRPVVSTQLLL |
| P03375 | VSTVQCTHGIRPVVSTQLLLNGSLAEEEVVIRSANFTDNAK |
| P03375 | STQLLLNGSLAEEEVVIRSANFTDNAKTIIVQLNQSVEINC |
| P03375 | EVVIRSANFTDNAKTIIVQLNQSVEINCTRPNNNTRKSIRI |
| P03375 | ANFTDNAKTIIVQLNQSVEINCTRPNNNTRKSIRIQRGPGR |
| P03375 | AKTIIVQLNQSVEINCTRPNNNTRKSIRIQRGPGRAFVTIG |
| P03375 | GPGRAFVTIGKIGNMRQAHCNISRAKWNNTLKQIDSKLREQ |
| P03375 | TIGKIGNMRQAHCNISRAKWNNTLKQIDSKLREQFGNNKTI |
| P03375 | AKWNNTLKQIDSKLREQFGNNKTIIFKQSSGGDPEIVTHSF |
| P03375 | GGDPEIVTHSFNCGGEFFYCNSTQLFNSTWFNSTWSTKGSN |
| P03375 | VTHSFNCGGEFFYCNSTQLFNSTWFNSTWSTKGSNNTEGSD |
| P03375 | NCGGEFFYCNSTQLFNSTWFNSTWSTKGSNNTEGSDTITLP |
| P03375 | NSTQLFNSTWFNSTWSTKGSNNTEGSDTITLPCRIKQIINM |
| P03375 | QEVGKAMYAPPISGQIRCSSNITGLLLTRDGGNSNNESEIF |
| P03375 | IRCSSNITGLLLTRDGGNSNNESEIFRPGGGDMRDNWRSEL |
| P03375 | QLLGIWGCSGKLICTTAVPWNASWSNKSLEQIWNNMTWMEW |
| P03375 | WGCSGKLICTTAVPWNASWSNKSLEQIWNNMTWMEWDREIN |
| P03375 | TTAVPWNASWSNKSLEQIWNNMTWMEWDREINNYTSLIHSL |
| P03375 | KSLEQIWNNMTWMEWDREINNYTSLIHSLIEESQNQQEKNE |
| P03375 | EKNEQELLELDKWASLWNWFNITNWLWYIKLFIMIVGGLVG |
| P04578 | VWATHACVPTDPNPQEVVLVNVTENFNMWKNDMVEQMHEDI |
| P04578 | LKPCVKLTPLCVSLKCTDLKNDTNTNSSSGRMIMEKGEIKN |
| P04578 | KLTPLCVSLKCTDLKNDTNTNSSSGRMIMEKGEIKNCSFNI |
| P04578 | NDTNTNSSSGRMIMEKGEIKNCSFNISTSIRGKVQKEYAFF |
| P04578 | TNSSSGRMIMEKGEIKNCSFNISTSIRGKVQKEYAFFYKLD |
| P04578 | RGKVQKEYAFFYKLDIIPIDNDTTSYKLTSCNTSVITQACP |
| P04578 | YKLDIIPIDNDTTSYKLTSCNTSVITQACPKVSFEPIPIHY |
| P04578 | FEPIPIHYCAPAGFAILKCNNKTFNGTGPCTNVSTVQCTHG |
| P04578 | PIHYCAPAGFAILKCNNKTFNGTGPCTNVSTVQCTHGIRPV |

| | |
|---|---|
| P04578 | AGFAILKCNNKTFNGTGPCTNVSTVQCTHGIRPVVSTQLLL |
| P04578 | VSTVQCTHGIRPVVSTQLLLNGSLAEEEVVIRSVNFTDNAK |
| P04578 | STQLLLNGSLAEEEVVIRSVNFTDNAKTIIVQLNTSVEINC |
| P04578 | EVVIRSVNFTDNAKTIIVQLNTSVEINCTRPNNNTRKRIRI |
| P04578 | VNFTDNAKTIIVQLNTSVEINCTRPNNNTRKRIRIQRGPGR |
| P04578 | AKTIIVQLNTSVEINCTRPNNNTRKRIRIQRGPGRAFVTIG |
| P04578 | GPGRAFVTIGKIGNMRQAHCNISRAKWNNTLKQIASKLREQ |
| P04578 | TIGKIGNMRQAHCNISRAKWNNTLKQIASKLREQFGNNKTI |
| P04578 | AKWNNTLKQIASKLREQFGNNKTIIFKQSSGGDPEIVTHSF |
| P04578 | GGDPEIVTHSFNCGGEFFYCNSTQLFNSTWFNSTWSTEGSN |
| P04578 | VTHSFNCGGEFFYCNSTQLFNSTWFNSTWSTEGSNNTEGSD |
| P04578 | NCGGEFFYCNSTQLFNSTWFNSTWSTEGSNNTEGSDTITLP |
| P04578 | NSTQLFNSTWFNSTWSTEGSNNTEGSDTITLPCRIKQIINM |
| P04578 | QKVGKAMYAPPISGQIRCSSNITGLLLTRDGGNSNNESEIF |
| P04578 | IRCSSNITGLLLTRDGGNSNNESEIFRPGGGDMRDNWRSEL |
| P04578 | QLLGIWGCSGKLICTTAVPWNASWSNKSLEQIWNHTTWMEW |
| P04578 | WGCSGKLICTTAVPWNASWSNKSLEQIWNHTTWMEWDREIN |
| P04578 | CTTAVPWNASWSNKSLEQIWNHTTWMEWDREINNYTSLIHS |
| P04578 | KSLEQIWNHTTWMEWDREINNYTSLIHSLIEESQNQQEKNE |
| P04578 | EKNEQELLELDKWASLWNWFNITNWLWYIKLFIMIVGGLVG |
| P03390 | FLSLKGARSAAPGSSPHQVYNITWEVTNGDRETVWAISGNH |
| P03390 | TTGRVYWKPSSSWDYITVDNNLTTSQAVQVCKDNKWCNPLA |
| P03390 | PPAGTGDRLLNLVQGAYQALNLTNPDKTQECWLCLVSGPPY |
| P03390 | LCLVSGPPYYEGVAVLGTYSNHTSAPANCSVASQHKLTLSE |
| P03390 | PYYEGVAVLGTYSNHTSAPANCSVASQHKLTLSEVTGRGLC |
| P03390 | EVTGRGLCIGTVPKTHQALCNTTLKIDKGSYYLVAPTGTTW |
| P03390 | TGTTWACNTGLTPCLSATVLNRTTDYCVLVELWPRVTYHPP |
| P03395 | TTGRAYWKPSSSWDYITVDNNLTTNQAVQVCKDNKWCNPLA |
| P03395 | QLSFPLPNPLPKPAKSPPVSNSTPTMISPSPTPTQPPPAGT |
| P03395 | PPAGTGDRLLNLVQGAYQALNLTNPDKTQECWLCLVSGPPY |
| P03395 | LCLVSGPPYYEGVAVLGTYSNHTSAPTNCSVASQHKLTLSE |
| P03395 | PYYEGVAVLGTYSNHTSAPTNCSVASQHKLTLSEVTGRGLC |
| P03395 | EVTGRGLCIGTVPKTHQALCNTTLKTNKGSYYLVAPAGTTW |
| P03395 | AGTTWACNTGLTPCLSATVLNRTTDYCVLVELWPRVTYHPP |
| Q39688 | LTILLFFIQRIDFCHTLVPANETFKFVNEGELGQYISEYFG |
| Q39688 | RTESLMRWVWEANRGNPVDENATLTFGPDGNLVLARSNGQV |
| Q39688 | TTSPKPIRYYSFSLFTKLNKNESLQNVTFEFENENDQGFAF |
| Q39688 | PIRYYSFSLFTKLNKNESLQNVTFEFENENDQGFAFLLSLK |
| Q39688 | LKYGTSNSLGGASILNRIKYNTTLSFLRLEIDGNVKIYTYN |
| P16422 | QNTVICSKLAAKCLVMKAEMNGSKLGRRAKPEGALQNNDGL |
| P16422 | NDGLYDPDCDESGLFKAKQCNGTSMCWCVNTAGVRRTDKDT |

| | |
|---|---|
| P16422 | PKFITSILYENNVITIDLVQNSSQKTQNDVDIADVAYYFEK |
| Q9UNN8 | RLHMLQISYFRDPYHVWYQGNASLGGHLTHVLEGPDTNTTI |
| Q9UNN8 | YQGNASLGGHLTHVLEGPDTNTTIIQLQPLQEPESWARTQS |
| Q9UNN8 | LGCELPPEGSRAHVFFEVAVNGSSFVSFRPERALWQADTQV |
| Q9UNN8 | ADTQVTSGVVTFTLQQLNAYNRTRYELREFLEDTCVQYVQK |
| Q9UM22 | TKQCSKMTLTQPWDPLDIPQNSTFEDQYSIGGPQEQITVQE |
| Q9UM22 | TWIGIYTVKDCYPVQETFTINYSVILSTRFFDIQLGIKDPS |
| P29317 | EPPHGLTRTSVTVSDLEPHMNYTFTVEARNGVSGLVTSRSF |
| P29317 | RNGVSGLVTSRSFRTASVSINQTEPPKVRLEGRSTTSLSVS |
| Q03145 | EPPHALTRTSVTVSDLEPHMNYTFAVEARNGVSGLVTSRSF |
| Q03145 | RNGVSGLVTSRSFRTASVSINQTEPPKVRLEDRSTTSLSVT |
| P54763 | GEWLVPIGRCMCKAGFEAVENGTVCRGCPSGTFKANQGDEA |
| P54763 | PLDMPCTTIPSAPQAVISSVNETSLMLEWTPPRDSGGREDL |
| P54763 | IQAVNGVTDQSPFSPQFASVNITTNQAAPSAVSIMHQVSRT |
| P54763 | PNGVILDYELQYYEKELSEYNATAIKSPTNTVTVQGLKAGA |
| P54761 | ACMALLSLHLFYKKCSWLITNLTYFPETVPRELVVPVAGSC |
| P54761 | PRSSPCTTPPSAPRSVVHHLNGSTLRLEWSAPLESGGREDL |
| P54761 | VAALNGVSTLATGPPPFEPVNVTTDREVPPAVSDIRVTRSS |
| Q21313 | CGHCNAGNENSHPAANMVDGNNSWWMSPPLSRGLQHNEVNI |
| Q21313 | GNNSWWMSPPLSRGLQHNEVNITIDLEQEFHVAYVWIQMAN |
| Q21313 | IRILEHRPSSRQFATSEALQNFTRATNVRLRLLGTRTLQGH |
| Q21313 | CERCCPGFVQKQWQAATAHNNFTCEACNCFGRSNECEYDAE |
| Q21313 | DQCASGYYDAPKCKPCECNVNGTIGDVCLPEDGQCPCKAGF |
| Q21313 | CPCKAGFGGTFCETCADGYTNVTAGCVECVCDATGSEHGNC |
| Q21313 | TNVTAGCVECVCDATGSEHGNCSASTGQCECKPAYAGLSCD |
| Q21313 | GAGITSPECDATSGQCPCNGNFTGRTCDKCAAGFYNYPDCR |
| Q21313 | TFWDLQYHHEDGCRSCDCNVNGTISGLNTCDLKTGQCMCKK |
| Q21313 | YEGTVLRQRAPQPCLSHSTKNTTCVDLIYPPIPSVSRQFVD |
| Q21313 | ERPEVIQFYMDDKYTATFYHNSSQKGPIYIDSITAVPYNSY |
| Q21313 | VPRGGRKSMNADADVRLTGANMTIEYWASEQPTNPEEQFTV |
| Q21313 | TAEGKTVTREELMKVLHSLQNITLKASYFDHPKTSTLYEFG |
| Q21313 | DCEHNTNGDHCEFCNEGHYGNATNGSPYDCMACACPFAPTN |
| Q21313 | FGHPQISGESCSPCQCNGNNNLTDSRSCHPNSGDCYLCEQN |
| Q21313 | DGRHCESCAAWFYGDAVTAKNCSSCECSQCGSQYCDNKSGG |
| Q21313 | VTAKNCSSCECSQCGSQYCDNKSGGCECKINVEGDSCDRCK |
| Q21313 | LIGDVDNLELEIDVLGTAIANISSATIVGARLARNKKEFND |
| Q21313 | LNDEENSFGNVFGDAQDILTNSTQIQNKLVRTKTHSQNSVS |
| Q21313 | IQNKLVRTKTHSQNSVSSAKNITLNGTEFLQEVMKRAQRAR |
| Q21313 | LVRTKTHSQNSVSSAKNITLNGTEFLQEVMKRAQRARQSVR |
| Q21313 | DLERVEAAKGEFQKLNVAIGNITENLKDKREEMTHAVTTLN |
| Q21313 | NITENLKDKREEMTHAVTTLNETRNDVAEALEAAKKRVRRD |

| | |
|---|---|
| Q21313 | TFDNNKDNTDQAVEAANAFSNLTDTLKNAKAQIDNAYEALS |
| Q21313 | TLSKPNSVETAETKSSVAGGNKSVLNLNQQISRLFVGGVPT |
| Q21313 | PLHSVRTVSFLSDRTTASFNNATEFSEDVSVTFKFKTRSIR |
| Q21313 | FANSEIKEISLNGCSLSDDENISTTTTAAPKPTDDSDVAVL |
| Q21313 | MKNGILFSVGVLEYITVEFVNGSIKTTVESGSGGEELWHHP |
| P14753 | QRLEDLVCFWEEAASSGMDFNYSFSYQLEGESRKSCSLHQA |
| P01588 | RLICDSRVLERYLLEAKEAENITTGCAEHCSLNENITVPDT |
| P01588 | EAKEAENITTGCAEHCSLNENITVPDTKVNFYAWKRMEVGQ |
| P01588 | VWQGLALLSEAVLRGQALLVNSSQPWEPLQLHVDKAVSGLR |
| P79119 | LHLQNNNIMEMHEDTFCNVKNLTYIRKALEDIRLDGNPINL |
| Q9NZ08 | NKIRLPEYVIPVHYDLLIHANLTTLTFWGTTKVEITASQPT |
| Q9NZ08 | LAPEPLLVGLPYTVVIHYAGNLSETFHGFYKSTYRTKEGEL |
| Q9NZ08 | LKVGDYFFGKCFDAMEVDALNSSHPVSTPVENPAQIREMFD |
| Q9NZ08 | YQPCVQRAEGYFRKWKESNGNLSLPVDVTLAVFAVGAQSTE |
| Q9NZ08 | QFSTRTRLEEVKGFFSSLKENGSQLRCVQQTIETIEENIGW |
| Q6P179 | QELRLPSVVIPLHYDLFVHPNLTSLDFVASEKIEVLVSNAT |
| Q6P179 | VLVSNATQFIILHSKDLEITNATLQSEEDSRYMKPGKELKV |
| Q6P179 | WWNDIWLKEGFAKYMELIAVNATYPELQFDDYFLNVCFEVI |
| Q6P179 | YYIVHYEGHGWDQLITQLNQNHTLLRPKDRVGLIHDVFQLV |
| P04626 | HLYQGCQVVQGNLELTYLPTNASLSFLQDIQEVQGYVLIAH |
| P04626 | GTQLFEDNYALAVLDNGDPLNNTTPVTGASPGGLRELQLRS |
| P04626 | ILWKDIFHKNNQLALTLIDTNRSRACHPCSPMCKGSRCWGE |
| P04626 | QCAAGCTGPKHSDCLACLHFNHSGICELHCPALVTYNTDTF |
| P04626 | ACHQLCARGHCWGPGPTQCVNCSQFLRGQECVEECRVLQGL |
| P04626 | PREYVNARHCLPCHPECQPQNGSVTCFGPEADQCVACAHYK |
| P04626 | SYMPIWKFPDEEGACQPCPINCTHSCVDLDDKGCPAEQRAS |
| P06494 | HLYQGCQVVQGNLELTYVPANASLSFLQDIQEVQGYMLIAH |
| P06494 | VLWKDVFRKNNQLAPVDIDTNRSRACPPCAPACKDNHCWGE |
| P06494 | QCAAGCTGPKHSDCLACLHFNHSGICELHCPALVTYNTDTF |
| P06494 | VCNSLCAHGHCWGPGPTQCVNCSHFLRGQECVEECRVWKGL |
| P06494 | PREYVSDKRCLPCHPECQPQNSSETCFGSEADQCAACAHYK |
| P06494 | SYMPIWKYPDEEGICQPCPINCTHSCVDLDERGCPAEQRAS |
| P21860 | RGTQVYDGKFAIFVMLNYNTNSSHALRQLRLTQLTEILSGG |
| P21860 | ECAGGCSGPQDTDCFACRHFNDSGACVPRCPQPLVYNKLTF |
| P21860 | GTGSGSRFQTVDSSNIDGFVNCTKILGNLDFLITGLNGDPW |
| P21860 | RTVREITGYLNIQSWPPHMHNFSVFSNLTTIGGRSLYNRGF |
| P21860 | TGYLNIQSWPPHMHNFSVFSNLTTIGGRSLYNRGFSLLIMK |
| P21860 | TIGGRSLYNRGFSLLIMKNLNVTSLGFRSLKEISAGRIYIS |
| P21860 | ISAGRIYISANRQLCYHHSLNWTKVLRGPTEERLDIKHNRP |
| P21860 | PLCSSGGCWGPGPGQCLSCRNYSRGGVCVTHCNFLNGEPRE |
| P21860 | EAECFSCHPECQPMEGTATCNGSGSDTCAQCAHFRDGPHCV |

| | |
|---|---|
| P21860 | AKGPIYKYPDVQNECRPCHENCTQGCKGPELQDCLGQTLVL |
| Q15303 | AIFLNYRKDGNFGLQELGLKNLTEILNGGVYVDQNKFLCYA |
| Q15303 | FLCYADTIHWQDIVRNPWPSNLTLVSTNGSSGCGRCHKSCT |
| Q15303 | IHWQDIVRNPWPSNLTLVSTNGSSGCGRCHKSCTGRCWGPT |
| Q15303 | ECAGGCSGPKDTDCFACMNFNDSGACVTQCPQTFVYNPTTF |
| Q15303 | GTGSLMSAQTVDSSNIDKFINCTKINGNLIFLVTGIHGDPY |
| Q15303 | NVFRTVREITGFLNIQSWPPNMTDFSVFSNLVTIGGRVLYS |
| Q15303 | ISAGNIYITDNSNLCYYHTINWTTLFSTINQRIVIRDNRKA |
| Q15303 | TTLFSTINQRIVIRDNRKAENCTAEGMVCNHLCSSDGCWGP |
| Q15303 | RGRICIESCNLYDGEFREFENGSICVECDPQCEKMEDGLLT |
| Q15303 | DPQCEKMEDGLLTCHGPGPDNCTKCSHFKDGPNCVEKCPDG |
| Q15303 | ANSFIFKYADPDRECHPCHPNCTQGCNGPTSHDCIYYPWTG |
| Q9Y282 | SFQQSHVHVHDLQSFGLDNINMTHYIQHLSFGEDYPGIVNP |
| Q9Y282 | IQHLSFGEDYPGIVNPLDHTNVTAPQASMMFQYFVKVVPTV |
| Q969X5 | NELYVDDPDKDSGGKIDVSLNISLPNLHCELVGLDIQDEMG |
| O94905 | FDRIEVVNFLVPNAVYDIVKNYTADYDKALIFNKIHHELNQ |
| O75477 | IDRIEVVNMLAPYAVFDIVRNYTADYDKTLIFNKIHHELNQ |
| Q7Z2K6 | QRPTGSFSIDFLGGFTSYYDNITNVVVKLEPRDGAQHAVLA |
| Q7Z2K6 | WINGFDYTGISHITPHIPEINDSIRAHCEENAPLCGFPWYL |
| Q86YB8 | ESKIPVGIKAGHSNKYLKMANNTKELEDCEQANKLGAINST |
| Q86YB8 | MANNTKELEDCEQANKLGAINSTLSNQSKEAFIDWARYDDS |
| Q86YB8 | KELEDCEQANKLGAINSTLSNQSKEAFIDWARYDDSRDHFC |
| Q86YB8 | FAGDKKGAKSLKEEFRLHFKNISRIMDCVGCDKCRLWGKLQ |
| Q96HE7 | VHLSARYLLQETWLEKKWGHNITEFQQRFDGILTEGEGPRR |
| Q96HE7 | FAGDKKEAHKLKEDFRLHFRNISRIMDCVGCFKCRLWGKLQ |
| Q09216 | NPYEYSHENPRLRRIIALHANRTIYDFEGHLTQKDNALFVH |
| Q09216 | DYRGASDLPDHSFLQGSSAPNCTGVVDEYCRMPYYTAIHEL |
| Q09216 | VLPYPIDLKLVSRHAMGENLNLTFEIRGGYDKMSLHVTPLN |
| Q18600 | VSGCFDIPAHDTEGMNICYRNVSNVMARLGKGEKKDKISVL |
| Q18600 | RTQFFHVNQMLYDRNGQISVNDTRFYAISHDYRGAEDIPFV |
| Q18600 | VIGTGQISVYIIPDTTWLITNTSVTQPKTPQENMFLYYTCS |
| Q96AP7 | INGVTTSKPGVSLVYSMPSRNLSLRLEGLQEKDSGPYSCSV |
| Q96AP7 | VLVPPAPPSCRLQGVPHVGANVTLSCQSPRSKPAVQYQWDR |
| Q96AP7 | SFQTFFAPALDVIRGSLSLTNLSSSMAGVYVCKAHNEVGTA |
| Q96AP7 | SSMAGVYVCKAHNEVGTAQCNVTLEVSTGPGAAVVAGAVVG |
| P23953 | PLGSLRFAPPQPAEPWSFVKNATSYPPMCSQDAGWAKILSD |
| P23953 | NVGKKNIQAVNEIIATLSQCNDTSSAAMVQCLRQKTESELL |
| P23953 | CLRQKTESELLEISGKLVQYNISLSTMIDGVVLPKAPEEIL |
| P23953 | EGKMNEETASLLLRRFHSELNISESMIPAVIEQYLRGVDDP |
| P23953 | EIFFVFGAPLLKEGASEEETNLSKMVMKFWANFARNGNPNG |
| Q04457 | FEKPVAPDPWEDVYPATQYRNDCTPHYRLVAQFSSYSGEDC |

| | |
|---|---|
| P23141 | PLGPLRFTPPQPAEPWSFVKNATSYPPMCTQDPKAGQLLSE |
| P12337 | PLGSLRFAPPQPAESWSHVKNTTSYPPMCSQDAVSGHMLSE |
| P12337 | EGKLDQKTATELLWKSYPIVNVSKELTPVATEKYLGGTDDP |
| O00748 | HPAMCLQDLTAVESEFLSQFNMTFPSDSMSEDCLYLSIYTP |
| O00748 | VALLPGLIASSADVISTVVANLSACDQVDSEALVGCLRGKS |
| P14943 | VALLPGLITSSSEVVSTVVANLSRCGQVDSETLVRCLRAKS |
| Q6UWW8 | EGVRDASTAPPMCLQDVESMNSSRFVLNGKQQIFSVSEDCL |
| Q8I034 | PLGPLRFKQPKPALPGNDFRNATSYPKLCFQDLEWLVSYQH |
| Q8I034 | KLEASEDCLYLNIYAPAHADNGSNLPVMVWFPGGAFKMGSA |
| Q8I034 | NNHECAFLLSTEFSEILGGSNRSLALYLVHTFLNIPTQYLH |
| Q8I034 | HRDAGAPVYFYEFQHPPQCLNDTRPAFVKADHSDEIRFVFG |
| P08171 | SDTDDPLLVQLPQGKLRGRDNGSYYSYESIPYAEPPTGDLR |
| P08171 | RFDIESYSELQRLFTDILFKNSTQESLDLHRKYGKSPAYAY |
| P08171 | PAYAYVYDNPAEKGIAQVLANRTDYDFGTVHGDDYFLIFEN |
| P08171 | DEQIISRNFINMLADFASSDNGSLKYGECDFKDNVGSEKFQ |
| O60883 | TSPNPGKDGGTPDSGQELRGNLTGAPGQRLQIQNPLYPVTE |
| P23776 | DPYVSGLQESYLDQAIGWARNNSLKVWVDLHGAAGSQNGFD |
| P23776 | LERSIDEHIKVACEWGTGVLNESHWTVCGEFAAALTDCTKW |
| P58738 | VLAALVAGGSCGPPKVPPGPNITTNYNGKWLTARATWYGQP |
| Q9UBQ6 | EIKSQGKSTMDSFTLIMQTYNRTDLLLKLLNHYQAVPNLHK |
| A0A1F4 | QAGFACLSNPCVFGVCIDGLNSSYSCYCIDGYTGIQCQTNW |
| A0A1F4 | ECWSSPCQNGGTCVDGVAYYNCTCPEGFSGSNCEENVDECM |
| A0A1F4 | PLLTGMLCECLMVGEESLDCNYTAPATQSPPRRTTTTSTMA |
| A0A1F4 | ETPVNTGHEITIRAELDFSRNYTHCNASLLVNDTLAMSGDQ |
| A0A1F4 | TGHEITIRAELDFSRNYTHCNASLLVNDTLAMSGDQPTWLK |
| A0A1F4 | IRAELDFSRNYTHCNASLLVNDTLAMSGDQPTWLKLLPPRL |
| A0A1F4 | YEIKVGRIGRQAWLSVDGKFNITGRSPGSGSRMDVLPILYL |
| A0A1F4 | YLYDGGDKLGAKRSQMVSYRNFTKKLMPPKPITTPSSHFVM |
| A0A1F4 | PIPFRGCVRGLVVSGTRIVLNETNIVESRNIRDCDGTACGG |
| A0A1F4 | DKRRGPSLYVRPREAIQVSLNFSTIEPDGLLLWSEHERSKF |
| A0A1F4 | LGLGLEAGHLKLASNLLGSTNDTVRAPASGFIADGAWHWTS |
| A0A1F4 | EGGRSLGSTTPRSTLAGRRKNSSKEPTISYEDVFYLGGFPN |
| P00488 | EYMGQLLEQASLHFFVTARINETRDVLAKQKSTVLTIPEII |
| P05160 | SQPTCRKEHETCLAPELYNGNYSTTQKTFKVKDKVQYECAT |
| P05160 | TSPPLIKHGVIISSTVDTYENGSSVEYRCFDHHFLEGSREA |
| Q07968 | SQPSCRKEQETCLAPELEHGNYSTTQRTFKVKDIVAYTCTA |
| Q07968 | ASPPVIRNGDIVSSAARTYENGSSVEYRCFDNHFLQGSQNV |
| Q5VUB5 | QGARPQPRVHFQRRALRLPENTSYSDLTAFLTAASSPSEVD |
| Q5VUB5 | TAASSPSEVDSFPYLRGLDGNGTGNSTRHDLTPVTAVSVHL |
| Q5VUB5 | SPSEVDSFPYLRGLDGNGTGNSTRHDLTPVTAVSVHLLSSN |
| A8MVW0 | QVYVSGELVPLARASVDVFGNRTLLAAGTTDSEGVATLPLS |

| | |
|---|---|
| A8MVW0 | TQQEMRAFPAFLGTEASSSGNGSWLELMPLTAVSVHLLTGN |
| A8MVW0 | NGSWLELMPLTAVSVHLLTGNGTEVPLSGPIHLSLPVPSET |
| A8MVW0 | TVGTSIPAWRFDPKSGLWVRNGTGVIRKEGRQLYWTFVSPQ |
| P00743 | EHYDPADLSPTESSLDLLGLNRTEPSAGEDGSQVVRIVGGR |
| P00742 | KPYDAADLDPTENPFDLLDFNQTQPERGDNNLTRIVGGQEC |
| P00742 | TENPFDLLDFNQTQPERGDNNLTRIVGGQECKDGECPWQAL |
| O88947 | TAPFPCGKITTGRRKRSVALNTSDSELDLEDALLDEDFLSP |
| O88947 | ALLDEDFLSPTENPIELLNLNETQPERSSDDLVRIVGGREC |
| P03951 | PTRWFTCVLKDSVTETLPRVNRTAAISGYSFKQCSHQISAC |
| P03951 | QISACNKDIYVDLDMKGINYNSSVAKSAQECQERCTDDVHC |
| P03951 | DVHCHFFTYATRQFPSLEHRNICLLKHTQTGTPTRITKLDK |
| P03951 | TPAQASCNEGKGKCYLKLSSNGSPTKILHGRGGISGYTLRL |
| P03951 | AHCFYGVESPKILRVYSGILNQSEIKEDTSFFGVQEIIIHD |
| P03951 | QYKMAESGYDIALLKLETTVNYTDSQRPICLPSKGDRNVIY |
| P00748 | ARTTLSGAPCQPWASEATYRNVTAEQARNWGLGGHAFCRNP |
| P00748 | CLQDRPAPEDLTVVLGQERRNHSCEPCQTLAVRSYRLHEAF |
| Q9XTW2 | LYDLTKNVDFDQLRQNECKKNITLSKFWEKSEQRNVPEDDN |
| Q9XTW2 | FHLDRVLGFRRAIPTVGRVLNMTTELFEKAEKKLKKTFFFS |
| Q8IXL6 | GWPNKHTLRILQDFSSDPSSNLSSHSLEKLPPAAEPAERAL |
| Q8IXL6 | FHLDRILDFRRVPPVAGRMVNMTKEIRDVTRDKKLWRTFFI |
| Q8IXL6 | FDFLMGNMDRHHYETFEKFGNETFIIHLDNGRGFGKYSHDE |
| Q28107 | KMFEKQHVLMFAVFDESKSWNQTSSLMYTVNGYVNGTMPDI |
| Q28107 | DESKSWNQTSSLMYTVNGYVNGTMPDITVCAHDHISWHLIG |
| Q28107 | EQNHHKISAITLVSATSTTANMTVSPEGRWTIASLIPRHFQ |
| Q28107 | DYAPIIPANMDKKYRSLHLDNFSNRIGKHYKKVVYKQYQDD |
| Q28107 | SRSYSIYPHGVTFSPYDNEVNSSSTSGSNTMIRAVRPGETY |
| Q28107 | RGIQRAADIEQQAVFAVFDENKSWYIEDNIYKFCENPEKVK |
| Q28107 | ENPEKVKRDDPKFYESNIMSNFTLPAINGYVPESIPILGFC |
| Q28107 | DADSDYQDELALILGLRSFRNSSLNQEKDELNLTALALEKD |
| Q28107 | LILGLRSFRNSSLNQEKDELNLTALALEKDSEFIPPSANRS |
| Q28107 | ELNLTALALEKDSEFIPPSANRSLDSNSSSRSHVSRLIAKN |
| Q28107 | LALEKDSEFIPPSANRSLDSNSSSRSHVSRLIAKNFAESLK |
| Q28107 | HKFSQTRFPAHKTRTRLSQDNSSSSRMGPWEDIPSDLLLLQ |
| Q28107 | GEWHLVSEKGSYEIIQDANENKTVNKLPNSPQNDSRTWGEN |
| Q28107 | EIIQDANENKTVNKLPNSPQNDSRTWGENIPFKNSHGKQSG |
| Q28107 | KPAYHVPLSPRSFHPLRGEVNASFSDRRHNHSLLLHASNET |
| Q28107 | PRSFHPLRGEVNASFSDRRHNHSLLLHASNETSLSIDLNQT |
| Q28107 | EVNASFSDRRHNHSLLLHASNETSLSIDLNQTFPSMNLSLA |
| Q28107 | RHNHSLLLHASNETSLSIDLNQTFPSMNLSLAASLPDHDQT |
| Q28107 | LHASNETSLSIDLNQTFPSMNLSLAASLPDHDQTSPNDTTS |
| Q28107 | FPSMNLSLAASLPDHDQTSPNDTTSQTSSPPDLYPTVSPEE |

| | |
|---|---|
| Q28107 | SLSPDLSQLDLKQTSPPLDLNQTSHTSESSQSLPLPEFGQT |
| Q28107 | QTFPNADIGQMPSPPPDSTLNNTFIPEEFNPLVVVGLSRDD |
| Q28107 | GEFEFVAYNDPYQTDLRTDINSSRNPDNIAAWYLRSNTGNR |
| Q28107 | GKTYEDDSPEWFKEDNAIQPNKTYTYVWHATTRSGPENPGS |
| Q28107 | NLGGSRDIHVVHFHGQTLLENGTQQHQLGVWPLLPGSFKTL |
| Q28107 | TTEFCVAYSLDRKNWRIFKGNSTRNVMYFGGNSDASTIKEN |
| P12259 | LRQFYVAAQGISWSYRPEPTNSSLNLSVTSFKKIVYREYEP |
| P12259 | YVAAQGISWSYRPEPTNSSLNLSVTSFKKIVYREYEPYFKK |
| P12259 | DESKSWSQSSSLMYTVNGYVNGTMPDITVCAHDHISWHLLG |
| P12259 | EQNHHKVSAITLVSATSTTANMTVGPEGKWIISSLTPKHLQ |
| P12259 | DYAPVIPANMDKKYRSQHLDNFSNQIGKHYKKVMYTQYEDE |
| P12259 | SRPYSIYPHGVTFSPYEDEVNSSFTSGRNNTMIRAVQPGET |
| P12259 | HGVTFSPYEDEVNSSFTSGRNNTMIRAVQPGETYTYKWNIL |
| P12259 | RGIQRAADIEQQAVFAVFDENKSWYLEDNINKFCENPDEVK |
| P12259 | DADYDYQNRLAAALGIRSFRNSSLNQEEEEFNLTALALENG |
| P12259 | AALGIRSFRNSSLNQEEEEFNLTALALENGTEFVSSNTDII |
| P12259 | RNSSLNQEEEEFNLTALALENGTEFVSSNTDIIVGSNYSSP |
| P12259 | LALENGTEFVSSNTDIIVGSNYSSPSNISKFTVNNLAEPQK |
| P12259 | TEFVSSNTDIIVGSNYSSPSNISKFTVNNLAEPQKAPSHQQ |
| P12259 | QQATTAGSPLRHLIGKNSVLNSSTAEHSSPYSEDPIEDPLQ |
| P12259 | PSRMRPWKDPPSDLLLLKQSNSSKILVGRWHLASEKGSYEI |
| P12259 | EIIQDTDEDTAVNNWLISPQNASRAWGESTPLANKPGKQSG |
| P12259 | EAYNTFSERRLKHSLVLHKSNETSLPTDLNQTLPSMDFGWI |
| P12259 | RLKHSLVLHKSNETSLPTDLNQTLPSMDFGWIASLPDHNQN |
| P12259 | NQTLPSMDFGWIASLPDHNQNSSNDTGQASCPPGLYQTVPP |
| P12259 | LPSMDFGWIASLPDHNQNSSNDTGQASCPPGLYQTVPPEEH |
| P12259 | DLDQIFYPSESSQSLLLQEFNESFPYPDLGQMPSPSSPTLN |
| P12259 | NESFPYPDLGQMPSPSSPTLNDTFLSKEFNPLVIVGLSKDG |
| P12259 | AEIDYVPYDDPYKTDVRTNINSSRDPDNIAAWYLRSNNGNR |
| P12259 | GKTYEDDSPEWFKEDNAVQPNSSYTYVWHATERSGPESPGS |
| P12259 | TTEFYVAYSSNQINWQIFKGNSTRNVMYFNGNSDASTIKEN |
| O88783 | DSGPTPDDPPCLTHIYYSYENLTQDFNSGLIGPLLICKKGT |
| O88783 | DESKSRSQSPSLMYTINGFVNKTMPDITVCAHDHVSWHLIG |
| O88783 | NYAPVIPANMDKIYRSQHLDNFSNQIGKHYKKVIYRQYEEE |
| O88783 | SSTEHRSSSYHENDMENPQSNITMVYLLPLGPKGSGNREQD |
| O88783 | IAANGEDTDVDKLTNSPQNQNITVPRGESTSHTNTTRKPSD |
| O88783 | TNSPQNQNITVPRGESTSHTNTTRKPSDLPTFSGVGHKSPH |
| O88783 | DPDQASYPPDSGQASSLPELNRTLPHPDLTHIPPPSPSPTL |
| O88783 | NMGGSRDIHVVHFHGQTLLDNRTKQHQLGVWPLLPGSFKTL |
| P08709 | VSCTPTVEYPCGKIPILEKRNASKPQGRIVGGKVCPKGECP |
| P08709 | VPRLMTQDCLQQSRKVGDSPNITEYMFCAGYSDGSKDSCKG |

| | |
|---|---|
| P22457 | VSCAPTVEYPCGKIPVLEKRNGSKPQGRIVGGHVCPKGECP |
| P22457 | VGPAWVVSAAHCFERLRSRGNLTAVLGEHDLSRVEGPEQER |
| P00451 | LGELPVDARFPPRVPKSFPFNTSVVYKKTLFVEFTDHLFNI |
| P00451 | DRDAASARAWPKMHTVNGYVNRSLPGLIGCHRKSVYWHVIG |
| P00451 | RGNQIMSDKRNVILFSVFDENRSWYLTENIQRFLPNPAGVQ |
| P00451 | IEPRSFSQNSRHPSTRQKQFNATTIPENDIEKTDPWFAHRT |
| P00451 | NDIEKTDPWFAHRTPMPKIQNVSSSDLLMLLRQSPTPHGLS |
| P00451 | LQEAKYETFSDDPSPGAIDSNNSLSEMTHFRPQLHHSGDMV |
| P00451 | STSNNLISTIPSDNLAAGTDNTSSLGPPSMPVHYDSQLDTT |
| P00451 | GKKSSPLTESGGPLSLSEENNDSKLLESGLMNSQESSWGKN |
| P00451 | NDSKLLESGLMNSQESSWGKNVSSTESGRLFKGKRAHGPAL |
| P00451 | PALLTKDNALFKVSISLLKTNKTSNNSATNRKTHIDGPSLL |
| P00451 | TKDNALFKVSISLLKTNKTSNNSATNRKTHIDGPSLLIENS |
| P00451 | SDTEFKKVTPLIHDRMLMDKNATALRLNHMSNKTTSSKNME |
| P00451 | IHDRMLMDKNATALRLNHMSNKTTSSKNMEMVQQKKEGPIP |
| P00451 | EMVFPSSRNLFLTNLDNLHENNTHNQEKKIQEEIEKKETLI |
| P00451 | NVEGSYDGAYAPVLQDFRSLNDSTNRTKKHTAHFSKKGEEE |
| P00451 | SYDGAYAPVLQDFRSLNDSTNRTKKHTAHFSKKGEEENLEG |
| P00451 | KKHTAHFSKKGEEENLEGLGNQTKQIVEKYACTTRISPNTS |
| P00451 | LGNQTKQIVEKYACTTRISPNTSQQNFVTQRSKRALKQFRL |
| P00451 | KVSSFPSIRPIYLTRVLFQDNSSHLPAASYRKKDSGVQESS |
| P00451 | RKKDSGVQESSHFLQGAKKNNLSLAILTLEMTGDQREVGSL |
| P00451 | ISYEEDQRQGAEPRKNFVKPNETKTYFWKVQHHMAPTKDEF |
| P00451 | ISQFIIMYSLDGKKWQTYRGNSTGTLMVFFGNVDSSGIKHN |
| P00741 | SHISKKLTRAETIFSNTNYENSSEAEIIWDNVTQSNQSFDE |
| P00741 | ETIFSNTNYENSSEAEIIWDNVTQSNQSFDEFSRVVGGEDA |
| P00741 | NTNYENSSEAEIIWDNVTQSNQSFDEFSRVVGGEDAERGQF |
| P00741 | KPEPTEQKRNVIRAIPYHSYNASINKYSHDIALLELDEPLE |
| P00740 | VSQTSKLTRAETVFPDVDYVNSTEAETILDNITQSTQSFND |
| P00740 | ETVFPDVDYVNSTEAETILDNITQSTQSFNDFTRVVGGEDA |
| O42807 | KEIITVFRGTGSDTNLQLDTNYTLTPFDTLPQCNDCEVHGG |
| Q96BQ1 | RMIMSPVKNNVGRGLNIALVNGTTGAVLGQKAFDMYSGDVM |
| P10674 | LRDDSELSQFYSLLESNQIANSTLSLRSCTIFVPTNEAFQR |
| P10674 | LDDINSLTEVTILAPSNEAWNSSNINNVLRDRNKMRQILNM |
| P10674 | NVDKIRQKNANLIAQVPTVNNNTFLYFNVRGEGSDTVITVE |
| P10674 | YFNVRGEGSDTVITVEGGGVNATVIQADVAQTNGYVHIIDH |
| P10674 | KLESDPMMSDTYKMGKFSHFNDQLNNTQRRFTYFVPRDKGW |
| P10674 | DPMMSDTYKMGKFSHFNDQLNNTQRRFTYFVPRDKGWQKTE |
| P34082 | CRPTVPEPSLVADLQWKDNRNNTILPKPNGRNQPPMYTETL |
| P34082 | PEIISLPTNLEAVEGKPFAANCTARGKPVPEISWIRDATQL |
| P34082 | VVDQKTKLNVLVRPQIYELYNVTGARTKEIAITCRAKGRPA |

| | |
|---|---|
| P34082 | PDFSHMKELPPVFSWEQRKANLSCLAMGIPNATIEWHWNGR |
| P34082 | PVFSWEQRKANLSCLAMGIPNATIEWHWNGRKIKDLYDTNL |
| P34082 | ILAYSVQYKEALNPDWSTAYNRSWSPDSPYIVEGLRPQTEY |
| Q14517 | DSDGSQRLEQTPLQFTHLEYNVTVQENSAAKTYVGHPVKMG |
| Q14517 | KEYKVKAIGGIDWDSHPFGYNLTLQAKDKGTPPQFSSVKVI |
| Q14517 | FHSLRITATDGENFATPLYINITVAASHKLVNLQCEETGVA |
| Q14517 | LPTGIQVKENQPVGSSVIFMNSTDLDTGFNGKLVYAVSGGN |
| Q14517 | TGMLKILSPLDRETTDKYTLNITVYDLGIPQKAAWRLLHVV |
| Q14517 | KLSGAVRIVQQLDFEKKQVYNLTVRAKDKGKPVSLSSTCYV |
| Q14517 | KGTGTIIVAKPLDAEQKSNYNLTVEATDGTTTILTQVFIKV |
| Q14517 | TVMVRDQDVPVKRNFARIVVNVSDTNDHAPWFTASSYKGRV |
| Q14517 | ETLPIYTLIIQGTNMAGLSTNTTVLVHLQDENDNAPVFMQA |
| Q14517 | HFTVQVHDMGTPRLFAEYAANVTVHVIDINDCPPVFAKPLY |
| Q14517 | PLYEASLLLPTYKGVKVITVNATDADSSAFSQLIYSITEGN |
| Q14517 | EGNIGEKFSMDYKTGALTVQNTTQLRSRYELTVRASDGRFA |
| Q14517 | ESKESHLKFTQDVYSAVVKENSTEAETLAVITAIGNPINEP |
| Q14517 | VRATDSDSEPNRGISYQMFGNHSKSHDHFHVDSSTGLISLL |
| Q14517 | TGIITLSNLHRHALKPFYSLNLSVSDGVFRSSTQVHVTVIG |
| Q14517 | TVEATDGGTPSLSDVATVNVNVTDINDNTPVFSQDTYTTVI |
| Q14517 | TKLLDRETISGYTLTVQASDNGSPPRVNTTTVNIDVSDVND |
| Q14517 | TISGYTLTVQASDNGSPPRVNTTTVNIDVSDVNDNAPVFSR |
| Q14517 | TTVNIDVSDVNDNAPVFSRGNYSVIIQENKPVGFSVLQLVV |
| Q14517 | SSTGGKLIAHKKLDIGQYLLNVSVTDGKFTTVADITVHIRQ |
| Q14517 | KFTTVADITVHIRQVTQEMLNHTIAIRFANLTPEEFVGDYW |
| Q14517 | LFVEKPGSAQISTKQLLHKINSSVTDIEEIIGVRILNVFQK |
| Q14517 | SGNPCLHGALCENTHGSYHCNCSHEYRGRHCEDAAPNQYVS |
| Q9NYQ8 | EKPLEGILSSSAWHFTHSHYNATIYENSSPKTYVESFEKMG |
| Q9NYQ8 | RSEMFAIHPTSGVVTVAGKLNVTWRGKHELQVLAVDRMRKI |
| Q9NYQ8 | VVTPPDSNDGTTYATVLVDANSSGAEVESVEVVGGDPGKHF |
| Q9NYQ8 | NEFSLVSVKDINWMEYLHGFNLSLQARSGSGPYFYSQIRGF |
| Q9NYQ8 | QASTVVVIDIVDCNNHAPLFNRSSYDGTLDENIPPGTSVLA |
| Q9NYQ8 | VSIFLQLRNLNDNQPMFEEVNCTGSIRQDWPVGKSIMTMSA |
| Q9NYQ8 | LEYFDLNHFSGVISLKRPFINLTAGQPTSYSLKITASDGKN |
| Q9NYQ8 | SYSLKITASDGKNYASPTTLNITVVKDPHFEVPVTCDKTGV |
| Q9NYQ8 | TGLLTVAAPLDYEATNFYILNVTVYDLGTPQKSSWKLLTVN |
| Q9NYQ8 | LMTGALILERELDFERRAGYNLSLWASDGGRPLARRTLCHV |
| Q9NYQ8 | SVLQLDAWDPDSSSKGKLTFNITSGNYMGFFMIHPVTGLLS |
| Q9NYQ8 | PVNHMVGVISVEGRPGLFWFNISGGDKDMDFDIEKTTGSIV |
| Q9NYQ8 | KTTGSIVIARPLDTRRRSNYNLTVEVTDGSRTIATQVHIFM |
| Q9NYQ8 | PIHPGMELLMVRASDEDSEVNYSIKTGNADEAVTIHPVTGS |
| Q9NYQ8 | KENLQDRKALVILGAQGNHLNDTLSYFLLNGTDMFHMVQSA |

| | |
|---|---|
| Q9NYQ8 | LVILGAQGNHLNDTLSYFLLNGTDMFHMVQSAGVLQTRGVA |
| Q9NYQ8 | ARDGGTPSLQSEEEVLVTVRNKSNPLFQSPYYKVRVPENIT |
| Q9NYQ8 | VRNKSNPLFQSPYYKVRVPENITLYTPILHTQARSPEGLRL |
| Q9NYQ8 | RDVSYQIVEDGSDVSKFFQINGSTGEMSTVQELDYEAQQHF |
| Q9NYQ8 | KVRAMDKGDPPLTGETLVVVNVSDINDNPPEFRQPQYEANV |
| Q9NYQ8 | VNVSDINDNPPEFRQPQYEANVSELATCGHLVLKVQAIDPD |
| Q9NYQ8 | DTSRLEYLILSGNQDRHFFINSSSGIISMFNLCKKHLDSSY |
| Q9NYQ8 | YNLRVGASDGVFRATVPVYINTTNANKYSPEFQQHLYEAEL |
| Q9NYQ8 | EKFSINPNGQIATLQKLDRENSTERVIAIKVMARDGGGRVA |
| Q9NYQ8 | DENDNPPQFKASEYTVSIQSNVSKDSPVIQVLAYDADEGQN |
| Q9NYQ8 | IEINPVTGVVKVKDSLVGLENQTLDFFIKAQDGGPPHWNSL |
| Q9NYQ8 | DVNDNAPRFFPSHCAVAVFDNTTVKTPVAVVFARDPDQGAN |
| Q9NYQ8 | VSGNEQGRFRLDARTGILYVNASLDFETSPKYFLSIECSRK |
| Q9NYQ8 | SIECSRKSSSSLSDVTTVMVNITDVNEHRPQFPQDPYSTRV |
| Q9NYQ8 | TDIAIQVADVNDNPPRFFQLNYSTTVQENSPIGSKVLQLIL |
| Q9NYQ8 | SDPDSPENGPPYSFRITKGNNGSAFRVTPDGWLVTAEGLSR |
| Q9NYQ8 | GAPDGKIIAAQGLPRGHYSFNVTVSDGTFTTTAGVHVYVWH |
| Q9NYQ8 | STARLSILTPRHHLQRSCSCNGTATRFSGQSYVRYRAPAAR |
| Q9NYQ8 | NWHIHFYLKTLQPQAILLFTNETASVSLKLASGVPQLEYHC |
| Q9NYQ8 | LKLASGVPQLEYHCLGGFYGNLSSQRHVNDHEWHSILVEEM |
| Q9NYQ8 | HSILVEEMDASIRLMVDSMGNTSLVVPENCRGLRPERHLLL |
| Q9NYQ8 | GLRPERHLLLGGLILLHSSSNVSQGFEGCLDAVVVNEEALD |
| Q9NYQ8 | GYVCKCPPQFSGKHCEQGRENCTFAPCLEGGTCILSPKGAS |
| Q56VR3 | VFCGGTILSPIYVLTAAHCINETETISVVVGEIDRSRAETG |
| P83370 | VFCGGTILSPIHVLTAAHCINQTKSVSVIVGEIDISRKETR |
| P81428 | VFCGGTILSPIHVLTAAHCINQTKSVSVIVGEIDISRKETR |
| O77469 | IADGYSCIKVCSTEDTECLGNHTREVLYQFRAVPSLKTIIS |
| P23142 | CATGISLANEQDRCATPHGDNASLEATFVKRCCHCCLLGRA |
| P23142 | RLAPNGRNCQDIDECVTGIHNCSINETCFNIQGGFRCLAFE |
| P23142 | NGRNCQDIDECVTGIHNCSINETCFNIQGGFRCLAFECPEN |
| P98095 | CHCPDAGGELICYQLPGCHGNFSDAEEGDPERHYEDPYSYD |
| P98095 | KSCMAGVLGAKEGETCGAEDNDSCGISLYKQCCDCCGLGLR |
| P98095 | TMTANGRSCKDVDECALGTHNCSEAETCHNIQGSFRCLRFE |
| P35555 | PRPPVEYLYPSREPPRVLPVNVTDYCQLVRYLCQNGRCIPT |
| P35555 | TIGSFKCRCDSGFALDSEERNCTDIDECRISPDLCGRGQCV |
| P35555 | CHNTEGSYRCECPPGHQLSPNISACIDINECELSAHLCPNG |
| P35555 | SCSPGWIGDGIKCTDLDECSNGTHMCSQHADCKNTMGSYRC |
| P35555 | LPGLFRCECEIGYELDRSGGNCTDVNECLDPTTCISGNCVN |
| P35555 | SCCCSLGKAWGTPCEMCPAVNTSEYKILCPGGEGFRPNPIT |
| P35555 | VNECETPGICGPGTCYNTVGNYTCICPPDYMQVNGGNNCMD |
| P35555 | GGNNCMDMRRSLCYRNYYADNQTCDGELLFNMTKKMCCCSY |

| | |
|---|---|
| P35555 | SLCYRNYYADNQTCDGELLFNMTKKMCCCSYNIGRAWNKPC |
| P35555 | TNDDQTMCLDINECERDACGNGTCRNTIGSFNCRCNHGFIL |
| P35555 | RMSYCYAKFEGGKCSSPKSRNHSKQECCCALKGEGWGDPCE |
| P35555 | ILAGNECVDTDECSVGNPCGNGTCKNVIGGFECTCEEGFEP |
| P35555 | ACYECKINGYPKRGRKRRSTNETDASNIEDQSETEANVSLA |
| P35555 | RRSTNETDASNIEDQSETEANVSLASWDVEKTAIFAFNISH |
| P35555 | TEANVSLASWDVEKTAIFAFNISHVSNKVRILELLPALTTL |
| P35556 | GGAGVGAGGQGPIITGLTILNQTIDICKHHANLCLNGRCIP |
| P35556 | TIGSFKCRCNSGFALDMEERNCTDIDECRISPDLCGSGICV |
| P35556 | SCREGWIGNGIKCIDLDECSNGTHQCSINAQCVNTPGSYRC |
| P35556 | LPGMFHCICDDGYELDRTGGNCTDIDECADPINCVNGLCVN |
| P35556 | SCCCSLGKAWGNPCETCPPVNSTEYYTLCPGGEGFRPNPIT |
| P35556 | DECFAHPGVCGPGTCYNTLGNYTCICPPEYMQVNGGHNCMD |
| P35556 | QVNGGHNCMDMRKSFCYRSYNGTTCENELPFNVTKRMCCCT |
| P35556 | RKSFCYRSYNGTTCENELPFNVTKRMCCCTYNVGKAWNKPC |
| P35556 | ASQDQTMCMDVDECERHPCGNGTCKNTVGSYNCLCYPGFEL |
| P35556 | RQSFCFTNFENGKCSVPKAFNTTKAKCCCSKMPGEGWGDPC |
| P35556 | DYTGVRCVDTDECSIGNPCGNGTCTNVIGSFECNCNEGFEP |
| P35556 | AVEQISLESVDMDSPVNMKFNLSHLGSKEHILELRPAIQPL |
| P24071 | VKIQCQAIREAYLTQLMIIKNSTYREIGRRLKFWNETDPEF |
| P24071 | QLMIIKNSTYREIGRRLKFWNETDPEFVIDHMDANKAGRYQ |
| P24071 | GLYGKPFLSADRGLVLMPGENISLTCSSAHIPFDRFSLAKE |
| P24071 | SLAKEGELSLPQHQSGEHPANFSLGPVDLNVSGIYRCYGWY |
| P24071 | LPQHQSGEHPANFSLGPVDLNVSGIYRCYGWYNRSPYLWSF |
| P24071 | FSLGPVDLNVSGIYRCYGWYNRSPYLWSFPSNALELVVTDS |
| P12319 | VPQKPKVSLNPPWNRIFKGENVTLTCNGNNFFEVSSTKWFH |
| P12319 | VTLTCNGNNFFEVSSTKWFHNGSLSEETNSSLNIVNAKFED |
| P12319 | NFFEVSSTKWFHNGSLSEETNSSLNIVNAKFEDSGEYKCQH |
| P12319 | NIVNAKFEDSGEYKCQHQQVNESEPVYLEVFSDWLLLQASA |
| P12319 | VYKVIYYKDGEALKYWYENHNISITNATVEDSGTYYCTGKV |
| P12319 | YYKDGEALKYWYENHNISITNATVEDSGTYYCTGKVWQLDY |
| P12319 | SGTYYCTGKVWQLDYESEPLNITVIKAPREKYWLQFFIPLL |
| P12318 | HNGNLIPTHTQPSYRFKANNNDSGEYTCQTGQTSLSDPVHL |
| P12318 | QNGKSQKFSHLDPTFSIPQANHSHSGDYHCTGNIGYTLFSS |
| P31995 | HNGNLIPTHTQPSYRFKANNNDSGEYTCQTGQTSLSDPVHL |
| P31995 | LVKVTFFQNGKSKKFSRSDPNFSIPQANHSHSGDYHCTGNI |
| P31995 | QNGKSKKFSRSDPNFSIPQANHSHSGDYHCTGNIGYTLYSS |
| P08637 | RVLEKDSVTLKCQGAYSPEDNSTQWFHNESLISSQASSYFI |
| P08637 | VTLKCQGAYSPEDNSTQWFHNESLISSQASSYFIDAATVDD |
| P08637 | SSYFIDAATVDDSGEYRCQTNLSTLSDPVQLEVHIGWLLLQ |
| P08637 | PKATLKDSGSYFCRGLFGSKNVSSETVNITITQGLAVSTIS |

| | |
|---|---|
| P08637 | SGSYFCRGLFGSKNVSSETVNITITQGLAVSTISSFFPPGY |
| Q9Y6R7 | LLISSLSESPASVSILSQADNTSKKVTVRPGESVMVNISAK |
| Q9Y6R7 | SQADNTSKKVTVRPGESVMVNISAKAEMIGSKIFQHAVVIH |
| Q9Y6R7 | NVAWGNGRVSVTRVITVQVANFTLRLEQRQWKVTVNGVDMK |
| Q9Y6R7 | NRGNPAVSYVRVVTVAALGTNISIHKDEIGKVRVNGVLTAL |
| Q9Y6R7 | NEHRGSQAVSYTRSVTLQIYNHSLTLSARWPRKLQVDGVFV |
| Q9Y6R7 | NVAWGNGRVSVTRVITVQVANFTLRLEQRQWKVTVNGVDMK |
| Q9Y6R7 | NVAWGNGRVSVTRVITVQVANFTLRLEQRQWKVTVNGVDMK |
| Q9Y6R7 | NRGNPAVSYVRVVTVAALGTNISIHKDEIGKVRVNGVLTAL |
| Q9Y6R7 | NEHRGSQAVSYTRSVTLQIYNHSLTLSARWPRKLQVDGVFV |
| P12314 | VTLHCEVLHLPGSSSTQWFLNGTATQTSTPSYRITSASVND |
| P12314 | LNGTATQTSTPSYRITSASVNDSGEYRCQRGLSGRSDPIQL |
| P12314 | VYNVLYYRNGKAFKFFHWNSNLTILKTNISHNGTYHCSGMG |
| P12314 | RNGKAFKFFHWNSNLTILKTNISHNGTYHCSGMGKHRYTSA |
| P12314 | AFKFFHWNSNLTILKTNISHNGTYHCSGMGKHRYTSAGISV |
| P12314 | RYTSAGISVTVKELFPAPVLNASVTSPLLEGNLVTLSCETK |
| P12314 | RPGLQLYFSFYMGSKTLRGRNTSSEYQILTARREDSGLYWC |
| O75636 | HQLTLQGNWELRVELEDFNGNRTFAHYATFRLLGEVDHYQL |
| P30440 | VFFAVANGNELLLDLSLTKVNATEPERTAMKKIQDCYVENG |
| P68512 | DGNLKQIDAGSGSVVGVNNLNETFVLIDNVFTKISGSLKHF |
| P38993 | SIAVLLFSMLSLAQAETHTFNWTTGWDYRNVDGLKSRPVIT |
| P38993 | WPDITVNKGDRVQIYLTNGMNNTNTSMHFHGLFQNGTASMD |
| P38993 | ITVNKGDRVQIYLTNGMNNTNTSMHFHGLFQNGTASMDGVP |
| P38993 | YLTNGMNNTNTSMHFHGLFQNGTASMDGVPFLTQCPIAPGS |
| P38993 | MDGVPFLTQCPIAPGSTMLYNFTVDYNVGTYWYHSHTDGQY |
| P38993 | SFMSVYNPTGAEPIPQNLIVNNTMNLTWEVQPDTTYLLRIV |
| P38993 | VYNPTGAEPIPQNLIVNNTMNLTWEVQPDTTYLLRIVNVGG |
| P38993 | FWIEDHEMTVVEIDGITTEKNVTDMLYITVAQRYTVLVHTK |
| P38993 | VTDMLYITVAQRYTVLVHTKNDTDKNFAIMQKFDDTMLDVI |
| P38993 | AIMQKFDDTMLDVIPSDLQLNATSYMVYNKTAALPTQNYVD |
| P38993 | TMLDVIPSDLQLNATSYMVYNKTAALPTQNYVDSIDNFLDD |
| P38993 | ITVDVVMDNLKNGVNYAFFNNITYTAPKVPTLMTVLSSGDQ |
| P38993 | TYTAPKVPTLMTVLSSGDQANNSEIYGSNTHTFILEKDEIV |
| P29695 | KRDVFVKCHSTPDSVENVRRNCSKCPILLPPNNPHVVDSVE |
| P29695 | RGQHKYEPEAYYLEFVIVEINCTAQEAHDDHHQCHPYTAGE |
| P29695 | HAHSHLIQQHIEKNSISPEHNITILNFVHPDDHTSTSHESH |
| P02771 | GTRTFQAITVTKLSQKFTKVNFTEIQKLVLDVAHVHEHCCR |
| Q5KQS4 | RGQHKYEPEAFYVEFAIVEVNCTAQEAHDDHHHCHPNTAGE |
| Q5KQS4 | HAHSHLIEHHIGKYSTSPGQNSTVECVAECPVAFVNKEVPT |
| Q5KQS1 | RGQHKYEPEAFYVEFAIVEVNCTAQEAHDDHHHCHPNTAGE |
| Q5KQS1 | HAHSHLIEHHIGKYSTSPGQNSTVECVAECPVAFVNKEVPT |

| | |
|---|---|
| P12763 | DIEIDTLETTCHVLDPTPLANCSVRQQTQHAVEGDCDIHVL |
| P12763 | PDSAEDVRKLCPDCPLLAPLNDSRVVHAVEVALATFNAESN |
| P12763 | NDSRVVHAVEVALATFNAESNGSYLQLVEISRAQFVPLPVS |
| P02765 | PDSAEDVRKVCQDCPLLAPLNDTRVVHAAKAALAAFNAQNN |
| P02765 | NDTRVVHAAKAALAAFNAQNNGSNFQLEEISRAQLVPLPPS |
| P29699 | EMEVDTLETTCHALDPTPLANCSVRQLTEHAVEGDCDFHIL |
| P29699 | PDSAEDVRKLCPRCPLLTPFNDTNVVHTVNTALAAFNTQNN |
| P29699 | NDTNVVHTVNTALAAFNTQNNGTYFKLVEISRAQNVPLPVS |
| Q58D62 | GATSPPQPAARPSSLLSLDCNSSYVLDIANDILQDINRDRK |
| Q58D62 | QCKAIFYINKEKRIFYLPAYNCTLRPVSQSAIIMTCPDCPS |
| Q58D62 | KIISVTCSFFNSQAPTPRGENATVNQRPANPSKTEELQQQN |
| Q9UGM5 | GAMSPPQLALNPSALLSRGCNDSDVLAVAGFALRDINKDRK |
| Q9UGM5 | QCKAIFYMNNPSRVLYLAAYNCTLRPVSKKKIYMTCPDCPS |
| Q9UGM5 | SSNHQVLEAATESLAKYNNENTSKQYSLFKVTRASSQWVVG |
| Q9QXC1 | SPPAPPLPQRPLSPLHPLGCNDSEVLAVAGFALQNINRDQK |
| Q9QXC1 | QCKAMFHINKPRRVLYLPAYNCTLRPVSKRKTHTTCPDCPS |
| P36386 | NRKLYCATKYHLQIHLNGKINGTLEKNSVFSILEITAVDVG |
| P05524 | RRKLYCATKYHLQLHPSGRVNGSLENSAYSILEITAVEVGV |
| P31371 | GILRRRQLYCRTGFHLEIFPNGTIQGTRKDHSRFGILEFIS |
| Q9MZ06 | SVLKTRVCRKKFPESNLKLVNSTLIRIKKPSQELMEPSPMD |
| P11362 | LRDDVQSINWLRDGVQLAESNRTRITGEEVEVQDSVPADSG |
| P11362 | GLYACVTSSPSGSDTTYFSVNVSDALPSSEDDDDDDDSSSE |
| P11362 | KVRYATWSIIMDSVVPSDKGNYTCIVENEYGSINHTYQLDV |
| P11362 | VVPSDKGNYTCIVENEYGSINHTYQLDVVERSPHRPILQAG |
| P11362 | QLDVVERSPHRPILQAGLPANKTVALGSNVEFMCKVYSDPQ |
| P11362 | MCKVYSDPQPHIQWLKHIEVNGSKIGPDNLPYVQILKTAGV |
| P11362 | GSKIGPDNLPYVQILKTAGVNTTDKEMEVLHLRNVSFEDAG |
| P11362 | ILKTAGVNTTDKEMEVLHLRNVSFEDAGEYTCLAGNSIGLS |
| P16092 | LRDDVQSINWLRDGVQLVESNRTRITGEEVEVRDSIPADSG |
| P16092 | GLYACVTSSPSGSDTTYFSVNVSDALPSSEDDDDDDDSSSE |
| P16092 | KVRYATWSIIMDSVVPSDKGNYTCIVENEYGSINHTYQLDV |
| P16092 | VVPSDKGNYTCIVENEYGSINHTYQLDVVERSPHRPILQAG |
| P16092 | QLDVVERSPHRPILQAGLPANKTVALGSNVEFMCKVYSDPQ |
| P16092 | MCKVYSDPQPHIQWLKHIEVNGSKIGPDNLPYVQILKTAGV |
| P16092 | GSKIGPDNLPYVQILKTAGVNTTDKEMEVLHLRNVSFEDAG |
| P16092 | ILKTAGVNTTDKEMEVLHLRNVSFEDAGEYTCLAGNSIGLS |
| Q03142 | LPEDAGRYLCLARGSMTVVHNLTLLMDDSLTSISNDEDPKT |
| Q03142 | LLDVLERSPHRPILQAGLPANTTAVVGSDVELLCKVYSDAQ |
| Q03142 | LCKVYSDAQPHIQWLKHVVINGSSFGADGFPYVQVLKTTDI |
| Q03142 | GSSFGADGFPYVQVLKTTDINSSEVEVLYLRNVSAEDAGEY |
| Q03142 | VQVLKTTDINSSEVEVLYLRNVSAEDAGEYTCLAGNSIGLS |

| | |
|---|---|
| Q14314 | NWYWLSSAVLATYGFLVVANNETEEIKDERAKDVCPVRLES |
| Q14314 | EKLNLVNMNNIENYVDSKVANLTFVVNSLDGKCSKCPSQEQ |
| Q14314 | DYYAIGKRSSETYRVTPDPKNSSFEVYCDMETMGGGWTVLQ |
| Q14314 | DMETMGGGWTVLQARLDGSTNFTRTWQDYKAGFGNLRREFW |
| Q14314 | YDQFYVANEFLKYRLHVGNYNGTAGDALRFNKHYNHDLKFF |
| Q03591 | EGDTVQIICNTGYRLQNNENNISCVERGWSTPPKCRSTDTS |
| Q03591 | YECRSPYEMFGDEEVMCLNGNWTEPPQCKDSTGKCGPPPPI |
| P36980 | EGDTVQIICNTGYRLQNNENNISCVERGWSTPPKCRSTISA |
| Q02985 | YFPYLENGYNQNYGRKFVQGNSTEVACHPGYGLPKAQTTVT |
| Q02985 | YILNKEIQYKCKPGYATADGNSSGSITCLQNGWSAQPICIN |
| Q02985 | NSSGSITCLQNGWSAQPICINSSEKCGPPPPISNGDTTSFL |
| Q02985 | KYYAKTGDTIEFMCKLGYNANTSILSFQAVCREGIVEYPRC |
| P02671 | LVTSKGDKELRTGKEKVTSGSTTTTRRSCSKTVTKTVIGPD |
| P02671 | ETSLGGWLLIQQRMDGSLNFNRTWQDYKRGFGSLNDEGEGE |
| P02676 | KYKGTAGNALIEGASQLVGENRTMTIHNSMFFSTYDRDNDG |
| P02675 | KYRGTAGNALMDGASQLMGENRTMTIHNGMFFSTYDRDNDG |
| P02678 | GQPENDYDTGDDBTAADPDSNNTAAALDVRRPLPSGTRVRR |
| P02679 | YQTKVDKDLQSLEDILHQVENKTSEVKQLIKAIQLTYNPDE |
| P02679 | DAFDGFDFGDDPSDKFFTSHNGMQFSTWDNDNDKFEGNCAE |
| P04115 | CEIENGNGWTVIQHRHDGSVNFTRDWVSYREGFGYLAPTLT |
| Q8TAL6 | SFFCHLCQGYFDGPLYPEMSNGTLHHYFVPDGDYEENDDPE |
| P07589 | TRGGNSNGALCHFPFLYNNHNYTDCTSEGRRDNMKWCGTTQ |
| P07589 | CVAYSQLRDQCIVDGITYNVNDTFHKRHEEGHMLNCTCFGQ |
| P07589 | GITYNVNDTFHKRHEEGHMLNCTCFGQGRGRWKCDPVDQCQ |
| P07589 | NLPETANSVTLSDLQPGVQYNITIYAVEENQESTPVFIQQE |
| P07589 | KPLTAQQATKLDAPTNLQFINETDTTVIVTWTPPRARIVGY |
| P07589 | VVHADQSSCTFENLSPGLEYNVSVYTVKDDKESVPISDTII |
| P07589 | TDLSFVDITDSSIGLRWTPLNSSTIIGYRITVVAAGEGIPI |
| P07589 | HVPRGDVDHHLYPHVVGLNPNASTGQEALSQTTISWTPFQE |
| P02751 | TRGGNSNGALCHFPFLYNNHNYTDCTSEGRRDNMKWCGTTQ |
| P02751 | CIAYSQLRDQCIVDDITYNVNDTFHKRHEEGHMLNCTCFGQ |
| P02751 | DITYNVNDTFHKRHEEGHMLNCTCFGQGRGRWKCDPVDQCQ |
| P02751 | NLPETANSVTLSDLQPGVQYNITIYAVEENQESTPVVIQQE |
| P02751 | KPLTAQQTTKLDAPTNLQFVNETDSTVLVRWTPPRAQITGY |
| P02751 | VVHADQSSCTFDNLSPGLEYNVSVYTVKDDKESVPISDTII |
| P02751 | HIPREDVDYHLYPHGPGLNPNASTGQEALSQTTISWAPFQD |
| P11276 | TRGGNSNGALCHFPFLYNNRNYTDCTSEGRRDNMKWCGTTQ |
| P11276 | CIPYSQLRDQCIVDDITYNVNDTFHKRHEEGHMLNCTCFGQ |
| P11276 | DITYNVNDTFHKRHEEGHMLNCTCFGQGRGRWKCDPIDQCQ |
| P11276 | NLPETANSVTLSDLQPGVQYNITIYAVEENQESTPVFIQQE |
| P11276 | QGRESNPLTAQQTTKLDAPTNLQFVNETDRTVLVTWTPPRA |

| | |
|---|---|
| P11276 | NPLTAQQTTKLDAPTNLQFVNETDRTVLVTWTPPRARIAGY |
| P11276 | VVHADQSSCTFENLNPGLEYNVSVYTVKDDKESAPISDTVV |
| P11276 | TDLSFVDITDSSIGLRWTPLNSSTIIGYRITVVAAGEGIPI |
| P11276 | HVPRGDVDYHLYPHVPGLNPNASTGQEALSQTTISWTPFQE |
| Q96AY3 | HIPRACPREVQMGDFVRYHYNGTFEDGKKFDSSYDRNTLVA |
| Q96AY3 | LRPPHCPRMVQDGDFVRYHYNGTLLDGTSFDTSYSKGGTYD |
| Q96AY3 | ELPPGCVRRAGAGDFMRYHYNGSLMDGTLFDSSYSRNHTYN |
| Q96AY3 | RYHYNGSLMDGTLFDSSYSRNHTYNTYIGQGYIIPGMDQGL |
| Q96AY3 | GACMGERRRITIPPHLAYGENGTGDKIPGSAVLIFNVHVID |
| Q96AY3 | FHNPADVVEIRTLSRPSETCNETTKLGDFVRYHYNCSLLDG |
| Q96AY3 | RPSETCNETTKLGDFVRYHYNCSLLDGTQLFTSHDYGAPQE |
| O95302 | FKPPSCPRTIQVSDFVRYHYNGTFLDGTLFDSSHNRMKTYD |
| O95302 | VVPENCERISQSGDFLRYHYNGTLLDGTLFDSSYSRNRTFD |
| O95302 | RYHYNGTLLDGTLFDSSYSRNRTFDTYIGQGYVIPGMDEGL |
| O95302 | YKPPDCSVLSKKGDYLKYHYNASLLDGTLLDSTWNLGKTYN |
| Q9Z247 | FKPPSCPRTIQVSDFVRYHYNGTFLDGTLFDSSHNRMKTYD |
| Q9Z247 | VVPENCERRSQSGDFLRYHYNGTLLDGTLFDSSYSRNHTFD |
| Q9Z247 | RYHYNGTLLDGTLFDSSYSRNHTFDTYIGQGYVIPGMDEGL |
| Q9Z247 | YKPPDCSVLSKKGDYLKYHYNASLLDGTLLDSTWNLGKTYN |
| Q9H9S5 | SARLVAAPVATANPARCLALNVSLREWTARYGAAPAAPRCD |
| Q9H9S5 | PRCDALDGDAVVLLRARDLFNLSAPLARPVGTSLFLQTALR |
| P27803 | TLIVFIAMVLVAAVAASVLINTSGFLQQKASTTGKESTEQV |
| P27803 | GKESTEQVASGLQVTGVTGVNSTDSENITHIVAYITPKAGS |
| P27803 | QVASGLQVTGVTGVNSTDSENITHIVAYITPKAGSSAIDLS |
| P27803 | DLSQAKLFVTYNGVSAVLKANESAIDGTSGTPDVLSATLLS |
| P27803 | ESAIDGTSGTPDVLSATLLSNTSATEYQVVSLQDFDGSVAK |
| P27802 | TLIVFIAMVLVAAVAASVLINTSGFLQQKASSTGTESTEQV |
| P27802 | GSCTAESPVIGKGDMAVITINCTNLDLAPRTRLNGYLQSEI |
| P27804 | TLIVFIAMVLVAAVAASVLINTSGFLQQKASTTGKESTEQV |
| P27804 | KESTEQVASGLQISQVMGMHNNSNINKTAIYISPNAGSSAI |
| P27804 | QVASGLQISQVMGMHNNSNINKTAIYISPNAGSSAIDLSQA |
| P27804 | DLSQAVIMLSDGSNKRVYKYNESSYKDLTNGGDIFDNANVE |
| P27804 | ESCTAANPVINKGDLVAITLNTTSFSTTPRTSITGTVQPEF |
| P27805 | TTVVTFSTDDRKMSLLYDSSNASNGGKVRLSTANGTSDIFK |
| P27805 | SLLYDSSNASNGGKVRLSTANGTSDIFKYDDVYAIGAWPFE |
| D4GWY0 | QQSVNKVTNRVDVVNAHGLVNKTGEERTVDQIFLTVRLAAG |
| D4GWY0 | SLEDTTVKYLSETTARTLTYNDTVTGSDTADPANLTTGNNF |
| D4GWY0 | EQSDRAEMVINTSTVEGDNTNGTATGQTVKLDITSRNGGTA |
| O43155 | LQELRVDENRIAVISDMAFQNLTSLERLIVDGNLLTNKGIA |
| O43155 | LERLDISNNQLRMLTQGVFDNLSNLKQLTARNNPWFCDCSI |
| O43155 | GRERVTPPISERIQLSIHFVNDTSIQVSWLSLFTVMAYKLT |

| | |
|---|---|
| O43155 | YRAVEDTICSEATTHASYLNNGSNTASSHEQTTSHSMGSPF |
| Q9NZU0 | LDGNLLNNHGLGDKVFFNLVNLTELSLVRNSLTAAPVNLPG |
| Q9NZU0 | VPPNAFSYLRQLYRLDMSNNNLSNLPQGIFDDLDNITQLIL |
| Q9NZU0 | LDMSNNNLSNLPQGIFDDLDNITQLILRNNPWYCGCKMKWV |
| Q9FL28 | SNDPLGVLSDWTIIGSLRHCNWTGITCDSTGHVVSVSLLEK |
| Q9FL28 | VVSVSLLEKQLEGVLSPAIANLTYLQVLDLTSNSFTGKIPA |
| Q9FL28 | DVPEEICKTSSLVLIGFDYNNLTGKIPECLGDLVHLQMFVA |
| Q9FL28 | FVAAGNHLTGSIPVSIGTLANLTDLDLSGNQLTGKIPRDFG |
| Q9FL28 | LQSLVLTENLLEGDIPAEIGNCSSLVQLELYDNQLTGKIPA |
| Q9FL28 | PISEEIGFLESLEVLTLHSNNFTGEFPQSITNLRNLTVLTV |
| Q9FL28 | LTLHSNNFTGEFPQSITNLRNLTVLTVGFNNISGELPADLG |
| Q9FL28 | EFPQSITNLRNLTVLTVGFNNISGELPADLGLLTNLRNLSA |
| Q9FL28 | GFNNISGELPADLGLLTNLRNLSAHDNLLTGPIPSSISNCT |
| Q9FL28 | LRNLSAHDNLLTGPIPSSISNCTGLKLLDLSHNQMTGEIPR |
| Q9FL28 | LLDLSHNQMTGEIPRGFGRMNLTFISIGRNHFTGEIPDDIF |
| Q9FL28 | LTFISIGRNHFTGEIPDDIFNCSNLETLSVADNNLTGTLKP |
| Q9FL28 | EIPDDIFNCSNLETLSVADNNLTGTLKPLIGKLQKLRILQV |
| Q9FL28 | LNILYLHSNGFTGRIPREMSNLTLLQGLRMYSNDLEGPIPE |
| Q9FL28 | PALFSKLESLTYLSLQGNKFNGSIPASLKSLSLLNTFDISD |
| Q9FL28 | LTGTIPGELLASLKNMQLYLNFSNNLLTGTIPKELGKLEMV |
| Q9FL28 | SIPRSLQACKNVFTLDFSQNNLSGHIPDEVFQGMDMIISLN |
| Q9FL28 | NLSGHIPDEVFQGMDMIISLNLSRNSFSGEIPQSFGNMTHL |
| Q9FL28 | IISLNLSRNSFSGEIPQSFGNMTHLVSLDLSSNNLTGEIPE |
| Q9FL28 | EIPQSFGNMTHLVSLDLSSNNLTGEIPESLANLSTLKHLKL |
| Q9FL28 | LVSLDLSSNNLTGEIPESLANLSTLKHLKLASNNLKGHVPE |
| Q9FL28 | KLASNNLKGHVPESGVFKNINASDLMGNTDLCGSKKPLKPC |
| P36888 | GTITNQDLPVIKCVLINHKNNDSSVGKSSSYPMVSESPEDL |
| P36888 | AVEVDVSASITLQVLVDAPGNISCLWVFKHSSLNCQPHFDL |
| P36888 | LKMTETQAGEYLLFIQSEATNYTILFTVSIRNTLLYTLRRP |
| P36888 | ELENKALEEGNYFEMSTYSTNRTMIRILFAFVSSVARNDTG |
| P36888 | YSTNRTMIRILFAFVSSVARNDTGYYTCSSSKHPSQSALVT |
| P36888 | SSSKHPSQSALVTIVEKGFINATNSSEDYEIDQYEEFCFSV |
| P36888 | KHPSQSALVTIVEKGFINATNSSEDYEIDQYEEFCFSVRFK |
| P36888 | FSDGYPLPSWTWKKCSDKSPNCTEEITEGVWNRKANRKVFG |
| P36888 | VWNRKANRKVFGQWVSSSTLNMSEAIKGFLVKCCAYNSLGT |
| P36888 | GTSCETILLNSPGPFPFIQDNISFYATIGVCLLFIVVLTLL |
| P16549 | LLKCIQFKTKVCSVTKHEDFNTTGQWDVVTLCEGKQESAVF |
| P13605 | MKYVYFQNNQISSIQEGVFDNATGLLWIALHGNQITSDKVG |
| P13605 | VGKKVFSKLRHLERLYLDHNNLTRIPSPLPRSLRELHLDHN |
| P13605 | LHLDHNQISRVPNNALEGLENLTALYLHHNEIQEVGSSMKG |
| P13605 | LYVRLSHNSLTNNGLASNTFNSSSLLELDLSYNQLQKIPPV |

| | |
|---|---|
| P13605 | QGNRINEFSISSFCTVVDVMNFSKLQVLRLDGNEIKRSAMP |
| Q04609 | GFFLLGFLFGWFIKSSNEATNITPKHNMKAFLDELKAENIK |
| Q04609 | HNMKAFLDELKAENIKKFLYNFTQIPHLAGTEQNFQLAKQI |
| Q04609 | KEFGLDSVELAHYDVLLSYPNKTHPNYISIINEDGNEIFNT |
| Q04609 | PNKTHPNYISIINEDGNEIFNTSLFEPPPPGYENVSDIVPP |
| Q04609 | EDGNEIFNTSLFEPPPPGYENVSDIVPPFSAFSPQGMPEGD |
| Q04609 | VYVNYARTEDFFKLERDMKINCSGKIVIARYGKVFRGNKVK |
| Q04609 | DSSWRGSLKVPYNVGPGFTGNFSTQKVKMHIHSTNEVTRIY |
| Q04609 | SRLLQERGVAYINADSSIEGNYTLRVDCTPLMYSLVHNLTK |
| Q04609 | IEGNYTLRVDCTPLMYSLVHNLTKELKSPDEGFEGKSLYES |
| Q04609 | HPQEMKTYSVSFDSLFSAVKNFTEIASKFSERLQDFDKSNP |
| P02702 | PEDSLHEQCSPWRKNACCSVNTSIEAHKDISYLYRFNWDHC |
| P02702 | SWWEDCRTSYTCKSNWHKGWNWTSGYNQCPVKAACHRFDFY |
| P14207 | IQDTCLYECSPNLGPWIQQVNQSWRKERFLDVPLCKEDCQR |
| P14207 | YFPTPAALCEGLWSHSYKVSNYSRGSGRCIQMWFDSAQGNP |
| P15328 | PEDKLHEQCRPWRKNACCSTNTSQEAHKDVSYLYRFNWNHC |
| P15328 | QWWEDCRTSYTCKSNWHKGWNWTSGFNKCAVGAACQPFHFY |
| P15328 | YFPTPTVLCNEIWTHSYKVSNYSRGSGRCIQMWFDPAQGNP |
| Q9P2B2 | VRVPTATLVRVVGTELVIPCNVSDYDGPSEQNFDWSFSSLG |
| Q9P2B2 | AVEVATVVIQPSVLRAAVPKNVSVAEGKELDLTCNITTDRA |
| Q9P2B2 | RAAVPKNVSVAEGKELDLTCNITTDRADDVRPEVTWSFSRM |
| Q9P2B2 | VSKENSGYYYCHVSLWAPGHNRSWHKVAEAVSSPAGVGVTW |
| Q9P2B2 | VSSPAGVGVTWLEPDYQVYLNASKVPGFADDPTELACRVVD |
| Q9P2B2 | TTEEDRGNYYCVVSAWTKQRNNSWVKSKDVFSKPVNIFWAL |
| Q9P2B2 | SPRYSVLIMAEKPVGDLSSPNETKYIISLDQDSVVKLENWT |
| Q9P2B2 | SPNETKYIISLDQDSVVKLENWTDASRVDGVVLEKVQEDEF |
| Q9P2B2 | AATSLSNPIEIDFQTSGPIFNASVHSDTPSVIRGDLIKLFC |
| Q9WV91 | VRVPAGTLVRVVGTELVIPCNVSDYDGPSEQNFDWSFSSSG |
| Q9WV91 | QLAVPRTVSVTEGKDLDLSCNITTDRVDDVRPEVTWYFKKT |
| Q9WV91 | VSKENSGYYLCLVALWAPGHNRSWHKVAEAMSAPSGVSVTW |
| Q9WV91 | MSAPSGVSVTWLEPEYQVYLNASKVPGFSDDPTELQCRVID |
| Q9WV91 | TTEEDRGNYYCVVSAWTRQRNNSWVKSKDVFSKPVNIFWAS |
| Q9WV91 | SPRYSVLITAEKPVGDLSSPNETKYIISLDQDSVVKLENWT |
| Q9WV91 | SPNETKYIISLDQDSVVKLENWTDASRVDGVVLEKVQEDEF |
| Q9WV91 | AATSLSNPIEIDFQTSGPTFNASVHSDTPSVTRGDLIKLFC |
| Q5SZK8 | LTPDMLAAEDAESPSDLLIFNLTSPFQPGQGYLVSTDDRSL |
| Q5SZK8 | DLTFTITQFPTHGHIMNQLINGTVLVESFTLDQIIESSSII |
| Q5SZK8 | HGLLQRRKPTGAFENITLGMNFTQDEVDRNLIQYVHLGQEG |
| Q5SZK8 | PDKLLKFTITQVPIHGHLLFNNTRPVMVFTKQDLNENLISY |
| Q5SZK8 | QFTQADIDDMKICYVLREGANATSDMFYFAVEDGGGNKLTY |
| Q9ZTA9 | SVISYHGGFLGLFPNANTLNNSSTSENQTTTKAASSNVVAV |

| | |
|---|---|
| Q9ZTA9 | GGFLGLFPNANTLNNSSTSENQTTTKAASSNVVAVEFDTYL |
| Q8K385 | EVTLSGPPFRGFLLEARDAENLSGPPIGSFTLIDSEESQLL |
| Q8K385 | TLEDMAWRLADGVIQCSFRRNITLPEAKNRFVLNESYYIFF |
| Q8K385 | IQCSFRRNITLPEAKNRFVLNESYYIFFAEGPSHDGRIFRH |
| Q8K385 | SHDGRIFRHSQQPLITYEKYNVTDTPKSVGGSRSSPLLKAH |
| P01226 | QFCFLFCCWKAVCCNSCELTNITIAVEKEECGFCISINTTW |
| P01226 | ELTNITIAVEKEECGFCISINTTWCAGYCYTRDLVYKDPAR |
| P01225 | QFFFLFCCWKAICCNSCELTNITIAIEKEECRFCISINTTW |
| P01225 | ELTNITIAIEKEECRFCISINTTWCAGYCYTRDLVYKDPAR |
| Q9PS36 | ELSNITIVLEKEECGACVSVNATWCSGYCYTKDANLMYPQK |
| P23945 | ESVILWLNKNGIQEIHNCAFNGTQLDELNLSDNNNLEELPN |
| P23945 | KNGIQEIHNCAFNGTQLDELNLSDNNNLEELPNDVFHGASG |
| P23945 | SHCCAFANWRRQISELHPICNKSILRQEVDYMTQARGQRSS |
| P23945 | RQEVDYMTQARGQRSSLAEDNESSYSRGFDMTYTEFDYDLC |
| P20395 | ESVILWLSKNGIEEIHNCAFNGTQLDELNLSDNNNLEELPN |
| P20395 | KNGIEEIHNCAFNGTQLDELNLSDNNNLEELPNDVFQGASG |
| P20395 | SHCCAFANLKRQISELHPICNKSILRQDIDDMTQIGDQRVS |
| Q12841 | RIIQWLEAEIIPDGWFSKGSNYSEILDKYFKNFDNGDSRLD |
| Q12841 | NFDNGDSRLDSSEFLKFVEQNETAINITTYPDQENNKLLRG |
| Q12841 | DSRLDSSEFLKFVEQNETAINITTYPDQENNKLLRGLCVDA |
| Q62356 | RLIQWLEAEIIPDGWFSKGSNYSEILDKYFKSFDNGDSHLD |
| Q62356 | SFDNGDSHLDSSEFLKFVEQNETAINITTYADQENNKLLRS |
| Q62356 | DSHLDSSEFLKFVEQNETAINITTYADQENNKLLRSLCVDA |
| O95633 | QTDVTRAECCASGNIDTAWSNLTHPGNKINLLGFLGLVHCL |
| O95633 | CRAAPCPVPSSPGQELCGNNNVTYISSCHMRQATCFLGRSI |
| P10669 | KKCRMNKKNKPRCVCAPDCSNITWKGPVCGLDGKTYRNECA |
| P10669 | SLCDELCPESKSEEPVCASDNATYASECAMKEAACSSGVLL |
| Q9DGI0 | FLKLNLLETECHVLDPTPVKNCTVRPQHNHAVEMDCDVKIM |
| Q9DGI0 | RGQHKYEPEAYYVEFAIVEVNCTAQELHDDHHHCHPNTAGE |
| Q9DGI0 | HAHSHLIQQHVEKDSISPEHNNTALNFVHPHNDTSTSHESH |
| Q9DGI0 | EKDSISPEHNNTALNFVHPHNDTSTSHESHEHLAEVPVAFV |
| Q9BTY2 | NAIDEGPKRDIVKELEVAIRNRTDLRFGLYYSLFEWFHPLF |
| Q9BTY2 | NYQPEVLWSDGDGGAPDQYWNSTGFLAWLYNESPVRGTVVT |
| Q9BTY2 | GSWLKVNGEAIYETHTWRSQNDTVTPDVWYTSKPKEKLVYA |
| P04066 | SYKPDLIWSDGEWECPDTYWNSTNFLSWLYNDSPVKDEVVV |
| P04066 | WLYNDSPVKDEVVVNDRWGQNCSCHHGGYYNCEDKFKPQSL |
| P04066 | LSINGEAIYASKPWRVQWEKNTTSVWYTSKGSAVYAIFLHW |
| P23188 | TSASAPLAAGIIALTLEANKNLTWRDMQHLVVQTSKPAHLN |
| P23188 | VSHSYGYGLLDAGAMVALAQNWTTVAPQRKCIVEILVEPKD |
| P23188 | FMTTHSWDEDPAGEWVLEIENTSEANNYGTLTKFTLVLYGT |
| P35936 | LLPNMPKDKEACAKAPLEAYNRTLTTLLTPLGDSIRRIQES |

| | |
|---|---|
| P35936 | GLSQLAVAVGKMQQFVNDQFNKTAQELDCIKITQQVGVELN |
| P35936 | YCTRIVTFPMSPGIYSCLSGNTSACMYSKTEGALTTPYMTL |
| P35936 | LSLDGITLRLSGEFDATYQKNISIQDSQVIVTGNLDISTEL |
| P35936 | QDSQVIVTGNLDISTELGNVNNSISNALDKLEESNSKLDKV |
| P04855 | RLLIPLRDALDLQEALITVTNDTTQNAGAPQSRFFGAVIGT |
| P04855 | FGTIGEKSLTLQALSSLYSANITEIMTTIKTGQSNIYDVIY |
| P04855 | VNGVELYANRRGHDATWGVQNLTVGPAIAIRPIDISLNLAD |
| Q495W5 | HEESPLNNFLLSHGPGIRLFNLTSTFSRHSDYPLSLQWLPG |
| Q495W5 | SHMDCPVPTPGFGNVEEIPENDSWKEMWLQDYWQGLDQGEA |
| Q8IWF2 | RDMVRYLGDFADTLGLRVQYNTTIAHVTLDKDRQAWNGHYF |
| Q9NQR9 | KWILFGHRPYWWVQETQIYPNHSSPCLEQFPTTCETGPGSP |
| P35575 | KWILFGQRPYWWVLDTDYYSNTSVPLIKQFPVTCETGPGSP |
| O95479 | GRIFYFSVPPFAYEDIARNINSSCRPGPGAWLRVVLEKPFG |
| O95479 | DVLQNHLTEVLTLVAMELPHNVSSAEAVLRHKLQVFQALRG |
| O95479 | CAEEDQGAQIYAREISALVANSSFDLVLLGMGADGHTASLF |
| P56201 | GRMFYFSVPPFAYADIARNINSSCRPGPGAWLRVVLEKPFG |
| P56201 | DTLQNHLTEILTLVAMELPANVSCSEAVLRHKLQAFRALRR |
| P39012 | FRESEWNILRGYRSQIKEMVNMTSMERNNLMGSWLQEFGTK |
| Q9UBS5 | LLLLAPLFLRPPGAGGAQTPNATSEGCQIIHPPWEGGIRYR |
| Q9UBS5 | EYVCRGEREVVGPKVRKCLANGSWTDMDTPSRCVRICSKSY |
| Q9UBS5 | VWFLIGWYADNWFKIYDPSINCTVDEMTEAVEGHITTEIVM |
| Q9UBS5 | EGHITTEIVMLNPANTRSISNMTSQEFVEKLTKRLKRHPEE |
| Q9UBS5 | GGFQEAPLAYDAIWALALALNKTSGGGGRSGVRLEDFNYNN |
| Q9UBS5 | NKTSGGGGRSGVRLEDFNYNNQTITDQIYRAMNSSSFEGVS |
| Q9UBS5 | RLEDFNYNNQTITDQIYRAMNSSSFEGVSGHVVFDASGSRM |
| O75899 | KGSIGRGVLPAVELAIEQIRNESLLRPYFLDLRLYDTECDN |
| O75899 | QWIIPGWYEPSWWEQVHTEANSSRCLRKNLLAAMEGYIGVD |
| O75899 | LQRAMETLHASSRHQRIQDFNYTDHTLGRIILNAMNETNFF |
| O75899 | RIQDFNYTDHTLGRIILNAMNETNFFGVTGQVVFRNGERMG |
| O75899 | DSREVKVGEYNAVADTLEIINDTIRFQGSEPPKDKTIILEQ |
| P54818 | NSNVGAGCWSRILNQNYINGNMTSTIAWNLVASYYEELPYG |
| P54818 | KTVGHLEKGGSYVALTDGLGNLTIIIETMSHQHSMCIRPYL |
| P54818 | IIETMSHQHSMCIRPYLPYYNVSHQLATFTLKGSLREIQEL |
| P54818 | ITWAADASSTISVIGDHHWTNMTVQCDVYIETPRSGGVFIA |
| P54818 | VNKGGILIRSATGVFFWIFANGSYRVTADLGGWITYASGHA |
| P54818 | AKRWYTLTLGIKGYFAFGMLNGTILWKNVRVKYPGHGWAAI |
| P34059 | WEMVGRYYEEFPINLKTGEANLTQIYLQEALDFIKRQARHH |
| P34059 | WTWTNSWENFRQGIDFCPGQNVSGVTTHNLEDHTKLPLIFH |
| O08912 | MKEMFKINQFNLMASEMIALNRSLPDVRLEGCKTKVYPDNL |
| O45293 | DPYENLEGWLDLKPLTERKCNHTLKENLTEAESKKSEWGIK |
| O45293 | EGWLDLKPLTERKCNHTLKENLTEAESKKSEWGIKSFAFDA |

| | |
|---|---|
| Q8BHN3 | HLIHEVTKVLLVLELQGLQKNMTRIRIDELEPRRPRYRVPD |
| Q14697 | HLIHEVTKVLLVLELQGLQKNMTRFRIDELEPRRPRYRVPD |
| P83691 | HQTTPAGWPSDINNLAWKLYNYTLDSMNRFADAGIQVDIVS |
| P83692 | HQTMPAGWPSDIDNLSWKLYNYTLDAANKLQNAGIQPTIVS |
| Q14393 | VMKIAVAGDLFQPERGLYHLNLTVGGIPFHEKDLVQPINPR |
| P28472 | FSAPVLVAVVCCAQSVNDPGNMSFVKETVDKLLKGYDIRLR |
| P28472 | TMYFQQYWRDKRLAYSGIPLNLTLDNRVADQLWVPDTYFLN |
| P28472 | RITTTAACMMDLRRYPLDEQNCTLEIESYGYTTDDIEFYWR |
| P03188 | HGDLFRFSSDIQCPSFGTRENHTEGLLMVFKDNIIPYSFKV |
| P03188 | NAVKMTKDGLTRVYVDRDGVNITVNLKPTGGLANGVRRYAS |
| P03188 | QGERRAFLDKGTYTLSWKLENRTAYCPLQHWQTFDSTIATE |
| P03188 | TETGKSIHFVTDEGTSSFVTNTTVGIELPDAFKCIEEQVNK |
| P03188 | TNTTVGIELPDAFKCIEEQVNKTMHEKYEAVQDRYTKGQEA |
| P03188 | SGGLLLAWLPLTPRSLATVKNLTELTTPTSSPPSSPSPPAP |
| P03188 | SAARGSTPAAVLRRRRRDAGNATTPVPPTAPGKSLGTLNNP |
| P03188 | RVPGSETMCYSRPLVSFSFINDTKTYEGQLGTDNEIFLTKK |
| P03188 | HHFKTIELDGIATLQTFISLNTSLIENIDFASLELYSRDEQ |
| P01865 | WFVNNVEVHTAQTQTHREDYNSTLRVVSALPIQHQDWMSGK |
| P01864 | WFVNNVEVHTAQTQTHREDYNSTLRVVSALPIQHQDWMSGK |
| P01863 | WFVNNVEVHTAQTQTHREDYNSTLRVVSALPIQHQDWMSGK |
| Q09324 | PEFFSVRHLELAGDDPYSNVNCTKILQGDPEEIQKVKLEIL |
| Q09324 | LEILTVQFKKRPRRTPHDYINMTRDCASFIRTRKYIVEPLT |
| P91406 | IIPDEQSGKYAGHQWLAYAGNGSASADVVYINHGTANDFKN |
| P91406 | ILKRLSGRPAPSDWQGFVGGNLTYKLGPGFVNGEKLSINVH |
| P91406 | DPNSGTAVLAEVARAMMQTINETSWKPARTIVFNAWDAEEF |
| P91406 | FVNILQKRAVVYINMDCIQGNISLHVDTVPILEHAVIEASK |
| P91406 | VPGGGSDHAPFLNFAGVPVINFTFKNYTTWDTYPLYHTMYE |
| P91406 | SDHAPFLNFAGVPVINFTFKNYTTWDTYPLYHTMYETPFSN |
| P91406 | PLYHTMYETPFSNIHLLDTDNLSVHKAIGQYWAELAKTFAD |
| P91406 | IGQYWAELAKTFADDVILPMNTTHFASVMLKTYLPQLKTTI |
| P91406 | FASVMLKTYLPQLKTTISGINVSRSDFEDIRTQYALLSKSA |
| P39905 | KGRRGQRGKNRGCVLTAIHLNVTDLGLGYETKEELIFRYCS |
| P39905 | FRYCSGSCDAAETTYDKILKNLSRNRRLVSDKVGQACCRPI |
| P57083 | PSEAPQIVRGASEDVRKQPYNLTIAWFRMGGNCAIPITVME |
| P57083 | RMGGNCAIPITVMEYTECSYNKSLGACPIRTQPRWNYYDSF |
| P57083 | HGPKAPYTSTLLPPELSETPNATQPELAPEDPEDSALLEDP |
| O60609 | LPSEEPSVPADCLEAAQQLRNSSLIGCMCHRRMKNQVACLD |
| O60609 | NYELDVSPYEDTVTSKPWKMNLSKLNMLKPDSDLCLKFAML |
| O60609 | LRAYLGLIGTAMTPNFVSNVNTSVALSCTCRGSGNLQEECE |
| Q92820 | PGGSVDLRRSDYAKVAKIFYNLSIQSFDDGDYFPVWGTCLG |
| Q92820 | LISGECLLTATDTVDVAMPLNFTGGQLHSRMFQNFPTELLL |

| | |
|---|---|
| Q92820 | LSLAVEPLTANFHKWSLSVKNFTMNEKLKKFFNVLTTNTDG |
| Q9Z0L8 | AEYEELFRSINGVLLPGGGANLTDSGYSRVAKIFFSKALES |
| Q9Z0L8 | LVSGENLLTSTDTKSKKLPLNFTEGARKSRMFKHFPTELLD |
| Q9Z0L8 | RKSRMFKHFPTELLDSLALENLTANFHKWSLSVKNFTENEK |
| Q9Z0L8 | DSLALENLTANFHKWSLSVKNFTENEKLKKFFNILTTNTDG |
| P19440 | VGLMNAHSMGIGGGLFLTIYNSTTRKAEVINAREVAPRLAF |
| P19440 | KAEVINAREVAPRLAFATMFNSSEQSQKGGLSVAVPGEIRG |
| P19440 | LPQLADTYETLAIEGAQAFYNGSLTAQIVKDIQAAGGIVTA |
| P19440 | GIVTAEDLNNYRAELIEHPLNISLGDVVLYMPSAPLSGPVL |
| P19440 | PSAPLSGPVLALILNILKGYNFSRESVESPEQKGLTYHRIV |
| P19440 | YAKRTLLGDPKFVDVTEVVRNMTSEFFAAQLRAQISDDTTH |
| P19440 | FGYDVKRAVEEPRLHNQLLPNVTTVERNIDQAVTAALETRH |
| Q60928 | MGLMNAHSMGIGGGLFFTIYNSTTGKVEVINAREVAPRLAN |
| Q60928 | NSTTGKVEVINAREVAPRLANTTMFNNSKDSEEGGLSVAVP |
| Q60928 | KVEVINAREVAPRLANTTMFNNSKDSEEGGLSVAVPGEIRG |
| Q60928 | VEVINAREVAPRLANTTMFNNSKDSEEGGLSVAVPGEIRGY |
| Q60928 | MPKLADTLQILAQEGAKAFYNGSLTAQIVKDIQEAGGIMTV |
| Q60928 | YAKRTMLGDPKFVDVSQVIRNMSSEFYATQLRARITDETTH |
| Q60928 | FGYDVKRAVEEPRLHNQLLPNTTTVEKDIDQVVTAGLKIRH |
| P36268 | LPRLADTYEMLAIEGAQAFYNGSLMAQIVKDIQAAGGIVTA |
| P36268 | FGYDVKRAVEEPRLHNKLLPNVTTVERNIDQAVTAALETRH |
| P36269 | TSVVNPQSMGLGGGVIFTIYNVTTGKVEVINARETVPASHA |
| P36269 | LHNSILRPSLQASTLRQLFFNGTEPLRPQDPLPWPALATTL |
| P36269 | PPPPAGGAILSFILNVLRGFNFSTESMARPEGRVNVYHHLV |
| P36269 | KFAKGQRWRLGDPRSHPKLQNASRDLLGETLAQLIRQQIDG |
| P36269 | AAIAAPILHVNSKGCVEYEPNFSQEVQRGLQDRGQNQTQRP |
| P36269 | VEYEPNFSQEVQRGLQDRGQNQTQRPFFLNVVQAVSQEGAC |
| P48546 | YRRECQETLAAAEPPSGLACNGSFDMYVCWDYAAPNATARA |
| P48546 | SGLACNGSFDMYVCWDYAAPNATARASCPWYLPWHHHVAAG |
| Q68CQ7 | IRKLSMKASTCSFNPGVFVANLTEWKRQNITNQLEKWMKLN |
| Q68CQ7 | STCSFNPGVFVANLTEWKRQNITNQLEKWMKLNVEEGLYSR |
| P28316 | LYKHMFENYPSMREAFKDRENYTAEDVQKDPFFVKQGQRIL |
| P25974 | RAELSEDDVFVIPAAYPFVVNATSNLNFLAFGINAENNQRN |
| O94923 | PQGYFYPIQIAQYGLSHYSKNLTEKPPHIEVYETAEDRDKN |
| O16580 | FSGRVYSYNDVANDYSMQNFNLTKEDFQWKIPYIKNAQKYN |
| P81447 | QPQNQNPKLPLSILKEKQLRNATLGSEETTEHAPSDASTTE |
| P80195 | QPQSQNPKLPLSILKEKHLRNATLGSEETTEHTPSDASTTE |
| P04062 | SGARPCIPKSFGYSSVVCVCNATYCDSFDPPTFPALGTFSR |
| P04062 | RYESTRSGRRMELSMGPIQANHTGTGLLLTLQPEQKFQKVK |
| P04062 | CDFSIRTYTYADTPDDFQLHNFSLPEEDTKLKIPLIHRALQ |
| P04062 | GFTPEHQRDFIARDLGPTLANSTHHNVRLLMLDDQRLLLPH |

| | |
|---|---|
| P04062 | NDLDAVALMHPDGSAVVVVLNRSSKDVPLTIKDPAVGFLET |
| P17439 | YGAQPCIPKSFGYSSVVCVCNASYCDSLDPVTLPALGTFSR |
| P17439 | RYESTRRGRRMELSVGAIQANRTGTGLLLTLQPEKKFQKVK |
| P17439 | CDFSIRVYTYADTPNDFQLSNFSLPEEDTKLKIPLIHQALK |
| P17439 | GFTPEHQRDFISRDLGPALANSSHDVKLLMLDDQRLLLPRW |
| P17439 | TDLETVALLRPDGSAVVVVLNRSSEDVPLTISDPDLGFLET |
| P37036 | CFSRAYPTPARSKKTMLVPKNITSZATCCVAKAFTKATVMG |
| P01217 | CFSRAYPTPARSKKTMLVPKNITSEATCCVAKAFTKATVMG |
| P01215 | CFSRAYPTPLRSKKTMLVQKNVTSESTCCVAKSYNRVTVMG |
| P01220 | CFSRAYPTPARSRKTMLVPKNITSESTCCVAKAFIRVTVMG |
| P80051 | CYSRAYPTPMRSKKTMLVPKNITSEAKCCVAKTQYRVTVMD |
| P25329 | CFSRAYPTPARSKKTMLVPKNITSZATCCVAKAFTKATVMG |
| P01219 | CFSRAYPTPARSKKTMLVPKNITSEATCCVAKAFTKATVMG |
| P07474 | CFSRAYPTPARSKKTMLVPKNITSEATCCVAKAFTKATVMG |
| P01218 | CFSRAYPTPARSKKTMLVPKNITSEATCCVAKAFTKATVMG |
| P37204 | CFSRAFPTPLKAMKTMTIPKNITSEATCCVAKHSYETEVAG |
| Q9JHJ3 | EVISGWPNPQNLLHIRAVGSNSTLHYVWSSLGPPAVVLVAT |
| Q9JHJ3 | STLHYVWSSLGPPAVVLVATNTTQSVLSVNWSLLLSPDPAG |
| Q9JHJ3 | SSIQFSSALVFTRLLEFDSTNASEGAQPPGKPYPPYSLAKF |
| Q9JHJ3 | GAQPPGKPYPPYSLAKFSWNNITNSLDLANLSADFQGRPVD |
| Q9JHJ3 | ANLSADFQGRPVDDPTGAFANGSLTFKVQAFSRSGRPAQPP |
| Q9JHJ3 | LHTADVCQLEVALVGASPRGNHSLFGLEVATLGQGPDCPSV |
| P43220 | RRQCQRSLTEDPPPATDLFCNRTFDEYACWPDGEPGSFVNV |
| P43220 | CNRTFDEYACWPDGEPGSFVNVSCPWYLPWASSVPQGHVYR |
| P43220 | VPQGHVYRFCTAEGLWLQKDNSSLPWRDLSECEESKRGERS |
| P02724 | VAMHTSTSSSVTKSYISSQTNDTHKRDTYAATPRAHEVSEI |
| P02725 | NATVTAGKPSATSPGVMTIKNTTAVVQKETGVPESYHQDFS |
| Q10914 | IGETSKKFPLRAFVASTDIDNDTAHAIIEMLRLAEMTFNAL |
| Q10914 | FKEFLNEFHRRISTQHTLKSNDSSEEIDEPIPVNVIVDLEG |
| Q10914 | VFSNFDVDETDLSGFHFSLINITIFRIFDKNNKKFLKTRAE |
| Q10914 | DKDGTLTGRIQFDKVTGKRTNFSAEIVEIKPGVNSLNSIWE |
| Q10914 | LKGNDRFEGYCIDLLNLLAKNITGFEYDVFISDGNKYGSRQ |
| Q10914 | NKYGSRQADGSWDGMIGYLLNETADVAVAPLTITQERERAV |
| Q10914 | WNFMVSTTQKQIELEKQSITNSTSNRIFVSSYADGIEKVRT |
| Q10914 | WYDRGQCDTGTSDGGTSSSLNLSKVAGIFYILLAGMVLSMC |
| P47871 | AQVMDFLFEKWKLYGDQCHHNLSLLPPPTELVCNRTFDKYS |
| P47871 | YGDQCHHNLSLLPPPTELVCNRTFDKYSCWPDTPANTTANI |
| P47871 | TELVCNRTFDKYSCWPDTPANTTANISCPWYLPWHHKVQHR |
| P47871 | CNRTFDKYSCWPDTPANTTANISCPWYLPWHHKVQHRFVFK |
| O45947 | ALSLYKANGYNAYISDMISLNRSIKDIRHKECKNMMYSAKL |
| O45947 | MMYSAKLPTVSVIFPFHEEHNSTLLRSVYSVINRSPPELLK |

| | |
|---|---|
| Q86SR1 | VDQAYRENGFNIYVSDKISLNRSLPDIRHPNCNSKRYLETL |
| Q86SR1 | SLPDIRHPNCNSKRYLETLPNTSIIIPFHNEGWSSLLRTVH |
| Q8MVS5 | FYSHSLRSSIRSAGWRIDEGNATPRAELSYQARVTVGCTPN |
| Q8MVS5 | NATPRAELSYQARVTVGCTPNASITTGESPAAPKPPSDPEQ |
| Q8MVS5 | SHIEVNQQWLEPLLRLIKSENATLAVPVIDLINADTFEYTP |
| Q17328 | QEILNALLKNYDMRVRPPPANSSTEGAVNVRVNIMIRMLSK |
| Q17328 | STPVQLKPGVGSDLPNFILKNYTTNADCTSHTNTGSYGCLR |
| G5EBR3 | HSPLQLKVGLSSSLPSFQLTNTSTTYCTSVTNTGIYSCLRT |
| P03524 | IDIHHLSCPNNLVVEDEGCTNLSGFSYMELKVGYILAIKMN |
| P03524 | LCGVLGLRLMDGTWVAMQTSNETKWCPPDQLVNLHDFRSDE |
| P03524 | RRLSHLRKLVPGFGKAYTIFNKTLMEADAHYKSVRTWNEIL |
| Q3T906 | KSFQNRLCLPMPIDVVYTWVNGTDLELLKELQQVREQMEEE |
| Q3T906 | QQVREQMEEEQKAMREILGKNTTEPTKKSEKQLECLLTHCI |
| Q3T906 | CLLTHCIKVPMLVLDPALPANITLKDLPSLYPSFHSASDIF |
| Q3T906 | PSFHSASDIFNVAKPKNPSTNVSVVVFDSTKDVEDAHSGLL |
| Q3T906 | LSGFPPTFKETNQLKTKLPENLSSKVKLLQLYSEASVALLK |
| Q3T906 | KWKTIHLIMHSGMNATTIHFNLTFQNTNDEEFKMQITVEVD |
| Q3T906 | KRHDVNSTRRAQEEVKIPLVNISLLPKDAQLSLNTLDLQLE |
| Q3T906 | LSLNTLDLQLEHGDITLKGYNLSKSALLRSFLMNSQHAKIK |
| Q3T906 | DLETTARFRVETHTQKTIGGNVTKEKPPSLIVPLESQMTKE |
| Q3T906 | EDMQFAFSYFYYLMSAVQPLNISQVFDEVDTDQSGVLSDRE |
| Q3T906 | NKYRFEIMGEEEIAFKMIRTNVSHVVGQLDDIRKNPRKFVC |
| Q6S5C2 | AGKCFSLVESTYKYEFCPFHNVTQHEQTFRWNAYSGILGIW |
| Q6S5C2 | TFRWNAYSGILGIWHEWEIINNTFKGMWMTDGDSCHSRSRQ |
| P15586 | CCPSRASILTGKYPHNHHVVNNTLEGNCSSKSWQKIQEPNT |
| P15586 | SILTGKYPHNHHVVNNTLEGNCSSKSWQKIQEPNTFPAILR |
| P15586 | EHVPLGWSYWYALEKNSKYYNYTLSINGKARKHGENYSVDY |
| P15586 | NSKYYNYTLSINGKARKHGENYSVDYLTDVLANVSLDFLDY |
| P15586 | GKARKHGENYSVDYLTDVLANVSLDFLDYKSNFEPFFMMIA |
| P15586 | FNIHGTNKHWLIRQAKTPMTNSSIQFLDNAFRKRWQTLLSV |
| P15586 | LSVDDLVEKLVKRLEFTGELNNTYIFYTSDNGYHTGQFSLP |
| P15586 | QLYEFDIKVPLLVRGPGIKPNQTSKMLVANIDLGPTILDIA |
| P15586 | MLVANIDLGPTILDIAGYDLNKTQMDGMSLLPILRGASNLT |
| P15586 | DLNKTQMDGMSLLPILRGASNLTWRSDVLVEYQGEGRNVTD |
| P15586 | GASNLTWRSDVLVEYQGEGRNVTDPTCPSLSPGVSQCFPDC |
| P15586 | PSLSPGVSQCFPDCVCEDAYNNTYACVRTMSALWNLQYCEF |
| P15586 | ALWNLQYCEFDDQEVFVEVYNLTADPDQITNIAKTIDPELL |
| Q91XA2 | HSFQLENVNKLHQDEKAVLVNNITTGEKLIRDLQDQLKALQ |
| Q91XA2 | SFQLENVNKLHQDEKAVLVNNITTGEKLIRDLQDQLKALQR |
| Q91XA2 | LKALQRSYSSLQQDIFQFQKNQTSLEKKFSYDLNQCISQMT |
| Q91XA2 | LQEEVPSEEQMPQEKGDVPRNKSQIPAPNSESLGLKPQVQN |

| | |
|---|---|
| Q8NBJ4 | NFQLESVNKLYQDEKAVLVNNITTGERLIRVLQDQLKTLQR |
| Q8NBJ4 | LKTLQRNYGRLQQDVLQFQKNQTNLERKFSYDLSQCINQMK |
| Q86XS8 | PARLAALALLTCSLWPARADNASQEYYTALINVTVQEPGRG |
| Q86XS8 | CSLWPARADNASQEYYTALINVTVQEPGRGAPLTFRIDRGR |
| Q86XS8 | QTRFFVPPNIKQWIALLQRGNCTFKEKISRAAFHNAVAVVI |
| Q86XS8 | FKEKISRAAFHNAVAVVIYNNKSKEEPVTMTHPGTGDIIAV |
| Q86XS8 | IIAVMITELRGKDILSYLEKNISVQMTIAVGTRMPPKNFSR |
| Q86XS8 | LEKNISVQMTIAVGTRMPPKNFSRGSLVFVSISFIVLMIIS |
| Q19791 | SDDAVYIVHLHRWNQIPDSHNKSVPHFSNSNFAPMVLYLDS |
| Q19791 | LYGMLALPSGIHTVEPIISGNGTEHDGASRHRQHLVRKFDP |
| Q19791 | HRQHLVRKFDPMHFKSFDHLNSTSVNETETTVATWQDQWED |
| Q19791 | VRKFDPMHFKSFDHLNSTSVNETETTVATWQDQWEDVIERK |
| Q19791 | IAFQDTVLEYSGSDAIIERINGTGPIRSDIYVHVLSVGSHP |
| Q19791 | FGVWSDCSAKCGDGVQYRDANCTDRHRSVLPEHRCLKMEKI |
| Q19791 | QCSVSCEDGWSSRRVSCVSGNGTEVDMSLCGTASDRPASHQ |
| Q19791 | DVPPIRWATGPWTACSATCGNGTQRRLLKCRDHVRDLPDEY |
| Q19791 | KMAEWEECPATCGTHVQQSRNVTCVSAEDGGRTILKDVDCD |
| Q19791 | ETRKPKMFDKCNEELCPPLTNNSWQISPWTHCSVSCGGGVQ |
| Q19791 | EIKPREQRDCEMPPCRSHYHNKTSSASMTSLSSSNSNTTSS |
| Q19791 | SHYHNKTSSASMTSLSSSNSNTTSSASASSLPILPPVVSWQ |
| Q19791 | SAKCGRGTKRRVVECVNPSLNVTVASTECDQTKKPVEEVRC |
| Q19791 | KSCGEGVQTREVRCRRKINFNSTIPIIFMLEDEPAVPKEKC |
| Q19791 | FMLEDEPAVPKEKCELFPKPNESQTCELNPCDSEFKWSFGP |
| Q19791 | NCLPSTCQELKSQNVKAKDGNYTILLDGFTIEIYCHRMNST |
| Q19791 | DGNYTILLDGFTIEIYCHRMNSTIPKAYLNVNPRTNFAEVY |
| Q19791 | AEVYGKKLIYPHTCPFNGDRNDSCHCSEDGDASAGLTRFNK |
| P13006 | GDIFGSSVDHAYETVELATNNQTALIRSGNGLGGSTLVNGG |
| Q86SQ4 | LDNGESQTKFCGATAKGLSFNSSANEMHVSFSSDFSIQKKG |
| Q86SQ4 | SANEMHVSFSSDFSIQKKGFNASYIRVAVSLRNQKVILPQT |
| Q86SQ4 | CFEATKVGHEDSDWTAFSYSNASFTQLLSFGKAKSGYFLSI |
| Q86SQ4 | PVKEKEDIFAESFEQLCLVWNNSLGSIGVNFKRNYETVPCD |
| Q86SQ4 | GSNQNEIVSLKGDIYNFRLWNFTMNAKILSNLSCNVKGNVV |
| Q86SQ4 | KGDIYNFRLWNFTMNAKILSNLSCNVKGNVVDWQNDFWNIP |
| Q86SQ4 | VVDWQNDFWNIPNLALKAESNLSCGSYLIPLPAAELASCAD |
| Q86SQ4 | IQNILRHPEVKVQSKVAEWLNSTFQNWNYTVYVVNISFHLS |
| Q86SQ4 | PEVKVQSKVAEWLNSTFQNWNYTVYVVNISFHLSAGEDKIK |
| Q86SQ4 | KVAEWLNSTFQNWNYTVYVVNISFHLSAGEDKIKVKRSLED |
| Q86SQ4 | KVKRSLEDEPRLVLWALLVYNATNNTNLEGKIIQQKLLKNN |
| Q86SQ4 | RSLEDEPRLVLWALLVYNATNNTNLEGKIIQQKLLKNNESL |
| Q86SQ4 | NATNNTNLEGKIIQQKLLKNNESLDEGLRLHTVNVRQLGHC |
| Q86SQ4 | EYVLPCPDKPGFSASRICFYNATNPLVTYWGPVDISNCLKE |

| | |
|---|---|
| Q86SQ4 | GPVDISNCLKEANEVANQILNLTADGQNLTSANITNIVEQV |
| Q86SQ4 | CLKEANEVANQILNLTADGQNLTSANITNIVEQVKRIVNKE |
| Q86SQ4 | NEVANQILNLTADGQNLTSANITNIVEQVKRIVNKEENIDI |
| Q86SQ4 | LESSSEALKTIDELAFKIDLNSTSHVNITTRNLALSVSSLL |
| Q86SQ4 | ALKTIDELAFKIDLNSTSHVNITTRNLALSVSSLLPGTNAI |
| Q86SQ4 | TTRNLALSVSSLLPGTNAISNFSIGLPSNNESYFQMDFESG |
| Q86SQ4 | SSLLPGTNAISNFSIGLPSNNESYFQMDFESGQVDPLASVI |
| Q86SQ4 | LENLSPEDSVLVRRAQFTFFNKTGLFQDVGPQRKTLVSYVM |
| Q86SQ4 | QDVGPQRKTLVSYVMACSIGNITIQNLKDPVQIKIKHTRTQ |
| Q86SQ4 | KHTRTQEVHHPICAFWDLNKNKSFGGWNTSGCVAHRDSDAS |
| Q86SQ4 | VHHPICAFWDLNKNKSFGGWNTSGCVAHRDSDASETVCLCN |
| Q6F3F9 | LDNGESQTKFCGATAKGLSFNSSVNEMHVSFSSDFSIQKKG |
| Q6F3F9 | SVNEMHVSFSSDFSIQKKGFNASYIRVAVSLRNQKVILPQT |
| Q6F3F9 | PVKDKEDIFTENLEQLCLVWNNSWGSIGINFKKNYETVPCD |
| Q6F3F9 | GSDRDEVASLRGSIYNFRLWNFTMDLKALSNLSCSVSGNVI |
| Q6F3F9 | RGSIYNFRLWNFTMDLKALSNLSCSVSGNVIDWHNDFWSIS |
| Q6F3F9 | VIDWHNDFWSISTQALKAEGNLSCGSYLIQLPAAELTNCSE |
| Q6F3F9 | AEGNLSCGSYLIQLPAAELTNCSELGTLCQDGIMYRISVVI |
| Q6F3F9 | IHNDFNHPEVKVQTKVAEWLNSTFQNWNYTVYVVNISFHQK |
| Q6F3F9 | PEVKVQTKVAEWLNSTFQNWNYTVYVVNISFHQKVGEDRMK |
| Q6F3F9 | KVAEWLNSTFQNWNYTVYVVNISFHQKVGEDRMKVKRDIMD |
| Q6F3F9 | VKRDIMDDDKRLVLWALLVYNATNNVSLNEEKIKQKLMTNN |
| Q6F3F9 | IMDDDKRLVLWALLVYNATNNVSLNEEKIKQKLMTNNASLE |
| Q6F3F9 | NATNNVSLNEEKIKQKLMTNNASLEDGLRLCEVDVNQLGMC |
| Q6F3F9 | VYKQPCPNKPGFFMTRACLSNGTSTFWGPVDTSNCSRQSNE |
| Q6F3F9 | MTRACLSNGTSTFWGPVDTSNCSRQSNEVANEILNQTGDGQ |
| Q6F3F9 | GPVDTSNCSRQSNEVANEILNQTGDGQNLTSANINSIVEKV |
| Q6F3F9 | CSRQSNEVANEILNQTGDGQNLTSANINSIVEKVKRIVNKE |
| Q6F3F9 | ETQNLALGVSSLIPGTNAPSNFSIGLPSNNESYFQMDFGNG |
| Q6F3F9 | SSLIPGTNAPSNFSIGLPSNNESYFQMDFGNGQTDPLASVI |
| Q6F3F9 | LENLSPEDSVLVRRAQFTFFNKTGLFQDVGSQRKVLVSYVM |
| Q6F3F9 | QDVGSQRKVLVSYVMACSIGNITIQNLKDPVQIKIKHTRTQ |
| Q6F3F9 | KHTRTQEVHHPICAFWDMNKNKSFGGWNTSGCVAHSDLDAG |
| Q6F3F9 | VHHPICAFWDMNKNKSFGGWNTSGCVAHSDLDAGETICLCS |
| P07359 | HPICEVSKVASHLEVNCDKRNLTALPPDLPKDTTILHLSEN |
| P07359 | LPPGLLTPTPKLEKLSLANNNLTELPAGLLNGLENLDTLLL |
| P13224 | WASLPTAFPVDTTELVLTGNNLTALPPGLLDALPALRTAHL |
| P28993 | PVALLCFFLYFCFICGSVGSNNTTICMHTTSDTSVHLFYAA |
| P28993 | VALLCFFLYFCFICGSVGSNNTTICMHTTSDTSVHLFYAAN |
| P28993 | NTTICMHTTSDTSVHLFYAANVTFPSHFQRHFAAAQDFVVH |
| P28993 | GVTMLVHLFANLVLTFPSLVNCSRPVNVFANASCVQVVCSH |

| | |
|---|---|
| P28993 | NLVLTFPSLVNCSRPVNVFANASCVQVVCSHTNSTTGLGQL |
| P28993 | SRPVNVFANASCVQVVCSHTNSTTGLGQLSFSFVDEDLRLH |
| P08148 | ARIVANVPNVRGKNFDVPVINSSTAVAKAREQYGCDTLEYL |
| P08148 | AFLTNKCMEQSVTQWPAMFCNESEDAIRCPTSRLSLGACGV |
| P08148 | QCDTATRTYSVQVHGSNDYTNCTPGLRVELSTVSNAFEGGG |
| Q52085 | GAVCDATANICEEGTVCIKPNSTAANTICFVLPTLNEDCSG |
| Q52085 | LPTLNEDCSGPLACADSYYCNTTSKICVEAYYLGVGESCSS |
| Q52085 | PLCGASNSRVGCKAGEGCAFNGTALVCSPFIANGAACNTST |
| Q52085 | CAFNGTALVCSPFIANGAACNTSTSGLCHPVSSCSNGVCTA |
| Q52085 | PVSSCSNGVCTAPLTGALNSNCTSNTDCNIANGLYCSSGKC |
| Q52085 | IANGLYCSSGKCTAVPEALNNCTTTPTVDNCLGYSACMCPS |
| Q99795 | SNNAEQSDASITIDQLTMADNGTYECSVSLMSDLEGNTKSR |
| Q99795 | ILNQEQPLAQPASGQPVSLKNISTDTSGYYICTSSNEEGTQ |
| Q99795 | TDTSGYYICTSSNEEGTQFCNITVAVRSPSMNVALYVGIAV |
| P35052 | LRICPQGYTCCTSEMEENLANRSHAELETALRDSSRVLQAM |
| P35052 | LQAMLATQLRSFDDHFQHLLNDSERTLQATFPGAFGELYTQ |
| Q9QZF2 | LRICPQGYTCCTSEMEENLANHSRMELESALHDSSRALQAT |
| Q9QZF2 | LQATLATQLHGIDDHFQRLLNDSERTLQEAFPGAFGDLYTQ |
| P38875 | ESRTTFPRRSYASDIGAPLFNSTEKLYLMRASLPNEPICTE |
| P49048 | VIDRYTHYTVNFLTKEVKALNSSANMQDYIDSCPARKCLSN |
| Q14956 | WKGGRVQAVLTSDSPALVGSNITFAVNLIFPRCQKEDANGN |
| Q14956 | IVYEKNCRNEAGLSADPYVYNWTAWSEDSDGENGTGQSHHN |
| Q14956 | LSADPYVYNWTAWSEDSDGENGTGQSHHNVFPDGKPFPHHP |
| Q14956 | LGQYFQKLGRCSVRVSVNTANVTLGPQLMEVTVYRRHGRAY |
| Q14956 | VYVVTDQIPVFVTMFQKNDRNSSDETFLKDLPIMFDVLIHD |
| Q14956 | FLKDLPIMFDVLIHDPSHFLNYSTINYKWSFGDNTGLFVST |
| Q14956 | YSTINYKWSFGDNTGLFVSTNHTVNHTYVLNGTFSLNLTVK |
| Q14956 | NYKWSFGDNTGLFVSTNHTVNHTYVLNGTFSLNLTVKAAAP |
| Q14956 | GDNTGLFVSTNHTVNHTYVLNGTFSLNLTVKAAAPGPCPPP |
| Q14956 | FVSTNHTVNHTYVLNGTFSLNLTVKAAAPGPCPPPPPPPRP |
| Q14956 | TVCSPVDVDEMCLLTVRRTFNGSGTYCVNLTLGDDTSLALT |
| Q14956 | DEMCLLTVRRTFNGSGTYCVNLTLGDDTSLALTSTLISVPD |
| Q8K209 | LVQGAHSGSPREDFRFCGQRNQTQQSTLHYDQSSEPHIFVW |
| Q8K209 | SLKQGAPLIATSVSSWQIPQNTSLPGAPSFIFSFHNAPHKV |
| Q8K209 | LPGAPSFIFSFHNAPHKVSHNASVDMCDLKKELQQLSRYLQ |
| Q8K209 | SKLTSVSFLGDTLSFEEDRVNATVWKLPPTAGLEDLHIHSQ |
| Q8K209 | DDAKRLLVVDFSSQALFQDKNSSQVLGEKVLGIVVQNTKVT |
| Q8K209 | SSQVLGEKVLGIVVQNTKVTNLSDPVVLTFQHQPQPKNVTL |
| Q8K209 | KVTNLSDPVVLTFQHQPQPKNVTLQCVFWVEDPASSSTGSW |
| Q9HCN6 | VLFIPAMKRSLAGRYRCSYQNGSLWSLPSDQLELVATGVFA |
| P40197 | DAAQCSGGDVARISALGLPTNLTHILLFGMGRGVLQSQSFS |

| | |
|---|---|
| P40197 | LPASLFTNLENLKLLDLSGNNLTHLPKGLLGAQAKLERLLL |
| P40197 | LQFHRNHIRSIAPGAFDRLPNLSSLTLSRNHLAFLPSALFL |
| P40197 | LTLSRNHLAFLPSALFLHSHNLTLLTLFENPLAELPGVLFG |
| P40197 | LAELPGVLFGEMGGLQELWLNRTQLRTLPAAAFRNLSRLRY |
| P40197 | LQELWLNRTQLRTLPAAAFRNLSRLRYLGVTLSPRLSALPQ |
| P40197 | LRQVSLRRNRLRALPRALFRNLSSLESVQLDHNQLETLPGD |
| P40197 | PPRPAADSSSEAPVHPALAPNSSEPWVWAQPVTTGKGQDHS |
| P00435 | NCLKYVRPGGGFEPNFMLFEKCEVNGEKAHPLFAFLREVLP |
| P67877 | AQLGPKIGKQFLKPKQCEITNQTVYDFQVQMLNGAQKSLAE |
| P67877 | TYCAYTMQYRDFNPILESNSNGTLNILGFPCNQFYLQEPAE |
| P67878 | AQLGPKIGKQFLKPKQCEITNQTVYDFQVQMLNGAQKSLAE |
| P67878 | TYCAYTMQYRDFNPILESNSNGTLNILGFPCNQFYLQEPAE |
| P12544 | VAGWGRTHNSASWSDTLREVNITIIDRKVCNDRNHYNFNPV |
| P10144 | GFLIRDDFVLTAAHCWGSSINVTLGAHNIKEQEPTQQFIPV |
| P10144 | PTQQFIPVKRPIPHPAYNPKNFSNDIMLLQLERKAKRTRAV |
| P42262 | IIANLGFTDGDLLKIQFGGANVSGFQIVDYDDSLVSKFIER |
| P42262 | QVQVEGLSGNIKFDQNGKRINYTINIMELKTNGPRKIGYWS |
| P42262 | IGYWSEVDKMVVTLTELPSGNDTSGLENKTVVVTTILESPY |
| P42262 | DKMVVTLTELPSGNDTSGLENKTVVVTTILESPYVMMKKNH |
| P19490 | RFALSQLTEPPKLLPQIDIVNISDSFEMTYRFCSQFSKGVY |
| P19490 | ILANLGFMDIDLNKFKESGANVTGFQLVNYTDTIPARIMQQ |
| P19490 | DIDLNKFKESGANVTGFQLVNYTDTIPARIMQQWRTSDSRD |
| P19490 | QVRFEGLTGNVQFNEKGRRTNYTLHVIEMKHDGIRKIGYWN |
| P19490 | YWNEDDKFVPAATDAQAGGDNSSVQNRTYIVTTILEDPYVM |
| P19490 | DKFVPAATDAQAGGDNSSVQNRTYIVTTILEDPYVMLKKNA |
| P19491 | IIANLGFTDGDLLKIQFGGANVSGFQIVDYDDSLVSKFIER |
| P19491 | QVQVEGLSGNIKFDQNGKRINYTINIMELKTNGPRKIGYWS |
| P19491 | IGYWSEVDKMVVTLTELPSGNDTSGLENKTVVVTTILESPY |
| P19491 | DKMVVTLTELPSGNDTSGLENKTVVVTTILESPYVMMKKNH |
| P19492 | NTVQEHSAFRFAVQLYNTNQNTTEKPFHLNYHVDHLDSSNS |
| P19492 | MLANLGFTDILLERVMHGGANITGFQIVNNENPMVQQFIQR |
| P19492 | MVQVQGMTGNIQFDTYGRRTNYTIDVYEMKVSGSRKAGYWN |
| P19492 | KAGYWNEYERFVPFSDQQISNDSSSSENRTIVVTTILESPY |
| P19492 | YERFVPFSDQQISNDSSSSENRTIVVTTILESPYVMYKKNH |
| P19493 | NTDQEYTAFRLAIFLHNTSPNASEAPFNLVPHVDNIETANS |
| P19493 | IIANLGFKDISLERFIHGGANVTGFQLVDFNTPMVTKLMDR |
| P19493 | QVRIQGLTGNVQFDHYGRRVNYTMDVFELKSTGPRKVGYWN |
| P19493 | VGYWNDMDKLVLIQDMPTLGNDTAAIENRTVVVTTIMESPY |
| P19493 | DKLVLIQDMPTLGNDTAAIENRTVVVTTIMESPYVMYKKNH |
| P42260 | SGPMGAEELAFRFAVNTINRNRTLLPNTTLTYDTQKINLYD |
| P42260 | EELAFRFAVNTINRNRTLLPNTTLTYDTQKINLYDSFEASK |

| | |
|---|---|
| P42260 | IFTTLDLFALDVEPYRYSGVNMTGFRILNTENTQVSSIIEK |
| P42260 | RFMSLIKEAHWEGLTGRITFNKTNGLRTDFDLDVISLKEEG |
| P42260 | ISLKEEGLEKIGTWDPASGLNMTESQKGKPANITDSLSNRS |
| P42260 | GTWDPASGLNMTESQKGKPANITDSLSNRSLIVTTILEEPY |
| P42260 | GLNMTESQKGKPANITDSLSNRSLIVTTILEEPYVLFKKSD |
| P42260 | VIDFSKPFMTLGISILYRKPNGTNPGVFSFLNPLSPDIWMY |
| P42260 | SDYAFLMESTTIEFVTQRNCNLTQIGGLIDSKGYGVGTPMG |
| P42264 | AQVMNAEEHAFRFSANIINRNRTLLPNTTLTYDIQRIHFHD |
| P42264 | EEHAFRFSANIINRNRTLLPNTTLTYDIQRIHFHDSFEATK |
| P42264 | IFTTLDLYALDLEPYRYSGVNLTGFRILNVDNAHVSAIVEK |
| P42264 | RFMNFIKEAQWEGLTGRIVFNKTSGLRTDFDLDIISLKEDG |
| P42264 | ISLKEDGLEKVGVWSPADGLNITEVAKGRGPNVTDSLTNRS |
| P42264 | GVWSPADGLNITEVAKGRGPNVTDSLTNRSLIVTTLLEEPF |
| P42264 | GLNITEVAKGRGPNVTDSLTNRSLIVTTLLEEPFVMFRKSD |
| P42264 | AIDFSKPFMTLGVSILYRKPNGTNPSVFSFLNPLSPDIWMY |
| Q63273 | PTPLLKEIRDDKVSTIIIDANASISHLVLRKASELGMTSAF |
| Q63273 | ILHLDGIVEDSSNILGFSMFNTSHPFYPEFVRSLNMSWREN |
| Q63273 | LGFSMFNTSHPFYPEFVRSLNMSWRENCEASTYPGPALSAA |
| Q63273 | LSAALMFDAVHVVVSAVRELNRSQEIGVKPLACTSANIWPH |
| Q63273 | MVEYDGLTGRVEFNSKGQRTNYTLRILEKSRQGHREIGVWY |
| Q63273 | TLRILEKSRQGHREIGVWYSNRTLAMNATTLDINLSQTLAN |
| Q63273 | KSRQGHREIGVWYSNRTLAMNATTLDINLSQTLANKTLVVT |
| Q63273 | EIGVWYSNRTLAMNATTLDINLSQTLANKTLVVTTILENPY |
| Q63273 | NRTLAMNATTLDINLSQTLANKTLVVTTILENPYVMRRPNF |
| Q63273 | LRFRYRLRLVEDGLYGAPEPNGSWTGMVGELINRKADLAVA |
| Q13255 | RVEAMFHTLDKINADPVLLPNITLGSEIRDSCWHSSVALEQ |
| Q13255 | RVVPSDTLQARAMLDIVKRYNWTYVSAVHTEGNYGESGMDA |
| Q13255 | QCRLPGHLLENPNFKRICTGNESLEENYVQDSKMGFVINAI |
| Q13255 | VHVGTWHEGVLNIDDYKIQMNKSGVVRSVCSEPCLKGQIKV |
| P23385 | RVEAMFHTLDKINADPVLLPNITLGSEIRDSCWHSSVALEQ |
| P23385 | RVVPSDTLQARAMLDIVKRYNWTYVSAVHTEGNYGESGMDA |
| P23385 | QCRLPGHLLENPNFKKVCTGNESLEENYVQDSKMGFVINAI |
| P23385 | VHVGTWHEGVLNIDDYKIQMNKSGMVRSVCSEPCLKGQIKV |
| P31422 | RTVPPDFYQAKAMAEILRFFNWTYVSTVASEGDYGETGIEA |
| P31422 | VVLFMRSDDSRELIAAANRVNASFTWVASDGWGAQESIVKG |
| P31422 | VNAVYAMAHALHKMQRTLCPNTTKLCDAMKILDGKKLYKEY |
| P31422 | CDAMKILDGKKLYKEYLLKINFTAPFNPNKGADSIVKFDTF |
| P41594 | RVEAMLHTLERINSDPTLLPNITLGCEIRDSCWHSAVALEQ |
| P41594 | RVVPSDAQQARAMVDIVKRYNWTYVSAVHTEGNYGESGMEA |
| P41594 | FWQHRFQCRLEGFPQENSKYNKTCNSSLTLKTHHVQDSKMG |
| P41594 | RFQCRLEGFPQENSKYNKTCNSSLTLKTHHVQDSKMGFVIN |

| | |
|---|---|
| P41594 | LCDAMKPIDGRKLLESLMKTNFTGVSGDTILFDENGDSPGR |
| P41594 | MEPPDIMHDYPSIREVYLICNTTNLGVVTPLGYNGLLILSC |
| P91685 | PGITIGVHILDTCSRDTYALNQSLQFVRASLNNLDTSGYEC |
| P91685 | NNLDTSGYECADGSSPQLRKNASSGPVFGVIGGSYSSVSLQ |
| P91685 | RTVPPDTFQSVALVDILKNFNWSYVSTIHSEGSYGEYGIEA |
| P91685 | VVLFTRAEDARRILQAAKRANLSQPFHWIASDGWGKQQKLL |
| P91685 | DTFNCVLTSLSVKPDTSNSANSTDNKIGVKAKTECDDSYRL |
| P91685 | QACPDMANYDGKEFYNNYLLNVSFIDLAGSEVKFDRQGDGL |
| P91685 | FDRQGDGLARYDILNYQRQENSSGYQYKVIGKWFNGLQLNS |
| P28799 | CCPRGFHCSADGRSCFQRSGNNSVGAIQCPDSQFECPDFST |
| P28799 | PDGSTCCELPSGKYGCCPMPNATCCSDHLHCCPQDTVCDLI |
| P28799 | HCCPQDTVCDLIQSKCLSKENATTDLLTKLPAHTVGDVKCD |
| P28799 | APAHLSLPDPQALKRDVPCDNVSSCPSSDTCCQLTSGEWGC |
| P28799 | RSPHVGVKDVECGEGHFCHDNQTCCRDNRQGWACCPYRQGV |
| Q92896 | REPENEISSDCNHLLWNYKLNLTTDPKFESVAREVCKSTIT |
| Q92896 | CADEPVGKGYMVSCLVDHRGNITEYQCHQYITKMTAIIFSD |
| Q92896 | PVLYRKCQGDASRLCHTHGWNETSEFMPQGAVFSCLYRHAY |
| Q92896 | LECLQDHLDDLVVECRDIVGNLTELESEDIQIEALLMRACE |
| Q92896 | LCPNIKKKVDVVICLSTTVRNDTLQEAKEHRVSLKCRRQLR |
| Q61543 | REPENEISSDCNHLLWNYKLNLTTDPKFESVAREVCKSTIS |
| Q61543 | CAEEPVGKGYMVSCLVDHRGNITEYQCHQYITKMTAIIFSD |
| Q61543 | PVLYRKCQGDASRLCHTHGWNETSELMPPGAVFSCLYRHAY |
| Q61543 | LECLQDHLDDLAVECRDIVGNLTELESEDIQIEALLMRACE |
| Q61543 | LCPNIKKKVDVVICLSTTVRNDTLQEAKEHRVSLKCRKQLR |
| Q19459 | LCTSQTQPGFALSCLMEFTKNVTETGKCHAFLARTERLAFS |
| Q19459 | GDIEAEGRTLHCLMEHAESRNETLKLGAQCLQAVQQVVKVA |
| Q8K297 | QLRHPRERTALWVATDHNTDNTSAILREWLVAVKGLYHSVE |
| Q8K297 | FMDIDNLITNPDTLSLLIAENKTVVAPMLDSRAAYSNFWCG |
| Q8K297 | ALHEQEIDCQLVEAVDGKAMNTSQVEAMGIQMLPGYRDPYH |
| Q8NBJ5 | RLRHPRERTALWVATDHNMDNTSTVLREWLVAVKSLYHSVE |
| Q8NBJ5 | FVDADNLILNPDTLSLLIAENKTVVAPMLDSRAAYSNFWCG |
| Q8NBJ5 | ALQAQEIECRLVEAVDGKAMNTSQVEALGIQMLPGYRDPYH |
| P10257 | MLWVTPVRAGTECRYGCRLNNMTIIVEREDCHGSITITTCA |
| P10256 | FLCILLEPVEGSLMQPCQPINQTVSLEKEGCPTCLVIQTPI |
| P17809 | QFGYNTGVINAPQKVIEEFYNQTWNHRYGEPIPSTTLTTLW |
| P17809 | NTGVINAPQKVIEEFYNQTWNHRYGEPIPSTTLTTLWSLSV |
| P11166 | QFGYNTGVINAPQKVIEEFYNQTWVHRYGESILPTTLTTLW |
| P32037 | QFGYNTGVINAPETILKDFLNYTLEERLEDLPSEGLLTALW |
| P14672 | QFGYNIGVINAPQKVIEQSYNETWLGRQGPEGPSSIPPGTL |
| P14142 | QFGYNIGVINAPQKVIEQSYNATWLGRQGPGGPDSIPQGTL |
| P12257 | YPYLAWAYNPSAMDMGYALFNASGTVVRDGAYGYQNLFDTT |

| | |
|---|---|
| P25092 | SLLFQPGWLSFSSQVSQNCHNGSYEISVLMMGNSAFAEPLK |
| P25092 | EDAVNEGLEIVRGRLQNAGLNVTVNATFMYSDGLIHNSGDC |
| P25092 | NEGLEIVRGRLQNAGLNVTVNATFMYSDGLIHNSGDCRSST |
| P25092 | WKTNDLPFKTYSWSTSYVYKNGTETEDCFWYLNALEASVSY |
| P25092 | VAEDIVIILVDLFNDQYFEDNVTAPDYMKNVLVLTLSPGNS |
| P25092 | APDYMKNVLVLTLSPGNSLLNSSFSRNLSPTKRDFALAYLN |
| P25092 | YLNGILLFGHMLKIFLENGENITTPKFAHAFRNLTFEGYDG |
| P25092 | LYTSVDTKKYKVLLTYDTHVNKTYPVDMSPTFTWKNSKLPN |
| P56680 | CAKNCIMEGIPDYSQYGVTTNGTSLRLQHILPDGRVPSPRV |
| P56680 | ECAFEGVCDKNGCGWNNYRVNVTDYYGRGEEFKVNTLKPFT |
| Q7Z9M7 | AILAALGCLAGSVLGHGQVQNFTINGQYNQGFILDYYYQKQ |
| P46237 | GIHGIRQKNGAGCGDWGQKPNATACPDEASCAKNCILSGMD |
| P46237 | ECGSSGICDKAGCGWNHNRINVTDFYGRGKQYKVDSTRKFT |
| P46237 | LIELHRHYIQDNKVIESAVVNISGPPKINFINDKYCAATGA |
| P07987 | SVPLEERQACSSVWGQCGGQNWSGPTCCASGSTCVYSNDYY |
| P07987 | WLGWPANQDPAAQLFANVYKNASSPRALRGLATNVANYNGW |
| P07987 | ASSPRALRGLATNVANYNGWNITSPPSYTQGNAVYNEKLYI |
| Q9C1S9 | ALRAAASAVAEVPSFQWLDRNVTVDTLLVETLSEIRAANQA |
| Q4G148 | DQLHHSFKGRLDNWSFLQTFNYTLYPITFPSENAAEWKKLF |
| Q4G148 | DTDILFLRPVDDIWSLLKKFNSTQIAAMAPEHEEPRIGWYN |
| Q4G148 | RFARHPYYGKTGVNSGVMLMNMTRMRRKYFKNDMTTVRLQW |
| P01898 | RAKGNEQSFHVSLRTLLGYYNQSESGSHTIQWMYGCKVGSD |
| P01898 | AGDGTFQKWASVVVPLGKEQNYTCHVYHEGLPEPLTLRWEP |
| P01899 | KAKGQEQWFRVSLRNLLGYYNQSAGGSHTLQQMSGCDLGSD |
| P01899 | YKAYLEGECVEWLHRYLKNGNATLLRTDSPKAHVTHHPRSK |
| P01899 | AGDGTFQKWASVVVPLGKEQNYTCRVYHEGLPEPLTLRWEP |
| P14426 | IAKGNEQSFRVDLRTLLRYYNQSEGGSHTIQRLSGCDVGSD |
| P14426 | DRAYLEGTCVEWLRRYLELGNATLLHTDSPKAHVTHHPRSK |
| P14426 | AGDGTFQKWASVVVPLGKEQNYTCHVYHEGLPEPLTLRWEP |
| P01901 | KAKGNEQSFRVDLRTLLGYYNQSKGGSHTIQVISGCEVGSD |
| P01901 | LRAYLEGTCVEWLRRYLKNGNATLLRTDSPKAHVTHHSRPE |
| P01902 | RAKSDEQWFRVSLRTAQRYYNQSKGGSHTFQRMFGCDVGSD |
| P01902 | YRAYLEGECVEWLRRYLELGNETLLRTDSPKAHVTYHPRSQ |
| P01902 | AGDGTFQKWAAVVVPLGKEQNYTCHVHHKGLPEPLTLRWKL |
| P04223 | IAKGNEQIFRVNLRTALRYYNQSAGGSHTFQRMYGCEVGSD |
| P04223 | DRAYLEGTCVEWLRRYLQLGNATLPRTDSPKAHVTRHSRPE |
| P01897 | IAKGQEQWFRVNLRTLLGYYNQSAGGTHTLQWMYGCDVGSD |
| P01897 | YRAYLEGECVEWLHRYLKNGNATLLRTDSPKAHVTHHPRSK |
| P01897 | AGDGTFQKWASVVVPLGKEQNYTCRVYHEGLPEPLTLRWEP |
| P14434 | LLGQPNTLICFVDNIFPPVINITWLRNSKSVADGVYETSFF |
| P23150 | GGLQEIATGKYNLEILIKDSNFTPAANEAPQATVFPKSPVL |

| | |
|---|---|
| P23150 | LLGQPNTLICFVDNIFPPVINITWLRNSKSVTDGVYETSFL |
| P01910 | GGLQNIATGKHNLEILTKRSNSTPATNEAPQATVFPKSPVL |
| P01910 | LLGQPNTLICFVDNIFPPVINITWLRNSKSVTDGVYETSFF |
| Q8TDQ0 | LTTRGHGPAETQTLGSLPDINLTQISTLANELRDSRLANDL |
| P18469 | LVRNSRPRFLEYSTSECHFYNGTQRVRFLERYIYNREEYVR |
| P06343 | EGGNSERHFVHQFQPFCYFTNGTQRIRLVIRYIYNREEYVR |
| P69905 | HAGEYGAEALERMFLSFPTTKTYFPHFDLSHGSAQVKGHGK |
| P69905 | TYFPHFDLSHGSAQVKGHGKKVADALTNAVAHVDDMPNALS |
| P68871 | GDLSTPDAVMGNPKVKAHGKKVLGAFSDGLAHLDNLKGTFA |
| P68871 | PENFRLLGNVLVCVLAHHFGKEFTPPVQAAYQKVVAGVANA |
| P03141 | RQPTPISPPLRDSHPQAMQWNSTAFHQTLQDPRVRGLYLPA |
| P03141 | TPAQGNSMFPSCCCTKPTDGNCTCIPIPSSWAFAKYLWEWA |
| P03138 | RQPTPLSPPLRNTHPQAMQWNSTTFHQTLQDPRVRGLYFPA |
| P03138 | TTAQGTSMYPSCCCTKPSDGNCTCIPIPSSWAFGKFLWEWA |
| P80960 | VDSLTEEEILTLQSVMRELQNDSSEHGFQSIASFHGSPPLC |
| P80960 | VELMREPLQPFAQTSATNPNNVTRAHSTPKSLFNYRQLAGY |
| P83040 | QEDLPAFFNDEIWDDPLFHANFTNPFNGADIDFNHQKIARD |
| P84293 | DMLLFEERIQDAIAHGYLRYNGSTINIRDNHGIDVLGDVFE |
| P04254 | NSEVIDKAYSAKMTQKQGTFNVSFTGTKKNREQRVAYFGED |
| P10787 | NSEVIDKAYSAKMTQKPGTFNVSFTGTKKNREQRVAYFGED |
| P12031 | AINLMRKPLQPFQDKKLNPRNITNIYSRPADTFDYRNHFHY |
| P80096 | TYIERLNHKKFSFLILVTNNNNTEVLATVRIFAWPLRDNNG |
| P56825 | MPTFKLNFDSHFTIKTVVAQNGTELPESILPEATIDRIPPS |
| O61363 | KSLGIPLDGNYYVHADVTEINGTLLPDGTIPRPTVSYIPHN |
| O61363 | AELGVDMHAEYSINLQINDINGTALPPTSIPDPIVIFSPGK |
| O61363 | PYNEANCALPLLNVPMRPFSNTTANHDRMTLTHSAPNDVFD |
| O61363 | KLLGLNQNSHFRGVTEVTAVNGSSINSDIFPHPTIIYVPKQ |
| O61363 | SDMNLAFDSAFTIKTKLVAQNGTELPASILPEATVIRIPPS |
| O61363 | PGTKLPRLLADSDYYDAWTDNVTENPFLRGYIKTEDTYTVR |
| O61363 | DNGYQKIASYHGIPLSCHYENGTAYACCQHGMVTFPNWHRL |
| P56823 | RLTVSETENLREALRRIKADNGSDGFQSIASFHGSPPGCEH |
| P56823 | SDGFQSIASFHGSPPGCEHENHSVACCIHGMANFPQWHRLY |
| P56823 | SKLHLRPDSEYHFNIHIVSVNGTELDSHLIRSPTVQFVPGV |
| P80888 | NSEVIDKAYSAKMTHKEGTFNMSFTGTQKNREQRVAYFGQD |
| Q9D1N2 | RDRAPLQCYFCQVLHSGESCNQTQSCSSSKPFCITLVSHSG |
| Q9ULI3 | GSADAAWKHWPESNTEAHVENITFYQNQEDFSTVSSKEGVM |
| Q9ULI3 | KEGVMVQTSGKSHAASDAPENLTLLAETADARGRSGSSSRT |
| Q9ULI3 | LTLLAETADARGRSGSSSRTNFTILPVGYSLEIATALTSQS |
| Q9ULI3 | AASSPLLDLSSSSESTEKLNNSTGLQSSSVSQTKTMHVATV |
| Q9ULI3 | VSRSVAPMRGGEITAHWLLTNSTTSADVTGSSASYPEGVNA |
| Q9ULI3 | TALGDRSYSESSSTSSSESLNSSAPRGERSIAGISYGQVRG |

| | |
|---|---|
| Q9ULI3 | ISNSSHSEYSSFFHAQTERSNISSYDGEYAQPSTESPVLHT |
| Q9ULI3 | ASRESNAVVISLQTTFSLASNVTLFDLADRMQKCVNSCKSS |
| P0C0V9 | LCPKFSSKSSSSMFLSLHWNNHSSFVSYDYFNCGVEKVFYE |
| P0C0V9 | GSTESHDYVCDYLFMEPGTYNASTVGKFLVYPTKSYCMDTM |
| P0C0V9 | ASTVGKFLVYPTKSYCMDTMNITVPVQAVQSIWSEQYASDD |
| P0C0V9 | QYASDDAIGQACKAPYCIFYNKTTPYTVTNGSDANHGDDEV |
| P0C0V9 | QACKAPYCIFYNKTTPYTVTNGSDANHGDDEVRMMMQGLLR |
| P0C0V9 | GSDANHGDDEVRMMMQGLLRNSSCISPQGSTPLALYSTEMI |
| P15776 | WFLFGDSRSDCNHVVNTNPRNYSYMDLNPALCDSGKISSKA |
| P15776 | KISSKAGNSIFRSFHFTDFYNYTGEGQQIIFYEGVNFTPYH |
| P15776 | VYKNMAVYRSLTFVNVPYVYNGSAQSTALCKSGSLVLNNPA |
| P15776 | KYYDDSQYYFNKDTGVIYGLNSTETITTGFDFNCHYLVLPS |
| P15776 | KDFTPVQVVDSRWNNARQSDNMTAVACQPPYCYFRNSTTNY |
| P15776 | ARQSDNMTAVACQPPYCYFRNSTTNYVGVYDINHGDAGFTS |
| P15776 | LSGLLYDSPCFSQQGVFRYDNVSSVWPLYSYGRCPTAADIN |
| P31614 | RISAKSGNSLFRSFHFIDFYNYSGEGDQVIFYEGVNFSPSH |
| P31614 | ARFYARVYEKMAQYRSLSFVNVSYAYGGNAKPTSICKDKTL |
| P31614 | NPTFISKESNYVDYYYESEANFTLQGCDEFIVPLCVFNGHS |
| P31614 | KYYTDSQSYYNMDTGVLYGFNSTLDVGNTVQNPGLDLTCRY |
| P31614 | AICLRKPKSFMPVQVVDSRWNSTRQSDNMTAVACQLPYCFF |
| P31614 | KSFMPVQVVDSRWNSTRQSDNMTAVACQLPYCFFRNTSADY |
| P31614 | TRQSDNMTAVACQLPYCFFRNTSADYSGGTHDVHHGDFHFR |
| P31614 | THDVHHGDFHFRQLLSGLLYNVSCIAQQGAFVYNNVSSSWP |
| P31614 | LSGLLYNVSCIAQQGAFVYNNVSSSWPAYGYGHCPTAANIG |
| P03459 | LVAVIPTNADKICLGHHAVSNGTKVNTLTERGVEVVNATET |
| P03459 | HAVSNGTKVNTLTERGVEVVNATETVERTNIPKICSKGKRT |
| P03459 | RGSGGIDKETMGFTYSGIRTNGTTSACRRSGSSFYAEMEWL |
| P03459 | CRRSGSSFYAEMEWLLSNTDNASFPQMTKSYKNTRRESALI |
| P03459 | RPQINGQSGRIDFHWLILDPNDTVTFSFNGAFIAPNRASFL |
| P03459 | QFELIDNEFTEVEKQIGNLINWTKDFITEVWSYNAELLVAM |
| P03459 | SEMNKLYERVRKQLRENAEEDGTGCFEIFHKCDDDCMASIR |
| P03459 | GTGCFEIFHKCDDDCMASIRNNTYDHSKYREEAMQNRIQID |
| P03437 | IIALSYIFCLALGQDLPGNDNSTATLCLGHHAVPNGTLVKT |
| P03437 | DLPGNDNSTATLCLGHHAVPNGTLVKTITDDQIEVTNATEL |
| P03437 | HAVPNGTLVKTITDDQIEVTNATELVQSSSTGKICNNPHRI |
| P03437 | GIDCTLIDALLGDPHCDVFQNETWDLFVERSKAFSNCYPYD |
| P03437 | SGFFSRLNWLTKSGSTYPVLNVTMPNNDNFDKLYIWGIHHP |
| P03437 | SSIMRSDAPIDTCISECITPNGSIPNDKPFQNVNKITYGAC |
| P03437 | GNGCFKIYHKCDNACIESIRNGTYDHDVYRDEALNNRFQIK |
| P07975 | LLVLGLTEAEKIKICLQKQVNSSFSLHNGFGGNLYATEEKR |
| P07975 | ATEEKRMFELVKPKAGASVLNQSTWIGFGDSRTDKSNSAFP |

| | |
|---|---|
| P07975 | KVFYEGVNWSPHAAINCYRKNWTDIKLNFQKNIYELASQSH |
| P07975 | RELLSGLDYEARCISQSGWVNETSPFTEKYLLPPKFGRCPL |
| P02790 | PDVTERCSDGWSFDATTLDDNGTMLFFKGEFVWKSHKWDRE |
| P02790 | EWFWDLATGTMKERSWPAVGNCSSALRWLGRYYCFQGNQFL |
| P02790 | YPRDVRDYFMPCPGRGHGHRNGTGHGNSTHHGPEYMRCSPH |
| P02790 | DYFMPCPGRGHGHRNGTGHGNSTHHGPEYMRCSPHLVLSAL |
| Q91X72 | WSLAVASPLPTANGRVAEVENGTKPDSDVPEHCLDTWSFDA |
| Q91X72 | SDVPEHCLDTWSFDAATMDHNGTMLFFKGEFVWRGHSGTRE |
| Q91X72 | KWFWDFATRTQKERSWSTVGNCTAALRWLERYYCFQGNKFL |
| Q91X72 | PLDARDYFVSCPGRGHGRPRNGTAHGNSTHPMHSRCSPDPG |
| Q91X72 | YFVSCPGRGHGRPRNGTAHGNSTHPMHSRCSPDPGLTALLS |
| Q91X72 | EKVDGALCLDKSLGPNTCSSNGSSLYFIHGPNLYCYSSIDK |
| P20058 | WGLCWSLATVNSVPLTSAHGNVTEGESGTKPEADVIEQCSD |
| P20058 | ADVIEQCSDGWSFDATTLDDNGTMLFFKDEFVWKSHRGIRE |
| P20058 | KWFWDLTTGTKKERSWPAVGNCTSALRWLGRYYCFQGNQFL |
| P20058 | LDVRDYFLSCPGRGHRSSHRNSTQHGHESTRCDPDLVLSAM |
| P05546 | EKGGETAQSADPQWEQLNNKNLSMPLLPADFHKENTVTNDW |
| P05546 | GLKGETHEQVHSILHFKDFVNASSKYEITTIHNLFRKLTHR |
| P05546 | KTLEAQLTPRVVERWQKSMTNRTREVLLPKFKLEKNYNLVE |
| P49182 | SLITFMCIGSKGLAEQLTNENLTTSFLPANFHKENTVTNDW |
| P49182 | GLRGETHEEVHSVLHFRDFVNASSKYEVTTIHNLFRKLTHR |
| P49182 | KTLEAQLTPQVVERWQKSMTNRTREVLLPKFKLEKNYNLVE |
| P49182 | YNLVEVLKSMGITKLFNKNGNMSGISDQRIAIDLFKHQSTI |
| P05981 | EMGFLRALTHSELDVRTAGANGTSGFFCVDEGRLPHTQRLL |
| Q22492 | DRWSVGGVWPLPKKIVYGSKNRTITYDKIGIDLGDKKDCDI |
| Q22492 | VECWERNKKIRKFMEEKGFGNDTVLLENYFFEKLYKIVENL |
| Q22492 | NAVIHIWKGNTHEEIYEQVKNITSQNFPVIVSACWYLNYIK |
| Q22492 | EIRGTAPSNSRYYYCDPTNFNGTVAQKELVWGGIAAIWGEL |
| P06865 | LVVSVVTPGCNQLPTLESVENYTLTINDDQCLLLSETVWGA |
| P06865 | RGLETFSQLVWKSAEGTFFINKTEIEDFPRFPHRGLLLDTS |
| P06865 | TPCYSGSEPSGTFGPVNPSLNNTYEFMSTFFLEVSSVFPDF |
| P07686 | KMTPNLLHLAPENFYISHSPNSTAGPSCTLLEEAFRRYHGY |
| P07686 | TQVQQLLVSITLQSECDAFPNISSDESYTLLVKEPVAVLKA |
| P07686 | RGLETFSQLVYQDSYGTFTINESTIIDSPRFSHRGILIDTS |
| P07686 | KDLLTPCYSRQNKLDSFGPINPTLNTTYSFLTTFFKEISEV |
| P07686 | TPCYSRQNKLDSFGPINPTLNTTYSFLTTFFKEISEVFPDQ |
| Q9W568 | QLTMISSCSNISKWTNLHVRNMTVEDMDLSNPIFRSLQSLA |
| Q9W568 | DMDLSNPIFRSLQSLAVTDGNITRLVNAFPRLSALKCLNIS |
| Q9W568 | DGNITRLVNAFPRLSALKCLNISNNNISEIHSRAVKDVPHL |
| Q9W568 | TRLVNAFPRLSALKCLNISNNNISEIHSRAVKDVPHLEFFG |
| Q9W568 | RLVNAFPRLSALKCLNISNNNISEIHSRAVKDVPHLEFFGM |

| | |
|---|---|
| P04441 | ATPLLMRPMSMDNMLLGPVKNVTKYGNMTQDHVMHLLTRSG |
| P04441 | RPMSMDNMLLGPVKNVTKYGNMTQDHVMHLLTRSGPLEYPQ |
| P04233 | ATPLLMQALPMGALPQGPMQNATKYGNMTEDHVMHLLQNAD |
| P04233 | QALPMGALPQGPMQNATKYGNMTEDHVMHLLQNADPLKVYP |
| P04233 | GNYLPLQCYGSIGYCWCVFPNGTEVPNTRSRGHHNCSESLE |
| Q04756 | LLPRGFQPQPGGNRTESPEPNATATPAIPTILVTSVTSETP |
| Q04756 | PPGFAGRLCNIEPDERCFLGNGTGYRGVASTSASGLSCLAW |
| Q04756 | CFSHSPPRDSVSVVLGQHFFNRTTDVTQTFGIEKYIPYTLY |
| Q04756 | DVTQTFGIEKYIPYTLYSVFNPSDHDLVLIRLKKKGDRCAT |
| Q04756 | GSTFPAGHKCQIAGWGHLDENVSGYSSSLREALVPLVADHK |
| Q9R098 | FLLLLLLLVVPRGAQPQAGRNHTEPPGPNVTATPVTPTIPV |
| Q9R098 | VVPRGAQPQAGRNHTEPPGPNVTATPVTPTIPVISGNVSTS |
| Q9R098 | PPGPNVTATPVTPTIPVISGNVSTSTESAPAAETEGPQSER |
| Q9R098 | PLGYAGRFCNIVPTEHCFLGNGTEYRGVASTAASGLSCLAW |
| Q9R098 | CFANSPPRDSITVVLGQHFFNRTTDVTQTFGIEKYVPYTLY |
| Q9R098 | GSSFPTGHKCQIAGWGHMDENVSSYSNSLLEALVPLVADHK |
| P26927 | DAEECAGRCGPLMDCRAFHYNVSSHGCQLLPWTQHSPHTRL |
| P26927 | QEATTVSCFRGKGEGYRGTANTTTAGVPCQRWDAQIPHQHR |
| P26927 | VVPPGTKCEIAGWGETKGTGNDTVLNVALLNVISNQECNIK |
| P14210 | LDPHTRWEYCAIKTCADNTMNDTDVPLETTECIQGQGEGYR |
| P14210 | NCDMSHGQDCYRGNGKNYMGNLSQTRSGLTCSMWDKNMEDL |
| P14210 | AWLGIHDVHGRGDEKCKQVLNVSQLVYGPEGSDLVLMKLAR |
| P14210 | HLYIMGNEKCSQHHRGKVTLNESEICAGAEKIGSGPCEGDY |
| Q68CP4 | HAELKMDQALLLIHNELLWTNLTVYWKSECCYHCLFQVLVN |
| Q68CP4 | GKPSAAAASVSTQHGSILQLNDTLEEKEVCRLEYRFGEFGN |
| Q68CP4 | NDTLEEKEVCRLEYRFGEFGNYSLLVKNIHNGVSEIACDLA |
| Q3UDW8 | AKPSVAPVSVSTQHGSILQVNSTSEERAACRLEYKFGEFGN |
| Q3UDW8 | NSTSEERAACRLEYKFGEFGNYSLLVQHASSGANKIACDII |
| Q96QV1 | CLRSDSPGLGRLENKIFSVTNNTECGKLLEEIKCALCSPHS |
| Q96QV1 | DVDTDMCNVPYSIPRSNPHFNSTNQPPEVFAHGLHDPGRCA |
| Q96QV1 | HGLHDPGRCAVDRHPTDININLTILCSDSNGKNRSSARILQ |
| Q96QV1 | RHPTDININLTILCSDSNGKNRSSARILQIIKGKDYESEPS |
| Q09101 | IGPEPEPLADQDLKDLQQYGNQSSSARVALLWQRVKRKSGK |
| Q09101 | PLYKSCHVNRIPSHLLLSYRNISVTPIPPNRGWRKTRLSKS |
| Q09101 | CSNGLWAPRMPSCVPTTVLTNYSEDSAPSIRIKIFNGSHSF |
| Q09101 | TTVLTNYSEDSAPSIRIKIFNGSHSFEPSGVMAVPPHSTVL |
| Q09101 | KLGQRAHYECPEGFRLDGAWNATCLASGNWSSPTPTCHAIQ |
| Q09101 | HAIQCPRLELDDPHLSLIELNTSAWGRAVFKCQWGFKLTGP |
| Q84TI7 | LISITERQNLQRDPINFNVLNITLEVISAYGNVGFTTGYSC |
| P13747 | SARDTAQIFRVNLRTLRGYYNQSEAGSHTLQWMHGCELGPD |
| Q967F4 | LQPSIFSTNFEVEDGYRIITNTTVTQFHGELFELFLNVKEQ |

| | |
|---|---|
| Q967F4 | VHFLLKSKKSIAKFASAIPANSTVFDVEKRNLSEPLLFHLE |
| Q967F4 | IAKFASAIPANSTVFDVEKRNLSEPLLFHLEEPSRFFKIDQ |
| Q967F4 | PMTSSRSRRHLDDIVFRIPENTTMEDIEKKDMKIPLFAGET |
| Q967F4 | YERGDTYRISAQAMDLSPSDNTTSQLSEVAILEILADERPP |
| Q967F4 | NVPTFTRPLYTAQVREDIPLNQTILKVTAVDKDTGDNSRIT |
| Q967F4 | TAVDKDTGDNSRITYSVDNHNFSINSNGEISAKVRLDADQL |
| Q967F4 | SAKVRLDADQLNERHFVYRFNVTARDHGEPVSLSSSAMIHI |
| Q967F4 | LVKLRHGVSAADLAEAENPINLTVIVQDDGSCCVYPSKTHT |
| Q967F4 | NGNEWVEVVIMEGLDYEQVNNYTLTLTATDMTSRVASTKTF |
| Q967F4 | LDFEASDQYHLVLIASDGRHNATTNVYIHIEDVNDNAPQFE |
| Q967F4 | EFIIEVRANDRGVPSREGFANVTIKVTDMNDNAPFFEKTRY |
| Q967F4 | PSFKYSQYLNVIFNANGDGGNGSMTITPLQEFDREAPVPGK |
| Q967F4 | CDAGCQTLNSADYDGIVVSANSTVIVGVNATSRDDCTCPVW |
| Q967F4 | NSADYDGIVVSANSTVIVGVNATSRDDCTCPVWRAPPACQH |
| Q967F4 | RSFGGNGFAWYKPMPACTSLNISFSFMTTQSDALLFYNGPL |
| Q967F4 | FMTTQSDALLFYNGPLETLRNDTHIEYSDYIFIQLRGGRIS |
| Q967F4 | GTGGRRARQELRVSEVLLKENASYWLQFTRNPTRASLSIDN |
| Q95460 | LLRGWQQMFKVELKRLQRHYNHSGSHTYQRMIGCELLEDGS |
| P52708 | NKAANILFAESPAGVVFSYSNTSSDLSMGDDKMAQDTYTFL |
| P52708 | AVFNSINYLNLPEVQTALHANVSGIVEYPWTVCSNTIFDQW |
| P04853 | IISARQGYSMKEYSMTVEALNMSSREVKESLTSLIRQEVIA |
| P04853 | TDAYPLSPDAANVATVTLYANTSRVNPTIMYSNTTNIINML |
| P04853 | VATVTLYANTSRVNPTIMYSNTTNIINMLRIKDVQLEAAYT |
| P04850 | NDNRYINGINQFYFSIAEGRNLTLGPLLNMPSFIPTATTPE |
| P04850 | ERDDYFSAAPPEQRIIIMYYNDTIVERIINPPGVLDVWATL |
| P04850 | FTGSYLNTATQRINPTMYIANNTQIISSQQFGSSGQEAAYG |
| P07354 | NGPRLLVVAEQAKIFSQRGGNVTLPCKFYHEHTSTAGSGTH |
| P03994 | NGPRLLVEAEQAKVFSHRGGNVTLPCKFYRDPTAFGSGIHK |
| Q6YGZ1 | VEWPFQELLLLREQYQKEFKNSTYSRSSVDMLYSFAKCSGL |
| Q6YGZ1 | SGLDLIFGLNALLRTPDLRWNSSNAQLLLDYCSSKGYNISW |
| Q6YGZ1 | LRWNSSNAQLLLDYCSSKGYNISWELGNEPNSFWKKAHILI |
| Q6YGZ1 | NVYHPRYQEGDLTLYVLNLHNVTKHLKVPPPLFRKPVDTYL |
| Q9Y251 | LEWPYQEQLLLREHYQKKFKNSTYSRSSVDVLYTFANCSGL |
| Q9Y251 | KKFKNSTYSRSSVDVLYTFANCSGLDLIFGLNALLRTADLQ |
| Q9Y251 | SGLDLIFGLNALLRTADLQWNSSNAQLLLDYCSSKGYNISW |
| Q9Y251 | LQWNSSNAQLLLDYCSSKGYNISWELGNEPNSFLKKADIFI |
| Q9Y251 | ISWELGNEPNSFLKKADIFINGSQLGEDFIQLHKLLRKSTF |
| Q9Y251 | NTDNPRYKEGDLTLYAINLHNVTKYLRLPYPFSNKQVDKYL |
| P19006 | GGSVDAKGSFPWQAKMVSHHNLTSGATLINEQWLLTTAKNL |
| P19006 | DYAEVGRIGYVSGWGRNSNFNFTELLKYVMLPVADQDKCVQ |
| P00738 | GGHLDAKGSFPWQAKMVSHHNLTTGATLINEQWLLTTAKNL |

| | |
|---|---|
| P00738 | TGATLINEQWLLTTAKNLFLNHSENATAKDIAPTLTLYVGK |
| P00738 | LINEQWLLTTAKNLFLNHSENATAKDIAPTLTLYVGKKQLV |
| P00738 | LTLYVGKKQLVEIEKVVLHPNYSQVDIGLIKLKQKVSVNER |
| Q61646 | TGATLISDQWLLTTAKNLFLNHSETASAKDITPTLTLYVGK |
| Q61646 | LTLYVGKNQLVEIEKVVLHPNHSVVDIGLIKLKQRVLVTER |
| Q61646 | RLKYVMLPVADQDKCVVHYENSTVPEKKNLTSPVGVQPILN |
| Q61646 | VADQDKCVVHYENSTVPEKKNLTSPVGVQPILNEHTFCAGL |
| Q8SPS7 | GGSLDAKGSFPWQAKMISHHNLTSGATLINEQWLLTTAKNL |
| Q8SPS7 | TLINEQWLLTTAKNLRLGHKNDTKAKDIAPTLRLYVGKKQE |
| Q8SPS7 | RLYVGKKQEVEIEKVIFHPDNSTVDIGLIKLKQKVPVNERV |
| Q8SPS7 | DYVNVGLVGYVSGWGRNANLNFTEHLKYVMLPVADQEKCVQ |
| P90859 | VFTTFRSQPELQKLIEEELRNNTRLSSAYGLNIEALSGHTF |
| P33433 | YLVESDCPVLSRKHWDDCELNVTVIGQCKLAGPEDLSVNDF |
| P33433 | VTVIGQCKLAGPEDLSVNDFNCTTSSVSSALTNMRARGGEG |
| P33433 | TNMRARGGEGTSYFLDFSVRNCSSHHFPRHHIFGFCRADLF |
| P33433 | DHGHPHESYNFRCPPPLEHKNHSDSPPFQARAPLPFPPPGL |
| P04196 | RDGYLFQLLRIADAHLDRVENTTVYYLVLDVQESDCSVLSR |
| P04196 | CKVIATRHSHESQDLRVIDFNCTTSSVSSALANTKDSPVLI |
| P04196 | CSSCQHATFGTNGAQRHSHNNNSSDLHPHKHHSHEQHPHGH |
| P04196 | SSCQHATFGTNGAQRHSHNNNSSDLHPHKHHSHEQHPHGHH |
| Q9ESB3 | SRWQSEIVIGQCKVIATRYSNESQDLSVNGYNCTTSSVSSA |
| Q9ESB3 | CKVIATRYSNESQDLSVNGYNCTTSSVSSALRNTKDSPVLL |
| Q9ESB3 | RVIRARGGERTNYYVEFSMRNCSTQHFPRSPLVFGFCRALL |
| Q9ESB3 | RLQEGALPQLPPGYPPHSGANRTHRPSYNHSCNEHPCHGHR |
| Q9ESB3 | QLPPGYPPHSGANRTHRPSYNHSCNEHPCHGHRPHGHHPHS |
| P11147 | YSCVGVFQHGKVEIIANDQGNRTTPSYVAFTDTERLIGDAA |
| Q08169 | LLKDPNGNVVARNGGVPQLGNLTKHLQVFRDHLINQIPDKS |
| Q08169 | KMSWLFESEDVLLPSVYLRWNLTSGERVGLVGGRVKEALRI |
| P49370 | LLSLKDGKYKKRNGGVPQEGNITIHLQKFIENLDKIYPNRN |
| P49370 | NITIHLQKFIENLDKIYPNRNFSGIGVIDFERWRPIFRQNW |
| P49370 | DFERWRPIFRQNWGNMKIHKNFSIDLVRNEHPTWNKKMIEL |
| Q5D7H4 | GETISLFYDPGNFPSMVLLKNGTYEIRNEGVPQKGNLTIHL |
| Q5D7H4 | MVLLKNGTYEIRNEGVPQKGNLTIHLEQFTKELDEIYPKKI |
| P01815 | PPGKGLEWLARIDWDDDKYYNTSLETRLTISKDTSRNQVVL |
| P01763 | GSGSTIYYADSVKGRFTISRNBSKNSLYLQMSSLRAEDTAV |
| P01773 | GGGVVQAGTSLRLSCTASAFNLSDYAMHWVRQAPGKGLZWV |
| P01756 | KWVKQSHGKSLEWIGDINPNNGGTSYNQKFKGKATLTVDKS |
| Q12794 | LGTYPYYTPTGEPVFGGLPQNASLIAHLARTFQDILAAIPA |
| Q12794 | RGLWGFYGFPDCYNYDFLSPNYTGQCPSGIRAQNDQLGWLW |
| Q12794 | KESCQAIKEYMDTTLGPFILNVTSGALLCSQALCSGHGRCV |
| Q2M3T9 | RLNLKMFPVIGSPLAKARGQNVTIFYVNRLGYYPWYTSQGV |

| | |
|---|---|
| Q2M3T9 | LGYYPWYTSQGVPINGGLPQNISLQVHLEKADQDINYYIPA |
| Q2M3T9 | WNSKDVYRQKSRKLISDMGKNVSATDIEYLAKVTFEESAKA |
| Q2M3T9 | STIGESAALGAAGIVIWGDMNLTASKANCTKVKQFVSSDLG |
| I0CME7 | FNHPDNDGETQRILKKHRPENFTGLGVLDFETWRAIYSTNF |
| I0CME7 | DFETWRAIYSTNFGPMTIYQNESVKLVKEQHPDYDQKKLTK |
| I0CME7 | QWQCQQLQEHIRTVLGPLVKNVTQMMTDCSRAICEGHGRCV |
| O35632 | PLDLRAFDVKATPNEGFFNQNITTFYYDRLGLYPRFDAAGT |
| O35632 | LGLYPRFDAAGTSVHGGVPQNGSLCAHLPMLKESVERYIQT |
| O35632 | METCQYLKNYLTQLLVPYIVNVSWATQYCSWTQCHGHGRCV |
| O35632 | CHGHGRCVRRNPSANTFLHLNASSFRLVPGHTPSEPQLRPE |
| O35632 | QCYLGWGGEQCQRNYKGAAGNASRAWAGSHLTSLLGLVAVA |
| Q7Z139 | PWWTDALYWLPRGVSWSDMYNKTTEPGYMYPHYSHLWMTVL |
| Q9JKR6 | RFQISPQLQFSPEEVLGMVLNYSRSLAEDFAEQPIKDAVIT |
| Q9JKR6 | LINDNTATALSYGVFRRKDINSTAQNVMFYDMGSGSTVCTI |
| Q9JKR6 | INYGDLGFLGPEDLRVFGSQNLTTVKLKGVGESFKKYPDYE |
| Q9JKR6 | KLGNTISSLFGGGTSSDAKENGTDAVQEEEESPAEGSKDEP |
| Q9JKR6 | FRVEERRKWPERLSALDNLLNHSSIFLKGARLIPEMDQVFT |
| Q9JKR6 | IPEMDQVFTEVEMTTLEKVINDTWAWKNATLAEQAKLPATE |
| Q9JKR6 | FTEVEMTTLEKVINDTWAWKNATLAEQAKLPATEKPVLLSK |
| Q9JKR6 | VQYLLNKAKFTKPRPRPKDKNGTRAEPPLNASAGDQEEKVI |
| Q9JKR6 | FTKPRPRPKDKNGTRAEPPLNASAGDQEEKVIPPAGQTEEA |
| Q9Y4L1 | HFQISSQLQFSPEEVLGMVLNYSRSLAEDFAEQPIKDAVIT |
| Q9Y4L1 | LINDNTATALSYGVFRRKDINTTAQNIMFYDMGSGSTVCTI |
| Q9Y4L1 | INYGDLGFLGPEDLRVFGSQNLTTVKLKGVGDSFKKYPDYE |
| Q9Y4L1 | KLGNTISSLFGGGTTPDAKENGTDTVQEEEESPAEGSKDEP |
| Q9Y4L1 | FRVEERKKWPERLSALDNLLNHSSMFLKGARLIPEMDQIFT |
| Q9Y4L1 | IPEMDQIFTEVEMTTLEKVINETWAWKNATLAEQAKLPATE |
| Q9Y4L1 | FTEVEMTTLEKVINETWAWKNATLAEQAKLPATEKPVLLSK |
| Q9Y4L1 | VQYLLNKAKFTKPRPRPKDKNGTRAEPPLNASASDQGEKVI |
| Q9Y4L1 | FTKPRPRPKDKNGTRAEPPLNASASDQGEKVIPPAGQTEDA |
| Q14627 | FDLNKGIEAKIHTLLPWQCTNGSEVQSSWAETTYWISPQGI |
| Q14627 | IGCRFPYLEASDYKDFYICVNGSSENKPIRSSYFTFQLQNI |
| Q14627 | FDYEIEIREDDTTLVTATVENETYTLKTTNETRQLCFVVRS |
| Q14627 | DDTTLVTATVENETYTLKTTNETRQLCFVVRSKVNIYCSDD |
| Q96F46 | GGASLRLLDHRALVCSQPGLNCTVKNSTCLDDSWIHPRNLT |
| Q96F46 | RLLDHRALVCSQPGLNCTVKNSTCLDDSWIHPRNLTPSSPK |
| Q96F46 | GLNCTVKNSTCLDDSWIHPRNLTPSSPKDLQIQLHFAHTQQ |
| Q96F46 | EHARMKVTTPCMSSGSLWDPNITVETLEAHQLRVSFTLWNE |
| Q96F46 | PNITVETLEAHQLRVSFTLWNESTHYQILLTSFPHMENHSC |
| Q96F46 | TLWNESTHYQILLTSFPHMENHSCFEHMHHIPAPRPEEFHQ |
| Q96F46 | CFEHMHHIPAPRPEEFHQRSNVTLTLRNLKGCCRHQVQIQP |

| | |
|---|---|
| O95998 | PAAKQCPALEVTWPEVEVPLNGTLSLSCVACSRFPNFSILY |
| O95998 | VEVPLNGTLSLSCVACSRFPNFSILYWLGNGSFIEHLPGRL |
| O95998 | SLSCVACSRFPNFSILYWLGNGSFIEHLPGRLWEGSTSRER |
| O95998 | GTQLCKALVLEQLTPALHSTNFSCVLVDPEQVVQRHVVLAQ |
| P62757 | SCQLGYSQGPCLGMFKRYFYNGTSMACETFYYGGCMGNGNN |
| P62756 | SCQLGYSQGPCLGMFKRYFYNGTSMACETFYYGGCMGNGNN |
| P04365 | SCQLDHAQGPCLGMISRYFYNGTSMACETFQYGGCLGNGNN |
| P17936 | PSPDEARPLQALLDGRGLCVNASAVSRLRAYLLPAPPAPGN |
| P17936 | NASAVSRLRAYLLPAPPAPGNASESEEDRSAGSVESPSVSS |
| P17936 | HAKDSQRYKVDYESQSTDTQNFSSESKRETEYGPCRREMED |
| Q28893 | EIEAIQESLQPSDKDEGDHPNNSFSPCSAHDRKCLQKHLAK |
| P00976 | LCQLPQARGPCKAALLRYFYNSTSNACEPFTYGGCQGNNBN |
| P05155 | LTLLTLLLLLLAGDRASSNPNATSSSSQDPESLQDRGEGKV |
| P05155 | VISKMLFVEPILEVSSLPTTNSTTNSATKITANTTDEPTTQ |
| P05155 | EVSSLPTTNSTTNSATKITANTTDEPTTQPTTEPTTQPTIQ |
| P05155 | VTSVSQIFHSPDLAIRDTFVNASRTLYSSSPRVLSNNSDAN |
| P05155 | RDTFVNASRTLYSSSPRVLSNNSDANLELINTWVAKNTNNK |
| P05155 | SNNSDANLELINTWVAKNTNNKISRLLDSLPSDTRLVLLNA |
| P05155 | VAHFIDQTLKAKVGQLQLSHNLSLVILVPQNLKHRLEDMEQ |
| P97290 | PEPTVLPSTWPTTSVAITITNDTMGKVANESFSQHSQPAAQ |
| P97290 | TWPTTSVAITITNDTMGKVANESFSQHSQPAAQLPTDSPGQ |
| P97290 | SQHSQPAAQLPTDSPGQPPLNSSSQPSTASDLPTQATTEPF |
| P97290 | VTSVSQIFHSPDLAIRDTYVNASQSLYGSSPRVLGPDSAAN |
| P97290 | VAQFDDHTLKAKVGQLQLSHNLSFVIVVPVFPKHQLKDVEK |
| P05362 | YWTPERVELAPLPSWQPVGKNLTLRCQVEGGAPRANLTVVL |
| P05362 | QPVGKNLTLRCQVEGGAPRANLTVVLLRGEKELKREPAVGE |
| P05362 | VGEPAEVTTTVLVRRDHHGANFSCRTELDLRPQGLELFENT |
| P05362 | ANFSCRTELDLRPQGLELFENTSAPYQLQTFVLPATPPQLV |
| P05362 | DGLFPVSEAQVHLALGDQRLNPTVTYGNDSFSAKASVSVTA |
| P05362 | EAQVHLALGDQRLNPTVTYGNDSFSAKASVSVTAEDEGTQR |
| P05362 | VSVTAEDEGTQRLTCAVILGNQSQETLQTVTIYSFPAPNVI |
| P05362 | NGRSFSCSATLEVAGQLIHKNQTRELRVLYGPRLDERDCPG |
| P05362 | QTRELRVLYGPRLDERDCPGNWTWPENSQQTPMCQAWGNPL |
| P13597 | AQVSIHPREAFLPQGGSVQVNCSSSCKEDLSLGLETQWLKD |
| P13597 | PKDPKEITFTVLASRGDHGANFSCRTELDLRPQGLALFSNV |
| P13597 | ANFSCRTELDLRPQGLALFSNVSEARSLRTFDLPATIPKLD |
| P13597 | ASEARIYLELGGQMPTQESTNSSDSVSATALVEVTEEFDRT |
| P13597 | RCVLELADQILETQRTLTVYNFSAPVLTLSQLEVSEGSQVT |
| P13597 | VVLLSGVEPRPPTPQVQFTLNASSEDHKRSFFCSAALEVAG |
| P13597 | HKRSFFCSAALEVAGKFLFKNQTLELHVLYGPRLDETDCLG |
| P13597 | QTLELHVLYGPRLDETDCLGNWTWQEGSQQTLKCQAWGNPS |

| | |
|---|---|
| P13597 | RKADGALLPIGVVKSVKQEMNGTYVCHAFSSHGNVTRNVYL |
| P13597 | KSVKQEMNGTYVCHAFSSHGNVTRNVYLTVLYHSQNNWTII |
| P13598 | FEVHVRPKKLAVEPKGSLEVNCSTTCNQPEVGGLETSLDKI |
| P13598 | TSLDKILLDEQAQWKHYLVSNISHDTVLQCHFTCSGKQESM |
| P13598 | HDTVLQCHFTCSGKQESMNSNVSVYQPPRQVILTLQPTLVA |
| P13598 | ECRVPTVEPLDSLTLFLFRGNETLHYETFGKAAPAPQEATA |
| P13598 | LHYETFGKAAPAPQEATATFNSTADREDGHRNFSCLAVLDL |
| P13598 | APQEATATFNSTADREDGHRNFSCLAVLDLMSRGGNIFHKH |
| P32942 | FLLRVEPQNPVLSAGGSLFVNCSTDCPSSEKIALETSLSKE |
| P32942 | ALETSLSKELVASGMGWAAFNLSNVTGNSRILCSVYCNGSQ |
| P32942 | TSLSKELVASGMGWAAFNLSNVTGNSRILCSVYCNGSQITG |
| P32942 | KELVASGMGWAAFNLSNVTGNSRILCSVYCNGSQITGSSNI |
| P32942 | AAFNLSNVTGNSRILCSVYCNGSQITGSSNITVYRLPERVE |
| P32942 | GNSRILCSVYCNGSQITGSSNITVYRLPERVELAPLPPWQP |
| P32942 | YRLPERVELAPLPPWQPVGQNFTLRCQVEDGSPRTSLTVVL |
| P32942 | APFSCRTELDMQPQGLGLFVNTSAPRQLRTFVLPVTPPRLV |
| P32942 | DGLFPASEAQVYLALGDQMLNATVMNHGDTLTATATATARA |
| P32942 | TATATATARADQEGAREIVCNVTLGGERREARENLTVFSFL |
| P32942 | GAREIVCNVTLGGERREARENLTVFSFLGPIVNLSEPTAHE |
| P32942 | GERREARENLTVFSFLGPIVNLSEPTAHEGSTVTVSCMAGA |
| P32942 | QVTLDGVPAAAPGQPAQLQLNATESDDGRSFFCSATLEVDG |
| P32942 | DGRSFFCSATLEVDGEFLHRNSSVQLRVLYGPKIDRATCPQ |
| P32942 | LRCLKEGSSREVPVGIPFFVNVTHNGTYQCQASSSRGKYTL |
| P32942 | KEGSSREVPVGIPFFVNVTHNGTYQCQASSSRGKYTLVVVM |
| Q9UMF0 | FWADLQPRVAFVERGGSLWLNCSTNCPRPERGGLETSLRRN |
| Q9UMF0 | NCSTNCPRPERGGLETSLRRNGTQRGLRWLARQLVDIREPE |
| Q9UMF0 | FQRPDRVELMPLPPWQPVGENFTLSCRVPGAGPRASLTLTL |
| Q9UMF0 | RARGAVLTATVLARREDHGANFSCRAELDLRPHGLGLFENS |
| Q9UMF0 | ANFSCRAELDLRPHGLGLFENSSAPRELRTFSLSPDAPRLA |
| Q9UMF0 | VATATATASAEQEGARQLVCNVTLGGENRETRENVTIYSFP |
| Q9UMF0 | GARQLVCNVTLGGENRETRENVTIYSFPAPLLTLSEPSVSE |
| Q9UMF0 | LVTLEGVPAAVPGQPAQLQLNATENDDRRSFFCDATLDVDG |
| Q9UMF0 | DRRSFFCDATLDVDGETLIKNRSAELRVLYAPRLDDSDCPR |
| Q9UMF0 | KNVAVTVEYGPRFEEPSCPSNWTWVEGSGRLFSCEVDGKPQ |
| Q9UMF0 | DPSPRAPRIPRVLAPGIYVCNATNRHGSVAKTVVVSAESPP |
| Q9UMF0 | YRPVVAELAASPPGGVRPGGNFTLTCRAEAWPPAQISWRAP |
| Q9UMF0 | PPAQISWRAPPGALNIGLSSNNSTLSVAGAMGSHGGEYECA |
| Q9UMF0 | PAQISWRAPPGALNIGLSSNNSTLSVAGAMGSHGGEYECAA |
| Q60625 | FWADLQPRVALVERGGSLWLNCSTNCPRPERGGLETSLRRN |
| Q60625 | NCSTNCPRPERGGLETSLRRNGTQRGLRWLARQLVDIREPE |
| Q60625 | FQRPDRVELVPLPSWQPVGENFTLSCRVPGAGPRASLTLTL |

| | |
|---|---|
| Q60625 | RARGAMLTARVLARREDHRVNFSCLAELDLRPHGLGLFANS |
| Q60625 | VNFSCLAELDLRPHGLGLFANSSAPRQLRTFAMPPHSPSLI |
| Q60625 | LFPAPEAGVYLSLGDQRLNPNVTLDGDSLVATATATASAEQ |
| Q60625 | GTKQLMCVVTLGGESRETQENLTVYSFPTPLLTLSEPEAPE |
| Q60625 | LVTLEGIPAAVPGQPAELQLNVTKNDDKRGFFCDAALDVDG |
| Q60625 | DKRGFFCDAALDVDGETLRKNQSSELRVLYAPRLDDLDCPR |
| Q60625 | KNVAVTVEYGPSFEELGCPSNWTWVEGSGKLFSCEVDGKPE |
| Q60625 | NSGPRNSMTPGNLSPGIYLCNATNRHGSTVKTVVVSAESPP |
| Q60625 | EYRPVVAELAASPPSVRPGGNFTLTCRAEAWPPAQISWRAP |
| Q60625 | PPAQISWRAPPGALNLGLSSNNSTLSVAGAMGSHGGEYECA |
| Q60625 | PAQISWRAPPGALNLGLSSNNSTLSVAGAMGSHGGEYECAA |
| Q9DBD0 | ESSAKDLLFSDDTECLSNLQNKTTYKTYLGPQYLTLMDNFR |
| O75144 | VYVYWQTSESKTVVTYHIPQNSSLENVDSRYRNRALMSPAG |
| O75144 | NRALMSPAGMLRGDFSLRLFNVTPQDEQKFHCLVLSQSLGF |
| O75144 | SQSLGFQEVLSVEVTLHVAANFSVPVVSAPHSPSQDELTFT |
| O75144 | ELTFTCTSINGYPRPNVYWINKTDNSLLDQALQNDTVFLNM |
| O75144 | RPNVYWINKTDNSLLDQALQNDTVFLNMRGLYDVVSVLRIA |
| O75144 | IARTPSVNIGCCIENVLLQQNLTVGSQTGNDIGERDKITEN |
| Q8MM24 | GGDRDVDEAHPNKYVELLEANRTAQQNFIDSSMILLKRNGF |
| Q8MM24 | IKNAFRSANLMLSLTVLPNVNSTWYFDVPKLHPQFDYINLA |
| Q8MM24 | QIQSAEGLLSWPEICSKLSQNASAQYRGELAPLRKVTDLTQ |
| Q9V3D4 | VKDSLRADGFLLSLTVLPNVNSTWYFDIPALNGLVDFVNLA |
| P22304 | RRPDTTRLYDFNSYWRVHAGNFSTIPQYFKENGYVTMSVGK |
| P22304 | KENGYVTMSVGKVFHPGISSNHTDDSPYSWSFPPYHPSSEK |
| P22304 | HKPHIPFRYPKEFQKLYPLENITLAPDPEVPDGLPPVAYNP |
| P22304 | PPVAYNPWMDIRQREDVQALNISVPYGPIPVDFQRKIRQSY |
| P22304 | SYLDTQVGRLLSALDDLQLANSTIIAFTSDHGWALGEHGEW |
| P22304 | RTIDYRYTVWVGFNPDEFLANFSDIHAGELYFVDSDPLQDH |
| P35475 | THWLLELVTTRGSTGRGLSYNFTHLDGYLDLLRENQLLPGF |
| P35475 | AHVSKWNFETWNEPDHHDFDNVSMTMQGFLNYYDACSEGLR |
| P35475 | VTYAAMVVKVIAQHQNLLLANTTSAFPYALLSNDNAFLSYH |
| P35475 | FLSYHPHPFAQRTLTARFQVNNTRPPHVQLLRKPVLTAMGL |
| P35475 | LLDEEQLWAEVSQAGTVLDSNHTVGVLASAHRPQGPADAWR |
| P35475 | ADAWRAAVLIYASDDTRAHPNRSVAVTLRLRGVPPGPGLVY |
| P84881 | YYVLPHIWPGPGGLSFEKTGNQTCPVSVFQLPRLPLEQNNG |
| P01570 | QAISVLHEMMQQTFNLFSTKNSSAAWDETLLEKFYIELFQQ |
| P01574 | YEMLQNIFAIFRQDSSSTGWNETIVENLLANVYHQINHLKT |
| P01575 | QERTNIRKCQELLEQLNGKINLTYRADFKIPMEMTEKMQKS |
| P01575 | SYTAFAIQEMLQNVFLVFRNNFSSTGWNETIVVRLLDELHQ |
| P01575 | QEMLQNVFLVFRNNFSSTGWNETIVVRLLDELHQQTVFLKT |
| P05000 | HEMLQQIFSLFHTERSSAAWNMTLLDQLHTGLHQQLQHLET |

| | |
|---|---|
| P01579 | VKEAENLKKYFNAGHSDVADNGTLFLGILKNWKEESDRKIM |
| P01579 | MNVKFFNSNKKKRDDFEKLTNYSVTDLNVQRKAIHELIQVM |
| P27352 | DHEVQPTLPSNPGPGPTSASNITVIYTINNQLRGVELLFNE |
| P27352 | SNITVIYTINNQLRGVELLFNETINVSVKSGSVLLVVLEEA |
| P27352 | VIYTINNQLRGVELLFNETINVSVKSGSVLLVVLEEAQRKN |
| O18023 | SKDVIWYKDGSQISKGSQFLNTTSEKAYKIQHSIEVDYEKG |
| O18023 | SCVDNQCCSCRREEFTLVLRNLTFDESGRYRCQLGNKSELL |
| O18023 | TLVLRNLTFDESGRYRCQLGNKSELLEFQVEVLESGLKGGF |
| O18023 | ELLEFQVEVLESGLKGGFHENISYDHSECCQEKGISPLCRG |
| O18023 | KVQIREKRSTMVIVTRDDVTNSTTIREFAFQNVNTTERCVT |
| O18023 | VTRDDVTNSTTIREFAFQNVNTTERCVTLSDLRSSTRYIVY |
| O18023 | GTSVPSVRNIASTNVHMVKNNASLPDSMKCCTDANVTSFCS |
| O18023 | VHMVKNNASLPDSMKCCTDANVTSFCSSKMCNVAEDPSSFS |
| O18023 | IATTCRAEWPKVSPCIADGRNHTDCCLKKGVQHDCLEICSG |
| O18023 | LQAIYQCIRQGYETHPSAPGNVTISELTAHSVTVQWTEPNS |
| O18023 | TAHSVTVQWTEPNSNAHLVENYTLFIRKNEHGEAVRTVKNV |
| O18023 | VELGLDPDSEYVLTLQSHSANGTSLPSTAKLFSTLPTTRPP |
| O18023 | FTSCCKEQKMPESCMSSCQYNMTLPESCKENLNTWVQCASE |
| O18023 | NIIVFRVQLFEKGGNLIKTENSSADIFRFIDLEPNKDYSVR |
| O18023 | KDYSVRVTAINFLGEGPPSWNATFTTKPAQIYEGDRPVAPE |
| O18023 | GDRPVAPEKLRISWNSGPRVNVTWDPVSVRRNAEVVTKPIE |
| O18023 | EYTIYYLDTEQSSTWTTLRTNQTWVVMRDLRKDALYYVYVT |
| O18023 | VMRDLRKDALYYVYVTAKEDNRTSRSSSIITILAQKDSPGL |
| O18023 | IVIEPDHKDGVFSPGEKISINCSLPNIKKHLNIDLTVGSHV |
| O18023 | TGDCLQACNIGRTSLSIKNQNCTRFAVSLLKCASDIRDHSD |
| O18016 | IATTCRAEWPKVSPCIADGRNHTDCCLKKGVQHDCLEICSG |
| O18016 | LQAIYQCIRQGYETHPSAPGNVTISELTAHSVTVQWTEPNS |
| O18016 | TAHSVTVQWTEPNSNAHLVENYTLFIRKNEHGEAVRTVKNV |
| O18016 | VELGLDPDSEYVLTLQSHSANGTSLPSTAKLFSTLPTTRPP |
| O18016 | FTSCCKEQKMPESCMSSCQYNMTLPESCKENLNTWVQCASE |
| O18016 | NIIVFRVQLFEKGGNLIKTENSSADIFRFIDLEPNKDYSVR |
| O18016 | KDYSVRVTAINFLGEGPPSWNATFTTKPAQIYEGDRPVAPE |
| O18016 | GDRPVAPEKLRISWNSGPRVNVTWDPVSVRRNAEVVTKPIE |
| O18016 | EYTIYYLDTEQSSTWTTLRTNQTWVVMRDLRKDALYYVYVT |
| O18016 | VMRDLRKDALYYVYVTAKEDNRTSRSSSIITILAQKDSPGL |
| O18016 | IVIEPDHKDGVFSPGEKISINCSLPNIKKHLNIDLTVGSHV |
| O18016 | TGDCLQACNIGRTSLSIKNQNCTRFAVSLLKCASDIRDHSD |
| P08069 | EICGPGIDIRNDYQQLKRLENCTVIEGYLHILLISKAEDYR |
| P08069 | TEYLLLFRVAGLESLGDLFPNLTVIRGWKLFYNYALVIFEM |
| P08069 | YALVIFEMTNLKDIGLYNLRNITRGAIRIEKNADLCYLSTV |
| P08069 | CTENNECCHPECLGSCSAPDNDTACVACRHYYYAGVCVPAC |

| | |
|---|---|
| P08069 | EGFVIHDGECMQECPSGFIRNGSQSMYCIPCEGPCPKVCEE |
| P08069 | VSLSFLKNLRLILGEEQLEGNYSFYVLDNQNLQQLWDWDHR |
| P08069 | YSFYVLDNQNLQQLWDWDHRNLTIKAGKMYFAFNPKLCVSE |
| P08069 | PPDYRDLISFTVYYKEAPFKNVTEYDGQDACGSNSWNMVDV |
| P08069 | TMVENDHIRGAKSEILYIRTNASVPSIPLDVLSASNSSSQL |
| P08069 | LYIRTNASVPSIPLDVLSASNSSSQLIVKWNPPSLPNGNLS |
| P08069 | ASNSSSQLIVKWNPPSLPNGNLSYYIVRWQRQPQDGYLYRH |
| P08069 | HNSIFVPRPERKRRDVMQVANTTMSSRSRNTTAADTYNITD |
| P08069 | ERKRRDVMQVANTTMSSRSRNTTAADTYNITDPEELETEYP |
| P08069 | QVANTTMSSRSRNTTAADTYNITDPEELETEYPFFESRVDN |
| P08069 | VSRQEYRKYGGAKLNRLNPGNYTARIQATSLSGNGSWTDPV |
| P08069 | LNRLNPGNYTARIQATSLSGNGSWTDPVFFYVQAKTGYENF |
| Q60751 | EICGPGIDIRNDYQQLKRLENCTVIEGFLHILLISKAEDYR |
| Q60751 | TEYLLLFRVAGLESLGDLFPNLTVIRGWKLFYNYALVIFEM |
| Q60751 | YALVIFEMTNLKDIGLYNLRNITRGAIRIEKNADLCYLSTI |
| Q60751 | TENNECCHPECLGSCHTPDDNTTCVACRHYYYKGVCVPACP |
| Q60751 | DGFVIHDDECMQECPSGFIRNSTQSMYCIPCEGPCPKVCGD |
| Q60751 | VSLSFLKNLRLILGEEQLEGNYSFYVLDNQNLQQLWDWNHR |
| Q60751 | YSFYVLDNQNLQQLWDWNHRNLTVRSGKMYFAFNPKLCVSE |
| Q60751 | PPDYRDLISFTVYYKEAPFKNVTEYDGQDACGSNSWNMVDV |
| Q60751 | TMVENDHIRGAKSEILYIRTNASVPSIPLDVLSASNSSSQL |
| Q60751 | LYIRTNASVPSIPLDVLSASNSSSQLIVKWNPPTLPNGNLS |
| Q60751 | LSASNSSSQLIVKWNPPTLPNGNLSYYIVRWQRQPQDGYLY |
| Q60751 | ASNSSSQLIVKWNPPTLPNGNLSYYIVRWQRQPQDGYLYRH |
| Q60751 | HNSIFVPRPERRRRDVMQVANTTMSSRSRNTTVADTYNITD |
| Q60751 | ERRRRDVMQVANTTMSSRSRNTTVADTYNITDPEEFETEYP |
| Q60751 | QVANTTMSSRSRNTTVADTYNITDPEEFETEYPFFESRVDN |
| Q60751 | VSRQEYRKYGGAKLNRLNPGNYTARIQATSLSGNGSWTDPV |
| Q60751 | LNRLNPGNYTARIQATSLSGNGSWTDPVFFYVPAKTTYENF |
| P01867 | WFVNNVEVHTAQTQTHREDYNSTIRVVSTLPIQHQDWMSGK |
| P01869 | WFVDDVEVHTAQTQPREEQFNSTFRSVSELPIMHQDWLNGK |
| P01876 | HPRLSLHRPALEDLLLGSEANLTCTLTGLRDASGVTFTWTP |
| P01878 | SSDPVIIGCLIHDYFPSGTMNVTWGKSGKDITTVNFPPALA |
| P01878 | GESVKCSVQHDSNPVQELDVNCSGPTPPPPITIPSCQPSLS |
| P01877 | LVQGFFPQEPLSVTWSESGQNVTARNFPPSQDASGDLYTTS |
| P01877 | LPATQCPDGKSVTCHVKHYTNPSQDVTVPCPVPPPPPCCHP |
| P01877 | HPRLSLHRPALEDLLLGSEANLTCTLTGLRDASGATFTWTP |
| P01877 | HGETFTCTAAHPELKTPLTANITKSGNTFRPEVHLLPPPSE |
| P01880 | EVAGKVPTGGVEEGLLERHSNGSQSQHSRLTLPRSLWNAGT |
| P01880 | VSGFSPPNILLMWLEDQREVNTSGFAPARPPPQPGSTTFWA |
| P01854 | CLATGYFPEPVMVTWDTGSLNGTTMTLPATTLTLSGHYATI |

| | |
|---|---|
| P01854 | AKQMFTCRVAHTPSSTDWVDNKTFSVCSRDFTPPTVKILQS |
| P01854 | HFPPTIQLLCLVSGYTPGTINITWLEDGQVMDVDLSTASTT |
| P01854 | RKSPTITCLVVDLAPSKGTVNLTWSRASGKPVNHSTRKEEK |
| P01854 | WSRASGKPVNHSTRKEEKQRNGTLTVTSTLPVGTRDWIEGE |
| P01857 | WYVDGVEVHNAKTKPREEQYNSTYRVVSVLTVLHQDWLNGK |
| P01868 | WFVDDVEVHTAQTQPREEQFNSTFRSVSELPIMHQDWLNGK |
| P01862 | WFVDNKPVGNAETKPRVEQYNTTFRVESVLPIQHQDWLRGK |
| P01859 | WYVDGVEVHNAKTKPREEQFNSTFRVVSVLTVVHQDWLNGK |
| P01860 | WYVDGVEVHNAKTKPREEQYNSTFRVVSVLTVLHQDWLNGK |
| P01860 | FYPSDIAVEWESSGQPENNYNTTPPMLDSDGSFFLYSKLTV |
| P01861 | WYVDGVEVHNAKTKPREEQFNSTYRVVSVLTVLHQDWLNGK |
| P03987 | WFVDNKEVHTAWTQPREAQYNSTFRVVSALPIQHQDWMRGK |
| P03987 | GEIFTCSVVHEALHNHHTQKNLSRSPELELNETCAEAQDGE |
| P03987 | EALHNHHTQKNLSRSPELELNETCAEAQDGELDGLWTTITI |
| P01871 | VGCLAQDFLPDSITLSWKYKNNSDISSTRGFPSVLRGGKYA |
| P01871 | WLGQSMFTCRVDHRGLTFQQNASSMCVPDQDTAIRVFAIPP |
| P01871 | YDSVTISWTRQNGEAVKTHTNISESHPNATFSAVGEASICE |
| P01871 | WTRQNGEAVKTHTNISESHPNATFSAVGEASICEDDWNSGE |
| P01872 | MGCLARDFLPSTISFTWNYQNNTEVIQGIRTFPTLRTGGKY |
| P01872 | WLNLNVYTCRVDHRGLTFLKNVSSTCAASPSTDILTFTIPP |
| P01872 | DILTFTIPPSFADIFLSKSANLTCLVSNLATYETLNISWAS |
| P01872 | LSKSANLTCLVSNLATYETLNISWASQSGEPLETKIKIMES |
| P01872 | WASQSGEPLETKIKIMESHPNGTFSAKGVASVCVEDWNNRK |
| P01591 | PNEDIVERNIRIIVPLNNRENISDPTSPLRTRFVYHLSDLC |
| P34595 | FAPFPQLEHIILRDGDLESLNGTVIHPTLKVLSIENSELTS |
| P34595 | EVPNTLRVLNVSRNRLTSFENISTKLTDLDISFNKLSLWPS |
| P34595 | KCLLQWSLPVYAQTSIRKDCNLTREEIESASCKIGVVANDT |
| P34595 | DCNLTREEIESASCKIGVVANDTGIQYGKYEKPTAISCFSY |
| Q6ZQA6 | VTVQEGPLYRTESSHITIWCNVSGYQGPSEQNFQWSIYLPS |
| Q6ZQA6 | KNIPIVVLPLKSSISVEVASNASVVLEGEDLHFSCTVRTVG |
| Q6ZQA6 | VQGSLSVLETRQIQLECVVLNRTSVASQLLVEWFVWKPNHP |
| Q6ZQA6 | RLSPLLYRLTVLEASPQDTGNYSCHVEEWLPSPQKEWYRLT |
| Q969P0 | VLVPEGPLYRVAGTAVSISCNVTGYEGPAQQNFEWFLYRPE |
| Q969P0 | VTVGPGERRIGPGEPLELLCNVSGALPPAGRHAAYSVGWEM |
| Q969P0 | AVAWLAGGTVYRGETASLLCNISVRGGPPGLRLAASWWVER |
| Q14623 | TQDPPRRLALTPAHLLFTADNHTEPAARFRATFASHVQPGQ |
| Q9VM64 | RSSRSPGAGFKYLKTGTAKANVSQEAAISRLRVLVRPDTLG |
| Q9VM64 | RNGVEIKPEFIGTNGNLFLSNVSSESSGSYSCQATNPASGE |
| Q9VM64 | HRPKPNQGSRQKQMYPPTPPNVTRLSDESVMLRWMVPRNDG |
| Q9VM64 | QHTYRFRILAVYSNNDNKESNTSAKFYLQPGAALDPMPVPE |
| Q9VM64 | STTEEPTLQMGDRDTTTPSHNETFNMSPMLTGTIGGGAVLI |

| | |
|---|---|
| Q70UQ0 | EEINEVKTWSNRITEKQDILNNSLTTLSQDITKVDQSTTSM |
| Q70UQ0 | LVTDLLQREKEIAFLSEKISNLTIVQAEIKDIKDEIAHISD |
| P29460 | DILKDQKEPKNKTFLRCEAKNYSGRFTCWWLTTISTDLTFS |
| P29460 | AEESLPIEVMVDAVHKLKYENYTSSFFIRDIIKPDPPKNLQ |
| Q96PD4 | NENQRVSMSRNIESRSTSPWNYTVTWDPNRYPSEVVQAQCR |
| Q9NPH3 | VFEDEPARIKCPLFEHFLKFNYSTAHSAGLTLIWYWTRQDR |
| Q9NPH3 | LPENRISKEKDVLWFRPTLLNDTGNYTCMLRNTTYCSKVAF |
| Q9NPH3 | RISKEKDVLWFRPTLLNDTGNYTCMLRNTTYCSKVAFPLEV |
| Q9NPH3 | VLWFRPTLLNDTGNYTCMLRNTTYCSKVAFPLEVVQKDSCF |
| Q9NPH3 | TWYMGCYKIQNFNNVIPEGMNLSFLIALISNNGNYTCVVTY |
| Q9NPH3 | NVIPEGMNLSFLIALISNNGNYTCVVTYPENGRTFHLTRTL |
| Q9NPH3 | EVWWTIDGKKPDDITIDVTINESISHSRTEDETRTQILSIK |
| P27930 | VALRCPQVPYWLWASVSPRINLTWHKNDSARTVPGEEETRM |
| P27930 | QVPYWLWASVSPRINLTWHKNDSARTVPGEEETRMWAQDGA |
| P27930 | ALWLLPALQEDSGTYVCTTRNASYCDKMSIELRVFENTDAF |
| P27930 | LEDAGYYRCVLTFAHEGQQYNITRSIELRIKKKKEETIPVI |
| P27930 | PCKVFLGTGTPLTTMLWWTANDTHIESAYPGGRVTEGPRQE |
| P27931 | ILWILPAVQQDSGTYICTFRNASHCEQMSVELKVFKNTEAS |
| P27931 | LDKGNKEFLSAGDPTRLLISNTSMDDAGYYRCVMTFTYNGQ |
| P27931 | MDDAGYYRCVMTFTYNGQEYNITRNIELRVKGTTTEPIPVI |
| P27931 | PCKVFLGTGTSSNTIVWWLANSTFISAAYPRGRVTEGLHHQ |
| P18510 | KMCLSCVKSGDETRLQLEAVNITDLSENRKQDKRFAFIRSD |
| Q9HBE5 | DQYEELKDEATSCSLHRSAHNATHATYTCHMDVFHFMADDI |
| Q9HBE5 | ATYTCHMDVFHFMADDIFSVNITDQSGNYSQECGSFLLAES |
| Q9HBE5 | DVFHFMADDIFSVNITDQSGNYSQECGSFLLAESIKPAPPF |
| Q9HBE5 | YSQECGSFLLAESIKPAPPFNVTVTFSGQYNISWRSDYEDP |
| Q9HBE5 | AESIKPAPPFNVTVTFSGQYNISWRSDYEDPAFYMLKGKLQ |
| Q9GZX6 | APISSHCRLDKSNFQQPYITNRTFMLAKEASLADNNTDVRL |
| Q9GZX6 | QQPYITNRTFMLAKEASLADNNTDVRLIGEKLFHGVSMSER |
| Q9GZX6 | EKLFHGVSMSERCYLMKQVLNFTLEEVLFPQSDRFQPYMQE |
| Q5VWK5 | DAVIALYILFSWCHGGITNINCSGHIWVEPATIFKMGMNIS |
| Q5VWK5 | NINCSGHIWVEPATIFKMGMNISIYCQAAIKNCQPRKLHFY |
| Q5VWK5 | PRKLHFYKNGIKERFQITRINKTTARLWYKNFLEPHASMYC |
| Q5VWK5 | SGYPPDIPDEVTCVIYEYSGNMTCTWNAGKLTYIDTKYVVH |
| Q5VWK5 | VHVKSLETEEEQQYLTSSYINISTDSLQGGKKYLVWVQAAN |
| Q5VWK5 | IHLDDIVIPSAAVISRAETINATVPKTIIYWDSQTTIEKVS |
| Q5VWK5 | WDSQTTIEKVSCEMRYKATTNQTWNVKEFDTNFTYVQQSEF |
| Q5VWK5 | CEMRYKATTNQTWNVKEFDTNFTYVQQSEFYLEPNIKYVFQ |
| P01589 | ECKRGFRRIKSGSLYMLCTGNSSHSSWDNQCQCTSSATRNT |
| P01589 | GNSSHSSWDNQCQCTSSATRNTTKQVTPQPEEQKERKTTEM |
| P14784 | RLPLLILLLPLATSWASAAVNGTSQFTCFYNSRANISCVWS |

| | |
|---|---|
| P14784 | WASAAVNGTSQFTCFYNSRANISCVWSQDGALQDTSCQVHA |
| P14784 | DGALQDTSCQVHAWPDRRRWNQTCELLPVSQASWACNLILG |
| P14784 | NLRLMAPISLQVVHVETHRCNISWEISQASHYFERHLEFEA |
| P31785 | PSLPFTSLLFLQLPLLGVGLNTTILTPNGNEDTTADFFLTT |
| P31785 | SVSTLPLPEVQCFVFNVEYMNCTWNSSSEPQPTNLTLHYWY |
| P31785 | LPLPEVQCFVFNVEYMNCTWNSSSEPQPTNLTLHYWYKNSD |
| P31785 | VFNVEYMNCTWNSSSEPQPTNLTLHYWYKNSDNDKVQKCSH |
| P31785 | RRQATQMLKLQNLVIPWAPENLTLHKLSESQLELNWNNRFL |
| P31785 | PLCGSAQHWSEWSHPIHWGSNTSKENPFLFALEAVVISVGS |
| P32927 | DYTSHITCRWADTQDAQRLVNVTLIRRVNEDLLEPVSCDLS |
| P32927 | FEVVYKRLQDSWEDAAILLSNTSQATLGPEHLMPSSTYVAR |
| P32927 | RRAEKHIKSSVNIQMAPPSLNVTKDGDSYSLRWETMKMRYE |
| P01586 | HLGLQASISGRDTHRLTRTLNCSSIVKEIIGKLPEPELKTD |
| P01586 | DRYVIKSNLQKLNCCLPTSANDSALPGVFIRDLDDFRKKLR |
| P16382 | VDCSSQLCLHYRLMFFEFSENLTCIPRNSASTVCVCHMEMN |
| P16382 | RQLWQGSFSPSGNVKPLAPDNLTLHTNVSDEWLLTWNNLYP |
| P16382 | SFSPSGNVKPLAPDNLTLHTNVSDEWLLTWNNLYPSNNLLY |
| P16382 | TWNNLYPSNNLLYKDLISMVNISREDNPAEFIVYNVTYKEP |
| P05112 | TEQKTLCTELTVTDIFAASKNTTEKETFCRAATVLRQFYSH |
| P04401 | STVVKETLTQLSAHRALLTSNETMRLPVPTHKNHQLCIGEI |
| P04401 | THKNHQLCIGEIFQGLDILKNQTVRGGTVEMLFQNLSLIKK |
| P04401 | GLDILKNQTVRGGTVEMLFQNLSLIKKYIDRQKEKCGEERR |
| P05113 | SALVKETLALLSTHRTLLIANETLRIPVPVHKNHQLCTEEI |
| P08887 | LTSLPGDSVTLTCPGVEPEDNATVHWVLRKPAAGSHPSRWA |
| P08887 | RWAGMGRRLLLRSVQLHDSGNYSCYRAGRPAGTVHLLVDVP |
| P08887 | KFSKTQTFQGCGILQPDPPANITVTAVARNPRWLSVTWQDP |
| Q00560 | QLLEPCGYIYPEFPVVQRGSNFTAICVLKEACLQHYYVNAS |
| Q00560 | GSNFTAICVLKEACLQHYYVNASYIVWKTNHAAVPREQVTV |
| Q00560 | SYIVWKTNHAAVPREQVTVINRTTSSVTFTDVVLPSVQLTC |
| Q00560 | IEQNVYGVTMLSGFPPDKPTNLTCIVNEGKNMLCQWDPGRE |
| Q00560 | NEGKNMLCQWDPGRETYLETNYTLKSEWATEKFPDCQSKHG |
| Q00560 | VSSESINFDPVDKVKPTPPYNLSVTNSEELSSILKLSWVSS |
| Q00560 | LTQSKSVSQTYTVTGTELTVNLTNDRYVASLAARNKVGKSA |
| Q00560 | CVLSENAPCVEDWQQEDATVNRTHLRGRLLESKCYQITVTP |
| Q00560 | NEAVLAWDQIPVDDQNGFIRNYSISYRTSVGKEMVVHVDSS |
| P40189 | ELLDPCGYISPESPVVQLHSNFTAVCVLKEKCMDYFHVNAN |
| P40189 | NYIVWKTNHFTIPKEQYTIINRTASSVTFTDIASLNIQLTC |
| P40189 | LEQNVYGITIISGLPPEKPKNLSCIVNEGKKMRCEWDGGRE |
| P40189 | NEGKKMRCEWDGGRETHLETNFTLKSEWATHKFADCKAKRD |
| P40189 | VTSDHINFDPVYKVKPNPPHNLSVINSEELSSILKLTWTNP |
| P40189 | ANGKILDYEVTLTRWKSHLQNYTVNATKLTVNLTNDRYLAT |

| | |
|---|---|
| P40189 | ILDYEVTLTRWKSHLQNYTVNATKLTVNLTNDRYLATLTVR |
| P40189 | LTRWKSHLQNYTVNATKLTVNLTNDRYLATLTVRNLVGKSD |
| P40189 | NEAVLEWDQLPVDVQNGFIRNYTIFYRTIIGNETAVNVDSS |
| P40189 | VDVQNGFIRNYTIFYRTIIGNETAVNVDSSHTEYTLSSLTS |
| P16871 | DLEDAELDDYSFSCYSQLEVNGSQHSLTCAFEDPDVNTTNL |
| P16871 | QLEVNGSQHSLTCAFEDPDVNTTNLEFEICGALVEVKCLNF |
| P16871 | APFDLSVIYREGANDFVVTFNTSHLQKKYVKVLMHDVAYRQ |
| P16871 | VLMHDVAYRQEKDENKWTHVNLSSTKLTLLQRKLQPAAMYE |
| P16871 | YFKGFWSEWSPSYYFRTPEINNSSGEMDPILLTISILSFFS |
| P16871 | FKGFWSEWSPSYYFRTPEINNSSGEMDPILLTISILSFFSV |
| P15247 | TNYLIENLKDDPPSKCSCSGNVTSCLCLSVPTDDCTTPCYR |
| P15247 | SVPTDDCTTPCYREGLLQLTNATQKSRLLPVFHRVKRIVEV |
| P15247 | QKSRLLPVFHRVKRIVEVLKNITCPSFSCEKPCNQTMAGNT |
| P15247 | RIVEVLKNITCPSFSCEKPCNQTMAGNTLSFLKSLLGTFQK |
| P05231 | IDKQIRYILDGISALRKETCNKSNMCESSKEALAENNLNLP |
| Q01638 | VRCPRQGKPSYTVDWYYSQTNKSIPTQERNRVFASGQLLKF |
| Q01638 | LPAAVADSGIYTCIVRSPTFNRTGYANVTIYKKQSDCNVPD |
| Q01638 | DSGIYTCIVRSPTFNRTGYANVTIYKKQSDCNVPDYLMYST |
| Q01638 | STVSGSEKNSKIYCPTIDLYNWTAPLEWFKNCQALQGSRYR |
| Q01638 | VMTEDAGDYTCKFIHNENGANYSVTATRSFTVKDEQGFSLF |
| Q01638 | PVIGAPAQNEIKEVEIGKNANLTCSACFGKGTQFLAAVLWQ |
| Q01638 | TCSACFGKGTQFLAAVLWQLNGTKITDFGEPRIQQEEGQNQ |
| Q01638 | LNGTKITDFGEPRIQQEEGQNQSFSNGLACLDMVLRIADVK |
| P49049 | LWVARVFQILRVPEEYVQKINSTIISYTANTTTTGPSEPFL |
| P49049 | LRVPEEYVQKINSTIISYTANTTTTGPSEPFLLRLASRIPQ |
| P82176 | HEYYECGGACDNVCADLHIQNKTNCPIINIRCNDKCYCEDG |
| P82176 | CIDSPPPSPGCSCNSGYLRLNLTSPCIPICDCPQMQHSPDC |
| Q03600 | HWCEQVRFEYENVEDYAKRPNETRGRTVHPLGKNDQLLAST |
| Q03600 | HPLGKNDQLLASTIVSKGTKNGSALVCAPLIRYHQTAAYPQ |
| Q03600 | LPFIQDSSSKLTLNEDKFIINGTAMGSAFGYSIEVVDLNGD |
| Q03600 | AIPFRADTTARGFIEGSHSHNYSWPCGSNSHVQKRTYRQLI |
| Q03600 | KLTRGRTEGIGKALKFMAHVNSTSQETEEELKDNKWEAEVQ |
| Q03600 | KARAESELELEEDIGTMVRHNYTIINHGPWTVRNVEAHISW |
| Q03600 | HFDFIDANSAVVIDLRARLWNATFIEDYSDVESVKIRSFGK |
| P48551 | SCTFKISLRNFRSILSWELKNHSIVPTHYTLLYTIMSKPED |
| P48551 | TLLYTIMSKPEDLKVVKNCANTTRSFCDLTDEWRSTHEAYV |
| P48551 | TDEWRSTHEAYVTVLEGFSGNTTLFSCSHNFWLAIDMSFEP |
| P48551 | LVIEEQSEGIVKKHKPEIKGNMSGNFTYIIDKLIPNTNYCV |
| P48551 | EQSEGIVKKHKPEIKGNMSGNFTYIIDKLIPNTNYCVSVYL |
| O35664 | SLETITPSAFDGYPDEPCTINITIRNSRLILSWELENKSGP |
| O35664 | PCTINITIRNSRLILSWELENKSGPPANYTLWYTVMSKDEN |

| | |
|---|---|
| O35664 | IRNSRLILSWELENKSGPPANYTLWYTVMSKDENLTKVKNC |
| O35664 | NKSGPPANYTLWYTVMSKDENLTKVKNCSDTTKSSCDVTDK |
| O35664 | ANYTLWYTVMSKDENLTKVKNCSDTTKSSCDVTDKWLEGME |
| O35664 | PANAPLEPPEFEIVGFTDHINVTMEFPPVTSKIIQEKMKTT |
| O35664 | FVIKEQIGDSVRKKHEPKVNNVTGNFTFVLRDLLPKTNYCV |
| O35664 | EQIGDSVRKKHEPKVNNVTGNFTFVLRDLLPKTNYCVSLYF |
| P17181 | LKSPQKVEVDIIDDNFILRWNRSDESVGNVTFSFDYQKTGM |
| P17181 | VDIIDDNFILRWNRSDESVGNVTFSFDYQKTGMDNWIKLSG |
| P17181 | FSFDYQKTGMDNWIKLSGCQNITSTKCNFSSLKLNVYEEIK |
| P17181 | TGMDNWIKLSGCQNITSTKCNFSSLKLNVYEEIKLRIRAEK |
| P17181 | SSLKLNVYEEIKLRIRAEKENTSSWYEVDSFTPFRKAQIGP |
| P17181 | DSVMWALDGLSFTYSLVIWKNSSGVEERIENIYSRHKIYKL |
| P17181 | NIEVSVQNQNYVLKWDYTYANMTFQVQWLHAFLKRNPGNHL |
| P17181 | FPQNVFQKGIYLLRVQASDGNNTSFWSEEIKFDTEIQAFLL |
| P17181 | PQNVFQKGIYLLRVQASDGNNTSFWSEEIKFDTEIQAFLLP |
| P17181 | SGNTPVIQDYPLIYEIIFWENTSNAERKIIEKKTDVTVPNL |
| P17181 | LKPLTVYCVKARAHTMDEKLNKSSVFSDAVCEKTKPGNTSK |
| P17181 | EKLNKSSVFSDAVCEKTKPGNTSKIWLIVGICIALFALPFV |
| Q9WU62 | QAISEPDEEQLEDEELQPCQNKTPSPPCPANKVVRPLRTFL |
| Q9VR07 | IDCNNRWNTPDCWVPQRKGINASAPDTSRTPSEEFFENKVL |
| P05111 | VTSAQLWFHTGLDRQGTAASNSSEPLLGLLALSPGGPVAVP |
| P05111 | LLQRPPEEPAAHANCHRVALNISFQELGWERWIVYPPSFIF |
| P05111 | YPPSFIFHYCHGGCGLHIPPNLSLPVPGAPPTPAQPYSLLP |
| P58166 | LRGEKSGVLKLQLDCRPLEGNSTVTGQPRRLLDTAGHQQPF |
| Q8IU57 | PLLLCLLQAAPGRPRLAPPQNVTLLSQNFSVYLTWLPGLGN |
| Q8IU57 | QAAPGRPRLAPPQNVTLLSQNFSVYLTWLPGLGNPQDVTYF |
| Q8IU57 | EVEPAPPVLVLTQTEEILSANATYQLPPCMPPLDLKYEVAF |
| Q8IU57 | PCMPPLDLKYEVAFWKEGAGNKTLFPVTPHGQPVQITLQPA |
| Q968Y9 | TPDSSMDCYEENPPSQKTSINYSWISKKSSMTSLMLLLLFA |
| Q968Y9 | PQWSKLGDPNEKDLAGQRMVNCTVVEGSLTISFVLKHKTKA |
| Q968Y9 | HYLEEKNQEQGVERVQSCWSNTTCQKSCAYDRLLPTKEIGP |
| Q968Y9 | LLQRRCVTREQCLQLNPVLSNKTVPIKATAGLCSDKCPDGY |
| Q968Y9 | HVIDTFPKAQAIRLCNIIDGNLTIEIRGKQDSGMASELKDI |
| Q968Y9 | PIDQSEGTNGEKAICEDMAINVSITAVNADSVFFSWPSFNI |
| Q968Y9 | INVSITAVNADSVFFSWPSFNITDIDQRKFLGYELFFKEVP |
| Q968Y9 | DQRKFLGYELFFKEVPRIDENMTIEEDRSACVDSWQSVFKQ |
| Q968Y9 | PGTRIRLYEIYEPLPGSWAINVSALALDNSYVIRNLKHYTL |
| Q968Y9 | YVIRNLKHYTLYAISLSACQNMTVPGASCSISHRAGALKRT |
| Q968Y9 | SHRAGALKRTKHITDIDKVLNETIEWRFMNNSQQVNVTWDP |
| Q968Y9 | TKHITDIDKVLNETIEWRFMNNSQQVNVTWDPPTEVNGGIF |
| Q968Y9 | IDKVLNETIEWRFMNNSQQVNVTWDPPTEVNGGIFGYVVKL |

| | |
|---|---|
| P09208 | RQAVEAVDSPASSEEAYSSSNSSSCQASSEISAEEVWFLSH |
| P09208 | HHYQHHHQQHHQQHHQRQQANVSYTKFLLLLQTLAAATTRL |
| P09208 | ATPQQKQQEKDRHKCFHYKHNYSYSPGISLLLFILLANTLA |
| P09208 | VKPCKSMDIRNMVSHFNQLENCTVIEGFLLIDLINDASPLN |
| P09208 | NCTVIEGFLLIDLINDASPLNRSFPKLTEVTDYIIIYRVTG |
| P09208 | TDYIIIYRVTGLHSLSKIFPNLSVIRGNKLFDGYALVVYSN |
| P09208 | EKNHKLCYDRTIDWLEILAENETQLVVLTENGKEKECRLSK |
| P09208 | CPGEIRIEEGHDTTAIEGELNASCQLHNNRRLCWNSKLCQT |
| P09208 | DEHTCCSQDCLGGCVIDKNGNESCISCRNVSFNNICMDSCP |
| P09208 | DCLGGCVIDKNGNESCISCRNVSFNNICMDSCPKGYYQFDS |
| P09208 | ADKYALYVLDNRDLDELWGPNQTVFIRKGGVFFHFNPKLCV |
| P09208 | KSDVGADSNGNRGSCGTAVLNVTLQSVGANSAMLNVTTKVE |
| P09208 | CGTAVLNVTLQSVGANSAMLNVTTKVEIGEPQKPSNATIVF |
| P09208 | NSAMLNVTTKVEIGEPQKPSNATIVFKDPRAFIGFVFYHMI |
| P09208 | FKDPRAFIGFVFYHMIDPYGNSTKSSDDPCDDRWKVSSPEK |
| P09208 | KVSSPEKSGVMVLSNLIPYTNYSYYVRTMAISSELTNAESD |
| P09208 | PGRPSKVTEVVATAISDSKINVTWSYLDKPYGVLTRYFIKA |
| P09208 | MEFENALQNFIFVPNIRKSKNGSSDKSDGAEGAALDSNAIP |
| P09208 | EEDLSSNKQFYEVFAKELPPNQTHFVFEKLRHFTRYAIFVV |
| P09208 | TKRKKFADIVMDLKVDLEHANNTESPVRVRWTPPVDPNGEI |
| P09208 | KRKKFADIVMDLKVDLEHANNTESPVRVRWTPPVDPNGEIV |
| P09208 | YKLQKPDQVEEKKCIPAADFNQTAGYLIKLNEGLYSFRVRA |
| P06213 | LGAAGHLYPGEVCPGMDIRNNLTRLHELENCSVIEGHLQIL |
| P06213 | GEVCPGMDIRNNLTRLHELENCSVIEGHLQILLMFKTRPED |
| P06213 | TDYLLLFRVYGLESLKDLFPNLTVIRGSRLFFNYALVIFEM |
| P06213 | YALVIFEMVHLKELGLYNLMNITRGSVRIEKNNELCYLATI |
| P06213 | ICKSHGCTAEGLCCHSECLGNCSQPDDPTKCVACRNFYLDG |
| P06213 | GRCVETCPPPYYHFQDWRCVNFSFCQDLHHKCKNSRRQGCH |
| P06213 | HQYVIHNNKCIPECPSGYTMNSSNLLCTPCLGPCPKVCHLL |
| P06213 | GEKTIDSVTSAQELRGCTVINGSLIINIRGGNNLAAELEAN |
| P06213 | VSLSFFRKLRLIRGETLEIGNYSFYALDNQNLRQLWDWSKH |
| P06213 | YSFYALDNQNLRQLWDWSKHNLTITQGKLFFHYNPKLCLSE |
| P06213 | PPDFRDLLGFMLFYKEAPYQNVTEFDGQDACGSNSWTVVDI |
| P06213 | IYVQTDATNPSVPLDPISVSNSSSQIILKWKPPSDPNGNIT |
| P06213 | VSNSSSQIILKWKPPSDPNGNITHYLVFWERQAEDSELFEL |
| P06213 | LKLPSRTWSPPFESEDSQKHNQSEYEDSAGECCSCPKTDSQ |
| P06213 | GTGAEDPRPSRKRRSLGDVGNVTVAVPTVAAFPNTSSTSVP |
| P06213 | RSLGDVGNVTVAVPTVAAFPNTSSTSVPTSPEEHRPFEKVV |
| P06213 | VSRKHFALERGCRLRGLSPGNYSVRIRATSLAGNGSWTEPT |
| P06213 | LRGLSPGNYSVRIRATSLAGNGSWTEPTYFYVTDYLDVPSN |
| P15208 | VGTAGHLYPGEVCPGMDIRNNLTRLHELENCSVIEGHLQIL |

| | |
|---|---|
| P15208 | GEVCPGMDIRNNLTRLHELENCSVIEGHLQILLMFKTRPED |
| P15208 | TDYLLLFRVYGLESLKDLFPNLTVIRGSRLFFNYALVIFEM |
| P15208 | YALVIFEMVHLKELGLYNLMNITRGSVRIEKNNELCYLATI |
| P15208 | ICKSHGCTAEGLCCHKECLGNCSEPDDPTKCVACRNFYLDG |
| P15208 | GQCVETCPPPYYHFQDWRCVNFSFCQDLHFKCRNSRKPGCH |
| P15208 | HQYVIHNNKCIPECPSGYTMNSSNLMCTPCLGPCPKVCQIL |
| P15208 | GEKTIDSVTSAQELRGCTVINGSLIINIRGGNNLAAELEAN |
| P15208 | VSLSFFRKLHLIRGETLEIGNYSFYALDNQNLRQLWDWSKH |
| P15208 | YSFYALDNQNLRQLWDWSKHNLTITQGKLFFHYNPKLCLSE |
| P15208 | PPDFRDLLGFMLFYKEAPYQNVTEFDGQDACGSNSWTVVDI |
| P15208 | IYVQTDATNPSVPLDPISVSNSSSQIILKWKPPSDPNGNIT |
| P15208 | VSNSSSQIILKWKPPSDPNGNITHYLVYWERQAEDSELFEL |
| P15208 | LKLPSRTWSPPFESDDSQKHNQSEYDDSASECCSCPKTDSQ |
| P15208 | HNVVFVPRPSRKRRSLEEVGNVTATTLTLPDFPNVSSTIVP |
| P15208 | RSLEEVGNVTATTLTLPDFPNVSSTIVPTSQEEHRPFEKVV |
| P15208 | VSRKHFALERGCRLRGLSPGNYSVRVRATSLAGNGSWTEPT |
| P15208 | LRGLSPGNYSVRVRATSLAGNGSWTEPTYFYVTDYLDVPSN |
| O94220 | TAIADENGVEAFTGTAYYDPNNTSGLGDSANPPYLAWFTGY |
| O94220 | AIADENGVEAFTGTAYYDPNNTSGLGDSANPPYLAWFTGYT |
| O94220 | MVLAHGGQDKLSFWTSADTINWTWQSDLKSTSINGLSSDIT |
| O94220 | FVQQPITELDTISTSLQILANQTITPGQTLLSSIRGTALDV |
| Q96TU3 | QYHFSPQKNWMNDPNGLLYHNGTYHLFFQYNPGGIEWGNIS |
| Q96TU3 | YHNGTYHLFFQYNPGGIEWGNISWGHAISEDLTHWEEKPVA |
| Q96TU3 | RGFGSDVTEMYFSGSAVADVNNTSGFGKDGKTPLVAMYTSY |
| Q96TU3 | GFGSDVTEMYFSGSAVADVNNTSGFGKDGKTPLVAMYTSYY |
| Q96TU3 | NAQGGVWECPGLVKLPLDSGNSTKWVITSGLNPGGPPGTVG |
| Q96TU3 | FVGEFDGTTFTPDADTVYPGNSTANWMDWGPDFYAAAGYNG |
| Q96TU3 | ISNKRPIYSRTFKTLSEGSTNTTTTGETFKVDLSFSAKSKA |
| Q96TU3 | LSFSAKSKASTFAIALRASANFTEQTLVGYDFAKQQIFLDR |
| Q43866 | ILYTGIDPKNQQVQNIAEPKNLSDPYLREWKKSPLNPLMAP |
| Q43866 | REWKKSPLNPLMAPDAVNGINASSFRDPTTAWLGQDKKWRV |
| Q43866 | YASKTFFDSAKNRRILWGWTNESSSVEDDVEKGWSGIQTIP |
| Q43866 | EKADVIEPSWTDPQLICSKMNVSVKSGLGPFGLMVLASKNL |
| P26792 | TGIVEGPPKNVQVQNYAIPANLSDPYLRKWIKPDNNPLVVA |
| P26792 | YLRKWIKPDNNPLVVANNGENATAFRDPTTAWLDKSGHWKM |
| P26792 | RYEYYTVGTYLTDKDRYIPDNTSVDGWAGLRYDYGNFYASK |
| P26792 | YASKTFFDPSKNRRILWGWANESDSTAHDVAKGWAGIQLIP |
| P26792 | SSRVYPTLAVYENAHLYVFNNGSETITVENLDAWSMKKPLR |
| P00724 | LQAFLFLLAGFAAKISASMTNETSDRPLVHFTPNKGWMNDP |
| P00724 | NGLWYDEKDAKWHLYFQYNPNDTVWGTPLFWGHATSDDLTN |
| P00724 | ATSDDLTNWEDQPIAIAPKRNDSGAFSGSMVVDYNNTSGFF |

| | |
|---|---|
| P00724 | AIAPKRNDSGAFSGSMVVDYNNTSGFFNDTIDPRQRCVAIW |
| P00724 | DSGAFSGSMVVDYNNTSGFFNDTIDPRQRCVAIWTYNTPES |
| P00724 | YSLDGGYTFTEYQKNPVLAANSTQFRDPKVFWYEPSQKWIM |
| P00724 | SINPGAPAGGSFNQYFVGSFNGTHFEAFDNQSRVVDFGKDY |
| P00724 | GSFNQYFVGSFNGTHFEAFDNQSRVVDFGKDYYALQTFFNT |
| P00724 | TEYQANPETELINLKAEPILNISNAGPWSRFATNTTLTKAN |
| P00724 | LKAEPILNISNAGPWSRFATNTTLTKANSYNVDLSNSTGTL |
| P00724 | SRFATNTTLTKANSYNVDLSNSTGTLEFELVYAVNTTQTIS |
| P00724 | YNVDLSNSTGTLEFELVYAVNTTQTISKSVFADLSLWFKGL |
| O01634 | FSEDKAERSYNWEQLVADKQNTTSLKQTNQIGYYQWVPFIL |
| Q21123 | RQQNFGFDMWKTIFYGSTNGNETWRENGVFPRVTLCDFETR |
| P01005 | LCGFLPDAAFGAEVDCSRFPNATDKEGKDVLVCNKDLRPIC |
| P01005 | DGVTYTNDCLLCAYSIEFGTNISKEHDGECKETVPMNCSSY |
| P01005 | EFGTNISKEHDGECKETVPMNCSSYANTTSEDGKVMVLCNR |
| P01005 | SKEHDGECKETVPMNCSSYANTTSEDGKVMVLCNRAFNPVC |
| P01003 | DGVTYNHECMLCFYNKEYGTNISKEQDGECGETVPMDCSRY |
| P01003 | SKEQDGECGETVPMDCSRYPNTTSEDGKVTILCTKDFSFVC |
| Q9N2I2 | TVVRVPMMKQQDQFYYLLDRNLSCKVVGVPYQGNATAFFIL |
| Q9N2I2 | FYYLLDRNLSCKVVGVPYQGNATAFFILPREGEMEQVENGL |
| Q9N2I2 | LPKLGIRDIFTSDADLTGISNHSSIRVSEMVHKAVVEVDES |
| P05154 | TVVRVPMMSREDQYHYLLDRNLSCRVVGVPYQGNATALFIL |
| P05154 | YHYLLDRNLSCRVVGVPYQGNATALFILPSEGKMQQVENGL |
| P05154 | LPSLGISNVFTSHADLSGISNHSNIQVSEMVHKAVVEVDES |
| O00105 | TGYLLTLSAAFQVAQAAVTANNSQLLTWWHNTGEINTQTPV |
| O00105 | VYLAIPGNGMSDQLQYTQGYNQTQAWTSFLYSHDATVKISR |
| O00105 | QTQAWTSFLYSHDATVKISRNGSSANSNVVIRPTSLNFPVR |
| O00105 | SANSNVVIRPTSLNFPVRYDNQSVYITVPYSPTGYRFSVEF |
| O00105 | APSGARQPENALLIFASPFENSSTKPQPGSPNSIAPAPGRV |
| O00105 | KPQPGSPNSIAPAPGRVLGLNTTSASTVVFNPGVYYFTGHD |
| O00105 | YQKASGANNNGLRMWRGTLGNSSQTFVLNGVTVSAPPFNSM |
| O00105 | TILQDVFYHTDDDGLKMYYSNVTARNIVMWKESVAPVVEFG |
| O00105 | NPGIFGAVNNYLYAPDGLSSNHSTGNSNMTVRNITWSNFRA |
| O00105 | VNNYLYAPDGLSSNHSTGNSNMTVRNITWSNFRAEGSSSAL |
| O00105 | YAPDGLSSNHSTGNSNMTVRNITWSNFRAEGSSSALFRINP |
| O00105 | FRAEGSSSALFRINPIQNLDNISIKNVSIESFEPLSINTTE |
| O00105 | SSSALFRINPIQNLDNISIKNVSIESFEPLSINTTESWMPV |
| O00105 | NLDNISIKNVSIESFEPLSINTTESWMPVWYDLNNGKQITV |
| O00105 | LNNGKQITVTDFSIEGFTVGNTTITASNAASVGRIDGVDPA |
| Q6GU68 | FQIADCAYRDLEGVPPGFPANVTTLSLSANRLPGLPEGAFR |
| Q6GU68 | DLEGVPPGFPANVTTLSLSANRLPGLPEGAFREVPLLQSLW |
| Q6GU68 | GRALPGALATSGQPRFQAFANGSLLIPDFGKLEEGTYSCLA |

| | |
|---|---|
| Q9TXD8 | VIGNYANGICVPKGVTNPEGNATVIGWGKISSGGKQVNTLQ |
| P61622 | METNGHQKTGDVYKCPVTQGNCTKLNLGRVTLSNVSERKDN |
| P61622 | KCPVTQGNCTKLNLGRVTLSNVSERKDNMRLGLSLATNPKD |
| P61622 | DGESHDSPDLEKVIRQSEKDNVTRYAVAVLGYYNRRGINPE |
| P61622 | ETFLNEIKYIASDPDDKHFFNVTDEAALKDIVDALGDRIFS |
| P61622 | LKDIVDALGDRIFSLEGTNKNETSFGLEMSQTGFSSHVVED |
| P61622 | VTSVVSSRQGRVYVAGAPRFNHTGKVILFSMHNNRSLTIHQ |
| P61622 | VAGAPRFNHTGKVILFSMHNNRSLTIHQALRGEQIGSYFGS |
| P61622 | EGRERGKVYVYNLRQNRFVYNGTLKDSHSYQNARFGSCIAS |
| P61622 | LAVGALGNAVVLWARPVVQINASLHFEPSKINIFHKDCKRN |
| P61622 | CFIPIFLAPHFQTATVGIRYNATMDERRYMPRAHLDEGGDQ |
| P61622 | RVAVEATLENRGENAYSAVLNISQSENLQFASLIQKDDSDN |
| P61622 | DSDNSIECVNEERRLHKKVCNVSYPFFRAKAKVAFRLDFEF |
| P61622 | YEADVLFTRSSSLSHFEVKANSSLESYDGIGPPFNCVFKVQ |
| P61622 | IATRGGNRLLMLRDFFTDQGNTSCNIWGNSTEYRSTPTEED |
| P61622 | LLMLRDFFTDQGNTSCNIWGNSTEYRSTPTEEDLSHAPQRN |
| P61622 | NSTEYRSTPTEEDLSHAPQRNHSNSDVVSIICNLRLAPSQE |
| Q9UKX5 | LETNGYQKTGDVYKCPVIHGNCTKLNLGRVTLSNVSERKDN |
| Q9UKX5 | KCPVIHGNCTKLNLGRVTLSNVSERKDNMRLGLSLATNPKD |
| Q9UKX5 | DGESHDSPDLEKVIQQSERDNVTRYAVAVLGYYNRRGINPE |
| Q9UKX5 | ETFLNEIKYIASDPDDKHFFNVTDEAALKDIVDALGDRIFS |
| Q9UKX5 | LKDIVDALGDRIFSLEGTNKNETSFGLEMSQTGFSSHVVED |
| Q9UKX5 | VTSVVSSRQGRVYVAGAPRFNHTGKVILFTMHNNRSLTIHQ |
| Q9UKX5 | VAGAPRFNHTGKVILFTMHNNRSLTIHQAMRGQQIGSYFGS |
| Q9UKX5 | EGRERGKVYVYELRQNLFVYNGTLKDSHSYQNARFGSSIAS |
| Q9UKX5 | LAVGALGNAVILWSRPVVQINASLHFEPSKINIFHRDCKRS |
| Q9UKX5 | CFTPIFLAPHFQTTTVGIRYNATMDERRYTPRAHLDEGGDR |
| Q9UKX5 | RVAVEATLENRGENAYSTVLNISQSANLQFASLIQKEDSDG |
| Q9UKX5 | DSDGSIECVNEERRLQKQVCNVSYPFFRAKAKVAFRLDFEF |
| Q9UKX5 | YEADVLFTRSSSLSHYEVKPNSSLERYDGIGPPFSCIFRIQ |
| Q9UKX5 | IATRSGNRLLKLRDFLTDEANTSCNIWGNSTEYRPTPVEED |
| Q9UKX5 | LLKLRDFLTDEANTSCNIWGNSTEYRPTPVEEDLRRAPQLN |
| Q9UKX5 | NSTEYRPTPVEEDLRRAPQLNHSNSDVVSINCNIRLVPNQE |
| P56199 | YENEEGKWVLIGSPLVGQPKNRTGDVYKCPVGRGESLPCVK |
| P56199 | YKCPVGRGESLPCVKLDLPVNTSIPNVTEVKENMTFGSTLV |
| P56199 | GRGESLPCVKLDLPVNTSIPNVTEVKENMTFGSTLVTNPNG |
| P56199 | CVKLDLPVNTSIPNVTEVKENMTFGSTLVTNPNGGFLACGP |
| P56199 | LERMDIGPKQTQVGIVQYGENVTHEFNLNKYSSTEEVLVAA |
| P56199 | CEDENIQRFSIAILGSYNRGNLSTEKFVEEIKSIASEPTEK |
| P56199 | EKFVEEIKSIASEPTEKHFFNVSDELALVTIVKTLGERIFA |
| P56199 | FSAHYSQDWVMLGAVGAYDWNGTVVMQKASQIIIPRNTTFN |

| | |
|---|---|
| P56199 | AYDWNGTVVMQKASQIIIPRNTTFNVESTKKNEPLASYLGY |
| P56199 | VNSATASSGDVLYIAGQPRYNHTGQVIIYRMEDGNIKILQT |
| P56199 | APMYMGTEKEEQGKVYVYALNQTRFEYQMSLEPIKQTCCSS |
| P56199 | NKVNIQKKNCHMEGKETVCINATVCFDVKLKSKEDTIYEAD |
| P56199 | SLRQISRSFFSGTQERKVQRNITVRKSECTKHSFYMLDKHD |
| P56199 | SFYMLDKHDFQDSVRITLDFNLTDPENGPVLDDSLPNSVHE |
| P56199 | LHVATTEKDLLIVRSQNDKFNVSLTVKNTKDSAYNTRTIVH |
| P56199 | PNLVFSGIEAIQKDSCESNHNITCKVGYPFLRRGEMVTFKI |
| P56199 | VGYPFLRRGEMVTFKILFQFNTSYLMENVTIYLSATSDSEE |
| P56199 | RGEMVTFKILFQFNTSYLMENVTIYLSATSDSEEPPETLSD |
| P56199 | YLSATSDSEEPPETLSDNVVNISIPVKYEVGLQFYSSASEY |
| P56199 | YEVGLQFYSSASEYHISIAANETVPEVINSTEDIGNEINIF |
| P56199 | SSASEYHISIAANETVPEVINSTEDIGNEINIFYLIRKSGS |
| P56199 | LIRKSGSFPMPELKLSISFPNMTSNGYPVLYPTGLSSSENA |
| P56199 | HLKRGTILDCNTCKFATITCNLTSSDISQVNVSLILWKPTF |
| P56199 | NTCKFATITCNLTSSDISQVNVSLILWKPTFIKSYFSSLNL |
| P56199 | VNVSLILWKPTFIKSYFSSLNLTIRGELRSENASLVLSSSN |
| P56199 | FIKSYFSSLNLTIRGELRSENASLVLSSSNQKRELAIQISK |
| Q24247 | PHSHFGYSVATHTIGEANGPNKTNCVLVGAPLDQNRQPNTT |
| Q24247 | GPNKTNCVLVGAPLDQNRQPNTTHSGALWRCPMTQRFDDCE |
| Q24247 | DEIKEDQWMGVTVRSNPLQANGSGGKVIVCAHRYMYIVREN |
| Q24247 | ISGNTDLDDNSYPDVVIGAFNSSAAVILLARPIISIQTSVQ |
| Q24247 | RKELHNMDPNTPGCLDDPASNLTCFTFRACCSIEPYDEKNK |
| Q24247 | QKDCGDDDLCESNLIIRVEPNITESSGNEYTLILDETELEV |
| Q24247 | ESSGNEYTLILDETELEVRINVSNLADSAYEAQLFIAHQAG |
| Q24247 | AQLFIAHQAGVSYVATKKPTNATCNSYNTTLVACSLGNPML |
| Q24247 | QAGVSYVATKKPTNATCNSYNTTLVACSLGNPMLRDTTTFV |
| Q24247 | RFQPKGLEPSEKIMLFHIFANTTSKLVGPERPERDLRVNVV |
| Q24247 | AYLSAPAQMRMFPSQSRHSFNKSLIHSQRSYYSSSHRDDHS |
| Q24247 | DILNMPALSEAQVVVKARLWNSTLVSEYPRVERVRIFSTAT |
| P08514 | APPAWALNLDPVQLTFYAGPNGSQFGFSLDFHKDSHGRVAI |
| P08514 | EYFDGYWGYSVAVGEFDGDLNTTEYVVGAPTWSWTLGAVEI |
| P08514 | AFLRDEADFRDKLSPIVLSLNVSLPPTEAGMAPAVVLHGDT |
| P08514 | MRALSNVEGFERLICNQKKENETRVVLCELGNPMKKNAQIG |
| P08514 | LPSLYQRPLDQFVLQSHAWFNVSSLPYAVPPLSLPRGEAQV |
| P17301 | VDLSTATCEKLNLQTSTSIPNVTEMKTNMSLGLILTRNMGT |
| P17301 | CEKLNLQTSTSIPNVTEMKTNMSLGLILTRNMGTGGFLTCG |
| P17301 | KNLIKEIKAIASIPTERYFFNVSDEAALLEKAGTLGEQIFS |
| P17301 | TSHGHLIFPKQAFDQILQDRNHSSYLGYSVAAISTGESTHF |
| P17301 | SVAAISTGESTHFVAGAPRANYTGQIVLYSVNENGNITVIQ |
| P17301 | GAPRANYTGQIVLYSVNENGNITVIQAHRGDQIGSYFGSVL |

| | |
|---|---|
| P17301 | LCFSAKFRPTKQNNQVAIVYNITLDADGFSSRVTSRGLFKE |
| P17301 | FKSENFRHTKELNCRTASCSNVTCWLKDVHMKGEYFVNVTT |
| P17301 | SCSNVTCWLKDVHMKGEYFVNVTTRIWNGTFASSTFQTVQL |
| P17301 | WLKDVHMKGEYFVNVTTRIWNGTFASSTFQTVQLTAAAEIN |
| O44386 | NFMPRPSRVINSPKHLKFHINQTRSSYFGYTLVIRQTSIIV |
| O44386 | TSIIVGAPRAQSTLESQRTINETGAIYRCSLTNGVCSPYVL |
| O44386 | PRFYAPSSRDNHLHGVCYWVNNTVASTPQHVTRISPLRLKS |
| O44386 | LGDINHDGYNDVAVGAPFAGNGTVFIYLGSENGLRDQPSQR |
| O44386 | TLRLNYEITNIGETAYLPQFNVTSTSRLAFAQVPGNCKVVD |
| O44386 | SFYIPIAYKVAGSTAIIPIINVTSLKMQASYDSQLLSIDLY |
| O44386 | SLKMQASYDSQLLSIDLYDQNNTMLVVDPVEVTTTLSGGLE |
| O44386 | ISNVKAHDLLSDDFKGKLPVNRTIVFNCRDPEMTICVRAEM |
| Q62470 | QRYLLLAGAPRDLAVGDDYTNRTGAVYLCPLTAHKDDCERM |
| Q62470 | LDPALCTATSCVQVELCFAYNQSAGNPNYRRNITLAYTLEA |
| Q62470 | VQVELCFAYNQSAGNPNYRRNITLAYTLEADRDRRPPRLRF |
| Q62470 | LELLLMDNVRDKLRPIVIAMNYSLPLRMPDRLKLGLRSLDA |
| Q62470 | LRMPDRLKLGLRSLDAYPVLNQAQAMENHTEVHFQKECGPD |
| Q62470 | KLGLRSLDAYPVLNQAQAMENHTEVHFQKECGPDNKCDSNL |
| Q62470 | LQPLSRLQYSRDTKKLFLSINVTNSPSSQRAGEDAHEALLT |
| Q62470 | EVPSALLLSSVRPSGTCQANNETILCELGNPFKRNQRMELL |
| Q62470 | PYEVTNGKWLLYPTEITIHSNGSWPCQPSGNLVNPLNLTLS |
| Q62470 | TIHSNGSWPCQPSGNLVNPLNLTLSDPGVTPLSPQRRRRQL |
| Q62470 | TCSNGRARCVWLECPLPDTSNITNVTVKARVWNSTFIEDYK |
| Q62470 | NGRARCVWLECPLPDTSNITNVTVKARVWNSTFIEDYKDFD |
| Q62470 | ECPLPDTSNITNVTVKARVWNSTFIEDYKDFDRVRVDGWAT |
| Q62470 | RVDGWATLFLRTSIPTINMENKTTWFSVDIDSELVEELPAE |
| P13612 | HSHGANRWLLVGAPTANWLANASVINPGAIYRCRIGKNPGQ |
| P13612 | CLEERDNQWLGVTLSRQPGENGSIVTCGHRWKNIFYIKNEN |
| P13612 | DLIVMGAPGSSYWTGSLFVYNITTNKYKAFLDKQNQVKFGS |
| P13612 | LLRTRPVVIVDASLSHPESVNRTKFDCVENGWPSVCIDLTL |
| P13612 | LTLCFSYKGKEVPGYIVLFYNMSLDVNRKAESPPRFYFSSN |
| P13612 | NMSLDVNRKAESPPRFYFSSNGTSDVITGSIQVSSREANCR |
| P13612 | KKEKDIMKKTINFARFCAHENCSADLQVSAKIGFLKPHENK |
| P13612 | ENCSADLQVSAKIGFLKPHENKTYLAVGSMKTLMLNVSLFN |
| P13612 | LKPHENKTYLAVGSMKTLMLNVSLFNAGDDAYETTLHVKLP |
| P13612 | SFVYGSNDENEPETCMVEKMNLTFHVINTGNSMAPNVSVEI |
| P13612 | MVEKMNLTFHVINTGNSMAPNVSVEIMVPNSFSPQTDKLFN |
| P08648 | VEFYRPGTDGVSVLVGAPKANTSQPGVLQGGAVYLCPWGAS |
| P08648 | WRTEKEPLSDPVGTCYLSTDNFTRILEYAPCRSDFSWAAGQ |
| P08648 | AVGEFSGDDTEDFVAGVPKGNLTYGYVTILNGSDIRSLYNF |
| P08648 | EDFVAGVPKGNLTYGYVTILNGSDIRSLYNFSGEQMASYFG |

| | |
|---|---|
| P08648 | GNLTYGYVTILNGSDIRSLYNFSGEQMASYFGYAVAATDVN |
| P08648 | AMFNPEERSCSLEGNPVACINLSFCLNASGKHVADSIGFTV |
| P08648 | ERSCSLEGNPVACINLSFCLNASGKHVADSIGFTVELQLDW |
| P08648 | TLLIQNGAREDCREMKIYLRNESEFRDKLSPIHIALNFSLD |
| P08648 | IYLRNESEFRDKLSPIHIALNFSLDPQAPVDSHGLRPALHY |
| P08648 | LQLEVFGEQNHVYLGDKNALNLTFHAQNVGEGGAYEAELRV |
| P08648 | ELRVTAPPEAEYSGLVRHPGNFSSLSCDYFAVNQSRLLVCD |
| P08648 | SGLVRHPGNFSSLSCDYFAVNQSRLLVCDLGNPMKAGASLW |
| P08648 | VPHLRDTKKTIQFDFQILSKNLNNSQSDVVSFRLSVEAQAQ |
| P08648 | HLRDTKKTIQFDFQILSKNLNNSQSDVVSFRLSVEAQAQVT |
| P08648 | SCPQALEGQQLLYVTRVTGLNCTTNHPINPKGLELDPEGSL |
| P11688 | VEFYRPGRDGVSVLVGAPKANTSQPGVLQGGAVYYCPWGTS |
| P11688 | WRTEKDPQNDPVGTCYLSTENFTRILEYAPCRSDFGSAAGQ |
| P11688 | AVGEFSGDDTEDFVAGVPKGNLTYGYVTVLNGSDIHSLYNV |
| P11688 | EDFVAGVPKGNLTYGYVTVLNGSDIHSLYNVSGEQMASYFG |
| P11688 | GNLTYGYVTVLNGSDIHSLYNVSGEQMASYFGYAVAATDTN |
| P11688 | SMFNPEERSCSLEGNPVSCINLSFCLNASGKHVPNSIGFEV |
| P11688 | ERSCSLEGNPVSCINLSFCLNASGKHVPNSIGFEVELQLDW |
| P11688 | TLLIQNGAREDCREMKIYLRNESEFRDKLSPIHIALNFSLD |
| P11688 | IYLRNESEFRDKLSPIHIALNFSLDPKAPMDSHGLRPVLHY |
| P11688 | VPDLQLDVYGEKKHVYLGDKNALNLTFHAQNLGEGGAYEAE |
| P11688 | LQLDVYGEKKHVYLGDKNALNLTFHAQNLGEGGAYEAELRV |
| P11688 | EKKHVYLGDKNALNLTFHAQNLGEGGAYEAELRVTAPLEAE |
| P11688 | ELRVTAPLEAEYSGLVRHPGNFSSLSCDYFAVNQSRQLVCD |
| P11688 | SGLVRHPGNFSSLSCDYFAVNQSRQLVCDLGNPMKAGTSLW |
| P11688 | HLQDTKKTIQFDFQILSKNLNNSQSNVVSFPLSVEAQAQVS |
| P11688 | CPQALEGQQLLYVTKVTGLSNCTSNYTPNSQGLELDPETSP |
| P11688 | LEGQQLLYVTKVTGLSNCTSNYTPNSQGLELDPETSPHHLQ |
| P23229 | DKRLLLVGAPRAEALPLQRANRTGGLYSCDITARGPCTRIE |
| P23229 | IVFGAPGTYNWKGIVRVEQKNNTFFDMNIFEDGPYEVGGET |
| P23229 | SGKGIVSKDEITFVSGAPRANHSGAVVLLKRDMKSAHLLPE |
| P23229 | AVYVYMNQQGRWNNVKPIRLNGTKDSMFGIAVKNIGDINQD |
| P23229 | SAYRELRAFPEKQLSCVANQNGSQADCELGNPFKRNSNVTF |
| P23229 | ANQNGSQADCELGNPFKRNSNVTFYLVLSTTEVTFDTPDLD |
| P23229 | VESKGLEKVTCEPQKEINSLNLTESHNSRKKREITEKQIDD |
| P23229 | KQIDDNRKFSLFAERKYQTLNCSVNVNCVNIRCPLRGLDSK |
| P23229 | RCPLRGLDSKASLILRSRLWNSTFLEEYSKLNYLDILMRAF |
| Q61739 | DKRLLLVGAPRAEALPLQRANRTGGLYSCDITSRGPCTRIE |
| Q61739 | IVFGAPGTYNWKGIVRVEQKNNTFFDMNIFEDGPYEVGGET |
| Q61739 | SGKGIVSKDDITFVSGAPRANHSGAVVLLKRDMKSAHLLPE |
| Q61739 | AVYVYINQQGKWSNVKPIRLNGTKDSMFGISVKNIGDINQD |

| | |
|---|---|
| Q61739 | SAYRELRAFPEKQLSCVANQNGSQADCELGNPFKRNSSVTF |
| Q61739 | CVANQNGSQADCELGNPFKRNSSVTFYLILSTTEVTFDTTD |
| Q61739 | KQIDDSRKFSLFPERKYQTLNCSVNVRCVNIRCPLRGLDSK |
| Q61739 | RCPLRGLDSKASLVLRSRLWNSTFLEEYSKLNYLDILLRAS |
| Q13683 | PQSWLLVGAPQALALPGQQANRTGGLFACPLSLEETDCYRV |
| Q13683 | HYSGVRALDPAEKPLCLSNENASHVECELGNPMKRGAQVTF |
| Q13683 | GERQEPSMSWWPVSSAEKKKNITLDCARGTANCVVFSCPLY |
| Q13683 | SCPLYSFDRAAVLHVWGRLWNSTFLEEYSAVKSLEVIVRAN |
| Q13683 | NSTFLEEYSAVKSLEVIVRANITVKSSIKNLMLRDASTVIP |
| Q61738 | PQSWLLVGAPQALALPGQQANRTGGLFACPLSLEETDCYRV |
| Q61738 | SLRYSGVRALDSVEKPLCLSNDSASHVECELGNPMKRGAQV |
| Q61738 | PPEKVEPSTSWWPVSSAEKRNMTLDCPRTAKCVVFSCPLYS |
| Q61738 | SCPLYSFDRAAVLHVWGRLWNSTFLEEYMAVKSLEVIVRAN |
| Q61738 | NSTFLEEYMAVKSLEVIVRANITVKSSIKNLLLRDASTVIP |
| Q13797 | ELVVMGAPGSFYWAGTIKVLNLTDNTYLKLNDEVIMNRRYT |
| Q13797 | LLRARPVITVDVSIFLPGSINITAPQCHDGQQPVNCLNVTT |
| Q13797 | GSINITAPQCHDGQQPVNCLNVTTCFSFHGKHVPGEIGLNY |
| Q13797 | ELPPLTPVLRWKKGQKIAQKNQTVFERNCRSEDCAADLQLQ |
| Q13797 | KLLLSSMDEKTLYLALGAVKNISLNISISNLGDDAYDANVS |
| Q13797 | SSMDEKTLYLALGAVKNISLNISISNLGDDAYDANVSFNVS |
| Q13797 | VKNISLNISISNLGDDAYDANVSFNVSRELFFINMWQKEEM |
| Q13797 | SLNISISNLGDDAYDANVSFNVSRELFFINMWQKEEMGISC |
| Q13797 | SVDAANFIQLDDLECHFQPINITLQVYNTGPSTLPGSSVSI |
| Q13797 | SSGGAEMFHVQEMVVGQEKGNCSFQKNPTPCIIPQEQENIF |
| Q13797 | SGRKVLDCEKPGISCLTAHCNFSALAKEESRTIDIYMLLNT |
| P20701 | FGYRVLQVGNGVIVGAPGEGNSTGSLYQCQSGTGHCLPVTL |
| P20701 | SLYQCQSGTGHCLPVTLRGSNYTSKYLGMTLATDPTDGSIL |
| P20701 | QPDEFQKILDFMKDVMKKLSNTSYQFAAVQFSTSYKTEFDF |
| P20701 | IPVHEVECSYSTSNKMKEGVNITICFQIKSLIPQFQGRLVA |
| P20701 | ITICFQIKSLIPQFQGRLVANLTYTLQLDGHRTRRRGLFPG |
| P20701 | SCTDFSFHFPVCVQDLISPINVSLNFSLWEEEGTPRDQRAQ |
| P20701 | FSFHFPVCVQDLISPINVSLNFSLWEEEGTPRDQRAQGKDI |
| P20701 | PVSCEELPEESRLLSRALSCNVSSPIFKAGHSVALQMMFNT |
| P20701 | SPIFKAGHSVALQMMFNTLVNSSWGDSVELHANVTCNNEDS |
| P20701 | QMMFNTLVNSSWGDSVELHANVTCNNEDSDLLEDNSATTII |
| P20701 | VGEIEASSMFSLCSSLSISFNSSKHFHLYGSNASLAQVVMK |
| P20701 | LCSSLSISFNSSKHFHLYGSNASLAQVVMKVDVVYEKQMLY |
| P11215 | CDYSTGSCEPIRLQVPVEAVNMSLGLSLAATTSPPQLLACG |
| P11215 | QLLGRTHTATGIRKVVRELFNITNGARKNAFKILVVITDGE |
| P11215 | SYDWAGGVFLYTSKEKSTFINMTRVDSDMNDAYLGYAAAII |
| P11215 | KGTQIGAYFGASLCSVDVDSNGSTDLVLIGAPHYYEQTRGG |

| | |
|---|---|
| P11215 | SVVTYDLALDSGRPHSRAVFNETKNSTRRQTQVLGLTQTCE |
| P11215 | YDLALDSGRPHSRAVFNETKNSTRRQTQVLGLTQTCETLKL |
| P11215 | LKLQLPNCIEDPVSPIVLRLNFSLVGTPLSAFGNLRPVLAE |
| P11215 | SITFSFMSLDCLVVGGPREFNVTVTVRNDGEDSYRTQVTFF |
| P11215 | KSTSCSINHPIFPENSEVTFNITFDVDSKASLGNKLLLKAN |
| P11215 | NITFDVDSKASLGNKLLLKANVTSENNMPRTNKTEFQLELP |
| P11215 | LGNKLLLKANVTSENNMPRTNKTEFQLELPVKYAVYMVVTS |
| P11215 | PVKYAVYMVVTSHGVSTKYLNFTASENTSRVMQHQYQVSNL |
| P11215 | YMVVTSHGVSTKYLNFTASENTSRVMQHQYQVSNLGQRSLP |
| P11215 | SNLGQRSLPISLVFLVPVRLNQTVIWDRPQVTFSENLSSTC |
| P11215 | VPVRLNQTVIWDRPQVTFSENLSSTCHTKERLPSHSDFLAE |
| P11215 | KERLPSHSDFLAELRKAPVVNCSIAVCQRIQCDIPFFGIQE |
| P11215 | IAVCQRIQCDIPFFGIQEEFNATLKGNLSFDWYIKTSHNHL |
| P11215 | IQCDIPFFGIQEEFNATLKGNLSFDWYIKTSHNHLLIVSTA |
| P11215 | WYIKTSHNHLLIVSTAEILFNDSVFTLLPGQGAFVRSQTET |
| P06756 | DFFVPSASSRMFLLVGAPKANTTQPGIVEGGQVLKCDWSST |
| P06756 | VSGVPRAARTLGMVYIYDGKNMSSLYNFTGEQMAAYFGFSV |
| P06756 | AARTLGMVYIYDGKNMSSLYNFTGEQMAAYFGFSVAATDIN |
| P06756 | RPVITVNAGLEVYPSILNQDNKTCSLPGTALKVSCFNVRFC |
| P06756 | QKGAIRRALFLYSRSPSHSKNMTISRGGLMQCEELIAYLRD |
| P06756 | YRTAADTTGLQPILNQFTPANISRQAHILLDCGEDNVCKPK |
| P06756 | IGVVRNNEALARLSCAFKTENQTRQVVCDLGNPMKAGTQLL |
| P06756 | NGPSSFSKAMLHLQWPYKYNNNTLLYILHYDIDGPMNCTSD |
| P06756 | YKYNNNTLLYILHYDIDGPMNCTSDMEINPLRIKISSLQTT |
| P06756 | SDMEINPLRIKISSLQTTEKNDTVAGQGERDHLITKRDLAL |
| P06756 | AILYVKSLLWTETFMNKENQNHSYSLKSSASFNVIEFPYKN |
| P06756 | SASFNVIEFPYKNLPIEDITNSTLVTTNVTWGIQPAPMPVP |
| P06756 | EFPYKNLPIEDITNSTLVTTNVTWGIQPAPMPVPVWVIILA |
| P43406 | DFFEPSTSSRMFLLVGAPKANTTQPGIVEGGQVLKCECSSS |
| P43406 | VSGVPRAARTLGMVYIYDGKNMSSLHNFTGEQMAAYFGFSV |
| P43406 | AARTLGMVYIYDGKNMSSLHNFTGEQMAAYFGFSVAATDIN |
| P43406 | QRTAADATGLQPILNQFTPANVSRQAHILLDCGEDNVCKPK |
| P43406 | IGVVRNNEALARLSCAFKTENQTRQVVCDLGNPMKAGTQLL |
| P43406 | NGPSSFSKAILNLQWPYKYNNNTLLYILHYDIDGPMNCTAD |
| P43406 | YKYNNNTLLYILHYDIDGPMNCTADTEINPLRIKTPEKNDT |
| P43406 | PMNCTADTEINPLRIKTPEKNDTAAAGQGERNHLITKRDLT |
| P43406 | KSAILYVKSLLWTETFMNKENQNHSYSLKSSASFNIIEFPY |
| P43406 | AILYVKSLLWTETFMNKENQNHSYSLKSSASFNIIEFPYKN |
| P43406 | SASFNIIEFPYKNLPIEDLFNSTLVTTNITWGIQPAPMPVP |
| P43406 | EFPYKNLPIEDLFNSTLVTTNITWGIQPAPMPVPVWVIILA |
| P20702 | VVQYANSWVVVGAPQKITAANQTGGLYQCGYSTGACEPIGL |

| | |
|---|---|
| P20702 | CGYSTGACEPIGLQVPPEAVNMSLGLSLASTTSPSQLLACG |
| P20702 | SFTWSGGAFLYPPNMSPTFINMSQENVDMRDSYLGYSTELA |
| P20702 | LDLALDPGRLSPRATFQETKNRSLSRVRVLGLKAHCENFNL |
| P20702 | FNLLLPSCVEDSVTPITLRLNFTLVGKPLLAFRNLRPMLAA |
| P20702 | LATFDVSPKAVLGDRLLLTANVSSENNTPRTSKTTFQLELP |
| P20702 | PVKYAVYTVVSSHEQFTKYLNFSESEEKESHVAMHRYQVNN |
| P20702 | FRCDVPSFSVQEELDFTLKGNLSFGWVRQILQKKVSVVSVA |
| P05556 | ANAKSCGECIQAGPNCGWCTNSTFLQEGMPTSARCDDLEAL |
| P05556 | GCPPDDIENPRGSKDIKKNKNVTNRSKGTAEKLKPEDITQI |
| P05556 | PDDIENPRGSKDIKKNKNVTNRSKGTAEKLKPEDITQIQPQ |
| P05556 | VMPYISTTPAKLRNPCTSEQNCTSPFSYKNVLSLTNKGEVF |
| P05556 | EGGFDAIMQVAVCGSLIGWRNVTRLLVFSTDAGFHFAGDGK |
| P05556 | PVYKELKNLIPKSAVGTLSANSSNVIQLIIDAYNSLSSEVI |
| P05556 | ILENGKLSEGVTISYKSYCKNGVNGTGENGRKCSNISIGDE |
| P05556 | NGKLSEGVTISYKSYCKNGVNGTGENGRKCSNISIGDEVQF |
| P05556 | EGVTISYKSYCKNGVNGTGENGRKCSNISIGDEVQFEISIT |
| P05556 | YKSYCKNGVNGTGENGRKCSNISIGDEVQFEISITSNKCPK |
| P05556 | ICECECQSEGIPESPKCHEGNGTFECGACRCNEGRVGRHCE |
| P05556 | CECSTDEVNSEDMDAYCRKENSSEICSNNGECVCGQCVCRK |
| P05556 | NGLICGGNGVCKCRVCECNPNYTGSACDCSLDTSTCEASNG |
| P05556 | CRAFNKGEKKDTCTQECSYFNITKVESRDKLPQPVQPDPVS |
| P09055 | ANAKSCGECIQAGPNCGWCTNTTFLQEGMPTSARCDDLEAL |
| P09055 | GCQPSDIENPRGSQTIKKNKNVTNRSKGMAEKLRPEDITQI |
| P09055 | PSDIENPRGSQTIKKNKNVTNRSKGMAEKLRPEDITQIQPQ |
| P09055 | VMPYISTTPAKLRNPCTSEQNCTSPFSYKNVLSLTDRGEFF |
| P09055 | EGGFDAIMQVAVCGSLIGWRNVTRLLVFSTDAGFHFAGDGK |
| P09055 | PVYKELKNLIPKSAVGTLSGNSSNVIQLIIDAYNSLSSEVI |
| P09055 | KELKNLIPKSAVGTLSGNSSNVIQLIIDAYNSLSSEVILEN |
| P09055 | AVGTLSGNSSNVIQLIIDAYNSLSSEVILENSKLPDGVTIN |
| P09055 | NSKLPDGVTINYKSYCKNGVNGTGENGRKCSNISIGDEVQF |
| P09055 | YKSYCKNGVNGTGENGRKCSNISIGDEVQFEISITANKCPN |
| P09055 | ICKCNCQSHGIPASPKCHEGNGTFECGACRCNEGRVGRHCE |
| P09055 | CECSTDEVNSEDMDAYCRKENSSEICSNNGECVCGQCVCRK |
| P09055 | NGLICGGNGVCRCRVCECYPNYTGSACDCSLDTGPCLASNG |
| P09055 | CRAFNKGEKKDTCAQECSHFNLTKVESREKLPQPVQVDPVT |
| P05107 | VSSCRECIESGPGCTWCQKLNFTGPGDPDSIRCDTRPQLLM |
| P05107 | KQLSPQKVTLYLRPGQAAAFNVTFRRAKGYPIDLYYLMDLS |
| P05107 | PNKEKECQPPFAFRHVLKLTNNSNQFQTEVGKQLISGNLDA |
| P05107 | NKEKECQPPFAFRHVLKLTNNSNQFQTEVGKQLISGNLDAP |
| P05107 | EKECQPPFAFRHVLKLTNNSNQFQTEVGKQLISGNLDAPEG |
| P05107 | EGGLDAMMQVAACPEEIGWRNVTRLLVFATDDGFHFAGDGK |

| | |
|---|---|
| P05107 | CECQTQGRSSQELEGSCRKDNNSIICSGLGDCVCGQCLCHT |
| P05107 | CGKYISCAECLKFEKGPFGKNCSAACPGLQLSNNPVKGRTC |
| P05106 | QVTQVSPQRIALRLRPDDSKNFSIQVRQVEDYPVDIYYLMD |
| P05106 | SQKNINLIFAVTENVVNLYQNYSELIPGTTVGVLSMDSSNV |
| P05106 | IRSKVELEVRDLPEELSLSFNATCLNNEVIPGLKSCMGLKI |
| P05106 | DCDCACQAQAEPNSHRCNNGNGTFECGVCRCGPGWLGSQCE |
| P05106 | HGQCSCGDCLCDSDWTGYYCNCTTRTDTCMSSNGLLCSGRG |
| P05106 | YCRDEIESVKELKDTGKDAVNCTYKNEDDCVVRFQYYEDSS |
| P16144 | VPTLVRLLAKHNIIPIFAVTNYSYSYYEKLHTYFPVSSLGV |
| P16144 | NGDFVCGQCVCSEGWSGQTCNCSTGSLSDIQPCLREGEDKP |
| P16144 | MGQCVCEPGWTGPSCDCPLSNATCIDSNGGICNGRGHCECG |
| P16144 | ECGRCHCHQQSLYTDTICEINYSAIHPGLCEDLRSCVQCQA |
| P16144 | DEDDDCTYSYTMEGDGAPGPNSTVLVHKKKDCPPGSFWWLI |
| Q9GYK2 | WCIDPHSSLTNRCQLKSKFTNETCTPHLVYSPQTAQVKIQQ |
| Q9GYK2 | CPLASHGVSKASELEDKCRFNSSSPVCSASGKCKCGQCQCN |
| Q9GYK2 | KCGQCQCNKPTVTGKFCQCDNDSCPLAVNGKVCSGNGVCDC |
| P11584 | TEGCAWCMQPDFKGQSRCYQNTSSLCPEEFAYSPITVEQIL |
| P11584 | EHPCENCKAPYGYQNHMPLNNNTESFSNEVKNATVSGNLDA |
| P11584 | GYQNHMPLNNNTESFSNEVKNATVSGNLDAPEGGFDAIMQA |
| P11584 | LSVYEKLVEHIQGSSAAKLDNDSSNVVELVKEEYRKISSSV |
| P11584 | VVELVKEEYRKISSSVEMKDNATGDVKITYFSSCLSNGPEV |
| P11584 | DSYFGNKCECSATDLTSKFANDTSCRADSTSTTDCSGRGHC |
| P11584 | ECHKRPNPIEIISGKHCECDNFSCERNRNQLCSGPDHGTCE |
| P11584 | CGRCKCKPGWTGSNCGCQESNDTCMPPGGGEICSGHGTCEC |
| P11584 | CVQCQMYKTGELKNGDDCARNCTQFVPVGVEKVEIDETKDE |
| Q8C0Z1 | FLALGDINKDKVQDVLFLYKNTNSSNNLTRSCADEGFSTPC |
| Q8C0Z1 | ALGDINKDKVQDVLFLYKNTNSSNNLTRSCADEGFSTPCAF |
| Q8C0Z1 | DINKDKVQDVLFLYKNTNSSNNLTRSCADEGFSTPCAFVVA |
| Q8C0Z1 | INKDKVQDVLFLYKNTNSSNNLTRSCADEGFSTPCAFVVAV |
| Q8C0Z1 | ERITGRDGHFKEDPYWENMLNHSVHRRLLHRLGAVRYLMNI |
| Q8C0Z1 | VLRKPILGHYKPDTLAVVIENGTSIDRQILLLDLSTGSILW |
| Q8C0Z1 | VQHSLYMFHPTLPGILLELANVSANIVAFDAVLLEPSRHAA |
| Q9H0X4 | AVDDINGDRIQDVLFLYKNTNSSNNFSRSCVDEGFSSPCTF |
| Q9H0X4 | EKVTGSGGPFKSDPHWESMLNATTRRMLSHSSGAVRYLMHV |
| Q9H0X4 | VLRKPIFGRYKPDTLAVAVENGTGTDRQILFLDLGTGAVLC |
| Q9H0X4 | ARHSLYMFHPTLPRVLLELANVSTHIVAFDAVLFEPSRHAA |
| Q0VCM5 | YLTIQELLAKRMKLEGQEKANVSAKALQMSLAYQFVTPLTS |
| Q0VCM5 | FGRLGIANPATDFQLEVTPQNITLNPGSGGPVFSWRDQAFL |
| P19827 | DKICDLLVANNHFAHFFAPQNLTNMNKNVVFVIDISGSMRG |
| P19827 | YLTIQELLAKRMKVDREERANLSSQALQMSLDYGFVTPLTS |
| P19827 | FGRLGIANPATDFQLEVTPQNITLNPGFGGPVFSWRDQAVL |

| | |
|---|---|
| Q61702 | DKLCDLLVANNYFTHFFAPKNLTNMSKNLVFVIDISGSMEG |
| Q61702 | CDLLVANNYFTHFFAPKNLTNMSKNLVFVIDISGSMEGQKV |
| Q61702 | YLTIQELLAKRMKTEGEERANLSSQVLKMSLDYHFVTPLTS |
| P19823 | KVQSTITSRMATTMIQSKVVNNSPQPQNVVFDVQIPKGAFI |
| P19823 | SPQPQNVVFDVQIPKGAFISNFSMTVDGKTFRSSIKEKTVG |
| P19823 | TVGELKLSKIQKNVKENIQDNISLFSLGMGFDVDYDFLKRL |
| Q61703 | KVQSTITSRVATTTIQSKLVNNSPLPQSVVFDVQIPKGAFI |
| Q61703 | SPLPQSVVFDVQIPKGAFISNFTMTVNGMTFTSSIKEKTVG |
| Q61703 | GQQKAHVSFKPTVAQQRKCPNCTETAVNGELVVMYDVNREE |
| Q61703 | TVGELKLSKIQKNVKQSIQDNISLFSLGIGFDVDYDFLKRL |
| Q61704 | ADTAKEVSFDVELPKTAFITNFTLTIDGVTYPGNVKEKEVA |
| Q61704 | YLTIEQLLEKRKNAKGDEKENITAEALDLSLKYHFVTPLTS |
| Q06033 | ADTAKEVSFDVELPKTAFITNFTLTIDGVTYPGNVKEKEVA |
| Q06033 | YLTIEQLLEKRKNAHGEEKENLTARALDLSLKYHFVTPLTS |
| Q14624 | ANTVQEATFQMELPKKAFITNFSMIIDGMTYPGIIKEKAEA |
| Q14624 | LETESTFMTNQLVDALTTWQNKTKAHIRFKPTLSQQQKSPE |
| Q14624 | IENGYFVHYFAPEGLTTMPKNVVFVIDKSGSMSGRKIQQTR |
| Q14624 | LQDRGPDVLTATVSGKLPTQNITFQTESSVAEQEAEFQSPK |
| Q14624 | LEQTVSASDADQQALRNQALNLSLAYSFVTPLTSMVVTKPD |
| Q3T052 | ADAVREATFQMELPKKAFITNFSMVIDGVTYPGNIKEKAAA |
| Q3T052 | LETESTFMTNKLAEALTTSQNKTKAHVRFKPTLSQQQKYPE |
| Q3T052 | LREQSPDVLLAQIRGQLHRENITYMMMSHVAEQEEMFRSPK |
| A6X935 | ADAVQEATFQVELPRKAFITNFSMIIDGVTYPGVVKEKAEA |
| A6X935 | LQDQGPDVLLAKVSGQMHMQNITFQTEASVAQQEKEFKSPK |
| A6X935 | LEQRISASGAELEALEAQVLNLSLKYNFVTPLTHMVVTKPE |
| A6X935 | KVTIGLLSLDDPQRGLMLLLNDTQHFSNNVKGELGQFYRDI |
| Q9Y287 | NKKLTAYLDLNLDKCYVIPLNTSIVMPPRNLLELLINIKAG |
| Q14643 | GESLASEFLFSDVCRVESGENCSSPAPREELVPAEETEQDK |
| Q9D9J7 | LSWHRASKGLTDYSFYRVWENSSETLIAKGKEPYLTKSMVG |
| Q9Y624 | TWFKDGIVMPTNPKSTRAFSNSSYVLNPTTGELVFDPLSAS |
| Q9Y624 | IVMPTNPKSTRAFSNSSYVLNPTTGELVFDPLSASDTGEYS |
| O88792 | SLVQGKGSVYTAQSDVQVPENESIKLTCTYSGFSSPRVEWK |
| O88792 | WFKDGISMLTADAKKTRAFMNSSFTIDPKSGDLIFDPVTAF |
| Q9D8B7 | IQGDLAGRTDVFGKTSLRIWNVTRSDSAIYRCEVVALNDRK |
| Q9D8B7 | SWYRNDVPLPTDSRANPRFQNSSFHVNSETGTLVFNAVHKD |
| Q9D8B7 | VPLPTDSRANPRFQNSSFHVNSETGTLVFNAVHKDDSGQYY |
| P57087 | TLQGDFKNRAEMIDFNIRIKNVTRSDAGKYRCEVSAPSEQG |
| P57087 | TWFKDGIRLLENPRLGSQSTNSSYTMNTKTGTLQFNTVSKL |
| P57087 | ARNSVGYRRCPGKRMQVDDLNISGIIAAVVVVALVISVCGL |
| Q9BX67 | IQGDLAGRAEILGKTSLKIWNVTRRDSALYRCEVVARNDRK |
| Q9BX67 | SWYRNDVPLPTDSRANPRFRNSSFHLNSETGTLVFTAVHKD |

| | |
|---|---|
| Q9BX67 | VPLPTDSRANPRFRNSSFHLNSETGTLVFTAVHKDDSGQYY |
| Q80UL9 | DWLFSKDKDDASEYVLFYYSNLSVPTGRFQNRSHLVGDTFH |
| Q80UL9 | ASEYVLFYYSNLSVPTGRFQNRSHLVGDTFHNDGSLLLQDV |
| Q80UL9 | LLQDVQKADEGIYTCEIRLKNESMVMKKPVELWVLPEEPKD |
| Q8BI36 | AVDIQPACLGLYCGKTLLFKNGSSEIYGECGVCPRGQRTNA |
| Q6NSJ0 | AFGGILERYWLSSRAAAIKVNDSVPFHLGWNSTERSLRLQA |
| Q6NSJ0 | LSSRAAAIKVNDSVPFHLGWNSTERSLRLQARYHDTPYKPP |
| Q6NSJ0 | TEMALPFFSLAEVRVGYQSQNISCFFRLVDRDSVWGYDLGL |
| Q69ZQ1 | AFGGILERYWLSSRAAAIKVNDSVPFHLGWNSTERSMRLQA |
| Q69ZQ1 | LSSRAAAIKVNDSVPFHLGWNSTERSMRLQARYHDTSYKPP |
| Q69ZQ1 | THPEAREWFQGHLRRLRLRYNVTSFKFDAGEVSYLPRDFST |
| P00756 | KDEPSAQHRFVSKAIPHPGFNMSLMRKHIRFLEYDYSNDLM |
| Q8IZA0 | ITISSPLTTDLTAELSGGPKNVSVQPEISEGLATTPSTQQV |
| Q8IZA0 | GLYEFKVIVEGQNAHGEGYVNVTVKPEPRKNRPPIAIVSPQ |
| Q8IZA0 | REEKISEDTAILKLSKLVPGNYTFSLTVVDSDGATNSTTAN |
| Q8IZA0 | KLVPGNYTFSLTVVDSDGATNSTTANLTVNKAVDYPPVANA |
| Q8IZA0 | ANAGPNQVITLPQNSITLFGNQSTDDHGITSYEWSLSPSSK |
| P29622 | GLLALSHGQLHVEHDGESCSNSSHQQILETGEGSPSLKIAP |
| P29622 | MLSLGACSHSRSQILEGLGFNLTELSESDVHRGFQHLLHTL |
| P29622 | TRVGSALFLSHNLKFLAKFLNDTMAVYEAKLFHTNFYDTVG |
| P29622 | LWEKPFISSRTTPKDFYVDENTTVRVPMMLQDQEHHWYLHD |
| Q86W47 | FIFGFCWLSPALQDLQATEANCTVLSVQQIGEVFECTFTCG |
| Q86W47 | FTCGADCRGTSQYPCVQVYVNNSESNSRALLHSDEHQLLTN |
| P63142 | IFRDENEDMHGGGVTFHTYSNSTIGYQQSTSFTDPFFIVET |
| P15384 | FRDEKDYPASPSQDVFEAANNSTSGASSGASSFSDPFFVVE |
| P08510 | LSIVIFCLETLPEFKHYKVFNTTTNGTKIEEDEVPDITDPF |
| P08510 | IFCLETLPEFKHYKVFNTTTNGTKIEEDEVPDITDPFFLIE |
| P15382 | TTAVTPFLTKLWQETVQQGGNMSGLARRSPRSSDGKLEALY |
| Q12809 | HMDSRIGWLHNLGDQIGKPYNSSGLGGPSIKDKYVTALYFT |
| Q9H252 | YLEHKIGWLDSLGVQLGKRYNGSDPASGPSVQDKYVTALYF |
| P48048 | LLWYAVAYIHKDLPEFHPSANHTPCVENINGLTSAFLFSLE |
| P48549 | ASMWWVIAYTRGDLNKAHVGNYTPCVANVYNFPSAFLFFIE |
| O00180 | LEQFLGRVLEASNYGVSVLSNASGNWNWDFTSALFFASTVL |
| Q7Z418 | LVAADDGEFEKFLEELCRILNCSETVVEDRKQDLQGHLQKV |
| Q6VV64 | AEENPELKKFLDDLCNILKCNLTVVEGSRKNLCEHLQHLKP |
| Q7Z4H8 | AAVVLPVRYFYLQAVNSEGQNLTRSPAGETPFKVVVKSLSP |
| Q7Z4H8 | MRGVTNDLLSIQGNTGPSWINKTERAFFRGRDSREERLQLV |
| P23276 | PRPCETSVCLDLRDHYLASGNTSVAPCTDFFSFACGRAKET |
| P23276 | TSVAPCTDFFSFACGRAKETNNSFQELATKNKNRLRRILEV |
| P23276 | QVIEELGGWRISGKWTSLNFNRTLRLLMSQYGHFPFFRAYL |
| P23276 | TPMSLSPSQSLVVHDVEYLKNMSQLVEEMLLKQRDFLQSHM |

| | |
|---|---|
| P23276 | AHLCLKRHYAAFPLPSRTSFNDSLTFLENAADVGGLAIALQ |
| O60938 | IWYLYLQNNLIETIPEKPFENATQLRWINLNKNKITNYGIE |
| O60938 | LQLARNKVSRIPQGTFSNLENLTLLDLQNNKLVDNAFQRDT |
| O60938 | NALRNMPPRLPANTMQLFLDNNSIEGIPENYFNVIPKVAFL |
| O60938 | PRISAHLQHLHLDHNKIKSVNVSVICPSPSMLPAERDSFSY |
| O42235 | IWYLYLQNNLIETISEKPFVNATHLRWINLNKNKITNNGIE |
| O42235 | LRLARNKISRIPEGVFSNLENLTMLDLHQNNLLDSALQSDT |
| O42235 | NSLKKMPLSIPANTLQLFLDNNSIEVIPENYFSAIPKVTFL |
| O42235 | TFLRLNYNKLSDDGIPPNGFNVSSILDLQLSHNQLTKIPPI |
| O42235 | PPINAHLEHLHLDHNRIKSVNGTQICPVSIAVAEDYGLYGN |
| P09620 | EDADEQDSSDLEYFFWKFTNNDSNGNVDRPLIIWLNGGPGC |
| P09620 | HKSKSTDDSEEFSGYVKYDRNLTFVSVYNASHMVPFDKSLV |
| P09620 | SEEFSGYVKYDRNLTFVSVYNASHMVPFDKSLVSRGIVDIY |
| P13134 | SSQQIPLKDHTSRQYFAVESNETLSRLEEMHPNWKYEHDVR |
| P13134 | HLVNPSFPGSDINVLDLWYNNITGAGVVAAIVDDGLDYENE |
| P13134 | TSAAAPLAAGVYTLLLEANPNLTWRDVQYLSILSAVGLEKN |
| P13134 | ENVNAQTWFYLPTLYVSQSTNSTEETLESVITISEKSLQDA |
| P43629 | HGRIFQESFNMSPVTTAHAGNYTCRGSHPHSPTGWSAPSNP |
| P43629 | GISKDPSRLVGQIHDGVSKANFSIGPMMLALAGTYRCYGSV |
| P43629 | YHLSREGGAHERRLPAVRKVNRTFQADFPLGPATHGGTYRC |
| Q96J84 | EPADQTVVAGQRAVLPCVLLNYSGIVQWTKDGLALGMGQGL |
| Q96J84 | PEDTRIDGGPVILLQAGTPHNLTCRAFNAKPAATIIWFRDG |
| Q96J84 | DYSFFTEPVSCEVHNKVGSTNVSTLVNVHFAPRIVVDPKPT |
| Q96J84 | GVLSTLTINNVMEADFQTHYNCTAWNSFGPGTAIIQLEERE |
| Q80W68 | EPADQTVVAGQRAVLPCVLLNYSGIVQWTKDGLALGMGQGL |
| Q80W68 | PEETRIDGGPVILLQAGTPYNLTCRAFNAKPAATIIWFRDG |
| Q80W68 | DYSFFTEPVSCEVYNKVGSTNVSTLVNVHFAPRIVVYPKPT |
| Q80W68 | GVLSTLTINNVMEADFQTHYNCTAWNSFGPGTAIIQLEERE |
| P10721 | FVRDPAKLFLVDRSLYGKEDNDTLVRCPLTDPEVTNYSLKG |
| P10721 | YGKEDNDTLVRCPLTDPEVTNYSLKGCQGKPLPKDLRFIPD |
| P10721 | HHGDFNYERQATLTISSARVNDSGVFMCYANNTFGSANVTT |
| P10721 | ATLTISSARVNDSGVFMCYANNTFGSANVTTTLEVVDKGFI |
| P10721 | ARVNDSGVFMCYANNTFGSANVTTTLEVVDKGFINIFPMIN |
| P10721 | NVTTTLEVVDKGFINIFPMINTTVFVNDGENVDLIVEYEAF |
| P10721 | DLIVEYEAFPKPEHQQWIYMNRTFTDKWEDYPKSENESNIR |
| P10721 | QWIYMNRTFTDKWEDYPKSENESNIRYVSELHLTRLKGTEG |
| P10721 | CPGTEQRCSASVLPVDVQTLNSSGPPFGKLVVQSSIDSSAF |
| P10721 | GPPFGKLVVQSSIDSSAFKHNGTVECKAYNDVGKTSAYFNF |
| P05532 | FGKEDSDALVRCPLTDPQVSNYSLIECDGKSLPTDLTFVPN |
| P05532 | ETLTISSARVDDSGVFMCYANNTFGSANVTTTLKVVEKGFI |
| P05532 | ARVDDSGVFMCYANNTFGSANVTTTLKVVEKGFINISPVKN |

| | |
|---|---|
| P05532 | NVTTTLKVVEKGFINISPVKNTTVFVTDGENVDLVVEYEAY |
| P05532 | DLVVEYEAYPKPEHQQWIYMNRTSANKGKDYVKSDNKSNIR |
| P05532 | QWIYMNRTSANKGKDYVKSDNKSNIRYVNQLRLTRLKGTEG |
| P05532 | CTGAEQRCTTPVSPVDVQVQNVSVSPFGKLVVQSSIDSSVF |
| P05532 | VSPFGKLVVQSSIDSSVFRHNGTVECKASNDVGKSSAFFNF |
| Q9UKR0 | SIFLLLCVLGLSQAATPKIFNGTECGRNSQPWQVGLFEGTS |
| Q9UKR0 | SGWGITNHPRNPFPDLLQCLNLSIVSHATCHGVYPGRITSN |
| P06870 | DDENTAQFVHVSESFPHPGFNMSLLENHTRQADEDYSHDLM |
| P06870 | QFVHVSESFPHPGFNMSLLENHTRQADEDYSHDLMLLRLTE |
| P06870 | EEPEVGSTCLASGWGSIEPENFSFPDDLQCVDLKILPNDEC |
| P12323 | EDEDTAQHFLVSQSVPHPDFNMSLLEPHNVLPNEDYSHDLM |
| P12323 | LQCVGLEILPSKNCDDAHIANVTGTMLCAGDLAGGKDTCVG |
| P00759 | QRRLVRQSFRHPDYIPLIVTNDTEQPVHDHSNDLMLLHLSE |
| P00759 | LQCVNIHLLSNEKCIETYKDNVTDVMLCAGEMEGGKDTCAG |
| P07288 | VCGGVLVHPQWVLTAAHCIRNKSVILLGRHSLFHPEDTGQV |
| Q9Y337 | DLGAGAGEDARSDDSSSRIINGSDCDMHTQPWQAALLLRPN |
| Q9Y337 | NLMLIKLNRRIRPTKDVRPINVSSHCPSAGTKCLVSGWGTT |
| Q9Y337 | SGWGTTKSPQVHFPKVLQCLNISVLSQKRCEDAYPRQIDDT |
| Q9Y337 | AGDKAGRDSCQGDSGGPVVCNGSLQGLVSWGDYPCARPNRP |
| P03952 | QISACHRDIYKGVDMRGVNFNVSKVSSVEECQKRCTNNIRC |
| P03952 | LPEPCHSKIYPGVDFGGEELNVTFVKGVNVCQETCTKMIRC |
| P03952 | NTGDNSVCTTKTSTRIVGGTNSSWGEWPWQVSLQVKLTAQR |
| P03952 | AHCFDGLPLQDVWRIYSGILNLSDITKDTPFSQIKEIIIHQ |
| P03952 | NYKVSEGNHDIALIKLQAPLNYTEFQKPICLPSKGDTSTIY |
| P26262 | QISACHRDIYKGLDMRGSNFNISKTDNIEECQKLCTNNFHC |
| P26262 | ALSEIGCPMDIFQHSAFADLNVSQVITPDAFVCRTICTFHP |
| P26262 | RPEPCHSKIYSGVDFEGEELNVTFVQGADVCQETCTKTIRC |
| P26262 | KLVDSPDCTTKINARIVGGTNASLGEWPWQVSLQVKLVSQT |
| P26262 | EYKVSEGNYDIALIKLQTPLNYTEFQKPICLPSKADTNTIY |
| P14272 | QLSACHQDIYEGLDMRGSNFNISKTDSIEECQKLCTNNIHC |
| P14272 | ALSEIGCPMDIFQHFAFADLNVSQVVTPDAFVCRTVCTFHP |
| P14272 | RPEPCHFKIYSGVAFEGEELNATFVQGADACQETCTKTIRC |
| P14272 | KVVESSDCTTKINARIVGGTNSSLGEWPWQVSLQVKLVSQN |
| P14272 | AHCFDGIPYPDVWRIYGGILNLSEITNKTPFSSIKELIIHQ |
| P14272 | IPYPDVWRIYGGILNLSEITNKTPFSSIKELIIHQKYKMSE |
| P14272 | KYKMSEGSYDIALIKLQTPLNYTEFQKPICLPSKADTNTIY |
| P01042 | CNDKDLFKAVDAALKKYNSQNQSNNQFVLYRITEATKTVGS |
| P01042 | ISTQSPDLEPILRHGIQYFNNNTQHSSLFMLNEVKRAQRQV |
| P01042 | AQRQVVAGLNFRITYSIVQTNCSKENFLFLTPDCKSLWNGD |
| P01042 | NSPELEETLTHTITKLNAENNATFYFKIDNVKKARVQVVAG |
| O08677 | GTKTDGSPTFYSFKYLIKEGNCSAQSGLAWQDCDFKDAEEA |

| | |
|---|---|
| O08677 | ISTDSPDLEPVLKHSIEHFNNNTDHSHLFTLRKVKSAHRQV |
| O08677 | AHRQVVAGLNFDITYTIVQTNCSKERFPSLHGDCVALPNGD |
| O08677 | NGDDGECRGNLFMDINNKIANFSQSCTLYSGDDLVEALPKP |
| P01044 | CNDQDVFKAVDAALTKYNSENKSGNQFVLYRITEVARMDNP |
| P01044 | PDTFYSLKYQIKEGDCPFQSNKTWQDCDYKDSAQAATGECT |
| P01044 | ISTKSPDLEPVLRYAIQYFNNNTSHSHLFDLKEVKRAQRQV |
| P01044 | STKSPDLEPVLRYAIQYFNNNTSHSHLFDLKEVKRAQRQVV |
| P01044 | DLKEVKRAQRQVVSGWNYEVNYSIAQTNCSKEEFSFLTPDC |
| P01044 | AQRQVVSGWNYEVNYSIAQTNCSKEEFSFLTPDCKSLSSGD |
| P01045 | PDTFYSLKYQIKEGDCPFQSNKTWQDCDYKDSAQAATGQCT |
| P01045 | ISTKSPDLEPVLRYAIQYFNNNTSHSHLFDLKEVKRAQKQV |
| P01045 | STKSPDLEPVLRYAIQYFNNNTSHSHLFDLKEVKRAQKQVV |
| P01045 | DLKEVKRAQKQVVSGWNYEVNYSIAQTNCSKEEFSFLTPDC |
| P01045 | AQKQVVSGWNYEVNYSIAQTNCSKEEFSFLTPDCKSLSSGD |
| P01045 | VCVGCPKPIPVDSPDLEEALNHSIAKLNAEHDGTFYFKIDT |
| P27809 | SSEVKFGILPKEHWSYPEWINQTKAAEIRADAATKYIYGGS |
| P01596 | PSTLSASVGDRVAITCRASQNISSWLAWYQQKPGKAPKVLI |
| P06315 | AETTLTQSPAFMSATPGDKVNISCKASQDIDDDMNWYQQKP |
| P32004 | EELGVTVYQSPHSGSFTITGNNSNFAQRFQGIYRCFASNKL |
| P32004 | ERVTMGQNGNLYFANVLTSDNHSDYICHAHFPGTRTIIQKE |
| P32004 | LRVKATNSMIDRKPRLLFPTNSSSHLVALQGQPLVLECIAE |
| P32004 | IKWLRPSGPMPADRVTYQNHNKTLQLLKVGEEDDGEYRCLA |
| P32004 | LLANAYIYVVQLPAKILTADNQTYMAVQGSTAYLLCKAFGA |
| P32004 | QWLDEDGTTVLQDERFFPYANGTLGIRDLQANDTGRYFCLA |
| P32004 | QDERFFPYANGTLGIRDLQANDTGRYFCLAANDQNNVTIMA |
| P32004 | RDLQANDTGRYFCLAANDQNNVTIMANLKVKDATQITQGPR |
| P32004 | KYFIEDGRLVIHSLDYSDQGNYSCVASTELDVVESRAQLLV |
| P32004 | EFEDKEMAPEKWYSLGKVPGNQTSTTLKLSPYVHYTFRVTA |
| P32004 | TVVTPEAAPEKNPVDVKGEGNETTNMVITWKPLRWMDWNAP |
| P32004 | QGTRGPWQEQIVSDPFLVVSNTSTFVPYEIKVQAVNSQGKG |
| P32004 | GYSGEDYPQAIPELEGIEILNSSAVLVKWRPVDLAQVKGHL |
| P32004 | VLVKWRPVDLAQVKGHLRGYNVTYWREGSQRKHSKRHIHKD |
| P32004 | GSQRKHSKRHIHKDHVVVPANTTSVILSGLRPYSSYHLEVQ |
| P32004 | DEGGKGQLSFNLRDPELRTHNLTDLSPHLRYRFQLQATTKE |
| P32004 | GEAIVREGGTMALSGISDFGNISATAGENYSVVSWVPKEGQ |
| P32004 | GTMALSGISDFGNISATAGENYSVVSWVPKEGQCNFRFHIL |
| P32004 | FKALGEEKGGASLSPQYVSYNQSSYTQWDLQPDTDYEIHLF |
| P32004 | DYEIHLFKERMFRHQMAVKTNGTGRVRLPPAGFATEGWFIG |
| P11627 | EELGVVVHEAPYSGSFTIEGNNSFAQRFQGIYRCYASNKLG |
| P11627 | ERVSMGQNGDLYFANVLTSDNHSDYICNAHFPGTRTIIQKE |
| P11627 | LRVKPTNSMIDRKPRLLFPTNSSSRLVALQGQSLILECIAE |

| | |
|---|---|
| P11627 | IKWLHPSDPMPTDRVIYQNHNKTLQLLNVGEEDDGEYTCLA |
| P11627 | LLANAYIYVVQLPARILTKDNQTYMAVEGSTAYLLCKAFGA |
| P11627 | QWLDEEGTTVLQDERFFPYANGTLSIRDLQANDTGRYFCQA |
| P11627 | QDERFFPYANGTLSIRDLQANDTGRYFCQAANDQNNVTILA |
| P11627 | RDLQANDTGRYFCQAANDQNNVTILANLQVKEATQITQGPR |
| P11627 | KYFIEDGKLVIQSLDYSDQGNYSCVASTELDEVESRAQLLV |
| P11627 | EFEDKEMAPEKWFSLGKVPGNQTSTTLKLSPYVHYTFRVTA |
| P11627 | SVVTPEAAPEKNPVDVRGEGNETNNMVITWKPLRWMDWNAP |
| P11627 | QGKQETWRKQTVSDPFLVVSNTSTFVPYEIKVQAVNNQGKG |
| P11627 | GYSGEDYPQVSPELEDITIFNSSTVLVRWRPVDLAQVKGHL |
| P11627 | VLVRWRPVDLAQVKGHLKGYNVTYWWKGSQRKHSKRHIHKS |
| P11627 | GSQRKHSKRHIHKSHIVVPANTTSAILSGLRPYSSYHVEVQ |
| P11627 | TGYLLSYHPVEGESKEQLFFNLSDPELRTHNLTNLNPDLQY |
| P11627 | EGESKEQLFFNLSDPELRTHNLTNLNPDLQYRFQLQATTQQ |
| P11627 | GEAIVREGGTMALFGKPDFGNISATAGENYSVVSWVPRKGQ |
| P11627 | GTMALFGKPDFGNISATAGENYSVVSWVPRKGQCNFRFHIL |
| P11627 | ALPEGKVSPDHQPQPQYVSYNQSSYTQWNLQPDTKYEIHLI |
| P11627 | KYEIHLIKEKVLLHHLDVKTNGTGPVRVSTTGSFASEGWFI |
| Q70KY3 | INTDYEVSTPDTGVTQSYVFNLTEVDNWMGPDGVVKEKVML |
| Q70KY3 | NIVANWGDTVEVTVINNLVTNGTSIHWHGIHQKDTNLHDGA |
| Q70KY3 | DDLVHFTQNNAPPFSDNVLINGTAVNPNTGEGQYANVTLTP |
| Q70KY3 | DNVLINGTAVNPNTGEGQYANVTLTPGKRHRLRILNTSTEN |
| Q70KY3 | RHRLRILNTSTENHFQVSLVNHTMTVIAADMVPVNAMTVDS |
| Q70KY3 | VGQRYDVVIDASRAPDNYWFNVTFGGQAACGGSLNPHPAAI |
| Q70KY3 | DNTLPVALDLTGTPLFVWKVNGSDINVDWGKPIIDYILTGN |
| Q70KY3 | NGSDINVDWGKPIIDYILTGNTSYPVSDNIVQVDAVDQWTY |
| D0VWU3 | TPGPLVAGNIGDRFQLNVIDNLTNHTMLKTTSVHWHGFFQQ |
| D0VWU3 | PLVAGNIGDRFQLNVIDNLTNHTMLKTTSVHWHGFFQQGTN |
| D0VWU3 | IKVTKGKRYRFRLVSLSCDPNFTFSIDGHNLTIIEVDSSNS |
| D0VWU3 | RFRLVSLSCDPNFTFSIDGHNLTIIEVDSSNSQPLSVDSIQ |
| D0VWU3 | GGINSAILRYDGAPAVEPTTNQTTSVKPLNEVNLHPLVSTP |
| D0VWU3 | VPGSPSSGGVDKAINMAFNFNGSNFFINGASFVPPSVPVLL |
| D0VWU3 | SGAQTAQDLLPSGSVYVLPSNASIEISFPATAAAPGAPHPF |
| D0VWU3 | PFHLHGHTFAVVRSAGSTVYNYSNPIFRDVVSTGTPAAGDN |
| D0VWU3 | NYSNPIFRDVVSTGTPAAGDNVTIRFLTNNPGPWFLHCHID |
| Q12739 | YNFDTPARRDVVNTGTGANDNVTIRFVTDNPGPWFLHCHID |
| Q12718 | PLITGKKGDRFQLNVDDTLTNHSMLKSTSIHWHGFFQAGTN |
| Q12718 | RGPFVVYDPKDPHASRYDVDNESTVITLTDWYHTAARLGPR |
| Q12718 | INVQHGKRYRFRLVSISCDPNYTFSIDGHNLTVIEVDGINS |
| Q12718 | RFRLVSISCDPNYTFSIDGHNLTVIEVDGINSQPLLVDSIQ |
| Q12718 | LLVDSIQIFAAQRYSFVLNANQTVGNYWVRANPNFGTVGFA |

| | |
|---|---|
| Q12718 | VPGSPTPGGVDKALNLAFNFNGTNFFINNATFTPPTVPVLL |
| Q12718 | GVDKALNLAFNFNGTNFFINNATFTPPTVPVLLQILSGAQT |
| Q12718 | NYNDPIFRDVVSTGTPAAGDNVTIRFQTDNPGPWFLHCHID |
| Q6GTX8 | CRGPVGVQTFRLERESRSTYNDTEDVSQASPSESEARFRID |
| P00709 | ELICTMFHTSGYDTQAIVENNESTEYGLFQISNKLWCKSSQ |
| P00709 | GLFQISNKLWCKSSQVPQSRNICDISCDKFLDDDITDDIMC |
| P30805 | EWLCVIFHESGYDSQALNYYNGSSSHGLFQINQPYWCDDXD |
| P00716 | EWICTLFHTSGLDTKITVNNNGSTEYGIFQISDKLWCVSKQ |
| P00714 | EWTCVLFHTSGYDSQAIVKNNGSTEYGLFQISNRNWCKSSE |
| Q18823 | DRHEGFSHPAKYLTDFNVGNNETWWQSDTMQEGQQYPTTTN |
| Q18823 | NETWWQSDTMQEGQQYPTTTNLTLVLGKSFDITYVRLKFIS |
| Q18823 | YNDRPWRSGTSVEANECIACNCSQLSNRCYFDQQLFEETGH |
| Q18823 | PCFCFGHSSICNTADGYFAMNVSSVFDQDKQKWAGQNRIGL |
| Q18823 | DPYFGWYPRINELDFIGILSNITAIKIRGTYSYKDIGYLSN |
| Q18823 | FRRETKFGGPFNHCIKCDCHNHSNSCEAESGSCICEHNTAG |
| Q18823 | CDCLPNVIGIQCDQCAHGFYNITSGLGCQECNCDPLGSEGN |
| Q18823 | EKVKSLDNTLQEIIENPAPVNDTKFDEKVKETSRAASEVWE |
| Q18823 | IENVLHYLETEGEERAQIAYNASQKYGEQSKRMSELASGTR |
| Q18823 | AEKHLKQASEIEQLSEQAIANATQANKEASDAIYGGEQISK |
| Q18823 | YGGEQISKQIAELKEKQNQLNESIHRTLDLAEEQKKSADEA |
| Q18823 | NLVDSSVKENSANDELFDEVNRSVADARNELQSSQDQQRVS |
| Q18823 | SLPIDKQFVIDYRKSADVLLNETHALADRYKDIIHSDVDTR |
| Q18823 | QMATEAVRKATLAKNSAIEANATILAEEDEIKKIINSLDTM |
| P25391 | RQRGLFPAILNLASNAHISTNATCGEKGPEMFCKLVEHVPG |
| P25391 | SPRKIPSQQDALGGRHQVSINNTAVMQRLAPKYYWAAPEAY |
| P25391 | FQDFHSKRQIDRDQLMTVLANVTHLLIRANYNSAKMALYRL |
| P25391 | ECHGHAAECNVHGVCIACAHNTTGVHCEQCLPGFYGEPSRG |
| P25391 | VKGSHSAVCHLETGLCDCKPNVTGQQCDQCLHGYYGLDSGH |
| P25391 | CDQCLHGYYGLDSGHGCRPCNCSVAGSVSDGCTDEGQCHCV |
| P25391 | CEECEDGHWGYDAEVGCQACNCSLVGSTHHRCDVVTGHCQC |
| P25391 | AGSDRGPRPLVAPCVPCSCNNHSDTCDPNTGKCLNCGDNTA |
| P25391 | ECVGVLLNDLDEIGDAVLSLNLTGIIPVPYGILSNLENTTK |
| P25391 | LSLNLTGIIPVPYGILSNLENTTKYLQESLLKENMQKDLGK |
| P25391 | AIAIERLQMSITEIMEKTTLNQTLDEDFLLPNSTLQNMQQN |
| P25391 | TEIMEKTTLNQTLDEDFLLPNSTLQNMQQNGTSLLEIMQIR |
| P25391 | NQTLDEDFLLPNSTLQNMQQNGTSLLEIMQIRDFTQLHQNA |
| P25391 | QNGTSLLEIMQIRDFTQLHQNATLELKAAEDLLSQIQENYQ |
| P25391 | MVNANLREFSDKKLHVQEEQNLTSELIVQGRGLIDAAAAQT |
| P25391 | HAAEFQRLADVLYSGLENIRNVSLNATSAAYVHYNIQSLIE |
| P25391 | FQRLADVLYSGLENIRNVSLNATSAAYVHYNIQSLIEESEE |
| P25391 | LVSNGKAAVQRSSRFLKEGNNLSRKLPGIALELSELRNKTN |

| | |
|---|---|
| P25391 | EGNNLSRKLPGIALELSELRNKTNRFQENAVEITRQTNESL |
| P25391 | ELRNKTNRFQENAVEITRQTNESLLILRAIPKGIRDKGAKT |
| P25391 | ASQSAVSTLRDVAGLSQELLNTSASLSRVNTTLRETHQLLQ |
| P25391 | RDVAGLSQELLNTSASLSRVNTTLRETHQLLQDSTMATLLA |
| P25391 | VEIQANLLFDRLKPLKMLEENLSRNLSEIKLLISQARKQAA |
| P25391 | ANLLFDRLKPLKMLEENLSRNLSEIKLLISQARKQAASIKV |
| P25391 | NQKSPTKTSKSPGTANVLDVNNSTLMFVGGLGGQIKKSPAV |
| P25391 | QKSPTKTSKSPGTANVLDVNNSTLMFVGGLGGQIKKSPAVK |
| P25391 | KVMTDLGSGPITLLTDRRYNNGTWYKIAFQRNRKQGVLAVI |
| P25391 | YKIAFQRNRKQGVLAVIDAYNTSNKETKQGETPGASSDLNR |
| P25391 | ELPPKSLSPESEWLVTFATTNSSGIILAALGGDVEKRGDRE |
| P25391 | LDENNPVEMKLGTLVESRTINVSNLYVGGIPEGEGTSLLTM |
| P25391 | YVPGAHQFGLTQNSHFILPFNQSAVRKKLSVELSIRTFASS |
| P25391 | VEGLFYLGGLPSQYQARKIGNITHSIPACIGDVTVNSKQLD |
| P25391 | FDGSGYAALVKEGYKVQSDVNITLEFRTSSQNGVLLGISTA |
| P25391 | AYEPKTATVLCDGKWHTLQANKSKHRITLIVDGNAVGAESP |
| P19137 | QQRGLFPAILNLATNAHISANATCGEKGPEMFCKLVEHVPG |
| P19137 | LVEHVPGRPVRHAQCRVCDGNSTNPRERHPISHAIDGTNNW |
| P19137 | AKERRSLNTAGQYSGGGVCVNCSQNTTGINCETCIDQYYRP |
| P19137 | RSLNTAGQYSGGGVCVNCSQNTTGINCETCIDQYYRPHKVS |
| P19137 | FCFGVSGVCDSLTWSISQVTNMSGWLVTDLMSTNKIRSQQD |
| P19137 | STNKIRSQQDVLGGHRQISINNTAVMQRLTSTYYWAAPEAY |
| P19137 | FRDFNTRREIDRDQLMTVLANVTHLLIRANYNSAKMALYRL |
| P19137 | ECHGHASECDIHGICSVCTHNTTGDHCEQCLPGFYGTPSRG |
| P19137 | RCADGYYGNPTVPGGTCVPCNCSGNVDPLEAGHCDSVTGEC |
| P19137 | DGFYGDAVTAKNCRACDCHENGSLSGVCHLETGLCDCKPHV |
| P19137 | CDQCLSGYYGLDTGLGCVPCNCSVEGSVSDNCTEEGQCHCG |
| P19137 | LDTGLGCVPCNCSVEGSVSDNCTEEGQCHCGPGVSGKQCDR |
| P19137 | CEECEEAYWGLDPEQGCQACNCSAVGSTSAQCDVLSGHCPC |
| P19137 | IDYILIKASYGQGLQQSRIANISMEVGRKAVELPAEGEAAL |
| P19137 | ESGGRGPRPLLAPCVPCNCNNHSDVCDPETGKCLSCRDHTS |
| P19137 | DCVGPLLNDLDSVGDAVLSLNLTGVSPAPYGILENLENTTK |
| P19137 | LSLNLTGVSPAPYGILENLENTTKYFQRYLIKENAKKIRAE |
| P19137 | ELTRVLARHQKVNAEMERTSNGTQALATFIEQLHANIKEIT |
| P19137 | TFIEQLHANIKEITEKVATLNQTARKDFQPPVSALQSMHQN |
| P19137 | NQTARKDFQPPVSALQSMHQNISSLLGLIKERNFTEMQQNA |
| P19137 | SALQSMHQNISSLLGLIKERNFTEMQQNATLELKAAKDLLS |
| P19137 | QNISSLLGLIKERNFTEMQQNATLELKAAKDLLSRIQKRFQ |
| P19137 | RFQKPQEKLKALKEANSLLSNHSEKLQAAEELLKEAGSKTQ |
| P19137 | VKANLKEEFQEKKLRVQEEQNVTSELIAKGREWVDAAGTHT |
| P19137 | HASELQSRAGALDRDLENVRNVSLNATSAAHVHSNIQTLTE |

| | |
|---|---|
| P19137 | LQSRAGALDRDLENVRNVSLNATSAAHVHSNIQTLTEEAEM |
| P19137 | NIQTLTEEAEMLAADAHKTANKTDLISESLASRGKAVLQRS |
| P19137 | ESVGTRRKQQGITMKLDELKNLTSQFQESVDNITKQANDSL |
| P19137 | ITMKLDELKNLTSQFQESVDNITKQANDSLAMLRESPGGMR |
| P19137 | ELKNLTSQFQESVDNITKQANDSLAMLRESPGGMREKGRKA |
| P19137 | ESPGGMREKGRKARELAAAANESAVKTLEDVLALSLRVFNT |
| P19137 | ANESAVKTLEDVLALSLRVFNTSEDLSRVNATVQETNDLLH |
| P19137 | EDVLALSLRVFNTSEDLSRVNATVQETNDLLHNSTMTTLLA |
| P19137 | LRVFNTSEDLSRVNATVQETNDLLHNSTMTTLLAGRKMKDM |
| P19137 | TSEDLSRVNATVQETNDLLHNSTMTTLLAGRKMKDMEMQAN |
| P19137 | MEMQANLLLDRLKPLKTLEENLSRNLSEIKLLISRARKQAA |
| P19137 | ANLLLDRLKPLKTLEENLSRNLSEIKLLISRARKQAASIKV |
| P19137 | AENPPVRTSKSPGPSKVLDINNSTLMFVGGLGGQIKKSPAV |
| P19137 | ENPPVRTSKSPGPSKVLDINNSTLMFVGGLGGQIKKSPAVK |
| P19137 | QIVILFSTFSPNGLLFYLASNGTKDFLSIELVRGRVKVMVD |
| P19137 | KVMVDLGSGPLTLMTDRRYNNGTWYKIAFQRNRKQGLLAVF |
| P19137 | EMPPKSLSPESSLLATFATKNSSGILLVALGKDAEEAGGAQ |
| P19137 | YVAGAHQFGLSQNSHLVLPLNQSDVRKRLQVQLSIRTFASS |
| P19137 | KRKAFMTVDGQESPSVTVVGNATTLDVERKLYLGGLPSHYR |
| P19137 | FEGSGYAALVKEGYKVRLDLNITLEFRTTSKNGVLLGISSA |
| P24043 | QQRGLFPAVLNLASNALITTNATCGEKGPEMYCKLVEHVPG |
| P24043 | LVEHVPGQPVRNPQCRICNQNSSNPNQRHPITNAIDGKNTW |
| P24043 | VGGMCICYGHARACPLDPATNKSRCECEHNTCGDSCDQCCP |
| P24043 | ACNCHGKAEECYYDENVARRNLSLNIRGKYIGGGVCINCTQ |
| P24043 | ARRNLSLNIRGKYIGGGVCINCTQNTAGINCETCTDGFFRP |
| P24043 | GVSCDRCARGYTGYPDCKACNCSGLGSKNEDPCFGPCICKE |
| P24043 | CPPGYTGSSCESCWPRHRRVNGTIFGGICEPCQCFGHAESC |
| P24043 | CSKCAPNTWGHSITTGCKACNCSTVGSLDFQCNVNTGQCNC |
| P24043 | ECTGLLLGDLARLEQMVMSINLTGPLPAPYKMLYGLENMTQ |
| P24043 | MSINLTGPLPAPYKMLYGLENMTQELKHLLSPQRAPERLIQ |
| P24043 | EFIKELARDAEAVNEKAIKLNETLGTRDEAFERNLEGLQKE |
| P24043 | LREATDKIREANRLFAVNQKNMTALEKKKEAVESGKRQIEN |
| P24043 | IKDRKLAEKVSQAESHAAQLNDSSAVLDGILDEAKNISFNA |
| P24043 | HAAQLNDSSAVLDGILDEAKNISFNATAAFKAYSNIKDYID |
| P24043 | LNDSSAVLDGILDEAKNISFNATAAFKAYSNIKDYIDEAEK |
| P24043 | GLKTRIENADARNGDLLRTLNDTLGKLSAIPNDTAAKLQAV |
| P24043 | RNGDLLRTLNDTLGKLSAIPNDTAAKLQAVKDKARQANDTA |
| P24043 | AIPNDTAAKLQAVKDKARQANDTAKDVLAQITELHQNLDGL |
| P24043 | ADRLIDKLKPIKELEDNLKKNISEIKELINQARKQANSIKV |
| P24043 | PDLTIDDSYWYRIVASRTGRNGTISVRALDGPKASIVPSTH |
| P24043 | TIQFDGEGYALVSRPIRWYPNISTVMFKFRTFSSSALLMYL |

| | |
|---|---|
| P24043 | QNHNDGKWKSFTLSRIQKQANISIVDIDTNQEENIATSSSG |
| P24043 | FGLDLKADDKIYFGGLPTLRNLSMKARPEVNLKKYSGCLKD |
| P24043 | FPKPGFVELSPVPIDVGTEINLSFSTKNESGIILLGSGGTP |
| P24043 | ELSPVPIDVGTEINLSFSTKNESGIILLGSGGTPAPPRRKR |
| P24043 | VERTRGIFTVQVDENRRYMQNLTVEQPIEVKKLFVGGAPPE |
| P24043 | HKIKIMRSKQEGILYVDGASNRTISPKKADILDVVGMLYVG |
| P24043 | PKKADILDVVGMLYVGGLPINYTTRRIGPVTYSIDGCVRNL |
| Q16363 | CNGNSNECLDGSGYCVHCQRNTTGEHCEKCLDGYIGDSIRG |
| Q16363 | PNLIFEDCDEVTGQCRNCLRNTTGFKCERCAPGYYGDARIA |
| Q16363 | KSGVLSVSSGAAAHRHVNEINATIYLLKTKLSERENQYALR |
| Q16363 | EEADEAYELLSQAESWQRLHNETRTLFPVVLEQLDDYNAKL |
| Q16363 | ARQRDHEKQQERVREQMEVVNMSLSTSADSLTTPRLTLSEL |
| Q16363 | SADSLTTPRLTLSELDDIIKNASGIYAEIDGAKSELQVKLS |
| Q16363 | ASGIYAEIDGAKSELQVKLSNLSNLSHDLVQEAIDHAQDLQ |
| Q16363 | IYAEIDGAKSELQVKLSNLSNLSHDLVQEAIDHAQDLQQEA |
| Q16363 | QKALDASNVYENIVNYVSEANETAEFALNTTDRIYDAVSGI |
| Q16363 | VYENIVNYVSEANETAEFALNTTDRIYDAVSGIDTQIIYHK |
| Q16363 | AERGDAQQRLGQSRLITEEANRTTMEVQQATAPMANNLTNW |
| Q16363 | TEEANRTTMEVQQATAPMANNLTNWSQNLQHFDSSAYNTAV |
| Q16363 | ANRTTMEVQQATAPMANNLTNWSQNLQHFDSSAYNTAVNSA |
| Q16363 | QHFDSSAYNTAVNSARDAVRNLTEVVPQLLDQLRTVEQKRP |
| Q16363 | EVVPQLLDQLRTVEQKRPASNVSASIQRIRELIAQTRSVAS |
| Q16363 | TRFDIEVRTPADNGLILLMVNGSMFFRLEMRNGYLHVFYDF |
| Q16363 | PNGLLFYYASGSDVFSISLDNGTVIMDVKGIKVQSVDKQYN |
| Q16363 | TQASEKKFYFGGSPISAQYANFTGCISNAYFTRVDRDVEVE |
| Q16363 | SLYECPIESSPLFLLHKKGKNLSKPKASQNKKGGKSKDAPS |
| O15230 | RPTEDLYCKLVGGPVAGGDPNQTIRGQYCDICTAANSNKAH |
| O15230 | GTERWWQSPPLSRGLEYNEVNVTLDLGQVFHVAYVLIKFAN |
| O15230 | VPLENGEIVVSLVNGRPGAMNFSYSPLLREFTKATNVRLRF |
| O15230 | ESDFTDGTCEDLTGRCYCRPNFSGERCDVCAEGFTGFPSCY |
| O15230 | DVCAEGFTGFPSCYPTPSSSNDTREQVLPAGQIVNCDCSAA |
| O15230 | EAATPEGHAVRFGFNPLEFENFSWRGYAQMAPVQPRIVARL |
| O15230 | FSWRGYAQMAPVQPRIVARLNLTSPDLFWLVFRYVNRGAMS |
| O15230 | AMSVSGRVSVREEGRSATCANCTAQSQPVAFPPSTEPAFIT |
| O15230 | HPTFPVEVLINAGRVWQGHANASFCPHGYGCRTLVVCEGQA |
| O15230 | CLLCQPQTFGCHPLVGCEECNCSGPGIQELTDPTCDTDSGQ |
| O15230 | IQELTDPTCDTDSGQCKCRPNVTGRRCDTCSPGFHGYPRCR |
| O15230 | DDLERAGALLPAIHEQLRGINASSMAWARLHRLNASIADLQ |
| O15230 | HEQLRGINASSMAWARLHRLNASIADLQSQLRSPLGPRHET |
| O15230 | RAVDRTLSELMSQTGHLGLANASAPSGEQLLRTLAEVERLL |
| O15230 | SRNQERLEEALQRKQELSRDNATLQATLHAARDTLASVFRL |

| | |
|---|---|
| O15230 | KLRLVEAAEAHAQQLGQLALNLSSIILDVNQDRLTQRAIEA |
| O15230 | HTWATVVRQGLVDRAQQLLANSTALEEAMLQEQQRLGLVWA |
| O15230 | EKTLPQLLAKLSILENRGVHNASLALSASIGRVRELIAQAR |
| O15230 | VRGCVKGIKALGKYVDLKRLNTTGVSAGCTADLLVGRAMTF |
| O15230 | EVKTQAGFADGAPHYVAFYSNATGVWLYVDDQLQQMKPHRG |
| O15230 | QPEGPPRLLLGGLPESGTIYNFSGCISNVFVQRLLGPQRVF |
| O15230 | VQRLLGPQRVFDLQQNLGSVNVSTGCAPALQAQTPGLGPRG |
| O15230 | HRLAVMKSGNVLRLEVDAQSNHTVGPLLAAAAGAPAPLYLG |
| Q61001 | RPTEDLYCKLVGGPVAGGDPNQTIQGQYCDICTAANSNKAH |
| Q61001 | GTERWWQSPPLSRGLEYNEVNVTLDLGQVFHVAYVLIKFAN |
| Q61001 | VPLENGEIVVSLVNGRPGALNFSYSPLLRDFTKATNIRLRF |
| Q61001 | SCNCHGHAYDCYYDPEVDRRNASQNQDNVYQGGGVCLDCQH |
| Q61001 | ESDFTDGTCEDLTGRCYCRPNFTGELCAACAEGYTDFPHCY |
| Q61001 | ACAEGYTDFPHCYPLPSFPHNDTREQVLPAGQIVNCDCNAA |
| Q61001 | EAATPEGHAVRFGFNPLEFENFSWRGYAHMMAIQPRIVARL |
| Q61001 | FSWRGYAHMMAIQPRIVARLNVTSPDLFRLVFRYVNRGSTS |
| Q61001 | STSVNGQISVREEGKLSSCTNCTEQSQPVAFPPSTEPAFVT |
| Q61001 | HPSFPVEVLINGGRIWQGHANASFCPHGYGCRTLVLCEGQT |
| Q61001 | CLVCQPQSFGCHPLVGCEECNCSGPGVQELTDPTCDMDSGQ |
| Q61001 | TGPHCERCAPGFYGNALLPGNCTRCDCSPCGTETCDPQSGR |
| Q61001 | DDLERAGALLPAIREQLQGINASSAAWARLHRLNASIADLQ |
| Q61001 | REQLQGINASSAAWARLHRLNASIADLQSKLRSPPGPRYQA |
| Q61001 | AEAQRLMARVQEQLTSFWEENQSLATHIRDQLAQYESGLMD |
| Q61001 | QLAQYESGLMDLREALNQAVNTTREAEELNSRNQERLKEAL |
| Q61001 | SRNQERLKEALQWKQELSQDNATLKATLQAASLILGHVSEL |
| Q61001 | KVDLVEAAEAHAQKLNQLAINLSGIILGINQDRFIQRAVEA |
| Q61001 | RTWEMVVQRGLAAGARQLLANSSALEETILGHQGRLGLAQG |
| Q61001 | EKTLPQLLAKLSRLENRGVHNASLALSANIGRVRKLIAQAR |
| Q61001 | VRGCIKGIKALGKYVDLKRLNTTGISFGCTADLLVGRTMTF |
| Q61001 | EVETQRVFADGAPHYVAFYSNVTGVWLYVDDQLQLVKSHER |
| Q61001 | QPEEPSRLLLGGLPVSGTFHNFSGCISNVFVQRLRGPQRVF |
| Q61001 | VQRLRGPQRVFDLHQNMGSVNVSVGCTPAQLIETSRATAQK |
| Q61001 | HRVAVIMGRDTLRLEVDTQSNHTTGRLPESLAGSPALLHLG |
| Q61001 | PELPAYRGCLRKLLINGAPVNVTASVQIQGAVGMRGCPSGT |
| Q00174 | GTEAWWQSPPLSRGMKFNEVNLTINFEQEFHVAYLFIRMGN |
| Q00174 | ENGEIPVMLLNERPSSTNYFNSTVLQEWTRATNVRIRLLRT |
| Q00174 | VGINCNKCKPKYYRPKGKHWNETDVCSPCQCDYFFSTGHCE |
| Q00174 | DSCAYGYYGYPNCRECECNLNGTNGYHCEAESGQQCPCKIN |
| Q00174 | GFPECKACECNKIGSITNDCNVTTGECKCLTNFGGDNCERC |
| Q00174 | GPRCDQCLPGFYNYPDCKPCNCSSTGSSAITCDNTGKCNCL |
| Q00174 | CKERVTGRICNECKPLYWNLNISNTEGCEICDCWTDGTISA |

| | |
|---|---|
| Q00174 | NVFKSSLYRIVLRYVNPNAENVTATISVTSDNPLEVDQHVK |
| Q00174 | FDQTKEFIQNCGHDHFHITHNASDFCKKSVFSLTADYNSGA |
| Q00174 | EECACNPMGIANGNSQCDLFNGTCECRQNIEGRACDVCSNG |
| Q00174 | TTCFCFGKTSRCDSAYLRVYNVSLLKHVSITTPEFHEESIK |
| Q00174 | FHEESIKFDMWPVPADEILLNETTLKADFTLREVNDERPAY |
| Q00174 | KCQHGTEGDHCERCVSGYYGNATNGTPGDCMICACPLPFDS |
| Q00174 | INPEDQGSCDTRTGECLRCLNNTFGAACNLCAPGFYGDAIK |
| Q00174 | DHYGFESGVGCRACDCGAASNSTQCDPHTGHCACKSGVTGR |
| Q00174 | PSKKANSELESDAKSYAKQVNQTLANAFDIRERSSTTLGNI |
| Q00174 | VNQTLANAFDIRERSSTTLGNITVAYDEAVKSADQAKEAIA |
| Q00174 | AASTKIDAALEQAQHILGQINGTSIELTPNEQVLEKARKLY |
| Q00174 | EKARKLYEEVNTLVLPIKAQNKSLNALKNDIGEFSDHLEDL |
| Q00174 | SLNALKNDIGEFSDHLEDLFNWSEASQAKSADVERRNVANQ |
| Q00174 | IVEAVEAAQKLSQDAISAAGNATDKTDGIEERAHLADTGST |
| Q00174 | LLQRARQSLQKVQDDLEPRLNASAGKVQKISAVNNATEHQL |
| Q00174 | DLEPRLNASAGKVQKISAVNNATEHQLKDINKLIDQLPAES |
| Q00174 | KLIDQLPAESQRDMWKNSNANASDALEILKNVLEILEPVSV |
| Q00174 | PSTILELKTPEKTKLLATRTNLSTYFRTTEPSGFLLYLGND |
| Q00174 | LSTYFRTTEPSGFLLYLGNDNKTAQKNNDFVAVEIVNGYPI |
| Q00174 | LEKKKPVTGLRFKGNGYVQLNATSNLKSRSSIQFSFKADKD |
| Q00174 | FYGRDKHYMSIEMIDGAIFFNISLGEGGGVQSGSQDRYNDN |
| Q00174 | ADLELPKLRRLYFGGHPRRLNTSISLQPNFDGCIDNVVINQ |
| Q00174 | AKFSTVVSYAPHEYGFLRMNNVSSDNNLHVVLHFKTTQPNG |
| Q00174 | AVELPIYLGGVNKFLESEVKNLTDFKTEVPYFNGCLKNIKF |
| Q00174 | YFTVKTDLKNIVSTNYKLPNNESFCDGKTRNVQAIKSKFVI |
| P11046 | YKTKPGTNIPTWWQSENGKENATIQLDLEAEFHFTHLIITF |
| P11046 | YRYFAYDCKESFPGVPTVLENITDVMCTSRYSNVEPSRNGE |
| P11046 | SNVEPSRNGEVIFRVLPPNINVTDPYAEHVQNQLKMTNLRI |
| P11046 | LQSDNPEGCEPCTCNPLGTLNNSGCVMRTGECKCKKYVTGK |
| P11046 | LPEVHEAEVVDECISYGANGNCSLVAETPDGSFTGIGFTRV |
| P11046 | FFGDALQQNCQQCECDFLGTNNTIAHCDRFTGQCPCLPNVQ |
| P11046 | YTSEFSELDKKLQHIRNLLQNTSVSLVDIEKLDYETQSLRD |
| P11046 | NLDDIYNSLSLSGVELESLQNHSRLVQQLSKELKENGIQLQ |
| P11046 | SKELKENGIQLQESNIEGALNLTRHAYERVSNLSTLKDEAN |
| P11046 | QESNIEGALNLTRHAYERVSNLSTLKDEANELASNTDRNCK |
| P11046 | ITSKKDQADQTIRALTQAKLNASEAYEKAKRGFEQSERYLN |
| P11046 | NASEAYEKAKRGFEQSERYLNQTNANIKLAENLFIALNNFQ |
| P11046 | TNANIKLAENLFIALNNFQENKTASPSESKELAQKTLDLDL |
| P11046 | AIIYRTKPDLDRVNNLQSIANATKEKADKILDSANSVVESL |
| P11046 | LAGQDLEKIDEETYSAEAPANNTAQQVEKLAKKVQKLQNNI |
| P11046 | LEAMRARGEANNLQSATSATNQTLTDRASRSENARERAKQL |

| | |
|---|---|
| P07942 | VTTFAPNRLKIWWQSENGVENVTIQLDLEAEFHFTHLIMTF |
| P07942 | NCNEHSISCHFDMAVYLATGNVSGGVCDDCQHNTMGRNCEQ |
| P07942 | GLSNDLDGCRPCDCDLGGALNNSCFAESGQCSCRPHMIGRQ |
| P07942 | LSPGSRYVVLPRPVCFEKGTNYTVRLELPQYTSSDSDVESP |
| P07942 | LQQDCRKCVCNYLGTVQEHCNGSDCQCDKATGQCLCLPNVI |
| P07942 | DCTPCHQCFALWDVIIAELTNRTHRFLEKAKALKISGVIGP |
| P07942 | DVTEMMAQVEVKLSDTTSQSNSTAKELDSLQTEAESLDNTV |
| P07942 | GALDSITKYFQMSLEAEERVNASTTEPNSTVEQSALMRDRV |
| P07942 | KYFQMSLEAEERVNASTTEPNSTVEQSALMRDRVEDVMMER |
| P07942 | EAKLRADEAKQSAEDILLKTNATKEKMDKSNEELRNLIKQI |
| P07942 | EAVANEVLKMEMPSTPQQLQNLTEDIRERVESLSQVEVILQ |
| P02469 | VTTFAPNRLKIWWQSENGVENVTIQLDLEAEFHFTHLIMTF |
| P02469 | NCNEHSSSCHFDMAVFLATGNVSGGVCDNCQHNTMGRNCEQ |
| P02469 | GLSNDLDGCRPCDCDLGGALNNSCSEDSGQCSCLPHMIGRQ |
| P02469 | LSPGSRYVVLPRPVCFEKGMNYTVRLELPQYTASGSDVESP |
| P02469 | LRQDCRKCVCNYLGTVKEHCNGSDCHCDKATGQCSCLPNVI |
| P02469 | DCTPCHQCFALWDAIIGELTNRTHKFLEKAKALKISGVIGP |
| P02469 | DVTEKMAQVEVKLTDTASQSNSTAGELGALQAEAESLDKTV |
| P02469 | GALDSITKYFQMSLEAEKRVNASTTDPNSTVEQSALTRDRV |
| P02469 | KYFQMSLEAEKRVNASTTDPNSTVEQSALTRDRVEDLMLER |
| P02469 | EAKVRADEAKQNAQDVLLKTNATKEKVDKSNEDLRNLIKQI |
| P02469 | EAVANEVLKMEMPSTPQQLQNLTEDIRERVETLSQVEVILQ |
| P02469 | TQNLLTSIESETAASEETLTNASQRISKLERNVEELKRKAA |
| P55268 | IPDPYSSRIQNLLKITNLRVNLTRLHTLGDNLLDPRREIRE |
| P55268 | ECHGHTHSCHFDMAVYLASGNVSGGVCDGCQHNTAGRHCEL |
| P55268 | CPCLPNVQGPSCDRCAPNFWNLTSGHGCQPCACHPSRARGP |
| P55268 | SSFWHMQEKLGIVQGIVGARNTSAASTAQLVEATEELRREI |
| P55268 | DENFNANHALSGLERDRLALNLTLRQLDQHLDLLKHSNFLG |
| P55268 | GAYDSIRHAHSQSAEAERRANTSALAVPSPVSNSASARHRT |
| P55268 | ETRRQASEAQQRAQAALDKANASRGQVEQANQELQELIQSV |
| P11047 | CTDEGGRPQRCMPEFVNAAFNVTVVATNTCGTPPEEYCVQT |
| P11047 | DTTWWQSQTMLAGVQYPSSINLTLHLGKAFDITYVRLKFHT |
| P11047 | PRYFIAPAKFLGKQVLSYGQNLSFSFRVDRRDTRLSAEDLV |
| P11047 | ATDYPWRPALTPFEFQKLLNNLTSIKIRGTYSERSAGYLDD |
| P11047 | ECREGFVGNRCDQCEENYFYNRSWPGCQECPACYRLVKDKV |
| P11047 | REAQDVKDVDQNLMDRLQRVNNTLSSQISRLQNIRNTIEET |
| P11047 | TERLIEIASRELEKAKVAAANVSVTQPESTGDPNNMTLLAE |
| P11047 | AKVAAANVSVTQPESTGDPNNMTLLAEEARKLAERHKQEAD |
| P11047 | KLAERHKQEADDIVRVAKTANDTSTEAYNLLLRTLAGENQT |
| P11047 | TANDTSTEAYNLLLRTLAGENQTAFEIEELNRKYEQAKNIS |
| P11047 | GENQTAFEIEELNRKYEQAKNISQDLEKQAARVHEEAKRAG |

| | |
|---|---|
| P11047 | LQEANDILNNLKDFDRRVNDNKTAAEEALRKIPAINQTITE |
| P11047 | RRVNDNKTAAEEALRKIPAINQTITEANEKTREAQQALGSA |
| P11047 | ATEAKNKAHEAERIASAVQKNATSTKAEAERTFAEVTDLDN |
| P02468 | CADEGGRPQRCMPEFVNAAFNVTVVATNTCGTPPEEYCVQT |
| P02468 | DTTWWQSQTMLAGVQYPNSINLTLHLGKAFDITYVRLKFHT |
| P02468 | PRYFIAPVKFLGNQVLSYGQNLSFSFRVDRRDTRLSAEDLV |
| P02468 | ATDYPWRPALSPFEFQKLLNNLTSIKIRGTYSERTAGYLDD |
| P02468 | ECREGFVGNRCDQCEENYFYNRSWPGCQECPACYRLVKDKA |
| P02468 | REAQEVKDVDQNLMDRLQRVNSSLHSQISRLQNIRNTIEET |
| P02468 | TEQLIEIASRELEKAKMAAANVSITQPESTGEPNNMTLLAE |
| P02468 | AKMAAANVSITQPESTGEPNNMTLLAEEARRLAERHKQEAD |
| P02468 | RLAERHKQEADDIVRVAKTANETSAEAYNLLLRTLAGENQT |
| P02468 | TANETSAEAYNLLLRTLAGENQTALEIEELNRKYEQAKNIS |
| P02468 | GENQTALEIEELNRKYEQAKNISQDLEKQAARVHEEAKRAG |
| P02468 | LQEANDILNNLKDFDRRVNDNKTAAEEALRRIPAINRTIAE |
| P02468 | RRVNDNKTAAEEALRRIPAINRTIAEANEKTREAQLALGNA |
| P02468 | ATEAKNKAHEAERIASAVQKNATSTKADAERTFGEVTDLDN |
| P11279 | LLLLGLMHCASAAMFMVKNGNGTACIMANFSAAFSVNYDTK |
| P11279 | CASAAMFMVKNGNGTACIMANFSAAFSVNYDTKSGPKNMTF |
| P11279 | IMANFSAAFSVNYDTKSGPKNMTFDLPSDATVVLNRSSCGK |
| P11279 | TKSGPKNMTFDLPSDATVVLNRSSCGKENTSDPSLVIAFGR |
| P11279 | TFDLPSDATVVLNRSSCGKENTSDPSLVIAFGRGHTLTLNF |
| P11279 | ENTSDPSLVIAFGRGHTLTLNFTRNATRYSVQLMSFVYNLS |
| P11279 | DPSLVIAFGRGHTLTLNFTRNATRYSVQLMSFVYNLSDTHL |
| P11279 | TLNFTRNATRYSVQLMSFVYNLSDTHLFPNASSKEIKTVES |
| P11279 | RYSVQLMSFVYNLSDTHLFPNASSKEIKTVESITDIRADID |
| P11279 | IRADIDKKYRCVSGTQVHMNNVTVTLHDATIQAYLSNSSFS |
| P11279 | VHMNNVTVTLHDATIQAYLSNSSFSRGETRCEQDRPSPTTA |
| P11279 | PAPPSPSPSPVPKSPSVDKYNVSGTNGTCLLASMGLQLNLT |
| P11279 | PSPSPVPKSPSVDKYNVSGTNGTCLLASMGLQLNLTYERKD |
| P11279 | KYNVSGTNGTCLLASMGLQLNLTYERKDNTTVTRLLNINPN |
| P11279 | GTCLLASMGLQLNLTYERKDNTTVTRLLNINPNKTSASGSC |
| P11279 | NLTYERKDNTTVTRLLNINPNKTSASGSCGAHLVTLELHSE |
| P11279 | LVTLELHSEGTTVLLFQFGMNASSSRFFLQGIQLNTILPDA |
| P11279 | QGIQLNTILPDARDPAFKAANGSLRALQATVGNSYKCNAEE |
| P11438 | LLLLLAGLAHGASALFEVKNNGTTCIMASFSASFLTTYETA |
| P11438 | GTTCIMASFSASFLTTYETANGSQIVNISLPASAEVLKNGS |
| P11438 | ASFSASFLTTYETANGSQIVNISLPASAEVLKNGSSCGKEN |
| P11438 | TANGSQIVNISLPASAEVLKNGSSCGKENVSDPSLTITFGR |
| P11438 | NISLPASAEVLKNGSSCGKENVSDPSLTITFGRGYLLTLNF |
| P11438 | ENVSDPSLTITFGRGYLLTLNFTKNTTRYSVQHMYFTYNLS |

| | |
|---|---|
| P11438 | DPSLTITFGRGYLLTLNFTKNTTRYSVQHMYFTYNLSDTEH |
| P11438 | TLNFTKNTTRYSVQHMYFTYNLSDTEHFPNAISKEIYTMDS |
| P11438 | IKADINKAYRCVSDIRVYMKNVTVVLRDATIQAYLSSGNFS |
| P11438 | MKNVTVVLRDATIQAYLSSGNFSKEETHCTQDGPSPTTGPP |
| P11438 | TGPPSPSPPLVPTNPTVSKYNVTGNNGTCLLASMALQLNIT |
| P11438 | PSPPLVPTNPTVSKYNVTGNNGTCLLASMALQLNITYLKKD |
| P11438 | KYNVTGNNGTCLLASMALQLNITYLKKDNKTVTRAFNISPN |
| P11438 | GTCLLASMALQLNITYLKKDNKTVTRAFNISPNDTSSGSCG |
| P11438 | NITYLKKDNKTVTRAFNISPNDTSSGSCGINLVTLKVENKN |
| P11438 | NLVTLKVENKNRALELQFGMNASSSLFFLQGVRLNMTLPDA |
| P11438 | ELQFGMNASSSLFFLQGVRLNMTLPDALVPTFSISNHSLKA |
| P11438 | QGVRLNMTLPDALVPTFSISNHSLKALQATVGNSYKCNTEE |
| P13473 | SGLVLVCLVLGAVRSYALELNLTDSENATCLYAKWQMNFTV |
| P13473 | CLVLGAVRSYALELNLTDSENATCLYAKWQMNFTVRYETTN |
| P13473 | LELNLTDSENATCLYAKWQMNFTVRYETTNKTYKTVTISDH |
| P13473 | NATCLYAKWQMNFTVRYETTNKTYKTVTISDHGTVTYNGSI |
| P13473 | ETTNKTYKTVTISDHGTVTYNGSICGDDQNGPKIAVQFGPG |
| P13473 | DDQNGPKIAVQFGPGFSWIANFTKAASTYSIDSVSFSYNTG |
| P13473 | TKAASTYSIDSVSFSYNTGDNTTFPDAEDKGILTVDELLAI |
| P13473 | LEKNDVVQHYWDVLVQAFVQNGTVSTNEFLCDKDKTSTVAP |
| P13473 | TTTPTPKEKPEAGTYSVNNGNDTCLLATMGLQLNITQDKVA |
| P13473 | TYSVNNGNDTCLLATMGLQLNITQDKVASVININPNTTHST |
| P13473 | MGLQLNITQDKVASVININPNTTHSTGSCRSHTALLRLNSS |
| P13473 | NPNTTHSTGSCRSHTALLRLNSSTIKYLDFVFAVKNENRFY |
| P13473 | KYLDFVFAVKNENRFYLKEVNISMYLVNGSVFSIANNNLSY |
| P13473 | AVKNENRFYLKEVNISMYLVNGSVFSIANNNLSYWDAPLGS |
| P13473 | KEVNISMYLVNGSVFSIANNNLSYWDAPLGSSYMCNKEQTV |
| P13473 | TVSVSGAFQINTFDLRVQPFNVTQGKYSTAQDCSADDDNFL |
| Q9UQV4 | KNTATTSPITYTLVTTQATPNNSHTAPPVTEVTVGPSLAPY |
| Q9UQV4 | ITPPAHTTGTSSSTVSHTTGNTTQPSNQTTLPATLSIALHK |
| Q9UQV4 | TTGTSSSTVSHTTGNTTQPSNQTTLPATLSIALHKSTTGQK |
| Q9UQV4 | TTGQKPVQPTHAPGTTAAAHNTTRTAAPASTVPGPTLAPQP |
| Q9UQV4 | PGPTLAPQPSSVKTGIYQVLNGSRLCIKAEMGIQLIVQDKE |
| Q9UQV4 | LIVQDKESVFSPRRYFNIDPNATQASGNCGTRKSNLLLNFQ |
| Q9UQV4 | SGNCGTRKSNLLLNFQGGFVNLTFTKDEESYYISEVGAYLT |
| Q9BMN8 | IKSLATGLSTLRFERVSLDDNATVVSCSADNGVANPVVAEA |
| Q9BMN8 | PPYFSYKLERQYVVGVGGNINLTCVAVGYPMPRVFWKKTDL |
| Q9BMN8 | PSTAPIGKNVLTLTHVESTENFTCVAVSALGNIEATTTVIA |
| Q9BMN8 | QLTWDKPLYSSPVVGYTVRYNTSDGEKELTLTSPHEKHVVT |
| Q9BMN8 | WMGVVYDVLLAAENREGRSQNATETIATPVGSPDGEPIDVQ |
| Q9BMN8 | VMKGKIVVSWRPPSEEKRNGNITSYKAILSAMDATADRYEQ |

| | |
|---|---|
| Q9BMN8 | NPDPAALVGPPTNVRVEATSNSTAVVQWDFESQKADSFVVK |
| Q9BMN8 | VVSDLNAHKPYAFCVLAVKNNLTLNEQFNKVRVTNYMTNFQ |
| Q9BMN8 | SNSVQLTWEYNGPRNVGFYVNHTGRKDYVNHELQEKTMSTP |
| Q9BMN8 | TPATEEYGPISHYWIILVPANYSTEDVVNLDPIELEKATAE |
| Q8BSM7 | ASLLIMLKKEGFYSSLCPAENRTNTTQDEQHQWTSCDQQEK |
| Q8BSM7 | LIMLKKEGFYSSLCPAENRTNTTQDEQHQWTSCDQQEKMLN |
| Q8BSM7 | IMDWRIKDCVDAPTEGTLNENASFGDARDGASTKFTRPRYR |
| P49291 | VSCSAQETMGCADRTAINDFNATLYMGKWYEYAKMGSMPYE |
| P49291 | SMPYEEGGVCVTAEYSMSSNNITVVNSMKDNTTHEVNTTTG |
| P49291 | VTAEYSMSSNNITVVNSMKDNTTHEVNTTTGWAEFASELHT |
| P49291 | MSSNNITVVNSMKDNTTHEVNTTTGWAEFASELHTDGKLSV |
| P49291 | VHFPNSPSVGNYWILSTDYDNYSIVWSCVKRPDSAASTEIS |
| P49291 | VKRPDSAASTEISWILLRSRNSSNMTLERVEDELKNLQLDL |
| P49291 | PDSAASTEISWILLRSRNSSNMTLERVEDELKNLQLDLNKY |
| P18428 | AISDYVFNTASLVYHEEGYLNFSITDDMIPPDSNIRLTTKS |
| P18428 | MNLELQGSVPSAPLLNFSPGNLSVDPYMEIDAFVLLPSSSK |
| P18428 | AFVLLPSSSKEPVFRLSVATNVSATLTFNTSKITGFLKPGK |
| P18428 | SKEPVFRLSVATNVSATLTFNTSKITGFLKPGKVKVELKES |
| Q8K1G2 | KCSLVSGQSESQTMHVQLSVNNTRTPTSVNLSNLLVLDEIT |
| Q8K1G2 | ESQTMHVQLSVNNTRTPTSVNLSNLLVLDEITGLAVKESPG |
| Q8K1G2 | LSNLLVLDEITGLAVKESPGNNTQDGIQTFRKSFLQVGECY |
| Q9UIQ6 | IMVIYLLPRCTFTKEGCHKKNQSIGLIQPFATNGKLFPWAQ |
| Q9UIQ6 | AQIRLPTAVVPLRYELSLHPNLTSMTFRGSVTISVQALQVT |
| Q9UIQ6 | TISVQALQVTWNIILHSTGHNISRVTFMSAVSSQEKQAEIL |
| Q9UIQ6 | EYAYHGQIAIVAPEALLAGHNYTLKIEYSANISSSYYGFYG |
| Q9UIQ6 | VAPEALLAGHNYTLKIEYSANISSSYYGFYGFSYTDESNEK |
| Q9UIQ6 | FSESVKMSTYLVAFIVGEMKNLSQDVNGTLVSIYAVPEKIG |
| Q9UIQ6 | MSTYLVAFIVGEMKNLSQDVNGTLVSIYAVPEKIGQVHYAL |
| Q9UIQ6 | GAMENWGLLTFREETLLYDSNTSSMADRKLVTKIIAHELAH |
| Q9UIQ6 | LSSYEDFLDARFKTMKKDSLNSSHPISSSVQSSEQIEEMFD |
| Q9UIQ6 | MLKTYLSEDVFQHAVVLYLHNHSYASIQSDDLWDSFNEVTN |
| Q9UIQ6 | NHSYASIQSDDLWDSFNEVTNQTLDVKRMMKTWTLQKGFPL |
| Q9UIQ6 | QPSDTSYLWHIPLSYVTEGRNYSKYQSVSLLDKKSGVINLT |
| Q9UIQ6 | GRNYSKYQSVSLLDKKSGVINLTEEVLWVKVNINMNGYYIV |
| Q9UIQ6 | GLGKVPLKRAFDLINYLGNENHTAPITEALFQTDLIYNLLE |
| Q9UIQ6 | PSMRELRSALLEFACTHNLGNCSTTAMKLFDDWMASNGTQS |
| Q9UIQ6 | HNLGNCSTTAMKLFDDWMASNGTQSLPTDVMTTVFKVGAKT |
| Q9UIQ6 | GSTYLFSTKTHLSEVQAFFENQSEATFRLRCVQEALEVIQL |
| Q8C129 | IMVIYLLPRCTFTKEGCHKTNQSAELIQPVATNGKVFPWAQ |
| Q8C129 | AQIRLPTAIIPLCYELSLHPNLTSMTFRGSVTISLQALQDT |
| Q8C129 | TISLQALQDTRDIILHSTGHNISRVTFMSAVSSQEKQVEIL |

| | |
|---|---|
| Q8C129 | EYPYHEQIAVVAPEPLLTGHNYTLKIEYSANISNSYYGFYG |
| Q8C129 | VAPEPLLTGHNYTLKIEYSANISNSYYGFYGITYTDKSNEK |
| Q8C129 | FSESVKMSTYLVAFIVGEMRNLSQDVNGTLVSVYAVPEKIG |
| Q8C129 | MSTYLVAFIVGEMRNLSQDVNGTLVSVYAVPEKIGQVHHAL |
| Q8C129 | AGAMENWGLLTFREETLLYDNATSSVADRKLVTKIIAHELA |
| Q8C129 | LNSYEDFLDARFKTMRKDSLNSSHPISSSVQSSEQIEEMFD |
| Q8C129 | MLKSYLSEDVFRHAVILYLHNHSYAAIQSDDLWDSFNEVTD |
| Q8C129 | QPSDTSHLWHIPISYVTDGRNYSEYRSVSLLDKKSDVINLT |
| Q8C129 | GRNYSEYRSVSLLDKKSDVINLTEQVQWVKVNSNMTGYYIV |
| Q8C129 | DKKSDVINLTEQVQWVKVNSNMTGYYIVHYAHDDWTALINQ |
| Q8C129 | LAGLGKVPLRMAFDLIDYLKNETHTAPITEALFQTNLIYNL |
| Q8C129 | PSMRELRSALLEFACAHSLENCTTMATNLFDSWMASNGTQS |
| Q8C129 | HSLENCTTMATNLFDSWMASNGTQSLPTDVMVTVFKVGART |
| Q8C129 | GSTHLFSTKTHLSEVQAFFENQSEATLKLRCVQEALEVIQL |
| P04180 | PFWLLNVLFPPHTTPKAELSNHTRPVILVPGCLGNQLEAKL |
| P04180 | LNMFLPLGVDCWIDNTRVVYNRSSGLVSNAPGVQIRVPGFG |
| P04180 | MFPSRMAWPEDHVFISTPSFNYTGRDFQRFFADLHFEEGWY |
| P04180 | PQPVHLLPLHGIQHLNMVFSNLTLEHINAILLGAYRQGPPA |
| P16301 | PFWLLNVLFPPHTTPKAELSNHTRPVILVPGCLGNRLEAKL |
| P16301 | FNLFLPLGVDCWIDNTRIVYNHSSGRVSNAPGVQIRVPGFG |
| P16301 | MLPAPHVWPEDHVFISTPNFNYTVQDFERFFTDLHFEEGWH |
| P16301 | TELCGQWQGRQSQPVHLLPMNETDHLNMVFSNKTLEHINAI |
| P16301 | SQPVHLLPMNETDHLNMVFSNKTLEHINAILLGAYRTPKSP |
| P01130 | GRVNRCIPQFWRCDGQVDCDNGSDEQGCPPKTCSQDEFRCH |
| P01130 | DGSDEASCPVLTCGPASFQCNSSTCIPQLWACDNDPDCEDG |
| P01130 | SRQCDREYDCKDMSDEVGCVNVTLCEGPNKFKCHSGECITL |
| P01130 | LGTVSVADTKGVKRKTLFRENGSKPRAIVVDPVHGFMYWTD |
| P01130 | SDVNLLAENLLSPEDMVLFHNLTQPRGVNWCERTTLSNGGC |
| P02873 | LALLSHANSATETSFIIDAFNKTNLILQGDATVSSNGNLQL |
| P02873 | YSAPIQIRDSTTGNVASFDTNFTMNIRTHRQANSAVGLDFV |
| P02873 | SVPWDVHDYDGQNAEVRITYNSSTKVFSVSLSNPSTGKSNN |
| Q41114 | LVLLTHANSASDTSFNFYSFNETNLILQGDATVSSKGYLQL |
| Q41114 | YSAPIQIRDSTTGNVASFDTNFTMNITTQREANSVIGLDFA |
| Q41114 | IQIRDSTTGNVASFDTNFTMNITTQREANSVIGLDFALVPV |
| P23558 | SQIPSGSSAGMFGLFCSSDYNSSNQIIAVEFDTYFGKAYNP |
| P22972 | PDSPLRRAGGYFGLFEDTKDNDSSYQTVAVEFDTIGSPVNF |
| O24313 | RISSNSVLELTKVVNGVPTWNSTGRALYAKPVQVWDSTTGN |
| O24313 | PESVNVGFSAATGDPSGKQRNATETHDILSWSFSASLPGTN |
| P29257 | DTQPQSAGGYLGLFEKDSSYNSSNQIVAVEFDTYYNSAWDP |
| P22971 | APPNSPLRRAGGYLGLFETSNKSDSSYQTVAVEFDTVGAPA |
| P17346 | EERVTQAFAELTTKLSELQENVTNTFHGCNHCPNGWVTSEN |

| | |
|---|---|
| P22973 | ANSQIPSGSSAGMFGLFCSSNDSKSSNQIIAVEFDSYFGKT |
| P19588 | LLLLTQAYSADIQSFSFKNFNSSSFILQGDATVSSSKLRLT |
| P19588 | KSTGAVASWATSFTANIFAPNKSSSADGIAFALVPVGSEPK |
| P18670 | TAIGDFQVVYDLNGSPYVGQNHKSFITGFTPVKISLDFPSE |
| P18670 | VKISLDFPSEYIMEVSGYTGNVSGYVVVRSLTFKTNKKTYG |
| U3KRG0 | PYYILPSTSRAGFSPDNLRKNTSQPSCPLDLITQLRFPRRI |
| U3KRG0 | LITQLRFPRRIGVPVIFTPQNSSLKVVPLSHNLNIHTCSDL |
| Q9AVB0 | GEGCQSQCEQYNWRCGVDFGNRTCPNDLCCSVGGWCGTTDD |
| Q9AVB0 | GEGCQSQCEQYNWHCGVDFGNRTCPNDLCCSEWGWCGITEG |
| Q9YGP1 | SFGLLVVFLSLSGAKGSCCTNDSLPMNGMCYKIFDEPKTWE |
| P02707 | LARIAALSSKLSTLQSEPKHNFSSRDSLLFPCGAQSRQWEY |
| P81371 | FFLAPPDTQPQKDGGFLGLFNDSNKSIQTVAVEFDTFSNTW |
| P81371 | VYISYEASTKTLTASLTYPSNATSYIVSANVDLKSALPEWV |
| P17404 | PTYPKEIQRERRELVRKIVTTTTTRRLRSGPQGTPAPGRPN |
| P17404 | TTTRRLRSGPQGTPAPGRPNNGTRPSVQEDAEPFNPDNPYH |
| P16030 | STSSTLTGFTFPNFWSNTQENGTEIIFLGNATYTPGALRLT |
| P16030 | TFPNFWSNTQENGTEIIFLGNATYTPGALRLTRIGEDGIPL |
| P16030 | DTWPNTEWSDLRYPHIGINVNSTVSVATTRWDNDDAYVTKS |
| P16030 | DLPKILPERVRIGFSGGTGFNETQYILSWSFTSTLNSTKIS |
| P83410 | FFMGPTKSKPAQGYGYLGVFNNSKQDNSYQTLAVEFDTFSN |
| P16404 | KVNSVETISFSFSEFEPGNDNLTLQGAALITQSGVLQLTKI |
| P16404 | FFMGPTKSKPAQGYGYLGIFNNSKQDNSYQTLGVEFDTFSN |
| P02874 | TDTQPKSGGGYLGIFKDAESNETVVAVEFDTFSNRWDPANS |
| P05046 | TPIHIWDKETGSVASFAASFNFTFYAPDTKRLADGLAFFLA |
| P16300 | AANLILQGNAVSSKGHLLLTNVTHNGEPSVASSGRALYSAP |
| P16300 | VASSGRALYSAPIQIRDSTGNASSTPTSHSYTLQQIFQNVT |
| P16300 | TGNASSTPTSHSYTLQQIFQNVTDDPAWLFALVPVDSQPKK |
| P16300 | FALVPVDSQPKKKGRLLGLFNKSENDINALTVAVEFDTCHN |
| P16300 | DSSTKFLAVSLFYPITGKRNNVSANVELEKVLDDWVSVGFS |
| P02871 | ISTKSWNLQNGEEAHVAISFNATTNVLSVTLLYPNLTGYTL |
| Q6P5S2 | LPSWVCVLVGSFSASLAGTSNLSETEPPLWKESPGQLSDYR |
| Q6P5S2 | MYIINPWVYLERMGMYKIILNQTARYFAKFAPDNEQNILWG |
| P48357 | CQKFCVVLLHWEFIYVITAFNLSYPITPWRFKLSCMPPNST |
| P48357 | AFNLSYPITPWRFKLSCMPPNSTYDYFLLPAGLSKNTSNSN |
| P48357 | SCMPPNSTYDYFLLPAGLSKNTSNSNGHYETAVEPKFNSSG |
| P48357 | LSKNTSNSNGHYETAVEPKFNSSGTHFSNLSKTTFHCCFRS |
| P48357 | NGHYETAVEPKFNSSGTHFSNLSKTTFHCCFRSEQDRNCSL |
| P48357 | HFSNLSKTTFHCCFRSEQDRNCSLCADNIEGKTFVSTVNSL |
| P48357 | EVLEDSPLVPQKGSFQMVHCNCSVHECCECLVPVPTAKLND |
| P48357 | CNCSVHECCECLVPVPTAKLNDTLLMCLKITSGGVIFQSPL |
| P48357 | SWSSPPLVPFPLQYQVKYSENSTTVIREADKIVSATSLLVD |

| | |
|---|---|
| P48357 | VFTTQDVIYFPPKILTSVGSNVSFHCIYKKENKIVPSKEIV |
| P48357 | PQSQYDVVSDHVSKVTFFNLNETKPRGKFTYDAVYCCNEHE |
| P48357 | YECIFQPIFLLSGYTMWIRINHSLGSLDSPPTCVLPDSVVK |
| P48357 | CAVYAVQVRCKRLDGLGYWSNWSNPAYTVVMDIKVPMRGPE |
| P48357 | PMRGPEFWRIINGDTMKKEKNVTLLWKPLMKNDSLCSVQRY |
| P48357 | MKNDSLCSVQRYVINHHTSCNGTWSEDVGNHTKFTFLWTEQ |
| P48357 | QRYVINHHTSCNGTWSEDVGNHTKFTFLWTEQAHTVTVLAI |
| P48357 | QAHTVTVLAINSIGASVANFNLTFSWPMSKVNIVQSLSAYP |
| P48357 | TFSWPMSKVNIVQSLSAYPLNSSCVIVSWILSPSDYKLMYF |
| P24348 | GNLEITWIEANEIKKWRESTNSTVDPKNEDSPLKSINFFDN |
| P24348 | YIHKNDKVHEVVMRELRVIRNGSVTIQDNPKMCYIGDKIDW |
| P24348 | DCQRVYRSVCPKSCSQCFYSNSTSSYECCDSACLGGCTGHG |
| P24348 | CEKCRSSSCPKICTVDGHLTNETLKNLEGCEQIDGHLIIEH |
| P24348 | KTWKSVGTCVEKCDTKGFLRNQTSMKCERCSPECETCNGLG |
| P24348 | PDSNLGYGGCKQCKYAVKYENDTIFCLQSSGMNNVCVENDL |
| P24348 | PSERICMDQCPVNSFMVPDTNNTVCKKCHHECDQNYHCANG |
| P24348 | ECDQNYHCANGQSTGCQKCKNFTVFKGDIAQCVSECPKNLP |
| P16150 | SGPQVSSVKLSTMMSPTTSTNASTVPFRNPDENSRGMLPVA |
| P19256 | LHCFGFISCFSQQIYGVVYGNVTFHVPSNVPLKEVLWKKQK |
| P19256 | FSSFKNRVYLDTVSGSLTIYNLTSSDEDEYEMESPNITDTM |
| P19256 | SLTIYNLTSSDEDEYEMESPNITDTMKFFLYVLESLPSPTL |
| P19256 | KFFLYVLESLPSPTLTCALTNGSIEVQCMIPEHYNSHRGLI |
| P19256 | NSHRGLIMYSWDCPMEQCKRNSTSIYFKMENDLPQKIQCTL |
| P19256 | FKMENDLPQKIQCTLSNPLFNTTSSIILTTCIPSSGHSRHR |
| P28175 | DAEKPFVWELMDRSNVVLNDNLTFWASGEPGNETNCVYLDI |
| P28175 | DRSNVVLNDNLTFWASGEPGNETNCVYLDIRDQLQPVWKTK |
| P28175 | RYSCEVLHYLSGTETVTCTTNGTWSAPKPRCIKVITCQNPP |
| P28175 | CESRYYELLGSQGRRCDSNGNWSGRPASCIPVCGRSDSPRS |
| P28175 | SCIPVCGRSDSPRSPFIWNGNSTEIGQWPWQAGISRWLADH |
| P28175 | TREHLKEGTLAVVTGWGLNENNTYSEMIQQAVLPVVAASTC |
| Q08380 | CDNLWDLTDASVVCRALGFENATQALGRAAFGQGSGPIMLD |
| Q08380 | SLGWLKSNCRHERDAGVVCTNETRSTHTLDLSRELSEALGQ |
| Q08380 | HTVILTANLEAQALWKEPGSNVTMSVDAECVPMVRDLLRYF |
| Q08380 | LVEKIRFPMMLPEELFELQFNLSLYWSHEALFQKKTLQALE |
| Q08380 | LQALEFHTVPFQLLARYKGLNLTEDTYKPRIYTSPTWSAFV |
| Q08380 | VADVTDFEGWKAAIPSALDTNSSKSTSSFPCPAGHFNGFRT |
| Q5RDA4 | CDNLWDLTDASVVCRALGFENATQALGRAAFGQGSGPIMLD |
| Q5RDA4 | SLGWLKSNCRHERDAGVVCTNETRSTHTLDLSRELSEALGQ |
| Q5RDA4 | HTVILTANLEAQALWKEPGSNVTMSVDAECVPMVRDLLRYF |
| Q5RDA4 | LVEKIRFPMMLPEELFELQFNLSLYWSHEALFQKKTLQALE |
| Q5RDA4 | LQALEFHTVPFQLLARYKGLNLTEDTYKPRIYTSPTWSAFV |

| | |
|---|---|
| Q5RDA4 | VADVTDFEGWKAAIPSALDTNSSKSTSSFPCPAGHFNGFRT |
| A7E3W2 | CENMWDLTDASVVCRALGFQNATEALGGAAFGPGYGPIMLD |
| A7E3W2 | SLGWMRSNCRHDKDASVICTNETRGVYTLDLSGELPAALEQ |
| A7E3W2 | LVGQVRFPMMPPQDLFSLQFNLSLYWSHEALFQKKILQALE |
| A7E3W2 | LQALEFHTVPFELLAQYWGLNLTEGTYQPRLYTSPTWSQSV |
| Q99538 | YDDIAYSEDNPTPGIVINRPNGTDVYQGVPKDYTGEDVTPQ |
| Q99538 | DHGSTGILVFPNEDLHVKDLNETIHYMYKHKMYRKMVFYIE |
| Q99538 | VEDLTKETLHKQYHLVKSHTNTSHVMQYGNKTISTMKVMQF |
| Q99538 | HKQYHLVKSHTNTSHVMQYGNKTISTMKVMQFQGMKRKASS |
| Q9BXB1 | LTAVPEGLSAFTQALDISMNNITQLPEDAFKNFPFLEELQL |
| Q9BXB1 | LQALTLALNKISSIPDFAFTNLSSLVVLHLHNNKIRSLSQH |
| Q9BXB1 | LRTIHLYDNPLSFVGNSAFHNLSDLHSLVIRGASMVQQFPN |
| Q9BXB1 | NLSDLHSLVIRGASMVQQFPNLTGTVHLESLTLTGTKISSI |
| Q9BXB1 | EDNSLQDHSVAQEKGTADAANVTSTLENEEHSQIIIHCTPS |
| O75473 | DGRMLLRVDCSDLGLSELPSNLSVFTSYLDLSMNNISQLLP |
| O75473 | LSELPSNLSVFTSYLDLSMNNISQLLPNPLPSLRFLEELRL |
| O75473 | LQAMTLALNKIHHIPDYAFGNLSSLVVLHLHNNRIHSLGKK |
| O75473 | CAFGVCENAYKISNQWNKGDNSSMDDLHKKDAGMFQAQDER |
| O75473 | LFTNCILNCPVAFLSFSSLINLTFISPEVIKFILLVVVPLP |
| P38571 | TLHSEGSGGKLTAVDPETNMNVSEIISYWGFPSEEYLVETE |
| P38571 | LVETEDGYILCLNRIPHGRKNHSDKGPKPVVFLQHGLLADS |
| P38571 | VVFLQHGLLADSSNWVTNLANSSLGFILADAGFDVWMGNSR |
| P38571 | AFSYDEMAKYDLPASINFILNKTGQEQVYYVGHSQGTTIGF |
| P38571 | LKELCGNLCFLLCGFNERNLNMSRVDVYTTHSPAGTSVQNM |
| P38571 | KFQKFQAFDWGSSAKNYFHYNQSYPPTYNVKDMLVPTAVWS |
| P42702 | SQKKGAPHDLKCVTNNLQVWNCSWKAPSGTGRGTDYEVCIE |
| P42702 | CSWKAPSGTGRGTDYEVCIENRSRSCYQLEKTSIKIPALSH |
| P42702 | TINSLHDFGSSTSKFTLNEQNVSLIPDTPEILNLSADFSTS |
| P42702 | SKFTLNEQNVSLIPDTPEILNLSADFSTSTLYLKWNDRGSV |
| P42702 | IWEIKVLRKESMELVKLVTHNTTLNGKDTLHHWSWASDMPL |
| P42702 | YIDNLHFSGLEEWSDWSPVKNISWIPDSQTKVFPQDKVILV |
| P42702 | GHTNCPLIHLDGENVAIKIRNISVSASSGTNVVFTTEDNIF |
| P42702 | TLVESFSGKYVRLKRAEAPTNESYQLLFQMLPNQEIYNFTL |
| P42702 | APTNESYQLLFQMLPNQEIYNFTLNAHNPLGRSQSTILVNI |
| P42702 | YNFTLNAHNPLGRSQSTILVNITEKVYPHTPTSFKVKDINS |
| P42702 | VNITEKVYPHTPTSFKVKDINSTAVKLSWHLPGNFAKINFL |
| P42702 | KINFLCEIEIKKSNSVQEQRNVTIKGVENSSYLVALDKLNP |
| P42702 | EIKKSNSVQEQRNVTIKGVENSSYLVALDKLNPYTLYTFRI |
| P42702 | LIIYWKPLPINEANGKILSYNVSCSSDEETQSLSEIPDPQH |
| P42702 | KIEQVVGMGKGILLTWHYDPNMTCDYVIKWCNSSRSEPCLM |
| P42702 | ILLTWHYDPNMTCDYVIKWCNSSRSEPCLMDWRKVPSNSTE |

| | |
|---|---|
| P42702 | KWCNSSRSEPCLMDWRKVPSNSTETVIESDEFRPGIRYNFF |
| P42702 | YQLLRSMIGYIEELAPIVAPNFTVEDTSADSILVKWEDIPV |
| P42702 | ERDTSKMRVLESGRSDIKVKNITDISQKTLRIADLQGKTSY |
| P42703 | FTSSLLLKWNDRGSALPHPSNATWEIKVLQNPRTEPVALVL |
| P42703 | PVALVLLNTMLSGKDTVQHWNWTSDLPLQCATHSVSIRWHI |
| P42703 | HIDSPHFSGYKEWSDWSPLKNISWIRNTETNVFPQDKVVLA |
| P42703 | WIRNTETNVFPQDKVVLAGSNMTICCMSPTKVLSGQIGNTL |
| P42703 | TLFESISGKSAVFHRIEGLTNETYRLGVQMHPGQEIHNFTL |
| P42703 | GLTNETYRLGVQMHPGQEIHNFTLTGRNPLGQAQSAVVINV |
| P42703 | HNFTLTGRNPLGQAQSAVVINVTERVAPHDPTSLKVKDINS |
| P42703 | INVTERVAPHDPTSLKVKDINSTVVTFSWYLPGNFTKINLL |
| P42703 | SLKVKDINSTVVTFSWYLPGNFTKINLLCQIEICKANSKKE |
| P42703 | KINLLCQIEICKANSKKEVRNATIRGAEDSTYHVAVDKLNP |
| P42703 | LIVYWKPLPINEANGKILSYNVSCSLNEETQSVLEIFDPQH |
| P42703 | TVEQAVGLGNRIFLTWRHDPNMTCDYVIKWCNSSRSEPCLL |
| P42703 | IFLTWRHDPNMTCDYVIKWCNSSRSEPCLLDWRKVPSNSTE |
| P42703 | KWCNSSRSEPCLLDWRKVPSNSTETVIESDQFQPGVRYNFY |
| P42703 | YQLLRSIIGYVEELAPIVAPNFTVEDTSADSILVKWDDIPV |
| P42703 | ERDTPKTRSLEPHHSDIKLKNITDISQKTLRIADLQGKTSY |
| P11542 | TDHLFARDSRTACEWQSFVNNQTKLQEDFQFIFTALSTLGH |
| P15018 | LLLVLHWKHGAGSPLPITPVNATCAIRHPCHNNLMNQIRSQ |
| P15018 | IRHPCHNNLMNQIRSQLAQLNGSANALFILYYTAQGEPFPN |
| P15018 | LYYTAQGEPFPNNLDKLCGPNVTDFPPFHANGTEKAKLVEL |
| P15018 | PNNLDKLCGPNVTDFPPFHANGTEKAKLVELYRIVVYLGTS |
| P15018 | EKAKLVELYRIVVYLGTSLGNITRDQKILNPSALSLHSKLN |
| P15018 | NITRDQKILNPSALSLHSKLNATADILRGLLSNVLCRLCSK |
| P09056 | LLLVLHWKHGAGSPLPITPVNATCAIRHPCHGNLMNQIKNQ |
| P09056 | IRHPCHGNLMNQIKNQLAQLNGSANALFISYYTAQGEPFPN |
| P09056 | SYYTAQGEPFPNNVEKLCAPNMTDFPSFHGNGTEKTKLVEL |
| P09056 | PNNVEKLCAPNMTDFPSFHGNGTEKTKLVELYRMVAYLSAS |
| P09056 | EKTKLVELYRMVAYLSASLTNITRDQKVLNPTAVSLQVKLN |
| P09056 | NITRDQKVLNPTAVSLQVKLNATIDVMRGLLSNVLCRLCNK |
| Q96FE5 | LGLRSNRLKLIPLGVFTGLSNLTKLDISENKIVILLDYMFQ |
| Q96FE5 | ISHRAFSGLNSLEQLTLEKCNLTSIPTEALSHLHGLIVLRL |
| Q96FE5 | LEISHWPYLDTMTPNCLYGLNLTSLSITHCNLTAVPYLAVR |
| Q96FE5 | TMTPNCLYGLNLTSLSITHCNLTAVPYLAVRHLVYLRFLNL |
| Q96FE5 | CNLTAVPYLAVRHLVYLRFLNLSYNPISTIEGSMLHELLRL |
| Q96FE5 | GQLAVVEPYAFRGLNYLRVLNVSGNQLTTLEESVFHSVGNL |
| Q96FE5 | GRLTVFPDGTLEVRYAQVQDNGTYLCIAANAGGNDSMPAHL |
| Q96FE5 | RYAQVQDNGTYLCIAANAGGNDSMPAHLHVRSYSPDWPHQP |
| Q96FE5 | DSMPAHLHVRSYSPDWPHQPNKTFAFISNQPGEGEANSTRA |

| | |
|---|---|
| Q96FE5 | PHQPNKTFAFISNQPGEGEANSTRATVPFPFDIKTLIIATT |
| P79066 | PDISYNRFAQYAGCDTSASANDTLECLRSKSSSVLHDAQNS |
| P79066 | VPYISGNQEDEGTAFAPVALNATTTPHVKKWLQYIFYDASE |
| P20261 | PGFLAYSSLRLSYLPRPDGVNITDDMYALVREGKYANIPVI |
| P20261 | IPVIIGDQNDEGTFFGTSSLNVTTDAQAREYFKQSFVHASD |
| Q9XTR8 | LFLLCINSVFGEFGPEDAQYNETEARMLLSLSAAAYSLDVT |
| Q9XTR8 | AAAYSLDVTPCIGRTFSPAENQTLLSTFSVRCDFVGNPCAG |
| Q9XTR8 | CDPVSTNGGYHHAIEIWYPGNMTQGDPFMVCTGLPRDEDFG |
| P32946 | VPVIIGDQNDEGTLFGLSSLNVTTDAQARAYFKQSFIHASD |
| P41365 | PLSTQLGYTPCWISPPPFMLNDTQVNTEYMVNAITALYAGS |
| Q9Y5X9 | EHEGCYLSVGHSQPLEDCSFNMTAKTFFIIHGWTMSGIFEN |
| Q9Y5X9 | ANVVVVDWLPLAHQLYTDAVNNTRVVGHSIARMLDWLQEKD |
| Q9Y5X9 | YGTNADSQTLPLEIVERIEQNATNTFLVYTEEDLGDLLKIQ |
| Q9Y5X9 | IRVKSGETQRKLTFCTEDPENTSISPGRELWFRKCRDGWRM |
| P07098 | LGTTHGLFGKLHPGSPEVTMNISQMITYWGYPNEEYEVVTE |
| P07098 | VVFLQHGLLASATNWISNLPNNSLAFILADAGYDVWLGNSR |
| P07098 | LNLLCSNALFIICGFDSKNFNTSRLDVYLSHNPAGTSVQNM |
| P07098 | DWGSPVQNRMHYDQSQPPYYNVTAMNVPIAVWNGGKDLLAD |
| P11151 | AEDTCHLIPGVTESVANCHFNHSSKTFVVIHGWTVTGMYES |
| P11151 | LYGTVAESENIPFTLPEVSTNKTYSFLLYTEVDIGELLMLK |
| P11602 | DEDVCYLVPGQMDSLAQCNFNHTSKTFVVIHGWTVTGMYES |
| P11602 | AQMPYKVFHYQVKIHFFGKTNVTKVDQPFLISLYGTLDESE |
| P11602 | LYGTLDESENIPFTLPEVSSNKTFSFLIYTEVDIGDLLMLK |
| P06858 | AEDTCHLIPGVAESVATCHFNHSSKTFMVIHGWTVTGMYES |
| P06858 | LYGTVAESENIPFTLPEVSTNKTYSFLIYTEVDIGELLMLK |
| P16233 | VHVIGHSLGAHAAGEAGRRTNGTIGRITGLDPAEPCFQGTP |
| P00591 | VHVIGHSLGSHAAGEAGRRTNGTIERITGLDPAEPCFQGTP |
| P06857 | ANNVRVVGAQVAQMLSMLSANYSYSPSQVQLIGHSLGAHVA |
| P54317 | FKGKTSAVEQTFFLNTGESGNFTSWRYKVSVTLSGKEKVNG |
| P54317 | VDFNVGKIQKVKFLWNKRGINLSEPKLGASQITVQSGEDGT |
| P54318 | FEGKTATVEQTVYLNTGDSGNFTRWRYKVSVTLSGAKKLSG |
| Q8N6C8 | GAYSKPTLSALPSPVVTSGGNVTIQCDSQVAFDGFILCKEG |
| Q8N6C8 | WGRDFLQRPGRQPQAGLSQANFTLGPVSRSYGGQYTCSGAY |
| Q8N6C8 | FTLGPVSRSYGGQYTCSGAYNLSSEWSAPSDPLDILITGQI |
| Q8N6C8 | QIRARPFLSVRPGPTVASGENVTLLCQSQGGMHTFLLTKEG |
| O75023 | GFYAEPTLLALPSPVVASGGNVTLQCDTLDGLLTFVLVEEE |
| O75023 | GEHDLVQGSGQQPQAGLSQANFTLGPVSRSHGGQYRCYGAH |
| O75023 | LIPDIPALSVQPGPKVASGENVTLLCQSWHQIDTFFLTKEG |
| Q9H0V9 | YPNEEKQQERVFPYISAMVNNGSLSYDHERDGRPTELGGCT |
| Q12907 | TYPNDETTERVFPYISVMVNNGSLSYDHSKDGRWTELAGCT |
| P49256 | TYPNDETTERVFPYISVMVNNGSLSYDHSKDGRWTELAGCT |

| | |
|---|---|
| Q7M1X5 | PGFVVTGRVYCDPCRAGFETNVSHNVEGATVAVDCRPFDGG |
| Q9Y4K0 | TEAHISSCKLGPQVSLDPMKNVTCENGLPAVVSCVPGQVFS |
| Q9Y4K0 | LRLNGGRNPYEGRVEVLVERNGSLVWGMVCGQNWGIVEAMV |
| Q9Y4K0 | DCHRHYHSMEVFTHYDLLNLNGTKVAEGHKASFCLEDTECE |
| Q99677 | RRFIDFQFQDSNSSLRPRLGNATANNTCIVDDSFKYNLNGA |
| Q99677 | DFQFQDSNSSLRPRLGNATANNTCIVDDSFKYNLNGAVYSV |
| Q99677 | WILVLSGGISASLFSTTNVNNATTTCFEGFSKRVWKTYLSK |
| A1Z7G7 | TTNRPSPPPWVLSNGPPIFGNGSGLIHPPGVGAGAPPPPRL |
| A1Z7G7 | HHGEDTASPTKPSSKLPAGGNATSPSNTRILTGVGGSGTDD |
| A1Z7G7 | TKSSPNRPPGTAASGSVVPGNGSVVRTINNINLNAAGMSGG |
| A1Z7G7 | GGDDESKLFCGPTHARNLYWNMTRVGDVNVQPCPGGAAGIA |
| A1Z7G7 | EYDDDISSTTPAPSGGDCLHNSSSCEPPVSMAHKVNQRLRN |
| A1Z7G7 | YDHFDLKSSRSYVRNTAILSNDSDVNAGEIQQRLRILNSKV |
| A1Z7G7 | GRHIQLSQPITLTLKHLKTENVTNPTCVFWNYIDHAWSANG |
| A1Z7G7 | VFWNYIDHAWSANGCSLESTNRTHSVCSCNHLTNFAILMDV |
| Q9DAX2 | MIGRLRPSFLAVCDPDWSQVNCSGYVQLEVCRGSPANVTEA |
| Q9DAX2 | WSQVNCSGYVQLEVCRGSPANVTEARLSFYSGHSSFGMYCM |
| Q2I0M4 | SANQLEALAPGTFAPLRALRNLSLAGNRLARLEPAALGALP |
| Q14392 | NVLMDIEDGAFEGLPRLTHLNLSRNSLTCISDFSLQQLRVL |
| Q14392 | RENKLLHFPDLAALPRLIYLNLSNNLIRLPTGPPQDSKGIH |
| Q14392 | KGIHAPSEGWSALPLSAPSGNASGRPLSQLLNLDLSYNEIE |
| Q14392 | NEIELIPDSFLEHLTSLCFLNLSRNCLRTFEARRLGSLPCL |
| Q14392 | RLSHLPAWTQAVSLEVLDLRNNSFSLLPGSAMGGLETSLRR |
| Q07954 | GVQDCMDGSDEGPHCRELQGNCSRLGCQHHCVPTLDGPTCY |
| Q07954 | SRLGCQHHCVPTLDGPTCYCNSSFQLQADGKTCKDFDECSV |
| Q07954 | TNTDGSFICGCVEGYLLQPDNRSCKAKNEPVDRPPVLLIAN |
| Q07954 | VSTITPTSTRQTTAMDFSYANETVCWVHVGDSAAQTQLKCA |
| Q07954 | TQLKCARMPGLKGFVDEHTINISLSLHHVEQMAIDWLTGNF |
| Q07954 | VFFTDYGQIPKVERCDMDGQNRTKLVDSKIVFPHGITLDLV |
| Q07954 | TNSDNANAQQKTSVIRVNRFNSTEYQVVTRVDKGGALHIYH |
| Q07954 | PAGRLYWVDAFYDRIETILLNGTDRKIVYEGPELNHAFGLC |
| Q07954 | IPNRWLCDGDNDCGNSEDESNATCSARTCPPNQFSCASGRC |
| Q07954 | EHWTCDGDNDCGDYSDETHANCTNQATRPPGGCHTDEFQCR |
| Q07954 | NSDEENCESLACRPPSHPCANNTSVCLPPDKLCDGNDDCGD |
| Q07954 | SDEENCESLACRPPSHPCANNTSVCLPPDKLCDGNDDCGDG |
| Q07954 | GSDEGELCDQCSLNNGGCSHNCSVAPGEGIVCSCPLGMELG |
| Q07954 | VAPGEGIVCSCPLGMELGPDNHTCQIQSYCAKHLKCSQKCD |
| Q07954 | VYWTDWRTNTLAKANKWTGHNVTVVQRTNTQPFDLQVYHPS |
| Q07954 | NPCEANGGQGPCSHLCLINYNRTVSCACPHLMKLHKDNTTC |
| Q07954 | INYNRTVSCACPHLMKLHKDNTTCYEFKKFLLYARQMEIRG |
| Q07954 | VDLDAPYYNYIISFTVPDIDNVTVLDYDAREQRVYWSDVRT |

| | |
|---|---|
| Q07954 | REQRVYWSDVRTQAIKRAFINGTGVETVVSADLPNAHGLAV |
| Q07954 | QPHGLVVHPLRGKLYWTDGDNISMANMDGSNRTLLFSGQKG |
| Q07954 | RGKLYWTDGDNISMANMDGSNRTLLFSGQKGPVGLAIDFPE |
| Q07954 | GPVGLAIDFPESKLYWISSGNHTINRCNLDGSGLEVIDAMR |
| Q07954 | VSEKMGTCSKADGSGSVVLRNSTTLVMHMKVYDESIQLDHK |
| Q07954 | DALVPVSGTSLAVGIDFHAENDTIYWVDMGLSTISRAKRDQ |
| Q07954 | IAGNIYWTDQGFDVIEVARLNGSFRYVVISQGLDKPRAITV |
| Q07954 | GQYPRIERSRLDGTERVVLVNVSISWPNGISVDYQDGKLYW |
| Q07954 | DMFSVSVFEDFIYWSDRTHANGSIKRGSKDNATDSVPLRTG |
| Q07954 | FIYWSDRTHANGSIKRGSKDNATDSVPLRTGIGVQLKDIKV |
| Q07954 | NMKLLRVDIPQQPMGIIAVANDTNSCELSPCRINNGGCQDL |
| Q07954 | CRINNGGCQDLCLLTHQGHVNCSCRGGRILQDDLTCRAVNS |
| Q07954 | VNCSCRGGRILQDDLTCRAVNSSCRAQDEFECANGECINFS |
| Q07954 | AVNSSCRAQDEFECANGECINFSLTCDGVPHCKDKSDEKPS |
| Q07954 | NMLWCNGADDCGDGSDEIPCNKTACGVGEFRCRDGTCIGNS |
| Q07954 | CNKTACGVGEFRCRDGTCIGNSSRCNQFVDCEDASDEMNCS |
| Q07954 | IGNSSRCNQFVDCEDASDEMNCSATDCSSYFRLGVKGVLFQ |
| Q07954 | DGDKDCADGADESIAAGCLYNSTCDDREFMCQNRQCIPKHF |
| Q07954 | HDQSDEAPKNPHCTSQEHKCNASSQFLCSSGRCVAEALLCN |
| Q07954 | EPFLIFANRYYLRKLNLDGSNYTLLKQGLNNAVALDFDYRE |
| Q07954 | QMIYWTDVTTQGSMIRRMHLNGSNVQVLHRTGLSNPDGLAV |
| Q07954 | VYWTDWETKSINRAHKTTGTNKTLLISTLHRPMDLHVFHAL |
| Q07954 | HKCACPTNFYLGSDGRTCVSNCTASQFVCKNDKCIPFWWKC |
| Q07954 | PRVWVCDRDNDCVDGSDEPANCTQMTCGVDEFRCKDSGRCI |
| Q07954 | SDEEACGTGVRTCPLDEFQCNNTLCKPLAWKCDGEDDCGDN |
| Q07954 | GDGSDEEDCSIDPKLTSCATNASICGDEARCVRTEKAAYCA |
| Q07954 | QPGCQDINECLRFGTCSQLCNNTKGGHLCSCARNFMKTHNT |
| Q07954 | APPTTSNRHRRQIDRGVTHLNISGLKMPRGIAIDWVAGNVY |
| Q07954 | HNERLYWADAKLSVIGSIRLNGTDPIVAADSKRGLSHPFSI |
| Q07954 | VTYINNRVFKIHKFGHSPLVNLTGGLSHASDVVLYHQHKQP |
| Q07954 | LCLLSPSGPVCTCPNGKRLDNGTCVPVPSPTPPPDAPRPGT |
| Q07954 | CPTGFTGPKCTQQVCAGYCANNSTCTVNQGNQPQCRCLPGF |
| Q07954 | PTGFTGPKCTQQVCAGYCANNSTCTVNQGNQPQCRCLPGFL |
| Q07954 | CSRCLEGACVVNKQSGDVTCNCTDGRVAPSCLTCVGHCSNG |
| Q91ZX7 | GIQDCMDGSDEGAHCRELRANCSRMGCQHHCVPTPSGPTCY |
| Q91ZX7 | SRMGCQHHCVPTPSGPTCYCNSSFQLQADGKTCKDFDECSV |
| Q91ZX7 | TNTDGSFTCGCVEGYLLQPDNRSCKAKNEPVDRPPVLLIAN |
| Q91ZX7 | VSTITPTSTRQTTAMDFSYANETVCWVHVGDSAAQTQLKCA |
| Q91ZX7 | TQLKCARMPGLKGFVDEHTINISLSLHHVEQMAIDWLTGNF |
| Q91ZX7 | VFFTDYGQIPKVERCDMDGQNRTKLVDSKIVFPHGITLDLV |
| Q91ZX7 | TNSDNANTQQKTSVIRVNRFNSTEYQVVTRVDKGGALHIYH |

| | |
|---|---|
| Q91ZX7 | PAGRLYWVDAFYDRIETILLNGTDRKIVYEGPELNHAFGLC |
| Q91ZX7 | IPNRWLCDGDNDCGNSEDESNATCSARTCPPNQFSCASGRC |
| Q91ZX7 | EHWTCDGDNDCGDYSDETHANCTNQATRPPGGCHSDEFQCR |
| Q91ZX7 | NSDEENCEALACRPPSHPCANNTSVCLPPDKLCDGKDDCGD |
| Q91ZX7 | SDEENCEALACRPPSHPCANNTSVCLPPDKLCDGKDDCGDG |
| Q91ZX7 | GSDEGELCDQCSLNNGGCSHNCSVAPGEGIVCSCPLGMELG |
| Q91ZX7 | VAPGEGIVCSCPLGMELGSDNHTCQIQSYCAKHLKCSQKCD |
| Q91ZX7 | VYWTDWRTNTLAKANKWTGHNVTVVQRTNTQPFDLQVYHPS |
| Q91ZX7 | NPCEANGGRGPCSHLCLINYNRTVSCACPHLMKLHKDNTTC |
| Q91ZX7 | INYNRTVSCACPHLMKLHKDNTTCYEFKKFLLYARQMEIRG |
| Q91ZX7 | VDLDAPYYNYIISFTVPDIDNVTVLDYDAREQRVYWSDVRT |
| Q91ZX7 | REQRVYWSDVRTQAIKRAFINGTGVETVVSADLPNAHGLAV |
| Q91ZX7 | QPHGLVVHPLRGKLYWTDGDNISMANMDGSNHTLLFSGQKG |
| Q91ZX7 | RGKLYWTDGDNISMANMDGSNHTLLFSGQKGPVGLAIDFPE |
| Q91ZX7 | GPVGLAIDFPESKLYWISSGNHTINRCNLDGSELEVIDTMR |
| Q91ZX7 | VSEKMGTCNKADGSGSVVLRNSTTLVMHMKVYDESIQLEHE |
| Q91ZX7 | DALVPVSGTSLAVGIDFHAENDTIYWVDMGLSTISRAKRDQ |
| Q91ZX7 | IAGNIYWTDQGFDVIEVARLNGSFRYVVISQGLDKPRAITV |
| Q91ZX7 | GHYPRIERSRLDGTERVVLVNVSISWPNGISVDYQGGKLYW |
| Q91ZX7 | DMFSVSVFEDFIYWSDRTHANGSIKRGCKDNATDSVPLRTG |
| Q91ZX7 | FIYWSDRTHANGSIKRGCKDNATDSVPLRTGIGVQLKDIKV |
| Q91ZX7 | DMKLLRVDIPQQPMGIIAVANDTNSCELSPCRINNGGCQDL |
| Q91ZX7 | CRINNGGCQDLCLLTHQGHVNCSCRGGRILQEDFTCRAVNS |
| Q91ZX7 | VNCSCRGGRILQEDFTCRAVNSSCRAQDEFECANGECISFS |
| Q91ZX7 | NMLWCNGVDDCGDGSDEIPCNKTACGVGEFRCRDGSCIGNS |
| Q91ZX7 | CNKTACGVGEFRCRDGSCIGNSSRCNQFVDCEDASDEMNCS |
| Q91ZX7 | IGNSSRCNQFVDCEDASDEMNCSATDCSSYFRLGVKGVLFQ |
| Q91ZX7 | DGDKDCTDGADESVTAGCLYNSTCDDREFMCQNRLCIPKHF |
| Q91ZX7 | HDHSDEAPKNPHCTSPEHKCNASSQFLCSSGRCVAEALLCN |
| Q91ZX7 | EPFLIFANRYYLRKLNLDGSNYTLLKQGLNNAVALDFDYRE |
| Q91ZX7 | QMIYWTDVTTQGSMIRRMHLNGSNVQVLHRTGLSNPDGLAV |
| Q91ZX7 | VYWTDWETKSINRAHKTTGANKTLLISTLHRPMDLHVFHAL |
| Q91ZX7 | HKCACPTNFYLGGDGRTCVSNCTASQFVCKNDKCIPFWWKC |
| Q91ZX7 | PRVWVCDRDNDCVDGSDEPANCTQMTCGVDEFRCKDSGRCI |
| Q91ZX7 | SDEEACGTGVRTCPLDEFQCNNTLCKPLAWKCDGEDDCGDN |
| Q91ZX7 | GDGSDEEDCSIDPKLTSCATNASMCGDEARCVRTEKAAYCA |
| Q91ZX7 | QPGCQDINECLRFGTCSQLCNNTKGGHLCSCARNFMKTHNT |
| Q91ZX7 | APPTTSNRHRRQIDRGVTHLNISGLKMPRGIAIDWVAGNVY |
| Q91ZX7 | HNERLYWADAKLSVIGSIRLNGTDPIVAADSKRGLSHPFSI |
| Q91ZX7 | VTYINNRVFKIHKFGHSPLINLTGGLSHASDVVLYHQHKQP |
| Q91ZX7 | LCLLSPSGPVCTCPNGKRLDNGTCVPVPSPTPPPDAPRPGT |

| | |
|---|---|
| Q91ZX7 | CPTGFTGPKCTAQVCAGYCSNNSTCTVNQGNQPQCRCLPGF |
| Q91ZX7 | PTGFTGPKCTAQVCAGYCSNNSTCTVNQGNQPQCRCLPGFL |
| Q91ZX7 | CSRCLQGACVVNKQTGDVTCNCTDGRVAPSCLTCIDHCSNG |
| A2ARV4 | ERNCYYPTCDQLTCANGACYNTSQKCDHKVDCRDSSDEANC |
| A2ARV4 | YNTSQKCDHKVDCRDSSDEANCTTLCSQKEFQCGSGECILR |
| A2ARV4 | ISMDKVCDGVPDCPEGEDENNATSGRYCGTGLCSILNCEYQ |
| A2ARV4 | CHQTPYGGECFCPPGHIINSNDSRTCIDFDDCQIWGICDQK |
| A2ARV4 | RHQCLCEEGYILERGQHCKSNDSFSAASIIFSNGRDLLVGD |
| A2ARV4 | ERGQHCKSNDSFSAASIIFSNGRDLLVGDLHGRNFRILAES |
| A2ARV4 | PMQAKVFSTDINGLNTQEILNVSIDAPENLAVDWINNKLYL |
| A2ARV4 | YHQSSLTPFGVTVYHALRQPNATNPCGNNNGGCAQICVLSH |
| A2ARV4 | FRPAKIMRAWSDGSHLMPIVNTSLGWPNGLAIDWSTSRLYW |
| A2ARV4 | RCDGVDDCHDNSDEHQCGALNNTCSSSAFTCVHGGQCIPGQ |
| A2ARV4 | YVCDGDKDCVDGSDEAGCVLNCTSSQFKCADGSSCINSRYR |
| A2ARV4 | TQPFHCPSSQWQCPGYSICVNLSALCDGVFDCPNGTDESPL |
| A2ARV4 | PGYSICVNLSALCDGVFDCPNGTDESPLCNQDSCLHFNGGC |
| A2ARV4 | RCIQGPFGATCVCPIGYQLANDTKTCEDVNECDIPGFCSQH |
| A2ARV4 | VTASENLLLVVASRDKIIMDNITAHTHNIYSLVQDVSFVVA |
| A2ARV4 | VTGRVFWSDLLEGKTWSAFQNGTDKRVVHDSGLSLTEMIAV |
| A2ARV4 | LETIEVSKIDGSHRTVLISKNVTKPRGLALDPRMGDNVMFW |
| A2ARV4 | VFWTDRGTHQVMQANKWHGRNQSVVMYSVPQPLGIIAIHPS |
| A2ARV4 | LCLLSAQEPRHYSCACPSGWNLSDDSVNCVRGDQPFLISVR |
| A2ARV4 | QFIYWVENPGEIHRVKTDGSNRTAFAPLSLLGSSLGLALDW |
| A2ARV4 | YWCDFSSSVRSSNGIRRIKPNGSNFTNIVTYGIGANGIRGV |
| A2ARV4 | DFSSSVRSSNGIRRIKPNGSNFTNIVTYGIGANGIRGVAVD |
| A2ARV4 | GNLYFTNAFVYETLIEVIRINTTYRRVLLKVSVDMPRHIVV |
| A2ARV4 | LFWADYGQKPKIERSFLDCTNRTVLVSEGIVTPRGLAVDHD |
| A2ARV4 | LEDDGKNCATSREDFLIYSLNNSLRSLHFDPQDHNLPFQAI |
| A2ARV4 | VTDGIAFDWINRRIYYSDFSNQTINSMAEDGSNRAVIARVS |
| A2ARV4 | GTNAKIERATLGGNFRVPIVNTSLVWPNGLTLDLETDLLYW |
| A2ARV4 | DFYNDCGDNSDEAGCLFRSCNSTTEFTCSNGRCIPLSYVCN |
| A2ARV4 | SNGRCIPLSYVCNGINNCHDNDTSDEKNCPPITCQPDFAKC |
| A2ARV4 | GDNDCGDMSDEDQRHHCELQNCSSTEFTCINSRPPNRRCIP |
| A2ARV4 | PQHWVCDGDADCADALDELQNCTMRACSTGEFSCANGRCIR |
| A2ARV4 | DEKGCGINECQDSSISHCDHNCTDTITSFYCSCLPGYKLMS |
| A2ARV4 | CRQNSNIEPYLVFSNRYYIRNLTIDGTSYSLILQGLGNVVA |
| A2ARV4 | VEERLYWIDAEKQIIERMFLNKTNQETIISHRLRRAESLAV |
| A2ARV4 | FVSDLEGRQRKMLAQHCVDANNTFCFENPRGIVLHPQRGYV |
| A2ARV4 | VYWADWGDHAYIARIGMDGTNKTVIISTKIEWPNAITIDYT |
| A2ARV4 | NTRTVEKGNKYDGSGRVVLVNTTHKPFDIHVLHPYRQPIMS |
| A2ARV4 | ESDLCPHRFCRLGQFQCRDGNCTSPQALCNARQDCADGSDE |

| | |
|---|---|
| A2ARV4 | CGDYSDEPIHECMTAAYNCDNHTEFSCKTNYRCIPQWAVCN |
| A2ARV4 | DGRADCLDASDESACPTRFPNGTYCPAAMFECKNHVCIQSF |
| A2ARV4 | CDNVDDCGDLSDETGCNLGENRTCAEKICEQNCTQLSNGGF |
| A2ARV4 | DETGCNLGENRTCAEKICEQNCTQLSNGGFICSCRPGFKPS |
| A2ARV4 | AADGSPPLLLLPENVRIRKYNISSEKFSEYLEEEEHIQAID |
| A2ARV4 | KLLVVNPWLTQVRIFHQLRYNQSVSNPCKQVCSHLCLLRPG |
| O75581 | LLLYANRRDLRLVDATNGKENATIVVGGLEDAAAVDFVFSH |
| O75581 | SHGLIYWSDVSEEAIKRTEFNKTESVQNVVVSGLLSPDGLA |
| O75581 | IHSDIFSPMDIHAFSQQRQPNATNPCGIDNGGCSHLCLMSP |
| O75581 | VARNLYWTDTGTDRIEVTRLNGTMRKILISEDLEEPRAIVL |
| O75581 | GEIPKIERAALDGSDRVVLVNTSLGWPNGLALDYDEGKIYW |
| O75581 | TDNRIYWTDISLKTISRAFMNGSALEHVVEFGLDYPEGMAV |
| O75581 | TQYQDYIYWTDWSRRSIERANKTSGQNRTIIQGHLDYVMDI |
| O75581 | IYWTDWSRRSIERANKTSGQNRTIIQGHLDYVMDILVFHSS |
| O75581 | AVPVGGFVCGCPAHYSLNADNRTCSAPTTFLLFSQKSAINR |
| O75581 | LSIDIYSRYIYWTCEATNVINVTRLDGRSVGVVLKGEQDRP |
| Q04833 | QCDTYTWHSVSCIAEYQRCDNITDCADGSDEKDCPASTVDC |
| Q04833 | KCDGKYDCRDLSDEKDSCSRNHTACFQYQFRCADKTQCIQK |
| Q04833 | TCSCLGDCFTLQMEHGPGKDNLTMRGYCVSNNADKMKLFVA |
| Q04833 | MRPAAIYRCHIDGQNCQVIRNTTLGRPSEMAIDFAENRLCW |
| Q04833 | PNELEAKCACRQGFMINKENNHSCQKDPAEKIEQLCSSNST |
| Q04833 | ENNHSCQKDPAEKIEQLCSSNSTQFQCKNGRCIPKEWKCDG |
| Q04833 | KCDSDNDCGDGSDEKLEMCGNATCAANQFSCANGRCIPIYW |
| Q04833 | KCHNSPNGPICSCPFGEQLVNKTKCEPENECLDSSSCSQRC |
| Q04833 | LTDRIYWSDIREKKILSANRNGTNATVFIADGLDITEGIAL |
| Q04833 | RIYWSDIREKKILSANRNGTNATVFIADGLDITEGIALDWV |
| Q04833 | NTIEVANLEDPKQRTLLVHQNVSQPRGIAVDPRKGVMFWTD |
| Q04833 | DMMYYSDRRLQKLQVYPKYPNGTTSEYPSHTFSKALGVVAV |
| Q04833 | KTLAQITTDSAIYRSTVNGGNKTKMFSSAVPDDAYCLGFDW |
| Q04833 | QNMYWTDNRLEKLFRATSKPNQTSLLLSPTTVAASLKDIGD |
| Q04833 | FDVDVNLRRVYFVVESPVGVNISWFSMNNAENPRLVFGASK |
| Q04833 | LCTVVADDIGLAASKVQCSCNDTYELVQEPGKDYPTQCVLR |
| Q04833 | CPSAVACAEGTFPCSNGHCINQTKVCDGHNDCHDEQVSDES |
| Q04833 | ETWQCDGDNDCSDGWDETHTNCTDTAGKKICVGDYLFQCDN |
| Q04833 | CDGEDDCGDGSDEHSRHGCGNRTCTDQEFHCTSNAKLAQPK |
| Q04833 | HSTWECDGDNDCLDGSDEHANCTYSSCQPDFFQCANHKCVP |
| Q04833 | LYWADWNLREVLRCDKWTGKNETILKKTVQLPNDLRIVHPM |
| Q04833 | YTCSCPDQFVLLSDQKTCEPNCTERQFACGGDDAKCIPKLW |
| Q04833 | GESICGQRICPVGEFQCTNHNCTRPFQICDGNDDCGDSSDE |
| Q04833 | DDDCGDRSDEADTLCMSAERNCTAEEFRCNNNKCIAKAWRC |
| Q04833 | FSEYGDCSSDQFKCANGKCVNGTVACDRKDDCGDASDEIGC |

| | |
|---|---|
| Q04833 | GFRPDPQSPKECIDIDECAGNNTCTQLCLNTKGSYLCRCHE |
| Q04833 | LMYWIDGSERTIYRSAIANGNQSHEGQKLDVDFAAMGVVPT |
| P59383 | PTDSLCVGLPAAGVTTLNLANRSLESLPSCLPRTLRSLDGS |
| P59383 | NPLRALLPRTFACFPALRLLNLSCSELGHIAQEAFAGVDGG |
| P59383 | VHTEIFQDTPNLQVLQFQNCNLSSFGPWNSSQVLSVSLFGN |
| P59383 | TPNLQVLQFQNCNLSSFGPWNSSQVLSVSLFGNPLICSCEL |
| P59383 | SLFGNPLICSCELAWLLVDVNKTVLHRAADTMCEPALGSTG |
| P59383 | QGPSIEQSTALSAQPGGSQQNITKVPSLTMTSPTQGSWMYK |
| P59383 | ALPGAASSGAEQTATHILEPNISSASTPLVSKYLEPLPTSP |
| P59383 | APNSVVLWYQIHYVAEGRSGNQSVVDIYATARQHPLYKLTP |
| Q13449 | LCLLPTGLPVRSVDFNRGTDNITVRQGDTAILRCVVEDKNS |
| Q13449 | GDTAILRCVVEDKNSKVAWLNRSGIIFAGHDKWSLDPRVEL |
| Q13449 | QHEPKTSQVYLIVQVPPKISNISSDVTVNEGSNVTLVCMAN |
| Q13449 | VQVPPKISNISSDVTVNEGSNVTLVCMANGRPEPVITWRHL |
| Q13449 | NSANGLEIKSTEGQSSLTVTNVTEEHYGNYTCVAANKLGVT |
| Q13449 | KSTEGQSSLTVTNVTEEHYGNYTCVAANKLGVTNASLVLFR |
| Q13449 | VTEEHYGNYTCVAANKLGVTNASLVLFRPGSVRGINGSISL |
| Q13449 | KLGVTNASLVLFRPGSVRGINGSISLAVPLWLLAASLLCLL |
| P04651 | LGVAGVWASRGPLRPLCQPINATLAAEKEACPVCITFTTSI |
| P08751 | LSVGGVWASRGPLRPLCRPINATLAAEKEACPICITFTTSI |
| P01229 | HPINAILAVEKEGCPVCITVNTTICAGYCPTMMRVLQAVLP |
| P01232 | LSVAGVWASRGPLRPLCRPINATLAAENEACPVCITFTTSI |
| P01231 | LGVAGVWASRGPLRPLCQPINATLAAEKEACPVCITFTTSI |
| P80691 | AIKNVKDNIVFESVDLNVKFNITINCNETTAWKVDRFPGVI |
| P80691 | DNIVFESVDLNVKFNITINCNETTAWKVDRFPGVIGWTVTL |
| Q14766 | EGSFPLRYVQDQVAAPFQLSNHTGRIKVVFTPSICKVTCTK |
| Q14766 | PSICKVTCTKGSCQNSCEKGNTTTLISENGHAADTLTATNF |
| Q14766 | HLPCMNGGQCSSRDKCQCPPNFTGKLCQIPVHGASVPKLYQ |
| Q14766 | KPSYHGYNQMMECLPGYKRVNNTFCQDINECQLQGVCPNGE |
| Q14766 | CINTAGSYDCTCPDGFQLDDNKTCQDINECEHPGLCGPQGE |
| Q14766 | QQGFSISADGRTCEDIDECVNNTVCDSHGFCDNTAGSFRCL |
| Q14766 | KKECYYNLNDASLCDNVLAPNVTKQECCCTSGVGWGDNCEI |
| P51884 | IKYLYLRNNQIDHIDEKAFENVTDLQWLILDHNLLENSKIK |
| P51884 | IKGRVFSKLKQLKKLHINHNNLTESVGPLPKSLEDLQLTHN |
| P51884 | EDLQLTHNKITKLGSFEGLVNLTFIHLQHNRLKEDAVSAAF |
| P51884 | QYLRLSHNELADSGIPGNSFNVSSLVELDLSYNKLKNIPTV |
| P51890 | IKYLYLRNNMIEAIEENTFDNVTDLQWLILDHNHLENSKIK |
| P51890 | IKGRVFSKLKNLKKLHINYNNLTEAVGPLPKTLDDLQLSHN |
| P51890 | LQLSHNKITKVNPGALEGLVNLTVIHLQNNQLKTDSISGAF |
| P51890 | QYLRLSHNKLTDSGIPGNVFNITSLVELDLSFNQLKSIPTV |
| P51890 | CKVVGPLTYSKITHLRLDGNNLTRADLPQEMYNCLRVAADI |

| | |
|---|---|
| Q9Z1Q4 | PEEPCREVFNETNHKLGLNYNTTCCDKDNCNSPAPRPTPAL |
| Q5SQ64 | GRPAPDPGKPGRESRLRLLGNYSLWLEGSKEEDAGRYWCAV |
| Q60767 | LYTAAQYRLALKDGYAVANTNTSDVWKKGGSEENLCAQPYH |
| Q60767 | GLWQSVSCESQQPYVCKKPLNNTLELPDVWTYTDTHCHVGW |
| Q60767 | TDTHCHVGWLPNNGFCYLLANESSSWDAAHLKCKAFGADLI |
| Q60767 | RHGETCYKIYEKEAPFGTNCNLTITSRFEQEFLNYMMKNYD |
| Q60767 | YWFVADPHLNYEEAVLYCASNHSFLATITSFTGLKAIKNKL |
| Q60767 | SFLATITSFTGLKAIKNKLANISGEEQKWWVKTSENPIDRY |
| Q60767 | VDLSKRADCNAKLPFICERYNVSSLEKYSPDPAAKVQCTEK |
| Q60767 | SHFHCALILNLKKSPLTGTWNFTSCSERHSLSLCQKYSETE |
| Q60767 | RHSLSLCQKYSETEDGQPWENTSKTVKYLNNLYKIISKPLT |
| Q60767 | FWKTADCDDNQPGAICYYPGNETEEEVRALDTAKCPSPVQS |
| Q60767 | EQLLYFNYIASWVMLGITYENNSLMWFDKTALSYTHWRTGR |
| Q60767 | FYQHSISACKIEMVDYEDKHNGTLPQFIPYKDGVYSVIQKK |
| Q60767 | VTIADENENKFVSRLMRENYNITMRVWLGLSQHSLDQSWSW |
| Q60767 | SLDQSWSWLDGLDVTFVKWENKTKDGDGKCSILIASNETWR |
| O88188 | QDIRKLFLDITLMAKGSSILNYSYPLCEEDQPKFSFCGRRK |
| Q9Y6Y9 | FFSTLFSSIFTEAQKQYWVCNSSDASISYTYCDKMQYPISI |
| Q9Y6Y9 | ICRGSDDDYSFCRALKGETVNTTISFSFKGIKFSKGKYKCV |
| Q9HBG7 | KDSAPTVVSGILGGSVTLPLNISVDTEIENVIWIGPKNALA |
| Q9HBG7 | IENVIWIGPKNALAFARPKENVTIMVKSYLGRLDITKWSYS |
| Q9HBG7 | VKSYLGRLDITKWSYSLCISNLTLNDAGSYKAQINQRNFEV |
| Q9HBG7 | FVYEQLQEPQVTMKSVKVSENFSCNITLMCSVKGAEKSVLY |
| Q9HBG7 | QLQEPQVTMKSVKVSENFSCNITLMCSVKGAEKSVLYSWTP |
| Q9HBG7 | VTLPLALPACRDTEKVVWLFNTSIISKEREEAATADPLIKS |
| Q9HBG7 | MYTWTPLQKEAVVSQGESHLNVSWRSSENHPNLTCTASNPV |
| Q9HBG7 | VVSQGESHLNVSWRSSENHPNLTCTASNPVSRSSHQFLSEN |
| Q9JHF9 | LFSTLLSPILTESEKQQWFCNSSDAIISYSYCDHLKFPISI |
| Q9JHF9 | LCHGHDDDYSFCRALKGETVNTSIPFSFEGILFPKGHYRCV |
| P10253 | AQMGQPWCFFPPSYPSYKLENLSSSEMGYTATLTRTTPTFF |
| P10253 | FSEEPFGVIVRRQLDGRVLLNTTVAPLFFADQFLQLSTSLP |
| P10253 | GFHLCRWGYSSTAITRQVVENMTRAHFPLDVQWNDLDYMDS |
| P10253 | GPAGSYRPYDEGLRRGVFITNETGQPLIGKVWPGSTAFPDF |
| P10253 | LQFNLLGVPLVGADVCGFLGNTSEELCVRWTQLGAFYPFMR |
| P10253 | GESLEVLERGAYTQVIFLARNNTIVNELVRVTSEGAGLQLQ |
| P10253 | TVLGVATAPQQVLSNGVPVSNFTYSPDTKVLDICVSLLMGE |
| P70699 | PQIGQPWCFFPPSYPSYRLENLSSTESGYTATLTRTSPTFF |
| P70699 | FSEEPFGVIVRRKLGGRVLLNTTVAPLFFADQFLQLSTSLP |
| P70699 | GFHLCRWGYSSTAIVRQVVENMTRTHFPLDVQWNDLDYMDA |
| P70699 | GPAGSYRPYDEGLRRGVFITNETGQPLIGKVWPGTTAFPDF |
| P70699 | GESLAVLERGAYTLVTFSAKNNTIVNKLVRVTKEGAELQLR |

| | |
|---|---|
| P70699 | TVLGVATAPTQVLSNGIPVSNFTYSPDNKSLAIPVSLLMGE |
| P14151 | TYHYSEKPMNWQRARRFCRDNYTDLVAIQNKAEIEYLEKTL |
| P14151 | RSYYWIGIRKIGGIWTWVGTNKSLTEEAENWGDGEPNNKKN |
| P14151 | TASCQPWSCSGHGECVEIINNYTCNCDVGYYGPQCQFVIQC |
| P14151 | HPLGNFSFSSQCAFSCSEGTNLTGIEETTCGPFGNWSSPEP |
| P14151 | SCSEGTNLTGIEETTCGPFGNWSSPEPTCQVIQCEPLSAPD |
| P14151 | EPTCQVIQCEPLSAPDLGIMNCSHPLASFSFTSACTFICSE |
| P14151 | EGTELIGKKKTICESSGIWSNPSPICQKLDKSFSMIKEGDY |
| P16109 | TNQKEVAAWTYHYSTKAYSWNISRKYCQNRYTDLVAIQNKN |
| P16109 | YLNKVLPYYSSYYWIGIRKNNKTWTWVGTKKALTNEAENWA |
| P16109 | TASCQDMSCSKQGECLETIGNYTCSCYPGFYGPECEYVREC |
| P16109 | PECEYVRECGELELPQHVLMNCSHPLGNFSFNSQCSFHCTD |
| P16109 | ECGELELPQHVLMNCSHPLGNFSFNSQCSFHCTDGYQVNGP |
| P16109 | SPVHGSMDCSPSLRAFQYDTNCSFRCAEGFMLRGADIVRCD |
| P16109 | APVCQALQCQDLPVPNEARVNCSHPFGAFRYQSVCSFTCNE |
| P16109 | WNSVPPECQAIPCTPLLSPQNGTMTCVQPLGSSSYKSTCQF |
| P16109 | LTTPGQGTMYCRHHPGTFGFNTTCYFGCNAGFTLIGDSTLS |
| P16109 | TPACRAVKCSELHVNKPIAMNCSNLWGNFSYGSICSFHCLE |
| P16109 | KCSELHVNKPIAMNCSNLWGNFSYGSICSFHCLEGQLLNGS |
| P16109 | WGNFSYGSICSFHCLEGQLLNGSAQTACQENGHWSTTVPTC |
| Q9Y5Y7 | QVSCRIMGITLVSKKANQQLNFTEAKEACRLLGLSLAGKDQ |
| Q9Y5Y7 | GVGVLIWKVPVSRQFAAYCYNSSDTWTNSCIPEIITTKDPI |
| Q8BHC0 | ISTCRIMGVALVGRNKNPQMNFTEANEACKMLGLTLASRDQ |
| Q8BHC0 | GKGVLIWNAPSSQKFKAYCHNSSDTWVNSCIPEIVTTFYPV |
| P45700 | RAQHYLELGAEIARTCHESYNRTYVKLGPEAFRFDGGVEAI |
| Q16706 | LERLLAENNEIISNIRDSVINLSESVEDGPKSSQSNFSQGA |
| Q16706 | RDSVINLSESVEDGPKSSQSNFSQGAGSHLLPSQLSLSVDT |
| Q29451 | TRRFIYVEIAFFSRWWRQQTNATQKIVRELVRQGRLEFANG |
| Q29451 | IRVNVLYSTPACYLWELNKANLSWSVKKDDFFPYADGPYMF |
| Q29451 | SNALAHLSGLKEDFAFCRKLNISICPLTQTAERFQVIVYNP |
| Q29451 | ELENLEQNLLLPVRQAFYWYNASTGNNLSSQASGAYIFRPN |
| Q29451 | QNLLLPVRQAFYWYNASTGNNLSSQASGAYIFRPNQNKPLF |
| Q29451 | VSHWAQTHLVKASLVQEVHQNFSAWCSQVVRLYPRQRHLEL |
| Q29451 | DSNGREILERRRNYRPTWKLNQTEPVAGNYYPVNSRIYITD |
| Q29451 | PETLLLRLEHQFAVGEDSGRNLSSPVTLDLTNLFSAFTITN |
| O00754 | TRRFIYVEIAFFSRWWHQQTNATQEVVRDLVRQGRLEFANG |
| O00754 | KELVDYFLNVATAQGRYYRTNHTVMTMGSDFQYENANMWFK |
| O00754 | SSVHVLYSTPACYLWELNKANLTWSVKHDDFFPYADGPHQF |
| O00754 | SNALARLRGFKDHFTFCQQLNISICPLSQTAARFQVIVYNP |
| O00754 | EIMNMNQQLLLPVRQTFFWYNASIGDNESDQASGAYIFRPN |
| O00754 | QQLLLPVRQTFFWYNASIGDNESDQASGAYIFRPNQQKPLP |

| | |
|---|---|
| O00754 | VSRWAQIHLVKTPLVQEVHQNFSAWCSQVVRLYPGQRHLEL |
| O00754 | DSNGREILERRRDYRPTWKLNQTEPVAGNYYPVNTRIYITD |
| O00754 | MVHRRLLKDDGRGVSEPLMENGSGAWVRGRHLVLLDTAQAA |
| O00754 | PEMVLLRLEHQFAVGEDSGRNLSAPVTLNLRDLFSTFTITR |
| O00754 | KWTTNTGPTPHQTPYQLDPANITLEPMEIRTFLASVQWKEV |
| Q9Y2E5 | IFTHIMDQYSYCTPSHIPFSNRSGFYWNGVAVFPKPPQDGV |
| Q9Y2E5 | GFYWNGVAVFPKPPQDGVYPNMSEPVTPANINLYAEALVAN |
| Q9Y2E5 | AAWFRTPHVLWPWGCDKQFFNASVQFANMDPLLDHINSHAA |
| Q9Y2E5 | LGVSVQYATLGDYFRALHALNVTWRVRDHHDFLPYSTEPFQ |
| Q9Y2E5 | GFPGVRVTDEAGHPVPSQIQNSTETPSAYDLLILTTIPGLS |
| Q9Y2E5 | YIVLLDQDTNLMHSIWERQSNRTVRVTQEFLEYHVNGDVKQ |
| Q9Y2E5 | VEMEIVAGQLVTEIRQYFYRNMTAQNYTYAIRSRLTHVPQG |
| Q9Y2E5 | VAGQLVTEIRQYFYRNMTAQNYTYAIRSRLTHVPQGHDGEL |
| Q9Y2E5 | VIYSDNNGYQMQRRPYVSYVNNSIARNYYPMVQSAFMEDGK |
| Q9Y2E5 | QVEVMLHRRLWNNFDWDLGYNLTLNDTSVVHPVLWLLLGSW |
| Q9Y2E5 | MLHRRLWNNFDWDLGYNLTLNDTSVVHPVLWLLLGSWSLTT |
| Q9Y2E5 | TLPPNLHLQILSIPGWRYSSNHTEHSQNLRKGHRGEAQADL |
| P20916 | QVVHESFQGRSRLLGDLGLRNCTLLLSNVSPELGGKYYFRG |
| P20916 | QGRSRLLGDLGLRNCTLLLSNVSPELGGKYYFRGDLGGYNQ |
| P20916 | HFVPTREANGHRLGCQASFPNTTLQFEGYASMDVKYPPVIV |
| P20916 | LQFEGYASMDVKYPPVIVEMNSSVEAIEGSHVSLLCGADSN |
| P20916 | VTPAEDGVYACLAENAYGQDNRTVGLSVMYAPWKPTVNGTM |
| P20916 | DDGEYWCVAENQYGQRATAFNLSVEFAPVLLLESHCAAARD |
| P20916 | CLCVVKSNPEPSVAFELPSRNVTVNESEREFVYSERSGLVL |
| P20916 | VKSNPEPSVAFELPSRNVTVNESEREFVYSERSGLVLTSIL |
| A2VE13 | FYFGAFLLEAATTSLHDLRCNRTMTVQPLLSDNQYNINVAA |
| Q969L2 | FYFGAFLLEAAATSLHDLHCNTTITGQPLLSDNQYNINVAA |
| Q18788 | NDLRRQKVKEMMIHAWEGYKNYSWGANELRPMSKKPNSQNI |
| Q18788 | TLFIMDLKDKYKEARDYIENNFSMAKSTSTLSVFETTIRFL |
| Q18788 | SLGALGDSFYEYLIKSYVQSNYTDTQAKNMYWDVSDAIQKH |
| Q18788 | MYWDVSDAIQKHMIKVSKQSNLTYTVELNNGQAQHKMGHLA |
| P31723 | LADVLKFAFDTPSGVPYNNINITSHGNDGATTNGLAVTGTL |
| P31723 | DRQDFIDFGLELVDGCEATYNSTLTKIGPDSWGWDPKKVPS |
| P31723 | HRVTGKEIYRDWVWNAFVAINSTCRTDSGFAAVSDVNKANG |
| C0HJB3 | GRVNALYSTPSLYTEAKNAANQTWPLKIDDYFPYADGRNAY |
| C0HJB3 | KQSADQCSAPASAFSQCHLFNISYCPPTESSLPDDKSLVVV |
| C0HJW7 | FDYNVQERVNDFVAAALSQANITRVNALYSTPSIYTDAKYA |
| Q93324 | TTHGYNTLVNLAGNWEFSSSNKTVNGTGTVPGDIYSDLYAS |
| Q93324 | YNTLVNLAGNWEFSSSNKTVNGTGTVPGDIYSDLYASGIID |
| Q93324 | VYVNGQKVLHSRNQFLPYHVNVTDIIALGENDITIKFKSSV |
| Q93324 | VFSGNNENEAAIRGHWWKASNYTESQQVKDYVLLYQRLAKI |

| | |
|---|---|
| Q93324 | CASEYGVQSYPMKETMLNWINESDWEYTSKAMFHRQHHPGG |
| Q93324 | SQVHQSIALKTQTLHYRRFRNTTTNEGLGNTMCAMYWQLND |
| Q93324 | DETDFNLKVFLLNDNPYLLHNITVNVQMLSWGNGLDPILTN |
| Q9UEW3 | LVVQVLNLQARLRVLEMYFLNDTLAAEDSPSFSLLQSAHPG |
| Q9UEW3 | LQAQLTWVRVSHEHLLQRVDNFTQNPGMFRIKGEQGAPGLQ |
| P48740 | GQIQSPGYPDSYPSDSEVTWNITVPDGFRIKLYFMHFNLES |
| P48740 | HNYIGGYYCSCRFGYILHTDNRTCRVECSDNLFTQRTGVIT |
| P48740 | VDCRAPGELEHGLITFSTRNNLTTYKSEIKYSCQEPYYKML |
| P48740 | TTYKSEIKYSCQEPYYKMLNNNTGIYTCSAQGVWMNKVLGR |
| Q9JJS8 | TKVLATLCGQESTDTERAPGNDTFYSLGPSLKVTFHSDYSN |
| Q9JJS8 | PRIETDSNKVTITFTTDESGNHTGWKIHYTSTAQPCPDPTA |
| Q9JJS8 | DRGGKDSCRGDSGGALVFLDNETQRWFVGGIVSWGSINCGG |
| Q91WP0 | TKVLATLCGQESTDTEQAPGNDTFYSLGPSLKVTFHSDYSN |
| Q91WP0 | PRIETDSHKVTITFATDESGNHTGWKIHYTSTARPCPDPTA |
| Q91WP0 | GWKIHYTSTARPCPDPTAPPNGSISPVQAIYVLKDRFSVFC |
| Q91WP0 | HGAGFDNDIALIKLKNKVTINGSIMPVCLPRKEAASLMRTD |
| Q91WP0 | ETGGKDSCRGDSGGALVFLDNETQRWFVGGIVSWGSINCGA |
| Q4ZIN3 | KFELDIEPKVFKPPSSTEALNDSQEFPFPETPTKVWPQDEY |
| Q8CIV2 | KFELDIEPKVFKPQSGADALNDSQDFPFPETPAKVWPQDEY |
| O43462 | QLSFPVRAYKRLDGSTECCNNHSLTDVCFSYRNNFNKRLHT |
| Q9GZU1 | TLLESSSSYKNLTLKFHKLVNVTIHFRLKTINLQSLINNEI |
| P15529 | KCKKGYFYIPPLATHTICDRNHTWLPVSDDACYRETCPYIR |
| P15529 | CYRETCPYIRDPLNGQAVPANGTYEFGYQMHFICNEGYYLI |
| P15529 | MFECDKGFYLDGSDTIVCDSNSTWDPPVPKCLKVLPPSSTK |
| O02839 | MPSDACDEPPKFESMRPQFLNTTYRPGDRVEYECRPGFQPM |
| Q8NFP4 | YAPAQAQIVHAGQACVVKEDNISERVYTIREGDTLMLQCLV |
| Q8NFP4 | VRWTKTAGSASDKFQETSVFNETLRIERIARTQGGRYYCKA |
| Q8NFP4 | QVSVRNVCGIPDKAITFRLTNTTAPPALKLSVNETLVVNPG |
| Q8NFP4 | KAITFRLTNTTAPPALKLSVNETLVVNPGENVTVQCLLTGG |
| Q8NFP4 | TAPPALKLSVNETLVVNPGENVTVQCLLTGGDPLPQLQWSH |
| Q8NFP4 | LAQGGTLSIPSVQARDSGYYNCTATNNVGNPAKKTVNLLVR |
| Q8NFP4 | TNNVGNPAKKTVNLLVRSMKNATFQITPDVIKESENIQLGQ |
| Q8NFP4 | TYLCMASFPGAPVPDLSVEVNISSETVPPTISVPKGRAVVT |
| Q8NFP4 | YSPEFYFDTPNPTRSHKLSKNYSYVLQWTQREPDAVDPVLN |
| Q8NFP4 | FGAGDMASRIIHYTEPINSPNLSDNTCHFEDEKICGYTQDL |
| Q8NFP4 | IETSRPRELGDRARLVSPLYNASAKFYCVSFFYHMYGKHIG |
| Q945K2 | VRGRVLGGTSIINAGVYARANTSIYSASGVDWDMDLVNQTY |
| Q945K2 | ARANTSIYSASGVDWDMDLVNQTYEWVEDTIVYKPNSQSWQ |
| Q945K2 | SLPFTTPPFGFFPSASYPLPNSTFAHFASKVAGPLSYGSLT |
| Q945K2 | TLKSSSNVRVSPNVKFNYYSNLTDLSHCVSGMKKIGELLST |
| P61870 | PLLSMGYVHVSPEYWITSPNNATVSTSDIKVIDGDVSFDGN |

| | |
|---|---|
| Q00748 | FGTSSNVHALPLFGGGKTLTNASREENNEALYDDSISYGIL |
| Q7Z7M0 | CKGQRQVLREAPGFVTDGAGNYSVNGNCEWLIEAPSPQHRI |
| Q7Z7M0 | FLGRACDLHLWENQGAGWWHNVSARDPAFSARIGAAGAFLS |
| Q7Z7M0 | DNPTLGRCLQGDFSGPLGGGNCSLWVGEGLGLPVALPARWA |
| Q7Z7M0 | GDPRAGGSCFRECGGRALLTNVSSVALGSRRVGGLLPPGGG |
| Q7Z7M0 | PMPVESSPPLPCPTPCHLLPNCTSCLDSKGADGGWQHCVWS |
| Q7Z7M0 | GYTMDNMTGLCRPVCAQGCVNGSCVEPDHCRCHFGFVGRNC |
| P04824 | LVADEQKFPNGMGHVADHLHNNSFLFGMYSSAGEYTCAGYP |
| P04824 | GQFGTPEISYHRYKAMSDALNKTGRPIFYSLCNWGQDLTFY |
| P04824 | GQNAGVGGWNDLDNLEVGVGNLTDDEEKAHFSMWAMVKSPL |
| P04824 | LDNGDQVVALLNGGSVSRPMNTTLEEIFFDSNLGSKKLTST |
| P04824 | GSKKLTSTWDIYDLWANRVDNSTASAILGRNKTATGILYNA |
| P04824 | IYDLWANRVDNSTASAILGRNKTATGILYNATEQSYKDGLS |
| P04824 | DNSTASAILGRNKTATGILYNATEQSYKDGLSKNDTRLFGQ |
| P04824 | TATGILYNATEQSYKDGLSKNDTRLFGQKIGSLSPNAILNT |
| P28825 | CLLSLIFSAHIAAVSIKHLLNGSDHDTDVGEQKDIFEINLA |
| P28825 | IIFQKLSGCWSMIGDQQVGQNISIGEGCDFKATIEHEILHA |
| P28825 | LNTPYDYESLMHYGPFSFNKNESIPTITTKIPEFNTIIGQL |
| P28825 | IIGQLPDFSAIDLIRLNRMYNCTATHTLLDHCDFEKTNVCG |
| P28825 | EQVDHTLVGQCKGAGYFMFFNTSLGARGEAALLESRILYPK |
| P28825 | TLNEEKKFRYVFLGTKGDPGNSSGGIYLDDITLTETPCPAG |
| P28825 | YLDDITLTETPCPAGVWTIRNISQILENTVKGDKLVSPRFY |
| P28825 | DQEADTRNRMSLTLMFTTSKNQTSSAINGSVIWDRPSKVGV |
| P28825 | NRMSLTLMFTTSKNQTSSAINGSVIWDRPSKVGVYDKDCDC |
| Q16820 | SLNVPYDYTSVMHYSKTAFQNGTEPTIVTRISDFEDVIGQR |
| Q16820 | VIGQRMDFSDSDLLKLNQLYNCSSSLSFMDSCSFELENVCG |
| Q16820 | SESDQLNIYIREYSADNVDGNLTLVEEIKEIPTGSWQLYHV |
| Q16820 | VFEGRKGSGASLGGLSIDDINLSETRCPHHIWHIRNFTQFI |
| Q16820 | SIDDINLSETRCPHHIWHIRNFTQFIGSPNGTLYSPPFYSS |
| Q16820 | TRCPHHIWHIRNFTQFIGSPNGTLYSPPFYSSKGYAFQIYL |
| Q16820 | DNGNYFWDRPSKVGTVALFSNGTQFRRGGGYGTSAFITHER |
| Q16820 | DFIKGDDVYILLTVEDISHLNSTQIQLTPAPSVQDLCSKTT |
| Q12866 | LAFKHTVGHIILSEHKGVKFNCSISVPNIYQDTTISWWKDG |
| Q12866 | DDEVTAIIASFSITSVQRSDNGSYICKMKINNEEIVSDPIY |
| Q12866 | DPIYIEVQGLPHFTKQPESMNVTRNTAFNLTCQAVGPPEPV |
| Q12866 | GLPHFTKQPESMNVTRNTAFNLTCQAVGPPEPVNIFWVQNS |
| Q12866 | FNLTCQAVGPPEPVNIFWVQNSSRVNEQPEKSPSVLTVPGL |
| Q12866 | KGVQINIKAIPSPPTEVSIRNSTAHSILISWVPGFDGYSPF |
| Q12866 | TAHSILISWVPGFDGYSPFRNCSIQVKEADPLSNGSVMIFN |
| Q12866 | DGYSPFRNCSIQVKEADPLSNGSVMIFNTSALPHLYQIKQL |
| Q12866 | NCSIQVKEADPLSNGSVMIFNTSALPHLYQIKQLQALANYS |

| | |
|---|---|
| Q12866 | IFNTSALPHLYQIKQLQALANYSIGVSCMNEIGWSAVSPWI |
| Q12866 | AVSPWILASTTEGAPSVAPLNVTVFLNESSDNVDIRWMKPP |
| Q12866 | LASTTEGAPSVAPLNVTVFLNESSDNVDIRWMKPPTKQQDG |
| Q12866 | ISHVWQSAGISKELLEEVGQNGSRARISVQVHNATCTVRIA |
| Q12866 | ELLEEVGQNGSRARISVQVHNATCTVRIAAVTRGGVGPFSD |
| Q0KHY3 | KLFVVCALACGFMAFVLANENISTEEFEDQIINAVPEPVKV |
| Q0KHY3 | SMTQLHKNLNFQLPFYGFRFNYTRLSLNGYLEFSDPPEYLT |
| Q0KHY3 | QGVVGADTFIPKHVVIATWKNVSFAGGIDNSLYTTNTFQMV |
| Q0KHY3 | GGDTTKGEGGVPAYVGFNAGNGTQAYEYNPYSQNMVIRDLA |
| Q0KHY3 | ALLPLTFAPESGNMLGGQVVNITGPCFDPAIRVTCHFDTES |
| Q0KHY3 | TTDDVHKKNPAEIRITWNQYNLTSNANANVMISLWGYRETK |
| Q0KHY3 | SNYINRNNINRDMQFGFLQINLTQPDQYSGLAISPVLWSRP |
| Q0KHY3 | PVNYYGEVKATQLTAVAMRGNTTTTIEVRLRPLHARWRYRL |
| Q0KHY3 | ESLKFQHFDGVTVYTPTYLLNQSQVVVQFDAGIGVEVVENE |
| Q0KHY3 | EVVENEGYMTGRVFLPWKFINKTAGLFGNWSFNKLDDFMLP |
| Q0KHY3 | MTGRVFLPWKFINKTAGLFGNWSFNKLDDFMLPNGQVAQLN |
| Q0KHY3 | PGVGAALFKREFGRMSGYYANATFQPNYVLDPADFLPANRS |
| Q0KHY3 | YANATFQPNYVLDPADFLPANRSYDLERAEELCGECMQCQY |
| P08581 | ECKEALAKSEMNVNMKYQLPNFTAETPIQNVILHEHHIFLG |
| P08581 | TGPVLEHPDCFPCQDCSSKANLSGGVWKDNINMALVVDTYY |
| P08581 | QLISCGSVNRGTCQRHVFPHNHTADIQSEVHCIFSPQIEEP |
| P08581 | AKVLSSVKDRFINFFVGNTINSSYFPDHPLHSISVRRLKET |
| P08581 | NKNNVRCLQHFYGPNHEHCFNRTLLRNSSGCEARRDEYRTE |
| P08581 | CLQHFYGPNHEHCFNRTLLRNSSGCEARRDEYRTEFTTALQ |
| P08581 | DFGFRRNNKFDLKKTRVLLGNESCTLTLSESTMNTLKCTVG |
| P08581 | SESTMNTLKCTVGPAMNKHFNMSIIISNGHGTTQYSTFSYV |
| P08581 | KNLNSVSVPRMVINVHEAGRNFTVACQHRSNSEIICCTTPS |
| P08581 | EIKGNDIDPEAVKGEVLKVGNKSCENIHLHSEAVLCTVPND |
| P08581 | EWKQAISSTVLGKVIVQPDQNFTGLIAGVVSISTALLLLLG |
| Q13361 | LAVLADIAPSTDDLASLSEKNTTAECWDEKFTCTRLYSVHR |
| Q9D1H9 | PVFCDMTTEGGKWTVFQKRFNGSVSFFRGWSDYKLGFGRAD |
| Q9D1H9 | LHLLTLKQKYELRVDLEDFENNTAYAKYIDFSISPNAISAE |
| P55083 | PVFCDMTTEGGKWTVFQKRFNGSVSFFRGWNDYKLGFGRAD |
| P55083 | MHLLTLKQKYELRVDLEDFENNTAYAKYADFSISPNAVSAE |
| Q95114 | EDRTPPFYCLCPEGFTGLLCNETEHGPCFPNPCHNDAECQV |
| Q95114 | QFQFIQVAGRSGDKIFIGNVNNSGLKINLFDTPLETQYVRL |
| Q08431 | YPTSCHTACTLRFELLGCELNGCANPLGLKNNSIPDKQITA |
| Q08431 | LRFELLGCELNGCANPLGLKNNSIPDKQITASSSYKTWGLH |
| Q08431 | QGARNFGSVQFVASYKVAYSNDSANWTEYQDPRTGSSKIFP |
| Q08431 | NFGSVQFVASYKVAYSNDSANWTEYQDPRTGSSKIFPGNWD |
| Q08431 | WTEYQDPRTGSSKIFPGNWDNHSHKKNLFETPILARYVRIL |

| | |
|---|---|
| Q8NHS3 | FFILLPWGNQFPKIQWEDLHNNSIPNTTFGEIIIGLWKSPM |
| Q8NHS3 | PWGNQFPKIQWEDLHNNSIPNTTFGEIIIGLWKSPMEDDNE |
| Q9XGM8 | KLNDVTVDWKAKDLGYLTEGNYTKYFSGLVRQARPIQGSDL |
| O43451 | RGCCWNPQGAVSVPWCYYSKNHSYHVEGNLVNTNAGFTARL |
| O43451 | RHDMNWKTWPIFNRDTTPNGNGTNLYGAQTFFLCLEDASGL |
| O43451 | VNELHNNGQKLVIIVDPAISNNSSSSKPYGPYDRGSDMKIW |
| O43451 | NELHNNGQKLVIIVDPAISNNSSSSKPYGPYDRGSDMKIWV |
| O43451 | SSSSKPYGPYDRGSDMKIWVNSSDGVTPLIGEVWPGQTVFP |
| O43451 | NGQGYKDQDPASFGADSLLLNSSRHYLNIRYTLLPYLYTLF |
| O43451 | RAHSRGDTVARPLLHEFYEDNSTWDVHQQFLWGPGLLITPV |
| O43451 | PGDKIGLHLRGGYIFPTQQPNTTTLASRKNPLGLIIALDEN |
| O43451 | VANKVYLLCEFSVTQNRLEVNISQSTYKDPNNLAFNEIKIL |
| O43451 | KDPNNLAFNEIKILGTEEPSNVTVKHNGVPSQTSPTVTYDS |
| O43451 | KIRDEEKIDCYPDENGASAENCTARGCIWEASNSSGVPFCY |
| O43451 | DENGASAENCTARGCIWEASNSSGVPFCYFVNDLYSVSDVQ |
| O43451 | RPVMVPYWSLGFQLCRYGYQNDSEIASLYDEMVAAQIPYDV |
| O43451 | NRMKADGMRVILILDPAISGNETQPYPAFTRGVEDDVFIKY |
| O43451 | PNDGDIVWGKVWPDFPDVVVNGSLDWDSQVELYRAYVAFPD |
| O43451 | DWDSQVELYRAYVAFPDFFRNSTAKWWKREIEELYNNPQNP |
| O43451 | NHNTIGTRRQDPVSWDVAFVNISRTVLQTRYTLLPYLYTLM |
| O43451 | SQFLLGPAFLVSPVLERNARNVTAYFPRARWYDYYTGVDIN |
| O77836 | QRSSELSAIVQQFKRVEAETNRSKDPVNKFSDDTLKILKEL |
| O77836 | NVESYLFHSGNQDHPGDILLNTTVEVLPLKSEGLDISKETK |
| Q5JRA6 | SFESFEEMLQDKLKVPESENNKTSNSSQVSNEQDKIDAYKL |
| Q5JRA6 | DRKIQQESLGSAPLMGDDHPNASRDSVEGDALVNGAKLHTL |
| Q20930 | SFVKRKVQDGLKFSRVIKYTNETIQGMKTNFNSNKTQELSL |
| Q20930 | SRVIKYTNETIQGMKTNFNSNKTQELSLDVLVVADFLSYQA |
| Q20930 | EYLHALFEQTKIIYDGISFGNETLHMVFAGTWIATQERDCP |
| Q20930 | EEERVLNEEIRRLEEKERDLNSTFVDDTFFMNSTDSDNSST |
| Q20930 | RLEEKERDLNSTFVDDTFFMNSTDSDNSSTDALISSDMPKK |
| Q20930 | RDLNSTFVDDTFFMNSTDSDNSSTDALISSDMPKKLRKFVD |
| Q20930 | SDMPKKLRKFVDITLEEMQENNSTEMTLKIDSKKAIDKFTI |
| Q20930 | DMPKKLRKFVDITLEEMQENNSTEMTLKIDSKKAIDKFTIW |
| Q20930 | AVAVSGSADRQSFLNSRRMSNCSINSIIENLKEPTANCVKK |
| P20774 | AKYNKIKSRGIKANAFKKLNNLTFLYLDHNALESVPLNLPE |
| P20774 | VIHLQFNNIASITDDTFCKANDTSYIRDRIEEIRLEGNPIV |
| P19879 | LESVPLNLPESLRVIHLQFNNITSITDDTFCKANDTSYIRD |
| P19879 | VIHLQFNNITSITDDTFCKANDTSYIRDRIEEIRLEGNPVI |
| Q9UNW1 | NDKLMRFFDHCEKFLTEVEKNATALYHVEAFKTGPEMQNIL |
| P13087 | YIVPVLRDHGGGLTVSATTPNGTFVCPPRVVQTRKEVDHDR |
| P81446 | APRGAETHLFTGTTRSSLPFNGSYPDLERYAGHRDQIPLGI |

| | |
|---|---|
| P81446 | KSNNDPNQLWTIKRDGTIRSNGSCLTTYGYTAGVYVMIFDC |
| P81446 | VMIFDCNTAVREATLWEIWGNGTIINPRSNLVLAASSGIKG |
| P81446 | GTTLTVQTLDYTLGQGWLAGNDTAPREVTIYGFRDLCMESN |
| Q6ITZ3 | FLSGPGTHLFTGTTRSSLPFNGSYPDLEQYAGHRKQIPLGI |
| Q6ITZ3 | KSNNDPNQLWTIKRDGTIRSNGSCLTTYGYTAGVYVMIFDC |
| Q6ITZ3 | VMIFDCNTAVREATIWQIWGNGTIINPRSNLALAASSGIKG |
| Q6ITZ3 | GTTLTVQTLDYTLGQGWLAGNDTAPREVTIYGFNDLCMESN |
| P82683 | VMIFDCNTAVREATLWQIWGNGTIINPRSNLVLGAASGSSG |
| P82683 | GTTLTVQTQVYSLGQGWLAGNDTAPREVTIYGFRDLCMEAN |
| Q23490 | FNRHFPAQYDDGKGEPISSLNQSRPSAREANRVMLSSAQSV |
| Q23490 | FRFGHGMIEEFYKRVDLSGNNITHGGFFFGDGVFKSGKILF |
| Q23490 | DDFADMILDRNLRAGLARNYNTTNDVDFYVGSMLEDPVIGG |
| P45452 | GVPDVGEYNVFPRTLKWSKMNLTYRIVNYTPDMTHSEVEKA |
| P45452 | SEVEKAFKKAFKVWSDVTPLNFTRLHDGIADIMISFGIKEH |
| P03956 | FVLTEGNPRWEQTHLTYRIENYTPDLPRADVDHAIEKAFQL |
| Q9H306 | AYLNQFYSLEIEGNHLVQSKNRSLIDDKIREMQAFFGLTVT |
| Q9H306 | RCGVPDVGQYGYTLPGWRKYNLTYRIINYTPDMARAAVDEA |
| Q9H306 | KGFFFFSRGSKQFEYDIKTKNITRIMRTNTWFQCKEPKNSS |
| P22894 | QDYLEKFYQLPSNQYQSTRKNGTNVIVEKLKEMQRFFGLNV |
| P22894 | KNGTNVIVEKLKEMQRFFGLNVTGKPNEETLDMMKKPRCGV |
| P22894 | GVPDSGGFMLTPGNPKWERTNLTYRIRNYTPQLSEAEVERA |
| P22894 | FQPGQGIGGDAHFDAEETWTNTSANYNLFLVAAHEFGHSLG |
| P22894 | AHSSDPGALMYPNYAFRETSNYSLPQDDIDGIQAIYGLSSN |
| Q9H8L6 | QNDVHRVADSLPGLWKALPGNLTAAVMEANQTGHEFPDRSL |
| Q9H8L6 | SLPGLWKALPGNLTAAVMEANQTGHEFPDRSLEQVLLPHVD |
| Q9H8L6 | LLPHVDTFLQVHFSPIWRSFNQSLHSLTQAIRNLSLDVEAN |
| Q9H8L6 | FSPIWRSFNQSLHSLTQAIRNLSLDVEANRQAISRVQDSAV |
| Q9H8L6 | QADVDTKLKRLHKAQEAPGTNGSLVLATPGAGARPEPDSLQ |
| Q9H8L6 | GAGARPEPDSLQARLGQLQRNLSELHMTTARREEELQYTLE |
| Q9H8L6 | SDETFDQISKVERQVEELQVNHTALRELRVILMEKSLIMEE |
| Q9H8L6 | EKSLIMEENKEEVERQLLELNLTLQHLQGGHADLIKYVKDC |
| Q9H8L6 | DVKNVGRCCEAEAGAGAASLNASLHGLHNALFATQRSLEQH |
| Q9H8L6 | EQHQRLFHSLFGNFQGLMEANVSLDLGKLQTMLSRKGKKQQ |
| Q9H8L6 | PVAFYASFSEGTAALQTVKFNTTYINIGSSYFPEHGYFRAP |
| Q13201 | SLLKSTLPPSETSAPAEGVRNQTLTSTEKAEGVVKLQNLTL |
| Q13201 | GVRNQTLTSTEKAEGVVKLQNLTLPTNASIKFNPGAESVVL |
| Q13201 | LTSTEKAEGVVKLQNLTLPTNASIKFNPGAESVVLSNSTLK |
| Q13201 | TLPTNASIKFNPGAESVVLSNSTLKFLQSFARKSNEQATSL |
| Q13201 | MTDQVNYQAMKLTLLQKKIDNISLTVNDVRNTYSSLEGKVS |
| Q13201 | KTVSSLSEDLESTRQIIQKVNESVVSIAAQQKFVLVQENRP |
| Q13201 | HLEGALEQEHSRSILYYESLNKTLSKLKEVHEQLLSTEQVS |

| | |
|---|---|
| Q13201 | LSTEQVSDQKNAPAAESVSNNVTEYMSTLHENIKKQSLMML |
| Q13201 | QSLMMLQMFEDLHIQESKINNLTVSLEMEKESLRGECEDML |
| Q13201 | KCRNDFKFQLKDTEENLHVLNQTLAEVLFPMDNKMDKMSEQ |
| Q13201 | RQTMTYEQPKEAIVIRKKIENLTSAVNSLNFIIKELTKRHN |
| Q13201 | RDDALERRINEYALEMEDGLNKTMTIINNAIDFIQDNYALK |
| Q13201 | HKCTSDMETILTFIPQFHRLNDSIQTLVNDNQRYNFVLQVA |
| Q13201 | YNFVLQVAKTLAGIPRDEKLNQSNFQKMYQMFNETTSQVRK |
| Q13201 | GIPRDEKLNQSNFQKMYQMFNETTSQVRKYQQNMSHLEEKL |
| Q13201 | FQKMYQMFNETTSQVRKYQQNMSHLEEKLLLTTKISKNFET |
| Q13201 | SRFKALEAKSIHLSINFFSLNKTLHEVLTMCHNASTSVSEL |
| Q13201 | LSINFFSLNKTLHEVLTMCHNASTSVSELNATIPKWIKHSL |
| Q13201 | KTLHEVLTMCHNASTSVSELNATIPKWIKHSLPDIQLLQKG |
| Q13201 | KGLTEFVEPIIQIKTQAALSNLTCCIDRSLPGSLANVVKSQ |
| Q13201 | SQKQVKSLPKKINALKKPTVNLTTVLIGRTQRNTDNIIYPE |
| Q13201 | TCINGRTSFTCACRHPFTGDNCTIKLVEENALAPDFSKGSY |
| P46557 | VITFLTLFNKNGDGPDMDWQNATEVPLTTTSKATVSTTTTQ |
| P46557 | PSQSPLTSDPSKFQPIHYVLNITIRDVRKPVLEGHMQLFAS |
| P46557 | NLCLNHGPSMSAIANSLINPNVSTSGISCFEKTVPLIAQQL |
| P46557 | PLIAQQLSFVAFEKTNPLFYNTTTMDGAYLPEIDMIFNLNA |
| P46557 | LQIVKKTRKMEVSKIRPRNLNETADSVRVLRQIKSQSSNLC |
| Q9ES58 | HVAVLLIWGVFAAESSCPDKNQTMQNNSSTMTEVNTTVFVQ |
| Q9ES58 | LIWGVFAAESSCPDKNQTMQNNSSTMTEVNTTVFVQMGKKA |
| Q9ES58 | SSCPDKNQTMQNNSSTMTEVNTTVFVQMGKKALLCCPSISL |
| Q9ES58 | RGQPSCIISYKADTRETHESNCSDRSITWASTPDLAPDLQI |
| Q9ES58 | QNIYDLQVLVPPEVTHFPGENRTAVCEAIAGKPAAQISWTP |
| Q9ES58 | IAGKPAAQISWTPDGDCVAKNESHSNGTVTVRSTCHWEQSH |
| Q9ES58 | AAQISWTPDGDCVAKNESHSNGTVTVRSTCHWEQSHVSVVF |
| Q9ES58 | HWEQSHVSVVFCVVSHLTTGNQSLSIELGRGGDQLLGSYIQ |
| Q9ES57 | ALAVLLIWGVFVAGSSCTDKNQTTQNNSSSPLTQVNTTVSV |
| Q9ES57 | LIWGVFVAGSSCTDKNQTTQNNSSSPLTQVNTTVSVQIGTK |
| Q9ES57 | IWGVFVAGSSCTDKNQTTQNNSSSPLTQVNTTVSVQIGTKA |
| Q9ES57 | SCTDKNQTTQNNSSSPLTQVNTTVSVQIGTKALLCCFSIPL |
| Q9ES57 | IIKLRGLPSCTIAYKVDTKTNETSCLGRNITWASTPDHSPE |
| Q9ES57 | SCTIAYKVDTKTNETSCLGRNITWASTPDHSPELQISAVTL |
| Q9ES57 | EKNYDLQVLVPPEVTYFPEKNRSAVCEAMAGKPAAQISWSP |
| Q9ES57 | AAQISWSPDGDCVTTSESHSNGTVTVRSTCHWEQNNVSDVS |
| Q9ES57 | SESHSNGTVTVRSTCHWEQNNVSDVSCIVSHLTGNQSLSIE |
| Q9ES57 | CHWEQNNVSDVSCIVSHLTGNQSLSIELSRGGNQSLRPYIP |
| Q9ES57 | CIVSHLTGNQSLSIELSRGGNQSLRPYIPYIIPSIIILIII |
| Q19426 | DDRLPGYYWYEADGRHFGKQNISEAHKGVIQTDWINDANGF |
| Q19426 | RIRSYNFNKKYENVFQLAGKNYTKTQLKMAKVSLSNMLGSV |

| | |
|---|---|
| Q19426 | HWIRITQSGPTRTTYRWRGRNETIKTELNPKTLSSGLDDFP |
| Q13724 | AESLDELHWAPELGVFADFGNHTKAVQLKPRPPQGLVRVVG |
| O88941 | AGSLDELHWAPELGVFADFGNHTKAVQLKSRPPQGLVRVVG |
| P00304 | RAYALWSARQQFKTTDVLWFNFTTGEDSVAEVWREEAYHAC |
| P43213 | VLFAVFLGSAYGIPKVPPGPNITATYGDKWLDAKSTWYGKP |
| P20645 | EKGKESEKELALVKRLKPLFNKSFESTVGQGSDTYIYIFRV |
| P20645 | TVGQGSDTYIYIFRVCREAGNHTSGAGLVQINKSNGKETVV |
| P20645 | IFRVCREAGNHTSGAGLVQINKSNGKETVVGRLNETHIFNG |
| P20645 | GAGLVQINKSNGKETVVGRLNETHIFNGSNWIMLIYKGGDE |
| P20645 | INKSNGKETVVGRLNETHIFNGSNWIMLIYKGGDEYDNHCG |
| P24668 | EKDKESKNEVALLERLRPLFNKSFESTVGQGSDTYSYIFRV |
| P24668 | TVGQGSDTYSYIFRVCREASNHSSGAGLVQINKSNDKETVV |
| P24668 | IFRVCREASNHSSGAGLVQINKSNDKETVVGRINETHIFNG |
| P24668 | GAGLVQINKSNDKETVVGRINETHIFNGSNWIMLIYKGGDE |
| P24668 | INKSNDKETVVGRINETHIFNGSNWIMLIYKGGDEYDNHCG |
| P08169 | SFHSVGDSLLKTASRSLLEFNTTVNCKQQNHKIQSSITFLC |
| P08169 | AVCQVKKADSTQVKVAGRPQNLTLRYSDGDLTLIYFGGEEC |
| P08169 | FGGEECSSGFQRMSVINFECNQTAGNNGRGAPVFTGEVDCT |
| P08169 | LQTGQARGCPEDAAVCAVDKNGSKNLGRFISSPTREKGNIQ |
| P08169 | NIQLSYSDGDECGGGQKIITNITLMCKPGDLESAPVLTTSR |
| P08169 | FYEFEWRTAAACVLSRTEGDNCTVFDSQAGFSFDLTPLTKK |
| P08169 | ITFLCDRDAGVGFPEYQEEDNSTYNFRWYTSYACPEEPLEC |
| P08169 | GVAKTGPMVEDSGSLLLEYVNGSACTTSDQRRTTYTTRIHL |
| P08169 | DQVCSIKDPNSGYVFDLNPLNNSRGYVVLGIGKTFLFNVCG |
| P08169 | GRLVGLEKSLQLSTEGFITLNYTGLPSHPNGRADAFIIRFV |
| P08169 | VTEDSKLNLGVVQISPQVGANGSLSLVYVNGDKCKNQRFST |
| P08169 | YCEVRDPRHGNLYNLIPLGLNDTVVRAGEYTYYFRVCGELT |
| P08169 | QKVAGLFNQKLTYENGVLKMNYTGGDTCHKVYQRSTTIFFY |
| P08169 | LFFSWHTPLACEQTTECSVRNGSSLIDLSPLIHRTGGYEAY |
| P08169 | ANEVYLNFESSTPCLADRHFNYTSLITFHCKRGVSMGTPKL |
| P08169 | PDEVKTDGCSLTDEQLYYSFNLSSLSKSTFKVTRGPHTYSV |
| P08169 | LDVVDDRVIVTYSKGHYCGDNKTASAVIELTCAKTVGRPSF |
| P08169 | HFSWDSRAACAVKPQEVQMVNGTITNPANGRSFSLGDIYFK |
| P08169 | ASICQRKANDQHFSRKVGTSNQTRYYVQDGDLDVVFTSSSK |
| P11717 | TYHSVGDSVLRSATRSLLEFNTTVSCDQQGTNHRVQSSIAF |
| P11717 | AVCQVKKSDTSQVKAAGRYHNQTLRYSDGDLTLIYFGGDEC |
| P11717 | FGGDECSSGFQRMSVINFECNKTAGNDGKGTPVFTGEVDCT |
| P11717 | LQEGKARGCPEDAAVCAVDKNGSKNLGKFISSPMKEKGNIQ |
| P11717 | NIQLSYSDGDDCGHGKKIKTNITLVCKPGDLESAPVLRTSG |
| P11717 | FYEFEWHTAAACVLSKTEGENCTVFDSQAGFSFDLSPLTKK |
| P11717 | ITFLCDRDAGVGFPEYQEEDNSTYNFRWYTSYACPEEPLEC |

| | |
|---|---|
| P11717 | GMAKTGPVVEDSGSLLLEYVNGSACTTSDGRQTTYTTRIHL |
| P11717 | DQACSIRDPNSGFVFNLNPLNSSQGYNVSGIGKIFMFNVCG |
| P11717 | RDPNSGFVFNLNPLNSSQGYNVSGIGKIFMFNVCGTMPVCG |
| P11717 | VSEGNSWNLGVVQMSPQAAANGSLSIMYVNGDKCGNQRFST |
| P11717 | NCEVKDPRHGNLYDLKPLGLNDTIVSAGEYTYYFRVCGKLS |
| P11717 | HKVAGLLTQKLTYENGLLKMNFTGGDTCHKVYQRSTAIFFY |
| P11717 | LFFSWHTPLACEQATECSVRNGSSIVDLSPLIHRTGGYEAY |
| P11717 | ANEIYLNFESSTPCLADKHFNYTSLIAFHCKRGVSMGTPKL |
| P11717 | PDEVRMDGCTLTDEQLLYSFNLSSLSTSTFKVTRDSRTYSV |
| P11717 | LGVIGDKVVVTYSKGYPCGGNKTASSVIELTCTKTVGRPAF |
| P11717 | YFSWDSRAACAVKPQEVQMVNGTITNPINGKSFSLGDIYFK |
| Q07113 | NCRSVGDSLLRSSARSLLEFNTTMGCQPSDSQHRIQTSITF |
| Q07113 | AVCQEKKADSTQVKIAGRHQNQTLRYSDGDLTLIYSGGDEC |
| Q07113 | SGGDECSSGFQRMSVINFECNKTAGKDGRGEPVFTGEVDCT |
| Q07113 | LQEGKARNCPEDAAVCAVDKNGSKNLGKFVSSPTKEKGHIQ |
| Q07113 | HIQLSYTDGDDCGSDKKISTNITLVCKPGDLESAPVLRAAR |
| Q07113 | FYEFEWHTAAACVLSKTEGENCTVLDAQAGFSFDLSLLTKK |
| Q07113 | ITFLCDRDAGVGFPEYQEEDNSTYNFRWYTSYACPEEPLEC |
| Q07113 | GVAKIGPVVEESGSLLLEYVNGSACTTSDGQLTTYSTRIHL |
| Q07113 | DQACSIRDPSSGFVFNLSPLNDSAQGHVVLGIGKTFVFNIC |
| Q07113 | VSEDNSFNLGVVQISPQATGNGSLSILYVNGDRCGDQRFST |
| Q07113 | NCQVKDPRHGNLYDLKPLGLNDTIVSVGEYTYYLRVCGKLS |
| Q07113 | QKVAGLLSQKLTFENGLLKMNYTGGDTCHKVYQRSTTIYFY |
| Q07113 | ETSDCSYMFEWRTQYACPPFNVTECSVQDAAGNSIDLSSLS |
| Q07113 | SLSPHAGTEPCPPEAAVCLLNGSKPVNLGKVRDGPQWTDGV |
| Q07113 | TNPSTGHLFDLSSLSGRAGINASYSEKGLVFMSICEENENC |
| Q07113 | LFFSWHTPLACEQATECTVRNGSSIIDLSPLIHRTGGYEAY |
| Q07113 | ANEVYLNFESSTHCLADRYMNYTSLITFHCKRGVSMGTPKL |
| Q07113 | PDEVKTQGCAVTDEQLLYSFNLTSLSTSTFKVTRDARTYSI |
| Q07113 | LEVIDETVIVTYSKGYPCGGNKTASSVIELTCAKTVGRPAF |
| Q07113 | YFYWYSRAACAVRPQEVTMVNGTLTNPVTGKSFSLGEIYFK |
| O95297 | LLTAGVSALEVYTPKEIFVANGTQGKLTCKFKSTSTTGGLT |
| O95297 | AGDLDKKDASINIENMQFIHNGTYICDVKNPPDIVVQPGHI |
| Q3TEW6 | LLTARISALEVHTPKEIFVVNGTQGKLTCTFDSPNTTGWLT |
| Q3TEW6 | AGDLDKKDASINIENIQAVHNGTYICDVKNPPDIVVRPGHI |
| O70255 | TALCPTEAVEIYTSGALEAVNGTDVRLKCTFSSFAPVGDAL |
| O70255 | DGNPERYDVSILLWKLQFDDNGTYTCQVKNPPDVDGLVGTI |
| O60487 | TALWPIAAVEIYTSRVLEAVNGTDARLKCTFSSFAPVGDAL |
| O60487 | DGNPERYDASILLWKLQFDDNGTYTCQVKNPPDVDGVIGEI |
| P22897 | VAITLYACDSKSEFQKWECKNDTLLGIKGEDLFFNYGNRQE |
| P22897 | NAKWENLECVQKLGYICKKGNTTLNSFVIPSESDVPTHCPS |

| | |
|---|---|
| P22897 | KKHHFYCYMIGHTLSTFAEANQTCNNENAYLTTIEDRYEQA |
| P22897 | SGFWNDINCGYPNAFICQRHNSSINATTVMPTMPSVPSGCK |
| P22897 | NDINCGYPNAFICQRHNSSINATTVMPTMPSVPSGCKEGWN |
| P22897 | AFAWLQMETSNERVWIALNSNLTDNQYTWTDKWRVRYTNWA |
| P22897 | KLKSACVYLDLDGYWKTAHCNESFYFLCKRSDEIPATEPPQ |
| P22897 | SKTNFWIGLFRNVEGTWLWINNSPVSFVNWNTGDPSGERND |
| Q9UBG0 | HGLQGCLEAQGGQVRVTPACNTSLPAQRWKWVSRNRLFNLG |
| Q9UBG0 | RWHCRTLGDQLSLLLGARTSNISKPGTLERGDQTRSGQWRI |
| Q9UBG0 | GGWQNRDCSIALPYVCKKKPNATAEPTPPDRWANVKVECEP |
| Q9UBG0 | VSSLIYNWEGEYFWTALQDLNSTGSFFWLSGDEVMYTHWNR |
| Q9UBG0 | REDWGDQRCLTALPYICKRSNVTKETQPPDLPTTALGGCPS |
| Q9UBG0 | AQLVTITNPLEQAFITASLPNVTFDLWIGLHASQRDFQWVE |
| Q9UBG0 | LGMNFNPKGGTLVWQDNTAVNYSNWGPPGLGPSMLSHNSCY |
| Q64449 | RNRLFNLGATQCLGTGWPVTNTTVSLGMYECDREALSLRWQ |
| Q64449 | RWQCRTLGDQLSLLLGARASNASKPGTLERGDQTRSGHWNI |
| Q64449 | GGWQNHDCSIALPYVCKKKPNATVEPIQPDRWTNVKVECDP |
| Q64449 | AQLVTIANPLEQAFITASLPNVTFDLWIGLHASQRDFQWIE |
| Q64449 | LGMNFNPKGGTLVWQDNTAVNYSNWGPPGLGPSMLSHNSCY |
| P33527 | LRGFCSADGSDPLWDWNVTWNTSNPDFTKCFQNTVLVWVPC |
| P33527 | HVSALASNYWLSLWTDDPIVNGTQEHTKVRLSVYGALGISQ |
| P21757 | AVLIPLIGIVAAQLLKWETKNCSVSSTNANDITQSLTGKGN |
| P21757 | NCSVSSTNANDITQSLTGKGNDSEEEMRFQEVFMEHMSNME |
| P21757 | KRIQHILDMEANLMDTEHFQNFSMTTDQRFNDILLQLSTLF |
| P21757 | SSVQGHGNAIDEISKSLISLNTTLLDLQLNIENLNGKIQEN |
| P21757 | IQENTFKQQEEISKLEERVYNVSAEIMAMKEEQVHLEQEIK |
| P21757 | MKEEQVHLEQEIKGEVKVLNNITNDLRLKDWEHSQTLRNIT |
| P21757 | LNNITNDLRLKDWEHSQTLRNITLIQGPPGPPGEKGDRGPT |
| O97148 | DILECDYFDTVDISAAQKLQNGSYLFEGLLVPAILTGEYDF |
| O97148 | PCVRFCCPHDHIMDNGVCYDNMSDEELAELDPFLNVTLDDG |
| O97148 | NGVCYDNMSDEELAELDPFLNVTLDDGSVSRRHFKNELIVQ |
| O97148 | CDGMFYLDNREEQDKYTLFENGTFFRHFDRVTLRKREYCLQ |
| O97148 | DRVTLRKREYCLQHLTFADGNATSIRIAPHNCLIVPSITGQ |
| O97148 | LFIIMGLSWSLEIGSYFSQSNQTWANVFLVADYLNWSQGII |
| P34576 | SDTFIMDGNWDCEDGSDEFINKTLTANCTRNNKVNIATNSL |
| P34576 | DGNWDCEDGSDEFINKTLTANCTRNNKVNIATNSLSLGKLK |
| P34576 | YGTIMTPPEFHFNRDDIRCGNKTCGLHESCQKNSESKYECI |
| P34576 | CRCQDDYVDVSREGARKPGRNCTQAINECASNLHNCDTHAI |
| P34576 | PQQSDIARVAIILTDGRSQDNVTGPADSARKLSINTFAIGV |
| P34576 | SLAPRYVNTKVDYITHPKTKNSSWDQGLLFKYEVQTTKSQS |
| P34576 | ELKNAFVTAEVNDIVNPVLMNASYDTGLLFNTTVHFRKGMV |
| P34576 | VNDIVNPVLMNASYDTGLLFNTTVHFRKGMVHVPSDAYYQL |

| | |
|---|---|
| P34576 | SVPGSLCVLDYCSDVNFCPTNTTCKNMEQQAECKCDAGFMD |
| P34576 | LGDDTLCMHVRDVDECALGLNNCSGVAHCIDRAVGYTCKCP |
| P34576 | GDDTLCMHVRDVDECALGLNNCSGVAHCIDRAVGYTCKCPD |
| P34576 | ALLCDLCNAHGDCVHNTATNNITCVCTDGWTGPQCQVAPSN |
| P34576 | NITCVCTDGWTGPQCQVAPSNASLVLLILLALLFLLLTLCC |
| Q8MI01 | ISILSSLLLFGSHGEEGQKTNTTESTAEDLKTMENQSVPLE |
| Q8MI01 | EEGQKTNTTESTAEDLKTMENQSVPLESKANLTSDKENRET |
| Q8MI01 | STAEDLKTMENQSVPLESKANLTSDKENRETSNPKASNFSF |
| Q8MI01 | SKANLTSDKENRETSNPKASNFSFEDPSNKTHETGFYSNLS |
| Q8MI01 | KENRETSNPKASNFSFEDPSNKTHETGFYSNLSTDNSSRSP |
| Q8MI01 | ASNFSFEDPSNKTHETGFYSNLSTDNSSRSPSLMPTLSPRS |
| Q8MI01 | FEDPSNKTHETGFYSNLSTDNSSRSPSLMPTLSPRSPSTHS |
| Q8MI01 | MPTLSPRSPSTHSFVSKLPWNSSIADNSLLPASAPPNTTVP |
| Q8MI01 | KLPWNSSIADNSLLPASAPPNTTVPVSSENFTLSSINDTMK |
| Q8MI01 | DNSLLPASAPPNTTVPVSSENFTLSSINDTMKAPDNSSITV |
| Q8MI01 | SAPPNTTVPVSSENFTLSSINDTMKAPDNSSITVSNLPSGP |
| Q8MI01 | PVSSENFTLSSINDTMKAPDNSSITVSNLPSGPNTTSVTPM |
| Q8MI01 | DTMKAPDNSSITVSNLPSGPNTTSVTPMVTEGWPTTTRESM |
| Q8MI01 | SMEGFTVYQETTLHPTLKFTNNSKIFPNTSDPQEENRNTGV |
| Q8MI01 | YQETTLHPTLKFTNNSKIFPNTSDPQEENRNTGVVFGAILG |
| P43121 | VEVEVGSTALLKCGLSQSQGNLSHVDWFSVHKEKRTLIFRV |
| P43121 | KREAGGGYRCVASVPSIPGLNRTQLVNVAIFGPPWMAFKER |
| P43121 | GPPWMAFKERKVWVKENMVLNLSCEASGHPRPTISWNVNGT |
| P43121 | VLNLSCEASGHPRPTISWNVNGTASEQDQDPQRVLSTLNVL |
| P43121 | VTPELLETGVECTASNDLGKNTSILFLELVNLTTLTPDSNT |
| P43121 | ECTASNDLGKNTSILFLELVNLTTLTPDSNTTTGLSTSTAS |
| P43121 | KNTSILFLELVNLTTLTPDSNTTTGLSTSTASPHTRANSTS |
| P43121 | PDSNTTTGLSTSTASPHTRANSTSTERKLPEPESRGVVIVA |
| Q8R2Y2 | VEAEVGSTALLKCGPSRASGNFSQVDWFLIHKERQILIFRV |
| Q8R2Y2 | VTPELLETGAECTASNSLGSNTTTIVLKLVTLTTLIPDSSQ |
| Q99102 | QTSTLTHRTTSTPSFSPSVHNVTGTVSQKTSPSGETATSSL |
| Q99102 | VSQKTSPSGETATSSLCSVTNTSMMTSEKITVTTSTGSTLG |
| Q99102 | SSSPMLDRHTSQQITTAPSTNHSTIHSTSTSPQESPAVSQR |
| Q99102 | GVSYTFNGLGDFLLVGAQDGNSSFLLQGRTAQTGSAQATNF |
| Q99102 | GPVTVQWLLEPHDAIRVLLDNQTVTFQPDHEDGGGQETFNA |
| Q99102 | DNQTVTFQPDHEDGGGQETFNATGVLLSRNGSEVSASFDGW |
| Q99102 | DHEDGGGQETFNATGVLLSRNGSEVSASFDGWATVSVIALS |
| Q99102 | SVIALSNILHASASLPPEYQNRTEGLLGVWNNNPEDDFRMP |
| Q99102 | RTEGLLGVWNNNPEDDFRMPNGSTIPPGSPEEMLFHFGMTW |
| Q99102 | TIPPGSPEEMLFHFGMTWQINGTGLLGKRNDQLPSNFTPVF |
| Q99102 | KRNDQLPSNFTPVFYSQLQKNSSWAEHLISNCDGDSSCIYD |

| | |
|---|---|
| Q99102 | HLISNCDGDSSCIYDTLALRNASIGLHTREVSKNYEQANAT |
| Q99102 | LRNASIGLHTREVSKNYEQANATLNQYPPSINGGRVIEAYK |
| Q99102 | IEAYKGQTTLIQYTSNAEDANFTLRDSCTDLELFENGTLLW |
| Q99102 | NAEDANFTLRDSCTDLELFENGTLLWTPKSLEPFTLEILAR |
| Q99102 | SALQPRTVVCHCNAESQCLYNQTSRVGNSSLEVAGCKCDGG |
| Q99102 | VVCHCNAESQCLYNQTSRVGNSSLEVAGCKCDGGTFGRYCE |
| Q99102 | EPCFPSVHCVPGKGCEACPPNLTGDGRHCAALGSSFLCQNQ |
| Q99102 | PNLTGDGRHCAALGSSFLCQNQSCPVNYCYNQGHCYISQTL |
| Q99102 | PTVNLELPLRVIQLLLSEEENASMAEVNASVAYRLGTLDMR |
| Q99102 | PLRVIQLLLSEEENASMAEVNASVAYRLGTLDMRAFLRNSQ |
| Q99102 | RNDVVFQPISGEDVRDVTALNVSTLKAYFRCDGYKGYDLVY |
| P98088 | LVLMWNHDDSLLLELDTKYANKTCGLCGDFNGMPVVSELLS |
| P98088 | LQKMDDPTDQCQDPVPEPPRNCSTGFGICEELLHGQLFSGC |
| P98088 | ACVYNGAAYAPGATYSTDCTNCTCSGGRWSCQEVPCPGTCS |
| P98088 | KASGEVFLNQIYTQLPISAANVTIFRPSTFFIIAQTSLGLQ |
| P98088 | DVIYHTTDGTGGCISARCGANGTIERRVYPCSPTTPVPPTT |
| P98088 | SAPTASTTSAPTSTSSAPTTNTTSAPTTSTTSAPITSTISA |
| P98088 | ELGCPNAVPPRKKGETWATPNCSEATCEGNNVISLRPRTCP |
| P98088 | SGWGDPHYITFDGTYYTFLDNCTYVLVQQIVPVYGHFRVLV |
| P98088 | VQVMFSGLIFSVEVPFSKFANNTEGQCGTCTNDRKDECRTP |
| P98088 | ECRTPRGTVVASCSEMSGLWNVSIPDQPACHRPHPTPTTVG |
| P98088 | CPADKVYQPCGPSNPSYCYGNDSASLGALPEAGPITEGCFC |
| P98088 | VPVPAAPQAGQCCPQYSCACNTSRCPAPVGCPEGARAIPTY |
| P98088 | CPEGARAIPTYQEGACCPVQNCSWTVCSINGTLYQPGAVVS |
| P98088 | TYQEGACCPVQNCSWTVCSINGTLYQPGAVVSSSLCETCRC |
| P98088 | EYQEQSGQCCGTCVQVACVTNTSKSPAHLFYPGETWSDAGN |
| P98088 | ARMSKDGCCRFCPPPPPPYQNQSTCAVYHRSLIIQQQGCSS |
| P98088 | EGNTVEHRCQCCQELRTSLRNVTLHCTDGSSRAFSYTEVEE |
| Q98UI9 | PCMFQGKVYSSGGTYSTPCQNCTCKGGHWSCISLPCSGSCS |
| Q98UI9 | IRVQMKPVMQLSITVDHSYQNRTSGLCGNFNNIQTDDFRTA |
| Q98UI9 | PCSNSVDKEKFAQHWCALLSNTSSTFAACHSVVDPSVYIKR |
| Q98UI9 | CDPSEECPETMVYNYSVKYCNQSCRSLDEPDPLCKVQIAPM |
| Q98UI9 | LDCIGETVLVKDCPAPMYYFNCSSAGPGAIGSECQKSCKTQ |
| Q98UI9 | KPGETIRVDCNTCTCNKRQWNCTDNPCKGTCTVYGNGHYMS |
| Q98UI9 | EMSIQEFGNSWKITSTCSNINMTDLCADQPFKSALGQKHCS |
| Q98UI9 | LCTEDSKDCLCCYNGKTYPLNETIYSQTEGTKCGNAFCGPN |
| Q98UI9 | PATALAPMSDCLGLIPPRKFNESWDFGNCQIATCLGEENNI |
| Q98UI9 | SGWGNEHYVTFDGTYYHFKENCTYVLVELIQPSSEKFWIHI |
| Q98UI9 | VYVSYSRLAFYIKLPFGKYYNNTMGLCGTCTNQKSDDARKR |
| Q98UI9 | PVKIENYQHCEPSELCKIIWNLTECHRVVPPQPYYEACVAS |
| Q98UI9 | KPREAWEHDCQYCTCDEETLNISCFPRPCAKSPPINCTKEG |

| | |
|---|---|
| Q98UI9 | DEETLNISCFPRPCAKSPPINCTKEGFVRKIKPRLDDPCCT |
| Q98UI9 | LDDPCCTETVCECDIKTCIINKTACDLGFQPVVAISEDGCC |
| Q98UI9 | NFPFGSVTIEVGKSYKAPYDNCTQYTCTESGGQFSLTSTVK |
| Q9HC84 | SRPVVTRVVIKAQGLVLEASNGSVLINGQREELPYSRTGLL |
| Q9HC84 | LTFLWNGEDSALLELDPKYANQTCGLCGDFNGLPAFNEFYA |
| Q9HC84 | LQKLDGPTEQCPDPLPLPAGNCTDEEGICHRTLLGPAFAEC |
| Q9HC84 | PLGQCPCTHGGRTYSPGTSFNTTCSSCTCSGGLWQCQDLPC |
| Q9HC84 | QADGGVFLNSIYTQLPLSAANITLFTPSSFFIVVQTGLGLQ |
| Q9HC84 | GASLQKSTGCAAPMVYLDCSNSSAGTPGAECLRSCHTLDVG |
| Q9HC84 | DRYSFEGSCEYILAQDYCGDNTTHGTFRIVTENIPCGTTGT |
| Q9HC84 | LEACTCTYEDRTYSYQDVIYNTTDGLGACLIAICGSNGTII |
| Q9HC84 | DVIYNTTDGLGACLIAICGSNGTIIRKAVACPGTPATTPFT |
| Q9HC84 | AGGHLCQQPKDIECQAESFPNWTLAQVGQKVHCDVHFGLVC |
| Q9HC84 | STSQAETSTPRTETTMSPLTNTTTSQGTTRCQPKCEWTEWF |
| Q9HC84 | TSSTVTPSSALGTTHTPPVPNTTATTHGRSLSPSSPHTVRT |
| Q9HC84 | TSSTVTPSSALGTTHTPPVPNTTATTHGRSLPPSSPHTVRT |
| Q9HC84 | TSSTVTPSSALGTTHTPPVPNTTATTHGRSLSPSSPHTVRT |
| Q9HC84 | PETTHTSTVLTTTATMTRATNSTATPSSTLGTTRILTELTT |
| Q9HC84 | STPCFCRAFGQFFSPGEVIYNKTDRAGCHFYAVCNQHCDID |
| Q9HC84 | PLSSPSPAPGCDNAIPLRQVNETWTLENCTVARCVGDNRVV |
| Q9HC84 | APGCDNAIPLRQVNETWTLENCTVARCVGDNRVVLLDPKPV |
| Q9HC84 | TVARCVGDNRVVLLDPKPVANVTCVNKHLPIKVSDPSQPCD |
| Q9HC84 | SMWGGSHYSTFDGTSYTFRGNCTYVLMREIHARFGNLSLYL |
| Q9HC84 | YTFRGNCTYVLMREIHARFGNLSLYLDNHYCTASATAAAAR |
| Q9HC84 | VSVTFNGQVFQARLPYSLFHNNTEGQCGTCTNNQRDDCLQR |
| Q9HC84 | VTVTRPRAENPCCPETVCVCNTTTCPQSLPVCPPGQESICT |
| Q9HC84 | TQEEGDCCPTFRCRPQLCSYNGTFYGVGATFPGALPCHMCT |
| Q9HC84 | CTCLSGDTQDPTVQCQEDACNNTTCPQGFEYKRVAGQCCGE |
| Q9HC84 | TCLSGDTQDPTVQCQEDACNNTTCPQGFEYKRVAGQCCGEC |
| Q9HC84 | CCGECVQTACLTPDGQPVQLNETWVNSHVDNCTVYLCEAEG |
| Q9HC84 | LTPDGQPVQLNETWVNSHVDNCTVYLCEAEGGVHLLTPQPA |
| Q9HC84 | SLRKTGCCYSCEEDSCQVRINTTILWHQGCETEVNITFCEG |
| Q9HC84 | SCQVRINTTILWHQGCETEVNITFCEGSCPGASKYSAEAQA |
| Q9HC84 | QCTCCQERRVHEETVPLHCPNGSAILHTYTHVDECGCTPFC |
| P12021 | EGAGTSGVGFKTEATTFPGENETTRVGIATGTTGIVSRKTL |
| P12021 | GTGSRPGTTGELSGTTIASGNATTEATTSTETRIGPQTGAQ |
| P12021 | SPKVSSPETTAGATEDQENENKTGCPAPLPPPPVCHGPLGE |
| P12021 | KECPSPPTCKTGERLIKFKANDTCCEIGHCEKRTCLFNNTD |
| P12021 | FKANDTCCEIGHCEKRTCLFNNTDYEVGSSFDDPNNPCVTY |
| P12021 | VYDSKQCCYTCKSSCKPSPVNVTVRYNGCTIKVEMARCVGE |
| P04220 | WTRQDGEAVKTHTNISESHPNATFSAVGEASICEDDWDSGE |

| | |
|---|---|
| P28665 | VPSQLYTETPEKICLHLYQLNETVTVTASLVSQSGRKNLFD |
| P28665 | TGCREVNSQLDNNGCSTQEVNITELQSKKRNYEVQLFHVNA |
| P28665 | VNITELQSKKRNYEVQLFHVNATVTEEGTGLEFSRSGTTKI |
| P28665 | LKELIFYYLVMAQGSIIQTGNHTHQVEPGEAPVKGKFALEI |
| P28665 | LTETIRKYFPETWVWDIVTVNSTGLAEVEMTVPDTITEWKA |
| P28665 | VEMTVPDTITEWKAGALCLSNDTGLGLSSVVPLQAFKPFFV |
| P28665 | SANGRHTSSWLVTPKSLGNVNFSVSAEAQQSSEPCGSEVAT |
| P28665 | GCGEQNMVLFAPNIYVLKYLNETQQLTQKIKTKALGFLRAG |
| P28665 | VVSKALSCLESSWKTIEQERNASFVYTKALMAYAFALAGNQ |
| P28665 | GNQNKRDEILKSLDEEAIKENNSIHWKRPQKSRKSEHHLYK |
| P28665 | HVSRTEVSNNNVLIYLDQVTNQTLAFSFIIQQDIPVRNLQP |
| P04939 | EASYEREKIEEHGSMRAFVENITVLENSLVFKFHLIVNEEC |
| Q62838 | KLVKLEVEVFARILRAPESHNVTFGSFVTLRCTAIGMPVPT |
| Q62838 | AQYRGEVCDAVLVKDSLVFFNTSYPDPEEAQELLIHTAWNE |
| Q62838 | SMHQDPTACTRLPYLDYKKENITTFPSITSSKPSVDIPNLP |
| P10522 | VGDPHRKDGSIVIHNLDYGDNGTFTCDVKNPPDIVGKTSQV |
| P29736 | IEGTIGRGLNIWDGFTHRYPNKSGPDHGNGDTTCDSFSYWQ |
| P29736 | DTTCDSFSYWQKDIDVLDELNATGYRFSIAWSRIIPRGKRS |
| P29736 | SALDAPGRCSPTVDPSCYAGNSSTEPYIVAHHQLLAHAKVV |
| P29736 | YIVAHHQLLAHAKVVDLYRKNYTHQGGKIGPTMITRWFLPY |
| P29736 | YTHQGGKIGPTMITRWFLPYNDTDRHSIAATERMKEFFLGW |
| P29736 | IAATERMKEFFLGWFMGPLTNGTYPQIMIDTVGERLPSFSP |
| P29736 | DFLGLNYYFTQYAQPSPNPVNSTNHTAMMDAGAKLTYINAS |
| P29736 | GLNYYFTQYAQPSPNPVNSTNHTAMMDAGAKLTYINASGHY |
| P29736 | PVNSTNHTAMMDAGAKLTYINASGHYIGPLFEKDKADSTDN |
| Q02083 | LLLLAGAGLSAASPPAAPRFNVSLDSVPELRWLPVLRHYDL |
| Q02083 | LERFLPQPFTGEIRGMCDFMNLSLADCLLVNLAYESSVFCT |
| Q02083 | DHWKPAPKEDDRRTSAIKALNATGQANLSLEALFQILSVVP |
| Q02083 | QANLSLEALFQILSVVPVYNNFTIYTTVMSAGSPDKYMTRI |
| Q9W4C5 | CVNSRPQEYVDNLLTGVSLANESARNLSGIVANDETEKLQS |
| Q9W4C5 | PQEYVDNLLTGVSLANESARNLSGIVANDETEKLQSSSELY |
| Q90744 | GCYSSGKEQAQGYPQMARALNSTGRPIVYSCSWPAYQGGLP |
| Q90744 | RPIVYSCSWPAYQGGLPPKVNYTLLGEICNLWRNYDDIQDS |
| Q90744 | AYEVQDVYSGKIISGLKTGDNFTVIINPSGVVMWYLCPKAL |
| P17050 | FLADYVHSLGLKLGIYADMGNFTCMGYPGTTLDKVVQDAQT |
| P17050 | GCFSTPEERAQGYPKMAAALNATGRPIAFSCSWPAYEGGLP |
| P17050 | RPIAFSCSWPAYEGGLPPRVNYSLLADICNLWRNYDDIQDS |
| P17050 | VFFSCRTDMPYRYHSSLGQLNFTGSVIYEAQDVYSGDIISG |
| P17050 | IYEAQDVYSGDIISGLRDETNFTVIINPSGVVMWYLYPIKN |
| Q9Y3Q0 | KKFGLDSAKLVHYDVLLSYPNETNANYISIVDEHETEIFKT |
| Q9Y3Q0 | EHETEIFKTSYLEPPPDGYENVTNIVPPYNAFSAQGMPEGD |

| | |
|---|---|
| Q9Y3Q0 | VYVNYARTEDFFKLEREMGINCTGKIVIARYGKIFRGNKVK |
| Q9Y3Q0 | ILLRYLGGIAPPDKSWKGALNVSYSIGPGFTGSDSFRKVRM |
| Q9Y3Q0 | VKILQERSIAYINSDSSIEGNYTLRVDCTPLLYQLVYKLTK |
| Q9Y3Q0 | IPFNIQDYAEALKNYAASIYNLSKKHDQQLTDHGVSFDSLF |
| Q9Y3Q0 | HDQQLTDHGVSFDSLFSAVKNFSEAASDFHKRLIQVDLNNP |
| Q9CZR2 | KKFGLDSANLVHYDVLLSYPNETNANYVSIVDEHGVEIFKT |
| Q9CZR2 | EHGVEIFKTSYLEPPPDGYENVTNIIPPYNAFSASGMPEGE |
| Q9CZR2 | VYVNYARTEDFFKLEREMNINCTGKIVIARYGKIFRGNKVK |
| Q9CZR2 | RLLRNLGGAAPPDKSWKGSLNVSYNIGPGFTGSEYSRNIRM |
| Q9CZR2 | NLGGAAPPDKSWKGSLNVSYNIGPGFTGSEYSRNIRMHVNN |
| Q9CZR2 | AKLLQERSIAYINSDSAIEGNYTLRVDCTPLLNQLVYKVAR |
| Q9CZR2 | IPFNIQDYAKALKNYAASIFNISKKHDQQLRNHAVSFDPLF |
| Q9CZR2 | HDQQLRNHAVSFDPLFSAVKNFSEAASDFHRRLTQVDLNNP |
| O09043 | LSKFMNTQYFGTIGLGTPPQNFTVVFDTGSSNLWVPSTRCH |
| O09043 | SLACWFHHRFNPKASSSFRPNGTKFAIQYGTGRLSGILSQD |
| O09043 | GTKFAIQYGTGRLSGILSQDNLTIGGIHDAFVTFGEALWEP |
| O09043 | QCSKTPTLPPVSFHLGGVWFNLTGQDYVIKILQSDVGLCLL |
| Q13508 | NEVFQVSQEGAGNNLILQSINKTCSHYECAFLGGLKTENCI |
| Q21432 | SVQLRFDRTLQIKEALLEKGNLTADIVPVDNGVYDLDTMLT |
| Q21432 | HALGVAHQHLRNDRDQFITINWSNIDPQQYDAFVVVDSKLY |
| Q22396 | FYNLPLEMQAMFRDAINYLENHTCLKFEYNENAETAVRIRK |
| Q22396 | VMLYARDPHSDKRIPIDPEYNFTMGSLRVAFYDMVLLNKFY |
| Q22396 | YKTDRRIVSPLVCCDNDNLWNKTRSSTNNPFIIAKYGNNRT |
| P98061 | GRQGVAPPVTFAALNYGPKNNQTKKFEELNQDINEYTFESD |
| P98061 | SMGQREAPAFSDIIGVNKLYNCTSQCKIQMKCSNCGITDSR |
| P98061 | SWETFDAKAGDPSSFSSSTDNSTNCYWHIKAPEGQQIEFKM |
| Q21059 | IGQRVAPSFADVKRINFAYCNSTCSNYLDCQNGGYINPNDC |
| P98060 | DGSHTQREQRIIELALEHWHNITCLNFQRNDQANSGNRIVF |
| P98060 | EFEMYCKIRHSLCMDYVEVRNSTDFANTGMRYCCYGTPPTR |
| P13590 | KDIQVIVNVPPSVRARQSTMNATANLSQSVTLACDADGFPE |
| P13590 | EQDATIHLKVFAKPKITYVENKTAMELEDQITLTCEASGDP |
| P13590 | LTCEASGDPIPSITWKTSTRNISNEEKTLDGRIVVRSHARV |
| P13590 | QYAPKLQGPVAVYTWEGNQVNITCEVFAYPSAVISWFRDGQ |
| P13590 | FAYPSAVISWFRDGQLLPSSNYSNIKIYNTPSASYLEVTPD |
| P13590 | TPSASYLEVTPDSENDFGNYNCTAVNRIGQESSEFILVQAD |
| P13591 | KDIQVIVNVPPTIQARQNIVNATANLGQSVTLVCDAEGFPE |
| P13591 | EQDATIHLKVFAKPKITYVENQTAMELEEQVTLTCEASGDP |
| P13591 | LTCEASGDPIPSITWRTSTRNISSEEKASWTRPEKQETLDG |
| P13591 | QYAPKLQGPVAVYTWEGNQVNITCEVFAYPSATISWFRDGQ |
| P13591 | FAYPSATISWFRDGQLLPSSNYSNIKIYNTPSASYLEVTPD |
| P13591 | TPSASYLEVTPDSENDFGNYNCTAVNRIGQESLEFILVQAD |

| | |
|---|---|
| P13595 | KDIQVIVNVPPTVQARQSIVNATANLGQSVTLVCDADGFPE |
| P13595 | EQDASIHLKVFAKPKITYVENQTAMELEEQVTLTCEASGDP |
| P13595 | LTCEASGDPIPSITWRTSTRNISSEEKTLDGHMVVRSHARV |
| P13595 | QYAPKLQGPVAVYTWEGNQVNITCEVFAYPSATISWFRDGQ |
| P13595 | FAYPSATISWFRDGQLLPSSNYSNIKIYNTPSASYLEVTPD |
| P13595 | PSATISWFRDGQLLPSSNYSNIKIYNTPSASYLEVTPDSEN |
| P13595 | TPSASYLEVTPDSENDFGNYNCTAVNRIGQESLEFILVQAD |
| O15394 | TISDNRFAMLANNNLQILNINKSDEGIYRCEGRVEARGEID |
| O15394 | RDIIVIVNVPPAISMPQKSFNATAERGEEMTFSCRASGSPE |
| O15394 | EDEKQAFLQVFVQPHIIQLKNETTYENGQVTLVCDAEGEPI |
| O15394 | IGGHQKSMYLDIEYAPKFISNQTIYYSWEGNPINISCDVKS |
| O15394 | YAPKFISNQTIYYSWEGNPINISCDVKSNPPASIHWRRDKL |
| O15394 | KSNPPASIHWRRDKLVLPAKNTTNLKTYSTGRKMILEIAPT |
| O15394 | TGRKMILEIAPTSDNDFGRYNCTATNHIGTRFQEYILALAD |
| O15394 | WKIVRSHGVQTMVVLNNLEPNTTYEIRVAAVNGKGQGDYSK |
| P55067 | RVAKGWQGRVSLPAYPRHRANATLLLGPLRASDSGLYRCQV |
| P55067 | TPRRRCGGSAPGVRTVYRFANRTGFPAPGARFDAYCFRAHH |
| P55067 | SSMQATKHPISGPWASLDSSNVTVNPVPSDAGILGTESGVL |
| P55067 | EDPTDPCENNPCLHGGTCRTNGTMYGCSCDQGYAGENCEID |
| P55067 | NLPYVCKKGTVLCGPPPAVENASLVGVRKVKYNVHATVRYQ |
| Q6PIU2 | KYWVDYFKGNYDFVQAMIVNNHTSLDVEEAAAVRARLNWTS |
| Q6PIU2 | IVNNHTSLDVEEAAAVRARLNWTSLLPASFTKNYKPVVQTT |
| O00533 | GDLPKGRETKENYGKTLKIENVSYQDKGNYRCTASNFLGTA |
| O00533 | SWQKVEEVKPLEGRRYHIYENGTLQINRTTEEDAGSYSCWV |
| O00533 | EVKPLEGRRYHIYENGTLQINRTTEEDAGSYSCWVENAIGK |
| O00533 | SHLKHSLKLSWSKDGEAFEINGTEDGRIIIDGANLTISNVT |
| O00533 | EINGTEDGRIIIDGANLTISNVTLEDQGIYCCSAHTALDSA |
| O00533 | YRVTWKPQGAPVEWEEETVTNHTLRVMTPAVYAPYDVKVQA |
| O00533 | LYSGEDYPDTAPVIHGVDVINSTLVKVTWSTVPKDRVHGRL |
| O00533 | IKVDKDTATLSWGLPKKLNGNLTGYLLQYQIINDTYEIGEL |
| O00533 | TEESSTLGEGSKGIGKISGVNLTQKTHPIEVFEPGAEHIVR |
| Q9UI40 | NDKILDYTPQPPLSKEGESENSTDHAQGDYPKDIFSLEERR |
| P29120 | QKGITGKGVVITVLDDGLEWNHTDIYANYDPEASYDFNDND |
| P29120 | TSASAPLAAGIFALALEANPNLTWRDMQHLVVWTSEYDPLA |
| Q94EG3 | DVCVADLHSKVKVNGFPCKTNFTAADFSSLAISKPGATNNK |
| Q6ZQE4 | KELEQFSIWNFFSSFLKEKLNDTYVNVGLYSTKTCLKVEMI |
| P97798 | FYFLVEPVDTLSVRGSSVILNCSAYSEPSPNIEWKKDGTFL |
| P97798 | QPLLLDDRIVKLPSGTLVISNATEGDGGLYRCIVESGGPPK |
| P97798 | EISDVTEDDAGTYFCIADNGNKTVEAQAELTVQVPPGFLKQ |
| P97798 | VSTRFIKLTWRTPASDPHGDNLTYSVFYTKEGVDRERVENT |
| P97798 | DNLTYSVFYTKEGVDRERVENTSQPGEMQVTIQNLMPATVY |

| | |
|---|---|
| P97798 | VSTQDVAVRTLSDVPSAAPQNLSLEVRNSKSIVIHWQPPSS |
| P97798 | LIEGLDRGTEYNFRVAALTVNGTGPATDWLSAETFESDLDE |
| P97798 | RYYTVRWKTNIPANTKYKNANATTLSYLVTGLKPNTLYEFS |
| Q92859 | FYFLVEPVDTLSVRGSSVILNCSAYSEPSPKIEWKKDGTFL |
| Q92859 | QPLLLDDRVIKLPSGMLVISNATEGDGGLYRCVVESGGPPK |
| Q92859 | EISDVTEDDAGTYFCIADNGNETIEAQAELTVQAQPEFLKQ |
| Q92859 | VSTRFIKLTWRTPASDPHGDNLTYSVFYTKEGIARERVENT |
| Q92859 | DNLTYSVFYTKEGIARERVENTSHPGEMQVTIQNLMPATVY |
| Q92859 | VSTPDVAVRTLSDVPSAAPQNLSLEVRNSKSIMIHWQPPAP |
| Q92859 | LIEGLDRGTEYNFRVAALTINGTGPATDWLSAETFESDLDE |
| Q92859 | RYYTVRWKTNIPANTKYKNANATTLSYLVTGLKPNTLYEFS |
| Q18673 | LILCGTVDALPRAPYFNDDINKTTTTSEDKTVGNTVVEEEK |
| Q18673 | YTVGDSEGYQEASRLLQKSLNLSLDPCDDFFEYACRAWVDS |
| Q18673 | SQFTATREKVLAEMRKLYEDNTSIPTSKSIALIKQIYNTCM |
| Q18673 | TIQSRDVSVFFDFGPAEDSRNVSRRLLSFDQGSLGLGYSTR |
| Q18673 | YRKYTIGKVRYYTEDAGMAVNESKIESDVDEIIAFEKEWAQ |
| Q18673 | EIIAFEKEWAQILVAEEDRRNYTKLYNVRRFDDLKEYMSII |
| Q18673 | KLSYASGSMYVRKYFDANAKNTTLDMITDLQEAFRNMMHAN |
| Q18673 | GFDDTGRQFDNVGNLRDWWDNTTSSKFNERTQCIIEQYADV |
| P08473 | PKTEDIVAVQKAKALYRSCINESAIDSRGGEPLLKLLPDIY |
| P08473 | DENQLALEMNKVMELEKEIANATAKPEDRNDPMLLYNKMTL |
| P08473 | LAQIQNNFSLEINGKPFSWLNFTNEIMSTVNISITNEEDVV |
| P08473 | TQQSASNFKEQSQCMVYQYGNFSWDLAGGQHLNGINTLGEN |
| Q61391 | PKTEDIVAVQKAKTLYRSCINESAIDSRGGQPLLKLLPDIY |
| Q61391 | LNSKYGKKVLINFFVGTDDKNSTQHIIHFDQPRLGLPSRDY |
| Q61391 | DENQLSLEMNKVMELEKEIANATTKPEDRNDPMLLYNKMTL |
| Q61391 | EDRNDPMLLYNKMTLAKLQNNFSLEVNGKSFSWSNFTNEIM |
| Q61391 | MLLYNKMTLAKLQNNFSLEVNGKSFSWSNFTNEIMSTVNIN |
| Q61391 | LAKLQNNFSLEVNGKSFSWSNFTNEIMSTVNINIQNEEEVV |
| Q61391 | TQQSANNFKDQSQCMVYQYGNFSWDLAGGQHLNGINTLGEN |
| P07861 | PKTEDIVAVQKAKTLYRSCINESAIDSRGGQPLLTLLPDIY |
| P07861 | LNSKYGKKVLINFFVGTDDKNSTQHIIHFDQPRLGLPSRDY |
| P07861 | DENQLSLEMNKVMELEKEIANATTKPEDRNDPMLLYNKMTL |
| P07861 | EDRNDPMLLYNKMTLAKLQNNFSLEINGKPFSWSNFTNEIM |
| P07861 | LAKLQNNFSLEINGKPFSWSNFTNEIMSTVNINIQNEEEVV |
| P07861 | TQQSANNFKDQSQCMVYQYGNFTWDLAGGQHLNGINTLGEN |
| P08049 | PKTEDIVAVQKAKTLYRSCVNETAIDSRGGQPLLKLLPDVY |
| P08049 | DENQISVEMNKVMELEKEIANATTKSEDRNDPMLLYNKMTL |
| P08049 | EDRNDPMLLYNKMTLAQIQNNFSLEINGKPFSWSNFTNEIM |
| P08049 | LAQIQNNFSLEINGKPFSWSNFTNEIMSTVNINIPNEEDVV |
| P08049 | TQQSANNFKEQSQCMVYQYGNFSWDLAGGQHLNGINTLGEN |

| | |
|---|---|
| P10769 | VIEPECREEFHRPVRAGDRSNVTQLDGPAGALLLRLMQLAG |
| P01180 | EPECREGVGFPRRVRANDRSNATLLDGPSGALLLRLVQLAG |
| P01185 | VTEPECREGFHRRARASDRSNATQLDGPAGALLLRLVQLAG |
| P01181 | EPECREGIGFPRRVXASDRSNATLLDGPSGALLLRLVQLAA |
| P01183 | EPECREGASFLRRARASDRSNATLLDGPSGALLLRLVQLAG |
| P01186 | VAEPECREGFFRLTRAREQSNATQLDGPARELLLRLVQLAG |
| Q99519 | VASTMLVWSKDDGVSWSTPRNLSLDIGTEVFAPGPGSGIQK |
| Q99519 | VVTSSGIVFFSNPAHPEFRVNLTLRWSFSNGTSWRKETVQL |
| Q99519 | FSNPAHPEFRVNLTLRWSFSNGTSWRKETVQLWPGPSGYSS |
| O94856 | GDLPSDKAKFENFNKALRITNVSEEDSGEYFCLASNKMGSI |
| O94856 | AGDTIIFRDTQISSRAVYQCNTSNEHGYLLANAFVSVLDVP |
| O94856 | LDVPPRMLSPRNQLIRVILYNRTRLDCPFFGSPIPTLRWFK |
| O94856 | RWFKNGQGSNLDGGNYHVYENGSLEIKMIRKEDQGIYTCVA |
| O94856 | GDVKGEGTRKNNMEITWTPMNATSAFGPNLRYIVKWRRRET |
| O94856 | GPNLRYIVKWRRRETREAWNNVTVWGSRYVVGQTPVYVPYE |
| O94856 | DHPEHPNGIMIGYTLKYVAFNGTKVGKQIVENFSPNQTKFT |
| O94856 | KYVAFNGTKVGKQIVENFSPNQTKFTVQRTDPVSRYRFTLS |
| P11672 | VQKKTEGSFTMYSTIYELQENNSYNVTSILVRDQDQGCRYW |
| P11672 | TEGSFTMYSTIYELQENNSYNVTSILVRDQDQGCRYWIRTF |
| P80188 | DKDPQKMYATIYELKEDKSYNVTSVLFRKKKCDYWIRTFVP |
| Q96E22 | CMAVGISYISVYDHQGIFKRNNSRLMDEILKQQQELLGLDC |
| Q96E22 | LGFLPWHIRLTEIVSLPSHLNISYEDFFSALRQYAACEQRL |
| Q04121 | FVNWIYRVKTIRSMSGITGENISVPDEIPYNYQMMTFWAGL |
| Q04121 | EEDTSDDEFDIEAPRAINLLNGSSIQTDLGPYSDNNSPDIS |
| Q04121 | NSPDISIDQFAVSSNKNLPNNISTTGGNTFGGLNETENTSP |
| Q04121 | SNKNLPNNISTTGGNTFGGLNETENTSPNPARSSMDKRNLR |
| Q23316 | SDQVFRTLFIGEGNACYRTFNKTHEFGCQANRENENGLIVR |
| Q23316 | AITRDGLMKIDWRIQMVFIDNSTDLEIIEKCYSMFNKPKED |
| Q23316 | GAMHSDNIFAFPTPIPTSPTNETIITSKYMMVTARMDSFGM |
| Q23316 | SVLAAARSMGTQIEKWQKASNTSNRNVFFAFFNGESLDYIG |
| Q23316 | IGVAKGRKYYVHVDGERYQQNKTQTDRVIDRIERGLRSHAF |
| Q23316 | GQFQAFQMYTYTWQPNPYTGNFSCLKSAIVKKVMVSPAVDS |
| P57716 | VVLAGLCGGNSVERKIYIPLNKTAPCVRLLNATHQIGCQSS |
| P57716 | SVERKIYIPLNKTAPCVRLLNATHQIGCQSSISGDTGVIHV |
| P57716 | EKLKGTTSRIAGLAVTLAKPNSTSSFSPSVQCPNDGFGIYS |
| P57716 | LGNGLAYEDFSFPIFLLEDENETKVIKQCYQDHNLGQNGSA |
| P57716 | EDENETKVIKQCYQDHNLGQNGSAPSFPLCAMQLFSHMHAV |
| P57716 | PEIVCDPLSDYNVWSMLKPINTSVGLEPDVRVVVAATRLDS |
| P57716 | QVALRTSLDLWMHTDPMSQKNESVKNQVEDLLATLEKSGAG |
| P57716 | RLAQSQALPPSSLQRFLRARNISGVVLADHSGSFHNRYYQS |
| P57716 | HSGSFHNRYYQSIYDTAENINVTYPEWQSPEEDLNFVTDTA |

| | |
|---|---|
| P57716 | ALANVATVLARALYELAGGTNFSSSIQADPQTVTRLLYGFL |
| P57716 | SIQADPQTVTRLLYGFLVRANNSWFQSILKHDLRSYLDDRP |
| P57716 | IQADPQTVTRLLYGFLVRANNSWFQSILKHDLRSYLDDRPL |
| P57716 | LRSYLDDRPLQHYIAVSSPTNTTYVVQYALANLTGKATNLT |
| P57716 | HYIAVSSPTNTTYVVQYALANLTGKATNLTREQCQDPSKVP |
| P57716 | PTNTTYVVQYALANLTGKATNLTREQCQDPSKVPNESKDLY |
| P57716 | LTGKATNLTREQCQDPSKVPNESKDLYEYSWVQGPWNSNRT |
| P57716 | VPNESKDLYEYSWVQGPWNSNRTERLPQCVRSTVRLARALS |
| Q92542 | VLLAGLCRGNSVERKIYIPLNKTAPCVRLLNATHQIGCQSS |
| Q92542 | SVERKIYIPLNKTAPCVRLLNATHQIGCQSSISGDTGVIHV |
| Q92542 | LGNGLAYEDFSFPIFLLEDENETKVIKQCYQDHNLSQNGSA |
| Q92542 | IFLLEDENETKVIKQCYQDHNLSQNGSAPTFPLCAMQLFSH |
| Q92542 | EDENETKVIKQCYQDHNLSQNGSAPTFPLCAMQLFSHMHAV |
| Q92542 | PEIVCDPLSDYNVWSMLKPINTTGTLKPDDRVVVAATRLDS |
| Q92542 | QVALRTSLELWMHTDPVSQKNESVRNQVEDLLATLEKSGAG |
| Q92542 | LLATLEKSGAGVPAVILRRPNQSQPLPPSSLQRFLRARNIS |
| Q92542 | RPNQSQPLPPSSLQRFLRARNISGVVLADHSGAFHNKYYQS |
| Q92542 | HSGAFHNKYYQSIYDTAENINVSYPEWLSPEEDLNFVTDTA |
| Q92542 | ALADVATVLGRALYELAGGTNFSDTVQADPQTVTRLLYGFL |
| Q92542 | TVQADPQTVTRLLYGFLIKANNSWFQSILRQDLRSYLGDGP |
| Q92542 | LRSYLGDGPLQHYIAVSSPTNTTYVVQYALANLTGTVVNLT |
| Q92542 | HYIAVSSPTNTTYVVQYALANLTGTVVNLTREQCQDPSKVP |
| Q92542 | PTNTTYVVQYALANLTGTVVNLTREQCQDPSKVPSENKDLY |
| Q92542 | VPSENKDLYEYSWVQGPLHSNETDRLPRCVRSTARLARALS |
| P10493 | SSSPAEEGKRNTFQAVLASSNSSSYAIFLYPEDGLQFFTTF |
| P10493 | CSVHAECRDYATGFCCRCVANYTGNGRQCVAEGSPQRVNGK |
| Q14112 | PEVDRDSLAPSWETPPPYPENGSIQPYPDGGPVPSEMDVPP |
| Q14112 | PENYLSIKTNIQGQVPYVSANFTAHISPYKELYHYSDSTVT |
| Q14112 | SDSTVTSTSSRDYSLTFGAINQTWSYRIHQNITYQVCRHAP |
| Q14112 | RDYSLTFGAINQTWSYRIHQNITYQVCRHAPRHPSFPTTQQ |
| Q14112 | PSVGTFLLYTQGQQIGYLPLNGTRLQKDAAKTLLSLHGSII |
| O88322 | PENYLSIKTNIEGQVPFIPANFTAHITPYKEFYHYRDSVVT |
| O88322 | RDSVVTSSSSRSFSLTSGSINQTWSYHIDQNITYQACRHAP |
| O88322 | RSFSLTSGSINQTWSYHIDQNITYQACRHAPRHLAIPATQQ |
| O88322 | PSVGTFLLYAQGQQIGHLPLNGSRLQKDAARTLLSLHGSIV |
| Q9XTG1 | LPVMVIVHGEEYGWGTGNAFNGTTLAAYGHIIVVTLNYRLG |
| Q9XTG1 | SQNPQQYFMQLAEELACAPKNRTSSFNDNVDTIVRCMQVHS |
| Q9XTG1 | SSFNDNVDTIVRCMQVHSSENITKAVLKIDVPTFLSGFAPI |
| Q99K10 | PTGEHRFQPPEPPSPWSDIRNATQFAPVCPQNIIDGRLPEV |
| Q99K10 | GGSCVNLLTLSHYSEGNRWSNSTKGLFQRAIAQSGTALSSW |
| Q99K10 | WAVSFQPAKYARILATKVGCNVSDTVELVECLQKKPYKELV |

| | |
|---|---|
| Q99K10 | DEVPYVLGIPMIGPTELFPCNFSKNDVMLSAVVMTYWTNFA |
| Q8N0W4 | PTGERRFQPPEPPSSWTGIRNTTQFAAVCPQHLDERSLLHD |
| Q8N0W4 | DEVPYVFGIPMIGPTELFSCNFSKNDVMLSAVVMTYWTNFA |
| Q62765 | PTGEHRFQPPEPPSPWSDIRNATQFAPVCPQNIIDGRLPEV |
| Q62765 | GGSCVNLLTLSHYSEGNRWSNSTKGLFQRAIAQSGTALSSW |
| Q62765 | WAVSFQPAKYARILATKVGCNVSDTVELVECLQKKPYKELV |
| Q62765 | DEVPYVLGIPMIGPTELFPCNFSKNDVMLSAVVMTYWTNFA |
| Q69ZK9 | PLGARRFQPPEAPASWPGVRNATTLPPACPQNLHGALPAIM |
| Q69ZK9 | AIMLPVWFTDNLEAAATYVQNQSEDCLYLNLYVPTEDGPLT |
| Q69ZK9 | DELPYVFGVPMVGATDLFPCNFSKNDVMLSAVVMTYWTNFA |
| Q24418 | YLMYASTEDAQVIFRDAGEYNMTGEGHVWIVTEQALFSNNT |
| Q24418 | KGHIRDSVYVLASAIKEMISNETIAEAPKDCGDSAVNWESG |
| Q24418 | GDSAVNWESGKRLFQYLKSRNITGETGQVAFDDNGDRIYAG |
| Q24418 | KHVVGKFSYDSMRAKMRMRINDSEIIWPGKQRRKPEGIMIP |
| Q24418 | RRMGDDEFRCEPDERPCPLFNNSDATANEFCCRGYCIDLLI |
| Q24418 | NEFCCRGYCIDLLIELSKRINFTYDLALSPDGQFGHYILRN |
| Q24418 | NFTYDLALSPDGQFGHYILRNNTGAMTLRKEWTGLIGELVN |
| Q24418 | ERPKTKLSGINDARLRNTMENLTCATVKGSSVDMYFRRQVE |
| Q00960 | HEKDDFHHLSVVPRVELVAMNETDPKSIITRICDLMSDRKI |
| Q00960 | CYNTHEKRIYQSNMLNRYLINVTFEGRNLSFSEDGYQMHPK |
| Q00960 | RIYQSNMLNRYLINVTFEGRNLSFSEDGYQMHPKLVIILLN |
| Q00960 | PLSGTCMRNTVPCQKRIISENKTDEEPGYIKKCCKGFCIDI |
| Q00960 | SVKFTYDLYLVTNGKHGKKINGTWNGMIGEVVMKRAYMAVG |
| Q00960 | VVDFSVPFIETGISVMVSRSNGTVSPSAFLEPFSADVWVMM |
| Q00960 | DKKFQRPNDFSPPFRFGTVPNGSTERNIRNNYAEMHAYMGK |
| Q8IW45 | GPMDSDDSHGSVLRLSQALGNVTVVQKGERDILSNGQQVLV |
| Q8IW45 | LSGSLGVLVHWALLAGPQKTNGSSPLLVAAFGACSLTRQCN |
| Q99784 | STMAMITNWMSQTLPSLVGLNTTKLSAAGGGTLDRSTGVLP |
| Q99784 | TMCSRDARTKQLRQLLEKVQNMSQSIEVLDRRTQRDLQYVE |
| Q99784 | VLEEYKADAKLVLQFKEEVQNLTSVLNELQEEIGAYDYDEL |
| Q99784 | YHNNRFVREYKSMVDFMNTDNFTSHRLPHPWSGTGQVVYNG |
| Q99784 | DNFTSHRLPHPWSGTGQVVYNGSIYFNKFQSHIIIRFDLKT |
| Q99784 | AGNIVVSRLDPVSLQTLQTWNTSYPKRSAGEAFIICGTLYV |
| Q99784 | TLYVTNGYSGGTKVHYAYQTNASTYEYIDIPFQNKYSHISM |
| O88998 | STMAMITNWMSQTLPSLVGLNTTRLSAASGGTLDRSTGVLP |
| O88998 | TMCSRDARTKQLRQLLEKVQNMSQSIEVLDRRTQRDLQYVE |
| O88998 | VLEEYKADAKLVLQFKEEVQNLTSVLNELQEEIGAYDYDEL |
| O88998 | YHNNRFVREYKSMVDFMNTDNFTSHRLPHPWSGTGQVVYNG |
| O88998 | DNFTSHRLPHPWSGTGQVVYNGSIYFNKFQSHIIIRFDLKT |
| O88998 | AGNIVISKLDPVSLQILQTWNTSYPKRSAGEAFIICGTLYV |
| O88998 | TLYVTNGYSGGTKVHYAYQTNASTYEYIDIPFQNKYSHISM |

| | |
|---|---|
| Q15155 | HGSEDIVVGCGGFVKSDVEINYSLIEIKLYTKHGTLKYQTD |
| Q15155 | TVRVTNSNANAASPLIVAGYNVSGSVRSDGEPMKGVKFLLF |
| Q15155 | HAITLEFYQDGNGRENVGIYNLSKGVNRFCLSKPGVYKVTP |
| P46531 | GPRCSQPGETCLNGGKCEAANGTEACVCGGAFVGPRCQDPN |
| P46531 | GTFCEEDINECASDPCRNGANCTDCVDSYTCTCPAGFSGIH |
| P46531 | TCTDYLGGYSCKCVAGYHGVNCSEEIDECLSHPCQNGGTCL |
| P46531 | NVDDCNPPVDPVSRSPKCFNNGTCVDQVGGYSCTCPPGFVG |
| P46531 | NHACGWDGGDCSLNFNDPWKNCTQSLQCWKYFSDGHCDSQC |
| P46531 | ERLAAGTLVVVVLMPPEQLRNSSFHFLRELSRVLHTNVVFK |
| Q01705 | VDINECVKSPCRHGASCQNTNGSYRCLCQAGYTGRNCESDI |
| Q01705 | GAFCEEDINECASNPCQNGANCTDCVDSYTCTCPVGFNGIH |
| Q01705 | TCTDYLGGFSCKCVAGYHGSNCSEEINECLSQPCQNGGTCI |
| Q01705 | NVDDCHPPLDPASRSPKCFNNGTCVDQVGGYTCTCPPGFVG |
| Q01705 | NHACGWDGGDCSLNFNDPWKNCTQSLQCWKYFSDGHCDSQC |
| Q01705 | ERLAAGTLVLVVLLPPDQLRNNSFHFLRELSHVLHTNVVFK |
| O35516 | LQCRGGQEPCVNEGTCVTYHNGTGFCRCPEGFLGEYCQHRD |
| O35516 | VGFTGKQCQWTDACLSHPCENGSTCTSVASQFSCKCPAGLT |
| O35516 | HHPSCYSQVNECLSNPCIHGNCTGGLSGYKCLCDAGWVGVN |
| O35516 | KARPHCLCPPGWDGAYCDVLNVSCKAAALQKGVPVEHLCQH |
| O35516 | SHACQWDGGDCSLTMEDPWANCTSTLRCWEYINNQCDEQCN |
| Q9VUX3 | MHSASGSGDHSRSLKRANLANTSITCNDGSHAGFYLRKHPS |
| Q9VUX3 | TPQQWNYIHEMGGALRSSLDNVSAVFAPSCIGHGVLFKRDW |
| Q9VUX3 | QPAAGVFLEASAPQKTRSSNNASAGTKSKKRHRVPRVPEKC |
| Q9VUX3 | PRVPEKCGLRLLERCSWPQCNHSCPTLTNPMTGEEMRFLEL |
| Q9Y5S8 | SSLSHDEKKGGSWLNPIQSRNTTVEYVTFTSIAGLTGVIMT |
| Q9Y5S8 | LGLGIHGIGGIVRGQTEESMNESHPRKCAESFEMWDDRDSH |
| Q19127 | CIMRGLCQKHTENAYGPCVTNDTNVEPTAFDKTHPAYEKMV |
| Q19127 | FLLYDPIKTPPSDRSTYMNVNFTGCDKSARVGWPACSTSEC |
| Q19127 | GPVFHKDIFEELFDILNAIKNISTQDSDGRTITLDDVCYRP |
| Q19127 | GLSCMGTYGGPSAPNMVFGKNSTNHQAANSIMMTILVTQRT |
| Q19127 | KSPCCKVYVHDPNTFCSTNRNKSALDDKACRTCMDFDYVAN |
| Q19127 | SRGRIQASQFMTFHKKLSISNSSDFIKAMDTARMVSRRLER |
| O15118 | PLPKDGYDLVQELCPGFFFGNVSLCCDVRQLQTLKDNLQLP |
| O15118 | YNLLNLFCELTCSPRQSQFLNVTATEDYVDPVTNQTKTNVK |
| O15118 | PRQSQFLNVTATEDYVDPVTNQTKTNVKELQYYVGQSFANA |
| O15118 | KTNVKELQYYVGQSFANAMYNACRDVEAPSSNDKALGLLCG |
| O15118 | APSSNDKALGLLCGKDADACNATNWIEYMFNKDNGQAPFTI |
| O15118 | PFTITPVFSDFPVHGMEPMNNATKGCDESVDEVTAPCSCQD |
| O15118 | GPPLDIQILHQVLDLQIAIENITASYDNETVTLQDICLAPL |
| O15118 | ILHQVLDLQIAIENITASYDNETVTLQDICLAPLSPYNTNC |
| O15118 | DNETVTLQDICLAPLSPYNTNCTILSVLNYFQNSHSVLDHK |

| | |
|---|---|
| O15118 | FVYADYHTHFLYCVRAPASLNDTSLLHDPCLGTFGGPVFPW |
| O15118 | SWIDDYFDWVKPQSSCCRVDNITDQFCNASVVDPACVRCRP |
| O15118 | DWVKPQSSCCRVDNITDQFCNASVVDPACVRCRPLTPEGKQ |
| O15118 | VLQTSADFIDALKKARLIASNVTETMGINGSAYRVFPYSVF |
| O15118 | IDALKKARLIASNVTETMGINGSAYRVFPYSVFYVFYEQYL |
| P61916 | NVSPCPTQPCQLSKGQSYSVNVTFTSNIQSKSSKAVVHGIL |
| Q9UHC9 | AFYDECGKNPELSGSLMTLSNVSCLSNTPARKITGDHLILL |
| Q9UHC9 | RCPACSDNFVNLHCHNTCSPNQSLFINVTRVAQLGAGQLPA |
| Q9UHC9 | DNFVNLHCHNTCSPNQSLFINVTRVAQLGAGQLPAVVAYEA |
| Q9UHC9 | EPGQAVGSGIQPLNEGVARCNESQGDDVATCSCQDCAASCP |
| Q9UHC9 | FHDQHFGPFFRTNQVILTAPNRSSYRYDSLLLGPKNFSGIL |
| Q9UHC9 | ILTAPNRSSYRYDSLLLGPKNFSGILDLDLLLELLELQERL |
| Q9UHC9 | LLELQERLRHLQVWSPEAQRNISLQDICYAPLNPDNTSLYD |
| Q9UHC9 | PEAQRNISLQDICYAPLNPDNTSLYDCCINSLLQYFQNNRT |
| Q9UHC9 | PDNTSLYDCCINSLLQYFQNNRTLLLLTANQTLMGQTSQVD |
| Q9UHC9 | CINSLLQYFQNNRTLLLLTANQTLMGQTSQVDWKDHFLYCA |
| Q9UHC9 | RMAGMFQVTFMAERSLEDEINRTTAEDLPIFATSYIVIFLY |
| Q22523 | VLDGYKTFTDGRPIYPLPFENSSVAVDRSAKNHNDVVCTSR |
| Q22523 | EGDKWQEWSHSWEEQIALVLNLTGVNAVILEMAVTHDPSNS |
| Q22523 | LNLTGVNAVILEMAVTHDPSNSSRSVIELDQPKWGAGSRYP |
| Q22523 | KWGAGSRYPYLSGANDPMLRNYTTLMKMTAVALGADPAIAE |
| Q22523 | DPAIAEKEMNEAMEFELKLVNFSADDMVRRDPERGNNRFEL |
| Q22523 | FINFEKYLKTVFKELVALSPNHTVIVREIDYFVGIQHVLQS |
| Q22523 | QGFSPFLPPSAREPFYQFKANQTGMFNSPPPDRWEDCVTLS |
| Q22523 | RAISKANMIEYKSGFPMVLFNDTWMEKNWGMIIKPREYLLH |
| Q22523 | NIADNGGVKTAFNAYKAWKSNTTGISEPALPGFQNFTSQQM |
| Q22523 | YKAWKSNTTGISEPALPGFQNFTSQQMFFLAYANNWCSLVR |
| O16796 | PEVCSTPGCVRAATHFLNAMNTSVDPCDDFFEFACGQWNDQ |
| O16796 | EVVTESESINMARATYRSCMNKTQLDELMTGPLFETLTELG |
| O16796 | TLTELGEWPLLQENWDKTKFNFTSLLVNSRRDYGVDVFFQL |
| O16796 | SRRDYGVDVFFQLYIYADSKNTSRNTLFIDQSTLALGRGTR |
| O16796 | TLFIDQSTLALGRGTRDYYLNTTLFSSHMTAYRKYLRQIAH |
| O16796 | HMTAYRKYLRQIAHLLKTDGNLTRSESEMNADIEKIIDFEI |
| O16796 | KIIDFEIELAKIIVAEDERRNNTRLYNKRQIQDLYNLLPQV |
| O16796 | VDWVPFFQSIAPSDLTHLFHNETEIIICEIEYLQHVSELIE |
| O16796 | SESSLNSFDTRRRCIVEQYGNYTVPKTNFRVNGKLTQGENI |
| Q9Y639 | VTSEEVIIRDSPVLPVTLQCNLTSSSHTLTYSYWTKNGVEL |
| Q9Y639 | HTLTYSYWTKNGVELSATRKNASNMEYRINKPRAEDSGEYH |
| Q9Y639 | RAEDSGEYHCVYHFVSAPKANATIEVKAAPDITGHKRSENK |
| Q9Y639 | YPHPDWIWRKKENGMPMDIVNTSGRFFIINKENYTELNIVN |
| Q9Y639 | NGMPMDIVNTSGRFFIINKENYTELNIVNLQITEDPGEYEC |

| | |
|---|---|
| Q9Y639 | YTELNIVNLQITEDPGEYECNATNAIGSASVVTVLRVRSHL |
| P97300 | IVTSEEVIIRESLLPVTLQCNLTSSSHTLMYSYWTRNGVEL |
| P97300 | TLQCNLTSSSHTLMYSYWTRNGVELTATRKNASNMEYRINK |
| P97300 | HTLMYSYWTRNGVELTATRKNASNMEYRINKPRAEDSGEYH |
| P97300 | MYSYWTRNGVELTATRKNASNMEYRINKPRAEDSGEYHCVY |
| P97300 | RAEDSGEYHCVYHFVSAPKANATIEVKAAPDITGHKRSENK |
| P97300 | MMYCKSVGYPHPEWIWRKKENGVFEEISNSSGRFFITNKEN |
| P97300 | YPHPEWIWRKKENGVFEEISNSSGRFFITNKENYTELSIVN |
| P97300 | NGVFEEISNSSGRFFITNKENYTELSIVNLQITEDPGEYEC |
| P97300 | YTELSIVNLQITEDPGEYECNATNSIGSASVSTVLRVRSHL |
| P97546 | IVTSEEVIIRDSLLPVTLQCNLTSSSHTLMYSYWTKNGVEL |
| P97546 | HTLMYSYWTKNGVELTATRKNASNMEYRINKPRAEDSGEYH |
| P97546 | RAEDSGEYHCVYHFVSAPKANATIEVKAAPDITGHKRSENK |
| P97546 | YPHPEWMWRKKENGVFEEISNSSGRFFIINKENYTELNIVN |
| P97546 | NGVFEEISNSSGRFFIINKENYTELNIVNLQITEDPGEYEC |
| P97546 | YTELNIVNLQITEDPGEYECNATNSIGSASVSTVLRVRSHL |
| Q68D85 | EGDLKVEMMAGGTQITPLNDNVTIFCNIFYSQPLNITSMGI |
| Q68D85 | ITPLNDNVTIFCNIFYSQPLNITSMGITWFWKSLTFDKEVK |
| Q68D85 | MKENEDKYMCESSGFYPEAINITWEKQTQKFPHPIEISEDV |
| Q68D85 | IEISEDVITGPTIKNMDGTFNVTSCLKLNSSQEDPGTVYQC |
| Q68D85 | TGPTIKNMDGTFNVTSCLKLNSSQEDPGTVYQCVVRHASLH |
| Q68D85 | PGTVYQCVVRHASLHTPLRSNFTLTAARHSLSETEKTDNFS |
| Q68D85 | RSNFTLTAARHSLSETEKTDNFSIHWWPISFIGVGLVLLIV |
| Q8CJ26 | QGQDGDREGMWVGAGGALAPNTSSLFPPEPPGASSNIIPVY |
| Q9IGQ6 | NISNTNVVAGQDATSVILTGNSSLCPISGWAIYSKDNGIRI |
| Q9IGQ6 | HLECRTFFLTQGALLNDKHSNGTVKDRSPYRTLMSCPVGEA |
| Q9IGQ6 | TIKSWRNNILRTQESECACVNGSCFTIMTDGPSNGQASYKI |
| Q6XV27 | LNIGLHYKVGDTPNVNIPNVNGTNSTTTIINNNTQNNFTNI |
| Q6XV27 | GLHYKVGDTPNVNIPNVNGTNSTTTIINNNTQNNFTNITNI |
| Q6XV27 | TPNVNIPNVNGTNSTTTIINNNTQNNFTNITNIIQSKGGER |
| Q6XV27 | IPNVNGTNSTTTIINNNTQNNFTNITNIIQSKGGERTFLNL |
| Q6XV27 | VNGTNSTTTIINNNTQNNFTNITNIIQSKGGERTFLNLTKP |
| Q6XV27 | NNFTNITNIIQSKGGERTFLNLTKPLCEVNSWHILSKDNAI |
| Q6XV27 | PQGCRMFALSQGTTLRGRHANGTIHDRSPFRALISWEMGQA |
| Q6XV27 | STSCHDGMSRMSICMSGPNNNASAVVWYGGRPITEIPSWAG |
| Q6XV27 | NAETDIQSGPISNQVIVNNQNWSGYSGAFIDYWANKECFNP |
| P06820 | ECDSPASNQVMPCEPIIIERNITEIVYLNNTTIEKEICPKV |
| P06820 | QVMPCEPIIIERNITEIVYLNNTTIEKEICPKVVEYRNWSK |
| P06820 | VMPCEPIIIERNITEIVYLNNTTIEKEICPKVVEYRNWSKP |
| P06820 | VYLNNTTIEKEICPKVVEYRNWSKPQCQITGFAPFSKDNSI |
| P06820 | PVKCYQFALGQGTTLDNKHSNDTVHDRIPHRTLLMNELGVP |

| | |
|---|---|
| P06820 | SSSCHDGKAWLHVCITGDDKNATASFIYDGRLVDSIGSWSQ |
| P06820 | SIGSWSQNILRTQESECVCINGTCTVVMTDGSASGRADTRI |
| P03472 | CSHSQPEATNASQTIINNYYNDTNITQISNTNIQVEERAIR |
| P03472 | SQPEATNASQTIINNYYNDTNITQISNTNIQVEERAIRDFN |
| P03472 | ITQISNTNIQVEERAIRDFNNLTKGLCTINSWHIYGKDNAV |
| P03472 | PDECRFYALSQGTTIRGKHSNGTIHDRSQYRALISWPLSSP |
| P03472 | STSCHDGKTRMSICISGPNNNASAVIWYNRRPVTEINTWAR |
| P05803 | VLIGIVNLGLNIGLHLKPSCNCSRSQPEATNASQTIINNYY |
| P05803 | NIGLHLKPSCNCSRSQPEATNASQTIINNYYNETNITQISN |
| P05803 | CSRSQPEATNASQTIINNYYNETNITQISNTNIQVEERASR |
| P05803 | SQPEATNASQTIINNYYNETNITQISNTNIQVEERASREFN |
| P05803 | ITQISNTNIQVEERASREFNNLTKGLCTINSWHIYGKDNAV |
| P05803 | PDECRFYALSQGTTIRGKHSNGTIHDRSQYRDLISWPLSSP |
| P05803 | STSCHDGRARMSICISGPNNNASAVIWYNRRPVTEINTWAR |
| P27907 | DILLKFSSKITAPTMTLDCANASNVQAVNRSATKEMTFLLP |
| P27907 | KITAPTMTLDCANASNVQAVNRSATKEMTFLLPEPEWTYPR |
| P27907 | EIFPTGRVEHTEECTCGFASNKTIECACRDNSYTAKRPFVK |
| Q92823 | NIVIQCEAKGKPPPSFSWTRNGTHFDIDKDPLVTMKPGTGT |
| Q92823 | YFSNVLPEDTREDYICYARFNHTQTIQQKQPISVKVISVDE |
| Q92823 | TQTIQQKQPISVKVISVDELNDTIAANLSDTEFYGAKSSRE |
| Q92823 | KQPISVKVISVDELNDTIAANLSDTEFYGAKSSRERPPTFL |
| Q92823 | EFYGAKSSRERPPTFLTPEGNASNKEELRGNVLSLECIAEG |
| Q92823 | AEGLPTPIIYWAKEDGMLPKNRTVYKNFEKTLQIIHVSEAD |
| Q92823 | DGDTIIFSNVQERSSAVYQCNASNEYGYLLANAFVNVLAEP |
| Q92823 | EWFKGAKGSALHEDIYVLHENGTLEIPVAQKDSTGTYTCVA |
| Q92823 | DHLVVADVSDDDSGTYTCVANTTLDSVSASAVLSVVAPTPT |
| Q92823 | HQTEVSGTQTTAQLKLSPYVNYSFRVMAVNSIGKSLPSEAS |
| Q92823 | YKVSWRQKDGDDEWTSVVVANVSKYIVSGTPTFVPYLIKVQ |
| Q92823 | GHSGEDLPMVAPGNVRVNVVNSTLAEVHWDPVPLKSIRGHL |
| Q92823 | DPPSHPNGILTEYTLKYQPINSTHELGPLVDLKIPANKTRW |
| Q92823 | YQPINSTHELGPLVDLKIPANKTRWTLKNLNFSTRYKFYFY |
| Q92823 | GPLVDLKIPANKTRWTLKNLNFSTRYKFYFYAQTSAGSGSQ |
| Q92823 | GILPPDVGAGKVQAVNPRISNLTAAAAETYANISWEYEGPE |
| Q92823 | VQAVNPRISNLTAAAAETYANISWEYEGPEHVNFYVEYGVA |
| Q92823 | NFYVEYGVAGSKEEWRKEIVNGSRSFFGLKGLMPGTAYKVR |
| Q09225 | YFGNMMSGPPDKHCYDKTVKNVTDDGFCFALFPVLKFGVCM |
| P20241 | PSPTVNWMIQESIDGSIKSINNSRMTLDPEGNLWFSNVTRE |
| P20241 | IKSINNSRMTLDPEGNLWFSNVTREDASSDFYYACSATSVF |
| P20241 | TDNTIRIINLVKGDTGNYGCNATNSLGYVYKDVYLNVQAEP |
| P20241 | TIRIINLVKGDTGNYGCNATNSLGYVYKDVYLNVQAEPPTI |
| P20241 | QAEPPTISEAPAAVSTVDGRNVTIKCRVNGSPKPLVKWLRA |

| | |
|---|---|
| P20241 | IHWEQQGDNRSPILHYTIQFNTSFTPASWDAAYEKVPNTDS |
| P20241 | AYEKVPNTDSSFVVQMSPWANYTFRVIAFNKIGASPPSAHS |
| P20241 | VAAEEVVGYSGEDRPLDAPTNFTMRQITSSTSGYMAWTPVS |
| P20241 | TESATQEIDTNTVEGPIMVANETVANAGWFIGMMLALAFII |
| O60462 | GAGFSLRYEIFKTGSEDCSKNFTSPNGTIESPGFPEKYPHN |
| O60462 | LRYEIFKTGSEDCSKNFTSPNGTIESPGFPEKYPHNLDCTF |
| O60462 | KSEETTTPYPTEEEATECGENCSFEDDKDLQLPSGFNCNFD |
| O60462 | IHEREGYEDEIDDEYEVDWSNSSSATSGSGAPSTDKEKSWL |
| O14786 | HGAGFSIRYEIFKRGPECSQNYTTPSGVIKSPGFPEKYPNS |
| O14786 | GILSMVFYTDSAIAKEGFSANYSVLQSSVSEDFKCMEALGM |
| O14786 | GMESGEIHSDQITASSQYSTNWSAERSRLNYPENGWTPGED |
| O14786 | GGKHRENKVFMRKFKIGYSNNGSDWKMIMDDSKRKAKSFEG |
| O14786 | IKIDETGSTPGYEGEGEGDKNISRKPGNVLKTLDPILITII |
| P97333 | HGAGFSIRYEIFKRGPECSQNYTAPTGVIKSPGFPEKYPNS |
| P97333 | GVLSMVFYTDSAIAKEGFSANYSVLQSSISEDFKCMEALGM |
| P97333 | GMESGEIHSDQITASSQYGTNWSVERSRLNYPENGWTPGED |
| P97333 | GGKHRENKVFMRKFKIAYSNNGSDWKTIMDDSKRKAKSFEG |
| P97333 | IKIDETGSTPGYEGEGEGDKNISRKPGNVLKTLDPILITII |
| Q28146 | TDTPVNDGAWHNVRIRRQFRNTTLFIDQVEAKWVEVKSKRR |
| Q28146 | TLASVREREPFKGWIRDVRVNSSLALPVDSGEVKLDDEPPN |
| Q28146 | ELDAGRVKLTVNLDCIRINCNSSKGPETLFAGYNLNDNEWH |
| Q28146 | SNAIINDGKYHVVRFTRSGGNATLQVDSWPVIERYPAGNND |
| Q94887 | RISMRVELYGCDYISENLYFNGTGLVRYDLRREPITSTKES |
| Q94887 | NRELYLGGVPNVQEGLIVQQNFSGCLENIYFNSTNFIRVMK |
| Q94887 | VQEGLIVQQNFSGCLENIYFNSTNFIRVMKDSTELGEGYLF |
| Q94887 | TFTTRSSFVRLKGYENSQRLNVSFYFRTYEETGVMLHHDFY |
| Q94887 | GSFEQSIMYDANQLQIEALLNRSHSCWQRLSYSCRSSRLFN |
| Q94887 | GLYGISTGCVGRCESNPCLNNGTCIERYDGYSCDCRWSAFK |
| Q94887 | NIRVGFTTTIPKGFLLGFSSNLTGEYLTIQISNSGHLRCVF |
| Q94887 | DIKASADAQFNNIQYMYIGKNESMTDGFVGCVSRVQFDDIY |
| Q96CW9 | GLATYWQSITWSRYPSPLEANITLSWNKTVELTDDVVMTFE |
| Q96CW9 | QSITWSRYPSPLEANITLSWNKTVELTDDVVMTFEYGRPTV |
| Q96CW9 | NLHANLCSMREGSLQCECEHNTTGPDCGKCKKNFRTRSWRA |
| Q96CW9 | SCKHNTRGQHCQHCRLGYYRNGSAELDDENVCIECNCNQIG |
| Q96CW9 | DENVCIECNCNQIGSVHDRCNETGFCECREGAAGPKCDDCL |
| P04629 | GLRCTRDGALDSLHHLPGAENLTELYIENQQHLQHLELRDL |
| P04629 | NQQHLQHLELRDLRGLGELRNLTIVKSGLRFVAPDAFHFTP |
| P04629 | SGLRFVAPDAFHFTPRLSRLNLSFNALESLSWKTVQGLSLQ |
| P04629 | GGVPEQKLQCHGQGPLAHMPNASCGVPTLKVQVPNASVDVG |
| P04629 | PLAHMPNASCGVPTLKVQVPNASVDVGDDVLLRCQVEGRGL |
| P04629 | EQSATVMKSGGLPSLGLTLANVTSDLNRKNVTCWAENDVGR |

| | |
|---|---|
| P04629 | GGLPSLGLTLANVTSDLNRKNVTCWAENDVGRAEVSVQVNV |
| P04629 | KNVTCWAENDVGRAEVSVQVNVSFPASVQLHTAVEMHHWCI |
| P04629 | HWCIPFSVDGQPAPSLRWLFNGSVLNETSFIFTEFLEPAAN |
| P04629 | FSVDGQPAPSLRWLFNGSVLNETSFIFTEFLEPAANETVRH |
| P04629 | NGSVLNETSFIFTEFLEPAANETVRHGCLRLNQPTHVNNGN |
| P04629 | NETVRHGCLRLNQPTHVNNGNYTLLAANPFGQASASIMAAF |
| P04629 | NPFEFNPEDPIPVSFSPVDTNSTSGDPVEKKDETPFGVSVA |
| Q16620 | DPSPGIVAFPRLEPNSVDPENITEIFIANQKRLEIINEDDV |
| Q16620 | NQKRLEIINEDDVEAYVGLRNLTIVDSGLKFVAHKAFLKNS |
| Q16620 | SGLKFVAHKAFLKNSNLQHINFTRNKLTSLSRKHFRHLDLS |
| Q16620 | WIKTLQEAKSSPDTQDLYCLNESSKNIPLANLQIPNCGLPS |
| Q16620 | PLANLQIPNCGLPSANLAAPNLTVEEGKSITLSCSVAGDPV |
| Q16620 | AGDPVPNMYWDVGNLVSKHMNETSHTQGSLRITNISSDDSG |
| Q16620 | NLVSKHMNETSHTQGSLRITNISSDDSGKQISCVAENLVGE |
| Q16620 | SGKQISCVAENLVGEDQDSVNLTVHFAPTITFLESPTSDHH |
| Q16620 | FTVKGNPKPALQWFYNGAILNESKYICTKIHVTNHTEYHGC |
| Q16620 | FYNGAILNESKYICTKIHVTNHTEYHGCLQLDNPTHMNNGD |
| Q16620 | YPDVIYEDYGTAANDIGDTTNRSNEIPSTDVTDKTGREHLS |
| P81204 | VGFNRHYWAYLEGFMRDLTQNFTDVYVYTGPLFLPSAASTG |
| P24289 | CNVDYERDCGSSGCSISAIANYTQRVSDSSLSSENHAEALR |
| P24289 | IGDMTQPLHDEAYAVGGNKINVTFDGYHDNLHSDWDTYMPQ |
| P24289 | HALSDAESWAKTLVQNIESGNYTAQAIGWIKGDNISEPITT |
| P24289 | VQNIESGNYTAQAIGWIKGDNISEPITTATRWASDANALVC |
| P24504 | CNVDYERDCGSSGCSISAIANYTQRVSDSSLSSENHAEALR |
| P24504 | IGDMTQPLHDEAYAVGGNKINVTFDGYHDNLHSDWDTYMPQ |
| P24504 | HALSDAESWAKTLVQNIESGNYTAQATGWIKGDNISEPITT |
| P24504 | VQNIESGNYTAQATGWIKGDNISEPITTATRWASDANALVC |
| P24021 | CGVDYDRDCGSAGCSISAIQNYTNILLESPNGSEALNALKF |
| P24021 | AADANTYVCSTVLDDGLAYINSTDLSGEYYDKSQPVFEELI |
| F1NSM7 | CNTKHGFYIFQYIYSHLMQKNQTQVKKEEGDHQGTIHGHWL |
| F1NSM7 | ISPEDEVKIFIGRANIQVGENDSSVGSAGATSEANVIPTVV |
| Q9U539 | GNPPHTCHIPEGKEYLRPLTNDTQILSCKQYNETQINVFRA |
| Q9U539 | GKEYLRPLTNDTQILSCKQYNETQINVFRAFTSAPVDTYSD |
| Q9U539 | VDTYSDRISLVPCQNGWDYDNSTYLDSLVTEFNLVCDQQAW |
| Q8WR51 | FQCVSIQGDSGTLKDLLKHSNFSESTSIMVDRAETILHEHY |
| Q9Y2G5 | QDKHEYYRGWFWGYEETRGLNVSCLSVQGSASIVAPLLLRN |
| Q9Y2G5 | NVSCLSVQGSASIVAPLLLRNTSARSVMLDRAENLLHDHYG |
| Q9Y2G5 | SMVFARHLREVGDEFRSRHLNSTDDADRIPFQEDWMKMKVK |
| Q9H488 | GRFGNQADHFLGSLAFAKLLNRTLAVPPWIEYQHHKPPFTN |
| Q9H488 | CPMKEGNPFGPFWDQFHVSFNKSELFTGISFSASYREQWSQ |
| Q8WWZ8 | AMPTFCIPENHCGTHAPVWLNGSHPLEGDGIVQRQACASFN |

| | |
|---|---|
| Q8WWZ8 | GDGIVQRQACASFNGNCCLWNTTVEVKACPGGYYVYRLTKP |
| Q8WWZ8 | NAIEVNIPRELVGGLELFLTNTSCRGVSNGTHVNILFSLKT |
| Q8WWZ8 | RELVGGLELFLTNTSCRGVSNGTHVNILFSLKTCGTVVDVV |
| Q07081 | EMQKLEEQDIILDTYSEKIINLTRRVEYLEKLHPESLVEIS |
| Q07081 | MRVKPNGNSVQVETLYNEVKNMSKTVGQLETLDKNNVLQAK |
| Q07081 | QLNWKGNAYKSGAWGKDAAWNTTKKSLYWVAPLNTDGRVLE |
| Q07081 | LQMYKNPIDLPLSMLIKNKLNNTFAGQGAGVVVHNNNLYYN |
| Q07081 | VVGKVNVATFTVDNIWITTQNKSDASNAFMICGVLYVTRSL |
| P78380 | IMVLGMQLSQVSDLLTQEQANLTHQKKKLEGQISARQQAEE |
| P78380 | EQMELHHQNLNLQETLKRVANCSAPCPQDWIWHGENCYLFS |
| P41145 | IQIFRGEPGPTCAPSACLPPNSSAWFPGWAEPDSNGSAGSE |
| P41145 | SACLPPNSSAWFPGWAEPDSNGSAGSEDAQLEPAHISPAIP |
| P06002 | MSSIWCLAPAFGWSRYVPEGNLTSCGIDYLERDWNPRSYLI |
| Q9VNB5 | AWTTITFFGDSVKMVVDHETNSSIPVEIPRLPIKSFYPWNA |
| Q9VNB5 | TNSSIPVEIPRLPIKSFYPWNASHGMFYMISFAFQIYYVLF |
| Q9VCA2 | KLAGVFLLAKPDFRCALPYENGSIYELSPHLWNLSYPENER |
| Q9VCA2 | FRCALPYENGSIYELSPHLWNLSYPENERCSYYDVDYTEEY |
| Q9VCA2 | SYPENERCSYYDVDYTEEYLNGSIPRSSNETKTCSSYVYDR |
| Q9VCA2 | SYYDVDYTEEYLNGSIPRSSNETKTCSSYVYDRSKYLNSAV |
| Q8GWH3 | FLCYLPKEEKATSGWTSSQQNISTVMMETQQLVKLKTPDEL |
| Q8GWH3 | NKIVQEFFLGTFDPEATAAFNQTVSDASTDASQRYHSHVYT |
| Q8GWH3 | QTVSDASTDASQRYHSHVYTNGTTCDLTGSPREVEVRFVCA |
| O70458 | VPALTHEELNMIFQIEISRLNISNTIWVENYSTTVKREEAV |
| O70458 | NTIWVENYSTTVKREEAVRWNWTSDIPLECVKHFIRIRALV |
| O70458 | FIRIRALVDDTKSLPQSSWGNWSSWKEVNAKVSVEPDKSLI |
| O70458 | SVEPDKSLIFPKDKVLEEGSNVTICLMYGQNVYNVSCKLQD |
| O70458 | GPKRIFGTVLFVSKVLEEPKNVSCETRDFKTLDCSWEPGVD |
| O70458 | DCSWEPGVDTTLTWRKQRFQNYTLCESFSKRCEVSNYRNSY |
| O70458 | VSNYRNSYTWQITEGSQEMYNFTLTAENQLRKRSVNINFNL |
| O70458 | YNFTLTAENQLRKRSVNINFNLTHRVHPKAPQDVTLKIIGA |
| O70458 | NNYTLLCQVKLQYGEVIHEHNVSVHMSANYLFSDLDPDTKY |
| O70458 | NEAKPTESEHYCVWAPALSTNLSLDLQPYKIRITTNNSMGA |
| O70458 | NDSGHEEVKEKTIKGIKDAFNISWEPVSGDTMGYVVDWCAH |
| O70458 | DWCAHSQDQRCDLQWKNLGPNTTSTTITSDDFKPGVRYNFR |
| O70458 | NQPWERTLLPDNSVLCKYDINGSETKTLTVENLQPESLYEF |
| O70458 | QPESLYEFFVTPYTSAGPGPNETFTKVTTPDARSHMLLQII |
| P10451 | NAVSSEETNDFKQETLPSKSNESHDHMDDMDDEDDDDHVDS |
| P10451 | DDMDDEDDDDHVDSQDSIDSNDSDDVDDTDDSHQSDESHHS |
| Q7RTW8 | KDIIIDLGEIRERALQSPGVNRSLFLITLERCFQMLNSLEC |
| Q7RTW8 | FLQPDITERLPRDLREDAFKNLSAVFKDLYDKTSAHSQRAL |
| Q7RTW8 | SAHSQRALYSWMTGILQTSSNATDDSASWVSAEHLWVLGRY |

| | |
|---|---|
| Q7RTW8 | SFEEITKISPIEIGLFISYDNATKQLDMVYDITPELAQAFL |
| Q7RTW8 | TPELAQAFLERISSSNFNMRNTSTIHRQAHELWALEPFPKM |
| Q7RTW8 | RGFQAGVQKLKAELLDIAMENQTLNETLGSLSDAVVGLTYS |
| Q7RTW8 | AGVQKLKAELLDIAMENQTLNETLGSLSDAVVGLTYSQLES |
| Q7RTW8 | SQVIILSAKYLAHEKVLSFYNVSQMGALLAGVSTQAFCSMK |
| Q7RTW8 | IQGAFFKEVSLFDLRRQPGFNSTVLKDKELGRSQALFLYEL |
| Q7RTW8 | KMARTLPTKEFLWAVFQSVRNSSDKIPSYDPMPGCHGVVAP |
| Q7RTW8 | MPSYWREHHIVSLGRIALALNESELEQLDLSSIDTVASLSW |
| Q7RTW8 | SIQDLKSFHLVGLGATLCAINITEIPLIKISEFRVVVARIG |
| P01012 | VMEERKIKVYLPRMKMEEKYNLTSVLMAMGITDVFSSSANL |
| O01739 | YRDLAEPELFGPTSLFDLPRNTTTRGIHYTAYTSEGLRFCP |
| O01739 | PVIDHVYMGTVKQEGAFGPNNVTSADIQDITSRYVALQPSF |
| O01739 | FVGYRPGRKQVRVEKQIRETNGSKKFTVVHNYGHSGNGFTL |
| P81382 | SAGQLYEESLGKVVEELKRTNCSYILNKYDTYSTKEYLIKE |
| P81382 | IHGGKSTTDLPSRFIYYPNHNFTNGVGVIIAYGIGDDANFF |
| Q6STF1 | SAGQLYEESLGKVVEELKRTNCSYILNKYDTYSTKEYLLKE |
| Q6STF1 | IHGGKSTTDLPSRFIYYPNHNFTSGVGVIIAYGIGDDANFF |
| Q96RQ9 | EKCMQDPDYEQLLKVVTWGLNRTLKPQRVIVVGAGVAGLVA |
| Q96RQ9 | AMRMPSSHRILHKLCQGLGLNLTKFTQYDKNTWTEVHEVKL |
| Q96RQ9 | RKAMKKFERHTLLEYLLGEGNLSRPAVQLLGDVMSEDGFFY |
| P0DI84 | SAGQLYEESLGSAVKDLKRTNCSYILNKYDTYSTKEYLIKE |
| P45850 | KPMSEAGDDFLFSSKLTKAGNTSTPNGSAVTELDVAEWPGT |
| P45850 | AGDDFLFSSKLTKAGNTSTPNGSAVTELDVAEWPGTNTLGV |
| Q99571 | QETDSVVSSVTTKVKGVAVTNTSKLGFRIWDVADYVIPAQE |
| Q99571 | VIPAQEENSLFVMTNVILTMNQTQGLCPEIPDATTVCKSDA |
| Q99571 | TAGSAGTHSNGVSTGRCVAFNGSVKTCEVAAWCPVEDDTHV |
| Q99571 | WCPVEDDTHVPQPAFLKAAENFTLLVKNNIWYPKFNFSKRN |
| Q99571 | LKAAENFTLLVKNNIWYPKFNFSKRNILPNITTTYLKSCIY |
| Q99571 | LVKNNIWYPKFNFSKRNILPNITTTYLKSCIYDAKTDPFCP |
| Q99572 | WCPIEAVEEAPRPALLNSAENFTVLIKNNIDFPGHNYTTRN |
| Q99572 | LNSAENFTVLIKNNIDFPGHNYTTRNILPGLNITCTFHKTQ |
| Q99572 | KNNIDFPGHNYTTRNILPGLNITCTFHKTQNPQCPIFRLGD |
| Q99572 | TQNPQCPIFRLGDIFRETGDNFSDVAIQGGIMGIEIYWDCN |
| Q99572 | RWFHHCRPKYSFRRLDDKTTNVSLYPGYNFRYAKYYKENNV |
| Q9Z1M0 | KEPVISSVHTKVKGIAEVTENVTEGGVTKLGHSIFDTADYT |
| Q9Z1M0 | WCPTEEEKEAPRPALLRSAENFTVLIKNNIHFPGHNYTTRN |
| Q9Z1M0 | LRSAENFTVLIKNNIHFPGHNYTTRNILPTMNGSCTFHKTW |
| Q9Z1M0 | KNNIHFPGHNYTTRNILPTMNGSCTFHKTWDPQCSIFRLGD |
| Q9Z1M0 | TWDPQCSIFRLGDIFQEAGENFTEVAVQGGIMGIEIYWDCN |
| Q96FE7 | CLNWLDAQSGLASAPVSGAGNHSYCRNPDEDPRGPWCYVSG |
| Q32P28 | FEDFLPSHYNYLQFAYYNIGNYTQAVECAKTYLLFFPNDEV |

| | |
|---|---|
| Q32P28 | REGGPLLYEGISLTMNSKLLNGSQRVVMDGVISDHECQELQ |
| Q32P28 | KALKLGQEGKVPLQSAHLYYNVTEKVRRIMESYFRLDTPLY |
| P13674 | TEWSELENLVLKDMSDGFISNLTIQRQYFPNDEDQVGAAKA |
| P13674 | QRANGNLKYFEYIMAKEKDVNKSASDDQSDQKTTPKKKGVA |
| Q10576 | DTYRLDTKDLADGKIYADQGNYTFSAKDCFEIARAAYNEHD |
| Q1RMU3 | TEWSELENLVLKDMSDGFISNLTIQRQYFPNDEDQVGAAKA |
| Q1RMU3 | QRANGNLKYFEYIMAKEKDANKSSSDDQSDQKTTLKKKGAA |
| Q06478 | FVGDPSSSNELDRFSVCPFSNDTVKMIFLTRENRKHDFYTL |
| Q06478 | HTSSNLGTERTLGTVDFYINNGSNQPGCRYIIGETCSHTRA |
| P23028 | PNEVFNVDYSYECNEGQLTCNESNNECEMAVCNCDRAAAIC |
| Q9NZ20 | EKRGWTMPGTLWCGVGDSAGNSSELGVFQGPDLCCREHDRC |
| Q9NZ20 | GGCRMYGTVPLARLQPRTFYNASWSSRATSPTPSSRSPAPP |
| Q9NZ20 | KQHLRKGPPHQKGSKRPSKANTTALQDPMVSPRLDVAPTGL |
| Q9NZ20 | GPREIEFQLLNSAQEPLFHCNCTRRLARFLRLHSPPEVTNM |
| Q9NZ20 | ELLGTTCFKLAPPLDCVEGKNCSRDPRAIRVSARHLRRLQQ |
| Q9U256 | PTTTTLSPQILKAKLPPVVKNATWECGTDEFTKSISEGEIQ |
| Q9U256 | PLFCDLVRTFGDGAYEASGPNASTTEESPAEKDDYDYESHV |
| P00616 | ECYAEAGKLSACKSVLSEPNNDTYSYECNEGQLTCNDDNDE |
| Q6PXP0 | EERFLIVSGTKWCGNNNIAANYSDLGFLEADKCCRDHDHCD |
| Q6PXP0 | TILNCDCDEAFDHCLKEISNNVTTDIRQKGGAENVWRFYFQ |
| P00630 | GDNELEERIIYPGTLWCGHGNKSSGPNELGRFKHTDACCRT |
| P09466 | QDLELPKLAGTWHSMAMATNNISLMATLKAPLRVHITSLLP |
| P09466 | ITSLLPTPEDNLEIVLHRWENNSCVEKKVLGEKTENPKKFK |
| Q8NCC3 | LELLLPVIIDCWIDNIRLVYNKTSRATQFPDGVDVRVPGFG |
| Q8NCC3 | PLKIREQQRSAVSTSWLLPYNYTWSPEKVFVQTPTINYTLR |
| Q8NCC3 | LLPYNYTWSPEKVFVQTPTINYTLRDYRKFFQDIGFEDGWL |
| P05121 | FHKSDGSTVSVPMMAQTNKFNYTEFTTPDGHYYDILELPYH |
| P05121 | VPLSALTNILSAQLISHWKGNMTRLPRLLVLPKFSLETEVD |
| P05121 | LSDQEPLHVAQALQKVKIEVNESGTVASSSTAVIVSARMAP |
| P91268 | WDEKSFNDHETFNKDLGVINNKTIAIISREKKVIDEFGAGL |
| Q6UXH9 | SHITVAGWNVLADVRSPGFKNDTLRSGVVSVVDSLLCEEQH |
| Q96RD7 | FSLSSLSDEFVCSIKSGILRNDSTVPDQFQCKLIAVGIFQL |
| Q9JIP4 | FSLSSLSDEFLCSIKSGVLKNDSTIPDRFQCKLIAVGIFQL |
| Q8CEG0 | SLAFAQEFSSGSPISCFSPSNFSVRQAAYVDSSCWDSLAHH |
| O64411 | GANWVEGVNGGKMNPIWPIVNSTLKLRNFRSDFDYLAQNVY |
| Q13219 | VTREQVDFQHHQLAEAFKQYNISWELDVLEVSNSSLRRRLI |
| Q13219 | LAEAFKQYNISWELDVLEVSNSSLRRRLILANCDISKIGDE |
| Q13219 | LILANCDISKIGDENCDPECNHTLTGHDGGDCRHLRHPAFV |
| Q13219 | DCNYERFNFDGGECCDPEITNVTQTCFDPDSPHRAYLDVNE |
| Q13219 | QSCSDPCMETEPSFETGDLCNDTNPAPKHKSCGDPGPGNDT |
| Q13219 | LCNDTNPAPKHKSCGDPGPGNDTCGFHSFFNTPYNNFMSYA |

| | |
|---|---|
| Q13219 | ELGSACHLCLEGRILVQYASNASSPMPCSPSGHWSPREAEG |
| Q13219 | TDWDSSGAVNDIKLLAVSGKNISLGPQNVFCDVPLTIRLWD |
| Q13219 | TSIKDCGVYTPQGFLDQWASNASVSHQDQQCPGWVIIGQPA |
| Q13219 | EQSCVHFACEKTDCPELAVENASLNCSSSDRYHGAQCTVSC |
| Q13219 | VHFACEKTDCPELAVENASLNCSSSDRYHGAQCTVSCRTGY |
| Q13219 | GTTFGSQCSFQCRHPAQLKGNNSLLTCMEDGLWSFPEALCE |
| Q13219 | SDASQGLGSNVIHCRKDGTWNGSFHVCQEMQGQCSVPNELN |
| Q13219 | GSECATSCLDHNSESIILPMNVTVRDIPHWLNPTRVERVVC |
| P30558 | NPSENTFELVPLGDEEEEEKNESVLLEGRAVYLNISLPPHT |
| P30558 | DEEEEEKNESVLLEGRAVYLNISLPPHTPPPPFISEDASGY |
| P30558 | AIMGVVPLLLKEQTTRVPGLNITTCHDVLSENLMQGFYSYY |
| P55085 | LGAAILLAASLSCSGTIQGTNRSSKGRSLIGKVDGTSHVTG |
| P55085 | ILLVTIPLYVVKQTIFIPALNITTCHDVLPEQLLVGDMFNY |
| P09791 | LTKGGKGKGGKGTKVSDDDTNGTDPDPEPEPEPEPEPEPEP |
| Q6UWI2 | PTTIWTSSPQNTDADTASPSNGTHNNSVLPVTASAPTSLLP |
| Q6UWI2 | WTSSPQNTDADTASPSNGTHNNSVLPVTASAPTSLLPKNIS |
| Q6UWI2 | THNNSVLPVTASAPTSLLPKNISIESREEEITSPGSNWEGT |
| Q6UWI2 | SSPSSLSTSPPEVFSASVTTNHSSTVTSTQPTGAPTAPESP |
| P34446 | NRATCREVHVDKMKGNLKKLNGSHLVPIEEKSHQFFGATVR |
| P34446 | FIGAPGVWYWQGAMFSQNIKNQTDRPNTEYGSKEYDHDMMG |
| P34446 | RGNDLHGKLVLYTSKLKMMINLTDEVSTQHGQYCGGSVAVA |
| P34446 | HRFFASSTMKDKLSPIHWSVNYTYVESKTGKLRGDKLEPAI |
| P34446 | TAVADREKFLLGTQDNTMLINVTVQNGGEDSYETKLYFDVP |
| P34446 | RVTASSDKPPLAPISINAHVNSSNDEEAHTVADNKVTFTIP |
| P34446 | KNQLSLNGRSNPEQVDFSMTNKTRVDAFDDNEIGPVVSHLY |
| Q27874 | EVTGKVVEKSEFPCYSLSRDNYTCSACIQYHESCAWCGAPM |
| Q27874 | VQIKPQEMYVEIRPKSRVRFNVTYRQAVDYPVDLYYLMDLS |
| Q27874 | SGNLDAPEGGFDAVVQALACNKTIGWRERARKMIVFSTDAG |
| Q27874 | LYTQLSNALPDVSSSVGVLANDSRNIVDLIEKEYLKISEKI |
| Q27874 | DLIEKEYLKISEKIIMVDNANASEGLKLTYRSMCLDGTTLK |
| Q27874 | CECNRPGMSTAALNEKCKRTNESAICEGRGVCNCGRCECNP |
| Q27874 | TKCVEYKNCVMCQQWQTGPLNETACDQCEFKVIPVEELPNL |
| Q27874 | ETACDQCEFKVIPVEELPNLNETTPCQFVDPADDCTFYYLY |
| Q27874 | VDPADDCTFYYLYYYDEATDNATVWVRKHKDCPPPVPVLAI |
| P15478 | KGIIPATILEFLEGQLQEVDNNTDARLADYFDVIGGTSTGG |
| P15478 | FDVIGGTSTGGLLTAMITTPNETNRPFAAAKDIVPFYFEHG |
| P15478 | DICYSTAAAPTFFPPHYFATNTSNGDKYEFNLVDGAVATVD |
| Q966B0 | RSLLISVISGTFKNNSSSKQNKTLFFFDDVPFHWTLKTQNE |
| Q966B0 | QNKTLFFFDDVPFHWTLKTQNESIQLGNRFKNRYFEFKYEI |
| Q966B0 | FDLKATDKVVKTLVIRMIGINQTLNGGGKAEACLETFENCT |
| Q966B0 | GINQTLNGGGKAEACLETFENCTEIPVDEDLIIDMPRADTL |

| | |
|---|---|
| Q966B0 | AYWGEFFSRLRWVSTQDHLLNISSTFHDDSGRVFALSPKTG |
| Q08174 | YEAELSENSPIGHSVIQVKANDSDQGANAEIEYTFHQAPEV |
| Q08174 | NAPTIEIRGIGLVTHQDGMANISEDVAEETAVALVQVSDRD |
| Q08174 | VLVNVLDCNDNDPKFMLSGYNFSVMENMPALSPVGMVTVID |
| Q08174 | GENAQVQLSVEQDNGDFVIQNGTGTILSSLSFDREQQSTYT |
| Q08174 | KVSDRGKPPRYGTALVHLYVNETLANRTLLETLLGHSLDTP |
| Q08174 | GKPPRYGTALVHLYVNETLANRTLLETLLGHSLDTPLDIDI |
| P21902 | TTERPPKQIPPNLPEVCGIHNTTTTRIIGGREAPIGAWPWM |
| P21902 | KHHEHFVLATYLNDIAILTLNDTVTFTDRIRPICLPYRKLR |
| P21902 | EVQLPIWEHEACRQAYEKDLNITNVYMCAGFADGGKDACQG |
| Q96NT5 | LQGPLTTQYLWHRFSADLGYNGTRQRGGCSNRSADPTMQEV |
| Q96NT5 | WHRFSADLGYNGTRQRGGCSNRSADPTMQEVETLTSHWTLY |
| Q6PEM8 | LQGPLTTQYLWHRFSTELGYNGTRHRENCGNQSADPLMKEV |
| Q6PEM8 | WHRFSTELGYNGTRHRENCGNQSADPLMKEVETLTSHWTLY |
| Q61398 | SFLGPFLLAWVLPLARGQTPNYTRPVFLCGGDVTGESGYVA |
| P34610 | MKLDHFTWGDTRTFDMRVMWNNTFYKPGGPIFFYTGNEGGL |
| P34610 | GGLESFVTATGMMFDLAPMFNASIIFAEHRFYGQTQPFGNQ |
| P34610 | FNASIIFAEHRFYGQTQPFGNQSYASLANVGYLTSEQALAD |
| P34610 | ITSRTYIDNGCNRFILANAWNATLNLSSTDAGRQWLNNNTV |
| P34610 | TYIDNGCNRFILANAWNATLNLSSTDAGRQWLNNNTVFKLD |
| P34610 | NAWNATLNLSSTDAGRQWLNNNTVFKLDPRTKIRNQTDGWN |
| P34610 | GRQWLNNNTVFKLDPRTKIRNQTDGWNLNAYLREAIEYMAM |
| P34610 | GFLEPLPAWPVTVACGYMNANGTSFSDKDLVKAVANAANIY |
| P34610 | DLVKAVANAANIYYNYNRDPNFTYCIDFSICGDQGTGGLGG |
| P34676 | LKAAVVFVEHRFYGKSQPFKNESYTDIRHLGYLSSQQALAD |
| P34676 | PGKTQEESAEQLYKIVNLYYNYTGDKSTHCANAAKCDSAYG |
| P34676 | PFTSEKYAEFCMQTFSSIHYNKTLLRPLAGGLAFGATSLPS |
| P84875 | VCDLPADRGQCTAYIPQWFFNKTTEDCEKFVYGGCQGNANR |
| P42785 | RALGSLHLPTNPTSLPAVAKNYSVLYFQQKVDHFGFNTVKT |
| P42785 | KNGGSILFYTGNEGDIIWFCNNTGFMWDVAEELKAMLVFAE |
| P42785 | FLQPLPAWPIKVVCQYLKNPNVSDSLLLQNIFQALNVYYNY |
| P42785 | PNVSDSLLLQNIFQALNVYYNYSGQVKCLNISETATSSLGT |
| P42785 | QNIFQALNVYYNYSGQVKCLNISETATSSLGTLGWSYQACT |
| P42785 | FQQWGVRPRPSWITTMYGGKNISSHTNIVFSNGELDPWSGG |
| Q8NBP7 | NAFGGEGVYAIARCCLLPQANCSVHTAPPAEASMGTRVHCH |
| Q9UHG3 | FSSVEKLLHALGGDDFLGMLNRTLLETLQKAGFSEKFLNEM |
| Q9UHG3 | TRSDFYDIVLVATPLNRKMSNITFLNFDPPIEEFHQYYQHI |
| Q9UHG3 | IEEFHQYYQHIVTTLVKGELNTSIFSSRPIDKFGLNTVLTT |
| Q9CQF9 | FSSVEKLMHAIGGDDYVRLLNQTLRENLKKAGFSETFLNEM |
| Q9CQF9 | THSDFYDIVLVAAPLNRKMSNITFRNFDPPIEEFNDPYQQL |
| Q9CQF9 | IEEFNDPYQQLVTTFIKGELNSTLFSSRPKDQFGLSAILVT |

| | |
|---|---|
| Q9NZQ7 | LLNAFTVTVPKDLYVVEYGSNMTIECKFPVEKQLDLAALIV |
| Q9NZQ7 | HQVLSGKTTTTNSKREEKLFNVTSTLRINTTTNEIFYCTFR |
| Q9NZQ7 | TTTNSKREEKLFNVTSTLRINTTTNEIFYCTFRRLDPEENH |
| Q9NZQ7 | INTTTNEIFYCTFRRLDPEENHTAELVIPELPLAHPPNERT |
| Q3L245 | FVTRVPGLASTLGAGSPIDWNYTTIPQDGLDGRSLDYPRAK |
| Q3L245 | HIDPSVHGFDGKLSVSAAYSNISFNDLLFETTKELNAEFPF |
| Q3L245 | GDNVHVLVNTLVTRVLSASGNGTDFRKVEFAVDANSPKKQL |
| Q3L245 | RKVLQAVGIDTLIDNPSVGKNLSDQGATSVMFDTTLPSTDF |
| Q17770 | ISLEEDMTKFKPDFEEITTENISKFTQNYLDGSVKPHLMSE |
| Q13087 | ELAEEFGVTEYPTLKFFRNGNRTHPEEYTGPRDAEGIAEWL |
| Q13087 | SQTSAKIFAARILNHLLLFVNQTLAAHRELLAGFGEAAPRF |
| Q9NZ53 | REKEEVEKQEEEEEEELLPVNGSQEEAKPQVRDFSLTSSSQ |
| Q9NZ53 | STQVICKDWSNLAGKNYIIILNMTENIDCEVFRQHRGPQLLA |
| P16284 | FTINSVDMKSLPDWTVQNGKNLTLQCFADVSTTSHVKPQHQ |
| P16284 | TSHVKPQHQMLFYKDDVLFYNISSMKSTESYFIPEVRIYDS |
| P16284 | PSPRVTLDKKEAIQGGIVRVNCSVPEEKAPIHFTIEKLELN |
| P16284 | HNRHGNKAVYSVMAMVEHSGNYTCKVESSRISKVSSIVVNI |
| P16284 | GNYTCKVESSRISKVSSIVVNITELFSKPELESSFTHLDQG |
| P16284 | LFSKPELESSFTHLDQGERLNLSCSIPGAPPANFTIQKEDT |
| P16284 | HLDQGERLNLSCSIPGAPPANFTIQKEDTIVSQTQDFTKIA |
| P16284 | SISGTLPISYQLLKTSKVLENSTKNSNDPAVFKDNPTEDVE |
| P16284 | PITYKFYREKEGKPFYQMTSNATQAFWTKQKASKEQEGEYY |
| Q08481 | TSKSRSQHRVLFYKDDAMVYNVTSREHTESYVIPQARVFHS |
| Q08481 | SKPKVTLDKKEVTEGGVVTVNCSLQEEKPPIFFKIEKLEVG |
| Q08481 | GHYTCKVESNRISKASSIMVNITELFPKPKLEFSSSRLDQG |
| Q08481 | RLDQGELLDLSCSVSGTPVANFTIQKEETVLSQYQNFSKIA |
| Q08481 | GTPVANFTIQKEETVLSQYQNFSKIAEESDSGEYSCTAGIG |
| Q08481 | HDAKSEIIKGHAIGISCQSENGTAPITYHLMKAKSDFQTLE |
| Q08481 | PITFQFYKEKEDRPFHQAVVNDTQAFWHNKQASKKQEGQYY |
| P36955 | QLPLTGSMSIIFFLPLKVTQNLTLIEESLTSEFIHDIDREL |
| Q01172 | APSVSVEYYNAGVLGITVTSNKSLIGEGSSGAIKGKGLRIV |
| Q02567 | GPKAGGGADGSMLLFPTVEPNFSANNGIDDSVNNLIPFMQK |
| Q02567 | AVALSNCPGAPRLEFLAGRPNKTIAAVDGLIPEPQDSVTKI |
| Q02567 | FDTQVFLEVLLKGVGFPGSANNTGEVASPLPLGSGSDTGEM |
| P27678 | LENYLDVEYFGTIGIGTPAQNFTVVFDTGSSNLWVPSVYCY |
| P00793 | SACSNHKRFDPSKSSTYVSTNETVYIAYGTGSMSGILGYDT |
| P16311 | GSCWAFSGVAATESAYLAYRNTSLDLSEQELVDCASQHGCH |
| P08176 | GSCWAFSGVAATESAYLAYRNQSLDLAEQELVDCASQHGCH |
| Q17758 | APDDSVRKPISFTGDDFFQPNITFDNLAPNCPKFTAEPMLA |
| O01840 | VNETMEFPPSLGRIYLQRVGNESLISDFRYKSDGRLIGDGM |
| O01840 | LPKGRTELDAGIYTFNTGLKNESQEIDISTPNKGYVMAVFR |

| | |
|---|---|
| P00433 | LHASLSDAQLTPTFYDNSCPNVSNIVRDTIVNELRSDPRIA |
| P00433 | LRLHFHDCFVNGCDASILLDNTTSFRTEKDAFGNANSARGF |
| P00433 | LPAPFFTLPQLKDSFRNVGLNRSSDLVALSGGHTFGKNQCR |
| P00433 | LSGGHTFGKNQCRFIMDRLYNFSNTGLPDPTLNTTYLQTLR |
| P00433 | RFIMDRLYNFSNTGLPDPTLNTTYLQTLRGLCPLNGNLSAL |
| P00433 | DPTLNTTYLQTLRGLCPLNGNLSALVDFDLRTPTIFDNKYY |
| P00433 | VNLEEQKGLIQSDQELFSSPNATDTIPLVRSFANSTQTFFN |
| P00433 | QELFSSPNATDTIPLVRSFANSTQTFFNAFVEAMDRMGNIT |
| P22195 | HFHDCFVQGCDASVLLDDTSNFTGEKTAGPNANSIRGFEVI |
| P22195 | RDSTTASLSSANSDLPAPFFNLSGLISAFSNKGFTTKELVT |
| P80679 | LTANLAGANSAIPSPFEGLSNITSKFSAVGLNTNDLVALSG |
| P80679 | LSGAHTFGRARCGVFNNRLFNFSGTNGPDPTLNSTLLSSLQ |
| P80679 | GVFNNRLFNFSGTNGPDPTLNSTLLSSLQQLCPQNGSASTI |
| P80679 | GPDPTLNSTLLSSLQQLCPQNGSASTITNLDLSTPDAFDNN |
| P80679 | QELFSTLGSATIAVVTSFASNQTLFFQAFAQSMINMGNISP |
| P82600 | WGMEGYPFDRVLEPAYEDGVWAPRIHSVTGNLLPSARVISV |
| P82600 | FTLSRGFTTKHGQAIECCTPNCTAPLFGPHRHFACFPIEVP |
| P82600 | TLFMREHNRLAVGLSKINPHWDDERLYQEARRILIAEYQNV |
| P82600 | LGEVGALLAQVYESPDDVDLWPGGVLEPPAEGAVVGSTFVA |
| P14222 | HTPPLHPDFKRALGDLPHHFNASTQPAYLRLISNYGTHFIR |
| P10820 | QKPPLHLDFKKALRALPRNFNSSTEHAYHRLISSYGTHFIT |
| P10820 | KREALRQAISHYIMSRARWQNCSRPCRSGQHKSSHDSCQCE |
| P80025 | RNGQVWEESLKRLRRDTTLTNVTDPSLDLTALSWEVGCGAP |
| P80025 | AREVSNKIVGYLDEEGVLDQNRSLLFMQWGQIVDHDLDFAP |
| P80025 | SFLDASLVYGSEPSLASRLRNLSSPLGLMAVNQEAWDHGLA |
| P80025 | DHGLAYLPFNNKKPSPCEFINTTARVPCFLAGDFRASEQIL |
| P80025 | YLPIVLGSEMQKWIPPYQGYNNSVDPRISNVFTFAFRFGHM |
| A5JUY8 | RNGQVWEESFKRLRRDTTLTNVTDPSLDLTALSWEVGCGAP |
| A5JUY8 | AREVSNKIVGYLDEEGVLDQNRSLLFMQWGQIVDHDLDFAP |
| A5JUY8 | SFLDASLVYGSEPSLASRLRNLSSPLGLMAVNQEAWDHGLA |
| A5JUY8 | DHGLAYLPFNNKKPSPCEFINTTARVPCFLAGDFRASEQIL |
| A5JUY8 | YLPIVLGSEMQKWIPPYQGYNNSVDPRISNVFTFAFRFGHM |
| P22079 | RNGQVWEESLKRLRQKASLTNVTDPSLDLTSLSLEVGCGAP |
| P22079 | AREVSNKIVGYLNEEGVLDQNRSLLFMQWGQIVDHDLDFAP |
| P22079 | SFLDASFVYSSEPSLASRLRNLSSPLGLMAVNQEVSDHGLP |
| P22079 | DHGLPYLPYDSKKPSPCEFINTTARVPCFLAGDSRASEHIL |
| P05164 | LHVALDLLERKLRSLWRRPFNVTDVLTPAQLNVLSKSSGCA |
| P05164 | IKNQADCIPFFRSCPACPGSNITIRNQINALTSFVDASMVY |
| P05164 | SFVDASMVYGSEEPLARNLRNMSNQLGLLAVNQRFQDNGRA |
| P05164 | DNGRALLPFDNLHDDPCLLTNRSARIPCFLAGDTRSSEMPE |
| P05164 | LPLVLGPTAMRKYLPTYRSYNDSVDPRIANVFTNAFRYGHT |

| | |
|---|---|
| P00434 | LSGAHTIGQSRCVNFRARVYNETNINAAFATLRQRSCPRAA |
| P09933 | RDTDQLPTDVLSEELLSTIANLSGCLPHMLPPSCPHTCLAN |
| P09933 | ADCQLTCENRSPCFPIQLPTNASGAAGATCLPFYRSSAACG |
| P09933 | LPFYRSSAACGSGRQGALVGNLSWAAPRQQMNGLTSFLDAS |
| P09933 | SFLDASTVYGSSPAQEQRLRNWTSAEGLLRVNTRHRDAGRA |
| P28313 | AVGMSNCPGSPRLEFLTGRSNSSQPSPPSLIPGPGNTVTAI |
| P28314 | AVGMSNCPGSPRLEFLTGRSNSSQPSPPSLIPGPGNTVTAI |
| Q3UUQ7 | LTGTSMWVPVKVSRWSYVAYNESDKIYFAFPLANHRKIYTH |
| Q3UUQ7 | THAYCQSTMLDTNSWIFGCINSTSMCRQGVDLSWKAELLPT |
| Q3UUQ7 | IAQVPSSTDISLKLHVAQPENDSHVALLKMYTSSDCQYEVT |
| Q7K0P4 | ASNGDRTQFFHNCRQNCERTNCSADGLEIQEQAVKFYQQSV |
| P98160 | LGSGDLGSGDFQMVYFRALVNFTRSIEYSPQLEDAGSREFR |
| P98160 | RRFRDQIRLRFDQPDDFKGVNVTMPAQPGTPPLSSTQLQID |
| P98160 | SVQPSDAGVYICTCRNLHQSNTSRAELLVTEAPSKPITVTV |
| P98160 | RLRLPQVSPADSGEYVCRVENGSGPKEASITVSVLHGTHSG |
| P98160 | ISLEWKTRNQELEDNVHISPNGSIITIVGTRPSNHGTYRCV |
| P98160 | NHGTYRCVASNAYGVAQSVVNLSVHGPPTVSVLPEGPVWVK |
| P98160 | EGDTLIIPRVAQQDSGQYICNATSPAGHAEATIILHVESPP |
| P98160 | VTLLRSLTQGSLIVGDLAPVNGTSQGKFQGLDLNEELYLGG |
| P98160 | FIGCVRELRIQGEEIVFHDLNLTAHGISHCPTCRDRPCQNG |
| P98160 | LHTLLYLGGVEPSVPLSPATNMSAHFRGCVGEVSVNGKRLD |
| Q05793 | LGSGDVGSGDFQMVYFRALVNFTRSIEYSPQLEDASAKEFR |
| Q05793 | ALKLWRCDGDFDCEDRTDEANCSVKQPGEVCGPTHFQCVST |
| Q05793 | LRFRDQIRLSFDQPNDFKGVNVTMPSQPGVPPLSSTQLQID |
| Q05793 | SCSPGHSGRHCERCAPGYYGNPSQGQPCHRDGQVPEVLGCG |
| Q05793 | PPQLTVQPGQQAEFRCSATGNPTPMLEWIGGPSGQLPAKAQ |
| Q05793 | AGSSHPEHEASFKLTVPSSQNSSFRLRSPVISIEPPSSTVQ |
| Q05793 | IKVEWKIRDQELEDNVHISPNGSIITIVAPGPATMEPTACV |
| Q05793 | TMEPTACVASNVYGMAQSVVNLSVHGPPTVSVLPEGPVHVK |
| Q05793 | EGNTLVIPRVAQQDSGQYICNATNSAGHTEATVVLHVESPP |
| Q05793 | VTLLRSLTQGSLIVGNLAPVNGTSQGKFQGLDLNEELYLGG |
| Q05793 | FVGCVRELRIQGEEIVFHDVNLTTHGISHCPTCQDRPCQNG |
| Q05793 | LHTLLYLGGVEPSVQLSPATNMSAHFHGCVGEVSVNGKRLD |
| P07898 | PEEEDTNALLTPRIKWSKLSNGTEIVLLVATGGKIRLNAEY |
| P07898 | LPNYPAIPTDATLEIKALRSNHTGIYRCEVMYGIEDRQDTI |
| P07898 | ARPNCGGNLVGVRTVYLNPANQTGYPHPSSRYDAICYSGDD |
| P07898 | GSAFTIQTVTQTEVELPLPRNVTEEEARGSIATLEPMEITA |
| P07898 | VLPDLFATSVTVETASPREENVTREEITGIWAVPEEVTTSV |
| P07898 | FATQPEQFTFQEAQLYCESQNATLASAGQLHAAWKQGLDRC |
| P07898 | SPRPACGGDAPGVRTIYQHHNQTGFPDPLSRHHAFCFRALP |
| P07898 | EGIPSGEETTVETELSSEPENQTAQGTEVFPTDVSLLSARP |

| | |
|---|---|
| P07898 | LSARPSAFPPATVIPEETSTNASIPEVSGEFPESGEHPTSG |
| P16112 | LPNYPAIPSDATLEVQSLRSNDSGVYRCEVMHGIEDSEATL |
| P16112 | GCYGDKDEFPGVRTYGIRDTNETYDVYCFAEEMEGEVFYAT |
| P16112 | KARPNCGGNLLGVRTVYVHANQTGYPDPSSRYDAICYTGED |
| P16112 | EEDITVQTVTWPDMELPLPRNITEGEARGSVILTVKPIFEV |
| P16112 | PEEPFTFAPEIGATAFAEVENETGEATRPWGFPTPGLGPAT |
| P16112 | FATRLEQFTFQEALEFCESHNATATTGQLYAAWSRGLDKCY |
| P16112 | TPRPACGGDKPGVRTVYLYPNQTGLPDPLSRHHAFCFRGIS |
| P16112 | TTAVPSGETTAILEFTTEPENQTEWEPAYTPVGTSPLPGIL |
| P16112 | EPSGEPPGTPYFSGDFASTTNVSGESSVAMGTSGEASGLPE |
| Q29011 | LPNYPAIPSDATLEIQNLRSNDSGIYRCEVMHGIEDSEATL |
| Q29011 | GCYGDKDEFPGVITYGIRDTNETYDVYCFAEEMEGEVFYAT |
| Q29011 | KARPNCGGNLLGVRTVYLHANQTGYPDPSSRYDAICYTGED |
| Q29011 | EEDITIQTVTWPDVELPLPRNITEGEARGTVILTVKPVFEF |
| Q29011 | PEEPFTFAPGTGATAFPEAENRTGEATRPWAFPEESTPGLG |
| P07897 | LPNYPAIPSDATLEIQNLRSNDSGIYRCEVMHGIEDSEATL |
| P07897 | GCYGDKDEFPGVRTYGIRDTNETYDVYCFAEEMEGEVFYAT |
| P07897 | KARPNCGGNLLGVRTVYLHANQTGYPDPSSRYDAICYTGED |
| P07897 | EEDITIQTVTWPDLELPLPRNITEGEARGNVILTAKPIFDM |
| P07897 | FATQMEQFTFQEAQAFCAAQNATLASTGQLYAAWSQGLDKC |
| P07897 | NPRPACGGDKPGVRTVYLYPNQTGLPDPLSKHHAFCFRGVS |
| P07897 | MTSGDRTEISGEWSDHTSEVNVTVSTTVPESRWAQSTQHPT |
| P05979 | VRFGLDRYQCDCTRTGYSGPNCTIPEIWTWLRTTLRPSPSF |
| P05979 | IPSPPTYNIAHDYISWESFSNVSYYTRILPSVPRDCPTPMD |
| P05979 | LMPDSFRVGPQDYSYEQFLFNTSMLVDYGVEALVDAFSRQP |
| P05622 | LGPQTSRGLVITPPGPEFVLNISSTFVLTCSGSAPVMWEQM |
| P05622 | PWQEAAMNQDGTFSSVLTLTNVTGGDTGEYFCVYNNSLGPE |
| P05622 | SVLTLTNVTGGDTGEYFCVYNNSLGPELSERKRIYIFVPDP |
| P05622 | GDREVDSDTYYVYSLQVSSINVSVNAVQTVVRQGESITIRC |
| P05622 | IGSILHIPTAELSDSGTYTCNVSVSVNDHGDEKAINISVIE |
| P05622 | GTYTCNVSVSVNDHGDEKAINISVIENGYVRLLETLGDVEI |
| P05622 | VSVSVNDHGDEKAINISVIENGYVRLLETLGDVEIAELHRS |
| P05622 | RTLRVVFEAYPMPSVLWLKDNRTLGDSGAGELVLSTRNMSE |
| P05622 | LKDNRTLGDSGAGELVLSTRNMSETRYVSELILVRVKVSEA |
| P05622 | SHPANGEQTIRCRGRGMPQPNVTWSTCRDLKRCPRKLSPTP |
| P05622 | WSTCRDLKRCPRKLSPTPLGNSSKEESQLETNVTFWEEDQE |
| P05622 | RKLSPTPLGNSSKEESQLETNVTFWEEDQEYEVVSTLRLRH |
| P09619 | LEPQISQGLVVTPPGPELVLNVSSTFVLTCSGSAPVVWERM |
| P09619 | PPQEMAKAQDGTFSSVLTLTNLTGLDTGEYFCTHNDSRGLE |
| P09619 | SVLTLTNLTGLDTGEYFCTHNDSRGLETDERKRLYIFVPDP |
| P09619 | GDREVDSDAYYVYRLQVSSINVSVNAVQTVVRQGENITLMC |

| | |
|---|---|
| P09619 | QVSSINVSVNAVQTVVRQGENITLMCIVIGNEVVNFEWTYP |
| P09619 | IRSILHIPSAELEDSGTYTCNVTESVNDHQDEKAINITVVE |
| P09619 | GTYTCNVTESVNDHQDEKAINITVVESGYVRLLGEVGTLQF |
| P09619 | RTLQVVFEAYPPPTVLWFKDNRTLGDSSAGEIALSTRNVSE |
| P09619 | FKDNRTLGDSSAGEIALSTRNVSETRYVSELTLVRVKVAEA |
| P09619 | WSACRDLKRCPRELPPTLLGNSSEEESQLETNVTYWEEEQE |
| P09619 | RELPPTLLGNSSEEESQLETNVTYWEEEQEFEVVSTLRLQH |
| Q05769 | MSTGFDQYKCDCTRTGFYGENCTTPEFLTRIKLLLKPTPNT |
| Q05769 | IDSPPTYNVHYGYKSWEAFSNLSYYTRALPPVADDCPTPMG |
| Q05769 | LLPDTFNIEDQEYSFKQFLYNNSILLEHGLTQFVESFTRQI |
| Q05769 | GCPFTSFNVQDPQPTKTATINASASHSRLDDINPTVLIKRR |
| P35354 | MSVGFDQYKCDCTRTGFYGENCSTPEFLTRIKLFLKPTPNT |
| P35354 | IDSPPTYNADYGYKSWEAFSNLSYYTRALPPVPDDCPTPLG |
| P35354 | LLPDTFQIHDQKYNYQQFIYNNSILLEHGITQFVESFTRQI |
| P35354 | GCPFTSFSVPDPELIKTVTINASSSRSGLDDINPTVLLKER |
| P58822 | KKDLGNPTTLSSWLPTTDCCNRTWLGVLCDTDTQTYRVNNL |
| P58822 | PIPPAIAKLTQLHYLYITHTNVSGAIPDFLSQIKTLVTLDF |
| O74213 | SGGHGLSIGSVGGRSDNTVKNVTFVDSTIINSDNGVRIKTN |
| P26213 | SGGHGLSIGSVGGRDDNTVKNVTISDSTVSNSANGVRIKTI |
| P26214 | IGGHGLSIGSVGDRSNNVVKNVTIEHSTVSNSENAVRIKTI |
| Q95T64 | VSEEGNIYVGRGWDWANTYANQTLAITFMGDYGRFKPGPKQ |
| Q95T64 | LLAHAVANRNIDVDYKLVAQNQTKVTRSPGAYVYQEIRNWP |
| Q9GNK5 | EIPDLELPVGLVIALPTNSENCSTQAICVLRVRLLQTYDIE |
| Q96PD5 | APNSGPHNRLYHFLLGAWSLNATELDPCPLSPELLGLTKEV |
| Q96PD5 | EPVHLQLQCMSQEQLAQVAANATKEFTEAFLGCPAIHPRCR |
| Q96PD5 | WVGAHTLGHNSRGFGVAIVGNYTAALPTEAALRTVRDTLPS |
| Q8VCS0 | QKVPVTEASITASAWILSAKNSSTHNSLHQRLLLKAPSHNT |
| Q8VCS0 | KNSSTHNSLHQRLLLKAPSHNTTEPDPHSLSPELQALISEV |
| Q8VCS0 | TGDTLANIRATWPGLMDAFPNASSPDVGATLPNDKAKTPTT |
| Q8VCS0 | QVWEALVLLQKLEPEHLQLQNISQEQLAQVATLATKEFTEA |
| Q8VCS0 | WVGAHTRGYNSRGFGVAFVGNYTGSLPNEAALNTVRDALPS |
| P21809 | LRYSKLYRLGLGHNQIRMIENGSLSFLPTLRELHLDNNKLS |
| P21809 | RVPAGLPDLKLLQVVYLHTNNITKVGVNDFCPVGFGVKRAY |
| P21810 | LRYSKLYRLGLGHNQIRMIENGSLSFLPTLRELHLDNNKLA |
| P21810 | RVPSGLPDLKLLQVVYLHSNNITKVGVNDFCPMGFGVKRAY |
| P21793 | IENGAFQGMKKLSYIRIADTNITTIPQGLPPSLTELHLDGN |
| P21793 | KGLNNLAKLGLSFNSISAVDNGSLANTPHLRELHLNNNKLV |
| P21793 | KVPGGLADHKYIQVVYLHNNNISAIGSNDFCPPGYNTKKAS |
| P07585 | IENGAFQGMKKLSYIRIADTNITSIPQGLPPSLTELHLDGN |
| P07585 | KGLNNLAKLGLSFNSISAVDNGSLANTPHLRELHLDNNKLT |
| P07585 | RVPGGLAEHKYIQVVYLHNNNISVVGSSDFCPPGHNTKKAS |

| | |
|---|---|
| P05088 | LVLLTHANSASQTSFSFQRFNETNLILQRDATVSSKGQLRL |
| P05088 | EPTLSSLGRAFYSAPIQIWDNTTGAVAASPTSFTFNIDVPN |
| P05088 | NTTGAVAASPTSFTFNIDVPNNSGPADGLAFVLLPVGSQPK |
| P05087 | LVLLTHANSSNDIYFNFQRFNETNLILQRDASVSSSGQLRL |
| P05087 | EPRVGSLGRAFYSAPIQIWDNTTGTVASFATSFTFNIQVPN |
| O70362 | ICKRGKYHDVSERTHWTPFLNASIHYIRENYPLPWEKDTEK |
| O70362 | LDDMAFWSTNIYRLTSFMLENGTSDCNLPENPLFISCDGRN |
| O70362 | NGTSDCNLPENPLFISCDGRNHTLSGSKVQKNDFHRNLTMF |
| O70362 | CDGRNHTLSGSKVQKNDFHRNLTMFISRDIRKNLNYTERGV |
| O70362 | KNDFHRNLTMFISRDIRKNLNYTERGVFYSTGSWARPESVT |
| O70362 | DGLPDLAVGAPSVGSGQLTYNGSVYVYYGSQQGRLSSSPNV |
| O70362 | YNGSVYVYYGSQQGRLSSSPNVTISCKDTYCNLGWTLLATD |
| O70362 | VNGEEDFSWFGYSLHGVTVANRSLLLIGSPTWKNVSRMARS |
| O70362 | LHGVTVANRSLLLIGSPTWKNVSRMARSSHKKNQEEKSLGK |
| O70362 | SGDKAMGKLGTSLSSGYVRVNGTLTQVLLVGAPTHDDVSKM |
| P80108 | ICKGGKFHDVSESTHWTPFLNASVHYIRENYPLPWEKDTEK |
| P80108 | LDDMAFWSTNIYHLTSFMLENGTSDCNLPENPLFIACGGQQ |
| P80108 | GTSDCNLPENPLFIACGGQQNHTQGSKMQKNDFHRNLTTSL |
| P80108 | CGGQQNHTQGSKMQKNDFHRNLTTSLTESVDRNINYTERGV |
| P80108 | KNDFHRNLTTSLTESVDRNINYTERGVFFSVNSWTPDSMSF |
| P80108 | YKGAVYVYFGSKQGGMSSSPNITISCQDIYCNLGWTLLAAD |
| P80108 | AAFYSGPSLSDKEKLNVEAANWTVRGEEDFSWFGYSLHGVT |
| P80108 | VRGEEDFSWFGYSLHGVTVDNRTLLLVGSPTWKNASRLGHL |
| P80108 | LHGVTVDNRTLLLVGSPTWKNASRLGHLLHIRDEKKSLGRV |
| P80108 | SGDKAMGKLGTSLSSGHVLMNGTLKQVLLVGAPTYDDVSKV |
| Q95XM2 | EPQPKLAATMLLVTGGTLPRNKTESFETACMIEEDVVMHPF |
| Q10944 | GSWLGEKFNRNAIYIRSSDYNRTLMSAQANMAGLFPPKYPI |
| Q10944 | NMELKATWRIFDNLFCEKQNNITWPSWMNSSIFERVDQLYN |
| Q10944 | RIFDNLFCEKQNNITWPSWMNSSIFERVDQLYNEVSQLEFH |
| Q10944 | FYDIYPKYATCLLIEMHKLANETRLIRVFHKNETDIDRLIE |
| Q10944 | LLIEMHKLANETRLIRVFHKNETDIDRLIEYSIPGCDDPCT |
| P07219 | KSSSRKSHSKQDNTIGNEFGNLTERTDNSLNVLISSIEMKE |
| P07219 | DDVFVIPAAYPVAIKATSNVNFTGFGINANNNNRNLLAGKT |
| P02853 | KSSSRKSLSKQDNTIGNEFGNLTERTDNSLNVLISSIEMEE |
| P02853 | DDVFVIPAAYPVAIKATSNVNFTGFGINANNNNRNLLAGKT |
| O00092 | RYPTSSKSKKYKKLVTAIQANATDFKGKFAFLKTYNYTLGA |
| O00092 | TAIQANATDFKGKFAFLKTYNYTLGADDLTPFGEQQLVNSG |
| O00092 | GATNRAAPAISVIIPESETFNNTLDHGVCTKFEASQLGDEV |
| O00092 | LDHGVCTKFEASQLGDEVAANFTALFAPDIRARAEKHLPGV |
| O00092 | FTNELIARLTRSPVQDHTSTNSTLVSNPATFPLNATMYVDF |
| O00092 | VQDHTSTNSTLVSNPATFPLNATMYVDFSHDNSMVSIFFAL |

| | |
|---|---|
| O00092 | YVDFSHDNSMVSIFFALGLYNGTEPLSRTSVESAKELDGYS |
| P34752 | LLPLYLLSGVTSGLAVPASRNQSSCDTVDQGYQCFSETSHL |
| P34752 | QCFSETSHLWGQYAPFFSLANESVISPEVPAGCRVTFAQVL |
| P34752 | RYPTDSKGKKYSALIEEIQQNATTFDGKYAFLKTYNYSLGA |
| P34752 | EEIQQNATTFDGKYAFLKTYNYSLGADDLTPFGEQELVNSG |
| P34752 | AQPGQSSPKIDVVISEASSSNNTLDPGTCTVFEDSELADTV |
| P34752 | LDPGTCTVFEDSELADTVEANFTATFVPSIRQRLENDLSGV |
| P34752 | YANELIARLTHSPVHDDTSSNHTLDSSPATFPLNSTLYADF |
| P34752 | VHDDTSSNHTLDSSPATFPLNSTLYADFSHDNGIISILFAL |
| P34752 | YADFSHDNGIISILFALGLYNGTKPLSTTTVENITQTDGFS |
| P34752 | ILFALGLYNGTKPLSTTTVENITQTDGFSSAWTVPFASRLY |
| P34755 | SGYGRVIETARKFGEGFFGYNYSTNAALNIISESEVMGADS |
| P34755 | YYYCAGPGDKNMAAVGAVYANASLTLLNQGPKEAGSLFFNF |
| P34755 | TTTCNVSASYPQYLSFWWNYNTTTELNYRSSPIACQEGDAM |
| Q92508 | LPPAGIWARVLGLKDFVGPTNCSSPHALVLNTGLDWPVYAS |
| Q92508 | SGALWRISPPSRAQMKRELYNGTADITLRFTWNFQRDLAKG |
| P01833 | GCITLISSEGYVSSKYAGRANLTNFPENGTFVVNIAQLSQD |
| P01833 | SEGYVSSKYAGRANLTNFPENGTFVVNIAQLSQDDSGRYKC |
| P01833 | NSRGLSFDVSLEVSQGPGLLNDTKVYTVDLGRTVTINCPFK |
| P01833 | YKQIGLYPVLVIDSSGYVNPNYTGRIRLDIQGTGQLLFSVV |
| P01833 | SEGWVKAQYEGRLSLLEEPGNGTFTVILNQLTSRDAGFYWC |
| P01833 | WRTTVEIKIIEGEPNLKVPGNVTAVLGETLKVPCHFPCKFS |
| P01833 | KVPCHFPCKFSSYEKYWCKWNNTGCQALPSQDEGPSKAFVN |
| Q6PD26 | IEGAVQRFVQPFLNRLSVAGNFSVDSQILYYAMLGVNPRFD |
| Q6PD26 | AFHSPRWGGIMVYNVDPKIYNASELPVRVEVDMVRVMEVFL |
| P01832 | GRCVTLASTGYTSQEYSGRGKLTDFPDKGEFVVTVDQLTQN |
| P01832 | KLTDFPDKGEFVVTVDQLTQNDSGSYKCGVGVNGRGLDFGV |
| P01832 | GEGLVQKDYTGRLALFEEPGNGTFSVVLNQLTAEDEGFYWC |
| Q8BXQ2 | FIDATNTVTPTASFKPLGLANDTDDYFLRYAVLPREVVCTE |
| Q8BXQ2 | ACPLASQSLVYVDITGYSQDNETLEVSPPPTSTYQDVILGT |
| Q8BXQ2 | VILGTRKTYAVYDLFDTAMINNSRNLNIQLKWKRPPDNEAL |
| Q969N2 | FIDSTNTVTPTASFKPLGLANDTDHYFLRYAVLPREVVCTE |
| Q969N2 | PCPLASESRVYVDITTYNQDNETLEVHPPPTTTYQDVILGT |
| Q969N2 | VILGTRKTYAIYDLLDTAMINNSRNLNIQLKWKRPPENEAP |
| Q9UKJ1 | SFYSTRPPSIHKDYVNRLFLNWTEGQKSGFLRISNLQKQDQ |
| P12273 | KYTACLCDDNPKTFYWDFYTNRTVQIAAVVDVIRELGICPD |
| Q13563 | FWKFTEGSLLDGLYWKMQPSNQTEADNRSFIFYENLLLGVP |
| Q13563 | GSLLDGLYWKMQPSNQTEADNRSFIFYENLLLGVPRIRQLR |
| Q13563 | FIFYENLLLGVPRIRQLRVRNGSCSIPQDLRDEIKECYDVY |
| Q13563 | KECYDVYSVSSEDRAPFGPRNGTAWIYTSEKDLNGSSHWGI |
| Q13563 | RAPFGPRNGTAWIYTSEKDLNGSSHWGIIATYSGAGYYLDL |

| | |
|---|---|
| Q9XWV2 | KTDEELCAKRLATAYFHDEVNQTGWAFLEVDVISPKIPHYL |
| Q9XWV2 | LKWMEQEIRENPEDEYWQQVNLTVNQLFGLIHGYENQLGAE |
| Q9XWV2 | KRDHNTVTDMSSMIALMRSNNYTKDPLSKCDCNPPYSAENA |
| Q9XWV2 | CNPPYSAENAIACRSDLNPLNGTYPFKSLGFRDHGAIDVKV |
| Q9GL30 | EKTIQVKNVLDRKGDAYGFYNNSVKTTGWGILEIKAGYGSQ |
| Q9GL30 | DDFYLLSSGLVLLQTTNSVYNKTLLQHVVPQSLLAWQRVRV |
| Q9GL30 | YNSGTYNNQYMVLDLKKVNLNHSLDEGTLYIVEQIPTYVEY |
| Q9GL30 | AVLRRGYWPSYNIPFHEKVYNWSGYPILVKKLGLDYSYDLA |
| Q9GL30 | YAISGPTVQGGLPVFHWSRFNKTLHEGMPEAYNFDFITMKP |
| Q6P4A8 | EKTVQVKNVMDKNGDAYGFYNNSVKTTGWGILEIRAGYGSQ |
| Q6P4A8 | DDFYILSSGLILLQTTNSVFNKTLLKQVIPETLLSWQRVRV |
| Q6P4A8 | YNSGTYNNQYMVLDLKKVKLNHSLDKGTLYIVEQIPTYVEY |
| Q6P4A8 | DVLRKGYWPSYNVPFHEKIYNWSGYPLLVQKLGLDYSYDLA |
| Q6P4A8 | YAISGPTVQGGLPVFRWDRFNKTLHQGMPEVYNFDFITMKP |
| Q8VCI0 | SKKVEIKTVLDKNGDAYGYYNDSIKTTGWGILEIRAGYGSQ |
| Q8VCI0 | DDFYILSSGLILLQTTNSVYNKTLLKQVVPKTLLAWQRVRV |
| Q8VCI0 | HNSGTYNNQYMVLDLKKVTINRSLDKGTLYIVEQIPTYVEY |
| Q8VCI0 | NVLRKGYWASYNIPFHKTIYNWSGYPLLVHKLGLDYSYDLA |
| Q8VCI0 | YAISGPTVQDGLPPFNWNRFNETLHRGMPEVFDFNFVTMKP |
| O62146 | VLDGFDCRNQVAVARWQNAVNTTGWTFLEVETKENYCPQLQ |
| O62146 | LLSKTVLTYHLKNAQEDYCKNFTGYCSRLSDFLTENQKWIQ |
| O62146 | KWIQSSLETVAPDDLYWGAVNRTYHQVSGLIDAYEGREFKP |
| O62146 | LHPILYLNLNGDFYDLEKKLNKTRDPAFEQTGGKCSGLIKV |
| O62146 | DDFALQTSGLAVIETTISVFNTSLFENTKPVGQLPTWIRAI |
| O62146 | CNPPYSAEAGISARGDLNPANGTYEFPGQGHVNHGALDYKG |
| Q8NHP8 | VSAGQLLMVDGRHPDAVAWANLTNAIRETGWAFLELGTSGQ |
| Q8NHP8 | TNAIRETGWAFLELGTSGQYNDSLQAYAAGVVEAAVSEELI |
| Q8NHP8 | PLGFLLLQLSGDLEDLELALNKTKIKPSLGSGSCSALIKLL |
| Q8NHP8 | ELYQKTYWASYNIPSFETVFNASGLQALVAQYGDWFSYDGS |
| Q8NHP8 | AQYGDWFSYDGSPRAQIFRRNQSLVQDMDSMVRLMRYNDFL |
| Q8NHP8 | CNPQPNGENAISARSDLNPANGSYPFQALRQRSHGGIDVKV |
| Q3TCN2 | AASGQLRLEDGFHPDAVAWANLTNAIRETGWAYLDLSTNGR |
| Q3TCN2 | TNAIRETGWAYLDLSTNGRYNDSLQAYAAGVVEASVSEELI |
| Q3TCN2 | PLGFLLLQISGDLEDLEPALNKTNTKPSLGSGSCSALIKLL |
| Q3TCN2 | ELYKTTYWASYNIPYFETVFNASGLQALVAQYGDWFSYTKN |
| Q3TCN2 | CNPKPNAENAISARSDLNPANGSYPFQALHQRAHGGIDVKV |
| Q9BL07 | QILNDHSSAKFDYTYVSVCVNSTDETLLDIVYAKECKNAAS |
| Q9BL07 | AKECKNAASRVALGKYSNQVNTTGWGILEIETFASHSYDVQ |
| Q9BL07 | ELTRLQIYYHYRNTIETMCNNHTLFCKRLYIYLQQNLDWMR |
| Q9BL07 | DWMRSQVQANPPTDPFWRQVNLTFAQLTGIYDAYSKRNLTP |
| Q9BL07 | LHPIYMMQLAGDMFDLNKLLNKTADPMEYPEGGRCSGFVKL |

| | |
|---|---|
| Q9BL07 | DDYTITSAGLTSIETTIAIFNQTLYTDKFMKPEGQVHCWIR |
| Q9BL07 | KMRFRAWGGPPYDPLPVFDWNHTNLTNVRHFGQPDVWNFTY |
| Q9BL07 | FRAWGGPPYDPLPVFDWNHTNLTNVRHFGQPDVWNFTYVDL |
| Q9BL07 | FDWNHTNLTNVRHFGQPDVWNFTYVDLEWQLAAQVQLTPYD |
| Q8IV08 | CYDPCEAVLVESIPEGLDFPNASTGNPSTSQAWLGLLAGAH |
| Q8IV08 | LLAGAHSSLDIASFYWTLTNNDTHTQEPSAQQGEEVLRQLQ |
| Q63434 | CFLQVLAGLAVHSQGALSAGNNSTEMEVVPFNEVWGRSYCR |
| Q63434 | FLQVLAGLAVHSQGALSAGNNSTEMEVVPFNEVWGRSYCRP |
| Q63434 | SRCSGCCGDEGLHCVALKTANITMQILKIPPNRDPHSYVEM |
| P21755 | FASVLERHNKAAYLVVGDSANFTNWAAGQPNEADGTCVKAD |
| P82142 | FANVLERHNKEAYLVVGNSANFTNWAEGQPKKADGTCVKAD |
| Q7LZI1 | GDIAYNIKGCISSCPELSLSNRTHEVDRNELIKVECTDAVK |
| P21756 | FANVLERHNKAAYLVVGDSANFTNWAAGQPNEADGTCVKAD |
| P06868 | AVTESGHTCQRWSEQTPHKHNRTPENFPCKNLEENYCRNPN |
| P00747 | AVTVSGHTCQHWSAQTPHTHNRTPENFPCKNLDENYCRNPD |
| P06867 | SVTASGHTCQRWSAQSPHKHNRTPENFPCKNLEENYCRNPD |
| Q9R0E2 | DSDQLFYTKIFLNPEKREQINISLDHRCRIFQNLDGALDEV |
| Q9R0E2 | NDLWEVFSNPEDWKEKYIHENYTKALAGKLVETPCPDVYWF |
| Q9R0E2 | IALNRVGEDYEGGGCRFLRYNCSVRAPRKGWALLHPGRLTH |
| O00469 | TVATKESDGFHRFMQSAKYFNYTVKVLGQGEEWRGGDGINS |
| O00469 | DDDQLFYTKVYIDPLKREAINITLDHKCKIFQTLNGAVDEV |
| O00469 | NGCTLCEFDTVDLSAVDVHPNVSIGVFIEQPTPFLPRFLDI |
| O00469 | VFFDKAKHEIKTIKIVGPEENLSQAEARNMGMDFCRQDEKC |
| O00469 | VFMYISNRHEFGRLLSTANYNTSHYNNDLWQIFENPVDWKE |
| O00469 | IALNNVGEDFQGGGCKFLRYNCSIESPRKGWSFMHPGRLTH |
| Q9R0E1 | TVATAETEGYRRFLQSAEFFNYTVRTLGLGQEWRGGDVART |
| Q9R0E1 | NYLGNYVPNGWTPQGGCGFCNQTLRTLPGGQPPPRVLLAVF |
| Q9R0E1 | PDLWQIFDNPVDWREQYIHENYSRALDGEGLVEQPCPDVYW |
| Q20679 | IALNKKGRDYEGGGVRYIRYNCTVPADEVGYAMMFPGRLTH |
| P72186 | PPIVEKDVKTKEPSLEPKMYNSTWVINALQFIQEFGYDGSG |
| P72186 | WKDFTDEGFVDTSFSFSKVVNGTLIINTTFQVASGLTLNES |
| P72186 | EGFVDTSFSFSKVVNGTLIINTTFQVASGLTLNESTGLMEY |
| P72186 | VVNGTLIINTTFQVASGLTLNESTGLMEYVVKTVYVSNVTI |
| P72186 | LTLNESTGLMEYVVKTVYVSNVTIGNITSANGIYHFGLLPE |
| P72186 | STGLMEYVVKTVYVSNVTIGNITSANGIYHFGLLPERYFDL |
| P72186 | RYFDLNFDGDQEDFYPVLLVNSTGNGYDIAYVDTDLDYDFT |
| P72186 | AYVDTDLDYDFTDEVPLGQYNVTYDVAVFSYYYGPLNYVLA |
| P72186 | LSMYSGEWEVFSRLYGWDYTNVTTDTVQGVAPGAQIMAIRV |
| P72186 | EGDPYTGQKYTELDQGHGLVNVTKSWEILKAINGTTLPIVD |
| P72186 | LDQGHGLVNVTKSWEILKAINGTTLPIVDHWADKSYSDFAE |
| P72186 | EIYATEPWIKPFVSGSVILENNTEFVLRVKYDVEGLEPGLY |

| | |
|---|---|
| P72186 | VIEDEILNTIVIPEKFTPENNYTLTWYDINGPEMVTHHFFT |
| P72186 | ESGFLVRIYGVEITPSVWYINRTYLDTNTEFSIEFNITNIY |
| P72186 | SVWYINRTYLDTNTEFSIEFNITNIYAPINATLIPIGLGTY |
| P72186 | LDTNTEFSIEFNITNIYAPINATLIPIGLGTYNASVESVGD |
| P72186 | ITNIYAPINATLIPIGLGTYNASVESVGDGEFFIKGIEVPE |
| P72186 | QMTLDNGNIKLDKDSIILGSNESVVVTANITIDRDHPTGVY |
| P72186 | IKLDKDSIILGSNESVVVTANITIDRDHPTGVYSGIIEIRD |
| P72186 | PTGVYSGIIEIRDNEVYQDTNTSIAKIPITLVIDKADFAVG |
| P72186 | DKADFAVGLTPAEGVLGEARNYTLIVKHALTLEPVPNATVI |
| P72186 | GEARNYTLIVKHALTLEPVPNATVIIGNYTYLTDENGTVTF |
| P72186 | LIVKHALTLEPVPNATVIIGNYTYLTDENGTVTFTYAPTKL |
| P72186 | LEPVPNATVIIGNYTYLTDENGTVTFTYAPTKLGSDEITVI |
| P72186 | PEITEEDINEPKLAMSSPEANATIVSVEMESEGGVKKTVTV |
| P72186 | SVEMESEGGVKKTVTVEITINGTANETATIVVPVPKKAENI |
| P72186 | ESEGGVKKTVTVEITINGTANETATIVVPVPKKAENIEVSG |
| P72186 | RLYSQKFDELYQKALELGVDNETLALALSYHEKAKEYYEKA |
| P55058 | QELETITIPDLRGKEGHFYYNISEVKVTELQLTSSELDFQP |
| P55058 | QLTSSELDFQPQQELMLQITNASLGLRFRRQLLYWFFYDGG |
| P55058 | LGLRFRRQLLYWFFYDGGYINASAEGVSIRTGLELSRDPAG |
| P55058 | VSIRTGLELSRDPAGRMKVSNVSCQASVSRMHAAFGGTFKK |
| P55058 | ASTSNLDMDFRGAFFPLTERNWSLPNRAVEPQLQEEERMVY |
| P55058 | ALRGKALRTQLDLRRFRIYSNHSALESLALIPLQAPLKTML |
| P55065 | QELETITIPDVYGAKGHFYYNISDVRVTQLHLISSELHFQP |
| P55065 | TQLHLISSELHFQPDQDLLLNISNASLGLHFRRQLLYWFLY |
| P55065 | HLISSELHFQPDQDLLLNISNASLGLHFRRQLLYWFLYDGG |
| P55065 | LGLHFRRQLLYWFLYDGGYINASAEGVSIRTGLQLSQDSSG |
| P55065 | VSIRTGLQLSQDSSGRIKVSNVSCEASVSKMNMAFGGTFRR |
| P55065 | VSNGNLDMEFRGAFFPLKEDNWSLPNRAVEPQLEDDERMVY |
| P55065 | TLRGKALRVKLDLRRFQIYSNQSALESLALIPLQAPLKTLL |
| Q9UIW2 | HEQTGEVYVGAVNRIYKLSGNLTLLRAHVTGPVEDNEKCYP |
| Q9UIW2 | LYLKSKETGKKFASVDFVFYNCSVHQSCLSCVNGSFPCHWC |
| Q9UIW2 | ASVDFVFYNCSVHQSCLSCVNGSFPCHWCKYRHVCTHNVAD |
| Q9UIW2 | KYRHVCTHNVADCAFLEGRVNVSEDCPQILPSTQIYVPVGV |
| Q9UIW2 | SAPIIININRAQLTNPEVKYNYTEDPTILRIDPEWSINSGG |
| Q9UIW2 | SPLILKGRNLLPPAPGNSRLNYTVLIGSTPCTLTVSETQLL |
| Q9UIW2 | IGSTPCTLTVSETQLLCEAPNLTGQHKVTVRAGGFEFSPGT |
| P70206 | HEQTGEVYVGAVNRIYKLSGNLTLLRAHVTGPVEDNEKCYP |
| P70206 | LYLKSKETGKKFASVDFVFYNCSVHQSCLACVNGSFPCHWC |
| P70206 | ASVDFVFYNCSVHQSCLACVNGSFPCHWCKYRHVCTNNAAD |
| P70206 | KYRHVCTNNAADCAFLEGRVNMSEDCPQILPSTHIYVPVGV |
| P70206 | SAPIVININRAQLSNPEVKYNYTEDPTILRIDPEWSINSGG |

| | |
|---|---|
| P70206 | SPLILKGRNLLPPAPGNSRLNYTVLIGSTPCILTVSETQLL |
| P70206 | IGSTPCILTVSETQLLCEAPNLTGQHKVTVRAGGFEFSPGM |
| O75051 | HQGTGAVYVGAINRVYKLTGNLTIQVAHKTGPEEDNKSCYP |
| O75051 | YKLTGNLTIQVAHKTGPEEDNKSCYPPLIVQPCSEVLTLTN |
| O75051 | YRLLQAAYLAKPGDSLAQAFNITSQDDVLFAIFSKGQKQYH |
| O75051 | LSLVVSDAPDLSAGIACAFGNLTEVEGQVSGSQVICISPGP |
| O75051 | KYRNLCTHDPTTCSFQEGRINISEDCPQLVPTEEILIPVGE |
| O75051 | GYECVLNIQGAIHRVPALRFNSSSVQCQNSSYQYDGMDISN |
| O75051 | IGETPCAVTVSETQLLCEPPNLTGQHKVMVHVGGMVFSPGS |
| P70207 | HRRTGAVYVGAINRVYKLTGNLTIQVAHKTGPEEDNKACYP |
| P70207 | DDLFILVEPSHKKEHYLSSVNKTGTMYGVIVRSEGEDGKLF |
| P70207 | YRLLQAAYLAKPGEALAQAFNISSDEDVLFAIFSKGQKQYH |
| P70207 | LSLVVNDAPNLSEGIACAFGNLTEVEGQVSGSQVICISPGP |
| P70207 | KYRNLCTHDPTTCSFQEGRINVSEDCPQLVPTEEILIPVGE |
| P70207 | GYECVLSIQGAVHRVPALRFNSSSVQCQNSSYQYDGMDISN |
| P70207 | SPIILKGKNLCPPASGGAKLNYTVMIGETPCTVTVSETQLL |
| P70207 | IGETPCTVTVSETQLLCEPPNLTGQHKVMVHVGGMVFSPGS |
| O15031 | KRLVECGSLFKGICALRALSNISLRLFYEDGSGEKSFVASN |
| O15031 | FEDGPYVFFVFNQQDKHPARNRTLLARMCREDPNYYSYLEM |
| O15031 | YPLGSRDGLRGTAVLQRGGLNLTAVTVAAENNHTVAFLGTS |
| O15031 | TAVLQRGGLNLTAVTVAAENNHTVAFLGTSDGRILKVYLTP |
| O15031 | SHWLWSRSKSCVAVTSAQPQNMSRRAQGEVQLTVSPLPALS |
| O15031 | VTMQESGTFAFRTPKLSHDANETLPLHLYVKSYGKNIDSKL |
| O15031 | HLYVKSYGKNIDSKLHVTLYNCSFGRSDCSLCRAANPDYRC |
| O15031 | PDYRCAWCGGQSRCVYEALCNTTSECPPPVITRIQPETGPL |
| O15031 | LGSNLGVQAGDIQRISVAGRNCSFQPERYSVSTRIVCVIEA |
| O15031 | NPVLRAFEPLRSFASGGRSINVTGQGFSLIQRFAMVVIAEP |
| O15031 | PREAESLQPMTVVGTDYVFHNDTKVVFLSPAVPEEPEAYNL |
| O15031 | HNDTKVVFLSPAVPEEPEAYNLTVLIEMDGHRALLRTEAGA |
| O15031 | RALLRTEAGAFEYVPDPTFENFTGGVKKQVNKLIHARGTNL |
| B2RXS4 | KRLVECGSLFKGICALRAMSNISVRLFYEDGSGEKSFVASN |
| B2RXS4 | FEDDFYVFFVFNHQDKHPAKNRTLLARMCKDDPSYYSYVEM |
| B2RXS4 | YPLGSRDGLVATAVLHRGGLNLTAVTVTAENDHTVAFLGTS |
| B2RXS4 | YGSIPVDINKKIKQDLALSGNLSSLYAMTQDKVFRLPVQEC |
| B2RXS4 | AYRCVWCRGQNRCVYEALCSNVTSECPPPVITRIQPETGPL |
| B2RXS4 | QPLSVEPRQGPQAGGTTLTINGTHLDTGSKEDVRVTLNDVP |
| B2RXS4 | RREAGALERVTVEGMEYVFYNDTKVVFLSPAVPEEPEAYNL |
| B2RXS4 | YNDTKVVFLSPAVPEEPEAYNLTVLIRMDGHCAPLRTEAGV |
| O43157 | ALWAGWVLTLQPLPPTAFTPNGTYLQHLARDPTSGTLYLGA |
| O43157 | GASGASALCAFPLDEVDRLANRTRDACYTREGRAEDGTEVA |
| O43157 | WLWSFQPELGCLQVAAMSPANISREETREVFLSVPDLPPLW |

| | |
|---|---|
| O43157 | KVHSIFPARGPRAGGTRLTLNGSKLLTGRLEDIRVVVGDQP |
| O43157 | WFGATERRLQRGQFKYTLDPNITSAGPTKSFLSGGREICVR |
| O43157 | TACSLGPSCSSQQFEEPCHVNSSQLITCRTPALPGLPEDPW |
| O60486 | DQLDYSLEHSLSRLYRDQAGNCTEPVSLAPPARPRPGSSFS |
| O60486 | GLLLTGWTFDRGACEVRPLGNLSRNSLRNGTEVVSCHPQGS |
| O60486 | FDRGACEVRPLGNLSRNSLRNGTEVVSCHPQGSTAGVVYRA |
| O60486 | GRLKLCEGAGSLHFVDAFLWNGSIYFPYYPYNYTSGAATGW |
| O60486 | LHFVDAFLWNGSIYFPYYPYNYTSGAATGWPSMARIAQSTE |
| O60486 | IASSTLIHSDLTSVYGTVVMNRTVLFLGTGDGQLLKVILGE |
| O60486 | RTVLFLGTGDGQLLKVILGENLTSNCPEVIYEIKEETPVFY |
| O60486 | SPRHSKCMVKNVDSSRELCQNKSQPNRTCTCSIPTRATYKD |
| O60486 | TRATYKDVSVVNVMFSFGSWNLSDRFNFTNCSSLKECPACV |
| O60486 | QCPVAVEKTSGGGRPKENKGNRTNQALQVFYIKSIEPQKVS |
| O60486 | VSTLGKSNVIVTGANFTRASNITMILKGTSTCDKDVIQVSH |
| O60486 | IALPHCSLIFPATTWISGGQNITMMGRNFDVIDNLIISHEL |
| O60486 | GRNFDVIDNLIISHELKGNINVSEYCVATYCGFLAPSLKSS |
| O60486 | CVATYCGFLAPSLKSSKVRTNVTVKLRVQDTYLDCGTLQYR |
| O60486 | VESEVDTELEVKIQKENDNFNISKKDIEITLFHGENGQLNC |
| O60486 | FNISKKDIEITLFHGENGQLNCSFENITRNQDLTTILCKIK |
| Q8CJH3 | VFWAGCVVTLRSPLPAAFTANGTHLQHLARDPTTGTLYVGA |
| Q8CJH3 | FLFQLSPGLQLEAVVSTGPVNDSRDCLPPVIPDECPQAQPT |
| Q8CJH3 | GASGTSVLCAFPLDEVDQLANYTRDACYTREGRAENGTKVA |
| Q8CJH3 | VDQLANYTRDACYTREGRAENGTKVADIAYDVLSDCAQLPV |
| Q8CJH3 | WLWSFQPELGCLRVVAVSPANISREERREVFLSVPGLPSLW |
| Q8CJH3 | LFGATERRLQHGQFKYTSDPNVTSVGPSKSFFSGGREIWVR |
| Q8CJH3 | QGLAQKQHVVPEKFEEPCLVNSSHLLMCRTPALPGPPWDSG |
| Q9Y4D7 | GAAGTVYLAAVNRLYQLSGANLSLEAEAAVGPVPDSPLCHA |
| Q9Y4D7 | GLVVVCGSIYQGFCQLRRRGNISAVAVRFPPAAPPAEPVTV |
| Q9Y4D7 | PPAEPVTVFPSMLNVAANHPNASTVGLVLPPAAGAGGSRLL |
| Q9Y4D7 | GSRLLVGATYTGYGSSFFPRNRSLEDHRFENTPEIAIRSLD |
| Q9Y4D7 | KATPVFRAPGLTSVAVASVNNYTAVFLGTVNGRLLKINLNE |
| Q9Y4D7 | NNYTAVFLGTVNGRLLKINLNESMQVVSRRVVTVAYGEPVH |
| Q9Y4D7 | DAYCGWCALETRCTLQQDCTNSSQQHFWTSASEGPSRCPAM |
| Q9Y4D7 | QDHVTVEMSVRVNGRNIVKANFTIYDCSRTAQVYPHTACTS |
| Q9Y4D7 | SCLSAQWPCFWCSQQHSCVSNQSRCEASPNPTSPQDCPRTL |
| Q9Y4D7 | QGAALECSFGLEEIFEAVWVNESVVRCDQVVLHTTRKSQVF |
| Q9Y4D7 | EEIVCVTGPAPGPLSGVVTVNASKEGKSRDRFSYVLPLVHS |
| Q9Y4D7 | TRITIHGNDLHVGSELQVLVNDTDPCTELMRTDTSIACTMP |
| Q9Y4D7 | ALPAPVPVCVRFERRGCVHGNLTFWYMQNPVITAISPRRSP |
| Q9Y4D7 | SPVSGGRTITVAGERFHMVQNVSMAVHHIGREPTLCKVLNS |
| Q9Y4D7 | QNVSMAVHHIGREPTLCKVLNSTLITCPSPGALSNASAPVD |

| | |
|---|---|
| Q9Y4D7 | TLCKVLNSTLITCPSPGALSNASAPVDFFINGRAYADEVAV |
| Q9Y4D7 | IGQVSCDIQIVSDRIIHCSVNESLGAAVGQLPITIQVGNFN |
| Q9Y4D7 | NESLGAAVGQLPITIQVGNFNQTIATLQLGGSETAIIVSIV |
| P18632 | IIHGLYLYGCSTSVLGNVLINESFGVEPVHPQDGDALTLRT |
| P18632 | GDALTLRTATNIWIDHNSFSNSSDGLVDVTLTSTGVTISNN |
| P18632 | AIGGSSNPTILSEGNSFTAPNESYKKQVTIRIGCKTSSSCS |
| P83947 | ELMDLSKERVVDSISILFHQNLTTRSHEDLHVWLSGVLTNH |
| P83947 | IDAYQDTLYPHNYRQFYRDRNITGTVDFIFGNAAVVFQNCN |
| P85076 | KKKTNLMFIGDGIGKTWIKGNRSVVDGWTTFRSSTVAVVGT |
| P85076 | GDFALTTLYYREYKNTGPGSNTTARVTWPGYAVTTNETEVI |
| P85076 | TGPGSNTTARVTWPGYAVTTNETEVIQFTVGNFIQGSQWLT |
| P07738 | CSWVDQKLNSEGMEEARNCGKQLKALNFEFDLVFTSVLNRS |
| P07738 | DRRYKVCDVPLDQLPRSESLKDVLERLLPYWNERIAPEVLR |
| P07738 | VLRGKTILISAHGNSSRALLKHLEGISDEDIINITLPTGVP |
| Q9TQZ3 | VSTIVSQWMVGNGHATDLWQNCSTSLMGSVQHCFSSSANEW |
| Q8TEM1 | AAAKLNIPKVLLPFTRATRVNFTLEASEGCYRWLSTRPEVA |
| Q8TEM1 | QSSLVLGHRSIRMQGASRLPNSTIYVVEPGYLGFTVHPGDR |
| Q8TEM1 | NIRIETVLPAEFFEVLSSSQNGSYHRIRALKRGQTAIDAAL |
| Q8TEM1 | PWQPKTGAYQYTIRAHGGSGNFSWSSSSHLVATVTVKGVMT |
| Q8TEM1 | EMLFEGGPRPWILEPSKFFQNVTAEDTDSIGLALFAPHSSR |
| Q8TEM1 | SHRNPRLDLAAYDQEGRRFDNFSSLSIQWESTRPVLASIEP |
| Q8TEM1 | NHPGIQAELRIREGSGYFFLNTSTADVVKVAYQEARGVAMV |
| Q8TEM1 | LKLRAASPIITLVALDEALDNYTITFLIRGVAIGQTSLTAS |
| Q8TEM1 | MQVTSEGGPQPQSNILFSISNESVALVSAAGLVQGLAIGNG |
| Q8TEM1 | SNESVALVSAAGLVQGLAIGNGTVSGLVQAVDAETGKVVII |
| Q8TEM1 | SGSMIGTSTIEVIAQEPFGANQTIIVAVKVSPVSYLRVSMS |
| Q8TEM1 | SVLNFATNRDDFVQIGKGPTNNTCVVRTVSVGLTLLRVWDA |
| Q7Z5L7 | RSVYLHNNKLADAGLPDNMFNGSSNVEVLILSSNFLRHVPK |
| Q7Z5L7 | LSSLRELYLQNNYLTDEGLDNETFWKLSSLEYLDLSSNNLS |
| Q7Z5L7 | LDNETFWKLSSLEYLDLSSNNLSRVPAGLPRSLVLLHLEKN |
| Q7Z5L7 | NQITGIGREDFATTYFLEELNLSYNRITSPQVHRDAFRKLR |
| Q52S86 | EKVSTNPTIATSDSKGIPDLNKSILPSATNSMKPDTPVTQT |
| Q52S86 | SITPALTSIITPTSPRQPSANSTTLKPPESSSESPDKSHTA |
| Q52S86 | GDDRIKCESPGRLTDKMLLLNLTRSGLCAGNNSDDKLITLL |
| Q52S86 | GRLTDKMLLLNLTRSGLCAGNNSDDKLITLLCRAAKATFNP |
| P29150 | KMTVQSTIPSGSYADVYNARNMTRVFRPQSVQGSSLAEAQF |
| P14340 | ITYKCPFLKQNEPEDIDCWCNSTSTWVTYGTCTTTGEHRRE |
| P14340 | ETEAKQPATLRKYCIEAKLTNTTTDSRCPTQGEPSLNEEQD |
| P14340 | ENLEYTIVITPHSGEEHAVGNDTGKHGKEIKITPQSSITEA |
| P14340 | AIKDNRAVHADMGYWIESALNDTWKIEKASFIEVKSCHWPK |
| P14340 | WTLYAVATTFVTPMLRHSIENSSVNVSLTAIANQATVLMGL |

| | |
|---|---|
| P14340 | AVATTFVTPMLRHSIENSSVNVSLTAIANQATVLMGLGKGW |
| P14340 | LTLATGPISTLWEGNPGRFWNTTIAVSMANIFRGSYLAGAG |
| P09866 | VTYKCPLLVNTEPEDIDCWCNLTSTWVMYGTCTQSGERRRE |
| P09866 | KTTAKEVALLRTYCIEASISNITTATRCPTQGEPYLKEEQD |
| P09866 | ENLEYTVVVTVHNGDTHAVGNDTSNHGVTAMITPRSPSVEV |
| P09866 | DLKYSWKTWGKAKIFTPEARNSTFLIDGPDTSECPNERRAW |
| P09866 | AIKDQKAVHADMGYWIESSKNQTWQIEKASLIEVKTCLWPK |
| P09866 | WTLYAVATTILTPMLRHTIENTSANLSLAAIANQAAVLMGL |
| P09866 | AVATTILTPMLRHTIENTSANLSLAAIANQAAVLMGLGKGW |
| P09866 | LTLATGPILTLWEGNPGRFWNTTIAVSTANIFRGSYLAGAG |
| P05769 | IGFAAALKLSTFQGKIMMTVNATDIADVIAIPTPKGPNQCW |
| P05769 | DIKYEVGVFVHGSTDSTSHGNYSTQIGANQAVRFTISPNAP |
| P05769 | ELDIGWKAWGKSLLFAAELANSTFVVDGPETAECPNSKRAW |
| P05769 | IEDFGFGITSTRGWLKLREENTSECDSTIIGTAVKGNHAVH |
| P05769 | AVKGNHAVHSDLSYWIESGLNGTWKLERAIFGEVKSCTWPE |
| P05769 | TTIREAGILCTAAALTLWDNNASAAWNSTTATGLCHVMRGS |
| P05769 | GILCTAAALTLWDNNASAAWNSTTATGLCHVMRGSWIAGAS |
| P07720 | DGTTVIRAEGKDAATQVRVENGTCVILATDMGSWCDDSLTY |
| P07720 | ANKIVYTVKVEPHTGDYVAANETHSGRKTASFTVSSERTIL |
| P07720 | MAMWRSSATELNLALVEGDANLTVVVDKLDPTDYRGGIPSL |
| P07720 | AVKNGMAVHTDQSLWMKSVRNDTGTYIVELLVTDLRNCSWP |
| P07720 | KSVRNDTGTYIVELLVTDLRNCSWPASHTIDNAEVVDSELF |
| P08563 | DVSCEGLGAWVPTAPCARIWNGTQRACTFWAVNAYSSGGYA |
| P08563 | VKFHTETRTVWQLSVAGVSCNVTTEHPFCNTPHGQLEVQVP |
| P08563 | HGQLEVQVPPDPGDLVEYIMNYTGNQQSRWGLGSPNCHGPD |
| P03315 | TPEGSEEWSAPLITAMCVLANATFPCFQPPCVPCCYENNAE |
| P03315 | EDNVDRPGYYDLLQAALTCRNGTRHRRSVSQHFNVYKATRP |
| P03315 | LLSQQSGNVKITVGGKKVKYNCTCGTGNVGTTNSDMTINTC |
| P03315 | VPRADEPARKGKVHIPFPLDNITCRVPMAREPTVIHGKREV |
| P03315 | AYKAHTASLKAKVRVMYGNVNQTVDVYVNGDHAVTIGGTQF |
| Q9Y6A1 | HDVAAPLSPHSQEVSCYIDYNISMPAQNLWRLEIVNRGSDT |
| Q9Y6A1 | RGSDTDVWKTILSEVRFVHVNTSAVLKLSGAHLPDWGYRQL |
| Q9Y6A1 | QEQRERERELHSPAQVDVSRNLSFMARFSELQWRMLALRSD |
| P27169 | YYSPSEVRVVAEGFDFANGINISPDGKYVYIAELLAHKIHV |
| P27169 | KYVYIAELLAHKIHVYEKHANWTLTPLKSLDFNTLVDNISV |
| P27169 | KHANWTLTPLKSLDFNTLVDNISVDPETGDLWVGCHPNGMK |
| P27169 | VLRIQNILTEEPKVTQVYAENGTVLQGSTVASVYKGKLLIG |
| P52430 | KYVYIAELLAHKIHVYEKHANWTLTPLKVLNFDTLVDNISV |
| P52430 | KHANWTLTPLKVLNFDTLVDNISVDPVTGDLWVGCHPNGMR |
| P52430 | VLRIQNILSEDPKITVVYAENGTVLQGTTVASVYKGKLLIG |
| Q15165 | IYVADILAHEIHVLEKHTNMNLTQLKVLELDTLVDNLSIDP |

| | |
|---|---|
| Q15165 | KHTNMNLTQLKVLELDTLVDNLSIDPSSGDIWVGCHPNGQK |
| Q15165 | VLRIQNILCEKPTVTTVYANNGSVLQGSSVASVYDGKLLIG |
| Q15166 | LLGVGLSLVGEMFLAFRERVNASREVEPVEPENCHLIEELE |
| Q15166 | KHDNWDLTQLKVIQLGTLVDNLTVDPATGDILAGCHPNPMK |
| Q15166 | VLRIQNVLSEKPRVSTVYANNGSVLQGTSVASVYHGKILIG |
| Q15063 | PGVTNILKTTQGSKIFLKEVNDTLLVNELKSKESDIMTTNG |
| P13686 | DRSLRKVPWYVLAGNHDHLGNVSAQIAYSKISKRWNFPSPF |
| P13686 | SKRWNFPSPFYRLHFKIPQTNVSVAIFMLDTVTLCGNSDDF |
| P29288 | DRALRNIPWYVLAGNHDHLGNVSAQIAYSKISKRWNFPSPY |
| P29288 | SKRWNFPSPYYRLRFKVPRSNITVAIFMLDTVMLCGNSDDF |
| P09889 | DPSLRNVPWHVLAGNHDHLGNVSAQIAYSKISKRWNFPSPY |
| P09889 | SKRWNFPSPYYRLRFKIPRSNVSVAIFMLDTVTLCGNSDDF |
| P80366 | SEKNGRKRIAKGKMSTYRFFNYSSGFIHHTTIRKLKYNTKY |
| P80366 | HTTIRKLKYNTKYYYEVGLRNTTRRFSFITPPQTGLDVPYT |
| P80366 | GLDVPYTFGLIGDLGQSFDSNTTLSHYELSPKKGQTVLFVG |
| P80366 | AYQPWIWTAGNHEIEFAPEINETEPFKPFSYRYHVPYEASQ |
| P80366 | QPEYSAFREASFGHGMFDIKNRTHAHFSWNRNQDGVAVEAD |
| P11117 | GMLQHWELGQALRQRYHGFLNTSYHRQEVYVRSTDFDRTLM |
| P11117 | SAEANLAGLFPPNGMQRFNPNISWQPIPVHTVPITEDRLLK |
| P11117 | TEDRLLKFPLGPCPRYEQLQNETRQTPEYQNESSRNAQFLD |
| P11117 | GPCPRYEQLQNETRQTPEYQNESSRNAQFLDMVANETGLTD |
| P11117 | QTPEYQNESSRNAQFLDMVANETGLTDLTLETVWNVYDTLF |
| P11117 | YQQAEKARLQGGVLLAQIRKNLTLMATTSQLPKLLVYSAHD |
| P11117 | GEQAPYASCHIFELYQEDSGNFSVEMYFRNESDKAPWPLSL |
| P11117 | HIFELYQEDSGNFSVEMYFRNESDKAPWPLSLPGCPHRCPL |
| P15309 | GMEQHYELGEYIRKRYRKFLNESYKHEQVYIRSTDVDRTLM |
| P15309 | QDLFGIWSKVYDPLYCESVHNFTLPSWATEDTMTKLRELSE |
| P15309 | CHLTELYFEKGEYFVEMYYRNETQHEPYPLMLPGCSPSCPL |
| P20646 | GMGQHYELGSYIRRRYGRFLNNSYKHDQVYIRSTDVDRTLM |
| P20646 | QDLFEIWSRLYDPLYCESVHNFTFRTWATEDAMTKLKELSE |
| P20646 | CHIMELYQDNGGTFVEMYYRNETQNEPYPLTLPGCTHSCPL |
| P15693 | GVKANYKTIGVSAAARFNQCNSTFGNEVFSVMHRAKKAGKS |
| P15693 | AVTRLMGLFEPTEMKYDVNRNASADPSLAEMTEVAVRLLSR |
| P15693 | YTSILYGNGPGYVLNSGNRPNVTDAESGDVNYKQQAAVPLS |
| P09242 | GVKANEGTVGVSAATERTRCNTTQGNEVTSILRWAKDAGKS |
| P09242 | HNIKDIDVIMGGGRKYMYPKNRTDVEYELDEKARGTRLDGL |
| P09242 | DLISIWKSFKPRHKHSHYVWNRTELLALDPSRVDYLLGLFE |
| P09242 | VDYLLGLFEPGDMQYELNRNNLTDPSLSEMVEVALRILTKN |
| P09242 | FTAILYGNGPGYKVVDGERENVSMVDYAHNNYQAQSAVPLR |
| P09242 | PGYKVVDGERENVSMVDYAHNNYQAQSAVPLRHETHGGEDV |
| P05187 | GVKGNFQTIGLSAAARFNQCNTTRGNEVISVMNRAKKAGKS |

| | |
|---|---|
| P05187 | DGKNLVQEWLAKRQGARYVWNRTELMQASLDPSVTHLMGLF |
| P05186 | GVKANEGTVGVSAATERSRCNTTQGNEVTSILRWAKDAGKS |
| P05186 | HNIRDIDVIMGGGRKYMYPKNKTDVEYESDEKARGTRLDGL |
| P05186 | DLVDTWKSFKPRYKHSHFIWNRTELLTLDPHNVDYLLGLFE |
| P05186 | VDYLLGLFEPGDMQYELNRNNVTDPSLSEMVVVAIQILRKN |
| P05186 | FTAILYGNGPGYKVVGGERENVSMVDYAHNNYQAQSAVPLR |
| P83456 | GVKANEGTVGVSAAAVRSQANTTQGNEVTSILRWAKDAGKS |
| P83456 | ENIPDIDVIMGGGRKYMYPKNTTDVEYPGQPKHSGTRKDGR |
| P83456 | FTAILYGNGPGYKLVNGARENVSTVDYQDNSYLAQAAVPLS |
| P11491 | GWQYVGDRKNFDSLLKSHGENVTLPFLGLFADNDIPFEIDR |
| P11491 | TSRQVTASYPQYVWYPQVLANATHSGEFLKRKLVDFVHEHK |
| P10619 | YLESPAGVGFSYSDDKFYATNDTEVAQSNFEALQDFFRLFP |
| P10619 | MWHQALLRSGDKVRMDPPCTNTTAASTYLNNPYVRKALNIP |
| P16675 | YIESPAGVGFSYSDDKMYVTNDTEVAENNYEALKDFFRLFP |
| P16675 | RFPEALMRSGDKVRLDPPCTNTTAPSNYLNNPYVRKALHIP |
| O76840 | GATNIKIQEARKSTNNLALKNGSDHFYLNGNGLIQVEKEVE |
| O76840 | LYCIDGKNKGRVEDDLCEENNATKPEFEKSCETVDCEAEWF |
| O76840 | RVVYCHQVFANGRRVTVEDGNCTVERPPVKQTCNRFACPEW |
| O76840 | LGPCEGLTFVTGEWNLCTRCNDTEETREVTCKDSQGRAYPL |
| O76840 | EVTCKDSQGRAYPLEKCLVDNSTEIPTDTRSCATQPPCEYE |
| O76840 | GGLEVVDEGHCQAEKPEGKTNCTNEEKCTGTWYTSSWSECT |
| O76840 | DCNVDDCPTCVDSEFGCCPDNSTFATGEFNFGCSNCSETEF |
| O76840 | FGCCPDNSTFATGEFNFGCSNCSETEFGCCADNVTVATGPN |
| O76840 | GEFNFGCSNCSETEFGCCADNVTVATGPNSKGCEEFVESPL |
| O76840 | VECATIAPITALLGDGELIGNDTDASNETIHCSKTEFGCCP |
| O76840 | APITALLGDGELIGNDTDASNETIHCSKTEFGCCPDWYTAA |
| O76840 | YTAASGKGNEGCPSFTLGGCNETQFGCCHDDVTLARGANLE |
| O76840 | PCELSDFGCCPDGETAALGKNGTGCGENCLTTKFGCCPDGK |
| O76840 | TTTPPFLMGGTVAPHKIAACNQTQESGTVCGAGYKLAWHYD |
| O76840 | NAFSSKADCESLCRVETLWSNNTDFCTLERSAGPCTDSISM |
| O76840 | SESERVGQLTSASTPVIYIVNKTAIFVGNTFRIRCNSYGVL |
| O76840 | VIKRLPGHRTTSRPMLTPSKNFSLGTPPTPSPSTVSTTPFR |
| O76840 | SAPSDARVSRPTSNSCMDVGNASTCDLIVKNGLCGKKRYGT |
| Q868Z9 | GATNIRIEETVPSSNYLACRNHSGHYYLNGDWRIDFPRPMF |
| Q868Z9 | LTCSGPISESLFIVMLVQEKNISLDYEYSIPESLSHSQQDT |
| Q868Z9 | HWVEGEWSKCSKGCGSDGFQNRSITCERISSSGEHTVEEDA |
| Q868Z9 | GKCSKPCGGGERVREVLCLSNGTKSVNCDEEKVEPLSEKCN |
| Q868Z9 | EASASESTDVSGASDSTGSTNASDSTPESSTEASSSTDDST |
| Q868Z9 | TPESSTEASSSTDDSTDSSDNSSNVSESSTEASSSSVSDSN |
| Q868Z9 | SSTEASSSTDDSTDSSDNSSNVSESSTEASSSSVSDSNDSS |
| Q868Z9 | VSDSNDSSDGSTDGVSSTTENSSDSTSDATSDSTASSDSTD |

| | |
|---|---|
| Q868Z9 | SDNTDITTDGSTDESTDGSSNASTEGSTEGASEDTTISTES |
| Q868Z9 | RGPNNEGCECHYTPYGCCPDNKSAATGYNQEGCACETTQYG |
| Q868Z9 | NVQEPPQKACGLPKETGTCNNYSVKYYFDTSYGGCARFWYG |
| Q868Z9 | CEQPVESGPCAGNFERWYYDNETDICRPFTYGGCKGNKNNY |
| Q868Z9 | QCSQPADPGQCDKWALHWNYNETEGRCQSFYYGGCGGNDNR |
| Q868Z9 | PVEHDTSKCFLAFEPGNCYNNVTRWFYNSAEGLCDEFVYTG |
| Q868Z9 | EPSAPTYSVCAEPPEAGECDNRTTAWFYDSENMACTAFTYT |
| Q868Z9 | QFAPVCRDIYKPGECPALSANASGCARECYTDADCRGDNKC |
| Q868Z9 | QTAIGGIAVLRCFATGNPAPNITWSLKNLVINTNKGRYVLT |
| Q868Z9 | EVALQVTEPVSQPAYIYGDKNVTQIVELNRPAVIRCPAGGF |
| Q868Z9 | SYTPGSTIVMSCSVQGYPEPNVTWIKDDVPLYNNERVQITY |
| Q868Z9 | DSGKYTCRASNAYTYANGEANVSIQSVVPVSPECVDNPYFA |
| P45478 | QERLVQAEYWHDPIREDIYRNHSIFLADINQERGVNESYKK |
| P45478 | EDIYRNHSIFLADINQERGVNESYKKNLMALKKFVMVKFLN |
| P45478 | NESYKKNLMALKKFVMVKFLNDTIVDPVDSEWFGFYRSGQA |
| P50897 | QERLVQAEYWHDPIKEDVYRNHSIFLADINQERGINESYKK |
| P50897 | EDVYRNHSIFLADINQERGINESYKKNLMALKKFVMVKFLN |
| P50897 | NESYKKNLMALKKFVMVKFLNDSIVDPVDSEWFGFYRSGQA |
| Q9UMR5 | IVVHGLFDSSYSFRHLLEYINETHPGTVVTVLDLFDGRESL |
| Q9UMR5 | GQEFSICNYWHDPHHDDLYLNASSFLALINGERDHPNATVW |
| Q9UMR5 | DLYLNASSFLALINGERDHPNATVWRKNFLRVGHLVLIGGP |
| Q9UMR5 | GPDDGVITPWQSSFFGFYDANETVLEMEEQLVYLRDSFGLK |
| P02812 | NKPQGPPPPGKPQGPPPQGDNKSRSSRSPPGKPQGPPPQGG |
| P02812 | NKPQGPPPPGKPQGPPPQGDNKSQSARSPPGKPQGPPPQGG |
| P02812 | QPQGPPPPPGKPQGPPPQGGNKSQGPPPPGKPQGPPPQGGS |
| Q04118 | QPQRTPPPPGKPEGRPPQGGNQSQGPPPRPGKPEGPPPQGG |
| Q04118 | QSQGPPPRPGKPEGPPPQGGNQSQGPPPRPGKPEGQPPQGG |
| Q04118 | QSQGPPPRPGKPEGQPPQGGNQSQGPPPRPGKPEGPPPQGG |
| Q04118 | QSQGPPPRPGKPEGPPPQGGNQSQGPPPRPGKPEGPPPQGG |
| Q04118 | QSQGPPPRPGKPEGPPPQGGNQSQGPPPHPGKPEGPPPQGG |
| Q04118 | QSQGPPPHPGKPEGPPPQGGNQSQGPPPRPGKPEGPPPQGG |
| Q04118 | QSQGPPPRPGKPEGPPPQGGNQSQGPPPRPGKPEGPPPQGG |
| Q04118 | QSQGPPPRPGKPEGPPPQGGNQSQGPPPRPGKPEGSPSQGG |
| Q04118 | KPQGPPPHPGKPQGPPPQEGNKPQRPPPPGRPQGPPPPGGN |
| P10163 | QPQRPPPPPGKPQGPPPQGGNQSQGPPPPPGKPEGRPPQGG |
| P10163 | QSQGPPPPPGKPEGRPPQGGNQSQGPPPHPGKPERPPPQGG |
| P10163 | QSQGPPPHPGKPERPPPQGGNQSQGPPPHPGKPESRPPQGG |
| P10163 | QSQGPPPTPGKPEGPPPQGGNQSQGTPPPPGKPEGRPPQGG |
| P10163 | QSQGTPPPPGKPEGRPPQGGNQSQGPPPHPGKPERPPPQGG |
| P10163 | QSQGPPPHPGKPERPPPQGGNQSHRPPPPPGKPERPPPQGG |
| P10163 | QSHRPPPPPGKPERPPPQGGNQSQGPPPHPGKPEGPPPQEG |

| | |
|---|---|
| P10163 | QSQGPPPHPGKPEGPPPQEGNKSRSARSPPGKPQGPPQQEG |
| P51888 | IHYLYLQNNFITELPVESFQNATGLRWINLDNNRIRKIDQR |
| P51888 | AFIRLNYNKLTDRGLPKNSFNISNLLVLHLSHNRISSVPAI |
| P51888 | HNRISSVPAINNRLEHLYLNNNSIEKINGTQICPNDLVAFH |
| P51888 | PAINNRLEHLYLNNNSIEKINGTQICPNDLVAFHDFSSDLE |
| P13727 | DASKKDGAVESISVPDMVDKNLTCPEEEDTVKVVGIPGCQT |
| Q92954 | KNSAANRELQKKLKVKDNKKNRTKKKPTPKPPVVDEAGSGL |
| Q92954 | LSDETNICNGKPVDGLTTLRNGTLVAFRGHYFWMLSPFSPP |
| Q86XR5 | PLPPPPPPPPPPRLLSAPAPNSTSCPTEESWWSGLVIIIAV |
| P04156 | VYYRPMDEYSNQNNFVHDCVNITIKQHTVTTTTKGENFTET |
| P04156 | HDCVNITIKQHTVTTTTKGENFTETDVKMMERVVEQMCITQ |
| P04273 | VYYRPVDQYNNQNNFVHDCVNITIKQHTVTTTTKGENFTET |
| P04273 | HDCVNITIKQHTVTTTTKGENFTETDIKIMERVVEQMCTTQ |
| P04925 | VYYRPVDQYSNQNNFVHDCVNITIKQHTVTTTTKGENFTET |
| P04925 | HDCVNITIKQHTVTTTTKGENFTETDVKMMERVVEQMCVTQ |
| P55751 | FDERYAQGRGFITKAVNGCHNASLTTPEDKEQAQQIHHEDL |
| P55753 | FDERYAQGRGFITKAVNGCHNASLTTPEDKEQAQQIHHEDL |
| P05710 | PDKETFTCWWNPGTDGGLPTNYSLTYSKEGEKTTYECPDYK |
| P05710 | NSCFFSKQYTSIWKIYIITVNATNQMGSSSSDPLYVDVTYI |
| P05710 | SSSDPLYVDVTYIVEPEPPRNLTLEVKQLKDKKTYLWVKWS |
| P22393 | CQVSLRDLFDRAVILSHYIHNLSSEMFNEFDKRYAQGRGFM |
| P12420 | CQVSLRELFDRAVILSHYIHNLSSEMFNEFDKRYAQGRGFV |
| P01236 | CQVTLRDLFDRAVVLSHYIHNLSSEMFSEFDKRYTHGRGFI |
| P01238 | CQVSLRDLFDRAVILSHYIHNLSSEMFNEFDKRYAQGRGFI |
| P01240 | CQVSLRDLFDRAVMVSHYIHNLSSEMFNEFDKRYAQGKGFI |
| Q9QUG3 | YAANYWQFPDGIYYEGCSEANVTKEMLVTSCVNATQAANQA |
| Q9QUG3 | YYEGCSEANVTKEMLVTSCVNATQAANQAEFSREKQDSKLH |
| P00745 | RCDCAEGWEGRFCLHEVRFSNCSAENGGCAHYCMEEEGRRH |
| P00745 | MRRWESWEVDLDIKEVIIHPNYTKSTSDNDIALLRLAKPAT |
| P00745 | TQVGQETVVTGWGYRDETKRNRTFVLSFIKVPVVPYNACVH |
| P00745 | ETKRNRTFVLSFIKVPVVPYNACVHAMENKISENMLCAGIL |
| P04070 | SCDCRSGWEGRFCQREVSFLNCSLDNGGCTHYCLEEVGWRR |
| P04070 | LRRWEKWELDLDIKEVFVHPNYSKSTTDNDIALLHLAQPAT |
| P04070 | QETLVTGWGYHSSREKEAKRNRTFVLNFIKIPVVPHNECSE |
| P04070 | EAKRNRTFVLNFIKIPVVPHNECSEVMSNMVSENMLCAGIL |
| P33587 | KILKRDTDLEDELEPDPRIVNGTLTKQGDSPWQAILLDSKK |
| P33587 | LRRRDHWELDLDIKEILVHPNYTRSSSDNDIALLRLAQPAT |
| P33587 | QETVVTGWGYQSDRIKDGRRNRTFILTFIRIPLVARNECVE |
| O54990 | VLEEIKAMATAIKQTKDALQNMSSSLKSLQDAATQLNTNLS |
| O54990 | LQNMSSSLKSLQDAATQLNTNLSSVRNSIENSLSSSDCTSD |
| O54990 | PASKICDSIRPSLSSLGSSLNSSQLPSVDRELNTVTEVDKT |

| | |
|---|---|
| O54990 | LESLVKRGYTTIDEIPNTIQNQTVDVIKDVKNTLDSISSNI |
| O54990 | KDMSQSIPIEDMLLQVSHYLNNSNRYLNQELPKLEEYDSYW |
| O54990 | LLKEQWQFYLSGMLFNNPDINMTFEQVYRDCKRGRGIYAAF |
| O54990 | YRDCKRGRGIYAAFQLENVVNVSDHFNIDQISENINTELEN |
| O54990 | PEKVKKILASLDSVQHFLTNNVSLIVIGETKKFGKTILGYF |
| P82295 | EQLGQPHWPPVQYTVYRPTTNYTKAPPPPTSAMNPIFNFTH |
| P82295 | PTTNYTKAPPPPTSAMNPIFNFTHFLYDKVLYRDEPIPEGY |
| P82295 | YFMLGMVTYQGACAPLRDQENNTLFRQLDASIDLNHYLPPS |
| P82295 | SNKEVVQPLKMSSAIKACHANQTIFDMMRQHNIYDINDLTR |
| P82295 | INSEQFIQTRGKDYINALGGNLTNSIDQMIDDYIDMIIKEA |
| P82295 | LTNSIDQMIDDYIDMIIKEANESVGHCAPLSYIYYRGVDLI |
| P82295 | ERRREQQDYFEDASPSVSRGNRSGGDRGGGGGDGAPGSSSM |
| P27918 | TPLLPKYPPTVSMVEGQGEKNVTFWGRPLPRCEELQGQKLV |
| P07224 | VEKGSYYPGTGVAQFSINYKNESNPEAWQINVSLNIRPSAG |
| P07224 | GVAQFSINYKNESNPEAWQINVSLNIRPSAGTGVMLALVSD |
| P07225 | VEKGSYYPGSGIAQFHIDYNNVSSAEGWHVNVTLNIRPSTG |
| P07225 | GIAQFHIDYNNVSSAEGWHVNVTLNIRPSTGTGVMLALVSG |
| P07225 | VTLNIRPSTGTGVMLALVSGNNTVPFAVSLVDSTSEKSQDI |
| P22891 | DEFWRRYKGGSPCISQPCLHNGSCQDSIWGYTCTCSPGYEG |
| P22891 | GVIIRENFVLTTAKCSLLHRNITVKTYFNRTSQDPLMIKIT |
| P22891 | VLTTAKCSLLHRNITVKTYFNRTSQDPLMIKITHVHVHMRY |
| P22891 | DFAEHLLIPRTRGLLSGWARNGTDLGNSLTTRPVTLVEGEE |
| P22891 | NSLTTRPVTLVEGEECGQVLNVTVTTRTYCERSSVAAMHWM |
| P00744 | DEFWRTYMGGSPCASQPCLNNGSCQDSIRGYACTCAPGYEG |
| P00744 | GVLIQDNFVLTTATCSLLYANISVKTRSHFRLHVRGVHVHT |
| P00744 | MVPLRLRVTHVEPAECGRALNATVTTRTSCERGAAAGAARW |
| Q9CQW3 | DEFWRQYGGGSPCVSQPCLNNGTCEDHIRSYSCTCSPGYEG |
| Q9CQW3 | GVLLQEDFVLTTAKCSLLHSNISVKANVDQRIRIKSTHVHM |
| Q9CQW3 | DFAEHVLIPGTEGLLSGWMLNGTHLATTPMLLSVTQADGEE |
| Q9CQW3 | TTPMLLSVTQADGEECGQTLNVTVTTRTSCEKGSVVMGPWV |
| Q5FWE3 | EAPGTPKSLIPGPSDPGPAVNRTESPMGALQPDEAEEWPGR |
| P24158 | NYDAENKLNDVLLIQLSSPANLSASVATVQLPQQDQPVPHG |
| P24158 | AMGWGRVGAHDPPAQVLQELNVTVVTFFCRPHNICTFVPRR |
| P04963 | AALPHSVRQEPGSGIGYPYDNNTLPYVAPGPTDSRAPCPAL |
| P04963 | TNAFVVCEYVTGSDCGDSLVNLTLLAEPHAFEHDHSFSRKD |
| P04963 | LPDNDENPLVRIDWWKYWFTNESFPYHLGWHPPSPAREIEF |
| P47033 | ALGYTDTGAVDAWYGEISKYNYSNPGFSESTGHFTQVVWKS |
| P47033 | TSTASSRSVTSNSVNSVKFANTTVFSAQTTSSVSASLSSSV |
| P47033 | VTASESITSETAQASSSTEKNISNSAATSSSIYSNSASVSG |
| P47033 | AEYAITSEQSSALATSVPATNCSSIVKTTTLENSSTTTITA |
| P47033 | LATSVPATNCSSIVKTTTLENSSTTTITAITKSTTTLATTA |

| | |
|---|---|
| P47033 | STTTITAITKSTTTLATTANNSTRAATAVTIDPTLDPTDNS |
| P47033 | SSTGASLDSLRTTTSISVSSNTTQLVSTCTSESDYSDSPSF |
| P35495 | CVWTLQVDPKYKLLVSIPTLNLTCGKEYVEILEGAPGSKSL |
| Q12355 | ATATAQSDLDKYSRCDTLVGNLTIGGGLKTGALANVKEING |
| Q12355 | GNLTIGGGLKTGALANVKEINGSLTIFNATNLTSFAADSLE |
| Q12355 | GLKTGALANVKEINGSLTIFNATNLTSFAADSLESITDSLN |
| Q12355 | TGALANVKEINGSLTIFNATNLTSFAADSLESITDSLNLQS |
| Q12355 | TSIKSPVETVSDSLQFSFNGNQTKITFDDLVWANNISLTDV |
| Q12355 | QFSFNGNQTKITFDDLVWANNISLTDVHSVSFANLQKINSS |
| Q12355 | ANNISLTDVHSVSFANLQKINSSLGFINNSISSLNFTKLNT |
| Q12355 | DVHSVSFANLQKINSSLGFINNSISSLNFTKLNTIGQTFSI |
| Q12355 | ANLQKINSSLGFINNSISSLNFTKLNTIGQTFSIVSNDYLK |
| Q12355 | FTKLNTIGQTFSIVSNDYLKNLSFSNLSTIGGALVVANNTG |
| Q12355 | TIGQTFSIVSNDYLKNLSFSNLSTIGGALVVANNTGLQKIG |
| Q12355 | YLKNLSFSNLSTIGGALVVANNTGLQKIGGLDNLTTIGGTL |
| Q12355 | IGGALVVANNTGLQKIGGLDNLTTIGGTLEVVGNFTSLNLD |
| Q12355 | QKIGGLDNLTTIGGTLEVVGNFTSLNLDSLKSVKGGADVES |
| Q12355 | LNLDSLKSVKGGADVESKSSNFSCNALKALQKKGGIKGESF |
| Q3YJS9 | KGIIPATILEFLEGQLQEVDNNTDARLADYFDVIGGTGTGG |
| Q3YJS9 | DICYSTAAAPTYFPPHYFATNTSNGDQYDFNLVDGDVAAVD |
| Q3YJS9 | NYKQMLLLSLGTGTNSEFAKNYTAEEAAKWGILQWMSPLWE |
| Q09614 | RFKQKPGRNSMKTGLDMDAWNETAAEQVLQAWQRNFTKSLY |
| Q09614 | RDWLISIQRGFDEEVAKGSFNLTSGTVIGSNVSEDARLAHA |
| Q09614 | FDEEVAKGSFNLTSGTVIGSNVSEDARLAHALMCSHGSLFG |
| Q6ISU1 | CLVLDVAPPGLDSPIWFSAGNGSALDAFTYGPSPATDGTWT |
| P41222 | QPNFQQDKFLGRWFSAGLASNSSWLREKKAALSMCKSVVAP |
| P41222 | KKAALSMCKSVVAPATDGGLNLTSTFLRKNQCETRTMLLQP |
| P22057 | QPNFQQDKFLGRWYSAGLASNSSWFREKKELLFMCQTVVAP |
| P22057 | KKELLFMCQTVVAPSTEGGLNLTSTFLRKNQCETKVMVLQP |
| Q6AWJ9 | SVLASSSRFQRVPQSQSVVENESVKFECESTDSYSELHYDW |
| Q6AWJ9 | FDSGKDALLREDARLVLHKQNGTLSFASIIASDAGQYQCQL |
| Q6AWJ9 | VHCKAQGTPTPQVQWVRDGENTTLPDHVEVDANGTLIFRNV |
| Q6AWJ9 | VQWVRDGENTTLPDHVEVDANGTLIFRNVNSEHRGNYTCLA |
| Q6AWJ9 | VEVDANGTLIFRNVNSEHRGNYTCLATNSQGQINATVAINV |
| Q6AWJ9 | VNSEHRGNYTCLATNSQGQINATVAINVVVTPKFSVPPVGP |
| Q6AWJ9 | AIGDPKPTIQWDKDLKYLSENNTDRERFRFLENGTLEIRNV |
| Q6AWJ9 | KDLKYLSENNTDRERFRFLENGTLEIRNVQVEDEGSYGCTI |
| Q13308 | SGTFQCVARDDVTGEEARSANASFNIKWIEAGPVVLKHPAS |
| Q13308 | HPRPTYQWFRDGTPLSDGQSNHTVSSKERNLTLRPAGPEHS |
| Q13308 | RDGTPLSDGQSNHTVSSKERNLTLRPAGPEHSGLYSCCAHS |
| Q13308 | HSGLYSCCAHSAFGQACSSQNFTLSIADESFARVVLAPQDV |

| | |
|---|---|
| Q13308 | FSAQPPPSLQWLFEDETPITNRSRPPHLRRATVFANGSLLL |
| Q13308 | ETPITNRSRPPHLRRATVFANGSLLLTQVRPRNAGIYRCIG |
| Q13308 | DAGVYTCHAANLAGQRRQDVNITVATVPSWLKKPQDSQLEE |
| Q13308 | VVWYRNQMLISEDSRFEVFKNGTLRINSVEVYDGTWYRCMS |
| Q13308 | WVTDNAGTLHFARVTRDDAGNYTCIASNGPQGQIRAHVQLT |
| Q13308 | KGKDRILDPTKLGPRMHIFQNGSLVIHDVAPEDSGRYTCIA |
| Q8BKG3 | SFAAVDRLQDSGAFQCVARDNVTGEEVRSTNASFNIKWIEA |
| Q8BKG3 | SGAFQCVARDNVTGEEVRSTNASFNIKWIEAGPVVLKHPAS |
| Q8BKG3 | RDGTPLSDDQSTHTVSSRERNLTLRPASPEHSGLYSCCAHN |
| Q8BKG3 | HSGLYSCCAHNAFGQACSSQNFTLSVADESFARVVLAPQDV |
| Q8BKG3 | FSAQPPPSLQWVFEDETPITNRSRPPHLRRAVVFANGSLLL |
| Q8BKG3 | ETPITNRSRPPHLRRAVVFANGSLLLTQVRPRNAGVYRCIG |
| Q8BKG3 | DTGVYTCHASNLAGQRRQDVNITVATVPTWLRKPQDSQLEE |
| Q8BKG3 | VIWYRNQMLISEDSRFEVSKNGTLRINSVEVYDGTLYRCVS |
| Q8BKG3 | WVTDNAGTLHFARVTRDDAGNYTCIASNEPQGQIRAHVQLT |
| Q8BKG3 | KGKDRILDPTKLGPRMHIFQNGSLVIHDVAPEDSGSYTCIA |
| P16620 | FLCAICVQGSVKQEWAEIGKNVSLECASENEAVAWKLGNQT |
| P16620 | GKNVSLECASENEAVAWKLGNQTINKNHTRYKIRTEPLKSN |
| P16620 | ECASENEAVAWKLGNQTINKNHTRYKIRTEPLKSNDDGSEN |
| P16620 | HTRYKIRTEPLKSNDDGSENNDSQDFIKYKNVLALLDVNIK |
| P16620 | DFIKYKNVLALLDVNIKDSGNYTCTAQTGQNHSTEFQVRPY |
| P16620 | LLDVNIKDSGNYTCTAQTGQNHSTEFQVRPYLPSKVLQSTP |
| P16620 | IKRKIKQDVMLYCLIEMYPQNETTNRNLKWLKDGSQFEFLD |
| P16620 | WLKDGSQFEFLDTFSSISKLNDTHLNFTLEFTEVYKKENGT |
| P16620 | SQFEFLDTFSSISKLNDTHLNFTLEFTEVYKKENGTYKCTV |
| P16620 | KLNDTHLNFTLEFTEVYKKENGTYKCTVFDDTGLEITSKEI |
| P16620 | EVPQVSIDFAKAVGANKIYLNWTVNDGNDPIQKFFITLQEA |
| P16620 | FFITLQEAGTPTFTYHKDFINGSHTSYILDHFKPNTTYFLR |
| P16620 | YHKDFINGSHTSYILDHFKPNTTYFLRIVGKNSIGNGQPTQ |
| P16620 | FRVRACSDLTKTCGPWSENVNGTTMDGVATKPTNLSIQCHH |
| P16620 | GPWSENVNGTTMDGVATKPTNLSIQCHHDNVTRGNSIAINW |
| P16620 | TTMDGVATKPTNLSIQCHHDNVTRGNSIAINWDVPKTPNGK |
| P16620 | IRRIDEPHHKTLYESVSPNTNYTVTVSAITRHKKNGEPATG |
| P16620 | KELPDPEKLNIATYQEVHSDNVTRSSAYIAEMISSKYFRPE |
| P16620 | SNSDSELPILSEKDNLIKGANLTEHALKILESKLRDKRNAV |
| P16620 | NVPLHDSSRDVFDGEIDINSNYTGFLEIIVRDRNNALMAYS |
| P08575 | ASTFERENDFSETTTSLSPDNTSTQVSPDSLDNASAFNTTG |
| P08575 | TTTSLSPDNTSTQVSPDSLDNASAFNTTGVSSVQTPHLPTH |
| P08575 | SPDNTSTQVSPDSLDNASAFNTTGVSSVQTPHLPTHADSQT |
| P08575 | LTTTLSLAHHSSAALPARTSNTTITANTSDAYLNASETTTL |
| P08575 | LAHHSSAALPARTSNTTITANTSDAYLNASETTTLSPSGSA |

| | |
|---|---|
| P08575 | ALPARTSNTTITANTSDAYLNASETTTLSPSGSAVISTTTI |
| P08575 | ISTTTIATTPSKPTCDEKYANITVDYLYNKETKLFTAKLNV |
| P08575 | TPSKPTCDEKYANITVDYLYNKETKLFTAKLNVNENVECGN |
| P08575 | NKETKLFTAKLNVNENVECGNNTCTNNEVHNLTECKNASVS |
| P08575 | LNVNENVECGNNTCTNNEVHNLTECKNASVSISHNSCTAPD |
| P08575 | VECGNNTCTNNEVHNLTECKNASVSISHNSCTAPDKTLILD |
| P08575 | TNNEVHNLTECKNASVSISHNSCTAPDKTLILDVPPGVEKF |
| P08575 | ADTTICLKWKNIETFTCDTQNITYRFQCGNMIFDNKEIKLE |
| P08575 | EPEHEYKCDSEILYNNHKFTNASKIIKTDFGSPGEPQIIFC |
| P08575 | RSEAAHQGVITWNPPQRSFHNFTLCYIKETEKDCLNLDKNL |
| P08575 | KPYTKYVLSLHAYIIAKVQRNGSAAMCHFTTKSAPPSQVWN |
| P08575 | NGSAAMCHFTTKSAPPSQVWNMTVSMTSDNSMHVKCRPPRD |
| P08575 | TTKSAPPSQVWNMTVSMTSDNSMHVKCRPPRDRNGPHERYH |
| P08575 | RNGPHERYHLEVEAGNTLVRNESHKNCDFRVKDLQYSTDYT |
| P10586 | RVQRDEAIYECTATNSLGEINTSAKLSVLEEEQLPPGFPSI |
| P10586 | VAPRFSIPPSSQEVMPGGSVNLTCVAVGAPMPYVKWMMGAE |
| P10586 | EDEMPVGRNVLELSNVVRSANYTCVAISSLGMIEATAQVTV |
| P10586 | VRTDEDVPSGPPRKVEVEPLNSTAVHVYWKLPVPSKQHGQI |
| P10586 | PVLAERNGRIISYTVVFRDINSQQELQNITTDTRFTLTGLK |
| P10586 | GRIISYTVVFRDINSQQELQNITTDTRFTLTGLKPDTTYDI |
| A2A8L5 | RVQRDEAIYECTATNSLGEINTSAKLSVLEEDQLPSGFPTI |
| A2A8L5 | VAPRFSIPPSSQEVMPGGSVNLTCVAVGAPMPYVKWMMGAE |
| A2A8L5 | EDEMPVGRNVLELSNVMRSANYTCVAISSLGMIEATAQVTV |
| A2A8L5 | VRTDEDVPSGPPRKVEVEPLNSTAVHVSWKLPVPNKQHGQI |
| A2A8L5 | TGLTTSTTELTWDPPVLAERNGHITNYTVVYRDINSQLELQ |
| A2A8L5 | STTELTWDPPVLAERNGHITNYTVVYRDINSQLELQNVTND |
| A2A8L5 | GHITNYTVVYRDINSQLELQNVTNDTHLTLLGLKPDTTYDI |
| A2A8L5 | TNYTVVYRDINSQLELQNVTNDTHLTLLGLKPDTTYDIKVR |
| P23470 | LDQYARVGEEYQELQLDGFDNESSNKTWMKNTGKTVAILLK |
| P23470 | ARVGEEYQELQLDGFDNESSNKTWMKNTGKTVAILLKDDYF |
| P23470 | GAGLPGRFKAEKVEFHWGHSNGSAGSEHSINGRRFPVEMQI |
| P23470 | GTEASKVCSSPPIHMKVQPLNQTALQVSWSQPETIYHPPIM |
| P23470 | QAVCRNDMRSDFSQTMLFQANTTRIFQGTRIVKTGVPTASP |
| P23470 | GEKDSEKKEKSGVTHAAEERNQTEPSPTPSSPNRTAEGGHQ |
| P23470 | VTHAAEERNQTEPSPTPSSPNRTAEGGHQTIPGHEQDHTAV |
| P23470 | SRGDRFSEDSRFITVNPAEKNTSGMISRPAPGRMEWIIPLI |
| Q12913 | ATGENGITQISSTAESFHKQNGTGTPQVETNTSEDGESSGA |
| Q12913 | SSTAESFHKQNGTGTPQVETNTSEDGESSGANDSLRTPEQG |
| Q12913 | GTGTPQVETNTSEDGESSGANDSLRTPEQGSNGTDGASQKT |
| Q12913 | SEDGESSGANDSLRTPEQGSNGTDGASQKTPSSTGPSPVFD |
| Q12913 | VFDIKAVSISPTNVILTWKSNDTAASEYKYVVKHKMENEKT |

| | |
|---|---|
| Q12913 | VVKHKMENEKTITVVHQPWCNITGLRPATSYVFSITPGIGN |
| Q12913 | NITGLRPATSYVFSITPGIGNETWGDPRVIKVITEPIPVSD |
| Q12913 | SDLRVALTGVRKAALSWSNGNGTASCRVLLESIGSHEELTQ |
| Q12913 | VLLESIGSHEELTQDSRLQVNISGLKPGVQYNINPYLLQSN |
| Q12913 | NISGLKPGVQYNINPYLLQSNKTKGDPLGTEGGLDASNTER |
| Q12913 | GQQSRDTEVLLVGLEPGTRYNATVYSQAANGTEGQPQAIEF |
| Q12913 | LLVGLEPGTRYNATVYSQAANGTEGQPQAIEFRTNAIQVFD |
| Q12913 | QPQAIEFRTNAIQVFDVTAVNISATSLTLIWKVSDNESSSN |
| Q12913 | DVTAVNISATSLTLIWKVSDNESSSNYTYKIHVAGETDSSN |
| Q12913 | NISATSLTLIWKVSDNESSSNYTYKIHVAGETDSSNLNVSE |
| Q12913 | SSSNYTYKIHVAGETDSSNLNVSEPRAVIPGLRSSTFYNIT |
| Q12913 | NLNVSEPRAVIPGLRSSTFYNITVCPVLGDIEGTPGFLQVH |
| Q12913 | SFQMHITQEGAGNSRVEITTNQSIIIGGLFPGTKYCFEIVP |
| Q12913 | IIGGLFPGTKYCFEIVPKGPNGTEGASRTVCNRTVPSAVFD |
| Q12913 | CFEIVPKGPNGTEGASRTVCNRTVPSAVFDIHVVYVTTTEM |
| Q12913 | SPDGASEYVYHLVIESKHGSNHTSTYDKAITLQGLIPGTLY |
| Q12913 | HTSTYDKAITLQGLIPGTLYNITISPEVDHVWGDPNSTAQY |
| Q12913 | PGTLYNITISPEVDHVWGDPNSTAQYTRPSNVSNIDVSTNT |
| Q12913 | PEVDHVWGDPNSTAQYTRPSNVSNIDVSTNTTAATLSWQNF |
| Q12913 | PNSTAQYTRPSNVSNIDVSTNTTAATLSWQNFDDASPTYSY |
| Q12913 | QNFDDASPTYSYCLLIEKAGNSSNATQVVTDIGITDATVTE |
| Q12913 | DDASPTYSYCLLIEKAGNSSNATQVVTDIGITDATVTELIP |
| Q12913 | TCPPGANAGFELEVSSGAWNNATHLESCSSENGTEYRTEVT |
| Q12913 | LEVSSGAWNNATHLESCSSENGTEYRTEVTYLNFSTSYNIS |
| Q12913 | HLESCSSENGTEYRTEVTYLNFSTSYNISITTVSCGKMAAP |
| Q12913 | SENGTEYRTEVTYLNFSTSYNISITTVSCGKMAAPTRNTCT |
| Q12913 | PTRNTCTTGITDPPPPDGSPNITSVSHNSVKVKFSGFEASH |
| Q12913 | EEKGRSQSLSEVLKYEIDVGNESTTLGYYNGKLEPLGSYRA |
| Q12913 | YYNGKLEPLGSYRACVAGFTNITFHPQNKGLIDGAESYVSF |
| Q64455 | PIFDIEAVVSPTSVLLTWKHNDSGASECRIENKMESNLTFP |
| Q64455 | TWKHNDSGASECRIENKMESNLTFPVKNQTSCNITGLSPGT |
| Q64455 | GASECRIENKMESNLTFPVKNQTSCNITGLSPGTSYTFSII |
| Q64455 | RIENKMESNLTFPVKNQTSCNITGLSPGTSYTFSIISVTTN |
| Q64455 | NITGLSPGTSYTFSIISVTTNETLNKTITTEPWPVSDLHVT |
| Q64455 | LSPGTSYTFSIISVTTNETLNKTITTEPWPVSDLHVTSVGV |
| Q64455 | SDLHVTSVGVTQARLTWSNANGTASYRMLIEELTTHSSVNI |
| Q64455 | ANGTASYRMLIEELTTHSSVNISGLKPGTNNSFAFPESNET |
| Q64455 | LIEELTTHSSVNISGLKPGTNNSFAFPESNETQADFAVAEE |
| Q64455 | SVNISGLKPGTNNSFAFPESNETQADFAVAEEVPDANGTKR |
| Q64455 | FPESNETQADFAVAEEVPDANGTKRIPVTNLSQLHKNSLVS |
| Q64455 | DFAVAEEVPDANGTKRIPVTNLSQLHKNSLVSVDPPSGQDP |

| | |
|---|---|
| Q64455 | GQDPSLTEILLTDLKPDTQYNATIYSQAANGTEGQPRNKVF |
| Q64455 | LLTDLKPDTQYNATIYSQAANGTEGQPRNKVFKTNSTQVSD |
| Q64455 | IYSQAANGTEGQPRNKVFKTNSTQVSDVRAMNISASSMTLT |
| Q64455 | QPRNKVFKTNSTQVSDVRAMNISASSMTLTWKSNYDGSRTS |
| Q64455 | DGSRTSIVYKIHVAGGTHSVNQTVNKTEAIILGLSSSTLYN |
| Q64455 | TSIVYKIHVAGGTHSVNQTVNKTEAIILGLSSSTLYNITVH |
| Q64455 | NQTVNKTEAIILGLSSSTLYNITVHPFLGQTEGTPGFLQVY |
| Q64455 | TPGFLQVYTSPDQVSDFRVTNVSTRAIGLAWRSNDSKSFEI |
| Q64455 | VSDFRVTNVSTRAIGLAWRSNDSKSFEIFIKQDGGEKHRNA |
| Q64455 | SNDSKSFEIFIKQDGGEKHRNASTGNQSYMVEDLKPGTSYH |
| Q64455 | SFEIFIKQDGGEKHRNASTGNQSYMVEDLKPGTSYHFEIIP |
| Q64455 | YHFEIIPRGPDGTEGLSSTVNGSTDPSAVTDIRVVNISTTE |
| Q64455 | LSSTVNGSTDPSAVTDIRVVNISTTEMQLEWQNTDDASGYT |
| Q64455 | ASGYTYHLVLESKSGSIIRTNSSQKWITVGSLTPGTLYNVT |
| Q64455 | RTNSSQKWITVGSLTPGTLYNVTIFPEVDQIQGISNSITQY |
| Q64455 | GISNSITQYTRPSSVSHIEVNTTTTTAAIRWKNEDAASASY |
| Q64455 | DAASASYAYSVLILKTGDGSNVTSNFTKDPSILIPELIPGV |
| Q64455 | ASYAYSVLILKTGDGSNVTSNFTKDPSILIPELIPGVSYTV |
| Q64455 | ACPFGMYTGFELGVRSDSWDNMTRLENCTSDDDTECRTEVA |
| Q64455 | YTGFELGVRSDSWDNMTRLENCTSDDDTECRTEVAYLNFST |
| Q64455 | RLENCTSDDDTECRTEVAYLNFSTSYNISIATLSCGKMALP |
| Q64455 | SDDDTECRTEVAYLNFSTSYNISIATLSCGKMALPAQNICT |
| Q64455 | PAQNICTTGITDPPTPDGSPNITSVSHNSVKVKFSGFEASH |
| Q64455 | EEKGQSQGLSEVLNYEIDVGNQSTTLGYYNGRLEPLGSYRA |
| Q64455 | YYNGRLEPLGSYRACVAGFTNITYNLQNDGLINGDESYVSF |
| Q15262 | DSSDHDPGEKARLQLPTMKENDTHCIDFSYLLYSQKGLNPG |
| Q15262 | PGTLNILVRVNKGPLANPIWNVTGFTGRDWLRAELAVSTFW |
| Q15262 | PCDKSPHFLRLGDVEVNAGQNATFQCIATGRDAVHNKLWLQ |
| Q15262 | LKIAEIQARRIAVDWESLGYNITRCHTFNVTICYHYFRGHN |
| Q15262 | RRIAVDWESLGYNITRCHTFNVTICYHYFRGHNESKADCLD |
| Q15262 | NITRCHTFNVTICYHYFRGHNESKADCLDMDPKAPQHVVNH |
| Q15262 | CLDMDPKAPQHVVNHLPPYTNVSLKMILTNPEGRKESEETI |
| Q15262 | RSFDPAVPVAGPPQTVSNLWNSTHHVFMHLHPGTTYQFFIR |
| Q15262 | TYQFFIRASTVKGFGPATAINVTTNISAPTLPDYEGVDASL |
| Q15262 | FIRASTVKGFGPATAINVTTNISAPTLPDYEGVDASLNETA |
| Q15262 | VTTNISAPTLPDYEGVDASLNETATTITVLLRPAQAKGAPI |
| Q15262 | FAAELPPGNLPEPAPFTVGDNRTYQGFWNPPLAPRKGYNIY |
| P35822 | DSSNHDPGEKARLQLPTMKENDTHCIDFSYLLYSQKGLNPG |
| P35822 | PGTLNILVRVNKGPLANPIWNVTGFTGRDWLRAELAVSTFW |
| P35822 | PCDKSPHFLRLGDVEVNAGQNATFQCIATGRDAVHNKLWLQ |
| P35822 | LKIAEIQARRIAVDWESLGYNITRCHTFNVTICYHYFRGHN |

| | |
|---|---|
| P35822 | RRIAVDWESLGYNITRCHTFNVTICYHYFRGHNESRADCLD |
| P35822 | NITRCHTFNVTICYHYFRGHNESRADCLDMDPKAPQHVVNH |
| P35822 | CLDMDPKAPQHVVNHLPPYTNVSLKMILTNPEGRKESEETI |
| P35822 | RSFDPAVPVAGPPQTVSNLWNSTHHVFMHLHPGTTYQFFIR |
| P35822 | TYQFFIRASTVKGFGPATAINVTTNISAPSLPDYEGVDASL |
| P35822 | FIRASTVKGFGPATAINVTTNISAPSLPDYEGVDASLNETA |
| P35822 | VTTNISAPSLPDYEGVDASLNETATTITVLLRPAQAKGAPI |
| P35822 | FAAELPPGNLPEPAPFTVGDNRTYKGFWNPPLAPRKGYNIY |
| P28827 | NTLTKPTSDPWMPSGSFMLVNASGRPEGQRAHLLLPQLKEN |
| P28827 | NASGRPEGQRAHLLLPQLKENDTHCIDFHYFVSSKSNSPPG |
| P28827 | PGLLNVYVKVNNGPLGNPIWNISGDPTRTWNRAELAISTFW |
| P28827 | PLKEIKVTSSRRFIASFNVVNTTKRDAGKYRCMIRTEGGVG |
| P28827 | LEVVEVKSRQITIRWEPFGYNVTRCHSYNLTVHYCYQVGGQ |
| P28827 | RQITIRWEPFGYNVTRCHSYNLTVHYCYQVGGQEQVREEVS |
| P28827 | SWDTENSHPQHTITNLSPYTNVSVKLILMNPEGRKESQELI |
| P28827 | TLYEITYKAVSSFDPEIDLSNQSGRVSKLGNETHFLFFGLY |
| P28827 | SSFDPEIDLSNQSGRVSKLGNETHFLFFGLYPGTTYSFTIR |
| P28827 | QFTTKISAPSMPAYELETPLNQTDNTVTVMLKPAHSRGAPV |
| P28827 | RRTKKTTEILKCYPVPIHFQNASLLNSQYYFAAEFPADSLQ |
| P28827 | FAAEFPADSLQAAQPFTIGDNKTYNGYWNTPLLPYKSYRIY |
| Q16849 | GVKLLEILAEHVHMSSGSFINISVVGPALTFRIRHNEQNLS |
| Q16849 | FINISVVGPALTFRIRHNEQNLSLADVTQQAGLVKSELEAQ |
| Q13332 | VAPRFSILPMSHEIMPGGNVNITCVAVGSPMPYVKWMQGAE |
| Q13332 | EDDMPVGRNVLELTDVKDSANYTCVAMSSLGVIEAVAQITV |
| Q13332 | VRTDEDVPSAPPRKVEAEALNATAIRVLWRSPAPGRQHGQI |
| Q13332 | VLSIPEDTPRGHPQILEAAGNASAGTVLLRWLPPVPAERNG |
| P23471 | WDKTSLENTFIHNTGKTVEINLTNDYRVSGGVSEMVFKASK |
| P23471 | GGVSEMVFKASKITFHWGKCNMSSDGSEHSLEGQKFPLEMQ |
| P23471 | VSRFGKQAALDPFILLNLLPNSTDKYYIYNGSLTSPPCTDT |
| P23471 | LDPFILLNLLPNSTDKYYIYNGSLTSPPCTDTVDWIVFKDT |
| P23471 | EEIHEAVCSSEPENVQADPENYTSLLVTWERPRVVYDTMIE |
| P23471 | HEFLTDGYQDLGAILNNLLPNMSYVLQIVAICTNGLYGKYS |
| P23471 | AKTNRSPTRGSEFSGKGDVPNTSLNSTSQPVTKLATEKDIS |
| P23471 | RSPTRGSEFSGKGDVPNTSLNSTSQPVTKLATEKDISLTSQ |
| P23471 | EGTSASLNDGSKTVLRSPHMNLSGTAESLNTVSITEYEEES |
| P23471 | GAEDSSGSSPATSAIPFISENISQGYIFSSENPETITYDVL |
| P23471 | FSSENPETITYDVLIPESARNASEDSTSSGSEESLKDPSME |
| P23471 | STDITAQPDVGSGRESFLQTNYTEIRVDESEKTTKSFSAGP |
| P23471 | SGASSDSEFLLPDTDGLTALNISSPVSVAEFTYTTSVFGDD |
| P23471 | TTSVFGDDNKALSKSEIIYGNETELQIPSFNEMVYPSESTV |
| P23471 | MVYPSESTVMPNMYDNVNKLNASLQETSVSISSTKGMFPGS |

| | |
|---|---|
| P23471 | SLAHTTTKVFDHEISQVPENNFSVQPTHTVSQASGDTSLKP |
| P23471 | LSIHKCMSCSSYRESQEKVMNDSDTHENSLMDQNNPISYSL |
| P23471 | VLTSDEESGSGQGTSDSLNENETSTDFSFADTNEKDADGIL |
| P23471 | TSSVTSENSEVFHVSEAEASNSSHESRIGLAEGLESEKKAV |
| Q9BVG9 | LGVPLPERDYGGNCLIYDPDNETDPFHNIWDKLDGFVPAHF |
| P17219 | KEIEDLGENSVPRFIETRNCNKTQQPTCRPPYICKESLYSI |
| Q15223 | LALGLTAFFLPGVHSQVVQVNDSMYGFIGTDVVLHCSFANP |
| Q15223 | SFANPLPSVKITQVTWQKSTNGSKQNVAIYNPSMGVSVLAP |
| Q15223 | EGVYICEFATFPTGNRESQLNLTVMAKPTNWIEGTQAVLRA |
| Q15223 | VVSWETRLKGEAEYQEIRNPNGTVTVISRYRLVPSREAHQQ |
| Q15223 | KLTCKADANPPATEYHWTTLNGSLPKGVEAQNRTLFFKGPI |
| Q15223 | ATEYHWTTLNGSLPKGVEAQNRTLFFKGPINYSLAGTYICE |
| Q15223 | GSLPKGVEAQNRTLFFKGPINYSLAGTYICEATNPIGTRSG |
| Q15223 | GTYICEATNPIGTRSGQVEVNITEFPYTPSPPEHGRRAGPV |
| Q9JKF6 | LALGLTAFFLPGTHTQVVQVNDSMYGFIGTDVVLHCSFANP |
| Q9JKF6 | SFANPLPSVKITQVTWQKASNGSKQNMAIYNPTMGVSVLPP |
| Q9JKF6 | EGMYICEFATFPTGNRESQLNLTVMAKPTNWIEGTRAVLRA |
| Q9JKF6 | PSAVSWETRLKGEAEYQEIRNPNGTVTVISRYRLVPSREAH |
| Q9JKF6 | AVSWETRLKGEAEYQEIRNPNGTVTVISRYRLVPSREAHRQ |
| Q9JKF6 | FDGNWYLQRTDVKLTCKADANPPATEYHWTTLNGSLPKGVE |
| Q9JKF6 | KLTCKADANPPATEYHWTTLNGSLPKGVEAQNRTLFFRGPI |
| Q9JKF6 | ATEYHWTTLNGSLPKGVEAQNRTLFFRGPITYSLAGTYICE |
| Q9JKF6 | GTYICEATNPIGTRSGQVEVNITEFPYTPTPEHGRRAGQMP |
| P32507 | NADLRDATLAFRGLRVEDEGNYTCEFATFPNGTRRGVTWLR |
| P32507 | FRGLRVEDEGNYTCEFATFPNGTRRGVTWLRVIAQPENHAE |
| P32507 | PASAVAQGSQLLVHSVDRMVNTTFICTATNAVGTGRAEQVI |
| Q9NQS3 | CGALAGPIIVEPHVTAVWGKNVSLKCLIEVNETITQISWEK |
| Q9NQS3 | EPHVTAVWGKNVSLKCLIEVNETITQISWEKIHGKSSQTVA |
| Q9NQS3 | HHPQYGFSVQGEYQGRVLFKNYSLNDATITLHNIGFSDSGK |
| Q9NQS3 | VLVEPTVSLIKGPDSLIDGGNETVAAICIAATGKPVAHIDW |
| Q9NQS3 | AHIDWEGDLGEMESTTTSFPNETATIISQYKLFPTRFARGR |
| Q9NQS3 | WPDGLLASDNTLHFVHPLTFNYSGVYICKVTNSLGQRSDQK |
| P15151 | SYSESKRLEFVAARLGAELRNASLRMFGLRVEDEGNYTCLF |
| P15151 | GAELRNASLRMFGLRVEDEGNYTCLFVTFPQGSRSVDIWLR |
| P15151 | STGGRPPAQITWHSDLGGMPNTSQVPGFLSGTVTVTSLWIL |
| P15151 | GTVTVTSLWILVPSSQVDGKNVTCKVEHESFEKPQLLTVNL |
| P15151 | KNVTCKVEHESFEKPQLLTVNLTVYYPPEVSISGYDNNWYL |
| P15151 | GQNEATLTCDARSNPEPTGYNWSTTMGPLPPFAVAQGAQLL |
| P15151 | PPFAVAQGAQLLIRPVDKPINTTLICNVTNALGARQAELTV |
| P15151 | QGAQLLIRPVDKPINTTLICNVTNALGARQAELTVQVKEGP |
| Q6UX71 | GQDSPEPRSFTDLLLDDGQDNNTQIEEDTDHNYYISRIYGP |

| | |
|---|---|
| Q6UX71 | KDKVKIHGILSNTHRQAARVNLSFDFPFYGHFLREITVATG |
| Q1ENI8 | CDKKGLVVDCSSSGLTRIPKNISRNVRSLVIRNNRIHKLKR |
| Q1ENI8 | CARPAEESWTGEEIKLTCAKNSSSKLVVWMYENVEVDSSSL |
| Q1ENI8 | LVREHVEKGLILNVDELHPKNVSYESVLHVTHVQALMGLSG |
| Q1ENI8 | DLTHTVTALSRHSYATGAFCNRTCENLDPCFNIPLSPNDPR |
| Q1ENI8 | SEAGKEYLPFEKDSNMDCRRNFSEENPIRCFLAGDLRANEQ |
| Q92626 | GDPFVATSIVEAIATVDRAINSTRTHLFDSRPRSPNDLLAL |
| Q92626 | FERTLQLIQEHVQHGLMVDLNGTSYHYNDLVSPQYLNLIAN |
| Q92626 | NGTSYHYNDLVSPQYLNLIANLSGCTAHRRVNNCSDMCFHQ |
| Q92626 | PQYLNLIANLSGCTAHRRVNNCSDMCFHQKYRTHDGTCNNL |
| Q92626 | QHCSNVCSNDPPCFSVMIPPNDSRARSGARCMFFVRSSPVC |
| Q92626 | NIQRGRDHGIPPYHDYRVYCNLSAAHTFEDLKNEIKNPEIR |
| Q92626 | AQLTQIKQTSLARILCDNADNITRVQSDVFRVAEFPHGYGS |
| Q92626 | TKKTRPRKIPSVGRQGEHLSNSTSAFSTRSDASGTNDFREF |
| Q92626 | KLESRLSTTECVDAGGESHANNTKWKKDACTICECKDGQVT |
| Q9VZZ4 | QWFVNGRQLLQSTPSLQLQANGSLILLQPNQLSAGTYRCEA |
| Q9VZZ4 | PNVQLAEKYQISGAGSLFVKNVTIPDGGRYECQLKNQFGRA |
| Q9VZZ4 | GDRYVRIAFAEAAKEIDLAINNTLDMLFSNRSDKAPPNYGE |
| Q9VZZ4 | AEAAKEIDLAINNTLDMLFSNRSDKAPPNYGELLRVFRFPT |
| Q9VZZ4 | AAEIYERTLVNIRKHVQEGDNLTMKSEEYEFRDLLSREHLH |
| Q9VZZ4 | REHLHLVAELSGCMEHREMPNCTDMCFHSRYRSIDGTCNNL |
| Q9VZZ4 | SYIDASQVYGYSTAFAQELRNLTSQEGLLRVGVHFPRQKDM |
| Q9VZZ4 | FATAALRFGHTIINPILHRLNETFQPIPQGHLLLHKAFFAP |
| Q9VZZ4 | NIQRGRDHGMPGYNVYRKLCNLTVAQDFEDLAGEISSAEIR |
| P20742 | DRLLHLCLVLLLILLSASDSNSTEPQYMVLVPSLLHTEAPK |
| P20742 | VPSLLHTEAPKKGCVLLSHLNETVTVSASLESGRENRSLFT |
| P20742 | LLSHLNETVTVSASLESGRENRSLFTDLVAEKDLFHCVSFT |
| P20742 | PKFEVKVQVPKIISIMDEKVNITVCGEYTYGKPVPGLATVS |
| P20742 | GVPIPNKLFFISVNDANYYSNATTNEQGLAQFSINTTSISV |
| P20742 | DANYYSNATTNEQGLAQFSINTTSISVNKLFVRVFTVHPNL |
| P20742 | VPETVRSYFPETWIWELVAVNSSGVAEVGVTVPDTITEWKA |
| P20742 | CGNERQTLSWTVTPKTLGNVNFSVSAEAMQSLELCGNEVVE |
| P20742 | EAEGIEQEKTFSSMTCASGANVSEQLSLKLPSNVVKESARA |
| P20742 | GCGEQNMVLFAPNIYVLNYLNETQQLTQEIKAKAVGYLITG |
| P20742 | SVSRTEVSNNHVLIYVEQVTNQTLSFSFMVLQDIPVGDLKP |
| Q7SIC2 | KHYENFYCNKGSFQLWAQSGNETQQTRVLSSGDYGSVPRNV |
| Q7SIC2 | GNETQQTRVLSSGDYGSVPRNVTHTFQIQDPDTEMTGVIVP |
| Q7SIC2 | EMTGVIVPGGFEDLFYYLGTNATDTTHTPYIPSSSDSSSTT |
| Q7SIC2 | TLQSFDVYAELSFTPRTDTVNGTAPANTVWHTGANALASTA |
| Q7SIC2 | SQYGYQIVAPFVTATQAQDTNYTLSTISMSTTPSTVTVPTW |
| Q9CYK2 | VVGTLHLLLLQATVLSLTAGNLSLVSAAWTQEKNHHQPAHL |

| | |
|---|---|
| Q9CYK2 | LSLVSAAWTQEKNHHQPAHLNSSSLQQVAEGTSISEMWQND |
| Q16769 | VSPSASAWPEEKNYHQPAILNSSALRQIAEGTSISEMWQND |
| Q16769 | HELHELGLLKDHSLEGRYFQNYSYGGVIQDDHIPFLRRGVP |
| O00391 | CRDFNIPGFPTVRFFKAFTKNGSGAVFPVAGADVQTLRERL |
| O00391 | ANVVRKFGVTDFPSCYLLFRNGSVSRVPVLMESRSFYTAYL |
| O00391 | VQNVAAAPELAMGALELESRNSTLDPGKPEMMKSPTNTTPH |
| P02752 | SPEPNMHECTLYSESSCCYANFTEQLAHSPIIKVSNSYWNR |
| P02752 | DESGENHCKSKCVPYSEMYANGTDMCQSMWGESFKVSESSC |
| Q05186 | VRKERVVRPDSELGERPPEDNQSFQYDHEAFLGKEDSKTFD |
| Q15293 | VRKERVVRPDSELGERPPEDNQSFQYDHEAFLGKEDSKTFD |
| Q96D15 | AAWDTYDTDRDGRVGWEELRNATYGHYAPGEEFHDVEDAET |
| P78509 | FGNQFMCSVVASHVSHLPTTNLSFIWIAPPAGTGCVNFMAT |
| P78509 | GNAVTFCEPYGPRELITTGLNTTTASVLQFSIGSGSCRFSY |
| P78509 | GSGSCRFSYSDPSIIVLYAKNNSADWIQLEKIRAPSNVSTI |
| P78509 | LYAKNNSADWIQLEKIRAPSNVSTIIHILYLPEDAKGENVQ |
| P78509 | CLPEICAGPHLPHSTVYSSENYSGWNRITIPLPNAALTRNT |
| P78509 | DYPMNQMSVWLMLANEGMVKNETFCAATPSAMIFGKSDGDR |
| P78509 | QPQHGKHSAQWALDDVLIGMNDSSQTGFQDKFDGSIDLQAN |
| P78509 | TVYLPLSTISPRTRFRWIQANYTVGADSWAIDNVVLASGCP |
| P78509 | FPQTTNILFINVPLPYTAQTNATRFRLWQPYNNGKKEEIWI |
| P78509 | DNVYIGPQCEEMCNGQGSCINGTKCICDPGYSGPTCKISTK |
| P78509 | LQYSLNGGLSWSLLQEFLFSNSSNVGRYIALEIPLKARSGS |
| P78509 | PSENGHFYSPWVIDQILIGGNISGNTVLEDDFTTLDSRKWL |
| P78509 | SGMALHFSGGCSRLLVTVDLNLTNAEFIQFYFMYGCLITPN |
| P78509 | DLDLRGAKFLQYWGRIGSENNMTSCHRPICRKEGVLLDYST |
| P78509 | YQKYISVRHDYILLPEDALTNTTRLRWWQPFVISNGIVVSG |
| P78509 | EINPSQLVDTFDDEGTSHEENWSFYPNAVRTAGFCGNPSFH |
| P78509 | SNSIGCSPFQFHEATIYNSVNSSSWKRITIQLPDHVSSSAT |
| P78509 | NVPLEARMKGVLLRWWQPRHNGTGHDQWALDHVEVVLVSTR |
| P78509 | WALDHVEVVLVSTRKQNYMMNFSRQHGLRHFYNRRRRSLRR |
| Q60841 | FGNQFMCSVVASHVSHLPTTNLSFVWIAPPAGTGCVNFMAT |
| Q60841 | GNAVTFCEPYGPRELTTTCLNTTTASVLQFSIGSGSCRFSY |
| Q60841 | GSGSCRFSYSDPSITVSYAKNNTADWIQLEKIRAPSNVSTV |
| Q60841 | SYAKNNTADWIQLEKIRAPSNVSTVIHILYLPEEAKGESVQ |
| Q60841 | CLPEICAGPHLPHSTVYSSENYSGWNRITIPLPNAALTRDT |
| Q60841 | DYPMNQMSVWLMLANEGMAKNDSFCATTPSAMVFGKSDGDR |
| Q60841 | VCFCDLGYTAAQGTCVSNTPNHSEMFDRFEGKLSPLWYKIT |
| Q60841 | QPQHGKHSAQWALDDVLIGVNDSSQTGFQDKFDGSIDLQAN |
| Q60841 | TVYLPLATNSPRTRFRWIQTNYTVGADSWAIDNVILASGCP |
| Q60841 | FPQTTSILFINVPLPYGAQTNATRFRLWQPYNNGKKEEIWI |
| Q60841 | DNVYIGPQCEEMCYGHGSCINGTKCICDPGYSGPTCKISTK |

| | |
|---|---|
| Q60841 | LQYSLNGGLSWSLLQEFLFSNSSNVGRYIALEMPLKARSGS |
| Q60841 | PSENGHFYSPWVIDQILIGGNISGNTVLEDDFSTLDSRKWL |
| Q60841 | SGLALHFSGGCSRLLVTVDLNLTNAEFIQFYFMYGCLITPS |
| Q60841 | DLDLRGAKFLQYWGRIGSENNMTSCHRPVCRKEGVLLDFST |
| Q60841 | FQKYISVRHDYILLPEGALTNTTRLRWWQPFVISNGLVVSG |
| Q60841 | EINPSQLVDTFDDEGSSHEENWSFYPNAVRTAGFCGNPSFH |
| Q60841 | SNSIGCSPFQFHEATIYNAVNSSSWKRITIQLPDHVSSSAT |
| Q60841 | NVPLEARMKGVLLRWWQPRHNGTGHDQWALDHVEVVLVSTR |
| Q60841 | WALDHVEVVLVSTRKQNYMMNFSRQHGLRHFYNRRRRSLRR |
| P81191 | QYVRAAISICGPNMQTMRPRNGSGPIVPPPDFLAMYGMARY |
| P00797 | VDMARLGPEWSQPMKRLTLGNTTSSVILTNYMDTQYYGEIG |
| P00797 | YTACVYHKLFDASDSSSYKHNGTELTLRYSTGTVSGFLSQD |
| Q6H3X3 | LHYDCGSKTVTPVSPLGKKLNVTTAWKAQNPVLREVVDILT |
| P30986 | LNCALGGNDLLSCLTFNGVRNHTVFSADSDSDFNRFLHLSI |
| P30986 | FTPFPHRSGTRLMVEYIVAWNQSEQKKKTEFLDWLEKVYEF |
| P30986 | NPRLGYVNHIDLDLGGIDWGNKTVVNNAIEISRSWGESYFL |
| P07949 | TRLHENNWICIQEDTGLLYLNRSLDHSSWEKLSVRNRGFPL |
| P07949 | SLREGECQWPGCARVYFSFFNTSFPACSSLKPRELCFPETR |
| P07949 | NRPPGTFHQFRLLPVQFLCPNISVAYRLLEGEGLPFRCAPD |
| P07949 | STLLPGDTWAQQTFRVEHWPNETSVQANGSFVRATVHDYRL |
| P07949 | TWAQQTFRVEHWPNETSVQANGSFVRATVHDYRLVLNRNLS |
| P07949 | QANGSFVRATVHDYRLVLNRNLSISENRTMQLAVLVNDSDF |
| P07949 | VRATVHDYRLVLNRNLSISENRTMQLAVLVNDSDFQGPGAG |
| P07949 | VLNRNLSISENRTMQLAVLVNDSDFQGPGAGVLLLHFNVSV |
| P07949 | VLVNDSDFQGPGAGVLLLHFNVSVLPVSLHLPSTYSLSVSR |
| P07949 | ENCQAFSGINVQYKLHSSGANCSTLGVVTSAEDTSGILFVN |
| P07949 | NCSTLGVVTSAEDTSGILFVNDTKALRRPKCAELHYMVVAT |
| P07949 | LGSPTGRCEWRQGDGKGITRNFSTCSPSTKTCPDGHCDVVE |
| Q00017 | GRTDCSGTGAEVCYSVYDGVNETILTFPAYLENAAKLFTAK |
| Q00017 | AGVEYVDHWSYVDSIYETLGNATVNSYFPIDHTHTSPAGAE |
| Q9UBD6 | VFVRYDFEADAHWWSERTHKNLSDMENEFYYRYPSFQDVHV |
| Q96CC6 | PHEWPEDITKWPICTKNSAGNHTNHPHMDCVITGRPCCIGT |
| Q00001 | ASTKGATKTCNILSYGAVADNSTDVGPAITSAWAACKSGGL |
| Q00001 | DIDGYWSSMTAVAGDGVQLNNITVKNWKGTEANGATRPPIR |
| P02879 | SGWSFTLEDNNIFPKQYPIINFTTAGATVQSYTNFIRAVRG |
| P02879 | TAIQESNQGAFASPIQLQRRNGSKFSVYDVSILIPIIALMV |
| P02879 | VMIYDCNTAATDATRWQIWDNGTIINPRSSLVLAATSGNSG |
| P02879 | GTTLTVQTNIYAVSQGWLPTNNTQPFVTTIVGLYGLCLQAN |
| P16094 | GIFRTPIVLVDNKGNRVQITNVTSKVVTSNIQLLLNTRNIA |
| P34967 | TERKDVETTLCPNASSRVSKNISYDSSYPALENKAGRSRSQ |
| P24817 | VYDIPLLYSTISDSRRFILLNLTSYAYETISVAIDVTNVYV |

| | |
|---|---|
| P33186 | RFTFIENQIRNNFQQRIRPANNTISLENKWGKLSFQIRTSG |
| P84853 | SLRNEAKDPSLQCYGIPMLPNNSSTIKYLLVKLQGASQKTI |
| P84531 | IANNVGTKFKPSQTIISLENNWSALSKQIQIAKNKNGQFET |
| P84530 | GSSSTSYSKFIGALRKALPSNGTVYNITLLLSSASGASRYT |
| P84530 | SYSKFIGALRKALPSNGTVYNITLLLSSASGASRYTLMKLS |
| P84530 | GKAITVAIDVTNVYIMGYLVNSTSYFFNESDAKLASQYVFA |
| P84530 | IDVTNVYIMGYLVNSTSYFFNESDAKLASQYVFAGSTIVTL |
| P84530 | ISLENEWSALSKQIQLAQTNNGTFKTPVVIMDAGGQRVEIG |
| Q9HB40 | TVRKDAYMFWWLYYATNSCKNFSELPLVMWLQGGPGGSSTG |
| Q9HB40 | LQAASLLFVDNPVGTGFSYVNGSGAYAKDLAMVASDMMVLL |
| Q9HB40 | EEDFMKPVISIVDELLEAGINVTVYNGQLDLIVDTMGQEAW |
| Q8TEB7 | ALSPQAPGSRGAEAVWTAYLNVSWRVPHTGVNRTVWELSEE |
| Q8TEB7 | AEAVWTAYLNVSWRVPHTGVNRTVWELSEEGVYGQDSPLEP |
| Q8TEB7 | AGVLVPPDGPGALNACNPHTNFTVPTVWGSTVQVSWLALIQ |
| Q6ZSG1 | PIPQHYQHYLATPRMHHFPRNSSSTQMVVHEIRNYPYPQLH |
| P00667 | DPSASSISSSNYCNQMMQSRNLTQDRCKPVNTFVHESLADV |
| P07847 | DSSTSSASSSNYCNQMMKSRNLTQSRCKPVNTFVHESLADV |
| P00668 | DSNPSSVSSSNYCNQMMKSRNLTQGRCKPVNTFVHESLADV |
| P00673 | AVCSQKNVLCKNGRTNCYESNSTMHITDCRQTGSSKYPNCA |
| P61823 | VLLSLLVLVLLLVRVQPSLGKETAAAKFERQHMDSSTSAAS |
| P61823 | VLVLLLVRVQPSLGKETAAAKFERQHMDSSTSAASSSNYCN |
| P61823 | DSSTSAASSSNYCNQMMKSRNLTKDRCKPVNTFVHESLADV |
| P61823 | TSAASSSNYCNQMMKSRNLTKDRCKPVNTFVHESLADVQAV |
| P61823 | SSSNYCNQMMKSRNLTKDRCKPVNTFVHESLADVQAVCSQK |
| P00657 | DSSTSSASSSNYCNQMMKSRNMTSDRCKPVNTFVHESLADV |
| P00664 | DPSPSSASSSNYCNQMMQSRNLTQDRCKPVNTFVHESLADV |
| P67926 | DSSTSSASSSNYCNQMMKSRNLTQDRCKPVNTFVHESLADV |
| P00675 | DSSGSPSTNANYCNEMMKGRNMTQGYCKPVNTFVHEPLADV |
| P00685 | DSGSSLSSSSDYCNKMMKVRNMTQESCKPVNTFVHESLQDV |
| P00659 | BSSTSSASSSBYCBZMMKSRNLTQDRCKPVBTFVHZSLABV |
| P00662 | DSSTSSVSSSNYCNEMMTSRNLTQDRCKPVNTFVHESLADV |
| P00672 | DTSSSLSNDSNYCNQMMVRRNMTQDRCKPVNTFVHESEADV |
| P00674 | DSGTSSSNPTYCNQMMKRRNMTQGWCKPVNTFVHEPLADV |
| P00674 | PVNTFVHEPLADVQAICLQKNITCKNGQSNCYQSSSSMHIT |
| P07998 | DSDSSPSSSSTYCNQMMRRRNMTQGRCKPVNTFVHEPLVDV |
| P07998 | NVCFQEKVTCKNGQGNCYKSNSSMHITDCRLTNGSRYPNCA |
| P07998 | GQGNCYKSNSSMHITDCRLTNGSRYPNCAYRTSPKERHIIV |
| P04060 | DSSGSPSSNSNYCNEMMRRRNMTQDRCKPVNTFVHEPLADV |
| P00682 | DSTVATSSSPTYCNQMMKRRNMTQGYCKPVNTFVHESLADV |
| P00676 | DSRGSPSTNPNYCNEMMKSRNMTQGRCKPVNTFVHEPLADV |
| P00671 | DPDSSSSNSSNYCNLMMSRRNMTQGRCKPVNTFVHESLADV |

| | |
|---|---|
| P00671 | AVCSQINVNCKNGQTNCYQSNSTMHITDCRQTGSSKYPNCA |
| P04059 | DSSGSPSTNPNYCNAMMKSRNMTQERCKPVNTFVHEPLADV |
| P19644 | DSGSSPSSSSTYCNQMMKLRNMTQGSCKSVNTFVHEPLVDV |
| P16414 | SIDSPGNLSPTYCNQMMLRRNMTKGSCKPVNTFVHEPLKDI |
| P67927 | DSSTSSASSSNYCNQMMKSRNLTQDRCKPVNTFVHESLADV |
| P10153 | SLHVKPPQFTWAQWFETQHINMTSQQCTNAMQVINNYQRRC |
| P10153 | NQNTFLLTTFANVVNVCGNPNMTCPSNKTRKNCHHSGSQVP |
| P10153 | LTTFANVVNVCGNPNMTCPSNKTRKNCHHSGSQVPLIHCNL |
| P10153 | SNKTRKNCHHSGSQVPLIHCNLTTPSPQNISNCRYAQTPAN |
| P10153 | HHSGSQVPLIHCNLTTPSPQNISNCRYAQTPANMFYIVACD |
| P81649 | TIQHIQPSPLQCNKAMNGVNNYTWHCKPQNTFLHDSFQDVA |
| P81649 | PQNTFLHDSFQDVATACNLPNITCKNGQNNCHQSAKPVSLT |
| P81649 | CKNGQNNCHQSAKPVSLTQCNFTGGNYPNCRYKDAAQYKFF |
| P39873 | PVNTFVHESLDDVKAVCSQKNITCKNGHPNCYQSKSTMSIT |
| O43567 | LINSKPENACEPIVPPPVKDNSSGTFIVLIRRLDCNFDIKV |
| P19791 | WPDNCDGSYQEYCDDSREYSNITSILEAQDRTELLSYMKEY |
| Q7SID5 | VLTWPASFCYANHCERIAPNNFTIHGLWPDNVKTRLHNCKP |
| P00679 | DPEGSPSNSSNYCNVMMIRRNMTQGRCKPVNTFVHESLADV |
| O80322 | RNGPDPEKCKTTALNSQKIGNMTAQLEIIWPNVLNRSDHVG |
| O80322 | NSQKIGNMTAQLEIIWPNVLNRSDHVGFWEKEWIKHGTCGY |
| O80322 | IKDDMHYLQTVIRMYITQKQNVSAILSKAAIQPNGTNRPLV |
| O80322 | MYITQKQNVSAILSKAAIQPNGTNRPLVDIENAIRRGTNNT |
| O80322 | QPNGTNRPLVDIENAIRRGTNNTKPKFKCQKNTRTTTELVE |
| P04007 | LVLTWPITFCRIKHCERTPTNFTIHGLWPDNHTTMLNYCDR |
| P04007 | RIKHCERTPTNFTIHGLWPDNHTTMLNYCDRSKPYNMFTDG |
| P04007 | LSSLRNHGISRGFSYTVQNLNNTIKAITGGFPNLTCSRLRE |
| Q40965 | KTKRYRKIQRLEPQLEIIWPNVSDRKANRGFWRKQWYKHGS |
| Q40965 | LPNQKHYFETVIRMFLAEKQNVSRILSMATIEPEGKNRTLL |
| Q40965 | AEKQNVSRILSMATIEPEGKNRTLLEIQNAIRAGTNNNMIPK |
| Q40965 | KCQKVNGMTELVEVTLCHDSNLTQFINCPRPLPQASPYFCP |
| O80323 | TVGYDYFQFTQQYQLAVCNSNRTLCKDPPDKLFTVHGLWPS |
| O80323 | IDNENHYFETVIKMYISKKQNVSRILSKAKIEPDGKKRALL |
| Q40966 | RNGPDPEKCKTTTMNSQKIGNMTAQLEIIWPNVLNRSDHVG |
| Q40966 | NSQKIGNMTAQLEIIWPNVLNRSDHVGFWEREWLKHGTCGY |
| Q40966 | IKDDMHYLKTVIKMYITQKQNVSAILSKATIQPNGNNRSLV |
| Q40966 | TQKQNVSAILSKATIQPNGNNRSLVDIENAIRSGNNNTKPK |
| Q40966 | QPNGNNRSLVDIENAIRSGNNNTKPKFKCQKNTRTTTELVE |
| P93460 | TVGYDYFQFTQQYQLAVCNSNRTPCKDPPDKLFTVHGLWPS |
| P93460 | IDNENHYFETVIKMYISKKQNVSRILSKAKIEPDGKKRALL |
| O80324 | FTVHGLWPSNDVGDDPIYCKNKTIKSQQIGNLTAQLIIIWP |
| O80324 | DVGDDPIYCKNKTIKSQQIGNLTAQLIIIWPNVLDRTDHVG |

| | |
|---|---|
| O80324 | IKDEMHYFKTVIKMYITQKQNVSEILSRAKIEPEGKIRRRD |
| O80324 | INAIRLGTKDKKPKLKCQKNNQTTELVEITICSDRNLTQFI |
| O80324 | KCQKNNQTTELVEITICSDRNLTQFIDCPRSSFKGSPFHCP |
| Q40381 | LVLQWPTAFCHTTPCKRIPNNFTIHGLWPDNVSTTLNYCAA |
| Q40381 | HTTPCKRIPNNFTIHGLWPDNVSTTLNYCAAKENFKNIEDD |
| Q40381 | SLRNHGIIPGRGMKYTVQKINSTIKKITQGYPNLSCTKGIM |
| O80325 | DKLFTVHGLWPSNLNGPHPENCTNATVNPHRIKNIQAQLKI |
| O80325 | FTVHGLWPSNLNGPHPENCTNATVNPHRIKNIQAQLKIIWP |
| O80325 | VGFWNKQWIKHGSCGYPAIMNDTHYFQTVINMYITQKQNVS |
| O80325 | IMNDTHYFQTVINMYITQKQNVSEILSKAKIEPLGIQRPLV |
| O80325 | AKIEPLGIQRPLVHIENAIRNSTNNKKPKFKCQKNSGVTEL |
| P10281 | AAGAVFEFPSCPKDIPFSCQNSTAVADSCCFNSPGGALLQT |
| P10281 | WPDNCDGSYGQFCDKSREYSNITAILQEQGRTELLSYMKKY |
| O00584 | RDPPDYWTIHGLWPDKSEGCNRSWPFNLEEIKDLLPEMRAY |
| O00584 | IKDLLPEMRAYWPDVIHSFPNRSRFWKHEWEKHGTCAAQVD |
| O00584 | EVQTIGQIELCLTKQDQQLQNCTEPGEQPSPKQEVWLANGA |
| P24657 | WPDNCDGSFPQTCDASRAYTNITDILTAMGADDTLQYMQTY |
| Q8WZ75 | QEPQDYTEPVELLAVRIQLENVTLLNPDPAEGPKPRPAVWL |
| Q8WZ75 | LRLPEKVPSAPPQEVTLKPGNGTVFVSWVPPPAENHNGIIR |
| Q8WZ75 | PPPAENHNGIIRGYQVWSLGNTSLPPANWTVVGEQTQLEIA |
| Q8WZ75 | NGIIRGYQVWSLGNTSLPPANWTVVGEQTQLEIATHMPGSY |
| Q8WZ75 | LRGSHSLELRACELGNRGSKNLSQSPGAVPQALVAWRALGP |
| Q8I7I5 | RNLAYPLDSGEVHWTGLAEYNYSSRISSCRHGCEDVDERES |
| Q8I7I5 | KPLNGILGWRWTSLPQNSFRNSSLSSEVHVPFEHGEHVEVR |
| Q8I7I5 | YVNQVEIELVSVASSRLIFSNGTHLLESEIDQYALGDSATV |
| Q8I7I5 | DTTIAIGSSKGSLWTYQMSANQTDEDQPSSVIQLKTVGEMD |
| Q8I7I5 | GHVYTLNADNKVYRTEMVAFNATGIETVASLQYLQDMSPSN |
| Q8I7I5 | SPSNGIFFDVSKFLLYSALQNGSMMTLNPVTDHVHIFKDVG |
| Q8I7I5 | RVSWDESNTLPFQAQGSSWRNFTYFLKVTAPDITDFSPIEI |
| Q8I7I5 | IPADDSVKIVEKLQYPHVLDNTTKTVYLAGDHSMGIFKKYL |
| Q8I7I5 | VMGISSDDTTGMVHFLTQARNGTITLWESDPENRTPRDIAS |
| Q8I7I5 | IFREKMGGQDPPIITIQSETNFTIPSEVLEAWSSAQRFDVS |
| Q8I7I5 | SAQRFDVSVQAMTPWATAVLNRTGLTAPVKPPTSPTQLRIF |
| Q8I7I5 | ISFFWGPPSEWNGTPYQYIVNCTKDDGSWIGGPVTTSQSHY |
| Q8I7I5 | TDAKQLAYDPFMNTAYLLTKNGSLFALDMNKNTEANLALTV |
| Q8I7I5 | AGISVPTSIAVIALSETVSSNISSDTTSCLINPCRVKIANL |
| Q8I7I5 | AVSSEAVARTLDVPGTLRPDNVTGSSLLLRWNGLEPEHRPS |
| Q8I7I5 | SIAVQYRESGGANNEWQFPMNVSFEPDVTTELVPITNLLSA |
| Q8I7I5 | KEPMSDGGSPITSYAVETRINKTAEWEIAERGLDGWKTWWR |
| Q04912 | VPSFSAGGLVQAMVTYEGDRNESAVFVAIRNRLHVLGPDLK |
| Q04912 | DFFQSPSFCPNPPGLEALSPNTSCRHFPLLVSSSFSRVDLF |

| | |
|---|---|
| Q04912 | LFNGLLGPVQVTALYVTRLDNVTVAHMGTMDGRILQVELVR |
| Q04912 | DGRILQVELVRSLNYLLYVSNFSLGDSGQPVQRDVSRLGDH |
| Q04912 | FVEEFECELEPLGTQAVGPTNVSLTVTNMPPGKHFRVDGTS |
| Q04912 | TCLTLEGQSLSVGTSRAVLVNGTECLLARVSEGQLLCATPP |
| Q04912 | QQLCRLPEYVVRDPQGWVAGNLSARGDGAAGFTLPGFRFLP |
| Q04912 | EHAIKFEYIGLGAVADCVGINVTVGGESCQHEFRGDMVVCP |
| Q03691 | GNPRNPTCPMASLIYQHGTYNISENGTLVLNPIEVDGRQLF |
| Q03691 | NPTCPMASLIYQHGTYNISENGTLVLNPIEVDGRQLFSDPC |
| Q03691 | GRQLFSDPCNDDGVSTYSRYNQTETFKEYAVGIDPYHGIYT |
| P04843 | SFKTILPAAAQDVYYRDEIGNVSTSHLLILDDSVEMEIRPR |
| P04844 | SLFYAAQASQALSGCEISISNETKDLLLAAVSEDSSVTQIY |
| Q9BZR6 | IFLHGNRISHVPAASFRACRNLTILWLHSNVLARIDAAAFT |
| Q9BZR6 | LYLQDNALQALPDDTFRDLGNLTHLFLHGNRISSVPERAFR |
| Q9HBX9 | YFSHGGGQDVKCSLGYFPCGNITKCLPQLLHCNGVDDCGNQ |
| Q9HBX9 | QGLELDCDETNLRAVPSVSSNVTAMSLQWNLIRKLPPDCFK |
| Q9HBX9 | QHMPRLHWLDLEGNHIHNLRNLTFISCSNLTVLVMRKNKIN |
| Q9HBX9 | LDLEGNHIHNLRNLTFISCSNLTVLVMRKNKINHLNENTFA |
| Q9HBX9 | NKIENLPPLIFKDLKELSQLNLSYNPIQKIQANQFDYLVKL |
| Q9HBX9 | LSLEGIEISNIQQRMFRPLMNLSHIYFKKFQYCGYAPHVRS |
| P55012 | VVYIGIAVSVGSCVVRDATGNVNDTITTELTNCTSAACKLN |
| P55012 | YIGIAVSVGSCVVRDATGNVNDTITTELTNCTSAACKLNFD |
| P55012 | SCVVRDATGNVNDTITTELTNCTSAACKLNFDFSYCESNTC |
| Q9JIS8 | YAGGIKSIFDPPVFPVCMLGNRTLSRDQFDICAKTVVVDNE |
| Q9JIS8 | GNRTLSRDQFDICAKTVVVDNETVATRLWTFFCHSPNLTAD |
| Q9JIS8 | VVVDNETVATRLWTFFCHSPNLTADSCDPYFLLNNVTEIPG |
| Q9JIS8 | SFTVLVGIFFPSVTGIMAGSNRSGDLRDAQKSIPVGTILAI |
| Q9WVL3 | YAGVIKTAFAPPDIPVCLLGNRTLANRNFDTCAKMQVVSNG |
| Q9WVL3 | GNRTLANRNFDTCAKMQVVSNGTVTTALWRLFCNGSSLGAT |
| Q9WVL3 | AKMQVVSNGTVTTALWRLFCNGSSLGATCDEYFAQNNVTEI |
| Q9WVL3 | RLFCNGSSLGATCDEYFAQNNVTEIQGIPGVASGVFLDNLW |
| Q99MR3 | LLVSGSLASVLVSFVAVGPRNIPLAPRPGTNASSVPHRHGH |
| Q99MR3 | LVSFVAVGPRNIPLAPRPGTNASSVPHRHGHFTGFNGSTLR |
| Q99MR3 | PRPGTNASSVPHRHGHFTGFNGSTLRDNLGAGYAEDYTTGA |
| Q9JIP7 | YYGMRALLVLYFRNFLGWDDNLSTAIYHTFVALCYLTPILG |
| Q9JIP7 | LPVFPGGNQVQIKVLNIGNNNMTVHFPGNSVTLAQMSQTDT |
| Q9JIP7 | AQMSQTDTFMTFDIDKLTSINISSSGSPGVTTVAHDFEQGH |
| Q9JIP7 | LNEMVTIKMSGKVYENVTSHNASGYQFFPSGEKQYTINTTA |
| Q9JIP7 | TSHNASGYQFFPSGEKQYTINTTAVAPTCLTDFKSSNLDFG |
| Q9BZV2 | FSMMRPSEPFLIPYLSGPDKNLTSAEITNEIFPVWTYSYLV |
| Q9BZV2 | VTLAAYTAGSVLAQLLVSLANMSYFYLNVISLASVSVAFLF |
| P21453 | KAHRSSVSDYVNYDIIVRHYNYTGKLNISADKENSIKLTSV |

| | |
|---|---|
| P21453 | VSDYVNYDIIVRHYNYTGKLNISADKENSIKLTSVVFILIC |
| Q08357 | LLGAKVGETIRKGIIDVNLYNETVETLMAGEVSAMVGSAVW |
| O76082 | LSSVFLIATPEHRCRVPDAANLSSAWRNHTVPLRLRDGREV |
| O76082 | ATPEHRCRVPDAANLSSAWRNHTVPLRLRDGREVPHSCRRY |
| O76082 | LRDGREVPHSCRRYRLATIANFSALGLEPGRDVDLGQLEQE |
| Q4U2R8 | IQVTLVVLPLLLMASHNTLQNFTAAIPTHHCRPPADANLSK |
| Q4U2R8 | TLQNFTAAIPTHHCRPPADANLSKNGGLEVWLPRDRQGQPE |
| Q4U2R8 | QGQPESCLRFTSPQWGLPFLNGTEANGTGATEPCTDGWIYD |
| Q4U2R8 | SCLRFTSPQWGLPFLNGTEANGTGATEPCTDGWIYDNSTFP |
| Q4U2R8 | GTEANGTGATEPCTDGWIYDNSTFPSTIVTEWDLVCSHRAL |
| Q8VC69 | IQVTMVVAPLLLMASHNTLQNFTAAIPAHHCRPPANANLSK |
| Q8VC69 | TLQNFTAAIPAHHCRPPANANLSKDGGLEAWLPLDKQGRPE |
| Q8VC69 | WLPLDKQGRPESCLRFPFPHNGTEANGTGVTEPCLDGWVYD |
| Q8VC69 | KQGRPESCLRFPFPHNGTEANGTGVTEPCLDGWVYDNSTFP |
| Q8VC69 | GTEANGTGVTEPCLDGWVYDNSTFPSTIVTEWNLVCSHRAF |
| Q8VC69 | VLILNYLQTAVSGTCAAYAPNYTVYCIFRLLSGMSLASIAI |
| Q9Y694 | REPDGTLSSCLRFAYPQALPNTTLGEERQSRGELEDEPATV |
| Q9UHI7 | AKAILALERWKCPPEEEIYGNWSLPLNTSHIWHPRIREVQG |
| Q9UHI7 | LERWKCPPEEEIYGNWSLPLNTSHIWHPRIREVQGAIMVSS |
| P40879 | LSMMVGLAVSGAVSKAVPDRNATTLGLPNNSNNSSLLDDER |
| P40879 | VSGAVSKAVPDRNATTLGLPNNSNNSSLLDDERVRVAAAAS |
| P40879 | VSKAVPDRNATTLGLPNNSNNSSLLDDERVRVAAAASVTVL |
| Q62674 | CVAGILYVPRGVEVDCVSLLNQTVSSSSFEVYLCCRQVFQS |
| Q99808 | PWNFFMTATQYFTNRLDMSQNVSLVTAELSKDAQASAAPAA |
| Q14542 | PWNFFITAIPYFQARLAGAGNSTARILSTNHTGPEDAFNFN |
| Q14542 | PYFQARLAGAGNSTARILSTNHTGPEDAFNFNNWVTLLSQL |
| Q14542 | MSIVCYLSLPHLKFARYYLANKSSQAQAQELETKAELLQSD |
| Q9JIM1 | PWNFFMTATKYFTNRLDVSQNVSSDTDQSCESTKALADPTV |
| Q969I6 | VIYKKFQIPCPLPVLDHSVGNLSFNNTLPMHVVMLPNNSES |
| Q969I6 | KFQIPCPLPVLDHSVGNLSFNNTLPMHVVMLPNNSESSDVN |
| Q969I6 | HSVGNLSFNNTLPMHVVMLPNNSESSDVNFMMDYTHRNPAG |
| Q13433 | DLAISTRQYHLQQLFYRYGENNSLSVEGFRKLLQNIGIDKI |
| Q13433 | LAISTRQYHLQQLFYRYGENNSLSVEGFRKLLQNIGIDKIK |
| Q13433 | STPPSVTSKSRVSRLAGRKTNESVSEPRKGFMYSRNTNENP |
| Q13433 | EPRKGFMYSRNTNENPQECFNASKLLTSHGMGIQVPLNATE |
| Q13433 | ECFNASKLLTSHGMGIQVPLNATEFNYLCPAIINQIDARSC |
| Q13433 | SAMLAYLGMATGIFIGHYAENVSMWIFALTAGLFMYVALVD |
| Q8C145 | DLAVTMQRHHLQQLFYRYGENDSLSVEGFRKLLQNIGIDKI |
| Q8C145 | PSITEKSRVGRLSRLARKKSNESVSEPRKSFMYSRNTNDNI |
| Q8C145 | EPRKSFMYSRNTNDNIQECFNTTKLLTSHGMSIQALLNATE |
| Q8C145 | ECFNTTKLLTSHGMSIQALLNATEFNYLCPAIINQIDARAC |

| | |
|---|---|
| Q8C145 | SAMLAYLGMATGIFIGHYAENVSMWIFALTAGLFMYVALVD |
| Q6P5F6 | LHDRNHRFHHRHRLHHHLDHNTTRHVHNDSVAHSEHGEPGH |
| Q6P5F6 | FHHRHRLHHHLDHNTTRHVHNDSVAHSEHGEPGHSPSPETN |
| Q6P5F6 | NDSVAHSEHGEPGHSPSPETNKTQEQSEVKSVKVRRKEKGK |
| Q6P5F6 | IYSTPSHKDQSEDDRQHECLNVTQLLKHFGLGPNSPISPDL |
| Q15043 | LTLQQLKALLNHLDVGVGRGNVTQHVQGHRNLSTCFSSGDL |
| Q15043 | VQGHRNLSTCFSSGDLFTAHNFSEQSRIGSSELQEFCPTIL |
| Q75N73 | GLPPLSATSFLEDLMDRYGKNDSLTLTQLKSLLDHLHVGVG |
| Q75N73 | LTLTQLKSLLDHLHVGVGRDNVSQPKEGPRNLSTCFSSGDL |
| Q75N73 | DHLHVGVGRDNVSQPKEGPRNLSTCFSSGDLFAAHNLSERS |
| Q75N73 | KEGPRNLSTCFSSGDLFAAHNLSERSQIGASEFQEFCPTIL |
| Q9Y6R1 | YNTLFSCTCVPPDPANISISNDTTLAPEYLPTMSSTDMYHN |
| Q9Y6R1 | NDTTLAPEYLPTMSSTDMYHNTTFDWAFLSKKECSKYGGNL |
| Q9JI66 | YNTHFSCACLPPDPVNLSVSNDTTLAPEDLPTVSSTDMYHN |
| Q9JI66 | NDTTLAPEDLPTVSSTDMYHNATFDWAYLSKKECVKFGGKL |
| Q9Y6M7 | KPYVATLSLHSLFELRSCILNGTVMLDMRASTLDEIADMVL |
| Q9Y6M7 | RNGEGLSASRHSLRTGLSASNLSLRGESPLSLLLGHLLPSS |
| Q9Y6M7 | ASPQSAPGNLDNSKSGEIKGNGSGGSRENSTVDFSKVDMNF |
| Q9Y6M7 | NLDNSKSGEIKGNGSGGSRENSTVDFSKVDMNFMRKIPTGA |
| Q9Y6M7 | NNLDKLTSYSCVCTEPPNPSNETLAQWKKDNITAHNISWRN |
| Q9Y6M7 | CVCTEPPNPSNETLAQWKKDNITAHNISWRNLTVSECKKLR |
| Q9Y6M7 | PPNPSNETLAQWKKDNITAHNISWRNLTVSECKKLRGVFLG |
| Q8BTY2 | KPYVATLSLHSLFELRSCILNGTVMLDMRASTLDEIADMVL |
| Q8BTY2 | NLDNSKSGEMKGNGSGGSRENSTVDFSKVDMNFMRKIPTGA |
| Q8BTY2 | NNLDELTSYTCVCAEPSNPSNETLELWKRKNITAYSVSWGN |
| Q8BTY2 | CVCAEPSNPSNETLELWKRKNITAYSVSWGNLTVSECKTFH |
| Q8BTY2 | NETLELWKRKNITAYSVSWGNLTVSECKTFHGMFVGSACGP |
| P35542 | KLISRSRVYLQGLIDCYLFGNSSTVLEDSKSNEKAEEWGRS |
| P20840 | IREGDEFTLSMPHVYRIKLLNSSQTATISLADGTEAFKCYV |
| P20840 | ADGTEAFKCYVSQQAAYLYENTTFTCTAQNDLSSYNTIDGS |
| P20840 | TAQNDLSSYNTIDGSITFSLNFSDGGSSYEYELENAKFFKS |
| P20840 | SSVQVYSSNDFNDWWFPQSYNDTNADVTCFGSNLWITLDEK |
| P20840 | NVNTIDHALEFQYTCLDTIANTTYATQFSTTREFIVYQGRN |
| P20840 | LTSINTSAYSTGSISTVETGNRTTSEVISHVVTTSTKLSPT |
| P20840 | SPTATTSLTIAQTSIYSTDSNITVGTDIHTTSEVISDVETI |
| P20840 | WTGAMNTYISQFTSSSFATINSTPIISSSAVFETSDASIVN |
| P20840 | ISSSAVFETSDASIVNVHTENITNTAAVPSEEPTFVNATRN |
| P20840 | VHTENITNTAAVPSEEPTFVNATRNSLNSFCSSKQPSSPSS |
| P20840 | SSLIAYPSSASGSQLSGIQQNFTSTSLMISTYEGKASIFFS |
| P81608 | ENIEENGSMRVFVEHIRVLDNSSLAFKFQRKVNGECTDFYA |
| P02743 | PRESVTDHVNLITPLEKPLQNFTLCFRAYSDLSRAYSLFSY |

| | |
|---|---|
| P12246 | PRESETDHVKLIPHLEKPLQNFTLCFRTYSDLSRSQSLFSY |
| P17900 | KDPAVIRSLTLEPDPIIVPGNVTLSVMGSTSVPLSSPLKVD |
| Q8C1C1 | GAVCHDCVQLISLLQDALESNLTLAEVTVQNQCQSMGPGLA |
| Q8C1C1 | GLTCDVCLNLVQELDKWLVTNSTEALISHTLERVCTVVPEP |
| P20097 | SVTCKACEYVVKKVMELIDNNRTEEKIIHALDSVCALLPES |
| P26779 | SLPCDICKDVITAAGNLLKDNATEQEILMYLERTCDWLPKP |
| P26779 | ATEQEILMYLERTCDWLPKPNMSASCKEIVDSYLPVILDMI |
| P26779 | GNVCQDCIQLVTDVQEALRTNSTFVEALVDHAKEECDRLGP |
| P26779 | DIYCEVCEFVVKEVAKLIDNNRTEEEILHALDKVCSKLPTS |
| P26779 | GGFCEVCKKLVGYLDRNLEKNSTKEQILAALEKGCSFLPDQ |
| P07602 | SLPCDICKDVVTAAGDMLKDNATEEEILVYLEKTCDWLPKP |
| P07602 | ATEEEILVYLEKTCDWLPKPNMSASCKEIVDSYLPVILDII |
| P07602 | GDVCQDCIQMVTDIQTAVRTNSTFVQALVEHVKEECDRLGP |
| P07602 | DVYCEVCEFLVKEVTKLIDNNKTEKEILDAFDKMCSKLPKS |
| P07602 | GGFCEVCKKLVGYLDRNLEKNSTKQEILAALEKGCSFLPDP |
| Q61207 | SLPCDICKTVVTEAGNLLKDNATQEEILHYLEKTCEWIHDS |
| Q61207 | EDVCQDCMKLVSDVQTAVKTNSSFIQGFVDHVKEDCDRLGP |
| Q61207 | VILCQTCQFVMNKFSELIVNNATEELLVKGLSNACALLPDP |
| Q61207 | GGFCEVCKKLVLYLEHNLEKNSTKEEILAALEKGCSFLPDP |
| P43007 | SNLVVAAFRTYATDYKVVTQNSSSGNVTHEKIPIGTEIEGM |
| P43007 | AAFRTYATDYKVVTQNSSSGNVTHEKIPIGTEIEGMNILGL |
| O35874 | NSGPPPVSKETVDSFLDLLRNLFPSNLVVAAFTTSATDYTV |
| O35874 | PVSKETVDSFLDLLRNLFPSNLVVAAFTTSATDYTVVTHNT |
| O35874 | SNLVVAAFTTSATDYTVVTHNTSSGNVTKEKIPVVTDVEGM |
| O35874 | AAFTTSATDYTVVTHNTSSGNVTKEKIPVVTDVEGMNILGL |
| P13866 | GYDAFMEKYMKAIPTIVSDGNTTFQEKCYTPRADSFHIFRD |
| P31641 | AWATYYLFQSFQKELPWAHCNHSWNTPHCMEDTMRKNKSVW |
| P31641 | WAHCNHSWNTPHCMEDTMRKNKSVWITISSTNFTSPVIEFW |
| P31641 | HCMEDTMRKNKSVWITISSTNFTSPVIEFWERNVLSLSPGI |
| P97260 | YHAKFLSSLRARLMLLHPSPNCSLRAENLVHVHFKEEIGIA |
| P97260 | ASRPDPAFSIFPPDAPKLPENQTVPGELPEHAAPAEGVHDS |
| P97260 | DEELWRRLSFRHWPTLFNYYNITLAKRYISLLPVIPVTLRL |
| P21583 | ISEMVVQLSDSLTDLLDKFSNISEGLSNYSIIDKLVNIVDD |
| P21583 | YSIIDKLVNIVDDLVECVKENSSKDLKKSFKSPEPRLFTPE |
| P21583 | KSFKSPEPRLFTPEEFFRIFNRSIDAFKDFVVASETSDCVV |
| P21583 | SRVSVTKPFMLPPVAASSLRNDSSSSNRKAKNPPGDSSLHW |
| P21581 | LRDMVTHLSVSLTTLLDKFSNISEGLSNYSIIDKLGKIVDD |
| P21581 | CMEENAPKNVKESLKKPETRNFTPEEFFSIFNRSIDAFKDF |
| P21581 | ESLKKPETRNFTPEEFFSIFNRSIDAFKDFMVASDTSDCVL |
| P21581 | SRVSVTKPFMLPPVAASSLRNDSSSSNRKAAKSPEDPGLQW |
| P37089 | EELEELDRITEQTLFDLYKYNSSYTRQAGARRRSSRDLLGA |

| | |
|---|---|
| P37089 | DNNPQVDRKDWKIGFQLCNQNKSDCFYQTYSSGVDAVREWY |
| P37089 | EALGNFIFTCRFNQAPCNQANYSKFHHPMYGNCYTFNDKNN |
| P37089 | ANYSKFHHPMYGNCYTFNDKNNSNLWMSSMPGVNNGLSLTL |
| P37089 | ISMRKEALDSLGGNYGDCTENGSDVPVKNLYPSKYTQQVCI |
| P37089 | SRWPSVKSQDWIFEMLSLQNNYTINNKRNGVAKLNIFFKEL |
| Q14108 | VARVFQKAVDQSIEKKIVLRNGTEAFDSWEKPPLPVYTQFY |
| Q14108 | EAFDSWEKPPLPVYTQFYFFNVTNPEEILRGETPRVEEVGP |
| Q14108 | EVGPYTYRELRNKANIQFGDNGTTISAVSNKAYVFERDQSV |
| Q14108 | SLIHVFRPDISPYFGLFYEKNGTNDGDYVFLTGEDSYLNFT |
| Q14108 | EKNGTNDGDYVFLTGEDSYLNFTKIVEWNGKTSLDWWITDK |
| Q14108 | VEWNGKTSLDWWITDKCNMINGTDGDSFHPLITKDEVLYVF |
| Q14108 | YESVQGLPAFRYKVPAEILANTSDNAGFCIPEGNCLGSGVL |
| Q14108 | TSDNAGFCIPEGNCLGSGVLNVSICKNGAPIIMSFPHFYQA |
| Q14108 | LDDFVETGDIRTMVFPVMYLNESVHIDKETASRLKSMINTT |
| Q14108 | YLNESVHIDKETASRLKSMINTTLIITNIPYIIMALGVFFG |
| Q8WTV0 | EKPQVRERGPYVYREFRHKSNITFNNNDTVSFLEYRTFQFQ |
| Q8WTV0 | ERGPYVYREFRHKSNITFNNNDTVSFLEYRTFQFQPSKSHG |
| Q8WTV0 | MTLKLIMTLAFTTLGERAFMNRTVGEIMWGYKDPLVNLINK |
| Q8WTV0 | NKYFPGMFPFKDKFGLFAELNNSDSGLFTVFTGVQNISRIH |
| Q8WTV0 | LFAELNNSDSGLFTVFTGVQNISRIHLVDKWNGLSKVDFWH |
| Q8WTV0 | DKWNGLSKVDFWHSDQCNMINGTSGQMWPPFMTPESSLEFY |
| Q8WTV0 | SGVFEGIPTYRFVAPKTLFANGSIYPPNEGFCPCLESGIQN |
| Q8WTV0 | NGSIYPPNEGFCPCLESGIQNVSTCRFSAPLFLSHPHFLNA |
| Q8WTV0 | PNQEAHSLFLDIHPVTGIPMNCSVKLQLSLYMKSVAGIGQT |
| O35114 | VARVFQKAVDQTIEKNMVLQNGTKVFNSWEKPPLPVYIQFY |
| O35114 | KVFNSWEKPPLPVYIQFYFFNVTNPEEILQGEIPLLEEVGP |
| O35114 | EVGPYTYRELRNKANIQFGENGTTISAVTNKAYVFERNQSV |
| O35114 | FGENGTTISAVTNKAYVFERNQSVGDPNVDLIRTINIPLLT |
| O35114 | SLVHIFKPDVSPNFGLFYERNGTNDGEYVFLTGEDNYLNFS |
| O35114 | ERNGTNDGEYVFLTGEDNYLNFSKIVEWNGKTSLDWWTTDT |
| O35114 | VEWNGKTSLDWWTTDTCNMINGTDGDSFHPLISKDEVLYLF |
| O35114 | FENVEGLPAFRYKVPAEILANTSENAGFCIPEGNCMDSGVL |
| O35114 | TSENAGFCIPEGNCMDSGVLNISICKNGAPIIMSFPHFYQA |
| O35114 | LDDFVETGDIRTMVFPVMYLNESVLIDKETANQLKSVINTT |
| O35114 | YLNESVLIDKETANQLKSVINTTLVVTNIPYIIMALGVFFG |
| P18828 | QIVAVNVPPEDQDGSGDDSDNFSGSGTGALPDTLSRQTPST |
| Q9N3X8 | EATNAAGADLASVWVKEGEANETVATEMSEDGMSLEEEISM |
| Q9N3X8 | VGPDNTGEYRCRVTVDGREENASAMLQIIEKPAMPERVRAE |
| Q9N3X8 | AMLQIIEKPAMPERVRAELHNETMPAKVRVRWNEGFDGNEP |
| Q9N3X8 | LGYVVRYRLAGYSSLTWNEKNLTTKDARNTLVDELITWREY |
| Q9N3X8 | VTTSEGRPTQAPKNVRVKVLNSTAVAIEFTAPEQQRIPGVN |

| | |
|---|---|
| Q9N3X8 | DPDRRQLTTVVNELEKFGHYNLTTLCFTTPGDGPRSNVVKV |
| Q9N3X8 | HWAASSPDVKTKHEVDGSTTNFTIDGLQPSTRYGVDVMAST |
| Q9N3X8 | IRQWVVEGKMADSSVFAHVFNVSAPKARSITVTGLRPFTQY |
| Q9N3X8 | KSTWNSDAIGYRIHYREYPSNETWQMEEIAIRDEHEDKEEK |
| Q9N3X8 | TYFFSVWAETSAGKGEQRSANVTIGPSKDGPPPPSKPQITS |
| O97394 | AGSIFSEKSDVVVAYMGIFENTTEGRLTVISGHPAIFDMPP |
| O97394 | RAKAINTQLGKEESSAFVHLNVSGDPYIEVAPEIIVRPQDV |
| O97394 | LKDGLAVETAGVRHTLNDPWNRTLALLQANSSHSGEYTCQV |
| O97394 | AGVRHTLNDPWNRTLALLQANSSHSGEYTCQVRLRSGGYPA |
| O97394 | SASTWLRVKTSAPIMELPPQNVTALDGKDATISCRAVGSPN |
| O97394 | TALDGKDATISCRAVGSPNPNITWIYNETQLVDISSRVQIL |
| O97394 | DATISCRAVGSPNPNITWIYNETQLVDISSRVQILESGDLL |
| O97394 | QAVRASDVGSYACVVTSPGGNETRAARLSVIELPFPPSNVK |
| O97394 | PFPPSNVKVERLPEPQQRSINVSWTPGFDGNSPISKFIIQR |
| O97394 | LEKFVGPVPDPLLNWITELSNVSADQRWILLENLKAATVYQ |
| O97394 | LGYILRYRLFGYNNVPWSYQNITNEAQRNFLIQELITWKDY |
| O97394 | IKTKEGVPEAPPTNVKVEAINSTAARCRWTPPNPQQINGIN |
| O97394 | IDPLAEQTAILGGLEKFTEYNISVLCFTDPGDGVASSQVAV |
| O97394 | LTGYTVRYQVKDRPDTLKSFNLTADDTELTVNQLQATTHYW |
| O97394 | ALSNIEAFSVVLQFTPGFDGNSSITKWKVEGQTARNMTWFT |
| O97394 | PGFDGNSSITKWKVEGQTARNMTWFTICEINDPDAETLTVT |
| O97394 | PSEATKDFQTIQARPKHPPFNVTVRAMSAQQLRVRWIPLQQ |
| O97394 | LRVRWIPLQQTEWYGNPRGYNISYKQLVKTPGTIKYVPRSV |
| O97394 | TAVERTREAVPSYGPLDVQANATSSTTVVVQWGEVPRQHRN |
| O97394 | KVFYAAADRGQQVLHKTIPNNATFTTTLTELKKYVVYHVQV |
| O97394 | ALSTPPIRVQTFEDTPGVPSNVSFPDVSLTMARIIWDVPVD |
| O97394 | WDVPVDPNGKILAYQVTYTLNGSAMLNYSREFPPSDRTFRA |
| O97394 | PNGKILAYQVTYTLNGSAMLNYSREFPPSDRTFRATELLPG |
| O97394 | TYETTEENEKFSKQVKQKVSNTTLRVQNLEEEVTYTFTVRA |
| O97394 | TYTFTVRAQTSVDYGPGISENVTTGPQDGSPVAPRDLILTK |
| Q9UBV2 | AKRRQMQEAEMMYQTGMKILNGSNKKSQKREAYRYLQKAAS |
| Q9UBV2 | SNKKSQKREAYRYLQKAASMNHTKALERVSYALLFGDYLPQ |
| Q9UBV2 | EGSPKGQTALGFLYASGLGVNSSQAKALVYYTFGALGGNLI |
| Q9UBV2 | HAMAFLGKMYSEGSDIVPQSNETALHYFKKAADMGNPVGQS |
| Q9UBV2 | VAQSNAAFILDQREASIVGENETYPRALLHWNRAASQGYTV |
| Q9BYH1 | YIDSSDYPLLPLNNFLECTYNVTVYTGYGVELQVKSVNLSD |
| Q9BYH1 | CTYNVTVYTGYGVELQVKSVNLSDGELLSIRGVDGPTLTVL |
| Q9BYH1 | SDGELLSIRGVDGPTLTVLANQTLLVEGQVIRSPTNTISVY |
| Q9BYH1 | AHFHCHLGYELQGAKMLTCINASKPHWSSQEPICSAPCGGA |
| Q9BYH1 | KPHWSSQEPICSAPCGGAVHNATIGRVLSPSYPENTNGSQF |
| Q9BYH1 | GAVHNATIGRVLSPSYPENTNGSQFCIWTIEAPEGQKLHLH |

| | |
|---|---|
| Q9BYH1 | HFERLLLHDKDRMTVHSGQTNKSALLYDSLQTESVPFEGLL |
| Q9BYH1 | NIRFEAFEKGHCYEPYIQNGNFTTSDPTYNIGTIVEFTCDP |
| Q9BYH1 | HSLEQGPAIIECINVRDPYWNDTEPLCRAMCGGELSAVAGV |
| Q9BYH1 | CIWKIHVGEEKRIFLDIQFLNLSNSDILTIYDGDEVMPHIL |
| Q9BYH1 | AGLIFGKGQGFIMNYIEVSRNDSCSDLPEIQNGWKTTSHTE |
| P30650 | DKICNCYNREYVYNIPEFALNSSRIKPLNHLRLDPPLPFQF |
| Q14242 | EYEYLDYDFLPETEPPEMLRNSTDTTPLTGPGTPESTTVEP |
| Q14242 | TGLDAGGAVTELTTELANMGNLSTDSAAMEIQTTQPAATEA |
| Q14242 | LFIPFSVSSVTHKGIPMAASNLSVNYPVGAPDHISVKQCLL |
| Q95XP4 | AGALFVGSEGAIFRLWAYNINDTGENVFAKKQLVLSESEES |
| Q95XP4 | LRDQQEPRTEIGICVVDPTFNFTAVVVDSGNPEDATSVYSG |
| Q95XP4 | DIGGRNVLRQVWTSFVKARLNCSVSANFPFYFDHIQSVKRV |
| Q24323 | EDRDTLYVGAMDRVFRVNLQNISSSNCNRDVINLEPTRDDV |
| Q24323 | DRLYVCGTNAHNPKDYVIYANLTHLPRSEYVIGVGLGIAKC |
| Q24323 | SEYVIGVGLGIAKCPYDPLDNSTAIYVENGNPGGLPGLYSG |
| Q24323 | SGTNAEFTKADTVIFRTDLYNTSAKRLEYKFKRTLKYDSKW |
| Q24323 | DVGGKNLLAHNWATYLKARLNCSISGEFPFYFNEIQSVYQL |
| Q24323 | SAWLPVLNSRVPEPRPGTCVNDTSNLPDTVLNFIRSHPLMD |
| Q24323 | WDKEANTCRPYELDLLQDVANETSDICDSSVLKKKIVVTYG |
| Q24323 | NEADGGRYDCHLGGSLLCSYNITVDAHRCTPPNKSNDYQKI |
| Q24323 | GGSLLCSYNITVDAHRCTPPNKSNDYQKIYSDWCHEFEKYK |
| O08665 | KLSYKEMLESNNVITFNGLANSSSYHTFLLDEERSRLYVGA |
| O08665 | WAGKDILKECANFIKVLEAYNQTHLYACGTGAFHPICTYIE |
| O08665 | QHHDNHHGPSLEERIIYGVENSSTFLECSPKSQRALVYWQF |
| Q62179 | PRISVPLGSEERLIRKFEAENISNYTALLLSQDGKTLYVGA |
| Q62179 | SVPLGSEERLIRKFEAENISNYTALLLSQDGKTLYVGAREA |
| Q62179 | SQDGKTLYVGAREALFALNSNLSFLPGGEYQELLWSADADR |
| Q62179 | FKGKDPKRDCQNYIKILLPLNSSHLLTCGTAAFSPLCAYIH |
| Q62179 | QVPTPRPGACITNSARERKINSSLQLPDRVLNFLKDHFLMD |
| Q62179 | SHGGLLYASSHSGVVQVPVANCSLYPTCGDCLLARDPYCAW |
| Q62179 | LASRPWTQDIEGASVKELCKNSSYKARFLVPGKPCKQVQIQ |
| Q62179 | ACPLLSNLATRLWVHNGAPVNASASCRVLPTGDLLLVGSQQ |
| Q62179 | VMEEGVMDQKNQRDGTPVIINTSRVSAPAGGRASWGADKSY |
| Q9NPR2 | SLPLGSEERPFLRFEAEHISNYTALLLSRDGRTLYVGAREA |
| Q9NPR2 | SRDGRTLYVGAREALFALSSNLSFLPGGEYQELLWGADAEK |
| Q9NPR2 | HLFTCGTAAFSPMCTYINMENFTLARDEKGNVLLEDGKGRC |
| Q9NPR2 | PVPTPRPGACITNSARERKINSSLQLPDRVLNFLKDHFLMD |
| Q9NPR2 | THRGLLYAASHSGVVQVPMANCSLYRSCGDCLLARDPYCAW |
| Q9NPR2 | ACPLLSNLATRLWLRNGAPVNASASCHVLPTGDLLLVGTQQ |
| O35464 | LHCAGAGFPEDSEPISISHGNYTKQYPVFVGHKPGRNTTQR |
| O35464 | ISHGNYTKQYPVFVGHKPGRNTTQRHRLDIQMIMIMNRTLY |

| | |
|---|---|
| O35464 | KPGRNTTQRHRLDIQMIMIMNRTLYVAARDHIYTVDIDTSH |
| O35464 | MGGSQRVLEKQWTSFLKARLNCSVPGDSHFYFNILQAVTDV |
| O35464 | TMVRYRLTKIAVDNAAGPYQNHTVVFLGSEKGIILKFLARI |
| O35464 | GSEKGIILKFLARIGSSGFLNGSLFLEEMNVYNPEKCSYDG |
| Q92854 | RITWEHREVHLVQFHEPDIYNYSALLLSEDKDTLYIGAREA |
| Q92854 | LLSEDKDTLYIGAREAVFAVNALNISEKQHEVYWKVSEDKK |
| Q92854 | EDKDTLYIGAREAVFAVNALNISEKQHEVYWKVSEDKKAKC |
| Q92854 | SATSLYVCGTNAFQPACDHLNLTSFKFLGKNEDGKGRCPFD |
| Q92854 | GELYSGTSYNFLGSEPIISRNSSHSPLRTEYAIPWLNEPSF |
| Q92854 | FYALFTPQLNNVGLSAVCAYNLSTAEEVFSHGKYMQSTTVE |
| Q92854 | NGPVPKPRPGACIDSEARAANYTSSLNLPDKTLQFVKDHPL |
| Q92854 | LMDDSVTPIDNRPRLIKKDVNYTQIVVDRTQALDGTVYDVM |
| Q92854 | VLKAESPKYGLMGRKNLLIFNLSEGDSGVYQCLSEERVKNK |
| Q92854 | FNLSEGDSGVYQCLSEERVKNKTVFQVVAKHVLEVKVVPKP |
| O09126 | RLTWEHGEVGLVQFHKPGIFNYSALLMSEDKDTLYVGAREA |
| O09126 | EDKDTLYVGAREAVFAVNALNISEKQHEVYWKVSEDKKSKC |
| O09126 | SSTSLYVCGTNAFQPTCDHLNLTSFKFLGKSEDGKGRCPFD |
| O09126 | GELYSGTSYNFLGSEPIISRNSSHSPLRTEYAIPWLNEPSF |
| O09126 | NGPVPTPRPGACIDSEARAANYTSSLNLPDKTLQFVKDHPL |
| O09126 | LMDDSVTPIDNRPKLIKKDVNYTQIVVDRTQALDGTFYDVM |
| O09126 | ELKAASPKYGFVGRKHLLIFNLSDGDSGVYQCLSEERVRNK |
| O09126 | FNLSDGDSGVYQCLSEERVRNKTVSQLLAKHVLEVKMVPRT |
| O75326 | SSVWVGGRGKVYLFDFPEGKNASVRTVNIGSTKGSCLDKRD |
| O75326 | SEGLLACGTNARHPSCWNLVNGTVVPLGEMRGYAPFSPDEN |
| O75326 | DKIYYFFREDNPDKNPEAPLNVSRVAQLCRGDQGGESSLSV |
| O75326 | PDPSGQWRDTRVYGVFSNPWNYSAVCVYSLGDIDKVFRTSS |
| O75326 | NVEQSCEPGHQSPNCILFIENLTAQQYGHYFCEAQEGSYFR |
| P49908 | SLCKQPPAWSIRDQDPMLNSNGSVTVVALLQASUYLCILQA |
| P49908 | ILQASKLEDLRVKLKKEGYSNISYIVVNHQGISSRLKYTHL |
| P49908 | KYTHLKNKVSEHIPVYQQEENQTDVWTLLNGSKDDFLIYDR |
| P49908 | SEHIPVYQQEENQTDVWTLLNGSKDDFLIYDRCGRLVYHLG |
| P49908 | LTFPYVEEAIKIAYCEKKCGNCSLTTLKDEDFCKRVSLATV |
| P49908 | UQCKENLPSLCSUQGLRAEENITESCQURLPPAAUQISQQL |
| P25236 | LLQASRLEDLRIKLENQGYFNISYIVVNHQGSPSQLKHAHL |
| P25236 | LTFPYVEEAIKIAYCEKRCGNCSFTSLEDEAFCKNVSSATA |
| P25236 | CEKRCGNCSFTSLEDEAFCKNVSSATASKTTEPSEEHNHHK |
| Q12884 | SRVHNSEENTMRALTLKDILNGTFSYKTFFPNWISGQEYLH |
| Q12884 | ADNNIVLYNIETGQSYTILSNRTMKSVNASNYGLSPDRQFV |
| Q12884 | YNIETGQSYTILSNRTMKSVNASNYGLSPDRQFVYLESDYS |
| Q12884 | ATKYALWWSPNGKFLAYAEFNDTDIPVIAYSYYGDEQYPRT |
| Q12884 | SWLTWVTDERVCLQWLKRVQNVSVLSICDFREDWQTWDCPK |

| | |
|---|---|
| Q12884 | VYTERFMGLPTKDDNLEHYKNSTVMARAEYFRNVDYLLIHG |
| Q9NRX5 | AMTNEPETNCNPSLLSIIGYNTTSTVPKEGQSVQWWHAQGI |
| Q12874 | PNEICVPMSVEFEELLKARENPSEEAQNLVEFTDEEGYGRY |
| Q8N474 | EMLKCDKFPEGDVCIAMTPPNATEASKPQGTTVCPPCDNEL |
| P35248 | GPMGLSGLPGPRGPVGPKGENGSAGEPGPKGERGLVGPPGS |
| P02780 | CCYASGSGCSILDEVIRGTINSTVTLHDYMKLVKPYVQDHF |
| Q16585 | TGNNQPIVFQQGTTKLSVENNKTSITSDIGMQFFDPRTQNI |
| Q16585 | LPSGVKSLNVQKASTERITSNATSDLNIKVDGRAIVRGNEG |
| Q16585 | TIEFHMGGNMELKAENSIILNGSVMVSTTRLPSSSSGDQLG |
| O43556 | LGDFLGAVKNVWQPERLNAINITSALDRGGRVPLPINDLKE |
| P04278 | SFCLNGLWAQGQRLDVDQALNRSHEIWTHSCPQSPGNGTDA |
| P15196 | SFCLDGLWAQGQKLDMDKALNRSQDIWTHSCPSSPGNGTDT |
| Q15465 | REPRERLLLTAAHLLFVAPHNDSATGEPEASSGSGPPSGGA |
| P97797 | LKVTQPEKSVSVAAGDSTVLNCTLTSLLPVGPIKWYRGVGQ |
| P97797 | VGQSRLLIYSFTGEHFPRVTNVSDATKRNNMDFSIRISNVT |
| P97797 | KPSPPEVSGPADRGIPDQKVNFTCKSHGFSPRNITLKWFKD |
| P97797 | RGIPDQKVNFTCKSHGFSPRNITLKWFKDGQELHHLETTVN |
| P97797 | WFKDGQELHHLETTVNPSGKNVSYNISSTVRVVLNSMDVHS |
| P97797 | GQELHHLETTVNPSGKNVSYNISSTVRVVLNSMDVHSKVIC |
| P97797 | KVICEVAHITLDRSPLRGIANLSNFIRVSPTVKVTQQSPTS |
| P97797 | IRVSPTVKVTQQSPTSMNQVNLTCRAERFYPEDLQLIWLEN |
| P97797 | TCRAERFYPEDLQLIWLENGNVSRNDTPKNLTKNTDGTYNY |
| P97797 | EDLQLIWLENGNVSRNDTPKNLTKNTDGTYNYTSLFLVNSS |
| P97797 | GNVSRNDTPKNLTKNTDGTYNYTSLFLVNSSAHREDVVFTC |
| P97797 | PKNLTKNTDGTYNYTSLFLVNSSAHREDVVFTCQVKHDQQP |
| P97797 | REDVVFTCQVKHDQQPAITRNHTVLGLAHSSDQGSMQTFPG |
| P97797 | TVLGLAHSSDQGSMQTFPGNNATHNWNVFIGVGVACALLVV |
| P78324 | QVICEVAHVTLQGDPLRGTANLSETIRVPPTLEVTQQPVRA |
| P78324 | IRVPPTLEVTQQPVRAENQVNVTCQVRKFYPQRLQLTWLEN |
| P78324 | TCQVRKFYPQRLQLTWLENGNVSRTETASTVTENKDGTYNW |
| P78324 | ASTVTENKDGTYNWMSWLLVNVSAHRDDVKLTCQVEHDGQP |
| P04148 | RGQIPSQYEIPVFQFEIPYFNATYVDHNLITRNHDQCRVSE |
| P04148 | NVRTLKTVLTVDCPWLNFESNRTLAQHMSFKEDVVLSFYIN |
| P04148 | NRTLAQHMSFKEDVVLSFYINGSYPLIRLTTVFDKGNNFDL |
| Q9Y274 | EFDNIPCKKCVVVGNGGVLKNKTLGEKIDSYDVIIRMNNGP |
| Q9Y274 | TTFRLFYPESVFSDPIHNDPNTTVILTAFKPHDLRWLLELL |
| Q9Y274 | IAITLAFYICHEVHLAGFKYNFSDLKSPLHYYGNATMSLMN |
| Q9Y274 | HLAGFKYNFSDLKSPLHYYGNATMSLMNKNAYHNVTAEQLF |
| Q9Y274 | SPLHYYGNATMSLMNKNAYHNVTAEQLFLKDIIEKNLVINL |
| Q92187 | EHQETQLIGDGELSLSRSLVNSSDKIIRKAGSSIFQHNVEG |
| Q92187 | KIIRKAGSSIFQHNVEGWKINSSLVLEIRKNILRFLDAERD |

| | |
|---|---|
| Q92187 | KSSFKPGDVIHYVLDRRRTLNISHDLHSLLPEVSPMKNRRF |
| Q92187 | KSDFITMNPSVVQRAFGGFRNESDREKFVHRLSMLNDSVLW |
| Q92187 | FGGFRNESDREKFVHRLSMLNDSVLWIPAFMVKGGEKHVEW |
| Q92187 | LNGKAVKYHYYDDLKYRYFSNASPHRMPLEFKTLNVLHNRG |
| O43173 | VPITNSLTQELQEKPSKWKFNRTAFLHQRQEILQHVDVIKN |
| O43173 | NRTAFLHQRQEILQHVDVIKNFSLTKNSVRIGQLMHYDYSS |
| O43173 | ISNNFRSLLPDVSPIMNKHYNICAVVGNSGILTGSQCGQEI |
| O43173 | VFRCNFAPTEAFQRDVGRKTNLTTFNPSILEKYYNNLLTIQ |
| Q9HAT2 | DPMKPGGPFEVMAQQTLEKINFTLRVHDVLFGDVWLCSGQS |
| Q9HAT2 | GDVWLCSGQSNMQMTVLQIFNATRELSNTAAYQSVRILSVS |
| Q9HAT2 | SVTGPSKHSVLWNAMIHPLCNMTLKGVVWYQGESNINYNTD |
| Q9HAT2 | LKGVVWYQGESNINYNTDLYNCTFPALIEDWRETFHRGSQG |
| Q9HAT2 | KQTVAYRLHLGARALAYGEKNLTFEGPLPEKIELLAHKGLL |
| Q9HAT2 | LTFEGPLPEKIELLAHKGLLNLTYYQQIQVQKKDNKIFEIS |
| P15907 | PGPGIKFSAEALRCHLRDHVNVSMVEVTDFPFNTSEWEGYL |
| P15907 | RCHLRDHVNVSMVEVTDFPFNTSEWEGYLPKESIRTKAGPW |
| P13721 | PGPGVKFSVEALRCHLRDHVNVSMIEATDFPFNTTEWEGYL |
| P13721 | RCHLRDHVNVSMIEATDFPFNTTEWEGYLPKENFRTKVGPW |
| P13721 | KWYQKPDYNFFETYKSYRRLNPSQPFYILKPQMPWELWDII |
| Q96JF0 | DGDRLYSSMSRAFLYRLWKGNVSSKMLNPRLQKAMKDYLTA |
| Q9GZC8 | LVIGLTQNNSTTPSPIITSSNSSVLVFEISSKMKMIEKKLE |
| Q9GZC8 | TTKTLSNQKLTFVKPIGFFLNCSEQNISQFHVTLYSEDDIC |
| Q9GZC8 | SNQKLTFVKPIGFFLNCSEQNISQFHVTLYSEDDICANLIT |
| Q9GZC8 | FHVTLYSEDDICANLITVPANESIYDRSVISDKTHNRRVLS |
| Q9GZC8 | MFKSFRIFVFIAPDDSGCSTNTSRKSFNEKKKISFEFKKLE |
| Q9GZC8 | TSRKSFNEKKKISFEFKKLENQSYAVPTALMMIFLTTPCLL |
| Q8CIF6 | PLCVLLVASVESHLGALGPKNVSQKDAEFERTYADDVNSEL |
| Q8CIF6 | EFERTYADDVNSELVNIYTFNHTVTRNRTEGVRVSVNVLNK |
| Q8CIF6 | ADDVNSELVNIYTFNHTVTRNRTEGVRVSVNVLNKQKGAPL |
| Q8CIF6 | YQRKYLYQKVERTLCQPPTKNESEIQFFYVDVSTLSPVNTT |
| Q8CIF6 | TKNESEIQFFYVDVSTLSPVNTTYQLRVNRVDNFVLRTGEL |
| Q8CIF6 | QLRVNRVDNFVLRTGELFTFNTTAAQPQYFKYEFPDGVDSV |
| Q8CIF6 | IAVFYALPVVQLVITYQTVVNVTGNQDICYYNFLCAHPLGN |
| Q8CIF6 | NVTGNQDICYYNFLCAHPLGNLSAFNNILSNLGYILLGLLF |
| Q8CIF6 | AMGTALMMEGLLSACYHVCPNYTNFQFDTSFMYMIAGLCML |
| Q8CIF6 | MYMIAGLCMLKLYQKRHPDINASAYSAYACLAIVIFFSVLG |
| Q9Y286 | WAVQEETRDRFHLLGDPQTKNCTLSIRDARMSDAGRYFFRM |
| Q9Y286 | FFRMEKGNIKWNYKYDQLSVNVTALTHRPNILIPGTLESGC |
| Q9Y286 | ALTHRPNILIPGTLESGCFQNLTCSVPWACEQGTPPMISWM |
| Q9Y286 | QHHGTSLTCQVTLPGAGVTTNRTIQLNVSYPPQNLTVTVFQ |
| Q9Y286 | LTCQVTLPGAGVTTNRTIQLNVSYPPQNLTVTVFQGEGTAS |

| | |
|---|---|
| Q9Y286 | PGAGVTTNRTIQLNVSYPPQNLTVTVFQGEGTASTALGNSS |
| Q9Y286 | PQNLTVTVFQGEGTASTALGNSSSLSVLEGQSLRLVCAVDS |
| Q9Y286 | DEGEFTCRAQNSLGSQHVSLNLSLQQEYTGKMRPVSGVLLG |
| Q9H173 | ELNVVIETDMQIMVRLINKFNSSSSSLEEKIAALFDLEYYV |
| Q9H173 | MDNAQDLLSFGGLQVVINGLNSTEPLVKEYAAFVLGAAFSS |
| O00241 | QVICEIAHITLQGDPLRGTANLSEAIRVPPTLEVTQQPMRA |
| O00241 | IRVPPTLEVTQQPMRAENQANVTCQVSNFYPRGLQLTWLEN |
| O00241 | TCQVSNFYPRGLQLTWLENGNVSRTETASTLIENKDGTYNW |
| Q9P1W8 | QVICEVAHVTLQGDPLRGTANLSEAIRVPPTLEVTQQPMRV |
| Q9P1W8 | IRVPPTLEVTQQPMRVGNQVNVTCQVRKFYPQSLQLTWSEN |
| Q9P1W8 | GNVCQRETASTLTENKDGTYNWTSWFLVNISDQRDDVVLTC |
| Q9P1W8 | ASTLTENKDGTYNWTSWFLVNISDQRDDVVLTCQVKHDGQL |
| Q9Y3P8 | PRLRAVCLWTLTSAAMSRGDNCTDLLALGIPSITQAWGLWV |
| Q03567 | RFALSLVMFFGCLVTYMMRTNMSFAVVCMVNENKTDTGVEK |
| Q03567 | LVTYMMRTNMSFAVVCMVNENKTDTGVEKVSRCGKEMTPVE |
| Q03567 | TDTGVEKVSRCGKEMTPVESNSSVIGEFDWDKQTTGMVLSS |
| Q03567 | WGAYTMLVSLPSFLKDVLGLNLSSLGAVASIPYIAYFLAIN |
| Q03567 | NTISALAGIISPAVSSYLTPNGTQEEWQMVLWLTAGILTIG |
| P19634 | RSIGDVTTAPPEVTPESRPVNHSVTDHGMKPRKAFPVLGID |
| Q92581 | YGLLVGLVLRYGIHVPSDVNNVTLSCEVQSSPTTLLVTFDP |
| Q8IVB4 | ESGTVYDCVKLTFSPSTLLVNITDQVYEYKYKREISQHNIN |
| Q96DU3 | VTLPLEFPAGEKVNFITWLFNETSLAFIVPHETKSPEIHVT |
| Q96DU3 | PHETKSPEIHVTNPKQGKRLNFTQSYSLQLSNLKMEDTGSY |
| Q96DU3 | AKLSSYTLRILRQLRNIQVTNHSQLFQNMTCELHLTCSVED |
| Q96DU3 | LRILRQLRNIQVTNHSQLFQNMTCELHLTCSVEDADDNVSF |
| Q96DU3 | LFQNMTCELHLTCSVEDADDNVSFRWEALGNTLSSQPNLTV |
| Q96DU3 | LTCSVEDADDNVSFRWEALGNTLSSQPNLTVSWDPRISSEQ |
| Q96DU3 | ADDNVSFRWEALGNTLSSQPNLTVSWDPRISSEQDYTCIAE |
| Q96DU3 | WDPRISSEQDYTCIAENAVSNLSFSVSAQKLCEDVKIQYTD |
| Q7T2Q0 | CPLGWSSFDQHCYKVFEPVKNWTEAEEICMQQHKGSRLASI |
| O94813 | LRSVPRNIPRNTERLDLNGNNITRITKTDFAGLRHLRVLQL |
| O94813 | IEDGAFRALRDLEVLTLNNNNITRLSVASFNHMPKLRTFRL |
| O94813 | EFTVLEATGIFKKLPQLRKINFSNNKITDIEEGAFEGASGV |
| O94813 | KGLESLKTLMLRSNRITCVGNDSFIGLSSVRLLSLYDNQIT |
| O94813 | SNYKHLTLIDLSNNRISTLSNQSFSNMTQLLTLILSYNRLR |
| O94813 | LTLIDLSNNRISTLSNQSFSNMTQLLTLILSYNRLRCIPPR |
| O94813 | GFEGENCEVNVDDCEDNDCENNSTCVDGINNYTCLCPPEYT |
| O94813 | FEGENCEVNVDDCEDNDCENNSTCVDGINNYTCLCPPEYTG |
| O94813 | VDDCEDNDCENNSTCVDGINNYTCLCPPEYTGELCEEKLDF |
| O94813 | NFINKESYLQIPSAKVRPQTNITLQIATDEDSGILLYKGDK |
| O94813 | LALDQSLSLSVDGGNPKIITNLSKQSTLNFDSPLYVGGMPG |

| | |
|---|---|
| O94813 | YVGGMPGKSNVASLRQAPGQNGTSFHGCIRNLYINSELQDF |
| Q4PRD1 | CPSGWLSYEQHCYKGFNDLKNWTDAEKFCTEQKKGSHLVSL |
| Q4PRD2 | GHLVSIESMEEAEFVAKLLSNTTGKFITHFWIGLMIKDKEQ |
| P23126 | LVNSEENSNSIITDEDYDHYNSSLDSSNNVKHSQEAFHRNS |
| P23126 | SQEAFHRNSDPDGFPEYEFLNETSIEIKEELGQELHQLQLI |
| Q9Y6L6 | LTALPHFFMGYYRYSKETNINSSENSTSTLSTCLINQILSL |
| Q9Y6L6 | PHFFMGYYRYSKETNINSSENSTSTLSTCLINQILSLNRAS |
| Q9Y6L6 | CFTAVMSLSFYLLYFFILCENKSVAGLTMTYDGNNPVTSHR |
| Q9Y6L6 | SPCLAGCKSSSGNKKPIVFYNCSCLEVTGLQNRNYSAHLGE |
| Q9Y6L6 | KKPIVFYNCSCLEVTGLQNRNYSAHLGECPRDDACTRKFYF |
| Q9Y6L6 | TTCIKWSTNNCGTRGSCRTYNSTSFSRVYLGLSSMLRVSSL |
| P46720 | SLPHFFMGRYEYETTISPTGNLSSNSFLCMENRTQTLKPTQ |
| P46720 | YETTISPTGNLSSNSFLCMENRTQTLKPTQDPAECVKEMKS |
| P46720 | FVGTGTNMVFQDCSCIQSLGNSSAVLGLCKKGPECANRLQY |
| P83684 | AVAVLRGDSKITGTVTFEQANESAPTTVSWNITGHDPNAER |
| P34461 | IPTELIGTIDFDQSGSFLKLNGSVSGLAAGKHGFHIHEKGD |
| P08294 | LAPRAKLDAFFALEGFPTEPNSSSRAIHVHQFGDLSQGCES |
| P21919 | PLVYLQTTLDRYDDVPDVLLNKTKWMSEKLISLEQGVVVLI |
| Q8WY21 | TFALTGDSAHNQAMVHWSGHNSSVILILTKLYDYNLGSITE |
| Q8WY21 | VHLEARTVDGHSHYLTCRMQNCTEANRNQPFPGYIDPDSLI |
| Q8WY21 | MHVISTDENQVFAAVQEWNQNDTYNLYISDTRGVYFTLALE |
| Q8WY21 | AFWFNPSSLSKDCSLGQSYLNSTGYRKVVSNNCTDGVREQY |
| Q8WY21 | DCSLGQSYLNSTGYRKVVSNNCTDGVREQYTAKPQKCPGKA |
| Q8WY21 | APRGLRIVTADGKLTAEQGHNVTLMVQLEEGDVQRTLIQVD |
| Q8WY21 | DVQRTLIQVDFGDGIAVSYVNLSSMEDGIKHVYQNVGIFRV |
| Q8WY21 | CPLEHVHLSLPFVTTKNKEVNATAVLWPSQVGTLTYVWWYG |
| Q8WY21 | ATAVLWPSQVGTLTYVWWYGNNTEPLITLEGSISFRFTSEG |
| Q9EPR5 | SFVLKGDATHNQAMVHWTGENSSVILILTKYYHADMGKVLE |
| Q9EPR5 | VHVEAQDLSGGYRYYTCLIYNCSAQPHIAPFSGPIDRGSLT |
| Q9EPR5 | IDRGSLTVQDEYIFLKATSTNRTKYYVSYRRSDFVLMKLPK |
| Q9EPR5 | SIPLKILKFSVDEGHTWSTHNFTSTSVFVDGLLSEPGDETL |
| Q9EPR5 | DVLTTKYQVDLGDGFKAMYVNLTLTGEPIRHHYESPGIYRV |
| Q9EPR5 | SPLQALYLEVVPVIGVNQEVNLTAVLLPLNPNLTVFYWWIG |
| Q9EPR5 | PVIGVNQEVNLTAVLLPLNPNLTVFYWWIGHSLQPLLSLDN |
| Q92673 | RKRSAALQPEPIKVYGQVSLNDSHNQMVVHWAGEKSNVIVA |
| Q92673 | SYDYGKSFKKISDKLNFGLGNRSEAVIAQFYHSPADNKRYI |
| Q92673 | EYYIADASEDQVFVCVSHSNNRTNLYISEAEGLKFSLSLEN |
| Q92673 | PFADFHRVEGLQGVYIATLINGSMNEENMRSVITFDKGGTW |
| Q92673 | VFTIFGSNKENVHSWLILQVNATDALGVPCTENDYKLWSPS |
| Q92673 | RTPHATCFNGEDFDRPVVVSNCSCTREDYECDFGFKMSEDL |
| Q92673 | EHNCLYWSDLALDVIQRLCLNGSTGQEVIINSGLETVEALA |

| | |
|---|---|
| Q92673 | AGFKKIEVANPDGDFRLTIVNSSVLDRPRALVLVPQEGVMF |
| Q92673 | NTGSNACVPRPCSLLCLPKANNSRSCRCPEDVSSSVLPSGD |
| Q92673 | SSVLPSGDLMCDCPQGYQLKNNTCVKQENTCLRNQYRCSNG |
| Q92673 | CGDNSDESHCEMHQCRSDEYNCSSGMCIRSSWVCDGDNDCR |
| Q92673 | IRSSWVCDGDNDCRDWSDEANCTAIYHTCEASNFQCRNGHC |
| Q92673 | DGSDEDPVNCEKKCNGFRCPNGTCIPSSKHCDGLRDCSDGS |
| Q92673 | MDDCGDYSDEANCENPTEAPNCSRYFQFRCENGHCIPNRWK |
| Q92673 | CDGYRDCADGSDEEACPLLANVTAASTPTQLGRCDRFEFEC |
| Q92673 | YNVYYRVVGESIWKTLETHSNKTNTVLKVLKPDTTYQVKVQ |
| Q92673 | YIVEYSRSGSKMWASQRAASNFTEIKNLLVNTLYTVRVAAV |
| Q92673 | LLVNTLYTVRVAAVTSRGIGNWSDSKSITTIKGKVIPPPDI |
| Q92673 | HKQERRTLNFRGSILSHKVGNLTAHTSYEISAWAKTDLGDS |
| Q92673 | EHVMTRGVRPPAPSLKAKAINQTAVECTWTGPRNVVYGIFY |
| Q92673 | YATSFLDLYRNPKSLTTSLHNKTVIVSKDEQYLFLVRVVVP |
| Q92673 | AVAVKDLIRKTDRSYKVKSRNSTVEYTLNKLEPGGKYHIIV |
| Q92673 | EYTLNKLEPGGKYHIIVQLGNMSKDSSIKITTVSLSAPDAL |
| Q92673 | TENDHVLLFWKSLALKEKHFNESRGYEIHMFDSAMNITAYL |
| Q92673 | KEKHFNESRGYEIHMFDSAMNITAYLGNTTDNFFKISNLKM |
| Q92673 | SRGYEIHMFDSAMNITAYLGNTTDNFFKISNLKMGHNYTFT |
| Q92673 | AYLGNTTDNFFKISNLKMGHNYTFTVQARCLFGNQICGEPA |
| Q99523 | SAPGEDEECGRVRDFVAKLANNTHQHVFDDLRGSVSLSWVG |
| Q99523 | SKLYRSEDYGKNFKDITDLINNTFIRTEFGMAIGPENSGKV |
| Q99523 | KLYRSEDYGKNFKDITDLINNTFIRTEFGMAIGPENSGKVV |
| Q99523 | KAVCLAKWGSDNTIFFTTYANGSCKADLGALELWRTSDLGK |
| Q99523 | YSKSLDRHLYTTTGGETDFTNVTSLRGVYITSVLSEDNSIQ |
| Q99523 | FTRDPIYFTGLASEPGARSMNISIWGFTESFLTSQWVSYTI |
| Q99523 | SICLCSLEDFLCDFGYYRPENDSKCVEQPELKGHDLEFCLY |
| Q80X76 | VFGCPDVTLGRNTAVREVQENITSVDSLTLASSNTDFAFSL |
| Q80X76 | LLSLGAKSNTLKEILEGLKFNLTETPEPDIHQGFRYLLDLL |
| Q80X76 | FTADFQQPLEATKLINDYVSNHTQGKIKELISDLDKRTLMV |
| Q80X76 | TPYFRDEELSCTVVELKYTGNASAMFILPDQGKMQQVEASL |
| P07759 | VLCFPDGTKEMDIVFHEHQDNGTQDDSLTLASVNTDFAFSL |
| P07759 | LVSLGAKGKTMEEILEGLKFNLTETPEADIHQGFGNLLQSL |
| P07759 | FTADFQQPTEAKNLINDYVSNQTQGMIKELISELDERTLMV |
| P07759 | TRHFRDEELSCSVLELKYTGNASALLILPDQGRMQQVEASL |
| Q03734 | LVSLGAKGNTLEEILEGLKFNLTETSEADIHQGFGHLLQRL |
| Q03734 | FTADFQQPTEATKLINDYVSNQTQGMIKKLISELDDRTLMV |
| Q03734 | TRHFRDEELSCSVLELKYTGNASALFILPDQGRMQQVEASL |
| P08723 | IRIFLTVIDLIKGIQLRFGGNWSDVYGSRSLKYKEFLLEDG |
| P34525 | IGVKSKYYFLDDGTNLLNHKNVTFVLRYNVIPNSGYLRLVQ |
| P28687 | KDMKSKYFFFDDGNGLKGNRNVTLTLSWNVVPNAGLLPLVT |

| | |
|---|---|
| P61008 | KDMKTKYFFFDDGNGLKGNRNVTLTLSWNVVPNAGILPLVT |
| P61009 | KDMKTKYFFFDDGNGLKGNRNVTLTLSWNVVPNAGILPLVT |
| P51688 | RPRNALLLLADDGGFESGAYNNSAIATPHLDALARRSLLFR |
| P51688 | GKKHVGPETVYPFDFAYTEENGSVLQVGRNITRIKLLVRKF |
| P51688 | VYPFDFAYTEENGSVLQVGRNITRIKLLVRKFLQTQDDRPF |
| P51688 | GRMDQGVGLVLQELRDAGVLNDTLVIFTSDNGIPFPSGRTN |
| P51688 | KMPFPIDQDFYVSPTFQDLLNRTTAGQPTGWYKDLRHYYYR |
| Q8CEK3 | PYSVFSVNIFAGPENVIKEPNCTMYKSKSECSNIAENPVCA |
| Q9R097 | EGTYLFQLTRTDSDQPEETANLTITVLTAKQTEDYCLASYK |
| O43291 | FCLVSKVVGRCRASMPRWWYNVTDGSCQLFVYGGCDGNSNN |
| O43291 | NSNNYLTKEECLKKCATVTENATGDLATSRNAADSSVPSAP |
| Q01083 | QAPVTSSTTMRTTTTTTPRPNITFPTYKCPETFDAWYCLND |
| Q9HCB6 | KPILDCCACGTAKYRLTFYGNWSEKTHPKDYPRRANHWSAI |
| Q9HCB6 | MLPECPIDCELTEWSQWSECNKSCGKGHVIRTRMIQMEPQF |
| Q8TCT8 | TKDYCMLYNPYWTALPSTLENATSISLMNLTSTPLCNLSDI |
| Q8TCT8 | NPYWTALPSTLENATSISLMNLTSTPLCNLSDIPPVGIKSK |
| Q8TCT8 | STLENATSISLMNLTSTPLCNLSDIPPVGIKSKAVVVPWGS |
| Q8TCT8 | HFLEKARIAQKGGAEAMLVVNNSVLFPPSGNRSEFPDVKIL |
| Q8TCT8 | KGGAEAMLVVNNSVLFPPSGNRSEFPDVKILIAFISYKDFR |
| Q8TCT8 | EFPDVKILIAFISYKDFRDMNQTLGDNITVKMYSPSWPNFD |
| Q8TCT8 | ILIAFISYKDFRDMNQTLGDNITVKMYSPSWPNFDYTMVVI |
| P09486 | QDPTSCPAPIGEFEKVCSNDNKTFDSSCHFFATKCTLEGTK |
| Q14515 | TDSNQQESITKREENQEQPRNYSHHQLNRSSKHSQGLRDQG |
| Q14515 | SITKREENQEQPRNYSHHQLNRSSKHSQGLRDQGNQEQDPN |
| Q14515 | NRSSKHSQGLRDQGNQEQDPNISNGEEEEEKEPGEVGTHND |
| Q14515 | EFDQGNQEQEDNSNAEMEEENASNVNKHIQETEWQSQEGKT |
| Q14515 | ENENIGTTEPGEHQEAKKAENSSNEEETSSEGNMRVHAVDS |
| Q14515 | CQDPVTCPPTKPLDQVCGTDNQTYASSCHLFATKCRLEGTK |
| P43307 | IVESLDASFRYPQDYQFYIQNFTALPLNTVVPPQRQATFEY |
| P43307 | LVINLNYKDLNGNVFQDAVFNQTVTVIEREDGLDGETIFMY |
| Q9CY50 | IVESLDASFRYPQDYQFYIQNFTALPLNTVVPPQRQATFEY |
| Q9CY50 | SFRYPQDYQFYIQNFTALPLNTVVPPQRQATFEYSFIPAEP |
| Q9CY50 | LVINLNYKDLNGNVFQDAVFNQTVTVIEREDGLDGETIFMY |
| P43308 | DFGIVSGMLNVKWDRIAPASNVSHTVVLRPLKAGYFNFTSA |
| P43308 | APASNVSHTVVLRPLKAGYFNFTSATITYLAQEDGPVVIGS |
| Q22169 | SFDIVKGLLFVHFEQIPAGSNVTHSVVIRPRAFGFFNYTAA |
| Q22169 | PAGSNVTHSVVIRPRAFGFFNYTAAQVTYYTDNENHHVTLT |
| Q9CPW5 | DFGIVSGMLNVKWDRIAPASNVSHTVVLRPLKAGYFNFTSA |
| Q9CPW5 | APASNVSHTVVLRPLKAGYFNFTSATITYLAQEDGPVVIGS |
| P56677 | QKVFNGHLRITNEIFLDAYENSTSTEFISLASQVKEALKLL |
| P56677 | LSPMEPHAVVRLCGTFSPSYNLTFLSSQNVFLVTLITNTDR |

| | |
|---|---|
| P56677 | SDTQGTFSSPYYPGHYPPNINCTWNIKVPNNRNVKVRFKLF |
| P56677 | YVEINGEKYCGERSQFVVSSNSSKITVHFHSDHSYTDTGFL |
| P56677 | LRCDGWADCPDYSDERYCRCNATHQFTCKNQFCKPLFWVCD |
| P56677 | HTKEGGTGALILQKGEIRVINQTTCEDLMPQQITPRMMCVG |
| Q9NY15 | PGGAETPCNGHGTCLDGMDRNGTCVCQENFRGSACQECQDP |
| Q9NY15 | DGMVCLPKDPCTDNLGGCPSNSTLCVYQKPGQAFCTCRPGL |
| Q9NY15 | YQKPGQAFCTCRPGLVSINSNASAGCFAFCSPFSCDRSATC |
| Q9NY15 | TAGPFTVLVPSVSSFSSRTMNASLAQQLCRQHIIAGQHILE |
| Q9NY15 | VEKLISKGRILTMANQVLAVNISEEGRILLGPEGVPLQRVD |
| Q9NY15 | HCSEEQHKIVAGSCVDCQALNTSTCPPNSVKLDIFPKECVY |
| Q9NY15 | VYIHDPTGLNVLKKGCASYCNQTIMEQGCCKGFFGPDCTQC |
| Q9NY15 | FGPDCTQCPGGFSNPCYGKGNCSDGIQGNGACLCFPDYKGI |
| Q9NY15 | GSGGVCQQGTCAPGFSGRFCNESMGDCGPTGLAQHCHLHAR |
| Q9NY15 | ARLGGQEVATLNPTTRWEIRNISGRVWVQNASVDVADLLAT |
| Q9NY15 | TLNPTTRWEIRNISGRVWVQNASVDVADLLATNGVLHILSQ |
| Q9NY15 | HHGLVPQIEAATAYTIFVPTNRSLEAQGNSSHLDADTVRHH |
| Q9NY15 | EAATAYTIFVPTNRSLEAQGNSSHLDADTVRHHVVLGEALS |
| Q9NY15 | LRKGGHRNSLLGPAHWIVFYNHSGQPEVNHVPLEGPMLEAP |
| Q9NY15 | TVGSSRCLHSHAEALREKCVNCTRRFRCTQGFQLQDTPRKS |
| Q9NY15 | CHEGFHGTACEVCELGRYGPNCTGVCDCAHGLCQEGLQGDG |
| Q9NY15 | IFCSEVDPCAHGHGGCSPHANCTKVAPGQRTCTCQDGYMGD |
| Q9NY15 | KELKGDGPFTIFVPHADLMSNLSQDELARIRAHRQLVFRYH |
| Q9NY15 | VLLPPEALHWEPDDAPIPRRNVTAAAQGFGYKIFSGLLKVA |
| Q9NY15 | RVCTVADLCQDGHGGCSEHANCSQVGTMVTCTCLPDYEGDG |
| Q9NY15 | FQEKRAGVFHLQATSGPYGLNFSEAEAACEAQGAVLASFPQ |
| Q9NY15 | PQLSAAQQLGFHLCLMGWLANGSTAHPVVFPVADCGNGRVG |
| Q9NY15 | FPVADCGNGRVGIVSLGARKNLSERWDAYCFRVQDVACRCR |
| Q9NY15 | VGDGISTCNGKLLDVLAATANFSTFYGMLLGYANATQRGLD |
| Q9NY15 | DVLAATANFSTFYGMLLGYANATQRGLDFLDFLDDELTYKT |
| Q9NY15 | LDDELTYKTLFVPVNEGFVDNMTLSGPDLELHASNATLLSA |
| Q9NY15 | NEGFVDNMTLSGPDLELHASNATLLSANASQGKLLPAHSGL |
| Q9NY15 | MTLSGPDLELHASNATLLSANASQGKLLPAHSGLSLIISDA |
| Q9NY15 | GKLLPAHSGLSLIISDAGPDNSSWAPVAPGTVVVSRIIVWD |
| Q8WWQ8 | PGGAGSPCNGRGSCAEGMEGNGTCSCQEGFGGTACETCADD |
| Q8WWQ8 | GVGCSMTDICKSDNPCHRNANCTTVAPGRTECICQKGYVGD |
| Q8WWQ8 | AAQYFVKLHIIAGQMNIEYMNNTDMFYTLTGKSGEIFNSDK |
| Q8WWQ8 | VATLISTPHIRSMANQLIQFNTTDNGQILANDVAMEEIEIT |
| Q8WWQ8 | HRCVYSGRFGSLKSGCARYCNATVKIPKCCKGFYGPDCNQC |
| Q8WWQ8 | PGGFSNPCSGNGQCADSLGGNGTCICEEGFQGSQCQFCSDP |
| Q8WWQ8 | TSACGPYVQFCHIHATCEYSNGTASCICKAGYEGDGTLCSE |
| Q8WWQ8 | GRDCSEINNCLLPSAGGCHDNASCLYVGPGQNECECKKGFR |

| | |
|---|---|
| Q8WWQ8 | NAAVELSFLSEAAIFNRWINNASLQPTLSATSNLTVLVPSQ |
| Q8WWQ8 | AIFNRWINNASLQPTLSATSNLTVLVPSQQATEDMDQDEKS |
| Q8WWQ8 | SDMLATSLQGNFLHLAKVDGNITIEGASIVDGDNAATNGVI |
| Q8WWQ8 | SYFLSFFLHNDQLYVNEAPINYTNVATDKGVIHGLGKVLEI |
| Q8WWQ8 | KGVIHGLGKVLEIQKNRCDNNDTTIIRGRCRTCSSELTCPF |
| Q8WWQ8 | PCPGNAQNVCFGNGICLDGVNGTGVCECGEGFSGTACETCT |
| Q8WWQ8 | GPLGDGSCDCDVGWRGVHCDNATTEDNCNGTCHTSANCLTN |
| Q8WWQ8 | DCDVGWRGVHCDNATTEDNCNGTCHTSANCLTNSDGTASCK |
| Q8WWQ8 | NCLTNSDGTASCKCAAGFQGNGTICTAINACEISNGGCSAK |
| Q8WWQ8 | CTLINVCLTKNGGCSEFAICNHTGQVERTCTCKPNYIGDGF |
| Q8WWQ8 | LRYHVVACHQLLLENLKLISNATSLQGEPIVISVSQSTVYI |
| Q8WWQ8 | LLSPKNLLITPKDNSGRILQNLTTLATNNGYIKFSNLIQDS |
| Q8WWQ8 | RGVCLDQYSATGECKCNTGFNGTACEMCWPGRFGPDCLPCG |
| Q8WWQ8 | QAVLPAVCTPPCSAHATCKENNTCECNLDYEGDGITCTVVD |
| Q8WWQ8 | FASQNCGSGVVGIVDYGPRPNKSEMWDVFCYRMKDVNCTCK |
| Q8WWQ8 | GPRPNKSEMWDVFCYRMKDVNCTCKVGYVGDGFSCSGNLLQ |
| Q8WWQ8 | QVLMSFPSLTNFLTEVLAYSNSSARGRAFLEHLTDLSIRGT |
| Q8WWQ8 | LTDLSIRGTLFVPQNSGLGENETLSGRDIEHHLANVSMFFY |
| Q8WWQ8 | NSGLGENETLSGRDIEHHLANVSMFFYNDLVNGTTLQTRLG |
| Q8WWQ8 | GRDIEHHLANVSMFFYNDLVNGTTLQTRLGSKLLITASQDP |
| Q08264 | VARCLNGALAVGCGTFACLENSTCDTDGMHDICQLFFHTAA |
| P43647 | VARCLNGALDVGCGTFACLENSTCDTDGMHDICQLFFHTAA |
| P43648 | VARCLNGALAVGCGTFACLENSTCDTDGMHDICQLFFHTAA |
| D6VTK4 | APSLSNLFYDPTYNPGQSTINYTSIYGNGSTITFDELQGLV |
| D6VTK4 | FYDPTYNPGQSTINYTSIYGNGSTITFDELQGLVNSTVTQA |
| Q687X5 | LHVLYTLVIPIRYYVRWRLGNLTVTQAILKKENPFSTSSAW |
| P00302 | SAGWKVPFFGDVDYDWKWASNKTFHIGDVLVFKYDRRFHNV |
| P00302 | KYDRRFHNVDKVTQKNYQSCNDTTPIASYNTGBBRINLKTV |
| Q13586 | EDKLISVEDLWKAWKSSEVYNWTVDEVVQWLITYVELPQYE |
| Q13586 | EETFRKLQLSGHAMPRLAVTNTTMTGTVLKMTDRSHRQKLQ |
| P08842 | PNIILVMADDLGIGDPGCYGNKTIRTPNIDRLASGGVKLTQ |
| P08842 | PLNCFMMRNYEIIQQPMSYDNLTQRLTVEAAQFIQRNTETP |
| P46978 | PEDAKVMSWWDYGYQITAMANRTILVDNNTWNNTHISRVGQ |
| P46978 | SWWDYGYQITAMANRTILVDNNTWNNTHISRVGQAMASTEE |
| P46978 | YGYQITAMANRTILVDNNTWNNTHISRVGQAMASTEEKAYE |
| P46977 | PEDAKVMSWWDYGYQITAMANRTILVDNNTWNNTHISRVGQ |
| P46977 | SWWDYGYQITAMANRTILVDNNTWNNTHISRVGQAMASTEE |
| P46977 | YGYQITAMANRTILVDNNTWNNTHISRVGQAMASTEEKAYE |
| Q3TDQ1 | YGYQIAGMANRTTLVDNNTWNNSHIALVGKAMSSNETAAYK |
| Q3TDQ1 | VDNNTWNNSHIALVGKAMSSNETAAYKIMRSLDVDYVLVIF |
| Q8TCJ2 | TVYPGLMITAGLIHWILNTLNITVHIRDVCVFLAPTFSGLT |

| | |
|---|---|
| Q8TCJ2 | DEHARVMSWWDYGYQIAGMANRTTLVDNNTWNNSHIALVGK |
| Q8TCJ2 | SWWDYGYQIAGMANRTTLVDNNTWNNSHIALVGKAMSSNET |
| Q8TCJ2 | YGYQIAGMANRTTLVDNNTWNNSHIALVGKAMSSNETAAYK |
| Q8TCJ2 | VDNNTWNNSHIALVGKAMSSNETAAYKIMRTLDVDYVLVIF |
| P46975 | ADDARVMSWWDYGYQIAGMANRTTLVDNNTWNNSHIALVGK |
| P46975 | SWWDYGYQIAGMANRTTLVDNNTWNNSHIALVGKAMSSNES |
| P46975 | YGYQIAGMANRTTLVDNNTWNNSHIALVGKAMSSNESAAYE |
| P46975 | VDNNTWNNSHIALVGKAMSSNESAAYEIMTELDVDYILVIF |
| P14410 | PEQFPTEGICAQRGCCWRPWNDSLIPWCFFVDNHGYNVQDM |
| P14410 | DHGQKYVIILDPAISIGRRANGTTYATYERGNTQHVWINES |
| P14410 | RANGTTYATYERGNTQHVWINESDGSTPIIGEVWPGLTVYP |
| P14410 | DVTTTASRKNPLGLIVALGENNTAKGDFFWDDGETKDTIQN |
| P14410 | GETKDTIQNGNYILYTFSVSNNTLDIVCTHSSYQEGTTLAF |
| P14410 | TDSVTEVRVAENNQPMNAHSNFTYDASNQVLLIADLKLNLG |
| P14410 | TYDASNQVLLIADLKLNLGRNFSVQWNQIFSENERFNCYPD |
| P14410 | HPVMPAYWALGFQLCRYGYANTSEVRELYDAMVAANIPYDV |
| P14410 | DKIRGEGMRYIIILDPAISGNETKTYPAFERGQQNDVFVKW |
| P14410 | FVKWPNTNDICWAKVWPDLPNITIDKTLTEDEAVNASRAHV |
| P14410 | VWPDLPNITIDKTLTEDEAVNASRAHVAFPDFFRTSTAEWW |
| P14410 | NEKMKFDGLWIDMNEPSSFVNGTTTNQCRNDELNYPPYFPE |
| P14410 | MMEFSLFGMSYTGADICGFFNNSEYHLCTRWMQLGAFYPYS |
| P14410 | YPYSRNHNIANTRRQDPASWNETFAEMSRNILNIRYTLLPY |
| P14410 | RWFDYHTGKDIGVRGQFQTFNASYDTINLHVRGGHILPCQE |
| P14410 | DGESIDTYERDLYLSVQFNLNQTTLTSTILKRGYINKSETR |
| P14410 | VQFNLNQTTLTSTILKRGYINKSETRLGSLHVWGKGTTPVN |
| Q9VEX0 | RSSLLTGMYVHNHMVFTNNDNCSSPQWQATHETRSYATYLS |
| Q9VEX0 | TYLSNAGYRTGYFGKYLNKYNGSYIPPGWREWGGLIMNSKY |
| Q9VEX0 | SYIPPGWREWGGLIMNSKYYNYSINLNGQKIKHGFDYAKDY |
| Q9VEX0 | GQKIKHGFDYAKDYYPDLIANDSIAFLRSSKQQNQRKPVLL |
| Q9VEX0 | SFPAPHGPEDSAPQYSHLFFNVTTHHTPSYDHAPNPDKQWI |
| Q9VEX0 | EQIAESRARLQIERRNMKLANSSLLEDFLEGAGESTTIVSS |
| Q9VEX0 | VVDQLVDHTHRSKRDLPASSNETIAQVIQQIQSTLEILELK |
| Q9VEX0 | IQSTLEILELKFNEHELHASNSSGNSYERGEKYTKSGGHRC |
| Q9VEX0 | EKYTKSGGHRCFVDATTAKVNCSNVIYDDEKTWRTSRTQID |
| Q9VEX0 | NDNRRRNDQSVLDSGAGPEFNMSYFTEISSTPRSNVVGQTE |
| Q9VEX0 | HWRTAPLWNDSPFCFCMNANNNTYSCLRTINGTHNFLYCEF |
| Q9VEX0 | SPFCFCMNANNNTYSCLRTINGTHNFLYCEFTTGLITFYNL |
| Q9VEX0 | INGTHNFLYCEFTTGLITFYNLTIDRFETINRAAGLTPGER |
| Q8IWU6 | NIILVLTDDQDVELGSLQVMNKTRKIMEHGGATFINAFVTT |
| Q8IWU6 | RSSMLTGKYVHNHNVYTNNENCSSPSWQAMHEPRTFAVYLN |
| Q8IWU6 | NCSSPSWQAMHEPRTFAVYLNNTGYRTAFFGKYLNEYNGSY |

| | |
|---|---|
| Q8IWU6 | VYLNNTGYRTAFFGKYLNEYNGSYIPPGWREWLGLIKNSRF |
| Q8IWU6 | SYIPPGWREWLGLIKNSRFYNYTVCRNGIKEKHGFDYAKDY |
| Q8IWU6 | GIKEKHGFDYAKDYFTDLITNESINYFKMSKRMYPHRPVMM |
| Q8IWU6 | SHAAPHGPEDSAPQFSKLYPNASQHITPSYNYAPNMDKHWI |
| Q8IWU6 | SNAVGPPTTVRVTHKCFILPNDSIHCERELYQSARAWKDHK |
| Q8IWU6 | HWQTAPFWNLGSFCACTSSNNNTYWCLRTVNETHNFLFCEF |
| Q8IWU6 | GSFCACTSSNNNTYWCLRTVNETHNFLFCEFATGFLEYFDM |
| Q8IWU5 | NIILVLTDDQDVELGSMQVMNKTRRIMEQGGAHFINAFVTT |
| Q8IWU5 | RSSILTGKYVHNHNTYTNNENCSSPSWQAQHESRTFAVYLN |
| Q8IWU5 | NCSSPSWQAQHESRTFAVYLNSTGYRTAFFGKYLNEYNGSY |
| Q8IWU5 | VYLNSTGYRTAFFGKYLNEYNGSYVPPGWKEWVGLLKNSRF |
| Q8IWU5 | SYVPPGWKEWVGLLKNSRFYNYTLCRNGVKEKHGSDYSKDY |
| Q8IWU5 | GVKEKHGSDYSKDYLTDLITNDSVSFFRTSKKMYPHRPVLM |
| Q8IWU5 | SHAAPHGPEDSAPQYSRLFPNASQHITPSYNYAPNPDKHWI |
| Q8IWU5 | AIEVDGRVYHVGLGDAAQPRNLTKRHWPGAPEDQDDKDGGD |
| Q8IWU5 | LPDYSAANPIKVTHRCYILENDTVQCDLDLYKSLQAWKDHK |
| Q8IWU5 | LLREQKRKKKLRKLLKRLQNNDTCSMPGLTCFTHDNQHWQT |
| Q8IWU5 | HWQTAPFWTLGPFCACTSANNNTYWCMRTINETHNFLFCEF |
| Q8IWU5 | GPFCACTSANNNTYWCMRTINETHNFLFCEFATGFLEYFDL |
| Q8NBK3 | IDAFYMDAYEVSNTEFEKFVNSTGYLTEAEKFGDSFVFEGM |
| Q8NBJ7 | FAARGGLKGQVYPWGNWFQPNRTNLWQGKFPKGDKAEDGFH |
| Q9UH99 | SARIRPTAVTLEHVPKALSPNSTISSAPKDFAIFGFDEDLQ |
| Q8BJS4 | SARIRPTAVTLEHVPKALSPNSTISSAPKDFAIFGFDEDLQ |
| Q17374 | KTESVIEERMEYGRMILLVCNKTCAKHRSDIPLWLKEFNQK |
| Q17374 | PETITYYYHTYRQAVSFIDTNETDVFPNLIYFIGVKRVVFN |
| Q17374 | LILLEPRVYEDLNVILSDTSNCSSKYLLLADRPKCPQPSWS |
| Q9DBX3 | GTWLAAHPYKVSESEKSQLVNETHWQYYGTSDTRGNLNLTW |
| Q9DBX3 | QLVNETHWQYYGTSDTRGNLNLTWDTSALPTPAVTIELWGY |
| Q9DBX3 | LETTLSDLRVQGRAQPGRMPNGTQARGTGLTAVAVQEDNSD |
| Q6UWL2 | GRTQCVDKNECQFGATLVCGNHTSCHNTPGGFYCICLEGYR |
| Q6UWL2 | HNTPGGFYCICLEGYRATNNNKTFIPNDGTFCTDIDECEVS |
| Q6UWL2 | SCTEIDCGTPPEVPDGYIIGNYTSSLGSQVRYACREGFFSV |
| Q6UWL2 | HCQEINCGNPPEMRHAILVGNHSSRLGGVARYVCQEGFESP |
| Q6UWL2 | VISIKGQRLDPMESVREETVNLTTDSRTPEVCLALYPGTNY |
| Q6UWL2 | VNLTTDSRTPEVCLALYPGTNYTVNISTAPPRRSMPAVIGF |
| Q6UWL2 | ISSSSRDPEVCLDLRPGTNYNVSLRALSSELPVVISLTTQI |
| Q9UGT4 | GTWLAVHPNKVSMMEKSELVNETRWQYYGTANTSGNLSLTW |
| Q9UGT4 | KSELVNETRWQYYGTANTSGNLSLTWHVKSLPTQTITIELW |
| Q9UGT4 | GLTAVAVQEGNSDVVEVRLANRTGGLEVLLNQEVLSFTEQS |
| P08471 | FEIGIVNSGSGACYTEPASQNLSKVSVYAQAAQRDRLSAFG |
| P08471 | AAQRDRLSAFGVLLAGAPVSNMTYLTPRWDSLNMTTISNLN |

| | |
|---|---|
| P08471 | LLAGAPVSNMTYLTPRWDSLNMTTISNLNFSKTQANGTRIC |
| P08471 | NMTYLTPRWDSLNMTTISNLNFSKTQANGTRICLELFKPTT |
| P08471 | RWDSLNMTTISNLNFSKTQANGTRICLELFKPTTINEFCER |
| Q8N3V7 | AADVNQNLASPSATLTTPTSNSSHNPPATDVNQNPPATVVP |
| Q16563 | CGGFKGQTEIQVNCPPAVTENKTVTATFGYPFRLNEASFQP |
| Q16563 | PCKKKAVLCYFGSVTSMGSLNVSVIFGFLNMILWGGNAWFV |
| O09117 | TCGGFKGKTEIQVNCPKVGVNKNQTVTATFGYPFRLNQASF |
| O09117 | GGFKGKTEIQVNCPKVGVNKNQTVTATFGYPFRLNQASFHT |
| O09117 | VTATFGYPFRLNQASFHTPPNVSVCDVNWEKHVLIGDYSSS |
| O09117 | CNPESGVSCYFVSVTSMGSLNVSVIFGFLNMILWGGNAWFV |
| P21707 | ASHPEALAAPVTTVATLVPHNATEPASPGEGKEDAFSKLKQ |
| Q9NUM4 | YIGVKSAYVSYDVQKRTIYLNITNTLNITNNNYYSVEVENI |
| Q9NUM4 | AYVSYDVQKRTIYLNITNTLNITNNNYYSVEVENITAQVQF |
| Q9NUM4 | LNITNTLNITNNNYYSVEVENITAQVQFSKTVIGKARLNNI |
| Q9NUM4 | ENITAQVQFSKTVIGKARLNNITIIGPLDMKQIDYTVPTVI |
| Q9NYV8 | LLLVTSVFLFLNIALINIHINASINGYRRNKTCSSDSSNFT |
| Q9NYV8 | FLNIALINIHINASINGYRRNKTCSSDSSNFTRFSSLIVLT |
| Q9NYV8 | HINASINGYRRNKTCSSDSSNFTRFSSLIVLTSTVFIFIPF |
| Q64302 | IVASLGLAEGPKCSDAHGVWNYTFASTEGQYLLNSSMWSKC |
| Q64302 | SDAHGVWNYTFASTEGQYLLNSSMWSKCYEPKHIVEWHVTL |
| P48230 | GYSFIISAISINKGPKCLMANSTWGYPFHDGDYLNDEALWN |
| P48230 | YLNDEALWNKCREPLNVVPWNLTLFSILLVVGGIQMVLCAI |
| P40200 | VWEKTVNTEENVYATLGSDVNLTCQTQTVGFFVQMQWSKVT |
| P40200 | FYCAYGRPCESLVTFTETPENGSKWTLHLRNMSCSVSGRYE |
| P40200 | SLVTFTETPENGSKWTLHLRNMSCSVSGRYECMLVLYPEGI |
| P40200 | QTKIYNLLIQTHVTADEWNSNHTIEIEINQTLEIPCFQNSS |
| P40200 | IQTHVTADEWNSNHTIEIEINQTLEIPCFQNSSSKISSEFT |
| P40200 | NSNHTIEIEINQTLEIPCFQNSSSKISSEFTYAWSVENSST |
| P40200 | SVENSSTDSWVLLSKGIKEDNGTQETLISQNHLISNSTLLK |
| P40200 | GIKEDNGTQETLISQNHLISNSTLLKDRVKLGTDYRLHLSP |
| P40200 | LRSSTTVKVFAKPEIPVIVENNSTDVLVERRFTCLLKNVFP |
| P40200 | RSSTTVKVFAKPEIPVIVENNSTDVLVERRFTCLLKNVFPK |
| P40200 | TDVLVERRFTCLLKNVFPKANITWFIDGSFLHDEKEGIYIT |
| P40200 | FLELKSVLTRVHSNKPAQSDNLTIWCMALSPVPGNKVWNIS |
| P40200 | SDNLTIWCMALSPVPGNKVWNISSEKITFLLGSEISSTDPP |
| P40200 | SSVTLVDVSALRPNTTPQPSNSSMTTRGFNYPWTSSGTDTK |
| P40200 | LHDNVFTSTARAFSEVPTTANGSTKTNHVHITGIVVNKPKD |
| Q19981 | SKGWYGSQCDHCFGRIRISDNASYISDGPLDYSPSAKCTWL |
| Q19981 | ASGKALVHFFSDLAINLNGFNVSYESNRCAYNCSNHGSCLN |
| Q19981 | DLAINLNGFNVSYESNRCAYNCSNHGSCLNGKCDCEDGYKG |
| Q19981 | WSIGGEFFDGSSDPNNIDVYNVTSRIWSKVEVSGDMPKPRF |

| | |
|---|---|
| Q19981 | TNELWIFDMGSKKWAQQIHKNETIIAAPFAVAGHSAHVIRS |
| Q19981 | FGFMHHVQIYNFETEEWTVANTSDHVYGRFKHSAVEYTTPT |
| Q19981 | VEYTTPTGATAILVYGGSMWNNTITDSLMQFDTSTKKWSNL |
| Q19981 | YLHAAAYLNGLMVVVGGRGSNVTAGSKSECFSNMVQSYDVA |
| Q19981 | KSECFSNMVQSYDVACKQWSNMSTAPVDLKRFGHSVHVIGQ |
| Q19981 | SVLADRNKFLSPSHFPSFFRNATECPMPCAQRNNCSDCTDL |
| Q19981 | SHFPSFFRNATECPMPCAQRNNCSDCTDLEQCMWCPSTNRC |
| Q19981 | HFPSFFRNATECPMPCAQRNNCSDCTDLEQCMWCPSTNRCI |
| Q19981 | TGLGLCIRGTSTGPLEPKPENSTWYFIDCPACQCNGHSTCF |
| Q19981 | CFTSVGSFPPVTIEKCQSCQNHTTGAHCERCAPGFYGDARN |
| Q19981 | FYELAVDFIFTFKLRSDDKDNHTSEIYLYSVPYKKDTDVTF |
| Q19981 | TDVTFQISCESPKGNALVALNMTSSYVNGLADKSQAMMVDT |
| Q19981 | KGFRRVYVASDKGYPFGPDSNTTFFVRVYNFNTPVQIVVSF |
| A2A690 | NAQNGHLLEDDYYSPHGMLANGSRGDLLERVSQASSYPDVK |
| Q7Z7G0 | ALGNAQKLPKGKRPNLKVHINTTSDSILLKFLRPSPNVKLE |
| P10636 | KSTPTAEDVTAPLVDEGAPGKQAAAQPHTEIPEGTTAEEAG |
| P10636 | PRGKPVSRVPQLKARMVSKSKDGTGSDDKKAKTSTRSSAKT |
| P10636 | SRTGSSGAKEMKLKGADGKTKIATPRGAAPPGQKGQANATR |
| P10636 | KGADGKTKIATPRGAAPPGQKGQANATRIPAKTPPAPKTPP |
| P10636 | PRGAAPPGQKGQANATRIPAKTPPAPKTPPSSGEPPKSGDR |
| P10636 | TPGSRSRTPSLPTPPTREPKKVAVVRTPPKSPSSAKSRLQT |
| P10636 | SLPTPPTREPKKVAVVRTPPKSPSSAKSRLQTAPVPMPDLK |
| P10636 | AKSRLQTAPVPMPDLKNVKSKIGSTENLKHQPGGGKVQIIN |
| P10636 | IGSTENLKHQPGGGKVQIINKKLDLSNVQSKCGSKDNIKHV |
| P10636 | GSTENLKHQPGGGKVQIINKKLDLSNVQSKCGSKDNIKHVP |
| P10636 | NIHHKPGGGQVEVKSEKLDFKDRVQSKIGSLDNITHVPGGG |
| P10636 | GGGQVEVKSEKLDFKDRVQSKIGSLDNITHVPGGGNKKIET |
| P10636 | RVQSKIGSLDNITHVPGGGNKKIETHKLTFRENAKAKTDHG |
| Q9XTQ6 | VAYCGGVVHAGEIVAELYHTNVTVKWHTDYERQLVDFSIWF |
| Q9XTQ6 | WFGASTPDVLFLGFSDFGDTNNSDVLMYYNSKKEIKDAYTN |
| Q9XTQ6 | VNYIYYYPASDVEVCKSAISNSTLRAYFSERHGMDGKRMQI |
| P01848 | RDSKSSDKSVCLFTDFDSQTNVSQSKDSDVYITDKTVLDMR |
| P01848 | SDVYITDKTVLDMRSMDFKSNSAVAWSNKSDFACANAFNNS |
| P01848 | KTVLDMRSMDFKSNSAVAWSNKSDFACANAFNNSIIPEDTF |
| P01848 | SCDVKLVEKSFETDTNLNFQNLSVIGFRILLLKVAGFNLLM |
| P20061 | PLTNYYQLSLDVLALCLFNGNYSTAEVVNHFTPENKNYYFG |
| P20061 | TCVKKSLINGQIKADEGSLKNISIYTKSLVEKILSEKKENG |
| P20061 | GKTFLDINKDSSCVSASGNFNISADEPITVTPPDSQSYISV |
| P20061 | ISADEPITVTPPDSQSYISVNYSVRINETYFTNVTVLNGSV |
| P20061 | ITVTPPDSQSYISVNYSVRINETYFTNVTVLNGSVFLSVME |
| P20061 | DSQSYISVNYSVRINETYFTNVTVLNGSVFLSVMEKAQKMN |

| | |
|---|---|
| P20061 | ISVNYSVRINETYFTNVTVLNGSVFLSVMEKAQKMNDTIFG |
| P20061 | NVTVLNGSVFLSVMEKAQKMNDTIFGFTMEERSWGPYITCI |
| Q2MV57 | LSLLQVEEGVLPVPTCGGRRNETVDWNVTVSPRESTLEVTI |
| Q2MV57 | EEGVLPVPTCGGRRNETVDWNVTVSPRESTLEVTIRWKRGL |
| Q2MV57 | SASHNASCLAHLLIQVEIYPNTSVTHNASENMTVIPNQVYQ |
| Q2MV57 | HLLIQVEIYPNTSVTHNASENMTVIPNQVYQPLGPCPCDLT |
| Q2MV57 | SVGSAPRNVNVEEHYVFRWQNNSISGLDITVIRAEISAQQR |
| Q9UIK5 | PVKLAAFPTSLSDCQTPTGWNCSGYDDRENDLFLCDTNTCK |
| Q9UIK5 | IDCSQTNFNPLCASDGKSYDNACQIKEASCQKQEKIEVMSL |
| Q9UIK5 | EASCQKQEKIEVMSLGRCQDNTTTTTKSEDGHYARTDYAEN |
| Q9WTS6 | LSYFIAMHLFGLNWHLQQTENDTFENGKVNSDTVPTNTVSL |
| Q9WTS6 | TNTVSLPSGDNGKLGGFTHENNTIDSGELDIGRRAIQEVPP |
| Q9WTS6 | PPGIFWRSQLFIDQPQFLKFNISLQKDALIGVYGRKGLPPS |
| Q9WTS6 | DQCSGHGTYLQESGSCTCDPNWTGPDCSNEICSVDCGSHGV |
| Q9WTS6 | RISFLIGSDSTHVLPGESPFNKSLASVIRGQVLTADGTPLI |
| Q9WTS6 | LASVIRGQVLTADGTPLIGVNVSFLHYSEYGYTITRQDGMF |
| Q9WTS6 | IDGSLYVGDFNYVRRIFPSGNVTSVLELRNKDFRHSSNPAH |
| Q9WTS6 | SMNFYEVASPTDQELYIFDINGTHQYTVSLVTGDYLYNFSY |
| Q9WTS6 | FDINGTHQYTVSLVTGDYLYNFSYSNDNDVTAVTDSNGNTL |
| Q9WTS6 | KSDETGWTTFFDYDSEGRLTNVTFPTGVVTNLHGDMDKAIT |
| Q9WTS6 | KAITVDIESSSREEDVSITSNLSSIDSFYTMVQDQLRNSYQ |
| Q9WTS6 | HYQTEPHVLAGTANPTVAKRNMTLPGENGQNLVEWRFRKEQ |
| Q9WTS6 | AYDTSGHPTLWLPSSKLMAVNVTYSSTGQIASIQRGTTSEK |
| Q9WTS6 | TMQTIRSIGYYRNIYNPPESNASIITDYNEEGLLLQTAFLG |
| Q9WTS6 | IQYDNMGRVTKREIKIGPFANTTKYAYEYDVDGQLQTVYLN |
| Q9WTS6 | QHLQFFYADLTYPTRITHVYNHSSSEITSLYYDLQGHLFAM |
| Q9WTS6 | ESDLGTLRLTSGRKALENGINVTVSQSTTVVNGRTRRFADV |
| Q3UHK6 | PPGTFWRSQVFIDHPVHLKFNVSLGKAALVGIYGRKGLPPS |
| Q3UHK6 | HACVIRGQVMTSDGTPLVGVNISFINNPLFGYTISRQDGSF |
| Q3UHK6 | SDGSLYVGDFNYIRRIFPSGNVTNILEMRNKDFRHSHSPAH |
| Q3UHK6 | FDTSGKHLYTQSLPTGDYLYNFTYTGDGDITHITDNNGNMV |
| Q3UHK6 | KSNENGWTTFYEYDSFGRLTNVTFPTGQVSSFRSDTDSSVH |
| Q3UHK6 | DSSVHVQVETSSKDDVTITTNLSASGAFYTLLQDQVRNSYY |
| Q3UHK6 | ALQTEPHLLAGTVNPTVGKRNVTLPIDNGLNLVEWRQRKEQ |
| Q3UHK6 | LYDQAGRPSLWSPSSRLNGVNVTYSPGGHIAGIQRGIMSER |
| Q3UHK6 | TLETIRSVGYYRNIYQPPEGNASVIQDFTEDGHLLHTFYLG |
| Q3UHK6 | VQYDNMGRVVKKELKVGPYANTTRYSYEYDADGQLQTVSIN |
| Q3UHK6 | HHLQFFYADLTNPTKVTHLYNHSSSEITSLYYDLQGHLFAM |
| Q3UHK6 | EGDLAILGLSGGRRTLENGVNVTVSQINTVLSGRTRRYTDI |
| P24821 | LATEGGVLKKVIRHKRQSGVNATLPEENQPVVFNHVYNIKL |
| P24821 | CCLQPATGRLDTRPFCSGRGNFSTEGCGCVCEPGWKGPNCS |

| | |
|---|---|
| P24821 | RGNFSTEGCGCVCEPGWKGPNCSEPECPGNCHLRGRCIDGQ |
| P24821 | GEDCSELICPNDCFDRGRCINGTCYCEEGFTGEDCGKPTCP |
| P24821 | YRQTGLAPGQEYEISLHIVKNNTRGPGLKRVTTTRLDAPSQ |
| P24821 | ETSLTLLWKTPLAKFDRYRLNYSLPTGQWVGVQLPRNTTSY |
| P24821 | RYRLNYSLPTGQWVGVQLPRNTTSYVLRGLEPGQEYNVLLT |
| P24821 | RHKSKPARVKASTEQAPELENLTVTEVGWDGLRLNWTAADQ |
| P24821 | QAPELENLTVTEVGWDGLRLNWTAADQAYEHFIIQVQEANK |
| P24821 | QAYEHFIIQVQEANKVEAARNLTVPGSLRAVDIPGLKAATP |
| P24821 | ETPNLGEVVVAEVGWDALKLNWTAPEGAYEYFFIQVQEADT |
| P24821 | GAYEYFFIQVQEADTVEAAQNLTVPGGLRSTDLPGLKAATH |
| P24821 | DFSTTPLSVEVLTEEVPDMGNLTVTEVSWDALRLNWTTPDG |
| P24821 | EVPDMGNLTVTEVSWDALRLNWTTPDGTYDQFTIQVQEADQ |
| P24821 | GTYDQFTIQVQEADQVEEAHNLTVPGSLRSMEIPGLRAGTP |
| P24821 | DLPQLGDLAVSEVGWDGLRLNWTAADNAYEHFVIQVQEVNK |
| P24821 | NAYEHFVIQVQEVNKVEAAQNLTLPGSLRAVDIPGLEAATP |
| P24821 | RTPVLSAEASTAKEPEIGNLNVSDITPESFNLSWMATDGIF |
| P24821 | TAKEPEIGNLNVSDITPESFNLSWMATDGIFETFTIEIIDS |
| P24821 | FETFTIEIIDSNRLLETVEYNISGAERTAHISGLPPSTDFI |
| P24821 | SIRTKTISATATTEALPLLENLTISDINPYGFTVSWMASEN |
| P24821 | EPVSGSFTTALDGPSGLVTANITDSEALARWQPAIATVDSY |
| P24821 | GAFWYRNCHRVNLMGRYGDNNHSQGVNWFHWKGHEHSIQFA |
| Q80YX1 | LTPEGGVLKKIIRHKRESGLNMTLPEENQPVVFNHIYNIKL |
| Q80YX1 | CCLQPAEGRLDTRPFCSGRGNFSAEGCGCVCEPGWKGPNCS |
| Q80YX1 | RGNFSAEGCGCVCEPGWKGPNCSEPDCPGNCNLRGQCLDGQ |
| Q80YX1 | GEDCSELICPNDCFDRGRCINGTCYCEEGFTGEDCGELTCP |
| Q80YX1 | YRQTGLAPGQEYEISLHIVKNNTRGPGLKKVTTTRLDAPSH |
| Q80YX1 | QDSLTFFWTTPLAKFDRYRLNYSLPTGQSMEVQLPKDATSH |
| Q80YX1 | RHKSKPARVKASTEEVPSLENLTVTEAGWDGLRLNWTADDL |
| Q80YX1 | EVPSLENLTVTEAGWDGLRLNWTADDLAYEYFVIQVQEANN |
| Q80YX1 | LAYEYFVIQVQEANNVETAHNFTVPGNLRAADIPGLKVATS |
| Q80YX1 | TTPNLGEVTVAEVGWDALTLNWTAPEGAYKNFFIQVLEADT |
| Q80YX1 | GAYKNFFIQVLEADTTQTVQNLTVPGGLRSVDLPGLKAATR |
| Q80YX1 | DLPQLGGLSVTEVSWDGLTLNWTTDDLAYKHFVVQVQEANN |
| Q80YX1 | LAYKHFVVQVQEANNVEAAQNLTVPGSLRAVDIPGLKADTP |
| Q80YX1 | RTPMLSTDVSTAREPEIGNLNVSDVTPKSFNLSWTATDGIF |
| Q80YX1 | TAREPEIGNLNVSDVTPKSFNLSWTATDGIFDMFTIEIIDS |
| Q80YX1 | FDMFTIEIIDSNRLLQTAEHNISGAERTAHISGLPPSTDFI |
| Q80YX1 | SIRTKTISTTATTEALPLLENLTISDTNPYGFTVSWTASEN |
| Q80YX1 | DPVSGTLITALDGPSGLLIANITDSEALAMWQPAIATVDSY |
| Q80YX1 | WKAYAAGFGDRREEFWLGLDNLSKITAQGQYELRVDLQDHG |
| Q80YX1 | GAFWYKNCHRVNLMGRYGDNNHSQGVNWFHWKGHEYSIQFA |

| | |
|---|---|
| O61307 | RMKFLIDESSLQNYAKLETFNESRSAVIRGRVVTSLGMGLV |
| Q9UQP3 | YVEGFGDPMKEFWLGLDKLHNLTTGTPARYEVRVDLQTANE |
| P10646 | CFLEEDPGICRGYITRYFYNNQTKQCERFKYGGCLGNMNNF |
| P10646 | CEDGPNGFQVDNYGTQLNAVNNSLTPQSTKVPSLFEFHGPS |
| P02786 | KLVHANFGTKKDFEDLYTPVNGSIVIVRAGKITFAEKVANA |
| P02786 | FFGHAHLGTGDPYTPGFPSFNHTQFPPSRSSGLPNIPVQTI |
| P02786 | HTLPALLENLKLRKQNNGAFNETLFRNQLALATWTIQGAAN |
| Q62351 | KLVHANFGTKKDFEELSYSVNGSLVIVRAGEITFAEKVANA |
| Q62351 | LFGHAHLGTGDPYTPGFPSFNHTQFPPSQSSGLPNIPVQTI |
| Q62351 | WGSGSHTLSALVENLKLRQKNITAFNETLFRNQLALATWTI |
| Q62351 | HTLSALVENLKLRQKNITAFNETLFRNQLALATWTIQGVAN |
| Q9UP52 | VGEQLPLEDPDVYCPYSAIGNVTGELVYAHYGRPEDLQDLR |
| Q9UP52 | VYGHVHLGTGDPYTPGFPSFNQTQFPPVASSGLPSIPAQPI |
| Q9UP52 | KTSPLLTSLIESVLKQVDSPNHSGQTLYEQVVFTNPSWDAE |
| Q9UP52 | MGRGDHTLGALLDHLRLLRSNSSGTPGATSSTGFQESRFRR |
| P20352 | FLGCLLLQVIAGAGIPEKAFNLTWISTDFKTILEWQPKPTN |
| P20352 | NLTWISTDFKTILEWQPKPTNYTYTVQISDRSRNWKNKCFS |
| P20352 | EQDGRKLNVVVKDSLTLVRKNGTFLTLRQVFGKDLGYIITY |
| P20352 | GKDLGYIITYRKGSSTGKKTNITNTNEFSIDVEEGVSYCFF |
| O88393 | AGVPRLFLVSEGSVVQFSSGNFSLTAETEERSFPQENEHLL |
| O88393 | TNGYSGMELTLLDPSCKAKMNGTHFVLESPLNGCGTRHRRS |
| O88393 | PGDTDEGETAPLSRAGVVVFNCSLRQLRSPSGFQDQLDGNA |
| O88393 | FNCSLRQLRSPSGFQDQLDGNATFNMELYNTDLFLVPSPGV |
| O88393 | PIPHAEVDKKRFSFVFKSVFNTSLLFLHCELTLCSRNKGSQ |
| P26342 | SACLATAGPEPSTRCELSPINASHPVQALMESFTVLSGCAS |
| P26342 | AGVPRLFLVSEGSVVQFPSGNFSLTAETEERNFPQENEHLL |
| P26342 | TNGYSGMELTLLDPSCKAKMNGTHFVLESPLNGCGTRHRRS |
| P26342 | PGDGDEGETAPLSRAGVVVFNCSLRQLRNPSGFQGQLDGNA |
| P26342 | FNCSLRQLRNPSGFQGQLDGNATFNMELYNTDLFLVPSPGV |
| P26342 | PIPHAEVDKKRFSFLFKSVFNTSLLFLHCELTLCSRKKGSL |
| P09533 | PPSQGEVPPGPLPEAVLALYNSTRDRVAGESAEPEPEPEAD |
| P09533 | THNEIYDKFKQSTHSIYMFFNTSELREAVPEPVLLSRAELR |
| P09533 | RLLRLKLKVEQHVELYQKYSNNSWRYLSNRLLAPSNSPEWL |
| P07200 | PPSQGDVPPGPLPEAVLALYNSTRDRVAGESVEPEPEPEAD |
| P07200 | SGNQIYDKFKGTPHSLYMLFNTSELREAVPEPVLLSRAELR |
| P07200 | RLLRLKLKVEQHVELYQKYSNDSWRYLSNRLLAPSDSPEWL |
| P01137 | PPSQGEVPPGPLPEAVLALYNSTRDRVAGESAEPEPEPEAD |
| P01137 | THNEIYDKFKQSTHSIYMFFNTSELREAVPEPVLLSRAELR |
| P01137 | RLLRLKLKVEQHVELYQKYSNNSWRYLSNRLLAPSDSPEWL |
| Q99041 | WCSDDSVFMASEEDRAEYVLNDTGYMYMGFAKQIKEKPWTF |
| Q99041 | VSRAICTMMCAANNFGVLVGNWTGDYSNGTAPYVWASSVPI |

| | |
|---|---|
| Q99041 | MMCAANNFGVLVGNWTGDYSNGTAPYVWASSVPILQQHYIT |
| Q99041 | QNQEREKNVLIAVETASIGKNISTKMVGENRRQDITLHYKF |
| Q99041 | NSRTLHISVLQNSVELGHPINLTIVLKRKTATPQNVNISCS |
| Q99041 | GHPINLTIVLKRKTATPQNVNISCSLDLQTYTGNKKTNLGV |
| Q62313 | ATDHSLGDSRRQPEKTDAELNETARPLSPVNPKLEKSDQSS |
| Q62313 | GTLTDSMKNEKDDLYKDSSGNTSAESSHFFAYLVTAAVLVA |
| P05543 | ATIHCASPEGKVTACHSSQPNATLYKMSSINADFAFNLYRR |
| P05543 | MLSFGACCSTQTEIVETLGFNLTDTPMVEIQHGFQHLICSL |
| P05543 | LGFNLTDTPMVEIQHGFQHLICSLNFPKKELELQIGNALFI |
| P05543 | KFLNDVKTLYETEVFSTDFSNISAAKQEINSHVEMQTKGKV |
| P05543 | VQVPMMHQMEQYYHLVDMELNCTVLQMDYSKNALALFVLPK |
| P00735 | KLNECLEGNCAEGVGMNYRGNVSVTRSGIECQLWRSRYPHK |
| P00735 | TRSGIECQLWRSRYPHKPEINSTTHPGADLRENFCRNPDGS |
| P00735 | ISDRWVLTAAHCLLYPPWDKNFTVDDLLVRIGKHSRTRYER |
| P00734 | AACLEGNCAEGLGTNYRGHVNITRSGIECQLWRSRYPHKPE |
| P00734 | TRSGIECQLWRSRYPHKPEINSTTHPGADLQENFCRNPDSS |
| P00734 | ISDRWVLTAAHCLLYPPWDKNFTENDLLVRIGKHSRTRYER |
| P19221 | MDCLEGRCAMDLGVNYLGTVNVTHTGIQCQLWRSRYPHKPE |
| P19221 | THTGIQCQLWRSRYPHKPEINSTTHPGADLKENFCRNPDSS |
| P19221 | ISDRWVLTAAHCILYPPWDKNFTENDLLVRIGKHSRTRYER |
| P19221 | CKASTRIRITDNMFCAGFKVNDTKRGDACEGDSGGPFVMKS |
| P04216 | VTSLTACLVDQSLRLDCRHENTSSSPIQYEFSLTRETKKHV |
| P04216 | KKHVLFGTVGVPEHTYRSRTNFTSKYNMKVLYLSAFTSKDE |
| P04216 | EGTYTCALHHSGHSPPISSQNVTVLRDKLVKCEGISLLAQN |
| P04216 | NVTVLRDKLVKCEGISLLAQNTSWLLLLLLSLSLLQATDFM |
| P01267 | AACLSFCQLQKQQILLSSYINSTATSYLPQCQDSGDYSPVQ |
| P01267 | AACLSFCQLQKQQILLSSYINSTATSYLPQCQDSGDYSPVQ |
| P01267 | AKRLQQNLFGGRFLVKVGQFNLSGALGTRGTFNFSHFFQQL |
| P01267 | AKRLQQNLFGGRFLVKVGQFNLSGALGTRGTFNFSHFFQQL |
| P01267 | FLVKVGQFNLSGALGTRGTFNFSHFFQQLGLPGFQDGRALA |
| P01267 | FLVKVGQFNLSGALGTRGTFNFSHFFQQLGLPGFQDGRALA |
| P01267 | SPCQLQAERAFLGTVRTLVSNPSTLPALSSIYIPQCSASGQ |
| P01267 | FPGLARYSSFQDVPVSVLEGNQTQPGGNVFLEPYLFWQILN |
| P01267 | FPGLARYSSFQDVPVSVLEGNQTQPGGNVFLEPYLFWQILN |
| P01267 | EVKLRVLQFIREAEEIVTYSNSSRFPLGESFLAAKGIRLTD |
| P01267 | EVKLRVLQFIREAEEIVTYSNSSRFPLGESFLAAKGIRLTD |
| P01267 | SEAGTWCVDPASGEGVPPGTNSSAQCPSLCEVLQSGVPSRR |
| P01267 | SEAGTWCVDPASGEGVPPGTNSSAQCPSLCEVLQSGVPSRR |
| P01267 | PVCDDSSVKVECLSRERLGVNITWKLQLVDAPPASLPDLQD |
| P01267 | SPDVLLCHVRDWRDPAEAQANASCPGVTYDQDSRQVTLRLG |
| P01267 | SPDVLLCHVRDWRDPAEAQANASCPGVTYDQDSRQVTLRLG |

| | |
|---|---|
| P01267 | TDLTTGLFSPVDLIQVIVDGNVSLPSQQHWLFKHLFSLQQA |
| P01267 | TDLTTGLFSPVDLIQVIVDGNVSLPSQQHWLFKHLFSLQQA |
| P01267 | FFECERLCDMDPCCTGFGFLNVSQLKGGEVTCLTLNSLGLQ |
| P01267 | IVDPSIRNFDVAHISTAAVGNFSAARDRCLWECSRHQDCLV |
| P01267 | GVPYAAPPLGEKRFRAPEHLNWTGSWEATKPRARCWQPGIR |
| P01267 | GVPYAAPPLGEKRFRAPEHLNWTGSWEATKPRARCWQPGIR |
| P01267 | PGVSEDCLYLNVFVPQNMAPNASVLVFFHNAAEGKGSGDRP |
| P01830 | VISLTACLVNQNLRLDCRHENNTNLPIQHEFSLTREKKKHV |
| P01830 | TYRSRVNLFSDRFIKVLTLANFTTKDEGDYMCELRVSGQNP |
| P01830 | KDEGDYMCELRVSGQNPTSSNKTINVIRDKLVKCGGISLLV |
| P01831 | VTSLTACLVNQNLRLDCRHENNTKDNSIQHEFSLTREKRKH |
| P01831 | TYRSRVTLSNQPYIKVLTLANFTTKDEGDYFCELQVSGANP |
| P01831 | KDEGDYFCELQVSGANPMSSNKSISVYRDKLVKCGGISLLV |
| P01266 | GSFQTVQCQNDGRSCWCVGANGSEVLGSRQPGRPVACLSFC |
| P01266 | KSPPQCSAEGEFMPVQCKFVNTTDMMIFDLVHSYNRFPDAF |
| P01266 | PKRLQQNLFGGKFLVNVGQFNLSGALGTRGTFNFSQFFQQL |
| P01266 | FLNGGRQEDLAKPLSVGLDSNSSTGTPEAAKKDGTMNKPTV |
| P01266 | TPCQLQSEQAFLRTVQALLSNSSMLPTLSDTYIPQCSTDGQ |
| P01266 | DLTPAKLLVKIMSYREAASGNFSLFIQSLYEAGQQDVFPVL |
| P01266 | EAKLRVLQFIRETEEIVSASNSSRFPLGESFLVAKGIRLRN |
| P01266 | GTRVTGGQPACESPRCPLPFNASEVVGGTILCETISGPTGS |
| P01266 | RGFCQIQVKTFGTLVSIPVCNNSSVQVGCLTRERLGVNVTW |
| P01266 | GFCQIQVKTFGTLVSIPVCNNSSVQVGCLTRERLGVNVTWK |
| P01266 | PVCNNSSVQVGCLTRERLGVNVTWKSRLEDIPVASLPDLHD |
| P01266 | GFQNMLSGLYNPIVFSASGANLTDAHLFCLLACDRDLCCDG |
| P01266 | SPSVLLCNVKDWMDPSEAWANATCPGVTYDQESHQVILRLG |
| P01266 | FFECERRCDADPCCTGFGFLNVSQLKGGEVTCLTLNSLGIQ |
| P01266 | GVPYAAPPLAERRFQAPEPLNWTGSWDASKPRASCWQPGTR |
| P01266 | PGVSEDCLYLNVFIPQNVAPNASVLVFFHNTMDREESEGWP |
| P01266 | WYYSLEHSTDDYASFSRALENATRDYFIICPIIDMASAWAK |
| Q02763 | QQASFLPATLTMTVDKGDNVNISFKKVLIKEEDAVIYKNGS |
| Q02763 | NVNISFKKVLIKEEDAVIYKNGSFIHSVPRHEVPDILEVHL |
| Q02763 | TNEEMTLVKPDGTVLHPKDFNHTDHFSVAIFTIHRILPPDS |
| Q02763 | DSGVWVCSVNTVAGMVEKPFNISVKVLPKPLNAPNVIDTGH |
| Q02763 | LPKPLNAPNVIDTGHNFAVINISSEPYFGDGPIKSKKLLYK |
| Q02763 | SIGLPPPRGLNLLPKSQTTLNLTWQPIFPSSEDDFYVEVER |
| Q02763 | VEVERRSVQKSDQQNIKVPGNLTSVLLNNLHPREQYVVRAR |
| Q02763 | LTAWTLSDILPPQPENIKISNITHSSAVISWTILDGYSISS |
| Q02763 | TIRYKVQGKNEDQHVDVKIKNATITQYQLKGLEPETAYQVD |
| P01033 | TAFCNSDLVIRAKFVGTPEVNQTTLYQRYEIKMTKMYKGFQ |
| P01033 | IRFVYTPAMESVCGYFHRSHNRSEEFLIAGKLQDGLLHITT |

| | |
|---|---|
| Q21265 | KGLNNLRYTVQHVEVFKKPSNMTTLPDEIFTPSEAPACGLK |
| Q9GZM7 | RADDCALPYLGAICYCDLFCNRTVSDCCPDFWDFCLGVPPP |
| Q9GZM7 | LVDPDMIKAINQGNYGWQAGNHSAFWGMTLDEGIRYRLGTI |
| Q8TB96 | PLLAGLALLGVGPVPARALHNVTAELFGAEAWGTLAAFGDL |
| Q8TB96 | VFLADQNAPYFKPKVKVSFKNHSALITSVVPGDYDGDSQMD |
| Q8TB96 | TYLPKNYAKSELGAVIFWGQNQTLDPNNMTILNRTFQDEPL |
| Q8TB96 | AKSELGAVIFWGQNQTLDPNNMTILNRTFQDEPLIMDFNGD |
| Q8TB96 | GAVIFWGQNQTLDPNNMTILNRTFQDEPLIMDFNGDLIPDI |
| Q8TB96 | DEPLIMDFNGDLIPDIFGITNESNQPQILLGGNLSWHPALT |
| Q8TB96 | IPDIFGITNESNQPQILLGGNLSWHPALTTTSKMRIPHSHA |
| Q8TB96 | SHAFIDLTEDFTADLFLTTLNATTSTFQFEIWENLDGNFSV |
| Q8TB96 | TTLNATTSTFQFEIWENLDGNFSVSTILEKPQNMMVVGQSA |
| Q8TB96 | TLHIGDYNMDGYPDALVILKNTSGSNQQAFLLENVPCNNAS |
| Q8TB96 | LKNTSGSNQQAFLLENVPCNNASCEEARRMFKVYWELTDLN |
| Q8TB96 | VNQPGPYIMYTTVDANGYLKNGSAGQLSQSAHLALQLPYNV |
| Q15399 | RSKNGLIHVPKDLSQKTTILNISQNYISELWTSDILSLSKL |
| Q15399 | KHLDLSFNAFDALPICKEFGNMSQLKFLGLSTTHLEKSSVL |
| Q15399 | FLGLSTTHLEKSSVLPIAHLNISKVLLVLGETYGEKEDPEG |
| Q15399 | DVFGFPQSYIYEIFSNMNIKNFTVSGTRMVHMLCPSKISPF |
| Q15399 | SVSYDEKKGDCSWTKSLLSLNMSSNILTDTIFRCLPPRIKV |
| Q15399 | YPESYRGTLLKDFHMSELSCNITLLIVTIVATMLVLAVTVT |
| O60603 | DSFSSLGSLEHLDLSYNYLSNLSSSWFKPLSSLTFLNLLGN |
| O60603 | LEIDASDLQSYEPKSLKSIQNVSHLILHMKQHILLLEIFVD |
| O60603 | LRQNHLASLEKTGETLLTLKNLTNIDISKNSFHSMPETCQW |
| O60603 | KNSFHSMPETCQWPEKMKYLNLSSTRIHSVTGCIPKTLEIL |
| Q9QUN7 | KYLNLMGNPYQTLGVTSLFPNLTNLQTLRIGNVETFSEIRR |
| Q9QUN7 | LSQNHLRSMQKTGEILLTLKNLTSLDISRNTFHPMPDSCQW |
| Q9QUN7 | RNTFHPMPDSCQWPEKMRFLNLSSTGIRVVKTCIPQTLEVL |
| O15455 | HEVADCSHLKLTQVPDDLPTNITVLNLTHNQLRRLPAANFT |
| O15455 | CSHLKLTQVPDDLPTNITVLNLTHNQLRRLPAANFTRYSQL |
| O15455 | PTNITVLNLTHNQLRRLPAANFTRYSQLTSLDVGFNTISKL |
| O15455 | LNLQHNELSQLSDKTFAFCTNLTELHLMSNSIQKIKNNPFV |
| O15455 | LLLSNNKIQALKSEELDIFANSSLKKLELSSNQIKEFSPGC |
| O15455 | FLNNVQLGPSLTEKLCLELANTSIRNLSLSNSQLSTTSNTT |
| O15455 | QLGPSLTEKLCLELANTSIRNLSLSNSQLSTTSNTTFLGLK |
| O15455 | LANTSIRNLSLSNSQLSTTSNTTFLGLKWTNLTMLDLSYNN |
| O15455 | LSNSQLSTTSNTTFLGLKWTNLTMLDLSYNNLNVVGNDSFA |
| O15455 | LKWTNLTMLDLSYNNLNVVGNDSFAWLPQLEYFFLEYNNIQ |
| O15455 | LINLKYLSLSNSFTSLRTLTNETFVSLAHSPLHILNLTKNK |
| O15455 | LRTLTNETFVSLAHSPLHILNLTKNKISKIESDAFSWLGHL |
| O15455 | LRRVALKNVDSSPSPFQPLRNLTILDLSNNNIANINDDMLE |

| | |
|---|---|
| O15455 | NLQKNLITSVEKKVFGPAFRNLTELDMRFNPFDCTCESIAW |
| O15455 | MRFNPFDCTCESIAWFVNWINETHTNIPELSSHYLCNTPPH |
| Q99MB1 | YNVADCSHLKLTHIPDDLPSNITVLNLTHNQLRRLPPTNFT |
| Q99MB1 | CSHLKLTHIPDDLPSNITVLNLTHNQLRRLPPTNFTRYSQL |
| Q99MB1 | PSNITVLNLTHNQLRRLPPTNFTRYSQLAILDAGFNSISKL |
| Q99MB1 | LNLQHNELSQISDQTFVFCTNLTELDLMSNSIHKIKSNPFK |
| Q99MB1 | LLLAKNKILALRSEELEFLGNSSLRKLDLSSNPLKEFSPGC |
| Q99MB1 | LLNNAQLNPHLTEKLCWELSNTSIQNLSLANNQLLATSEST |
| Q99MB1 | QLNPHLTEKLCWELSNTSIQNLSLANNQLLATSESTFSGLK |
| Q99MB1 | LANNQLLATSESTFSGLKWTNLTQLDLSYNNLHDVGNGSFS |
| Q99MB1 | LKWTNLTQLDLSYNNLHDVGNGSFSYLPSLRYLSLEYNNIQ |
| Q99MB1 | LVSLKYLSLSKTFTSLQTLTNETFVSLAHSPLLTLNLTKNH |
| Q99MB1 | LQTLTNETFVSLAHSPLLTLNLTKNHISKIANGTFSWLGQL |
| Q99MB1 | LAHSPLLTLNLTKNHISKIANGTFSWLGQLRILDLGLNEIE |
| Q99MB1 | LRRVALKNVDISPSPFRPLRNLTILDLSNNNIANINEDLLE |
| Q99MB1 | MRFNPFDCTCESISWFVNWINQTHTNISELSTHYLCNTPHH |
| Q99MB1 | FDCTCESISWFVNWINQTHTNISELSTHYLCNTPHHYYGFP |
| O00206 | MAFLSCVRPESWEPCVEVVPNITYQCMELNFYKIPDNLPFS |
| O00206 | KELNVAHNLIQSFKLPEYFSNLTNLEHLDLSSNKIQSIYCT |
| O00206 | NKIQSIYCTDLRVLHQMPLLNLSLDLSLNPMNFIQPGAFKE |
| O00206 | EFRNEGNLEKFDKSALEGLCNLTIEEFRLAYLDYYLDDIID |
| O00206 | RLAYLDYYLDDIIDLFNCLTNVSSFSLVSVTIERVKDFSYN |
| O00206 | KMAGNSFQENFLPDIFTELRNLTFLDLSQCQLEQLSPTAFN |
| O00206 | CQLEQLSPTAFNSLSSLQVLNMSHNNFFSLDTFPYKCLNSL |
| O00206 | HIMTSKKQELQHFPSSLAFLNLTQNDFACTCEHQSFLQWIK |
| O00206 | VERMECATPSDKQGMPVLSLNITCQMNKTIIGVSVLSVLVV |
| O00206 | ATPSDKQGMPVLSLNITCQMNKTIIGVSVLSVLVVSVVAVL |
| Q9QUK6 | ALFFSCLTPGSLNPCIEVVPNITYQCMDQKLSKVPDDIPSS |
| Q9QUK6 | TKNIDLSFNPLKILKSYSFSNFSELQWLDLSRCEIETIEDK |
| Q9QUK6 | KKLNVAHNFIHSCKLPAYFSNLTNLVHVDLSYNYIQTITVN |
| Q9QUK6 | NYIQTITVNDLQFLRENPQVNLSLDMSLNPIDFIQDQAFQG |
| Q9QUK6 | IQDQAFQGIKLHELTLRGNFNSSNIMKTCLQNLAGLHVHRL |
| Q9QUK6 | FRLTYTNDFSDDIVKFHCLANVSAMSLAGVSIKYLEDVPKH |
| Q9QUK6 | NTLKMAGNSFKDNTLSNVFANTTNLTFLDLSKCQLEQISWG |
| Q9QUK6 | KMAGNSFKDNTLSNVFANTTNLTFLDLSKCQLEQISWGVFD |
| Q9QUK6 | CQLEQISWGVFDTLHRLQLLNMSHNNLLFLDSSHYNQLYSL |
| Q9QUK6 | NRIETSKGILQHFPKSLAFFNLTNNSVACICEHQKFLQWVK |
| Q9QUK6 | ETSKGILQHFPKSLAFFNLTNNSVACICEHQKFLQWVKEQK |
| Q9QUK6 | EQKQFLVNVEQMTCATPVEMNTSLVLDFNNSTCYMYKTIIS |
| Q9QUK6 | VEQMTCATPVEMNTSLVLDFNNSTCYMYKTIISVSVVSVIV |
| Q9QUK6 | EQMTCATPVEMNTSLVLDFNNSTCYMYKTIISVSVVSVIVV |

| | |
|---|---|
| O60602 | PVFGIPSCSFDGRIAFYRFCNLTQVPQVLNTTERLLLSFNY |
| O60602 | FDGRIAFYRFCNLTQVPQVLNTTERLLLSFNYIRTVTASSF |
| O60602 | NMVLEILDVSGNGWTVDITGNFSNAISKSQAFSLILAHHIM |
| O60602 | NKINKIADEAFYGLDNLQVLNLSYNLLGELYSSNFYGLPKV |
| O60602 | IPSIPDIFLSGNKLVTLPKINLTANLIHLSENRLENLDILY |
| O60602 | DITHNKFICECELSTFINWLNHTNVTIAGPPADIYCVYPDS |
| O60602 | HNKFICECELSTFINWLNHTNVTIAGPPADIYCVYPDSFSG |
| Q9EPW9 | VGSMTPFSNELESMVDYSNRNLTHVPKDLPPRTKALSLSQN |
| Q9EPW9 | HVFLFNQDLEYLDVSHNRLQNISCCPMASLRHLDLSFNDFD |
| Q9EPW9 | RHLDLSFNDFDVLPVCKEFGNLTKLTFLGLSAAKFRQLDLL |
| Q9EPW9 | LLDLVSYHIKGGETESLQIPNTTVLHLVFHPNSLFSVQVNM |
| Q9EPW9 | PNTTVLHLVFHPNSLFSVQVNMSVNALGHLQLSNIKLNDEN |
| Q9EPW9 | ENCQRLMTFLSELTRGPTLLNVTLQHIETTWKCSVKLFQFF |
| Q9EPW9 | CSVKLFQFFWPRPVEYLNIYNLTITERIDREEFTYSETALK |
| Q9EPW9 | SDTPFIHMVCPPSPSSFTFLNFTQNVFTDSVFQGCSTLKRL |
| Q9EPW9 | TLILQRNGLKNFFKVALMTKNMSSLETLDVSLNSLNSHAYD |
| Q9EPW9 | SLNSHAYDRTCAWAESILVLNLSSNMLTGSVFRCLPPKVKV |
| Q9NR97 | SMLTCIFLLISGSCELCAEENFSRSYPCDEKKQNDSVIAEC |
| Q9NR97 | CELCAEENFSRSYPCDEKKQNDSVIAECSNRRLQEVPQTVG |
| Q9NR97 | TVGKYVTELDLSDNFITHITNESFQGLQNLTKINLNHNPNV |
| Q9NR97 | LDLSDNFITHITNESFQGLQNLTKINLNHNPNVQHQNGNPG |
| Q9NR97 | NHNPNVQHQNGNPGIQSNGLNITDGAFLNLKNLRELLLEDN |
| Q9NR97 | IPSGLPESLTELSLIQNNIYNITKEGISRLINLKNLYLAWN |
| Q9NR97 | LFLSNTQIKYISEEDFKGLINLTLLDLSGNCPRCFNAPFPC |
| Q9NR97 | FPCVPCDGGASINIDRFAFQNLTQLRYLNLSSTSLRKINAA |
| Q9NR97 | GASINIDRFAFQNLTQLRYLNLSSTSLRKINAAWFKNMPHL |
| Q9NR97 | RLEILDLSFNYIKGSYPQHINISRNFSKLLSLRALHLRGYV |
| Q9NR97 | LDLSFNYIKGSYPQHINISRNFSKLLSLRALHLRGYVFQEL |
| Q9NR97 | RGYVFQELREDDFQPLMQLPNLSTINLGINFIKQIDFKLFQ |
| Q9NR97 | LSTINLGINFIKQIDFKLFQNFSNLEIIYLSENRISPLVKD |
| Q9NR97 | IYLSENRISPLVKDTRQSYANSSSFQRHIRKRRSTDFEFDP |
| Q9NR97 | NSIFFIGPNQFENLPDIACLNLSANSNAQVLSGTEFSAIPH |
| Q9NR97 | FSAIPHVKYLDLTNNRLDFDNASALTELSDLEVLDLSYNSH |
| Q9NR97 | SYNSHYFRIAGVTHHLEFIQNFTNLKVLNLSHNNIYTLTDK |
| Q9NR97 | IAGVTHHLEFIQNFTNLKVLNLSHNNIYTLTDKYNLESKSL |
| Q9NR97 | LDILWNDDDNRYISIFKGLKNLTRLDLSLNRLKHIPNEAFL |
| Q9NR97 | LNLPASLTELHINDNMLKFFNWTLLQQFPRLELLDLRGNKL |
| Q9NR97 | LSEVSSLKHLDLSSNLLKTINKSALETKTTTKLSMLELHGN |
| Q17598 | LAIYSTTVPKSAMNIVCATINASTVKSSDPCHESWPEILTR |
| Q17598 | GAFAFEHVQDIYTLNFFHDGNTSFIYSIIDYFLALFPIITL |
| Q9D563 | IIDTERLERSSQRMRDQGPANRSQQEPLSSHSGLWRTCRVQ |

| | |
|---|---|
| Q9D563 | WRTCRVQSSCTPLMNPFWQENVTVSDSSRQLLTMHGTFVIL |
| Q92545 | RASFSSREADNHTAFIRIKTNASDSTEFIILPVEVEVTTAP |
| Q9H813 | IPPLTSPGQPGDMNCTTQRINYTDPFSNQTVKSALIVQGPR |
| Q9H813 | GQPGDMNCTTQRINYTDPFSNQTVKSALIVQGPREVKKREL |
| Q9H330 | SSLWIWTLVVGYVLTVSFKWNASTERYLRAVSIPVWIILLF |
| Q9H330 | LTAKVHQESVHMIEVTSNLINETLANHPEWANWLPEAQVVQ |
| Q9H330 | YQYGREWITHKLHKILGDKVNNTAVIEKQVLELWDRLYHSW |
| Q9H330 | VIEKQVLELWDRLYHSWFVKNVTHSGRHKGQKLHVSRQNSW |
| P61228 | ILCEDPVPLYGPGQTGQQPANESFRNEGKCLKMGGYRAEDV |
| Q8BXN9 | LFRNTTILLKFDGEPCDQSLNITWFLKSADCYNEIYNFKAD |
| Q8BXN9 | FSGDFTHRLPLLGEKQEAKENATNVTFTGDKIAMHEPLQTW |
| Q8BXN9 | DFTHRLPLLGEKQEAKENATNVTFTGDKIAMHEPLQTWQDA |
| Q8NBN3 | LFRNTTIFLKFDGEPCDLSLNITWYLKSADCYNEIYNFKAE |
| Q8NBN3 | KLKEKRGLSGKYQTSSKLFQNCSELFKTQTFSGDFMHRLPL |
| Q8NBN3 | FSGDFMHRLPLLGEKQEAKENGTNLTFIGDKTAMHEPLQTW |
| Q8NBN3 | DFMHRLPLLGEKQEAKENGTNLTFIGDKTAMHEPLQTWQDA |
| Q9HD45 | YLWTYKKLEIGFNGNRIVDVNLTSEGKVKLVPNTKIQMSYS |
| Q9HD45 | CCICFFVILPLNLVGTILGRNLSGQPNFPCRVNAVPRPIPE |
| Q9ET30 | YLWTYKKLEIGFNGNRIVDVNLTSEGKVKLVPNTKIQMSYS |
| Q9ET30 | CCICFFVILPLNLVGTILGRNLSGQPNFPCRVNAVPRPIPE |
| Q11069 | FVVIPEWLADSRTQFGDDRYNKTKAIKVMPPAVRARADELS |
| Q11069 | LPVGFVIASKTPSKSCGPFGNQSFFYSVITDVLHENLDKTL |
| Q9BVK6 | EGRFTFTSHTPGEHQICLHSNSTKFSLFAGGMLRVHLDIQV |
| Q7Z7H5 | EGRFTFTSHTPGDHQICLHSNSTRMALFAGGKLRVHLDIQV |
| Q5FWI3 | DFSRGLNVRVIDQDTARVLENEKFDTHEYHNESRRLQEFLR |
| Q5FWI3 | IDQDTARVLENEKFDTHEYHNESRRLQEFLRAQEPGRIVAI |
| Q5FWI3 | RYSSAIGFLMKNSWQTTPRNNVSLVKFGPQVSLNVFFGKPG |
| Q5FWI3 | RFQSPGKAEIQRGDPSIISVNGTDFTFRSAGALLLIVDACS |
| P43298 | VTRIQIGHSGLQGTLSPDLRNLSELERLELQWNNISGPVPS |
| P43298 | TLSPDLRNLSELERLELQWNNISGPVPSLSGLASLQVLMLS |
| P43298 | QSVEIDNNPFKSWEIPESLRNASALQNFSANSANVSGSLPG |
| P43298 | NNPFKSWEIPESLRNASALQNFSANSANVSGSLPGFLGPDE |
| P43298 | EIPESLRNASALQNFSANSANVSGSLPGFLGPDEFPGLSIL |
| P43298 | QVQSLWLNGQKLTGDITVLQNMTGLKEVWLHSNKFSGPLPD |
| P43298 | NSFTGPVPASLLSLESLKVVNLTNNHLQGPVPVFKSSVSVD |
| P43298 | ESWKGNDPCTNWIGIACSNGNITVISLEKMELTGTISPEFG |
| P43298 | TISPEFGAIKSLQRIILGINNLTGMIPQELTTLPNLKTLDV |
| Q6ZUK4 | QYCSIQAEGTSQNTSRKEDFNQTLTSNEQTSRADDLIETAK |
| Q6ZUK4 | AEGTSQNTSRKEDFNQTLTSNEQTSRADDLIETAKVFVNNL |
| Q0P6H9 | KYSAVRRDGSFHYVHSTPFGNYSFICVDATVNPGPKRPYNF |
| Q0P6H9 | ILIIFRYRGYPELKEPSGFINLTSFSLHVLSKINIFYYSVL |

| | |
|---|---|
| Q96JJ7 | LLYELGDTGKLVALAVIDEKNTSVEHTRLKSIIQEVARDYR |
| Q96JJ7 | NDYINTLLMDELTVPTVVVLNTSNQQYFLLDRQIKNVEDMV |
| Q9Y275 | EAPAVTAGLKIFEPPAPGEGNSSQNSRNKRAVQGPEETVTQ |
| Q9Y275 | DELSLVTLFRCIQNMPETLPNNSCYSAGIAKLEEGDELQLA |
| O43508 | QDGAQAGVDGTVSGWEEARINSSSPLRYNRQIGEFIVTRAG |
| O43557 | QLIQERRSHEVNPAAHLTGANSSLTGSGGPLLWETQLGLAF |
| P06804 | NGLPLISSMAQTLTLRSSSQNSSDKPVAHVVANHQVEEQLE |
| P01374 | SLLWRANTDRAFLQDGFSLSNNSLLVPTSGIYFVYSQVVFS |
| P43488 | EDGQLFISSYKNEYQTMEVQNNSVVIKCDGLYIIYLKGSFF |
| P23510 | KKEKGFILTSQKEDEIMKVQNNSVIINCDGFYLISLKGYFS |
| P23510 | IINCDGFYLISLKGYFSQEVNISLHYQKDEEPLFQLKKVRS |
| P23510 | VRSVNSLMVASLTYKDKVYLNVTTDNTSLDDFHVNGGELIL |
| P23510 | SLMVASLTYKDKVYLNVTTDNTSLDDFHVNGGELILIHQNP |
| O35305 | KCLLHKVCDAGKALVAVDPGNHTAPRRCACTAGYHWNSDCE |
| O35305 | CLLGFFSDVFSSTDKCKPWTNCTLLGKLEAHQGTTESDVVC |
| Q92956 | LSKCLQCQMCDPAMGLRASRNCSRTENAVCGCSPGHFCIVQ |
| Q92956 | GGTESQDTLCQNCPPGTFSPNGTLEECQHQTKCSWLVTKAG |
| P20333 | RPGTETSDVVCKPCAPGTFSNTTSSTDICRPHQICNVVAIP |
| P20333 | TSSTDICRPHQICNVVAIPGNASMDAVCTSTSPTRSMAPGA |
| O75509 | GQVLTCDKCPAGTYVSEHCTNTSLRVCSSCPVGTFTRHENG |
| O75509 | LPCAALTDRECTCPPGMFQSNATCAPHTVCPVGWGVRKKGT |
| O75509 | RPEHMETHEVPSSTYVPKGMNSTESNSSASVRPKVLSSIQE |
| O75509 | ETHEVPSSTYVPKGMNSTESNSSASVRPKVLSSIQEGTVPD |
| O75509 | SSASVRPKVLSSIQEGTVPDNTSSARGKEDVNKTLPNLQVV |
| O75509 | SIQEGTVPDNTSSARGKEDVNKTLPNLQVVNHQQGPHHRHI |
| P25445 | SSKCRRCRLCDEGHGLEVEINCTRTQNTKCRCKPNFFCNST |
| P25445 | EINCTRTQNTKCRCKPNFFCNSTVCEHCDPCTKCEHGIIKE |
| Q5JTV8 | YQGQDEKLWKRSQTFLEKHLNSSHPRSQPAILLLTAARDAE |
| Q8BYU6 | QSSFLWQRGRKFLQKHLNASNPSEPATIIFTAAREGKETLK |
| O14656 | GLNSDYVHLFVATLHFPHASNITLYKDQLQLWIRGNVSACA |
| O14656 | FPHASNITLYKDQLQLWIRGNVSACARSIFIFDEMDKMHAG |
| Q68G38 | GLNSDYVHLFVATLHFPHASNITLYKDQLQMWIRGNVSACA |
| Q68G38 | FPHASNITLYKDQLQMWIRGNVSACARSIFIFDEMDKMHAG |
| Q9ER38 | TAWLCRFQDCCSGGGDCRISNNLTGLESDLRVRLHGQHLAS |
| Q9ER38 | AWLCRFQDCCSGGGDCRISNNLTGLESDLRVRLHGQHLASK |
| Q95NU5 | VLSFHGYTGSGKNYVAEIIANNTFRLGLRSTFVQHIVATND |
| Q95NU5 | YESGYPREQLRLEAFERELMNFSYNEKGGLQMSELISNHLI |
| P00750 | GISYRGTWSTAESGAECTNWNSSALAQKPYSGRRPDAIRLG |
| P00750 | SSEFCSTPACSEGNSDCYFGNGSAYRGTHSLTESGASCLPW |
| P00750 | LKEAHVRLYPSSRCTSQHLLNRTVTDNMLCAGDTRSGGPQA |
| Q13641 | QCPALCECSEAARTVKCVNRNLTEVPTDLPAYVRNLFLTGN |

| | |
|---|---|
| Q13641 | AVLPAGAFARRPPLAELAALNLSGSRLDEVRAGAFEHLPSL |
| Q13641 | RNLTHLESLHLEDNALKVLHNGTLAELQGLPHIRVFLDNNP |
| Q9Z0L0 | TVLPAGAFARQPPLADLEALNLSGNHLKEVCAGAFEHLPGL |
| Q9Z0L0 | RNLTHLESLHLEDNALKVLHNSTLAEWHGLAHVKVFLDNNP |
| Q8BWC0 | YYVFAMIGINLFRGVIVPPGNSSLVPDNNSAVCGSFEQLGY |
| Q8BWC0 | GINLFRGVIVPPGNSSLVPDNNSAVCGSFEQLGYWPNNFDD |
| P40225 | PPTTAVPSRTSLVLTLNELPNRTSGLLETNFTASARTTGSG |
| P40225 | TSLVLTLNELPNRTSGLLETNFTASARTTGSGLLKWQQGFR |
| P40225 | TGSGLLKWQQGFRAKIPGLLNQTSRSLDQIPGYLNRIHELL |
| P40225 | QTSRSLDQIPGYLNRIHELLNGTRGLFPGPSRRTLGAPDIS |
| O14773 | VTGTVGLHLGVTPSVIRKRYNLTSQDVGSGTSNNSQACAQF |
| O14773 | PSVIRKRYNLTSQDVGSGTSNNSQACAQFLEQYFHDSDLAQ |
| O14773 | RGRAGIEASLDVQYLMSAGANISTWVYSSPGRHEGQEPFLQ |
| O14773 | SSPGRHEGQEPFLQWLMLLSNESALPHVHTVSYGDDEDSLS |
| O14773 | EEAVTKFLSSSPHLPPSSYFNASGRAYPDVAALSDGYWVVS |
| O89023 | QVGTVSLHLGVTPSVLRQRYNLTAKDVGSGTTNNSQACAQF |
| O89023 | PSVLRQRYNLTAKDVGSGTTNNSQACAQFLEQYFHNSDLTE |
| O89023 | RGRAGIEASLDVEYLMSAGANISTWVYSSPGRHEAQEPFLQ |
| O89023 | SSPGRHEAQEPFLQWLLLLSNESSLPHVHTVSYGDDEDSLS |
| O89023 | EEAVAQFLKSSSHLPPSSYFNASGRAYPDVAALSDGYWVVS |
| Q18936 | ERESQLSVKTSPEWIDELFPNVSFIDSPTHQVIRGFCRDVF |
| Q18936 | VLYPPIIYFTFLRKKLKTPPNNTSGLFMYRKHKDEQGSGDL |
| Q18936 | LYPPIIYFTFLRKKLKTPPNNTSGLFMYRKHKDEQGSGDLP |
| O15533 | EGAVAFAAWDDDEPWGPWTGNGTFWLPRVQPFQEGTYLATI |
| O88856 | LGYDPYANPPNYGNPDPIVINNTHRVLKGDYKTPANLKGYF |
| O77081 | DGDESLPFHQLTSVRSDDGYNRTSPFIFIGGVPRSGTTLMR |
| O00300 | DECLYCSPVCKELQYVKQECNRTHNRVCECKEGRYLEIEFC |
| O00300 | QAGTPERNTVCKRCPDGFFSNETSSKAPCRKHTNCSVFGLL |
| O00300 | CPDGFFSNETSSKAPCRKHTNCSVFGLLLTQKGNATHDNIC |
| O00300 | APCRKHTNCSVFGLLLTQKGNATHDNICSGNSESTQKCGID |
| O00300 | KIIQDIDLCENSVQRHIGHANLTFEQLRSLMESLPGKKVGA |
| P07204 | GSQCVEHDCFALYPGPATFLNASQICDGLRGHLMTVRSSVA |
| P07204 | GCGDPKRLGPLRGFQWVTGDNNTSYSRWARLDLNGAPLCGP |
| P07204 | CGDPKRLGPLRGFQWVTGDNNTSYSRWARLDLNGAPLCGPL |
| P07204 | CVEPVDPCFRANCEYQCQPLNQTSYLCVCAEGFAPIPHEPH |
| P07204 | VCAEGFAPIPHEPHRCQMFCNQTACPADCDPNTQASCECPE |
| P15306 | GCDDPVHLGPLRGFQWVTGDNHTSYSRWARPNDQTAPLCGP |
| P15306 | CRAPPGTSEGHWAWEATGAWNCSVENGGCEYLCNRSTNEPR |
| P15306 | WEATGAWNCSVENGGCEYLCNRSTNEPRCLCPRDMDLQADG |
| P15306 | ICAPGFAPKPDEPHKCEMFCNETSCPADCDPNSPTVCECPE |
| P82179 | ALIITWSAVAVVMFDLVDYKNFSASSLSKIGSDPLKLVHDA |

| | |
|---|---|
| P82179 | EMKHLKEEKVSTRKESLQSHNVTKAEKPARVSREDLEDVSA |
| Q28820 | ALIITWSAVAVVMFDLVDYKNFSASSIAKMGSDPLKLVHDA |
| Q28820 | IEKSEKEIKVPARRESHQLQNVTKAEKPARGSKEGFEDVPA |
| Q9W2M2 | FAIDALDTLEHEFEFFVNNHNVTVKNHSLCVYRDSSSGPRP |
| Q9W2M2 | LDTLEHEFEFFVNNHNVTVKNHSLCVYRDSSSGPRPESYRE |
| Q9W2M2 | ESGMDFSSRWFISPTGTNDGNRSALSTTSIVPVDLNAYLYW |
| Q9W2M2 | QKPRDYYTPTNLSPLWVKAFNISESEKISASVMAYIERNKL |
| Q9W2M2 | APMQYILVEGLNNLNTPEAKNMSLKWATRWVKTNFAAFSKD |
| P02789 | SCHTAVGRTAGWVIPMGLIHNRTGTCNFDEYFSEGCAPGSP |
| P02787 | GGFVYIAGKCGLVPVLAENYNKSDNCEDTPEAGYFAIAVVK |
| P02787 | TAVGRTAGWNIPMGLLYNKINHCRFDEFFSEGCAPGSKKDS |
| P02787 | KDKEACVHKILRQQQHLFGSNVTDCSGNFCLFRSETKDLLF |
| Q921I1 | INHCKFDEFFSQGCAPGYEKNSTLCDLCIGPLKCAPNNKEE |
| P19134 | INHCRFDEFFRQGCAPGSQKNSSLCELCVGPSVCAPNNREG |
| O77698 | TVFENLPEKADRDQYELLCLNNTRAPVDAFKECHLAQVPSH |
| O77698 | DGKEDLIWKLLSKAQEKFGKNKSGSFQLFGSPPGQRDLLFK |
| O77698 | SCHTAVDRTAGWNIPMGLIANQTGSCAFDEFFSQSCAPGAD |
| O77698 | GYTGAFRCLAEDVGDVAFVKNDTVWENTNGESTADWAKNLN |
| Q9TUM0 | TVFESLPAKADRDQYELLCPNNTRKPVDAFQECHLARVPSH |
| Q9TUM0 | VWCAVGSDEQLKCQEWSRQSNQSVVCATASTTEDCIALVLK |
| Q9TUM0 | RSKLCALCAGNEEGQNKCVPNSSERYYGYTGAFRCLAENVG |
| Q9TUM0 | KNTEQWAKDLKLGDFELLCLNGTRKPVTEAESCHLAVAPNH |
| P24627 | TVFENLPEKADRDQYELLCLNNSRAPVDAFKECHLAQVPSH |
| P24627 | CAVGPEEQKKCQQWSQQSGQNVTCATASTTDDCIVLVLKGE |
| P24627 | SCHTAVDRTAGWNIPMGLIVNQTGSCAFDEFFSQSCAPGAD |
| P24627 | GYTGAFRCLAEDVGDVAFVKNDTVWENTNGESTADWAKNLN |
| P02788 | TGLRRTAGWNVPIGTLRPFLNWTGPPEPIEAAVARFFSASC |
| P02788 | SCHTAVDRTAGWNIPMGLLFNQTGSCKFDEYFSQSCAPGSD |
| P02788 | MDKVERLKQVLLHQQAKFGRNGSDCPDKFCLFQSETKNLLF |
| Q13507 | FLGLLVFNASDRFEGITTLPNITVTDYPKQIFRVKTTQFTW |
| Q9Y210 | FLGLLVMNAADRFEGTKLLPNETSTDNAKQLFRMKTSCFSW |
| Q9Y210 | FIARFMAFWHASKAQSIIDANDTLKDLTKVTLGDNVKYYNL |
| Q8R4D5 | QVPSDVDSTTYDFSHCTFSGNESKPLCVELDEHNLPRFPEW |
| Q7Z2W7 | QVPSDVDGTTYDFAHCTFTGNESKPLCVELDEHNLPRFPEW |
| O35433 | YLVFLFGFSTAVVTLIEDGKNNSLPMESTPHKCRGSACKPG |
| Q9NQA5 | LYMICFTTCCVYRPLKFRGGNRTHSRDITILQQKLLQEAYE |
| Q15661 | QFYTAQIGADIALLELEEPVNVSSHVHTVTLPPASETFPPG |
| Q15661 | GNTRRDSCQGDSGGPLVCKVNGTWLQAGVVSWGEGCAQPNR |
| P01222 | IPTEYTMHIERRECAYCLTINTTICAGYCMTRDINGKLFLP |
| P01224 | IPTEYMMHVERKECAYCLTINTTICAGYCMTRDFNGKLFLP |
| P01223 | IPTEYMMHVERKECAYCLTINTTVCAGYCMTRDVNGKLFLP |

| | |
|---|---|
| P16473 | LKLIETHLRTIPSHAFSNLPNISRIYVSIDVTLQQLESHSF |
| P16473 | SRIYVSIDVTLQQLESHSFYNLSKVTHIEIRNTRNLTYIDP |
| P16473 | ESHSFYNLSKVTHIEIRNTRNLTYIDPDALKELPLLKFLGI |
| P16473 | EITDNPYMTSIPVNAFQGLCNETLTLKLYNNGFTSVQGYAF |
| P16473 | ETLTLKLYNNGFTSVQGYAFNGTKLDAVYLNKNKYLTVIDK |
| P16473 | HCCAFKNQKKIRGILESLMCNESSMQSLRQRKSVNALNSPL |
| P40689 | NDNSEPDRWVFKEPTKNIYRNLSGLAESHEDTTPGIFHGDF |
| P40689 | PWEAFTGGWFPDNAAKKLGINTSFIQGDYSYVLVRVVRFRE |
| P40689 | PVHQPLEPDVRSRMDQLQIGNITSAVRFMEDVGTHYVNSYT |
| P40689 | YVNSYTTGNSLYQVFVYSRKNYSMIKERIKSKGLNGLSKLD |
| P40689 | YNYFAPWFAAHLGQIRSASANATVERWARRKLQYEYYVVKY |
| P40689 | RKLQYEYYVVKYVTLLKLHGNSTLLRSLDSLLGNDAILQLD |
| O60635 | LVVPAIKKDYGSQEDFTQVWNTTMKGLKCCGFTNYTDFEDS |
| O60635 | EDFTQVWNTTMKGLKCCGFTNYTDFEDSPYFKENSAFPPFC |
| O60635 | FEDSPYFKENSAFPPFCCNDNVTNTANETCTKQKAHDQKVE |
| O60635 | FKENSAFPPFCCNDNVTNTANETCTKQKAHDQKVEGCFNQL |
| Q9CQ88 | QFGISCSCLAINRNTQADVINASWSVLSNSTRHELERSFDC |
| Q9CQ88 | LAINRNTQADVINASWSVLSNSTRHELERSFDCCGLFNLTT |
| Q9CQ88 | VLSNSTRHELERSFDCCGLFNLTTLRLQDDTSCSAVCKTKS |
| O14817 | VGLTNAWSIIQTDFRCCGVSNYTDWFEVYNATRVPDSCCLE |
| O14817 | IQTDFRCCGVSNYTDWFEVYNATRVPDSCCLEFSESCGLHA |
| P19075 | LQVATGILGAVFKSKSDRIVNETLYENTKLLSATGESEKQF |
| O70401 | FRHEIKNSFKSNYENALKEYNSTGDYRSEAVDKIQSTLHCC |
| Q8BJU2 | LGENTVPDRCCMENSQGCGRNSTTPLWRTGCYEKVKLWFDD |
| P35441 | RNKGCSSSTNVLLTLDNNVVNGSSPAIRTNYIGHKTKDLQA |
| P35441 | HCQNSVTICKKVSCPIMPCSNATVPDGECCPRCWPSDSADD |
| P35441 | ICGEDTDLDGWPNENLVCVANATYHCKKDNCPNLPNSGQED |
| P35441 | YWDTNPTRAQGYSGLSVKVVNSTTGPGEHLRNALWHTGNTP |
| P07996 | RNKGCSSSTSVLLTLDNNVVNGSSPAIRTNYIGHKTKDLQA |
| P07996 | HCQNSVTICKKVSCPIMPCSNATVPDGECCPRCWPSDSADD |
| P07996 | ICGEDTDLDGWPNENLVCVANATYHCKKDNCPNLPNSGQED |
| P07996 | YWDTNPTRAQGYSGLSVKVVNSTTGPGEHLRNALWHTGNTP |
| P35442 | DGTRHVVSLEDVGLADSQWKNVTVQVAGETYSLHVGCDLID |
| P35442 | RVSNDNQFLWELIGGPPKTRNMSACWQDGRFFAENETWVVD |
| P35442 | GPPKTRNMSACWQDGRFFAENETWVVDSCTTCTCKKFKTIC |
| P35442 | DGGWSHWSPWSSCSVTCGVGNITRIRLCNSPVPQMGGKNCK |
| P35442 | CSSFPDGSWSCGSCPVGFLGNGTHCEDLDECALVPDICFST |
| P35442 | ICGEDSDLDGWPNLNLVCATNATYHCIKDNCPHLPNSGQED |
| P35442 | YWEDQPTRAYGYSGVSLKVVNSTTGTGEHLRNALWHTGNTP |
| P02766 | YWKALGISPFHEHAEVVFTANDSGPRRYTIAALLSPYSYST |
| P07309 | YWKTLGISPFHEFADVVFTANDSGHRHYTIAALLSPYSYST |

| | |
|---|---|
| P55955 | SLVVCLLLAACALECTARLQNVTVKGVAVCNKKRLANVEVQ |
| P34500 | GFLDSDDKMASGKADSHGEFNLSGSTKEITGIEPYLVVFHD |
| Q20332 | TKKSYQISTAQISQFQEAALNTTVHLKNLEDTVHVESKKTK |
| Q20332 | TTVHLKNLEDTVHVESKKTKNATIVQQIDQILTNITDEIDI |
| Q20332 | VESKKTKNATIVQQIDQILTNITDEIDIIAKNGKLFQAKHG |
| Q20332 | HDNLPLPVARHVALLNNIPANKSQLDRLMEVAFNSSAAIVN |
| Q20332 | LLNNIPANKSQLDRLMEVAFNSSAAIVNSSSAVGDDITKML |
| Q20332 | NKSQLDRLMEVAFNSSAAIVNSSSAVGDDITKMLKLAGAVS |
| Q20332 | SSSSCYVFHDDVVNFYYGTCNQSVAGMSIYMLSILLLGVFL |
| Q9C0H2 | YGNGETSDGIHRATYSLRHANRTVAGVQDRVWDTAVGLNHT |
| Q9C0H2 | HANRTVAGVQDRVWDTAVGLNHTAEPSLQTLERQLAGRPEP |
| Q9C0H2 | TVPWEQPATKDPLLRVQEVLNGTEVNLQHLTALVDCRSLHL |
| P01739 | QNVQQSPESLIVPEGARTSLNCTFSDSASQYFWWYRQHSGK |
| P01738 | YSGDPVVQGVNGFEAEFSKSNSSFHLRKASVHWSDSAVYFC |
| P01733 | NNVPIDDSGMPEDRFSAKMPNASFSTLKIQPSEPRDSAVYF |
| Q19907 | AWLFRTYELQADIKRRSVFGNTTNLVRRQLAERWIEMHKDA |
| Q19907 | LVRRQLAERWIEMHKDAVLRNDSALRFRRAAEAVEWLLDEL |
| Q19907 | DSALRFRRAAEAVEWLLDELNLSDHIRDLSEETPWTWTGAM |
| Q18120 | KINQLQIKRQRRLMTAEEEYNRTAKVLTTFQETLGIVPADM |
| Q96J42 | TESLKSPKVNCEERNITGLENFTLKILNMSQDLMDFLNPNG |
| Q96J42 | KVNCEERNITGLENFTLKILNMSQDLMDFLNPNGSDCTLVL |
| Q96J42 | ENFTLKILNMSQDLMDFLNPNGSDCTLVLFYTPWCRFSASL |
| Q96J42 | PMARFNHTDRTLETLKIFIFNQTGIEAKKNVVVTQADQIGP |
| Q6P6J9 | TDSLKSPKVNCEERNVTGLENFTLKILNMSQDLMDFLNPNG |
| Q6P6J9 | KVNCEERNVTGLENFTLKILNMSQDLMDFLNPNGSDCTLVL |
| Q6P6J9 | ENFTLKILNMSQDLMDFLNPNGSDCTLVLFYTPWCRFSASL |
| Q6P6J9 | PMARFNHTDRTLETLKIFIFNQTGIEAKKNVVVTQADQMGP |
| Q17688 | NVEELTEEGMPFLIYFRDPDNKTTDKVFGEAVARELYDQRS |
| P55144 | LKLMGAPVKMTVSQGQPVKLNCSVEGMEDPDIHWMKDGTVV |
| P55144 | SVEGMEDPDIHWMKDGTVVQNASQVSISISEHSWIGLLSLK |
| P55144 | IYWWRGLTKVGGPAPSPSVLNVTGVTQRTEFSCEARNIKGL |
| P55144 | GLATSRPAIVRLQAPPAAPFNTTVTTISSYNASVAWVPGAD |
| P55144 | RLQAPPAAPFNTTVTTISSYNASVAWVPGADGLALLHSCTV |
| P55144 | AVVVPVPPFTCLLRNLAPATNYSLRVRCANALGPSPYGDWV |
| P55144 | IPEDPGEGPLGPYKLSWVQENGTQDELMVEGTRANLTDWDP |
| P55144 | LSWVQENGTQDELMVEGTRANLTDWDPQKDLILRVCASNAI |
| O62053 | SRLVLLLAVSVALVSAIAVGNRSSHLKKSKECSSDGHTERC |
| O62053 | GHTERCEYTGDLTVKTDSTCNHSTYIMTKVTPPNESPSNGV |
| O45944 | AIISGSFSSISEEWKAVCECNLSKLNNHAKTGNCKTTALWK |
| O45944 | NHAKTGNCKTTALWKVTSDTNCTASEYLKITVFPANDDPLN |
| Q22957 | ILFLGSTYGASVATEKLKASNCTKTEGFQAIACVLRMADFA |

| | |
|---|---|
| Q22957 | DAVVYVAKDFADCSEKLENKNSTCYQNWDPFPDAIDEETDE |
| O45160 | VMVFVGSAHCASQIEKLKSENCTEADKFLEMSCQLFDKSCT |
| O45160 | RKRKLKLIRNYCDGIVYVTQNFTECDKKLESKNSTCYSAWD |
| O45160 | DGIVYVTQNFTECDKKLESKNSTCYSAWDPFPENFEEELDE |
| O45160 | RKQKLNLIRTYCDSIVFISNNFTDCDAKLERKNSTCYTDWD |
| O45160 | DSIVFISNNFTDCDAKLERKNSTCYTDWDPFPENYEEELDE |
| O16406 | TSFLKLAIIGTVLLGVAHGANLTAAEKETYCELRSAIQTFL |
| O16406 | EFKKTCDSLKNCFAITRCGPNSTMDEAELFRMVEKNCEAVV |
| O01454 | TVLLGVANGASVATASAKGSNCTVQDGYAALMCLVRLSDFA |
| O01454 | TKLKEFKRSCDSLHSCYSNLNCTTKSDDEKDKYVESIKQYC |
| P22309 | PFQREDVKESFVSLGHNVFENDSFLQRVIKTYKKIKKDSAM |
| P22309 | VGGINCLHQNPLSQEFEAYINASGEHGIVVFSLGSMVSEIP |
| P22309 | GKIPQTVLWRYTGTRPSNLANNTILVKWLPQNDLLGHPMTR |
| P35503 | LYFETEHFLKKFFRSMAMLNNMSLVYHRSCVELLHNEALIR |
| P35503 | LVYHRSCVELLHNEALIRHLNATSFDVVLTDPVNLCAAVLA |
| P35503 | IGGINCANRKPLSQEFEAYINASGEHGIVVFSLGSMVSEIP |
| P35503 | GKIPQTVLWRYTGTRPSNLANNTILVKWLPQNDLLGHPMTR |
| P22310 | GFFETEHLLKRYSRSMAIMNNVSLALHRCCVELLHNEALIR |
| P22310 | LALHRCCVELLHNEALIRHLNATSFDVVLTDPVNLCGAVLA |
| P22310 | IGGINCANGKPLSQEFEAYINASGEHGIVVFSLGSMVSEIP |
| P22310 | GKIPQTVLWRYTGTRPSNLANNTILVKWLPQNDLLGHPMTR |
| Q9HAW9 | RGHEVVVVMPEVSWQLGKSLNCTVKTYSTSYTLEDLDREFM |
| Q9HAW9 | IGGINCHQGKPLPMEFEAYINASGEHGIVVFSLGSMVSEIP |
| Q9HAW9 | GKIPQTVLWRYTGTRPSNLANNTILVKWLPQNDLLGHPMTR |
| O60656 | RGHEVVVVMPEVSWQLGRSLNCTVKTYSTSYTLEDLDREFK |
| O60656 | IGGINCHQGKPLPMEFEAYINASGEHGIVVFSLGSMVSEIP |
| O60656 | GKIPQTVLWRYTGTRPSNLANNTILVKWLPQNDLLGHPMTR |
| P06133 | FVQSSGENGVVVFSLGSMVSNTSEERANVIASALAKIPQKV |
| P16662 | IQRGHEVTVLASSASILFDPNNSSALKIEIYPTSLTKTELE |
| P16662 | QRGHEVTVLASSASILFDPNNSSALKIEIYPTSLTKTELEN |
| P16662 | FVQSSGENGVVVFSLGSMVSNMTEERANVIASALAQIPQKV |
| P36537 | VQRGHEVTVLASSASILFDPNDSSTLKLEVYPTSLTKTEFE |
| P36537 | FVQSSGENGVVVFSLGSMVSNMTEERANVIATALAKIPQKV |
| P36537 | FWIEFVMRHKGAKHLRVAAHNLTWFQYHSLDVIGFLLACVA |
| P54855 | ELVQRGHEVTVLTSSASTLVNASKSSAIKLEVYPTSLTKNY |
| P54855 | FVQSSGENGIVVFSLGSMISNMSEESANMIASALAQIPQKV |
| P54855 | FWIEFVMRHKGAKHLRVAAHNLTWIQYHSLDVIAFLLACVA |
| Q9XU84 | CMPEGYQLTEWEYALKFWNINGSSSHSFESCYGTTKNFVES |
| Q9XU84 | TANEFQLTEGAIKTALSSEPNSTVVDQIFDLVYSGTNQVLS |
| Q18081 | IADELANAGHEVVNFEPDFLNLTDKFVPCKKCRRWPVTGLN |
| Q18081 | FVKIMEKGKGVILFSLGTIANTTNLPPTIMENLMKITQKFK |

| | |
|---|---|
| Q21706 | SKSHLISNGRIADELAKAGHNVTLLEIDFLGIVDSTKSAKL |
| Q21706 | EAEMKKGDKGVIYFSLGTIANTSTIDKKVMESFLEIVKKFP |
| Q10941 | ASEMSKGKEGVIYFSLGTIANTTKIDSKVMRTVLDIVKKFP |
| Q22295 | YSKLLDNYDGAKHAKVWRLHNVTEAYDTKLGTLANVMENSH |
| Q22295 | LNLFRKEVSSDFPSIAEIIRNVSLVLVNTDEIFDLPRSYSS |
| Q22295 | PRSYSSKFVYVGMLEAGKDENVTLPKKQDDYFKKGKSGSVF |
| Q22295 | KWIDFVLKYETSEHFDLESNNLSIIEHNHLDLFFYLCIISL |
| Q6RJQ3 | AVKGHRLMNGLQYRLPYATWNFSQLHLGQIFSLTFNVSTDT |
| Q6RJQ3 | PYATWNFSQLHLGQIFSLTFNVSTDTAGMYECVLRNYSHGL |
| Q6RJQ3 | FSLTFNVSTDTAGMYECVLRNYSHGLIMQRFVILTQLETLS |
| P42849 | NCKKENPISHMTTPPVKIMLNTTGPQYYICTVGDHCRVGQK |
| Q09274 | TGDRKGSCFAAFKGKIEIDTNNTAGFMNLHTSRCLCQLDTV |
| Q09274 | DRDFTLHYDNTFGNCYTFNYNRTAEVASHRAGANYGLRVLL |
| Q09274 | AEVASHRAGANYGLRVLLYANVSEYLPTTEAVGFRITVHDK |
| Q09274 | VIVEACGCADPMYPVAEMFGNNTKPCQAVNMDQRECLRNTT |
| Q09274 | FGNNTKPCQAVNMDQRECLRNTTLWLGELYSKGKEAIIPDC |
| P34374 | KVRSQFAYKAKISLEARSVRNDSTVNDPQSKSFIRFMSAKQ |
| P34374 | QGNDGTTIYESNHLGKRLKVNETKSFNLTQNANFYTLPTSS |
| P34374 | TIYESNHLGKRLKVNETKSFNLTQNANFYTLPTSSVSSAVH |
| P34374 | HNDYFNIMTFSKNTFLLDGCNGTNGLLQATMRNKKALRRKM |
| P34374 | YRERLYLPRPEIFAEPVPITNQSFAVMNKMASRRKIRLQKS |
| P34374 | QKSEARSRMFVTTVSYPVIVNETFMGVAAVNIPLTEVAQKS |
| P34374 | MDWMGDKRWRLHPHWRYCFLNDTDTHMSKEEAFEIYAQQMS |
| P34374 | KNNLIHLAFFATPSGMIRYYNLTLQDYDYIDPYWSIFEHIG |
| P34374 | DDRYYRRAVRMKDTIMFDVSNNSKIWYKSETQLTGYGLNEN |
| P34374 | NNSKIWYKSETQLTGYGLNENLTMLGQAFKAIYLDKAVLGV |
| P34374 | FYFSNKDGKNRPGTTHLVNGNRSERPCKMNAKCSVKMEASF |
| Q06561 | LNIRDPAPPQRGAAPQIDPPNQTVNVNDPAQFRCWVPGQPR |
| Q06561 | VYTVGQPAYLSCIGKTETKPNQSVVWTKEEGDLPSGSRVEQ |
| Q06561 | QSFQGCVYEILINSQDVDLQNLSSSGDISSCEESQFPVEED |
| Q06561 | SEEIVVGDVYSTQEPNNICANSTCGMNGQCVPRNMTHYTCE |
| Q06561 | EPNNICANSTCGMNGQCVPRNMTHYTCECKLYYDGPTCSLF |
| Q22271 | TRTELLARSKKLVRLTNLMSNFTAESWKMLNNAQHGVSNMD |
| Q26261 | VIRASDGSLIMSAARLSDSGNYTCEATNVANSRKTDPVEVQ |
| P35456 | LLATTCVPASQGLQCMQCESNQSCLVEECALGQDLCRTTVL |
| P35456 | WQDDRELEVVTRGCAHSEKTNRTMSYRMGSMIISLTETVCA |
| P35456 | DYTRGCGSLPGCPGTAGFHSNQTFHFLKCCNYTHCNGGPVL |
| P35456 | GCPGTAGFHSNQTFHFLKCCNYTHCNGGPVLDLQSFPPNGF |
| P35456 | PVLDLQSFPPNGFQCYSCEGNNTLGCSSEEASLINCRGPMN |
| P35456 | INCRGPMNQCLVATGLDVLGNRSYTVRGCATASWCQGSHVA |
| P35456 | CATASWCQGSHVADSFPTHLNVSVSCCHGSGCNSPTGGAPR |

| | |
|---|---|
| Q03405 | WEEGEELELVEKSCTHSEKTNRTLSYRTGLKITSLTEVVCG |
| Q03405 | RHLRGCGYLPGCPGSNGFHNNDTFHFLKCCNTTKCNEGPIL |
| Q03405 | GCPGSNGFHNNDTFHFLKCCNTTKCNEGPILELENLPQNGR |
| Q03405 | PILELENLPQNGRQCYSCKGNSTHGCSSEETFLIDCRGPMN |
| Q03405 | IDCRGPMNQCLVATGTHEPKNQSYMVRGCATASMCQHAHLG |
| P80607 | STPFFFNTLYDPYREGADFVRGYPFSLREGAHTAVSHGLWL |
| P00749 | SMYNDPQFGTSCEITGFGKENSTDYLYPEQLKMTVVKLISH |
| P07911 | FITTAATDTSEARWCSECHSNATCTEDEAVTTCTCQEGFTG |
| P07911 | FTGDGLTCVDLDECAIPGAHNCSANSSCVNTPGSFSCVCPE |
| P07911 | GLTCVDLDECAIPGAHNCSANSSCVNTPGSFSCVCPEGFRL |
| P07911 | RMAETCVPVLRCNTAAPMWLNGTHPSSDEGIVSRKACAHWS |
| P07911 | CCLWDASVQVKACAGGYYVYNLTAPPECHLAYCTDPSSVEG |
| P07911 | IDEDCKSNNGRWHCQCKQDFNITDISLLEHRLECGANDMKV |
| P07911 | DWVSVVTPARDGPCGTVLTRNETHATYSNTLYLADEIIIRD |
| P98119 | GVTYRGTWSTAESRVECINWNSSLLTRRTYNGRMPDAFNLG |
| P98119 | LKEGHVRLYPSSRCAPKFLFNKTVTNNMLCAGDTRSGEIYP |
| P10379 | VLIQILAPAKAAEHSVFTHKNASSVLGQLVTSSTLVWESYD |
| P10379 | PFRYELRDIKLDRLRTDIQENMTELVTRKLENLEDKYSTVE |
| P10379 | TEKRSENKAVHTKLWPGTAINVTLRGNYVTLEAPYSGKLFA |
| P10379 | VRKSYLKEVKLEFSPVYWIENGTLVPTTTTTTTTSTSTTTH |
| P10379 | EKTLHDSPSSNELNSHEAPENMSSDPGKDVALAGFGVNAAG |
| O61460 | RFSEDIDNWKNIGRLARSNSNMTTETLGMEIDSDTKTIRIA |
| O61460 | QLVYFRPQVTGPKETDMVRMNGSCIPNASKKIPGVDLIGLC |
| O61460 | PQVTGPKETDMVRMNGSCIPNASKKIPGVDLIGLCMSTGSG |
| O61460 | FEVEVRPAIVKKRTFETRHVNMTYTTFIGLNPETVYQFRVR |
| O61460 | LGRGLMSSPSSNEVEESQFLNQTGSALLIIIALILIVIAVA |
| Q9R1Q9 | EKLGASPLHVDLATLKELKLNASLPALLLIRLPYTASSGLM |
| Q9R1Q9 | GRQLLQTQVASPAIHPPVSYNDTAPRILFWAQNFSVAYKDE |
| Q9R1Q9 | AIHPPVSYNDTAPRILFWAQNFSVAYKDEWKDLTSLTFGVE |
| Q9R1Q9 | VAYKDEWKDLTSLTFGVENLNLTGSFWNDSFAMLSLTYEPL |
| Q9R1Q9 | KDLTSLTFGVENLNLTGSFWNDSFAMLSLTYEPLFGATVTF |
| Q9R1Q9 | RFYPVSARYWFAMERLEIHSNGSVAHFNVSQVTGPSIYSFH |
| Q9R1Q9 | RYWFAMERLEIHSNGSVAHFNVSQVTGPSIYSFHCEYVSSV |
| Q9R1Q9 | VTNVPSVWQMTLHNFQIQAFNVTGEQFSYASDCAGFFSPGI |
| Q15904 | EKLGASPLHVDLATLRELKLNASLPALLLIRLPYTASSGLM |
| Q15904 | GRQLLQKQPVSPVIHPPVSYNDTAPRILFWAQNFSVAYKDQ |
| Q15904 | VIHPPVSYNDTAPRILFWAQNFSVAYKDQWEDLTPLTFGVQ |
| Q15904 | VAYKDQWEDLTPLTFGVQELNLTGSFWNDSFARLSLTYERL |
| Q15904 | EDLTPLTFGVQELNLTGSFWNDSFARLSLTYERLFGTTVTF |
| Q15904 | RLYPVSARHWFTMERLEVHSNGSVAYFNASQVTGPSIYSFH |
| Q15904 | RHWFTMERLEVHSNGSVAYFNASQVTGPSIYSFHCEYVSSL |

| | |
|---|---|
| Q6EMK4 | LDLSQNQIASLPSGVFQPLANLSNLDLTANRLHEITNETFR |
| Q6EMK4 | QPLANLSNLDLTANRLHEITNETFRGLRRLERLYLGKNRIR |
| Q6EMK4 | AQLRPEDLAGLAALQELDVSNLSLQALPGDLSGLFPRLRLL |
| Q6EMK4 | LQRYLQGSSVQLRSLRLTYRNLSGPDKRLVTLRLPASLAEY |
| Q6EMK4 | LVTLRLPASLAEYTVTQLRPNATYSVCVMPLGPGRVPEGEE |
| P19320 | APEIFWSKKLDNGNLQHLSGNATLTLIAMRMEDSGIYVCEG |
| P19320 | FSWRTQIDSPLSGKVRSEGTNSTLTLSPVSFENEHSYLCTV |
| P19320 | QVELYSFPRDPEIEMSGGLVNGSSVTVSCKVPSVYPLDRLE |
| P19320 | GETILENIEFLEDTDMKSLENKSLEMTFIPTIEDTGKALVC |
| P19320 | PRDTTVLVSPSSILEEGSSVNMTCLSQGFPAPKILWSRQLP |
| P19320 | APKILWSRQLPNGELQPLSENATLTLISTKMEDSGVYLCEG |
| P29533 | DSTLKERETVKELQVYISPRNTTISVHPSTRLQEGGAVTMT |
| P29533 | MTCSSEGLPAPEIFWGRKLDNEVLQLLSGNATLTLIAMRME |
| P29533 | APEIFWGRKLDNEVLQLLSGNATLTLIAMRMEDSGVYVCEG |
| P29533 | PEDPVIKMSGPLVHGRPVTVNCTVPNVYPFDHLEIELLKGE |
| P29533 | PKETTIWVSPSPILEEGSPVNLTCSSDGIPAPKILWSRQLN |
| P29533 | LTCSSDGIPAPKILWSRQLNNGELQPLSENTTLTFMSTKRD |
| P29533 | APKILWSRQLNNGELQPLSENTTLTFMSTKRDDSGIYVCEG |
| P02854 | RAKLSPGDVFVIPAGHPVAINASSDLNLIGLGINAENNERN |
| Q91132 | DKGIYTPGSPVLYRVFSMDHNTSKMNKTVIVEFQTPEGILV |
| Q91132 | TPGSPVLYRVFSMDHNTSKMNKTVIVEFQTPEGILVSSNSV |
| Q91132 | PDLVSLGTWRIVAKYEHSPENYTAYFDVRKYVLPSFEVRLQ |
| Q91132 | LTFYNAQLQEKANVCNKFHLNVSVENIHLNAMGAKGALMLK |
| P16612 | MRCAGCCNDEALECVPTSESNVTMQIMRIKPHQSQHIGEMS |
| P49767 | PCVSVYRCGGCCNSEGLQCMNTSTSYLSKTLFEITVPLSQG |
| P49767 | LFEITVPLSQGPKPVTISFANHTSCRCMSKLDVYRQVHSII |
| P49767 | QVHSIIRRSLPATLPQCQAANKTCPTNYMWNNHICRCLAQE |
| O43915 | PCVNVFRCGGCCNEESLICMNTSTSYISKQLFEISVPLTSV |
| O43915 | LFEISVPLTSVPELVPVKVANHTGCKCLPTAPRHPYSIIRR |
| O43915 | DRCECVCKTPCPKDLIQHPKNCSCFECKESLETCCQKHKLF |
| P15692 | MRCGGCCNDEGLECVPTEESNITMQIMRIKPHQGQHIGEMS |
| P35968 | SVSLDLPRLSIQKDILTIKANTTLQITCRGQRDLDWLWPNN |
| P35968 | NTTLQITCRGQRDLDWLWPNNQSGSEQRVEVTECSDGLFCK |
| P35968 | VTECSDGLFCKTLTIPKVIGNDTGAYKCFYRETDLASVIYV |
| P35968 | SPFIASVSDQHGVVYITENKNKTVVIPCLGSISNLNVSLCA |
| P35968 | ITENKNKTVVIPCLGSISNLNVSLCARYPEKRFVPDGNRIS |
| P35968 | DVVLSPSHGIELSVGEKLVLNCTARTELNVGIDFNWEYPSS |
| P35968 | TRSDQGLYTCAASSGLMTKKNSTFVRVHEKPFVAFGSGMES |
| P35968 | YLGYPPPEIKWYKNGIPLESNHTIKAGHVLTIMEVSERDTG |
| P35968 | HTIKAGHVLTIMEVSERDTGNYTVILTNPISKEKQSHVVSL |
| P35968 | FQGGNKIEVNKNQFALIEGKNKTVSTLVIQAANVSALYKCE |

| | |
|---|---|
| P35968 | QFALIEGKNKTVSTLVIQAANVSALYKCEAVNKVGRGERVI |
| P35968 | QPTEQESVSLWCTADRSTFENLTWYKLGPQPLPIHVGELPT |
| P35968 | IHVGELPTPVCKNLDTLWKLNATMFSNSTNDILIMELKNAS |
| P35968 | PTPVCKNLDTLWKLNATMFSNSTNDILIMELKNASLQDQGD |
| P35968 | KLNATMFSNSTNDILIMELKNASLQDQGDYVCLAQDRKTKK |
| P35968 | VVRQLTVLERVAPTITGNLENQTTSIGESIEVSCTASGNPP |
| P35968 | IEVSCTASGNPPPQIMWFKDNETLVEDSGIVLKDGNRNLTI |
| P35968 | FKDNETLVEDSGIVLKDGNRNLTIRRVRKEDEGLYTCQACS |
| P35916 | LCLGLLDGLVSGYSMTPPTLNITEESHVIDTGDSLSISCRG |
| P35916 | CEGTDARPYCKVLLLHEVHANDTGSYVCYYKYIKARIEGTT |
| P35916 | LLVNRKDAMWVPCLVSIPGLNVTLRSQSSVLWPDGQEVVWD |
| P35916 | DIQLLPRKSLELLVGEKLVLNCTVWAEFNSGVTFDWDYPGK |
| P35916 | VPERRSQQTHTELSSILTIHNVSQHDLGSYVCKANNGIQRF |
| P35916 | EASTGTYTLALWNSAAGLRRNISLELVVNVPPQIHEKEASS |
| P35916 | QDAVNPIESLDTWTEFVEGKNKTVSKLVIQNANVSAMYKCV |
| P35916 | WTEFVEGKNKTVSKLVIQNANVSAMYKCVVSNKVGQDERLI |
| P35916 | VLLSCQADSYKYEHLRWYRLNLSTLHDAHGNPLLLDCKNVH |
| P35916 | KHCHKKYLSVQALEAPRLTQNLTDLLVNVSDSLEMQCLVAG |
| P35916 | LSVQALEAPRLTQNLTDLLVNVSDSLEMQCLVAGAHAPSIV |
| P35916 | VREEDAGRYLCSVCNAKGCVNSSASVAVEGSEDKGSMEIVI |
| P35917 | LCLGLLQGLANGYSMTPPTLNITEDSYVIDTGDSLSISCRG |
| P35917 | CEGTEARPYCKVLLLAQTHANNTGSYHCYYKYIKARIEGTT |
| P35917 | LLVNRKDSMWVPCLVSIPGLNITLRSQSSALHPDGQEVLWD |
| P35917 | DIQLYPKKSMELLVGEKLVLNCTVWAEFDSGVTFDWDYPGK |
| P35917 | VPERRSQQTHTELSSILTIHNVSQNDLGPYVCEANNGIQRF |
| P35917 | EASAGVYTLALWNSAAGLRQNISLELVVNVPPHIHEKEASS |
| P35917 | QDAVNPIESLDSWTEFVEGKNKTVSKLVIQDANVSAMYKCV |
| P35917 | WTEFVEGKNKTVSKLVIQDANVSAMYKCVVVNKVGQDERLI |
| P35917 | SEPSEDPLEGQSVRLSCRADNYTYEHLRWYRLNLSTLHDAQ |
| P35917 | VRLSCRADNYTYEHLRWYRLNLSTLHDAQGNPLLLDCKNVH |
| P35917 | KHCHKKYLSVQALEAPRLTQNLTDLLVNVSDSLEMRCPVAG |
| P35917 | LSVQALEAPRLTQNLTDLLVNVSDSLEMRCPVAGAHVPSIV |
| P35917 | VREEDAGRYLCSVCNAKGCVNSSASVAVEGSEDKGSMEIVI |
| P05690 | NENDRVVVQLTVEPMSRQYVNITMQSPMERIELKNVQVPRV |
| P02845 | ASFVYSHMKSLSKSRLPFMYNISSACNIALKLLSPKLDSMS |
| P02845 | EDIQAKLKRILGIDSMFKVANKTRHPKNRPSKKGNTVLAEF |
| P02845 | QVKQARNKDASSSSRSSKSSNSSKRSSSKSSNSSKRSSSSS |
| P02845 | SSSRSSKSSNSSKRSSSKSSNSSKRSSSSSSSSSSSSRSSS |
| P02845 | SSSSSKSSSHHSHSHHSGHLNGSSSSSSSSRSVSHHSHEHH |
| P02845 | QLVVYADTDSVRPRVQVFVTNLTDSSKWKLCADASVRNAHK |
| P02845 | LKARCSVSYNKIKTFNEVKFNYSMPANCYHILVQDCSSELK |

| | |
|---|---|
| P02845 | MHPVNGQVKLLVDGAESPTANISLISAGASLWIHNENQGFA |
| P55155 | NENDRVVVQLTVEPMSRQYVNITMQSPIERVELKNVQVPRV |
| Q9N4J2 | NEDRRVVLQLSVEPLSRQYMNMTIQTPEQEVELKNVRIPRV |
| P18948 | CKDCQQTLEQDKMSSTVLNYNITGTSSSFLINSVELRSQHL |
| P18948 | SRKNMIRATINVEPRQRLTVNMTIETPMETTVLERVELPFR |
| P18947 | NEDRRVVLQLSVEPLSRQYMNMTIQTPEQEVELKNVRIPRV |
| P38435 | DHADMLKQYATCLSRLLPKYNVTEPQIYFDIWVSINDRFQQ |
| P38435 | VFIADFPGLHLENFVSEDLGNTSIQLLQGEVTVELVAEQKN |
| P0C6E3 | KSASSVTLASFANWRESVLPNRTSHDNAQLLTAIVFNRGVI |
| P0C6E3 | LQVAITMAHEIGHNLGMGHDNNSCTCGGYSCIMLPALSDQP |
| P0C6E3 | GGYSCIMLPALSDQPSKYFSNCSYIQYRDFIMNQDPQCILN |
| P30403 | SYETLKAFGKWRESDLIKHVNHSNAQFLMDMKFIKNIIGKA |
| P30403 | GSSECIMSSHISDPPSKYFSNCSYYQFWKYIENQNPQCILN |
| P0DM89 | SLNILVALVYLEIWSKEDLINVTSAAKDTLASFGNWRATDL |
| P0DM89 | NCGTCVMAPTISDQISKLFSNCSKNDYENFLTLYKPQCILN |
| P0DM89 | YCYNGKCPIMLNQCISFYGSNATVAPDICFNYNLKGEGNFY |
| P0DM90 | SLNILVALVYLEIWSKEDLINVTSAAKDTLASFGNWRATDL |
| P0DM90 | NCGTCVMAPTISDQISKLFSNCSKNDYENFLTLYKPQCILN |
| O93523 | GDYPCIMGPTISNEPSKFFSNCSYIQCWDFIMKENPQCILN |
| Q7LZ61 | FNKIFIELVIIVDHSMAKKCNSTATNTKIYEIVNSANEIFN |
| Q7LZ61 | PLNIHVTLIGVEFWCDRDLINVTSSADETLNSFGEWRASDL |
| Q7LZ61 | MAHELSHNLGMYHDGKNCICNDSSCVMSPVLSDQPSKLFSN |
| Q7LZ61 | NDSSCVMSPVLSDQPSKLFSNCSIHDYQRYLTRYKPKCIFN |
| P0C7B0 | GDYACIMGATISHEPSTFFSNCSYIQCWDFIMDHNPECIVN |
| Q8JIR2 | RDLLKRKSHDNAQLLTAIDFNGTIIGLAHVASMCDPKCSTG |
| Q8JIR2 | VAVIMAHEMGHNLGIRHDRENCTCHANSCIMSAVISDQPSK |
| Q8JIR2 | HANSCIMSAVISDQPSKYFSNCSHVQYWNYINDDEPQCILN |
| Q8JIR2 | YCYNGNCPIMYHQCYALWGANATVAKDSCFEDNQKGNDYGY |
| P20164 | ALVYLEIWSKQNKITVQSASNVTLDLFGDWRESVLLKQRSH |
| P20164 | GGFPCIMSPMISDPPSELFSNCSKAYYQTFLTDHKPQCILN |
| P20164 | YCYNGKCPIMFYQCYFLFGSNATVAEDDCFNNNKKGDKYFY |
| P20164 | QEDVKCGRLFCDNKKYPCHYNYSEDLDFGMVDHGTKCADGK |
| D3TTC2 | VEVGEECDCGSPADCQSACCNATTCKLQHEAQCDSEECCEK |
| P82942 | TVLLPRKRNDNAQLLTGINLNGTAVGIAYPGSLCTQRSVFV |
| D3TTC1 | GIDFNGNTVGRAYIGSLCKTNESVAIVQDYNRRISLVASTI |
| D3TTC1 | PTDSLQRNGHPCQNNQSYCYNGTCPTLTNQCITLLGPHFTV |
| Q9DGB9 | KKYVKLFLVADYIMYLKYGRNLTAVRTRMYDIVNVITPIYH |
| Q90282 | GDYACIMRPEISPEPSTFFSNCSYFECWDFIMNHNPECILN |
| Q58CQ9 | RASSLDTFIAAVYEHAVILPNATLVPVSPEEALAVMNRNLD |
| Q58CQ9 | SASKQGAHIIVTPEDGIYGFNFTRESIYPYLEDIPDPQVNW |
| Q58CQ9 | CLAKDNSIYIVANIGDKKSCNASDPQCPPDGRYQYNTDVVF |

| | |
|---|---|
| Q58CQ9 | NLFLNEDQFNAPKEPEVVTFNTTFGKFGIFTCFDILFHDPA |
| Q58CQ9 | EEGKLLLAQLDSHPHPTPVVNWTSYASGVEAHSVGNQEFTG |
| Q58CQ9 | FTGIIFFDEFTFLELKEIGGNYTVCQRDLCCHLSYKMSEKR |
| O95497 | RASCQDTFTAAVYEHAAILPNATLTPVSREEALALMNRNLD |
| O95497 | NNRNRFGQTPVQERLSCLAKNNSIYVVANIGDKKPCDTSDP |
| O95497 | NLFMGENQFNVPKEPEIVTFNTTFGSFGIFTCFDILFHDPA |
| O95497 | LASNIHYPSKKMTGSGIYAPNSSRAFHYDMKTEEGKLLLSQ |
| O95497 | EEGKLLLSQLDSHPSHSAVVNWTSYASSIEALSSGNKEFKG |
| O95497 | FKGTVFFDEFTFVKLTGVAGNYTVCQKDLCCHLSYKMSENI |
| O95498 | QVGTQDSFIAAVYEHAVILPNKTETPVSQEDALNLMNENID |
| O95498 | AWAMGMGVNLLVANTHHVSLNMTGSGIYAPNGPKVYHYDMK |
| O95498 | IKPFPVQKNTFRGFISRDGFNFTELFENAGNLTVCQKELCC |
| O95498 | FRGFISRDGFNFTELFENAGNLTVCQKELCCHLSYRMLQKE |
| O95498 | HGRRRREYWQVCTLLKCKTTNLTTCGRPVETASTRFEMFSL |
| O95498 | EIHLSPGKFEVLKDGRLVNKNGSSGPILTVSLFGRWYTKDS |
| Q9NFP1 | LSLSAWSDSLAGYVEIINSENASATDIIVFPESTLNSAGST |
| Q9NFP1 | GSTTFVPNPEDQINPCLSDPNATYYEEFLVTLSCAARNASK |
| Q9NFP1 | SDPNATYYEEFLVTLSCAARNASKYIVINLTEKQKCEDIPE |
| Q9NFP1 | EEFLVTLSCAARNASKYIVINLTEKQKCEDIPEDTRPCASN |
| Q9NFP1 | DRQGVVVSRYRKVHLYGEAKNSTFLPELITFETDFGVTFGH |
| Q9NFP1 | YVENYESELLKLDEGTSGAINRTICQGSFCCNFDIAWRSLG |
| Q9NFP1 | QGSFCCNFDIAWRSLGTATENGSYYSYRLGTYDGWRNENNV |
| P26332 | WIDAWKAKKALTGAETAEFRNETAGIAGKTGVTKLVEEALL |
| P26332 | KEEAKKVADETAKDGKTGNTNTTGSSNSFVISKTPLWLAVL |
| P02896 | EDKICSWHKEVKAGEKHCKFNSTKAKEKGVSVTQTQTAGGT |
| P26334 | NADKKCSYETETDGTKKCKFNATKAEKSGAPVTQAQTVGET |
| P81824 | IVVGGRPCKINVHRSLVLLYNSSSLLCSGTLINQEWVLTAA |
| P85109 | NFDDEQRRSPKEKYFFRCSNNFTKWDKDIMLIRLDSPVNNS |
| P85109 | SNNFTKWDKDIMLIRLDSPVNNSAHIAPLSLPSNPPSVGSV |
| P85109 | TTSPQEDLSDVPRCANINLFNFTVCRAAYPWLPATSRVLCA |
| P81176 | PNKDEQTRVPKEKFFCLSSKNYTLWDKDIMLIRLDSPVKNS |
| P81176 | KNYTLWDKDIMLIRLDSPVKNSTHIEPFSLPSSPPSVGSVC |
| Q9PSN3 | SFLCGGTLINQEWVLTAAHCNMSNIYIYLGMHNQSVQFDDE |
| Q9PSN3 | WVLTAAHCNMSNIYIYLGMHNQSVQFDDEERRYPKEKYLFR |
| Q9PSN3 | QFDDEERRYPKEKYLFRCSKNFTKWDKDIMLIRLNKPVRNS |
| Q9PSN3 | SPPIVGSVCRVMGWGTITSPNETLPDVPRCVNINLFNYTVC |
| Q9PSN3 | ITSPNETLPDVPRCVNINLFNYTVCRGVFPRLPERSRILCA |
| P09872 | LNKDALRRFPKEKYFCLNTRNDTIWDKDIMLIRLNRPVRNS |
| P09872 | NPPSVGSVCRIMGWGTITSPNATLPDVPHCANINILDYAVC |
| P26324 | GGDECNINEHRFLVAVYEGTNWTFICGGVLIHPEWVITAEH |
| P26324 | SEKFDDEQERYPKKRYFIRCNKTRTSWDEDIMLIRLNKPVN |

| | |
|---|---|
| P26324 | NKTRTSWDEDIMLIRLNKPVNNSEHIAPLSLPSNPPIVGSD |
| P26324 | INRRIDVLSDEPRCANINLHNFTMCHGLFRKMPKKGRVLCA |
| P04971 | ITTSEDTYPDVPHCANINLFNNTVCREAYNGLPAKTLCAGV |
| Q9I8X1 | LVIGGNECDINEHRFLVAFFNTTGFFCGGTLINPEWVVTAA |
| P20948 | AGKAACEKTAASIEDVFMKLNFSEPSAVVTTLDGTRVELQN |
| P20948 | LVAALQARAEHLEDAAFTIFNESVLETQIAWESSRPPSTDA |
| P20948 | ESVLETQIAWESSRPPSTDANTSQKGPLQRPEKSGESSHLP |
| P21614 | STSEDCMASELPEHTIKICQNLSKKNSKFEECCQENTPMNI |
| P04004 | DVFTMPEDEYTVYDDGEEKNNATVHEQVGGPSLTSDLQAQS |
| P04004 | PPAEEELCSGKPFDAFTDLKNGSLFAFRGQYCYELDEKAVR |
| P04004 | FKGSQYWRFEDGVLDPDYPRNISDGFDGIPDNVDAALALPA |
| P29788 | DVFTMPEDDYWSYDYVEEPKNNTNTGVQPENTSPPGDLNPR |
| P29788 | EFPEEELCSGKPFDAFTDLKNGSLFAFRGQYCYELDETAVR |
| P29788 | FKGSQYWRFEDGVLDPGYPRNISEGFSGIPDNVDAAFALPA |
| P04275 | GKRVSLSVYLGEFFDIHLFVNGTVTQGDQRVSMPYASKGLY |
| P04275 | FVARIDGSGNFQVLLSDRYFNKTCGLCGNFNIFAEDDFMTQ |
| P04275 | WALSSGEQWCERASPPSSSCNISSGEMQKGLWEQCQLLKST |
| P04275 | GRCELNCPKGQVYLQCGTPCNLTCRSLSYPDEECNEACLEG |
| P04275 | APGETVKIGCNTCVCQDRKWNCTDHVCDATCSTIGMAHYLT |
| P04275 | PQSCEERNLRENGYECEWRYNSCAPACQVTCQHPEPLACPV |
| P04275 | KVTLNPSDPEHCQICHCDVVNLTCEACQEPGGLVVPPTDAP |
| P04275 | MVLDVAFVLEGSDKIGEADFNRSKEFMEEVIQRMDVGQDSI |
| P04275 | EAQSKGDILQRVREIRYQGGNRTNTGLALRYLSDHSFLVSQ |
| P04275 | CPPSLVYNHCEHGCPRHCDGNVSSCGDHPSEGCFCPPDKVM |
| P04275 | AWVPDHQPCQICTCLSGRKVNCTTQPCPTAKAPTCGLCEVA |
| P04275 | VPHCERGLQPTLTNPGECRPNFTCACRKEECKRVSPPSCPP |
| P04275 | LPTLRKTQCCDEYECACNCVNSTVSCPLGYLASTATNDCGC |
| P04275 | NPCLINECVRVKEEVFIQQRNVSCPQLEVPVCPSGFQLSCK |
| P04275 | CKTSACCPSCRCERMEACMLNGTVIGPGKTVMIDVCTTCRC |
| P04275 | QCSCCSPTRTEPMQVALHCTNGSVVYHEVLNAMECKCSPRK |
| Q14508 | LVSGTGAEKTGVCPELQADQNCTQECVSDSECADNLKCCSA |
| P56695 | TAILLFCWFYVYRSEGMKVYNSTLTWQQYGFLCGPRAWKET |
| P56695 | FLGDWMRCLYGEAYPSCSSGNTSTAEEELCRLKQLAKHPCH |
| P49893 | LLLLCCSGICGAIQWLGLTVNGSRVAWNESEHCRLLDGLVP |
| P49893 | GICGAIQWLGLTVNGSRVAWNESEHCRLLDGLVPDQSQLCK |
| P49893 | VNAAKQTKLTCQMTLSDMRWNCSSVENAPSFTPDLSKGTRE |
| P49893 | PDYCTKNPKLGSYGTQDRLCNKTSVGSDSCNLMCCGRGYNA |
| Q93097 | GEGAREWIRECQHQFRHHRWNCTTLDRDHTVFGRVMLRSSR |
| Q93097 | DYLRRRYDGAVQVMATQDGANFTAARQGYRRATRTDLVYFD |
| P56704 | AEGIKIGIQECQHQFRGRRWNCTTVHDSLAIFGPVLDKATR |
| P56704 | PNFCEPNPETGSFGTRDRTCNVSSHGIDGCDLLCCGRGHNA |

| | |
|---|---|
| P28026 | RETSFVHAISSAGVMYTLTRNCSMGDFDNCGCDDSRNGRIG |
| P28026 | SSVAGSELIFLEDSPDYCLKNISLGLQGTEGRECLQSGKNL |
| P28026 | KNISLGLQGTEGRECLQSGKNLSQWERRSCKRLCTDCGLRV |
| P22725 | GEGAKTGIKECQYQFRHRRWNCSTVDNTSVFGRVMQIGSRE |
| P22725 | GIKECQYQFRHRRWNCSTVDNTSVFGRVMQIGSRETAFTYA |
| P22725 | NSPTTQDLVYIDPSPDYCVRNESTGSLGTQGRLCNKTSEGM |
| P22725 | PDYCVRNESTGSLGTQGRLCNKTSEGMDGCELMCCGRGYDQ |
| P09615 | VKGANLAISECQHQFRNRRWNCSTRNFSRGKNLFGKIVDRG |
| P09615 | LAISECQHQFRNRRWNCSTRNFSRGKNLFGKIVDRGCRETS |
| P09615 | PSFCEKNLRQGILGTHGRQCNETSLGVDGCGLMCCGRGYRR |
| P91573 | FKEIVCVKDLQWTSGLVEINNGTHRTLKTECCSYEGMSDAK |
| P78423 | ATFCHLTVLLAGQHHGVTKCNITCSKMTSKIPVALLIHYQQ |
| P47989 | ALKIPTSKIYISETSTNTVPNTSPTAASVSADLNGQAVYAA |
| Q8L5C6 | KGVPVSLSIGGYGTGYSLPSNRSALDLFDHLWNSYFGGSKP |
| Q95333 | LICACAWGHPKSLNQREDVRNCSTSPPYLPVTAVNTTAQLT |
| Q95333 | QREDVRNCSTSPPYLPVTAVNTTAQLTALREQMLTQNLSAY |
| Q95333 | VTAVNTTAQLTALREQMLTQNLSAYIIPDTDAHMSEYIGEC |
| Q95333 | YNPFFYSYTLLTDSSIRLFANKSRFSSETLQYLNSSCNSSM |
| Q95333 | SSIRLFANKSRFSSETLQYLNSSCNSSMCVQLEDYSQIRDS |
| Q95333 | LFANKSRFSSETLQYLNSSCNSSMCVQLEDYSQIRDSIQAY |
| Q6YDN9 | AFYLSSTNNEHDEIDFEFLGNRTGQPVILQTNVFTGGKGNR |
| Q965Q8 | SRAITPSCKAKLQLEACQLKNGTFTINFPENQCPNHDSRLI |
| Q965Q8 | GFLYFGLEFGHECFCGNDVSNATAVDDVECRAYKCPGNENS |
| Q965Q8 | LQVIRDSMKIEKFKDWDYIINFSESDFPILPISDFERLITV |
| Q965Q8 | KIETTDGATFEFLIHRLSHVNLTENEEKLVEHGYLLRAVSF |
| Q965Q8 | GTKFEWKEELCREYMGFVTDNDTLHTRLQWHPTEHVKKVGD |
| Q965Q8 | PYDSVFGGQFDSWNVGKKLSNLTTCSNFFVDIISPSSPDDA |
| B3A0S5 | CHTLVWHSQLPSWVANGNWNNQTLQAVMRDHINAVMGRYRG |
| Q9BPN8 | DQMPEKWIQELTGAKLTSFINVTSGECIGNVSKPDLFKISR |
| Q9BPN8 | ELTGAKLTSFINVTSGECIGNVSKPDLFKISRFESIIGADT |
| Q9BPN8 | EVVGGVNAQKWVTCIDGATANDTKVLLEVRYAGEETIRPAQ |
| Q9BPN8 | QSPFSNPLLLSIRLAELPNFNSTVALNHVSVELDRYELPVG |
| Q9BPN8 | YELPVGNEAAVVHGVWCKNRNDTVLVLKPLDEYAAILNYFD |
| Q9BPN8 | LVLKPLDEYAAILNYFDPSTNRSDVVEVLYSKTKKVIHIAG |
| Q9BPN8 | AGDSFENGVKLFKATPKRYKNGTDYILHDFNYGYEFTMSHG |
| Q9BPN8 | HGACQSFSPLDDSSEDVLVKNGTDHFIMNNMEWLLVDPGLR |
| Q9BPN8 | SRLNMKTVQLSNCYDEQKFANNTLMVQIKDKTLSDLSQVGL |
| Q9BPN8 | EMIVRVAEKTEKKPGNVVGYNYTTELTTAEIFKLMNDSMTL |
| Q9BPN8 | NVVGYNYTTELTTAEIFKLMNDSMTLDKMPIEVLKNNGVKE |
| Q20762 | LTAGQTIDLHMYFPFYGGLYNYSTLSVNGYIGFATVLDQGP |
| Q20762 | AADFRQNSRSGRCQALFNGGNHTGLVPVDPTQDFKNTPKVL |

| | |
|---|---|
| Q20762 | MVRGRYMFRVDDVVRPAGCSNKTGGTYPMLIYPNIVNMLGE |
| Q20762 | YDWFKNPLPYTTMPLVWYPRNFTNPEMTQHMDQVRMNDDTL |
| Q20762 | CHDWYDEDGAQWNFIRDTETNSSCPCIERQAIADIGRFMPH |
| Q20762 | RVDFSLLGRYMAQQDLVQPTNATVVTGVVLEATGTDRVHVV |
| O44443 | GCGKGETLFGTSCTPHMCQHNGTIAVGKKEIECICPPPWDG |
| Q18594 | VGLPRGCVGTECNFAFSSISNGTHTEIEIFGNSVIDKTWLA |
| Q18594 | DEQTLYCKMAHRVEPIIDRFNVSKVEILMAKGTWMKGGLSY |
| Q18594 | ILMAKGTWMKGGLSYHGNTRNNTGIIDLSGESKHKRKNSAV |
| P90850 | LIQPDILEKIHTGRYSWSARNYSAFWGRSLSDGIKYRLGTL |
| P90850 | SVSTTAISSDRLAIISEGRINSTLSSQQLLSCNQHRQKGCE |
| O16887 | CSIQKKCVNLPVLSKLVDGKNISAEMYDDIDKYTDYGCIFT |
| Q18474 | FFAIMVTNPTVVQQVNQIEVNLTQSNPQFVKFVHNISHLHI |
| Q18474 | VNQIEVNLTQSNPQFVKFVHNISHLHIKLNTVELNETRLPI |
| Q18474 | FVKFVHNISHLHIKLNTVELNETRLPIVQKFLKELPKAMCN |
| Q18474 | NETRLPIVQKFLKELPKAMCNVSYSIPALFEGTAVMANTTL |
| Q18474 | AMCNVSYSIPALFEGTAVMANTTLYGKVNPMSSSVISSVHQ |
| Q18474 | VNPMSSSVISSVHQKIFSLLNNTEIPSVDVHDIFHPRMNIV |
| Q18474 | RMNIVELDNRKHEDLLQIMSNRTGDFKLNTGDFIKEIVLFN |
| Q18474 | NRTGDFKLNTGDFIKEIVLFNSTESGSFHEEIGRAVLAPCS |
| Q18474 | STESGSFHEEIGRAVLAPCSNFTLLRFKKPELISNRVPIFP |
| Q19040 | FLAEMFVSGVNGDCACYEANNVTGKSAPISNSYMTEDFSPC |
| Q19040 | ITIKYSQTGSGASMFILNINNGTFVTNVYYGSISTAAGLAP |
| Q19040 | EGDIDSALIGLADLAFTVNKNASTDTRNNVQRSVVLLTAEW |
| Q19040 | GDNVASKFTELGLNLLVVGYNLTDAETSQLLRTDRWYNAIN |
| Q19040 | NLTDAETSQLLRTDRWYNAINSSDSKITNVAAFVNPFYFNN |
| Q19040 | PPFGITNSVDGTYSWFQEPYNYTGPHGINGVWTDPFDGQTG |
| Q19077 | VGKNSVVAEEKDFDYCATMKNISTKEITYSGRTGATRVFEK |
| Q21874 | LTSGMTIDLYMFFPYYGGLYNYTTISVNGYLGFATVLDQGP |
| Q21874 | AQDFRGNSRSGRCRAVFNGGNHTGTVEVDPTQPYKNTPKVL |
| Q21874 | MVRGRYMFRVDDVVRPAGCSNKTGGTYPIMIYPNIVNMLGD |
| Q21874 | YEWYKNPMTNYLMPITWYPRNFTNPDILTNGNNMGVRISDD |
| Q21874 | TLYTYRVGFFKLAPINPNDANGTQLLPGLVSAPISLHWLWT |
| Q21874 | CHDWYDEDGALWNFIRDTETNTSCPCIETQALLDLGRFMPH |
| Q21874 | KVDFGLLGRYISQYELVQPTNATVITGIALEATGTERVIVM |
| O76565 | LIARDLETGNFPVVSVEFDLNNSAEALEEFPEDAFRDKISS |
| O76565 | SCVGTDDTLTIQFVILKKDTNSSALPFAIEDLIDAESIATR |
| Q9GUC9 | IAKHILMVNPDVVALQEVYANVTRNLTLMLGHPWVAVERNH |
| Q9GUC9 | ILMVNPDVVALQEVYANVTRNLTLMLGHPWVAVERNHEYPD |
| Q9GUC9 | RNHEYPDTAILTRHVLIPNTNLSTSGAVGVKIMLRTGFMIH |
| Q9GUC9 | NVVSDPGITWSTVNKFNPEWNYTIPEPQDRIDFLFYKGPVV |
| Q23307 | ENNEVITVQNAFRIKCLTEDNGSWKTEIIGCVTPDGTEINA |

| | |
|---|---|
| Q23307 | IPAGETGKIGGFDVKCEQHANGTITMQAANDPKSYDCTAKD |
| Q23307 | QGKIIGCHAENVGNTIGINQNVTSGDIVYSCTKDGSNYSFK |
| Q11101 | PVLCQYACSSELKFGTACSENKTSTKWYYDSKLLFCYPYKY |
| P34434 | APTTPGSDEDCDWGMQQRIDNCSEPIVHFLSQIERLNLKNM |
| P90893 | RLTASDGASIQETYPNLQVHNFTQKLDHFDPYNTKTWNQKY |
| P90893 | DPYNTKTWNQKYFYNPVFSRNNSIIFLMIGGEGPENGKWAA |
| P90893 | TAEGRNSLNNHFNLQPPFDANTTKLDINNFFGNIFNTYQGM |
| P90893 | FGNIFNTYQGMTQYTYDGQSNSTHSDKTVRKMCDIMTNATE |
| P90893 | GQSNSTHSDKTVRKMCDIMTNATETDVVMRVENLFLWFNQM |
| P90893 | VVMRVENLFLWFNQMEPASANLTVMPNSYWDVISQVGSGDL |
| P90893 | SQIMGGNKKSQNYYGGADFYNATNVVLPNGSLDPWHALGTY |
| P90893 | KSQNYYGGADFYNATNVVLPNGSLDPWHALGTYGTIKSQSL |
| P90893 | WHALGTYGTIKSQSLLPYLINGTAHCGDMYPSYDGEPGSLL |
| P34501 | AGRETQTNLGFDCPFFGFRFNYTMVYPMGMLSFGLPPFSAP |
| P34501 | RQMPGRVSQPGMVVDPWLLDNITRHIQDGYTGANGFRAEHA |
| P34501 | QVVLASDEIRTFAIFNYARLNWTSSNEAGGLDGFGGKQAAM |
| P34501 | AGGLDGFGGKQAAMAGFNGGNGTGWYGLPYSGEGRLWKLGY |
| P34501 | LTPGRWIHRVDEVIIPAGCTNASNGGMMTAPPWGPMHGGMA |
| P34501 | SNGGMMTAPPWGPMHGGMAINVSGPCLRPADSVKVNFENWQ |
| P34501 | FYVVNSERAPASVSLKDSVDNKTNRWYEPYAQELALGWQAM |
| P34501 | KTNRWYEPYAQELALGWQAMNLTWNTGARVDISLFGYWEDA |
| P34501 | KLMVQVRLEQPDDTLWHAHVNATVITGVAVQENDSSIVQVY |
| P34501 | DTLWHAHVNATVITGVAVQENDSSIVQVYARKPMRRWRYRT |
| P34501 | PHWKHQQFKHLDIRNPLQNMNQSEIVIMLKSGVGIRIFEGF |
| P34501 | IRIFEGFGMLDVMVTLPPSYNTTCRPGESLSSSLNAPRGQR |
| P34501 | PLLFAESDYRPVYWPQTIDMNASRVFTMEQVVSTCQNNPEC |
| P34501 | VVFSCKPKYYIHGDIERVCRNGTWSPGWWAWCRDRNLEYAL |
| Q21268 | GFPFYRNRAEQLAECWKAVPNDTNVLITHTPPLGYLDQFGD |
| P34572 | QKVGLLAKDINDIVASQVDSNSTCTKYGFTRQQQKKSHDDD |
| Q23570 | EALPNFEDYMEVVTCIQGKQNISMAAEVCFEGPTKLDRTKM |
| Q23570 | QENIVAQMAPEMDWAPWILINGTRYKEAEEDLWQFLCDRFI |
| P34639 | CYSRMIVVQNRTDNHNIDMTNSTLLLADEGNTYYIRMHDVK |
| P52716 | PGLLFKANFKSYSGYVDANANGTWKMHYMLTESRSNPDTDP |
| P52716 | AAQNYQALTNFFNVAQPKYTNRTFYLSGESYAGIYIPMLTD |
| P52716 | TLWSAYHGRVSEQNWADIKANCSKGADVDSFDFSQFTTSQN |
| P52716 | PQAWKDQKNTWEDCRMSIYNNYTLKYNTTNRFFNNIITNLT |
| P52716 | QKNTWEDCRMSIYNNYTLKYNTTNRFFNNIITNLTTDFRFL |
| P52716 | YNNYTLKYNTTNRFFNNIITNLTTDFRFLIYNGDVDTVCNY |
| P52716 | AGPSVQMISNFVWAPKNVVINYTSQDNFNPNIQLSDLVDSG |
| Q19948 | GSTGCVVTGNVLYANGIRLRNLTSSEQSELATYQTEVEQYK |
| Q17405 | AFLGHYLTKQNYEMMQFKSANESMTTCKNFTRSHKKHQDIV |

| | |
|---|---|
| Q17405 | KQNYEMMQFKSANESMTTCKNFTRSHKKHQDIVNEEDADEN |
| Q17405 | NFTRSHKKHQDIVNEEDADENASIKQPTKEELALPKNVQPV |
| Q17405 | LPKNVQPVWYDVSLSPKVGGNGTMGLAHVKLNIEEPTNKIV |
| Q17405 | KIQLSKEVTKRAKKSVDSGTNSTSEMPEGSGEEAMATTATT |
| Q17405 | TLNKVLDPDFVIGDLVASEVNTTSGITIRIWTRPEVKHSTE |
| Q17405 | AKYWFNWEVPLFLKSSGPVGNVSWLHEAFRLPLNTSDSIYL |
| Q17405 | KSSGPVGNVSWLHEAFRLPLNTSDSIYLNTDSNGVYRVNYE |
| Q17405 | LISDVFALANSGALPFETALNVTSYLPMETATVPWLIATRI |
| Q17405 | NEKLNELFVEGFLAPCQFSGNFSSDCSEVPGDLREKVYCNG |
| Q17405 | CSEVPGDLREKVYCNGVEFGNDTVFETVRELAEKEVDGAEK |
| Q17405 | NSLACFRDPRALRRLILDNLNSTSTVTLLLRKMNSRPVGKE |
| Q17405 | LRKMNSRPVGKEIATNWIIDNWSTVLKKKFKNDPETLNAIA |
| Q09288 | VTYRASPGHYRACEHDFFSGNTSAVQADPDMDPVEPFRIRK |
| Q09322 | CQKYVEKLAVVQSEMVACATNWSIPPVVCTKCFQNYINFKQ |
| Q09322 | INFKQFEYETKNLNNVYSLDNRTCSQVIYDNYLLSYSTDIS |
| Q09322 | EIWEKSRCDSCITIKWNFPQNKSEVSFSERTMQFQNRMYEW |
| Q09322 | FSERTMQFQNRMYEWRNCVVNYTSGGVLDDNLTNGSKICNL |
| Q09322 | RMYEWRNCVVNYTSGGVLDDNLTNGSKICNLCKTTFDELFG |
| Q09322 | EWRNCVVNYTSGGVLDDNLTNGSKICNLCKTTFDELFGYYW |
| Q09322 | YWKIYTTPDVDFCVDVETTMNDTIHLWDDVWKCAEKQDRNR |
| Q09606 | YAAFLYANPPMKNVMKFNLFNVTNPDEVKYLGAKPELIEVG |
| Q09606 | YGSQLNYIPEMKTFAYLSGYNNSYDENYWINTGYNDFNKLG |
| Q09606 | AGFRYKNQENVDYFPDWCDKNTTTSLSQCQKTANGTFLLPP |
| Q09606 | FPDWCDKNTTTSLSQCQKTANGTFLLPPGIFPLVCYPGHNA |
| Q09347 | TYQVEKRNDEHVLWRSGKCLNSTVNYRIGCKFDDPFETQFK |
| Q09365 | CITHCADRKSVKAAVRFQFANQSTAVPTYKVRRTFTRYSGM |
| Q09364 | CITHCADRKSVKVAARFQFANQSTAVPTYNVRRHYTRKSGV |
| Q19374 | HTPMTKFSPPGFPNADIFFKNETATATRTLKHRFAWALLLW |
| Q19374 | VLSDSQYSLFYDYVIGHKFTNQSGAGIWTPPGIAGIGYGYD |
| Q10011 | EVITIRDKGWLNAYATGIQMNISAAAYQLDGQPQVKIGDCT |
| Q10011 | FDLAADIVYGKSLCHPINIGNWTALLALNILYSSDASSTPG |
| Q10011 | FFHTYSTPYFVMSEKEGVMLNMSLAVDLFINPYAKTKQNIL |
| Q10011 | KQNILARLVVDTFSTVEPFLNHTRIHGRLQNSTITARVDFS |
| Q10011 | DTFSTVEPFLNHTRIHGRLQNSTITARVDFSNIGDIPKAFL |
| Q10011 | TAREAVRSVLGVGIPIPSYDNVTLADSSTIEVFDQFLRANI |
| Q10019 | SPSSSVYNASTLILPSKTTKNRSSNSSIDSGIRDEQLTPSP |
| Q10022 | VTKHVVGRLRPHFLDVCKLANDTCVTGDSHRYITDYTCTGP |
| P54002 | IHNNYGSHVEIYRSLNAELTNRTNFKNFHDPIRVCVGKEWH |
| P52717 | ASVVYIESPAGVGYSYATDGNITTNDDLTSLENYEAVKQFF |
| P52717 | MKHMLRGVAPAMAHFDELLKNQTKTSLYQFLKNKSQSQKPL |
| P52717 | AHFDELLKNQTKTSLYQFLKNKSQSQKPLKADVPCLNDTEM |

| | |
|---|---|
| P52717 | QFLKNKSQSQKPLKADVPCLNDTEMLSYMNNPKVRKAIHIP |
| O17861 | KCGFASFCDKHRAATHNQKINITVLIEALCPDCQNFLTKQL |
| Q10904 | MIYFPSVFQKKLGVVTDALTNKTIYTIVRNEDVEALQVFKF |
| P52714 | DYYDFMQFMKTTNKMDYLADNSTECGRLIEPLLGQFSETFD |
| P25311 | LQKAREDIFMETLKDIVEYYNDSNGSHVLQGRFGCEIENNR |
| P25311 | AREDIFMETLKDIVEYYNDSNGSHVLQGRFGCEIENNRSSG |
| P25311 | YNDSNGSHVLQGRFGCEIENNRSSGAFWKYYYDGKDYIEFN |
| P25311 | WTRAGEVQEPELRGDVLHNGNGTYQSWVVVAVPPQDTAPYS |
| Q64726 | YKDTTGSHTFQGMFGCEITNNRSSGAVWRYAYDGEDFIEFN |
| Q64726 | VQRAKAYLEEECPEMLKRYLNYSRSHLDRIDPPTVTITSRV |
| Q64726 | WNKANKKLAFEPERGVFPNGNGTYLSWAEVEVSPQDIDPFF |
| Q62005 | FEYSYDCGVRGMQLLVFPRPNQTVQFKVLDEFGNRFEVNNC |
| Q62005 | PNQTVQFKVLDEFGNRFEVNNCSICYHWVTSEAQEHTVFSA |
| Q62005 | GTHLPQERCQVASGHIPCMVNGSSKETCQQAGCCYDSTKEE |
| Q62005 | GPQGSITRDSAFRLHVRCIFNASDFLPIQASIFSPQPPAPV |
| Q62005 | TCSTTCDSGIARRRRSSGHHNITLRALDIVSSPGAVGFEDA |
| Q62005 | SPGAVGFEDAAKLEPSGSSRNSSSRMLLLLLAITLALAAGI |
| O54766 | FQYSYDCGVQGMQLLVFPRPNQTIQFKVLDEFGNRFEVNNC |
| O54766 | PNQTIQFKVLDEFGNRFEVNNCSICYHWVISEAQKPAVFSA |
| O54766 | GPQGSITRDGVFRLHVRCIFNASDFLPIRASIFSPQPPAPV |
| Q9BH10 | TLGNKIQHASVVDSLGLKMLNCTYVLDPEKLTLKAPYESCT |
| Q9BH10 | AGPKPQMGWTVTVGDGERAQNLTLQEALTQGYNLLIENQKM |
| Q9BH10 | PGQTIILSSRLICASDPVTCNATHMTLTIPEFPGKLKSVSF |
| Q9BH10 | KDYPVVRYLRQPIYLEVRVLNRTDPNIKLVLDDCWATSTMD |
| P20239 | RFDMEKWNPSVVDTLGSEILNCTYALDLERFVLKFPYETCT |
| P20239 | RDLISFSFPQLFSRLADENQNVSEMGWIVKIGNGTRAHILP |
| P20239 | SRLADENQNVSEMGWIVKIGNGTRAHILPLKDAIVQGFNLL |
| P20239 | IVQGFNLLIDSQKVTLHVPANATGIVHYVQESSYLYTVQLE |
| P20239 | GQKIVFSSHAICAPDLSVACNATHMTLTIPEFPGKLESVDF |
| P20239 | FEVYSHQTKPALNLDTLLVGNSSCQPIFKVQSVGLARFHIP |
| O54767 | RFDMEKWNPSLVDTFGNEISNCTYALDLEKFILKFPYETCT |
| O54767 | EDLISFSFPQLFSRLADENQNVSEMGWIIKIGNGTRVHTLP |
| O54767 | SRLADENQNVSEMGWIIKIGNGTRVHTLPLKDAIVQGFNLL |
| O54767 | IVQGFNLLIDSQKITLHVPANATGVAHYVQESSYLYTVQLK |
| O54767 | GQKITFSSQAICAPDLSVACNVTHMSLTIPEFPGKLKSVGF |
| O54767 | FEVYSHQTKPALNLESLLVGNSSCQPIFKVQSLGLARFHIP |
| O54767 | IIMDGCEYELDNYRTTFHAANSSAAHSGHYQRFDVKTFAFV |
| P79762 | VIIRTNPAVIPIECHYPRRENVSSNAIRPTWSPFNSALSAE |
| P21754 | VTDDALVYSTFLLHDPRPVGNLSIVRTNRAEIPIECRYPRQ |
| P21754 | SIVRTNRAEIPIECRYPRQGNVSSQAILPTWLPFRTTVFSE |
| P21754 | SHVPLRLFVDHCVATPTPDQNASPYHTIVDFHGCLVDGLTD |

| | |
|---|---|
| P21754 | KVPRPGPDTLQFTVDVFHFANDSRNMIYITCHLKVTLAEQD |
| Q07287 | TATPPALVVWDRRGRLHKLQNDSGCGTWVHKGPGSSMGVEA |
| Q07287 | GNTVTSRCTQDGHFSIAVSRNVTSPPLLWDSVHLAFRNDSE |
| Q07287 | VSRNVTSPPLLWDSVHLAFRNDSECKPVMETHTFVLFRFPF |
| Q07287 | PGPLTLELQIAKDERYGSYYNASDYPVVKLLREPIYVEVSI |
| Q07287 | ICVTTCPAARRRRSSDIHFQNGTASISSKGPMILLQATRDS |
| P97708 | SILRTNRVEVPIECRYPRQGNVSSHPIQPTWVPFSATVSSE |
| P97708 | QVPRPRPETLQFTVDVFHFANSSRNTVYITCHLKVAPANQI |
| P97708 | HLKVAPANQIPDKLNKACSFNKTSQSWLPVEGDADICDCCS |
| P97708 | SQSWLPVEGDADICDCCSNGNCSNSSSSEFETHEPAQWSTL |
| P97708 | WLPVEGDADICDCCSNGNCSNSSSSEFETHEPAQWSTLVSR |
| P10761 | SILRTNRVEVPIECRYPRQGNVSSHPIQPTWVPFRATVSSE |
| P10761 | QVPRPRPETLQFTVDVFHFANSSRNTLYITCHLKVAPANQI |
| P10761 | HLKVAPANQIPDKLNKACSFNKTSQSWLPVEGDADICDCCS |
| P10761 | SQSWLPVEGDADICDCCSHGNCSNSSSSQFQIHGPRQWSKL |
| P10761 | WLPVEGDADICDCCSHGNCSNSSSSQFQIHGPRQWSKLVSR |
| Q8R121 | EEKEEGKDEEYWLRASQQLSNETSSFGFNLLRKISMRHDGN |
| Q8R121 | LSQGSFAFIHKDFDIKETYFNLSKKYFDIEYVSINFQNSSQ |
| Q8R121 | DTFHLDKYRAIKVPMMYREGNFTSTFDKKFRCHILKLPYQG |
| Q8R121 | FTSTFDKKFRCHILKLPYQGNATMLVVLMEKTGDYLALEDY |
| P42098 | VTDDALVYSTFLRHDPRPAGNLSILRTNRAEVPIECHYPRQ |
| P42098 | SILRTNRAEVPIECHYPRQGNVSSWAILPTWVPFRTTVFSE |
| P42098 | FRTTVFSEEKLVFSLRLMEENWSAEKMTPTFQLGDRAHLQA |
| P42098 | KAPRPGPETLQFTVDVFHFANDSRNTIYITCHLKVTPADRV |
| Q9UK55 | VWLVPGLAPSPQSPETPAPQNQTSRVVQAPKEEEEDEQEAS |
| Q9UK55 | LTQGSFAFIHKDFDVKETFFNLSKRYFDTECVPMNFRNASQ |
| Q9UK55 | TFFNLSKRYFDTECVPMNFRNASQAKRLMNHYINKETRGKI |
| Q9UK55 | FASTFDKNFRCHVLKLPYQGNATMLVVLMEKMGDHLALEDY |
| P05532 | ARVDDSGVFMCYANNTFGSANVTTTLKVVEKGFINISPVKN |
| P05532 | NVTTTLKVVEKGFINISPVKNTTVFVTDGENVDLVVEYEAY |
| P05532 | DLVVEYEAYPKPEHQQWIYMNRTSANKGKDYVKSDNKSNIR |
| P05532 | QWIYMNRTSANKGKDYVKSDNKSNIRYVNQLRLTRLKGTEG |
| P05532 | CTGAEQRCTTPVSPVDVQVQNVSVSPFGKLVVQSSIDSSVF |
| P05532 | VSPFGKLVVQSSIDSSVFRHNGTVECKASNDVGKSSAFFNF |
| Q9UKR0 | SIFLLLCVLGLSQAATPKIFNGTECGRNSQPWQVGLFEGTS |
| Q9UKR0 | SGWGITNHPRNPFPDLLQCLNLSIVSHATCHGVYPGRITSN |
| P06870 | DDENTAQFVHVSESFPHPGFNMSLLENHTRQADEDYSHDLM |
| P06870 | QFVHVSESFPHPGFNMSLLENHTRQADEDYSHDLMLLRLTE |
| P06870 | EEPEVGSTCLASGWGSIEPENFSFPDDLQCVDLKILPNDEC |
| P12323 | EDEDTAQHFLVSQSVPHPDFNMSLLEPHNVLPNEDYSHDLM |
| P12323 | LQCVGLEILPSKNCDDAHIANVTGTMLCAGDLAGGKDTCVG |

| | |
|---|---|
| P00759 | QRRLVRQSFRHPDYIPLIVTNDTEQPVHDHSNDLMLLHLSE |
| P00759 | LQCVNIHLLSNEKCIETYKDNVTDVMLCAGEMEGGKDTCAG |
| P07288 | VCGGVLVHPQWVLTAAHCIRNKSVILLGRHSLFHPEDTGQV |
| O75882 | PEVVATSGYALLHFFSDAAYNLTGFNITYSFDMCPNNCSGR |
| O75882 | TSGYALLHFFSDAAYNLTGFNITYSFDMCPNNCSGRGECKI |
| O75882 | DAAYNLTGFNITYSFDMCPNNCSGRGECKISNSSDTVECEC |
| O75882 | TYSFDMCPNNCSGRGECKISNSSDTVECECSENWKGEACDI |
| O75882 | EACDIPHCTDNCGFPHRGICNSSDVRGCSCFSDWQGPGCSV |
| O75882 | RGCSCFSDWQGPGCSVPVPANQSFWTREEYSNLKLPRASHK |
| O75882 | ASHKAVVNGNIMWVVGGYMFNHSDYNMVLAYDLASREWLPL |
| O75882 | HSDYNMVLAYDLASREWLPLNRSVNNVVVRYGHSLALYKDK |
| O75882 | SLALYKDKIYMYGGKIDSTGNVTNELRVFHIHNESWVLLTP |
| O75882 | GGKIDSTGNVTNELRVFHIHNESWVLLTPKAKEQYAVVGHS |
| O75882 | YLHTAVIVSGTMLVFGGNTHNDTSMSHGAKCFSSDFMAYDI |
| O75882 | LPRPDLHHDVNRFGHSAVLHNSTMYVFGGFNSLLLSDILVF |
| O75882 | YSCTANTNDCHWCNDHCVPRNHSCSEGQISIFRYENCPKDN |
| O75882 | QSSQSMSKLTLTPWVGLRKINVSYWCWEDMSPFTNSLLQWM |
| O75882 | GILSEPSTRGLKAATCINPLNGSVCERPANHSAKQCRTPCA |
| O75882 | GLKAATCINPLNGSVCERPANHSAKQCRTPCALRTACGDCT |
| O75882 | ASFPFGQCMEWYTMSTCPPENCSGYCTCSHCLEQPGCGWCT |
| O75882 | GPVKMPSQAPTGNFYPQPLLNSSMCLEDSRYNWSFIHCPAC |
| O75882 | GNFYPQPLLNSSMCLEDSRYNWSFIHCPACQCNGHSKCINQ |
| O75882 | YNWSFIHCPACQCNGHSKCINQSICEKCENLTTGKHCETCI |
| O75882 | ACQCNGHSKCINQSICEKCENLTTGKHCETCISGFYGDPTN |
| O75882 | TAINFVATPDEQNRDLDMFINASKNFNLNITWAASFSAGTQ |
| O75882 | PDEQNRDLDMFINASKNFNLNITWAASFSAGTQAGEEMPVV |
| O75882 | NIKEYKDSFSNEKFDFRNHPNITFFVYVSNFTWPIKIQIAF |
| O75882 | SNEKFDFRNHPNITFFVYVSNFTWPIKIQIAFSQHSNFMDL |
| Q76LX8 | AQFRVHLVKMVILTEPEGAPNITANLTSSLLSVCGWSQTIN |
| Q8IUA7 | LKGRTIMGWPDEKSMDELDLNYSIDAVRVIFTDTFSYHLKF |
| Q8IUA7 | EKGFVAFQAAINAAIIEIATNHSVMEQLMSVTGVHMKILPF |
| Q8IUA7 | FLHSLRRQNIAIEVDAFGTRNGTDDPSYNGAIIVSGDEKDH |
| O94911 | LGRVDTFNESRFSVVYTPVTNTTQQIMNKVASTPFLAGKEV |
| O94911 | DLYLFSCIISFSSFIYYASVNVTRERKRMKALMTMMGLRDS |
| O94911 | HDSFEQAPPEFQGKEAIRIRNVTKEYKGKPDKIEALKDLVF |
| O94911 | PTKGSVTIYNNKLSEMADLENLSKLTGVCPQSNVQFDFLTV |
| O94911 | FIQSVEHQNIALEVDAFGTRNGTDDPSYNGAITVCCNEKNY |
| O95342 | VFIDYDVELQELQIPGKACVNNTIVWTNSSLNQNMTNGTRC |
| O95342 | ELQELQIPGKACVNNTIVWTNSSLNQNMTNGTRCGLLNIES |
| O95342 | IPGKACVNNTIVWTNSSLNQNMTNGTRCGLLNIESEMIKFA |
| O95342 | KACVNNTIVWTNSSLNQNMTNGTRCGLLNIESEMIKFASYY |

| | |
|---|---|
| Q09427 | IDYWLAKWTDSALVLSPAARNCSLSQECDLDQSVYAMVFTL |
| Q09429 | IDYWLAKWTDSALVLSPAARNCSLSQECALDQSVYAMVFTV |
| Q9UNQ0 | YGFTALQHNEFLGQNFCPGLNATGNNPCNYATCTGEEYLVK |
| U6A629 | LPLLASYASAVTISVAKSGGNVTTGLQYGAMEEEINHCGEG |
| U6A629 | TYPSNLDAWSAVGGSTLSLQNLSNPLSSALPTSVRITGKGT |
| U6A629 | ANDWVQHEFTLTPKKKASSSNNTFVLTFDASKASGGSLDFN |
| U6A629 | LRFPGGNNLEGQTIEGRWKWNETIGPLTQRPGRATTWGYEE |
| U6A629 | AKYGHPKPWTIRYVEVGNEDNLSSGLSSYKSYRFQAFYDAI |
| U6A629 | KSTSWHVYNNHMTNTLPATSNDTFGPPLLCHRCETAKTNSH |
| U6A629 | LCHRCETAKTNSHIFKAAVYNSTADVPVSLTFEGVGRGTTA |
| Q8NK89 | ISPLTAGGIADASAQDTFCANTTCLITIIYDQSGNGNHLTQ |
| Q8NK89 | NAETSSTDTGAGHMEAIYLGNSTTWGYGAGDGPWIMVDMEN |
| U6BLZ9 | KRGSDGATTDIAPRHAGGVANATHQDTFCAGTTCLITIIYD |
| U6BLZ9 | PNKWAIRGGNAASGPLSTFYNGSRPNARGYNPMSKEGAIIL |
| U6BLZ9 | GSSISLRATTLCCTTRYVAHNGSTVNTQVVSSSSSAALKQQ |
| P42254 | LLSLVQNAHGISLKVSTQGGNSSSPILYGFMFEDINHSGDG |
| P42254 | STQGGNSSSPILYGFMFEDINHSGDGGIYGQMLQNPGLQGT |
| P42254 | GDGGIYGQMLQNPGLQGTAPNLTAWAAVGDATIAIDGDSPL |
| P42254 | HSSFWIKGDYSGDITVRLVGNYTGTEYGSTTITHTSTADNF |
| P42254 | GNYTGTEYGSTTITHTSTADNFTQASVKFPTTKAPDGNVLY |
| P42254 | LRFPGGNNLEGNSAENRWKWNETIGDLCDRPGREGTWTYYN |
| P42254 | GDTSTTYGAWRAANGQEEPWNLTMVEIGNEDMLGGGCESYA |
| P42254 | YHDYSTPDGLVGQFNYFDNLNRSVPYFIGEYSRWEIDWPNM |
| P42254 | FERNSDVVKMAAYAPLLQLINSTQWTPDLIGYTQSPGDIFL |
| P13689 | LMGSSSLKYPGQPQEIPFFQNTTFSIPVNDPHQVWNSDEHE |
| P11140 | VMIYDCTSAVAEATYWEIWDNGTIINPKSALVLSAESSSMG |
| P11140 | GGTLTVQTNEYLMRQGWRTGNNTSPFVTSISGYSDLCMQAQ |
| P38433 | PVGNLRFKKPKPKQPWRIPLNATTPPNSCIQSEDTYFGDFY |
| P38433 | EPRDVALARAVILYNAMKCGNMSLINPDYDRILDCFQRADA |
| P38433 | VLHGYEINFIFGEPLNQKRFNYTDEERELSNRFMRYWANFA |
| P38433 | DGSFTQDVWPKYNSVSMEYMNMTVESSYPSMKRIGHGPRRK |
| Q9BYF1 | NHEAEDLFYQSSLASWNYNTNITEENVQNMNNAGDKWSAFL |
| Q9BYF1 | SAFLKEQSTLAQMYPLQEIQNLTVKLQLQALQQNGSSVLSE |
| Q9BYF1 | YPLQEIQNLTVKLQLQALQQNGSSVLSEDKSKRLNTILNTM |
| Q9BYF1 | WDAQRIFKEAEKFFVSVGLPNMTQGFWENSMLTDPGNVQKA |
| Q9BYF1 | AATPKHLKSIGLLSPDFQEDNETEINFLLKQALTIVGTLPF |
| Q9BYF1 | QEALCQAAKHEGPLHKCDISNSTEAGQKLFNMLRLGKSEPW |
| Q9BYF1 | VRVANLKPRISFNFFVTAPKNVSDIIPRTEVEKAIRMSRSR |
| P07140 | QERYEYFPGFSGEEIWNPNTNVSEDCLYINVWAPAKARLRH |
| P07140 | EHPNGKQADTDHLIHNGNPQNTTNGLPILIWIYGGGFMTGS |
| P07140 | HMTSEKAVEIGKALINDCNCNASMLKTNPAHVMSCMRSVDA |

| | |
|---|---|
| P07140 | GEWMGVLHGDEIEYFFGQPLNNSLQYRPVERELGKRMLSAV |
| P21836 | RRRATLLARLVGCPPGGAGGNDTELIACLRTRPAQDLVDHE |
| P21836 | VVKDEGSYFLVYGVPGFSKDNESLISRAQFLAGVRIGVPQA |
| P21836 | GVPHGYEIEFIFGLPLDPSLNYTTEERIFAQRLMKYWTNFA |
| P04058 | PVGNMRFRRPEPKKPWSGVWNASTYPNNCQQYVDEQFPGFS |
| P04058 | GDHNVICPLMHFVNKYTKFGNGTYLYFFNHRASNLVWPEWM |
| P04058 | GVIHGYEIEFVFGLPLVKELNYTAEEEALSRRIMHYWATFA |
| P04058 | HQRLRVQMCVFWNQFLPKLLNATACDGELSSSGTSSSKGII |
| P12821 | LLLLLPPQPALALDPGLQPGNFSADEAGAQLFAQSYNSSAE |
| P12821 | LQPGNFSADEAGAQLFAQSYNSSAEQVLFQSVAASWAHDTN |
| P12821 | NSSAEQVLFQSVAASWAHDTNITAENARRQEEAALLSQEFA |
| P12821 | QEFAEAWGQKAKELYEPIWQNFTDPQLRRIIGAVRTLGSAN |
| P12821 | TLGSANLPLAKRQQYNALLSNMSRIYSTAKVCLPNKTATCW |
| P12821 | YNALLSNMSRIYSTAKVCLPNKTATCWSLDPDLTNILASSR |
| P12821 | VPFPDKPNLDVTSTMLQQGWNATHMFRVAEEFFTSLELSPM |
| Q5RDA4 | SLGWLKSNCRHERDAGVVCTNETRSTHTLDLSRELSEALGQ |
| Q5RDA4 | HTVILTANLEAQALWKEPGSNVTMSVDAECVPMVRDLLRYF |
| Q5RDA4 | LVEKIRFPMMLPEELFELQFNLSLYWSHEALFQKKTLQALE |
| Q5RDA4 | LQALEFHTVPFQLLARYKGLNLTEDTYKPRIYTSPTWSAFV |
| Q5RDA4 | VADVTDFEGWKAAIPSALDTNSSKSTSSFPCPAGHFNGFRT |
| A7E3W2 | CENMWDLTDASVVCRALGFQNATEALGGAAFGPGYGPIMLD |
| A7E3W2 | SLGWMRSNCRHDKDASVICTNETRGVYTLDLSGELPAALEQ |
| A7E3W2 | LVGQVRFPMMPPQDLFSLQFNLSLYWSHEALFQKKILQALE |
| A7E3W2 | LQALEFHTVPFELLAQYWGLNLTEGTYQPRLYTSPTWSQSV |
| Q99538 | YDDIAYSEDNPTPGIVINRPNGTDVYQGVPKDYTGEDVTPQ |
| Q99538 | DHGSTGILVFPNEDLHVKDLNETIHYMYKHKMYRKMVFYIE |
| Q99538 | VEDLTKETLHKQYHLVKSHTNTSHVMQYGNKTISTMKVMQF |
| Q99538 | HKQYHLVKSHTNTSHVMQYGNKTISTMKVMQFQGMKRKASS |
| Q9BXB1 | LTAVPEGLSAFTQALDISMNNITQLPEDAFKNFPFLEELQL |
| Q9BXB1 | LQALTLALNKISSIPDFAFTNLSSLVVLHLHNNKIRSLSQH |
| Q9BXB1 | LRTIHLYDNPLSFVGNSAFHNLSDLHSLVIRGASMVQQFPN |
| Q9BXB1 | NLSDLHSLVIRGASMVQQFPNLTGTVHLESLTLTGTKISSI |
| Q9BXB1 | EDNSLQDHSVAQEKGTADAANVTSTLENEEHSQIIIHCTPS |
| O75473 | DGRMLLRVDCSDLGLSELPSNLSVFTSYLDLSMNNISQLLP |
| O75473 | LSELPSNLSVFTSYLDLSMNNISQLLPNPLPSLRFLEELRL |
| O75473 | LQAMTLALNKIHHIPDYAFGNLSSLVVLHLHNNRIHSLGKK |
| O75473 | CAFGVCENAYKISNQWNKGDNSSMDDLHKKDAGMFQAQDER |
| O75473 | LFTNCILNCPVAFLSFSSLINLTFISPEVIKFILLVVVPLP |
| P38571 | TLHSEGSGGKLTAVDPETNMNVSEIISYWGFPSEEYLVETE |
| P38571 | LVETEDGYILCLNRIPHGRKNHSDKGPKPVVFLQHGLLADS |
| P38571 | VVFLQHGLLADSSNWVTNLANSSLGFILADAGFDVWMGNSR |

| | |
|---|---|
| P38571 | AFSYDEMAKYDLPASINFILNKTGQEQVYYVGHSQGTTIGF |
| P38571 | LKELCGNLCFLLCGFNERNLNMSRVDVYTTHSPAGTSVQNM |
